# An integrated computational and experimental study to investigate *Staphylococcus aureus* metabolism

**DOI:** 10.1038/s41540-019-0122-3

**Published:** 2020-01-30

**Authors:** Mohammad Mazharul Islam, Vinai C. Thomas, Matthew Van Beek, Jong-Sam Ahn, Abdulelah A. Alqarzaee, Chunyi Zhou, Paul D. Fey, Kenneth W. Bayles, Rajib Saha

**Affiliations:** 1 Department of Chemical and Biomolecular Engineering, University of Nebraska-Lincoln, Lincoln, NE USA; 2 Center for Staphylococcal Research, Department of Pathology and Microbiology, University of Nebraska Medical Center, Omaha, NE USA

**Keywords:** Biochemical networks, Systems analysis

## Abstract

*Staphylococcus aureus* is a metabolically versatile pathogen that colonizes nearly all organs of the human body. A detailed and comprehensive knowledge of staphylococcal metabolism is essential to understand its pathogenesis. To this end, we have reconstructed and experimentally validated an updated and enhanced genome-scale metabolic model of *S. aureus* USA300_FPR3757. The model combined genome annotation data, reaction stoichiometry, and regulation information from biochemical databases and previous strain-specific models. Reactions in the model were checked and fixed to ensure chemical balance and thermodynamic consistency. To further refine the model, growth assessment of 1920 nonessential mutants from the Nebraska Transposon Mutant Library was performed, and metabolite excretion profiles of important mutants in carbon and nitrogen metabolism were determined. The growth and no-growth inconsistencies between the model predictions and in vivo essentiality data were resolved using extensive manual curation based on optimization-based reconciliation algorithms. Upon intensive curation and refinements, the model contains 863 metabolic genes, 1379 metabolites (including 1159 unique metabolites), and 1545 reactions including transport and exchange reactions. To improve the accuracy and predictability of the model to environmental changes, condition-specific regulation information curated from the existing knowledgebase was incorporated. These critical additions improved the model performance significantly in capturing gene essentiality, substrate utilization, and metabolite production capabilities and increased the ability to generate model-based discoveries of therapeutic significance. Use of this highly curated model will enhance the functional utility of omics data, and therefore, serve as a resource to support future investigations of *S. aureus* and to augment staphylococcal research worldwide.

## Systems Biology: Integrated study of Staphylococcus aureus metabolism

Integration of in vivo experiment with a newly developed model of *Staphylococcus aureus* metabolism helps explore its metabolic versatility. A multidisciplinary team led by Rajib Saha at the University of Nebraska developed a new genome-scale metabolic model of the multi-drug resistant pathogen *S. aureus* by combining genome annotation data, reaction stoichiometry, and condition- and mutant-specific regulations from biochemical databases and previous strain-specific models. Extensive manual curation and incorporation of newly generated experimental data on growth and metabolite production improved the accuracy and predictability of the model and increased its ability to generate model-based discoveries of therapeutic significance. Use of this highly curated model will enhance the functional utility of omics data and, therefore, serve as a resource to support future investigations of *S. aureus* and to augment staphylococcal research worldwide.

## Introduction

*S. aureus* is a versatile human pathogen that has emerged as one of the most successful infectious agents of recent times, affecting approximately 20% of the world’s population.^1–3^ The incidence of methicillin resistance at low fitness cost has significantly contributed to the rise in **c**ommunity-**a**ssociated **m**ethicillin **r**esistant *S. aureus* (CA-MRSA) infections, which significantly limit therapeutic options and increase rates of mortality, morbidity and costs associated with its treatment.^1,4,5^ This threat to human health has resulted in a steady interest and focus on understanding how staphylococcal metabolism relates to antibiotic resistance and pathogenesis. A number of studies have attempted to explore the metabolic aspects of antimicrobial functionality of MRSA, including nitric oxide metabolism, oxidative stress, carbon overflow metabolism, redox imbalance etc.^6–11^ However, a complete mechanistic understanding of staphylococcal metabolism is still missing, making the identification of therapeutic targets challenging.

The increase in knowledge of macromolecular structures, availability of numerous biochemical database resources, advances in high-throughput genome sequencing, and increase in computational efficiency have accelerated the use of in silico methods for metabolic model development and analysis, strain design, therapeutic target discovery, and drug development.^12–17^ There have been a number of attempts to reconstruct the metabolism of multiple strains of *S. aureus* using semi-automated methods.^18–22^ However, the absence of organism-specific metabolic functions and the inclusion of genes without any specified reactions still limit the utility of these models. These models need to be continually refined and updated to accurately predict biological phenotypes by addressing these issues, as well as by reducing metabolic network gaps, elemental imbalance, and missing physiological information. Since the predictive genome-scale metabolic models of several microorganisms were useful in performing in silico gene essentiality and synthetic lethality analyses and yielded promising results in pinpointing metabolic bottlenecks and potential drug targets,^14,23–26^ the potential for accurately modeling *S. aureus* metabolism is immense. To this end, Seif et al. recently developed an updated genome-scale model of *S. aureus* strain JE2, incorporated 3D protein structures, evaluated gene essentiality predictions against experimental physiological data, and assessed flux distributions in different media types.^21^ Their model was informed by multilevel omics data and a significant step toward deciphering the metabolic differences of this organism under different environmental conditions. Given the vast knowledgebase of experimental data, incorporation of the latest strain-specific annotation information, addition of condition-specific and mutant-specific regulations, and removal of spurious functions could result in a refined and more useful metabolic model for *S. aureus* USA300_FPR3757.

Several other studies have been dedicated to elucidating the metabolic aspects of staphylococcal virulence and to pinpoint the key metabolic “hubs” in carbon and nitrogen metabolism.^11,27–32^ However, a majority of these studies were focused on specific segments of staphylococcal metabolism and overlooked a system-wide interdependence that drives fitness, metabolic robustness, virulence, and antimicrobial resistance. Hence, a holistic approach of in silico genome-scale modeling and in vivo experimentation is crucial for gaining an improved mechanistic understanding of staphylococcal metabolism, and thereby, facilitating the development of novel therapeutic strategies to combat staphylococcal infections.

In this study, a comprehensive genome-scale metabolic model of *S. aureus* USA300_FPR3757, namely *i*SA863, was reconstructed using annotation information from biochemical databases^33,34^ and previous strain-specific models,^19,20,34^ and validated through experimental observations and published phenotypic data. Strain USA300 FPR3757 is one of the common MRSA strains with available genome annotation (GenBank accession number NC_007793.1) and is closely related to the strain JE2 (with only 11 SNPs between these strains).^35^ The Nebraska Transposon Mutant Library (NTML)^36^ was developed for JE2; however, the *S. aureus* USA300 FPR3757 chromosomal genome sequence was used to map transpositions of *bursa aurealis* into the genome of *S. aureus* JE2, since the annotated genome sequence of strain JE2 was not available at that time. Therefore, the modeling framework took advantage of the existing knowledgebase. The model underwent extensive manual curation to ensure chemical and charge balance, thermodynamic consistency, and biomass precursor production. To test and inform the model, the fitness level of 1920 mutants from NTML^36^ was assessed, and the metabolite excretion profiles of eight important mutants distributed across several pathways of the carbon and nitrogen metabolism were measured. The growth-phenotyping results of the NTML mutants were utilized via GrowMatch procedure^37^ to reconcile in silico versus in vivo growth inconsistencies. Upon incorporating conditional regulations in the model gleaned from existing “omics” datasets,^30,38,39^ the predictive capability of the model in terms of gene essentiality and metabolite excretions in different environmental conditions was further improved. Furthermore, the growth predictions from the model on 69 different carbon sources were validated against the existing growth experiment.^21^ Overall, this model is extensively tested by multiple available and newly developed experimental datasets on staphylococcal metabolism and subsequently refined to pave a way forward to advance system-wide analysis of fitness and virulence.

## Results

### Preliminary reconstruction utilizing the existing knowledgebase

A collection of 1511 metabolic reactions obtained from a consensus of recently published strain-specific models^19,20^ was assembled into a preliminary model of *S. aureus*. Out of 842 genes in the latest strain-specific USA300_FPR3757_uid58555 model by Bosi et al.,^19^ 109 did not have any reactions associated with them, which were not included in our model at this stage. Checking reactions from the *S. aureus* N315 model *i*SB619^20^ against the annotations of strain USA300_FPR3757 in the KEGG database^40^ resulted in the inclusion of seven unique reactions to the preliminary model. In addition, every reaction in the model was verified for correct gene annotations in the NCBI, KEGG, and UniProt databases and published resources^19,21,40–43^ to amend the model with 90 metabolic reactions and annotate 75 reactions with correct Gene-Protein-Reaction (GPR) rules.

These amendments resulted in a preliminary model that contained 858 metabolic genes catalyzing 1608 reactions involving 1499 metabolites. This model included reactions for central carbon metabolism, secondary biosynthesis pathway, energy and cofactor metabolism, lipid synthesis, elongation and degradation, nucleotide metabolism, amino acid biosynthesis, and degradation. The protocol outlined by Thiele et al. 2010^44^ was followed when developing the biomass equation according to experimental measurements of macromolecular composition^22^ and transcriptomic data^45^ and the biomass compositions by previous models.^19–21^ Biomass precursors that do not have either experimental measurements or any literature evidence of synthesis in *S. aureus* were excluded. For example, *S. aureus* lacks an identifiable polyamine biosynthetic pathway and therefore cannot produce putrescine.^28,46^ In addition, phosphatidylethanolamine is not produced in *S. aureus*.^47,48^ Therefore, these components are not included in our biomass equation (see Supplementary Data 1 for the detailed list of the biomass precursors).

### Model curation to ensure chemical balance and thermodynamic consistency

The preliminary reconstruction underwent extensive manual curation steps as outlined in the “Methods” section. In total, 197 reactions (excluding the biomass reaction, demand, sink, and exchange reactions) were found to be imbalanced in terms of proton, carbon, nitrogen, oxygen, or sulfur. Most of these reactions (i.e.,182 reactions) were fixed for proton imbalance and four reactions were fixed for imbalance in other elements (see Supplementary Data 2 for details). Nonetheless, a few mass- and charge-imbalanced reactions remained in the model, primarily due to the presence of macromolecules with unspecified “R”-groups and gaps in knowledge about the correct reaction mechanisms. These remaining reaction imbalances are common in published genome-scale metabolic models,^49^ and given that the overall stoichiometry of the reactions involving these macromolecules is correct, these imbalances do not significantly affect the performance of the model.

In addition to charge and elemental imbalances, the preliminary model had 291 reaction fluxes unnecessarily hitting the upper or lower bounds during a Flux Variability Analysis (FVA) when no nutrients were provided (see the Methods section). Also, the inconsistent dissipation of ATP and other cofactors, which was persistent in earlier models,^19^ also existed in the preliminary reconstruction. These two phenomena are observed when the reaction network contains thermodynamically infeasible cycles (as defined in the Methods section).^50^ To resolve these cycles, 42 reactions were made irreversible, and four reactions were reversed in directionality either when thermodynamic information and literature evidence were available, or the restrictions assumed did not conflict with any literature evidence but resolved an infeasible cycle (details in Supplementary Data 3 and Supplementary Figs. S1–S3). Furthermore, 72 reactions were turned off either due to their improper annotations or to remove lumped or duplicate reactions from the model. For example, the irreversible duplicates for several reactions including acetolactate synthase, aconitase, phosphoribosylaminoimidazole carboxylase, alcohol-NAD oxidoreductase, arginine deiminase, d-ribitol-5-phosphate NAD 2-oxidoreductase, glycerate dehydrogenase, methionine synthase, and ribokinase were removed. Also, based on available cofactor specificity information,^51,52^ reactions such as cytidine kinase (GTP), glycerol-3-phosphate dehydrogenase (NAD), guanylate kinase (GMP:dATP), and homoserine dehydrogenase (NADH) were turned off to ensure correct cofactor usage in these reactions. Reactions involved in polyamine synthesis and degradation were removed due to the lack of convincing evidence of polyamine metabolism in *S. aureus* USA300_FPR3757.^28,46^ After these manual curation steps, the number of unbounded reactions (reaction fluxes hitting either the upper or the lower bound without any nutrient uptake) was reduced to seven. At this step, the model was checked for erroneous generation of energetic cofactors and confirmed that it could not produce unlimited amount of them without any nutrient input, as described by Zomorrodi and Maranas^53^ and followed in previous modeling studies by us^54–58^ and other groups.^59,60^

**Fig. 1:**
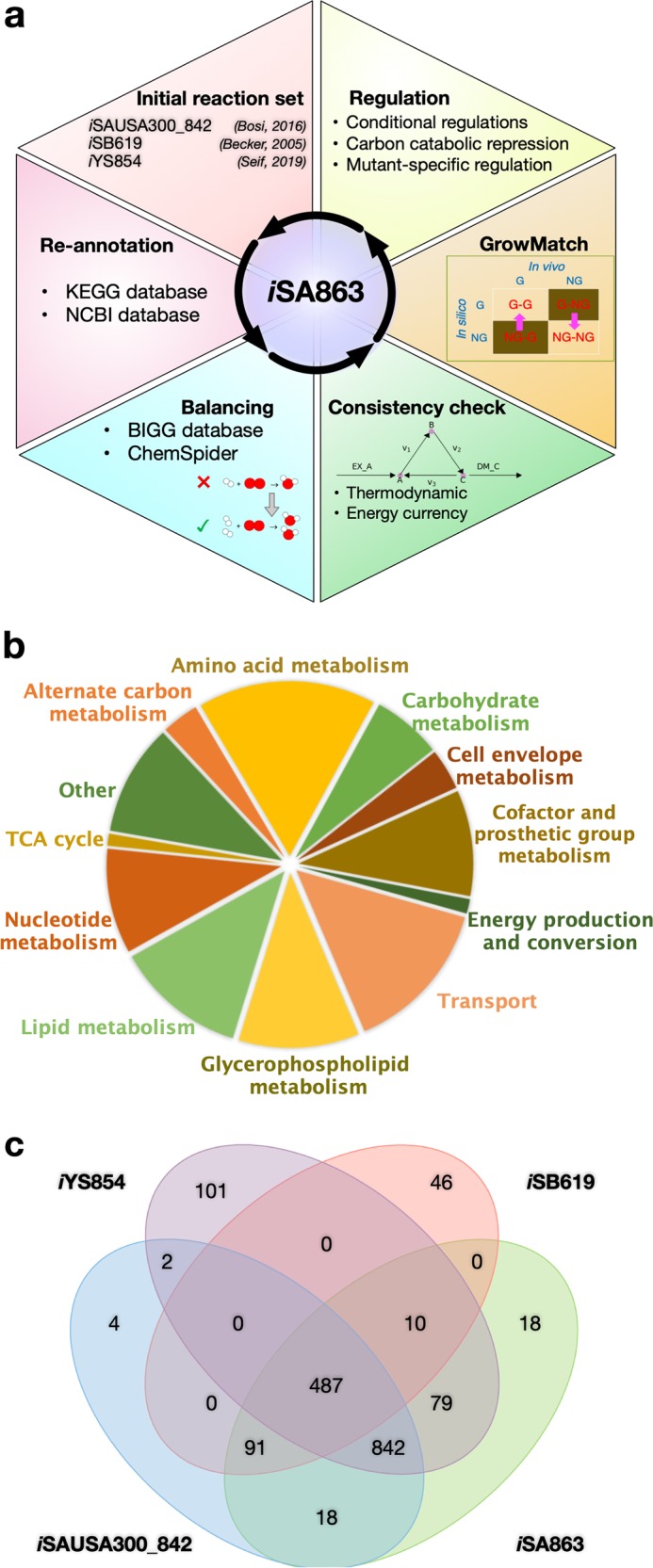
The overall view of the model reconstruction. **a** The schematic of the reconstruction and curation process for *i*SA863. **b** Pathway distribution of metabolic reactions. **c** Overlap of reactions between recent genome-scale metabolic reconstructions of *S. aureus*.

The annotation of *S. aureus* USA300_FPR3757 genome in the KEGG database was next used to bridge several network gaps in the model. At this stage, the model contained 528 blocked reactions compared with 784 in the preliminary reconstruction. While this was a significant improvement, the model still contained a greater number of blocked reactions than other similar-sized models.^21^ The blocked reactions were not removed at the current stage because they either contained proper gene annotation information and their terminal dead-end metabolite was beyond the scope of the model or no convincing evidence (e.g., high-score annotations) for filling the gap was available. A detailed list of the corrections and additions/removals made is given in Supplementary Data 3. The model reconstruction process, pathway distribution, and overlap of reactions with other *S. aureus* models are shown in Fig. 1 and the comparative model statistics are presented in Table 1. The model is available in systems-biology markup language format (SBML level 3 version 1 with fbc version 2) in Dataset 1. Metabolite InChI Keys, elemental formulas, and metabolite ID mapping to KEGG and Modelseed databases are included in Supplementary Data 4.

**Table 1:** Comparison of model statistics between recent *S. aureus* metabolic models.

	*i*SB619^20^	*i*SAUSA300_FPR3757^19^	*i*YS854^21^	*i*SA863 (this work)
Genes	619	842^a^	854	863
Reactions	640	1517	1440	1545
Metabolites	571	1431	1327	1379 (1159 internal)
Imbalanced reactions^b^	–	490	–	0
Blocked reactions	108 (~175)	784 (~52%)	428 (~30%)	528 (~34%)
Unbounded reactions	–	291 (~19%)	53 (~19%)	7 (~0.5%)

^a^In total, 732 associated with reactions.

^b^Excluding reactions with unspecified macromolecular formula.

### Identifying essential genes from existing knowledgebase

Disagreement regarding gene essentiality was persistent among existing datasets.^18,61–65^ For example, our growth evaluation study of the viable *S. aureus* mutants from NTML^36^ found varying degrees of growth inhibition (see Supplementary Data 5 for details), while 41 of them were reported to be essential in other recent studies.^18,61–65^ Therefore, the set of essential genes was a consensus of multiple literature sources^18,61–65^ and our experiments (see Methods and Supplementary Information 1 for details). Briefly, the common essential gene set (comprising 319 genes) from transposon mutagenesis followed by growth experiments by Valentino et al.^62^ and Chaudhuri et al.^64^ was considered to be essential. Of these genes, the 48 mutants, which were viable in our growth experiment, were filtered out from the consensus, unless they were reported to be domain-essential genes (explanation in Supplementary Information 1). Santiago et al.^61^ demonstrated that gene essentiality derived from transposon libraries can be affected by the high temperatures used to remove the plasmid delivery vehicle and also by the polar effect in disrupting expression of essential genes in the vicinity of a nonessential gene. Therefore, following their results, these false-positive genes (30 in total) were excluded from the essential gene list. Finally, for the modeling purpose, only the 167 metabolic genes (excluding 74 non-metabolic genes) present in the model were considered to be the core set of essential genes in the current study (see Supplementary Data 6 for the full list of the essential genes).

### Model refinement to reconcile growth and no-growth inconsistencies

**Fig. 2:**
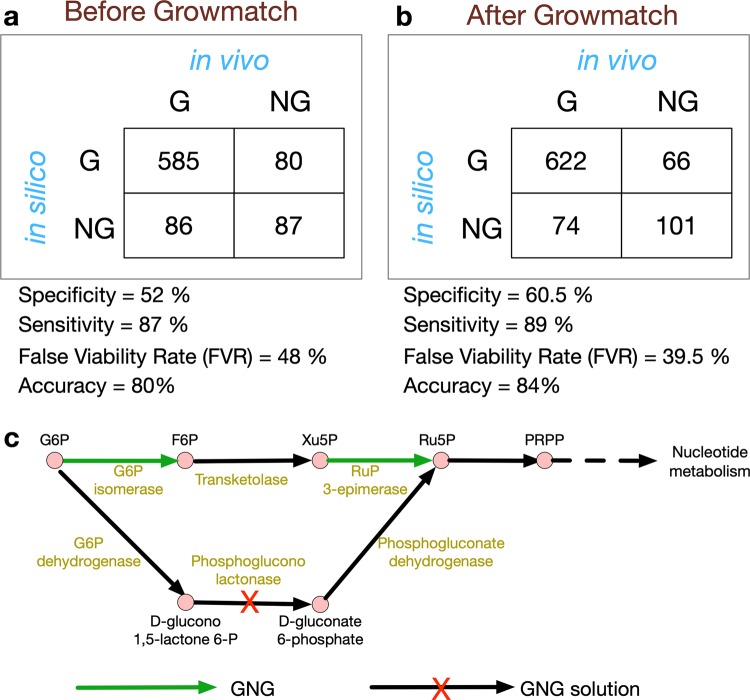
Growth–no-growth (G–NG) prediction matrices and the impact of Growmatch application. **a** Before reconciliation of growth–no-growth inconsistency by GrowMatch procedure. **b** After reconciliation of growth–no-growth inconsistency by GrowMatch procedure. Here, specificity = #NGNG/(#NGNG + #GNG), sensitivity or true viable rate (TVR) = #GG/(#GG + #NGG), false viable rate (FVR) = #GNG/(#GNG + #NGNG), and accuracy = (#GG + #NGNG)/(#GG + #GNG + #NGG + #NGNG). **c** A case study of GNG inconsistency and the corresponding Growmatch solution.

Comparison of essential and nonessential genes between the experimental (in vivo) and model-based (in silico) gene essentiality analysis (see Methods section for details) showed some disagreements (Fig. 2a). Correct model predictions for nonessential and essential genes were denoted by GG and NGNG, while wrong model predictions for nonessential and essential genes were denoted by NGG and GNG, respectively, in which the first of the two terms (“G” or “NG”) corresponds to in silico and the second term refers to in vivo observations. An optimization-based procedure called Growmatch was used to reconcile the GNG inconsistencies by suppressing spurious functionalities and the NGG inconsistencies by adding misannotated functionalities to the model.^37^ The overall impact of applying Growmatch is shown in Fig. 2b. The specificity increased from 52 to 60.5%, the sensitivity increased from 87 to 89%, the false viability rate decreased from 48 to 39.5%, and the accuracy increased from 80 to 84%. In comparison, the specificity, sensitivity, false viability rate, and accuracy of *i*YS854^21^ could be calculated to be 50.6, 93.2, 49.4, and 85%, respectively (see Supplementary Data 6). To resolve the NGG inconsistencies, metabolic reactions were added from highly curated metabolic models^66,67^ as well as the Modelseed database.^34^ A total of five reactions were added to the model, and three reactions were allowed to go in the reverse direction based on literature evidence or thermodynamic information (detailed procedure outlined in Supplementary Information 1), which reduced the number of NGGs by 12. It should be noted here that while Growmatch could suggest multiple solutions to fix an NGG inconsistency^68–71^, every suggestion needs to be manually scrutinized and filtered out if it does not have strong literature suggestion, indicating a possible gap in the genome annotation, or worsens the thermodynamic infeasibility in the model. Model predictions of essential genes were further improved upon the removal of spurious reactions and genes. To this end, six reactions that did not have either any gene associated with them (orphan reactions) or proper gene annotations, were removed from the model, resulting in an 8% reduction in GNGs. In total, 81 of the GrowMatch predicted resolution strategies were not accepted because they resulted in conflicts with correct growth (GG) and no-growth (NGNG) predictions in the model. The details of the GrowMatch results are presented in Supplementary Data 6.

It was observed that the majority of the GNG inconsistencies fall in the category of metal ion/proton antiporters and amino-acid-tRNA ligases, which indicates that non-*S. aureus*-specific and/or incorrectly annotated reactions might be present in the network. Figure 2c shows an example case of GNG inconsistency in the Pentose Phosphate Pathway, where erroneous reactions were present in the model. For example, glucose-6-phosphate isomerase and ribulose phosphate-3 epimerase are both essential genes (green highlighted genes in Fig. 2c) in *S. aureus*, while they were predicted to be nonessential by the model. The reason was the presence of an alternate pathway to convert glucose-6-phosphate (G6P) to ribulose-5-phosphate (Ru5P) in the model. Since literature and database searches failed to identify the presence of phospho-glucono lactonase in *S. aureus*, it was removed, and the model was made consistent with experimental essentiality prediction of glucose-6-phosphate isomerase and ribulose phosphate-3-epimerase genes. On the other hand, amino acid synthases, ATP-binding cassettes, and phosphoribosyltransferases are found among the NGG inconsistencies, which indicate possible cases for missing annotations. The details of NGG fixes made in this work can be found in Supplementary Data 6. It should be noted that attempting to reconcile every GNG and NGG inconsistency is out of scope of this work and not tractable with the existing knowledgebase. Since Growmatch solutions are only preliminary in silico suggestions, these gene functions need to be further verified by experimentation to enrich our knowledge about the correct genome annotation and regulatory effects.

### Model validation and refinement

An automated procedure like GrowMatch can significantly improve the gene essentiality predictions in the model. However, without extensive validation against experimental data and manual curation, it is difficult to obtain biologically significant and meaningful prediction capability from the model. Hence, the model was validated against multiple experimental observations from previous studies and results obtained in the current work for further refinements. In this step, conditional regulations, via a valve approach^72^ (see the Methods section for details), were incorporated into the model to achieve biologically meaningful distribution of fluxes that sharpened the model predictions of mutant growth phenotype and metabolite excretion behavior. The full list of regulations can be found in Supplementary Data 7. A major regulatory system that was incorporated into the model was the carbon catabolite repression, which is a well-studied global regulatory process in low-GC Gram-positive bacteria in the presence of a preferred carbon source (e.g., glucose) that induces the repression of genes involved in the metabolism of alternative carbon sources (e.g., amino acids).^30,39^ In addition, SrrAB and Rex-dependent transcriptional regulation are prominent driving forces of metabolic flux through respiratory metabolism that was integrated into the model.^73–75^ Furthermore, mutant-specific repression of respiration, histidine and ornithine metabolism, and pyruvate metabolism was imposed on the model for the *menD* mutant.^38^ In addition to the repressions mentioned above, reactions were added and constrained in flux values and directionalities as part of the refinement process.

### Model validation and refinement: growth phenotype study

**Fig. 3:**
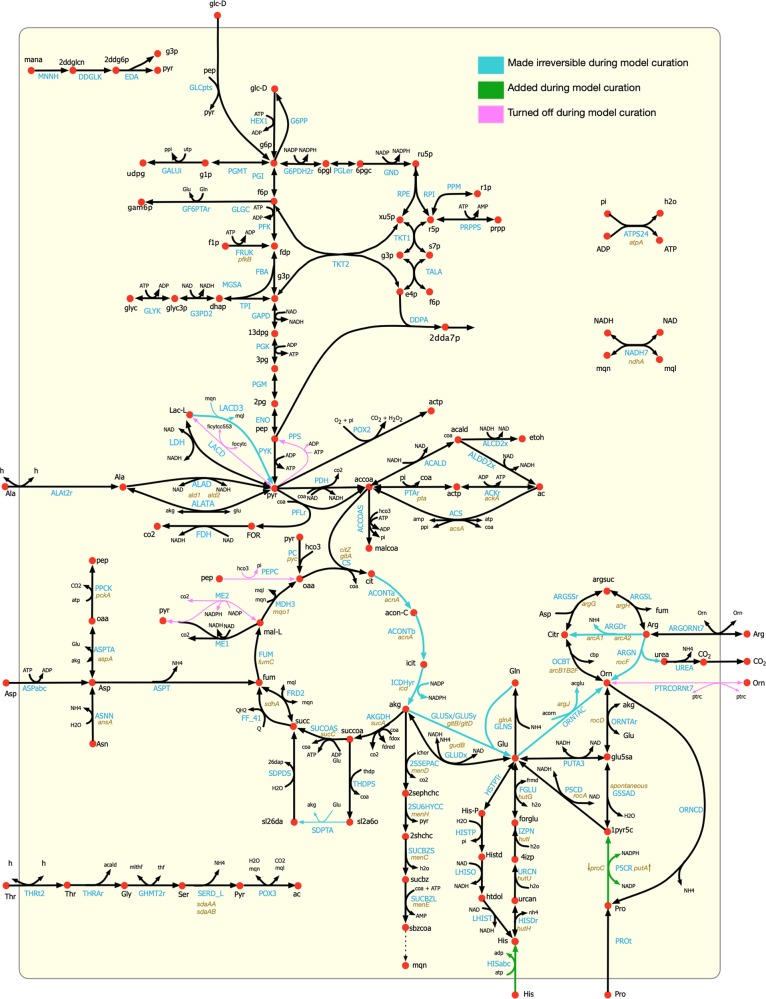
Refinements in the central metabolic pathway of the model *i*SA863. These include corrections of reaction directionality, additions, and deletions.

The essentiality predictions for 29 amino acid catabolic pathway genes in the model were validated against the mutant growth phenotypes evaluated in a previous study.^29^ The mutants were grown in a chemically defined medium (CDM) supplemented with 18 amino acids but lacking glucose. That study^29^ found that 11 of the mutations did not cause any growth defect, while 11 mutations caused intermediate growth defect and seven mutations were lethal. The model failed to recapitulate growth phenotype for nine (*ald1/ald2*—aldehyde dehydrogenase, *aspA*—aspartate aminotransferase, *gltA*—citrate synthase, *sdhA*—succinate dehydrogenase*, sdaAA/sdaAB*—serine dehydratase, *ansA*—asparaginase, *arcA1/arcA2*—arginine deiminase, and *rocF*— arginase) out of the 29 mutants, which warranted further investigation and refinements in the relevant pathways in the model. The *gudB* mutant did not appear to be an essential gene in the model simulation because other genes including d-alanine transaminase (*dat*) and aspartate transaminase (*aspA*) could convert glutamate to alpha-ketoglutarate. Based on information about kinetic limitation on alanine uptake^76^ and the experimentally measured uptake values reported by Seif et al.,^21^ a tighter constraint on alanine uptake of 0.4 mmol/gDW.h was imposed in the model, which resulted in a correct prediction of the essentiality of the *gudB* gene. The essentiality of *sucC* and *sucA* genes was ensured in the model by rectifying the direction of the alternate pathway consisting of succinyldiaminopimelate transaminase (*dapE*) and tetrahydrodipicolinate succinylase (*dapD*). In addition to that, the TCA cycle reactions converting citrate to succinyl-CoA were constrained to allow flux toward the forward direction only. Two of the gaps in the histidine transport pathway and proline catabolism were filled during the refinement process to allow for utilization of these alternate carbon sources in the absence of glucose. Ornithine–putrescine antiport, lactate dehydrogenase (ferricytochrome), malic enzyme (NADP), and succinyldiaminopimelate transaminase were removed from the model due to the lack of evidence in *S. aureus*. Upon these refinements, the model was able to correctly predict 24 (out of 29) of the mutant phenotypes, except *gltA*, *acnA*, *icd*, *fumC*, and *rocF* mutants. In comparison, the previous *S. aureus* model *i*YS854 failed to predict the growth phenotype for *gudB*, *ald1*, *ald2, pyc*, *argD*, and *gltA* mutants.^21^ The model refinements in the central metabolic pathway in terms of correction of reaction directionality, additions, and deletions are shown in Fig. 3.

### Model validation and refinement: metabolite excretion profiles of mutants

In addition to the model refinements mentioned in the preceding section, we determined the metabolite excretion profiles of eight mutants during exponential growth (Table 2) in CDM and CDMG (CDM media with added glucose) media. The mutants considered were *pyc* (pyruvate carboxylase), *citZ* (citrate synthase), *sucA* (2-oxoglutarate dehydrogenase), *ackA* (acetate kinase), *gudB* (glutamate dehydrogenase), *ndhA* (NADH dehydrogenase), *menD* (menaquinone biosynthesis protein), and *atpA* (a subunit of ATPase). These mutants were selected for their potential in affecting glycolysis, TCA cycle, gluconeogenesis, electron transport chain (ETC), cellular redox potential, overflow metabolism, and fitness, as was evident by the growth inhibition of these mutants in our experiment. In general, supplementation of glucose (CDMG) as the primary carbon source resulted in the excretion of acetate as the major by-product in all mutants (Table 2). In CDM, the *ackA*, *gudB, ndhA, atpA*, *and menD* mutants displayed delayed growth kinetics (see Supplementary Fig. S4). Acetate remained a major by-product of strains in CDM due to amino acid deamination, as evidenced by ammonia excretion (Table 2). As carbon flux through the ATP-generating Pta–AckA pathway is significant in *S. aureus*,^11,29^ we also observed the excretion of pyruvate and redirection of 75% of the carbon flux toward acetoin and α-ketoglutarate in the *ackA* mutant (Table 2). Mutations that affected respiration (*ndhA* and *menD*) of *S. aureus* resulted in increased levels of lactate production to maintain cellular redox when grown in CDMG (Table 2). The disruption of ATP production due to mutation of *atpA* was offset by increased acetate production and glucose consumption. The increased flux of glucose through the Pta–AckA pathway to generate acetate likely compensated for the decrease in ATP production due to a faulty ATPase.

**Table 2:** Metabolite excretion rates of multiple *S. aureus* mutants with altered carbon and nitrogen metabolism in CDMG and CDM culture supernatants (μM/OD_600_/h).

Strain	A-KG	Pyruvate	Lactate	Acetate	Acetoin	Glucose^a^	Urea	Ammonia
*CDMG media*								
WT	10 ± 1	─	150 ± 10	1120 ± 50	1 ± 1	3070 ± 164	60 ± 37	20 ± 22
* ackA*	20 ± 3	150 ± 18	80 ± 9	330 ± 13	170 ± 10	2070 ± 440	70 ± 38	─
* sucA*	10 ± 0	─	150 ± 12	1110 ± 32	1 ± 2	3520 ± 142	120 ± 5	160 ± 284
* gudB*	10 ± 0	─	140 ± 2	1120 ± 63	─	3400 ± 275	70 ± 70	─
* ndhA*	10 ± 1	─	500 ± 13	620 ± 19	─	2240 ± 140	20 ± 19	─
* citZ*	10 ± 0	─	120 ± 11	1250 ± 9	4 ± 6	3750 ± 199	30 ± 58	─
* pyc*	─	─	140 ± 10	1220 ± 96	─	3320 ± 99	20 ± 34	─
* atpA*	10 ± 9	10 ± 13	185 ± 13	1760 ± 16	─	2000 ± 627	─	10 ± 17
* menD*	─	2 ± 4	1300 ± 152	30 ± 59	─	500 ± 68	16 ± 22	65 ± 90
*CDM media*								
WT	1 ± 2	─	─	300 ± 15	─	─	─	790 ± 22
* ackA*	10 ± 2	─	─	─	─	─	20 ± 19	520 ± 141
* sucA*	170 ± 3	─	─	290 ± 6	─	─	20 ± 18	570 ± 132
* gudB*	─	─	─	210 ± 14	1 ± 1	─	20 ± 25	420 ± 74
* ndhA*	─	─	─	─	─	─	─	710 ± 55
* citZ*	─	─	─	670 ± 11	1 ± 2	─	─	850 ± 97
* pyc*	─	─	─	─	─	─	10 ± 9	680 ± 76
* atpA*	─	─	─	─	─	─	40 ± 14	630 ± 8
* menD*^b^	─	─	─	─	─	─	─	─

Not measured.

^a^Rate of glucose consumption.

^b^Not determined due to lack of growth of *menD* mutant in this media.

**Fig. 4:**
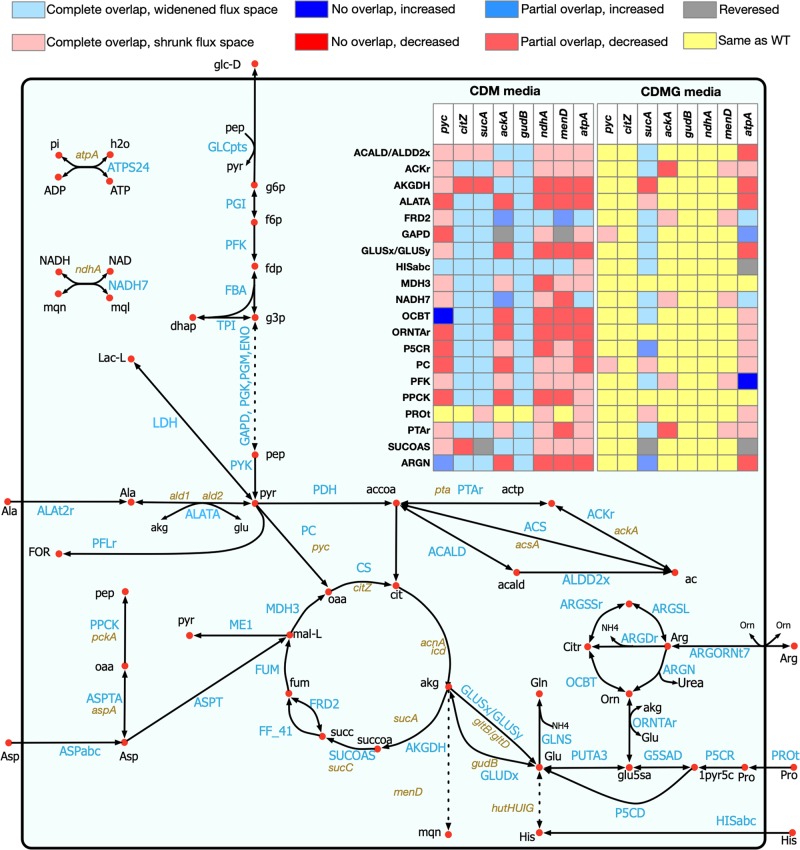
Shifts in flux space for 8 mutants in the central carbon and nitrogen metabolic pathway. Every row in the table (inset) denotes a reaction as identified in the pathway map. The relative shifts compared with the wild-type flux space are color-coded according to the legend in the figure.

Each of the mutants exhibited a deviation of the metabolic flux space (defined as the range between the minimum and maximum flux through reactions, see the Methods section for details) compared with the wild-type strain, as illustrated in Fig. 4. The redistribution of flux dictates how the different mutants excrete different metabolites. Among the eight mutants, the model-predicted excretion patterns for acetate and lactate in *sucA* and *ackA* mutants agreed with the experimental results of decreased excretion in CDMG media, compared with the wild-type strain. The Pta–AckA pathway is known to supply a major portion of the ATP required for growth.^27^ With the *atpA* gene turned off in the model, Pta–AckA pathway supplied most of the ATP demand, which increased the acetate production in CDMG media for the *atpA* mutant compared with the wild type. In CDMG media, the model-predicted excretion profile for urea in all the mutants matched with the experimental observations. In CDM media, the model predictions of higher urea excretion compared with the wild-type strain agreed with the experimental observations for *pyc*, *gudB*, *ndhA*, and *menD* mutants. Similar to the experimental results, excretion of ammonia was predicted by the model in all mutants when glucose was absent (CDM media). These correct predictions can be attributed to the deamination of the amino acids consumed in CDM media when the cell adapts to amino acids due to CcpA-mediated control of amino acid metabolism. The Rex and SrrAB repression on central carbon metabolism allowed the model to correctly simulate the oxygen deprivation in the model, which, in turn, resulted in correct predictions of decreased acetate excretion by the *ndhA* mutant in both CDM and CDMG media. Rex and SrrAB-mediated repression of pyruvate formate lyase (PFLr), alcohol dehydrogenase (ACALD, ALDD2x), and other pathways downstream of pyruvate shifted carbon flux away from the acetate production. At the same time, the flux space for lactate dehydrogenase (LDH) widened, which allowed for more lactate excretion in the CDMG media. Mutant-specific regulations and refinements also improved the model’s predictive capacity for *menD* and *pyc* mutants, which are discussed in detail in Supplementary Information 1. Incorporation of condition-specific and mutant-specific regulations were important to capture the biologically meaningful phenotypic behavior, which is evident from the observation that the unregulated model could only predict approximately 10 out of 24 cases in CDMG and 16 out of 24 cases in CDM media, while incorporation of those regulations resulted in 18 out of 24 correct predictions in CDMG and 20 out of 24 correct predictions in CDM media.

While the incorporation of the CcpA, Rex, and SrrAB regulations was critical in capturing the physiological behavior of *S. aureus* by the model, it should be noted that there are still gaps in our knowledge about the quantitative repression effect on the reaction fluxes in the presence of these regulators. To explore the quantitative effect of repression on the mutant phenotypes, different levels of repression (10, 25, 50, and 90% of the maximum wild-type flux space) were imposed on the model, and metabolite excretion behavior was observed (data not shown). The different levels of repression showed varied degrees of agreement with experimental observation, with the 50% of the wild-type cutoff preforming the best overall. However, there were specific cases when the 50% cutoff was not highly predictive. For example, in CDMG media, ammonia production was not predicted in the *menD*, *atpA*, and *sucA* mutants by the model with a 50% cutoff, which was observed experimentally. Upon further investigation, it was observed that relaxing the repressions (to 90% of the wild-type flux space), the discrepancies were removed. In addition, a stronger repression effect (10–25% of wild type) on the reactions downstream of pyruvate redirected a portion of the carbon flux to acetolactate and resulted in acetoin excretion, which was not observed with a more relaxed (50–90% of wild-type) repression effect. In CDMG media, the *citZ* mutant correctly predicted the excretion pattern of acetate, because with the reduced flux space for the TCA cycle reactions, more carbon could be directed to the Pta–AckA pathway. However, in the CDM media, when amino acids were the primary source of carbon, deletion of the *citZ* gene did change the model-predicted flux space in the Pta–AckA pathway, and hence could not capture the decrease in acetate excretion rate. The reason for these inconsistencies could be either the lack of a complete understanding of the regulatory processes that affects the relationship between amino acid catabolism, urea cycle, TCA cycle, and pyruvate metabolism, or the inherent nonlinearity that exists between gene expressions and flux levels in some cases (i.e., a limitation of any regulation-incorporating methods in a metabolic model^72^), and therefore, warrants further investigation.

### Model validation and refinement: carbon catabolism capacity

In order to further test the accuracy of the model, the growth-predictive capability of the model was validated against a recent study of carbon source utilization by *S. aureus* strain USA300-TCH1516 by Seif et al.^21^ Out of the 69 carbon sources tested, the authors observed growth on 53 metabolites and no growth on 16 metabolites in their BIOLOG experiment. Our model correctly predicted growth on 41 and no growth on 12 of the carbon sources, and falsely predicted growth on four and no growth on 12 carbon sources (see Supplementary Data 8 for details). In comparison, *i*YS854 correctly predicted growth on 42 and no growth on five of the carbon sources, and falsely predicted growth on 11 and no growth on 11 carbon sources. Overall, our model achieved a specificity of 75%, a precision of 91%, and an accuracy of 77%, which in general are either at par with or better than previously developed models,^21^ and further demonstrates the improved predictive capability of this new model.

## Discussion

In the current study, an updated and comprehensive genome-scale metabolic model of the methicillin-resistant human pathogen *S. aureus* USA300_FPR3757 was reconstructed from the previous strain-specific models,^19–21^ amended using annotations based on KEGG database,^40^ and refined and validated based on published and new experimental results. Strain USA300 FPR3757 is one of the common MRSA strains with available genome annotation (GenBank accession number NC_007793.1) and is closely related to the strain JE2.^35^ While the Nebraska Transposon Mutant Library^36^ was developed for JE2, the *S. aureus* USA300 FPR3757 chromosomal genome sequence was used to map transpositions of *bursa aurealis* into the genome of *S. aureus* JE2, since the annotated genome sequence of strain JE2 was not available at that time. Therefore, we chose to utilize the existing knowledgebase. Reactions were examined and fixed to ensure chemical and charge balance and thermodynamic consistencies. The extensive manual curation performed on the preliminary reconstruction resulted in improved prediction capabilities and successful capture of experimentally observed metabolic traits. All these demonstrate the necessity of exhaustive manual scrutiny and rectification of automated reconstructions. Further experimental results from gene essentiality, mutant growth, and metabolite excretion studies enabled high-resolution model refinements to further enhance the predictive capabilities of the model. The final genome-scale metabolic reconstruction (*i*SA863) is therefore a product of the series of automated and manual curation steps.

Our growth evaluation experiment revealed varying degrees of growth inhibition of the NTML mutants compared with the wild-type strain and identified subtle disagreements in gene essentiality predictions of other studies.^18,61–65^ Therefore, the true set of essential genes required further scrutiny, which is why, as a conservative estimate, we used a consensus set of essential genes by utilizing the existing knowledgebase and our own experimental findings (more details in Supplementary Information 1). Moreover, several mutants compromised in growth could be found in all the different methods, which did not appear to inhibit growth significantly during model simulations. Instead, the model either predicted growth at full capacity or became completely growth-inhibited. This phenomenon suggests that the model has degeneracy in the flux space that may compensate for lost functionality by redirecting or shifting metabolic fluxes. This issue calls for a more rigorous study of the regulatory influences and necessitates further future studies in enzymatic efficiencies and kinetics associated with important metabolic pathways. The growth and no-growth analysis and the resolution of inconsistencies between in silico growth predictions and in vivo results using the Growmatch algorithm^37,77^ further reinforces the importance of the iterative procedure of model refinement using experimental observations.

The growth-phenotyping studies of mutations in the amino acid catabolic pathway^29^ revealed shifts in *S. aureus* metabolism in the absence of a preferred carbon source and elucidated the extent of carbon catabolic repression, which allowed us to make necessary amendments to the model in terms of correction of reaction directionality, removal, and addition of reactions, and specifying cofactor utilization across the central metabolic pathway (see Fig. 3 for details). The change in media components (CDM vs. CDMG) resulted in a significant redistribution of metabolic flux in the model, as was evident from the shifts in flux space for different mutants in the carbon and nitrogen metabolic pathways. These shifts predicted how inactivation and/or repression of TCA cycle, respiration, electron transport, and ATP generation could impact the cellular redox balance, metabolite production, and fitness. While the model predictions for acetate and lactate production in the *ackA* and *sucA* mutants and ammonia and urea production in *ackA*, *pyc*, *gudB*, *ndhA*, and *menD* mutants matched with experimental results, other mutants showed deviations in their metabolite excretion behavior. The prediction capability of the model was improved upon the addition of regulatory information obtained from existing “omics” datasets.^30,38,39^ For example, incorporation of Rex and SrrAB regulation caused repression on pyruvate metabolism and alcohol dehydrogenase pathways, which resulted in correct predictions of acetate excretion by the *ndhA* mutant in both CDM and CDMG media, and by the *citZ* and *pyc* mutants in CDMG media. Moreover, imposing mutant-specific repressions was critical to achieve predictive results for the acetate and lactate excretion in the *menD* mutant and ammonia and urea excretion in the *atpA* mutant. However, the current knowledge of the regulatory landscape in *S. aureus* or the inherent limitation of existing regulation-incorporating methods is not sufficient to explain some of the inconsistent metabolite production trends in the remainder of the mutants, thus, warranting the need for further investigation. Although the model performed reasonably well in predicting growth on different nutrient sources, the current discrepancies are mostly due to the lack of knowledge of either synthesis routes for several biomass precursors (when it failed to produce biomass on a few carbon sources such as formate, lysine, methionine, and valine) or potential redundancies/missing regulations in the model (when it erroneously showed biomass production on several amino acids such as alanine, proline, and threonine).

*S. aureus* remains a significant threat to human health, which drives a growing number of studies toward understanding how staphylococcal metabolism relates to antibiotic resistance and pathogenesis. Very few studies have addressed these interrelationships from a systems-biology perspective, which requires a predictive in silico metabolic model capable of capturing the biochemical features of the pathogen. This work addresses these gaps through the development of a detailed metabolic model informed not only from existing resources, such as the NTML, in silico genome sequences, annotation databases, and theoretical metabolic stoichiometry but also from our own experimental studies on mutant fitness, gene essentiality, and metabolite excretion profile. The results presented in this work demonstrate the predictive capacity of the new genome-scale metabolic reconstruction of *S. aureus* USA300_FPR3757, *i*SA863, in different environments, utilizing different substrates, and with perturbed genetic contents, which paves the way for a mechanistic understanding of *S. aureus* metabolism. This latest genome-scale model of *S. aureus* demonstrates high performance in capturing gene essentiality, mutant phenotype, and substrate utilization behavior observed in experiments. However, the accuracy and prediction capability, as well as the ability to generate model-based drug-target discoveries, can be further enhanced by incorporating extensively vetted flux measurements, quantitative proteomics, and kinetic measurements of metabolic intermediates. The development of a more accurate systems-level metabolic model for *S. aureus* will have a tremendous impact on future scientific discoveries and will be a valuable resource shared among the staphylococcal research community for the identification and implementation of intervention strategies that are successful against a wide range of pathogenic strains.

## Methods

### Preliminary model reconstruction and flux balance analysis

The primary reaction set was obtained from the genome-scale metabolic reconstruction of *S. aureus* USA300_FPR3757 by Bosi et al.^19^ Reactions from the *S. aureus* N315 model *i*SB619^20^ were checked against annotations of *S. aureus* USA300_FPR3757 based on the KEGG database^40^ and merged with the reaction set to get the preliminary model. Flux balance analysis (FBA)^78–80^ was employed during model testing, validation, and analyzing flux distributions at different stages of the study. For performing FBA, the reconstruction was represented in a mathematical form of stoichiometric coefficients (known as stoichiometric matrix or S-matrix), where each column represents a metabolite and each row signifies a reaction. In addition to the mass balance constraints,^81^ environmental constraints based on nutrient availability, the relational constraint of reaction rates with concentrations of metabolites, and thermodynamic constraints were imposed as necessary. The effects of gene expressions were incorporated as regulatory constraints on the model as the cell adapted to a change in media or gene knockouts.^82^ The non-growth-associated ATP maintenance demand was estimated to be 5.00 mmol/gDCW.h in CDM media and 7.91 mmol/gDCW.h in CDMG media in this study, according to the established protocol in the absence of chemostat growth data.^44^ In CDMG media, glucose uptake rate was limited to 10 mmol/gDW.h with other nutrients set to be in excess (see Supplementary Data 9 for details). In CDM media, glucose uptake rate was set to zero.

### Rectification of reaction imbalances

To ensure that each of the reactions in the model is chemically balanced, the metabolite formula and the stoichiometry of the reactions were checked against biochemical databases.^34,40,83,84^ For balancing the reactions imbalanced in protons, the protonation state consistent with the reaction set in the preliminary model was checked, and additions/deletions of one or multiple protons or water on either the reactant or the product side were performed. For the other elements, correct stoichiometry was incorporated into the S-matrix. Reactions with the unspecified macromolecule formula were not rectified.

### Identification and elimination of thermodynamically infeasible cycles

One of the limitations of constraint-based genome-scale models is that the mass balance constraints only describe the net accumulation or consumption of metabolites, without restricting the individual reaction fluxes. Therefore, they have an inherent tendency to ignore the loop low for electric circuits, which states that there can be no flow through a closed loop in any network at steady state.^50^ While biochemical conversion cycles like TCA or urea cycle are ubiquitous in a metabolic network model, there can be cycles that do not have any net consumption or production of any metabolite. Therefore, the overall thermodynamic driving force of these cycles is zero, implying that no net flux can flow around these cycles.^50^ It is important to identify and eliminate these thermodynamically infeasible cycles (TICs) to achieve sensible and realistic metabolic flux distributions.

To identify thermodynamically infeasible cycles in the model, all the nutrient uptakes to the cell were turned off, and an optimization formulation called Flux Variability Analysis (FVA) was used.^85^ FVA maximizes and minimizes each of the reaction fluxes subject to mass balance, environmental, and any artificial (i.e., biomass threshold) constraints.^85^ The reaction fluxes which hit either the lower or upper bounds, are defined as unbounded reactions, and were grouped as a linear combination of the null basis of their stoichiometric matrix. These groups are indicative of possible thermodynamically infeasible cycles.^53^ To eliminate/destroy the cycles, duplicate reactions were removed, lumped reactions were turned off, or reactions were selectively turned on/off based on available cofactor specificity information (see Supplementary Information 1 for details).

### Simulation software

The General Algebraic Modeling System (GAMS) version 24.7.4 with IBM CPLEX solver was used to run FBA and FVA, estimate gene essentiality, calculate metabolite excretion, and run Growmatch algorithm on the model. For each of the algorithms, the required optimization algorithm was scripted in GAMS and then run on a Linux-based high-performance cluster computing system at the University of Nebraska-Lincoln. The model was parsed from Systems-Biology Markup Language (SBML) level 3 version 1 document using standard programming languages (i.e., Python) to generate the input files required by GAMS.

### Evaluation of growth profiles of mutants in NTML

Pre-cultures of wild-type and isogenic transposon mutant strains were grown overnight aerobically in 384-well plates containing 100 μL of Tryptic Soy Broth (TSB)/well with 14 mM glucose. The overnight cultures (1 μL) were seeded into a fresh 384-well plate containing TSB (100 μL/well) using a solid 384-pin tool (V & P Scientific) and cultured for 24 h at 37 °C under maximum agitation in a TECAN microplate reader. Preculture ODs were not specifically standardized due to the large number of mutants in this collection. Growth was monitored by recording the optical density (OD_600_) of cultures for 24 h at 30-min intervals. The area under the growth curve (AUC) was calculated as a measure of growth for each strain and used for comparative analyses.

### Gene essentiality analyses

Metabolic robustness of an organism in the event of genetic manipulations is attributed to the essentiality of the respective gene(s) under a specific nutrient medium or regulatory condition.^24^ In any metabolic reconstruction, there are either missing necessary functionalities in the model or erroneous pathways present in the model, mainly due to missing or wrong annotation information. To identify these inconsistencies in the model, in silico essential and nonessential genes were identified by turning off the reaction(s) catalyzed by the gene following the Boolean logic of the Gene-Protein-Reaction (GPR) relationships and estimating growth as a result of the deletion. Isozymes (i.e., proteins/genes with an “OR” relationship) for essential reactions are not considered as essential, and for reactions catalyzed by proteins with multiple subunits (i.e., proteins/genes with an “AND” relationship), each gene responsible for each subunit is considered essential. A mutant was classified as lethal if its growth rate is below a preset threshold. Essential genes with the threshold values of 1, 10, 25, and 50% of the wild-type growth rate were estimated. A 1 or 10% threshold did not have any difference in the number of essential genes, and following conventions^37,56^ used in the community, the 10% threshold was used in this study.

In vivo essential genes were curated from multiple sources,^18,61–65^ as explained in detail in Supplementary Information 1. Most of the essential genes were determined by randomly inserting transposons into *S. aureus* and excluding mutations that remained after growing the cells.^61,62,64^ An adaptation of data from multiple sources using antisense RNA was also used to determine essential enzymes and thus essential genes through the Boolean relationships.^18,63,65^ Genes reported to be essential in any source were considered essential unless there was evidence suggesting otherwise.^18,61–65^ There were three types of positive evidence. First, mutants obtained from Nebraska’s Transposon Mutant Library^36,86^ were not considered essential unless it was found to be domain-essential.^61^ This is because the transposon may have inserted in a nonessential part of the gene, allowing a partially functional protein to be formed. Second, if the gene was found to be essential at only 43 °C, then it is evident that the gene was incorrectly found to be essential in literature because of a high-temperature plasmid-curing step in the processes used in the other literature sources.^61^ Third, if the gene was found to be essential using a promoterless transposon insert, but not with promoter-containing methodologies, then the gene is upstream of an essential gene, and other sources found it to be essential due to polar effects that disrupt expression.^61^ The step-by-step methodology used in determining core essential gene set is illustrated in Supplementary Fig. S5.

Out of the consensus set of the essential genes, 167 metabolic genes that are present in the *i*SA863 metabolic model were considered for further model refinements. The results of the in silico growth estimation were compared with these experimental evidences, and the genes were classified based on the matches and mismatches between in silico and in vivo results. Correct model predictions for nonessential and essential genes are denoted by GG and NGNG, while wrong model predictions for nonessential and essential genes are denoted by NGG and GNG, respectively. GNG inconsistencies imply that the metabolic model erroneously contains reactions that complement for the lost gene function. In contrast, NGG inconsistencies are generally indicative of missing or poor annotations in the model.

### Using GrowMatch to resolve growth and no-growth inconsistencies

To resolve the growth and no-growth inconsistencies in the model, an automated procedure called GrowMatch was used.^37^ GrowMatch tries to reconcile GNG predictions by suppressing spurious functionalities that were mistakenly included in the model and NGG predictions by adding missing functionalities to the model while maintaining the already-identified correct growth and no-growth predictions.^37^ Every suggested GrowMatch modification was filtered for the resolution of conflict following the procedure of Henry et al. in 2009.^67^ Details of these cases can be found in Supplementary Data 6.

### Determination of metabolite excretion profiles of mutants

Chemically defined media (CDM) was prepared as previously described by Hussain, Hastings, and White^87^ with minor modifications to amino acid content. Amino acids were diluted to final concentrations in the media from working stocks, as described by Vitko and Richardson.^88^ Briefly, the media contained the following components: Na_2_HPO_4_.2H_2_O, 10 g/L; KH_2_PO_4_, 3 g/L; MgSO_4_.7H_2_O, 0.5 g/L biotin, 0.1 mg/L; nicotinic acid, 2 mg/L; D-pantothenic acid Ca salt, 2 mg/L; pyridoxal, 4 mg/L; pyridoxamine dihydrochloride, 4 mg/L; riboflavin, 2 mg/L; thiamin hydrochloride, 2 mg/L; adenine sulfate, 20 mg/L; guanine hydrochloride, 20 mg/L; CaCl_2_.6H_2_O, 10 mg/L; MnSO_4_, 5 mg/L; (NH_4_)_2_SO_4_.FeSO_4_.6H_2_O, 6 mg/L. The individual amino acids were diluted 100-fold into CDM from stock solutions prepared as follows: L-aspartic acid, 15 g/L in 1 N HCl; L-alanine, 10 g/L in dH_2_O; L-arginine, 10 g/L in 1 N HCl; L-cystine, 5 g/L in 1 N HCl; glycine, 10 g/L in dH_2_O; L-glutamic acid, 15 g/L in 1 N HCl; L-histidine, 10 g/L in 1 N HCl; L-isoleucine, 15 g/L in 1 M NH_4_OH; L-lysine, 10 g/L in 1 N HCl; L-leucine, 15 g/L in 1 N HCl; L-methionine, 10 g/L in 1 N HCl; L-phenylalanine, 10 g/L in 1 M NH_4_OH; L-proline, 15 g/L in dH_2_O; L-serine, 10 g/L in dH_2_O; L-threonine, 15 g/L in dH_2_O; L-tryptophan, 10 g/L in 1 N HCl; L-tyrosine, 10 g/L in 1 N HCl; L-valine, 15 g/L in dH_2_O. In all, 2.5 g/L glucose was added for CDMG media. Cultures were cultivated in 250-ml flasks with a 10:1 flask:volume ratio and aerated at 250 rpm at 37 °C. To determine the metabolite excretion profile of various strains, cell-free culture supernatants were analyzed by HPLC for multiple weak acids, acetoin, and sugars as previously described. Briefly, the analysis was performed isocratically at 0.5 mL/min and 65 °C using a Biorad Aminex HPX-87H cation exchange column with 0.13 N H_2_SO_4_ as the mobile phase. The peaks corresponding to various metabolites were identified by their retention time obtained by using genuine standards. Absolute concentrations were determined from calibration curves specific to each metabolite. The excretion rates were calculated from the concentration values at two time points (0 and 3 h), and normalizing the slope against the difference in optical densities corresponding to those time points (data not shown). Ammonia and urea were measured using a kit (R-biopharm) according to the manufacturer’s protocol. Since the metabolite excretion rates are semiquantitative due to only two data points being considered, a qualitative comparison approach between model predictions and experimental measurements was employed in this work.

### Incorporation of regulation in the model

Regulation information for *S. aureus* in terms of differential expression of genes or high/low abundance of the corresponding proteins was accumulated from multiple sources as listed in Supplementary Data 7. While there were numerous frameworks developed to regulated metabolic flux in a genome-scale model previously,^72^ our condition-specific and mutant-specific repressions were incorporated using a “valve” approach similar to several other researchers.^89,90^ Gene-Protein-Reaction (GPR) Boolean relationships for each of the genes were used to determine the corresponding reactions to be regulated in model simulations in different conditions. If a reaction in catalyzed by multiple isozymes, the reaction was only suppressed if all the isozymes were downregulated in a certain condition. For a reaction catalyzed by multiple subunit proteins, it was suppressed if any of the genes responsible for a subunit was downregulated. To simulate the condition- and mutant-specific repressions, the allowable flux ranges were limited to a fraction of their maximum wild-type flux range. To assess the effect of the level of repression, we performed a sensitivity analysis using the repression effect simulating 10, 25, 50, and 90% of maximum wild-type flux space.

### Reporting summary

Further information on research design is available in the Nature Research Reporting Summary linked to this article.

## Supplementary information

Supplementary Data 7

Supplementary Data 8

Supplementary Data 9

Supplementary Information 1

Supplementary Data 1

Supplementary Data 2

Supplementary Data 3

Supplementary Data 4

Supplementary Data 5

Supplementary Data 6

reporting summary

Dataset 1

## Data Availability

All data generated or analyzed during this study are included in this published article and its supplementary information files.

## Code availability

All computer codes used to analyze data during this study are available at a public github repository at 10.5281/zenodo.3518501 under GNU General Public License v3.0.
