# Supplementary material for: An integrated computational and experimental study to investigate Staphylococcus aureus metabolism
Source: NPJ Syst Biol Appl. 2020 Jan 30;6:3. doi: 10.1038/s41540-019-0122-3 (PMC6992624; doi:10.1038/s41540-019-0122-3)
Supplement: Supplementary file 4 — Supplementary Information 1 [file 41540_2019_122_MOESM4_ESM.docx]

**Supplementary Information 1**

**An integrated computational and experimental study to investigate *Staphylococcus aureus* metabolism**

Mohammad Mazharul Islam^1^, Vinai C. Thomas^2^, Matthew Van Beek^1^, Jong-Sam Ahn^2^, Abdulelah A. Alqarzaee^2^, Chunyi Zhou^2^, Paul D. Fey^2^, Kenneth W. Bayles^2^, and Rajib Saha^1*^

^1^Department of Chemical and Biomolecular Engineering, University of Nebraska-Lincoln

^2^Center for Staphylococcal Research, Department of Pathology and Microbiology, University of Nebraska Medical Center

*Corresponding author:

Rajib Saha

Assistant Professor

Chemical and Biomolecular Engineering

University of Nebraska-Lincoln

Lincoln, NE-68588, USA

Email: [rsaha2@unl.edu](mailto:rsaha2@unl.edu)

1. **Strategies for fixing thermodynamically infeasible cycles**

To fix the thermodynamically infeasible cycles in the models, three distinct cases were addressed.

**Case 1: Duplicate reactions that run in opposite direction**

In this case the model contains duplicates of the same reaction, often one being irreversible and one being reversible. The cycle can be broken by removing or turning off one of the reactions, usually the irreversible one if no concrete thermodynamic information is available.

Example:

Phosphoglycerate dehydrogenase (PGCD): nad_c[c] + 3pg_c[c] -> h_c[c] + nadh_c[c] + 3php_c[c]

and

Phosphoglycerate dehydrogenase reversible (PGCDr): nad_c[c] + 3pg_c[c] <=> h_c[c] + nadh_c[c] + 3php_c[c]

Solution:


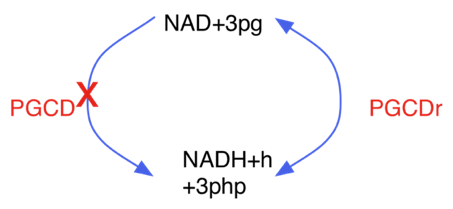
Turn off PGCD. Kept directionality of PGCDr as reversible.

Figure S1: Example of fixing cycles involving duplicate reactions.

**Case II: Lumped reactions**

In this case, multiple reactions in a pathway are lumped together to represent the overall conversion. If both the individual reactions and the lumped reaction are present in the model, they can potentially create thermodynamically infeasible cycles. The cycle can be broken by removing or turning off the lumped reaction and assigning proper annotation information to the individual reactions.

Example:

Aconitase (ACONT): cit_c[c] -> icit_c[c]

Aconitase (half-reaction A, Citrate hydro-lyase, ACONTa): cit_c[c] <=> h2o_c[c] + acon-C_c[c]

Aconitase (half-reaction B, Isocitrate hydro-lyase, ACONTb): icit_c[c] <=> h2o_c[c] + acon-C_c[c]

Solution:

The lumped reaction (ACONT) can be turned off.

Figure S2: Example of fixing cycles involving lumped reactions.

**Case III: Cofactor specificity**

In this case, the same biochemical conversion is carried out by different cofactors in the model, while in reality the organism only uses one of the cofactors. If the cofactor specificity information is available, the reaction with non-specific cofactor can be removed or turned off.

Example:

D-Ribitol-5-phosphate NAD 2-oxidoreductase (DR1ORx ): nad_c[c] + dr5p[c] <=> h_c[c] + nadh_c[c] + ru5p-D_c[c]

and

D-Ribitol-5-phosphate NADP 2-oxidoreductase (DR1ORy): nadp_c[c] + dr5p[c] <=> h_c[c] + nadph_c[c] + ru5p-D_c[c]

both catalyzes the conversion of D-Ribitol-5-phosphate to Ribulose-5-phosphate in S. aureus.

Solution:

Upon extensive search for evidence in literature for cofactor specificity of S. aureus for this reaction, DR1ORx was turned off.

Figure S3: Example of fixing cycles involving non-specific cofactors.

**2. Growth behavior of mutants in CDMG and CDM media**


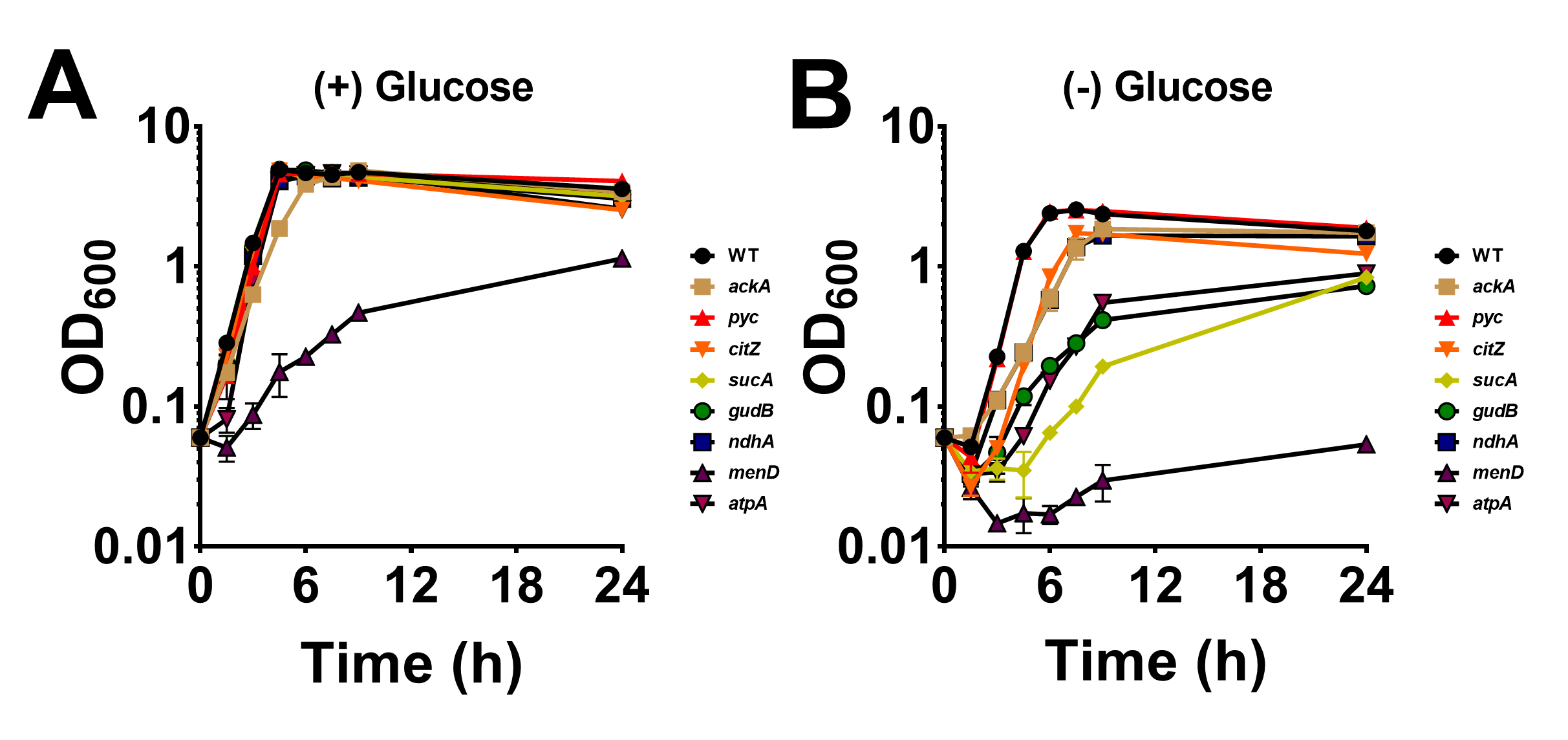


Figure S4: Growth curve for the mutants in (A) CDMG and (B) CDM media. **3. Consensus of *in vivo* essentiality information**

**Consensus on gene essentiality information**

Valentino et. al ^1^ hypothesized that large scale DNA sequencing combined with transposons can determine the essentiality and fitness criticality (non-essential but important for optimal growth) of genes. They determined gene essentiality by finding genes with fewer than 1% of the insertions expected from a random distribution and thereafter found 319 essential genes. The study quantified the effect of a mutation on fitness and found 106 genes that were important to fitness; these genes were defined as genes with 1-10% of the expected insertions. The study tested the mutants in a competitive growth environment with Brain Heart Infusion (BHI) Broth and found 420 essential genes. 30 of the 108 new essential genes were determined to be fitness compromised from before. The study found 91.1% of the previously reported essential genes, such as those by Chaudhuri et. al ^2^, to be essential. This suggests that this is an effective method for determining essential reactions. Of the remaining 8.9%, many were close to the essential cutoff, or were very far from being essential, suggesting that the results from the previous studies were inconsistent. One reason can be the presence of different false-positives within the different methods; and the 1% cutoff appears to have been arbitrarily determined, and genes important to fitness but not essential may fall within this cutoff. This work presumably incorporated true positives that were not included in Chaudhuri et. al ^2^ because Chaudhuri’s analysis made it hard to detect and classify essentiality in small genes or genes with few restriction sites. In general, Valentino was probably more accurate because they used 70,000 inserts compared to only about 350.

Santiago et. al ^3^ hypothesized that gene essentiality derived from transposon libraries can be affected by the high temperatures used to remove the plasmid delivery vehicle. As a result, previous methods found genes that were essential at high temperatures and/or at normal temperatures. They used a different methodology and was able to determine essentiality at 23°C, 30°C, 37°C, and 43°C. Santiago’s list of essential genes was determined at 30°C, instead of 37°C ^1^. Santiago had more inserts than Valentino (690,000 compared to 70,000), and also used a different methodology to determine essentiality, EL-ARTIST, instead of an arbitrary 1% cut-off. The increased number of inserts and EL-ARTIST allowed Santiago to determine if a gene was essential, had essential domains, or was non-essential. For the purpose of a metabolic model, the domain essential genes should be considered essential because knocking out the whole gene would kill the bacterium.

**Sources of Error**

There are two systematic sources of false positives in Chaudhuri et. al ^2^ and Valentino et. al ^1^. First, transposons can be incorrectly labeled essential if a polar effect from the transposon affects an essential gene immediately downstream. Second, the plasmid curing step requires high temperatures during a step of the experiment. This causes heat-essential genes to be incorrectly classified as essential genes. Santiago et. al ^3^ had evidence for likely false positive genes created by the polar effect. Santiago was able to upregulate and downregulate genes with transposons, enabling them to determine if a transposon was on an essential gene or just near an essential gene; these transposons were referred to as “erm” and “promoter”, respectively. However, the final data analysis uses a set of transposons referred to as “blunt” that affect only the downstream genes. 18 of the 20 essential genes found using the blunt methodology were found to be immediately upstream of an essential gene. Thus, genes found to be essential in the “blunt” dataset but non-essential in the “erm” and “promoter” sets are likely false positives, even if previous studies ^1,2^ found them to be essential. Santiago et. al ^3^ was unique because they were able to determine essentiality without a high temperature curing step. As a result, the study did not incorrectly label heat-essential genes as essential at 30°C. The study performed a test at 43°C to determine the heat-essential genes. They concluded that genes that were essential at 43°C but not 30°C are likely false positives. Valentino and Santiago randomly added transposons and then sequenced the junctions to determine which transposons remained after a number of generations.

The Nebraska Transposon Mutant Library (NTML) randomly generated transposon mutants and considered a gene to be knocked out if the transposon insertion was close to the 5’ end. NTML dataset was compared to the genes considered domain essential in Santiago et al ^3^. There appeared to be a relationship in which the domain essential genes have lower growth than normal. The average domain essential gene mutants have approximately 0.7 standard deviations of growth less than other mutants. The transposon may not disrupt the essential domain, allowing the gene to produce a slightly less functional protein. One would assume that there may be a relationship between genes considered fitness critical in Valentino et al ^1^ and the mutants with low growth in the mutant growth test. However, after looking at the fitness critical genes in agar conditions versus the growth in our mutant study, there appeared to be no correlation.

**Conclusions**

For metabolic modeling purpose, there are a few takeaways from the above discussion. First, the true list of essential genes may require a combination of many pools of knowledge. Genes found to be essential in any of the three data sets ^1-3^ should be considered essential unless 1) there was a growth mutant, 2) a gene was found to only be essential at 43°C, and 3) a gene was found essential with the blunt promoters but not under the other two methodologies. One exception is that domain essential genes were considered essential for the purposes of our model. This is because the mutant library may have only knocked out a portion of the gene, allowing it to still produce a functional protein. The second takeaway involves the number of fitness compromised mutants found in Valentino et al ^1^ and in the current study. These generally are not found in the *in silico* model; instead, the model generally shows full growth or no growth. This may suggest that some of the upper bounds in the model are too high, and the model may compensate for lost functionality by redirecting more flux through a pathway than possible *in vivo*.

**4. Growmatch Results**

*In silico* essential genes are found by turning off each gene individually and turning off the reaction(s) catalyzed by the gene by following the Boolean logic of the GPR relationships. *In vivo* essential genes were curated from multiple sources ^1-6^. Most of the essential genes were determined by randomly inserting transposons into Staphylococcus aureus and excluding the transposons which remained after growing the cells ^1-3^. An adaptation of data from two sources using antisense RNA was also used to determine essential enzymes and thus essential genes through the Boolean GPR relationships ^4-6^. The procedure for determining gene essentiality from the pool of literature is shown in Figure S4.

Figure S5. The methodology to determine gene-essentiality. The numbers around the boxes represent number of gene accepted/rejected from the essential gene list at different steps.

Genes found to be essential in any of the previous works were considered essential unless there was positive evidence suggesting the gene was non-essential ^1-6^. There were three types of positive evidence. First, mutants that were obtained from Nebraska’s Transposon Mutant Library ^7,8^ were not considered essential. Each individual mutant was grown in a 384-well plate to confirm that the mutant was able to grow. The least-fit mutant grew to an optical density (OD) of about 40% less than the average of the wild type control. As a result, all of these genes with a mutant were considered non-essential. An exception was made if the gene was found to be domain-essential ^3^. This is because the transposon may have inserted in a non-essential part of the gene, allowing a partially functional protein to be formed. Second, if the gene was found to be essential at only 43⁰C, then it is evident that the gene was incorrectly found to be essential in literature because of a high-temperature plasmid curing step in the processes used in the other literature sources ^3^. Third, if the gene was found to be essential using a promoterless transposon insert, but not with promoter-containing methodologies, then the gene is upstream of an essential gene, and other sources found it to be essential due to polar effects that disrupt expression ^3^.

After determining the list of *in vivo* essential genes, the growth and no-growth inconsistencies between experimental observations and the model predictions were reconciled and model performance was improved. Reactions and genes were categorized as G or NG, meaning that growth occurs when the gene or the corresponding reaction(s) was removed, or no growth occurs, respectively. GGs and NGNGs mean the model agrees with experimental evidence. GNGs are knockouts where the model predicts growth that does not occur in experiments, suggesting the model has spurious extra functionality; NGGs are knockouts upon which the model predicts no growth while experiments predict growth, suggesting the model lacks certain functionality. Growmatch is an optimization-based framework used to resolve metabolic models’ growth predictions with experimental evidence^9^. Growmatch consists of two algorithms: GrowmatchNGG and GrowMatchGNG.

**GrowmatchNGG:**

GrowmatchNGG was used to resolve NGGs. The algorithm turns off an NGG gene, and then maximizes growth by adding the minimal amount of reactions (minimum one to maximum four was allowed in this work) from a database of reactions. These solutions come from three sources: 1) The backwards directions of irreversible reactions (because the reversibility of the reaction may be uncertain) 2) Transport reactions of metabolites ( either diffusion through the cell membrane or via a non-specific transporter), and 3) The reactions from taxonomically similar organisms (*E. coli* and *B. subtilis*) from the BIGG database ^10^. Growmatch suggested various solutions (a set of one to four reactions) to solve most of the NGGs. Solutions were added one by one and checked to ensure they do not invalidate any NGNGs or create any new thermodynamically infeasible reactions cycles. Solutions were prioritized if they resolved NGGs in the central metabolism or amino acid biosynthesis. The rest of the solutions were arranged in order of increasing ranks. The reactions were ranked from one (the most likely) to three (the least likely). Database reactions from *Staphylococcus aureus* models and the backwards direction of irreversible reactions were given a ranking of one. Reactions from the phylum Firmicutes were given a ranking of two 2. Reactions from other bacteria were given a ranking of three 3.

Table 1: Ranking scheme for GrowMatchNGG solutions.

| Rank | Origin | Rationale |
| --- | --- | --- |
| 1 | *S. aureus* or changing direction | The values of ΔG have a low certainty in the cell, so the reactions could be reversible. |
| 2 | Phylum Firmicutes | Same lower taxonomic group, secondary functions predicted in literature |
| 3 | Phylum Firmicutes | Same lower taxonomic group, secondary functions not predicted |
| 4 | Other Bacteria | Same higher taxonomic group |

After checking to ensure the solutions did not invalidate NGNGs or create any new thermodynamically infeasible cycles, one solution for each NGG was added to the model.

**GrowmatchGNG :**

GrowmatchGNG was used to suggest reactions or reaction directions to remove to resolve GNGs. GrowmatchGNG turns off a GNG gene and attempts to minimize the maximum growth by turning off one or multiple reactions (the candidate solutions). GrowMatchGNG produces multiple solutions that can resolve the GNG. In order to minimize the solution space for two reaction knockouts, reactions from iSB619 were not considered for removal. This was justified because the reactions in iSB619 had a gene associated with the reaction or a rationale for each reaction ^11^. The solutions were pruned by ensuring they did not violate any GGs.

**5.** **Incorporation of conditional regulations in the model**

A major regulatory system that was incorporated in the model was the carbon catabolite repression, which is a well-studied global regulatory process in low-GC Gram-positive bacteria in the presence of a preferred carbon source that induces the repression of genes involved in the metabolism of alternative carbon sources ^12^. CcpA, the carbon catabolite control protein*,* is known to repress genes involved in the utilization of amino acids as alternative carbon sources in the presence of glucose^13^. In addition, SrrAB and Rex-dependent transcriptional regulation are prominent driving forces of metabolic flux through respiratory metabolism that was integrated into the model^14-16^. Furthermore, mutant-specific repression of respiration, histidine and ornithine metabolism, and pyruvate metabolism were imposed on the model for the *menD* mutant^17^.

Among the eight mutants, the model-predicted excretion patterns for acetate and lactate in *sucA* and *ackA* mutants agreed with the experimental results of decreased excretion in CDMG media, compared to the wild-type strain. In CDM media, while no significant change in lactate excretion was observed, acetate excretion was decreased in the *ackA* mutant compared to the wild-type strain, due to inactivation of the Pta-AckA pathway. On the other hand, the *sucA* mutant in CDM media showed increased production of acetate due to increased flux space in the Pta-AckA pathway (see Figure 4 in the manuscript). The Pta-AckA pathway is known to supply a major portion of the ATP required for growth ^18^. With the *atpA* gene turned off in the model Pta-AckA pathway supplied most of the ATP demand, which increased the acetate production in CDMG media for the *atpA* mutant compared to the wild-type. However, in CDM media, the model could not sustain the ATP maintenance demand of the *atpA* mutant and therefore, did not produce any acetate. In CDMG media, the model-predicted excretion profile for urea in all of the mutants matched with the experimental observations. In CDM media, the model predictions of higher urea excretion compared to the wild-type strain agreed with the experimental observations for *pyc*, *gudB*, *ndhA*, and *menD* mutants. Similar to the experimental results, excretion of ammonia was predicted by the model in all mutants when glucose was absent (CDM media). These correct predictions can be attributed to the deamination of the amino acids consumed in CDM media when the cell adapts to amino acids due to CcpA-mediated control of amino acid metabolism.

The incorporation of regulatory information improved the predictive capabilities of other mutants. For example, incorporation of regulation based on the Rex and SrrAB repressors' effect on central carbon metabolism allowed the model to correctly simulate the oxygen deprivation in the model, which, in turn, resulted in correct predictions of decreased acetate excretion by the *ndhA* mutant in both CDM and CDMG media. Rex and SrrAB-mediated repression of pyruvate formate lyase (PFLr), alcohol dehydrogenase (ACALD, ALDD2x) and other pathways downstream of pyruvate shifted carbon flux away from the acetate production. At the same time, the flux space for lactate dehydrogenase (LDH) widened, which allowed for more lactate excretion in the CDMG media. In the *menD* mutant, mutant-specific regulation information from Kohler *et al* ^17^ resulted in the correct prediction of lactate and acetate excretion. A mutation in *menD* or any other gene in the menaquinone biosynthesis pathway resulted in weakened respiratory functions and emulated anaerobic condition in the cell, which in turn caused a significant increase in the excretion of lactate in CDMG media. However, although the respiratory functions were downregulated in CDM media (apparent from the shrinkage of the flux space), there was no change in acetate excretion compared to the wild-type strain. In CDMG media, the conversion of pyruvate to oxaloacetate by pyruvate carboxylase was not active in the wild-type model. A small amount of phosphoenol pyruvate was converted to oxaloacetate (via PEPC), which was then used in the conversion of glutamate to aspartate. However, since no convincing evidence for phosphoenol pyruvate carboxylase was found in *S. aureus*, PEPC was removed. This refinement shifted carbon flux through pyruvate carboxylase in the wild-type model and also resulted in correct model prediction of acetate excretion in CDMG media when pyruvate carboxylase was turned off.

While the incorporation of the CcpA, Rex and SrrAB regulations was critical in capturing the physiological behavior of *S. aureus* by the model, it should be noted that there are still gaps in our knowledge about the quantitative repression effect on the reaction fluxes in the presence of these regulators. For example, in CDMG media, ammonia production was not predicted in the *menD*, *atpA,* and *sucA* mutants by the model, which was observed experimentally. However, upon further investigation, it was observed that relaxing the repressions of reaction fluxes that were imposed on the model due to CcpA, Rex, and SrrAB regulators, the discrepancies were removed. In CDMG media, the *citZ* mutant correctly predicted the excretion pattern of acetate because, with the reduced flux space for the TCA cycle reactions, more carbon could be directed to the Pta-AckA pathway. However, in the CDM media, when amino acids were the primary source of carbon, deletion of the *citZ* gene did change the model predicted flux space in the Pta-AckA pathway, and hence couldn’t capture the decrease in acetate excretion rate. In the *pyc* mutant, carbon flux to oxaloacetate was directed through malate dehydrogenase (MDH3) in the model, which involved the consumption of menaquinone produced by cytochrome oxidase BD. When the *pyc* gene was active, the same conversion was mediated through malic enzyme (ME1) and the pyruvate carboxylase (PC). However, since the model could accommodate the metabolic shift in both the wild-type and *pyc* mutant, no change in the excretion rate of acetate or lactate was observed. Also, while the CcpA repression was active, the deletion of the *gudB* gene in the model did not have any effect on the lactate and acetate excretion profiles in CDMG media. In CDM media, the model prediction for no lactate production was consistent with experimental observations. However, but no increase in acetate production was predicted by the model, which was observed in the experiment. Also, the model predicted a lower urea production rate in the *atpA* mutant compared to the wild-type strain, while it was higher in our experiments. Also, no urea excretion was observed in the *citZ* mutant in our experiments, but model predicted urea excretion at the same rate as the wild-type strain. The reason for these inconsistencies could be the lack of a complete understanding of the regulatory processes that affects the relationship between amino acid catabolism, urea cycle, TCA cycle and pyruvate metabolism.

**6.** **Estimation of biomass composition**

The protocol outlined by Thiele *et al* 2010^19^ was followed when developing the biomass equation according to experimental measurements of macromolecular composition^20^ and transcriptomic data^21^. Previous metabolic reconstructions of *S. aureus*^11,20,22,23^ used biomass equations similar to that of *Bacillus subtilis*^24^ and *Escherichia coli*^25^, with additional adjustments to accommodate lipid, cofactors, and other molecular compositions based on intracellular metabolomic data. *Bacillus subtilis* and *Escherichia coli* are not phylogenetically very close to *S. aureus^26^*, and only major macromolecules and components needed for the survival of the organism^19^, not necessarily other intracellular metabolites, even if they are measured, should be part of the biomass equation^19^. To address this issue, our model excludes biomass precursors that do not have either experimental measurements or any literature evidence of synthesis in *S. aureus*. For example, *S. aureus* lacks an identifiable polyamine biosynthetic pathway and therefore cannot produce putrescine^27,28^. In addition, phosphatidylethanolamine is not produced in *S. aureus^29,30^*. Therefore, the non-*S. aureus*-specific reactions and biomass precursors (for example, putrescine, phosphatydilethanolamine, phosphatidylglycine, phosphatidylleucine phosphatidylalanine etc.) were removed from the model. To recalculate the coefficient for each of the biomass precursors, the lost biomass due to removal of non-*S.aureus*-specific component was calculated. Then the coefficients were normalized to account for the lost mass using the equation below. See Supplementary Table 1 for the detailed list of the biomass precursors.

$$New coefficient= \frac{total biomass*old coefficient}{biomass after removal of precursors}$$

**Supplementary Data:**

# Supplementary Data 1: Biomass composition.

# Supplementary Data 2: Fixed reactions imbalanced in carbon, hydrogen and oxygen.

# Supplementary Data 3: All the reactions turned off or directionality changed or removed as a duplicate during model curation steps.

# Supplementary Data 4: Metabolite information.

# Supplementary Data 5: NTML mutant growth data and statistical analysis.

# Supplementary Data 6: Gene essentiality information, comparison of model and experimental essentiality results, GNG tables, Growmatch results, and rejected Growmatch suggestions.

# Supplementary Data 7: Regulations and repressions imposed on the model.

# Supplementary Data 8: Model predictions on utilization of different carbon sources and comparison with BIOLOG experimental results.

# Supplementary Data 9: Growth medium definition.

# Dataset 1: The *S. aureus* USA300_FPR3757 metabolic model (iSA863) in systems biology markup language format.

**References:**

1 Valentino, M. D. *et al.* Genes contributing to Staphylococcus aureus fitness in abscess- and infection-related ecologies. *Mbio* **5**, e01729-01714, doi:10.1128/mBio.01729-14 (2014).

2 Chaudhuri, R. R. *et al.* Comprehensive identification of essential Staphylococcus aureus genes using Transposon-Mediated Differential Hybridisation (TMDH). *BMC Genomics* **10**, 291, doi:10.1186/1471-2164-10-291 (2009).

3 Santiago, M. *et al.* A new platform for ultra-high density Staphylococcus aureus transposon libraries. *BMC Genomics* **16**, 252, doi:10.1186/s12864-015-1361-3 (2015).

4 Lee, D. S. *et al.* Comparative genome-scale metabolic reconstruction and flux balance analysis of multiple Staphylococcus aureus genomes identify novel antimicrobial drug targets. *J Bacteriol* **191**, 4015-4024, doi:10.1128/JB.01743-08 (2009).

5 Forsyth, R. A. *et al.* A genome-wide strategy for the identification of essential genes in Staphylococcus aureus. *Molecular Microbiology* **43**, 1387-1400, doi:DOI 10.1046/j.1365-2958.2002.02832.x (2002).

6 Ji, Y. D. *et al.* Identification of critical staphylococcal genes using conditional phenotypes generated by antisense RNA. *Science* **293**, 2266-2269, doi:DOI 10.1126/science.1063566 (2001).

7 Fey, P. D. *et al.* A genetic resource for rapid and comprehensive phenotype screening of nonessential Staphylococcus aureus genes. *Mbio* **4**, e00537-00512, doi:10.1128/mBio.00537-12 (2013).

8 Bae, T., Glass, E. M., Schneewind, O. & Missiakas, D. Generating a collection of insertion mutations in the Staphylococcus aureus genome using bursa aurealis. *Methods Mol Biol* **416**, 103-116, doi:10.1007/978-1-59745-321-9_7 (2008).

9 Kumar, V. S. & Maranas, C. D. GrowMatch: an automated method for reconciling in silico/in vivo growth predictions. *PLoS computational biology* **5**, e1000308, doi:10.1371/journal.pcbi.1000308 (2009).

10 Schellenberger, J., Park, J. O., Conrad, T. M. & Palsson, B. O. BiGG: a Biochemical Genetic and Genomic knowledgebase of large scale metabolic reconstructions. *BMC bioinformatics* **11**, 213, doi:10.1186/1471-2105-11-213 (2010).

11 Becker, S. A. & Palsson, B. O. Genome-scale reconstruction of the metabolic network in Staphylococcus aureus N315: an initial draft to the two-dimensional annotation. *BMC microbiology* **5**, 8, doi:10.1186/1471-2180-5-8 (2005).

12 Leiba, J. *et al.* A novel mode of regulation of the Staphylococcus aureus catabolite control protein A (CcpA) mediated by Stk1 protein phosphorylation. *J Biol Chem* **287**, 43607-43619, doi:10.1074/jbc.M112.418913 (2012).

13 Seidl, K. *et al.* Effect of a glucose impulse on the CcpA regulon in Staphylococcus aureus. *BMC microbiology* **9**, 95, doi:10.1186/1471-2180-9-95 (2009).

14 Kinkel, T. L., Roux, C. M., Dunman, P. M. & Fang, F. C. The Staphylococcus aureus SrrAB two-component system promotes resistance to nitrosative stress and hypoxia. *Mbio* **4**, e00696-00613, doi:10.1128/mBio.00696-13 (2013).

15 Pagels, M. *et al.* Redox sensing by a Rex-family repressor is involved in the regulation of anaerobic gene expression in Staphylococcus aureus. *Mol Microbiol* **76**, 1142-1161, doi:10.1111/j.1365-2958.2010.07105.x (2010).

16 Liu, X. *et al.* Redox-sensing regulator Rex regulates aerobic metabolism, morphological differentiation, and avermectin production in Streptomyces avermitilis. *Sci Rep* **7**, 44567, doi:10.1038/srep44567 (2017).

17 Kohler, C. *et al.* A defect in menadione biosynthesis induces global changes in gene expression in Staphylococcus aureus. *J Bacteriol* **190**, 6351-6364, doi:10.1128/JB.00505-08 (2008).

18 Sadykov, M. R. *et al.* Inactivation of the Pta-AckA pathway causes cell death in Staphylococcus aureus. *J Bacteriol* **195**, 3035-3044, doi:10.1128/JB.00042-13 (2013).

19 Thiele, I. & Palsson, B. O. A protocol for generating a high-quality genome-scale metabolic reconstruction. *Nat Protoc* **5**, 93-121, doi:10.1038/nprot.2009.203 (2010).

20 Heinemann, M., Kummel, A., Ruinatscha, R. & Panke, S. In silico genome-scale reconstruction and validation of the Staphylococcus aureus metabolic network. *Biotechnology and bioengineering* **92**, 850-864, doi:10.1002/bit.20663 (2005).

21 Carvalho, S. M., de Jong, A., Kloosterman, T. G., Kuipers, O. P. & Saraiva, L. M. The Staphylococcus aureus alpha-Acetolactate Synthase ALS Confers Resistance to Nitrosative Stress. *Frontiers in microbiology* **8**, 1273, doi:10.3389/fmicb.2017.01273 (2017).

22 Bosi, E. *et al.* Comparative genome-scale modelling of Staphylococcus aureus strains identifies strain-specific metabolic capabilities linked to pathogenicity. *Proc Natl Acad Sci U S A* **113**, E3801-3809, doi:10.1073/pnas.1523199113 (2016).

23 Seif, Y. *et al.* A computational knowledge-base elucidates the response of Staphylococcus aureus to different media types. *PLoS computational biology* **15**, e1006644, doi:10.1371/journal.pcbi.1006644 (2019).

24 Henry, C. S., Zinner, J. F., Cohoon, M. P. & Stevens, R. L. iBsu1103: a new genome-scale metabolic model of Bacillus subtilis based on SEED annotations. *Genome Biol* **10**, R69, doi:10.1186/gb-2009-10-6-r69 (2009).

25 Feist, A. M. *et al.* A genome-scale metabolic reconstruction for Escherichia coli K-12 MG1655 that accounts for 1260 ORFs and thermodynamic information. *Mol Syst Biol* **3**, 121, doi:10.1038/msb4100155 (2007).

26 Lamers, R. P. *et al.* Phylogenetic relationships among Staphylococcus species and refinement of cluster groups based on multilocus data. *Bmc Evol Biol* **12**, 171, doi:10.1186/1471-2148-12-171 (2012).

27 Harper, L. *et al.* Staphylococcus aureus Responds to the Central Metabolite Pyruvate To Regulate Virulence. *Mbio* **9**, doi:10.1128/mBio.02272-17 (2018).

28 Joshi, G. S., Spontak, J. S., Klapper, D. G. & Richardson, A. R. Arginine catabolic mobile element encoded speG abrogates the unique hypersensitivity of Staphylococcus aureus to exogenous polyamines. *Mol Microbiol* **82**, 9-20, doi:10.1111/j.1365-2958.2011.07809.x (2011).

29 Oku, Y., Kurokawa, K., Ichihashi, N. & Sekimizu, K. Characterization of the Staphylococcus aureus mprF gene, involved in lysinylation of phosphatidylglycerol. *Microbiology* **150**, 45-51, doi:10.1099/mic.0.26706-0 (2004).

30 Sohlenkamp, C. & Geiger, O. Bacterial membrane lipids: diversity in structures and pathways. *FEMS microbiology reviews* **40**, 133-159, doi:10.1093/femsre/fuv008 (2016).
