# Supplementary material for: An integrated computational and experimental study to investigate Staphylococcus aureus metabolism
Source: NPJ Syst Biol Appl. 2020 Jan 30;6:3. doi: 10.1038/s41540-019-0122-3 (PMC6992624; doi:10.1038/s41540-019-0122-3)
Supplement: Supplementary file 12 — Dataset 1 [file 41540_2019_122_MOESM12_ESM.pdf]

```
<?xml version="1.0" encoding="UTF-8"?>
<sbml xmlns="http://www.sbml.org/sbml/level3/version1/core"
xmlns:fbc="http://www.sbml.org/sbml/level3/version1/fbc/version2" level="3"
version="1" fbc:required="false">
<model id="SAUSA300_FPR3757" name="Staphylococcus aureus subsp.
SAUSA300_FPR3757" fbc:strict = "true">

<listOfUnitDefinitions>
      <unitDefinition id="mmol_per_gDW_per_hr">
            <listOfUnits>
                  <unit kind="mole" exponent="1" scale="-3" multiplier="1"/>
                  <unit kind="gram" exponent="-1" scale="0" multiplier="1"/>
                  <unit kind="second" exponent="-1" scale="0"
multiplier="3600"/>
            </listOfUnits>
      </unitDefinition>
</listOfUnitDefinitions>

<listOfCompartments>
      <compartment metaid="c" id="c" name="Cytosol" constant="false"/>
      <compartment metaid="p" id="p" name="Periplasm" constant="false"/>
      <compartment metaid="e" id="e" name="Extracellular" constant="false"/>
</listOfCompartments>

<listOfSpecies>
      <species metaid="M_1ag3p_c" id="M_1ag3p_c"
name="1__Acyl__sn__glycerol_3__phosphate" compartment="c" charge="-1"
initialConcentration="0" hasOnlySubstanceUnits="false"
boundaryCondition="false" constant="false" fbc:chemicalFormula="C28H54NO9P"/>
      <species metaid="M_1agpe181_c" id="M_1agpe181_c"
name="1__Acyl__sn__glycero__3__phosphoethanolamine (n__C18:1)"
compartment="c" charge="0" initialConcentration="0"
hasOnlySubstanceUnits="false" boundaryCondition="false" constant="false"
fbc:chemicalFormula="C23H46NO7P1"/>
      <species metaid="M_1agpg180_c" id="M_1agpg180_c"
name="1__octadecanoyl__sn__glycero__3__phospho__(1'__sn__glycerol)"
compartment="c" charge="-1" initialConcentration="0"
hasOnlySubstanceUnits="false" boundaryCondition="false" constant="false"
fbc:chemicalFormula="C24H48O9P"/>
      <species metaid="M_1agpg181_c" id="M_1agpg181_c"
name="1__(9Z__octadecenoyl)__sn__glycero__3__phospho__(1'__sn__glycerol)"
compartment="c" charge="-1" initialConcentration="0"
hasOnlySubstanceUnits="false" boundaryCondition="false" constant="false"
fbc:chemicalFormula="C24H46O9P"/>
      <species metaid="M_1aipsg3p_c" id="M_1aipsg3p_c"
name="1__anteisopentadecanoyl__sn__glycerol_3__phosphate" compartment="c"
charge="-1" initialConcentration="0" hasOnlySubstanceUnits="false"
boundaryCondition="false" constant="false" fbc:chemicalFormula=""/>
      <species metaid="M_1ddecg3p_c" id="M_1ddecg3p_c"
name="1__dodecanoyl__sn__glycerol 3__phosphate" compartment="c" charge="-2"
```

```
initialConcentration="0" hasOnlySubstanceUnits="false"
boundaryCondition="false" constant="false" fbc:chemicalFormula="C15H29O7P1"/>
      <species metaid="M_1h2nap_c" id="M_1h2nap_c"
name="1__Hydroxy__2__naphthoate" compartment="c" charge="-1"
initialConcentration="0" hasOnlySubstanceUnits="false"
boundaryCondition="false" constant="false" fbc:chemicalFormula="C11H7O3"/>
      <species metaid="M_1hdec9eg3p_c" id="M_1hdec9eg3p_c"
name="1__hexadec__9__enoyl__sn__glycerol 3__phosphate" compartment="c"
charge="-1" initialConcentration="0" hasOnlySubstanceUnits="false"
boundaryCondition="false" constant="false" fbc:chemicalFormula="C19H35O7P1"/>
      <species metaid="M_1hdecg3p_c" id="M_1hdecg3p_c"
name="1__hexadecanoyl__sn__glycerol 3__phosphate" compartment="c" charge="-1"
initialConcentration="0" hasOnlySubstanceUnits="false"
boundaryCondition="false" constant="false" fbc:chemicalFormula="C19H37O7P1"/>
      <species metaid="M_1ihgly3p_c" id="M_1ihgly3p_c"
name="1__isohexadecanoyl__sn__glycerol_3__phosphate" compartment="c"
charge="-1" initialConcentration="0" hasOnlySubstanceUnits="false"
boundaryCondition="false" constant="false" fbc:chemicalFormula=""/>
      <species metaid="M_1ihsg3p_c" id="M_1ihsg3p_c"
name="1__isoheptadecanoyl__sn__glycerol_3__phosphate" compartment="c"
charge="-1" initialConcentration="0" hasOnlySubstanceUnits="false"
boundaryCondition="false" constant="false" fbc:chemicalFormula=""/>
      <species metaid="M_1ipsg3p_c" id="M_1ipsg3p_c"
name="1__isopentadecanoyl__sn__glycerol_3__phosphate" compartment="c"
charge="-1" initialConcentration="0" hasOnlySubstanceUnits="false"
boundaryCondition="false" constant="false" fbc:chemicalFormula=""/>
      <species metaid="M_1odec11eg3p_c" id="M_1odec11eg3p_c"
name="1__octadec__11__enoyl__sn__glycerol 3__phosphate" compartment="c"
charge="-1" initialConcentration="0" hasOnlySubstanceUnits="false"
boundaryCondition="false" constant="false" fbc:chemicalFormula="C21H39O7P1"/>
      <species metaid="M_1odecg3p_c" id="M_1odecg3p_c"
name="1__octadecanoyl__sn__glycerol 3__phosphate" compartment="c" charge="-2"
initialConcentration="0" hasOnlySubstanceUnits="false"
boundaryCondition="false" constant="false" fbc:chemicalFormula="C21H41O7P1"/>
      <species metaid="M_1p3h5c_c" id="M_1p3h5c_c"
name="L__1__Pyrroline__3__hydroxy__5__carboxylate" compartment="c" charge="-
1" initialConcentration="0" hasOnlySubstanceUnits="false"
boundaryCondition="false" constant="false" fbc:chemicalFormula="C5H6NO3"/>
      <species metaid="M_1pyr5c_c" id="M_1pyr5c_c"
name="1__Pyrroline__5__carboxylate" compartment="c" charge="-1"
initialConcentration="0" hasOnlySubstanceUnits="false"
boundaryCondition="false" constant="false" fbc:chemicalFormula="C5H6NO2"/>
      <species metaid="M_1stsg3p_c" id="M_1stsg3p_c"
name="1__isotetradecanoyl__sn__glycerol_3__phosphate" compartment="c"
charge="-1" initialConcentration="0" hasOnlySubstanceUnits="false"
boundaryCondition="false" constant="false" fbc:chemicalFormula=""/>
      <species metaid="M_1tdec7eg3p_c" id="M_1tdec7eg3p_c"
name="1__tetradec__7__enoyl__sn__glycerol 3__phosphate" compartment="c"
charge="-1" initialConcentration="0" hasOnlySubstanceUnits="false"
boundaryCondition="false" constant="false" fbc:chemicalFormula="C17H31O7P1"/>
      <species metaid="M_1tdecg3p_c" id="M_1tdecg3p_c"
name="1__tetradecanoyl__sn__glycerol 3__phosphate" compartment="c" charge="-
2" initialConcentration="0" hasOnlySubstanceUnits="false"
boundaryCondition="false" constant="false" fbc:chemicalFormula="C17H33O7P1"/>
      <species metaid="M_2a3pp_c" id="M_2a3pp_c"
name="2__Amino__3__phosphonopropanoate" compartment="c" charge="-1"
initialConcentration="0" hasOnlySubstanceUnits="false"
boundaryCondition="false" constant="false" fbc:chemicalFormula="C3H6NO5P"/>
```

<species metaid="M_2agpe141_c" id="M_2agpe141_c"
name="2__Acyl__sn__glycero__3__phosphoethanolamine (n__C14:1)"
compartment="c" charge="0" initialConcentration="0"
hasOnlySubstanceUnits="false" boundaryCondition="false" constant="false"
fbc:chemicalFormula="C19H38NO7P1"/>
<species metaid="M_2agpe160_c" id="M_2agpe160_c"
name="2__Acyl__sn__glycero__3__phosphoethanolamine (n__C16:0)"
compartment="c" charge="0" initialConcentration="0"
hasOnlySubstanceUnits="false" boundaryCondition="false" constant="false"
fbc:chemicalFormula="C21H44NO7P1"/>
<species metaid="M_2agpg160_c" id="M_2agpg160_c"
name="2__Acyl__sn__glycero__3__phosphoglycerol (n__C16:0)" compartment="c"
charge="-1" initialConcentration="0" hasOnlySubstanceUnits="false"
boundaryCondition="false" constant="false" fbc:chemicalFormula="C22H44O9P1"/>
<species metaid="M_2agpg180_c" id="M_2agpg180_c"
name="2__Acyl__sn__glycero__3__phosphoglycerol (n__C18:0)" compartment="c"
charge="-1" initialConcentration="0" hasOnlySubstanceUnits="false"
boundaryCondition="false" constant="false" fbc:chemicalFormula="C24H48O9P1"/>
<species metaid="M_2ahbut_c" id="M_2ahbut_c"
name="(S)__2__Aceto__2__hydroxybutanoate" compartment="c" charge="-1"
initialConcentration="0" hasOnlySubstanceUnits="false"
boundaryCondition="false" constant="false" fbc:chemicalFormula="C6H9O4"/>
<species metaid="M_2aobut_c" id="M_2aobut_c"
name="L__2__Amino__3__oxobutanoate" compartment="c" charge="0"
initialConcentration="0" hasOnlySubstanceUnits="false"
boundaryCondition="false" constant="false" fbc:chemicalFormula="C4H7NO3"/>
<species metaid="M_2bdgsglyc_c" id="M_2bdgsglyc_c"
name="2__beta__D__Glucosyl__sn__glycerol" compartment="c" charge="0"
initialConcentration="0" hasOnlySubstanceUnits="false"
boundaryCondition="false" constant="false" fbc:chemicalFormula=""/>
<species metaid="M_2bdgsglyc_e" id="M_2bdgsglyc_e"
name="2__beta__D__Glucosyl__sn__glycerol_b" compartment="e" charge="0"
initialConcentration="0" hasOnlySubstanceUnits="false"
boundaryCondition="true" constant="false" fbc:chemicalFormula=""/>
<species metaid="M_2cpr5p_c" id="M_2cpr5p_c"
name="1__(2__Carboxyphenylamino)__1__deoxy__D__ribulose 5__phosphate"
compartment="c" charge="-3" initialConcentration="0"
hasOnlySubstanceUnits="false" boundaryCondition="false" constant="false"
fbc:chemicalFormula="C12H13NO9P"/>
<species metaid="M_2dda7p_c" id="M_2dda7p_c"
name="2__Dehydro__3__deoxy__D__arabino__heptonate 7__phosphate"
compartment="c" charge="-3" initialConcentration="0"
hasOnlySubstanceUnits="false" boundaryCondition="false" constant="false"
fbc:chemicalFormula="C7H10O10P"/>
<species metaid="M_2ddg6p_c" id="M_2ddg6p_c"
name="2__Dehydro__3__deoxy__D__gluconate 6__phosphate" compartment="c"
charge="-3" initialConcentration="0" hasOnlySubstanceUnits="false"
boundaryCondition="false" constant="false" fbc:chemicalFormula="C6H8O9P"/>
<species metaid="M_2ddglcn_c" id="M_2ddglcn_c"
name="2__Dehydro__3__deoxy__D__gluconate" compartment="c" charge="-1"
initialConcentration="0" hasOnlySubstanceUnits="false"
boundaryCondition="false" constant="false" fbc:chemicalFormula="C6H9O6"/>
<species metaid="M_2dhp_c" id="M_2dhp_c" name="2__Dehydropantoate"
compartment="c" charge="-1" initialConcentration="0"
hasOnlySubstanceUnits="false" boundaryCondition="false" constant="false"
fbc:chemicalFormula="C6H9O4"/>
<species metaid="M_2dmmq7_c" id="M_2dmmq7_c" name="2
Demethylmenaquinone 7" compartment="c" charge="-2" initialConcentration="0"

hasOnlySubstanceUnits="false" boundaryCondition="false" constant="false" fbc:chemicalFormula="C45H62O2"/>
<species metaid="M_2dr1p_c" id="M_2dr1p_c" name="2__Deoxy__D__ribose 1__phosphate" compartment="c" charge="-2" initialConcentration="0" hasOnlySubstanceUnits="false" boundaryCondition="false" constant="false" fbc:chemicalFormula="C5H9O7P"/>
<species metaid="M_2dr5p_c" id="M_2dr5p_c" name="2__Deoxy__D__ribose 5__phosphate" compartment="c" charge="-2" initialConcentration="0" hasOnlySubstanceUnits="false" boundaryCondition="false" constant="false" fbc:chemicalFormula="C5H9O7P"/>
<species metaid="M_2fe1s_c" id="M_2fe1s_c" name="[2Fe__1S] desulfurated iron__sulfur cluster" compartment="c" charge="0" initialConcentration="0" hasOnlySubstanceUnits="false" boundaryCondition="false" constant="false" fbc:chemicalFormula="SFe2"/>
<species metaid="M_2fe2s_c" id="M_2fe2s_c" name="[2Fe__2S] iron__sulfur cluster" compartment="c" charge="0" initialConcentration="0" hasOnlySubstanceUnits="false" boundaryCondition="false" constant="false" fbc:chemicalFormula="S2Fe2"/>
<species metaid="M_2h3oppan_c" id="M_2h3oppan_c" name="2__Hydroxy__3__oxopropanoate" compartment="c" charge="-1" initialConcentration="0" hasOnlySubstanceUnits="false" boundaryCondition="false" constant="false" fbc:chemicalFormula="C3H3O4"/>
<species metaid="M_2hdec9eg3p_c" id="M_2hdec9eg3p_c" name="2__hexadec__9__enoyl__sn__glycerol 3__phosphate" compartment="c" charge="-1" initialConcentration="0" hasOnlySubstanceUnits="false" boundaryCondition="false" constant="false" fbc:chemicalFormula="C19H36O7P1"/>
<species metaid="M_2hmc_c" id="M_2hmc_c" name="2__Hydroxymuconate" compartment="c" charge="-2" initialConcentration="0" hasOnlySubstanceUnits="false" boundaryCondition="false" constant="false" fbc:chemicalFormula="C6H4O5"/>
<species metaid="M_2hmcnsad_c" id="M_2hmcnsad_c" name="2__Hydroxymuconate semialdehyde" compartment="c" charge="-1" initialConcentration="0" hasOnlySubstanceUnits="false" boundaryCondition="false" constant="false" fbc:chemicalFormula="C6H6O4"/>
<species metaid="M_2hymeph_c" id="M_2hymeph_c" name="2__(Hydroxymethyl)phenol" compartment="c" charge="0" initialConcentration="0" hasOnlySubstanceUnits="false" boundaryCondition="false" constant="false" fbc:chemicalFormula="C7H8O2"/>
<species metaid="M_2ippm_c" id="M_2ippm_c" name="2__Isopropylmaleate" compartment="c" charge="-2" initialConcentration="0" hasOnlySubstanceUnits="false" boundaryCondition="false" constant="false" fbc:chemicalFormula="C7H8O4"/>
<species metaid="M_2kmb_c" id="M_2kmb_c" name="2__keto__4__methylthiobutyrate" compartment="c" charge="-1" initialConcentration="0" hasOnlySubstanceUnits="false" boundaryCondition="false" constant="false" fbc:chemicalFormula="C5H7O3S"/>
<species metaid="M_2maacoa_c" id="M_2maacoa_c" name="2__Methyl__3__acetoacetyl__CoA" compartment="c" charge="-4" initialConcentration="0" hasOnlySubstanceUnits="false" boundaryCondition="false" constant="false" fbc:chemicalFormula="C26H38N7O18P3S"/>
<species metaid="M_2mahmp_c" id="M_2mahmp_c" name="2__Methyl__4__amino__5__hydroxymethylpyrimidine diphosphate" compartment="c" charge="-3" initialConcentration="0" hasOnlySubstanceUnits="false" boundaryCondition="false" constant="false" fbc:chemicalFormula="C6H8N3O7P2"/>
<species metaid="M_2mb2coa_c" id="M_2mb2coa_c" name="trans__2__Methylbut__2__enoyl__CoA" compartment="c" charge="-4"

initialConcentration="0" hasOnlySubstanceUnits="false"
boundaryCondition="false" constant="false"
fbc:chemicalFormula="C26H38N7O17P3S"/>
<species metaid="M_2mbcoa_c" id="M_2mbcoa_c"
name="2__Methylbutanoyl__CoA" compartment="c" charge="-4"
initialConcentration="0" hasOnlySubstanceUnits="false"
boundaryCondition="false" constant="false"
fbc:chemicalFormula="C26H40N7O17P3S"/>
<species metaid="M_2mbdhl_c" id="M_2mbdhl_c"
name="S__(2__Methylbutanoyl)__dihydrolipoamide" compartment="c" charge="0"
initialConcentration="0" hasOnlySubstanceUnits="false"
boundaryCondition="false" constant="false"
fbc:chemicalFormula="C13H25NO2S2"/>
<species metaid="M_2mbutACP_c" id="M_2mbutACP_c"
name="2__methylbutyryl__ACP" compartment="c" charge="-1"
initialConcentration="0" hasOnlySubstanceUnits="false"
boundaryCondition="false" constant="false"
fbc:chemicalFormula="C16H29N2O8PRS"/>
<species metaid="M_2me4p_c" id="M_2me4p_c"
name="2__C__methyl__D__erythritol 4__phosphate" compartment="c" charge="-2"
initialConcentration="0" hasOnlySubstanceUnits="false"
boundaryCondition="false" constant="false" fbc:chemicalFormula="C5H11O7P"/>
<species metaid="M_2mop_c" id="M_2mop_c"
name="2__Methyl__3__oxopropanoate" compartment="c" charge="-1"
initialConcentration="0" hasOnlySubstanceUnits="false"
boundaryCondition="false" constant="false" fbc:chemicalFormula="C4H5O3"/>
<species metaid="M_2mp2coa_c" id="M_2mp2coa_c"
name="2__Methylprop__2__enoyl__CoA" compartment="c" charge="-4"
initialConcentration="0" hasOnlySubstanceUnits="false"
boundaryCondition="false" constant="false"
fbc:chemicalFormula="C25H36N7O17P3S"/>
<species metaid="M_2mpdhl_c" id="M_2mpdhl_c"
name="S__(2__Methylpropanoyl)__dihydrolipoamide" compartment="c" charge="0"
initialConcentration="0" hasOnlySubstanceUnits="false"
boundaryCondition="false" constant="false"
fbc:chemicalFormula="C12H23NO2S2"/>
<species metaid="M_2o3mpt_c" id="M_2o3mpt_c"
name="R__2__Oxo__3__methylpentanoate" compartment="c" charge="-1"
initialConcentration="0" hasOnlySubstanceUnits="false"
boundaryCondition="false" constant="false" fbc:chemicalFormula="C6H9O3"/>
<species metaid="M_2obut_c" id="M_2obut_c" name="2__Oxobutanoate"
compartment="c" charge="-1" initialConcentration="0"
hasOnlySubstanceUnits="false" boundaryCondition="false" constant="false"
fbc:chemicalFormula="C4H5O3"/>
<species metaid="M_2obut_e" id="M_2obut_e" name="2__Oxobutanoate_b"
compartment="e" charge="-1" initialConcentration="0"
hasOnlySubstanceUnits="false" boundaryCondition="true" constant="false"
fbc:chemicalFormula="C4H5O3"/>
<species metaid="M_2ohph_c" id="M_2ohph_c"
name="2__Octaprenyl__6__hydroxyphenol" compartment="c" charge="0"
initialConcentration="0" hasOnlySubstanceUnits="false"
boundaryCondition="false" constant="false" fbc:chemicalFormula="C46H70O2"/>
<species metaid="M_2ombz_c" id="M_2ombz_c"
name="2__Octaprenyl__6__methoxy__1,4__benzoquinone" compartment="c"
charge="0" initialConcentration="0" hasOnlySubstanceUnits="false"
boundaryCondition="false" constant="false" fbc:chemicalFormula="C47H70O3"/>
<species metaid="M_2ommb_c" id="M_2ommb_c"
name="2__Octaprenyl__3__methyl__6__methoxy__1,4__benzoquinone"

compartment="c" charge="0" initialConcentration="0"
hasOnlySubstanceUnits="false" boundaryCondition="false" constant="false"
fbc:chemicalFormula="C48H72O3"/>
<species metaid="M_2oph_c" id="M_2oph_c" name="2__Octaprenylphenol"
compartment="c" charge="0" initialConcentration="0"
hasOnlySubstanceUnits="false" boundaryCondition="false" constant="false"
fbc:chemicalFormula="C46H70O"/>
<species metaid="M_2p4c2me_c" id="M_2p4c2me_c"
name="2__phospho__4__(cytidine 5'__diphospho)__2__C__methyl__D__erythritol"
compartment="c" charge="-4" initialConcentration="0"
hasOnlySubstanceUnits="false" boundaryCondition="false" constant="false"
fbc:chemicalFormula="C14H22N3O17P3"/>
<species metaid="M_2pg_c" id="M_2pg_c" name="D__Glycerate 2__phosphate"
compartment="c" charge="-3" initialConcentration="0"
hasOnlySubstanceUnits="false" boundaryCondition="false" constant="false"
fbc:chemicalFormula="C3H4O7P"/>
<species metaid="M_2pglyc_c" id="M_2pglyc_c" name="2__Phosphoglycolate"
compartment="c" charge="-3" initialConcentration="0"
hasOnlySubstanceUnits="false" boundaryCondition="false" constant="false"
fbc:chemicalFormula="C2H2O6P"/>
<species metaid="M_2sephchc_c" id="M_2sephchc_c"
name="2__succinyl__5__enolpyruvyl__6__hydroxy__3__cyclohexene__1__carboxylate
" compartment="c" charge="-3" initialConcentration="0"
hasOnlySubstanceUnits="false" boundaryCondition="false" constant="false"
fbc:chemicalFormula="C14H13O9"/>
<species metaid="M_2shchc_c" id="M_2shchc_c"
name="2__Succinyl__6__hydroxy__2,4__cyclohexadiene__1__carboxylate"
compartment="c" charge="-2" initialConcentration="0"
hasOnlySubstanceUnits="false" boundaryCondition="false" constant="false"
fbc:chemicalFormula="C11H10O6"/>
<species metaid="M_3amp_c" id="M_3amp_c" name="3'__AMP" compartment="c"
charge="-2" initialConcentration="0" hasOnlySubstanceUnits="false"
boundaryCondition="false" constant="false"
fbc:chemicalFormula="C10H12N5O7P"/>
<species metaid="M_3amp_e" id="M_3amp_e" name="3'__AMP_b"
compartment="e" charge="-2" initialConcentration="0"
hasOnlySubstanceUnits="false" boundaryCondition="true" constant="false"
fbc:chemicalFormula="C10H12N5O7P"/>
<species metaid="M_3c1ht_c" id="M_3c1ht_c"
name="3__Carboxy__1__hydroxypropyl__ThPP" compartment="c" charge="-2"
initialConcentration="0" hasOnlySubstanceUnits="false"
boundaryCondition="false" constant="false"
fbc:chemicalFormula="C16H22N4O10P2S"/>
<species metaid="M_3c2hmp_c" id="M_3c2hmp_c"
name="3__Carboxy__2__hydroxy__4__methylpentanoate" compartment="c" charge="-
2" initialConcentration="0" hasOnlySubstanceUnits="false"
boundaryCondition="false" constant="false" fbc:chemicalFormula="C7H10O5"/>
<species metaid="M_3c3hmp_c" id="M_3c3hmp_c"
name="3__Carboxy__3__hydroxy__4__methylpentanoate" compartment="c" charge="-
2" initialConcentration="0" hasOnlySubstanceUnits="false"
boundaryCondition="false" constant="false" fbc:chemicalFormula="C7H10O5"/>
<species metaid="M_3c4mop_c" id="M_3c4mop_c"
name="3__Carboxy__4__methyl__2__oxopentanoate" compartment="c" charge="-2"
initialConcentration="0" hasOnlySubstanceUnits="false"
boundaryCondition="false" constant="false" fbc:chemicalFormula="C7H8O5"/>
<species metaid="M_3cmp_c" id="M_3cmp_c" name="3'__CMP" compartment="c"
charge="-2" initialConcentration="0" hasOnlySubstanceUnits="false"
boundaryCondition="false" constant="false" fbc:chemicalFormula="C9H12N3O8P"/>

<species metaid="M_3dhq_c" id="M_3dhq_c" name="3__Dehydroquinate"
compartment="c" charge="-1" initialConcentration="0"
hasOnlySubstanceUnits="false" boundaryCondition="false" constant="false"
fbc:chemicalFormula="C7H9O6"/>
<species metaid="M_3dhsk_c" id="M_3dhsk_c" name="3__Dehydroshikimate"
compartment="c" charge="-1" initialConcentration="0"
hasOnlySubstanceUnits="false" boundaryCondition="false" constant="false"
fbc:chemicalFormula="C7H7O5"/>
<species metaid="M_3g12dgr_SA2_c" id="M_3g12dgr_SA2_c"
name="3__D__Glucosyl__1,2__diacylglycerol" compartment="c" charge="0"
initialConcentration="0" hasOnlySubstanceUnits="false"
boundaryCondition="false" constant="false"
fbc:chemicalFormula="C1860H3615O292"/>
<species metaid="M_3gmp_c" id="M_3gmp_c" name="3'__GMP" compartment="c"
charge="-2" initialConcentration="0" hasOnlySubstanceUnits="false"
boundaryCondition="false" constant="false"
fbc:chemicalFormula="C10H12N5O8P"/>
<species metaid="M_3gmp_e" id="M_3gmp_e" name="3'__GMP_b"
compartment="e" charge="-2" initialConcentration="0"
hasOnlySubstanceUnits="false" boundaryCondition="true" constant="false"
fbc:chemicalFormula="C10H12N5O8P"/>
<species metaid="M_3gmp_p" id="M_3gmp_p" name="3'__GMP" compartment="p"
charge="-2" initialConcentration="0" hasOnlySubstanceUnits="false"
boundaryCondition="false" constant="false"
fbc:chemicalFormula="C10H12N5O8P"/>
<species metaid="M_3h3mop_c" id="M_3h3mop_c"
name="(R)__3__Hydroxy__3__methyl__2__oxopentanoate" compartment="c" charge="-
1" initialConcentration="0" hasOnlySubstanceUnits="false"
boundaryCondition="false" constant="false" fbc:chemicalFormula="C6H9O4"/>
<species metaid="M_3h5ox" id="M_3h5ox"
name="1__O__(alk__1__enyl)__2__acyl__sn__glycero__3__phosphoethanolamine"
compartment="c" charge="0" initialConcentration="0"
hasOnlySubstanceUnits="false" boundaryCondition="false" constant="false"
fbc:chemicalFormula="C8H14NO7PR2"/>
<species metaid="M_3hadpcoa_c" id="M_3hadpcoa_c"
name="(3S)__3__Hydroxyadipyl__CoA" compartment="c" charge="-5"
initialConcentration="0" hasOnlySubstanceUnits="false"
boundaryCondition="false" constant="false"
fbc:chemicalFormula="C27H39N7O20P3S"/>
<species metaid="M_3hbcoa_c" id="M_3hbcoa_c"
name="(S)__3__Hydroxybutanoyl__CoA" compartment="c" charge="-4"
initialConcentration="0" hasOnlySubstanceUnits="false"
boundaryCondition="false" constant="false"
fbc:chemicalFormula="C25H38N7O18P3S"/>
<species metaid="M_3hdcoa_c" id="M_3hdcoa_c"
name="(S)__3__Hydroxydecanoyl__CoA" compartment="c" charge="-4"
initialConcentration="0" hasOnlySubstanceUnits="false"
boundaryCondition="false" constant="false"
fbc:chemicalFormula="C31H50N7O18P3S"/>
<species metaid="M_3hddcoa_c" id="M_3hddcoa_c"
name="(S)__3__Hydroxydodecanoyl__CoA" compartment="c" charge="-4"
initialConcentration="0" hasOnlySubstanceUnits="false"
boundaryCondition="false" constant="false"
fbc:chemicalFormula="C33H54N7O18P3S"/>
<species metaid="M_3hddecACP_c" id="M_3hddecACP_c"
name="(R)__3__Hydroxydodecanoyl__[acyl__carrier protein]" compartment="c"
charge="0" initialConcentration="0" hasOnlySubstanceUnits="false"

```
boundaryCondition="false" constant="false"
fbc:chemicalFormula="C23H43N2O9PRS"/>
      <species metaid="M_3hdecACP_c" id="M_3hdecACP_c"
name="(R)__3__Hydroxydecanoyl__[acyl__carrier protein]" compartment="c"
charge="-1" initialConcentration="0" hasOnlySubstanceUnits="false"
boundaryCondition="false" constant="false"
fbc:chemicalFormula="C21H39N2O9PRS"/>
      <species metaid="M_3hgmeACP_c" id="M_3hgmeACP_c"
name="3__Hydroxyglutaryl__ACP methyl ester" compartment="c" charge="1"
initialConcentration="0" hasOnlySubstanceUnits="false"
boundaryCondition="false" constant="false"
fbc:chemicalFormula="C17H29N2O11PRS"/>
      <species metaid="M_3hhcoa_c" id="M_3hhcoa_c"
name="(S)__3__Hydroxyhexanoyl__CoA" compartment="c" charge="-4"
initialConcentration="0" hasOnlySubstanceUnits="false"
boundaryCondition="false" constant="false"
fbc:chemicalFormula="C27H42N7O18P3S"/>
      <species metaid="M_3hhdcoa_c" id="M_3hhdcoa_c"
name="(S)__3__Hydroxyhexadecanoyl__CoA" compartment="c" charge="-4"
initialConcentration="0" hasOnlySubstanceUnits="false"
boundaryCondition="false" constant="false"
fbc:chemicalFormula="C37H62N7O18P3S"/>
      <species metaid="M_3hhexACP_c" id="M_3hhexACP_c"
name="(R)__3__Hydroxyhexanoyl__[acyl__carrier protein]" compartment="c"
charge="0" initialConcentration="0" hasOnlySubstanceUnits="false"
boundaryCondition="false" constant="false"
fbc:chemicalFormula="C17H31N2O9PRS"/>
      <species metaid="M_3hmbcoa_c" id="M_3hmbcoa_c"
name="(S)__3__Hydroxy__2__methylbutyryl__CoA" compartment="c" charge="-4"
initialConcentration="0" hasOnlySubstanceUnits="false"
boundaryCondition="false" constant="false"
fbc:chemicalFormula="C26H40N7O18P3S"/>
      <species metaid="M_3hmcat_c" id="M_3hmcat_c"
name="4__Hydroxymethylcatechol" compartment="c" charge="0"
initialConcentration="0" hasOnlySubstanceUnits="false"
boundaryCondition="false" constant="false" fbc:chemicalFormula="C7H8O3"/>
      <species metaid="M_3hmp_c" id="M_3hmp_c"
name="3__Hydroxy__2__methylpropanoate" compartment="c" charge="-1"
initialConcentration="0" hasOnlySubstanceUnits="false"
boundaryCondition="false" constant="false" fbc:chemicalFormula="C4H7O3"/>
      <species metaid="M_3hmrsACP_c" id="M_3hmrsACP_c"
name="(R)__3__Hydroxytetradecanoyl__[acyl__carrier protein]" compartment="c"
charge="0" initialConcentration="0" hasOnlySubstanceUnits="false"
boundaryCondition="false" constant="false"
fbc:chemicalFormula="C25H47N2O9PRS"/>
      <species metaid="M_3hocACP_c" id="M_3hocACP_c"
name="R__3__Hydroxyoctanoyl__acyl__carrier_protein" compartment="c" charge="-
1" initialConcentration="0" hasOnlySubstanceUnits="false"
boundaryCondition="false" constant="false"
fbc:chemicalFormula="C19H35N2O9PRS"/>
      <species metaid="M_3hocoa_c" id="M_3hocoa_c"
name="(S)__3__Hydroxyoctanoyl__CoA" compartment="c" charge="-4"
initialConcentration="0" hasOnlySubstanceUnits="false"
boundaryCondition="false" constant="false"
fbc:chemicalFormula="C29H46N7O18P3S"/>
      <species metaid="M_3hodACP_c" id="M_3hodACP_c"
name="3__Hydroxyoctodecanoyl__ACP" compartment="c" charge="-1"
initialConcentration="0" hasOnlySubstanceUnits="false"
```

boundaryCondition="false" constant="false"
fbc:chemicalFormula="C29H55N2O9PRS"/>
<species metaid="M_3hoddecACP_c" id="M_3hoddecACP_c"
name="10__methyl__3__hydroxy__dodecanoyl__ACP" compartment="c" charge="-1"
initialConcentration="0" hasOnlySubstanceUnits="false"
boundaryCondition="false" constant="false"
fbc:chemicalFormula="C24H45N2O9PRS"/>
<species metaid="M_3hpaACP_c" id="M_3hpaACP_c"
name="R__3__hydroxypalmitoyl__acyl__carrierprotein" compartment="c" charge="-
1" initialConcentration="0" hasOnlySubstanceUnits="false"
boundaryCondition="false" constant="false"
fbc:chemicalFormula="C27H51N2O9PRS"/>
<species metaid="M_3hpmeACP_c" id="M_3hpmeACP_c"
name="3__Hydroxypimeloyl__ACP methyl ester" compartment="c" charge="1"
initialConcentration="0" hasOnlySubstanceUnits="false"
boundaryCondition="false" constant="false"
fbc:chemicalFormula="C19H33N2O11PRS"/>
<species metaid="M_3htdcoa_c" id="M_3htdcoa_c"
name="(S)__3__Hydroxytetradecanoyl__CoA" compartment="c" charge="-4"
initialConcentration="0" hasOnlySubstanceUnits="false"
boundaryCondition="false" constant="false"
fbc:chemicalFormula="C35H58N7O18P3S"/>
<species metaid="M_3ig3p_c" id="M_3ig3p_c"
name="C'__(3__Indolyl)__glycerol 3__phosphate" compartment="c" charge="-2"
initialConcentration="0" hasOnlySubstanceUnits="false"
boundaryCondition="false" constant="false" fbc:chemicalFormula="C11H12NO6P"/>
<species metaid="M_3mbdhl_c" id="M_3mbdhl_c"
name="S__(3__Methylbutanoyl)__dihydrolipoamide" compartment="c" charge="0"
initialConcentration="0" hasOnlySubstanceUnits="false"
boundaryCondition="false" constant="false"
fbc:chemicalFormula="C13H25NO2S2"/>
<species metaid="M_3mcat_c" id="M_3mcat_c" name="3__Methylcatechol"
compartment="c" charge="0" initialConcentration="0"
hasOnlySubstanceUnits="false" boundaryCondition="false" constant="false"
fbc:chemicalFormula="C7H8O2"/>
<species metaid="M_3mehacp_c" id="M_3mehacp_c"
name="5__methyl__hexanoyl__ACP" compartment="c" charge="-1"
initialConcentration="0" hasOnlySubstanceUnits="false"
boundaryCondition="false" constant="false"
fbc:chemicalFormula="C18H33N2O8PRS"/>
<species metaid="M_3mob_c" id="M_3mob_c"
name="3__Methyl__2__oxobutanoate" compartment="c" charge="-1"
initialConcentration="0" hasOnlySubstanceUnits="false"
boundaryCondition="false" constant="false" fbc:chemicalFormula="C5H7O3"/>
<species metaid="M_3mop_c" id="M_3mop_c"
name="(S)__3__Methyl__2__oxopentanoate" compartment="c" charge="-1"
initialConcentration="0" hasOnlySubstanceUnits="false"
boundaryCondition="false" constant="false" fbc:chemicalFormula="C6H9O3"/>
<species metaid="M_3o3pc" id="M_3o3pc"
name="3__oxo__3__phenylpropanoyl__CoA" compartment="c" charge="-4"
initialConcentration="0" hasOnlySubstanceUnits="false"
boundaryCondition="false" constant="false"
fbc:chemicalFormula="C30H38N7O18P3S"/>
<species metaid="M_3odcoa_c" id="M_3odcoa_c" name="3__Oxodecanoyl__CoA"
compartment="c" charge="-4" initialConcentration="0"
hasOnlySubstanceUnits="false" boundaryCondition="false" constant="false"
fbc:chemicalFormula="C31H48N7O18P3S"/>

<species metaid="M_3oddcoa_c" id="M_3oddcoa_c" name="3__Oxododecanoyl__CoA" compartment="c" charge="-4" initialConcentration="0" hasOnlySubstanceUnits="false" boundaryCondition="false" constant="false" fbc:chemicalFormula="C33H52N7O18P3S"/>
<species metaid="M_3oddecACP_c" id="M_3oddecACP_c" name="3__Oxododecanoyl__[acyl__carrier protein]" compartment="c" charge="0" initialConcentration="0" hasOnlySubstanceUnits="false" boundaryCondition="false" constant="false" fbc:chemicalFormula="C23H41N2O9PRS"/>
<species metaid="M_3odecACP_c" id="M_3odecACP_c" name="3__Oxodecanoyl__[acyl__carrier protein]" compartment="c" charge="0" initialConcentration="0" hasOnlySubstanceUnits="false" boundaryCondition="false" constant="false" fbc:chemicalFormula="C21H37N2O9PRS"/>
<species metaid="M_3ohcoa_c" id="M_3ohcoa_c" name="3__Oxohexanoyl__CoA" compartment="c" charge="-4" initialConcentration="0" hasOnlySubstanceUnits="false" boundaryCondition="false" constant="false" fbc:chemicalFormula="C27H40N7O18P3S"/>
<species metaid="M_3ohdcoa_c" id="M_3ohdcoa_c" name="3__Oxohexadecanoyl__CoA" compartment="c" charge="-4" initialConcentration="0" hasOnlySubstanceUnits="false" boundaryCondition="false" constant="false" fbc:chemicalFormula="C37H60N7O18P3S"/>
<species metaid="M_3ohexACP_c" id="M_3ohexACP_c" name="3__Oxohexanoyl__[acyl__carrier protein]" compartment="c" charge="0" initialConcentration="0" hasOnlySubstanceUnits="false" boundaryCondition="false" constant="false" fbc:chemicalFormula="C17H29N2O9PRS"/>
<species metaid="M_3omrsACP_c" id="M_3omrsACP_c" name="3__Oxotetradecanoyl__[acyl__carrier protein]" compartment="c" charge="0" initialConcentration="0" hasOnlySubstanceUnits="false" boundaryCondition="false" constant="false" fbc:chemicalFormula="C25H45N2O9PRS"/>
<species metaid="M_3oocoa_c" id="M_3oocoa_c" name="3__Oxooctanoyl__CoA" compartment="c" charge="-4" initialConcentration="0" hasOnlySubstanceUnits="false" boundaryCondition="false" constant="false" fbc:chemicalFormula="C29H44N7O18P3S"/>
<species metaid="M_3oodACP_c" id="M_3oodACP_c" name="3__Oxooctodecanoyl__ACP" compartment="c" charge="-1" initialConcentration="0" hasOnlySubstanceUnits="false" boundaryCondition="false" constant="false" fbc:chemicalFormula="C29H53N2O9PRS"/>
<species metaid="M_3ophb_c" id="M_3ophb_c" name="3__Octaprenyl__4__hydroxybenzoate" compartment="c" charge="-1" initialConcentration="0" hasOnlySubstanceUnits="false" boundaryCondition="false" constant="false" fbc:chemicalFormula="C47H69O3"/>
<species metaid="M_3otdcoa_c" id="M_3otdcoa_c" name="3__Oxotetradecanoyl__CoA" compartment="c" charge="-4" initialConcentration="0" hasOnlySubstanceUnits="false" boundaryCondition="false" constant="false" fbc:chemicalFormula="C35H56N7O18P3S"/>
<species metaid="M_3oxddACP_c" id="M_3oxddACP_c" name="3__oxododecanoyl__acp" compartment="c" charge="-1" initialConcentration="0" hasOnlySubstanceUnits="false" boundaryCondition="false" constant="false" fbc:chemicalFormula="C23H41N2O9PRS"/>

<species metaid="M_3oxhdACP_c" id="M_3oxhdACP_c" name="3__oxohexadecanoyl__acp" compartment="c" charge="-1" initialConcentration="0" hasOnlySubstanceUnits="false" boundaryCondition="false" constant="false" fbc:chemicalFormula="C27H49N2O9PRS"/>
<species metaid="M_3oxocACP_c" id="M_3oxocACP_c" name="3__oxooctanoyl__acp" compartment="c" charge="-1" initialConcentration="0" hasOnlySubstanceUnits="false" boundaryCondition="false" constant="false" fbc:chemicalFormula="C19H33N2O9PRS"/>
<species metaid="M_3pg_c" id="M_3pg_c" name="3__Phospho__D__glycerate" compartment="c" charge="-3" initialConcentration="0" hasOnlySubstanceUnits="false" boundaryCondition="false" constant="false" fbc:chemicalFormula="C3H4O7P"/>
<species metaid="M_3php_c" id="M_3php_c" name="3__Phosphohydroxypyruvate" compartment="c" charge="-3" initialConcentration="0" hasOnlySubstanceUnits="false" boundaryCondition="false" constant="false" fbc:chemicalFormula="C3H2O7P"/>
<species metaid="M_3pop_c" id="M_3pop_c" name="3__Phosphonopyruvate" compartment="c" charge="-2" initialConcentration="0" hasOnlySubstanceUnits="false" boundaryCondition="false" constant="false" fbc:chemicalFormula="C3H2O6P"/>
<species metaid="M_3psme_c" id="M_3psme_c" name="5__O__(1__Carboxyvinyl)__3__phosphoshikimate" compartment="c" charge="-4" initialConcentration="0" hasOnlySubstanceUnits="false" boundaryCondition="false" constant="false" fbc:chemicalFormula="C10H9O10P"/>
<species metaid="M_3ump_c" id="M_3ump_c" name="3'__UMP" compartment="c" charge="-2" initialConcentration="0" hasOnlySubstanceUnits="false" boundaryCondition="false" constant="false" fbc:chemicalFormula="C9H11N2O9P"/>
<species metaid="M_4aabutn_c" id="M_4aabutn_c" name="4__Acetamidobutanoate" compartment="c" charge="0" initialConcentration="0" hasOnlySubstanceUnits="false" boundaryCondition="false" constant="false" fbc:chemicalFormula="C6H10NO3"/>
<species metaid="M_4abut_c" id="M_4abut_c" name="4__Aminobutanoate" compartment="c" charge="0" initialConcentration="0" hasOnlySubstanceUnits="false" boundaryCondition="false" constant="false" fbc:chemicalFormula="C4H9NO2"/>
<species metaid="M_4abut_e" id="M_4abut_e" name="4__Aminobutanoate_b" compartment="e" charge="0" initialConcentration="0" hasOnlySubstanceUnits="false" boundaryCondition="true" constant="false" fbc:chemicalFormula="C4H9NO2"/>
<species metaid="M_4abutn_c" id="M_4abutn_c" name="4__Aminobutanal" compartment="c" charge="1" initialConcentration="0" hasOnlySubstanceUnits="false" boundaryCondition="false" constant="false" fbc:chemicalFormula="C4H10NO"/>
<species metaid="M_4abz_c" id="M_4abz_c" name="4__Aminobenzoate" compartment="c" charge="-1" initialConcentration="0" hasOnlySubstanceUnits="false" boundaryCondition="false" constant="false" fbc:chemicalFormula="C7H6NO2"/>
<species metaid="M_4abz_e" id="M_4abz_e" name="4__Aminobenzoate_b" compartment="e" charge="-1" initialConcentration="0" hasOnlySubstanceUnits="false" boundaryCondition="true" constant="false" fbc:chemicalFormula="C7H6NO2"/>
<species metaid="M_4adcho_c" id="M_4adcho_c" name="4__amino__4__deoxychorismate" compartment="c" charge="-1" initialConcentration="0" hasOnlySubstanceUnits="false" boundaryCondition="false" constant="false" fbc:chemicalFormula="C10H10NO5"/>

<species metaid="M_4ahmmp_c" id="M_4ahmmp_c" name="4__Amino__5__hydroxymethyl__2__methylpyrimidine" compartment="c" charge="0" initialConcentration="0" hasOnlySubstanceUnits="false" boundaryCondition="false" constant="false" fbc:chemicalFormula="C6H9N3O"/>
<species metaid="M_4ampm_c" id="M_4ampm_c" name="4__Amino__2__methyl__5__phosphomethylpyrimidine" compartment="c" charge="-2" initialConcentration="0" hasOnlySubstanceUnits="false" boundaryCondition="false" constant="false" fbc:chemicalFormula="C6H8N3O4P"/>
<species metaid="M_4c2me_c" id="M_4c2me_c" name="4__(cytidine 5'__diphospho)__2__C__methyl__D__erythritol" compartment="c" charge="-2" initialConcentration="0" hasOnlySubstanceUnits="false" boundaryCondition="false" constant="false" fbc:chemicalFormula="C14H23N3O14P2"/>
<species metaid="M_4fe4s_c" id="M_4fe4s_c" name="[4Fe__4S] iron__sulfur cluster" compartment="c" charge="0" initialConcentration="0" hasOnlySubstanceUnits="false" boundaryCondition="false" constant="false" fbc:chemicalFormula="S4Fe4"/>
<species metaid="M_4gudbd_c" id="M_4gudbd_c" name="4__Guanidinobutanamide" compartment="c" charge="1" initialConcentration="0" hasOnlySubstanceUnits="false" boundaryCondition="false" constant="false" fbc:chemicalFormula="C5H13N4O"/>
<species metaid="M_4gudbutn_c" id="M_4gudbutn_c" name="4__Guanidinobutanoate" compartment="c" charge="0" initialConcentration="0" hasOnlySubstanceUnits="false" boundaryCondition="false" constant="false" fbc:chemicalFormula="C5H11N3O2"/>
<species metaid="M_4h2oglt_c" id="M_4h2oglt_c" name="4__Hydroxy__2__oxoglutarate" compartment="c" charge="-2" initialConcentration="0" hasOnlySubstanceUnits="false" boundaryCondition="false" constant="false" fbc:chemicalFormula="C5H4O6"/>
<species metaid="M_4h5pone" id="M_4h5pone" name="4__Hydroxy__5__phenyltetrahydro__1,3__oxazin__2__one" compartment="c" charge="0" initialConcentration="0" hasOnlySubstanceUnits="false" boundaryCondition="false" constant="false" fbc:chemicalFormula="C10H11NO3"/>
<species metaid="M_4hba_c" id="M_4hba_c" name="4__Hydroxy__benzyl alcohol" compartment="c" charge="0" initialConcentration="0" hasOnlySubstanceUnits="false" boundaryCondition="false" constant="false" fbc:chemicalFormula="C7H8O2"/>
<species metaid="M_4hba_e" id="M_4hba_e" name="4__Hydroxy__benzyl alcohol_b" compartment="e" charge="0" initialConcentration="0" hasOnlySubstanceUnits="false" boundaryCondition="true" constant="false" fbc:chemicalFormula="C7H8O2"/>
<species metaid="M_4hbz_c" id="M_4hbz_c" name="4__Hydroxybenzoate" compartment="c" charge="-1" initialConcentration="0" hasOnlySubstanceUnits="false" boundaryCondition="false" constant="false" fbc:chemicalFormula="C7H5O3"/>
<species metaid="M_4hmsial_c" id="M_4hmsial_c" name="4__Hydroxymethylsalicylate" compartment="c" charge="-1" initialConcentration="0" hasOnlySubstanceUnits="false" boundaryCondition="false" constant="false" fbc:chemicalFormula="C8H7O4"/>
<species metaid="M_4hoxpacd_e" id="M_4hoxpacd_e" name="4__Hydroxyphenylacetaldehyde_b" compartment="e" charge="0" initialConcentration="0" hasOnlySubstanceUnits="false" boundaryCondition="true" constant="false" fbc:chemicalFormula="C8H8O2"/>
<species metaid="M_4hpro__LT_c" id="M_4hpro__LT_c" name="trans__4__Hydroxy__L__proline" compartment="c" charge="-1" initialConcentration="0" hasOnlySubstanceUnits="false" boundaryCondition="false" constant="false" fbc:chemicalFormula="C5H9NO3"/>

<species metaid="M_4hthr_c" id="M_4hthr_c"
name="4__Hydroxy__L__threonine" compartment="c" charge="0"
initialConcentration="0" hasOnlySubstanceUnits="false"
boundaryCondition="false" constant="false" fbc:chemicalFormula="C4H9NO4"/>
<species metaid="M_4izp_c" id="M_4izp_c"
name="4__Imidazolone__5__propanoate" compartment="c" charge="-1"
initialConcentration="0" hasOnlySubstanceUnits="false"
boundaryCondition="false" constant="false" fbc:chemicalFormula="C6H7N2O3"/>
<species metaid="M_4m3hhexACP_c" id="M_4m3hhexACP_c"
name="4__methyl__3__hydroxy__hexanoyl__ACP" compartment="c" charge="-1"
initialConcentration="0" hasOnlySubstanceUnits="false"
boundaryCondition="false" constant="false"
fbc:chemicalFormula="C18H33N2O9PRS"/>
<species metaid="M_4m3hpAPC_c" id="M_4m3hpAPC_c"
name="4__methyl__3__hydroxy__pentanoyl__ACP" compartment="c" charge="-1"
initialConcentration="0" hasOnlySubstanceUnits="false"
boundaryCondition="false" constant="false"
fbc:chemicalFormula="C17H31N2O9PRS"/>
<species metaid="M_4m3ohexACP_c" id="M_4m3ohexACP_c"
name="4__methyl__3__oxo__hexanoyl__ACP" compartment="c" charge="-1"
initialConcentration="0" hasOnlySubstanceUnits="false"
boundaryCondition="false" constant="false"
fbc:chemicalFormula="C18H31N2O9PRS"/>
<species metaid="M_4m3opACP_c" id="M_4m3opACP_c"
name="4__methyl__3__oxo__pentanoyl__ACP" compartment="c" charge="-1"
initialConcentration="0" hasOnlySubstanceUnits="false"
boundaryCondition="false" constant="false"
fbc:chemicalFormula="C17H29N2O9PRS"/>
<species metaid="M_4mcat_c" id="M_4mcat_c" name="4__Methylcatechol"
compartment="c" charge="0" initialConcentration="0"
hasOnlySubstanceUnits="false" boundaryCondition="false" constant="false"
fbc:chemicalFormula="C7H8O2"/>
<species metaid="M_4methex2eACP_c" id="M_4methex2eACP_c"
name="4__methyl__trans__hex__2__enoyl__ACP" compartment="c" charge="-1"
initialConcentration="0" hasOnlySubstanceUnits="false"
boundaryCondition="false" constant="false"
fbc:chemicalFormula="C18H31N2O8PRS"/>
<species metaid="M_4methexACP_c" id="M_4methexACP_c"
name="4__methyl__hexanoyl__ACP" compartment="c" charge="-1"
initialConcentration="0" hasOnlySubstanceUnits="false"
boundaryCondition="false" constant="false"
fbc:chemicalFormula="C18H33N2O8PRS"/>
<species metaid="M_4mhetz_c" id="M_4mhetz_c"
name="4__Methyl__5__(2__hydroxyethyl)__thiazole" compartment="c" charge="0"
initialConcentration="0" hasOnlySubstanceUnits="false"
boundaryCondition="false" constant="false" fbc:chemicalFormula="C6H9NOS"/>
<species metaid="M_4mop_c" id="M_4mop_c"
name="4__Methyl__2__oxopentanoate" compartment="c" charge="-1"
initialConcentration="0" hasOnlySubstanceUnits="false"
boundaryCondition="false" constant="false" fbc:chemicalFormula="C6H9O3"/>
<species metaid="M_4mpACP_c" id="M_4mpACP_c"
name="4__methyl__pentanoyl__ACP" compartment="c" charge="-1"
initialConcentration="0" hasOnlySubstanceUnits="false"
boundaryCondition="false" constant="false"
fbc:chemicalFormula="C17H31N2O8PRS"/>
<species metaid="M_4mpetz_c" id="M_4mpetz_c"
name="4__Methyl__5__(2__phosphoethyl)__thiazole" compartment="c" charge="-2"

initialConcentration="0" hasOnlySubstanceUnits="false"
boundaryCondition="false" constant="false" fbc:chemicalFormula="C6H8NO4PS"/>
<species metaid="M_4mtpACP_c" id="M_4mtpACP_c"
name="4__methyl__trans__pent__2__enoyl__ACP" compartment="c" charge="-1"
initialConcentration="0" hasOnlySubstanceUnits="false"
boundaryCondition="false" constant="false"
fbc:chemicalFormula="C17H29N2O8PRS"/>
<species metaid="M_4nph_c" id="M_4nph_c" name="4__Nitrophenol"
compartment="c" charge="-1" initialConcentration="0"
hasOnlySubstanceUnits="false" boundaryCondition="false" constant="false"
fbc:chemicalFormula="C6H5NO3"/>
<species metaid="M_4nphp_c" id="M_4nphp_c" name="4__Nitrophenyl
phosphate" compartment="c" charge="-1" initialConcentration="0"
hasOnlySubstanceUnits="false" boundaryCondition="false" constant="false"
fbc:chemicalFormula="C6H4NO6P"/>
<species metaid="M_4pasp_c" id="M_4pasp_c"
name="4__Phospho__L__aspartate" compartment="c" charge="-2"
initialConcentration="0" hasOnlySubstanceUnits="false"
boundaryCondition="false" constant="false" fbc:chemicalFormula="C4H6NO7P"/>
<species metaid="M_4per_c" id="M_4per_c"
name="4__Phospho__D__erythronate" compartment="c" charge="-3"
initialConcentration="0" hasOnlySubstanceUnits="false"
boundaryCondition="false" constant="false" fbc:chemicalFormula="C4H6O8P"/>
<species metaid="M_4ppan_c" id="M_4ppan_c"
name="D__4'__Phosphopantothenate" compartment="c" charge="-3"
initialConcentration="0" hasOnlySubstanceUnits="false"
boundaryCondition="false" constant="false" fbc:chemicalFormula="C9H15NO8P"/>
<species metaid="M_4ppcys_c" id="M_4ppcys_c"
name="N__((R)__4__Phosphopantothenoyl)__L__cysteine" compartment="c"
charge="-3" initialConcentration="0" hasOnlySubstanceUnits="false"
boundaryCondition="false" constant="false"
fbc:chemicalFormula="C12H20N2O9PS"/>
<species metaid="M_4r5au_c" id="M_4r5au_c"
name="4__(1__D__Ribitylamino)__5__aminouracil" compartment="c" charge="0"
initialConcentration="0" hasOnlySubstanceUnits="false"
boundaryCondition="false" constant="false" fbc:chemicalFormula="C9H16N4O6"/>
<species metaid="M_5aizc_c" id="M_5aizc_c"
name="5__amino__1__(5__phospho__D__ribosyl)imidazole__4__carboxylate"
compartment="c" charge="-2" initialConcentration="0"
hasOnlySubstanceUnits="false" boundaryCondition="false" constant="false"
fbc:chemicalFormula="C9H11N3O9P"/>
<species metaid="M_5aop_c" id="M_5aop_c"
name="5__Amino__4__oxopentanoate" compartment="c" charge="0"
initialConcentration="0" hasOnlySubstanceUnits="false"
boundaryCondition="false" constant="false" fbc:chemicalFormula="C5H9NO3"/>
<species metaid="M_5aprbu_c" id="M_5aprbu_c"
name="5__Amino__6__(5'__phosphoribitylamino)uracil" compartment="c" charge="-
2" initialConcentration="0" hasOnlySubstanceUnits="false"
boundaryCondition="false" constant="false" fbc:chemicalFormula="C9H15N4O9P"/>
<species metaid="M_5apru_c" id="M_5apru_c"
name="5__Amino__6__(5'__phosphoribosylamino)uracil" compartment="c" charge="-
2" initialConcentration="0" hasOnlySubstanceUnits="false"
boundaryCondition="false" constant="false" fbc:chemicalFormula="C9H13N4O9P"/>
<species metaid="M_5dglcn_e" id="M_5dglcn_e"
name="5__Dehydro__D__gluconate_b" compartment="e" charge="-1"
initialConcentration="0" hasOnlySubstanceUnits="false"
boundaryCondition="true" constant="false" fbc:chemicalFormula="C6H9O7"/>

<species metaid="M_5dh4dglc_c" id="M_5dh4dglc_c" name="5__Dehydro__4__deoxy__D__glucarate" compartment="c" charge="-2" initialConcentration="0" hasOnlySubstanceUnits="false" boundaryCondition="false" constant="false" fbc:chemicalFormula="C6H6O7"/>
<species metaid="M_5dpmev_c" id="M_5dpmev_c" name="(R)__5__Diphosphomevalonate" compartment="c" charge="-4" initialConcentration="0" hasOnlySubstanceUnits="false" boundaryCondition="false" constant="false" fbc:chemicalFormula="C6H10O10P2"/>
<species metaid="M_5drib_c" id="M_5drib_c" name="5'__deoxyribose" compartment="c" charge="0" initialConcentration="0" hasOnlySubstanceUnits="false" boundaryCondition="false" constant="false" fbc:chemicalFormula="C5H10O4"/>
<species metaid="M_5fthf_c" id="M_5fthf_c" name="5__Formyltetrahydrofolate" compartment="c" charge="-2" initialConcentration="0" hasOnlySubstanceUnits="false" boundaryCondition="false" constant="false" fbc:chemicalFormula="C20H21N7O7"/>
<species metaid="M_5m3hACP_c" id="M_5m3hACP_c" name="5__methyl__3__hydroxy__hexanoyl__ACP" compartment="c" charge="-1" initialConcentration="0" hasOnlySubstanceUnits="false" boundaryCondition="false" constant="false" fbc:chemicalFormula="C18H33N2O9PRS"/>
<species metaid="M_5m3hhACP_c" id="M_5m3hhACP_c" name="5__methyl__3__oxo__hexanoyl__ACP" compartment="c" charge="-1" initialConcentration="0" hasOnlySubstanceUnits="false" boundaryCondition="false" constant="false" fbc:chemicalFormula="C18H31N2O9PRS"/>
<species metaid="M_5mdr1p_c" id="M_5mdr1p_c" name="5__Methylthio__5__deoxy__D__ribose 1__phosphate" compartment="c" charge="-2" initialConcentration="0" hasOnlySubstanceUnits="false" boundaryCondition="false" constant="false" fbc:chemicalFormula="C6H11O7PS"/>
<species metaid="M_5mdru1p_c" id="M_5mdru1p_c" name="5__Methylthio__5__deoxy__D__ribulose 1__phosphate" compartment="c" charge="-2" initialConcentration="0" hasOnlySubstanceUnits="false" boundaryCondition="false" constant="false" fbc:chemicalFormula="C6H11O7PS"/>
<species metaid="M_5mhACP_c" id="M_5mhACP_c" name="5__methyl__trans__hex__2__enoyl__ACP" compartment="c" charge="-1" initialConcentration="0" hasOnlySubstanceUnits="false" boundaryCondition="false" constant="false" fbc:chemicalFormula="C18H31N2O8PRS"/>
<species metaid="M_5mta_c" id="M_5mta_c" name="5__Methylthioadenosine" compartment="c" charge="0" initialConcentration="0" hasOnlySubstanceUnits="false" boundaryCondition="false" constant="false" fbc:chemicalFormula="C11H15N5O3S"/>
<species metaid="M_5mthf_c" id="M_5mthf_c" name="5__Methyltetrahydrofolate" compartment="c" charge="-2" initialConcentration="0" hasOnlySubstanceUnits="false" boundaryCondition="false" constant="false" fbc:chemicalFormula="C20H24N7O6"/>
<species metaid="M_5mtr_c" id="M_5mtr_c" name="5__Methylthio__D__ribose" compartment="c" charge="0" initialConcentration="0" hasOnlySubstanceUnits="false" boundaryCondition="false" constant="false" fbc:chemicalFormula="C6H12O4S"/>
<species metaid="M_5mtr_e" id="M_5mtr_e" name="5__Methylthio__D__ribose_b" compartment="e" charge="0" initialConcentration="0" hasOnlySubstanceUnits="false" boundaryCondition="true" constant="false" fbc:chemicalFormula="C6H12O4S"/>
<species metaid="M_5pmev_c" id="M_5pmev_c" name="(R)__5__Phosphomevalonate" compartment="c" charge="-3"

```
initialConcentration="0" hasOnlySubstanceUnits="false"
boundaryCondition="false" constant="false" fbc:chemicalFormula="C6H10O7P"/>
      <species metaid="M_5podio" id="M_5podio"
name="5__Phenyl__1,3__oxazinane__2,4__dione" compartment="c" charge="0"
initialConcentration="0" hasOnlySubstanceUnits="false"
boundaryCondition="false" constant="false" fbc:chemicalFormula="C10H9NO3"/>
      <species metaid="M_6hmhpt_c" id="M_6hmhpt_c" name="6__hydroxymethyl
dihydropterin" compartment="c" charge="0" initialConcentration="0"
hasOnlySubstanceUnits="false" boundaryCondition="false" constant="false"
fbc:chemicalFormula="C7H9N5O2"/>
      <species metaid="M_6hmhptpp_c" id="M_6hmhptpp_c"
name="6__hydroxymethyl__dihydropterin pyrophosphate" compartment="c"
charge="-3" initialConcentration="0" hasOnlySubstanceUnits="false"
boundaryCondition="false" constant="false" fbc:chemicalFormula="C7H8N5O8P2"/>
      <species metaid="M_6m3hhACP_c" id="M_6m3hhACP_c"
name="6__methyl__3__hydroxy__heptanoyl__ACP" compartment="c" charge="-1"
initialConcentration="0" hasOnlySubstanceUnits="false"
boundaryCondition="false" constant="false"
fbc:chemicalFormula="C19H35N2O9PRS"/>
      <species metaid="M_6m3hoACP_c" id="M_6m3hoACP_c"
name="6__methyl__3__hydroxy__octanoyl__ACP" compartment="c" charge="-1"
initialConcentration="0" hasOnlySubstanceUnits="false"
boundaryCondition="false" constant="false"
fbc:chemicalFormula="C20H37N2O9PRS"/>
      <species metaid="M_6m3oACP_c" id="M_6m3oACP_c"
name="6__methyl__3__oxo__octanoyl__ACP" compartment="c" charge="-1"
initialConcentration="0" hasOnlySubstanceUnits="false"
boundaryCondition="false" constant="false"
fbc:chemicalFormula="C20H35N2O9PRS"/>
      <species metaid="M_6m3ohACP_c" id="M_6m3ohACP_c"
name="6__methyl__3__oxo__heptanoyl__ACP" compartment="c" charge="-1"
initialConcentration="0" hasOnlySubstanceUnits="false"
boundaryCondition="false" constant="false"
fbc:chemicalFormula="C19H33N2O9PRS"/>
      <species metaid="M_6mhACP_c" id="M_6mhACP_c"
name="6__methyl__heptanoyl__ACP" compartment="c" charge="-1"
initialConcentration="0" hasOnlySubstanceUnits="false"
boundaryCondition="false" constant="false"
fbc:chemicalFormula="C19H35N2O8PRS"/>
      <species metaid="M_6moACP_c" id="M_6moACP_c"
name="6__methyl__octanoyl__ACP" compartment="c" charge="-1"
initialConcentration="0" hasOnlySubstanceUnits="false"
boundaryCondition="false" constant="false"
fbc:chemicalFormula="C20H37N2O8PRS"/>
      <species metaid="M_6mth2eACP_c" id="M_6mth2eACP_c"
name="6__methyl__trans__hept__2__enoyl__ACP" compartment="c" charge="-1"
initialConcentration="0" hasOnlySubstanceUnits="false"
boundaryCondition="false" constant="false"
fbc:chemicalFormula="C19H33N2O8PRS"/>
      <species metaid="M_6mto2eACP_c" id="M_6mto2eACP_c"
name="6__methyl__trans__oct__2__enoyl__ACP" compartment="c" charge="-1"
initialConcentration="0" hasOnlySubstanceUnits="false"
boundaryCondition="false" constant="false"
fbc:chemicalFormula="C20H35N2O8PRS"/>
      <species metaid="M_6pgc_c" id="M_6pgc_c"
name="6__Phospho__D__gluconate" compartment="c" charge="-3"
initialConcentration="0" hasOnlySubstanceUnits="false"
boundaryCondition="false" constant="false" fbc:chemicalFormula="C6H10O10P"/>
```

```
      <species metaid="M_6pgl_c" id="M_6pgl_c"
name="6__phospho__D__glucono__1,5__lactone" compartment="c" charge="-2"
initialConcentration="0" hasOnlySubstanceUnits="false"
boundaryCondition="false" constant="false" fbc:chemicalFormula="C6H9O9P"/>
      <species metaid="M_6pthp_c" id="M_6pthp_c"
name="6__Pyruvoyl__5,6,7,8__tetrahydropterin" compartment="c" charge="0"
initialConcentration="0" hasOnlySubstanceUnits="false"
boundaryCondition="false" constant="false" fbc:chemicalFormula="C9H11N5O3"/>
      <species metaid="M_7m3hoACP_c" id="M_7m3hoACP_c"
name="7__methyl__3__hydroxy__octanoyl__ACP" compartment="c" charge="-1"
initialConcentration="0" hasOnlySubstanceUnits="false"
boundaryCondition="false" constant="false"
fbc:chemicalFormula="C20H37N2O9PRS"/>
      <species metaid="M_7me3oxoacp_c" id="M_7me3oxoacp_c"
name="7__methyl__3__oxo__octanoyl__ACP" compartment="c" charge="-1"
initialConcentration="0" hasOnlySubstanceUnits="false"
boundaryCondition="false" constant="false"
fbc:chemicalFormula="C20H35N2O9PRS"/>
      <species metaid="M_7moACP_c" id="M_7moACP_c"
name="7__methyl__octanoyl__ACP" compartment="c" charge="-1"
initialConcentration="0" hasOnlySubstanceUnits="false"
boundaryCondition="false" constant="false"
fbc:chemicalFormula="C20H37N2O8PRS"/>
      <species metaid="M_7mto2eACP_c" id="M_7mto2eACP_c"
name="7__methyl__trans__oct__2__enoyl__ACP" compartment="c" charge="-1"
initialConcentration="0" hasOnlySubstanceUnits="false"
boundaryCondition="false" constant="false"
fbc:chemicalFormula="C20H35N2O8PRS"/>
      <species metaid="M_8aonn_c" id="M_8aonn_c"
name="8__Amino__7__oxononanoate" compartment="c" charge="0"
initialConcentration="0" hasOnlySubstanceUnits="false"
boundaryCondition="false" constant="false" fbc:chemicalFormula="C9H17NO3"/>
      <species metaid="M_8m3hdACP_c" id="M_8m3hdACP_c"
name="8__methyl__3__hydroxy__decanoyl__ACP" compartment="c" charge="-1"
initialConcentration="0" hasOnlySubstanceUnits="false"
boundaryCondition="false" constant="false"
fbc:chemicalFormula="C22H41N2O9PRS"/>
      <species metaid="M_8m3hnACP_c" id="M_8m3hnACP_c"
name="8__methyl__3__hydroxy__nonanoyl__ACP" compartment="c" charge="-1"
initialConcentration="0" hasOnlySubstanceUnits="false"
boundaryCondition="false" constant="false"
fbc:chemicalFormula="C21H39N2O9PRS"/>
      <species metaid="M_8m3odACP_c" id="M_8m3odACP_c"
name="8__methyl__3__oxo__decanoyl__ACP" compartment="c" charge="-1"
initialConcentration="0" hasOnlySubstanceUnits="false"
boundaryCondition="false" constant="false"
fbc:chemicalFormula="C22H39N2O9PRS"/>
      <species metaid="M_8m3oxACP_c" id="M_8m3oxACP_c"
name="8__methyl__3__oxo__nonanoyl__ACP" compartment="c" charge="-1"
initialConcentration="0" hasOnlySubstanceUnits="false"
boundaryCondition="false" constant="false"
fbc:chemicalFormula="C21H37N2O9PRS"/>
      <species metaid="M_8mcACP_c" id="M_8mcACP_c"
name="8__methyl__nonanoyl__ACP" compartment="c" charge="-1"
initialConcentration="0" hasOnlySubstanceUnits="false"
boundaryCondition="false" constant="false"
fbc:chemicalFormula="C21H39N2O8PRS"/>
```

```
      <species metaid="M_8mdACP_c" id="M_8mdACP_c"
name="8__methyl__decanoyl__ACP" compartment="c" charge="-1"
initialConcentration="0" hasOnlySubstanceUnits="false"
boundaryCondition="false" constant="false"
fbc:chemicalFormula="C22H41N2O8PRS"/>
      <species metaid="M_8mtd2eACP_c" id="M_8mtd2eACP_c"
name="8__methyl__trans__dec__2__enoyl__ACP" compartment="c" charge="-1"
initialConcentration="0" hasOnlySubstanceUnits="false"
boundaryCondition="false" constant="false"
fbc:chemicalFormula="C22H39N2O8PRS"/>
      <species metaid="M_8mtn2eACP_c" id="M_8mtn2eACP_c"
name="8__methyl__trans__non__2__enoyl__ACP" compartment="c" charge="-1"
initialConcentration="0" hasOnlySubstanceUnits="false"
boundaryCondition="false" constant="false"
fbc:chemicalFormula="C21H37N2O8PRS"/>
      <species metaid="M_9m3hdACP_c" id="M_9m3hdACP_c"
name="9__methyl__3__hydroxy__decanoyl__ACP" compartment="c" charge="-1"
initialConcentration="0" hasOnlySubstanceUnits="false"
boundaryCondition="false" constant="false"
fbc:chemicalFormula="C22H41N2O9PRS"/>
      <species metaid="M_9m3odACP_c" id="M_9m3odACP_c"
name="9__methyl__3__oxo__decanoyl__ACP" compartment="c" charge="-1"
initialConcentration="0" hasOnlySubstanceUnits="false"
boundaryCondition="false" constant="false"
fbc:chemicalFormula="C22H39N2O9PRS"/>
      <species metaid="M_9mdACP_c" id="M_9mdACP_c"
name="9__methyl__decanoyl__ACP" compartment="c" charge="-1"
initialConcentration="0" hasOnlySubstanceUnits="false"
boundaryCondition="false" constant="false"
fbc:chemicalFormula="C22H41N2O8PRS"/>
      <species metaid="M_9mtd2eACP_c" id="M_9mtd2eACP_c"
name="9__methyl__trans__dec__2__enoyl__ACP" compartment="c" charge="-1"
initialConcentration="0" hasOnlySubstanceUnits="false"
boundaryCondition="false" constant="false"
fbc:chemicalFormula="C22H39N2O8PRS"/>
      <species metaid="M_10fthf_c" id="M_10fthf_c"
name="10__Formyltetrahydrofolate" compartment="c" charge="-2"
initialConcentration="0" hasOnlySubstanceUnits="false"
boundaryCondition="false" constant="false" fbc:chemicalFormula="C20H21N7O7"/>
      <species metaid="M_10m3ouACP_c" id="M_10m3ouACP_c"
name="10__methyl__3__oxo__undecanoyl__ACP" compartment="c" charge="-1"
initialConcentration="0" hasOnlySubstanceUnits="false"
boundaryCondition="false" constant="false"
fbc:chemicalFormula="C23H41N2O9PRS"/>
      <species metaid="M_10m3uACP_c" id="M_10m3uACP_c"
name="10__methyl__3__hydroxy__undecanoyl__ACP" compartment="c" charge="-1"
initialConcentration="0" hasOnlySubstanceUnits="false"
boundaryCondition="false" constant="false"
fbc:chemicalFormula="C23H43N2O9PRS"/>
      <species metaid="M_10mdACP_c" id="M_10mdACP_c"
name="10__methyl__dodecanoyl__ACP" compartment="c" charge="-1"
initialConcentration="0" hasOnlySubstanceUnits="false"
boundaryCondition="false" constant="false"
fbc:chemicalFormula="C24H45N2O8PRS"/>
      <species metaid="M_10mtd2eACP_c" id="M_10mtd2eACP_c"
name="10__methyl__trans__dodec__2__enoyl__ACP" compartment="c" charge="-1"
initialConcentration="0" hasOnlySubstanceUnits="false"
```

```
boundaryCondition="false" constant="false"
fbc:chemicalFormula="C24H43N2O8PRS"/>
      <species metaid="M_10mtu2eACP_c" id="M_10mtu2eACP_c"
name="10__methyl__trans__undec__2__enoyl__ACP" compartment="c" charge="-1"
initialConcentration="0" hasOnlySubstanceUnits="false"
boundaryCondition="false" constant="false"
fbc:chemicalFormula="C23H41N2O8PRS"/>
      <species metaid="M_10muACP_c" id="M_10muACP_c"
name="10__methyl__undecanoyl__ACP" compartment="c" charge="-1"
initialConcentration="0" hasOnlySubstanceUnits="false"
boundaryCondition="false" constant="false"
fbc:chemicalFormula="C23H43N2O8PRS"/>
      <species metaid="M_11m3hdACP_c" id="M_11m3hdACP_c"
name="11__methyl__3__hydroxy__dodecanoyl__ACP" compartment="c" charge="-1"
initialConcentration="0" hasOnlySubstanceUnits="false"
boundaryCondition="false" constant="false"
fbc:chemicalFormula="C24H45N2O9PRS"/>
      <species metaid="M_11m3odACP_c" id="M_11m3odACP_c"
name="11__methyl__3__oxo__dodecanoyl__ACP" compartment="c" charge="-1"
initialConcentration="0" hasOnlySubstanceUnits="false"
boundaryCondition="false" constant="false"
fbc:chemicalFormula="C24H43N2O9PRS"/>
      <species metaid="M_11mdACP_c" id="M_11mdACP_c"
name="11__methyl__dodecanoyl__ACP" compartment="c" charge="-1"
initialConcentration="0" hasOnlySubstanceUnits="false"
boundaryCondition="false" constant="false"
fbc:chemicalFormula="C24H45N2O8PRS"/>
      <species metaid="M_11mtdeACP_c" id="M_11mtdeACP_c"
name="11__methyl__trans__dodec__2__enoyl__ACP" compartment="c" charge="-1"
initialConcentration="0" hasOnlySubstanceUnits="false"
boundaryCondition="false" constant="false"
fbc:chemicalFormula="C24H43N2O8PRS"/>
      <species metaid="M_12ddgly3p_c" id="M_12ddgly3p_c"
name="1_2__dianteisoheptadecanoyl__sn__glycerol_3__phosphate" compartment="c"
charge="-1" initialConcentration="0" hasOnlySubstanceUnits="false"
boundaryCondition="false" constant="false" fbc:chemicalFormula=""/>
      <species metaid="M_12dgr_SA_c" id="M_12dgr_SA_c"
name="1,2__Daicylglycerol (Saureus)" compartment="c" charge="0"
initialConcentration="0" hasOnlySubstanceUnits="false"
boundaryCondition="false" constant="false"
fbc:chemicalFormula="C1560H3065O42"/>
      <species metaid="M_12dgr120_c" id="M_12dgr120_c"
name="1,2__Diacyl__sn__glycerol (didodecanoyl, n__C12:0)" compartment="c"
charge="0" initialConcentration="0" hasOnlySubstanceUnits="false"
boundaryCondition="false" constant="false" fbc:chemicalFormula="C27H52O5"/>
      <species metaid="M_12dgr140_c" id="M_12dgr140_c"
name="1,2__Diacyl__sn__glycerol (ditetradecanoyl, n__C14:0)" compartment="c"
charge="0" initialConcentration="0" hasOnlySubstanceUnits="false"
boundaryCondition="false" constant="false" fbc:chemicalFormula="C31H60O5"/>
      <species metaid="M_12dgr141_c" id="M_12dgr141_c"
name="1,2__Diacyl__sn__glycerol (ditetradec__7__enoyl, n__C14:1)"
compartment="c" charge="0" initialConcentration="0"
hasOnlySubstanceUnits="false" boundaryCondition="false" constant="false"
fbc:chemicalFormula="C31H56O5"/>
      <species metaid="M_12dgr160_c" id="M_12dgr160_c"
name="1,2__Diacyl__sn__glycerol (dihexadecanoyl, n__C16:0)" compartment="c"
charge="0" initialConcentration="0" hasOnlySubstanceUnits="false"
boundaryCondition="false" constant="false" fbc:chemicalFormula="C35H68O5"/>
```

<species metaid="M_12dgr161_c" id="M_12dgr161_c"
name="1,2__Diacyl__sn__glycerol (dihexadec__9__enoyl, n__C16:1)"
compartment="c" charge="0" initialConcentration="0"
hasOnlySubstanceUnits="false" boundaryCondition="false" constant="false"
fbc:chemicalFormula="C35H64O5"/>
<species metaid="M_12dgr180_c" id="M_12dgr180_c"
name="1,2__Diacyl__sn__glycerol (dioctadecanoyl, n__C18:0)" compartment="c"
charge="0" initialConcentration="0" hasOnlySubstanceUnits="false"
boundaryCondition="false" constant="false" fbc:chemicalFormula="C39H76O5"/>
<species metaid="M_12dgr181_c" id="M_12dgr181_c"
name="1,2__Diacyl__sn__glycerol (dioctadec__11__enoyl, n__C18:1)"
compartment="c" charge="0" initialConcentration="0"
hasOnlySubstanceUnits="false" boundaryCondition="false" constant="false"
fbc:chemicalFormula="C39H72O5"/>
<species metaid="M_12dhsgly3p_c" id="M_12dhsgly3p_c"
name="1_2__diisohexadecanoyl__sn__glycerol_3__phosphate" compartment="c"
charge="-1" initialConcentration="0" hasOnlySubstanceUnits="false"
boundaryCondition="false" constant="false" fbc:chemicalFormula=""/>
<species metaid="M_12diasglyc_c" id="M_12diasglyc_c"
name="1_2__Dianteisopentadecanoyl__sn__glycerol" compartment="c" charge="0"
initialConcentration="0" hasOnlySubstanceUnits="false"
boundaryCondition="false" constant="false" fbc:chemicalFormula=""/>
<species metaid="M_12diasn3_c" id="M_12diasn3_c"
name="1_2__dianteisopentadecanoyl__sn__glycerol_3__phosphate" compartment="c"
charge="-1" initialConcentration="0" hasOnlySubstanceUnits="false"
boundaryCondition="false" constant="false" fbc:chemicalFormula=""/>
<species metaid="M_12dihexsgly_c" id="M_12dihexsgly_c"
name="1_2__Diisohexadecanoyl__sn__glycerol" compartment="c" charge="0"
initialConcentration="0" hasOnlySubstanceUnits="false"
boundaryCondition="false" constant="false" fbc:chemicalFormula=""/>
<species metaid="M_12diidgly3p_c" id="M_12diidgly3p_c"
name="1_2__diisotetradecanoyl__sn__glycerol_3__phosphate" compartment="c"
charge="-1" initialConcentration="0" hasOnlySubstanceUnits="false"
boundaryCondition="false" constant="false" fbc:chemicalFormula=""/>
<species metaid="M_12diisgly_c" id="M_12diisgly_c"
name="1_2__Diisoheptadecanoyl__sn__glycerol" compartment="c" charge="0"
initialConcentration="0" hasOnlySubstanceUnits="false"
boundaryCondition="false" constant="false" fbc:chemicalFormula=""/>
<species metaid="M_12disgly_c" id="M_12disgly_c"
name="1_2__Dianteisoheptadecanoyl__sn__glycerol" compartment="c" charge="0"
initialConcentration="0" hasOnlySubstanceUnits="false"
boundaryCondition="false" constant="false" fbc:chemicalFormula=""/>
<species metaid="M_12ditetgly_c" id="M_12ditetgly_c"
name="1_2__Diisotetradecanoyl__sn__glycerol" compartment="c" charge="0"
initialConcentration="0" hasOnlySubstanceUnits="false"
boundaryCondition="false" constant="false" fbc:chemicalFormula=""/>
<species metaid="M_12dpgly_c" id="M_12dpgly_c"
name="1_2__Diisopentadecanoyl__sn__glycerol" compartment="c" charge="0"
initialConcentration="0" hasOnlySubstanceUnits="false"
boundaryCondition="false" constant="false" fbc:chemicalFormula=""/>
<species metaid="M_12dpsgly3p_c" id="M_12dpsgly3p_c"
name="1_2__diisopentadecanoyl__sn__glycerol_3__phosphate" compartment="c"
charge="-1" initialConcentration="0" hasOnlySubstanceUnits="false"
boundaryCondition="false" constant="false" fbc:chemicalFormula=""/>
<species metaid="M_12dsgly3p_c" id="M_12dsgly3p_c"
name="1_2__diisoheptadecanoyl__sn__glycerol_3__phosphate" compartment="c"
charge="-1" initialConcentration="0" hasOnlySubstanceUnits="false"
boundaryCondition="false" constant="false" fbc:chemicalFormula=""/>

<species metaid="M_12m3htdACP_c" id="M_12m3htdACP_c" name="12__methyl__3__hydroxy__tetra__decanoyl__ACP" compartment="c" charge="-1" initialConcentration="0" hasOnlySubstanceUnits="false" boundaryCondition="false" constant="false" fbc:chemicalFormula="C26H49N2O9PRS"/>
<species metaid="M_12m3otACP_c" id="M_12m3otACP_c" name="12__methyl__3__oxo__tridecanoyl__ACP" compartment="c" charge="-1" initialConcentration="0" hasOnlySubstanceUnits="false" boundaryCondition="false" constant="false" fbc:chemicalFormula="C25H45N2O9PRS"/>
<species metaid="M_12mdhtACP_c" id="M_12mdhtACP_c" name="12__methyl__3__hydroxy__tridecanoyl__ACP" compartment="c" charge="-1" initialConcentration="0" hasOnlySubstanceUnits="false" boundaryCondition="false" constant="false" fbc:chemicalFormula="C25H47N2O9PRS"/>
<species metaid="M_12methedec_c" id="M_12methedec_c" name="12__methyl__tetra__decanoyl__ACP" compartment="c" charge="-1" initialConcentration="0" hasOnlySubstanceUnits="false" boundaryCondition="false" constant="false" fbc:chemicalFormula="C26H49N2O8PRS"/>
<species metaid="M_12methtetdec2_c" id="M_12methtetdec2_c" name="12__methyl__trans__tetra__dec__2__enoyl__ACP" compartment="c" charge="-1" initialConcentration="0" hasOnlySubstanceUnits="false" boundaryCondition="false" constant="false" fbc:chemicalFormula="C26H47N2O8PRS"/>
<species metaid="M_12mtACP_c" id="M_12mtACP_c" name="12__methyl__tridecanoyl__ACP" compartment="c" charge="-1" initialConcentration="0" hasOnlySubstanceUnits="false" boundaryCondition="false" constant="false" fbc:chemicalFormula="C25H47N2O8PRS"/>
<species metaid="M_12mtt2eACP_c" id="M_12mtt2eACP_c" name="12__methyl__trans__tridec__2__enoyl__ACP" compartment="c" charge="-1" initialConcentration="0" hasOnlySubstanceUnits="false" boundaryCondition="false" constant="false" fbc:chemicalFormula="C25H45N2O8PRS"/>
<species metaid="M_12napdol_c" id="M_12napdol_c" name="1_2__Naphthalenediol" compartment="c" charge="0" initialConcentration="0" hasOnlySubstanceUnits="false" boundaryCondition="false" constant="false" fbc:chemicalFormula=""/>
<species metaid="M_12ppd__R_e" id="M_12ppd__R_e" name="(R)__Propane__1,2__diol_b" compartment="e" charge="0" initialConcentration="0" hasOnlySubstanceUnits="false" boundaryCondition="true" constant="false" fbc:chemicalFormula="C3H8O2"/>
<species metaid="M_12ppd__S_e" id="M_12ppd__S_e" name="(S)__Propane__1,2__diol_b" compartment="e" charge="0" initialConcentration="0" hasOnlySubstanceUnits="false" boundaryCondition="true" constant="false" fbc:chemicalFormula="C3H8O2"/>
<species metaid="M_13dpg_c" id="M_13dpg_c" name="3__Phospho__D__glyceroyl phosphate" compartment="c" charge="-4" initialConcentration="0" hasOnlySubstanceUnits="false" boundaryCondition="false" constant="false" fbc:chemicalFormula="C3H4O10P2"/>
<species metaid="M_13m3htdACP_c" id="M_13m3htdACP_c" name="13__methyl__3__hydroxy__tetra__decanoyl__ACP" compartment="c" charge="-1" initialConcentration="0" hasOnlySubstanceUnits="false" boundaryCondition="false" constant="false" fbc:chemicalFormula="C26H49N2O9PRS"/>
<species metaid="M_13m3otdACP_c" id="M_13m3otdACP_c" name="13__methyl__3__oxo__tetra__decanoyl__ACP" compartment="c" charge="-1"

initialConcentration="0" hasOnlySubstanceUnits="false"
boundaryCondition="false" constant="false"
fbc:chemicalFormula="C26H47N2O9PRS"/>
<species metaid="M_13mtdACP_c" id="M_13mtdACP_c"
name="13__methyl__tetra__decanoyl__ACP" compartment="c" charge="-1"
initialConcentration="0" hasOnlySubstanceUnits="false"
boundaryCondition="false" constant="false"
fbc:chemicalFormula="C26H49N2O8PRS"/>
<species metaid="M_13mttd2eACP_c" id="M_13mttd2eACP_c"
name="13__methyl__trans__tetra__dec__2__enoyl__ACP" compartment="c" charge="-
1" initialConcentration="0" hasOnlySubstanceUnits="false"
boundaryCondition="false" constant="false"
fbc:chemicalFormula="C26H47N2O8PRS"/>
<species metaid="M_14dhncoa_c" id="M_14dhncoa_c"
name="1,4__dihydroxy__2__napthoyl__CoA" compartment="c" charge="-3"
initialConcentration="0" hasOnlySubstanceUnits="false"
boundaryCondition="false" constant="false"
fbc:chemicalFormula="C32H38N7O19P3S"/>
<species metaid="M_14m3hdACP_c" id="M_14m3hdACP_c"
name="14__methyl__3__hydroxy__hexa__decanoyl__ACP" compartment="c" charge="-
1" initialConcentration="0" hasOnlySubstanceUnits="false"
boundaryCondition="false" constant="false"
fbc:chemicalFormula="C28H53N2O9PRS"/>
<species metaid="M_14m3hpACP_c" id="M_14m3hpACP_c"
name="14__methyl__3__hydroxy__pentadecanoyl__ACP" compartment="c" charge="-1"
initialConcentration="0" hasOnlySubstanceUnits="false"
boundaryCondition="false" constant="false"
fbc:chemicalFormula="C27H51N2O9PRS"/>
<species metaid="M_14m3ohdACP_c" id="M_14m3ohdACP_c"
name="14__methyl__3__oxo__hexa__decanoyl__ACP" compartment="c" charge="-1"
initialConcentration="0" hasOnlySubstanceUnits="false"
boundaryCondition="false" constant="false"
fbc:chemicalFormula="C28H51N2O9PRS"/>
<species metaid="M_14m3opACP_c" id="M_14m3opACP_c"
name="14__methyl__3__oxo__pentadecanoyl__ACP" compartment="c" charge="-1"
initialConcentration="0" hasOnlySubstanceUnits="false"
boundaryCondition="false" constant="false"
fbc:chemicalFormula="C27H49N2O9PRS"/>
<species metaid="M_14mhdACP_c" id="M_14mhdACP_c"
name="14__methyl__hexa__decanoyl__ACP" compartment="c" charge="-1"
initialConcentration="0" hasOnlySubstanceUnits="false"
boundaryCondition="false" constant="false"
fbc:chemicalFormula="C28H53N2O8PRS"/>
<species metaid="M_14mpACP_c" id="M_14mpACP_c"
name="14__methyl__pentadecanoyl__ACP" compartment="c" charge="-1"
initialConcentration="0" hasOnlySubstanceUnits="false"
boundaryCondition="false" constant="false"
fbc:chemicalFormula="C27H51N2O8PRS"/>
<species metaid="M_14mthdeACP_c" id="M_14mthdeACP_c"
name="14__methyl__trans__hexa__dec__2__enoyl__ACP" compartment="c" charge="-
1" initialConcentration="0" hasOnlySubstanceUnits="false"
boundaryCondition="false" constant="false"
fbc:chemicalFormula="C28H51N2O8PRS"/>
<species metaid="M_14mtp2eACP_c" id="M_14mtp2eACP_c"
name="14__methyl__trans__pentadec__2__enoyl__ACP" compartment="c" charge="-1"
initialConcentration="0" hasOnlySubstanceUnits="false"
boundaryCondition="false" constant="false"
fbc:chemicalFormula="C27H49N2O8PRS"/>

<species metaid="M_15dap_c" id="M_15dap_c" name="1,5__Diaminopentane"
compartment="c" charge="2" initialConcentration="0"
hasOnlySubstanceUnits="false" boundaryCondition="false" constant="false"
fbc:chemicalFormula="C5H16N2"/>
<species metaid="M_15m3hdACP_c" id="M_15m3hdACP_c"
name="15__methyl__3__hydroxy__hexa__decanoyl__ACP" compartment="c" charge="-
1" initialConcentration="0" hasOnlySubstanceUnits="false"
boundaryCondition="false" constant="false"
fbc:chemicalFormula="C28H53N2O9PRS"/>
<species metaid="M_15m3ohdACP_c" id="M_15m3ohdACP_c"
name="15__methyl__3__oxo__hexa__decanoyl__ACP" compartment="c" charge="-1"
initialConcentration="0" hasOnlySubstanceUnits="false"
boundaryCondition="false" constant="false"
fbc:chemicalFormula="C28H51N2O9PRS"/>
<species metaid="M_15methexeACP_c" id="M_15methexeACP_c"
name="15__methyl__trans__hexa__dec__2__enoyl__ACP" compartment="c" charge="-
1" initialConcentration="0" hasOnlySubstanceUnits="false"
boundaryCondition="false" constant="false"
fbc:chemicalFormula="C28H51N2O8PRS"/>
<species metaid="M_15mhdACP_c" id="M_15mhdACP_c"
name="15__methyl__hexa__decanoyl__ACP" compartment="c" charge="-1"
initialConcentration="0" hasOnlySubstanceUnits="false"
boundaryCondition="false" constant="false"
fbc:chemicalFormula="C28H53N2O8PRS"/>
<species metaid="M_23camp_c" id="M_23camp_c" name="2',3'__Cyclic AMP"
compartment="c" charge="-1" initialConcentration="0"
hasOnlySubstanceUnits="false" boundaryCondition="false" constant="false"
fbc:chemicalFormula="C10H11N5O6P"/>
<species metaid="M_23ccmp_c" id="M_23ccmp_c" name="2',3'__cyclic CMP"
compartment="c" charge="-1" initialConcentration="0"
hasOnlySubstanceUnits="false" boundaryCondition="false" constant="false"
fbc:chemicalFormula="C9H11N3O7P"/>
<species metaid="M_23ccmp_e" id="M_23ccmp_e" name="2',3'__Cyclic CMP_b"
compartment="e" charge="-1" initialConcentration="0"
hasOnlySubstanceUnits="false" boundaryCondition="true" constant="false"
fbc:chemicalFormula="C9H11N3O7P"/>
<species metaid="M_23cgmp_c" id="M_23cgmp_c" name="2',3'__cyclic GMP"
compartment="c" charge="-1" initialConcentration="0"
hasOnlySubstanceUnits="false" boundaryCondition="false" constant="false"
fbc:chemicalFormula="C10H11N5O7P"/>
<species metaid="M_23cump_c" id="M_23cump_c" name="2',3'__cyclic UMP"
compartment="c" charge="-1" initialConcentration="0"
hasOnlySubstanceUnits="false" boundaryCondition="false" constant="false"
fbc:chemicalFormula="C9H10N2O8P"/>
<species metaid="M_23ddhb_c" id="M_23ddhb_c"
name="2,3__Dihydro__2,3__dihydroxybenzoate" compartment="c" charge="-1"
initialConcentration="0" hasOnlySubstanceUnits="false"
boundaryCondition="false" constant="false" fbc:chemicalFormula="C7H7O4"/>
<species metaid="M_23dhdp_c" id="M_23dhdp_c"
name="2,3__Dihydrodipicolinate" compartment="c" charge="-2"
initialConcentration="0" hasOnlySubstanceUnits="false"
boundaryCondition="false" constant="false" fbc:chemicalFormula="C7H5NO4"/>
<species metaid="M_23dhmb_c" id="M_23dhmb_c"
name="(R)__2,3__Dihydroxy__3__methylbutanoate" compartment="c" charge="-1"
initialConcentration="0" hasOnlySubstanceUnits="false"
boundaryCondition="false" constant="false" fbc:chemicalFormula="C5H9O4"/>
<species metaid="M_23dhmp_c" id="M_23dhmp_c"
name="(R)__2,3__Dihydroxy__3__methylpentanoate" compartment="c" charge="-1"

initialConcentration="0" hasOnlySubstanceUnits="false" boundaryCondition="false" constant="false" fbc:chemicalFormula="C6H11O4"/>
<species metaid="M_23dmphol_c" id="M_23dmphol_c" name="2,3__dimethyl__6__phytylbenzene__1,4__diol" compartment="c" charge="0" initialConcentration="0" hasOnlySubstanceUnits="false" boundaryCondition="false" constant="false" fbc:chemicalFormula="C28H48O2"/>
<species metaid="M_25aics_c" id="M_25aics_c" name="(S)__2__[5__Amino__1__(5__phospho__D__ribosyl)imidazole__4__carboxamido]succinate" compartment="c" charge="-4" initialConcentration="0" hasOnlySubstanceUnits="false" boundaryCondition="false" constant="false" fbc:chemicalFormula="C13H15N4O12P"/>
<species metaid="M_25dhpp_c" id="M_25dhpp_c" name="2,5__Diamino__6__hydroxy__4__(5'__phosphoribosylamino)__pyrimidine" compartment="c" charge="-1" initialConcentration="0" hasOnlySubstanceUnits="false" boundaryCondition="false" constant="false" fbc:chemicalFormula="C9H14N5O8P"/>
<species metaid="M_25dthpp_c" id="M_25dthpp_c" name="2,5__diamino__6__ribitylamino__4(3H)__pyrimidinone 5'__phosphate" compartment="c" charge="0" initialConcentration="0" hasOnlySubstanceUnits="false" boundaryCondition="false" constant="false" fbc:chemicalFormula="C9H16N5O8P"/>
<species metaid="M_26dap__LL_c" id="M_26dap__LL_c" name="LL__2,6__Diaminoheptanedioate" compartment="c" charge="0" initialConcentration="0" hasOnlySubstanceUnits="false" boundaryCondition="false" constant="false" fbc:chemicalFormula="C7H14N2O4"/>
<species metaid="M_26dap__M_c" id="M_26dap__M_c" name="meso__2,6__Diaminoheptanedioate" compartment="c" charge="0" initialConcentration="0" hasOnlySubstanceUnits="false" boundaryCondition="false" constant="false" fbc:chemicalFormula="C7H14N2O4"/>
<species metaid="M_26dap__M_e" id="M_26dap__M_e" name="meso__2,6__Diaminoheptanedioate_b" compartment="e" charge="0" initialConcentration="0" hasOnlySubstanceUnits="false" boundaryCondition="true" constant="false" fbc:chemicalFormula="C7H14N2O4"/>
<species metaid="M_33hmeoxobut_c" id="M_33hmeoxobut_c" name="2__Oxo__3__hydroxyisovalerate" compartment="c" charge="-1" initialConcentration="0" hasOnlySubstanceUnits="false" boundaryCondition="false" constant="false" fbc:chemicalFormula="C5H7O4"/>
<species metaid="M_34hpp_c" id="M_34hpp_c" name="3__(4__Hydroxyphenyl)pyruvate" compartment="c" charge="-1" initialConcentration="0" hasOnlySubstanceUnits="false" boundaryCondition="false" constant="false" fbc:chemicalFormula="C9H7O4"/>
<species metaid="M_35oxcoa" id="M_35oxcoa" name="3__hydroxy__5__oxohexanoyl__CoA" compartment="c" charge="-4" initialConcentration="0" hasOnlySubstanceUnits="false" boundaryCondition="false" constant="false" fbc:chemicalFormula="C27H40N7O19P3S"/>
<species metaid="M_44dneu_c" id="M_44dneu_c" name="4_4'__Diaponeurosporene" compartment="c" charge="0" initialConcentration="0" hasOnlySubstanceUnits="false" boundaryCondition="false" constant="false" fbc:chemicalFormula=""/>
<species metaid="M_123oxtACP_c" id="M_123oxtACP_c" name="12__methyl__3__oxo__tetra__decanoyl__ACP" compartment="c" charge="-1" initialConcentration="0" hasOnlySubstanceUnits="false" boundaryCondition="false" constant="false" fbc:chemicalFormula="C26H47N2O9PRS"/>
<species metaid="M_a2as3p" id="M_a2as3p" name="a 2__acyl__sn__glycerol 3__phosphate" compartment="c" charge="-2" initialConcentration="0" hasOnlySubstanceUnits="false" boundaryCondition="false" constant="false" fbc:chemicalFormula="C4H6O7PR"/>

<species metaid="M_aa_c" id="M_aa_c" name="acrylamide" compartment="c"
charge="0" initialConcentration="0" hasOnlySubstanceUnits="false"
boundaryCondition="false" constant="false" fbc:chemicalFormula="C3H5NO"/>
<species metaid="M_aacald_c" id="M_aacald_c" name="Aminoacetaldehyde"
compartment="c" charge="1" initialConcentration="0"
hasOnlySubstanceUnits="false" boundaryCondition="false" constant="false"
fbc:chemicalFormula="C2H6NO"/>
<species metaid="M_aacoa_c" id="M_aacoa_c" name="Acetoacetyl__CoA"
compartment="c" charge="-4" initialConcentration="0"
hasOnlySubstanceUnits="false" boundaryCondition="false" constant="false"
fbc:chemicalFormula="C25H36N7O18P3S"/>
<species metaid="M_aact_c" id="M_aact_c" name="Aminoacetone"
compartment="c" charge="1" initialConcentration="0"
hasOnlySubstanceUnits="false" boundaryCondition="false" constant="false"
fbc:chemicalFormula="C3H8NO"/>
<species metaid="M_abt_c" id="M_abt_c" name="L__Arabinitol"
compartment="c" charge="0" initialConcentration="0"
hasOnlySubstanceUnits="false" boundaryCondition="false" constant="false"
fbc:chemicalFormula="C5H12O5"/>
<species metaid="M_abt_e" id="M_abt_e" name="L__Arabinitol_b"
compartment="e" charge="0" initialConcentration="0"
hasOnlySubstanceUnits="false" boundaryCondition="true" constant="false"
fbc:chemicalFormula="C5H12O5"/>
<species metaid="M_ac_c" id="M_ac_c" name="Acetate" compartment="c"
charge="-1" initialConcentration="0" hasOnlySubstanceUnits="false"
boundaryCondition="false" constant="false" fbc:chemicalFormula="C2H3O2"/>
<species metaid="M_ac_e" id="M_ac_e" name="Acetate_b" compartment="e"
charge="-1" initialConcentration="0" hasOnlySubstanceUnits="false"
boundaryCondition="true" constant="false" fbc:chemicalFormula="C2H3O2"/>
<species metaid="M_acac_e" id="M_acac_e" name="Acetoacetate_b"
compartment="e" charge="-1" initialConcentration="0"
hasOnlySubstanceUnits="false" boundaryCondition="true" constant="false"
fbc:chemicalFormula="C4H5O3"/>
<species metaid="M_acACP_c" id="M_acACP_c" name="Acetyl__ACP"
compartment="c" charge="0" initialConcentration="0"
hasOnlySubstanceUnits="false" boundaryCondition="false" constant="false"
fbc:chemicalFormula="C13H23N2O8PRS"/>
<species metaid="M_acadl_c" id="M_acadl_c" name="Acetyl_adenylate"
compartment="c" charge="-1" initialConcentration="0"
hasOnlySubstanceUnits="false" boundaryCondition="false" constant="false"
fbc:chemicalFormula=""/>
<species metaid="M_acald_c" id="M_acald_c" name="Acetaldehyde"
compartment="c" charge="0" initialConcentration="0"
hasOnlySubstanceUnits="false" boundaryCondition="false" constant="false"
fbc:chemicalFormula="C2H4O"/>
<species metaid="M_accoa_c" id="M_accoa_c" name="Acetyl__CoA"
compartment="c" charge="-4" initialConcentration="0"
hasOnlySubstanceUnits="false" boundaryCondition="false" constant="false"
fbc:chemicalFormula="C23H34N7O17P3S"/>
<species metaid="M_acg5p_c" id="M_acg5p_c" name="N__Acetyl__L__glutamyl
5__phosphate" compartment="c" charge="-3" initialConcentration="0"
hasOnlySubstanceUnits="false" boundaryCondition="false" constant="false"
fbc:chemicalFormula="C7H9NO8P"/>
<species metaid="M_acg5sa_c" id="M_acg5sa_c"
name="N__Acetyl__L__glutamate 5__semialdehyde" compartment="c" charge="-1"
initialConcentration="0" hasOnlySubstanceUnits="false"
boundaryCondition="false" constant="false" fbc:chemicalFormula="C7H10NO4"/>

```
      <species metaid="M_acgal_c" id="M_acgal_c"
name="N__Acetyl__D__galactosamine" compartment="c" charge="0"
initialConcentration="0" hasOnlySubstanceUnits="false"
boundaryCondition="false" constant="false" fbc:chemicalFormula="C8H15NO6"/>
      <species metaid="M_acgal_e" id="M_acgal_e"
name="N__Acetyl__D__galactosamine_b" compartment="e" charge="0"
initialConcentration="0" hasOnlySubstanceUnits="false"
boundaryCondition="true" constant="false" fbc:chemicalFormula="C8H15NO6"/>
      <species metaid="M_acgam_c" id="M_acgam_c"
name="N__Acetyl__D__glucosamine" compartment="c" charge="0"
initialConcentration="0" hasOnlySubstanceUnits="false"
boundaryCondition="false" constant="false" fbc:chemicalFormula="C8H15NO6"/>
      <species metaid="M_acgam_e" id="M_acgam_e"
name="N__Acetyl__D__glucosamine_b" compartment="e" charge="0"
initialConcentration="0" hasOnlySubstanceUnits="false"
boundaryCondition="true" constant="false" fbc:chemicalFormula="C8H15NO6"/>
      <species metaid="M_acgam1p_c" id="M_acgam1p_c"
name="N__Acetyl__D__glucosamine 1__phosphate" compartment="c" charge="-2"
initialConcentration="0" hasOnlySubstanceUnits="false"
boundaryCondition="false" constant="false" fbc:chemicalFormula="C8H14NO9P"/>
      <species metaid="M_acgam6p_c" id="M_acgam6p_c"
name="N__Acetyl__D__glucosamine 6__phosphate" compartment="c" charge="-2"
initialConcentration="0" hasOnlySubstanceUnits="false"
boundaryCondition="false" constant="false" fbc:chemicalFormula="C8H14NO9P"/>
      <species metaid="M_acglu_c" id="M_acglu_c"
name="N__Acetyl__L__glutamate" compartment="c" charge="-2"
initialConcentration="0" hasOnlySubstanceUnits="false"
boundaryCondition="false" constant="false" fbc:chemicalFormula="C7H9NO5"/>
      <species metaid="M_acgly" id="M_acgly"
name="1__alkenyl__2__acylglycerol" compartment="c" charge="0"
initialConcentration="0" hasOnlySubstanceUnits="false"
boundaryCondition="false" constant="false" fbc:chemicalFormula="C6H8O4R2"/>
      <species metaid="M_achms_c" id="M_achms_c"
name="O__Acetyl__L__homoserine" compartment="c" charge="0"
initialConcentration="0" hasOnlySubstanceUnits="false"
boundaryCondition="false" constant="false" fbc:chemicalFormula="C6H11NO4"/>
      <species metaid="M_acmalt_c" id="M_acmalt_c" name="Acetyl__maltose"
compartment="c" charge="0" initialConcentration="0"
hasOnlySubstanceUnits="false" boundaryCondition="false" constant="false"
fbc:chemicalFormula="C14H24O12"/>
      <species metaid="M_acmam_c" id="M_acmam_c"
name="N__Acetyl__D__muramoate" compartment="c" charge="-1"
initialConcentration="0" hasOnlySubstanceUnits="false"
boundaryCondition="false" constant="false" fbc:chemicalFormula="C11H18NO8"/>
      <species metaid="M_acmama_c" id="M_acmama_c"
name="N__Acetyl__D__muramoyl__L__alanine" compartment="c" charge="-1"
initialConcentration="0" hasOnlySubstanceUnits="false"
boundaryCondition="false" constant="false" fbc:chemicalFormula="C14H23N2O9"/>
      <species metaid="M_acmana_c" id="M_acmana_c"
name="N__Acetyl__D__mannosamine" compartment="c" charge="0"
initialConcentration="0" hasOnlySubstanceUnits="false"
boundaryCondition="false" constant="false" fbc:chemicalFormula="C8H15NO6"/>
      <species metaid="M_acmana_e" id="M_acmana_e"
name="N__Acetyl__D__mannosamine_b" compartment="e" charge="0"
initialConcentration="0" hasOnlySubstanceUnits="false"
boundaryCondition="true" constant="false" fbc:chemicalFormula="C8H15NO6"/>
      <species metaid="M_acmana_p" id="M_acmana_p"
name="N__Acetyl__D__mannosamine" compartment="p" charge="0"
```

```
initialConcentration="0" hasOnlySubstanceUnits="false"
boundaryCondition="false" constant="false" fbc:chemicalFormula="C8H15NO6"/>
      <species metaid="M_acmanap_c" id="M_acmanap_c"
name="N__Acetyl__D__mannosamine 6__phosphate" compartment="c" charge="-2"
initialConcentration="0" hasOnlySubstanceUnits="false"
boundaryCondition="false" constant="false" fbc:chemicalFormula="C8H14NO9P"/>
      <species metaid="M_acmum6p_c" id="M_acmum6p_c" name="N__acetylmuramate
6__phosphate" compartment="c" charge="-3" initialConcentration="0"
hasOnlySubstanceUnits="false" boundaryCondition="false" constant="false"
fbc:chemicalFormula="C11H17NO11P"/>
      <species metaid="M_acnam_c" id="M_acnam_c" name="N__Acetylneuraminate"
compartment="c" charge="-1" initialConcentration="0"
hasOnlySubstanceUnits="false" boundaryCondition="false" constant="false"
fbc:chemicalFormula="C11H18NO9"/>
      <species metaid="M_acnam_e" id="M_acnam_e"
name="N__Acetylneuraminate_b" compartment="e" charge="-1"
initialConcentration="0" hasOnlySubstanceUnits="false"
boundaryCondition="true" constant="false" fbc:chemicalFormula="C11H18NO9"/>
      <species metaid="M_acoa_c" id="M_acoa_c" name="Acyl__CoA"
compartment="c" charge="-4" initialConcentration="0"
hasOnlySubstanceUnits="false" boundaryCondition="false" constant="false"
fbc:chemicalFormula="C22H31N7O17P3RS"/>
      <species metaid="M_acon__C_c" id="M_acon__C_c" name="cis__Aconitate"
compartment="c" charge="-3" initialConcentration="0"
hasOnlySubstanceUnits="false" boundaryCondition="false" constant="false"
fbc:chemicalFormula="C6H3O6"/>
      <species metaid="M_acorn_c" id="M_acorn_c"
name="N2__Acetyl__L__ornithine" compartment="c" charge="0"
initialConcentration="0" hasOnlySubstanceUnits="false"
boundaryCondition="false" constant="false" fbc:chemicalFormula="C7H14N2O3"/>
      <species metaid="M_ACP_c" id="M_ACP_c" name="acyl carrier protein"
compartment="c" charge="-1" initialConcentration="0"
hasOnlySubstanceUnits="false" boundaryCondition="false" constant="false"
fbc:chemicalFormula="C11H21N2O7PRS"/>
      <species metaid="M_acryl_c" id="M_acryl_c" name="Acrylate"
compartment="c" charge="-1" initialConcentration="0"
hasOnlySubstanceUnits="false" boundaryCondition="false" constant="false"
fbc:chemicalFormula="C3H4O2"/>
      <species metaid="M_acser_c" id="M_acser_c" name="O__Acetyl__L__serine"
compartment="c" charge="0" initialConcentration="0"
hasOnlySubstanceUnits="false" boundaryCondition="false" constant="false"
fbc:chemicalFormula="C5H9NO4"/>
      <species metaid="M_actACP_c" id="M_actACP_c" name="Acetoacetyl__ACP"
compartment="c" charge="0" initialConcentration="0"
hasOnlySubstanceUnits="false" boundaryCondition="false" constant="false"
fbc:chemicalFormula="C15H25N2O9PRS"/>
      <species metaid="M_actn__R_c" id="M_actn__R_c" name="(R)__Acetoin"
compartment="c" charge="0" initialConcentration="0"
hasOnlySubstanceUnits="false" boundaryCondition="false" constant="false"
fbc:chemicalFormula="C4H8O2"/>
      <species metaid="M_actn__R_e" id="M_actn__R_e" name="(R)__Acetoin_b"
compartment="e" charge="0" initialConcentration="0"
hasOnlySubstanceUnits="false" boundaryCondition="true" constant="false"
fbc:chemicalFormula="C4H8O2"/>
      <species metaid="M_actp_c" id="M_actp_c" name="Acetyl phosphate"
compartment="c" charge="-2" initialConcentration="0"
hasOnlySubstanceUnits="false" boundaryCondition="false" constant="false"
fbc:chemicalFormula="C2H3O5P"/>
```

<species metaid="M_ad_c" id="M_ad_c" name="acetamide" compartment="c" charge="0" initialConcentration="0" hasOnlySubstanceUnits="false" boundaryCondition="false" constant="false" fbc:chemicalFormula="C2H5NO"/>
<species metaid="M_ad_e" id="M_ad_e" name="acetamide_b" compartment="e" charge="0" initialConcentration="0" hasOnlySubstanceUnits="false" boundaryCondition="true" constant="false" fbc:chemicalFormula="C2H5NO"/>
<species metaid="M_adcobdam_c" id="M_adcobdam_c" name="Adenosyl cobyrinate diamide" compartment="c" charge="-4" initialConcentration="0" hasOnlySubstanceUnits="false" boundaryCondition="false" constant="false" fbc:chemicalFormula="C55H68CoN11O15"/>
<species metaid="M_adcobhex_c" id="M_adcobhex_c" name="adenosyl__cobyric acid" compartment="c" charge="0" initialConcentration="0" hasOnlySubstanceUnits="false" boundaryCondition="false" constant="false" fbc:chemicalFormula="C55H76CoN15O11"/>
<species metaid="M_ade_c" id="M_ade_c" name="Adenine" compartment="c" charge="0" initialConcentration="0" hasOnlySubstanceUnits="false" boundaryCondition="false" constant="false" fbc:chemicalFormula="C5H5N5"/>
<species metaid="M_ade_e" id="M_ade_e" name="Adenine_b" compartment="e" charge="0" initialConcentration="0" hasOnlySubstanceUnits="false" boundaryCondition="true" constant="false" fbc:chemicalFormula="C5H5N5"/>
<species metaid="M_adhlam_c" id="M_adhlam_c" name="S__Acetyldihydrolipoamide" compartment="c" charge="0" initialConcentration="0" hasOnlySubstanceUnits="false" boundaryCondition="false" constant="false" fbc:chemicalFormula="C10H19NO2S2"/>
<species metaid="M_adn_c" id="M_adn_c" name="Adenosine" compartment="c" charge="0" initialConcentration="0" hasOnlySubstanceUnits="false" boundaryCondition="false" constant="false" fbc:chemicalFormula="C10H13N5O4"/>
<species metaid="M_adn_e" id="M_adn_e" name="Adenosine_b" compartment="e" charge="0" initialConcentration="0" hasOnlySubstanceUnits="false" boundaryCondition="true" constant="false" fbc:chemicalFormula="C10H13N5O4"/>
<species metaid="M_adp_c" id="M_adp_c" name="ADP" compartment="c" charge="-3" initialConcentration="0" hasOnlySubstanceUnits="false" boundaryCondition="false" constant="false" fbc:chemicalFormula="C10H12N5O10P2"/>
<species metaid="M_adpglc_c" id="M_adpglc_c" name="ADPglucose" compartment="c" charge="-2" initialConcentration="0" hasOnlySubstanceUnits="false" boundaryCondition="false" constant="false" fbc:chemicalFormula="C16H23N5O15P2"/>
<species metaid="M_adphep__DD_c" id="M_adphep__DD_c" name="ADP__D__glycero__D__manno__heptose" compartment="c" charge="-2" initialConcentration="0" hasOnlySubstanceUnits="false" boundaryCondition="false" constant="false" fbc:chemicalFormula="C17H25N5O16P2"/>
<species metaid="M_adphep__LD_c" id="M_adphep__LD_c" name="ADP__L__glycero__D__manno__heptose" compartment="c" charge="-2" initialConcentration="0" hasOnlySubstanceUnits="false" boundaryCondition="false" constant="false" fbc:chemicalFormula="C17H25N5O16P2"/>
<species metaid="M_adprib_c" id="M_adprib_c" name="ADPribose" compartment="c" charge="-2" initialConcentration="0" hasOnlySubstanceUnits="false" boundaryCondition="false" constant="false" fbc:chemicalFormula="C15H21N5O14P2"/>
<species metaid="M_agm_e" id="M_agm_e" name="Agmatine_b" compartment="e" charge="2" initialConcentration="0"

hasOnlySubstanceUnits="false" boundaryCondition="true" constant="false"
fbc:chemicalFormula="C5H16N4"/>
<species metaid="M_ah6p__D_c" id="M_ah6p__D_c"
name="Arabino__3__hexulose__6__P" compartment="c" charge="-1"
initialConcentration="0" hasOnlySubstanceUnits="false"
boundaryCondition="false" constant="false" fbc:chemicalFormula="C6H11O9P"/>
<species metaid="M_ahcys_c" id="M_ahcys_c"
name="S__Adenosyl__L__homocysteine" compartment="c" charge="0"
initialConcentration="0" hasOnlySubstanceUnits="false"
boundaryCondition="false" constant="false"
fbc:chemicalFormula="C14H20N6O5S"/>
<species metaid="M_ahdt_c" id="M_ahdt_c"
name="2__Amino__4__hydroxy__6__(erythro__1,2,3__trihydroxypropyl)dihydropteri
dine triphosphate" compartment="c" charge="-4" initialConcentration="0"
hasOnlySubstanceUnits="false" boundaryCondition="false" constant="false"
fbc:chemicalFormula="C9H12N5O13P3"/>
<species metaid="M_aicar_c" id="M_aicar_c"
name="5__Amino__1__(5__Phospho__D__ribosyl)imidazole__4__carboxamide"
compartment="c" charge="-2" initialConcentration="0"
hasOnlySubstanceUnits="false" boundaryCondition="false" constant="false"
fbc:chemicalFormula="C9H13N4O8P"/>
<species metaid="M_air_c" id="M_air_c"
name="5__amino__1__(5__phospho__D__ribosyl)imidazole" compartment="c"
charge="-1" initialConcentration="0" hasOnlySubstanceUnits="false"
boundaryCondition="false" constant="false" fbc:chemicalFormula="C8H12N3O7P"/>
<species metaid="M_akg_c" id="M_akg_c" name="2__Oxoglutarate"
compartment="c" charge="-2" initialConcentration="0"
hasOnlySubstanceUnits="false" boundaryCondition="false" constant="false"
fbc:chemicalFormula="C5H4O5"/>
<species metaid="M_akg_e" id="M_akg_e" name="2__Oxoglutarate_b"
compartment="e" charge="-2" initialConcentration="0"
hasOnlySubstanceUnits="false" boundaryCondition="true" constant="false"
fbc:chemicalFormula="C5H4O5"/>
<species metaid="M_ala__B_c" id="M_ala__B_c" name="beta__Alanine"
compartment="c" charge="0" initialConcentration="0"
hasOnlySubstanceUnits="false" boundaryCondition="false" constant="false"
fbc:chemicalFormula="C3H7NO2"/>
<species metaid="M_ala__D_c" id="M_ala__D_c" name="D__Alanine"
compartment="c" charge="0" initialConcentration="0"
hasOnlySubstanceUnits="false" boundaryCondition="false" constant="false"
fbc:chemicalFormula="C3H7NO2"/>
<species metaid="M_ala__D_e" id="M_ala__D_e" name="D__Alanine_b"
compartment="e" charge="0" initialConcentration="0"
hasOnlySubstanceUnits="false" boundaryCondition="true" constant="false"
fbc:chemicalFormula="C3H7NO2"/>
<species metaid="M_ala__L_asp__L_c" id="M_ala__L_asp__L_c"
name="ala__L__asp__L" compartment="c" charge="-1" initialConcentration="0"
hasOnlySubstanceUnits="false" boundaryCondition="false" constant="false"
fbc:chemicalFormula="C7H11N2O5"/>
<species metaid="M_ala__L_asp__L_e" id="M_ala__L_asp__L_e"
name="ala__L__asp__L_b" compartment="e" charge="-1" initialConcentration="0"
hasOnlySubstanceUnits="false" boundaryCondition="true" constant="false"
fbc:chemicalFormula="C7H11N2O5"/>
<species metaid="M_ala__L_c" id="M_ala__L_c" name="L__Alanine"
compartment="c" charge="0" initialConcentration="0"
hasOnlySubstanceUnits="false" boundaryCondition="false" constant="false"
fbc:chemicalFormula="C3H7NO2"/>

<species metaid="M_ala__L_e" id="M_ala__L_e" name="L__Alanine_b" compartment="e" charge="0" initialConcentration="0" hasOnlySubstanceUnits="false" boundaryCondition="true" constant="false" fbc:chemicalFormula="C3H7NO2"/>
<species metaid="M_ala__L_glu__L_c" id="M_ala__L_glu__L_c" name="ala__L__glu__L" compartment="c" charge="-1" initialConcentration="0" hasOnlySubstanceUnits="false" boundaryCondition="false" constant="false" fbc:chemicalFormula="C8H13N2O5"/>
<species metaid="M_ala__L_glu__L_e" id="M_ala__L_glu__L_e" name="ala__L__glu__L_b" compartment="e" charge="-1" initialConcentration="0" hasOnlySubstanceUnits="false" boundaryCondition="true" constant="false" fbc:chemicalFormula="C8H13N2O5"/>
<species metaid="M_ala__L_thr__L_c" id="M_ala__L_thr__L_c" name="ala__L__Thr__L" compartment="c" charge="0" initialConcentration="0" hasOnlySubstanceUnits="false" boundaryCondition="false" constant="false" fbc:chemicalFormula="C7H14N2O4"/>
<species metaid="M_ala__L_thr__L_e" id="M_ala__L_thr__L_e" name="ala__L__Thr__L_b" compartment="e" charge="0" initialConcentration="0" hasOnlySubstanceUnits="false" boundaryCondition="true" constant="false" fbc:chemicalFormula="C7H14N2O4"/>
<species metaid="M_ala_gln_c" id="M_ala_gln_c" name="Ala__Gln" compartment="c" charge="0" initialConcentration="0" hasOnlySubstanceUnits="false" boundaryCondition="false" constant="false" fbc:chemicalFormula="C8H15N3O4"/>
<species metaid="M_ala_gln_e" id="M_ala_gln_e" name="Ala__Gln_b" compartment="e" charge="0" initialConcentration="0" hasOnlySubstanceUnits="false" boundaryCondition="true" constant="false" fbc:chemicalFormula="C8H15N3O4"/>
<species metaid="M_ala_his_c" id="M_ala_his_c" name="Ala__His" compartment="c" charge="0" initialConcentration="0" hasOnlySubstanceUnits="false" boundaryCondition="false" constant="false" fbc:chemicalFormula="C9H14N4O3"/>
<species metaid="M_ala_his_e" id="M_ala_his_e" name="Ala__His_b" compartment="e" charge="0" initialConcentration="0" hasOnlySubstanceUnits="false" boundaryCondition="true" constant="false" fbc:chemicalFormula="C9H14N4O3"/>
<species metaid="M_ala_leu_c" id="M_ala_leu_c" name="Ala__Leu" compartment="c" charge="0" initialConcentration="0" hasOnlySubstanceUnits="false" boundaryCondition="false" constant="false" fbc:chemicalFormula="C9H18N2O3"/>
<species metaid="M_ala_leu_e" id="M_ala_leu_e" name="Ala__Leu_b" compartment="e" charge="0" initialConcentration="0" hasOnlySubstanceUnits="false" boundaryCondition="true" constant="false" fbc:chemicalFormula="C9H18N2O3"/>
<species metaid="M_alaACP_c" id="M_alaACP_c" name=" D__alanyl__[D__alanyl carrier protein]" compartment="c" charge="1" initialConcentration="0" hasOnlySubstanceUnits="false" boundaryCondition="false" constant="false" fbc:chemicalFormula="C14H27N3O8PRS"/>
<species metaid="M_alaala_c" id="M_alaala_c" name="D__Alanyl__D__alanine" compartment="c" charge="0" initialConcentration="0" hasOnlySubstanceUnits="false" boundaryCondition="false" constant="false" fbc:chemicalFormula="C6H12N2O3"/>
<species metaid="M_alac__S_c" id="M_alac__S_c" name="(S)__2__Acetolactate" compartment="c" charge="-1" initialConcentration="0" hasOnlySubstanceUnits="false" boundaryCondition="false" constant="false" fbc:chemicalFormula="C5H7O4"/>

<species metaid="M_alagly_c" id="M_alagly_c" name="L__alanylglycine"
compartment="c" charge="0" initialConcentration="0"
hasOnlySubstanceUnits="false" boundaryCondition="false" constant="false"
fbc:chemicalFormula="C5H10N2O3"/>
<species metaid="M_alagly_e" id="M_alagly_e" name="L__alanylglycine_b"
compartment="e" charge="0" initialConcentration="0"
hasOnlySubstanceUnits="false" boundaryCondition="true" constant="false"
fbc:chemicalFormula="C5H10N2O3"/>
<species metaid="M_alatrna_c" id="M_alatrna_c"
name="L__Alanyl__tRNA(Ala)" compartment="c" charge="1"
initialConcentration="0" hasOnlySubstanceUnits="false"
boundaryCondition="false" constant="false" fbc:chemicalFormula="C3H6NOR"/>
<species metaid="M_all__D_e" id="M_all__D_e" name="D__Allose_b"
compartment="e" charge="0" initialConcentration="0"
hasOnlySubstanceUnits="false" boundaryCondition="true" constant="false"
fbc:chemicalFormula="C6H12O6"/>
<species metaid="M_allphn_c" id="M_allphn_c" name="Allophanate"
compartment="c" charge="-1" initialConcentration="0"
hasOnlySubstanceUnits="false" boundaryCondition="false" constant="false"
fbc:chemicalFormula="C2H3N2O3"/>
<species metaid="M_alltn_e" id="M_alltn_e" name="Allantoin_b"
compartment="e" charge="0" initialConcentration="0"
hasOnlySubstanceUnits="false" boundaryCondition="true" constant="false"
fbc:chemicalFormula="C4H6N4O3"/>
<species metaid="M_alpro_c" id="M_alpro_c"
name="S__Aminomethyldihydrolipoylprotein" compartment="c" charge="1"
initialConcentration="0" hasOnlySubstanceUnits="false"
boundaryCondition="false" constant="false" fbc:chemicalFormula="CH6NS2X"/>
<species metaid="M_amdglc_c" id="M_amdglc_c"
name="alpha__Methyl__D__glucoside" compartment="c" charge="0"
initialConcentration="0" hasOnlySubstanceUnits="false"
boundaryCondition="false" constant="false" fbc:chemicalFormula="C7H14O6"/>
<species metaid="M_amet_c" id="M_amet_c"
name="S__Adenosyl__L__methionine" compartment="c" charge="1"
initialConcentration="0" hasOnlySubstanceUnits="false"
boundaryCondition="false" constant="false"
fbc:chemicalFormula="C15H23N6O5S"/>
<species metaid="M_amob_c" id="M_amob_c"
name="S__Adenosyl__4__methylthio__2__oxobutanoate" compartment="c" charge="0"
initialConcentration="0" hasOnlySubstanceUnits="false"
boundaryCondition="false" constant="false"
fbc:chemicalFormula="C15H19N5O6S"/>
<species metaid="M_amp_c" id="M_amp_c" name="AMP" compartment="c"
charge="-2" initialConcentration="0" hasOnlySubstanceUnits="false"
boundaryCondition="false" constant="false"
fbc:chemicalFormula="C10H12N5O7P"/>
<species metaid="M_amp_e" id="M_amp_e" name="AMP_b" compartment="e"
charge="-2" initialConcentration="0" hasOnlySubstanceUnits="false"
boundaryCondition="true" constant="false" fbc:chemicalFormula="C10H12N5O7P"/>
<species metaid="M_anipcard_c" id="M_anipcard_c"
name="Anteisopentadecanoylcardiolipin_B._subtilis" compartment="c" charge="-
2" initialConcentration="0" hasOnlySubstanceUnits="false"
boundaryCondition="false" constant="false" fbc:chemicalFormula=""/>
<species metaid="M_ant24ds_c" id="M_ant24ds_c"
name="Anteisoheptadecanoyllipoteichoic_acid_n=24__linked__D__alanine_substitu
ted" compartment="c" charge="-24" initialConcentration="0"
hasOnlySubstanceUnits="false" boundaryCondition="false" constant="false"
fbc:chemicalFormula=""/>

<species metaid="M_ant24glcs_c" id="M_ant24glcs_c"
name="Anteisopentadecanoyllipoteichoic_acid_n=24__linked__glucose_substituted
" compartment="c" charge="-24" initialConcentration="0"
hasOnlySubstanceUnits="false" boundaryCondition="false" constant="false"
fbc:chemicalFormula=""/>
<species metaid="M_ant24Nacds_c" id="M_ant24Nacds_c"
name="Anteisoheptadecanoyllipoteichoic_acid_n=24__linked__N__acetyl__D__gluco
samine" compartment="c" charge="-24" initialConcentration="0"
hasOnlySubstanceUnits="false" boundaryCondition="false" constant="false"
fbc:chemicalFormula=""/>
<species metaid="M_ant24u_c" id="M_ant24u_c"
name="Anteisoheptadecanoyllipoteichoic_acid_n=24__linked__unsubstituted"
compartment="c" charge="-24" initialConcentration="0"
hasOnlySubstanceUnits="false" boundaryCondition="false" constant="false"
fbc:chemicalFormula=""/>
<species metaid="M_antcard_c" id="M_antcard_c"
name="Anteisoheptadecanoylcardiolipin_B._subtilis" compartment="c" charge="-
2" initialConcentration="0" hasOnlySubstanceUnits="false"
boundaryCondition="false" constant="false" fbc:chemicalFormula=""/>
<species metaid="M_anth_c" id="M_anth_c" name="Anthranilate"
compartment="c" charge="-1" initialConcentration="0"
hasOnlySubstanceUnits="false" boundaryCondition="false" constant="false"
fbc:chemicalFormula="C7H6NO2"/>
<species metaid="M_antl24ds_c" id="M_antl24ds_c"
name="Anteisopentadecanoyllipoteichoic_acid_n=24__linked__D__alanine_substitu
ted" compartment="c" charge="-23" initialConcentration="0"
hasOnlySubstanceUnits="false" boundaryCondition="false" constant="false"
fbc:chemicalFormula=""/>
<species metaid="M_antla24s_c" id="M_antla24s_c"
name="Anteisoheptadecanoyllipoteichoic_acid_n=24__linked__glucose_substituted
" compartment="c" charge="-24" initialConcentration="0"
hasOnlySubstanceUnits="false" boundaryCondition="false" constant="false"
fbc:chemicalFormula=""/>
<species metaid="M_antp24s_c" id="M_antp24s_c"
name="Anteisopentadecanoyllipoteichoic_acid_n=24__linked__N__acetyl__D__gluco
samine" compartment="c" charge="-24" initialConcentration="0"
hasOnlySubstanceUnits="false" boundaryCondition="false" constant="false"
fbc:chemicalFormula=""/>
<species metaid="M_antp24u_c" id="M_antp24u_c"
name="Anteisopentadecanoyllipoteichoic_acid_n=24__linked__unsubstituted"
compartment="c" charge="-24" initialConcentration="0"
hasOnlySubstanceUnits="false" boundaryCondition="false" constant="false"
fbc:chemicalFormula=""/>
<species metaid="M_apoACP_c" id="M_apoACP_c" name="apoprotein [acyl
carrier protein]" compartment="c" charge="0" initialConcentration="0"
hasOnlySubstanceUnits="false" boundaryCondition="false" constant="false"
fbc:chemicalFormula="RHO"/>
<species metaid="M_aproa_c" id="M_aproa_c" name="3__Aminopropanal"
compartment="c" charge="0" initialConcentration="0"
hasOnlySubstanceUnits="false" boundaryCondition="false" constant="false"
fbc:chemicalFormula="C3H8NO"/>
<species metaid="M_aprut_c" id="M_aprut_c" name="N__Acetylputrescine"
compartment="c" charge="0" initialConcentration="0"
hasOnlySubstanceUnits="false" boundaryCondition="false" constant="false"
fbc:chemicalFormula="C6H15N2O"/>
<species metaid="M_aps_c" id="M_aps_c" name="Adenosine
5'__phosphosulfate" compartment="c" charge="-2" initialConcentration="0"

hasOnlySubstanceUnits="false" boundaryCondition="false" constant="false" fbc:chemicalFormula="C10H12N5O10PS"/>
<species metaid="M_arab__L_c" id="M_arab__L_c" name="L__Arabinose" compartment="c" charge="0" initialConcentration="0" hasOnlySubstanceUnits="false" boundaryCondition="false" constant="false" fbc:chemicalFormula="C5H10O5"/>
<species metaid="M_arab__L_e" id="M_arab__L_e" name="L__Arabinose_b" compartment="e" charge="0" initialConcentration="0" hasOnlySubstanceUnits="false" boundaryCondition="true" constant="false" fbc:chemicalFormula="C5H10O5"/>
<species metaid="M_arbt_c" id="M_arbt_c" name="hydroquinone O__beta__D__glucopyranoside" compartment="c" charge="0" initialConcentration="0" hasOnlySubstanceUnits="false" boundaryCondition="false" constant="false" fbc:chemicalFormula="C12H16O7"/>
<species metaid="M_arbt_e" id="M_arbt_e" name="Arbutin_b" compartment="e" charge="0" initialConcentration="0" hasOnlySubstanceUnits="false" boundaryCondition="true" constant="false" fbc:chemicalFormula="C12H16O7"/>
<species metaid="M_arbt6p_c" id="M_arbt6p_c" name="Arbutin 6__phosphate" compartment="c" charge="-2" initialConcentration="0" hasOnlySubstanceUnits="false" boundaryCondition="false" constant="false" fbc:chemicalFormula="C12H15O10P"/>
<species metaid="M_arg__L_c" id="M_arg__L_c" name="L__Arginine" compartment="c" charge="1" initialConcentration="0" hasOnlySubstanceUnits="false" boundaryCondition="false" constant="false" fbc:chemicalFormula="C6H15N4O2"/>
<species metaid="M_arg__L_e" id="M_arg__L_e" name="L__Arginine_b" compartment="e" charge="1" initialConcentration="0" hasOnlySubstanceUnits="false" boundaryCondition="true" constant="false" fbc:chemicalFormula="C6H15N4O2"/>
<species metaid="M_argsuc_c" id="M_argsuc_c" name="N(omega)__(L__Arginino)succinate" compartment="c" charge="-1" initialConcentration="0" hasOnlySubstanceUnits="false" boundaryCondition="false" constant="false" fbc:chemicalFormula="C10H17N4O6"/>
<species metaid="M_argtrna_c" id="M_argtrna_c" name="L__Arginyl__tRNA(Arg)" compartment="c" charge="2" initialConcentration="0" hasOnlySubstanceUnits="false" boundaryCondition="false" constant="false" fbc:chemicalFormula="C6H14N4OR"/>
<species metaid="M_arsbet_c" id="M_arsbet_c" name="ARSENOBETAINE" compartment="c" charge="0" initialConcentration="0" hasOnlySubstanceUnits="false" boundaryCondition="false" constant="false" fbc:chemicalFormula="C5H11AsO2"/>
<species metaid="M_arsbet_e" id="M_arsbet_e" name="ARSENOBETAINE_b" compartment="e" charge="0" initialConcentration="0" hasOnlySubstanceUnits="false" boundaryCondition="true" constant="false" fbc:chemicalFormula="C5H11AsO2"/>
<species metaid="M_ascb6p_c" id="M_ascb6p_c" name="L__ascorbate__6__phosphate" compartment="c" charge="-3" initialConcentration="0" hasOnlySubstanceUnits="false" boundaryCondition="false" constant="false" fbc:chemicalFormula="C6H7O9P"/>
<species metaid="M_asn__L_c" id="M_asn__L_c" name="L__Asparagine" compartment="c" charge="0" initialConcentration="0" hasOnlySubstanceUnits="false" boundaryCondition="false" constant="false" fbc:chemicalFormula="C4H8N2O3"/>
<species metaid="M_asn__L_e" id="M_asn__L_e" name="L__Asparagine_b" compartment="e" charge="0" initialConcentration="0" hasOnlySubstanceUnits="false" boundaryCondition="true" constant="false" fbc:chemicalFormula="C4H8N2O3"/>

<species metaid="M_asntrna_c" id="M_asntrna_c" name="L__Asparaginyl__tRNA(Asn)" compartment="c" charge="1" initialConcentration="0" hasOnlySubstanceUnits="false" boundaryCondition="false" constant="false" fbc:chemicalFormula="C14H24N2O12PR2"/>
<species metaid="M_aso3_c" id="M_aso3_c" name="arsenite" compartment="c" charge="-1" initialConcentration="0" hasOnlySubstanceUnits="false" boundaryCondition="false" constant="false" fbc:chemicalFormula="AsO3"/>
<species metaid="M_aso3_e" id="M_aso3_e" name="arsenite_b" compartment="e" charge="-1" initialConcentration="0" hasOnlySubstanceUnits="false" boundaryCondition="true" constant="false" fbc:chemicalFormula="AsO3"/>
<species metaid="M_aso4_c" id="M_aso4_c" name="arsenate" compartment="c" charge="-2" initialConcentration="0" hasOnlySubstanceUnits="false" boundaryCondition="false" constant="false" fbc:chemicalFormula="AsO4"/>
<species metaid="M_aso4_e" id="M_aso4_e" name="arsenate_b" compartment="e" charge="-2" initialConcentration="0" hasOnlySubstanceUnits="false" boundaryCondition="true" constant="false" fbc:chemicalFormula="AsO4"/>
<species metaid="M_asp__L_c" id="M_asp__L_c" name="L__Aspartate" compartment="c" charge="-1" initialConcentration="0" hasOnlySubstanceUnits="false" boundaryCondition="false" constant="false" fbc:chemicalFormula="C4H6NO4"/>
<species metaid="M_asp__L_e" id="M_asp__L_e" name="L__Aspartate_b" compartment="e" charge="-1" initialConcentration="0" hasOnlySubstanceUnits="false" boundaryCondition="true" constant="false" fbc:chemicalFormula="C4H6NO4"/>
<species metaid="M_aspsa_c" id="M_aspsa_c" name="L__Aspartate 4__semialdehyde" compartment="c" charge="0" initialConcentration="0" hasOnlySubstanceUnits="false" boundaryCondition="false" constant="false" fbc:chemicalFormula="C4H7NO3"/>
<species metaid="M_asptrna_c" id="M_asptrna_c" name="L__Aspartyl__tRNA(Asp)" compartment="c" charge="0" initialConcentration="0" hasOnlySubstanceUnits="false" boundaryCondition="false" constant="false" fbc:chemicalFormula="C4H5NO3R"/>
<species metaid="M_atp_c" id="M_atp_c" name="ATP" compartment="c" charge="-4" initialConcentration="0" hasOnlySubstanceUnits="false" boundaryCondition="false" constant="false" fbc:chemicalFormula="C10H12N5O13P3"/>
<species metaid="M_b2coa_c" id="M_b2coa_c" name="Crotonoyl__CoA" compartment="c" charge="-4" initialConcentration="0" hasOnlySubstanceUnits="false" boundaryCondition="false" constant="false" fbc:chemicalFormula="C25H36N7O17P3S"/>
<species metaid="M_b12_e" id="M_b12_e" name="Vitamin_B12_b" compartment="e" charge="6" initialConcentration="0" hasOnlySubstanceUnits="false" boundaryCondition="true" constant="false" fbc:chemicalFormula=""/>
<species metaid="M_benzcoa_c" id="M_benzcoa_c" name="Benzoyl__CoA" compartment="c" charge="-4" initialConcentration="0" hasOnlySubstanceUnits="false" boundaryCondition="false" constant="false" fbc:chemicalFormula="C28H40N7O17P3S"/>
<species metaid="M_betald_c" id="M_betald_c" name="Betaine aldehyde" compartment="c" charge="1" initialConcentration="0" hasOnlySubstanceUnits="false" boundaryCondition="false" constant="false" fbc:chemicalFormula="C5H12NO"/>

<species metaid="M_bgly_c" id="M_bgly_c" name="N__Benzoylglycine" compartment="c" charge="-1" initialConcentration="0" hasOnlySubstanceUnits="false" boundaryCondition="false" constant="false" fbc:chemicalFormula="C9H8NO3"/>
<species metaid="M_btbet_c" id="M_btbet_c" name="Butyro__betaine" compartment="c" charge="1" initialConcentration="0" hasOnlySubstanceUnits="false" boundaryCondition="false" constant="false" fbc:chemicalFormula="C8H18NO"/>
<species metaid="M_btbet_e" id="M_btbet_e" name="Butyro__betaine_b" compartment="e" charge="1" initialConcentration="0" hasOnlySubstanceUnits="false" boundaryCondition="true" constant="false" fbc:chemicalFormula="C8H18NO"/>
<species metaid="M_btcoa_c" id="M_btcoa_c" name="Butanoyl__CoA" compartment="c" charge="-4" initialConcentration="0" hasOnlySubstanceUnits="false" boundaryCondition="false" constant="false" fbc:chemicalFormula="C25H38N7O17P3S"/>
<species metaid="M_btd_RR_c" id="M_btd_RR_c" name=" R R 2 3 Butanediol C4H10O2" compartment="c" charge="0" initialConcentration="0" hasOnlySubstanceUnits="false" boundaryCondition="false" constant="false" fbc:chemicalFormula="C4H10O2"/>
<species metaid="M_btd_RR_e" id="M_btd_RR_e" name=" R R 2 3 Butanediol C4H10O2_b" compartment="e" charge="0" initialConcentration="0" hasOnlySubstanceUnits="false" boundaryCondition="true" constant="false" fbc:chemicalFormula="C4H10O2"/>
<species metaid="M_btn_c" id="M_btn_c" name="Biotin" compartment="c" charge="-1" initialConcentration="0" hasOnlySubstanceUnits="false" boundaryCondition="false" constant="false" fbc:chemicalFormula="C10H15N2O3S"/>
<species metaid="M_btn_e" id="M_btn_e" name="Biotin_b" compartment="e" charge="-1" initialConcentration="0" hasOnlySubstanceUnits="false" boundaryCondition="true" constant="false" fbc:chemicalFormula="C10H15N2O3S"/>
<species metaid="M_but_e" id="M_but_e" name="Butyrate (n__C4:0)_b" compartment="e" charge="-1" initialConcentration="0" hasOnlySubstanceUnits="false" boundaryCondition="true" constant="false" fbc:chemicalFormula="C4H7O2"/>
<species metaid="M_but2eACP_c" id="M_but2eACP_c" name="But__2__enoyl__[acyl__carrier protein]" compartment="c" charge="-1" initialConcentration="0" hasOnlySubstanceUnits="false" boundaryCondition="false" constant="false" fbc:chemicalFormula="C15H25N2O8PRS"/>
<species metaid="M_butACP_c" id="M_butACP_c" name="Butyryl__ACP (n__C4:0ACP)" compartment="c" charge="0" initialConcentration="0" hasOnlySubstanceUnits="false" boundaryCondition="false" constant="false" fbc:chemicalFormula="C15H27N2O8PRS"/>
<species metaid="M_butso3_e" id="M_butso3_e" name="butanesulfonate_b" compartment="e" charge="-1" initialConcentration="0" hasOnlySubstanceUnits="false" boundaryCondition="true" constant="false" fbc:chemicalFormula="C4H9O3S"/>
<species metaid="M_bz_c" id="M_bz_c" name="Benzoate" compartment="c" charge="-1" initialConcentration="0" hasOnlySubstanceUnits="false" boundaryCondition="false" constant="false" fbc:chemicalFormula="C7H5O2"/>
<species metaid="M_bzmd_c" id="M_bzmd_c" name="benzamide" compartment="c" charge="0" initialConcentration="0" hasOnlySubstanceUnits="false" boundaryCondition="false" constant="false" fbc:chemicalFormula="C7H7NO"/>
<species metaid="M_c12dihgly_c" id="M_c12dihgly_c" name="CDP__1_2__dianteisoheptadecanoylglycerol" compartment="c" charge="-2"

initialConcentration="0" hasOnlySubstanceUnits="false"
boundaryCondition="false" constant="false" fbc:chemicalFormula=""/>
<species metaid="M_c12diidgly_c" id="M_c12diidgly_c"
name="CDP__1_2__diisohexadecanoylglycerol" compartment="c" charge="-2"
initialConcentration="0" hasOnlySubstanceUnits="false"
boundaryCondition="false" constant="false" fbc:chemicalFormula=""/>
<species metaid="M_c12diigly_c" id="M_c12diigly_c"
name="CDP__1_2__diisoheptadecanoylglycerol" compartment="c" charge="-2"
initialConcentration="0" hasOnlySubstanceUnits="false"
boundaryCondition="false" constant="false" fbc:chemicalFormula=""/>
<species metaid="M_c12dipgly_c" id="M_c12dipgly_c"
name="CDP__1_2__diisopentadecanoylglycerol" compartment="c" charge="-2"
initialConcentration="0" hasOnlySubstanceUnits="false"
boundaryCondition="false" constant="false" fbc:chemicalFormula=""/>
<species metaid="M_c12ditgly_c" id="M_c12ditgly_c"
name="CDP__1_2__diisotetradecanoylglycerol" compartment="c" charge="-2"
initialConcentration="0" hasOnlySubstanceUnits="false"
boundaryCondition="false" constant="false" fbc:chemicalFormula=""/>
<species metaid="M_c12dpdgly_c" id="M_c12dpdgly_c"
name="CDP__1_2__dianteisopentadecanoylglycerol" compartment="c" charge="-2"
initialConcentration="0" hasOnlySubstanceUnits="false"
boundaryCondition="false" constant="false" fbc:chemicalFormula=""/>
<species metaid="M_c15811_c" id="M_c15811_c" name="C15811"
compartment="c" charge="0" initialConcentration="0"
hasOnlySubstanceUnits="false" boundaryCondition="false" constant="false"
fbc:chemicalFormula="C4H6N2O2R2S"/>
<species metaid="M_c15812_c" id="M_c15812_c" name="C15812"
compartment="c" charge="0" initialConcentration="0"
hasOnlySubstanceUnits="false" boundaryCondition="false" constant="false"
fbc:chemicalFormula="C4H6N2O2R2S2"/>
<species metaid="M_ca2_c" id="M_ca2_c" name="Calcium" compartment="c"
charge="2" initialConcentration="0" hasOnlySubstanceUnits="false"
boundaryCondition="false" constant="false" fbc:chemicalFormula="Ca"/>
<species metaid="M_ca2_e" id="M_ca2_e" name="Calcium_b" compartment="e"
charge="2" initialConcentration="0" hasOnlySubstanceUnits="false"
boundaryCondition="true" constant="false" fbc:chemicalFormula="Ca"/>
<species metaid="M_camp_c" id="M_camp_c" name="cAMP" compartment="c"
charge="-1" initialConcentration="0" hasOnlySubstanceUnits="false"
boundaryCondition="false" constant="false"
fbc:chemicalFormula="C10H11N5O6P"/>
<species metaid="M_carP_c" id="M_carP_c"
name="Carboxybiotin__carboxyl__carrier_protein" compartment="c" charge="-1"
initialConcentration="0" hasOnlySubstanceUnits="false"
boundaryCondition="false" constant="false" fbc:chemicalFormula=""/>
<species metaid="M_catechol_c" id="M_catechol_c" name="Catechol"
compartment="c" charge="0" initialConcentration="0"
hasOnlySubstanceUnits="false" boundaryCondition="false" constant="false"
fbc:chemicalFormula="C6H6O2"/>
<species metaid="M_cbasp_c" id="M_cbasp_c"
name="N__Carbamoyl__L__aspartate" compartment="c" charge="-2"
initialConcentration="0" hasOnlySubstanceUnits="false"
boundaryCondition="false" constant="false" fbc:chemicalFormula="C5H6N2O5"/>
<species metaid="M_cbl1_e" id="M_cbl1_e" name="Cob(I)alamin_b"
compartment="e" charge="-1" initialConcentration="0"
hasOnlySubstanceUnits="false" boundaryCondition="true" constant="false"
fbc:chemicalFormula="C62H88CoN13O14P"/>
<species metaid="M_cbl2_e" id="M_cbl2_e" name="cob(II)alamin_b"
compartment="e" charge="0" initialConcentration="0"

```
hasOnlySubstanceUnits="false" boundaryCondition="true" constant="false"
fbc:chemicalFormula="C62H88N13O14PCo"/>
      <species metaid="M_cbp_c" id="M_cbp_c" name="Carbamoyl phosphate"
compartment="c" charge="-2" initialConcentration="0"
hasOnlySubstanceUnits="false" boundaryCondition="false" constant="false"
fbc:chemicalFormula="CH2NO5P"/>
      <species metaid="M_cchoxod_c" id="M_cchoxod_c"
name="2__Hydroxy__6__keto__2,4__heptadienoate" compartment="c" charge="-1"
initialConcentration="0" hasOnlySubstanceUnits="false"
boundaryCondition="false" constant="false" fbc:chemicalFormula="C7H7O4"/>
      <species metaid="M_cd2_e" id="M_cd2_e" name="Cadmium_b" compartment="e"
charge="2" initialConcentration="0" hasOnlySubstanceUnits="false"
boundaryCondition="true" constant="false" fbc:chemicalFormula="Cd"/>
      <species metaid="M_cdp_c" id="M_cdp_c" name="CDP" compartment="c"
charge="-3" initialConcentration="0" hasOnlySubstanceUnits="false"
boundaryCondition="false" constant="false"
fbc:chemicalFormula="C9H12N3O11P2"/>
      <species metaid="M_cdpdag_SA_c" id="M_cdpdag_SA_c"
name="CDPdiacylglycerol (Saureus)" compartment="c" charge="-100"
initialConcentration="0" hasOnlySubstanceUnits="false"
boundaryCondition="false" constant="false"
fbc:chemicalFormula="C2010H3615N150O542P100"/>
      <species metaid="M_cdpdddecg_c" id="M_cdpdddecg_c"
name="CDP__1,2__didodecanoylglycerol" compartment="c" charge="-2"
initialConcentration="0" hasOnlySubstanceUnits="false"
boundaryCondition="false" constant="false"
fbc:chemicalFormula="C36H63N3O15P2"/>
      <species metaid="M_cdpdhdec9eg_c" id="M_cdpdhdec9eg_c"
name="CDP__1,2__dihexadec__9__enoylglycerol" compartment="c" charge="-2"
initialConcentration="0" hasOnlySubstanceUnits="false"
boundaryCondition="false" constant="false"
fbc:chemicalFormula="C44H75N3O15P2"/>
      <species metaid="M_cdpdhdecg_c" id="M_cdpdhdecg_c"
name="CDP__1,2__dihexadecanoylglycerol" compartment="c" charge="-2"
initialConcentration="0" hasOnlySubstanceUnits="false"
boundaryCondition="false" constant="false"
fbc:chemicalFormula="C44H79N3O15P2"/>
      <species metaid="M_cdpdodec11eg_c" id="M_cdpdodec11eg_c"
name="CDP__1,2__dioctadec__11__enoylglycerol" compartment="c" charge="-2"
initialConcentration="0" hasOnlySubstanceUnits="false"
boundaryCondition="false" constant="false"
fbc:chemicalFormula="C48H83N3O15P2"/>
      <species metaid="M_cdpdodecg_c" id="M_cdpdodecg_c"
name="CDP__1,2__dioctadecanoylglycerol" compartment="c" charge="-2"
initialConcentration="0" hasOnlySubstanceUnits="false"
boundaryCondition="false" constant="false"
fbc:chemicalFormula="C48H87N3O15P2"/>
      <species metaid="M_cdpdtdec7eg_c" id="M_cdpdtdec7eg_c"
name="CDP__1,2__ditetradec__7__enoylglycerol" compartment="c" charge="-2"
initialConcentration="0" hasOnlySubstanceUnits="false"
boundaryCondition="false" constant="false"
fbc:chemicalFormula="C40H67N3O15P2"/>
      <species metaid="M_cdpdtdecg_c" id="M_cdpdtdecg_c"
name="CDP__1,2__ditetradecanoylglycerol" compartment="c" charge="-2"
initialConcentration="0" hasOnlySubstanceUnits="false"
boundaryCondition="false" constant="false"
fbc:chemicalFormula="C40H71N3O15P2"/>
```

```
      <species metaid="M_cdpglyc_c" id="M_cdpglyc_c" name="CDPglycerol"
compartment="c" charge="-2" initialConcentration="0"
hasOnlySubstanceUnits="false" boundaryCondition="false" constant="false"
fbc:chemicalFormula="C12H19N3O13P2"/>
      <species metaid="M_cdprib_c" id="M_cdprib_c" name="CDP__ribitol"
compartment="c" charge="-2" initialConcentration="0"
hasOnlySubstanceUnits="false" boundaryCondition="false" constant="false"
fbc:chemicalFormula="C14H23N3O15P2"/>
      <species metaid="M_cellb_c" id="M_cellb_c" name="cellobiose"
compartment="c" charge="0" initialConcentration="0"
hasOnlySubstanceUnits="false" boundaryCondition="false" constant="false"
fbc:chemicalFormula="C12H22O11"/>
      <species metaid="M_cellb_e" id="M_cellb_e" name="cellobiose_b"
compartment="e" charge="0" initialConcentration="0"
hasOnlySubstanceUnits="false" boundaryCondition="true" constant="false"
fbc:chemicalFormula="C12H22O11"/>
      <species metaid="M_cgly_c" id="M_cgly_c" name="Cys__Gly"
compartment="c" charge="0" initialConcentration="0"
hasOnlySubstanceUnits="false" boundaryCondition="false" constant="false"
fbc:chemicalFormula="C5H10N2O3S"/>
      <species metaid="M_cgly_e" id="M_cgly_e" name="Cys__Gly_b"
compartment="e" charge="0" initialConcentration="0"
hasOnlySubstanceUnits="false" boundaryCondition="true" constant="false"
fbc:chemicalFormula="C5H10N2O3S"/>
      <species metaid="M_chol_c" id="M_chol_c" name="Choline" compartment="c"
charge="1" initialConcentration="0" hasOnlySubstanceUnits="false"
boundaryCondition="false" constant="false" fbc:chemicalFormula="C5H14NO"/>
      <species metaid="M_chol_e" id="M_chol_e" name="Choline_b"
compartment="e" charge="1" initialConcentration="0"
hasOnlySubstanceUnits="false" boundaryCondition="true" constant="false"
fbc:chemicalFormula="C5H14NO"/>
      <species metaid="M_cholate_c" id="M_cholate_c" name="Cholate"
compartment="c" charge="-1" initialConcentration="0"
hasOnlySubstanceUnits="false" boundaryCondition="false" constant="false"
fbc:chemicalFormula="C24H39O5"/>
      <species metaid="M_chols_c" id="M_chols_c" name="Choline sulfate"
compartment="c" charge="0" initialConcentration="0"
hasOnlySubstanceUnits="false" boundaryCondition="false" constant="false"
fbc:chemicalFormula="C5H13NO4S"/>
      <species metaid="M_chols_e" id="M_chols_e" name="Choline sulfate_b"
compartment="e" charge="0" initialConcentration="0"
hasOnlySubstanceUnits="false" boundaryCondition="true" constant="false"
fbc:chemicalFormula="C5H13NO4S"/>
      <species metaid="M_chor_c" id="M_chor_c" name="chorismate"
compartment="c" charge="-2" initialConcentration="0"
hasOnlySubstanceUnits="false" boundaryCondition="false" constant="false"
fbc:chemicalFormula="C10H8O6"/>
      <species metaid="M_Cit__Mg_c" id="M_Cit__Mg_c" name="Citrate__Mg"
compartment="c" charge="-1" initialConcentration="0"
hasOnlySubstanceUnits="false" boundaryCondition="false" constant="false"
fbc:chemicalFormula="C6H5O7Mg"/>
      <species metaid="M_Cit__Mg_e" id="M_Cit__Mg_e" name="Citrate__Mg_b"
compartment="e" charge="0" initialConcentration="0"
hasOnlySubstanceUnits="false" boundaryCondition="true" constant="false"
fbc:chemicalFormula="C6H5O7Mg"/>
      <species metaid="M_cit_c" id="M_cit_c" name="Citrate" compartment="c"
charge="-3" initialConcentration="0" hasOnlySubstanceUnits="false"
boundaryCondition="false" constant="false" fbc:chemicalFormula="C6H5O7"/>
```

```
      <species metaid="M_cit_e" id="M_cit_e" name="Citrate_b" compartment="e"
charge="-3" initialConcentration="0" hasOnlySubstanceUnits="false"
boundaryCondition="true" constant="false" fbc:chemicalFormula="C6H5O7"/>
      <species metaid="M_citr__L_c" id="M_citr__L_c" name="L__Citrulline"
compartment="c" charge="0" initialConcentration="0"
hasOnlySubstanceUnits="false" boundaryCondition="false" constant="false"
fbc:chemicalFormula="C6H13N3O3"/>
      <species metaid="M_citr__L_e" id="M_citr__L_e" name="L__Citrulline_b"
compartment="e" charge="0" initialConcentration="0"
hasOnlySubstanceUnits="false" boundaryCondition="true" constant="false"
fbc:chemicalFormula="C6H13N3O3"/>
      <species metaid="M_cl_c" id="M_cl_c" name="Chloride" compartment="c"
charge="-1" initialConcentration="0" hasOnlySubstanceUnits="false"
boundaryCondition="false" constant="false" fbc:chemicalFormula="Cl"/>
      <species metaid="M_cl_e" id="M_cl_e" name="Chloride_b" compartment="e"
charge="-1" initialConcentration="0" hasOnlySubstanceUnits="false"
boundaryCondition="true" constant="false" fbc:chemicalFormula="Cl"/>
      <species metaid="M_clpn_SA_c" id="M_clpn_SA_c" name="Cardiolipin
(Saureus)" compartment="c" charge="-100" initialConcentration="0"
hasOnlySubstanceUnits="false" boundaryCondition="false" constant="false"
fbc:chemicalFormula="C3270H6330O434P100"/>
      <species metaid="M_clpn160_c" id="M_clpn160_c" name="cardiolipin
(tetrahexadecanoyl, n__C16:0)" compartment="c" charge="-2"
initialConcentration="0" hasOnlySubstanceUnits="false"
boundaryCondition="false" constant="false"
fbc:chemicalFormula="C73H140O17P2"/>
      <species metaid="M_cmp_c" id="M_cmp_c" name="CMP" compartment="c"
charge="-2" initialConcentration="0" hasOnlySubstanceUnits="false"
boundaryCondition="false" constant="false" fbc:chemicalFormula="C9H12N3O8P"/>
      <species metaid="M_co2_c" id="M_co2_c" name="CO2" compartment="c"
charge="0" initialConcentration="0" hasOnlySubstanceUnits="false"
boundaryCondition="false" constant="false" fbc:chemicalFormula="CO2"/>
      <species metaid="M_co2_e" id="M_co2_e" name="CO2_b" compartment="e"
charge="0" initialConcentration="0" hasOnlySubstanceUnits="false"
boundaryCondition="true" constant="false" fbc:chemicalFormula="CO2"/>
      <species metaid="M_coa_c" id="M_coa_c" name="Coenzyme A"
compartment="c" charge="-4" initialConcentration="0"
hasOnlySubstanceUnits="false" boundaryCondition="false" constant="false"
fbc:chemicalFormula="C21H32N7O16P3S"/>
      <species metaid="M_cobalt2_c" id="M_cobalt2_c" name="Co2+"
compartment="c" charge="2" initialConcentration="0"
hasOnlySubstanceUnits="false" boundaryCondition="false" constant="false"
fbc:chemicalFormula="Co"/>
      <species metaid="M_cobalt2_e" id="M_cobalt2_e" name="Co2+_b"
compartment="e" charge="2" initialConcentration="0"
hasOnlySubstanceUnits="false" boundaryCondition="true" constant="false"
fbc:chemicalFormula="Co"/>
      <species metaid="M_cpp3_c" id="M_cpp3_c" name="Coproporphyrin III"
compartment="c" charge="-5" initialConcentration="0"
hasOnlySubstanceUnits="false" boundaryCondition="false" constant="false"
fbc:chemicalFormula="C36H38N4O8"/>
      <species metaid="M_cpppg1_c" id="M_cpppg1_c" name="Coproporphyrinogen
I" compartment="c" charge="-4" initialConcentration="0"
hasOnlySubstanceUnits="false" boundaryCondition="false" constant="false"
fbc:chemicalFormula="C36H40N4O8"/>
      <species metaid="M_cpppg3_c" id="M_cpppg3_c" name="Coproporphyrinogen
III" compartment="c" charge="-4" initialConcentration="0"
```

hasOnlySubstanceUnits="false" boundaryCondition="false" constant="false" fbc:chemicalFormula="C36H40N4O8"/>
<species metaid="M_crn_c" id="M_crn_c" name="L__Carnitine" compartment="c" charge="0" initialConcentration="0" hasOnlySubstanceUnits="false" boundaryCondition="false" constant="false" fbc:chemicalFormula="C7H15NO3"/>
<species metaid="M_crn_e" id="M_crn_e" name="L__Carnitine_b" compartment="e" charge="0" initialConcentration="0" hasOnlySubstanceUnits="false" boundaryCondition="true" constant="false" fbc:chemicalFormula="C7H15NO3"/>
<species metaid="M_csn_c" id="M_csn_c" name="Cytosine" compartment="c" charge="0" initialConcentration="0" hasOnlySubstanceUnits="false" boundaryCondition="false" constant="false" fbc:chemicalFormula="C4H5N3O"/>
<species metaid="M_csn_e" id="M_csn_e" name="Cytosine_b" compartment="e" charge="0" initialConcentration="0" hasOnlySubstanceUnits="false" boundaryCondition="true" constant="false" fbc:chemicalFormula="C4H5N3O"/>
<species metaid="M_ctbt_c" id="M_ctbt_c" name="crotonobetaine" compartment="c" charge="0" initialConcentration="0" hasOnlySubstanceUnits="false" boundaryCondition="false" constant="false" fbc:chemicalFormula="C7H13NO2"/>
<species metaid="M_ctbt_e" id="M_ctbt_e" name="crotono__betaine_b" compartment="e" charge="0" initialConcentration="0" hasOnlySubstanceUnits="false" boundaryCondition="true" constant="false" fbc:chemicalFormula="C7H13NO2"/>
<species metaid="M_ctp_c" id="M_ctp_c" name="CTP" compartment="c" charge="-4" initialConcentration="0" hasOnlySubstanceUnits="false" boundaryCondition="false" constant="false" fbc:chemicalFormula="C9H12N3O14P3"/>
<species metaid="M_cu2_c" id="M_cu2_c" name="Cu2+" compartment="c" charge="2" initialConcentration="0" hasOnlySubstanceUnits="false" boundaryCondition="false" constant="false" fbc:chemicalFormula="Cu"/>
<species metaid="M_cu2_e" id="M_cu2_e" name="Cu2+_b" compartment="e" charge="2" initialConcentration="0" hasOnlySubstanceUnits="false" boundaryCondition="true" constant="false" fbc:chemicalFormula="Cu"/>
<species metaid="M_cyoala_c" id="M_cyoala_c" name="L__3__Cyanoalanine" compartment="c" charge="0" initialConcentration="0" hasOnlySubstanceUnits="false" boundaryCondition="false" constant="false" fbc:chemicalFormula="C4H6N2O2"/>
<species metaid="M_cys__D_e" id="M_cys__D_e" name="D__Cysteine_b" compartment="e" charge="0" initialConcentration="0" hasOnlySubstanceUnits="false" boundaryCondition="true" constant="false" fbc:chemicalFormula="C3H7NO2S"/>
<species metaid="M_cys__L_c" id="M_cys__L_c" name="L__Cysteine" compartment="c" charge="0" initialConcentration="0" hasOnlySubstanceUnits="false" boundaryCondition="false" constant="false" fbc:chemicalFormula="C3H7NO2S"/>
<species metaid="M_cys__L_e" id="M_cys__L_e" name="L__Cysteine_b" compartment="e" charge="0" initialConcentration="0" hasOnlySubstanceUnits="false" boundaryCondition="true" constant="false" fbc:chemicalFormula="C3H7NO2S"/>
<species metaid="M_cyst__L_c" id="M_cyst__L_c" name="L__Cystathionine" compartment="c" charge="0" initialConcentration="0" hasOnlySubstanceUnits="false" boundaryCondition="false" constant="false" fbc:chemicalFormula="C7H14N2O4S"/>
<species metaid="M_cystrna_c" id="M_cystrna_c" name="L__Cysteinyl__tRNA(Cys)" compartment="c" charge="1"

initialConcentration="0" hasOnlySubstanceUnits="false"
boundaryCondition="false" constant="false" fbc:chemicalFormula="C3H6NOSR"/>
<species metaid="M_cytd_c" id="M_cytd_c" name="Cytidine"
compartment="c" charge="0" initialConcentration="0"
hasOnlySubstanceUnits="false" boundaryCondition="false" constant="false"
fbc:chemicalFormula="C9H13N3O5"/>
<species metaid="M_cytd_e" id="M_cytd_e" name="Cytidine_b"
compartment="e" charge="0" initialConcentration="0"
hasOnlySubstanceUnits="false" boundaryCondition="true" constant="false"
fbc:chemicalFormula="C9H13N3O5"/>
<species metaid="M_dad__2_c" id="M_dad__2_c" name="Deoxyadenosine"
compartment="c" charge="0" initialConcentration="0"
hasOnlySubstanceUnits="false" boundaryCondition="false" constant="false"
fbc:chemicalFormula="C10H13N5O3"/>
<species metaid="M_dad__2_e" id="M_dad__2_e" name="Deoxyadenosine_b"
compartment="e" charge="0" initialConcentration="0"
hasOnlySubstanceUnits="false" boundaryCondition="true" constant="false"
fbc:chemicalFormula="C10H13N5O3"/>
<species metaid="M_dad__5_c" id="M_dad__5_c" name="5'__Deoxyadenosine"
compartment="c" charge="0" initialConcentration="0"
hasOnlySubstanceUnits="false" boundaryCondition="false" constant="false"
fbc:chemicalFormula="C10H13N5O3"/>
<species metaid="M_dadp_c" id="M_dadp_c" name="dADP" compartment="c"
charge="-3" initialConcentration="0" hasOnlySubstanceUnits="false"
boundaryCondition="false" constant="false"
fbc:chemicalFormula="C10H12N5O9P2"/>
<species metaid="M_dag_SA_c" id="M_dag_SA_c"
name="1,2__diacyl__sn__glycerol 3__phosphate (MRSA)" compartment="c"
charge="2" initialConcentration="0" hasOnlySubstanceUnits="false"
boundaryCondition="false" constant="false"
fbc:chemicalFormula="C53H103N2O13"/>
<species metaid="M_damp_c" id="M_damp_c" name="dAMP" compartment="c"
charge="-2" initialConcentration="0" hasOnlySubstanceUnits="false"
boundaryCondition="false" constant="false"
fbc:chemicalFormula="C10H12N5O6P"/>
<species metaid="M_dann_c" id="M_dann_c" name="7,8__Diaminononanoate"
compartment="c" charge="1" initialConcentration="0"
hasOnlySubstanceUnits="false" boundaryCondition="false" constant="false"
fbc:chemicalFormula="C9H21N2O2"/>
<species metaid="M_datp_c" id="M_datp_c" name="dATP" compartment="c"
charge="-4" initialConcentration="0" hasOnlySubstanceUnits="false"
boundaryCondition="false" constant="false"
fbc:chemicalFormula="C10H12N5O12P3"/>
<species metaid="M_dattoo_c" id="M_dattoo_c"
name="2_5__Diamino__6__5'__triphosphoryl__3'_4'__trihydroxy__2'__oxopentyl___
amino__4__oxopyrimidine" compartment="c" charge="-3" initialConcentration="0"
hasOnlySubstanceUnits="false" boundaryCondition="false" constant="false"
fbc:chemicalFormula=""/>
<species metaid="M_db4p_c" id="M_db4p_c"
name="3,4__dihydroxy__2__butanone 4__phosphate" compartment="c" charge="-2"
initialConcentration="0" hasOnlySubstanceUnits="false"
boundaryCondition="false" constant="false" fbc:chemicalFormula="C4H7O6P"/>
<species metaid="M_dc2coa_c" id="M_dc2coa_c"
name="trans__Dec__2__enoyl__CoA" compartment="c" charge="-4"
initialConcentration="0" hasOnlySubstanceUnits="false"
boundaryCondition="false" constant="false"
fbc:chemicalFormula="C31H48N7O17P3S"/>

<species metaid="M_dca_e" id="M_dca_e" name="Decanoate (n__C10:0)_b" compartment="e" charge="-1" initialConcentration="0" hasOnlySubstanceUnits="false" boundaryCondition="true" constant="false" fbc:chemicalFormula="C10H19O2"/>
<species metaid="M_dcaACP_c" id="M_dcaACP_c" name="Decanoyl__ACP (n__C10:0ACP)" compartment="c" charge="0" initialConcentration="0" hasOnlySubstanceUnits="false" boundaryCondition="false" constant="false" fbc:chemicalFormula="C21H39N2O8PRS"/>
<species metaid="M_dcacoa_c" id="M_dcacoa_c" name="Decanoyl__CoA (n__C10:0CoA)" compartment="c" charge="-4" initialConcentration="0" hasOnlySubstanceUnits="false" boundaryCondition="false" constant="false" fbc:chemicalFormula="C31H50N7O17P3S"/>
<species metaid="M_dcamp_c" id="M_dcamp_c" name="N6__(1,2__Dicarboxyethyl)__AMP" compartment="c" charge="-4" initialConcentration="0" hasOnlySubstanceUnits="false" boundaryCondition="false" constant="false" fbc:chemicalFormula="C14H14N5O11P"/>
<species metaid="M_dcdp_c" id="M_dcdp_c" name="dCDP" compartment="c" charge="-3" initialConcentration="0" hasOnlySubstanceUnits="false" boundaryCondition="false" constant="false" fbc:chemicalFormula="C9H12N3O10P2"/>
<species metaid="M_dcmp_c" id="M_dcmp_c" name="dCMP" compartment="c" charge="-2" initialConcentration="0" hasOnlySubstanceUnits="false" boundaryCondition="false" constant="false" fbc:chemicalFormula="C9H12N3O7P"/>
<species metaid="M_dctp_c" id="M_dctp_c" name="dCTP" compartment="c" charge="-4" initialConcentration="0" hasOnlySubstanceUnits="false" boundaryCondition="false" constant="false" fbc:chemicalFormula="C9H12N3O13P3"/>
<species metaid="M_dcyt_c" id="M_dcyt_c" name="Deoxycytidine" compartment="c" charge="0" initialConcentration="0" hasOnlySubstanceUnits="false" boundaryCondition="false" constant="false" fbc:chemicalFormula="C9H13N3O4"/>
<species metaid="M_dcyt_e" id="M_dcyt_e" name="Deoxycytidine_b" compartment="e" charge="0" initialConcentration="0" hasOnlySubstanceUnits="false" boundaryCondition="true" constant="false" fbc:chemicalFormula="C9H13N3O4"/>
<species metaid="M_dd2coa_c" id="M_dd2coa_c" name="trans__Dodec__2__enoyl__CoA" compartment="c" charge="-4" initialConcentration="0" hasOnlySubstanceUnits="false" boundaryCondition="false" constant="false" fbc:chemicalFormula="C33H52N7O17P3S"/>
<species metaid="M_ddcaACP_c" id="M_ddcaACP_c" name="Dodecanoyl__ACP (n__C12:0ACP)" compartment="c" charge="-1" initialConcentration="0" hasOnlySubstanceUnits="false" boundaryCondition="false" constant="false" fbc:chemicalFormula="C23H43N2O8PRS"/>
<species metaid="M_ddcacoa_c" id="M_ddcacoa_c" name="Dodecanoyl__CoA (n__C12:0CoA)" compartment="c" charge="-4" initialConcentration="0" hasOnlySubstanceUnits="false" boundaryCondition="false" constant="false" fbc:chemicalFormula="C33H54N7O17P3S"/>
<species metaid="M_decdp_c" id="M_decdp_c" name="all trans Decaprenyl diphosphate" compartment="c" charge="-3" initialConcentration="0" hasOnlySubstanceUnits="false" boundaryCondition="false" constant="false" fbc:chemicalFormula="C50H81O7P2"/>
<species metaid="M_dg15l" id="M_dg15l" name="D__glucono__1,5__lactone" compartment="c" charge="0" initialConcentration="0" hasOnlySubstanceUnits="false" boundaryCondition="false" constant="false" fbc:chemicalFormula="C6H10O6"/>

<species metaid="M_dgal6p_c" id="M_dgal6p_c" name="D__Galactose 6__phosphate" compartment="c" charge="-1" initialConcentration="0" hasOnlySubstanceUnits="false" boundaryCondition="false" constant="false" fbc:chemicalFormula="C6H11O9P"/>
<species metaid="M_dgdcg_SA2_c" id="M_dgdcg_SA2_c" name="Diglucosyl__diacylglycerol (SA) 2" compartment="c" charge="100" initialConcentration="0" hasOnlySubstanceUnits="false" boundaryCondition="false" constant="false" fbc:chemicalFormula="C2160H4165O542"/>
<species metaid="M_dgdp_c" id="M_dgdp_c" name="dGDP" compartment="c" charge="-3" initialConcentration="0" hasOnlySubstanceUnits="false" boundaryCondition="false" constant="false" fbc:chemicalFormula="C10H12N5O10P2"/>
<species metaid="M_dglu12d_c" id="M_dglu12d_c" name="Diglucosyl__1_2_dipalmitoylglycerol" compartment="c" charge="0" initialConcentration="0" hasOnlySubstanceUnits="false" boundaryCondition="false" constant="false" fbc:chemicalFormula=""/>
<species metaid="M_dglu12dgly_c" id="M_dglu12dgly_c" name="Diglucosyl__1_2_diisohexadecanoylglycerol" compartment="c" charge="0" initialConcentration="0" hasOnlySubstanceUnits="false" boundaryCondition="false" constant="false" fbc:chemicalFormula=""/>
<species metaid="M_dglu12did_c" id="M_dglu12did_c" name="Diglucosyl__1_2_dianteisoheptadecanoylglycerol" compartment="c" charge="0" initialConcentration="0" hasOnlySubstanceUnits="false" boundaryCondition="false" constant="false" fbc:chemicalFormula=""/>
<species metaid="M_dglu12dig_c" id="M_dglu12dig_c" name="Diglucosyl__1_2_diisoheptadecanoylglycerol" compartment="c" charge="0" initialConcentration="0" hasOnlySubstanceUnits="false" boundaryCondition="false" constant="false" fbc:chemicalFormula=""/>
<species metaid="M_dglu12dit_c" id="M_dglu12dit_c" name="Diglucosyl__1_2_diisotetradecanoylglycerol" compartment="c" charge="0" initialConcentration="0" hasOnlySubstanceUnits="false" boundaryCondition="false" constant="false" fbc:chemicalFormula=""/>
<species metaid="M_dglu12dpg_c" id="M_dglu12dpg_c" name="Diglucosyl__1_2_diisopentadecanoylglycerol" compartment="c" charge="0" initialConcentration="0" hasOnlySubstanceUnits="false" boundaryCondition="false" constant="false" fbc:chemicalFormula=""/>
<species metaid="M_dglu12dpgly_c" id="M_dglu12dpgly_c" name="Diglucosyl__1_2_dianteisopentadecanoylglycerol" compartment="c" charge="0" initialConcentration="0" hasOnlySubstanceUnits="false" boundaryCondition="false" constant="false" fbc:chemicalFormula=""/>
<species metaid="M_dglu12g_c" id="M_dglu12g_c" name="Diglucosyl__1_2_distearoylglycerol" compartment="c" charge="0" initialConcentration="0" hasOnlySubstanceUnits="false" boundaryCondition="false" constant="false" fbc:chemicalFormula=""/>
<species metaid="M_dglu12m_c" id="M_dglu12m_c" name="Diglucosyl__1_2_dimyristoylglycerol" compartment="c" charge="0" initialConcentration="0" hasOnlySubstanceUnits="false" boundaryCondition="false" constant="false" fbc:chemicalFormula=""/>
<species metaid="M_dgmp_c" id="M_dgmp_c" name="dGMP" compartment="c" charge="-2" initialConcentration="0" hasOnlySubstanceUnits="false" boundaryCondition="false" constant="false" fbc:chemicalFormula="C10H12N5O7P"/>
<species metaid="M_dgsn_c" id="M_dgsn_c" name="Deoxyguanosine" compartment="c" charge="0" initialConcentration="0" hasOnlySubstanceUnits="false" boundaryCondition="false" constant="false" fbc:chemicalFormula="C10H13N5O4"/>

<species metaid="M_dgsn_e" id="M_dgsn_e" name="Deoxyguanosine_b"
compartment="e" charge="0" initialConcentration="0"
hasOnlySubstanceUnits="false" boundaryCondition="true" constant="false"
fbc:chemicalFormula="C10H13N5O4"/>
<species metaid="M_dgtp_c" id="M_dgtp_c" name="dGTP" compartment="c"
charge="-4" initialConcentration="0" hasOnlySubstanceUnits="false"
boundaryCondition="false" constant="false"
fbc:chemicalFormula="C10H12N5O13P3"/>
<species metaid="M_dha_c" id="M_dha_c" name="Dihydroxyacetone"
compartment="c" charge="0" initialConcentration="0"
hasOnlySubstanceUnits="false" boundaryCondition="false" constant="false"
fbc:chemicalFormula="C3H6O3"/>
<species metaid="M_dha_e" id="M_dha_e" name="Dihydroxyacetone_b"
compartment="e" charge="0" initialConcentration="0"
hasOnlySubstanceUnits="false" boundaryCondition="true" constant="false"
fbc:chemicalFormula="C3H6O3"/>
<species metaid="M_dhap_c" id="M_dhap_c" name="Dihydroxyacetone
phosphate" compartment="c" charge="-2" initialConcentration="0"
hasOnlySubstanceUnits="false" boundaryCondition="false" constant="false"
fbc:chemicalFormula="C3H5O6P"/>
<species metaid="M_dhap_e" id="M_dhap_e" name="Dihydroxyacetone
phosphate_b" compartment="e" charge="-2" initialConcentration="0"
hasOnlySubstanceUnits="false" boundaryCondition="true" constant="false"
fbc:chemicalFormula="C3H5O6P"/>
<species metaid="M_dhf_c" id="M_dhf_c" name="7,8__Dihydrofolate"
compartment="c" charge="-2" initialConcentration="0"
hasOnlySubstanceUnits="false" boundaryCondition="false" constant="false"
fbc:chemicalFormula="C19H19N7O6"/>
<species metaid="M_dhlam_c" id="M_dhlam_c" name="Dihydrolipoamide"
compartment="c" charge="0" initialConcentration="0"
hasOnlySubstanceUnits="false" boundaryCondition="false" constant="false"
fbc:chemicalFormula="C8H17NOS2"/>
<species metaid="M_dhlpro_c" id="M_dhlpro_c" name="Dihydrolipolprotein"
compartment="c" charge="0" initialConcentration="0"
hasOnlySubstanceUnits="false" boundaryCondition="false" constant="false"
fbc:chemicalFormula="H2S2X"/>
<species metaid="M_dhna_c" id="M_dhna_c"
name="1,4__Dihydroxy__2__naphthoate" compartment="c" charge="-1"
initialConcentration="0" hasOnlySubstanceUnits="false"
boundaryCondition="false" constant="false" fbc:chemicalFormula="C11H7O4"/>
<species metaid="M_dhnpt_c" id="M_dhnpt_c" name="Dihydroneopterin"
compartment="c" charge="0" initialConcentration="0"
hasOnlySubstanceUnits="false" boundaryCondition="false" constant="false"
fbc:chemicalFormula="C9H13N5O4"/>
<species metaid="M_dhor__S_c" id="M_dhor__S_c"
name="(S)__Dihydroorotate" compartment="c" charge="-1"
initialConcentration="0" hasOnlySubstanceUnits="false"
boundaryCondition="false" constant="false" fbc:chemicalFormula="C5H5N2O4"/>
<species metaid="M_dhpmp_c" id="M_dhpmp_c" name="Dihydroneopterin
monophosphate" compartment="c" charge="-2" initialConcentration="0"
hasOnlySubstanceUnits="false" boundaryCondition="false" constant="false"
fbc:chemicalFormula="C9H12N5O7P"/>
<species metaid="M_dhpt_c" id="M_dhpt_c" name="Dihydropteroate"
compartment="c" charge="-1" initialConcentration="0"
hasOnlySubstanceUnits="false" boundaryCondition="false" constant="false"
fbc:chemicalFormula="C14H13N6O3"/>
<species metaid="M_dhptd_c" id="M_dhptd_c"
name="4,5__dihydroxy__2,3__pentanedione" compartment="c" charge="0"

```
initialConcentration="0" hasOnlySubstanceUnits="false"
boundaryCondition="false" constant="false" fbc:chemicalFormula="C5H8O4"/>
      <species metaid="M_dhsq_c" id="M_dhsq_c"
name="15__cis__dehydrosqualene" compartment="c" charge="0"
initialConcentration="0" hasOnlySubstanceUnits="false"
boundaryCondition="false" constant="false" fbc:chemicalFormula="C30H48"/>
      <species metaid="M_dhsql_c" id="M_dhsql_c" name="Dehydrosqualene"
compartment="c" charge="0" initialConcentration="0"
hasOnlySubstanceUnits="false" boundaryCondition="false" constant="false"
fbc:chemicalFormula="C30H48"/>
      <species metaid="M_dianethal_c" id="M_dianethal_c"
name="Dianteisoheptadecanoylphosphatidylethanolamine" compartment="c"
charge="0" initialConcentration="0" hasOnlySubstanceUnits="false"
boundaryCondition="false" constant="false" fbc:chemicalFormula="C39H78NO8P"/>
      <species metaid="M_dianhppglyc_c" id="M_dianhppglyc_c"
name="Dianteisoheptadecanoylphosphatidylglycerol" compartment="c" charge="-1"
initialConcentration="0" hasOnlySubstanceUnits="false"
boundaryCondition="false" constant="false" fbc:chemicalFormula="C40H78O10P"/>
      <species metaid="M_dianphaser_c" id="M_dianphaser_c"
name="Dianteisoheptadecanoylphosphatidylserine" compartment="c" charge="-2"
initialConcentration="0" hasOnlySubstanceUnits="false"
boundaryCondition="false" constant="false"
fbc:chemicalFormula="C40H76NO10P"/>
      <species metaid="M_dianppglyc_c" id="M_dianppglyc_c"
name="Dianteisopentadecanoylphosphatidylglycerol" compartment="c" charge="-1"
initialConcentration="0" hasOnlySubstanceUnits="false"
boundaryCondition="false" constant="false" fbc:chemicalFormula="C36H70O10P"/>
      <species metaid="M_diapdpgp_c" id="M_diapdpgp_c"
name="Dianteisopentadecanoylphosphatidylglycerophosphate" compartment="c"
charge="-2" initialConcentration="0" hasOnlySubstanceUnits="false"
boundaryCondition="false" constant="false"
fbc:chemicalFormula="C36H70O13P2"/>
      <species metaid="M_didp_c" id="M_didp_c" name="DIDP" compartment="c"
charge="0" initialConcentration="0" hasOnlySubstanceUnits="false"
boundaryCondition="false" constant="false"
fbc:chemicalFormula="C10H11N4O10P2"/>
      <species metaid="M_diidpgp_c" id="M_diidpgp_c"
name="Diisotetradecanoylphosphatidylglycerophosphate" compartment="c"
charge="-2" initialConcentration="0" hasOnlySubstanceUnits="false"
boundaryCondition="false" constant="false"
fbc:chemicalFormula="C34H66O13P2"/>
      <species metaid="M_diihexpglyc_c" id="M_diihexpglyc_c"
name="Diisohexadecanoylphosphatidylglycerol" compartment="c" charge="-1"
initialConcentration="0" hasOnlySubstanceUnits="false"
boundaryCondition="false" constant="false" fbc:chemicalFormula="C38H74O10P"/>
      <species metaid="M_diihexpgp_c" id="M_diihexpgp_c"
name="Diisohexadecanoylphosphatidylglycerophosphate" compartment="c"
charge="-2" initialConcentration="0" hasOnlySubstanceUnits="false"
boundaryCondition="false" constant="false"
fbc:chemicalFormula="C38H74O13P2"/>
      <species metaid="M_diihpgly_c" id="M_diihpgly_c"
name="Diisoheptadecanoylphosphatidylglycerol" compartment="c" charge="-1"
initialConcentration="0" hasOnlySubstanceUnits="false"
boundaryCondition="false" constant="false" fbc:chemicalFormula="C40H78O10P"/>
      <species metaid="M_diihpgp_c" id="M_diihpgp_c"
name="Dianteisoheptadecanoylphosphatidylglycerophosphate" compartment="c"
charge="-2" initialConcentration="0" hasOnlySubstanceUnits="false"
```

boundaryCondition="false" constant="false"
fbc:chemicalFormula="C40H78O13P2"/>
<species metaid="M_diipdpgly_c" id="M_diipdpgly_c"
name="Diisopentadecanoylphosphatidylglycerol" compartment="c" charge="-1"
initialConcentration="0" hasOnlySubstanceUnits="false"
boundaryCondition="false" constant="false" fbc:chemicalFormula="C36H70O10P"/>
<species metaid="M_diippgp_c" id="M_diippgp_c"
name="Diisopentadecanoylphosphatidylglycerophosphate" compartment="c"
charge="-2" initialConcentration="0" hasOnlySubstanceUnits="false"
boundaryCondition="false" constant="false"
fbc:chemicalFormula="C36H70O13P2"/>
<species metaid="M_diispgp_c" id="M_diispgp_c"
name="Diisoheptadecanoylphosphatidylglycerophosphate" compartment="c"
charge="-2" initialConcentration="0" hasOnlySubstanceUnits="false"
boundaryCondition="false" constant="false"
fbc:chemicalFormula="C40H78O13P2"/>
<species metaid="M_diitpgly_c" id="M_diitpgly_c"
name="Diisotetradecanoylphosphatidylglycerol" compartment="c" charge="-1"
initialConcentration="0" hasOnlySubstanceUnits="false"
boundaryCondition="false" constant="false" fbc:chemicalFormula="C34H66O10P"/>
<species metaid="M_dimp_c" id="M_dimp_c" name="dIMP" compartment="c"
charge="-2" initialConcentration="0" hasOnlySubstanceUnits="false"
boundaryCondition="false" constant="false"
fbc:chemicalFormula="C10H11N4O7P"/>
<species metaid="M_din_c" id="M_din_c" name="Deoxyinosine"
compartment="c" charge="0" initialConcentration="0"
hasOnlySubstanceUnits="false" boundaryCondition="false" constant="false"
fbc:chemicalFormula="C10H12N4O4"/>
<species metaid="M_din_e" id="M_din_e" name="Deoxyinosine_b"
compartment="e" charge="0" initialConcentration="0"
hasOnlySubstanceUnits="false" boundaryCondition="true" constant="false"
fbc:chemicalFormula="C10H12N4O4"/>
<species metaid="M_ditp_c" id="M_ditp_c" name="dITP" compartment="c"
charge="-4" initialConcentration="0" hasOnlySubstanceUnits="false"
boundaryCondition="false" constant="false"
fbc:chemicalFormula="C10H11N4O13P3"/>
<species metaid="M_dkmpp_c" id="M_dkmpp_c"
name="2,3__diketo__5__methylthio__1__phosphopentane" compartment="c"
charge="-2" initialConcentration="0" hasOnlySubstanceUnits="false"
boundaryCondition="false" constant="false" fbc:chemicalFormula="C6H9O6PS"/>
<species metaid="M_dmlz_c" id="M_dmlz_c"
name="6,7__Dimethyl__8__(1__D__ribityl)lumazine" compartment="c" charge="-1"
initialConcentration="0" hasOnlySubstanceUnits="false"
boundaryCondition="false" constant="false" fbc:chemicalFormula="C13H18N4O6"/>
<species metaid="M_dmpp_c" id="M_dmpp_c" name="Dimethylallyl
diphosphate" compartment="c" charge="-3" initialConcentration="0"
hasOnlySubstanceUnits="false" boundaryCondition="false" constant="false"
fbc:chemicalFormula="C5H9O7P2"/>
<species metaid="M_dnad_c" id="M_dnad_c" name="Deamino__NAD+"
compartment="c" charge="-2" initialConcentration="0"
hasOnlySubstanceUnits="false" boundaryCondition="false" constant="false"
fbc:chemicalFormula="C21H24N6O15P2"/>
<species metaid="M_dnspal_c" id="M_dnspal_c"
name="4,4'__diaponeurosporenal" compartment="c" charge="0"
initialConcentration="0" hasOnlySubstanceUnits="false"
boundaryCondition="false" constant="false" fbc:chemicalFormula="C30H40O"/>
<species metaid="M_dnspate_c" id="M_dnspate_c"
name="4,4'__diaponeurosporenoate" compartment="c" charge="0"

initialConcentration="0" hasOnlySubstanceUnits="false"
boundaryCondition="false" constant="false" fbc:chemicalFormula="C30H39O2"/>
      <species metaid="M_dnspen_c" id="M_dnspen_c"
name="all__trans__4,4'__diaponeurosporene" compartment="c" charge="0"
initialConcentration="0" hasOnlySubstanceUnits="false"
boundaryCondition="false" constant="false" fbc:chemicalFormula="C30H42"/>
      <species metaid="M_dpcoa_c" id="M_dpcoa_c" name="Dephospho__CoA"
compartment="c" charge="-2" initialConcentration="0"
hasOnlySubstanceUnits="false" boundaryCondition="false" constant="false"
fbc:chemicalFormula="C21H33N7O13P2S"/>
      <species metaid="M_dphytol_c" id="M_dphytol_c"
name="all__trans__4,4'__diapophytofluene" compartment="c" charge="0"
initialConcentration="0" hasOnlySubstanceUnits="false"
boundaryCondition="false" constant="false" fbc:chemicalFormula="C30H46"/>
      <species metaid="M_dr5p" id="M_dr5p" name="D__ribitol 5__phosphate"
compartment="c" charge="-2" initialConcentration="0"
hasOnlySubstanceUnits="false" boundaryCondition="false" constant="false"
fbc:chemicalFormula="C5H11O8P"/>
      <species metaid="M_drib_c" id="M_drib_c" name="Deoxyribose"
compartment="c" charge="0" initialConcentration="0"
hasOnlySubstanceUnits="false" boundaryCondition="false" constant="false"
fbc:chemicalFormula="C5H10O4"/>
      <species metaid="M_drib_e" id="M_drib_e" name="Deoxyribose_b"
compartment="e" charge="0" initialConcentration="0"
hasOnlySubstanceUnits="false" boundaryCondition="true" constant="false"
fbc:chemicalFormula="C5H10O4"/>
      <species metaid="M_dscl_c" id="M_dscl_c" name="dihydrosirohydrochlorin"
compartment="c" charge="-7" initialConcentration="0"
hasOnlySubstanceUnits="false" boundaryCondition="false" constant="false"
fbc:chemicalFormula="C42H41N4O16"/>
      <species metaid="M_dtbt_c" id="M_dtbt_c" name="Dethiobiotin"
compartment="c" charge="-1" initialConcentration="0"
hasOnlySubstanceUnits="false" boundaryCondition="false" constant="false"
fbc:chemicalFormula="C10H17N2O3"/>
      <species metaid="M_dtdp_c" id="M_dtdp_c" name="dTDP" compartment="c"
charge="-3" initialConcentration="0" hasOnlySubstanceUnits="false"
boundaryCondition="false" constant="false"
fbc:chemicalFormula="C10H13N2O11P2"/>
      <species metaid="M_dtmp_c" id="M_dtmp_c" name="dTMP" compartment="c"
charge="-2" initialConcentration="0" hasOnlySubstanceUnits="false"
boundaryCondition="false" constant="false"
fbc:chemicalFormula="C10H13N2O8P"/>
      <species metaid="M_dttp_c" id="M_dttp_c" name="dTTP" compartment="c"
charge="-4" initialConcentration="0" hasOnlySubstanceUnits="false"
boundaryCondition="false" constant="false"
fbc:chemicalFormula="C10H13N2O14P3"/>
      <species metaid="M_dudp_c" id="M_dudp_c" name="dUDP" compartment="c"
charge="-3" initialConcentration="0" hasOnlySubstanceUnits="false"
boundaryCondition="false" constant="false"
fbc:chemicalFormula="C9H11N2O11P2"/>
      <species metaid="M_dump_c" id="M_dump_c" name="dUMP" compartment="c"
charge="-2" initialConcentration="0" hasOnlySubstanceUnits="false"
boundaryCondition="false" constant="false" fbc:chemicalFormula="C9H11N2O8P"/>
      <species metaid="M_duri_c" id="M_duri_c" name="Deoxyuridine"
compartment="c" charge="0" initialConcentration="0"
hasOnlySubstanceUnits="false" boundaryCondition="false" constant="false"
fbc:chemicalFormula="C9H12N2O5"/>

<species metaid="M_duri_e" id="M_duri_e" name="Deoxyuridine_b"
compartment="e" charge="0" initialConcentration="0"
hasOnlySubstanceUnits="false" boundaryCondition="true" constant="false"
fbc:chemicalFormula="C9H12N2O5"/>
<species metaid="M_dutp_c" id="M_dutp_c" name="dUTP" compartment="c"
charge="-4" initialConcentration="0" hasOnlySubstanceUnits="false"
boundaryCondition="false" constant="false"
fbc:chemicalFormula="C9H11N2O14P3"/>
<species metaid="M_dxyl5p_c" id="M_dxyl5p_c"
name="1__deoxy__D__xylulose 5__phosphate" compartment="c" charge="-2"
initialConcentration="0" hasOnlySubstanceUnits="false"
boundaryCondition="false" constant="false" fbc:chemicalFormula="C5H9O7P"/>
<species metaid="M_e4hglu_c" id="M_e4hglu_c"
name="L__erythro__4__Hydroxyglutamate" compartment="c" charge="-1"
initialConcentration="0" hasOnlySubstanceUnits="false"
boundaryCondition="false" constant="false" fbc:chemicalFormula="C5H8NO5"/>
<species metaid="M_e4p_c" id="M_e4p_c" name="D__Erythrose 4__phosphate"
compartment="c" charge="-2" initialConcentration="0"
hasOnlySubstanceUnits="false" boundaryCondition="false" constant="false"
fbc:chemicalFormula="C4H7O7P"/>
<species metaid="M_egmeACP_c" id="M_egmeACP_c" name="Enoylglutaryl__ACP
methyl ester" compartment="c" charge="1" initialConcentration="0"
hasOnlySubstanceUnits="false" boundaryCondition="false" constant="false"
fbc:chemicalFormula="C17H27N2O10PRS"/>
<species metaid="M_eig3p_c" id="M_eig3p_c"
name="D__erythro__1__(Imidazol__4__yl)glycerol 3__phosphate" compartment="c"
charge="-2" initialConcentration="0" hasOnlySubstanceUnits="false"
boundaryCondition="false" constant="false" fbc:chemicalFormula="C6H9N2O6P"/>
<species metaid="M_elaid_c" id="M_elaid_c" name="elaidic acid"
compartment="c" charge="-1" initialConcentration="0"
hasOnlySubstanceUnits="false" boundaryCondition="false" constant="false"
fbc:chemicalFormula="C18H33O2"/>
<species metaid="M_epmeACP_c" id="M_epmeACP_c" name="Enoylpimeloyl__ACP
methyl ester" compartment="c" charge="1" initialConcentration="0"
hasOnlySubstanceUnits="false" boundaryCondition="false" constant="false"
fbc:chemicalFormula="C19H31N2O10PRS"/>
<species metaid="M_etha_c" id="M_etha_c" name="Ethanolamine"
compartment="c" charge="1" initialConcentration="0"
hasOnlySubstanceUnits="false" boundaryCondition="false" constant="false"
fbc:chemicalFormula="C2H8NO"/>
<species metaid="M_etha_e" id="M_etha_e" name="Ethanolamine_b"
compartment="e" charge="1" initialConcentration="0"
hasOnlySubstanceUnits="false" boundaryCondition="true" constant="false"
fbc:chemicalFormula="C2H8NO"/>
<species metaid="M_ethamp_c" id="M_ethamp_c" name="Ethanolamine
phosphate" compartment="c" charge="-1" initialConcentration="0"
hasOnlySubstanceUnits="false" boundaryCondition="false" constant="false"
fbc:chemicalFormula="C2H7NO4P"/>
<species metaid="M_etoh_c" id="M_etoh_c" name="Ethanol" compartment="c"
charge="0" initialConcentration="0" hasOnlySubstanceUnits="false"
boundaryCondition="false" constant="false" fbc:chemicalFormula="C2H6O"/>
<species metaid="M_etoh_e" id="M_etoh_e" name="Ethanol_b"
compartment="e" charge="0" initialConcentration="0"
hasOnlySubstanceUnits="false" boundaryCondition="true" constant="false"
fbc:chemicalFormula="C2H6O"/>
<species metaid="M_f1p_c" id="M_f1p_c" name="D__Fructose 1__phosphate"
compartment="c" charge="-2" initialConcentration="0"

```
hasOnlySubstanceUnits="false" boundaryCondition="false" constant="false"
fbc:chemicalFormula="C6H11O9P"/>
      <species metaid="M_f6p_c" id="M_f6p_c" name="D__Fructose 6__phosphate"
compartment="c" charge="-2" initialConcentration="0"
hasOnlySubstanceUnits="false" boundaryCondition="false" constant="false"
fbc:chemicalFormula="C6H11O9P"/>
      <species metaid="M_f6p_e" id="M_f6p_e" name="D__Fructose
6__phosphate_b" compartment="e" charge="-2" initialConcentration="0"
hasOnlySubstanceUnits="false" boundaryCondition="true" constant="false"
fbc:chemicalFormula="C6H11O9P"/>
      <species metaid="M_f6p_p" id="M_f6p_p" name="D__Fructose 6__phosphate"
compartment="p" charge="-2" initialConcentration="0"
hasOnlySubstanceUnits="false" boundaryCondition="false" constant="false"
fbc:chemicalFormula="C6H11O9P"/>
      <species metaid="M_fa1_c" id="M_fa1_c" name="Fatty acid (Iso__C14:0)"
compartment="c" charge="-1" initialConcentration="0"
hasOnlySubstanceUnits="false" boundaryCondition="false" constant="false"
fbc:chemicalFormula="C14H27O2"/>
      <species metaid="M_fa1coa_c" id="M_fa1coa_c" name="fa1coa"
compartment="c" charge="-3" initialConcentration="0"
hasOnlySubstanceUnits="false" boundaryCondition="false" constant="false"
fbc:chemicalFormula="C35H59N7O17P3S"/>
      <species metaid="M_fa3_c" id="M_fa3_c" name="Fatty acid (Iso__C15:0)"
compartment="c" charge="-1" initialConcentration="0"
hasOnlySubstanceUnits="false" boundaryCondition="false" constant="false"
fbc:chemicalFormula="C15H29O2"/>
      <species metaid="M_fa3coa_c" id="M_fa3coa_c" name="fa3coa"
compartment="c" charge="-3" initialConcentration="0"
hasOnlySubstanceUnits="false" boundaryCondition="false" constant="false"
fbc:chemicalFormula="C36H61N7O17P3S"/>
      <species metaid="M_fa4_c" id="M_fa4_c" name="Fatty acid
(Anteiso__C15:0)" compartment="c" charge="-1" initialConcentration="0"
hasOnlySubstanceUnits="false" boundaryCondition="false" constant="false"
fbc:chemicalFormula="C15H29O2"/>
      <species metaid="M_fa4coa_c" id="M_fa4coa_c" name="fa4coa"
compartment="c" charge="-3" initialConcentration="0"
hasOnlySubstanceUnits="false" boundaryCondition="false" constant="false"
fbc:chemicalFormula="C36H61N7O17P3S"/>
      <species metaid="M_fa5_c" id="M_fa5_c" name="Fatty acid (Iso__C16:1)"
compartment="c" charge="-1" initialConcentration="0"
hasOnlySubstanceUnits="false" boundaryCondition="false" constant="false"
fbc:chemicalFormula="C16H29O2"/>
      <species metaid="M_fa6_c" id="M_fa6_c" name="Fatty acid (iso__C16:0)"
compartment="c" charge="-1" initialConcentration="0"
hasOnlySubstanceUnits="false" boundaryCondition="false" constant="false"
fbc:chemicalFormula="C16H31O2"/>
      <species metaid="M_fa6coa_c" id="M_fa6coa_c" name="fa6coa"
compartment="c" charge="-3" initialConcentration="0"
hasOnlySubstanceUnits="false" boundaryCondition="false" constant="false"
fbc:chemicalFormula="C37H63N7O17P3S"/>
      <species metaid="M_fa9_c" id="M_fa9_c" name="Fatty acid (Iso__C17:1)"
compartment="c" charge="-1" initialConcentration="0"
hasOnlySubstanceUnits="false" boundaryCondition="false" constant="false"
fbc:chemicalFormula="C17H31O2"/>
      <species metaid="M_fa10_c" id="M_fa10_c" name="Fatty acid
(Anteiso__C17:1)" compartment="c" charge="-1" initialConcentration="0"
hasOnlySubstanceUnits="false" boundaryCondition="false" constant="false"
fbc:chemicalFormula="C17H31O2"/>
```

```
      <species metaid="M_fa11_c" id="M_fa11_c" name="Fatty acid (Iso__C17:0)"
compartment="c" charge="-1" initialConcentration="0"
hasOnlySubstanceUnits="false" boundaryCondition="false" constant="false"
fbc:chemicalFormula="C17H33O2"/>
      <species metaid="M_fa11coa_c" id="M_fa11coa_c" name="fa11coa"
compartment="c" charge="-3" initialConcentration="0"
hasOnlySubstanceUnits="false" boundaryCondition="false" constant="false"
fbc:chemicalFormula="C38H65N7O17P3S"/>
      <species metaid="M_fa12_c" id="M_fa12_c" name="Fatty acid
(Anteiso__C17:0)" compartment="c" charge="-1" initialConcentration="0"
hasOnlySubstanceUnits="false" boundaryCondition="false" constant="false"
fbc:chemicalFormula="C17H33O2"/>
      <species metaid="M_fa12coa_c" id="M_fa12coa_c" name="fa12coa"
compartment="c" charge="-3" initialConcentration="0"
hasOnlySubstanceUnits="false" boundaryCondition="false" constant="false"
fbc:chemicalFormula="C38H65N7O17P3S"/>
      <species metaid="M_fa19a_c" id="M_fa19a_c" name="Fatty Acid
(Anteiso__C19:0)" compartment="c" charge="-1" initialConcentration="0"
hasOnlySubstanceUnits="false" boundaryCondition="false" constant="false"
fbc:chemicalFormula="C19H37O2"/>
      <species metaid="M_fa20n_c" id="M_fa20n_c" name="Fatty Acid (C20:0)"
compartment="c" charge="-1" initialConcentration="0"
hasOnlySubstanceUnits="false" boundaryCondition="false" constant="false"
fbc:chemicalFormula="C20H39O2"/>
      <species metaid="M_fad_c" id="M_fad_c" name="Flavin adenine
dinucleotide oxidized" compartment="c" charge="-3" initialConcentration="0"
hasOnlySubstanceUnits="false" boundaryCondition="false" constant="false"
fbc:chemicalFormula="C27H31N9O15P2"/>
      <species metaid="M_fadh2_c" id="M_fadh2_c" name="Flavin adenine
dinucleotide reduced" compartment="c" charge="-2" initialConcentration="0"
hasOnlySubstanceUnits="false" boundaryCondition="false" constant="false"
fbc:chemicalFormula="C27H33N9O15P2"/>
      <species metaid="M_fald_c" id="M_fald_c" name="Formaldehyde"
compartment="c" charge="0" initialConcentration="0"
hasOnlySubstanceUnits="false" boundaryCondition="false" constant="false"
fbc:chemicalFormula="CH2O"/>
      <species metaid="M_fapnt_c" id="M_fapnt_c"
name="Formamidopyrimidine_nucleoside_triphosphate" compartment="c" charge="-
3" initialConcentration="0" hasOnlySubstanceUnits="false"
boundaryCondition="false" constant="false" fbc:chemicalFormula=""/>
      <species metaid="M_fdp_c" id="M_fdp_c" name="D__Fructose
1,6__bisphosphate" compartment="c" charge="-4" initialConcentration="0"
hasOnlySubstanceUnits="false" boundaryCondition="false" constant="false"
fbc:chemicalFormula="C6H10O12P2"/>
      <species metaid="M_fdxox_c" id="M_fdxox_c" name="Oxidized ferredoxin"
compartment="c" charge="6" initialConcentration="0"
hasOnlySubstanceUnits="false" boundaryCondition="false" constant="false"
fbc:chemicalFormula="X"/>
      <species metaid="M_fdxrd_c" id="M_fdxrd_c" name="Reduced ferredoxin"
compartment="c" charge="4" initialConcentration="0"
hasOnlySubstanceUnits="false" boundaryCondition="false" constant="false"
fbc:chemicalFormula="XH2"/>
      <species metaid="M_fe2_c" id="M_fe2_c" name="Fe2+" compartment="c"
charge="2" initialConcentration="0" hasOnlySubstanceUnits="false"
boundaryCondition="false" constant="false" fbc:chemicalFormula="Fe"/>
      <species metaid="M_fe2_e" id="M_fe2_e" name="Fe2+_b" compartment="e"
charge="2" initialConcentration="0" hasOnlySubstanceUnits="false"
boundaryCondition="true" constant="false" fbc:chemicalFormula="Fe"/>
```

<species metaid="M_fe3_c" id="M_fe3_c" name="Fe3+" compartment="c"
charge="3" initialConcentration="0" hasOnlySubstanceUnits="false"
boundaryCondition="false" constant="false" fbc:chemicalFormula="Fe"/>
<species metaid="M_fe3_e" id="M_fe3_e" name="Fe3+_b" compartment="e"
charge="3" initialConcentration="0" hasOnlySubstanceUnits="false"
boundaryCondition="true" constant="false" fbc:chemicalFormula="Fe"/>
<species metaid="M_fe3dcit_e" id="M_fe3dcit_e"
name="Fe(III)dicitrate_b" compartment="e" charge="-3"
initialConcentration="0" hasOnlySubstanceUnits="false"
boundaryCondition="true" constant="false" fbc:chemicalFormula="C12H10FeO14"/>
<species metaid="M_fecpp3_c" id="M_fecpp3_c" name="Fe__Coproporphyrin
III" compartment="c" charge="-5" initialConcentration="0"
hasOnlySubstanceUnits="false" boundaryCondition="false" constant="false"
fbc:chemicalFormula="C36H36FeN4O8"/>
<species metaid="M_fecrm_c" id="M_fecrm_c" name="Ferrichrome"
compartment="c" charge="0" initialConcentration="0"
hasOnlySubstanceUnits="false" boundaryCondition="false" constant="false"
fbc:chemicalFormula="C27H42FeN9O12"/>
<species metaid="M_fecrm_e" id="M_fecrm_e" name="Ferrichrome_b"
compartment="e" charge="0" initialConcentration="0"
hasOnlySubstanceUnits="false" boundaryCondition="true" constant="false"
fbc:chemicalFormula="C27H42FeN9O12"/>
<species metaid="M_fgam_c" id="M_fgam_c"
name="N2__Formyl__N1__(5__phospho__D__ribosyl)glycinamide" compartment="c"
charge="-2" initialConcentration="0" hasOnlySubstanceUnits="false"
boundaryCondition="false" constant="false" fbc:chemicalFormula="C8H13N2O9P"/>
<species metaid="M_ficytcb1_c" id="M_ficytcb1_c"
name="Ferricytochrome_b1" compartment="c" charge="5" initialConcentration="0"
hasOnlySubstanceUnits="false" boundaryCondition="false" constant="false"
fbc:chemicalFormula=""/>
<species metaid="M_ficytcc553_c" id="M_ficytcc553_c"
name="Ferricytochrome c__553" compartment="c" charge="5"
initialConcentration="0" hasOnlySubstanceUnits="false"
boundaryCondition="false" constant="false"
fbc:chemicalFormula="C42H54FeN8O6S2"/>
<species metaid="M_fmn_c" id="M_fmn_c" name="FMN" compartment="c"
charge="-3" initialConcentration="0" hasOnlySubstanceUnits="false"
boundaryCondition="false" constant="false"
fbc:chemicalFormula="C17H19N4O9P"/>
<species metaid="M_fmnh2_c" id="M_fmnh2_c" name="Reduced FMN"
compartment="c" charge="-2" initialConcentration="0"
hasOnlySubstanceUnits="false" boundaryCondition="false" constant="false"
fbc:chemicalFormula="C17H21N4O9P"/>
<species metaid="M_focytc_c" id="M_focytc_c" name="Ferrocytochrome c"
compartment="c" charge="2" initialConcentration="0"
hasOnlySubstanceUnits="false" boundaryCondition="false" constant="false"
fbc:chemicalFormula="C42H53FeN8O6S2"/>
<species metaid="M_focytcb1_c" id="M_focytcb1_c"
name="Ferrocytochrome_b1" compartment="c" charge="4" initialConcentration="0"
hasOnlySubstanceUnits="false" boundaryCondition="false" constant="false"
fbc:chemicalFormula=""/>
<species metaid="M_fol_c" id="M_fol_c" name="Folate" compartment="c"
charge="-2" initialConcentration="0" hasOnlySubstanceUnits="false"
boundaryCondition="false" constant="false" fbc:chemicalFormula="C19H18N7O6"/>
<species metaid="M_for_c" id="M_for_c" name="Formate" compartment="c"
charge="-1" initialConcentration="0" hasOnlySubstanceUnits="false"
boundaryCondition="false" constant="false" fbc:chemicalFormula="CH1O2"/>

```
      <species metaid="M_for_e" id="M_for_e" name="Formate_b" compartment="e"
charge="-1" initialConcentration="0" hasOnlySubstanceUnits="false"
boundaryCondition="true" constant="false" fbc:chemicalFormula="CH1O2"/>
      <species metaid="M_forglu_c" id="M_forglu_c"
name="N__Formimidoyl__L__glutamate" compartment="c" charge="-1"
initialConcentration="0" hasOnlySubstanceUnits="false"
boundaryCondition="false" constant="false" fbc:chemicalFormula="C6H8N2O4"/>
      <species metaid="M_fpram_c" id="M_fpram_c"
name="2__(Formamido)__N1__(5__phospho__D__ribosyl)acetamidine"
compartment="c" charge="-2" initialConcentration="0"
hasOnlySubstanceUnits="false" boundaryCondition="false" constant="false"
fbc:chemicalFormula="C8H15N3O8P"/>
      <species metaid="M_fprica_c" id="M_fprica_c"
name="5__Formamido__1__(5__phospho__D__ribosyl)imidazole__4__carboxamide"
compartment="c" charge="-2" initialConcentration="0"
hasOnlySubstanceUnits="false" boundaryCondition="false" constant="false"
fbc:chemicalFormula="C10H13N4O9P"/>
      <species metaid="M_frdp_c" id="M_frdp_c" name="Farnesyl diphosphate"
compartment="c" charge="-3" initialConcentration="0"
hasOnlySubstanceUnits="false" boundaryCondition="false" constant="false"
fbc:chemicalFormula="C15H25O7P2"/>
      <species metaid="M_frmd_c" id="M_frmd_c" name="Formamide"
compartment="c" charge="0" initialConcentration="0"
hasOnlySubstanceUnits="false" boundaryCondition="false" constant="false"
fbc:chemicalFormula="CH3NO"/>
      <species metaid="M_fru_c" id="M_fru_c" name="D__Fructose"
compartment="c" charge="0" initialConcentration="0"
hasOnlySubstanceUnits="false" boundaryCondition="false" constant="false"
fbc:chemicalFormula="C6H12O6"/>
      <species metaid="M_fru_e" id="M_fru_e" name="D__Fructose_b"
compartment="e" charge="0" initialConcentration="0"
hasOnlySubstanceUnits="false" boundaryCondition="true" constant="false"
fbc:chemicalFormula="C6H12O6"/>
      <species metaid="M_fruur_c" id="M_fruur_c" name="D__Fructuronate"
compartment="c" charge="-1" initialConcentration="0"
hasOnlySubstanceUnits="false" boundaryCondition="false" constant="false"
fbc:chemicalFormula="C6H9O7"/>
      <species metaid="M_fuc__L_e" id="M_fuc__L_e" name="L__Fucose_b"
compartment="e" charge="0" initialConcentration="0"
hasOnlySubstanceUnits="false" boundaryCondition="true" constant="false"
fbc:chemicalFormula="C6H12O5"/>
      <species metaid="M_fuc1p__L_e" id="M_fuc1p__L_e" name="L__Fucose
1__phosphate_b" compartment="e" charge="-1" initialConcentration="0"
hasOnlySubstanceUnits="false" boundaryCondition="true" constant="false"
fbc:chemicalFormula="C6H11O8P"/>
      <species metaid="M_fum_c" id="M_fum_c" name="Fumarate" compartment="c"
charge="-2" initialConcentration="0" hasOnlySubstanceUnits="false"
boundaryCondition="false" constant="false" fbc:chemicalFormula="C4H2O4"/>
      <species metaid="M_fum_e" id="M_fum_e" name="Fumarate_b"
compartment="e" charge="-2" initialConcentration="0"
hasOnlySubstanceUnits="false" boundaryCondition="true" constant="false"
fbc:chemicalFormula="C4H2O4"/>
      <species metaid="M_g1p_c" id="M_g1p_c" name="D__Glucose 1__phosphate"
compartment="c" charge="-2" initialConcentration="0"
hasOnlySubstanceUnits="false" boundaryCondition="false" constant="false"
fbc:chemicalFormula="C6H11O9P"/>
      <species metaid="M_g1p_e" id="M_g1p_e" name="D__Glucose 1__phosphate_b"
compartment="e" charge="-2" initialConcentration="0"
```

```
hasOnlySubstanceUnits="false" boundaryCondition="true" constant="false"
fbc:chemicalFormula="C6H11O9P"/>
      <species metaid="M_g3p_c" id="M_g3p_c" name="Glyceraldehyde
3__phosphate" compartment="c" charge="-2" initialConcentration="0"
hasOnlySubstanceUnits="false" boundaryCondition="false" constant="false"
fbc:chemicalFormula="C3H5O6P"/>
      <species metaid="M_g3pc_c" id="M_g3pc_c"
name="sn__Glycero__3__phosphocholine" compartment="c" charge="0"
initialConcentration="0" hasOnlySubstanceUnits="false"
boundaryCondition="false" constant="false" fbc:chemicalFormula="C8H20NO6P"/>
      <species metaid="M_g3pe_c" id="M_g3pe_c"
name="sn__Glycero__3__phosphoethanolamine" compartment="c" charge="0"
initialConcentration="0" hasOnlySubstanceUnits="false"
boundaryCondition="false" constant="false" fbc:chemicalFormula="C5H14NO6P"/>
      <species metaid="M_g3pg_c" id="M_g3pg_c" name="Glycerophosphoglycerol"
compartment="c" charge="-1" initialConcentration="0"
hasOnlySubstanceUnits="false" boundaryCondition="false" constant="false"
fbc:chemicalFormula="C6H14O8P"/>
      <species metaid="M_g3pi_c" id="M_g3pi_c"
name="sn__Glycero__3__phospho__1__inositol" compartment="c" charge="-1"
initialConcentration="0" hasOnlySubstanceUnits="false"
boundaryCondition="false" constant="false" fbc:chemicalFormula="C9H18O11P"/>
      <species metaid="M_g3ps_c" id="M_g3ps_c" name="Glycerophosphoserine"
compartment="c" charge="-1" initialConcentration="0"
hasOnlySubstanceUnits="false" boundaryCondition="false" constant="false"
fbc:chemicalFormula="C6H13NO8P"/>
      <species metaid="M_g6p__B_c" id="M_g6p__B_c" name="beta__D__glucose
6__phosphate" compartment="c" charge="-1" initialConcentration="0"
hasOnlySubstanceUnits="false" boundaryCondition="false" constant="false"
fbc:chemicalFormula="C6H11O9P"/>
      <species metaid="M_g6p_c" id="M_g6p_c" name="D__Glucose 6__phosphate"
compartment="c" charge="-2" initialConcentration="0"
hasOnlySubstanceUnits="false" boundaryCondition="false" constant="false"
fbc:chemicalFormula="C6H11O9P"/>
      <species metaid="M_g6p_e" id="M_g6p_e" name="D__Glucose 6__phosphate_b"
compartment="e" charge="-2" initialConcentration="0"
hasOnlySubstanceUnits="false" boundaryCondition="true" constant="false"
fbc:chemicalFormula="C6H11O9P"/>
      <species metaid="M_g6p_p" id="M_g6p_p" name="D__Glucose 6__phosphate"
compartment="p" charge="-2" initialConcentration="0"
hasOnlySubstanceUnits="false" boundaryCondition="false" constant="false"
fbc:chemicalFormula="C6H11O9P"/>
      <species metaid="M_gal_c" id="M_gal_c" name="D__Galactose"
compartment="c" charge="0" initialConcentration="0"
hasOnlySubstanceUnits="false" boundaryCondition="false" constant="false"
fbc:chemicalFormula="C6H12O6"/>
      <species metaid="M_gal_e" id="M_gal_e" name="D__Galactose_b"
compartment="e" charge="0" initialConcentration="0"
hasOnlySubstanceUnits="false" boundaryCondition="true" constant="false"
fbc:chemicalFormula="C6H12O6"/>
      <species metaid="M_gal_p" id="M_gal_p" name="D__Galactose"
compartment="p" charge="0" initialConcentration="0"
hasOnlySubstanceUnits="false" boundaryCondition="false" constant="false"
fbc:chemicalFormula="C6H12O6"/>
      <species metaid="M_gal1p_c" id="M_gal1p_c" name="alpha__D__Galactose
1__phosphate" compartment="c" charge="-2" initialConcentration="0"
hasOnlySubstanceUnits="false" boundaryCondition="false" constant="false"
fbc:chemicalFormula="C6H11O9P"/>
```

<species metaid="M_galct__D_e" id="M_galct__D_e" name="D__Galactarate_b" compartment="e" charge="-2" initialConcentration="0" hasOnlySubstanceUnits="false" boundaryCondition="true" constant="false" fbc:chemicalFormula="C6H8O8"/>
<species metaid="M_galctn__D_e" id="M_galctn__D_e" name="D__Galactonate_b" compartment="e" charge="-1" initialConcentration="0" hasOnlySubstanceUnits="false" boundaryCondition="true" constant="false" fbc:chemicalFormula="C6H11O7"/>
<species metaid="M_galt_e" id="M_galt_e" name="Galactitol_b" compartment="e" charge="0" initialConcentration="0" hasOnlySubstanceUnits="false" boundaryCondition="true" constant="false" fbc:chemicalFormula="C6H14O6"/>
<species metaid="M_galt1p_c" id="M_galt1p_c" name="Galactitol 1__phosphate" compartment="c" charge="-2" initialConcentration="0" hasOnlySubstanceUnits="false" boundaryCondition="false" constant="false" fbc:chemicalFormula="C6H13O9P"/>
<species metaid="M_galur_e" id="M_galur_e" name="D__Galacturonate_b" compartment="e" charge="-1" initialConcentration="0" hasOnlySubstanceUnits="false" boundaryCondition="true" constant="false" fbc:chemicalFormula="C6H9O7"/>
<species metaid="M_gam_e" id="M_gam_e" name="D__Glucosamine_b" compartment="e" charge="1" initialConcentration="0" hasOnlySubstanceUnits="false" boundaryCondition="true" constant="false" fbc:chemicalFormula="C6H14NO5"/>
<species metaid="M_gam1p_c" id="M_gam1p_c" name="D__Glucosamine 1__phosphate" compartment="c" charge="-1" initialConcentration="0" hasOnlySubstanceUnits="false" boundaryCondition="false" constant="false" fbc:chemicalFormula="C6H13NO8P"/>
<species metaid="M_gam6p_c" id="M_gam6p_c" name="D__Glucosamine 6__phosphate" compartment="c" charge="-1" initialConcentration="0" hasOnlySubstanceUnits="false" boundaryCondition="false" constant="false" fbc:chemicalFormula="C6H13NO8P"/>
<species metaid="M_gam6p_e" id="M_gam6p_e" name="D__Glucosamine 6__phosphate_b" compartment="e" charge="-1" initialConcentration="0" hasOnlySubstanceUnits="false" boundaryCondition="true" constant="false" fbc:chemicalFormula="C6H13NO8P"/>
<species metaid="M_gam6p_p" id="M_gam6p_p" name="D__Glucosamine 6__phosphate" compartment="p" charge="-1" initialConcentration="0" hasOnlySubstanceUnits="false" boundaryCondition="false" constant="false" fbc:chemicalFormula="C6H13NO8P"/>
<species metaid="M_gar_c" id="M_gar_c" name="N1__(5__Phospho__D__ribosyl)glycinamide" compartment="c" charge="-1" initialConcentration="0" hasOnlySubstanceUnits="false" boundaryCondition="false" constant="false" fbc:chemicalFormula="C7H14N2O8P"/>
<species metaid="M_gcald_c" id="M_gcald_c" name="Glycolaldehyde" compartment="c" charge="0" initialConcentration="0" hasOnlySubstanceUnits="false" boundaryCondition="false" constant="false" fbc:chemicalFormula="C2H4O2"/>
<species metaid="M_gchola_c" id="M_gchola_c" name="glycocholate" compartment="c" charge="-1" initialConcentration="0" hasOnlySubstanceUnits="false" boundaryCondition="false" constant="false" fbc:chemicalFormula="C26H43NO6"/>
<species metaid="M_gdnspate_c" id="M_gdnspate_c" name="glucosyl__4,4'__diaponeurosporenoate" compartment="c" charge="0" initialConcentration="0" hasOnlySubstanceUnits="false" boundaryCondition="false" constant="false" fbc:chemicalFormula="C36H50O7"/>
<species metaid="M_gdp_c" id="M_gdp_c" name="GDP" compartment="c" charge="-3" initialConcentration="0" hasOnlySubstanceUnits="false"

boundaryCondition="false" constant="false"
fbc:chemicalFormula="C10H12N5O11P2"/>
<species metaid="M_gdptp_c" id="M_gdptp_c" name="Guanosine
3'__diphosphate 5'__triphosphate" compartment="c" charge="-6"
initialConcentration="0" hasOnlySubstanceUnits="false"
boundaryCondition="false" constant="false"
fbc:chemicalFormula="C10H11N5O20P5"/>
<species metaid="M_gg3apn_c" id="M_gg3apn_c"
name="gamma__Glutamyl__3__aminopropiononitrile" compartment="c" charge="0"
initialConcentration="0" hasOnlySubstanceUnits="false"
boundaryCondition="false" constant="false" fbc:chemicalFormula="C8H13N3O3"/>
<species metaid="M_ggbca_c" id="M_ggbca_c"
name="gamma__Glutamyl__beta__cyanoalanine" compartment="c" charge="-1"
initialConcentration="0" hasOnlySubstanceUnits="false"
boundaryCondition="false" constant="false" fbc:chemicalFormula="C9H12N3O5"/>
<species metaid="M_ggdp_c" id="M_ggdp_c" name="Geranylgeranyl
diphosphate" compartment="c" charge="-3" initialConcentration="0"
hasOnlySubstanceUnits="false" boundaryCondition="false" constant="false"
fbc:chemicalFormula="C20H33O7P2"/>
<species metaid="M_glc__D__B_c" id="M_glc__D__B_c"
name="beta__D__Glucose" compartment="c" charge="0" initialConcentration="0"
hasOnlySubstanceUnits="false" boundaryCondition="false" constant="false"
fbc:chemicalFormula="C6H12O6"/>
<species metaid="M_glc__D_c" id="M_glc__D_c" name="D__Glucose"
compartment="c" charge="0" initialConcentration="0"
hasOnlySubstanceUnits="false" boundaryCondition="false" constant="false"
fbc:chemicalFormula="C6H12O6"/>
<species metaid="M_glc__D_e" id="M_glc__D_e" name="D__Glucose_b"
compartment="e" charge="0" initialConcentration="0"
hasOnlySubstanceUnits="false" boundaryCondition="true" constant="false"
fbc:chemicalFormula="C6H12O6"/>
<species metaid="M_glcn_c" id="M_glcn_c" name="D__Gluconate"
compartment="c" charge="-1" initialConcentration="0"
hasOnlySubstanceUnits="false" boundaryCondition="false" constant="false"
fbc:chemicalFormula="C6H11O7"/>
<species metaid="M_glcn_e" id="M_glcn_e" name="D__Gluconate_b"
compartment="e" charge="-1" initialConcentration="0"
hasOnlySubstanceUnits="false" boundaryCondition="true" constant="false"
fbc:chemicalFormula="C6H11O7"/>
<species metaid="M_glcp_SA_c" id="M_glcp_SA_c" name="Glucosyl
Phosphoglycerol (SA)" compartment="c" charge="-100" initialConcentration="0"
hasOnlySubstanceUnits="false" boundaryCondition="false" constant="false"
fbc:chemicalFormula="C996H1868N62O1116P150"/>
<species metaid="M_glcr_c" id="M_glcr_c" name="D__Glucarate"
compartment="c" charge="-2" initialConcentration="0"
hasOnlySubstanceUnits="false" boundaryCondition="false" constant="false"
fbc:chemicalFormula="C6H8O8"/>
<species metaid="M_glcr_e" id="M_glcr_e" name="D__Glucarate_b"
compartment="e" charge="-2" initialConcentration="0"
hasOnlySubstanceUnits="false" boundaryCondition="true" constant="false"
fbc:chemicalFormula="C6H8O8"/>
<species metaid="M_glcr_p" id="M_glcr_p" name="D__Glucarate"
compartment="p" charge="-2" initialConcentration="0"
hasOnlySubstanceUnits="false" boundaryCondition="false" constant="false"
fbc:chemicalFormula="C6H8O8"/>
<species metaid="M_glcur_c" id="M_glcur_c" name="D__Glucuronate"
compartment="c" charge="-1" initialConcentration="0"

hasOnlySubstanceUnits="false" boundaryCondition="false" constant="false"
fbc:chemicalFormula="C6H9O7"/>
<species metaid="M_glcur_e" id="M_glcur_e" name="D__Glucuronate_b"
compartment="e" charge="-1" initialConcentration="0"
hasOnlySubstanceUnits="false" boundaryCondition="true" constant="false"
fbc:chemicalFormula="C6H9O7"/>
<species metaid="M_glcur_p" id="M_glcur_p" name="D__Glucuronate"
compartment="p" charge="-1" initialConcentration="0"
hasOnlySubstanceUnits="false" boundaryCondition="false" constant="false"
fbc:chemicalFormula="C6H9O7"/>
<species metaid="M_glmeACP_c" id="M_glmeACP_c" name="Glutaryl__ACP
methyl ester" compartment="c" charge="1" initialConcentration="0"
hasOnlySubstanceUnits="false" boundaryCondition="false" constant="false"
fbc:chemicalFormula="C17H29N2O10PRS"/>
<species metaid="M_gln__L_c" id="M_gln__L_c" name="L__Glutamine"
compartment="c" charge="0" initialConcentration="0"
hasOnlySubstanceUnits="false" boundaryCondition="false" constant="false"
fbc:chemicalFormula="C5H10N2O3"/>
<species metaid="M_gln__L_e" id="M_gln__L_e" name="L__Glutamine_b"
compartment="e" charge="0" initialConcentration="0"
hasOnlySubstanceUnits="false" boundaryCondition="true" constant="false"
fbc:chemicalFormula="C5H10N2O3"/>
<species metaid="M_glu__D_c" id="M_glu__D_c" name="D__Glutamate"
compartment="c" charge="-1" initialConcentration="0"
hasOnlySubstanceUnits="false" boundaryCondition="false" constant="false"
fbc:chemicalFormula="C5H8NO4"/>
<species metaid="M_glu__L_c" id="M_glu__L_c" name="L__Glutamate"
compartment="c" charge="-1" initialConcentration="0"
hasOnlySubstanceUnits="false" boundaryCondition="false" constant="false"
fbc:chemicalFormula="C5H8NO4"/>
<species metaid="M_glu__L_e" id="M_glu__L_e" name="L__Glutamate_b"
compartment="e" charge="-1" initialConcentration="0"
hasOnlySubstanceUnits="false" boundaryCondition="true" constant="false"
fbc:chemicalFormula="C5H8NO4"/>
<species metaid="M_glu1sa_c" id="M_glu1sa_c" name="L__Glutamate
1__semialdehyde" compartment="c" charge="0" initialConcentration="0"
hasOnlySubstanceUnits="false" boundaryCondition="false" constant="false"
fbc:chemicalFormula="C5H9NO3"/>
<species metaid="M_glu5sa_c" id="M_glu5sa_c" name="L__Glutamate
5__semialdehyde" compartment="c" charge="0" initialConcentration="0"
hasOnlySubstanceUnits="false" boundaryCondition="false" constant="false"
fbc:chemicalFormula="C5H9NO3"/>
<species metaid="M_glutcoa_c" id="M_glutcoa_c" name="Glutaryl__CoA"
compartment="c" charge="-5" initialConcentration="0"
hasOnlySubstanceUnits="false" boundaryCondition="false" constant="false"
fbc:chemicalFormula="C26H37N7O19P3S"/>
<species metaid="M_glutrna_c" id="M_glutrna_c"
name="L__Glutamyl__tRNA(Glu)" compartment="c" charge="0"
initialConcentration="0" hasOnlySubstanceUnits="false"
boundaryCondition="false" constant="false" fbc:chemicalFormula="C5H7NO3R"/>
<species metaid="M_glx_c" id="M_glx_c" name="Glyoxylate"
compartment="c" charge="-1" initialConcentration="0"
hasOnlySubstanceUnits="false" boundaryCondition="false" constant="false"
fbc:chemicalFormula="C2H1O3"/>
<species metaid="M_gly__pro__L_c" id="M_gly__pro__L_c"
name="gly__pro__L" compartment="c" charge="0" initialConcentration="0"
hasOnlySubstanceUnits="false" boundaryCondition="false" constant="false"
fbc:chemicalFormula="C7H12N2O3"/>

<species metaid="M_gly__pro__L_e" id="M_gly__pro__L_e"
name="gly__pro__L_b" compartment="e" charge="0" initialConcentration="0"
hasOnlySubstanceUnits="false" boundaryCondition="true" constant="false"
fbc:chemicalFormula="C7H12N2O3"/>
<species metaid="M_gly_asn__L_c" id="M_gly_asn__L_c" name="gly__asn__L"
compartment="c" charge="0" initialConcentration="0"
hasOnlySubstanceUnits="false" boundaryCondition="false" constant="false"
fbc:chemicalFormula="C6H11N3O4"/>
<species metaid="M_gly_asn__L_e" id="M_gly_asn__L_e"
name="gly__asn__L_b" compartment="e" charge="0" initialConcentration="0"
hasOnlySubstanceUnits="false" boundaryCondition="true" constant="false"
fbc:chemicalFormula="C6H11N3O4"/>
<species metaid="M_gly_asp__L_c" id="M_gly_asp__L_c" name="gly__asp__L"
compartment="c" charge="-1" initialConcentration="0"
hasOnlySubstanceUnits="false" boundaryCondition="false" constant="false"
fbc:chemicalFormula="C6H9N2O5"/>
<species metaid="M_gly_asp__L_e" id="M_gly_asp__L_e"
name="gly__asp__L_b" compartment="e" charge="-1" initialConcentration="0"
hasOnlySubstanceUnits="false" boundaryCondition="true" constant="false"
fbc:chemicalFormula="C6H9N2O5"/>
<species metaid="M_gly_c" id="M_gly_c" name="Glycine" compartment="c"
charge="0" initialConcentration="0" hasOnlySubstanceUnits="false"
boundaryCondition="false" constant="false" fbc:chemicalFormula="C2H5NO2"/>
<species metaid="M_gly_cys_c" id="M_gly_cys_c" name="Gly__Cys"
compartment="c" charge="0" initialConcentration="0"
hasOnlySubstanceUnits="false" boundaryCondition="false" constant="false"
fbc:chemicalFormula="C5H10N2O3S"/>
<species metaid="M_gly_cys_e" id="M_gly_cys_e" name="Gly__Cys_b"
compartment="e" charge="0" initialConcentration="0"
hasOnlySubstanceUnits="false" boundaryCondition="true" constant="false"
fbc:chemicalFormula="C5H10N2O3S"/>
<species metaid="M_gly_e" id="M_gly_e" name="Glycine_b" compartment="e"
charge="0" initialConcentration="0" hasOnlySubstanceUnits="false"
boundaryCondition="true" constant="false" fbc:chemicalFormula="C2H5NO2"/>
<species metaid="M_gly_gln_c" id="M_gly_gln_c" name="Gly__Gln"
compartment="c" charge="0" initialConcentration="0"
hasOnlySubstanceUnits="false" boundaryCondition="false" constant="false"
fbc:chemicalFormula="C7H13N3O4"/>
<species metaid="M_gly_gln_e" id="M_gly_gln_e" name="Gly__Gln_b"
compartment="e" charge="0" initialConcentration="0"
hasOnlySubstanceUnits="false" boundaryCondition="true" constant="false"
fbc:chemicalFormula="C7H13N3O4"/>
<species metaid="M_gly_glu__L_c" id="M_gly_glu__L_c" name="gly__glu__L"
compartment="c" charge="-1" initialConcentration="0"
hasOnlySubstanceUnits="false" boundaryCondition="false" constant="false"
fbc:chemicalFormula="C7H11N2O5"/>
<species metaid="M_gly_glu__L_e" id="M_gly_glu__L_e"
name="gly__glu__L_b" compartment="e" charge="-1" initialConcentration="0"
hasOnlySubstanceUnits="false" boundaryCondition="true" constant="false"
fbc:chemicalFormula="C7H11N2O5"/>
<species metaid="M_gly_leu_c" id="M_gly_leu_c" name="Gly__Leu"
compartment="c" charge="0" initialConcentration="0"
hasOnlySubstanceUnits="false" boundaryCondition="false" constant="false"
fbc:chemicalFormula="C8H16N2O3"/>
<species metaid="M_gly_leu_e" id="M_gly_leu_e" name="Gly__Leu_b"
compartment="e" charge="0" initialConcentration="0"
hasOnlySubstanceUnits="false" boundaryCondition="true" constant="false"
fbc:chemicalFormula="C8H16N2O3"/>

```
      <species metaid="M_gly_met_c" id="M_gly_met_c" name="Gly__Met"
compartment="c" charge="0" initialConcentration="0"
hasOnlySubstanceUnits="false" boundaryCondition="false" constant="false"
fbc:chemicalFormula="C7H14N2O3S"/>
      <species metaid="M_gly_met_e" id="M_gly_met_e" name="Gly__Met_b"
compartment="e" charge="0" initialConcentration="0"
hasOnlySubstanceUnits="false" boundaryCondition="true" constant="false"
fbc:chemicalFormula="C7H14N2O3S"/>
      <species metaid="M_gly_phe_c" id="M_gly_phe_c" name="Gly__Phe"
compartment="c" charge="0" initialConcentration="0"
hasOnlySubstanceUnits="false" boundaryCondition="false" constant="false"
fbc:chemicalFormula="C11H14N2O3"/>
      <species metaid="M_gly_phe_e" id="M_gly_phe_e" name="Gly__Phe_b"
compartment="e" charge="0" initialConcentration="0"
hasOnlySubstanceUnits="false" boundaryCondition="true" constant="false"
fbc:chemicalFormula="C11H14N2O3"/>
      <species metaid="M_gly_tyr_c" id="M_gly_tyr_c" name="Gly__Tyr"
compartment="c" charge="0" initialConcentration="0"
hasOnlySubstanceUnits="false" boundaryCondition="false" constant="false"
fbc:chemicalFormula="C11H14N2O4"/>
      <species metaid="M_gly_tyr_e" id="M_gly_tyr_e" name="Gly__Tyr_b"
compartment="e" charge="0" initialConcentration="0"
hasOnlySubstanceUnits="false" boundaryCondition="true" constant="false"
fbc:chemicalFormula="C11H14N2O4"/>
      <species metaid="M_glyald_c" id="M_glyald_c" name="D__Glyceraldehyde"
compartment="c" charge="0" initialConcentration="0"
hasOnlySubstanceUnits="false" boundaryCondition="false" constant="false"
fbc:chemicalFormula="C3H6O3"/>
      <species metaid="M_glyald_e" id="M_glyald_e" name="D__Glyceraldehyde_b"
compartment="e" charge="0" initialConcentration="0"
hasOnlySubstanceUnits="false" boundaryCondition="true" constant="false"
fbc:chemicalFormula="C3H6O3"/>
      <species metaid="M_glyb_c" id="M_glyb_c" name="Glycine betaine"
compartment="c" charge="0" initialConcentration="0"
hasOnlySubstanceUnits="false" boundaryCondition="false" constant="false"
fbc:chemicalFormula="C5H11NO2"/>
      <species metaid="M_glyb_e" id="M_glyb_e" name="Glycine betaine_b"
compartment="e" charge="0" initialConcentration="0"
hasOnlySubstanceUnits="false" boundaryCondition="true" constant="false"
fbc:chemicalFormula="C5H11NO2"/>
      <species metaid="M_glyc__R_c" id="M_glyc__R_c" name="(R)__Glycerate"
compartment="c" charge="-1" initialConcentration="0"
hasOnlySubstanceUnits="false" boundaryCondition="false" constant="false"
fbc:chemicalFormula="C3H5O4"/>
      <species metaid="M_glyc_c" id="M_glyc_c" name="Glycerol"
compartment="c" charge="0" initialConcentration="0"
hasOnlySubstanceUnits="false" boundaryCondition="false" constant="false"
fbc:chemicalFormula="C3H8O3"/>
      <species metaid="M_glyc_e" id="M_glyc_e" name="Glycerol_b"
compartment="e" charge="0" initialConcentration="0"
hasOnlySubstanceUnits="false" boundaryCondition="true" constant="false"
fbc:chemicalFormula="C3H8O3"/>
      <species metaid="M_glyc3p_c" id="M_glyc3p_c" name="Glycerol
3__phosphate" compartment="c" charge="-2" initialConcentration="0"
hasOnlySubstanceUnits="false" boundaryCondition="false" constant="false"
fbc:chemicalFormula="C3H7O6P"/>
      <species metaid="M_glyc3p_e" id="M_glyc3p_e" name="Glycerol
3__phosphate_b" compartment="e" charge="-2" initialConcentration="0"
```

```
hasOnlySubstanceUnits="false" boundaryCondition="true" constant="false"
fbc:chemicalFormula="C3H7O6P"/>
      <species metaid="M_glyclt_c" id="M_glyclt_c" name="Glycolate"
compartment="c" charge="-1" initialConcentration="0"
hasOnlySubstanceUnits="false" boundaryCondition="false" constant="false"
fbc:chemicalFormula="C2H3O3"/>
      <species metaid="M_glyclt_e" id="M_glyclt_e" name="Glycolate_b"
compartment="e" charge="-1" initialConcentration="0"
hasOnlySubstanceUnits="false" boundaryCondition="true" constant="false"
fbc:chemicalFormula="C2H3O3"/>
      <species metaid="M_glycogen_c" id="M_glycogen_c" name="glycogen"
compartment="c" charge="0" initialConcentration="0"
hasOnlySubstanceUnits="false" boundaryCondition="false" constant="false"
fbc:chemicalFormula="C6H10O5"/>
      <species metaid="M_glyteala_c" id="M_glyteala_c"
name="glycerol_teichoic_acid_n=45__linked__D__ala_substituted"
compartment="c" charge="-48" initialConcentration="0"
hasOnlySubstanceUnits="false" boundaryCondition="false" constant="false"
fbc:chemicalFormula=""/>
      <species metaid="M_glytglc_c" id="M_glytglc_c"
name="glycerol_teichoic_acid_n=45__linked__glucose_substituted"
compartment="c" charge="-48" initialConcentration="0"
hasOnlySubstanceUnits="false" boundaryCondition="false" constant="false"
fbc:chemicalFormula=""/>
      <species metaid="M_glytrna_c" id="M_glytrna_c" name="Glycyl__tRNA(Gly)"
compartment="c" charge="1" initialConcentration="0"
hasOnlySubstanceUnits="false" boundaryCondition="false" constant="false"
fbc:chemicalFormula="C2H4NOR"/>
      <species metaid="M_glytu_c" id="M_glytu_c"
name="glycerol_teichoic_acid_n=45__linked__unsubstituted" compartment="c"
charge="-48" initialConcentration="0" hasOnlySubstanceUnits="false"
boundaryCondition="false" constant="false" fbc:chemicalFormula=""/>
      <species metaid="M_gmhep1p_c" id="M_gmhep1p_c"
name="D__Glycero__D__manno__heptose 1__phosphate" compartment="c" charge="-2"
initialConcentration="0" hasOnlySubstanceUnits="false"
boundaryCondition="false" constant="false" fbc:chemicalFormula="C7H13O10P"/>
      <species metaid="M_gmhep7p_c" id="M_gmhep7p_c"
name="D__Glycero__D__manno__heptose 7__phosphate" compartment="c" charge="-2"
initialConcentration="0" hasOnlySubstanceUnits="false"
boundaryCondition="false" constant="false" fbc:chemicalFormula="C7H13O10P"/>
      <species metaid="M_gmhep17bp_c" id="M_gmhep17bp_c"
name="D__Glycero__D__manno__heptose 1,7__bisphosphate" compartment="c"
charge="-4" initialConcentration="0" hasOnlySubstanceUnits="false"
boundaryCondition="false" constant="false" fbc:chemicalFormula="C7H12O13P2"/>
      <species metaid="M_gmp_c" id="M_gmp_c" name="GMP" compartment="c"
charge="-2" initialConcentration="0" hasOnlySubstanceUnits="false"
boundaryCondition="false" constant="false"
fbc:chemicalFormula="C10H12N5O8P"/>
      <species metaid="M_grdp_c" id="M_grdp_c" name="Geranyl diphosphate"
compartment="c" charge="-3" initialConcentration="0"
hasOnlySubstanceUnits="false" boundaryCondition="false" constant="false"
fbc:chemicalFormula="C10H17O7P2"/>
      <species metaid="M_gsn_c" id="M_gsn_c" name="Guanosine" compartment="c"
charge="0" initialConcentration="0" hasOnlySubstanceUnits="false"
boundaryCondition="false" constant="false" fbc:chemicalFormula="C10H13N5O5"/>
      <species metaid="M_gsn_e" id="M_gsn_e" name="Guanosine_b"
compartment="e" charge="0" initialConcentration="0"
```

hasOnlySubstanceUnits="false" boundaryCondition="true" constant="false" fbc:chemicalFormula="C10H13N5O5"/>
<species metaid="M_gsn_p" id="M_gsn_p" name="Guanosine" compartment="p" charge="0" initialConcentration="0" hasOnlySubstanceUnits="false" boundaryCondition="false" constant="false" fbc:chemicalFormula="C10H13N5O5"/>
<species metaid="M_gtca1_c" id="M_gtca1_c" name="glycerol teichoic acid (n=25), unlinked, unsubstituted" compartment="c" charge="-25" initialConcentration="0" hasOnlySubstanceUnits="false" boundaryCondition="false" constant="false" fbc:chemicalFormula="C91H178N2O136P25"/>
<species metaid="M_gtca2_c" id="M_gtca2_c" name="glycerol teichoic acid (n=25), unlinked, D__ala substituted" compartment="c" charge="0" initialConcentration="0" hasOnlySubstanceUnits="false" boundaryCondition="false" constant="false" fbc:chemicalFormula="C166H328N27O161P25"/>
<species metaid="M_gtca3_c" id="M_gtca3_c" name="glycerol teichoic acid (n=25), unlinked, glucose substituted" compartment="c" charge="-25" initialConcentration="0" hasOnlySubstanceUnits="false" boundaryCondition="false" constant="false" fbc:chemicalFormula="C241H428N2O261P25"/>
<species metaid="M_gthrd_c" id="M_gthrd_c" name="Reduced glutathione" compartment="c" charge="-1" initialConcentration="0" hasOnlySubstanceUnits="false" boundaryCondition="false" constant="false" fbc:chemicalFormula="C10H16N3O6S"/>
<species metaid="M_gthrd2_c" id="M_gthrd2_c" name="Oxidized_glutathione" compartment="c" charge="-2" initialConcentration="0" hasOnlySubstanceUnits="false" boundaryCondition="false" constant="false" fbc:chemicalFormula=""/>
<species metaid="M_gtp_c" id="M_gtp_c" name="GTP" compartment="c" charge="-4" initialConcentration="0" hasOnlySubstanceUnits="false" boundaryCondition="false" constant="false" fbc:chemicalFormula="C10H12N5O14P3"/>
<species metaid="M_gua_c" id="M_gua_c" name="Guanine" compartment="c" charge="0" initialConcentration="0" hasOnlySubstanceUnits="false" boundaryCondition="false" constant="false" fbc:chemicalFormula="C5H5N5O"/>
<species metaid="M_gua_e" id="M_gua_e" name="Guanine_b" compartment="e" charge="0" initialConcentration="0" hasOnlySubstanceUnits="false" boundaryCondition="true" constant="false" fbc:chemicalFormula="C5H5N5O"/>
<species metaid="M_h_c" id="M_h_c" name="H+" compartment="c" charge="1" initialConcentration="0" hasOnlySubstanceUnits="false" boundaryCondition="false" constant="false" fbc:chemicalFormula="H"/>
<species metaid="M_h_e" id="M_h_e" name="H+_b" compartment="e" charge="1" initialConcentration="0" hasOnlySubstanceUnits="false" boundaryCondition="true" constant="false" fbc:chemicalFormula="H"/>
<species metaid="M_h_p" id="M_h_p" name="H+" compartment="p" charge="1" initialConcentration="0" hasOnlySubstanceUnits="false" boundaryCondition="false" constant="false" fbc:chemicalFormula="H"/>
<species metaid="M_h2o_c" id="M_h2o_c" name="H2O" compartment="c" charge="0" initialConcentration="0" hasOnlySubstanceUnits="false" boundaryCondition="false" constant="false" fbc:chemicalFormula="H2O"/>
<species metaid="M_h2o_e" id="M_h2o_e" name="H2O_b" compartment="e" charge="0" initialConcentration="0" hasOnlySubstanceUnits="false" boundaryCondition="true" constant="false" fbc:chemicalFormula="H2O"/>
<species metaid="M_h2o_p" id="M_h2o_p" name="H2O" compartment="p" charge="0" initialConcentration="0" hasOnlySubstanceUnits="false" boundaryCondition="false" constant="false" fbc:chemicalFormula="H2O"/>
<species metaid="M_h2o2_c" id="M_h2o2_c" name="Hydrogen peroxide" compartment="c" charge="0" initialConcentration="0"

```
hasOnlySubstanceUnits="false" boundaryCondition="false" constant="false"
fbc:chemicalFormula="H2O2"/>
      <species metaid="M_h2s_c" id="M_h2s_c" name="Hydrogen sulfide"
compartment="c" charge="-1" initialConcentration="0"
hasOnlySubstanceUnits="false" boundaryCondition="false" constant="false"
fbc:chemicalFormula="H2S"/>
      <species metaid="M_hcarb_c" id="M_hcarb_c" name="Holo__carboxylase"
compartment="c" charge="0" initialConcentration="0"
hasOnlySubstanceUnits="false" boundaryCondition="false" constant="false"
fbc:chemicalFormula=""/>
      <species metaid="M_hco3_c" id="M_hco3_c" name="Bicarbonate"
compartment="c" charge="-1" initialConcentration="0"
hasOnlySubstanceUnits="false" boundaryCondition="false" constant="false"
fbc:chemicalFormula="CHO3"/>
      <species metaid="M_hcys__L_c" id="M_hcys__L_c" name="L__Homocysteine"
compartment="c" charge="0" initialConcentration="0"
hasOnlySubstanceUnits="false" boundaryCondition="false" constant="false"
fbc:chemicalFormula="C4H9NO2S"/>
      <species metaid="M_hdca_c" id="M_hdca_c" name="Hexadecanoate
(n__C16:0)" compartment="c" charge="-1" initialConcentration="0"
hasOnlySubstanceUnits="false" boundaryCondition="false" constant="false"
fbc:chemicalFormula="C16H31O2"/>
      <species metaid="M_hdca_e" id="M_hdca_e" name="Hexadecanoate
(n__C16:0)_b" compartment="e" charge="-1" initialConcentration="0"
hasOnlySubstanceUnits="false" boundaryCondition="true" constant="false"
fbc:chemicalFormula="C16H31O2"/>
      <species metaid="M_hdcea_c" id="M_hdcea_c" name="Hexadecenoate
(n__C16:1)" compartment="c" charge="-1" initialConcentration="0"
hasOnlySubstanceUnits="false" boundaryCondition="false" constant="false"
fbc:chemicalFormula="C16H29O2"/>
      <species metaid="M_hdd2coa_c" id="M_hdd2coa_c"
name="trans__Hexadec__2__enoyl__CoA" compartment="c" charge="-4"
initialConcentration="0" hasOnlySubstanceUnits="false"
boundaryCondition="false" constant="false"
fbc:chemicalFormula="C37H60N7O17P3S"/>
      <species metaid="M_hdeACP_c" id="M_hdeACP_c"
name="cis__hexadec__9__enoyl__[acyl__carrier protein] (n__C16:1)"
compartment="c" charge="-1" initialConcentration="0"
hasOnlySubstanceUnits="false" boundaryCondition="false" constant="false"
fbc:chemicalFormula="C27H49N2O8PRS"/>
      <species metaid="M_hedacp_c" id="M_hedacp_c" name="hexadecanoyl__acp"
compartment="c" charge="-1" initialConcentration="0"
hasOnlySubstanceUnits="false" boundaryCondition="false" constant="false"
fbc:chemicalFormula="C27H51N2O8PRS"/>
      <species metaid="M_hemeA__1_c" id="M_hemeA__1_c" name="Heme A"
compartment="c" charge="-6" initialConcentration="0"
hasOnlySubstanceUnits="false" boundaryCondition="false" constant="false"
fbc:chemicalFormula="C49H54FeN4O6"/>
      <species metaid="M_hemeO_c" id="M_hemeO_c" name="Heme O"
compartment="c" charge="0" initialConcentration="0"
hasOnlySubstanceUnits="false" boundaryCondition="false" constant="false"
fbc:chemicalFormula="C49H56FeN4O5"/>
      <species metaid="M_hepdp_c" id="M_hepdp_c"
name="all__trans__Heptaprenyl diphosphate" compartment="c" charge="-3"
initialConcentration="0" hasOnlySubstanceUnits="false"
boundaryCondition="false" constant="false" fbc:chemicalFormula="C35H57O7P2"/>
      <species metaid="M_hethmpp_c" id="M_hethmpp_c" name="hydroxyethyl
thiamin diphosphate" compartment="c" charge="-1" initialConcentration="0"
```

```
hasOnlySubstanceUnits="false" boundaryCondition="false" constant="false"
fbc:chemicalFormula="C14H20N4O8P2S"/>
      <species metaid="M_hexACP_c" id="M_hexACP_c" name="Hexanoyl__ACP
(n__C6:0ACP)" compartment="c" charge="0" initialConcentration="0"
hasOnlySubstanceUnits="false" boundaryCondition="false" constant="false"
fbc:chemicalFormula="C17H31N2O8PRS"/>
      <species metaid="M_hexdp_c" id="M_hexdp_c" name="all__trans__Hexaprenyl
diphosphate" compartment="c" charge="-3" initialConcentration="0"
hasOnlySubstanceUnits="false" boundaryCondition="false" constant="false"
fbc:chemicalFormula="C30H49O7P2"/>
      <species metaid="M_hg2_e" id="M_hg2_e" name="Hg2+_b" compartment="e"
charge="2" initialConcentration="0" hasOnlySubstanceUnits="false"
boundaryCondition="true" constant="false" fbc:chemicalFormula="Hg"/>
      <species metaid="M_his__L_c" id="M_his__L_c" name="L__Histidine"
compartment="c" charge="0" initialConcentration="0"
hasOnlySubstanceUnits="false" boundaryCondition="false" constant="false"
fbc:chemicalFormula="C6H9N3O2"/>
      <species metaid="M_his__L_e" id="M_his__L_e" name="L__Histidine_b"
compartment="e" charge="0" initialConcentration="0"
hasOnlySubstanceUnits="false" boundaryCondition="true" constant="false"
fbc:chemicalFormula="C6H9N3O2"/>
      <species metaid="M_hisp_c" id="M_hisp_c" name="L__Histidinol phosphate"
compartment="c" charge="-1" initialConcentration="0"
hasOnlySubstanceUnits="false" boundaryCondition="false" constant="false"
fbc:chemicalFormula="C6H11N3O4P"/>
      <species metaid="M_histd_c" id="M_histd_c" name="L__Histidinol"
compartment="c" charge="1" initialConcentration="0"
hasOnlySubstanceUnits="false" boundaryCondition="false" constant="false"
fbc:chemicalFormula="C6H12N3O"/>
      <species metaid="M_hmbil_c" id="M_hmbil_c" name="Hydroxymethylbilane"
compartment="c" charge="-8" initialConcentration="0"
hasOnlySubstanceUnits="false" boundaryCondition="false" constant="false"
fbc:chemicalFormula="C40H38N4O17"/>
      <species metaid="M_hmccms_c" id="M_hmccms_c"
name="2__Hydroxy__5__methyl__cis,cis__muconic semialdehyde" compartment="c"
charge="-1" initialConcentration="0" hasOnlySubstanceUnits="false"
boundaryCondition="false" constant="false" fbc:chemicalFormula="C7H7O4"/>
      <species metaid="M_hmgcoa_c" id="M_hmgcoa_c"
name="Hydroxymethylglutaryl__CoA" compartment="c" charge="-5"
initialConcentration="0" hasOnlySubstanceUnits="false"
boundaryCondition="false" constant="false"
fbc:chemicalFormula="C27H39N7O20P3S"/>
      <species metaid="M_hom__L_c" id="M_hom__L_c" name="L__Homoserine"
compartment="c" charge="0" initialConcentration="0"
hasOnlySubstanceUnits="false" boundaryCondition="false" constant="false"
fbc:chemicalFormula="C4H9NO3"/>
      <species metaid="M_hom__L_e" id="M_hom__L_e" name="L__Homoserine_b"
compartment="e" charge="0" initialConcentration="0"
hasOnlySubstanceUnits="false" boundaryCondition="true" constant="false"
fbc:chemicalFormula="C4H9NO3"/>
      <species metaid="M_hpglu_c" id="M_hpglu_c"
name="Tetrahydropteroyltri__L__glutamate" compartment="c" charge="-4"
initialConcentration="0" hasOnlySubstanceUnits="false"
boundaryCondition="false" constant="false"
fbc:chemicalFormula="C24H34N8O12"/>
      <species metaid="M_hpyr_c" id="M_hpyr_c" name="Hydroxypyruvate"
compartment="c" charge="-1" initialConcentration="0"
```

```
hasOnlySubstanceUnits="false" boundaryCondition="false" constant="false"
fbc:chemicalFormula="C3H3O4"/>
      <species metaid="M_hqn_c" id="M_hqn_c" name="Hydroquinone"
compartment="c" charge="0" initialConcentration="0"
hasOnlySubstanceUnits="false" boundaryCondition="false" constant="false"
fbc:chemicalFormula="C6H6O2"/>
      <species metaid="M_htdol__L_c" id="M_htdol__L_c" name="L__Histidinal"
compartment="c" charge="1" initialConcentration="0"
hasOnlySubstanceUnits="false" boundaryCondition="false" constant="false"
fbc:chemicalFormula="C6H10N3O"/>
      <species metaid="M_hx2coa_c" id="M_hx2coa_c"
name="trans__Hex__2__enoyl__CoA" compartment="c" charge="-4"
initialConcentration="0" hasOnlySubstanceUnits="false"
boundaryCondition="false" constant="false"
fbc:chemicalFormula="C27H40N7O17P3S"/>
      <species metaid="M_hxa_e" id="M_hxa_e" name="Hexanoate (n__C6:0)_b"
compartment="e" charge="-1" initialConcentration="0"
hasOnlySubstanceUnits="false" boundaryCondition="true" constant="false"
fbc:chemicalFormula="C6H11O2"/>
      <species metaid="M_hxan_c" id="M_hxan_c" name="Hypoxanthine"
compartment="c" charge="0" initialConcentration="0"
hasOnlySubstanceUnits="false" boundaryCondition="false" constant="false"
fbc:chemicalFormula="C5H4N4O"/>
      <species metaid="M_hxan_e" id="M_hxan_e" name="Hypoxanthine_b"
compartment="e" charge="0" initialConcentration="0"
hasOnlySubstanceUnits="false" boundaryCondition="true" constant="false"
fbc:chemicalFormula="C5H4N4O"/>
      <species metaid="M_hxcoa_c" id="M_hxcoa_c" name="Hexanoyl__CoA
(n__C6:0CoA)" compartment="c" charge="-4" initialConcentration="0"
hasOnlySubstanceUnits="false" boundaryCondition="false" constant="false"
fbc:chemicalFormula="C27H42N7O17P3S"/>
      <species metaid="M_iad_c" id="M_iad_c" name="Indole__3__acetamide"
compartment="c" charge="0" initialConcentration="0"
hasOnlySubstanceUnits="false" boundaryCondition="false" constant="false"
fbc:chemicalFormula="C10H10N2O"/>
      <species metaid="M_iaidsg3p_c" id="M_iaidsg3p_c"
name="1__anteisoheptadecanoyl__sn__glycerol_3__phosphate" compartment="c"
charge="-1" initialConcentration="0" hasOnlySubstanceUnits="false"
boundaryCondition="false" constant="false" fbc:chemicalFormula=""/>
      <species metaid="M_ibcoa_c" id="M_ibcoa_c" name="Isobutyryl__CoA"
compartment="c" charge="-4" initialConcentration="0"
hasOnlySubstanceUnits="false" boundaryCondition="false" constant="false"
fbc:chemicalFormula="C25H38N7O17P3S"/>
      <species metaid="M_ichor_c" id="M_ichor_c" name="Isochorismate"
compartment="c" charge="-2" initialConcentration="0"
hasOnlySubstanceUnits="false" boundaryCondition="false" constant="false"
fbc:chemicalFormula="C10H8O6"/>
      <species metaid="M_icit_c" id="M_icit_c" name="Isocitrate"
compartment="c" charge="-3" initialConcentration="0"
hasOnlySubstanceUnits="false" boundaryCondition="false" constant="false"
fbc:chemicalFormula="C6H5O7"/>
      <species metaid="M_idp_c" id="M_idp_c" name="IDP" compartment="c"
charge="-3" initialConcentration="0" hasOnlySubstanceUnits="false"
boundaryCondition="false" constant="false"
fbc:chemicalFormula="C10H11N4O11P2"/>
      <species metaid="M_ile__L_c" id="M_ile__L_c" name="L__Isoleucine"
compartment="c" charge="0" initialConcentration="0"
```

```
hasOnlySubstanceUnits="false" boundaryCondition="false" constant="false"
fbc:chemicalFormula="C6H13NO2"/>
      <species metaid="M_ile__L_e" id="M_ile__L_e" name="L__Isoleucine_b"
compartment="e" charge="0" initialConcentration="0"
hasOnlySubstanceUnits="false" boundaryCondition="true" constant="false"
fbc:chemicalFormula="C6H13NO2"/>
      <species metaid="M_iletrna_c" id="M_iletrna_c"
name="L__Isoleucyl__tRNA(Ile)" compartment="c" charge="1"
initialConcentration="0" hasOnlySubstanceUnits="false"
boundaryCondition="false" constant="false" fbc:chemicalFormula="C6H12NOR"/>
      <species metaid="M_im4ac_c" id="M_im4ac_c" name="Imidazole__4__acetate"
compartment="c" charge="-1" initialConcentration="0"
hasOnlySubstanceUnits="false" boundaryCondition="false" constant="false"
fbc:chemicalFormula="C5H5N2O2"/>
      <species metaid="M_im4act_c" id="M_im4act_c"
name="Imidazole__4__acetaldehyde" compartment="c" charge="0"
initialConcentration="0" hasOnlySubstanceUnits="false"
boundaryCondition="false" constant="false" fbc:chemicalFormula="C5H6N2O"/>
      <species metaid="M_imacp_c" id="M_imacp_c"
name="3__(Imidazol__4__yl)__2__oxopropyl phosphate" compartment="c" charge="-
2" initialConcentration="0" hasOnlySubstanceUnits="false"
boundaryCondition="false" constant="false" fbc:chemicalFormula="C6H7N2O5P"/>
      <species metaid="M_imp_c" id="M_imp_c" name="IMP" compartment="c"
charge="-2" initialConcentration="0" hasOnlySubstanceUnits="false"
boundaryCondition="false" constant="false"
fbc:chemicalFormula="C10H11N4O8P"/>
      <species metaid="M_ind3ac_c" id="M_ind3ac_c" name="Indole__3__acetate"
compartment="c" charge="-1" initialConcentration="0"
hasOnlySubstanceUnits="false" boundaryCondition="false" constant="false"
fbc:chemicalFormula="C10H8NO2"/>
      <species metaid="M_indole_c" id="M_indole_c" name="Indole"
compartment="c" charge="0" initialConcentration="0"
hasOnlySubstanceUnits="false" boundaryCondition="false" constant="false"
fbc:chemicalFormula="C8H7N"/>
      <species metaid="M_inost_c" id="M_inost_c" name="myo__Inositol"
compartment="c" charge="0" initialConcentration="0"
hasOnlySubstanceUnits="false" boundaryCondition="false" constant="false"
fbc:chemicalFormula="C6H12O6"/>
      <species metaid="M_inost_e" id="M_inost_e" name="myo__Inositol_b"
compartment="e" charge="0" initialConcentration="0"
hasOnlySubstanceUnits="false" boundaryCondition="true" constant="false"
fbc:chemicalFormula="C6H12O6"/>
      <species metaid="M_ins_c" id="M_ins_c" name="Inosine" compartment="c"
charge="0" initialConcentration="0" hasOnlySubstanceUnits="false"
boundaryCondition="false" constant="false" fbc:chemicalFormula="C10H12N4O5"/>
      <species metaid="M_ins_e" id="M_ins_e" name="Inosine_b" compartment="e"
charge="0" initialConcentration="0" hasOnlySubstanceUnits="false"
boundaryCondition="true" constant="false" fbc:chemicalFormula="C10H12N4O5"/>
      <species metaid="M_ipdp_c" id="M_ipdp_c" name="Isopentenyl diphosphate"
compartment="c" charge="-3" initialConcentration="0"
hasOnlySubstanceUnits="false" boundaryCondition="false" constant="false"
fbc:chemicalFormula="C5H9O7P2"/>
      <species metaid="M_isetac_e" id="M_isetac_e" name="Isethionic acid_b"
compartment="e" charge="-1" initialConcentration="0"
hasOnlySubstanceUnits="false" boundaryCondition="true" constant="false"
fbc:chemicalFormula="C2H5O4S"/>
      <species metaid="M_ish24s_c" id="M_ish24s_c"
name="Isohexadecanoyllipoteichoic_acid_n=24__linked__N__acetyl__D__glucosamin
```

e" compartment="c" charge="-24" initialConcentration="0" hasOnlySubstanceUnits="false" boundaryCondition="false" constant="false" fbc:chemicalFormula=""/>
<species metaid="M_iso24ds_c" id="M_iso24ds_c" name="Isoheptadecanoyllipoteichoic_acid_n=24__linked__D__alanine_substituted" compartment="c" charge="-24" initialConcentration="0" hasOnlySubstanceUnits="false" boundaryCondition="false" constant="false" fbc:chemicalFormula=""/>
<species metaid="M_isobutACP_c" id="M_isobutACP_c" name="isobutyryl__ACP" compartment="c" charge="-1" initialConcentration="0" hasOnlySubstanceUnits="false" boundaryCondition="false" constant="false" fbc:chemicalFormula="C15H27N2O8PRS"/>
<species metaid="M_isod24s_c" id="M_isod24s_c" name="Isotetradecanoyllipoteichoic_acid_n=24__linked__glucose_substituted" compartment="c" charge="-24" initialConcentration="0" hasOnlySubstanceUnits="false" boundaryCondition="false" constant="false" fbc:chemicalFormula=""/>
<species metaid="M_isodec24s_c" id="M_isodec24s_c" name="Isohexadecanoyllipoteichoic_acid_n=24__linked__glucose_substituted" compartment="c" charge="-24" initialConcentration="0" hasOnlySubstanceUnits="false" boundaryCondition="false" constant="false" fbc:chemicalFormula=""/>
<species metaid="M_isoh24ds_c" id="M_isoh24ds_c" name="Isohexadecanoyllipoteichoic_acid_n=24__linked__D__alanine_substituted" compartment="c" charge="-24" initialConcentration="0" hasOnlySubstanceUnits="false" boundaryCondition="false" constant="false" fbc:chemicalFormula=""/>
<species metaid="M_isoh24s_c" id="M_isoh24s_c" name="Isoheptadecanoyllipoteichoic_acid_n=24__linked__N__acetyl__D__glucosamine" compartment="c" charge="-24" initialConcentration="0" hasOnlySubstanceUnits="false" boundaryCondition="false" constant="false" fbc:chemicalFormula=""/>
<species metaid="M_isohcard_c" id="M_isohcard_c" name="Isoheptadecanoylcardiolipin_B._subtilis" compartment="c" charge="-2" initialConcentration="0" hasOnlySubstanceUnits="false" boundaryCondition="false" constant="false" fbc:chemicalFormula=""/>
<species metaid="M_isohdcard_c" id="M_isohdcard_c" name="Isohexadecanoylcardiolipin_B._subtilis" compartment="c" charge="-2" initialConcentration="0" hasOnlySubstanceUnits="false" boundaryCondition="false" constant="false" fbc:chemicalFormula=""/>
<species metaid="M_isohdt24u_c" id="M_isohdt24u_c" name="Isohexadecanoyllipoteichoic_acid_n=24__linked__unsubstituted" compartment="c" charge="-24" initialConcentration="0" hasOnlySubstanceUnits="false" boundaryCondition="false" constant="false" fbc:chemicalFormula=""/>
<species metaid="M_isohep24s_c" id="M_isohep24s_c" name="Isoheptadecanoyllipoteichoic_acid_n=24__linked__glucose_substituted" compartment="c" charge="-24" initialConcentration="0" hasOnlySubstanceUnits="false" boundaryCondition="false" constant="false" fbc:chemicalFormula=""/>
<species metaid="M_isolp24u_c" id="M_isolp24u_c" name="Isoheptadecanoyllipoteichoic_acid_n=24__linked__unsubstituted" compartment="c" charge="-24" initialConcentration="0" hasOnlySubstanceUnits="false" boundaryCondition="false" constant="false" fbc:chemicalFormula=""/>
<species metaid="M_isop24ds_c" id="M_isop24ds_c" name="Isopentadecanoyllipoteichoic_acid_n=24__linked__D__alanine_substituted" compartment="c" charge="-24" initialConcentration="0"

```
hasOnlySubstanceUnits="false" boundaryCondition="false" constant="false"
fbc:chemicalFormula=""/>
      <species metaid="M_isop24glcs_c" id="M_isop24glcs_c"
name="Isopentadecanoyllipoteichoic_acid_n=24__linked__glucose_substituted"
compartment="c" charge="-24" initialConcentration="0"
hasOnlySubstanceUnits="false" boundaryCondition="false" constant="false"
fbc:chemicalFormula=""/>
      <species metaid="M_isop24gsms_c" id="M_isop24gsms_c"
name="Isopentadecanoyllipoteichoic_acid_n=24__linked__N__acetyl__D__glucosami
ne" compartment="c" charge="-24" initialConcentration="0"
hasOnlySubstanceUnits="false" boundaryCondition="false" constant="false"
fbc:chemicalFormula=""/>
      <species metaid="M_isop24u_c" id="M_isop24u_c"
name="Isopentadecanoyllipoteichoic_acid_n=24__linked__unsubstituted"
compartment="c" charge="-24" initialConcentration="0"
hasOnlySubstanceUnits="false" boundaryCondition="false" constant="false"
fbc:chemicalFormula=""/>
      <species metaid="M_isopcard_c" id="M_isopcard_c"
name="Isopentadecanoylcardiolipin_B._subtilis" compartment="c" charge="-2"
initialConcentration="0" hasOnlySubstanceUnits="false"
boundaryCondition="false" constant="false" fbc:chemicalFormula=""/>
      <species metaid="M_isot24ds_c" id="M_isot24ds_c"
name="Isotetradecanoyllipoteichoic_acid_n=24__linked__D__alanine_substituted"
compartment="c" charge="-24" initialConcentration="0"
hasOnlySubstanceUnits="false" boundaryCondition="false" constant="false"
fbc:chemicalFormula=""/>
      <species metaid="M_isot24s_c" id="M_isot24s_c"
name="Isotetradecanoyllipoteichoic_acid_n=24__linked__N__acetyl__D__glucosami
ne" compartment="c" charge="-24" initialConcentration="0"
hasOnlySubstanceUnits="false" boundaryCondition="false" constant="false"
fbc:chemicalFormula=""/>
      <species metaid="M_isot24u_c" id="M_isot24u_c"
name="Isotetradecanoyllipoteichoic_acid_n=24__linked__unsubstituted"
compartment="c" charge="-24" initialConcentration="0"
hasOnlySubstanceUnits="false" boundaryCondition="false" constant="false"
fbc:chemicalFormula=""/>
      <species metaid="M_isotcard_c" id="M_isotcard_c"
name="Isotetradecanoylcardiolipin_B._subtilis" compartment="c" charge="-2"
initialConcentration="0" hasOnlySubstanceUnits="false"
boundaryCondition="false" constant="false" fbc:chemicalFormula=""/>
      <species metaid="M_isovACP_c" id="M_isovACP_c" name="isovaleryl__ACP"
compartment="c" charge="-1" initialConcentration="0"
hasOnlySubstanceUnits="false" boundaryCondition="false" constant="false"
fbc:chemicalFormula="C16H29N2O8PRS"/>
      <species metaid="M_itp_c" id="M_itp_c" name="ITP" compartment="c"
charge="-4" initialConcentration="0" hasOnlySubstanceUnits="false"
boundaryCondition="false" constant="false"
fbc:chemicalFormula="C10H11N4O14P3"/>
      <species metaid="M_ivcoa_c" id="M_ivcoa_c" name="Isovaleryl__CoA"
compartment="c" charge="-4" initialConcentration="0"
hasOnlySubstanceUnits="false" boundaryCondition="false" constant="false"
fbc:chemicalFormula="C26H40N7O17P3S"/>
      <species metaid="M_k_c" id="M_k_c" name="potassium" compartment="c"
charge="1" initialConcentration="0" hasOnlySubstanceUnits="false"
boundaryCondition="false" constant="false" fbc:chemicalFormula="K"/>
      <species metaid="M_k_e" id="M_k_e" name="potassium_b" compartment="e"
charge="1" initialConcentration="0" hasOnlySubstanceUnits="false"
boundaryCondition="true" constant="false" fbc:chemicalFormula="K"/>
```

<species metaid="M_l23uo" id="M_l23uo" name="L__xylo__hex__3__ulonolactone" compartment="c" charge="-1" initialConcentration="0" hasOnlySubstanceUnits="false" boundaryCondition="false" constant="false" fbc:chemicalFormula="C6H7O6"/>
<species metaid="M_lac__D_c" id="M_lac__D_c" name="D__Lactate" compartment="c" charge="-1" initialConcentration="0" hasOnlySubstanceUnits="false" boundaryCondition="false" constant="false" fbc:chemicalFormula="C3H5O3"/>
<species metaid="M_lac__L_c" id="M_lac__L_c" name="L__Lactate" compartment="c" charge="-1" initialConcentration="0" hasOnlySubstanceUnits="false" boundaryCondition="false" constant="false" fbc:chemicalFormula="C3H5O3"/>
<species metaid="M_lac__L_e" id="M_lac__L_e" name="L__Lactate_b" compartment="e" charge="-1" initialConcentration="0" hasOnlySubstanceUnits="false" boundaryCondition="true" constant="false" fbc:chemicalFormula="C3H5O3"/>
<species metaid="M_lac6p_c" id="M_lac6p_c" name="Lactose__6__phosphate" compartment="c" charge="-2" initialConcentration="0" hasOnlySubstanceUnits="false" boundaryCondition="false" constant="false" fbc:chemicalFormula="C12H21O14P"/>
<species metaid="M_lald__L_c" id="M_lald__L_c" name="L__Lactaldehyde" compartment="c" charge="0" initialConcentration="0" hasOnlySubstanceUnits="false" boundaryCondition="false" constant="false" fbc:chemicalFormula="C3H6O2"/>
<species metaid="M_Largn_c" id="M_Largn_c" name="L__Arogenate" compartment="c" charge="-1" initialConcentration="0" hasOnlySubstanceUnits="false" boundaryCondition="false" constant="false" fbc:chemicalFormula="C10H13NO5"/>
<species metaid="M_lcts_e" id="M_lcts_e" name="Lactose_b" compartment="e" charge="0" initialConcentration="0" hasOnlySubstanceUnits="false" boundaryCondition="true" constant="false" fbc:chemicalFormula="C12H22O11"/>
<species metaid="M_leu__L_c" id="M_leu__L_c" name="L__Leucine" compartment="c" charge="0" initialConcentration="0" hasOnlySubstanceUnits="false" boundaryCondition="false" constant="false" fbc:chemicalFormula="C6H13NO2"/>
<species metaid="M_leu__L_e" id="M_leu__L_e" name="L__Leucine_b" compartment="e" charge="0" initialConcentration="0" hasOnlySubstanceUnits="false" boundaryCondition="true" constant="false" fbc:chemicalFormula="C6H13NO2"/>
<species metaid="M_lgt__S_c" id="M_lgt__S_c" name="(R)__S__Lactoylglutathione" compartment="c" charge="-1" initialConcentration="0" hasOnlySubstanceUnits="false" boundaryCondition="false" constant="false" fbc:chemicalFormula="C13H20N3O8S"/>
<species metaid="M_lip2_c" id="M_lip2_c" name="ditrans,octacis__undecaprenyldiphospho__N__acetyl__(N__acetyl__beta__D__glucosaminyl)muramoyl__L__alanyl__gamma__D__glutamyl__L__lysyl__D__alanyl__D__alanine" compartment="c" charge="-4" initialConcentration="0" hasOnlySubstanceUnits="false" boundaryCondition="false" constant="false" fbc:chemicalFormula="C94H153N8O26P2"/>
<species metaid="M_lip2g_c" id="M_lip2g_c" name="ditrans,octacis__undecaprenyldiphospho__N__acetyl__(N__acetylglucosaminyl)muramoyl__L__alanyl__gamma__D__isoglutaminyl__L__lysyl__(N6__glycyl)__D__alanyl__D__alanine" compartment="c" charge="-2" initialConcentration="0" hasOnlySubstanceUnits="false" boundaryCondition="false" constant="false" fbc:chemicalFormula="C96H158N9O27P2"/>

<species metaid="M_lip2g3_c" id="M_lip2g3_c" name="ditrans,octacis__undecaprenyldiphospho__N__acetyl__(N__acetylglucosaminyl)muramoyl__L__alanyl__gamma__D__isoglutaminyl__L__lysyl__(glycyl)3__D__alanyl__D__alanine" compartment="c" charge="-2" initialConcentration="0" hasOnlySubstanceUnits="false" boundaryCondition="false" constant="false" fbc:chemicalFormula="C100H164N11O29P2"/>
<species metaid="M_lipoamp_c" id="M_lipoamp_c" name="lipoyl__AMP" compartment="c" charge="-1" initialConcentration="0" hasOnlySubstanceUnits="false" boundaryCondition="false" constant="false" fbc:chemicalFormula="C18H25N5O8PS2"/>
<species metaid="M_lipoate_c" id="M_lipoate_c" name="Lipoate" compartment="c" charge="-1" initialConcentration="0" hasOnlySubstanceUnits="false" boundaryCondition="false" constant="false" fbc:chemicalFormula="C8H14O2S2"/>
<species metaid="M_lipoate_e" id="M_lipoate_e" name="Lipoate_b" compartment="e" charge="-1" initialConcentration="0" hasOnlySubstanceUnits="false" boundaryCondition="true" constant="false" fbc:chemicalFormula="C8H14O2S2"/>
<species metaid="M_lipopb_c" id="M_lipopb_c" name="Lipoate (protein bound)" compartment="c" charge="0" initialConcentration="0" hasOnlySubstanceUnits="false" boundaryCondition="false" constant="false" fbc:chemicalFormula=""/>
<species metaid="M_lpam_c" id="M_lpam_c" name="Lipoamide" compartment="c" charge="0" initialConcentration="0" hasOnlySubstanceUnits="false" boundaryCondition="false" constant="false" fbc:chemicalFormula="C8H15NOS2"/>
<species metaid="M_lpro_c" id="M_lpro_c" name="Lipoylprotein" compartment="c" charge="0" initialConcentration="0" hasOnlySubstanceUnits="false" boundaryCondition="false" constant="false" fbc:chemicalFormula="S2X"/>
<species metaid="M_LTA_c" id="M_LTA_c" name="Lipoteichoic acid " compartment="c" charge="-106" initialConcentration="0" hasOnlySubstanceUnits="false" boundaryCondition="false" constant="false" fbc:chemicalFormula="C386H754N8O555P120"/>
<species metaid="M_lys__L_c" id="M_lys__L_c" name="L__Lysine" compartment="c" charge="1" initialConcentration="0" hasOnlySubstanceUnits="false" boundaryCondition="false" constant="false" fbc:chemicalFormula="C6H15N2O2"/>
<species metaid="M_lys__L_e" id="M_lys__L_e" name="L__Lysine_b" compartment="e" charge="1" initialConcentration="0" hasOnlySubstanceUnits="false" boundaryCondition="true" constant="false" fbc:chemicalFormula="C6H15N2O2"/>
<species metaid="M_lystrna_c" id="M_lystrna_c" name="L__Lysine__tRNA (Lys)" compartment="c" charge="2" initialConcentration="0" hasOnlySubstanceUnits="false" boundaryCondition="false" constant="false" fbc:chemicalFormula="C6H14N2OR"/>
<species metaid="M_lyx__L_e" id="M_lyx__L_e" name="L__Lyxose_b" compartment="e" charge="0" initialConcentration="0" hasOnlySubstanceUnits="false" boundaryCondition="true" constant="false" fbc:chemicalFormula="C5H10O5"/>
<species metaid="M_m12daglyc_c" id="M_m12daglyc_c" name="Monoglucosyl__1_2_distearoylglycerol" compartment="c" charge="0" initialConcentration="0" hasOnlySubstanceUnits="false" boundaryCondition="false" constant="false" fbc:chemicalFormula=""/>
<species metaid="M_m12dglyc_c" id="M_m12dglyc_c" name="Monoglucosyl__1_2_dimyristoylglycerol" compartment="c" charge="0" initialConcentration="0" hasOnlySubstanceUnits="false" boundaryCondition="false" constant="false" fbc:chemicalFormula=""/>

<species metaid="M_m12dhdeglyc_c" id="M_m12dhdeglyc_c" name="Monoglucosyl__1_2_dianteisoheptadecanoylglycerol" compartment="c" charge="0" initialConcentration="0" hasOnlySubstanceUnits="false" boundaryCondition="false" constant="false" fbc:chemicalFormula=""/>
<species metaid="M_m12dhdglyc_c" id="M_m12dhdglyc_c" name="Monoglucosyl__1_2_diisoheptadecanoylglycerol" compartment="c" charge="0" initialConcentration="0" hasOnlySubstanceUnits="false" boundaryCondition="false" constant="false" fbc:chemicalFormula=""/>
<species metaid="M_m12diagly_c" id="M_m12diagly_c" name="Monoglucosyl__1_2_dianteisopentadecanoylglycerol" compartment="c" charge="0" initialConcentration="0" hasOnlySubstanceUnits="false" boundaryCondition="false" constant="false" fbc:chemicalFormula=""/>
<species metaid="M_m12diidgly_c" id="M_m12diidgly_c" name="Monoglucosyl__1_2_diisotetradecanoylglycerol" compartment="c" charge="0" initialConcentration="0" hasOnlySubstanceUnits="false" boundaryCondition="false" constant="false" fbc:chemicalFormula=""/>
<species metaid="M_m12diihgly_c" id="M_m12diihgly_c" name="Monoglucosyl__1_2_diisohexadecanoylglycerol" compartment="c" charge="0" initialConcentration="0" hasOnlySubstanceUnits="false" boundaryCondition="false" constant="false" fbc:chemicalFormula=""/>
<species metaid="M_m12dipgly_c" id="M_m12dipgly_c" name="Monoglucosyl__1_2_diisopentadecanoylglycerol" compartment="c" charge="0" initialConcentration="0" hasOnlySubstanceUnits="false" boundaryCondition="false" constant="false" fbc:chemicalFormula=""/>
<species metaid="M_mal__D_c" id="M_mal__D_c" name="D__Malate" compartment="c" charge="-2" initialConcentration="0" hasOnlySubstanceUnits="false" boundaryCondition="false" constant="false" fbc:chemicalFormula="C4H4O5"/>
<species metaid="M_mal__D_e" id="M_mal__D_e" name="D__Malate_b" compartment="e" charge="-2" initialConcentration="0" hasOnlySubstanceUnits="false" boundaryCondition="true" constant="false" fbc:chemicalFormula="C4H4O5"/>
<species metaid="M_mal__L_c" id="M_mal__L_c" name="L__Malate" compartment="c" charge="-2" initialConcentration="0" hasOnlySubstanceUnits="false" boundaryCondition="false" constant="false" fbc:chemicalFormula="C4H4O5"/>
<species metaid="M_mal__L_e" id="M_mal__L_e" name="L__Malate_b" compartment="e" charge="-2" initialConcentration="0" hasOnlySubstanceUnits="false" boundaryCondition="true" constant="false" fbc:chemicalFormula="C4H4O5"/>
<species metaid="M_malACP_c" id="M_malACP_c" name="Malonyl__[acyl__carrier protein]" compartment="c" charge="0" initialConcentration="0" hasOnlySubstanceUnits="false" boundaryCondition="false" constant="false" fbc:chemicalFormula="C14H22N2O10PRS"/>
<species metaid="M_malcoa_c" id="M_malcoa_c" name="Malonyl__CoA" compartment="c" charge="-5" initialConcentration="0" hasOnlySubstanceUnits="false" boundaryCondition="false" constant="false" fbc:chemicalFormula="C24H33N7O19P3S"/>
<species metaid="M_male_c" id="M_male_c" name="Maleate" compartment="c" charge="-2" initialConcentration="0" hasOnlySubstanceUnits="false" boundaryCondition="false" constant="false" fbc:chemicalFormula="C4H2O4"/>
<species metaid="M_malm_c" id="M_malm_c" name="Maleamate" compartment="c" charge="-1" initialConcentration="0" hasOnlySubstanceUnits="false" boundaryCondition="false" constant="false" fbc:chemicalFormula="C4H4NO3"/>
<species metaid="M_malmeACP_c" id="M_malmeACP_c" name="Malonyl__ACP methyl ester" compartment="c" charge="1" initialConcentration="0"

```
hasOnlySubstanceUnits="false" boundaryCondition="false" constant="false"
fbc:chemicalFormula="C15H25N2O10PRS"/>
      <species metaid="M_malt_c" id="M_malt_c" name="Maltose" compartment="c"
charge="0" initialConcentration="0" hasOnlySubstanceUnits="false"
boundaryCondition="false" constant="false" fbc:chemicalFormula="C12H22O11"/>
      <species metaid="M_malt_e" id="M_malt_e" name="Maltose_b"
compartment="e" charge="0" initialConcentration="0"
hasOnlySubstanceUnits="false" boundaryCondition="true" constant="false"
fbc:chemicalFormula="C12H22O11"/>
      <species metaid="M_malt6p_c" id="M_malt6p_c" name="Maltose
6'__phosphate" compartment="c" charge="-2" initialConcentration="0"
hasOnlySubstanceUnits="false" boundaryCondition="false" constant="false"
fbc:chemicalFormula="C12H21O14P"/>
      <species metaid="M_malthx_c" id="M_malthx_c" name="Maltohexaose"
compartment="c" charge="0" initialConcentration="0"
hasOnlySubstanceUnits="false" boundaryCondition="false" constant="false"
fbc:chemicalFormula="C36H62O31"/>
      <species metaid="M_malthx_e" id="M_malthx_e" name="Maltohexaose_b"
compartment="e" charge="0" initialConcentration="0"
hasOnlySubstanceUnits="false" boundaryCondition="true" constant="false"
fbc:chemicalFormula="C36H62O31"/>
      <species metaid="M_malttr_c" id="M_malttr_c" name="Maltotriose"
compartment="c" charge="0" initialConcentration="0"
hasOnlySubstanceUnits="false" boundaryCondition="false" constant="false"
fbc:chemicalFormula="C18H32O16"/>
      <species metaid="M_malttr_e" id="M_malttr_e" name="Maltotriose_b"
compartment="e" charge="0" initialConcentration="0"
hasOnlySubstanceUnits="false" boundaryCondition="true" constant="false"
fbc:chemicalFormula="C18H32O16"/>
      <species metaid="M_man_e" id="M_man_e" name="D__Mannose_b"
compartment="e" charge="0" initialConcentration="0"
hasOnlySubstanceUnits="false" boundaryCondition="true" constant="false"
fbc:chemicalFormula="C6H12O6"/>
      <species metaid="M_man1p_c" id="M_man1p_c" name="D__Mannose
1__phosphate" compartment="c" charge="-2" initialConcentration="0"
hasOnlySubstanceUnits="false" boundaryCondition="false" constant="false"
fbc:chemicalFormula="C6H11O9P"/>
      <species metaid="M_man6p_c" id="M_man6p_c" name="D__Mannose
6__phosphate" compartment="c" charge="-2" initialConcentration="0"
hasOnlySubstanceUnits="false" boundaryCondition="false" constant="false"
fbc:chemicalFormula="C6H11O9P"/>
      <species metaid="M_man6p_e" id="M_man6p_e" name="D__Mannose
6__phosphate_b" compartment="e" charge="-2" initialConcentration="0"
hasOnlySubstanceUnits="false" boundaryCondition="true" constant="false"
fbc:chemicalFormula="C6H11O9P"/>
      <species metaid="M_man6p_p" id="M_man6p_p" name="D__Mannose
6__phosphate" compartment="p" charge="-2" initialConcentration="0"
hasOnlySubstanceUnits="false" boundaryCondition="false" constant="false"
fbc:chemicalFormula="C6H11O9P"/>
      <species metaid="M_mana_c" id="M_mana_c" name="D__Mannonate"
compartment="c" charge="-1" initialConcentration="0"
hasOnlySubstanceUnits="false" boundaryCondition="false" constant="false"
fbc:chemicalFormula="C6H11O7"/>
      <species metaid="M_manl1p_c" id="M_manl1p_c" name="D mannose 1-
phosphate" compartment="c" charge="0" initialConcentration="0"
hasOnlySubstanceUnits="false" boundaryCondition="false" constant="false"
fbc:chemicalFormula="C6H13O9P"/>
```

<species metaid="M_manl1p_e" id="M_manl1p_e" name="D mannose 1-phosphate_b" compartment="e" charge="0" initialConcentration="0" hasOnlySubstanceUnits="false" boundaryCondition="true" constant="false" fbc:chemicalFormula="C6H13O9P"/>
<species metaid="M_mcres_c" id="M_mcres_c" name="m__Cresotic_acid" compartment="c" charge="-1" initialConcentration="0" hasOnlySubstanceUnits="false" boundaryCondition="false" constant="false" fbc:chemicalFormula=""/>
<species metaid="M_melib_c" id="M_melib_c" name="Melibiose" compartment="c" charge="0" initialConcentration="0" hasOnlySubstanceUnits="false" boundaryCondition="false" constant="false" fbc:chemicalFormula="C12H22O11"/>
<species metaid="M_melib_e" id="M_melib_e" name="Melibiose_b" compartment="e" charge="0" initialConcentration="0" hasOnlySubstanceUnits="false" boundaryCondition="true" constant="false" fbc:chemicalFormula="C12H22O11"/>
<species metaid="M_meoh_c" id="M_meoh_c" name="Methanol" compartment="c" charge="0" initialConcentration="0" hasOnlySubstanceUnits="false" boundaryCondition="false" constant="false" fbc:chemicalFormula="CH4O1"/>
<species metaid="M_met__D_c" id="M_met__D_c" name="D__Methionine" compartment="c" charge="0" initialConcentration="0" hasOnlySubstanceUnits="false" boundaryCondition="false" constant="false" fbc:chemicalFormula="C5H10NO2S"/>
<species metaid="M_met__D_e" id="M_met__D_e" name="D__Methionine_b" compartment="e" charge="0" initialConcentration="0" hasOnlySubstanceUnits="false" boundaryCondition="true" constant="false" fbc:chemicalFormula="C5H10NO2S"/>
<species metaid="M_met__L_ala__L_c" id="M_met__L_ala__L_c" name="met__L__ala__L" compartment="c" charge="-1" initialConcentration="0" hasOnlySubstanceUnits="false" boundaryCondition="false" constant="false" fbc:chemicalFormula="C8H15N2O3S"/>
<species metaid="M_met__L_ala__L_e" id="M_met__L_ala__L_e" name="met__L__ala__L_b" compartment="e" charge="-1" initialConcentration="0" hasOnlySubstanceUnits="false" boundaryCondition="true" constant="false" fbc:chemicalFormula="C8H15N2O3S"/>
<species metaid="M_met__L_c" id="M_met__L_c" name="L__Methionine" compartment="c" charge="0" initialConcentration="0" hasOnlySubstanceUnits="false" boundaryCondition="false" constant="false" fbc:chemicalFormula="C5H11NO2S"/>
<species metaid="M_met__L_e" id="M_met__L_e" name="L__Methionine_b" compartment="e" charge="0" initialConcentration="0" hasOnlySubstanceUnits="false" boundaryCondition="true" constant="false" fbc:chemicalFormula="C5H11NO2S"/>
<species metaid="M_methf_c" id="M_methf_c" name="5,10__Methenyltetrahydrofolate" compartment="c" charge="-1" initialConcentration="0" hasOnlySubstanceUnits="false" boundaryCondition="false" constant="false" fbc:chemicalFormula="C20H20N7O6"/>
<species metaid="M_metsox__R__L_c" id="M_metsox__R__L_c" name="L__methionine__R__sulfoxide" compartment="c" charge="0" initialConcentration="0" hasOnlySubstanceUnits="false" boundaryCondition="false" constant="false" fbc:chemicalFormula="C5H11NO3S"/>
<species metaid="M_metsox__R__L_e" id="M_metsox__R__L_e" name="L__methionine__R__sulfoxide_b" compartment="e" charge="0" initialConcentration="0" hasOnlySubstanceUnits="false" boundaryCondition="true" constant="false" fbc:chemicalFormula="C5H11NO3S"/>
<species metaid="M_metsox__S__L_c" id="M_metsox__S__L_c" name="L__Methionine Sulfoxide" compartment="c" charge="0"

initialConcentration="0" hasOnlySubstanceUnits="false"
boundaryCondition="false" constant="false" fbc:chemicalFormula="C5H11NO3S"/>
<species metaid="M_metsox__S__L_e" id="M_metsox__S__L_e"
name="L__Methionine Sulfoxide_b" compartment="e" charge="0"
initialConcentration="0" hasOnlySubstanceUnits="false"
boundaryCondition="true" constant="false" fbc:chemicalFormula="C5H11NO3S"/>
<species metaid="M_mettrna_c" id="M_mettrna_c" name="L__Methionyl__tRNA
(Met)" compartment="c" charge="1" initialConcentration="0"
hasOnlySubstanceUnits="false" boundaryCondition="false" constant="false"
fbc:chemicalFormula="C5H10NOSR"/>
<species metaid="M_mev__R_c" id="M_mev__R_c" name="(R)__Mevalonate"
compartment="c" charge="-1" initialConcentration="0"
hasOnlySubstanceUnits="false" boundaryCondition="false" constant="false"
fbc:chemicalFormula="C6H11O4"/>
<species metaid="M_mg2_c" id="M_mg2_c" name="magnesium" compartment="c"
charge="2" initialConcentration="0" hasOnlySubstanceUnits="false"
boundaryCondition="false" constant="false" fbc:chemicalFormula="Mg"/>
<species metaid="M_mg2_e" id="M_mg2_e" name="magnesium_b"
compartment="e" charge="2" initialConcentration="0"
hasOnlySubstanceUnits="false" boundaryCondition="true" constant="false"
fbc:chemicalFormula="Mg"/>
<species metaid="M_mg12dpgly_c" id="M_mg12dpgly_c"
name="Monoglucosyl__1_2_dipalmitoylglycerol" compartment="c" charge="0"
initialConcentration="0" hasOnlySubstanceUnits="false"
boundaryCondition="false" constant="false" fbc:chemicalFormula=""/>
<species metaid="M_mhpglu_c" id="M_mhpglu_c"
name="5__Methyltetrahydropteroyltri__L__glutamate" compartment="c" charge="-
4" initialConcentration="0" hasOnlySubstanceUnits="false"
boundaryCondition="false" constant="false"
fbc:chemicalFormula="C25H36N8O12"/>
<species metaid="M_mi1p__D_c" id="M_mi1p__D_c" name="1D__myo__Inositol
1__phosphate" compartment="c" charge="-1" initialConcentration="0"
hasOnlySubstanceUnits="false" boundaryCondition="false" constant="false"
fbc:chemicalFormula="C6H11O9P"/>
<species metaid="M_mi3p__D_c" id="M_mi3p__D_c" name="1D__myo__Inositol
3__phosphate" compartment="c" charge="-1" initialConcentration="0"
hasOnlySubstanceUnits="false" boundaryCondition="false" constant="false"
fbc:chemicalFormula="C6H11O9P"/>
<species metaid="M_mi4p__D_c" id="M_mi4p__D_c" name="1D__myo__Inositol
4__phosphate" compartment="c" charge="-1" initialConcentration="0"
hasOnlySubstanceUnits="false" boundaryCondition="false" constant="false"
fbc:chemicalFormula="C6H11O9P"/>
<species metaid="M_mlthf_c" id="M_mlthf_c"
name="5,10__Methylenetetrahydrofolate" compartment="c" charge="-2"
initialConcentration="0" hasOnlySubstanceUnits="false"
boundaryCondition="false" constant="false" fbc:chemicalFormula="C20H21N7O6"/>
<species metaid="M_mmcoa__S_c" id="M_mmcoa__S_c"
name="(S)__Methylmalonyl__CoA" compartment="c" charge="-4"
initialConcentration="0" hasOnlySubstanceUnits="false"
boundaryCondition="false" constant="false"
fbc:chemicalFormula="C25H35N7O19P3S"/>
<species metaid="M_mn2_c" id="M_mn2_c" name="Mn2+" compartment="c"
charge="2" initialConcentration="0" hasOnlySubstanceUnits="false"
boundaryCondition="false" constant="false" fbc:chemicalFormula="Mn"/>
<species metaid="M_mn2_e" id="M_mn2_e" name="Mn2+_b" compartment="e"
charge="2" initialConcentration="0" hasOnlySubstanceUnits="false"
boundaryCondition="true" constant="false" fbc:chemicalFormula="Mn"/>

```
      <species metaid="M_mnl_e" id="M_mnl_e" name="D__Mannitol_b"
compartment="e" charge="0" initialConcentration="0"
hasOnlySubstanceUnits="false" boundaryCondition="true" constant="false"
fbc:chemicalFormula="C6H14O6"/>
      <species metaid="M_mnl1p_c" id="M_mnl1p_c" name="D__Mannitol
1__phosphate" compartment="c" charge="-2" initialConcentration="0"
hasOnlySubstanceUnits="false" boundaryCondition="false" constant="false"
fbc:chemicalFormula="C6H13O9P"/>
      <species metaid="M_mobd_c" id="M_mobd_c" name="Molybdate"
compartment="c" charge="-2" initialConcentration="0"
hasOnlySubstanceUnits="false" boundaryCondition="false" constant="false"
fbc:chemicalFormula="MoO4"/>
      <species metaid="M_mobd_e" id="M_mobd_e" name="Molybdate_b"
compartment="e" charge="-2" initialConcentration="0"
hasOnlySubstanceUnits="false" boundaryCondition="true" constant="false"
fbc:chemicalFormula="MoO4"/>
      <species metaid="M_mql8_c" id="M_mql8_c" name="Menaquinol 8"
compartment="c" charge="0" initialConcentration="0"
hasOnlySubstanceUnits="false" boundaryCondition="false" constant="false"
fbc:chemicalFormula="C51H74O2"/>
      <species metaid="M_mqn8_c" id="M_mqn8_c" name="Menaquinone 8"
compartment="c" charge="0" initialConcentration="0"
hasOnlySubstanceUnits="false" boundaryCondition="false" constant="false"
fbc:chemicalFormula="C51H72O2"/>
      <species metaid="M_msa_c" id="M_msa_c" name="Malonate semialdehyde"
compartment="c" charge="-1" initialConcentration="0"
hasOnlySubstanceUnits="false" boundaryCondition="false" constant="false"
fbc:chemicalFormula="C3H3O3"/>
      <species metaid="M_msial_c" id="M_msial_c" name="3__Methylsalicylate"
compartment="c" charge="-1" initialConcentration="0"
hasOnlySubstanceUnits="false" boundaryCondition="false" constant="false"
fbc:chemicalFormula="C8H7O3"/>
      <species metaid="M_mso3_e" id="M_mso3_e" name="methanesulfonate_b"
compartment="e" charge="-1" initialConcentration="0"
hasOnlySubstanceUnits="false" boundaryCondition="true" constant="false"
fbc:chemicalFormula="CH3O3S"/>
      <species metaid="M_mthgxl_c" id="M_mthgxl_c" name="Methylglyoxal"
compartment="c" charge="0" initialConcentration="0"
hasOnlySubstanceUnits="false" boundaryCondition="false" constant="false"
fbc:chemicalFormula="C3H4O2"/>
      <species metaid="M_mttdca_c" id="M_mttdca_c"
name="12__methyltetradecanoate" compartment="c" charge="0"
initialConcentration="0" hasOnlySubstanceUnits="false"
boundaryCondition="false" constant="false" fbc:chemicalFormula="C15H29O2"/>
      <species metaid="M_my424ds_c" id="M_my424ds_c"
name="Myristoyllipoteichoic_acid_n=24__linked__D__alanine_substituted"
compartment="c" charge="-26" initialConcentration="0"
hasOnlySubstanceUnits="false" boundaryCondition="false" constant="false"
fbc:chemicalFormula=""/>
      <species metaid="M_mycard_c" id="M_mycard_c"
name="Myristoylcardiolipin_B._subtilis" compartment="c" charge="-2"
initialConcentration="0" hasOnlySubstanceUnits="false"
boundaryCondition="false" constant="false" fbc:chemicalFormula=""/>
      <species metaid="M_myrs24s_c" id="M_myrs24s_c"
name="Myristoyllipoteichoic_acid_n=24__linked__N__acetyl__D__glucosamine"
compartment="c" charge="-24" initialConcentration="0"
hasOnlySubstanceUnits="false" boundaryCondition="false" constant="false"
fbc:chemicalFormula=""/>
```

<species metaid="M_myrs24u_c" id="M_myrs24u_c" name="Myristoyllipoteichoic_acid_n=24__linked__unsubstituted" compartment="c" charge="-24" initialConcentration="0" hasOnlySubstanceUnits="false" boundaryCondition="false" constant="false" fbc:chemicalFormula=""/>
<species metaid="M_myrsACP_c" id="M_myrsACP_c" name="Myristoyl__ACP (n__C14:0ACP)" compartment="c" charge="-1" initialConcentration="0" hasOnlySubstanceUnits="false" boundaryCondition="false" constant="false" fbc:chemicalFormula="C25H47N2O8PRS"/>
<species metaid="M_myrst24s_c" id="M_myrst24s_c" name="Myristoyllipoteichoic_acid_n=24__linked__glucose_substituted" compartment="c" charge="-24" initialConcentration="0" hasOnlySubstanceUnits="false" boundaryCondition="false" constant="false" fbc:chemicalFormula=""/>
<species metaid="M_N1aspmd_c" id="M_N1aspmd_c" name="N1__Acetylspermidine" compartment="c" charge="2" initialConcentration="0" hasOnlySubstanceUnits="false" boundaryCondition="false" constant="false" fbc:chemicalFormula="C9H23N3O"/>
<species metaid="M_n4abutn_c" id="M_n4abutn_c" name="N4__Acetylaminobutanal" compartment="c" charge="0" initialConcentration="0" hasOnlySubstanceUnits="false" boundaryCondition="false" constant="false" fbc:chemicalFormula="C6H11NO2"/>
<species metaid="M_n6all26d_c" id="M_n6all26d_c" name="N6__Acetyl__LL__2,6__diaminoheptanedioate" compartment="c" charge="-1" initialConcentration="0" hasOnlySubstanceUnits="false" boundaryCondition="false" constant="false" fbc:chemicalFormula="C9H15N2O5"/>
<species metaid="M_n8aspmd_c" id="M_n8aspmd_c" name="N8__Acetylspermidine" compartment="c" charge="2" initialConcentration="0" hasOnlySubstanceUnits="false" boundaryCondition="false" constant="false" fbc:chemicalFormula="C9H23N3O"/>
<species metaid="M_na1_c" id="M_na1_c" name="Sodium" compartment="c" charge="1" initialConcentration="0" hasOnlySubstanceUnits="false" boundaryCondition="false" constant="false" fbc:chemicalFormula="Na"/>
<species metaid="M_na1_e" id="M_na1_e" name="Sodium_b" compartment="e" charge="1" initialConcentration="0" hasOnlySubstanceUnits="false" boundaryCondition="true" constant="false" fbc:chemicalFormula="Na"/>
<species metaid="M_nac_c" id="M_nac_c" name="Nicotinate" compartment="c" charge="-1" initialConcentration="0" hasOnlySubstanceUnits="false" boundaryCondition="false" constant="false" fbc:chemicalFormula="C6H4NO2"/>
<species metaid="M_nac_e" id="M_nac_e" name="Nicotinate_b" compartment="e" charge="-1" initialConcentration="0" hasOnlySubstanceUnits="false" boundaryCondition="true" constant="false" fbc:chemicalFormula="C6H4NO2"/>
<species metaid="M_nacetlbdgl_c" id="M_nacetlbdgl_c" name="N__Acetyl__beta__D__mannosaminyl__1_4__N__acetyl__D___glucosaminyldiphosphoundecaprenol" compartment="c" charge="-2" initialConcentration="0" hasOnlySubstanceUnits="false" boundaryCondition="false" constant="false" fbc:chemicalFormula=""/>
<species metaid="M_nacetlbdglg_c" id="M_nacetlbdglg_c" name="Gro__P__ManNAc__GlcNAc__PP__undecaprenol" compartment="c" charge="-2" initialConcentration="0" hasOnlySubstanceUnits="false" boundaryCondition="false" constant="false" fbc:chemicalFormula="C3H6O5P"/>
<species metaid="M_nacetlbdglg2_c" id="M_nacetlbdglg2_c" name="Gro__P__Gro__P__ManNAc__GlcNAc__PP__undecaprenol" compartment="c" charge="-2" initialConcentration="0" hasOnlySubstanceUnits="false" boundaryCondition="false" constant="false" fbc:chemicalFormula="C6H12O10P2"/>
<species metaid="M_nad_c" id="M_nad_c" name="Nicotinamide adenine dinucleotide" compartment="c" charge="-1" initialConcentration="0"

```
hasOnlySubstanceUnits="false" boundaryCondition="false" constant="false"
fbc:chemicalFormula="C21H26N7O14P2"/>
      <species metaid="M_nadh_c" id="M_nadh_c" name="Nicotinamide adenine
dinucleotide __ reduced" compartment="c" charge="-2" initialConcentration="0"
hasOnlySubstanceUnits="false" boundaryCondition="false" constant="false"
fbc:chemicalFormula="C21H27N7O14P2"/>
      <species metaid="M_nadp_c" id="M_nadp_c" name="Nicotinamide adenine
dinucleotide phosphate" compartment="c" charge="-3" initialConcentration="0"
hasOnlySubstanceUnits="false" boundaryCondition="false" constant="false"
fbc:chemicalFormula="C21H25N7O17P3"/>
      <species metaid="M_nadph_c" id="M_nadph_c" name="Nicotinamide adenine
dinucleotide phosphate __ reduced" compartment="c" charge="-4"
initialConcentration="0" hasOnlySubstanceUnits="false"
boundaryCondition="false" constant="false"
fbc:chemicalFormula="C21H26N7O17P3"/>
      <species metaid="M_nal2a6o_c" id="M_nal2a6o_c"
name="N__Acetyl__L__2__amino__6__oxopimelate" compartment="c" charge="-2"
initialConcentration="0" hasOnlySubstanceUnits="false"
boundaryCondition="false" constant="false" fbc:chemicalFormula="C9H11NO6"/>
      <species metaid="M_ncam_c" id="M_ncam_c" name="Nicotinamide"
compartment="c" charge="0" initialConcentration="0"
hasOnlySubstanceUnits="false" boundaryCondition="false" constant="false"
fbc:chemicalFormula="C6H6N2O"/>
      <species metaid="M_ncam_e" id="M_ncam_e" name="Nicotinamide_b"
compartment="e" charge="0" initialConcentration="0"
hasOnlySubstanceUnits="false" boundaryCondition="true" constant="false"
fbc:chemicalFormula="C6H6N2O"/>
      <species metaid="M_nh4_c" id="M_nh4_c" name="Ammonium" compartment="c"
charge="1" initialConcentration="0" hasOnlySubstanceUnits="false"
boundaryCondition="false" constant="false" fbc:chemicalFormula="H4N"/>
      <species metaid="M_nh4_e" id="M_nh4_e" name="Ammonium_b"
compartment="e" charge="1" initialConcentration="0"
hasOnlySubstanceUnits="false" boundaryCondition="true" constant="false"
fbc:chemicalFormula="H4N"/>
      <species metaid="M_nh4oh_c" id="M_nh4oh_c" name="Ammonium hydroxide"
compartment="c" charge="0" initialConcentration="0"
hasOnlySubstanceUnits="false" boundaryCondition="false" constant="false"
fbc:chemicalFormula="H5NO"/>
      <species metaid="M_ni2_c" id="M_ni2_c" name="nickel" compartment="c"
charge="2" initialConcentration="0" hasOnlySubstanceUnits="false"
boundaryCondition="false" constant="false" fbc:chemicalFormula="Ni"/>
      <species metaid="M_ni2_e" id="M_ni2_e" name="nickel_b" compartment="e"
charge="2" initialConcentration="0" hasOnlySubstanceUnits="false"
boundaryCondition="true" constant="false" fbc:chemicalFormula="Ni"/>
      <species metaid="M_nicrns_c" id="M_nicrns_c" name="Nicotinate
D__ribonucleoside" compartment="c" charge="0" initialConcentration="0"
hasOnlySubstanceUnits="false" boundaryCondition="false" constant="false"
fbc:chemicalFormula="C11H13NO6"/>
      <species metaid="M_nicrnt_c" id="M_nicrnt_c" name="Nicotinate
D__ribonucleotide" compartment="c" charge="-2" initialConcentration="0"
hasOnlySubstanceUnits="false" boundaryCondition="false" constant="false"
fbc:chemicalFormula="C11H12NO9P"/>
      <species metaid="M_nmn_c" id="M_nmn_c" name="NMN" compartment="c"
charge="-1" initialConcentration="0" hasOnlySubstanceUnits="false"
boundaryCondition="false" constant="false"
fbc:chemicalFormula="C11H14N2O8P"/>
```

<species metaid="M_nmn_e" id="M_nmn_e" name="NMN_b" compartment="e"
charge="-1" initialConcentration="0" hasOnlySubstanceUnits="false"
boundaryCondition="true" constant="false" fbc:chemicalFormula="C11H14N2O8P"/>
<species metaid="M_no_c" id="M_no_c" name="Nitric oxide"
compartment="c" charge="0" initialConcentration="0"
hasOnlySubstanceUnits="false" boundaryCondition="false" constant="false"
fbc:chemicalFormula="NO"/>
<species metaid="M_no2_c" id="M_no2_c" name="Nitrite" compartment="c"
charge="0" initialConcentration="0" hasOnlySubstanceUnits="false"
boundaryCondition="false" constant="false" fbc:chemicalFormula="NO2"/>
<species metaid="M_no2_e" id="M_no2_e" name="Nitrite_b" compartment="e"
charge="0" initialConcentration="0" hasOnlySubstanceUnits="false"
boundaryCondition="true" constant="false" fbc:chemicalFormula="NO2"/>
<species metaid="M_no3_c" id="M_no3_c" name="Nitrate" compartment="c"
charge="-1" initialConcentration="0" hasOnlySubstanceUnits="false"
boundaryCondition="false" constant="false" fbc:chemicalFormula="NO3"/>
<species metaid="M_no3_e" id="M_no3_e" name="Nitrate_b" compartment="e"
charge="-1" initialConcentration="0" hasOnlySubstanceUnits="false"
boundaryCondition="true" constant="false" fbc:chemicalFormula="NO3"/>
<species metaid="M_nop_c" id="M_nop_c" name="D__Nopaline"
compartment="c" charge="-1" initialConcentration="0"
hasOnlySubstanceUnits="false" boundaryCondition="false" constant="false"
fbc:chemicalFormula="C11H19N4O6"/>
<species metaid="M_nwharg_c" id="M_nwharg_c"
name="N__(omega)__Hydroxyarginine" compartment="c" charge="0"
initialConcentration="0" hasOnlySubstanceUnits="false"
boundaryCondition="false" constant="false" fbc:chemicalFormula="C6H15N4O3"/>
<species metaid="M_o2_c" id="M_o2_c" name="O2" compartment="c"
charge="0" initialConcentration="0" hasOnlySubstanceUnits="false"
boundaryCondition="false" constant="false" fbc:chemicalFormula="O2"/>
<species metaid="M_o2_e" id="M_o2_e" name="O2_b" compartment="e"
charge="0" initialConcentration="0" hasOnlySubstanceUnits="false"
boundaryCondition="true" constant="false" fbc:chemicalFormula="O2"/>
<species metaid="M_o2s_c" id="M_o2s_c" name="Superoxide anion"
compartment="c" charge="-1" initialConcentration="0"
hasOnlySubstanceUnits="false" boundaryCondition="false" constant="false"
fbc:chemicalFormula="O2"/>
<species metaid="M_oaa_c" id="M_oaa_c" name="Oxaloacetate"
compartment="c" charge="-2" initialConcentration="0"
hasOnlySubstanceUnits="false" boundaryCondition="false" constant="false"
fbc:chemicalFormula="C4H2O5"/>
<species metaid="M_oc2coa_c" id="M_oc2coa_c"
name="trans__Oct__2__enoyl__CoA" compartment="c" charge="-4"
initialConcentration="0" hasOnlySubstanceUnits="false"
boundaryCondition="false" constant="false"
fbc:chemicalFormula="C29H44N7O17P3S"/>
<species metaid="M_ocACP_c" id="M_ocACP_c" name="Octanoyl__ACP
(n__C8:0ACP)" compartment="c" charge="0" initialConcentration="0"
hasOnlySubstanceUnits="false" boundaryCondition="false" constant="false"
fbc:chemicalFormula="C19H35N2O8PRS"/>
<species metaid="M_occoa_c" id="M_occoa_c" name="Octanoyl__CoA
(n__C8:0CoA)" compartment="c" charge="-4" initialConcentration="0"
hasOnlySubstanceUnits="false" boundaryCondition="false" constant="false"
fbc:chemicalFormula="C29H46N7O17P3S"/>
<species metaid="M_ocdca_c" id="M_ocdca_c" name="octadecanoate
(n__C18:0)" compartment="c" charge="-1" initialConcentration="0"
hasOnlySubstanceUnits="false" boundaryCondition="false" constant="false"
fbc:chemicalFormula="C18H35O2"/>

```
      <species metaid="M_ocdcaACP_c" id="M_ocdcaACP_c"
name="Octadecanoyl__ACP (n__C18:0ACP)" compartment="c" charge="-1"
initialConcentration="0" hasOnlySubstanceUnits="false"
boundaryCondition="false" constant="false"
fbc:chemicalFormula="C29H55N2O8PRS"/>
      <species metaid="M_ocdcea_c" id="M_ocdcea_c" name="octadecenoate
(n__C18:1)" compartment="c" charge="-1" initialConcentration="0"
hasOnlySubstanceUnits="false" boundaryCondition="false" constant="false"
fbc:chemicalFormula="C18H33O2"/>
      <species metaid="M_octa_c" id="M_octa_c" name="Octanoate (n__C8:0)"
compartment="c" charge="0" initialConcentration="0"
hasOnlySubstanceUnits="false" boundaryCondition="false" constant="false"
fbc:chemicalFormula=""/>
      <species metaid="M_octACP_c" id="M_octACP_c" name="Octodecanoyl__ACP"
compartment="c" charge="-1" initialConcentration="0"
hasOnlySubstanceUnits="false" boundaryCondition="false" constant="false"
fbc:chemicalFormula="C29H55N2O8PRS"/>
      <species metaid="M_octapb_c" id="M_octapb_c" name="Octanoate (protein
bound)" compartment="c" charge="0" initialConcentration="0"
hasOnlySubstanceUnits="false" boundaryCondition="false" constant="false"
fbc:chemicalFormula=""/>
      <species metaid="M_octdp_c" id="M_octdp_c" name="all__trans__Octaprenyl
diphosphate" compartment="c" charge="-3" initialConcentration="0"
hasOnlySubstanceUnits="false" boundaryCondition="false" constant="false"
fbc:chemicalFormula="C40H65O7P2"/>
      <species metaid="M_octeACP_c" id="M_octeACP_c"
name="cis__octadec__11__enoyl__[acyl__carrier protein] (n__C18:1)"
compartment="c" charge="-1" initialConcentration="0"
hasOnlySubstanceUnits="false" boundaryCondition="false" constant="false"
fbc:chemicalFormula="C29H53N2O8PRS"/>
      <species metaid="M_octp_c" id="M_octp_c" name="Octopine"
compartment="c" charge="0" initialConcentration="0"
hasOnlySubstanceUnits="false" boundaryCondition="false" constant="false"
fbc:chemicalFormula="C9H18N4O4"/>
      <species metaid="M_orn_c" id="M_orn_c" name="Ornithine" compartment="c"
charge="1" initialConcentration="0" hasOnlySubstanceUnits="false"
boundaryCondition="false" constant="false" fbc:chemicalFormula="C5H13N2O2"/>
      <species metaid="M_orn_e" id="M_orn_e" name="Ornithine_b"
compartment="e" charge="1" initialConcentration="0"
hasOnlySubstanceUnits="false" boundaryCondition="true" constant="false"
fbc:chemicalFormula="C5H13N2O2"/>
      <species metaid="M_orot_c" id="M_orot_c" name="Orotate" compartment="c"
charge="-1" initialConcentration="0" hasOnlySubstanceUnits="false"
boundaryCondition="false" constant="false" fbc:chemicalFormula="C5H3N2O4"/>
      <species metaid="M_orot5p_c" id="M_orot5p_c" name="Orotidine
5'__phosphate" compartment="c" charge="-3" initialConcentration="0"
hasOnlySubstanceUnits="false" boundaryCondition="false" constant="false"
fbc:chemicalFormula="C10H10N2O11P"/>
      <species metaid="M_osuc_c" id="M_osuc_c" name="Oxalosuccinate"
compartment="c" charge="-3" initialConcentration="0"
hasOnlySubstanceUnits="false" boundaryCondition="false" constant="false"
fbc:chemicalFormula="C6H3O7"/>
      <species metaid="M_oxa_c" id="M_oxa_c" name="Oxalate" compartment="c"
charge="-2" initialConcentration="0" hasOnlySubstanceUnits="false"
boundaryCondition="false" constant="false" fbc:chemicalFormula="C2O4"/>
      <species metaid="M_oxadpcoa_c" id="M_oxadpcoa_c"
name="3__Oxoadipyl__CoA" compartment="c" charge="-5" initialConcentration="0"
```

hasOnlySubstanceUnits="false" boundaryCondition="false" constant="false" fbc:chemicalFormula="C27H37N7O20P3S"/>
<species metaid="M_oxalc_c" id="M_oxalc_c" name="4__oxalocrotonate" compartment="c" charge="-2" initialConcentration="0" hasOnlySubstanceUnits="false" boundaryCondition="false" constant="false" fbc:chemicalFormula="C6H4O5"/>
<species metaid="M_pa_SA_c" id="M_pa_SA_c" name="phosphatidate (Saureus)" compartment="c" charge="-100" initialConcentration="0" hasOnlySubstanceUnits="false" boundaryCondition="false" constant="false" fbc:chemicalFormula="C1560H3015O192P50"/>
<species metaid="M_pa120_c" id="M_pa120_c" name="1,2__didodecanoyl__sn__glycerol 3__phosphate" compartment="c" charge="-1" initialConcentration="0" hasOnlySubstanceUnits="false" boundaryCondition="false" constant="false" fbc:chemicalFormula="C27H51O8P1"/>
<species metaid="M_pa140_c" id="M_pa140_c" name="1,2__ditetradecanoyl__sn__glycerol 3__phosphate" compartment="c" charge="-2" initialConcentration="0" hasOnlySubstanceUnits="false" boundaryCondition="false" constant="false" fbc:chemicalFormula="C31H59O8P1"/>
<species metaid="M_pa141_c" id="M_pa141_c" name="1,2__ditetradec__7__enoyl__sn__glycerol 3__phosphate" compartment="c" charge="-1" initialConcentration="0" hasOnlySubstanceUnits="false" boundaryCondition="false" constant="false" fbc:chemicalFormula="C31H55O8P1"/>
<species metaid="M_pa160_c" id="M_pa160_c" name="1,2__dihexadecanoyl__sn__glycerol 3__phosphate" compartment="c" charge="-2" initialConcentration="0" hasOnlySubstanceUnits="false" boundaryCondition="false" constant="false" fbc:chemicalFormula="C35H67O8P1"/>
<species metaid="M_pa161_c" id="M_pa161_c" name="1,2__dihexadec__9__enoyl__sn__glycerol 3__phosphate" compartment="c" charge="-1" initialConcentration="0" hasOnlySubstanceUnits="false" boundaryCondition="false" constant="false" fbc:chemicalFormula="C35H63O8P1"/>
<species metaid="M_pa180_c" id="M_pa180_c" name="1,2__dioctadecanoyl__sn__glycerol 3__phosphate" compartment="c" charge="-2" initialConcentration="0" hasOnlySubstanceUnits="false" boundaryCondition="false" constant="false" fbc:chemicalFormula="C39H75O8P1"/>
<species metaid="M_pa181_c" id="M_pa181_c" name="1,2__dioctadec__11__enoyl__sn__glycerol 3__phosphate" compartment="c" charge="-1" initialConcentration="0" hasOnlySubstanceUnits="false" boundaryCondition="false" constant="false" fbc:chemicalFormula="C39H71O8P1"/>
<species metaid="M_pac_c" id="M_pac_c" name="Phenylacetic acid" compartment="c" charge="-1" initialConcentration="0" hasOnlySubstanceUnits="false" boundaryCondition="false" constant="false" fbc:chemicalFormula="C8H7O2"/>
<species metaid="M_pad_c" id="M_pad_c" name="2__Phenylacetamide" compartment="c" charge="0" initialConcentration="0" hasOnlySubstanceUnits="false" boundaryCondition="false" constant="false" fbc:chemicalFormula="C8H9NO"/>
<species metaid="M_pala_SA2_c" id="M_pala_SA2_c" name="Phosphatidylalanine_SA2" compartment="c" charge="-50" initialConcentration="0" hasOnlySubstanceUnits="false" boundaryCondition="false" constant="false" fbc:chemicalFormula="C1710H3315N50O242P50"/>
<species metaid="M_palm24alas_c" id="M_palm24alas_c" name="Palmitoyllipoteichoic_acid_n=24__linked__D__alanine_substituted" compartment="c" charge="-24" initialConcentration="0" hasOnlySubstanceUnits="false" boundaryCondition="false" constant="false" fbc:chemicalFormula=""/>
<species metaid="M_palm24nacs_c" id="M_palm24nacs_c" name="Palmitoyllipoteichoic_acid_n=24__linked__N__acetyl__D__glucosamine"

compartment="c" charge="-24" initialConcentration="0"
hasOnlySubstanceUnits="false" boundaryCondition="false" constant="false"
fbc:chemicalFormula=""/>
<species metaid="M_palm24u_c" id="M_palm24u_c"
name="Palmitoyllipoteichoic_acid_n=24__linked__unsubstituted" compartment="c"
charge="-24" initialConcentration="0" hasOnlySubstanceUnits="false"
boundaryCondition="false" constant="false" fbc:chemicalFormula=""/>
<species metaid="M_palmACP_c" id="M_palmACP_c" name="Palmitoyl__ACP
(n__C16:0ACP)" compartment="c" charge="-1" initialConcentration="0"
hasOnlySubstanceUnits="false" boundaryCondition="false" constant="false"
fbc:chemicalFormula="C27H51N2O8PRS"/>
<species metaid="M_palmt24s_c" id="M_palmt24s_c"
name="Palmitoyllipoteichoic_acid_n=24__linked__glucose_substituted"
compartment="c" charge="-24" initialConcentration="0"
hasOnlySubstanceUnits="false" boundaryCondition="false" constant="false"
fbc:chemicalFormula=""/>
<species metaid="M_pan4p_c" id="M_pan4p_c" name="Pantetheine
4'__phosphate" compartment="c" charge="-2" initialConcentration="0"
hasOnlySubstanceUnits="false" boundaryCondition="false" constant="false"
fbc:chemicalFormula="C11H21N2O7PS"/>
<species metaid="M_pant__R_c" id="M_pant__R_c" name="(R)__Pantoate"
compartment="c" charge="-1" initialConcentration="0"
hasOnlySubstanceUnits="false" boundaryCondition="false" constant="false"
fbc:chemicalFormula="C6H11O4"/>
<species metaid="M_pap_c" id="M_pap_c" name="Adenosine
3',5'__bisphosphate" compartment="c" charge="-4" initialConcentration="0"
hasOnlySubstanceUnits="false" boundaryCondition="false" constant="false"
fbc:chemicalFormula="C10H11N5O10P2"/>
<species metaid="M_paps_c" id="M_paps_c" name="3'__Phosphoadenylyl
sulfate" compartment="c" charge="-4" initialConcentration="0"
hasOnlySubstanceUnits="false" boundaryCondition="false" constant="false"
fbc:chemicalFormula="C10H11N5O13P2S"/>
<species metaid="M_pb_e" id="M_pb_e" name="Pb_b" compartment="e"
charge="2" initialConcentration="0" hasOnlySubstanceUnits="false"
boundaryCondition="true" constant="false" fbc:chemicalFormula="Pb"/>
<species metaid="M_pdx5p_c" id="M_pdx5p_c" name="Pyridoxine
5'__phosphate" compartment="c" charge="-2" initialConcentration="0"
hasOnlySubstanceUnits="false" boundaryCondition="false" constant="false"
fbc:chemicalFormula="C8H10NO6P"/>
<species metaid="M_pe_SA_c" id="M_pe_SA_c"
name="Phosphatidylethanolamine (Saureus)" compartment="c" charge="0"
initialConcentration="0" hasOnlySubstanceUnits="false"
boundaryCondition="false" constant="false"
fbc:chemicalFormula="C1660H3365N50O192P50"/>
<species metaid="M_pe140_c" id="M_pe140_c"
name="phosphatidylethanolamine (ditetradecanoyl, n__C14:0)" compartment="c"
charge="0" initialConcentration="0" hasOnlySubstanceUnits="false"
boundaryCondition="false" constant="false"
fbc:chemicalFormula="C33H66N1O8P1"/>
<species metaid="M_pe160_c" id="M_pe160_c"
name="phosphatidylethanolamine (dihexadecanoyl, n__C16:0)" compartment="c"
charge="0" initialConcentration="0" hasOnlySubstanceUnits="false"
boundaryCondition="false" constant="false"
fbc:chemicalFormula="C37H74N1O8P1"/>
<species metaid="M_pe180_c" id="M_pe180_c"
name="phosphatidylethanolamine (dioctadecanoyl, n__C18:0)" compartment="c"
charge="0" initialConcentration="0" hasOnlySubstanceUnits="false"

```
boundaryCondition="false" constant="false"
fbc:chemicalFormula="C41H82N1O8P1"/>
      <species metaid="M_peamn_e" id="M_peamn_e" name="Phenethylamine_b"
compartment="e" charge="1" initialConcentration="0"
hasOnlySubstanceUnits="false" boundaryCondition="true" constant="false"
fbc:chemicalFormula="C8H12N"/>
      <species metaid="M_pendp_c" id="M_pendp_c"
name="all__trans__Pentaprenyl diphosphate" compartment="c" charge="-3"
initialConcentration="0" hasOnlySubstanceUnits="false"
boundaryCondition="false" constant="false" fbc:chemicalFormula="C25H41O7P2"/>
      <species metaid="M_pep_c" id="M_pep_c" name="Phosphoenolpyruvate"
compartment="c" charge="-3" initialConcentration="0"
hasOnlySubstanceUnits="false" boundaryCondition="false" constant="false"
fbc:chemicalFormula="C3H2O6P"/>
      <species metaid="M_pep_met__L_c" id="M_pep_met__L_c"
name="Peptide__L__methionine" compartment="c" charge="0"
initialConcentration="0" hasOnlySubstanceUnits="false"
boundaryCondition="false" constant="false"
fbc:chemicalFormula="C6H10N2O2R2S"/>
      <species metaid="M_pep_met__LRS_c" id="M_pep_met__LRS_c"
name="Peptide__L__methionine_R__S__oxide" compartment="c" charge="0"
initialConcentration="0" hasOnlySubstanceUnits="false"
boundaryCondition="false" constant="false" fbc:chemicalFormula=""/>
      <species metaid="M_pep_p_c" id="M_pep_p_c"
name="Peptidoglycan_polymer_n_subunits" compartment="c" charge="-4"
initialConcentration="0" hasOnlySubstanceUnits="false"
boundaryCondition="false" constant="false" fbc:chemicalFormula=""/>
      <species metaid="M_pep_p1_c" id="M_pep_p1_c"
name="Peptidoglycan_polymer_n__1_subunits" compartment="c" charge="-2"
initialConcentration="0" hasOnlySubstanceUnits="false"
boundaryCondition="false" constant="false" fbc:chemicalFormula=""/>
      <species metaid="M_pepd_c" id="M_pepd_c" name="peptidoglycan dimer"
compartment="c" charge="-1" initialConcentration="0"
hasOnlySubstanceUnits="false" boundaryCondition="false" constant="false"
fbc:chemicalFormula="C153H250N26O55P2"/>
      <species metaid="M_pepm_c" id="M_pepm_c" name="peptidoglycan monomer:
ditrans,octacis__undecaprenyldiphospho__N__acetyl__(N__acetylglucosaminyl)mur
amoyl__L__alanyl__gamma__D__isoglutaminyl__L__lysyl__(glycyl)5__D__alanyl__D_
_alanine" compartment="c" charge="-2" initialConcentration="0"
hasOnlySubstanceUnits="false" boundaryCondition="false" constant="false"
fbc:chemicalFormula="C104H170N13O31P2"/>
      <species metaid="M_pg_SA_ala_c" id="M_pg_SA_ala_c"
name="2__O__D__alanyl__1__O__phosphatidylglycerol" compartment="c" charge="1"
initialConcentration="0" hasOnlySubstanceUnits="false"
boundaryCondition="false" constant="false"
fbc:chemicalFormula="C59H115N3O22P2"/>
      <species metaid="M_pg_SA_c" id="M_pg_SA_c" name="Phosphatidylglycerol
(Saureus)" compartment="c" charge="-50" initialConcentration="0"
hasOnlySubstanceUnits="false" boundaryCondition="false" constant="false"
fbc:chemicalFormula="C1710H3365O292P50"/>
      <species metaid="M_PG_ST_c" id="M_PG_ST_c"
name="Acetylglucosamine____N__acetylmuramoyl__(tetrapeptide)
diphospho__undecaprenol dimer with L__(gly)5__D cross__link strand length 6"
compartment="c" charge="9" initialConcentration="0"
hasOnlySubstanceUnits="false" boundaryCondition="false" constant="false"
fbc:chemicalFormula="C607H983N144O280P2"/>
      <species metaid="M_pg140_c" id="M_pg140_c" name="Phosphatidylglycerol
(ditetradecanoyl, n__C14:0)" compartment="c" charge="-1"
```

initialConcentration="0" hasOnlySubstanceUnits="false"
boundaryCondition="false" constant="false"
fbc:chemicalFormula="C34H66O10P1"/>
<species metaid="M_pg160_c" id="M_pg160_c" name="Phosphatidylglycerol
(dihexadecanoyl, n__C16:0)" compartment="c" charge="-1"
initialConcentration="0" hasOnlySubstanceUnits="false"
boundaryCondition="false" constant="false"
fbc:chemicalFormula="C38H74O10P1"/>
<species metaid="M_pg180_c" id="M_pg180_c" name="Phosphatidylglycerol
(dioctadecanoyl, n__C18:0)" compartment="c" charge="-1"
initialConcentration="0" hasOnlySubstanceUnits="false"
boundaryCondition="false" constant="false"
fbc:chemicalFormula="C42H82O10P1"/>
<species metaid="M_pgly_SA2_c" id="M_pgly_SA2_c"
name="Phosphatidylglycine_SA2" compartment="c" charge="-50"
initialConcentration="0" hasOnlySubstanceUnits="false"
boundaryCondition="false" constant="false"
fbc:chemicalFormula="C1660H3215N50O242P50"/>
<species metaid="M_pglys_SA_c" id="M_pglys_SA_c"
name="2__O__D__alanyl__1__O__phosphatidylglycerolysine" compartment="c"
charge="1" initialConcentration="0" hasOnlySubstanceUnits="false"
boundaryCondition="false" constant="false"
fbc:chemicalFormula="C62H124N4O23P2"/>
<species metaid="M_pgp_SA_c" id="M_pgp_SA_c"
name="Phosphatidylglycerophosphate (Saureus)" compartment="c" charge="-150"
initialConcentration="0" hasOnlySubstanceUnits="false"
boundaryCondition="false" constant="false"
fbc:chemicalFormula="C1710H3315O442P100"/>
<species metaid="M_pgp120_c" id="M_pgp120_c"
name="Phosphatidylglycerophosphate (didodecanoyl, n__C12:0)" compartment="c"
charge="-2" initialConcentration="0" hasOnlySubstanceUnits="false"
boundaryCondition="false" constant="false"
fbc:chemicalFormula="C30H57O13P2"/>
<species metaid="M_pgp140_c" id="M_pgp140_c"
name="Phosphatidylglycerophosphate (ditetradecanoyl, n__C14:0)"
compartment="c" charge="-2" initialConcentration="0"
hasOnlySubstanceUnits="false" boundaryCondition="false" constant="false"
fbc:chemicalFormula="C34H65O13P2"/>
<species metaid="M_pgp141_c" id="M_pgp141_c"
name="Phosphatidylglycerophosphate (ditetradec__7__enoyl, n__C14:1)"
compartment="c" charge="-2" initialConcentration="0"
hasOnlySubstanceUnits="false" boundaryCondition="false" constant="false"
fbc:chemicalFormula="C34H61O13P2"/>
<species metaid="M_pgp160_c" id="M_pgp160_c"
name="Phosphatidylglycerophosphate (dihexadecanoyl, n__C16:0)"
compartment="c" charge="-3" initialConcentration="0"
hasOnlySubstanceUnits="false" boundaryCondition="false" constant="false"
fbc:chemicalFormula="C38H73O13P2"/>
<species metaid="M_pgp161_c" id="M_pgp161_c"
name="Phosphatidylglycerophosphate (dihexadec__9__enoyl, n__C16:1)"
compartment="c" charge="-3" initialConcentration="0"
hasOnlySubstanceUnits="false" boundaryCondition="false" constant="false"
fbc:chemicalFormula="C38H69O13P2"/>
<species metaid="M_pgp180_c" id="M_pgp180_c"
name="Phosphatidylglycerophosphate (dioctadecanoyl, n__C18:0)"
compartment="c" charge="-3" initialConcentration="0"
hasOnlySubstanceUnits="false" boundaryCondition="false" constant="false"
fbc:chemicalFormula="C42H81O13P2"/>

```
      <species metaid="M_pgp181_c" id="M_pgp181_c"
name="Phosphatidylglycerophosphate (dioctadec__11__enoyl, n__C18:1)"
compartment="c" charge="-2" initialConcentration="0"
hasOnlySubstanceUnits="false" boundaryCondition="false" constant="false"
fbc:chemicalFormula="C42H77O13P2"/>
      <species metaid="M_phe__L_c" id="M_phe__L_c" name="L__Phenylalanine"
compartment="c" charge="0" initialConcentration="0"
hasOnlySubstanceUnits="false" boundaryCondition="false" constant="false"
fbc:chemicalFormula="C9H11NO2"/>
      <species metaid="M_phe__L_e" id="M_phe__L_e" name="L__Phenylalanine_b"
compartment="e" charge="0" initialConcentration="0"
hasOnlySubstanceUnits="false" boundaryCondition="true" constant="false"
fbc:chemicalFormula="C9H11NO2"/>
      <species metaid="M_pheme_c" id="M_pheme_c" name="Protoheme"
compartment="c" charge="0" initialConcentration="0"
hasOnlySubstanceUnits="false" boundaryCondition="false" constant="false"
fbc:chemicalFormula="C34H30FeN4O4"/>
      <species metaid="M_pheme_e" id="M_pheme_e" name="Protoheme_b"
compartment="e" charge="0" initialConcentration="0"
hasOnlySubstanceUnits="false" boundaryCondition="true" constant="false"
fbc:chemicalFormula="C34H30FeN4O4"/>
      <species metaid="M_phis__L" id="M_phis__L" name="a
[protein]__L__histidine" compartment="c" charge="0" initialConcentration="0"
hasOnlySubstanceUnits="false" boundaryCondition="false" constant="false"
fbc:chemicalFormula="C7H8N4O2R2"/>
      <species metaid="M_phom_c" id="M_phom_c"
name="O__Phospho__L__homoserine" compartment="c" charge="-2"
initialConcentration="0" hasOnlySubstanceUnits="false"
boundaryCondition="false" constant="false" fbc:chemicalFormula="C4H8NO6P"/>
      <species metaid="M_phpyr_c" id="M_phpyr_c" name="Phenylpyruvate"
compartment="c" charge="-1" initialConcentration="0"
hasOnlySubstanceUnits="false" boundaryCondition="false" constant="false"
fbc:chemicalFormula="C9H7O3"/>
      <species metaid="M_phthr_c" id="M_phthr_c"
name="O__Phospho__4__hydroxy__L__threonine" compartment="c" charge="-2"
initialConcentration="0" hasOnlySubstanceUnits="false"
boundaryCondition="false" constant="false" fbc:chemicalFormula="C4H8NO7P"/>
      <species metaid="M_phydp_c" id="M_phydp_c" name="Phytyl diphosphate"
compartment="c" charge="-2" initialConcentration="0"
hasOnlySubstanceUnits="false" boundaryCondition="false" constant="false"
fbc:chemicalFormula="C20H39O7P2"/>
      <species metaid="M_phyQ_c" id="M_phyQ_c" name="Phylloquinone"
compartment="c" charge="0" initialConcentration="0"
hasOnlySubstanceUnits="false" boundaryCondition="false" constant="false"
fbc:chemicalFormula="C31H46O2"/>
      <species metaid="M_pi_c" id="M_pi_c" name="Phosphate" compartment="c"
charge="-2" initialConcentration="0" hasOnlySubstanceUnits="false"
boundaryCondition="false" constant="false" fbc:chemicalFormula="HO4P"/>
      <species metaid="M_pi_e" id="M_pi_e" name="Phosphate_b" compartment="e"
charge="-2" initialConcentration="0" hasOnlySubstanceUnits="false"
boundaryCondition="true" constant="false" fbc:chemicalFormula="HO4P"/>
      <species metaid="M_pi_p" id="M_pi_p" name="Phosphate" compartment="p"
charge="-2" initialConcentration="0" hasOnlySubstanceUnits="false"
boundaryCondition="false" constant="false" fbc:chemicalFormula="HO4P"/>
      <species metaid="M_pime_c" id="M_pime_c" name="Pimelate"
compartment="c" charge="-2" initialConcentration="0"
hasOnlySubstanceUnits="false" boundaryCondition="false" constant="false"
fbc:chemicalFormula="C7H10O4"/>
```

```
      <species metaid="M_pleu_SA2_c" id="M_pleu_SA2_c"
name="Phosphatidylleucine_SA2" compartment="c" charge="-50"
initialConcentration="0" hasOnlySubstanceUnits="false"
boundaryCondition="false" constant="false"
fbc:chemicalFormula="C1860H3615N50O242P50"/>
      <species metaid="M_plys_SA2_c" id="M_plys_SA2_c"
name="Phosphatidyllysine_SA2" compartment="c" charge="0"
initialConcentration="0" hasOnlySubstanceUnits="false"
boundaryCondition="false" constant="false"
fbc:chemicalFormula="C1860H3715N100O242P50"/>
      <species metaid="M_pmACP_c" id="M_pmACP_c" name="Pimeloyl__ACP "
compartment="c" charge="0" initialConcentration="0"
hasOnlySubstanceUnits="false" boundaryCondition="false" constant="false"
fbc:chemicalFormula=""/>
      <species metaid="M_pmcoa_c" id="M_pmcoa_c" name="Pimeloyl__CoA"
compartment="c" charge="-5" initialConcentration="0"
hasOnlySubstanceUnits="false" boundaryCondition="false" constant="false"
fbc:chemicalFormula="C28H41N7O19P3S"/>
      <species metaid="M_pmlmeACP_c" id="M_pmlmeACP_c" name="Pimeloyl__ACP
methyl ester" compartment="c" charge="1" initialConcentration="0"
hasOnlySubstanceUnits="false" boundaryCondition="false" constant="false"
fbc:chemicalFormula="C19H33N2O10PRS"/>
      <species metaid="M_pmtcoa_c" id="M_pmtcoa_c" name="Palmitoyl__CoA
(n__C16:0CoA)" compartment="c" charge="-4" initialConcentration="0"
hasOnlySubstanceUnits="false" boundaryCondition="false" constant="false"
fbc:chemicalFormula="C37H62N7O17P3S"/>
      <species metaid="M_pnto__R_c" id="M_pnto__R_c" name="(R)__Pantothenate"
compartment="c" charge="-1" initialConcentration="0"
hasOnlySubstanceUnits="false" boundaryCondition="false" constant="false"
fbc:chemicalFormula="C9H16NO5"/>
      <species metaid="M_ppa_c" id="M_ppa_c" name="Propionate (n__C3:0)"
compartment="c" charge="-1" initialConcentration="0"
hasOnlySubstanceUnits="false" boundaryCondition="false" constant="false"
fbc:chemicalFormula="C3H5O2"/>
      <species metaid="M_ppa_e" id="M_ppa_e" name="Propionate (n__C3:0)_b"
compartment="e" charge="-1" initialConcentration="0"
hasOnlySubstanceUnits="false" boundaryCondition="true" constant="false"
fbc:chemicalFormula="C3H5O2"/>
      <species metaid="M_ppadn_c" id="M_ppadn_c" name="Propionyladenylate"
compartment="c" charge="-1" initialConcentration="0"
hasOnlySubstanceUnits="false" boundaryCondition="false" constant="false"
fbc:chemicalFormula="C13H17N5O8P"/>
      <species metaid="M_ppap_c" id="M_ppap_c" name="Propanoyl phosphate"
compartment="c" charge="-2" initialConcentration="0"
hasOnlySubstanceUnits="false" boundaryCondition="false" constant="false"
fbc:chemicalFormula="C3H5O5P"/>
      <species metaid="M_ppbng_c" id="M_ppbng_c" name="Porphobilinogen"
compartment="c" charge="-1" initialConcentration="0"
hasOnlySubstanceUnits="false" boundaryCondition="false" constant="false"
fbc:chemicalFormula="C10H13N2O4"/>
      <species metaid="M_ppcoa_c" id="M_ppcoa_c" name="Propanoyl__CoA"
compartment="c" charge="-4" initialConcentration="0"
hasOnlySubstanceUnits="false" boundaryCondition="false" constant="false"
fbc:chemicalFormula="C24H36N7O17P3S"/>
      <species metaid="M_pphn_c" id="M_pphn_c" name="Prephenate"
compartment="c" charge="-2" initialConcentration="0"
hasOnlySubstanceUnits="false" boundaryCondition="false" constant="false"
fbc:chemicalFormula="C10H8O6"/>
```

```
      <species metaid="M_ppi_c" id="M_ppi_c" name="Diphosphate"
compartment="c" charge="-3" initialConcentration="0"
hasOnlySubstanceUnits="false" boundaryCondition="false" constant="false"
fbc:chemicalFormula="HO7P2"/>
      <species metaid="M_ppi_e" id="M_ppi_e" name="Diphosphate_b"
compartment="e" charge="-3" initialConcentration="0"
hasOnlySubstanceUnits="false" boundaryCondition="true" constant="false"
fbc:chemicalFormula="HO7P2"/>
      <species metaid="M_pplhis" id="M_pplhis" name="Protein
N(pi)__phospho__L__histidine" compartment="c" charge="-2"
initialConcentration="0" hasOnlySubstanceUnits="false"
boundaryCondition="false" constant="false"
fbc:chemicalFormula="C7H9N4O5PR2"/>
      <species metaid="M_ppp9_c" id="M_ppp9_c" name="Protoporphyrin"
compartment="c" charge="-2" initialConcentration="0"
hasOnlySubstanceUnits="false" boundaryCondition="false" constant="false"
fbc:chemicalFormula="C34H32N4O4"/>
      <species metaid="M_pppg9_c" id="M_pppg9_c" name="Protoporphyrinogen IX"
compartment="c" charge="-2" initialConcentration="0"
hasOnlySubstanceUnits="false" boundaryCondition="false" constant="false"
fbc:chemicalFormula="C34H38N4O4"/>
      <species metaid="M_pppi_c" id="M_pppi_c" name="Inorganic triphosphate"
compartment="c" charge="-4" initialConcentration="0"
hasOnlySubstanceUnits="false" boundaryCondition="false" constant="false"
fbc:chemicalFormula="HO10P3"/>
      <species metaid="M_pqq_c" id="M_pqq_c" name="Pyrroloquinoline__quinone"
compartment="c" charge="-3" initialConcentration="0"
hasOnlySubstanceUnits="false" boundaryCondition="false" constant="false"
fbc:chemicalFormula="C14H3N2O8"/>
      <species metaid="M_pqqh2_c" id="M_pqqh2_c" name="Reduced
pyrroloquinoline__quinone" compartment="c" charge="-4"
initialConcentration="0" hasOnlySubstanceUnits="false"
boundaryCondition="false" constant="false" fbc:chemicalFormula="C14H5N2O8"/>
      <species metaid="M_pram_c" id="M_pram_c"
name="5__Phospho__beta__D__ribosylamine" compartment="c" charge="-1"
initialConcentration="0" hasOnlySubstanceUnits="false"
boundaryCondition="false" constant="false" fbc:chemicalFormula="C5H11NO7P"/>
      <species metaid="M_pran_c" id="M_pran_c"
name="N__(5__Phospho__D__ribosyl)anthranilate" compartment="c" charge="-3"
initialConcentration="0" hasOnlySubstanceUnits="false"
boundaryCondition="false" constant="false" fbc:chemicalFormula="C12H13NO9P"/>
      <species metaid="M_prbamp_c" id="M_prbamp_c"
name="1__(5__Phosphoribosyl)__AMP" compartment="c" charge="-4"
initialConcentration="0" hasOnlySubstanceUnits="false"
boundaryCondition="false" constant="false"
fbc:chemicalFormula="C15H19N5O14P2"/>
      <species metaid="M_prbatp_c" id="M_prbatp_c"
name="1__(5__Phosphoribosyl)__ATP" compartment="c" charge="-6"
initialConcentration="0" hasOnlySubstanceUnits="false"
boundaryCondition="false" constant="false"
fbc:chemicalFormula="C15H19N5O20P4"/>
      <species metaid="M_pren45_c" id="M_pren45_c"
name="Prenol__45n_teichoic_acid" compartment="c" charge="-47"
initialConcentration="0" hasOnlySubstanceUnits="false"
boundaryCondition="false" constant="false" fbc:chemicalFormula=""/>
      <species metaid="M_pren45glc_c" id="M_pren45glc_c"
name="Prenol__45n_teichoic_acid__glucose_substituted" compartment="c"
```

```
charge="-47" initialConcentration="0" hasOnlySubstanceUnits="false"
boundaryCondition="false" constant="false" fbc:chemicalFormula=""/>
      <species metaid="M_pretechala_c" id="M_pretechala_c"
name="Prenol__45n_teichoic_acid__alanine_substituted" compartment="c"
charge="-47" initialConcentration="0" hasOnlySubstanceUnits="false"
boundaryCondition="false" constant="false" fbc:chemicalFormula=""/>
      <species metaid="M_prfp_c" id="M_prfp_c"
name="1__(5__Phosphoribosyl)__5__[(5__phosphoribosylamino)methylideneamino]im
idazole__4__carboxamide" compartment="c" charge="-4" initialConcentration="0"
hasOnlySubstanceUnits="false" boundaryCondition="false" constant="false"
fbc:chemicalFormula="C15H21N5O15P2"/>
      <species metaid="M_prlp_c" id="M_prlp_c"
name="5__[(5__phospho__1__deoxyribulos__1__ylamino)methylideneamino]__1__(5__
phosphoribosyl)imidazole__4__carboxamide" compartment="c" charge="-4"
initialConcentration="0" hasOnlySubstanceUnits="false"
boundaryCondition="false" constant="false"
fbc:chemicalFormula="C15H21N5O15P2"/>
      <species metaid="M_pro__L_c" id="M_pro__L_c" name="L__Proline"
compartment="c" charge="-1" initialConcentration="0"
hasOnlySubstanceUnits="false" boundaryCondition="false" constant="false"
fbc:chemicalFormula="C5H9NO2"/>
      <species metaid="M_pro__L_e" id="M_pro__L_e" name="L__Proline_b"
compartment="e" charge="-1" initialConcentration="0"
hasOnlySubstanceUnits="false" boundaryCondition="true" constant="false"
fbc:chemicalFormula="C5H9NO2"/>
      <species metaid="M_prpp_c" id="M_prpp_c"
name="5__Phospho__alpha__D__ribose 1__diphosphate" compartment="c" charge="-
5" initialConcentration="0" hasOnlySubstanceUnits="false"
boundaryCondition="false" constant="false" fbc:chemicalFormula="C5H8O14P3"/>
      <species metaid="M_ps_SA_c" id="M_ps_SA_c" name="phosphatidylserine
(Saureus)" compartment="c" charge="-50" initialConcentration="0"
hasOnlySubstanceUnits="false" boundaryCondition="false" constant="false"
fbc:chemicalFormula="C1710H3315N50O292P50"/>
      <species metaid="M_psd5p_c" id="M_psd5p_c" name="Pseudouridine
5'__phosphate" compartment="c" charge="-1" initialConcentration="0"
hasOnlySubstanceUnits="false" boundaryCondition="false" constant="false"
fbc:chemicalFormula="C9H11N2O9P"/>
      <species metaid="M_psdp_c" id="M_psdp_c" name="presqualene diphosphate"
compartment="c" charge="0" initialConcentration="0"
hasOnlySubstanceUnits="false" boundaryCondition="false" constant="false"
fbc:chemicalFormula="C30H49O7P2"/>
      <species metaid="M_pser__L_c" id="M_pser__L_c"
name="O__Phospho__L__serine" compartment="c" charge="-1"
initialConcentration="0" hasOnlySubstanceUnits="false"
boundaryCondition="false" constant="false" fbc:chemicalFormula="C3H6NO6P"/>
      <species metaid="M_psqldp_c" id="M_psqldp_c" name="presqualene
diphosphate" compartment="c" charge="-3" initialConcentration="0"
hasOnlySubstanceUnits="false" boundaryCondition="false" constant="false"
fbc:chemicalFormula="C30H49O7P2"/>
      <species metaid="M_ptp_c" id="M_ptp_c" name="triaminopyrimidine
triphosphate" compartment="c" charge="0" initialConcentration="0"
hasOnlySubstanceUnits="false" boundaryCondition="false" constant="false"
fbc:chemicalFormula="C9H14N5O14P3"/>
      <species metaid="M_ptrc_c" id="M_ptrc_c" name="Putrescine"
compartment="c" charge="2" initialConcentration="0"
hasOnlySubstanceUnits="false" boundaryCondition="false" constant="false"
fbc:chemicalFormula="C4H14N2"/>
```

<species metaid="M_ptrc_e" id="M_ptrc_e" name="Putrescine_b" compartment="e" charge="2" initialConcentration="0" hasOnlySubstanceUnits="false" boundaryCondition="true" constant="false" fbc:chemicalFormula="C4H14N2"/>
<species metaid="M_pyam5p_c" id="M_pyam5p_c" name="Pyridoxamine 5'__phosphate" compartment="c" charge="-1" initialConcentration="0" hasOnlySubstanceUnits="false" boundaryCondition="false" constant="false" fbc:chemicalFormula="C8H12N2O5P"/>
<species metaid="M_pydam_c" id="M_pydam_c" name="Pyridoxamine" compartment="c" charge="1" initialConcentration="0" hasOnlySubstanceUnits="false" boundaryCondition="false" constant="false" fbc:chemicalFormula="C8H13N2O2"/>
<species metaid="M_pydx_c" id="M_pydx_c" name="Pyridoxal" compartment="c" charge="0" initialConcentration="0" hasOnlySubstanceUnits="false" boundaryCondition="false" constant="false" fbc:chemicalFormula="C8H9NO3"/>
<species metaid="M_pydx_e" id="M_pydx_e" name="Pyridoxal_b" compartment="e" charge="0" initialConcentration="0" hasOnlySubstanceUnits="false" boundaryCondition="true" constant="false" fbc:chemicalFormula="C8H9NO3"/>
<species metaid="M_pydx5p_c" id="M_pydx5p_c" name="Pyridoxal 5'__phosphate" compartment="c" charge="-2" initialConcentration="0" hasOnlySubstanceUnits="false" boundaryCondition="false" constant="false" fbc:chemicalFormula="C8H8NO6P"/>
<species metaid="M_pydxn_c" id="M_pydxn_c" name="Pyridoxine" compartment="c" charge="0" initialConcentration="0" hasOnlySubstanceUnits="false" boundaryCondition="false" constant="false" fbc:chemicalFormula="C8H11NO3"/>
<species metaid="M_pyr_c" id="M_pyr_c" name="Pyruvate" compartment="c" charge="-1" initialConcentration="0" hasOnlySubstanceUnits="false" boundaryCondition="false" constant="false" fbc:chemicalFormula="C3H3O3"/>
<species metaid="M_pyr_e" id="M_pyr_e" name="Pyruvate_b" compartment="e" charge="-1" initialConcentration="0" hasOnlySubstanceUnits="false" boundaryCondition="true" constant="false" fbc:chemicalFormula="C3H3O3"/>
<species metaid="M_q_c" id="M_q_c" name="Ubiquinone" compartment="c" charge="0" initialConcentration="0" hasOnlySubstanceUnits="false" boundaryCondition="false" constant="false" fbc:chemicalFormula="C14H18O4"/>
<species metaid="M_qh2_c" id="M_qh2_c" name="Ubiquinol" compartment="c" charge="0" initialConcentration="0" hasOnlySubstanceUnits="false" boundaryCondition="false" constant="false" fbc:chemicalFormula="C14H20O4"/>
<species metaid="M_quin_e" id="M_quin_e" name="Quinate_b" compartment="e" charge="-1" initialConcentration="0" hasOnlySubstanceUnits="false" boundaryCondition="true" constant="false" fbc:chemicalFormula="C7H12O6"/>
<species metaid="M_r1p_c" id="M_r1p_c" name="alpha__D__Ribose 1__phosphate" compartment="c" charge="-2" initialConcentration="0" hasOnlySubstanceUnits="false" boundaryCondition="false" constant="false" fbc:chemicalFormula="C5H9O8P"/>
<species metaid="M_r3hbACP_c" id="M_r3hbACP_c" name="R__3__Hydroxybutanoyl__acyl__carrier_protein" compartment="c" charge="-1" initialConcentration="0" hasOnlySubstanceUnits="false" boundaryCondition="false" constant="false" fbc:chemicalFormula=""/>
<species metaid="M_r5p_c" id="M_r5p_c" name="alpha__D__Ribose 5__phosphate" compartment="c" charge="-2" initialConcentration="0" hasOnlySubstanceUnits="false" boundaryCondition="false" constant="false" fbc:chemicalFormula="C5H9O8P"/>

<species metaid="M_raffin_c" id="M_raffin_c" name="Raffinose" compartment="c" charge="0" initialConcentration="0" hasOnlySubstanceUnits="false" boundaryCondition="false" constant="false" fbc:chemicalFormula="C18H32O16"/>
<species metaid="M_rbflvrd_c" id="M_rbflvrd_c" name="Reduced riboflavin" compartment="c" charge="0" initialConcentration="0" hasOnlySubstanceUnits="false" boundaryCondition="false" constant="false" fbc:chemicalFormula="C17H22N4O6"/>
<species metaid="M_rbl__B_c" id="M_rbl__B_c" name="Salicin" compartment="c" charge="0" initialConcentration="0" hasOnlySubstanceUnits="false" boundaryCondition="false" constant="false" fbc:chemicalFormula="C13H18O7"/>
<species metaid="M_rbl__B_e" id="M_rbl__B_e" name="Salicin_b" compartment="e" charge="0" initialConcentration="0" hasOnlySubstanceUnits="false" boundaryCondition="true" constant="false" fbc:chemicalFormula="C13H18O7"/>
<species metaid="M_rbl__D_c" id="M_rbl__D_c" name="D__Ribulose" compartment="c" charge="0" initialConcentration="0" hasOnlySubstanceUnits="false" boundaryCondition="false" constant="false" fbc:chemicalFormula="C5H10O5"/>
<species metaid="M_rbl__L_c" id="M_rbl__L_c" name="L__Ribulose" compartment="c" charge="0" initialConcentration="0" hasOnlySubstanceUnits="false" boundaryCondition="false" constant="false" fbc:chemicalFormula="C5H10O5"/>
<species metaid="M_rbt5p_c" id="M_rbt5p_c" name="D__ribitol 5__phosphate" compartment="c" charge="-1" initialConcentration="0" hasOnlySubstanceUnits="false" boundaryCondition="false" constant="false" fbc:chemicalFormula="C5H11O8P"/>
<species metaid="M_retinal_c" id="M_retinal_c" name="Retinal" compartment="c" charge="0" initialConcentration="0" hasOnlySubstanceUnits="false" boundaryCondition="false" constant="false" fbc:chemicalFormula="C20H28O"/>
<species metaid="M_retinol_c" id="M_retinol_c" name="Retinol" compartment="c" charge="0" initialConcentration="0" hasOnlySubstanceUnits="false" boundaryCondition="false" constant="false" fbc:chemicalFormula="C20H30O"/>
<species metaid="M_rhcys_c" id="M_rhcys_c" name="S__Ribosyl__L__homocysteine" compartment="c" charge="0" initialConcentration="0" hasOnlySubstanceUnits="false" boundaryCondition="false" constant="false" fbc:chemicalFormula="C9H17NO6S"/>
<species metaid="M_rib__D_c" id="M_rib__D_c" name="D__Ribose" compartment="c" charge="0" initialConcentration="0" hasOnlySubstanceUnits="false" boundaryCondition="false" constant="false" fbc:chemicalFormula="C5H10O5"/>
<species metaid="M_rib__D_e" id="M_rib__D_e" name="D__Ribose_b" compartment="e" charge="0" initialConcentration="0" hasOnlySubstanceUnits="false" boundaryCondition="true" constant="false" fbc:chemicalFormula="C5H10O5"/>
<species metaid="M_ribflv_c" id="M_ribflv_c" name="Riboflavin" compartment="c" charge="-1" initialConcentration="0" hasOnlySubstanceUnits="false" boundaryCondition="false" constant="false" fbc:chemicalFormula="C17H20N4O6"/>
<species metaid="M_ribflv_e" id="M_ribflv_e" name="Riboflavin_b" compartment="e" charge="-1" initialConcentration="0" hasOnlySubstanceUnits="false" boundaryCondition="true" constant="false" fbc:chemicalFormula="C17H20N4O6"/>
<species metaid="M_rmn_e" id="M_rmn_e" name="L__Rhamnose_b" compartment="e" charge="0" initialConcentration="0"

hasOnlySubstanceUnits="false" boundaryCondition="true" constant="false"
fbc:chemicalFormula="C6H12O5"/>
<species metaid="M_rnam_c" id="M_rnam_c" name="N__Ribosylnicotinamide"
compartment="c" charge="1" initialConcentration="0"
hasOnlySubstanceUnits="false" boundaryCondition="false" constant="false"
fbc:chemicalFormula="C11H15N2O5"/>
<species metaid="M_ru5p__D_c" id="M_ru5p__D_c" name="D__Ribulose
5__phosphate" compartment="c" charge="-1" initialConcentration="0"
hasOnlySubstanceUnits="false" boundaryCondition="false" constant="false"
fbc:chemicalFormula="C5H9O8P"/>
<species metaid="M_ru5p__L_c" id="M_ru5p__L_c" name="L__Ribulose
5__phosphate" compartment="c" charge="-1" initialConcentration="0"
hasOnlySubstanceUnits="false" boundaryCondition="false" constant="false"
fbc:chemicalFormula="C5H9O8P"/>
<species metaid="M_s_c" id="M_s_c" name="Sulfur" compartment="c"
charge="0" initialConcentration="0" hasOnlySubstanceUnits="false"
boundaryCondition="false" constant="false" fbc:chemicalFormula="S"/>
<species metaid="M_s7p_c" id="M_s7p_c" name="Sedoheptulose
7__phosphate" compartment="c" charge="-2" initialConcentration="0"
hasOnlySubstanceUnits="false" boundaryCondition="false" constant="false"
fbc:chemicalFormula="C7H13O10P"/>
<species metaid="M_s17bp_c" id="M_s17bp_c" name="Sedoheptulose
1,7__bisphosphate" compartment="c" charge="-4" initialConcentration="0"
hasOnlySubstanceUnits="false" boundaryCondition="false" constant="false"
fbc:chemicalFormula="C7H12O13P2"/>
<species metaid="M_SA_FREE_FA_c" id="M_SA_FREE_FA_c" name="SA Free
Fatty Acids" compartment="c" charge="0" initialConcentration="0"
hasOnlySubstanceUnits="false" boundaryCondition="false" constant="false"
fbc:chemicalFormula="X"/>
<species metaid="M_salc_c" id="M_salc_c" name="Salicylate"
compartment="c" charge="-1" initialConcentration="0"
hasOnlySubstanceUnits="false" boundaryCondition="false" constant="false"
fbc:chemicalFormula="C7H5O3"/>
<species metaid="M_salc6p_c" id="M_salc6p_c" name="Salicin
6__phosphate" compartment="c" charge="-2" initialConcentration="0"
hasOnlySubstanceUnits="false" boundaryCondition="false" constant="false"
fbc:chemicalFormula="C13H17O10P"/>
<species metaid="M_salcn_e" id="M_salcn_e" name="Salicin_b"
compartment="e" charge="0" initialConcentration="0"
hasOnlySubstanceUnits="false" boundaryCondition="true" constant="false"
fbc:chemicalFormula="C13H18O7"/>
<species metaid="M_sbt__D_c" id="M_sbt__D_c" name="D__Sorbitol"
compartment="c" charge="0" initialConcentration="0"
hasOnlySubstanceUnits="false" boundaryCondition="false" constant="false"
fbc:chemicalFormula="C6H14O6"/>
<species metaid="M_sbt__D_e" id="M_sbt__D_e" name="D__Sorbitol_b"
compartment="e" charge="0" initialConcentration="0"
hasOnlySubstanceUnits="false" boundaryCondition="true" constant="false"
fbc:chemicalFormula="C6H14O6"/>
<species metaid="M_sbt6p_c" id="M_sbt6p_c" name="D__Sorbitol
6__phosphate" compartment="c" charge="-2" initialConcentration="0"
hasOnlySubstanceUnits="false" boundaryCondition="false" constant="false"
fbc:chemicalFormula="C6H13O9P"/>
<species metaid="M_sbzcoa_c" id="M_sbzcoa_c"
name="O__Succinylbenzoyl__CoA" compartment="c" charge="-5"
initialConcentration="0" hasOnlySubstanceUnits="false"
boundaryCondition="false" constant="false"
fbc:chemicalFormula="C32H39N7O20P3S"/>

<species metaid="M_scl_c" id="M_scl_c" name="sirohydrochlorin"
compartment="c" charge="-8" initialConcentration="0"
hasOnlySubstanceUnits="false" boundaryCondition="false" constant="false"
fbc:chemicalFormula="C42H39N4O16"/>
<species metaid="M_sdhlam_c" id="M_sdhlam_c"
name="S__Succinyldihydrolipoamide" compartment="c" charge="-1"
initialConcentration="0" hasOnlySubstanceUnits="false"
boundaryCondition="false" constant="false"
fbc:chemicalFormula="C12H20NO4S2"/>
<species metaid="M_ser__D_c" id="M_ser__D_c" name="D__Serine"
compartment="c" charge="0" initialConcentration="0"
hasOnlySubstanceUnits="false" boundaryCondition="false" constant="false"
fbc:chemicalFormula="C3H7NO3"/>
<species metaid="M_ser__D_e" id="M_ser__D_e" name="D__Serine_b"
compartment="e" charge="0" initialConcentration="0"
hasOnlySubstanceUnits="false" boundaryCondition="true" constant="false"
fbc:chemicalFormula="C3H7NO3"/>
<species metaid="M_ser__L_c" id="M_ser__L_c" name="L__Serine"
compartment="c" charge="0" initialConcentration="0"
hasOnlySubstanceUnits="false" boundaryCondition="false" constant="false"
fbc:chemicalFormula="C3H7NO3"/>
<species metaid="M_ser__L_e" id="M_ser__L_e" name="L__Serine_b"
compartment="e" charge="0" initialConcentration="0"
hasOnlySubstanceUnits="false" boundaryCondition="true" constant="false"
fbc:chemicalFormula="C3H7NO3"/>
<species metaid="M_sertrna_c" id="M_sertrna_c"
name="L__Seryl__tRNA(Ser)" compartment="c" charge="1"
initialConcentration="0" hasOnlySubstanceUnits="false"
boundaryCondition="false" constant="false" fbc:chemicalFormula="C3H6NO2R"/>
<species metaid="M_sheme_c" id="M_sheme_c" name="Siroheme"
compartment="c" charge="-8" initialConcentration="0"
hasOnlySubstanceUnits="false" boundaryCondition="false" constant="false"
fbc:chemicalFormula="C42H36FeN4O16"/>
<species metaid="M_skgmeACP_c" id="M_skgmeACP_c"
name="3__Ketoglutaryl__ACP methyl ester" compartment="c" charge="1"
initialConcentration="0" hasOnlySubstanceUnits="false"
boundaryCondition="false" constant="false"
fbc:chemicalFormula="C17H27N2O11PRS"/>
<species metaid="M_skm_c" id="M_skm_c" name="Shikimate" compartment="c"
charge="-1" initialConcentration="0" hasOnlySubstanceUnits="false"
boundaryCondition="false" constant="false" fbc:chemicalFormula="C7H9O5"/>
<species metaid="M_skm5p_c" id="M_skm5p_c" name="Shikimate
5__phosphate" compartment="c" charge="-3" initialConcentration="0"
hasOnlySubstanceUnits="false" boundaryCondition="false" constant="false"
fbc:chemicalFormula="C7H8O8P"/>
<species metaid="M_skpmeACP_c" id="M_skpmeACP_c"
name="3__Ketopimeloyl__ACP methyl ester" compartment="c" charge="1"
initialConcentration="0" hasOnlySubstanceUnits="false"
boundaryCondition="false" constant="false"
fbc:chemicalFormula="C19H31N2O11PRS"/>
<species metaid="M_sl2a6o_c" id="M_sl2a6o_c"
name="N__Succinyl__2__L__amino__6__oxoheptanedioate" compartment="c"
charge="-3" initialConcentration="0" hasOnlySubstanceUnits="false"
boundaryCondition="false" constant="false" fbc:chemicalFormula="C11H12NO8"/>
<species metaid="M_sl26da_c" id="M_sl26da_c"
name="N__Succinyl__LL__2,6__diaminoheptanedioate" compartment="c" charge="-2"
initialConcentration="0" hasOnlySubstanceUnits="false"
boundaryCondition="false" constant="false" fbc:chemicalFormula="C11H16N2O7"/>

<species metaid="M_so3_c" id="M_so3_c" name="Sulfite" compartment="c" charge="-2" initialConcentration="0" hasOnlySubstanceUnits="false" boundaryCondition="false" constant="false" fbc:chemicalFormula="O3S"/>
<species metaid="M_so4_c" id="M_so4_c" name="Sulfate" compartment="c" charge="-2" initialConcentration="0" hasOnlySubstanceUnits="false" boundaryCondition="false" constant="false" fbc:chemicalFormula="O4S"/>
<species metaid="M_so4_e" id="M_so4_e" name="Sulfate_b" compartment="e" charge="-2" initialConcentration="0" hasOnlySubstanceUnits="false" boundaryCondition="true" constant="false" fbc:chemicalFormula="O4S"/>
<species metaid="M_spmd_c" id="M_spmd_c" name="Spermidine" compartment="c" charge="3" initialConcentration="0" hasOnlySubstanceUnits="false" boundaryCondition="false" constant="false" fbc:chemicalFormula="C7H22N3"/>
<species metaid="M_spmd_e" id="M_spmd_e" name="Spermidine_b" compartment="e" charge="3" initialConcentration="0" hasOnlySubstanceUnits="false" boundaryCondition="true" constant="false" fbc:chemicalFormula="C7H22N3"/>
<species metaid="M_sql_c" id="M_sql_c" name="Squalene" compartment="c" charge="0" initialConcentration="0" hasOnlySubstanceUnits="false" boundaryCondition="false" constant="false" fbc:chemicalFormula="C30H50"/>
<species metaid="M_ssaltpp_c" id="M_ssaltpp_c" name="Succinate semialdehyde__thiamin diphosphate anion" compartment="c" charge="-2" initialConcentration="0" hasOnlySubstanceUnits="false" boundaryCondition="false" constant="false" fbc:chemicalFormula="C16H21N4O10P2S"/>
<species metaid="M_stcoa_c" id="M_stcoa_c" name="Stearoyl__CoA (n__C18:0CoA)" compartment="c" charge="-4" initialConcentration="0" hasOnlySubstanceUnits="false" boundaryCondition="false" constant="false" fbc:chemicalFormula="C39H66N7O17P3S"/>
<species metaid="M_stear24ds_c" id="M_stear24ds_c" name="Stearoyllipoteichoic_acid_n=24__linked__D__alanine_substituted" compartment="c" charge="-24" initialConcentration="0" hasOnlySubstanceUnits="false" boundaryCondition="false" constant="false" fbc:chemicalFormula=""/>
<species metaid="M_stear24s_c" id="M_stear24s_c" name="Stearoyllipoteichoic_acid_n=24__linked__N__acetyl__D__glucosamine" compartment="c" charge="-24" initialConcentration="0" hasOnlySubstanceUnits="false" boundaryCondition="false" constant="false" fbc:chemicalFormula=""/>
<species metaid="M_stear24u_c" id="M_stear24u_c" name="Stearoyllipoteichoic_acid_n=24__linked__unsubstituted" compartment="c" charge="-24" initialConcentration="0" hasOnlySubstanceUnits="false" boundaryCondition="false" constant="false" fbc:chemicalFormula=""/>
<species metaid="M_steart24s_c" id="M_steart24s_c" name="Stearoyllipoteichoic_acid_n=24__linked__glucose_substituted" compartment="c" charge="-24" initialConcentration="0" hasOnlySubstanceUnits="false" boundaryCondition="false" constant="false" fbc:chemicalFormula=""/>
<species metaid="M_stecard_c" id="M_stecard_c" name="Stearoylcardiolipin_B._subtilis" compartment="c" charge="-2" initialConcentration="0" hasOnlySubstanceUnits="false" boundaryCondition="false" constant="false" fbc:chemicalFormula=""/>
<species metaid="M_stphxln_c" id="M_stphxln_c" name="staphyloxanthin" compartment="c" charge="0" initialConcentration="0" hasOnlySubstanceUnits="false" boundaryCondition="false" constant="false" fbc:chemicalFormula="C51H78O9"/>
<species metaid="M_suc6p_c" id="M_suc6p_c" name="Sucrose 6__phosphate" compartment="c" charge="-2" initialConcentration="0"

hasOnlySubstanceUnits="false" boundaryCondition="false" constant="false"
fbc:chemicalFormula="C12H21O14P"/>
<species metaid="M_sucbz_c" id="M_sucbz_c" name="o__Succinylbenzoate"
compartment="c" charge="-2" initialConcentration="0"
hasOnlySubstanceUnits="false" boundaryCondition="false" constant="false"
fbc:chemicalFormula="C11H8O5"/>
<species metaid="M_succ_c" id="M_succ_c" name="Succinate"
compartment="c" charge="-2" initialConcentration="0"
hasOnlySubstanceUnits="false" boundaryCondition="false" constant="false"
fbc:chemicalFormula="C4H4O4"/>
<species metaid="M_succ_e" id="M_succ_e" name="Succinate_b"
compartment="e" charge="-2" initialConcentration="0"
hasOnlySubstanceUnits="false" boundaryCondition="true" constant="false"
fbc:chemicalFormula="C4H4O4"/>
<species metaid="M_succoa_c" id="M_succoa_c" name="Succinyl__CoA"
compartment="c" charge="-5" initialConcentration="0"
hasOnlySubstanceUnits="false" boundaryCondition="false" constant="false"
fbc:chemicalFormula="C25H35N7O19P3S"/>
<species metaid="M_suchms_c" id="M_suchms_c"
name="O__Succinyl__L__homoserine" compartment="c" charge="-1"
initialConcentration="0" hasOnlySubstanceUnits="false"
boundaryCondition="false" constant="false" fbc:chemicalFormula="C8H12NO6"/>
<species metaid="M_sucr_c" id="M_sucr_c" name="Sucrose" compartment="c"
charge="0" initialConcentration="0" hasOnlySubstanceUnits="false"
boundaryCondition="false" constant="false" fbc:chemicalFormula="C12H22O11"/>
<species metaid="M_sucr_e" id="M_sucr_e" name="Sucrose_b"
compartment="e" charge="0" initialConcentration="0"
hasOnlySubstanceUnits="false" boundaryCondition="true" constant="false"
fbc:chemicalFormula="C12H22O11"/>
<species metaid="M_sucsal_c" id="M_sucsal_c" name="Succinic
semialdehyde" compartment="c" charge="-1" initialConcentration="0"
hasOnlySubstanceUnits="false" boundaryCondition="false" constant="false"
fbc:chemicalFormula="C4H5O3"/>
<species metaid="M_sufbcd_2fe2s_c" id="M_sufbcd_2fe2s_c" name="SufBCD
with bound [2Fe__2S] cluster" compartment="c" charge="0"
initialConcentration="0" hasOnlySubstanceUnits="false"
boundaryCondition="false" constant="false" fbc:chemicalFormula="H4O2S8Fe2R"/>
<species metaid="M_sufbcd_2fe2s2_c" id="M_sufbcd_2fe2s2_c" name="SufBCD
with two bound [2Fe__2S] clusters" compartment="c" charge="0"
initialConcentration="0" hasOnlySubstanceUnits="false"
boundaryCondition="false" constant="false" fbc:chemicalFormula="O2S10Fe4R"/>
<species metaid="M_sufbcd_4fe4s_c" id="M_sufbcd_4fe4s_c" name="SufBCD
with bound [4Fe__4S] cluster" compartment="c" charge="0"
initialConcentration="0" hasOnlySubstanceUnits="false"
boundaryCondition="false" constant="false"
fbc:chemicalFormula="H4O2S10Fe4R"/>
<species metaid="M_sufbcd_c" id="M_sufbcd_c" name="SufBCD scaffold
complex" compartment="c" charge="0" initialConcentration="0"
hasOnlySubstanceUnits="false" boundaryCondition="false" constant="false"
fbc:chemicalFormula="H8O2S6R"/>
<species metaid="M_sufse_c" id="M_sufse_c" name="SufSE sulfur acceptor
complex" compartment="c" charge="0" initialConcentration="0"
hasOnlySubstanceUnits="false" boundaryCondition="false" constant="false"
fbc:chemicalFormula="HSR"/>
<species metaid="M_sufsesh_c" id="M_sufsesh_c" name="SufSE with bound
sulfur" compartment="c" charge="0" initialConcentration="0"
hasOnlySubstanceUnits="false" boundaryCondition="false" constant="false"
fbc:chemicalFormula="HS2R"/>

```
      <species metaid="M_tag6p__D_c" id="M_tag6p__D_c" name="D__Tagatose
6__phosphate" compartment="c" charge="-1" initialConcentration="0"
hasOnlySubstanceUnits="false" boundaryCondition="false" constant="false"
fbc:chemicalFormula="C6H11O9P"/>
      <species metaid="M_tagdp__D_c" id="M_tagdp__D_c" name="D__Tagatose
1,6__biphosphate" compartment="c" charge="-2" initialConcentration="0"
hasOnlySubstanceUnits="false" boundaryCondition="false" constant="false"
fbc:chemicalFormula="C6H10O12P2"/>
      <species metaid="M_tartr__D_e" id="M_tartr__D_e" name="D__tartrate_b"
compartment="e" charge="-2" initialConcentration="0"
hasOnlySubstanceUnits="false" boundaryCondition="true" constant="false"
fbc:chemicalFormula="C4H4O6"/>
      <species metaid="M_tartr__L_e" id="M_tartr__L_e" name="L__tartrate_b"
compartment="e" charge="-2" initialConcentration="0"
hasOnlySubstanceUnits="false" boundaryCondition="true" constant="false"
fbc:chemicalFormula="C4H4O6"/>
      <species metaid="M_taur_c" id="M_taur_c" name="Taurine" compartment="c"
charge="0" initialConcentration="0" hasOnlySubstanceUnits="false"
boundaryCondition="false" constant="false" fbc:chemicalFormula="C2H7NO3S"/>
      <species metaid="M_taur_e" id="M_taur_e" name="Taurine_b"
compartment="e" charge="0" initialConcentration="0"
hasOnlySubstanceUnits="false" boundaryCondition="true" constant="false"
fbc:chemicalFormula="C2H7NO3S"/>
      <species metaid="M_tcam_c" id="M_tcam_c" name="minor teichoic acid
(acetylgalactosamine glucose phosphate, n=30)" compartment="c" charge="-30"
initialConcentration="0" hasOnlySubstanceUnits="false"
boundaryCondition="false" constant="false"
fbc:chemicalFormula="C420H750N30O420P30"/>
      <species metaid="M_tchola_c" id="M_tchola_c" name="taurocholic acid"
compartment="c" charge="-1" initialConcentration="0"
hasOnlySubstanceUnits="false" boundaryCondition="false" constant="false"
fbc:chemicalFormula="C26H45NO7S"/>
      <species metaid="M_td2coa_c" id="M_td2coa_c"
name="trans__Tetradec__2__enoyl__CoA" compartment="c" charge="-4"
initialConcentration="0" hasOnlySubstanceUnits="false"
boundaryCondition="false" constant="false"
fbc:chemicalFormula="C35H56N7O17P3S"/>
      <species metaid="M_tdcoa_c" id="M_tdcoa_c" name="Tetradecanoyl__CoA
(n__C14:0CoA)" compartment="c" charge="-4" initialConcentration="0"
hasOnlySubstanceUnits="false" boundaryCondition="false" constant="false"
fbc:chemicalFormula="C35H58N7O17P3S"/>
      <species metaid="M_tddec2eACP_c" id="M_tddec2eACP_c"
name="trans__Dodec__2__enoyl__[acyl__carrier protein]" compartment="c"
charge="0" initialConcentration="0" hasOnlySubstanceUnits="false"
boundaryCondition="false" constant="false"
fbc:chemicalFormula="C23H41N2O8PRS"/>
      <species metaid="M_tdeACP_c" id="M_tdeACP_c"
name="cis__tetradec__7__enoyl__[acyl__carrier protein] (n__C14:1)"
compartment="c" charge="0" initialConcentration="0"
hasOnlySubstanceUnits="false" boundaryCondition="false" constant="false"
fbc:chemicalFormula="C25H45N2O8PRS"/>
      <species metaid="M_tdec2eACP_c" id="M_tdec2eACP_c"
name="trans__Dec__2__enoyl__[acyl__carrier protein]" compartment="c"
charge="0" initialConcentration="0" hasOnlySubstanceUnits="false"
boundaryCondition="false" constant="false"
fbc:chemicalFormula="C21H37N2O8PRS"/>
      <species metaid="M_teich__45_c" id="M_teich__45_c"
name="teichuronic_acid_GlcA__GalNac__45_repeating_unit" compartment="c"
```

```
charge="-45" initialConcentration="0" hasOnlySubstanceUnits="false"
boundaryCondition="false" constant="false" fbc:chemicalFormula=""/>
      <species metaid="M_thdcar_c" id="M_thdcar_c"
name="all__trans__4,4'__diapo__?__carotene" compartment="c" charge="0"
initialConcentration="0" hasOnlySubstanceUnits="false"
boundaryCondition="false" constant="false" fbc:chemicalFormula="C30H44"/>
      <species metaid="M_thdp_c" id="M_thdp_c"
name="2,3,4,5__Tetrahydrodipicolinate" compartment="c" charge="-2"
initialConcentration="0" hasOnlySubstanceUnits="false"
boundaryCondition="false" constant="false" fbc:chemicalFormula="C7H7NO4"/>
      <species metaid="M_thex2eACP_c" id="M_thex2eACP_c"
name="trans__Hex__2__enoyl__[acyl__carrier protein]" compartment="c"
charge="0" initialConcentration="0" hasOnlySubstanceUnits="false"
boundaryCondition="false" constant="false"
fbc:chemicalFormula="C17H29N2O8PRS"/>
      <species metaid="M_thf_c" id="M_thf_c" name="5,6,7,8__Tetrahydrofolate"
compartment="c" charge="-2" initialConcentration="0"
hasOnlySubstanceUnits="false" boundaryCondition="false" constant="false"
fbc:chemicalFormula="C19H21N7O6"/>
      <species metaid="M_thfglu_c" id="M_thfglu_c"
name="Tetrahydrofolyl__[Glu](2)" compartment="c" charge="-3"
initialConcentration="0" hasOnlySubstanceUnits="false"
boundaryCondition="false" constant="false" fbc:chemicalFormula="C24H27N8O9"/>
      <species metaid="M_thm_c" id="M_thm_c" name="Thiamin" compartment="c"
charge="1" initialConcentration="0" hasOnlySubstanceUnits="false"
boundaryCondition="false" constant="false" fbc:chemicalFormula="C12H17N4OS"/>
      <species metaid="M_thm_e" id="M_thm_e" name="Thiamin_b" compartment="e"
charge="1" initialConcentration="0" hasOnlySubstanceUnits="false"
boundaryCondition="true" constant="false" fbc:chemicalFormula="C12H17N4OS"/>
      <species metaid="M_thmmp_c" id="M_thmmp_c" name="Thiamin monophosphate"
compartment="c" charge="-1" initialConcentration="0"
hasOnlySubstanceUnits="false" boundaryCondition="false" constant="false"
fbc:chemicalFormula="C12H16N4O4PS"/>
      <species metaid="M_thmpp_c" id="M_thmpp_c" name="Thiamine diphosphate"
compartment="c" charge="-2" initialConcentration="0"
hasOnlySubstanceUnits="false" boundaryCondition="false" constant="false"
fbc:chemicalFormula="C12H16N4O7P2S"/>
      <species metaid="M_thr__L_c" id="M_thr__L_c" name="L__Threonine"
compartment="c" charge="0" initialConcentration="0"
hasOnlySubstanceUnits="false" boundaryCondition="false" constant="false"
fbc:chemicalFormula="C4H9NO3"/>
      <species metaid="M_thr__L_e" id="M_thr__L_e" name="L__Threonine_b"
compartment="e" charge="0" initialConcentration="0"
hasOnlySubstanceUnits="false" boundaryCondition="true" constant="false"
fbc:chemicalFormula="C4H9NO3"/>
      <species metaid="M_thrtrna_c" id="M_thrtrna_c"
name="L__Threonyl__tRNA(Thr)" compartment="c" charge="1"
initialConcentration="0" hasOnlySubstanceUnits="false"
boundaryCondition="false" constant="false" fbc:chemicalFormula="C4H8NO2R"/>
      <species metaid="M_thym_c" id="M_thym_c" name="Thymine" compartment="c"
charge="0" initialConcentration="0" hasOnlySubstanceUnits="false"
boundaryCondition="false" constant="false" fbc:chemicalFormula="C5H6N2O2"/>
      <species metaid="M_thym_e" id="M_thym_e" name="Thymine_b"
compartment="e" charge="0" initialConcentration="0"
hasOnlySubstanceUnits="false" boundaryCondition="true" constant="false"
fbc:chemicalFormula="C5H6N2O2"/>
```

<species metaid="M_thym_p" id="M_thym_p" name="Thymine" compartment="p" charge="0" initialConcentration="0" hasOnlySubstanceUnits="false" boundaryCondition="false" constant="false" fbc:chemicalFormula="C5H6N2O2"/>
<species metaid="M_thymd_c" id="M_thymd_c" name="Thymidine" compartment="c" charge="0" initialConcentration="0" hasOnlySubstanceUnits="false" boundaryCondition="false" constant="false" fbc:chemicalFormula="C10H14N2O5"/>
<species metaid="M_thymd_e" id="M_thymd_e" name="Thymidine_b" compartment="e" charge="0" initialConcentration="0" hasOnlySubstanceUnits="false" boundaryCondition="true" constant="false" fbc:chemicalFormula="C10H14N2O5"/>
<species metaid="M_tmrs2eACP_c" id="M_tmrs2eACP_c" name="trans__Tetradec__2__enoyl__[acyl__carrier protein]" compartment="c" charge="0" initialConcentration="0" hasOnlySubstanceUnits="false" boundaryCondition="false" constant="false" fbc:chemicalFormula="C25H45N2O8PRS"/>
<species metaid="M_to2eACP_c" id="M_to2eACP_c" name="trans__Octodec__2__enoyl__ACP" compartment="c" charge="-1" initialConcentration="0" hasOnlySubstanceUnits="false" boundaryCondition="false" constant="false" fbc:chemicalFormula="C29H53N2O8PRS"/>
<species metaid="M_toct2eACP_c" id="M_toct2eACP_c" name="trans__Oct__2__enoyl__[acyl__carrier protein]" compartment="c" charge="0" initialConcentration="0" hasOnlySubstanceUnits="false" boundaryCondition="false" constant="false" fbc:chemicalFormula="C19H33N2O8PRS"/>
<species metaid="M_tpalm2eACP_c" id="M_tpalm2eACP_c" name="trans__Hexadec__2__enoyl__[acyl__carrier protein]" compartment="c" charge="0" initialConcentration="0" hasOnlySubstanceUnits="false" boundaryCondition="false" constant="false" fbc:chemicalFormula="C27H49N2O8PRS"/>
<species metaid="M_trdox_c" id="M_trdox_c" name="Oxidized thioredoxin" compartment="c" charge="0" initialConcentration="0" hasOnlySubstanceUnits="false" boundaryCondition="false" constant="false" fbc:chemicalFormula="X"/>
<species metaid="M_trdrd_c" id="M_trdrd_c" name="Reduced thioredoxin" compartment="c" charge="0" initialConcentration="0" hasOnlySubstanceUnits="false" boundaryCondition="false" constant="false" fbc:chemicalFormula="XH2"/>
<species metaid="M_tre_c" id="M_tre_c" name="Trehalose" compartment="c" charge="0" initialConcentration="0" hasOnlySubstanceUnits="false" boundaryCondition="false" constant="false" fbc:chemicalFormula="C12H22O11"/>
<species metaid="M_tre_e" id="M_tre_e" name="Trehalose_b" compartment="e" charge="0" initialConcentration="0" hasOnlySubstanceUnits="false" boundaryCondition="true" constant="false" fbc:chemicalFormula="C12H22O11"/>
<species metaid="M_tre6p_c" id="M_tre6p_c" name="alpha,alpha'__Trehalose 6__phosphate" compartment="c" charge="-2" initialConcentration="0" hasOnlySubstanceUnits="false" boundaryCondition="false" constant="false" fbc:chemicalFormula="C12H21O14P"/>
<species metaid="M_trnaala_c" id="M_trnaala_c" name="tRNA(Ala)" compartment="c" charge="0" initialConcentration="0" hasOnlySubstanceUnits="false" boundaryCondition="false" constant="false" fbc:chemicalFormula="R"/>
<species metaid="M_trnaarg_c" id="M_trnaarg_c" name="tRNA(Arg)" compartment="c" charge="0" initialConcentration="0" hasOnlySubstanceUnits="false" boundaryCondition="false" constant="false" fbc:chemicalFormula="R"/>

<species metaid="M_trnaasn_c" id="M_trnaasn_c" name="tRNA(Asn)" compartment="c" charge="0" initialConcentration="0" hasOnlySubstanceUnits="false" boundaryCondition="false" constant="false" fbc:chemicalFormula="C10H17O10PR2"/>
<species metaid="M_trnaasp_c" id="M_trnaasp_c" name="tRNA(Asp)" compartment="c" charge="0" initialConcentration="0" hasOnlySubstanceUnits="false" boundaryCondition="false" constant="false" fbc:chemicalFormula="R"/>
<species metaid="M_trnacys_c" id="M_trnacys_c" name="tRNA(Cys)" compartment="c" charge="-1" initialConcentration="0" hasOnlySubstanceUnits="false" boundaryCondition="false" constant="false" fbc:chemicalFormula="R"/>
<species metaid="M_trnaglu_c" id="M_trnaglu_c" name="tRNA (Glu)" compartment="c" charge="0" initialConcentration="0" hasOnlySubstanceUnits="false" boundaryCondition="false" constant="false" fbc:chemicalFormula="R"/>
<species metaid="M_trnagly_c" id="M_trnagly_c" name="tRNA(Gly)" compartment="c" charge="0" initialConcentration="0" hasOnlySubstanceUnits="false" boundaryCondition="false" constant="false" fbc:chemicalFormula="R"/>
<species metaid="M_trnaile_c" id="M_trnaile_c" name="tRNA(Ile)" compartment="c" charge="0" initialConcentration="0" hasOnlySubstanceUnits="false" boundaryCondition="false" constant="false" fbc:chemicalFormula="R"/>
<species metaid="M_trnalys_c" id="M_trnalys_c" name="tRNA(Lys)" compartment="c" charge="0" initialConcentration="0" hasOnlySubstanceUnits="false" boundaryCondition="false" constant="false" fbc:chemicalFormula="R"/>
<species metaid="M_trnamet_c" id="M_trnamet_c" name="tRNA(Met)" compartment="c" charge="0" initialConcentration="0" hasOnlySubstanceUnits="false" boundaryCondition="false" constant="false" fbc:chemicalFormula="R"/>
<species metaid="M_trnaser_c" id="M_trnaser_c" name="tRNA(Ser)" compartment="c" charge="0" initialConcentration="0" hasOnlySubstanceUnits="false" boundaryCondition="false" constant="false" fbc:chemicalFormula="R"/>
<species metaid="M_trnathr_c" id="M_trnathr_c" name="tRNA(Thr)" compartment="c" charge="0" initialConcentration="0" hasOnlySubstanceUnits="false" boundaryCondition="false" constant="false" fbc:chemicalFormula="R"/>
<species metaid="M_trp__L_c" id="M_trp__L_c" name="L__Tryptophan" compartment="c" charge="0" initialConcentration="0" hasOnlySubstanceUnits="false" boundaryCondition="false" constant="false" fbc:chemicalFormula="C11H12N2O2"/>
<species metaid="M_trp__L_e" id="M_trp__L_e" name="L__Tryptophan_b" compartment="e" charge="0" initialConcentration="0" hasOnlySubstanceUnits="false" boundaryCondition="true" constant="false" fbc:chemicalFormula="C11H12N2O2"/>
<species metaid="M_tsul_c" id="M_tsul_c" name="Thiosulfate" compartment="c" charge="-1" initialConcentration="0" hasOnlySubstanceUnits="false" boundaryCondition="false" constant="false" fbc:chemicalFormula="O3S2"/>
<species metaid="M_tsul_e" id="M_tsul_e" name="Thiosulfate_b" compartment="e" charge="-1" initialConcentration="0" hasOnlySubstanceUnits="false" boundaryCondition="true" constant="false" fbc:chemicalFormula="O3S2"/>
<species metaid="M_ttdca_c" id="M_ttdca_c" name="tetradecanoate (n__C14:0)" compartment="c" charge="-1" initialConcentration="0"

hasOnlySubstanceUnits="false" boundaryCondition="false" constant="false" fbc:chemicalFormula="C14H27O2"/>
<species metaid="M_ttdcea_c" id="M_ttdcea_c" name="tetradecenoate (n__C14:1)" compartment="c" charge="-1" initialConcentration="0" hasOnlySubstanceUnits="false" boundaryCondition="false" constant="false" fbc:chemicalFormula="C14H25O2"/>
<species metaid="M_tym_e" id="M_tym_e" name="Tyramine_b" compartment="e" charge="1" initialConcentration="0" hasOnlySubstanceUnits="false" boundaryCondition="true" constant="false" fbc:chemicalFormula="C8H12NO"/>
<species metaid="M_tyr__L_c" id="M_tyr__L_c" name="L__Tyrosine" compartment="c" charge="0" initialConcentration="0" hasOnlySubstanceUnits="false" boundaryCondition="false" constant="false" fbc:chemicalFormula="C9H11NO3"/>
<species metaid="M_tyr__L_e" id="M_tyr__L_e" name="L__Tyrosine_b" compartment="e" charge="0" initialConcentration="0" hasOnlySubstanceUnits="false" boundaryCondition="true" constant="false" fbc:chemicalFormula="C9H11NO3"/>
<species metaid="M_uaaAgla_c" id="M_uaaAgla_c" name="Undecaprenyl__diphospho__N__acetylmuramoyl__(N__acetylglucosamine)__L__alanyl__D__glutamyl__L__lysyl__D__alanyl__D__alanine" compartment="c" charge="-3" initialConcentration="0" hasOnlySubstanceUnits="false" boundaryCondition="false" constant="false" fbc:chemicalFormula="C94H153N8O26P2"/>
<species metaid="M_uaaAgtla_c" id="M_uaaAgtla_c" name="Undecaprenyl__diphospho__N__acetylmuramoyl__(N__acetylglucosamine)__L__alanyl__D__glutaminyl__L__lysyl__D__alanyl__D__alanine" compartment="c" charge="-2" initialConcentration="0" hasOnlySubstanceUnits="false" boundaryCondition="false" constant="false" fbc:chemicalFormula="C94H155N9O25P2"/>
<species metaid="M_uaaGgla_c" id="M_uaaGgla_c" name="Undecaprenyl__diphospho__N__acetylmuramoyl__(N__acetylglucosamine)__L__alanyl__gamma__D__glutamyl__L__lysyl__D__alanyl__D__alanine" compartment="c" charge="-3" initialConcentration="0" hasOnlySubstanceUnits="false" boundaryCondition="false" constant="false" fbc:chemicalFormula="C94H153N8O26P2"/>
<species metaid="M_uaaGgtla_c" id="M_uaaGgtla_c" name="Undecaprenyl__diphospho__N__acetylmuramoyl__(N__acetylglucosamine)__L__alanyl__D__isoglutaminyl__L__lysyl__D__alanyl__D__alanine" compartment="c" charge="-2" initialConcentration="0" hasOnlySubstanceUnits="false" boundaryCondition="false" constant="false" fbc:chemicalFormula="C94H155N9O25P2"/>
<species metaid="M_uaagmda_c" id="M_uaagmda_c" name="Undecaprenyl__diphospho__N__acetylmuramoyl__(N__acetylglucosamine)__L__ala__D__glu__meso__2,6__diaminopimeloyl__D__ala__D__ala" compartment="c" charge="-4" initialConcentration="0" hasOnlySubstanceUnits="false" boundaryCondition="false" constant="false" fbc:chemicalFormula="C95H152N8O28P2"/>
<species metaid="M_uaagtmda_c" id="M_uaagtmda_c" name="Undecaprenyl__diphospho__N__acetylmuramoyl__(N__acetylglucosamine)__L__alanyl__D__glutaminyl__meso__2,6__diaminopimeloyl__D__alanyl__D__alanine" compartment="c" charge="-3" initialConcentration="0" hasOnlySubstanceUnits="false" boundaryCondition="false" constant="false" fbc:chemicalFormula="C95H154N9O27P2"/>
<species metaid="M_uaccg_c" id="M_uaccg_c" name="UDP__N__acetyl__3__O__(1__carboxyvinyl)__D__glucosamine" compartment="c" charge="-3" initialConcentration="0"

```
hasOnlySubstanceUnits="false" boundaryCondition="false" constant="false"
fbc:chemicalFormula="C20H26N3O19P2"/>
      <species metaid="M_uacgam_c" id="M_uacgam_c"
name="UDP__N__acetyl__D__glucosamine" compartment="c" charge="-2"
initialConcentration="0" hasOnlySubstanceUnits="false"
boundaryCondition="false" constant="false"
fbc:chemicalFormula="C17H25N3O17P2"/>
      <species metaid="M_uacmam_c" id="M_uacmam_c"
name="UDP__N__acetyl__D__mannosamine" compartment="c" charge="-2"
initialConcentration="0" hasOnlySubstanceUnits="false"
boundaryCondition="false" constant="false"
fbc:chemicalFormula="C17H25N3O17P2"/>
      <species metaid="M_uacmamu_c" id="M_uacmamu_c"
name="UDP__N__acetyl__D__mannosaminouronate" compartment="c" charge="-3"
initialConcentration="0" hasOnlySubstanceUnits="false"
boundaryCondition="false" constant="false"
fbc:chemicalFormula="C17H22N3O18P2"/>
      <species metaid="M_uaGgla_c" id="M_uaGgla_c"
name="Undecaprenyl__diphospho__N__acetylmuramoyl__L__alanyl__gamma__D__glutam
yl__L__lysyl__D__alanyl__D__alanine" compartment="c" charge="-3"
initialConcentration="0" hasOnlySubstanceUnits="false"
boundaryCondition="false" constant="false"
fbc:chemicalFormula="C86H140N7O21P2"/>
      <species metaid="M_uagmda_c" id="M_uagmda_c"
name="Undecaprenyl__diphospho__N__acetylmuramoyl__L__alanyl__D__glutamyl__mes
o__2,6__diaminopimeloyl__D__alanyl__D__alanine" compartment="c" charge="-4"
initialConcentration="0" hasOnlySubstanceUnits="false"
boundaryCondition="false" constant="false"
fbc:chemicalFormula="C87H139N7O23P2"/>
      <species metaid="M_uama_c" id="M_uama_c"
name="UDP__N__acetylmuramoyl__L__alanine" compartment="c" charge="-3"
initialConcentration="0" hasOnlySubstanceUnits="false"
boundaryCondition="false" constant="false"
fbc:chemicalFormula="C23H33N4O20P2"/>
      <species metaid="M_uamag_c" id="M_uamag_c"
name="UDP__N__acetylmuramoyl__L__alanyl__D__glutamate" compartment="c"
charge="-4" initialConcentration="0" hasOnlySubstanceUnits="false"
boundaryCondition="false" constant="false"
fbc:chemicalFormula="C28H39N5O23P2"/>
      <species metaid="M_uamagl_c" id="M_uamagl_c"
name="UDP__N__acetyl__alpha__D__muramoyl__L__alanyl__gamma__D__glutamyl__L__l
ysine" compartment="c" charge="-4" initialConcentration="0"
hasOnlySubstanceUnits="false" boundaryCondition="false" constant="false"
fbc:chemicalFormula="C34H52N7O24P2"/>
      <species metaid="M_uamaglaa_c" id="M_uamaglaa_c"
name="UDP__N__acetyl__alpha__D__muramoyl__L__alanyl__gamma__D__glutamyl__L__l
ysyl__D__alanyl__D__alanine" compartment="c" charge="-4"
initialConcentration="0" hasOnlySubstanceUnits="false"
boundaryCondition="false" constant="false"
fbc:chemicalFormula="C40H62N9O26P2"/>
      <species metaid="M_uamr_c" id="M_uamr_c" name="UDP__N__acetylmuramate"
compartment="c" charge="-3" initialConcentration="0"
hasOnlySubstanceUnits="false" boundaryCondition="false" constant="false"
fbc:chemicalFormula="C20H28N3O19P2"/>
      <species metaid="M_ubpeth_c" id="M_ubpeth_c"
name="Unbranched_phosphatidylethanolamine" compartment="c" charge="0"
initialConcentration="0" hasOnlySubstanceUnits="false"
boundaryCondition="false" constant="false" fbc:chemicalFormula=""/>
```

<species metaid="M_udcp_c" id="M_udcp_c" name="Undecaprenol"
compartment="c" charge="0" initialConcentration="0"
hasOnlySubstanceUnits="false" boundaryCondition="false" constant="false"
fbc:chemicalFormula="C55H90O"/>
<species metaid="M_udcpdp_c" id="M_udcpdp_c" name="Undecaprenyl
diphosphate" compartment="c" charge="-3" initialConcentration="0"
hasOnlySubstanceUnits="false" boundaryCondition="false" constant="false"
fbc:chemicalFormula="C55H89O7P2"/>
<species metaid="M_udcpp_c" id="M_udcpp_c" name="Undecaprenyl
phosphate" compartment="c" charge="-2" initialConcentration="0"
hasOnlySubstanceUnits="false" boundaryCondition="false" constant="false"
fbc:chemicalFormula="C55H89O4P"/>
<species metaid="M_udp_c" id="M_udp_c" name="UDP" compartment="c"
charge="-3" initialConcentration="0" hasOnlySubstanceUnits="false"
boundaryCondition="false" constant="false"
fbc:chemicalFormula="C9H11N2O12P2"/>
<species metaid="M_udpacgal_c" id="M_udpacgal_c"
name="UDP__N__acetyl__D__galactosamine" compartment="c" charge="-2"
initialConcentration="0" hasOnlySubstanceUnits="false"
boundaryCondition="false" constant="false"
fbc:chemicalFormula="C17H25N3O17P2"/>
<species metaid="M_udpg_c" id="M_udpg_c" name="UDPglucose"
compartment="c" charge="-2" initialConcentration="0"
hasOnlySubstanceUnits="false" boundaryCondition="false" constant="false"
fbc:chemicalFormula="C15H22N2O17P2"/>
<species metaid="M_udpgal_c" id="M_udpgal_c" name="UDPgalactose"
compartment="c" charge="-2" initialConcentration="0"
hasOnlySubstanceUnits="false" boundaryCondition="false" constant="false"
fbc:chemicalFormula="C15H22N2O17P2"/>
<species metaid="M_udpglcur_c" id="M_udpglcur_c"
name="UDP__D__glucuronate" compartment="c" charge="-3"
initialConcentration="0" hasOnlySubstanceUnits="false"
boundaryCondition="false" constant="false"
fbc:chemicalFormula="C15H19N2O18P2"/>
<species metaid="M_uGgl_c" id="M_uGgl_c"
name="UDP__N__acetylmuramoyl__L__alanyl__gamma__D__glutamyl__L__lysine"
compartment="c" charge="-3" initialConcentration="0"
hasOnlySubstanceUnits="false" boundaryCondition="false" constant="false"
fbc:chemicalFormula="C34H52N7O24P2"/>
<species metaid="M_uGgla_c" id="M_uGgla_c"
name="UDP__N__acetylmuramoyl__L__alanyl__gamma__D__glutamyl__L__lysyl__D__ala
nyl__D__alanine" compartment="c" charge="-3" initialConcentration="0"
hasOnlySubstanceUnits="false" boundaryCondition="false" constant="false"
fbc:chemicalFormula="C40H62N9O26P2"/>
<species metaid="M_ugmd_c" id="M_ugmd_c"
name="UDP__N__acetylmuramoyl__L__alanyl__D__gamma__glutamyl__meso__2,6__diami
nopimelate" compartment="c" charge="-4" initialConcentration="0"
hasOnlySubstanceUnits="false" boundaryCondition="false" constant="false"
fbc:chemicalFormula="C35H51N7O26P2"/>
<species metaid="M_ugmda_c" id="M_ugmda_c"
name="UDP__N__acetylmuramoyl__L__alanyl__D__glutamyl__meso__2,6__diaminopimel
oyl__D__alanyl__D__alanine" compartment="c" charge="-4"
initialConcentration="0" hasOnlySubstanceUnits="false"
boundaryCondition="false" constant="false"
fbc:chemicalFormula="C41H61N9O28P2"/>
<species metaid="M_ump_c" id="M_ump_c" name="UMP" compartment="c"
charge="-2" initialConcentration="0" hasOnlySubstanceUnits="false"
boundaryCondition="false" constant="false" fbc:chemicalFormula="C9H11N2O9P"/>

<species metaid="M_unaga_c" id="M_unaga_c" name="Undecaprenyl diphospho N__acetyl__glucosamine" compartment="c" charge="-2" initialConcentration="0" hasOnlySubstanceUnits="false" boundaryCondition="false" constant="false" fbc:chemicalFormula="C63H103NO12P2"/>
<species metaid="M_updpamaglaa_c" id="M_updpamaglaa_c" name="undecaprenyl__diphospho__N__acetylmuramoyl__L__alanyl__gamma__D__glutamyl__L__lysyl__ D__alanyl__D__alanine" compartment="c" charge="-4" initialConcentration="0" hasOnlySubstanceUnits="false" boundaryCondition="false" constant="false" fbc:chemicalFormula="C86H140N7O21P2"/>
<species metaid="M_uppg1_c" id="M_uppg1_c" name="Uroporphyrinogen I" compartment="c" charge="-8" initialConcentration="0" hasOnlySubstanceUnits="false" boundaryCondition="false" constant="false" fbc:chemicalFormula="C40H36N4O16"/>
<species metaid="M_uppg3_c" id="M_uppg3_c" name="Uroporphyrinogen III" compartment="c" charge="-8" initialConcentration="0" hasOnlySubstanceUnits="false" boundaryCondition="false" constant="false" fbc:chemicalFormula="C40H36N4O16"/>
<species metaid="M_ura_c" id="M_ura_c" name="Uracil" compartment="c" charge="0" initialConcentration="0" hasOnlySubstanceUnits="false" boundaryCondition="false" constant="false" fbc:chemicalFormula="C4H4N2O2"/>
<species metaid="M_ura_e" id="M_ura_e" name="Uracil_b" compartment="e" charge="0" initialConcentration="0" hasOnlySubstanceUnits="false" boundaryCondition="true" constant="false" fbc:chemicalFormula="C4H4N2O2"/>
<species metaid="M_urcan_c" id="M_urcan_c" name="Urocanate" compartment="c" charge="-1" initialConcentration="0" hasOnlySubstanceUnits="false" boundaryCondition="false" constant="false" fbc:chemicalFormula="C6H5N2O2"/>
<species metaid="M_urea_c" id="M_urea_c" name="Urea" compartment="c" charge="0" initialConcentration="0" hasOnlySubstanceUnits="false" boundaryCondition="false" constant="false" fbc:chemicalFormula="CH4N2O"/>
<species metaid="M_urea_e" id="M_urea_e" name="Urea_b" compartment="e" charge="0" initialConcentration="0" hasOnlySubstanceUnits="false" boundaryCondition="true" constant="false" fbc:chemicalFormula="CH4N2O"/>
<species metaid="M_uri_c" id="M_uri_c" name="Uridine" compartment="c" charge="0" initialConcentration="0" hasOnlySubstanceUnits="false" boundaryCondition="false" constant="false" fbc:chemicalFormula="C9H12N2O6"/>
<species metaid="M_uri_e" id="M_uri_e" name="Uridine_b" compartment="e" charge="0" initialConcentration="0" hasOnlySubstanceUnits="false" boundaryCondition="true" constant="false" fbc:chemicalFormula="C9H12N2O6"/>
<species metaid="M_utp_c" id="M_utp_c" name="UTP" compartment="c" charge="-4" initialConcentration="0" hasOnlySubstanceUnits="false" boundaryCondition="false" constant="false" fbc:chemicalFormula="C9H11N2O15P3"/>
<species metaid="M_val__L_c" id="M_val__L_c" name="L__Valine" compartment="c" charge="0" initialConcentration="0" hasOnlySubstanceUnits="false" boundaryCondition="false" constant="false" fbc:chemicalFormula="C5H11NO2"/>
<species metaid="M_val__L_e" id="M_val__L_e" name="L__Valine_b" compartment="e" charge="0" initialConcentration="0" hasOnlySubstanceUnits="false" boundaryCondition="true" constant="false" fbc:chemicalFormula="C5H11NO2"/>
<species metaid="M_WTA40r_ala_c" id="M_WTA40r_ala_c" name="0.0" compartment="c" charge="18" initialConcentration="0" hasOnlySubstanceUnits="false" boundaryCondition="false" constant="false" fbc:chemicalFormula="C266H532N20O310P42"/>
<species metaid="M_WTA40r_c" id="M_WTA40r_c" name="(Rbo__P)40__[2__(alphaGlcNac)__Rbo__P]__Gro__P__Gro__P__ManNAc__GlcNAc_

_PP__undecaprenol" compartment="c" charge="-2" initialConcentration="0" hasOnlySubstanceUnits="false" boundaryCondition="false" constant="false" fbc:chemicalFormula="C206H412O290P42"/>
<species metaid="M_WTA40r_glcnac_c" id="M_WTA40r_glcnac_c" name="[2__(betaGlcNac)__Rbo__P]n__[2__(alphaGlcNac)__Rbo__P]__Gro__P__Gro__P__ManNAc__GlcNAc__PP__undecaprenol" compartment="c" charge="18" initialConcentration="0" hasOnlySubstanceUnits="false" boundaryCondition="false" constant="false" fbc:chemicalFormula="C366H691N20O390P42"/>
<species metaid="M_WTA40raPG_c" id="M_WTA40raPG_c" name="Peptidoglycan bound wall teichoic acid (alanine charged)" compartment="c" charge="9" initialConcentration="0" hasOnlySubstanceUnits="false" boundaryCondition="false" constant="false" fbc:chemicalFormula="C873H1515N164O590P44"/>
<species metaid="M_WTA40rgPG_c" id="M_WTA40rgPG_c" name="Peptidoglycan bound wall teichoic acid (N__acetyl__glucosamine charged)" compartment="c" charge="9" initialConcentration="0" hasOnlySubstanceUnits="false" boundaryCondition="false" constant="false" fbc:chemicalFormula="C973H1674N164O670P44"/>
<species metaid="M_WTA40rPG_c" id="M_WTA40rPG_c" name="Peptidoglycan bound wall teichoic acid" compartment="c" charge="3" initialConcentration="0" hasOnlySubstanceUnits="false" boundaryCondition="false" constant="false" fbc:chemicalFormula="C813H1395O570P44"/>
<species metaid="M_xan_c" id="M_xan_c" name="Xanthine" compartment="c" charge="0" initialConcentration="0" hasOnlySubstanceUnits="false" boundaryCondition="false" constant="false" fbc:chemicalFormula="C5H4N4O2"/>
<species metaid="M_xan_e" id="M_xan_e" name="Xanthine_b" compartment="e" charge="0" initialConcentration="0" hasOnlySubstanceUnits="false" boundaryCondition="true" constant="false" fbc:chemicalFormula="C5H4N4O2"/>
<species metaid="M_xmp_c" id="M_xmp_c" name="Xanthosine 5'__phosphate" compartment="c" charge="-2" initialConcentration="0" hasOnlySubstanceUnits="false" boundaryCondition="false" constant="false" fbc:chemicalFormula="C10H11N4O9P"/>
<species metaid="M_xtp_c" id="M_xtp_c" name="XTP" compartment="c" charge="-4" initialConcentration="0" hasOnlySubstanceUnits="false" boundaryCondition="false" constant="false" fbc:chemicalFormula="C10H11N4O15P3"/>
<species metaid="M_xtsn_c" id="M_xtsn_c" name="Xanthosine" compartment="c" charge="0" initialConcentration="0" hasOnlySubstanceUnits="false" boundaryCondition="false" constant="false" fbc:chemicalFormula="C10H12N4O6"/>
<species metaid="M_xtsn_e" id="M_xtsn_e" name="Xanthosine_b" compartment="e" charge="0" initialConcentration="0" hasOnlySubstanceUnits="false" boundaryCondition="true" constant="false" fbc:chemicalFormula="C10H12N4O6"/>
<species metaid="M_xu5p__D_c" id="M_xu5p__D_c" name="D__Xylulose 5__phosphate" compartment="c" charge="-1" initialConcentration="0" hasOnlySubstanceUnits="false" boundaryCondition="false" constant="false" fbc:chemicalFormula="C5H9O8P"/>
<species metaid="M_xyl__D_e" id="M_xyl__D_e" name="D__Xylose_b" compartment="e" charge="0" initialConcentration="0" hasOnlySubstanceUnits="false" boundaryCondition="true" constant="false" fbc:chemicalFormula="C5H10O5"/>
<species metaid="M_xylt_c" id="M_xylt_c" name="Xylitol" compartment="c" charge="0" initialConcentration="0" hasOnlySubstanceUnits="false" boundaryCondition="false" constant="false" fbc:chemicalFormula="C5H12O5"/>

```
      <species metaid="M_zn2_c" id="M_zn2_c" name="Zinc" compartment="c"
charge="2" initialConcentration="0" hasOnlySubstanceUnits="false"
boundaryCondition="false" constant="false" fbc:chemicalFormula="Zn"/>
      <species metaid="M_zn2_e" id="M_zn2_e" name="Zinc_b" compartment="e"
charge="2" initialConcentration="0" hasOnlySubstanceUnits="false"
boundaryCondition="true" constant="false" fbc:chemicalFormula="Zn"/>
</listOfSpecies>

<listOfParameters>
      <parameter id="irr_lb" value="0" constant="true"/>
      <parameter id="irr_ub" value="1000" constant="true"/>
      <parameter id="rev_lb" value="-1000" constant="true"/>
      <parameter id="rev_ub" value="1000" constant="true"/>
</listOfParameters>

<listOfReactions>
      <reaction metaid="R_10M3HDAHL" id="R_10M3HDAHL" name="stearyl-ACP acyl-
carrier-protein transferase " reversible="true" fast="false"
fbc:lowerFluxBound="rev_lb" fbc:upperFluxBound="rev_ub">
            <listOfReactants>
                  <speciesReference species="M_coa_c" stoichiometry="1"
constant="true"/>
                  <speciesReference species="M_octACP_c" stoichiometry="1"
constant="true"/>
            </listOfReactants>
            <listOfProducts>
                  <speciesReference species="M_ACP_c" stoichiometry="1"
constant="true"/>
                  <speciesReference species="M_stcoa_c" stoichiometry="1"
constant="true"/>
            </listOfProducts>
            <fbc:geneProductAssociation>
                  <fbc:geneProductRef fbc:geneProduct="SAUSA300_1123"/>
            </fbc:geneProductAssociation>
      </reaction>

      <reaction metaid="R_10M3HDHL" id="R_10M3HDHL" name="10-methyl-3-
hydroxy-undecanoyl-ACP hydro__Lyase " reversible="true" fast="false"
fbc:lowerFluxBound="rev_lb" fbc:upperFluxBound="rev_ub">
            <listOfReactants>
                  <speciesReference species="M_10m3uACP_c" stoichiometry="1"
constant="true"/>
            </listOfReactants>
            <listOfProducts>
                  <speciesReference species="M_h2o_c" stoichiometry="1"
constant="true"/>
                  <speciesReference species="M_10mtu2eACP_c"
stoichiometry="1" constant="true"/>
            </listOfProducts>
            <fbc:geneProductAssociation>
                  <fbc:geneProductRef fbc:geneProduct="SAUSA300_2054"/>
            </fbc:geneProductAssociation>
      </reaction>

      <reaction metaid="R_10M3OACPO" id="R_10M3OACPO" name="10-methyl-3-
oxo__Dodecanoyl-ACP NADP oxidoreductase " reversible="true" fast="false"
fbc:lowerFluxBound="rev_lb" fbc:upperFluxBound="rev_ub">
            <listOfReactants>
```

```
                  <speciesReference species="M_h_c" stoichiometry="1"
constant="true"/>
                  <speciesReference species="M_nadph_c" stoichiometry="1"
constant="true"/>
                  <speciesReference species="M_3oddecACP_c" stoichiometry="1"
constant="true"/>
            </listOfReactants>
            <listOfProducts>
                  <speciesReference species="M_nadp_c" stoichiometry="1"
constant="true"/>
                  <speciesReference species="M_3hoddecACP_c"
stoichiometry="1" constant="true"/>
            </listOfProducts>
            <fbc:geneProductAssociation>
                  <fbc:or>
                        <fbc:geneProductRef fbc:geneProduct="SAUSA300_1124"/>
                        <fbc:geneProductRef fbc:geneProduct="SAUSA300_1173"/>
                  </fbc:or>
            </fbc:geneProductAssociation>
      </reaction>

      <reaction metaid="R_10M3OUO" id="R_10M3OUO" name="10-methyl-3-oxo-
undecanoyl-ACP NADP oxidoreductase " reversible="true" fast="false"
fbc:lowerFluxBound="rev_lb" fbc:upperFluxBound="rev_ub">
            <listOfReactants>
                  <speciesReference species="M_h_c" stoichiometry="1"
constant="true"/>
                  <speciesReference species="M_nadph_c" stoichiometry="1"
constant="true"/>
                  <speciesReference species="M_10m3ouACP_c" stoichiometry="1"
constant="true"/>
            </listOfReactants>
            <listOfProducts>
                  <speciesReference species="M_nadp_c" stoichiometry="1"
constant="true"/>
                  <speciesReference species="M_10m3uACP_c" stoichiometry="1"
constant="true"/>
            </listOfProducts>
            <fbc:geneProductAssociation>
                  <fbc:or>
                        <fbc:geneProductRef fbc:geneProduct="SAUSA300_1124"/>
                        <fbc:geneProductRef fbc:geneProduct="SAUSA300_1173"/>
                  </fbc:or>
            </fbc:geneProductAssociation>
      </reaction>

      <reaction metaid="R_10MDOD" id="R_10MDOD" name="10-methyl__Dodecanoyl-
ACP malonyl-acyl-carrier-protein C-acyltransferase decarboxylating "
reversible="false" fast="false" fbc:lowerFluxBound="irr_lb"
fbc:upperFluxBound="irr_ub">
            <listOfReactants>
                  <speciesReference species="M_h_c" stoichiometry="1"
constant="true"/>
                  <speciesReference species="M_malACP_c" stoichiometry="1"
constant="true"/>
                  <speciesReference species="M_10mdACP_c" stoichiometry="1"
constant="true"/>
            </listOfReactants>
```

```
            <listOfProducts>
                  <speciesReference species="M_ACP_c" stoichiometry="1"
constant="true"/>
                  <speciesReference species="M_co2_c" stoichiometry="1"
constant="true"/>
                  <speciesReference species="M_123oxtACP_c" stoichiometry="1"
constant="true"/>
            </listOfProducts>
            <fbc:geneProductAssociation>
                  <fbc:and>
                        <fbc:geneProductRef fbc:geneProduct="SAUSA300_0886"/>
                        <fbc:geneProductRef fbc:geneProduct="SAUSA300_0885"/>
                  </fbc:and>
            </fbc:geneProductAssociation>
      </reaction>

      <reaction metaid="R_10MTDAO" id="R_10MTDAO" name="10-methyl-
trans__Dodec-2-enoyl-ACP NAD oxidoreductase A-specific " reversible="false"
fast="false" fbc:lowerFluxBound="irr_lb" fbc:upperFluxBound="irr_ub">
            <listOfReactants>
                  <speciesReference species="M_h_c" stoichiometry="1"
constant="true"/>
                  <speciesReference species="M_nadh_c" stoichiometry="1"
constant="true"/>
                  <speciesReference species="M_10mtd2eACP_c"
stoichiometry="1" constant="true"/>
            </listOfReactants>
            <listOfProducts>
                  <speciesReference species="M_nad_c" stoichiometry="1"
constant="true"/>
                  <speciesReference species="M_10mdACP_c" stoichiometry="1"
constant="true"/>
            </listOfProducts>
            <fbc:geneProductAssociation>
                  <fbc:geneProductRef fbc:geneProduct="SAUSA300_0912"/>
            </fbc:geneProductAssociation>
      </reaction>

      <reaction metaid="R_10MTU2A" id="R_10MTU2A" name="10-methyl-trans-
undec-2-enoyl-ACP NAD oxidoreductase A-specific " reversible="false"
fast="false" fbc:lowerFluxBound="irr_lb" fbc:upperFluxBound="irr_ub">
            <listOfReactants>
                  <speciesReference species="M_h_c" stoichiometry="1"
constant="true"/>
                  <speciesReference species="M_nadh_c" stoichiometry="1"
constant="true"/>
                  <speciesReference species="M_10mtu2eACP_c"
stoichiometry="1" constant="true"/>
            </listOfReactants>
            <listOfProducts>
                  <speciesReference species="M_nad_c" stoichiometry="1"
constant="true"/>
                  <speciesReference species="M_10muACP_c" stoichiometry="1"
constant="true"/>
            </listOfProducts>
            <fbc:geneProductAssociation>
                  <fbc:geneProductRef fbc:geneProduct="SAUSA300_0912"/>
            </fbc:geneProductAssociation>
```

```
      </reaction>

      <reaction metaid="R_10MUAM" id="R_10MUAM" name="10-methyl-undecanoyl-
ACP malonyl-acyl-carrier-protein C-acyltransferase decarboxylating "
reversible="false" fast="false" fbc:lowerFluxBound="irr_lb"
fbc:upperFluxBound="irr_ub">
            <listOfReactants>
                  <speciesReference species="M_h_c" stoichiometry="1"
constant="true"/>
                  <speciesReference species="M_malACP_c" stoichiometry="1"
constant="true"/>
                  <speciesReference species="M_10muACP_c" stoichiometry="1"
constant="true"/>
            </listOfReactants>
            <listOfProducts>
                  <speciesReference species="M_ACP_c" stoichiometry="1"
constant="true"/>
                  <speciesReference species="M_co2_c" stoichiometry="1"
constant="true"/>
                  <speciesReference species="M_12m3otACP_c" stoichiometry="1"
constant="true"/>
            </listOfProducts>
            <fbc:geneProductAssociation>
                  <fbc:and>
                        <fbc:geneProductRef fbc:geneProduct="SAUSA300_0886"/>
                        <fbc:geneProductRef fbc:geneProduct="SAUSA300_0885"/>
                  </fbc:and>
            </fbc:geneProductAssociation>
      </reaction>

      <reaction metaid="R_11M3ODO" id="R_11M3ODO" name="11-methyl-3-
oxo__Dodecanoyl-ACP NADP oxidoreductase " reversible="true" fast="false"
fbc:lowerFluxBound="rev_lb" fbc:upperFluxBound="rev_ub">
            <listOfReactants>
                  <speciesReference species="M_h_c" stoichiometry="1"
constant="true"/>
                  <speciesReference species="M_nadph_c" stoichiometry="1"
constant="true"/>
                  <speciesReference species="M_11m3odACP_c" stoichiometry="1"
constant="true"/>
            </listOfReactants>
            <listOfProducts>
                  <speciesReference species="M_nadp_c" stoichiometry="1"
constant="true"/>
                  <speciesReference species="M_11m3hdACP_c" stoichiometry="1"
constant="true"/>
            </listOfProducts>
            <fbc:geneProductAssociation>
                  <fbc:or>
                        <fbc:geneProductRef fbc:geneProduct="SAUSA300_1124"/>
                        <fbc:geneProductRef fbc:geneProduct="SAUSA300_1173"/>
                  </fbc:or>
            </fbc:geneProductAssociation>
      </reaction>

      <reaction metaid="R_11MDAMCA" id="R_11MDAMCA" name="11-
methyl__Dodecanoyl-ACP malonyl-acyl-carrier-protein C-acyltransferase
```

```
decarboxylating " reversible="false" fast="false" fbc:lowerFluxBound="irr_lb"
fbc:upperFluxBound="irr_ub">
            <listOfReactants>
                  <speciesReference species="M_h_c" stoichiometry="1"
constant="true"/>
                  <speciesReference species="M_malACP_c" stoichiometry="1"
constant="true"/>
                  <speciesReference species="M_11mdACP_c" stoichiometry="1"
constant="true"/>
            </listOfReactants>
            <listOfProducts>
                  <speciesReference species="M_ACP_c" stoichiometry="1"
constant="true"/>
                  <speciesReference species="M_co2_c" stoichiometry="1"
constant="true"/>
                  <speciesReference species="M_13m3otdACP_c"
stoichiometry="1" constant="true"/>
            </listOfProducts>
            <fbc:geneProductAssociation>
                  <fbc:and>
                        <fbc:geneProductRef fbc:geneProduct="SAUSA300_0886"/>
                        <fbc:geneProductRef fbc:geneProduct="SAUSA300_0885"/>
                  </fbc:and>
            </fbc:geneProductAssociation>
      </reaction>

      <reaction metaid="R_11MHDOD" id="R_11MHDOD" name="11-methyl-3-
hydroxy__Dodecanoyl-ACP hydro__Lyase " reversible="true" fast="false"
fbc:lowerFluxBound="rev_lb" fbc:upperFluxBound="rev_ub">
            <listOfReactants>
                  <speciesReference species="M_11m3hdACP_c" stoichiometry="1"
constant="true"/>
            </listOfReactants>
            <listOfProducts>
                  <speciesReference species="M_h2o_c" stoichiometry="1"
constant="true"/>
                  <speciesReference species="M_11mtdeACP_c" stoichiometry="1"
constant="true"/>
            </listOfProducts>
            <fbc:geneProductAssociation>
                  <fbc:geneProductRef fbc:geneProduct="SAUSA300_2054"/>
            </fbc:geneProductAssociation>
      </reaction>

      <reaction metaid="R_11MTRDOC" id="R_11MTRDOC" name="11-methyl-
trans__Dodec-2-enoyl-ACP NAD oxidoreductase A-specific " reversible="false"
fast="false" fbc:lowerFluxBound="irr_lb" fbc:upperFluxBound="irr_ub">
            <listOfReactants>
                  <speciesReference species="M_h_c" stoichiometry="1"
constant="true"/>
                  <speciesReference species="M_nadh_c" stoichiometry="1"
constant="true"/>
                  <speciesReference species="M_11mtdeACP_c" stoichiometry="1"
constant="true"/>
            </listOfReactants>
            <listOfProducts>
                  <speciesReference species="M_nad_c" stoichiometry="1"
constant="true"/>
```

```
                  <speciesReference species="M_11mdACP_c" stoichiometry="1"
constant="true"/>
            </listOfProducts>
            <fbc:geneProductAssociation>
                  <fbc:geneProductRef fbc:geneProduct="SAUSA300_0912"/>
            </fbc:geneProductAssociation>
      </reaction>

      <reaction metaid="R_12M3HTDH" id="R_12M3HTDH" name="12-methyl-3-
hydroxy-tetra__Decanoyl-ACP hydro__Lyase " reversible="true" fast="false"
fbc:lowerFluxBound="rev_lb" fbc:upperFluxBound="rev_ub">
            <listOfReactants>
                  <speciesReference species="M_12m3htdACP_c"
stoichiometry="1" constant="true"/>
            </listOfReactants>
            <listOfProducts>
                  <speciesReference species="M_h2o_c" stoichiometry="1"
constant="true"/>
                  <speciesReference species="M_12methtetdec2_c"
stoichiometry="1" constant="true"/>
            </listOfProducts>
            <fbc:geneProductAssociation>
                  <fbc:geneProductRef fbc:geneProduct="SAUSA300_2054"/>
            </fbc:geneProductAssociation>
      </reaction>

      <reaction metaid="R_12M3HTHL" id="R_12M3HTHL" name="12-methyl-3-
hydroxy-tridecanoyl-ACP hydro__Lyase " reversible="true" fast="false"
fbc:lowerFluxBound="rev_lb" fbc:upperFluxBound="rev_ub">
            <listOfReactants>
                  <speciesReference species="M_12mdhtACP_c" stoichiometry="1"
constant="true"/>
            </listOfReactants>
            <listOfProducts>
                  <speciesReference species="M_h2o_c" stoichiometry="1"
constant="true"/>
                  <speciesReference species="M_12mtt2eACP_c"
stoichiometry="1" constant="true"/>
            </listOfProducts>
            <fbc:geneProductAssociation>
                  <fbc:geneProductRef fbc:geneProduct="SAUSA300_2054"/>
            </fbc:geneProductAssociation>
      </reaction>

      <reaction metaid="R_12M3OXOT" id="R_12M3OXOT" name="12-methyl-3-oxo-
tridecanoyl-ACP NADP oxidoreductase " reversible="true" fast="false"
fbc:lowerFluxBound="rev_lb" fbc:upperFluxBound="rev_ub">
            <listOfReactants>
                  <speciesReference species="M_h_c" stoichiometry="1"
constant="true"/>
                  <speciesReference species="M_nadph_c" stoichiometry="1"
constant="true"/>
                  <speciesReference species="M_12m3otACP_c" stoichiometry="1"
constant="true"/>
            </listOfReactants>
            <listOfProducts>
                  <speciesReference species="M_nadp_c" stoichiometry="1"
constant="true"/>
```

```
                  <speciesReference species="M_12mdhtACP_c" stoichiometry="1"
constant="true"/>
            </listOfProducts>
            <fbc:geneProductAssociation>
                  <fbc:or>
                        <fbc:geneProductRef fbc:geneProduct="SAUSA300_1124"/>
                        <fbc:geneProductRef fbc:geneProduct="SAUSA300_1173"/>
                  </fbc:or>
            </fbc:geneProductAssociation>
      </reaction>

      <reaction metaid="R_12M3TDA" id="R_12M3TDA" name="12-methyl-3-oxo-
tetra__Decanoyl-ACP NADP oxidoreductase " reversible="true" fast="false"
fbc:lowerFluxBound="rev_lb" fbc:upperFluxBound="rev_ub">
            <listOfReactants>
                  <speciesReference species="M_h_c" stoichiometry="1"
constant="true"/>
                  <speciesReference species="M_nadph_c" stoichiometry="1"
constant="true"/>
                  <speciesReference species="M_123oxtACP_c" stoichiometry="1"
constant="true"/>
            </listOfReactants>
            <listOfProducts>
                  <speciesReference species="M_nadp_c" stoichiometry="1"
constant="true"/>
                  <speciesReference species="M_12m3htdACP_c"
stoichiometry="1" constant="true"/>
            </listOfProducts>
            <fbc:geneProductAssociation>
                  <fbc:or>
                        <fbc:geneProductRef fbc:geneProduct="SAUSA300_1124"/>
                        <fbc:geneProductRef fbc:geneProduct="SAUSA300_1173"/>
                  </fbc:or>
            </fbc:geneProductAssociation>
      </reaction>

      <reaction metaid="R_12MEDAT" id="R_12MEDAT" name="12-methyl-
tetra__Decanoyl-ACP acyl-carrier-protein transferase " reversible="true"
fast="false" fbc:lowerFluxBound="rev_lb" fbc:upperFluxBound="rev_ub">
            <listOfReactants>
                  <speciesReference species="M_12methedec_c"
stoichiometry="1" constant="true"/>
                  <speciesReference species="M_coa_c" stoichiometry="1"
constant="true"/>
            </listOfReactants>
            <listOfProducts>
                  <speciesReference species="M_ACP_c" stoichiometry="1"
constant="true"/>
                  <speciesReference species="M_fa4coa_c" stoichiometry="1"
constant="true"/>
            </listOfProducts>
            <fbc:geneProductAssociation>
                  <fbc:geneProductRef fbc:geneProduct="SAUSA300_1123"/>
            </fbc:geneProductAssociation>
      </reaction>
```

```
      <reaction metaid="R_12METRAT" id="R_12METRAT" name="12-methyl-
tridecanoyl-ACP acyl-carrier-protein transferase " reversible="true"
fast="false" fbc:lowerFluxBound="rev_lb" fbc:upperFluxBound="rev_ub">
            <listOfReactants>
                  <speciesReference species="M_coa_c" stoichiometry="1"
constant="true"/>
                  <speciesReference species="M_12mtACP_c" stoichiometry="1"
constant="true"/>
            </listOfReactants>
            <listOfProducts>
                  <speciesReference species="M_ACP_c" stoichiometry="1"
constant="true"/>
                  <speciesReference species="M_fa1coa_c" stoichiometry="1"
constant="true"/>
            </listOfProducts>
            <fbc:geneProductAssociation>
                  <fbc:geneProductRef fbc:geneProduct="SAUSA300_1123"/>
            </fbc:geneProductAssociation>
      </reaction>

      <reaction metaid="R_12MTACPM" id="R_12MTACPM" name="12-methyl-
tridecanoyl-ACP malonyl-acyl-carrier-protein C-acyltransferase
decarboxylating " reversible="false" fast="false" fbc:lowerFluxBound="irr_lb"
fbc:upperFluxBound="irr_ub">
            <listOfReactants>
                  <speciesReference species="M_h_c" stoichiometry="1"
constant="true"/>
                  <speciesReference species="M_malACP_c" stoichiometry="1"
constant="true"/>
                  <speciesReference species="M_12mtACP_c" stoichiometry="1"
constant="true"/>
            </listOfReactants>
            <listOfProducts>
                  <speciesReference species="M_ACP_c" stoichiometry="1"
constant="true"/>
                  <speciesReference species="M_co2_c" stoichiometry="1"
constant="true"/>
                  <speciesReference species="M_14m3opACP_c" stoichiometry="1"
constant="true"/>
            </listOfProducts>
            <fbc:geneProductAssociation>
                  <fbc:and>
                        <fbc:geneProductRef fbc:geneProduct="SAUSA300_0886"/>
                        <fbc:geneProductRef fbc:geneProduct="SAUSA300_0885"/>
                  </fbc:and>
            </fbc:geneProductAssociation>
      </reaction>

      <reaction metaid="R_12MTT2E" id="R_12MTT2E" name="12-methyl-trans-
tridec-2-enoyl-ACP NAD oxidoreductase A-specific " reversible="false"
fast="false" fbc:lowerFluxBound="irr_lb" fbc:upperFluxBound="irr_ub">
            <listOfReactants>
                  <speciesReference species="M_h_c" stoichiometry="1"
constant="true"/>
                  <speciesReference species="M_nadh_c" stoichiometry="1"
constant="true"/>
                  <speciesReference species="M_12mtt2eACP_c"
stoichiometry="1" constant="true"/>
```

```
            &lt;/listOfReactants&gt;
            &lt;listOfProducts&gt;
                  <speciesReference species="M_nad_c" stoichiometry="1"
constant="true"/>
                  <speciesReference species="M_12mtACP_c" stoichiometry="1"
constant="true"/>
            &lt;/listOfProducts&gt;
            &lt;fbc:geneProductAssociation&gt;
                  &lt;fbc:geneProductRef fbc:geneProduct="SAUSA300_0912"/&gt;
            &lt;/fbc:geneProductAssociation&gt;
      &lt;/reaction&gt;

      <reaction metaid="R_12TTACA" id="R_12TTACA" name="12-methyl-trans-
tetra__Dec-2-enoyl-ACP NAD oxidoreductase A-specific " reversible="false"
fast="false" fbc:lowerFluxBound="irr_lb" fbc:upperFluxBound="irr_ub">
            &lt;listOfReactants&gt;
                  <speciesReference species="M_h_c" stoichiometry="1"
constant="true"/>
                  <speciesReference species="M_12methtetdec2_c"
stoichiometry="1" constant="true"/>
                  <speciesReference species="M_nadh_c" stoichiometry="1"
constant="true"/>
            &lt;/listOfReactants&gt;
            &lt;listOfProducts&gt;
                  <speciesReference species="M_12methedec_c"
stoichiometry="1" constant="true"/>
                  <speciesReference species="M_nad_c" stoichiometry="1"
constant="true"/>
            &lt;/listOfProducts&gt;
            &lt;fbc:geneProductAssociation&gt;
                  &lt;fbc:geneProductRef fbc:geneProduct="SAUSA300_0912"/&gt;
            &lt;/fbc:geneProductAssociation&gt;
      &lt;/reaction&gt;

      <reaction metaid="R_13M3HTDAHL" id="R_13M3HTDAHL" name="13-methyl-3-
hydroxy-tetra__Decanoyl-ACP hydro__Lyase " reversible="true" fast="false"
fbc:lowerFluxBound="rev_lb" fbc:upperFluxBound="rev_ub">
            &lt;listOfReactants&gt;
                  <speciesReference species="M_13m3htdACP_c"
stoichiometry="1" constant="true"/>
            &lt;/listOfReactants&gt;
            &lt;listOfProducts&gt;
                  <speciesReference species="M_h2o_c" stoichiometry="1"
constant="true"/>
                  <speciesReference species="M_13mttd2eACP_c"
stoichiometry="1" constant="true"/>
            &lt;/listOfProducts&gt;
            &lt;fbc:geneProductAssociation&gt;
                  &lt;fbc:geneProductRef fbc:geneProduct="SAUSA300_2054"/&gt;
            &lt;/fbc:geneProductAssociation&gt;
      &lt;/reaction&gt;

      <reaction metaid="R_13M3OTDAO" id="R_13M3OTDAO" name="13-methyl-3-oxo-
tetra__Decanoyl-ACP NADP oxidoreductase " reversible="true" fast="false"
fbc:lowerFluxBound="rev_lb" fbc:upperFluxBound="rev_ub">
            &lt;listOfReactants&gt;
                  <speciesReference species="M_h_c" stoichiometry="1"
constant="true"/>
```

```
                  <speciesReference species="M_nadph_c" stoichiometry="1"
constant="true"/>
                  <speciesReference species="M_13m3otdACP_c"
stoichiometry="1" constant="true"/>
            </listOfReactants>
            <listOfProducts>
                  <speciesReference species="M_nadp_c" stoichiometry="1"
constant="true"/>
                  <speciesReference species="M_13m3htdACP_c"
stoichiometry="1" constant="true"/>
            </listOfProducts>
            <fbc:geneProductAssociation>
                  <fbc:or>
                        <fbc:geneProductRef fbc:geneProduct="SAUSA300_1124"/>
                        <fbc:geneProductRef fbc:geneProduct="SAUSA300_1173"/>
                  </fbc:or>
            </fbc:geneProductAssociation>
      </reaction>

      <reaction metaid="R_13MTDAM" id="R_13MTDAM" name="13-methyl-
tetra__Decanoyl-ACP malonyl-acyl-carrier-protein C-acyltransferase
decarboxylating " reversible="false" fast="false" fbc:lowerFluxBound="irr_lb"
fbc:upperFluxBound="irr_ub">
            <listOfReactants>
                  <speciesReference species="M_h_c" stoichiometry="1"
constant="true"/>
                  <speciesReference species="M_malACP_c" stoichiometry="1"
constant="true"/>
                  <speciesReference species="M_13mtdACP_c" stoichiometry="1"
constant="true"/>
            </listOfReactants>
            <listOfProducts>
                  <speciesReference species="M_ACP_c" stoichiometry="1"
constant="true"/>
                  <speciesReference species="M_co2_c" stoichiometry="1"
constant="true"/>
                  <speciesReference species="M_15m3ohdACP_c"
stoichiometry="1" constant="true"/>
            </listOfProducts>
            <fbc:geneProductAssociation>
                  <fbc:and>
                        <fbc:geneProductRef fbc:geneProduct="SAUSA300_0886"/>
                        <fbc:geneProductRef fbc:geneProduct="SAUSA300_0885"/>
                  </fbc:and>
            </fbc:geneProductAssociation>
      </reaction>

      <reaction metaid="R_13MTDAT" id="R_13MTDAT" name="13-methyl-
tetra__Decanoyl-ACP acyl-carrier-protein transferase " reversible="true"
fast="false" fbc:lowerFluxBound="rev_lb" fbc:upperFluxBound="rev_ub">
            <listOfReactants>
                  <speciesReference species="M_coa_c" stoichiometry="1"
constant="true"/>
                  <speciesReference species="M_13mtdACP_c" stoichiometry="1"
constant="true"/>
            </listOfReactants>
            <listOfProducts>
```

```
                  <speciesReference species="M_ACP_c" stoichiometry="1"
constant="true"/>
                  <speciesReference species="M_fa3coa_c" stoichiometry="1"
constant="true"/>
            </listOfProducts>
            <fbc:geneProductAssociation>
                  <fbc:geneProductRef fbc:geneProduct="SAUSA300_1123"/>
            </fbc:geneProductAssociation>
      </reaction>

      <reaction metaid="R_13MTTDAO" id="R_13MTTDAO" name="13-methyl-trans-
tetra__Dec-2-enoyl-ACP NAD oxidoreductase A-specific " reversible="false"
fast="false" fbc:lowerFluxBound="irr_lb" fbc:upperFluxBound="irr_ub">
            <listOfReactants>
                  <speciesReference species="M_h_c" stoichiometry="1"
constant="true"/>
                  <speciesReference species="M_nadh_c" stoichiometry="1"
constant="true"/>
                  <speciesReference species="M_13mttd2eACP_c"
stoichiometry="1" constant="true"/>
            </listOfReactants>
            <listOfProducts>
                  <speciesReference species="M_nad_c" stoichiometry="1"
constant="true"/>
                  <speciesReference species="M_13mtdACP_c" stoichiometry="1"
constant="true"/>
            </listOfProducts>
            <fbc:geneProductAssociation>
                  <fbc:geneProductRef fbc:geneProduct="SAUSA300_0912"/>
            </fbc:geneProductAssociation>
      </reaction>

      <reaction metaid="R_14DH2NOT" id="R_14DH2NOT" name="rxn04673 "
reversible="false" fast="false" fbc:lowerFluxBound="irr_lb"
fbc:upperFluxBound="irr_ub">
            <listOfReactants>
                  <speciesReference species="M_dhna_c" stoichiometry="1"
constant="true"/>
                  <speciesReference species="M_phydp_c" stoichiometry="1"
constant="true"/>
            </listOfReactants>
            <listOfProducts>
                  <speciesReference species="M_h_c" stoichiometry="1"
constant="true"/>
                  <speciesReference species="M_ppi_c" stoichiometry="1"
constant="true"/>
                  <speciesReference species="M_co2_c" stoichiometry="1"
constant="true"/>
                  <speciesReference species="M_23dmphol_c" stoichiometry="1"
constant="true"/>
            </listOfProducts>
            <fbc:geneProductAssociation>
                  <fbc:geneProductRef fbc:geneProduct="SAUSA300_0944"/>
            </fbc:geneProductAssociation>
      </reaction>
```

```
      <reaction metaid="R_14M3HDEC" id="R_14M3HDEC" name="14-methyl-3-
hydroxy-hexa__Decanoyl-ACP hydro__Lyase " reversible="true" fast="false"
fbc:lowerFluxBound="rev_lb" fbc:upperFluxBound="rev_ub">
            <listOfReactants>
                  <speciesReference species="M_14m3hdACP_c" stoichiometry="1"
constant="true"/>
            </listOfReactants>
            <listOfProducts>
                  <speciesReference species="M_h2o_c" stoichiometry="1"
constant="true"/>
                  <speciesReference species="M_14mthdeACP_c"
stoichiometry="1" constant="true"/>
            </listOfProducts>
            <fbc:geneProductAssociation>
                  <fbc:geneProductRef fbc:geneProduct="SAUSA300_2054"/>
            </fbc:geneProductAssociation>
      </reaction>

      <reaction metaid="R_14M3HPAHL" id="R_14M3HPAHL" name="14-methyl-3-
hydroxy-pentadecanoyl-ACP hydro__Lyase " reversible="true" fast="false"
fbc:lowerFluxBound="rev_lb" fbc:upperFluxBound="rev_ub">
            <listOfReactants>
                  <speciesReference species="M_14m3hpACP_c" stoichiometry="1"
constant="true"/>
            </listOfReactants>
            <listOfProducts>
                  <speciesReference species="M_h2o_c" stoichiometry="1"
constant="true"/>
                  <speciesReference species="M_14mtp2eACP_c"
stoichiometry="1" constant="true"/>
            </listOfProducts>
            <fbc:geneProductAssociation>
                  <fbc:geneProductRef fbc:geneProduct="SAUSA300_2054"/>
            </fbc:geneProductAssociation>
      </reaction>

      <reaction metaid="R_14M3OHO" id="R_14M3OHO" name="14-methyl-3-oxo-
hexa__Decanoyl-ACP NADP oxidoreductase " reversible="true" fast="false"
fbc:lowerFluxBound="rev_lb" fbc:upperFluxBound="rev_ub">
            <listOfReactants>
                  <speciesReference species="M_h_c" stoichiometry="1"
constant="true"/>
                  <speciesReference species="M_nadph_c" stoichiometry="1"
constant="true"/>
                  <speciesReference species="M_14m3ohdACP_c"
stoichiometry="1" constant="true"/>
            </listOfReactants>
            <listOfProducts>
                  <speciesReference species="M_nadp_c" stoichiometry="1"
constant="true"/>
                  <speciesReference species="M_14m3hdACP_c" stoichiometry="1"
constant="true"/>
            </listOfProducts>
            <fbc:geneProductAssociation>
                  <fbc:or>
                        <fbc:geneProductRef fbc:geneProduct="SAUSA300_1124"/>
                        <fbc:geneProductRef fbc:geneProduct="SAUSA300_1173"/>
                  </fbc:or>
```

```
            </fbc:geneProductAssociation>
      </reaction>

      <reaction metaid="R_14M3OP" id="R_14M3OP" name="14-methyl-3-oxo-
pentadecanoyl-ACP NADP oxidoreductase " reversible="true" fast="false"
fbc:lowerFluxBound="rev_lb" fbc:upperFluxBound="rev_ub">
            <listOfReactants>
                  <speciesReference species="M_h_c" stoichiometry="1"
constant="true"/>
                  <speciesReference species="M_nadph_c" stoichiometry="1"
constant="true"/>
                  <speciesReference species="M_14m3opACP_c" stoichiometry="1"
constant="true"/>
            </listOfReactants>
            <listOfProducts>
                  <speciesReference species="M_nadp_c" stoichiometry="1"
constant="true"/>
                  <speciesReference species="M_14m3hpACP_c" stoichiometry="1"
constant="true"/>
            </listOfProducts>
            <fbc:geneProductAssociation>
                  <fbc:or>
                        <fbc:geneProductRef fbc:geneProduct="SAUSA300_1124"/>
                        <fbc:geneProductRef fbc:geneProduct="SAUSA300_1173"/>
                  </fbc:or>
            </fbc:geneProductAssociation>
      </reaction>

      <reaction metaid="R_14MHDAT" id="R_14MHDAT" name="14-methyl-
hexa__Decanoyl-ACP acyl-carrier-protein transferase " reversible="true"
fast="false" fbc:lowerFluxBound="rev_lb" fbc:upperFluxBound="rev_ub">
            <listOfReactants>
                  <speciesReference species="M_coa_c" stoichiometry="1"
constant="true"/>
                  <speciesReference species="M_14mhdACP_c" stoichiometry="1"
constant="true"/>
            </listOfReactants>
            <listOfProducts>
                  <speciesReference species="M_ACP_c" stoichiometry="1"
constant="true"/>
                  <speciesReference species="M_fa12coa_c" stoichiometry="1"
constant="true"/>
            </listOfProducts>
            <fbc:geneProductAssociation>
                  <fbc:geneProductRef fbc:geneProduct="SAUSA300_1123"/>
            </fbc:geneProductAssociation>
      </reaction>

      <reaction metaid="R_14MPACPT" id="R_14MPACPT" name="14-methyl-
pentadecanoyl-ACP acyl-carrier-protein transferase " reversible="true"
fast="false" fbc:lowerFluxBound="rev_lb" fbc:upperFluxBound="rev_ub">
            <listOfReactants>
                  <speciesReference species="M_coa_c" stoichiometry="1"
constant="true"/>
                  <speciesReference species="M_14mpACP_c" stoichiometry="1"
constant="true"/>
            </listOfReactants>
            <listOfProducts>
```

```
                  <speciesReference species="M_ACP_c" stoichiometry="1"
constant="true"/>
                  <speciesReference species="M_fa6coa_c" stoichiometry="1"
constant="true"/>
            </listOfProducts>
            <fbc:geneProductAssociation>
                  <fbc:geneProductRef fbc:geneProduct="SAUSA300_1123"/>
            </fbc:geneProductAssociation>
      </reaction>

      <reaction metaid="R_14MTHEAO" id="R_14MTHEAO" name="14-methyl-trans-
hexa__Dec-2-enoyl-ACP NAD oxidoreductase A-specific " reversible="false"
fast="false" fbc:lowerFluxBound="irr_lb" fbc:upperFluxBound="irr_ub">
            <listOfReactants>
                  <speciesReference species="M_h_c" stoichiometry="1"
constant="true"/>
                  <speciesReference species="M_nadh_c" stoichiometry="1"
constant="true"/>
                  <speciesReference species="M_14mthdeACP_c"
stoichiometry="1" constant="true"/>
            </listOfReactants>
            <listOfProducts>
                  <speciesReference species="M_nad_c" stoichiometry="1"
constant="true"/>
                  <speciesReference species="M_14mhdACP_c" stoichiometry="1"
constant="true"/>
            </listOfProducts>
            <fbc:geneProductAssociation>
                  <fbc:geneProductRef fbc:geneProduct="SAUSA300_0912"/>
            </fbc:geneProductAssociation>
      </reaction>

      <reaction metaid="R_14MTP2EA" id="R_14MTP2EA" name="14-methyl-trans-
pentadec-2-enoyl-ACP NAD oxidoreductase A-specific " reversible="false"
fast="false" fbc:lowerFluxBound="irr_lb" fbc:upperFluxBound="irr_ub">
            <listOfReactants>
                  <speciesReference species="M_h_c" stoichiometry="1"
constant="true"/>
                  <speciesReference species="M_nadh_c" stoichiometry="1"
constant="true"/>
                  <speciesReference species="M_14mtp2eACP_c"
stoichiometry="1" constant="true"/>
            </listOfReactants>
            <listOfProducts>
                  <speciesReference species="M_nad_c" stoichiometry="1"
constant="true"/>
                  <speciesReference species="M_14mpACP_c" stoichiometry="1"
constant="true"/>
            </listOfProducts>
            <fbc:geneProductAssociation>
                  <fbc:geneProductRef fbc:geneProduct="SAUSA300_0912"/>
            </fbc:geneProductAssociation>
      </reaction>

      <reaction metaid="R_15M3HEXDA" id="R_15M3HEXDA" name="15-methyl-3-
hydroxy-hexa__Decanoyl-ACP hydro__Lyase " reversible="true" fast="false"
fbc:lowerFluxBound="rev_lb" fbc:upperFluxBound="rev_ub">
            <listOfReactants>
```

```
                  <speciesReference species="M_15m3hdACP_c" stoichiometry="1"
constant="true"/>
            </listOfReactants>
            <listOfProducts>
                  <speciesReference species="M_h2o_c" stoichiometry="1"
constant="true"/>
                  <speciesReference species="M_15methexeACP_c"
stoichiometry="1" constant="true"/>
            </listOfProducts>
            <fbc:geneProductAssociation>
                  <fbc:geneProductRef fbc:geneProduct="SAUSA300_2054"/>
            </fbc:geneProductAssociation>
      </reaction>

      <reaction metaid="R_15M3OHAO" id="R_15M3OHAO" name="15-methyl-3-oxo-
hexa__Decanoyl-ACP NADP oxidoreductase " reversible="true" fast="false"
fbc:lowerFluxBound="rev_lb" fbc:upperFluxBound="rev_ub">
            <listOfReactants>
                  <speciesReference species="M_h_c" stoichiometry="1"
constant="true"/>
                  <speciesReference species="M_nadph_c" stoichiometry="1"
constant="true"/>
                  <speciesReference species="M_15m3ohdACP_c"
stoichiometry="1" constant="true"/>
            </listOfReactants>
            <listOfProducts>
                  <speciesReference species="M_nadp_c" stoichiometry="1"
constant="true"/>
                  <speciesReference species="M_15m3hdACP_c" stoichiometry="1"
constant="true"/>
            </listOfProducts>
            <fbc:geneProductAssociation>
                  <fbc:or>
                        <fbc:geneProductRef fbc:geneProduct="SAUSA300_1124"/>
                        <fbc:geneProductRef fbc:geneProduct="SAUSA300_1173"/>
                  </fbc:or>
            </fbc:geneProductAssociation>
      </reaction>

      <reaction metaid="R_15MHDAT" id="R_15MHDAT" name="15-methyl-
hexa__Decanoyl-ACP acyl-carrier-protein transferase " reversible="true"
fast="false" fbc:lowerFluxBound="rev_lb" fbc:upperFluxBound="rev_ub">
            <listOfReactants>
                  <speciesReference species="M_coa_c" stoichiometry="1"
constant="true"/>
                  <speciesReference species="M_15mhdACP_c" stoichiometry="1"
constant="true"/>
            </listOfReactants>
            <listOfProducts>
                  <speciesReference species="M_ACP_c" stoichiometry="1"
constant="true"/>
                  <speciesReference species="M_fa11coa_c" stoichiometry="1"
constant="true"/>
            </listOfProducts>
            <fbc:geneProductAssociation>
                  <fbc:geneProductRef fbc:geneProduct="SAUSA300_1123"/>
            </fbc:geneProductAssociation>
      </reaction>
```

```
      <reaction metaid="R_15MTHACP" id="R_15MTHACP" name="15-methyl-trans-
hexa__Dec-2-enoyl-ACP NAD oxidoreductase A-specific " reversible="false"
fast="false" fbc:lowerFluxBound="irr_lb" fbc:upperFluxBound="irr_ub">
            <listOfReactants>
                  <speciesReference species="M_h_c" stoichiometry="1"
constant="true"/>
                  <speciesReference species="M_nadh_c" stoichiometry="1"
constant="true"/>
                  <speciesReference species="M_15methexeACP_c"
stoichiometry="1" constant="true"/>
            </listOfReactants>
            <listOfProducts>
                  <speciesReference species="M_nad_c" stoichiometry="1"
constant="true"/>
                  <speciesReference species="M_15mhdACP_c" stoichiometry="1"
constant="true"/>
            </listOfProducts>
            <fbc:geneProductAssociation>
                  <fbc:geneProductRef fbc:geneProduct="SAUSA300_0912"/>
            </fbc:geneProductAssociation>
      </reaction>

      <reaction metaid="R_23PDE2pp" id="R_23PDE2pp" name="2',3'-cyclic-
nucleotide phosphodiesterase (UMP) (periplasm)" reversible="false"
fast="false" fbc:lowerFluxBound="irr_lb" fbc:upperFluxBound="irr_ub">
            <listOfReactants>
                  <speciesReference species="M_h2o_c" stoichiometry="1"
constant="true"/>
                  <speciesReference species="M_23cump_c" stoichiometry="1"
constant="true"/>
            </listOfReactants>
            <listOfProducts>
                  <speciesReference species="M_3ump_c" stoichiometry="1"
constant="true"/>
            </listOfProducts>
            <fbc:geneProductAssociation>
                  <fbc:or>
                        <fbc:geneProductRef fbc:geneProduct="SAUSA300_0147"/>
                        <fbc:geneProductRef fbc:geneProduct="SAUSA300_0925"/>
                  </fbc:or>
            </fbc:geneProductAssociation>
      </reaction>

      <reaction metaid="R_23PDE4pp" id="R_23PDE4pp" name="2',3'-cyclic-
nucleotide phosphodiesterase (CMP) (periplasm)" reversible="false"
fast="false" fbc:lowerFluxBound="irr_lb" fbc:upperFluxBound="irr_ub">
            <listOfReactants>
                  <speciesReference species="M_h2o_c" stoichiometry="1"
constant="true"/>
                  <speciesReference species="M_23ccmp_c" stoichiometry="1"
constant="true"/>
            </listOfReactants>
            <listOfProducts>
                  <speciesReference species="M_3cmp_c" stoichiometry="1"
constant="true"/>
            </listOfProducts>
            <fbc:geneProductAssociation>
```

```
                  <fbc:or>
                        <fbc:geneProductRef fbc:geneProduct="SAUSA300_0147"/>
                        <fbc:geneProductRef fbc:geneProduct="SAUSA300_0925"/>
                  </fbc:or>
            </fbc:geneProductAssociation>
      </reaction>

      <reaction metaid="R_23PDE7pp" id="R_23PDE7pp" name="2',3'-cyclic-
nucleotide phosphodiesterase (AMP) (periplasm)" reversible="false"
fast="false" fbc:lowerFluxBound="irr_lb" fbc:upperFluxBound="irr_ub">
            <listOfReactants>
                  <speciesReference species="M_h2o_c" stoichiometry="1"
constant="true"/>
                  <speciesReference species="M_23camp_c" stoichiometry="1"
constant="true"/>
            </listOfReactants>
            <listOfProducts>
                  <speciesReference species="M_3amp_c" stoichiometry="1"
constant="true"/>
            </listOfProducts>
            <fbc:geneProductAssociation>
                  <fbc:or>
                        <fbc:geneProductRef fbc:geneProduct="SAUSA300_0147"/>
                        <fbc:geneProductRef fbc:geneProduct="SAUSA300_0925"/>
                  </fbc:or>
            </fbc:geneProductAssociation>
      </reaction>

      <reaction metaid="R_23PDE9pp" id="R_23PDE9pp" name="2',3'-cyclic-
nucleotide phosphodiesterase (GMP) (periplasm)" reversible="false"
fast="false" fbc:lowerFluxBound="irr_lb" fbc:upperFluxBound="irr_ub">
            <listOfReactants>
                  <speciesReference species="M_h2o_c" stoichiometry="1"
constant="true"/>
                  <speciesReference species="M_23cgmp_c" stoichiometry="1"
constant="true"/>
            </listOfReactants>
            <listOfProducts>
                  <speciesReference species="M_3gmp_c" stoichiometry="1"
constant="true"/>
            </listOfProducts>
            <fbc:geneProductAssociation>
                  <fbc:or>
                        <fbc:geneProductRef fbc:geneProduct="SAUSA300_0147"/>
                        <fbc:geneProductRef fbc:geneProduct="SAUSA300_0925"/>
                  </fbc:or>
            </fbc:geneProductAssociation>
      </reaction>

      <reaction metaid="R_2A2HBPL" id="R_2A2HBPL" name="(S)-2-Aceto-2-
hydroxybutanoate pyruvate__Lyase (carboxylating)" reversible="true"
fast="false" fbc:lowerFluxBound="rev_lb" fbc:upperFluxBound="rev_ub">
            <listOfReactants>
                  <speciesReference species="M_2obut_c" stoichiometry="1"
constant="true"/>
                  <speciesReference species="M_hethmpp_c" stoichiometry="1"
constant="true"/>
            </listOfReactants>
```

```
            <listOfProducts>
                  <speciesReference species="M_thmpp_c" stoichiometry="1"
constant="true"/>
                  <speciesReference species="M_2ahbut_c" stoichiometry="1"
constant="true"/>
            </listOfProducts>
            <fbc:geneProductAssociation>
                  <fbc:and>
                        <fbc:geneProductRef fbc:geneProduct="SAUSA300_2007"/>
                        <fbc:geneProductRef fbc:geneProduct="SAUSA300_2008"/>
                  </fbc:and>
            </fbc:geneProductAssociation>
      </reaction>

      <reaction metaid="R_2A4H6ET" id="R_2A4H6ET" name="2-Amino-4-hydroxy-6-
erythro-1 2 3-trihydroxypropyl " reversible="false" fast="false"
fbc:lowerFluxBound="irr_lb" fbc:upperFluxBound="irr_ub">
            <listOfReactants>
                  <speciesReference species="M_dattoo_c" stoichiometry="1"
constant="true"/>
            </listOfReactants>
            <listOfProducts>
                  <speciesReference species="M_h2o_c" stoichiometry="1"
constant="true"/>
                  <speciesReference species="M_ahdt_c" stoichiometry="1"
constant="true"/>
            </listOfProducts>
            <fbc:geneProductAssociation>
                  <fbc:geneProductRef fbc:geneProduct="SAUSA300_0551"/>
            </fbc:geneProductAssociation>
      </reaction>

      <reaction metaid="R_2ACLMM" id="R_2ACLMM" name="2-Acetolactate
methylmutase " reversible="false" fast="false" fbc:lowerFluxBound="irr_lb"
fbc:upperFluxBound="irr_ub">
            <listOfReactants>
                  <speciesReference species="M_alac__S_c" stoichiometry="1"
constant="true"/>
            </listOfReactants>
            <listOfProducts>
                  <speciesReference species="M_33hmeoxobut_c"
stoichiometry="1" constant="true"/>
            </listOfProducts>
            <fbc:geneProductAssociation>
                  <fbc:geneProductRef fbc:geneProduct="SAUSA300_2009"/>
            </fbc:geneProductAssociation>
      </reaction>

      <reaction metaid="R_2BDabc" id="R_2BDabc" name="2-beta__D-Glucosyl-sn-
glycerol ABC transport " reversible="false" fast="false"
fbc:lowerFluxBound="irr_lb" fbc:upperFluxBound="irr_ub">
            <listOfReactants>
                  <speciesReference species="M_h2o_c" stoichiometry="1"
constant="true"/>
                  <speciesReference species="M_atp_c" stoichiometry="1"
constant="true"/>
                  <speciesReference species="M_2bdgsglyc_e" stoichiometry="1"
constant="true"/>
```

```
            </listOfReactants>
            <listOfProducts>
                  <speciesReference species="M_h_c" stoichiometry="1"
constant="true"/>
                  <speciesReference species="M_pi_c" stoichiometry="1"
constant="true"/>
                  <speciesReference species="M_adp_c" stoichiometry="1"
constant="true"/>
                  <speciesReference species="M_2bdgsglyc_c" stoichiometry="1"
constant="true"/>
            </listOfProducts>
            <fbc:geneProductAssociation>
                  <fbc:geneProductRef fbc:geneProduct="SAUSA300_0208"/>
            </fbc:geneProductAssociation>
      </reaction>

      <reaction metaid="R_2MACPT" id="R_2MACPT" name="2-methylbutanoyl-CoA
acyl-carrier-protein transferase " reversible="true" fast="false"
fbc:lowerFluxBound="rev_lb" fbc:upperFluxBound="rev_ub">
            <listOfReactants>
                  <speciesReference species="M_ACP_c" stoichiometry="1"
constant="true"/>
                  <speciesReference species="M_2mbcoa_c" stoichiometry="1"
constant="true"/>
            </listOfReactants>
            <listOfProducts>
                  <speciesReference species="M_coa_c" stoichiometry="1"
constant="true"/>
                  <speciesReference species="M_2mbutACP_c" stoichiometry="1"
constant="true"/>
            </listOfProducts>
            <fbc:geneProductAssociation>
                  <fbc:geneProductRef fbc:geneProduct="SAUSA300_1123"/>
            </fbc:geneProductAssociation>
      </reaction>

      <reaction metaid="R_2MPCAC" id="R_2MPCAC" name="2-methylpropionyl-CoA
acyl-carrier-protein transferase " reversible="true" fast="false"
fbc:lowerFluxBound="rev_lb" fbc:upperFluxBound="rev_ub">
            <listOfReactants>
                  <speciesReference species="M_ACP_c" stoichiometry="1"
constant="true"/>
                  <speciesReference species="M_ibcoa_c" stoichiometry="1"
constant="true"/>
            </listOfReactants>
            <listOfProducts>
                  <speciesReference species="M_coa_c" stoichiometry="1"
constant="true"/>
                  <speciesReference species="M_isobutACP_c" stoichiometry="1"
constant="true"/>
            </listOfProducts>
            <fbc:geneProductAssociation>
                  <fbc:geneProductRef fbc:geneProduct="SAUSA300_1123"/>
            </fbc:geneProductAssociation>
      </reaction>
```

```
      <reaction metaid="R_2MPCTF" id="R_2MPCTF" name="10-methyl-3-
hydroxy__Dodecanoyl-ACP hydro__Lyase " reversible="true" fast="false"
fbc:lowerFluxBound="rev_lb" fbc:upperFluxBound="rev_ub">
            &lt;listOfReactants&gt;
                  <speciesReference species="M_3hoddecACP_c"
stoichiometry="1" constant="true"/>
            &lt;/listOfReactants&gt;
            &lt;listOfProducts&gt;
                  <speciesReference species="M_h2o_c" stoichiometry="1"
constant="true"/>
                  <speciesReference species="M_10mtd2eACP_c"
stoichiometry="1" constant="true"/>
            &lt;/listOfProducts&gt;
            &lt;fbc:geneProductAssociation&gt;
                  &lt;fbc:geneProductRef fbc:geneProduct="SAUSA300_2054"/&gt;
            &lt;/fbc:geneProductAssociation&gt;
      &lt;/reaction&gt;

      <reaction metaid="R_2OBUT_Et" id="R_2OBUT_Et" name="2-oxobutanoate
transport" reversible="true" fast="false" fbc:lowerFluxBound="rev_lb"
fbc:upperFluxBound="rev_ub">
            &lt;listOfReactants&gt;
                  <speciesReference species="M_2obut_e" stoichiometry="1"
constant="true"/>
            &lt;/listOfReactants&gt;
            &lt;listOfProducts&gt;
                  <speciesReference species="M_2obut_c" stoichiometry="1"
constant="true"/>
            &lt;/listOfProducts&gt;
      &lt;/reaction&gt;

      <reaction metaid="R_2OXOTHP" id="R_2OXOTHP" name="2-Oxoglutarate
Thiamin diphosphate 2-oxidoreductasedecarboxylating " reversible="false"
fast="false" fbc:lowerFluxBound="irr_lb" fbc:upperFluxBound="irr_ub">
            &lt;listOfReactants&gt;
                  <speciesReference species="M_h_c" stoichiometry="1"
constant="true"/>
                  <speciesReference species="M_akg_c" stoichiometry="1"
constant="true"/>
                  <speciesReference species="M_thmpp_c" stoichiometry="1"
constant="true"/>
            &lt;/listOfReactants&gt;
            &lt;listOfProducts&gt;
                  <speciesReference species="M_co2_c" stoichiometry="1"
constant="true"/>
                  <speciesReference species="M_3c1ht_c" stoichiometry="1"
constant="true"/>
            &lt;/listOfProducts&gt;
            &lt;fbc:geneProductAssociation&gt;
                  &lt;fbc:geneProductRef fbc:geneProduct="SAUSA300_1306"/&gt;
            &lt;/fbc:geneProductAssociation&gt;
      &lt;/reaction&gt;

      <reaction metaid="R_2S5EPAC" id="R_2S5EPAC" name="2-succinyl-5-
enolpyruvyl-6-hydroxy-3-cyclohexene-1-carboxylate " reversible="false"
fast="false" fbc:lowerFluxBound="irr_lb" fbc:upperFluxBound="irr_ub">
            &lt;listOfReactants&gt;
```

```
                  <speciesReference species="M_h_c" stoichiometry="1"
constant="true"/>
                  <speciesReference species="M_akg_c" stoichiometry="1"
constant="true"/>
                  <speciesReference species="M_ichor_c" stoichiometry="1"
constant="true"/>
            </listOfReactants>
            <listOfProducts>
                  <speciesReference species="M_co2_c" stoichiometry="1"
constant="true"/>
                  <speciesReference species="M_2sephchc_c" stoichiometry="1"
constant="true"/>
            </listOfProducts>
            <fbc:geneProductAssociation>
                  <fbc:geneProductRef fbc:geneProduct="SAUSA300_0946"/>
            </fbc:geneProductAssociation>
      </reaction>

      <reaction metaid="R_2SU6HYCC" id="R_2SU6HYCC" name="2-succinyl-6-
hydroxy-2 4-cyclohexadiene-1-carboxylate synthase " reversible="false"
fast="false" fbc:lowerFluxBound="irr_lb" fbc:upperFluxBound="irr_ub">
            <listOfReactants>
                  <speciesReference species="M_2sephchc_c" stoichiometry="1"
constant="true"/>
            </listOfReactants>
            <listOfProducts>
                  <speciesReference species="M_2shchc_c" stoichiometry="1"
constant="true"/>
                  <speciesReference species="M_pyr_c" stoichiometry="1"
constant="true"/>
            </listOfProducts>
            <fbc:geneProductAssociation>
                  <fbc:geneProductRef fbc:geneProduct="SAUSA300_0947"/>
            </fbc:geneProductAssociation>
      </reaction>

      <reaction metaid="R_3A2OA" id="R_3A2OA" name="3-Aminopropanoate 2-
oxoglutarate aminotransferase " reversible="true" fast="false"
fbc:lowerFluxBound="rev_lb" fbc:upperFluxBound="rev_ub">
            <listOfReactants>
                  <speciesReference species="M_akg_c" stoichiometry="1"
constant="true"/>
                  <speciesReference species="M_ala__B_c" stoichiometry="1"
constant="true"/>
            </listOfReactants>
            <listOfProducts>
                  <speciesReference species="M_glu__L_c" stoichiometry="1"
constant="true"/>
                  <speciesReference species="M_msa_c" stoichiometry="1"
constant="true"/>
            </listOfProducts>
            <fbc:geneProductAssociation>
                  <fbc:geneProductRef fbc:geneProduct="SAUSA300_2539"/>
            </fbc:geneProductAssociation>
      </reaction>
```

```
      <reaction metaid="R_3CRBL" id="R_3CRBL" name="3-Carboxy-1-
hydroxypropyl-ThPP lipoamde " reversible="true" fast="false"
fbc:lowerFluxBound="rev_lb" fbc:upperFluxBound="rev_ub">
            <listOfReactants>
                  <speciesReference species="M_lpam_c" stoichiometry="1"
constant="true"/>
                  <speciesReference species="M_3c1ht_c" stoichiometry="1"
constant="true"/>
            </listOfReactants>
            <listOfProducts>
                  <speciesReference species="M_thmpp_c" stoichiometry="1"
constant="true"/>
                  <speciesReference species="M_sdhlam_c" stoichiometry="1"
constant="true"/>
            </listOfProducts>
            <fbc:geneProductAssociation>
                  <fbc:geneProductRef fbc:geneProduct="SAUSA300_1306"/>
            </fbc:geneProductAssociation>
      </reaction>

      <reaction metaid="R_3GMPtex" id="R_3GMPtex" name="3GMP transport via
diffusion (extracellular to periplasm)" reversible="true" fast="false"
fbc:lowerFluxBound="rev_lb" fbc:upperFluxBound="rev_ub">
            <listOfReactants>
                  <speciesReference species="M_3gmp_e" stoichiometry="1"
constant="true"/>
            </listOfReactants>
            <listOfProducts>
                  <speciesReference species="M_3gmp_p" stoichiometry="1"
constant="true"/>
            </listOfProducts>
      </reaction>

      <reaction metaid="R_3HACPH" id="R_3HACPH" name="3R-3-Hydroxybutanoyl-
acyl-carrier-protein hydro__Lyase " reversible="true" fast="false"
fbc:lowerFluxBound="rev_lb" fbc:upperFluxBound="rev_ub">
            <listOfReactants>
                  <speciesReference species="M_r3hbACP_c" stoichiometry="1"
constant="true"/>
            </listOfReactants>
            <listOfProducts>
                  <speciesReference species="M_h2o_c" stoichiometry="1"
constant="true"/>
                  <speciesReference species="M_but2eACP_c" stoichiometry="1"
constant="true"/>
            </listOfProducts>
            <fbc:geneProductAssociation>
                  <fbc:geneProductRef fbc:geneProduct="SAUSA300_2054"/>
            </fbc:geneProductAssociation>
      </reaction>

      <reaction metaid="R_3HAD100" id="R_3HAD100" name="3-hydroxyacyl-[acyl-
carrier-protein] dehydratase (n-C10:0)" reversible="false" fast="false"
fbc:lowerFluxBound="irr_lb" fbc:upperFluxBound="irr_ub">
            <listOfReactants>
                  <speciesReference species="M_3hdecACP_c" stoichiometry="1"
constant="true"/>
            </listOfReactants>
```

```
            <listOfProducts>
                  <speciesReference species="M_h2o_c" stoichiometry="1"
constant="true"/>
                  <speciesReference species="M_tdec2eACP_c" stoichiometry="1"
constant="true"/>
            </listOfProducts>
            <fbc:geneProductAssociation>
                  <fbc:geneProductRef fbc:geneProduct="SAUSA300_2054"/>
            </fbc:geneProductAssociation>
      </reaction>

      <reaction metaid="R_3HAD120" id="R_3HAD120" name="3-hydroxyacyl-[acyl-
carrier-protein] dehydratase (n-C12:0)" reversible="false" fast="false"
fbc:lowerFluxBound="irr_lb" fbc:upperFluxBound="irr_ub">
            <listOfReactants>
                  <speciesReference species="M_3hddecACP_c" stoichiometry="1"
constant="true"/>
            </listOfReactants>
            <listOfProducts>
                  <speciesReference species="M_h2o_c" stoichiometry="1"
constant="true"/>
                  <speciesReference species="M_tddec2eACP_c"
stoichiometry="1" constant="true"/>
            </listOfProducts>
            <fbc:geneProductAssociation>
                  <fbc:geneProductRef fbc:geneProduct="SAUSA300_2054"/>
            </fbc:geneProductAssociation>
      </reaction>

      <reaction metaid="R_3HAD140" id="R_3HAD140" name="3-hydroxyacyl-[acyl-
carrier-protein] dehydratase (n-C14:0)" reversible="false" fast="false"
fbc:lowerFluxBound="irr_lb" fbc:upperFluxBound="irr_ub">
            <listOfReactants>
                  <speciesReference species="M_3hmrsACP_c" stoichiometry="1"
constant="true"/>
            </listOfReactants>
            <listOfProducts>
                  <speciesReference species="M_h2o_c" stoichiometry="1"
constant="true"/>
                  <speciesReference species="M_tmrs2eACP_c" stoichiometry="1"
constant="true"/>
            </listOfProducts>
            <fbc:geneProductAssociation>
                  <fbc:geneProductRef fbc:geneProduct="SAUSA300_2054"/>
            </fbc:geneProductAssociation>
      </reaction>

      <reaction metaid="R_3HAD160" id="R_3HAD160" name="3-hydroxyacyl-[acyl-
carrier-protein] dehydratase (n-C16:0)" reversible="false" fast="false"
fbc:lowerFluxBound="irr_lb" fbc:upperFluxBound="irr_ub">
            <listOfReactants>
                  <speciesReference species="M_3hpaACP_c" stoichiometry="1"
constant="true"/>
            </listOfReactants>
            <listOfProducts>
                  <speciesReference species="M_h2o_c" stoichiometry="1"
constant="true"/>
```

```
                  <speciesReference species="M_tpalm2eACP_c"
stoichiometry="1" constant="true"/>
            </listOfProducts>
            <fbc:geneProductAssociation>
                  <fbc:geneProductRef fbc:geneProduct="SAUSA300_2054"/>
            </fbc:geneProductAssociation>
      </reaction>

      <reaction metaid="R_3HAD60" id="R_3HAD60" name="3-hydroxyacyl-[acyl-
carrier-protein] dehydratase (n-C6:0)" reversible="false" fast="false"
fbc:lowerFluxBound="irr_lb" fbc:upperFluxBound="irr_ub">
            <listOfReactants>
                  <speciesReference species="M_3hhexACP_c" stoichiometry="1"
constant="true"/>
            </listOfReactants>
            <listOfProducts>
                  <speciesReference species="M_h2o_c" stoichiometry="1"
constant="true"/>
                  <speciesReference species="M_thex2eACP_c" stoichiometry="1"
constant="true"/>
            </listOfProducts>
            <fbc:geneProductAssociation>
                  <fbc:geneProductRef fbc:geneProduct="SAUSA300_2054"/>
            </fbc:geneProductAssociation>
      </reaction>

      <reaction metaid="R_3HAD80" id="R_3HAD80" name="3-hydroxyacyl-[acyl-
carrier-protein] dehydratase (n-C8:0)" reversible="false" fast="false"
fbc:lowerFluxBound="irr_lb" fbc:upperFluxBound="irr_ub">
            <listOfReactants>
                  <speciesReference species="M_3hocACP_c" stoichiometry="1"
constant="true"/>
            </listOfReactants>
            <listOfProducts>
                  <speciesReference species="M_h2o_c" stoichiometry="1"
constant="true"/>
                  <speciesReference species="M_toct2eACP_c" stoichiometry="1"
constant="true"/>
            </listOfProducts>
            <fbc:geneProductAssociation>
                  <fbc:geneProductRef fbc:geneProduct="SAUSA300_2054"/>
            </fbc:geneProductAssociation>
      </reaction>

      <reaction metaid="R_3HAOR" id="R_3HAOR" name="3-Hydroxyoctodecanoyl-ACP
NADP oxidoreductase " reversible="true" fast="false"
fbc:lowerFluxBound="rev_lb" fbc:upperFluxBound="rev_ub">
            <listOfReactants>
                  <speciesReference species="M_h_c" stoichiometry="1"
constant="true"/>
                  <speciesReference species="M_nadph_c" stoichiometry="1"
constant="true"/>
                  <speciesReference species="M_3oodACP_c" stoichiometry="1"
constant="true"/>
            </listOfReactants>
            <listOfProducts>
                  <speciesReference species="M_nadp_c" stoichiometry="1"
constant="true"/>
```

```
                  <speciesReference species="M_3hodACP_c" stoichiometry="1"
constant="true"/>
            </listOfProducts>
            <fbc:geneProductAssociation>
                  <fbc:or>
                        <fbc:geneProductRef fbc:geneProduct="SAUSA300_1124"/>
                        <fbc:geneProductRef fbc:geneProduct="SAUSA300_1173"/>
                  </fbc:or>
            </fbc:geneProductAssociation>
      </reaction>

      <reaction metaid="R_3HOXTPP" id="R_3HOXTPP" name="3R-3-Hydroxybutanoyl-
acyl-carrier protein NADP oxidoreductase " reversible="true" fast="false"
fbc:lowerFluxBound="rev_lb" fbc:upperFluxBound="rev_ub">
            <listOfReactants>
                  <speciesReference species="M_nadp_c" stoichiometry="1"
constant="true"/>
                  <speciesReference species="M_r3hbACP_c" stoichiometry="1"
constant="true"/>
            </listOfReactants>
            <listOfProducts>
                  <speciesReference species="M_h_c" stoichiometry="1"
constant="true"/>
                  <speciesReference species="M_nadph_c" stoichiometry="1"
constant="true"/>
                  <speciesReference species="M_actACP_c" stoichiometry="1"
constant="true"/>
            </listOfProducts>
            <fbc:geneProductAssociation>
                  <fbc:or>
                        <fbc:geneProductRef fbc:geneProduct="SAUSA300_1124"/>
                        <fbc:geneProductRef fbc:geneProduct="SAUSA300_1173"/>
                  </fbc:or>
            </fbc:geneProductAssociation>
      </reaction>

      <reaction metaid="R_3M2OBLOXRD" id="R_3M2OBLOXRD" name="3-Methyl-2-
oxobutanoate:lipoamide oxidoreductase(decarboxylating and acceptor-2-
methylpropanoylating)" reversible="true" fast="false"
fbc:lowerFluxBound="rev_lb" fbc:upperFluxBound="rev_ub">
            <listOfReactants>
                  <speciesReference species="M_h_c" stoichiometry="1"
constant="true"/>
                  <speciesReference species="M_3mob_c" stoichiometry="1"
constant="true"/>
                  <speciesReference species="M_lpam_c" stoichiometry="1"
constant="true"/>
            </listOfReactants>
            <listOfProducts>
                  <speciesReference species="M_co2_c" stoichiometry="1"
constant="true"/>
                  <speciesReference species="M_2mpdhl_c" stoichiometry="1"
constant="true"/>
            </listOfProducts>
            <fbc:geneProductAssociation>
                  <fbc:and>
                        <fbc:geneProductRef fbc:geneProduct="SAUSA300_1466"/>
                        <fbc:geneProductRef fbc:geneProduct="SAUSA300_1465"/>
```

```
                  </fbc:and>
            </fbc:geneProductAssociation>
      </reaction>

      <reaction metaid="R_3M2OPLOXRD" id="R_3M2OPLOXRD" name="3-Methyl-2-
oxopentanoate:lipoamide oxidoreductase(decarboxylating and acceptor-2-
methylpropanoylating)" reversible="true" fast="false"
fbc:lowerFluxBound="rev_lb" fbc:upperFluxBound="rev_ub">
            <listOfReactants>
                  <speciesReference species="M_h_c" stoichiometry="1"
constant="true"/>
                  <speciesReference species="M_lpam_c" stoichiometry="1"
constant="true"/>
                  <speciesReference species="M_3mop_c" stoichiometry="1"
constant="true"/>
            </listOfReactants>
            <listOfProducts>
                  <speciesReference species="M_co2_c" stoichiometry="1"
constant="true"/>
                  <speciesReference species="M_2mbdhl_c" stoichiometry="1"
constant="true"/>
            </listOfProducts>
            <fbc:geneProductAssociation>
                  <fbc:and>
                        <fbc:geneProductRef fbc:geneProduct="SAUSA300_1466"/>
                        <fbc:geneProductRef fbc:geneProduct="SAUSA300_1465"/>
                  </fbc:and>
            </fbc:geneProductAssociation>
      </reaction>

      <reaction metaid="R_3MBCOT" id="R_3MBCOT" name="3-methylbutanoyl-CoA
acyl-carrier-protein transferase " reversible="true" fast="false"
fbc:lowerFluxBound="rev_lb" fbc:upperFluxBound="rev_ub">
            <listOfReactants>
                  <speciesReference species="M_ACP_c" stoichiometry="1"
constant="true"/>
                  <speciesReference species="M_ivcoa_c" stoichiometry="1"
constant="true"/>
            </listOfReactants>
            <listOfProducts>
                  <speciesReference species="M_coa_c" stoichiometry="1"
constant="true"/>
                  <speciesReference species="M_isovACP_c" stoichiometry="1"
constant="true"/>
            </listOfProducts>
            <fbc:geneProductAssociation>
                  <fbc:geneProductRef fbc:geneProduct="SAUSA300_1123"/>
            </fbc:geneProductAssociation>
      </reaction>

      <reaction metaid="R_3METHMCAC" id="R_3METHMCAC" name="3-methylbutanoyl-
ACP malonyl-acyl-carrier-protein C-acyltransferase decarboxylating "
reversible="false" fast="false" fbc:lowerFluxBound="irr_lb"
fbc:upperFluxBound="irr_ub">
            <listOfReactants>
                  <speciesReference species="M_h_c" stoichiometry="1"
constant="true"/>
```

```
                  <speciesReference species="M_malACP_c" stoichiometry="1"
constant="true"/>
                  <speciesReference species="M_isovACP_c" stoichiometry="1"
constant="true"/>
            </listOfReactants>
            <listOfProducts>
                  <speciesReference species="M_ACP_c" stoichiometry="1"
constant="true"/>
                  <speciesReference species="M_co2_c" stoichiometry="1"
constant="true"/>
                  <speciesReference species="M_5m3hhACP_c" stoichiometry="1"
constant="true"/>
            </listOfProducts>
            <fbc:geneProductAssociation>
                  <fbc:and>
                        <fbc:geneProductRef fbc:geneProduct="SAUSA300_0886"/>
                        <fbc:geneProductRef fbc:geneProduct="SAUSA300_0885"/>
                  </fbc:and>
            </fbc:geneProductAssociation>
      </reaction>

      <reaction metaid="R_3MTDC" id="R_3MTDC" name="3-Methylcatechol oxygen 2
3-oxidoreductasedecyclizing " reversible="true" fast="false"
fbc:lowerFluxBound="rev_lb" fbc:upperFluxBound="rev_ub">
            <listOfReactants>
                  <speciesReference species="M_o2_c" stoichiometry="1"
constant="true"/>
                  <speciesReference species="M_3mcat_c" stoichiometry="1"
constant="true"/>
            </listOfReactants>
            <listOfProducts>
                  <speciesReference species="M_h_c" stoichiometry="1"
constant="true"/>
                  <speciesReference species="M_cchoxod_c" stoichiometry="1"
constant="true"/>
            </listOfProducts>
            <fbc:geneProductAssociation>
                  <fbc:geneProductRef fbc:geneProduct="SAUSA300_2461"/>
            </fbc:geneProductAssociation>
      </reaction>

      <reaction metaid="R_3NTD9pp" id="R_3NTD9pp" name="3'-nucleotidase (GMP)
(periplasm)" reversible="false" fast="false" fbc:lowerFluxBound="irr_lb"
fbc:upperFluxBound="irr_ub">
            <listOfReactants>
                  <speciesReference species="M_3gmp_p" stoichiometry="1"
constant="true"/>
                  <speciesReference species="M_h2o_p" stoichiometry="1"
constant="true"/>
            </listOfReactants>
            <listOfProducts>
                  <speciesReference species="M_pi_p" stoichiometry="1"
constant="true"/>
                  <speciesReference species="M_gsn_p" stoichiometry="1"
constant="true"/>
            </listOfProducts>
            <fbc:geneProductAssociation>
                  <fbc:geneProductRef fbc:geneProduct="SAUSA300_0147"/>
```

```
        </fbc:geneProductAssociation>
    </reaction>

    <reaction metaid="R_3OAR100" id="R_3OAR100" name="3-oxoacyl-[acyl-
carrier-protein] reductase (n-C10:0)" reversible="true" fast="false"
fbc:lowerFluxBound="rev_lb" fbc:upperFluxBound="rev_ub">
        <listOfReactants>
            <speciesReference species="M_nadp_c" stoichiometry="1"
constant="true"/>
            <speciesReference species="M_3hdecACP_c" stoichiometry="1"
constant="true"/>
        </listOfReactants>
        <listOfProducts>
            <speciesReference species="M_h_c" stoichiometry="1"
constant="true"/>
            <speciesReference species="M_nadph_c" stoichiometry="1"
constant="true"/>
            <speciesReference species="M_3odecACP_c" stoichiometry="1"
constant="true"/>
        </listOfProducts>
        <fbc:geneProductAssociation>
            <fbc:or>
                <fbc:geneProductRef fbc:geneProduct="SAUSA300_1124"/>
                <fbc:geneProductRef fbc:geneProduct="SAUSA300_1173"/>
            </fbc:or>
        </fbc:geneProductAssociation>
    </reaction>

    <reaction metaid="R_3OAR120" id="R_3OAR120" name="3-oxoacyl-[acyl-
carrier-protein] reductase (n-C12:0)" reversible="true" fast="false"
fbc:lowerFluxBound="rev_lb" fbc:upperFluxBound="rev_ub">
        <listOfReactants>
            <speciesReference species="M_nadp_c" stoichiometry="1"
constant="true"/>
            <speciesReference species="M_3hddecACP_c" stoichiometry="1"
constant="true"/>
        </listOfReactants>
        <listOfProducts>
            <speciesReference species="M_h_c" stoichiometry="1"
constant="true"/>
            <speciesReference species="M_nadph_c" stoichiometry="1"
constant="true"/>
            <speciesReference species="M_3oxddACP_c" stoichiometry="1"
constant="true"/>
        </listOfProducts>
        <fbc:geneProductAssociation>
            <fbc:or>
                <fbc:geneProductRef fbc:geneProduct="SAUSA300_1124"/>
                <fbc:geneProductRef fbc:geneProduct="SAUSA300_1173"/>
            </fbc:or>
        </fbc:geneProductAssociation>
    </reaction>

    <reaction metaid="R_3OAR140" id="R_3OAR140" name="3-oxoacyl-[acyl-
carrier-protein] reductase (n-C14:0)" reversible="true" fast="false"
fbc:lowerFluxBound="rev_lb" fbc:upperFluxBound="rev_ub">
        <listOfReactants>
```

```
                  <speciesReference species="M_nadp_c" stoichiometry="1"
constant="true"/>
                  <speciesReference species="M_3hmrsACP_c" stoichiometry="1"
constant="true"/>
            </listOfReactants>
            <listOfProducts>
                  <speciesReference species="M_h_c" stoichiometry="1"
constant="true"/>
                  <speciesReference species="M_nadph_c" stoichiometry="1"
constant="true"/>
                  <speciesReference species="M_3omrsACP_c" stoichiometry="1"
constant="true"/>
            </listOfProducts>
            <fbc:geneProductAssociation>
                  <fbc:or>
                        <fbc:geneProductRef fbc:geneProduct="SAUSA300_1124"/>
                        <fbc:geneProductRef fbc:geneProduct="SAUSA300_1173"/>
                  </fbc:or>
            </fbc:geneProductAssociation>
      </reaction>

      <reaction metaid="R_3OAR160" id="R_3OAR160" name="3-oxoacyl-[acyl-
carrier-protein] reductase (n-C16:0)" reversible="true" fast="false"
fbc:lowerFluxBound="rev_lb" fbc:upperFluxBound="rev_ub">
            <listOfReactants>
                  <speciesReference species="M_nadp_c" stoichiometry="1"
constant="true"/>
                  <speciesReference species="M_3hpaACP_c" stoichiometry="1"
constant="true"/>
            </listOfReactants>
            <listOfProducts>
                  <speciesReference species="M_h_c" stoichiometry="1"
constant="true"/>
                  <speciesReference species="M_nadph_c" stoichiometry="1"
constant="true"/>
                  <speciesReference species="M_3oxhdACP_c" stoichiometry="1"
constant="true"/>
            </listOfProducts>
            <fbc:geneProductAssociation>
                  <fbc:or>
                        <fbc:geneProductRef fbc:geneProduct="SAUSA300_1124"/>
                        <fbc:geneProductRef fbc:geneProduct="SAUSA300_1173"/>
                  </fbc:or>
            </fbc:geneProductAssociation>
      </reaction>

      <reaction metaid="R_3OAR60" id="R_3OAR60" name="3-oxoacyl-[acyl-
carrier-protein] reductase (n-C6:0)" reversible="true" fast="false"
fbc:lowerFluxBound="rev_lb" fbc:upperFluxBound="rev_ub">
            <listOfReactants>
                  <speciesReference species="M_nadp_c" stoichiometry="1"
constant="true"/>
                  <speciesReference species="M_3hhexACP_c" stoichiometry="1"
constant="true"/>
            </listOfReactants>
            <listOfProducts>
                  <speciesReference species="M_h_c" stoichiometry="1"
constant="true"/>
```

```
                  <speciesReference species="M_nadph_c" stoichiometry="1"
constant="true"/>
                  <speciesReference species="M_3ohexACP_c" stoichiometry="1"
constant="true"/>
            </listOfProducts>
            <fbc:geneProductAssociation>
                  <fbc:or>
                        <fbc:geneProductRef fbc:geneProduct="SAUSA300_1124"/>
                        <fbc:geneProductRef fbc:geneProduct="SAUSA300_1173"/>
                  </fbc:or>
            </fbc:geneProductAssociation>
      </reaction>

      <reaction metaid="R_3OAR80" id="R_3OAR80" name="3-oxoacyl-[acyl-
carrier-protein] reductase (n-C8:0)" reversible="true" fast="false"
fbc:lowerFluxBound="rev_lb" fbc:upperFluxBound="rev_ub">
            <listOfReactants>
                  <speciesReference species="M_nadp_c" stoichiometry="1"
constant="true"/>
                  <speciesReference species="M_3hocACP_c" stoichiometry="1"
constant="true"/>
            </listOfReactants>
            <listOfProducts>
                  <speciesReference species="M_h_c" stoichiometry="1"
constant="true"/>
                  <speciesReference species="M_nadph_c" stoichiometry="1"
constant="true"/>
                  <speciesReference species="M_3oxocACP_c" stoichiometry="1"
constant="true"/>
            </listOfProducts>
            <fbc:geneProductAssociation>
                  <fbc:or>
                        <fbc:geneProductRef fbc:geneProduct="SAUSA300_1124"/>
                        <fbc:geneProductRef fbc:geneProduct="SAUSA300_1173"/>
                  </fbc:or>
            </fbc:geneProductAssociation>
      </reaction>

      <reaction metaid="R_3OAS100" id="R_3OAS100" name="3-oxoacyl-[acyl-
carrier-protein] synthase (n-C10:0)" reversible="false" fast="false"
fbc:lowerFluxBound="irr_lb" fbc:upperFluxBound="irr_ub">
            <listOfReactants>
                  <speciesReference species="M_h_c" stoichiometry="1"
constant="true"/>
                  <speciesReference species="M_malACP_c" stoichiometry="1"
constant="true"/>
                  <speciesReference species="M_ocACP_c" stoichiometry="1"
constant="true"/>
            </listOfReactants>
            <listOfProducts>
                  <speciesReference species="M_ACP_c" stoichiometry="1"
constant="true"/>
                  <speciesReference species="M_co2_c" stoichiometry="1"
constant="true"/>
                  <speciesReference species="M_3odecACP_c" stoichiometry="1"
constant="true"/>
            </listOfProducts>
            <fbc:geneProductAssociation>
```

```
                    <fbc:and>
                          <fbc:geneProductRef fbc:geneProduct="SAUSA300_0886"/>
                          <fbc:geneProductRef fbc:geneProduct="SAUSA300_0885"/>
                    </fbc:and>
              </fbc:geneProductAssociation>
        </reaction>

        <reaction metaid="R_3OAS120" id="R_3OAS120" name="3-oxoacyl-[acyl-
carrier-protein] synthase (n-C12:0)" reversible="false" fast="false"
fbc:lowerFluxBound="irr_lb" fbc:upperFluxBound="irr_ub">
              <listOfReactants>
                    <speciesReference species="M_h_c" stoichiometry="1"
constant="true"/>
                    <speciesReference species="M_malACP_c" stoichiometry="1"
constant="true"/>
                    <speciesReference species="M_dcaACP_c" stoichiometry="1"
constant="true"/>
              </listOfReactants>
              <listOfProducts>
                    <speciesReference species="M_ACP_c" stoichiometry="1"
constant="true"/>
                    <speciesReference species="M_co2_c" stoichiometry="1"
constant="true"/>
                    <speciesReference species="M_3oxddACP_c" stoichiometry="1"
constant="true"/>
              </listOfProducts>
              <fbc:geneProductAssociation>
                    <fbc:and>
                          <fbc:geneProductRef fbc:geneProduct="SAUSA300_0886"/>
                          <fbc:geneProductRef fbc:geneProduct="SAUSA300_0885"/>
                    </fbc:and>
              </fbc:geneProductAssociation>
        </reaction>

        <reaction metaid="R_3OAS140" id="R_3OAS140" name="3-oxoacyl-[acyl-
carrier-protein] synthase (n-C14:0)" reversible="false" fast="false"
fbc:lowerFluxBound="irr_lb" fbc:upperFluxBound="irr_ub">
              <listOfReactants>
                    <speciesReference species="M_h_c" stoichiometry="1"
constant="true"/>
                    <speciesReference species="M_malACP_c" stoichiometry="1"
constant="true"/>
                    <speciesReference species="M_ddcaACP_c" stoichiometry="1"
constant="true"/>
              </listOfReactants>
              <listOfProducts>
                    <speciesReference species="M_ACP_c" stoichiometry="1"
constant="true"/>
                    <speciesReference species="M_co2_c" stoichiometry="1"
constant="true"/>
                    <speciesReference species="M_3omrsACP_c" stoichiometry="1"
constant="true"/>
              </listOfProducts>
              <fbc:geneProductAssociation>
                    <fbc:geneProductRef fbc:geneProduct="SAUSA300_0886"/>
              </fbc:geneProductAssociation>
        </reaction>
```

```
      <reaction metaid="R_3OAS160" id="R_3OAS160" name="3-oxoacyl-[acyl-
carrier-protein] synthase (n-C16:0)" reversible="false" fast="false"
fbc:lowerFluxBound="irr_lb" fbc:upperFluxBound="irr_ub">
            <listOfReactants>
                  <speciesReference species="M_h_c" stoichiometry="1"
constant="true"/>
                  <speciesReference species="M_malACP_c" stoichiometry="1"
constant="true"/>
                  <speciesReference species="M_myrsACP_c" stoichiometry="1"
constant="true"/>
            </listOfReactants>
            <listOfProducts>
                  <speciesReference species="M_ACP_c" stoichiometry="1"
constant="true"/>
                  <speciesReference species="M_co2_c" stoichiometry="1"
constant="true"/>
                  <speciesReference species="M_3oxhdACP_c" stoichiometry="1"
constant="true"/>
            </listOfProducts>
            <fbc:geneProductAssociation>
                  <fbc:and>
                        <fbc:geneProductRef fbc:geneProduct="SAUSA300_0886"/>
                        <fbc:geneProductRef fbc:geneProduct="SAUSA300_0885"/>
                  </fbc:and>
            </fbc:geneProductAssociation>
      </reaction>

      <reaction metaid="R_3OAS60" id="R_3OAS60" name="3-oxoacyl-[acyl-
carrier-protein] synthase (n-C6:0)" reversible="false" fast="false"
fbc:lowerFluxBound="irr_lb" fbc:upperFluxBound="irr_ub">
            <listOfReactants>
                  <speciesReference species="M_h_c" stoichiometry="1"
constant="true"/>
                  <speciesReference species="M_malACP_c" stoichiometry="1"
constant="true"/>
                  <speciesReference species="M_butACP_c" stoichiometry="1"
constant="true"/>
            </listOfReactants>
            <listOfProducts>
                  <speciesReference species="M_ACP_c" stoichiometry="1"
constant="true"/>
                  <speciesReference species="M_co2_c" stoichiometry="1"
constant="true"/>
                  <speciesReference species="M_3ohexACP_c" stoichiometry="1"
constant="true"/>
            </listOfProducts>
            <fbc:geneProductAssociation>
                  <fbc:and>
                        <fbc:geneProductRef fbc:geneProduct="SAUSA300_0886"/>
                        <fbc:geneProductRef fbc:geneProduct="SAUSA300_0885"/>
                  </fbc:and>
            </fbc:geneProductAssociation>
      </reaction>

      <reaction metaid="R_3OAS80" id="R_3OAS80" name="3-oxoacyl-[acyl-
carrier-protein] synthase (n-C8:0)" reversible="false" fast="false"
fbc:lowerFluxBound="irr_lb" fbc:upperFluxBound="irr_ub">
            <listOfReactants>
```

```
                  <speciesReference species="M_h_c" stoichiometry="1"
constant="true"/>
                  <speciesReference species="M_malACP_c" stoichiometry="1"
constant="true"/>
                  <speciesReference species="M_hexACP_c" stoichiometry="1"
constant="true"/>
            </listOfReactants>
            <listOfProducts>
                  <speciesReference species="M_ACP_c" stoichiometry="1"
constant="true"/>
                  <speciesReference species="M_co2_c" stoichiometry="1"
constant="true"/>
                  <speciesReference species="M_3oxocACP_c" stoichiometry="1"
constant="true"/>
            </listOfProducts>
            <fbc:geneProductAssociation>
                  <fbc:and>
                        <fbc:geneProductRef fbc:geneProduct="SAUSA300_0886"/>
                        <fbc:geneProductRef fbc:geneProduct="SAUSA300_0885"/>
                  </fbc:and>
            </fbc:geneProductAssociation>
      </reaction>

      <reaction metaid="R_3OXCOAT" id="R_3OXCOAT" name="3-oxoadipyl-CoA
thiolase" reversible="false" fast="false" fbc:lowerFluxBound="irr_lb"
fbc:upperFluxBound="irr_ub">
            <listOfReactants>
                  <speciesReference species="M_accoa_c" stoichiometry="1"
constant="true"/>
                  <speciesReference species="M_succoa_c" stoichiometry="1"
constant="true"/>
            </listOfReactants>
            <listOfProducts>
                  <speciesReference species="M_coa_c" stoichiometry="1"
constant="true"/>
                  <speciesReference species="M_oxadpcoa_c" stoichiometry="1"
constant="true"/>
            </listOfProducts>
            <fbc:geneProductAssociation>
                  <fbc:geneProductRef fbc:geneProduct="SAUSA300_0225"/>
            </fbc:geneProductAssociation>
      </reaction>

      <reaction metaid="R_3OXDAM" id="R_3OXDAM" name="3-Oxooctodecanoyl-ACP
malonyl-acyl-carrier-protein C-acyltransferase decarboxylating "
reversible="false" fast="false" fbc:lowerFluxBound="irr_lb"
fbc:upperFluxBound="irr_ub">
            <listOfReactants>
                  <speciesReference species="M_h_c" stoichiometry="1"
constant="true"/>
                  <speciesReference species="M_malACP_c" stoichiometry="1"
constant="true"/>
                  <speciesReference species="M_hedacp_c" stoichiometry="1"
constant="true"/>
            </listOfReactants>
            <listOfProducts>
                  <speciesReference species="M_ACP_c" stoichiometry="1"
constant="true"/>
```

```xml
                  <speciesReference species="M_co2_c" stoichiometry="1"
constant="true"/>
                  <speciesReference species="M_3oodACP_c" stoichiometry="1"
constant="true"/>
            </listOfProducts>
            <fbc:geneProductAssociation>
                  <fbc:and>
                        <fbc:geneProductRef fbc:geneProduct="SAUSA300_0886"/>
                        <fbc:geneProductRef fbc:geneProduct="SAUSA300_0885"/>
                  </fbc:and>
            </fbc:geneProductAssociation>
      </reaction>

      <reaction metaid="R_4HPOO" id="R_4HPOO" name="4-hydroxy-5-
phenyltetrahydro-1 3-oxazin-2-one NAD oxidoreductase " reversible="true"
fast="false" fbc:lowerFluxBound="rev_lb" fbc:upperFluxBound="rev_ub">
            <listOfReactants>
                  <speciesReference species="M_nad_c" stoichiometry="1"
constant="true"/>
                  <speciesReference species="M_4h5pone" stoichiometry="1"
constant="true"/>
            </listOfReactants>
            <listOfProducts>
                  <speciesReference species="M_h_c" stoichiometry="1"
constant="true"/>
                  <speciesReference species="M_nadh_c" stoichiometry="1"
constant="true"/>
                  <speciesReference species="M_5podio" stoichiometry="1"
constant="true"/>
            </listOfProducts>
            <fbc:geneProductAssociation>
                  <fbc:geneProductRef fbc:geneProduct="SAUSA300_2147"/>
            </fbc:geneProductAssociation>
      </reaction>

      <reaction metaid="R_4HTHRS" id="R_4HTHRS" name="4-Hydroxy__L-threonine
synthase" reversible="false" fast="false" fbc:lowerFluxBound="irr_lb"
fbc:upperFluxBound="irr_ub">
            <listOfReactants>
                  <speciesReference species="M_h2o_c" stoichiometry="1"
constant="true"/>
                  <speciesReference species="M_phthr_c" stoichiometry="1"
constant="true"/>
            </listOfReactants>
            <listOfProducts>
                  <speciesReference species="M_h_c" stoichiometry="1"
constant="true"/>
                  <speciesReference species="M_pi_c" stoichiometry="1"
constant="true"/>
                  <speciesReference species="M_4hthr_c" stoichiometry="1"
constant="true"/>
            </listOfProducts>
            <fbc:geneProductAssociation>
                  <fbc:geneProductRef fbc:geneProduct="SAUSA300_1227"/>
            </fbc:geneProductAssociation>
      </reaction>
```

```
      <reaction metaid="R_4ISOHELS" id="R_4ISOHELS" name="4-methyl-3-hydroxy-
hexanoyl-ACP hydro__Lyase " reversible="true" fast="false"
fbc:lowerFluxBound="rev_lb" fbc:upperFluxBound="rev_ub">
            <listOfReactants>
                  <speciesReference species="M_4m3hhexACP_c"
stoichiometry="1" constant="true"/>
            </listOfReactants>
            <listOfProducts>
                  <speciesReference species="M_h2o_c" stoichiometry="1"
constant="true"/>
                  <speciesReference species="M_4methex2eACP_c"
stoichiometry="1" constant="true"/>
            </listOfProducts>
            <fbc:geneProductAssociation>
                  <fbc:geneProductRef fbc:geneProduct="SAUSA300_2054"/>
            </fbc:geneProductAssociation>
      </reaction>

      <reaction metaid="R_4M2OPLOXRD" id="R_4M2OPLOXRD" name="4-Methyl-2-
oxopentanoate:lipoamide oxidoreductase(decarboxylating and acceptor-2-
methylpropanoylating)" reversible="true" fast="false"
fbc:lowerFluxBound="rev_lb" fbc:upperFluxBound="rev_ub">
            <listOfReactants>
                  <speciesReference species="M_h_c" stoichiometry="1"
constant="true"/>
                  <speciesReference species="M_4mop_c" stoichiometry="1"
constant="true"/>
                  <speciesReference species="M_lpam_c" stoichiometry="1"
constant="true"/>
            </listOfReactants>
            <listOfProducts>
                  <speciesReference species="M_co2_c" stoichiometry="1"
constant="true"/>
                  <speciesReference species="M_3mbdhl_c" stoichiometry="1"
constant="true"/>
            </listOfProducts>
            <fbc:geneProductAssociation>
                  <fbc:and>
                        <fbc:geneProductRef fbc:geneProduct="SAUSA300_1466"/>
                        <fbc:geneProductRef fbc:geneProduct="SAUSA300_1465"/>
                  </fbc:and>
            </fbc:geneProductAssociation>
      </reaction>

      <reaction metaid="R_4M3G3P" id="R_4M3G3P" name="4-methyl-3-hydroxy-
pentanoyl-ACP hydro__Lyase " reversible="true" fast="false"
fbc:lowerFluxBound="rev_lb" fbc:upperFluxBound="rev_ub">
            <listOfReactants>
                  <speciesReference species="M_4m3hpAPC_c" stoichiometry="1"
constant="true"/>
            </listOfReactants>
            <listOfProducts>
                  <speciesReference species="M_h2o_c" stoichiometry="1"
constant="true"/>
                  <speciesReference species="M_4mtpACP_c" stoichiometry="1"
constant="true"/>
            </listOfProducts>
            <fbc:geneProductAssociation>
```

```
                  <fbc:geneProductRef fbc:geneProduct="SAUSA300_2054"/>
            </fbc:geneProductAssociation>
      </reaction>

      <reaction metaid="R_4M3OHAT" id="R_4M3OHAT" name="4-methyl-3-oxo-
hexanoyl-ACP malonyl-acyl-carrier-protein C-acyltransferase decarboxylating "
reversible="false" fast="false" fbc:lowerFluxBound="irr_lb"
fbc:upperFluxBound="irr_ub">
            <listOfReactants>
                  <speciesReference species="M_h_c" stoichiometry="1"
constant="true"/>
                  <speciesReference species="M_malACP_c" stoichiometry="1"
constant="true"/>
                  <speciesReference species="M_2mbutACP_c" stoichiometry="1"
constant="true"/>
            </listOfReactants>
            <listOfProducts>
                  <speciesReference species="M_ACP_c" stoichiometry="1"
constant="true"/>
                  <speciesReference species="M_co2_c" stoichiometry="1"
constant="true"/>
                  <speciesReference species="M_4m3ohexACP_c"
stoichiometry="1" constant="true"/>
            </listOfProducts>
            <fbc:geneProductAssociation>
                  <fbc:and>
                        <fbc:geneProductRef fbc:geneProduct="SAUSA300_0886"/>
                        <fbc:geneProductRef fbc:geneProduct="SAUSA300_0885"/>
                  </fbc:and>
            </fbc:geneProductAssociation>
      </reaction>

      <reaction metaid="R_4M3OPAO" id="R_4M3OPAO" name="4-methyl-3-oxo-
pentanoyl-ACP NADP oxidoreductase " reversible="true" fast="false"
fbc:lowerFluxBound="rev_lb" fbc:upperFluxBound="rev_ub">
            <listOfReactants>
                  <speciesReference species="M_h_c" stoichiometry="1"
constant="true"/>
                  <speciesReference species="M_nadph_c" stoichiometry="1"
constant="true"/>
                  <speciesReference species="M_4m3opACP_c" stoichiometry="1"
constant="true"/>
            </listOfReactants>
            <listOfProducts>
                  <speciesReference species="M_nadp_c" stoichiometry="1"
constant="true"/>
                  <speciesReference species="M_4m3hpAPC_c" stoichiometry="1"
constant="true"/>
            </listOfProducts>
            <fbc:geneProductAssociation>
                  <fbc:or>
                        <fbc:geneProductRef fbc:geneProduct="SAUSA300_1124"/>
                        <fbc:geneProductRef fbc:geneProduct="SAUSA300_1173"/>
                  </fbc:or>
            </fbc:geneProductAssociation>
      </reaction>
```

```
      <reaction metaid="R_4M3OXHA" id="R_4M3OXHA" name="4-methyl-3-oxo-
hexanoyl-ACP NADP oxidoreductase " reversible="true" fast="false"
fbc:lowerFluxBound="rev_lb" fbc:upperFluxBound="rev_ub">
            <listOfReactants>
                  <speciesReference species="M_h_c" stoichiometry="1"
constant="true"/>
                  <speciesReference species="M_nadph_c" stoichiometry="1"
constant="true"/>
                  <speciesReference species="M_4m3ohexACP_c"
stoichiometry="1" constant="true"/>
            </listOfReactants>
            <listOfProducts>
                  <speciesReference species="M_nadp_c" stoichiometry="1"
constant="true"/>
                  <speciesReference species="M_4m3hhexACP_c"
stoichiometry="1" constant="true"/>
            </listOfProducts>
            <fbc:geneProductAssociation>
                  <fbc:or>
                        <fbc:geneProductRef fbc:geneProduct="SAUSA300_1124"/>
                        <fbc:geneProductRef fbc:geneProduct="SAUSA300_1173"/>
                  </fbc:or>
            </fbc:geneProductAssociation>
      </reaction>

      <reaction metaid="R_4MCO2D" id="R_4MCO2D" name="4-Methylcatechol oxygen
2 3-oxidoreductasedecyclizing " reversible="true" fast="false"
fbc:lowerFluxBound="rev_lb" fbc:upperFluxBound="rev_ub">
            <listOfReactants>
                  <speciesReference species="M_o2_c" stoichiometry="1"
constant="true"/>
                  <speciesReference species="M_4mcat_c" stoichiometry="1"
constant="true"/>
            </listOfReactants>
            <listOfProducts>
                  <speciesReference species="M_h_c" stoichiometry="1"
constant="true"/>
                  <speciesReference species="M_hmccms_c" stoichiometry="1"
constant="true"/>
            </listOfProducts>
            <fbc:geneProductAssociation>
                  <fbc:geneProductRef fbc:geneProduct="SAUSA300_2461"/>
            </fbc:geneProductAssociation>
      </reaction>

      <reaction metaid="R_4METPAC" id="R_4METPAC" name="4-methyl-pentanoyl-
ACP malonyl-acyl-carrier-protein C-acyltransferase decarboxylating "
reversible="false" fast="false" fbc:lowerFluxBound="irr_lb"
fbc:upperFluxBound="irr_ub">
            <listOfReactants>
                  <speciesReference species="M_h_c" stoichiometry="1"
constant="true"/>
                  <speciesReference species="M_malACP_c" stoichiometry="1"
constant="true"/>
                  <speciesReference species="M_4mpACP_c" stoichiometry="1"
constant="true"/>
            </listOfReactants>
            <listOfProducts>
```

```
                  <speciesReference species="M_ACP_c" stoichiometry="1"
constant="true"/>
                  <speciesReference species="M_co2_c" stoichiometry="1"
constant="true"/>
                  <speciesReference species="M_6m3ohACP_c" stoichiometry="1"
constant="true"/>
            </listOfProducts>
            <fbc:geneProductAssociation>
                  <fbc:and>
                        <fbc:geneProductRef fbc:geneProduct="SAUSA300_0886"/>
                        <fbc:geneProductRef fbc:geneProduct="SAUSA300_0885"/>
                  </fbc:and>
            </fbc:geneProductAssociation>
      </reaction>

      <reaction metaid="R_4MHEAMA" id="R_4MHEAMA" name="4-methyl-hexanoyl-ACP
malonyl-acyl-carrier-protein C-acyltransferase decarboxylating "
reversible="false" fast="false" fbc:lowerFluxBound="irr_lb"
fbc:upperFluxBound="irr_ub">
            <listOfReactants>
                  <speciesReference species="M_h_c" stoichiometry="1"
constant="true"/>
                  <speciesReference species="M_malACP_c" stoichiometry="1"
constant="true"/>
                  <speciesReference species="M_4methexACP_c"
stoichiometry="1" constant="true"/>
            </listOfReactants>
            <listOfProducts>
                  <speciesReference species="M_ACP_c" stoichiometry="1"
constant="true"/>
                  <speciesReference species="M_co2_c" stoichiometry="1"
constant="true"/>
                  <speciesReference species="M_6m3oACP_c" stoichiometry="1"
constant="true"/>
            </listOfProducts>
            <fbc:geneProductAssociation>
                  <fbc:and>
                        <fbc:geneProductRef fbc:geneProduct="SAUSA300_0886"/>
                        <fbc:geneProductRef fbc:geneProduct="SAUSA300_0885"/>
                  </fbc:and>
            </fbc:geneProductAssociation>
      </reaction>

      <reaction metaid="R_4MTHACP" id="R_4MTHACP" name="4-methyl-trans-hex-2-
enoyl-ACP NAD oxidoreductase A-specific " reversible="false" fast="false"
fbc:lowerFluxBound="irr_lb" fbc:upperFluxBound="irr_ub">
            <listOfReactants>
                  <speciesReference species="M_h_c" stoichiometry="1"
constant="true"/>
                  <speciesReference species="M_nadh_c" stoichiometry="1"
constant="true"/>
                  <speciesReference species="M_4methex2eACP_c"
stoichiometry="1" constant="true"/>
            </listOfReactants>
            <listOfProducts>
                  <speciesReference species="M_nad_c" stoichiometry="1"
constant="true"/>
```

<speciesReference species="M_4methexACP_c"
stoichiometry="1" constant="true"/>
</listOfProducts>
<fbc:geneProductAssociation>
<fbc:geneProductRef fbc:geneProduct="SAUSA300_0912"/>
</fbc:geneProductAssociation>
</reaction>

<reaction metaid="R_4MTRPE" id="R_4MTRPE" name="4-methyl-trans-pent-2-enoyl-ACP NAD oxidoreductase A-specific " reversible="false" fast="false"
fbc:lowerFluxBound="irr_lb" fbc:upperFluxBound="irr_ub">
<listOfReactants>
<speciesReference species="M_h_c" stoichiometry="1"
constant="true"/>
<speciesReference species="M_nadh_c" stoichiometry="1"
constant="true"/>
<speciesReference species="M_4mtpACP_c" stoichiometry="1"
constant="true"/>
</listOfReactants>
<listOfProducts>
<speciesReference species="M_nad_c" stoichiometry="1"
constant="true"/>
<speciesReference species="M_4mpACP_c" stoichiometry="1"
constant="true"/>
</listOfProducts>
<fbc:geneProductAssociation>
<fbc:geneProductRef fbc:geneProduct="SAUSA300_0912"/>
</fbc:geneProductAssociation>
</reaction>

<reaction metaid="R_4NPPP" id="R_4NPPP" name="4-Nitrophenyl phosphate
phosphohydrolase " reversible="false" fast="false"
fbc:lowerFluxBound="irr_lb" fbc:upperFluxBound="irr_ub">
<listOfReactants>
<speciesReference species="M_h2o_c" stoichiometry="1"
constant="true"/>
<speciesReference species="M_4nphp_c" stoichiometry="1"
constant="true"/>
</listOfReactants>
<listOfProducts>
<speciesReference species="M_h_c" stoichiometry="1"
constant="true"/>
<speciesReference species="M_pi_c" stoichiometry="1"
constant="true"/>
<speciesReference species="M_4nph_c" stoichiometry="1"
constant="true"/>
</listOfProducts>
<fbc:geneProductAssociation>
<fbc:geneProductRef fbc:geneProduct="SAUSA300_2561"/>
</fbc:geneProductAssociation>
</reaction>

<reaction metaid="R_4OT" id="R_4OT" name="4-oxalocrotonate tautomerase"
reversible="true" fast="false" fbc:lowerFluxBound="rev_lb"
fbc:upperFluxBound="rev_ub">
<listOfReactants>
<speciesReference species="M_2hmc_c" stoichiometry="1"
constant="true"/>

```
            </listOfReactants>
            <listOfProducts>
                  <speciesReference species="M_oxalc_c" stoichiometry="1"
constant="true"/>
            </listOfProducts>
            <fbc:geneProductAssociation>
                  <fbc:geneProductRef fbc:geneProduct="SAUSA300_1258"/>
            </fbc:geneProductAssociation>
      </reaction>

      <reaction metaid="R_5GPAGC" id="R_5GPAGC" name="5-Glutamyl-peptide
amino-acid 5-glutamyltransferase " reversible="false" fast="false"
fbc:lowerFluxBound="irr_lb" fbc:upperFluxBound="irr_ub">
            <listOfReactants>
                  <speciesReference species="M_h_c" stoichiometry="1"
constant="true"/>
                  <speciesReference species="M_glu__L_c" stoichiometry="1"
constant="true"/>
                  <speciesReference species="M_cyoala_c" stoichiometry="1"
constant="true"/>
            </listOfReactants>
            <listOfProducts>
                  <speciesReference species="M_h2o_c" stoichiometry="1"
constant="true"/>
                  <speciesReference species="M_co2_c" stoichiometry="1"
constant="true"/>
                  <speciesReference species="M_gg3apn_c" stoichiometry="1"
constant="true"/>
            </listOfProducts>
            <fbc:geneProductAssociation>
                  <fbc:geneProductRef fbc:geneProduct="SAUSA300_0204"/>
            </fbc:geneProductAssociation>
      </reaction>

      <reaction metaid="R_5M3HHAL" id="R_5M3HHAL" name="5-methyl-3-hydroxy-
hexanoyl-ACP hydro__Lyase " reversible="true" fast="false"
fbc:lowerFluxBound="rev_lb" fbc:upperFluxBound="rev_ub">
            <listOfReactants>
                  <speciesReference species="M_5m3hACP_c" stoichiometry="1"
constant="true"/>
            </listOfReactants>
            <listOfProducts>
                  <speciesReference species="M_h2o_c" stoichiometry="1"
constant="true"/>
                  <speciesReference species="M_5mhACP_c" stoichiometry="1"
constant="true"/>
            </listOfProducts>
            <fbc:geneProductAssociation>
                  <fbc:geneProductRef fbc:geneProduct="SAUSA300_2054"/>
            </fbc:geneProductAssociation>
      </reaction>

      <reaction metaid="R_5MET3OH" id="R_5MET3OH" name="5-methyl-3-oxo-
hexanoyl-ACP NADP oxidoreductase " reversible="true" fast="false"
fbc:lowerFluxBound="rev_lb" fbc:upperFluxBound="rev_ub">
            <listOfReactants>
                  <speciesReference species="M_h_c" stoichiometry="1"
constant="true"/>
```

```
                  <speciesReference species="M_nadph_c" stoichiometry="1"
constant="true"/>
                  <speciesReference species="M_5m3hhACP_c" stoichiometry="1"
constant="true"/>
            </listOfReactants>
            <listOfProducts>
                  <speciesReference species="M_nadp_c" stoichiometry="1"
constant="true"/>
                  <speciesReference species="M_5m3hACP_c" stoichiometry="1"
constant="true"/>
            </listOfProducts>
            <fbc:geneProductAssociation>
                  <fbc:or>
                        <fbc:geneProductRef fbc:geneProduct="SAUSA300_1124"/>
                        <fbc:geneProductRef fbc:geneProduct="SAUSA300_1173"/>
                  </fbc:or>
            </fbc:geneProductAssociation>
      </reaction>

      <reaction metaid="R_5MHACC" id="R_5MHACC" name="5-methyl-hexanoyl-ACP
malonyl-acyl-carrier-protein C-acyltransferase decarboxylating "
reversible="false" fast="false" fbc:lowerFluxBound="irr_lb"
fbc:upperFluxBound="irr_ub">
            <listOfReactants>
                  <speciesReference species="M_h_c" stoichiometry="1"
constant="true"/>
                  <speciesReference species="M_3mehacp_c" stoichiometry="1"
constant="true"/>
                  <speciesReference species="M_malACP_c" stoichiometry="1"
constant="true"/>
            </listOfReactants>
            <listOfProducts>
                  <speciesReference species="M_ACP_c" stoichiometry="1"
constant="true"/>
                  <speciesReference species="M_co2_c" stoichiometry="1"
constant="true"/>
                  <speciesReference species="M_7me3oxoacp_c"
stoichiometry="1" constant="true"/>
            </listOfProducts>
            <fbc:geneProductAssociation>
                  <fbc:and>
                        <fbc:geneProductRef fbc:geneProduct="SAUSA300_0886"/>
                        <fbc:geneProductRef fbc:geneProduct="SAUSA300_0885"/>
                  </fbc:and>
            </fbc:geneProductAssociation>
      </reaction>

      <reaction metaid="R_5MTH2EO" id="R_5MTH2EO" name="5-methyl-trans-hex-2-
enoyl-ACP NAD oxidoreductase A-specific " reversible="false" fast="false"
fbc:lowerFluxBound="irr_lb" fbc:upperFluxBound="irr_ub">
            <listOfReactants>
                  <speciesReference species="M_h_c" stoichiometry="1"
constant="true"/>
                  <speciesReference species="M_nadh_c" stoichiometry="1"
constant="true"/>
                  <speciesReference species="M_5mhACP_c" stoichiometry="1"
constant="true"/>
            </listOfReactants>
```

```
            <listOfProducts>
                  <speciesReference species="M_3mehacp_c" stoichiometry="1"
constant="true"/>
                  <speciesReference species="M_nad_c" stoichiometry="1"
constant="true"/>
            </listOfProducts>
            <fbc:geneProductAssociation>
                  <fbc:geneProductRef fbc:geneProduct="SAUSA300_0912"/>
            </fbc:geneProductAssociation>
      </reaction>

      <reaction metaid="R_6M3HHL" id="R_6M3HHL" name="6-methyl-3-hydroxy-
heptanoyl-ACP hydro__Lyase " reversible="true" fast="false"
fbc:lowerFluxBound="rev_lb" fbc:upperFluxBound="rev_ub">
            <listOfReactants>
                  <speciesReference species="M_6m3hhACP_c" stoichiometry="1"
constant="true"/>
            </listOfReactants>
            <listOfProducts>
                  <speciesReference species="M_h2o_c" stoichiometry="1"
constant="true"/>
                  <speciesReference species="M_6mth2eACP_c" stoichiometry="1"
constant="true"/>
            </listOfProducts>
            <fbc:geneProductAssociation>
                  <fbc:geneProductRef fbc:geneProduct="SAUSA300_2054"/>
            </fbc:geneProductAssociation>
      </reaction>

      <reaction metaid="R_6M3HOAHL" id="R_6M3HOAHL" name="6-methyl-3-hydroxy-
octanoyl-ACP hydro__Lyase " reversible="true" fast="false"
fbc:lowerFluxBound="rev_lb" fbc:upperFluxBound="rev_ub">
            <listOfReactants>
                  <speciesReference species="M_6m3hoACP_c" stoichiometry="1"
constant="true"/>
            </listOfReactants>
            <listOfProducts>
                  <speciesReference species="M_h2o_c" stoichiometry="1"
constant="true"/>
                  <speciesReference species="M_6mto2eACP_c" stoichiometry="1"
constant="true"/>
            </listOfProducts>
            <fbc:geneProductAssociation>
                  <fbc:geneProductRef fbc:geneProduct="SAUSA300_2054"/>
            </fbc:geneProductAssociation>
      </reaction>

      <reaction metaid="R_6M3OHAO" id="R_6M3OHAO" name="6-methyl-3-oxo-
heptanoyl-ACP NADP oxidoreductase " reversible="true" fast="false"
fbc:lowerFluxBound="rev_lb" fbc:upperFluxBound="rev_ub">
            <listOfReactants>
                  <speciesReference species="M_h_c" stoichiometry="1"
constant="true"/>
                  <speciesReference species="M_nadph_c" stoichiometry="1"
constant="true"/>
                  <speciesReference species="M_6m3ohACP_c" stoichiometry="1"
constant="true"/>
            </listOfReactants>
```

```
            <listOfProducts>
                  <speciesReference species="M_nadp_c" stoichiometry="1"
constant="true"/>
                  <speciesReference species="M_6m3hhACP_c" stoichiometry="1"
constant="true"/>
            </listOfProducts>
            <fbc:geneProductAssociation>
                  <fbc:or>
                        <fbc:geneProductRef fbc:geneProduct="SAUSA300_1124"/>
                        <fbc:geneProductRef fbc:geneProduct="SAUSA300_1173"/>
                  </fbc:or>
            </fbc:geneProductAssociation>
      </reaction>

      <reaction metaid="R_6M3OXO" id="R_6M3OXO" name="6-methyl-3-oxo-
octanoyl-ACP NADP oxidoreductase " reversible="true" fast="false"
fbc:lowerFluxBound="rev_lb" fbc:upperFluxBound="rev_ub">
            <listOfReactants>
                  <speciesReference species="M_h_c" stoichiometry="1"
constant="true"/>
                  <speciesReference species="M_nadph_c" stoichiometry="1"
constant="true"/>
                  <speciesReference species="M_6m3oACP_c" stoichiometry="1"
constant="true"/>
            </listOfReactants>
            <listOfProducts>
                  <speciesReference species="M_nadp_c" stoichiometry="1"
constant="true"/>
                  <speciesReference species="M_6m3hoACP_c" stoichiometry="1"
constant="true"/>
            </listOfProducts>
            <fbc:geneProductAssociation>
                  <fbc:or>
                        <fbc:geneProductRef fbc:geneProduct="SAUSA300_1124"/>
                        <fbc:geneProductRef fbc:geneProduct="SAUSA300_1173"/>
                  </fbc:or>
            </fbc:geneProductAssociation>
      </reaction>

      <reaction metaid="R_6MHACPA" id="R_6MHACPA" name="6-methyl-heptanoyl-
ACP malonyl-acyl-carrier-protein C-acyltransferase decarboxylating "
reversible="false" fast="false" fbc:lowerFluxBound="irr_lb"
fbc:upperFluxBound="irr_ub">
            <listOfReactants>
                  <speciesReference species="M_h_c" stoichiometry="1"
constant="true"/>
                  <speciesReference species="M_malACP_c" stoichiometry="1"
constant="true"/>
                  <speciesReference species="M_6mhACP_c" stoichiometry="1"
constant="true"/>
            </listOfReactants>
            <listOfProducts>
                  <speciesReference species="M_ACP_c" stoichiometry="1"
constant="true"/>
                  <speciesReference species="M_co2_c" stoichiometry="1"
constant="true"/>
                  <speciesReference species="M_8m3oxACP_c" stoichiometry="1"
constant="true"/>
```

```
            </listOfProducts>
            <fbc:geneProductAssociation>
                  <fbc:and>
                        <fbc:geneProductRef fbc:geneProduct="SAUSA300_0886"/>
                        <fbc:geneProductRef fbc:geneProduct="SAUSA300_0885"/>
                  </fbc:and>
            </fbc:geneProductAssociation>
      </reaction>

      <reaction metaid="R_6MOAMCA" id="R_6MOAMCA" name="6-methyl-octanoyl-ACP
malonyl-acyl-carrier-protein C-acyltransferase decarboxylating "
reversible="false" fast="false" fbc:lowerFluxBound="irr_lb"
fbc:upperFluxBound="irr_ub">
            <listOfReactants>
                  <speciesReference species="M_h_c" stoichiometry="1"
constant="true"/>
                  <speciesReference species="M_malACP_c" stoichiometry="1"
constant="true"/>
                  <speciesReference species="M_6moACP_c" stoichiometry="1"
constant="true"/>
            </listOfReactants>
            <listOfProducts>
                  <speciesReference species="M_ACP_c" stoichiometry="1"
constant="true"/>
                  <speciesReference species="M_co2_c" stoichiometry="1"
constant="true"/>
                  <speciesReference species="M_8m3odACP_c" stoichiometry="1"
constant="true"/>
            </listOfProducts>
            <fbc:geneProductAssociation>
                  <fbc:and>
                        <fbc:geneProductRef fbc:geneProduct="SAUSA300_0886"/>
                        <fbc:geneProductRef fbc:geneProduct="SAUSA300_0885"/>
                  </fbc:and>
            </fbc:geneProductAssociation>
      </reaction>

      <reaction metaid="R_6MTHAO" id="R_6MTHAO" name="6-methyl-trans-hept-2-
enoyl-ACP NAD oxidoreductase A-specific " reversible="false" fast="false"
fbc:lowerFluxBound="irr_lb" fbc:upperFluxBound="irr_ub">
            <listOfReactants>
                  <speciesReference species="M_h_c" stoichiometry="1"
constant="true"/>
                  <speciesReference species="M_nadh_c" stoichiometry="1"
constant="true"/>
                  <speciesReference species="M_6mth2eACP_c" stoichiometry="1"
constant="true"/>
            </listOfReactants>
            <listOfProducts>
                  <speciesReference species="M_nad_c" stoichiometry="1"
constant="true"/>
                  <speciesReference species="M_6mhACP_c" stoichiometry="1"
constant="true"/>
            </listOfProducts>
            <fbc:geneProductAssociation>
                  <fbc:geneProductRef fbc:geneProduct="SAUSA300_0912"/>
            </fbc:geneProductAssociation>
      </reaction>
```

```
      <reaction metaid="R_6MTR2EO" id="R_6MTR2EO" name="6-methyl-trans-oct-2-
enoyl-ACP NAD oxidoreductase A-specific " reversible="false" fast="false"
fbc:lowerFluxBound="irr_lb" fbc:upperFluxBound="irr_ub">
            <listOfReactants>
                  <speciesReference species="M_h_c" stoichiometry="1"
constant="true"/>
                  <speciesReference species="M_nadh_c" stoichiometry="1"
constant="true"/>
                  <speciesReference species="M_6mto2eACP_c" stoichiometry="1"
constant="true"/>
            </listOfReactants>
            <listOfProducts>
                  <speciesReference species="M_nad_c" stoichiometry="1"
constant="true"/>
                  <speciesReference species="M_6moACP_c" stoichiometry="1"
constant="true"/>
            </listOfProducts>
            <fbc:geneProductAssociation>
                  <fbc:geneProductRef fbc:geneProduct="SAUSA300_0912"/>
            </fbc:geneProductAssociation>
      </reaction>

      <reaction metaid="R_6PGALSZ" id="R_6PGALSZ" name="6-phospho-beta-
galactosidase" reversible="true" fast="false" fbc:lowerFluxBound="rev_lb"
fbc:upperFluxBound="rev_ub">
            <listOfReactants>
                  <speciesReference species="M_h2o_c" stoichiometry="1"
constant="true"/>
                  <speciesReference species="M_lac6p_c" stoichiometry="1"
constant="true"/>
            </listOfReactants>
            <listOfProducts>
                  <speciesReference species="M_dgal6p_c" stoichiometry="1"
constant="true"/>
                  <speciesReference species="M_glc__D__B_c" stoichiometry="1"
constant="true"/>
            </listOfProducts>
            <fbc:geneProductAssociation>
                  <fbc:geneProductRef fbc:geneProduct="SAUSA300_2149"/>
            </fbc:geneProductAssociation>
      </reaction>

      <reaction metaid="R_6PHBG" id="R_6PHBG" name="6-phospho-beta-
glucosidase" reversible="false" fast="false" fbc:lowerFluxBound="irr_lb"
fbc:upperFluxBound="irr_ub">
            <listOfReactants>
                  <speciesReference species="M_h2o_c" stoichiometry="1"
constant="true"/>
                  <speciesReference species="M_salc6p_c" stoichiometry="1"
constant="true"/>
            </listOfReactants>
            <listOfProducts>
                  <speciesReference species="M_g6p_c" stoichiometry="1"
constant="true"/>
                  <speciesReference species="M_2hymeph_c" stoichiometry="1"
constant="true"/>
            </listOfProducts>
```

```
            <fbc:geneProductAssociation>
                  <fbc:geneProductRef fbc:geneProduct="SAUSA300_0260"/>
            </fbc:geneProductAssociation>
      </reaction>

      <reaction metaid="R_6PHBG2" id="R_6PHBG2" name="Salicin 6-phosphate
glucohydrolase " reversible="false" fast="false" fbc:lowerFluxBound="irr_lb"
fbc:upperFluxBound="irr_ub">
            <listOfReactants>
                  <speciesReference species="M_h2o_c" stoichiometry="1"
constant="true"/>
                  <speciesReference species="M_salc6p_c" stoichiometry="1"
constant="true"/>
            </listOfReactants>
            <listOfProducts>
                  <speciesReference species="M_g6p__B_c" stoichiometry="1"
constant="true"/>
                  <speciesReference species="M_2hymeph_c" stoichiometry="1"
constant="true"/>
            </listOfProducts>
            <fbc:geneProductAssociation>
                  <fbc:geneProductRef fbc:geneProduct="SAUSA300_0260"/>
            </fbc:geneProductAssociation>
      </reaction>

      <reaction metaid="R_7M3HOACPL" id="R_7M3HOACPL" name="7-methyl-3-
hydroxy-octanoyl-ACP hydro__Lyase " reversible="true" fast="false"
fbc:lowerFluxBound="rev_lb" fbc:upperFluxBound="rev_ub">
            <listOfReactants>
                  <speciesReference species="M_7m3hoACP_c" stoichiometry="1"
constant="true"/>
            </listOfReactants>
            <listOfProducts>
                  <speciesReference species="M_h2o_c" stoichiometry="1"
constant="true"/>
                  <speciesReference species="M_7mto2eACP_c" stoichiometry="1"
constant="true"/>
            </listOfProducts>
            <fbc:geneProductAssociation>
                  <fbc:geneProductRef fbc:geneProduct="SAUSA300_2054"/>
            </fbc:geneProductAssociation>
      </reaction>

      <reaction metaid="R_7M3ODO" id="R_7M3ODO" name="7-methyl-3-oxo-
octanoyl-ACP NADP oxidoreductase " reversible="true" fast="false"
fbc:lowerFluxBound="rev_lb" fbc:upperFluxBound="rev_ub">
            <listOfReactants>
                  <speciesReference species="M_h_c" stoichiometry="1"
constant="true"/>
                  <speciesReference species="M_nadph_c" stoichiometry="1"
constant="true"/>
                  <speciesReference species="M_7me3oxoacp_c"
stoichiometry="1" constant="true"/>
            </listOfReactants>
            <listOfProducts>
                  <speciesReference species="M_nadp_c" stoichiometry="1"
constant="true"/>
```

```
                  <speciesReference species="M_7m3hoACP_c" stoichiometry="1"
constant="true"/>
            </listOfProducts>
            <fbc:geneProductAssociation>
                  <fbc:or>
                        <fbc:geneProductRef fbc:geneProduct="SAUSA300_1124"/>
                        <fbc:geneProductRef fbc:geneProduct="SAUSA300_1173"/>
                  </fbc:or>
            </fbc:geneProductAssociation>
      </reaction>

      <reaction metaid="R_7MOACA" id="R_7MOACA" name="7-methyl-octanoyl-ACP
malonyl-acyl-carrier-protein C-acyltransferase decarboxylating "
reversible="false" fast="false" fbc:lowerFluxBound="irr_lb"
fbc:upperFluxBound="irr_ub">
            <listOfReactants>
                  <speciesReference species="M_h_c" stoichiometry="1"
constant="true"/>
                  <speciesReference species="M_malACP_c" stoichiometry="1"
constant="true"/>
                  <speciesReference species="M_7moACP_c" stoichiometry="1"
constant="true"/>
            </listOfReactants>
            <listOfProducts>
                  <speciesReference species="M_ACP_c" stoichiometry="1"
constant="true"/>
                  <speciesReference species="M_co2_c" stoichiometry="1"
constant="true"/>
                  <speciesReference species="M_9m3odACP_c" stoichiometry="1"
constant="true"/>
            </listOfProducts>
            <fbc:geneProductAssociation>
                  <fbc:and>
                        <fbc:geneProductRef fbc:geneProduct="SAUSA300_0886"/>
                        <fbc:geneProductRef fbc:geneProduct="SAUSA300_0885"/>
                  </fbc:and>
            </fbc:geneProductAssociation>
      </reaction>

      <reaction metaid="R_7MTROCAC" id="R_7MTROCAC" name="7-methyl-trans-oct-
2-enoyl-ACP NAD oxidoreductase A-specific " reversible="false" fast="false"
fbc:lowerFluxBound="irr_lb" fbc:upperFluxBound="irr_ub">
            <listOfReactants>
                  <speciesReference species="M_h_c" stoichiometry="1"
constant="true"/>
                  <speciesReference species="M_nadh_c" stoichiometry="1"
constant="true"/>
                  <speciesReference species="M_7mto2eACP_c" stoichiometry="1"
constant="true"/>
            </listOfReactants>
            <listOfProducts>
                  <speciesReference species="M_nad_c" stoichiometry="1"
constant="true"/>
                  <speciesReference species="M_7moACP_c" stoichiometry="1"
constant="true"/>
            </listOfProducts>
            <fbc:geneProductAssociation>
                  <fbc:geneProductRef fbc:geneProduct="SAUSA300_0912"/>
```

```
            </fbc:geneProductAssociation>
      </reaction>

      <reaction metaid="R_8M3HDAL" id="R_8M3HDAL" name="8-methyl-3-
hydroxy__Decanoyl-ACP hydro__Lyase " reversible="true" fast="false"
fbc:lowerFluxBound="rev_lb" fbc:upperFluxBound="rev_ub">
            <listOfReactants>
                  <speciesReference species="M_8m3hdACP_c" stoichiometry="1"
constant="true"/>
            </listOfReactants>
            <listOfProducts>
                  <speciesReference species="M_h2o_c" stoichiometry="1"
constant="true"/>
                  <speciesReference species="M_8mtd2eACP_c" stoichiometry="1"
constant="true"/>
            </listOfProducts>
            <fbc:geneProductAssociation>
                  <fbc:geneProductRef fbc:geneProduct="SAUSA300_2054"/>
            </fbc:geneProductAssociation>
      </reaction>

      <reaction metaid="R_8M3HNAHL" id="R_8M3HNAHL" name="8-methyl-3-hydroxy-
nonanoyl-ACP hydro__Lyase " reversible="true" fast="false"
fbc:lowerFluxBound="rev_lb" fbc:upperFluxBound="rev_ub">
            <listOfReactants>
                  <speciesReference species="M_8m3hnACP_c" stoichiometry="1"
constant="true"/>
            </listOfReactants>
            <listOfProducts>
                  <speciesReference species="M_h2o_c" stoichiometry="1"
constant="true"/>
                  <speciesReference species="M_8mtn2eACP_c" stoichiometry="1"
constant="true"/>
            </listOfProducts>
            <fbc:geneProductAssociation>
                  <fbc:geneProductRef fbc:geneProduct="SAUSA300_2054"/>
            </fbc:geneProductAssociation>
      </reaction>

      <reaction metaid="R_8M3OAO" id="R_8M3OAO" name="8-methyl-3-
oxo__Decanoyl-ACP NADP oxidoreductase " reversible="true" fast="false"
fbc:lowerFluxBound="rev_lb" fbc:upperFluxBound="rev_ub">
            <listOfReactants>
                  <speciesReference species="M_h_c" stoichiometry="1"
constant="true"/>
                  <speciesReference species="M_nadph_c" stoichiometry="1"
constant="true"/>
                  <speciesReference species="M_8m3odACP_c" stoichiometry="1"
constant="true"/>
            </listOfReactants>
            <listOfProducts>
                  <speciesReference species="M_nadp_c" stoichiometry="1"
constant="true"/>
                  <speciesReference species="M_8m3hdACP_c" stoichiometry="1"
constant="true"/>
            </listOfProducts>
            <fbc:geneProductAssociation>
                  <fbc:or>
```

```
                        &lt;fbc:geneProductRef fbc:geneProduct="SAUSA300_1124"/&gt;
                        &lt;fbc:geneProductRef fbc:geneProduct="SAUSA300_1173"/&gt;
                  &lt;/fbc:or&gt;
            &lt;/fbc:geneProductAssociation&gt;
      &lt;/reaction&gt;

      <reaction metaid="R_8M3ONO" id="R_8M3ONO" name="8-methyl-3-oxo-
nonanoyl-ACP NADP oxidoreductase " reversible="true" fast="false"
fbc:lowerFluxBound="rev_lb" fbc:upperFluxBound="rev_ub">
            &lt;listOfReactants&gt;
                  <speciesReference species="M_h_c" stoichiometry="1"
constant="true"/>
                  <speciesReference species="M_nadph_c" stoichiometry="1"
constant="true"/>
                  <speciesReference species="M_8m3oxACP_c" stoichiometry="1"
constant="true"/>
            &lt;/listOfReactants&gt;
            &lt;listOfProducts&gt;
                  <speciesReference species="M_nadp_c" stoichiometry="1"
constant="true"/>
                  <speciesReference species="M_8m3hnACP_c" stoichiometry="1"
constant="true"/>
            &lt;/listOfProducts&gt;
            &lt;fbc:geneProductAssociation&gt;
                  &lt;fbc:or&gt;
                        &lt;fbc:geneProductRef fbc:geneProduct="SAUSA300_1124"/&gt;
                        &lt;fbc:geneProductRef fbc:geneProduct="SAUSA300_1173"/&gt;
                  &lt;/fbc:or&gt;
            &lt;/fbc:geneProductAssociation&gt;
      &lt;/reaction&gt;

      <reaction metaid="R_8MDAM" id="R_8MDAM" name="8-methyl__Decanoyl-ACP
malonyl-acyl-carrier-protein C-acyltransferase decarboxylating "
reversible="false" fast="false" fbc:lowerFluxBound="irr_lb"
fbc:upperFluxBound="irr_ub">
            &lt;listOfReactants&gt;
                  <speciesReference species="M_h_c" stoichiometry="1"
constant="true"/>
                  <speciesReference species="M_malACP_c" stoichiometry="1"
constant="true"/>
                  <speciesReference species="M_8mdACP_c" stoichiometry="1"
constant="true"/>
            &lt;/listOfReactants&gt;
            &lt;listOfProducts&gt;
                  <speciesReference species="M_ACP_c" stoichiometry="1"
constant="true"/>
                  <speciesReference species="M_co2_c" stoichiometry="1"
constant="true"/>
                  <speciesReference species="M_3oddecACP_c" stoichiometry="1"
constant="true"/>
            &lt;/listOfProducts&gt;
            &lt;fbc:geneProductAssociation&gt;
                  &lt;fbc:and&gt;
                        &lt;fbc:geneProductRef fbc:geneProduct="SAUSA300_0886"/&gt;
                        &lt;fbc:geneProductRef fbc:geneProduct="SAUSA300_0885"/&gt;
                  &lt;/fbc:and&gt;
            &lt;/fbc:geneProductAssociation&gt;
      &lt;/reaction&gt;
```

```
      <reaction metaid="R_8METNO" id="R_8METNO" name="8-methyl-trans-non-2-
enoyl-ACP NAD oxidoreductase A-specific " reversible="false" fast="false"
fbc:lowerFluxBound="irr_lb" fbc:upperFluxBound="irr_ub">
            <listOfReactants>
                  <speciesReference species="M_h_c" stoichiometry="1"
constant="true"/>
                  <speciesReference species="M_nadh_c" stoichiometry="1"
constant="true"/>
                  <speciesReference species="M_8mtn2eACP_c" stoichiometry="1"
constant="true"/>
            </listOfReactants>
            <listOfProducts>
                  <speciesReference species="M_nad_c" stoichiometry="1"
constant="true"/>
                  <speciesReference species="M_8mcACP_c" stoichiometry="1"
constant="true"/>
            </listOfProducts>
            <fbc:geneProductAssociation>
                  <fbc:geneProductRef fbc:geneProduct="SAUSA300_0912"/>
            </fbc:geneProductAssociation>
      </reaction>

      <reaction metaid="R_8MNAMC" id="R_8MNAMC" name="8-methyl-nonanoyl-ACP
malonyl-acyl-carrier-protein C-acyltransferase decarboxylating "
reversible="false" fast="false" fbc:lowerFluxBound="irr_lb"
fbc:upperFluxBound="irr_ub">
            <listOfReactants>
                  <speciesReference species="M_h_c" stoichiometry="1"
constant="true"/>
                  <speciesReference species="M_malACP_c" stoichiometry="1"
constant="true"/>
                  <speciesReference species="M_8mcACP_c" stoichiometry="1"
constant="true"/>
            </listOfReactants>
            <listOfProducts>
                  <speciesReference species="M_ACP_c" stoichiometry="1"
constant="true"/>
                  <speciesReference species="M_co2_c" stoichiometry="1"
constant="true"/>
                  <speciesReference species="M_10m3ouACP_c" stoichiometry="1"
constant="true"/>
            </listOfProducts>
            <fbc:geneProductAssociation>
                  <fbc:and>
                        <fbc:geneProductRef fbc:geneProduct="SAUSA300_0886"/>
                        <fbc:geneProductRef fbc:geneProduct="SAUSA300_0885"/>
                  </fbc:and>
            </fbc:geneProductAssociation>
      </reaction>

      <reaction metaid="R_8MT2ACP" id="R_8MT2ACP" name="8-methyl-trans__Dec-
2-enoyl-ACP NAD oxidoreductase A-specific " reversible="false" fast="false"
fbc:lowerFluxBound="irr_lb" fbc:upperFluxBound="irr_ub">
            <listOfReactants>
                  <speciesReference species="M_h_c" stoichiometry="1"
constant="true"/>
```

```
                  <speciesReference species="M_nadh_c" stoichiometry="1"
constant="true"/>
                  <speciesReference species="M_8mtd2eACP_c" stoichiometry="1"
constant="true"/>
            </listOfReactants>
            <listOfProducts>
                  <speciesReference species="M_nad_c" stoichiometry="1"
constant="true"/>
                  <speciesReference species="M_8mdACP_c" stoichiometry="1"
constant="true"/>
            </listOfProducts>
            <fbc:geneProductAssociation>
                  <fbc:geneProductRef fbc:geneProduct="SAUSA300_0912"/>
            </fbc:geneProductAssociation>
      </reaction>

      <reaction metaid="R_9M3HDL" id="R_9M3HDL" name="9-methyl-3-
hydroxy__Decanoyl-ACP hydro__Lyase " reversible="true" fast="false"
fbc:lowerFluxBound="rev_lb" fbc:upperFluxBound="rev_ub">
            <listOfReactants>
                  <speciesReference species="M_9m3hdACP_c" stoichiometry="1"
constant="true"/>
            </listOfReactants>
            <listOfProducts>
                  <speciesReference species="M_h2o_c" stoichiometry="1"
constant="true"/>
                  <speciesReference species="M_9mtd2eACP_c" stoichiometry="1"
constant="true"/>
            </listOfProducts>
            <fbc:geneProductAssociation>
                  <fbc:geneProductRef fbc:geneProduct="SAUSA300_2054"/>
            </fbc:geneProductAssociation>
      </reaction>

      <reaction metaid="R_9M3OXPA" id="R_9M3OXPA" name="9-methyl-3-
oxo__Decanoyl-ACP NADP oxidoreductase " reversible="true" fast="false"
fbc:lowerFluxBound="rev_lb" fbc:upperFluxBound="rev_ub">
            <listOfReactants>
                  <speciesReference species="M_h_c" stoichiometry="1"
constant="true"/>
                  <speciesReference species="M_nadph_c" stoichiometry="1"
constant="true"/>
                  <speciesReference species="M_9m3odACP_c" stoichiometry="1"
constant="true"/>
            </listOfReactants>
            <listOfProducts>
                  <speciesReference species="M_nadp_c" stoichiometry="1"
constant="true"/>
                  <speciesReference species="M_9m3hdACP_c" stoichiometry="1"
constant="true"/>
            </listOfProducts>
            <fbc:geneProductAssociation>
                  <fbc:or>
                        <fbc:geneProductRef fbc:geneProduct="SAUSA300_1124"/>
                        <fbc:geneProductRef fbc:geneProduct="SAUSA300_1173"/>
                  </fbc:or>
            </fbc:geneProductAssociation>
      </reaction>
```

```
      <reaction metaid="R_9MDACP" id="R_9MDACP" name="9-methyl-trans__Dec-2-
enoyl-ACP NAD oxidoreductase A-specific " reversible="false" fast="false"
fbc:lowerFluxBound="irr_lb" fbc:upperFluxBound="irr_ub">
            <listOfReactants>
                  <speciesReference species="M_h_c" stoichiometry="1"
constant="true"/>
                  <speciesReference species="M_nadh_c" stoichiometry="1"
constant="true"/>
                  <speciesReference species="M_9mtd2eACP_c" stoichiometry="1"
constant="true"/>
            </listOfReactants>
            <listOfProducts>
                  <speciesReference species="M_nad_c" stoichiometry="1"
constant="true"/>
                  <speciesReference species="M_9mdACP_c" stoichiometry="1"
constant="true"/>
            </listOfProducts>
            <fbc:geneProductAssociation>
                  <fbc:geneProductRef fbc:geneProduct="SAUSA300_0912"/>
            </fbc:geneProductAssociation>
      </reaction>

      <reaction metaid="R_9MDAM" id="R_9MDAM" name="9-methyl__Decanoyl-ACP
malonyl-acyl-carrier-protein C-acyltransferase decarboxylating "
reversible="false" fast="false" fbc:lowerFluxBound="irr_lb"
fbc:upperFluxBound="irr_ub">
            <listOfReactants>
                  <speciesReference species="M_h_c" stoichiometry="1"
constant="true"/>
                  <speciesReference species="M_malACP_c" stoichiometry="1"
constant="true"/>
                  <speciesReference species="M_9mdACP_c" stoichiometry="1"
constant="true"/>
            </listOfReactants>
            <listOfProducts>
                  <speciesReference species="M_ACP_c" stoichiometry="1"
constant="true"/>
                  <speciesReference species="M_co2_c" stoichiometry="1"
constant="true"/>
                  <speciesReference species="M_11m3odACP_c" stoichiometry="1"
constant="true"/>
            </listOfProducts>
            <fbc:geneProductAssociation>
                  <fbc:and>
                        <fbc:geneProductRef fbc:geneProduct="SAUSA300_0886"/>
                        <fbc:geneProductRef fbc:geneProduct="SAUSA300_0885"/>
                  </fbc:and>
            </fbc:geneProductAssociation>
      </reaction>

      <reaction metaid="R_AABTN" id="R_AABTN" name="4-acetamidobutyrate
deacetylase" reversible="false" fast="false" fbc:lowerFluxBound="irr_lb"
fbc:upperFluxBound="irr_ub">
            <listOfReactants>
                  <speciesReference species="M_h2o_c" stoichiometry="1"
constant="true"/>
```

```
                  <speciesReference species="M_4aabutn_c" stoichiometry="1"
constant="true"/>
            </listOfReactants>
            <listOfProducts>
                  <speciesReference species="M_ac_c" stoichiometry="1"
constant="true"/>
                  <speciesReference species="M_4abut_c" stoichiometry="1"
constant="true"/>
            </listOfProducts>
            <fbc:geneProductAssociation>
                  <fbc:and>
                        <fbc:geneProductRef fbc:geneProduct="SAUSA300_0170"/>
                        <fbc:geneProductRef fbc:geneProduct="SAUSA300_1901"/>
                        <fbc:geneProductRef fbc:geneProduct="SAUSA300_2076"/>
                  </fbc:and>
            </fbc:geneProductAssociation>
      </reaction>

      <reaction metaid="R_AACLAM" id="R_AACLAM" name="Acetyl adenylate CoA
ligase AMP-forming " reversible="false" fast="false"
fbc:lowerFluxBound="irr_lb" fbc:upperFluxBound="irr_ub">
            <listOfReactants>
                  <speciesReference species="M_coa_c" stoichiometry="1"
constant="true"/>
                  <speciesReference species="M_acadl_c" stoichiometry="1"
constant="true"/>
            </listOfReactants>
            <listOfProducts>
                  <speciesReference species="M_amp_c" stoichiometry="1"
constant="true"/>
                  <speciesReference species="M_accoa_c" stoichiometry="1"
constant="true"/>
            </listOfProducts>
            <fbc:geneProductAssociation>
                  <fbc:or>
                        <fbc:geneProductRef fbc:geneProduct="SAUSA300_2542"/>
                        <fbc:geneProductRef fbc:geneProduct="SAUSA300_1679"/>
                  </fbc:or>
            </fbc:geneProductAssociation>
      </reaction>

      <reaction metaid="R_AB6PGH" id="R_AB6PGH" name="Arbutin 6-phosphate
glucohydrolase" reversible="false" fast="false" fbc:lowerFluxBound="irr_lb"
fbc:upperFluxBound="irr_ub">
            <listOfReactants>
                  <speciesReference species="M_h2o_c" stoichiometry="1"
constant="true"/>
                  <speciesReference species="M_arbt6p_c" stoichiometry="1"
constant="true"/>
            </listOfReactants>
            <listOfProducts>
                  <speciesReference species="M_g6p__B_c" stoichiometry="1"
constant="true"/>
                  <speciesReference species="M_hqn_c" stoichiometry="1"
constant="true"/>
            </listOfProducts>
            <fbc:geneProductAssociation>
                  <fbc:geneProductRef fbc:geneProduct="SAUSA300_0260"/>
```

```
            </fbc:geneProductAssociation>
      </reaction>

      <reaction metaid="R_ABT_Et" id="R_ABT_Et" name="L-Arabinitol transport
" reversible="true" fast="false" fbc:lowerFluxBound="rev_lb"
fbc:upperFluxBound="rev_ub">
            <listOfReactants>
                  <speciesReference species="M_abt_e" stoichiometry="1"
constant="true"/>
            </listOfReactants>
            <listOfProducts>
                  <speciesReference species="M_abt_c" stoichiometry="1"
constant="true"/>
            </listOfProducts>
      </reaction>

      <reaction metaid="R_ABTA" id="R_ABTA" name="4-aminobutyrate
transaminase" reversible="false" fast="false" fbc:lowerFluxBound="irr_lb"
fbc:upperFluxBound="irr_ub">
            <listOfReactants>
                  <speciesReference species="M_akg_c" stoichiometry="1"
constant="true"/>
                  <speciesReference species="M_4abut_c" stoichiometry="1"
constant="true"/>
            </listOfReactants>
            <listOfProducts>
                  <speciesReference species="M_glu__L_c" stoichiometry="1"
constant="true"/>
                  <speciesReference species="M_sucsal_c" stoichiometry="1"
constant="true"/>
            </listOfProducts>
            <fbc:geneProductAssociation>
                  <fbc:geneProductRef fbc:geneProduct="SAUSA300_2539"/>
            </fbc:geneProductAssociation>
      </reaction>

      <reaction metaid="R_ABTAr" id="R_ABTAr" name="4-aminobutyrate
transaminase" reversible="false" fast="false" fbc:lowerFluxBound="irr_lb"
fbc:upperFluxBound="irr_ub">
            <listOfReactants>
                  <speciesReference species="M_akg_c" stoichiometry="1"
constant="true"/>
                  <speciesReference species="M_4abut_c" stoichiometry="1"
constant="true"/>
            </listOfReactants>
            <listOfProducts>
                  <speciesReference species="M_glu__L_c" stoichiometry="1"
constant="true"/>
                  <speciesReference species="M_sucsal_c" stoichiometry="1"
constant="true"/>
            </listOfProducts>
            <fbc:geneProductAssociation>
                  <fbc:geneProductRef fbc:geneProduct="SAUSA300_2539"/>
            </fbc:geneProductAssociation>
      </reaction>
```

```
      <reaction metaid="R_ABUTD" id="R_ABUTD" name="Aminobutyraldehyde
dehydrogenase" reversible="false" fast="false" fbc:lowerFluxBound="irr_lb"
fbc:upperFluxBound="irr_ub">
            &lt;listOfReactants&gt;
                  <speciesReference species="M_h2o_c" stoichiometry="1"
constant="true"/>
                  <speciesReference species="M_nad_c" stoichiometry="1"
constant="true"/>
                  <speciesReference species="M_4abutn_c" stoichiometry="1"
constant="true"/>
            &lt;/listOfReactants&gt;
            &lt;listOfProducts&gt;
                  <speciesReference species="M_h_c" stoichiometry="2"
constant="true"/>
                  <speciesReference species="M_nadh_c" stoichiometry="1"
constant="true"/>
                  <speciesReference species="M_4abut_c" stoichiometry="1"
constant="true"/>
            &lt;/listOfProducts&gt;
            &lt;fbc:geneProductAssociation&gt;
                  &lt;fbc:or&gt;
                        &lt;fbc:geneProductRef fbc:geneProduct="SAUSA300_1901"/&gt;
                        &lt;fbc:geneProductRef fbc:geneProduct="SAUSA300_2076"/&gt;
                  &lt;/fbc:or&gt;
            &lt;/fbc:geneProductAssociation&gt;
      &lt;/reaction&gt;

      <reaction metaid="R_ABUTDy" id="R_ABUTDy" name="4-Aminobutyraldehyde
NAD oxidoreductase " reversible="false" fast="false"
fbc:lowerFluxBound="irr_lb" fbc:upperFluxBound="irr_ub">
            &lt;listOfReactants&gt;
                  <speciesReference species="M_h2o_c" stoichiometry="1"
constant="true"/>
                  <speciesReference species="M_nadp_c" stoichiometry="1"
constant="true"/>
                  <speciesReference species="M_4abutn_c" stoichiometry="1"
constant="true"/>
            &lt;/listOfReactants&gt;
            &lt;listOfProducts&gt;
                  <speciesReference species="M_h_c" stoichiometry="2"
constant="true"/>
                  <speciesReference species="M_nadph_c" stoichiometry="1"
constant="true"/>
                  <speciesReference species="M_4abut_c" stoichiometry="1"
constant="true"/>
            &lt;/listOfProducts&gt;
            &lt;fbc:geneProductAssociation&gt;
                  &lt;fbc:or&gt;
                        &lt;fbc:geneProductRef fbc:geneProduct="SAUSA300_1901"/&gt;
                        &lt;fbc:geneProductRef fbc:geneProduct="SAUSA300_2076"/&gt;
                  &lt;/fbc:or&gt;
            &lt;/fbc:geneProductAssociation&gt;
      &lt;/reaction&gt;

      <reaction metaid="R_ACACT1r" id="R_ACACT1r" name="acetyl-CoA C-
acetyltransferase" reversible="true" fast="false" fbc:lowerFluxBound="rev_lb"
fbc:upperFluxBound="rev_ub">
            &lt;listOfReactants&gt;
```

```
                  <speciesReference species="M_accoa_c" stoichiometry="2"
constant="true"/>
            </listOfReactants>
            <listOfProducts>
                  <speciesReference species="M_coa_c" stoichiometry="1"
constant="true"/>
                  <speciesReference species="M_aacoa_c" stoichiometry="1"
constant="true"/>
            </listOfProducts>
            <fbc:geneProductAssociation>
                  <fbc:or>
                        <fbc:geneProductRef fbc:geneProduct="SAUSA300_0355"/>
                        <fbc:geneProductRef fbc:geneProduct="SAUSA300_0560"/>
                        <fbc:geneProductRef fbc:geneProduct="SAUSA300_0225"/>
                  </fbc:or>
            </fbc:geneProductAssociation>
      </reaction>

      <reaction metaid="R_ACACT2r" id="R_ACACT2r" name="acetyl-CoA C-
acyltransferase (butanoyl-CoA) (r)" reversible="true" fast="false"
fbc:lowerFluxBound="rev_lb" fbc:upperFluxBound="rev_ub">
            <listOfReactants>
                  <speciesReference species="M_accoa_c" stoichiometry="1"
constant="true"/>
                  <speciesReference species="M_btcoa_c" stoichiometry="1"
constant="true"/>
            </listOfReactants>
            <listOfProducts>
                  <speciesReference species="M_coa_c" stoichiometry="1"
constant="true"/>
                  <speciesReference species="M_3ohcoa_c" stoichiometry="1"
constant="true"/>
            </listOfProducts>
            <fbc:geneProductAssociation>
                  <fbc:or>
                        <fbc:geneProductRef fbc:geneProduct="SAUSA300_0355"/>
                        <fbc:geneProductRef fbc:geneProduct="SAUSA300_0560"/>
                  </fbc:or>
            </fbc:geneProductAssociation>
      </reaction>

      <reaction metaid="R_ACACT3r" id="R_ACACT3r" name="acetyl-CoA C-
acyltransferase (hexanoyl-CoA) (r)" reversible="true" fast="false"
fbc:lowerFluxBound="rev_lb" fbc:upperFluxBound="rev_ub">
            <listOfReactants>
                  <speciesReference species="M_accoa_c" stoichiometry="1"
constant="true"/>
                  <speciesReference species="M_hxcoa_c" stoichiometry="1"
constant="true"/>
            </listOfReactants>
            <listOfProducts>
                  <speciesReference species="M_coa_c" stoichiometry="1"
constant="true"/>
                  <speciesReference species="M_3oocoa_c" stoichiometry="1"
constant="true"/>
            </listOfProducts>
            <fbc:geneProductAssociation>
                  <fbc:or>
```

```
                        <fbc:geneProductRef fbc:geneProduct="SAUSA300_0225"/>
                        <fbc:geneProductRef fbc:geneProduct="SAUSA300_0560"/>
                  </fbc:or>
            </fbc:geneProductAssociation>
      </reaction>

      <reaction metaid="R_ACACT5r" id="R_ACACT5r" name="acetyl-CoA C-
acyltransferase (decanoyl-CoA) (r)" reversible="true" fast="false"
fbc:lowerFluxBound="rev_lb" fbc:upperFluxBound="rev_ub">
            <listOfReactants>
                  <speciesReference species="M_accoa_c" stoichiometry="1"
constant="true"/>
                  <speciesReference species="M_dcacoa_c" stoichiometry="1"
constant="true"/>
            </listOfReactants>
            <listOfProducts>
                  <speciesReference species="M_coa_c" stoichiometry="1"
constant="true"/>
                  <speciesReference species="M_3oddcoa_c" stoichiometry="1"
constant="true"/>
            </listOfProducts>
            <fbc:geneProductAssociation>
                  <fbc:or>
                        <fbc:geneProductRef fbc:geneProduct="SAUSA300_0225"/>
                        <fbc:geneProductRef fbc:geneProduct="SAUSA300_0560"/>
                  </fbc:or>
            </fbc:geneProductAssociation>
      </reaction>

      <reaction metaid="R_ACACT7r" id="R_ACACT7r" name="acetyl-CoA C-
acyltransferase (tetradecanoyl-CoA) (r)" reversible="true" fast="false"
fbc:lowerFluxBound="rev_lb" fbc:upperFluxBound="rev_ub">
            <listOfReactants>
                  <speciesReference species="M_accoa_c" stoichiometry="1"
constant="true"/>
                  <speciesReference species="M_tdcoa_c" stoichiometry="1"
constant="true"/>
            </listOfReactants>
            <listOfProducts>
                  <speciesReference species="M_coa_c" stoichiometry="1"
constant="true"/>
                  <speciesReference species="M_3ohdcoa_c" stoichiometry="1"
constant="true"/>
            </listOfProducts>
            <fbc:geneProductAssociation>
                  <fbc:or>
                        <fbc:geneProductRef fbc:geneProduct="SAUSA300_0225"/>
                        <fbc:geneProductRef fbc:geneProduct="SAUSA300_0560"/>
                  </fbc:or>
            </fbc:geneProductAssociation>
      </reaction>

      <reaction metaid="R_ACALD" id="R_ACALD" name="acetaldehyde
dehydrogenase (acetylating)" reversible="false" fast="false"
fbc:lowerFluxBound="irr_lb" fbc:upperFluxBound="irr_ub">
            <listOfReactants>
                  <speciesReference species="M_nad_c" stoichiometry="1"
constant="true"/>
```

```
                  <speciesReference species="M_acald_c" stoichiometry="1"
constant="true"/>
                  <speciesReference species="M_coa_c" stoichiometry="1"
constant="true"/>
            </listOfReactants>
            <listOfProducts>
                  <speciesReference species="M_h_c" stoichiometry="1"
constant="true"/>
                  <speciesReference species="M_nadh_c" stoichiometry="1"
constant="true"/>
                  <speciesReference species="M_accoa_c" stoichiometry="1"
constant="true"/>
            </listOfProducts>
            <fbc:geneProductAssociation>
                  <fbc:geneProductRef fbc:geneProduct="SAUSA300_0151"/>
            </fbc:geneProductAssociation>
      </reaction>

      <reaction metaid="R_ACCO2L" id="R_ACCO2L" name="Acetyl-CoA
carbon__Dioxide ligase ADP-forming " reversible="true" fast="false"
fbc:lowerFluxBound="rev_lb" fbc:upperFluxBound="rev_ub">
            <listOfReactants>
                  <speciesReference species="M_accoa_c" stoichiometry="1"
constant="true"/>
                  <speciesReference species="M_carP_c" stoichiometry="1"
constant="true"/>
            </listOfReactants>
            <listOfProducts>
                  <speciesReference species="M_hcarb_c" stoichiometry="1"
constant="true"/>
                  <speciesReference species="M_malcoa_c" stoichiometry="1"
constant="true"/>
            </listOfProducts>
            <fbc:geneProductAssociation>
                  <fbc:and>
                        <fbc:geneProductRef fbc:geneProduct="SAUSA300_1475"/>
                        <fbc:geneProductRef fbc:geneProduct="SAUSA300_1647"/>
                        <fbc:geneProductRef fbc:geneProduct="SAUSA300_1646"/>
                        <fbc:geneProductRef fbc:geneProduct="SAUSA300_1476"/>
                        <fbc:geneProductRef fbc:geneProduct="SAUSA300_1564"/>
                  </fbc:and>
            </fbc:geneProductAssociation>
      </reaction>

      <reaction metaid="R_ACGAL_Et" id="R_ACGAL_Et" name="N-Acetyl__D-
galactosamine transport " reversible="true" fast="false"
fbc:lowerFluxBound="rev_lb" fbc:upperFluxBound="rev_ub">
            <listOfReactants>
                  <speciesReference species="M_acgal_e" stoichiometry="1"
constant="true"/>
            </listOfReactants>
            <listOfProducts>
                  <speciesReference species="M_acgal_c" stoichiometry="1"
constant="true"/>
            </listOfProducts>
      </reaction>
```

```
      <reaction metaid="R_ACGAMT" id="R_ACGAMT" name="UDP-N-
acetylglucosamine:undecaprenylphosphate N-acetylglucosamine -1-phosphate
transferase" reversible="false" fast="false" fbc:lowerFluxBound="irr_lb"
fbc:upperFluxBound="irr_ub">
            <listOfReactants>
                  <speciesReference species="M_udcpp_c" stoichiometry="1"
constant="true"/>
                  <speciesReference species="M_uacgam_c" stoichiometry="1"
constant="true"/>
            </listOfReactants>
            <listOfProducts>
                  <speciesReference species="M_ump_c" stoichiometry="1"
constant="true"/>
                  <speciesReference species="M_unaga_c" stoichiometry="1"
constant="true"/>
            </listOfProducts>
            <fbc:geneProductAssociation>
                  <fbc:geneProductRef fbc:geneProduct="SAUSA300_0731"/>
            </fbc:geneProductAssociation>
      </reaction>

      <reaction metaid="R_ACGApts" id="R_ACGApts" name="N-Acetyl__D-
glucosamine transport via PEP:Pyr PTS" reversible="true" fast="false"
fbc:lowerFluxBound="rev_lb" fbc:upperFluxBound="rev_ub">
            <listOfReactants>
                  <speciesReference species="M_pep_c" stoichiometry="1"
constant="true"/>
                  <speciesReference species="M_acgam_e" stoichiometry="1"
constant="true"/>
            </listOfReactants>
            <listOfProducts>
                  <speciesReference species="M_pyr_c" stoichiometry="1"
constant="true"/>
                  <speciesReference species="M_acgam6p_c" stoichiometry="1"
constant="true"/>
            </listOfProducts>
            <fbc:geneProductAssociation>
                  <fbc:and>
                        <fbc:geneProductRef fbc:geneProduct="SAUSA300_0984"/>
                        <fbc:geneProductRef fbc:geneProduct="SAUSA300_0259"/>
                        <fbc:geneProductRef fbc:geneProduct="SAUSA300_0191"/>
                        <fbc:geneProductRef fbc:geneProduct="SAUSA300_1672"/>
                        <fbc:geneProductRef fbc:geneProduct="SAUSA300_0983"/>
                  </fbc:and>
            </fbc:geneProductAssociation>
      </reaction>

      <reaction metaid="R_ACGK" id="R_ACGK" name="acetylglutamate kinase"
reversible="false" fast="false" fbc:lowerFluxBound="irr_lb"
fbc:upperFluxBound="irr_ub">
            <listOfReactants>
                  <speciesReference species="M_h_c" stoichiometry="1"
constant="true"/>
                  <speciesReference species="M_atp_c" stoichiometry="1"
constant="true"/>
                  <speciesReference species="M_acglu_c" stoichiometry="1"
constant="true"/>
            </listOfReactants>
```

```
            <listOfProducts>
                  <speciesReference species="M_adp_c" stoichiometry="1"
constant="true"/>
                  <speciesReference species="M_acg5p_c" stoichiometry="1"
constant="true"/>
            </listOfProducts>
            <fbc:geneProductAssociation>
                  <fbc:geneProductRef fbc:geneProduct="SAUSA300_0184"/>
            </fbc:geneProductAssociation>
      </reaction>

      <reaction metaid="R_ACGS" id="R_ACGS" name="N-acetylglutamate synthase"
reversible="false" fast="false" fbc:lowerFluxBound="irr_lb"
fbc:upperFluxBound="irr_ub">
            <listOfReactants>
                  <speciesReference species="M_glu__L_c" stoichiometry="1"
constant="true"/>
                  <speciesReference species="M_accoa_c" stoichiometry="1"
constant="true"/>
            </listOfReactants>
            <listOfProducts>
                  <speciesReference species="M_h_c" stoichiometry="1"
constant="true"/>
                  <speciesReference species="M_coa_c" stoichiometry="1"
constant="true"/>
                  <speciesReference species="M_acglu_c" stoichiometry="1"
constant="true"/>
            </listOfProducts>
            <fbc:geneProductAssociation>
                  <fbc:geneProductRef fbc:geneProduct="SAUSA300_0185"/>
            </fbc:geneProductAssociation>
      </reaction>

      <reaction metaid="R_ACHBS" id="R_ACHBS" name="2-aceto-2-
hydroxybutanoate synthase" reversible="false" fast="false"
fbc:lowerFluxBound="irr_lb" fbc:upperFluxBound="irr_ub">
            <listOfReactants>
                  <speciesReference species="M_h_c" stoichiometry="1"
constant="true"/>
                  <speciesReference species="M_2obut_c" stoichiometry="1"
constant="true"/>
                  <speciesReference species="M_pyr_c" stoichiometry="1"
constant="true"/>
            </listOfReactants>
            <listOfProducts>
                  <speciesReference species="M_co2_c" stoichiometry="1"
constant="true"/>
                  <speciesReference species="M_2ahbut_c" stoichiometry="1"
constant="true"/>
            </listOfProducts>
            <fbc:geneProductAssociation>
                  <fbc:and>
                        <fbc:geneProductRef fbc:geneProduct="SAUSA300_2008"/>
                        <fbc:or>
                              <fbc:geneProductRef
fbc:geneProduct="SAUSA300_2007"/>
                              <fbc:geneProductRef
fbc:geneProduct="SAUSA300_2166"/>
```

```
                        </fbc:or>
                    </fbc:and>
                </fbc:geneProductAssociation>
            </reaction>

            <reaction metaid="R_ACKr" id="R_ACKr" name="acetate kinase"
reversible="true" fast="false" fbc:lowerFluxBound="rev_lb"
fbc:upperFluxBound="rev_ub">
                <listOfReactants>
                    <speciesReference species="M_h_c" stoichiometry="1"
constant="true"/>
                    <speciesReference species="M_ac_c" stoichiometry="1"
constant="true"/>
                    <speciesReference species="M_atp_c" stoichiometry="1"
constant="true"/>
                </listOfReactants>
                <listOfProducts>
                    <speciesReference species="M_adp_c" stoichiometry="1"
constant="true"/>
                    <speciesReference species="M_actp_c" stoichiometry="1"
constant="true"/>
                </listOfProducts>
                <fbc:geneProductAssociation>
                    <fbc:geneProductRef fbc:geneProduct="SAUSA300_1657"/>
                </fbc:geneProductAssociation>
            </reaction>

            <reaction metaid="R_ACLDC" id="R_ACLDC" name="acetolactate
decarboxylase" reversible="false" fast="false" fbc:lowerFluxBound="irr_lb"
fbc:upperFluxBound="irr_ub">
                <listOfReactants>
                    <speciesReference species="M_h_c" stoichiometry="1"
constant="true"/>
                    <speciesReference species="M_alac__S_c" stoichiometry="1"
constant="true"/>
                </listOfReactants>
                <listOfProducts>
                    <speciesReference species="M_co2_c" stoichiometry="1"
constant="true"/>
                    <speciesReference species="M_actn__R_c" stoichiometry="1"
constant="true"/>
                </listOfProducts>
                <fbc:geneProductAssociation>
                    <fbc:or>
                        <fbc:geneProductRef fbc:geneProduct="SAUSA300_2536"/>
                        <fbc:geneProductRef fbc:geneProduct="SAUSA300_2165"/>
                    </fbc:or>
                </fbc:geneProductAssociation>
            </reaction>

            <reaction metaid="R_ACLS" id="R_ACLS" name="acetolactate synthase"
reversible="false" fast="false" fbc:lowerFluxBound="irr_lb"
fbc:upperFluxBound="irr_ub">
                <listOfReactants>
                    <speciesReference species="M_h_c" stoichiometry="1"
constant="true"/>
                    <speciesReference species="M_pyr_c" stoichiometry="2"
constant="true"/>
```

```
            </listOfReactants>
            <listOfProducts>
                  <speciesReference species="M_co2_c" stoichiometry="1"
constant="true"/>
                  <speciesReference species="M_alac__S_c" stoichiometry="1"
constant="true"/>
            </listOfProducts>
            <fbc:geneProductAssociation>
                  <fbc:and>
                        <fbc:geneProductRef fbc:geneProduct="SAUSA300_2008"/>
                        <fbc:or>
                              <fbc:geneProductRef
fbc:geneProduct="SAUSA300_2007"/>
                              <fbc:geneProductRef
fbc:geneProduct="SAUSA300_2166"/>
                        </fbc:or>
                  </fbc:and>
            </fbc:geneProductAssociation>
      </reaction>

      <reaction metaid="R_ACMANAptspp" id="R_ACMANAptspp" name="N-acetyl__D-
mannosamine transport via PTS  (periplasm)" reversible="false" fast="false"
fbc:lowerFluxBound="irr_lb" fbc:upperFluxBound="irr_ub">
            <listOfReactants>
                  <speciesReference species="M_pep_c" stoichiometry="1"
constant="true"/>
                  <speciesReference species="M_acmana_p" stoichiometry="1"
constant="true"/>
            </listOfReactants>
            <listOfProducts>
                  <speciesReference species="M_pyr_c" stoichiometry="1"
constant="true"/>
                  <speciesReference species="M_acmanap_c" stoichiometry="1"
constant="true"/>
            </listOfProducts>
            <fbc:geneProductAssociation>
                  <fbc:and>
                        <fbc:geneProductRef fbc:geneProduct="SAUSA300_0983"/>
                        <fbc:geneProductRef fbc:geneProduct="SAUSA300_0984"/>
                  </fbc:and>
            </fbc:geneProductAssociation>
      </reaction>

      <reaction metaid="R_ACMANAtex" id="R_ACMANAtex" name="N-acetyl__D-
mannosamine transport via diffusion (extracellular to periplasm)"
reversible="true" fast="false" fbc:lowerFluxBound="rev_lb"
fbc:upperFluxBound="rev_ub">
            <listOfReactants>
                  <speciesReference species="M_acmana_e" stoichiometry="1"
constant="true"/>
            </listOfReactants>
            <listOfProducts>
                  <speciesReference species="M_acmana_p" stoichiometry="1"
constant="true"/>
            </listOfProducts>
      </reaction>
```

```
      <reaction metaid="R_ACNMLr" id="R_ACNMLr" name="N-Acetylneuraminate
lyase (reversible)" reversible="true" fast="false"
fbc:lowerFluxBound="rev_lb" fbc:upperFluxBound="rev_ub">
            <listOfReactants>
                  <speciesReference species="M_acnam_c" stoichiometry="1"
constant="true"/>
            </listOfReactants>
            <listOfProducts>
                  <speciesReference species="M_pyr_c" stoichiometry="1"
constant="true"/>
                  <speciesReference species="M_acmana_c" stoichiometry="1"
constant="true"/>
            </listOfProducts>
            <fbc:geneProductAssociation>
                  <fbc:geneProductRef fbc:geneProduct="SAUSA300_0315"/>
            </fbc:geneProductAssociation>
      </reaction>

      <reaction metaid="R_ACOAD1" id="R_ACOAD1" name="acyl-CoA dehydrogenase
(butanoyl-CoA)" reversible="false" fast="false" fbc:lowerFluxBound="irr_lb"
fbc:upperFluxBound="irr_ub">
            <listOfReactants>
                  <speciesReference species="M_h_c" stoichiometry="1"
constant="true"/>
                  <speciesReference species="M_b2coa_c" stoichiometry="1"
constant="true"/>
                  <speciesReference species="M_nadh_c" stoichiometry="1"
constant="true"/>
            </listOfReactants>
            <listOfProducts>
                  <speciesReference species="M_nad_c" stoichiometry="1"
constant="true"/>
                  <speciesReference species="M_btcoa_c" stoichiometry="1"
constant="true"/>
            </listOfProducts>
            <fbc:geneProductAssociation>
                  <fbc:or>
                        <fbc:geneProductRef fbc:geneProduct="SAUSA300_0177"/>
                        <fbc:geneProductRef fbc:geneProduct="SAUSA300_2236"/>
                  </fbc:or>
            </fbc:geneProductAssociation>
      </reaction>

      <reaction metaid="R_ACOAD2" id="R_ACOAD2" name="acyl-CoA dehydrogenase
(hexanoyl-CoA)" reversible="false" fast="false" fbc:lowerFluxBound="irr_lb"
fbc:upperFluxBound="irr_ub">
            <listOfReactants>
                  <speciesReference species="M_h_c" stoichiometry="1"
constant="true"/>
                  <speciesReference species="M_nadh_c" stoichiometry="1"
constant="true"/>
                  <speciesReference species="M_hx2coa_c" stoichiometry="1"
constant="true"/>
            </listOfReactants>
            <listOfProducts>
                  <speciesReference species="M_nad_c" stoichiometry="1"
constant="true"/>
```

```
                  <speciesReference species="M_hxcoa_c" stoichiometry="1"
constant="true"/>
            &lt;/listOfProducts&gt;
            &lt;fbc:geneProductAssociation&gt;
                  &lt;fbc:geneProductRef fbc:geneProduct="SAUSA300_0177"/&gt;
            &lt;/fbc:geneProductAssociation&gt;
      &lt;/reaction&gt;

      <reaction metaid="R_ACOAD3" id="R_ACOAD3" name="acyl-CoA dehydrogenase
(octanoyl-CoA)" reversible="false" fast="false" fbc:lowerFluxBound="irr_lb"
fbc:upperFluxBound="irr_ub">
            &lt;listOfReactants&gt;
                  <speciesReference species="M_h_c" stoichiometry="1"
constant="true"/>
                  <speciesReference species="M_nadh_c" stoichiometry="1"
constant="true"/>
                  <speciesReference species="M_oc2coa_c" stoichiometry="1"
constant="true"/>
            &lt;/listOfReactants&gt;
            &lt;listOfProducts&gt;
                  <speciesReference species="M_nad_c" stoichiometry="1"
constant="true"/>
                  <speciesReference species="M_occoa_c" stoichiometry="1"
constant="true"/>
            &lt;/listOfProducts&gt;
            &lt;fbc:geneProductAssociation&gt;
                  &lt;fbc:geneProductRef fbc:geneProduct="SAUSA300_0177"/&gt;
            &lt;/fbc:geneProductAssociation&gt;
      &lt;/reaction&gt;

      <reaction metaid="R_ACOAD4" id="R_ACOAD4" name="acyl-CoA dehydrogenase
(decanoyl-CoA)" reversible="false" fast="false" fbc:lowerFluxBound="irr_lb"
fbc:upperFluxBound="irr_ub">
            &lt;listOfReactants&gt;
                  <speciesReference species="M_h_c" stoichiometry="1"
constant="true"/>
                  <speciesReference species="M_nadh_c" stoichiometry="1"
constant="true"/>
                  <speciesReference species="M_dc2coa_c" stoichiometry="1"
constant="true"/>
            &lt;/listOfReactants&gt;
            &lt;listOfProducts&gt;
                  <speciesReference species="M_nad_c" stoichiometry="1"
constant="true"/>
                  <speciesReference species="M_dcacoa_c" stoichiometry="1"
constant="true"/>
            &lt;/listOfProducts&gt;
            &lt;fbc:geneProductAssociation&gt;
                  &lt;fbc:geneProductRef fbc:geneProduct="SAUSA300_0177"/&gt;
            &lt;/fbc:geneProductAssociation&gt;
      &lt;/reaction&gt;

      <reaction metaid="R_ACOAD5" id="R_ACOAD5" name="acyl-CoA dehydrogenase
(dodecanoyl-CoA)" reversible="false" fast="false" fbc:lowerFluxBound="irr_lb"
fbc:upperFluxBound="irr_ub">
            &lt;listOfReactants&gt;
                  <speciesReference species="M_h_c" stoichiometry="1"
constant="true"/>
```

```
                  <speciesReference species="M_nadh_c" stoichiometry="1"
constant="true"/>
                  <speciesReference species="M_dd2coa_c" stoichiometry="1"
constant="true"/>
            </listOfReactants>
            <listOfProducts>
                  <speciesReference species="M_nad_c" stoichiometry="1"
constant="true"/>
                  <speciesReference species="M_ddcacoa_c" stoichiometry="1"
constant="true"/>
            </listOfProducts>
            <fbc:geneProductAssociation>
                  <fbc:geneProductRef fbc:geneProduct="SAUSA300_0177"/>
            </fbc:geneProductAssociation>
      </reaction>

      <reaction metaid="R_ACOAD6" id="R_ACOAD6" name="acyl-CoA dehydrogenase
(tetradecanoyl-CoA)" reversible="false" fast="false"
fbc:lowerFluxBound="irr_lb" fbc:upperFluxBound="irr_ub">
            <listOfReactants>
                  <speciesReference species="M_h_c" stoichiometry="1"
constant="true"/>
                  <speciesReference species="M_nadh_c" stoichiometry="1"
constant="true"/>
                  <speciesReference species="M_td2coa_c" stoichiometry="1"
constant="true"/>
            </listOfReactants>
            <listOfProducts>
                  <speciesReference species="M_nad_c" stoichiometry="1"
constant="true"/>
                  <speciesReference species="M_tdcoa_c" stoichiometry="1"
constant="true"/>
            </listOfProducts>
            <fbc:geneProductAssociation>
                  <fbc:geneProductRef fbc:geneProduct="SAUSA300_0177"/>
            </fbc:geneProductAssociation>
      </reaction>

      <reaction metaid="R_ACOAD7" id="R_ACOAD7" name="acyl-CoA dehydrogenase
(hexadecanoyl-CoA)" reversible="false" fast="false"
fbc:lowerFluxBound="irr_lb" fbc:upperFluxBound="irr_ub">
            <listOfReactants>
                  <speciesReference species="M_h_c" stoichiometry="1"
constant="true"/>
                  <speciesReference species="M_nadh_c" stoichiometry="1"
constant="true"/>
                  <speciesReference species="M_hdd2coa_c" stoichiometry="1"
constant="true"/>
            </listOfReactants>
            <listOfProducts>
                  <speciesReference species="M_nad_c" stoichiometry="1"
constant="true"/>
                  <speciesReference species="M_pmtcoa_c" stoichiometry="1"
constant="true"/>
            </listOfProducts>
            <fbc:geneProductAssociation>
                  <fbc:geneProductRef fbc:geneProduct="SAUSA300_0177"/>
            </fbc:geneProductAssociation>
```

```
      </reaction>

      <reaction metaid="R_ACOADH2" id="R_ACOADH2" name="acyl-CoA
dehydrogenase (Isobutiryl-CoA)" reversible="true" fast="false"
fbc:lowerFluxBound="rev_lb" fbc:upperFluxBound="rev_ub">
            <listOfReactants>
                  <speciesReference species="M_o2_c" stoichiometry="1"
constant="true"/>
                  <speciesReference species="M_ibcoa_c" stoichiometry="2"
constant="true"/>
            </listOfReactants>
            <listOfProducts>
                  <speciesReference species="M_h2o_c" stoichiometry="2"
constant="true"/>
                  <speciesReference species="M_2mp2coa_c" stoichiometry="2"
constant="true"/>
            </listOfProducts>
            <fbc:geneProductAssociation>
                  <fbc:geneProductRef fbc:geneProduct="SAUSA300_2236"/>
            </fbc:geneProductAssociation>
      </reaction>

      <reaction metaid="R_ACOAM" id="R_ACOAM" name="Acetate CoA ligase AMP-
forming " reversible="false" fast="false" fbc:lowerFluxBound="irr_lb"
fbc:upperFluxBound="irr_ub">
            <listOfReactants>
                  <speciesReference species="M_ppi_c" stoichiometry="1"
constant="true"/>
                  <speciesReference species="M_acadl_c" stoichiometry="1"
constant="true"/>
            </listOfReactants>
            <listOfProducts>
                  <speciesReference species="M_h_c" stoichiometry="1"
constant="true"/>
                  <speciesReference species="M_ac_c" stoichiometry="1"
constant="true"/>
                  <speciesReference species="M_atp_c" stoichiometry="1"
constant="true"/>
            </listOfProducts>
            <fbc:geneProductAssociation>
                  <fbc:or>
                        <fbc:geneProductRef fbc:geneProduct="SAUSA300_2542"/>
                        <fbc:geneProductRef fbc:geneProduct="SAUSA300_1679"/>
                  </fbc:or>
            </fbc:geneProductAssociation>
      </reaction>

      <reaction metaid="R_ACOATA" id="R_ACOATA" name="Acetyl-CoA ACP
transacylase" reversible="true" fast="false" fbc:lowerFluxBound="rev_lb"
fbc:upperFluxBound="rev_ub">
            <listOfReactants>
                  <speciesReference species="M_ACP_c" stoichiometry="1"
constant="true"/>
                  <speciesReference species="M_accoa_c" stoichiometry="1"
constant="true"/>
            </listOfReactants>
            <listOfProducts>
```

```
                  <speciesReference species="M_coa_c" stoichiometry="1"
constant="true"/>
                  <speciesReference species="M_acACP_c" stoichiometry="1"
constant="true"/>
            </listOfProducts>
            <fbc:geneProductAssociation>
                  <fbc:and>
                        <fbc:geneProductRef fbc:geneProduct="SAUSA300_0886"/>
                        <fbc:geneProductRef fbc:geneProduct="SAUSA300_0885"/>
                  </fbc:and>
            </fbc:geneProductAssociation>
      </reaction>

      <reaction metaid="R_ACODA" id="R_ACODA" name="acetylornithine
deacetylase" reversible="false" fast="false" fbc:lowerFluxBound="irr_lb"
fbc:upperFluxBound="irr_ub">
            <listOfReactants>
                  <speciesReference species="M_h2o_c" stoichiometry="1"
constant="true"/>
                  <speciesReference species="M_acorn_c" stoichiometry="1"
constant="true"/>
            </listOfReactants>
            <listOfProducts>
                  <speciesReference species="M_ac_c" stoichiometry="1"
constant="true"/>
                  <speciesReference species="M_orn_c" stoichiometry="1"
constant="true"/>
            </listOfProducts>
            <fbc:geneProductAssociation>
                  <fbc:geneProductRef fbc:geneProduct="SAUSA300_1976"/>
            </fbc:geneProductAssociation>
      </reaction>

      <reaction metaid="R_ACONTa" id="R_ACONTa" name="aconitase (half-
reaction A, Citrate hydro__Lyase)" reversible="true" fast="false"
fbc:lowerFluxBound="rev_lb" fbc:upperFluxBound="rev_ub">
            <listOfReactants>
                  <speciesReference species="M_cit_c" stoichiometry="1"
constant="true"/>
            </listOfReactants>
            <listOfProducts>
                  <speciesReference species="M_h2o_c" stoichiometry="1"
constant="true"/>
                  <speciesReference species="M_acon__C_c" stoichiometry="1"
constant="true"/>
            </listOfProducts>
            <fbc:geneProductAssociation>
                  <fbc:geneProductRef fbc:geneProduct="SAUSA300_1246"/>
            </fbc:geneProductAssociation>
      </reaction>

      <reaction metaid="R_ACONTb" id="R_ACONTb" name="aconitase (half-
reaction B, Isocitrate hydro__Lyase)" reversible="true" fast="false"
fbc:lowerFluxBound="rev_lb" fbc:upperFluxBound="rev_ub">
            <listOfReactants>
                  <speciesReference species="M_icit_c" stoichiometry="1"
constant="true"/>
            </listOfReactants>
```

```
            <listOfProducts>
                  <speciesReference species="M_h2o_c" stoichiometry="1"
constant="true"/>
                  <speciesReference species="M_acon__C_c" stoichiometry="1"
constant="true"/>
            </listOfProducts>
            <fbc:geneProductAssociation>
                  <fbc:geneProductRef fbc:geneProduct="SAUSA300_1246"/>
            </fbc:geneProductAssociation>
      </reaction>

      <reaction metaid="R_ACOTA" id="R_ACOTA" name="acetylornithine
transaminase" reversible="true" fast="false" fbc:lowerFluxBound="rev_lb"
fbc:upperFluxBound="rev_ub">
            <listOfReactants>
                  <speciesReference species="M_akg_c" stoichiometry="1"
constant="true"/>
                  <speciesReference species="M_acorn_c" stoichiometry="1"
constant="true"/>
            </listOfReactants>
            <listOfProducts>
                  <speciesReference species="M_glu__L_c" stoichiometry="1"
constant="true"/>
                  <speciesReference species="M_acg5sa_c" stoichiometry="1"
constant="true"/>
            </listOfProducts>
            <fbc:geneProductAssociation>
                  <fbc:geneProductRef fbc:geneProduct="SAUSA300_0187"/>
            </fbc:geneProductAssociation>
      </reaction>

      <reaction metaid="R_ACPpds" id="R_ACPpds" name="[acyl-carrier-protein]
phosphodiesterase" reversible="false" fast="false"
fbc:lowerFluxBound="irr_lb" fbc:upperFluxBound="irr_ub">
            <listOfReactants>
                  <speciesReference species="M_h2o_c" stoichiometry="1"
constant="true"/>
                  <speciesReference species="M_ACP_c" stoichiometry="1"
constant="true"/>
            </listOfReactants>
            <listOfProducts>
                  <speciesReference species="M_h_c" stoichiometry="1"
constant="true"/>
                  <speciesReference species="M_apoACP_c" stoichiometry="1"
constant="true"/>
                  <speciesReference species="M_pan4p_c" stoichiometry="1"
constant="true"/>
            </listOfProducts>
            <fbc:geneProductAssociation>
                  <fbc:geneProductRef fbc:geneProduct="SAUSA300_0206"/>
            </fbc:geneProductAssociation>
      </reaction>

      <reaction metaid="R_ACPS1" id="R_ACPS1" name="acyl-carrier protein
synthase" reversible="false" fast="false" fbc:lowerFluxBound="irr_lb"
fbc:upperFluxBound="irr_ub">
            <listOfReactants>
```

```
                  <speciesReference species="M_coa_c" stoichiometry="1"
constant="true"/>
                  <speciesReference species="M_apoACP_c" stoichiometry="1"
constant="true"/>
            </listOfReactants>
            <listOfProducts>
                  <speciesReference species="M_ACP_c" stoichiometry="1"
constant="true"/>
                  <speciesReference species="M_pap_c" stoichiometry="1"
constant="true"/>
            </listOfProducts>
            <fbc:geneProductAssociation>
                  <fbc:geneProductRef fbc:geneProduct="SAUSA300_2028"/>
            </fbc:geneProductAssociation>
      </reaction>

      <reaction metaid="R_ACS" id="R_ACS" name="acetyl-CoA synthetase"
reversible="false" fast="false" fbc:lowerFluxBound="irr_lb"
fbc:upperFluxBound="irr_ub">
            <listOfReactants>
                  <speciesReference species="M_h_c" stoichiometry="1"
constant="true"/>
                  <speciesReference species="M_ac_c" stoichiometry="1"
constant="true"/>
                  <speciesReference species="M_atp_c" stoichiometry="1"
constant="true"/>
                  <speciesReference species="M_coa_c" stoichiometry="1"
constant="true"/>
            </listOfReactants>
            <listOfProducts>
                  <speciesReference species="M_ppi_c" stoichiometry="1"
constant="true"/>
                  <speciesReference species="M_amp_c" stoichiometry="1"
constant="true"/>
                  <speciesReference species="M_accoa_c" stoichiometry="1"
constant="true"/>
            </listOfProducts>
            <fbc:geneProductAssociation>
                  <fbc:geneProductRef fbc:geneProduct="SAUSA300_1679"/>
            </fbc:geneProductAssociation>
      </reaction>

      <reaction metaid="R_ACS2" id="R_ACS2" name="acetyl-CoA synthase
(propionate)" reversible="false" fast="false" fbc:lowerFluxBound="irr_lb"
fbc:upperFluxBound="irr_ub">
            <listOfReactants>
                  <speciesReference species="M_coa_c" stoichiometry="1"
constant="true"/>
                  <speciesReference species="M_ppadn_c" stoichiometry="1"
constant="true"/>
            </listOfReactants>
            <listOfProducts>
                  <speciesReference species="M_amp_c" stoichiometry="1"
constant="true"/>
                  <speciesReference species="M_ppcoa_c" stoichiometry="1"
constant="true"/>
            </listOfProducts>
            <fbc:geneProductAssociation>
```

<fbc:geneProductRef fbc:geneProduct="SAUSA300_1679"/>
</fbc:geneProductAssociation>
</reaction>

<reaction metaid="R_ACSERHS" id="R_ACSERHS" name="O3-Acetyl__L-serine acetate__Lyase (adding hydrogen sulfide)" reversible="true" fast="false" fbc:lowerFluxBound="rev_lb" fbc:upperFluxBound="rev_ub">
<listOfReactants>
<speciesReference species="M_acser_c" stoichiometry="1" constant="true"/>
<speciesReference species="M_trdrd_c" stoichiometry="1" constant="true"/>
<speciesReference species="M_tsul_c" stoichiometry="1" constant="true"/>
</listOfReactants>
<listOfProducts>
<speciesReference species="M_ac_c" stoichiometry="1" constant="true"/>
<speciesReference species="M_so3_c" stoichiometry="1" constant="true"/>
<speciesReference species="M_trdox_c" stoichiometry="1" constant="true"/>
<speciesReference species="M_cys__L_c" stoichiometry="1" constant="true"/>
</listOfProducts>
<fbc:geneProductAssociation>
<fbc:geneProductRef fbc:geneProduct="SAUSA300_0491"/>
</fbc:geneProductAssociation>
</reaction>

<reaction metaid="R_ACSNG" id="R_ACSNG" name="Acyl-CoA sn-glycerol-3-phosphate 2-O-acyltransferase " reversible="true" fast="false" fbc:lowerFluxBound="rev_lb" fbc:upperFluxBound="rev_ub">
<listOfReactants>
<speciesReference species="M_glyc3p_c" stoichiometry="1" constant="true"/>
<speciesReference species="M_acoa_c" stoichiometry="1" constant="true"/>
</listOfReactants>
<listOfProducts>
<speciesReference species="M_coa_c" stoichiometry="1" constant="true"/>
<speciesReference species="M_a2as3p" stoichiometry="1" constant="true"/>
</listOfProducts>
<fbc:geneProductAssociation>
<fbc:geneProductRef fbc:geneProduct="SAUSA300_2505"/>
</fbc:geneProductAssociation>
</reaction>

<reaction metaid="R_ACTD2" id="R_ACTD2" name="Acetoin dehydrogenase" reversible="true" fast="false" fbc:lowerFluxBound="rev_lb" fbc:upperFluxBound="rev_ub">
<listOfReactants>
<speciesReference species="M_coa_c" stoichiometry="1" constant="true"/>
<speciesReference species="M_nad_c" stoichiometry="1" constant="true"/>

```
                  <speciesReference species="M_actn__R_c" stoichiometry="1"
constant="true"/>
            </listOfReactants>
            <listOfProducts>
                  <speciesReference species="M_acald_c" stoichiometry="1"
constant="true"/>
                  <speciesReference species="M_accoa_c" stoichiometry="1"
constant="true"/>
                  <speciesReference species="M_h_c" stoichiometry="1"
constant="true"/>
                  <speciesReference species="M_nadh_c" stoichiometry="1"
constant="true"/>
            </listOfProducts>
            <fbc:geneProductAssociation>
                  <fbc:or>
                        <fbc:geneProductRef fbc:geneProduct="SAUSA300_0129"/>
                        <fbc:geneProductRef fbc:geneProduct="SAUSA300_1681"/>
                  </fbc:or>
            </fbc:geneProductAssociation>
      </reaction>

      <reaction metaid="R_ACTt2r" id="R_ACTt2r" name="acetate transport"
reversible="true" fast="false" fbc:lowerFluxBound="rev_lb"
fbc:upperFluxBound="rev_ub">
            <listOfReactants>
                  <speciesReference species="M_ac_c" stoichiometry="1"
constant="true"/>
            </listOfReactants>
            <listOfProducts>
                  <speciesReference species="M_ac_e" stoichiometry="1"
constant="true"/>
            </listOfProducts>
      </reaction>

      <reaction metaid="R_ACYP_2" id="R_ACYP_2" name="acylphosphatase (2)"
reversible="false" fast="false" fbc:lowerFluxBound="irr_lb"
fbc:upperFluxBound="irr_ub">
            <listOfReactants>
                  <speciesReference species="M_h2o_c" stoichiometry="1"
constant="true"/>
                  <speciesReference species="M_actp_c" stoichiometry="1"
constant="true"/>
            </listOfReactants>
            <listOfProducts>
                  <speciesReference species="M_h_c" stoichiometry="2"
constant="true"/>
                  <speciesReference species="M_ac_c" stoichiometry="1"
constant="true"/>
                  <speciesReference species="M_pi_c" stoichiometry="1"
constant="true"/>
            </listOfProducts>
            <fbc:geneProductAssociation>
                  <fbc:geneProductRef fbc:geneProduct="SAUSA300_1297"/>
            </fbc:geneProductAssociation>
      </reaction>
```

```
      <reaction metaid="R_AD_Et" id="R_AD_Et" name="acetamide transport"
reversible="true" fast="false" fbc:lowerFluxBound="rev_lb"
fbc:upperFluxBound="rev_ub">
            <listOfReactants>
                  <speciesReference species="M_ad_e" stoichiometry="1"
constant="true"/>
            </listOfReactants>
            <listOfProducts>
                  <speciesReference species="M_ad_c" stoichiometry="1"
constant="true"/>
            </listOfProducts>
      </reaction>

      <reaction metaid="R_ADA" id="R_ADA" name="Adenosine deaminase"
reversible="false" fast="false" fbc:lowerFluxBound="irr_lb"
fbc:upperFluxBound="irr_ub">
            <listOfReactants>
                  <speciesReference species="M_h_c" stoichiometry="1"
constant="true"/>
                  <speciesReference species="M_h2o_c" stoichiometry="1"
constant="true"/>
                  <speciesReference species="M_adn_c" stoichiometry="1"
constant="true"/>
            </listOfReactants>
            <listOfProducts>
                  <speciesReference species="M_nh4_c" stoichiometry="1"
constant="true"/>
                  <speciesReference species="M_ins_c" stoichiometry="1"
constant="true"/>
            </listOfProducts>
            <fbc:geneProductAssociation>
                  <fbc:geneProductRef fbc:geneProduct="SAUSA300_0543"/>
            </fbc:geneProductAssociation>
      </reaction>

      <reaction metaid="R_ADAPAT" id="R_ADAPAT" name="e N-
acetyl__L,L__Diaminopimelate aminotransferase" reversible="true" fast="false"
fbc:lowerFluxBound="rev_lb" fbc:upperFluxBound="rev_ub">
            <listOfReactants>
                  <speciesReference species="M_n6all26d_c" stoichiometry="1"
constant="true"/>
                  <speciesReference species="M_akg_c" stoichiometry="1"
constant="true"/>
            </listOfReactants>
            <listOfProducts>
                  <speciesReference species="M_glu__L_c" stoichiometry="1"
constant="true"/>
                  <speciesReference species="M_nal2a6o_c" stoichiometry="1"
constant="true"/>
            </listOfProducts>
            <fbc:geneProductAssociation>
                  <fbc:geneProductRef fbc:geneProduct="SAUSA300_0952"/>
            </fbc:geneProductAssociation>
      </reaction>

      <reaction metaid="R_ADCL" id="R_ADCL" name="4-aminobenzoate synthase"
reversible="false" fast="false" fbc:lowerFluxBound="irr_lb"
fbc:upperFluxBound="irr_ub">
```

```
            <listOfReactants>
                  <speciesReference species="M_4adcho_c" stoichiometry="1"
constant="true"/>
            </listOfReactants>
            <listOfProducts>
                  <speciesReference species="M_h_c" stoichiometry="1"
constant="true"/>
                  <speciesReference species="M_pyr_c" stoichiometry="1"
constant="true"/>
                  <speciesReference species="M_4abz_c" stoichiometry="1"
constant="true"/>
            </listOfProducts>
            <fbc:geneProductAssociation>
                  <fbc:geneProductRef fbc:geneProduct="SAUSA300_0700"/>
            </fbc:geneProductAssociation>
      </reaction>

      <reaction metaid="R_ADCS" id="R_ADCS" name="4-amino-4__Deoxychorismate
synthase" reversible="false" fast="false" fbc:lowerFluxBound="irr_lb"
fbc:upperFluxBound="irr_ub">
            <listOfReactants>
                  <speciesReference species="M_gln__L_c" stoichiometry="1"
constant="true"/>
                  <speciesReference species="M_chor_c" stoichiometry="1"
constant="true"/>
            </listOfReactants>
            <listOfProducts>
                  <speciesReference species="M_glu__L_c" stoichiometry="1"
constant="true"/>
                  <speciesReference species="M_4adcho_c" stoichiometry="1"
constant="true"/>
            </listOfProducts>
            <fbc:geneProductAssociation>
                  <fbc:or>
                        <fbc:geneProductRef fbc:geneProduct="SAUSA300_1263"/>
                        <fbc:geneProductRef fbc:geneProduct="SAUSA300_0699"/>
                  </fbc:or>
            </fbc:geneProductAssociation>
      </reaction>

      <reaction metaid="R_ADCYRS" id="R_ADCYRS" name="adenosylcobyric acid
synthase (glutamine-hydrolysing)" reversible="false" fast="false"
fbc:lowerFluxBound="irr_lb" fbc:upperFluxBound="irr_ub">
            <listOfReactants>
                  <speciesReference species="M_h2o_c" stoichiometry="4"
constant="true"/>
                  <speciesReference species="M_gln__L_c" stoichiometry="4"
constant="true"/>
                  <speciesReference species="M_atp_c" stoichiometry="4"
constant="true"/>
                  <speciesReference species="M_adcobdam_c" stoichiometry="1"
constant="true"/>
            </listOfReactants>
            <listOfProducts>
                  <speciesReference species="M_h_c" stoichiometry="4"
constant="true"/>
                  <speciesReference species="M_glu__L_c" stoichiometry="4"
constant="true"/>
```

```
                  <speciesReference species="M_pi_c" stoichiometry="4"
constant="true"/>
                  <speciesReference species="M_adp_c" stoichiometry="4"
constant="true"/>
                  <speciesReference species="M_adcobhex_c" stoichiometry="1"
constant="true"/>
            </listOfProducts>
            <fbc:geneProductAssociation>
                  <fbc:geneProductRef fbc:geneProduct="SAUSA300_1872"/>
            </fbc:geneProductAssociation>
      </reaction>

      <reaction metaid="R_ADK1" id="R_ADK1" name="adenylate kinase"
reversible="true" fast="false" fbc:lowerFluxBound="rev_lb"
fbc:upperFluxBound="rev_ub">
            <listOfReactants>
                  <speciesReference species="M_atp_c" stoichiometry="1"
constant="true"/>
                  <speciesReference species="M_amp_c" stoichiometry="1"
constant="true"/>
            </listOfReactants>
            <listOfProducts>
                  <speciesReference species="M_adp_c" stoichiometry="2"
constant="true"/>
            </listOfProducts>
            <fbc:geneProductAssociation>
                  <fbc:geneProductRef fbc:geneProduct="SAUSA300_2183"/>
            </fbc:geneProductAssociation>
      </reaction>

      <reaction metaid="R_ADK2" id="R_ADK2" name="adenylate kinase (Inorganic
triphosphate)" reversible="true" fast="false" fbc:lowerFluxBound="rev_lb"
fbc:upperFluxBound="rev_ub">
            <listOfReactants>
                  <speciesReference species="M_h_c" stoichiometry="1"
constant="true"/>
                  <speciesReference species="M_amp_c" stoichiometry="1"
constant="true"/>
                  <speciesReference species="M_pppi_c" stoichiometry="1"
constant="true"/>
            </listOfReactants>
            <listOfProducts>
                  <speciesReference species="M_adp_c" stoichiometry="1"
constant="true"/>
                  <speciesReference species="M_ppi_c" stoichiometry="1"
constant="true"/>
            </listOfProducts>
            <fbc:geneProductAssociation>
                  <fbc:geneProductRef fbc:geneProduct="SAUSA300_2183"/>
            </fbc:geneProductAssociation>
      </reaction>

      <reaction metaid="R_ADKd" id="R_ADKd" name="adenylate kinase (d form)"
reversible="true" fast="false" fbc:lowerFluxBound="rev_lb"
fbc:upperFluxBound="rev_ub">
            <listOfReactants>
                  <speciesReference species="M_datp_c" stoichiometry="1"
constant="true"/>
```

```
                  <speciesReference species="M_damp_c" stoichiometry="1"
constant="true"/>
            </listOfReactants>
            <listOfProducts>
                  <speciesReference species="M_dadp_c" stoichiometry="2"
constant="true"/>
            </listOfProducts>
            <fbc:geneProductAssociation>
                  <fbc:geneProductRef fbc:geneProduct="SAUSA300_2183"/>
            </fbc:geneProductAssociation>
      </reaction>

      <reaction metaid="R_ADNCYC" id="R_ADNCYC" name="adenylate cyclase"
reversible="false" fast="false" fbc:lowerFluxBound="irr_lb"
fbc:upperFluxBound="irr_ub">
            <listOfReactants>
                  <speciesReference species="M_atp_c" stoichiometry="1"
constant="true"/>
            </listOfReactants>
            <listOfProducts>
                  <speciesReference species="M_ppi_c" stoichiometry="1"
constant="true"/>
                  <speciesReference species="M_camp_c" stoichiometry="1"
constant="true"/>
            </listOfProducts>
            <fbc:geneProductAssociation>
                  <fbc:geneProductRef fbc:geneProduct="SAUSA300_0905"/>
            </fbc:geneProductAssociation>
      </reaction>

      <reaction metaid="R_ADNt2" id="R_ADNt2" name="adenosine transport in
via proton symport" reversible="false" fast="false"
fbc:lowerFluxBound="irr_lb" fbc:upperFluxBound="irr_ub">
            <listOfReactants>
                  <speciesReference species="M_h_e" stoichiometry="1"
constant="true"/>
                  <speciesReference species="M_adn_e" stoichiometry="1"
constant="true"/>
            </listOfReactants>
            <listOfProducts>
                  <speciesReference species="M_h_c" stoichiometry="1"
constant="true"/>
                  <speciesReference species="M_adn_c" stoichiometry="1"
constant="true"/>
            </listOfProducts>
      </reaction>

      <reaction metaid="R_ADNUC" id="R_ADNUC" name="adenosine hydrolase"
reversible="false" fast="false" fbc:lowerFluxBound="irr_lb"
fbc:upperFluxBound="irr_ub">
            <listOfReactants>
                  <speciesReference species="M_h2o_c" stoichiometry="1"
constant="true"/>
                  <speciesReference species="M_adn_c" stoichiometry="1"
constant="true"/>
            </listOfReactants>
            <listOfProducts>
```

```
                  <speciesReference species="M_ade_c" stoichiometry="1"
constant="true"/>
                  <speciesReference species="M_rib__D_c" stoichiometry="1"
constant="true"/>
            </listOfProducts>
            <fbc:geneProductAssociation>
                  <fbc:or>
                        <fbc:geneProductRef fbc:geneProduct="SAUSA300_2234"/>
                        <fbc:geneProductRef fbc:geneProduct="SAUSA300_0237"/>
                  </fbc:or>
            </fbc:geneProductAssociation>
      </reaction>

      <reaction metaid="R_ADPRDP" id="R_ADPRDP" name="ADPribose
diphosphatase" reversible="false" fast="false" fbc:lowerFluxBound="irr_lb"
fbc:upperFluxBound="irr_ub">
            <listOfReactants>
                  <speciesReference species="M_h2o_c" stoichiometry="1"
constant="true"/>
                  <speciesReference species="M_adprib_c" stoichiometry="1"
constant="true"/>
            </listOfReactants>
            <listOfProducts>
                  <speciesReference species="M_r5p_c" stoichiometry="1"
constant="true"/>
                  <speciesReference species="M_amp_c" stoichiometry="1"
constant="true"/>
            </listOfProducts>
            <fbc:geneProductAssociation>
                  <fbc:geneProductRef fbc:geneProduct="SAUSA300_1449"/>
            </fbc:geneProductAssociation>
      </reaction>

      <reaction metaid="R_ADPT" id="R_ADPT" name="adenine
phosphoribosyltransferase" reversible="false" fast="false"
fbc:lowerFluxBound="irr_lb" fbc:upperFluxBound="irr_ub">
            <listOfReactants>
                  <speciesReference species="M_ade_c" stoichiometry="1"
constant="true"/>
                  <speciesReference species="M_prpp_c" stoichiometry="1"
constant="true"/>
            </listOfReactants>
            <listOfProducts>
                  <speciesReference species="M_ppi_c" stoichiometry="1"
constant="true"/>
                  <speciesReference species="M_amp_c" stoichiometry="1"
constant="true"/>
            </listOfProducts>
            <fbc:geneProductAssociation>
                  <fbc:geneProductRef fbc:geneProduct="SAUSA300_1591"/>
            </fbc:geneProductAssociation>
      </reaction>

      <reaction metaid="R_ADSK" id="R_ADSK" name="adenylyl-sulfate kinase"
reversible="false" fast="false" fbc:lowerFluxBound="irr_lb"
fbc:upperFluxBound="irr_ub">
            <listOfReactants>
```

```
                  <speciesReference species="M_atp_c" stoichiometry="1"
constant="true"/>
                  <speciesReference species="M_aps_c" stoichiometry="1"
constant="true"/>
            </listOfReactants>
            <listOfProducts>
                  <speciesReference species="M_h_c" stoichiometry="1"
constant="true"/>
                  <speciesReference species="M_adp_c" stoichiometry="1"
constant="true"/>
                  <speciesReference species="M_paps_c" stoichiometry="1"
constant="true"/>
            </listOfProducts>
            <fbc:geneProductAssociation>
                  <fbc:geneProductRef fbc:geneProduct="SAUSA300_2597"/>
            </fbc:geneProductAssociation>
      </reaction>

      <reaction metaid="R_ADSL1" id="R_ADSL1" name="adenylosuccinate lyase"
reversible="true" fast="false" fbc:lowerFluxBound="rev_lb"
fbc:upperFluxBound="rev_ub">
            <listOfReactants>
                  <speciesReference species="M_dcamp_c" stoichiometry="1"
constant="true"/>
            </listOfReactants>
            <listOfProducts>
                  <speciesReference species="M_fum_c" stoichiometry="1"
constant="true"/>
                  <speciesReference species="M_amp_c" stoichiometry="1"
constant="true"/>
            </listOfProducts>
            <fbc:geneProductAssociation>
                  <fbc:geneProductRef fbc:geneProduct="SAUSA300_1889"/>
            </fbc:geneProductAssociation>
      </reaction>

      <reaction metaid="R_ADSL2r" id="R_ADSL2r" name="adenylosuccinate lyase"
reversible="true" fast="false" fbc:lowerFluxBound="rev_lb"
fbc:upperFluxBound="rev_ub">
            <listOfReactants>
                  <speciesReference species="M_25aics_c" stoichiometry="1"
constant="true"/>
            </listOfReactants>
            <listOfProducts>
                  <speciesReference species="M_fum_c" stoichiometry="1"
constant="true"/>
                  <speciesReference species="M_aicar_c" stoichiometry="1"
constant="true"/>
            </listOfProducts>
            <fbc:geneProductAssociation>
                  <fbc:geneProductRef fbc:geneProduct="SAUSA300_1889"/>
            </fbc:geneProductAssociation>
      </reaction>

      <reaction metaid="R_ADSS" id="R_ADSS" name="adenylosuccinate synthase"
reversible="false" fast="false" fbc:lowerFluxBound="irr_lb"
fbc:upperFluxBound="irr_ub">
            <listOfReactants>
```

```
                  <speciesReference species="M_asp__L_c" stoichiometry="1"
constant="true"/>
                  <speciesReference species="M_imp_c" stoichiometry="1"
constant="true"/>
                  <speciesReference species="M_gtp_c" stoichiometry="1"
constant="true"/>
            </listOfReactants>
            <listOfProducts>
                  <speciesReference species="M_h_c" stoichiometry="2"
constant="true"/>
                  <speciesReference species="M_pi_c" stoichiometry="1"
constant="true"/>
                  <speciesReference species="M_gdp_c" stoichiometry="1"
constant="true"/>
                  <speciesReference species="M_dcamp_c" stoichiometry="1"
constant="true"/>
            </listOfProducts>
            <fbc:geneProductAssociation>
                  <fbc:geneProductRef fbc:geneProduct="SAUSA300_0017"/>
            </fbc:geneProductAssociation>
      </reaction>

      <reaction metaid="R_AGDC_r" id="R_AGDC_r" name="N-acetylglucosamine-6-
phosphate deacetylase (reversible)" reversible="true" fast="false"
fbc:lowerFluxBound="rev_lb" fbc:upperFluxBound="rev_ub">
            <listOfReactants>
                  <speciesReference species="M_h2o_c" stoichiometry="1"
constant="true"/>
                  <speciesReference species="M_acgam6p_c" stoichiometry="1"
constant="true"/>
            </listOfReactants>
            <listOfProducts>
                  <speciesReference species="M_ac_c" stoichiometry="1"
constant="true"/>
                  <speciesReference species="M_gam6p_c" stoichiometry="1"
constant="true"/>
            </listOfProducts>
            <fbc:geneProductAssociation>
                  <fbc:geneProductRef fbc:geneProduct="SAUSA300_0686"/>
            </fbc:geneProductAssociation>
      </reaction>

      <reaction metaid="R_AGLYPT" id="R_AGLYPT" name="anteisopentadecanoyl-
glycerol-3-phosphate O-acyltransferase " reversible="false" fast="false"
fbc:lowerFluxBound="irr_lb" fbc:upperFluxBound="irr_ub">
            <listOfReactants>
                  <speciesReference species="M_glyc3p_c" stoichiometry="1"
constant="true"/>
                  <speciesReference species="M_fa4coa_c" stoichiometry="1"
constant="true"/>
            </listOfReactants>
            <listOfProducts>
                  <speciesReference species="M_coa_c" stoichiometry="1"
constant="true"/>
                  <speciesReference species="M_1aipsg3p_c" stoichiometry="1"
constant="true"/>
            </listOfProducts>
            <fbc:geneProductAssociation>
```

```
                  <fbc:and>
                        <fbc:geneProductRef fbc:geneProduct="SAUSA300_1249"/>
                        <fbc:or>
                              <fbc:geneProductRef
fbc:geneProduct="SAUSA300_1122"/>
                              <fbc:geneProductRef
fbc:geneProduct="SAUSA300_1121"/>
                        </fbc:or>
                  </fbc:and>
            </fbc:geneProductAssociation>
      </reaction>

      <reaction metaid="R_AGMHE" id="R_AGMHE" name="ADP__D-glycero__D-manno-
heptose epimerase" reversible="false" fast="false"
fbc:lowerFluxBound="irr_lb" fbc:upperFluxBound="irr_ub">
            <listOfReactants>
                  <speciesReference species="M_adphep__DD_c"
stoichiometry="1" constant="true"/>
            </listOfReactants>
            <listOfProducts>
                  <speciesReference species="M_adphep__LD_c"
stoichiometry="1" constant="true"/>
            </listOfProducts>
            <fbc:geneProductAssociation>
                  <fbc:geneProductRef fbc:geneProduct="SAUSA300_0130"/>
            </fbc:geneProductAssociation>
      </reaction>

      <reaction metaid="R_AGPAT120" id="R_AGPAT120" name="1-tetradecanoyl-sn-
glycerol 3-phosphate O-acyltransferase (n-C12:0)" reversible="false"
fast="false" fbc:lowerFluxBound="irr_lb" fbc:upperFluxBound="irr_ub">
            <listOfReactants>
                  <speciesReference species="M_ddcaACP_c" stoichiometry="1"
constant="true"/>
                  <speciesReference species="M_1ddecg3p_c" stoichiometry="1"
constant="true"/>
            </listOfReactants>
            <listOfProducts>
                  <speciesReference species="M_ACP_c" stoichiometry="1"
constant="true"/>
                  <speciesReference species="M_pa120_c" stoichiometry="1"
constant="true"/>
            </listOfProducts>
            <fbc:geneProductAssociation>
                  <fbc:geneProductRef fbc:geneProduct="SAUSA300_1673"/>
            </fbc:geneProductAssociation>
      </reaction>

      <reaction metaid="R_AGPAT140" id="R_AGPAT140" name="1-tetradecanoyl-sn-
glycerol 3-phosphate O-acyltransferase (n-C14:0)" reversible="false"
fast="false" fbc:lowerFluxBound="irr_lb" fbc:upperFluxBound="irr_ub">
            <listOfReactants>
                  <speciesReference species="M_myrsACP_c" stoichiometry="1"
constant="true"/>
                  <speciesReference species="M_1tdecg3p_c" stoichiometry="1"
constant="true"/>
            </listOfReactants>
            <listOfProducts>
```

```
                  <speciesReference species="M_ACP_c" stoichiometry="1"
constant="true"/>
                  <speciesReference species="M_pa140_c" stoichiometry="1"
constant="true"/>
            </listOfProducts>
            <fbc:geneProductAssociation>
                  <fbc:geneProductRef fbc:geneProduct="SAUSA300_1673"/>
            </fbc:geneProductAssociation>
      </reaction>

      <reaction metaid="R_AGPAT140d" id="R_AGPAT140d" name="myristoyl-1-
acylglycerol-3-phosphate O-acyltransferase " reversible="false" fast="false"
fbc:lowerFluxBound="irr_lb" fbc:upperFluxBound="irr_ub">
            <listOfReactants>
                  <speciesReference species="M_1tdecg3p_c" stoichiometry="1"
constant="true"/>
                  <speciesReference species="M_tdcoa_c" stoichiometry="1"
constant="true"/>
            </listOfReactants>
            <listOfProducts>
                  <speciesReference species="M_pa140_c" stoichiometry="1"
constant="true"/>
                  <speciesReference species="M_coa_c" stoichiometry="1"
constant="true"/>
            </listOfProducts>
            <fbc:geneProductAssociation>
                  <fbc:geneProductRef fbc:geneProduct="SAUSA300_1673"/>
            </fbc:geneProductAssociation>
      </reaction>

      <reaction metaid="R_AGPAT141" id="R_AGPAT141" name="1-tetradec-7-enoyl-
sn-glycerol 3-phosphate O-acyltransferase (n-C14:1)" reversible="false"
fast="false" fbc:lowerFluxBound="irr_lb" fbc:upperFluxBound="irr_ub">
            <listOfReactants>
                  <speciesReference species="M_1tdec7eg3p_c"
stoichiometry="1" constant="true"/>
                  <speciesReference species="M_tdeACP_c" stoichiometry="1"
constant="true"/>
            </listOfReactants>
            <listOfProducts>
                  <speciesReference species="M_ACP_c" stoichiometry="1"
constant="true"/>
                  <speciesReference species="M_pa141_c" stoichiometry="1"
constant="true"/>
            </listOfProducts>
            <fbc:geneProductAssociation>
                  <fbc:geneProductRef fbc:geneProduct="SAUSA300_1673"/>
            </fbc:geneProductAssociation>
      </reaction>

      <reaction metaid="R_AGPAT160" id="R_AGPAT160" name="1-hexadecanoyl-sn-
glycerol 3-phosphate O-acyltransferase (n-C16:0)" reversible="false"
fast="false" fbc:lowerFluxBound="irr_lb" fbc:upperFluxBound="irr_ub">
            <listOfReactants>
                  <speciesReference species="M_palmACP_c" stoichiometry="1"
constant="true"/>
                  <speciesReference species="M_1hdecg3p_c" stoichiometry="1"
constant="true"/>
```

```
            </listOfReactants>
            <listOfProducts>
                  <speciesReference species="M_ACP_c" stoichiometry="1"
constant="true"/>
                  <speciesReference species="M_pa160_c" stoichiometry="1"
constant="true"/>
            </listOfProducts>
            <fbc:geneProductAssociation>
                  <fbc:geneProductRef fbc:geneProduct="SAUSA300_1673"/>
            </fbc:geneProductAssociation>
      </reaction>

      <reaction metaid="R_AGPAT160d" id="R_AGPAT160d" name="palmitoyl-
glycerol-3-phosphate O-acyltransferase " reversible="false" fast="false"
fbc:lowerFluxBound="irr_lb" fbc:upperFluxBound="irr_ub">
            <listOfReactants>
                  <speciesReference species="M_glyc3p_c" stoichiometry="1"
constant="true"/>
                  <speciesReference species="M_pmtcoa_c" stoichiometry="1"
constant="true"/>
            </listOfReactants>
            <listOfProducts>
                  <speciesReference species="M_coa_c" stoichiometry="1"
constant="true"/>
                  <speciesReference species="M_1hdecg3p_c" stoichiometry="1"
constant="true"/>
            </listOfProducts>
            <fbc:geneProductAssociation>
                  <fbc:and>
                        <fbc:geneProductRef fbc:geneProduct="SAUSA300_1249"/>
                        <fbc:or>
                              <fbc:geneProductRef
fbc:geneProduct="SAUSA300_1122"/>
                              <fbc:geneProductRef
fbc:geneProduct="SAUSA300_1121"/>
                        </fbc:or>
                  </fbc:and>
            </fbc:geneProductAssociation>
      </reaction>

      <reaction metaid="R_AGPAT161" id="R_AGPAT161" name="1-hexadec-7-enoyl-
sn-glycerol 3-phosphate O-acyltransferase (n-C16:1)" reversible="false"
fast="false" fbc:lowerFluxBound="irr_lb" fbc:upperFluxBound="irr_ub">
            <listOfReactants>
                  <speciesReference species="M_1hdec9eg3p_c"
stoichiometry="1" constant="true"/>
                  <speciesReference species="M_hdeACP_c" stoichiometry="1"
constant="true"/>
            </listOfReactants>
            <listOfProducts>
                  <speciesReference species="M_ACP_c" stoichiometry="1"
constant="true"/>
                  <speciesReference species="M_pa161_c" stoichiometry="1"
constant="true"/>
            </listOfProducts>
            <fbc:geneProductAssociation>
                  <fbc:geneProductRef fbc:geneProduct="SAUSA300_1673"/>
            </fbc:geneProductAssociation>
```

```
      </reaction>

      <reaction metaid="R_AGPAT180" id="R_AGPAT180" name="1-octadecanoyl-sn-
glycerol 3-phosphate O-acyltransferase (n-C18:0)" reversible="false"
fast="false" fbc:lowerFluxBound="irr_lb" fbc:upperFluxBound="irr_ub">
            <listOfReactants>
                  <speciesReference species="M_1odecg3p_c" stoichiometry="1"
constant="true"/>
                  <speciesReference species="M_ocdcaACP_c" stoichiometry="1"
constant="true"/>
            </listOfReactants>
            <listOfProducts>
                  <speciesReference species="M_ACP_c" stoichiometry="1"
constant="true"/>
                  <speciesReference species="M_pa180_c" stoichiometry="1"
constant="true"/>
            </listOfProducts>
            <fbc:geneProductAssociation>
                  <fbc:geneProductRef fbc:geneProduct="SAUSA300_1673"/>
            </fbc:geneProductAssociation>
      </reaction>

      <reaction metaid="R_AGPAT181" id="R_AGPAT181" name="1-octadec-7-enoyl-
sn-glycerol 3-phosphate O-acyltransferase (n-C18:1)" reversible="false"
fast="false" fbc:lowerFluxBound="irr_lb" fbc:upperFluxBound="irr_ub">
            <listOfReactants>
                  <speciesReference species="M_octeACP_c" stoichiometry="1"
constant="true"/>
                  <speciesReference species="M_1odec11eg3p_c"
stoichiometry="1" constant="true"/>
            </listOfReactants>
            <listOfProducts>
                  <speciesReference species="M_ACP_c" stoichiometry="1"
constant="true"/>
                  <speciesReference species="M_pa181_c" stoichiometry="1"
constant="true"/>
            </listOfProducts>
            <fbc:geneProductAssociation>
                  <fbc:geneProductRef fbc:geneProduct="SAUSA300_1673"/>
            </fbc:geneProductAssociation>
      </reaction>

      <reaction metaid="R_AGPR" id="R_AGPR" name="N-acetyl-g-glutamyl-
phosphate reductase" reversible="true" fast="false"
fbc:lowerFluxBound="rev_lb" fbc:upperFluxBound="rev_ub">
            <listOfReactants>
                  <speciesReference species="M_nadp_c" stoichiometry="1"
constant="true"/>
                  <speciesReference species="M_pi_c" stoichiometry="1"
constant="true"/>
                  <speciesReference species="M_acg5sa_c" stoichiometry="1"
constant="true"/>
            </listOfReactants>
            <listOfProducts>
                  <speciesReference species="M_h_c" stoichiometry="1"
constant="true"/>
                  <speciesReference species="M_nadph_c" stoichiometry="1"
constant="true"/>
```

```
                  <speciesReference species="M_acg5p_c" stoichiometry="1"
constant="true"/>
            </listOfProducts>
            <fbc:geneProductAssociation>
                  <fbc:or>
                        <fbc:geneProductRef fbc:geneProduct="SAUSA300_0186"/>
                        <fbc:geneProductRef fbc:geneProduct="SA451515_255"/>
                  </fbc:or>
            </fbc:geneProductAssociation>
      </reaction>

      <reaction metaid="R_AH6PI" id="R_AH6PI" name="Arabino-3-hexulose-6-P
Isomerase" reversible="true" fast="false" fbc:lowerFluxBound="rev_lb"
fbc:upperFluxBound="rev_ub">
            <listOfReactants>
                  <speciesReference species="M_ah6p__D_c" stoichiometry="1"
constant="true"/>
            </listOfReactants>
            <listOfProducts>
                  <speciesReference species="M_f6p_c" stoichiometry="1"
constant="true"/>
            </listOfProducts>
            <fbc:geneProductAssociation>
                  <fbc:geneProductRef fbc:geneProduct="SAUSA300_0556"/>
            </fbc:geneProductAssociation>
      </reaction>

      <reaction metaid="R_AHCYSNS" id="R_AHCYSNS" name="S-
adenosylhomocysteine nucleosidase" reversible="false" fast="false"
fbc:lowerFluxBound="irr_lb" fbc:upperFluxBound="irr_ub">
            <listOfReactants>
                  <speciesReference species="M_h2o_c" stoichiometry="1"
constant="true"/>
                  <speciesReference species="M_ahcys_c" stoichiometry="1"
constant="true"/>
            </listOfReactants>
            <listOfProducts>
                  <speciesReference species="M_ade_c" stoichiometry="1"
constant="true"/>
                  <speciesReference species="M_rhcys_c" stoichiometry="1"
constant="true"/>
            </listOfProducts>
            <fbc:geneProductAssociation>
                  <fbc:geneProductRef fbc:geneProduct="SAUSA300_1558"/>
            </fbc:geneProductAssociation>
      </reaction>

      <reaction metaid="R_AICART" id="R_AICART"
name="phosphoribosylaminoimidazolecarboxamide formyltransferase"
reversible="true" fast="false" fbc:lowerFluxBound="rev_lb"
fbc:upperFluxBound="rev_ub">
            <listOfReactants>
                  <speciesReference species="M_aicar_c" stoichiometry="1"
constant="true"/>
                  <speciesReference species="M_10fthf_c" stoichiometry="1"
constant="true"/>
            </listOfReactants>
            <listOfProducts>
```

```
                  <speciesReference species="M_thf_c" stoichiometry="1"
constant="true"/>
                  <speciesReference species="M_fprica_c" stoichiometry="1"
constant="true"/>
            </listOfProducts>
            <fbc:geneProductAssociation>
                  <fbc:geneProductRef fbc:geneProduct="SAUSA300_0975"/>
            </fbc:geneProductAssociation>
      </reaction>

      <reaction metaid="R_AKGDa" id="R_AKGDa" name="oxoglutarate
dehydrogenase (lipoamide)" reversible="false" fast="false"
fbc:lowerFluxBound="irr_lb" fbc:upperFluxBound="irr_ub">
            <listOfReactants>
                  <speciesReference species="M_h_c" stoichiometry="1"
constant="true"/>
                  <speciesReference species="M_akg_c" stoichiometry="1"
constant="true"/>
                  <speciesReference species="M_lpam_c" stoichiometry="1"
constant="true"/>
            </listOfReactants>
            <listOfProducts>
                  <speciesReference species="M_co2_c" stoichiometry="1"
constant="true"/>
                  <speciesReference species="M_sdhlam_c" stoichiometry="1"
constant="true"/>
            </listOfProducts>
            <fbc:geneProductAssociation>
                  <fbc:geneProductRef fbc:geneProduct="SAUSA300_1306"/>
            </fbc:geneProductAssociation>
      </reaction>

      <reaction metaid="R_AKGDb" id="R_AKGDb" name="oxoglutarate
dehydrogenase (dihydrolipoamide S-succinyltransferase)" reversible="false"
fast="false" fbc:lowerFluxBound="irr_lb" fbc:upperFluxBound="irr_ub">
            <listOfReactants>
                  <speciesReference species="M_dhlam_c" stoichiometry="1"
constant="true"/>
                  <speciesReference species="M_succoa_c" stoichiometry="1"
constant="true"/>
            </listOfReactants>
            <listOfProducts>
                  <speciesReference species="M_coa_c" stoichiometry="1"
constant="true"/>
                  <speciesReference species="M_sdhlam_c" stoichiometry="1"
constant="true"/>
            </listOfProducts>
            <fbc:geneProductAssociation>
                  <fbc:geneProductRef fbc:geneProduct="SAUSA300_1305"/>
            </fbc:geneProductAssociation>
      </reaction>

      <reaction metaid="R_AKGDH" id="R_AKGDH" name="2-Oxoglutarate
dehydrogenase" reversible="false" fast="false" fbc:lowerFluxBound="irr_lb"
fbc:upperFluxBound="irr_ub">
            <listOfReactants>
                  <speciesReference species="M_akg_c" stoichiometry="1"
constant="true"/>
```

```
                  <speciesReference species="M_nad_c" stoichiometry="1"
constant="true"/>
                  <speciesReference species="M_coa_c" stoichiometry="1"
constant="true"/>
            </listOfReactants>
            <listOfProducts>
                  <speciesReference species="M_co2_c" stoichiometry="1"
constant="true"/>
                  <speciesReference species="M_nadh_c" stoichiometry="1"
constant="true"/>
                  <speciesReference species="M_succoa_c" stoichiometry="1"
constant="true"/>
            </listOfProducts>
            <fbc:geneProductAssociation>
                  <fbc:and>
                        <fbc:geneProductRef fbc:geneProduct="SAUSA300_0993"/>
                        <fbc:geneProductRef fbc:geneProduct="SAUSA300_0994"/>
                  </fbc:and>
            </fbc:geneProductAssociation>
      </reaction>

      <reaction metaid="R_AKGMAL" id="R_AKGMAL" name="alpha-
ketoglutarate/malate transporter" reversible="true" fast="false"
fbc:lowerFluxBound="rev_lb" fbc:upperFluxBound="rev_ub">
            <listOfReactants>
                  <speciesReference species="M_akg_c" stoichiometry="1"
constant="true"/>
                  <speciesReference species="M_mal__L_e" stoichiometry="1"
constant="true"/>
            </listOfReactants>
            <listOfProducts>
                  <speciesReference species="M_akg_e" stoichiometry="1"
constant="true"/>
                  <speciesReference species="M_mal__L_c" stoichiometry="1"
constant="true"/>
            </listOfProducts>
            <fbc:geneProductAssociation>
                  <fbc:geneProductRef fbc:geneProduct="SAUSA300_2627"/>
            </fbc:geneProductAssociation>
      </reaction>

      <reaction metaid="R_AKGt2r" id="R_AKGt2r" name="2-oxoglutarate
reversible transport via symport" reversible="false" fast="false"
fbc:lowerFluxBound="irr_lb" fbc:upperFluxBound="irr_ub">
            <listOfReactants>
                  <speciesReference species="M_h_e" stoichiometry="1"
constant="true"/>
                  <speciesReference species="M_akg_e" stoichiometry="1"
constant="true"/>
            </listOfReactants>
            <listOfProducts>
                  <speciesReference species="M_h_c" stoichiometry="1"
constant="true"/>
                  <speciesReference species="M_akg_c" stoichiometry="1"
constant="true"/>
            </listOfProducts>
            <fbc:geneProductAssociation>
                  <fbc:geneProductRef fbc:geneProduct="SAUSA300_2627"/>
```

```
            </fbc:geneProductAssociation>
      </reaction>

      <reaction metaid="R_AKP1" id="R_AKP1" name="alkaline phosphatase
(Dihydroneopterin)" reversible="false" fast="false"
fbc:lowerFluxBound="irr_lb" fbc:upperFluxBound="irr_ub">
            <listOfReactants>
                  <speciesReference species="M_h2o_c" stoichiometry="3"
constant="true"/>
                  <speciesReference species="M_ahdt_c" stoichiometry="1"
constant="true"/>
            </listOfReactants>
            <listOfProducts>
                  <speciesReference species="M_h_c" stoichiometry="3"
constant="true"/>
                  <speciesReference species="M_pi_c" stoichiometry="3"
constant="true"/>
                  <speciesReference species="M_dhnpt_c" stoichiometry="1"
constant="true"/>
            </listOfProducts>
            <fbc:geneProductAssociation>
                  <fbc:geneProductRef fbc:geneProduct="SAUSA300_2561"/>
            </fbc:geneProductAssociation>
      </reaction>

      <reaction metaid="R_ALAALAr" id="R_ALAALAr" name="D-alanine__D-alanine
ligase (reversible)" reversible="true" fast="false"
fbc:lowerFluxBound="rev_lb" fbc:upperFluxBound="rev_ub">
            <listOfReactants>
                  <speciesReference species="M_atp_c" stoichiometry="1"
constant="true"/>
                  <speciesReference species="M_ala__D_c" stoichiometry="2"
constant="true"/>
            </listOfReactants>
            <listOfProducts>
                  <speciesReference species="M_h_c" stoichiometry="1"
constant="true"/>
                  <speciesReference species="M_pi_c" stoichiometry="1"
constant="true"/>
                  <speciesReference species="M_adp_c" stoichiometry="1"
constant="true"/>
                  <speciesReference species="M_alaala_c" stoichiometry="1"
constant="true"/>
            </listOfProducts>
            <fbc:geneProductAssociation>
                  <fbc:geneProductRef fbc:geneProduct="SAUSA300_2039"/>
            </fbc:geneProductAssociation>
      </reaction>

      <reaction metaid="R_ALAASPtr" id="R_ALAASPtr" name="Dipeptide transport
via ABC system ala-asp " reversible="false" fast="false"
fbc:lowerFluxBound="irr_lb" fbc:upperFluxBound="irr_ub">
            <listOfReactants>
                  <speciesReference species="M_h2o_c" stoichiometry="1"
constant="true"/>
                  <speciesReference species="M_atp_c" stoichiometry="1"
constant="true"/>
```

```
                  <speciesReference species="M_ala__L_asp__L_e"
stoichiometry="1" constant="true"/>
            </listOfReactants>
            <listOfProducts>
                  <speciesReference species="M_h_c" stoichiometry="1"
constant="true"/>
                  <speciesReference species="M_pi_c" stoichiometry="1"
constant="true"/>
                  <speciesReference species="M_adp_c" stoichiometry="1"
constant="true"/>
                  <speciesReference species="M_ala__L_asp__L_c"
stoichiometry="1" constant="true"/>
            </listOfProducts>
            <fbc:geneProductAssociation>
                  <fbc:or>
                        <fbc:geneProductRef fbc:geneProduct="SAUSA300_2411"/>
                        <fbc:geneProductRef fbc:geneProduct="SAUSA300_0200"/>
                        <fbc:geneProductRef fbc:geneProduct="SAUSA300_0889"/>
                        <fbc:geneProductRef fbc:geneProduct="SAUSA300_2410"/>
                        <fbc:geneProductRef fbc:geneProduct="SAUSA300_0890"/>
                        <fbc:geneProductRef fbc:geneProduct="SAUSA300_0888"/>
                        <fbc:geneProductRef fbc:geneProduct="SAUSA300_2409"/>
                        <fbc:geneProductRef fbc:geneProduct="SAUSA300_0887"/>
                        <fbc:geneProductRef fbc:geneProduct="SAUSA300_0893"/>
                        <fbc:geneProductRef fbc:geneProduct="SAUSA300_0895"/>
                        <fbc:geneProductRef fbc:geneProduct="SAUSA300_0891"/>
                        <fbc:geneProductRef fbc:geneProduct="SAUSA300_0896"/>
                  </fbc:or>
            </fbc:geneProductAssociation>
      </reaction>

      <reaction metaid="R_ALAD_L" id="R_ALAD_L" name="L-alanine
dehydrogenase" reversible="false" fast="false" fbc:lowerFluxBound="irr_lb"
fbc:upperFluxBound="irr_ub">
            <listOfReactants>
                  <speciesReference species="M_h2o_c" stoichiometry="1"
constant="true"/>
                  <speciesReference species="M_nad_c" stoichiometry="1"
constant="true"/>
                  <speciesReference species="M_ala__L_c" stoichiometry="1"
constant="true"/>
            </listOfReactants>
            <listOfProducts>
                  <speciesReference species="M_h_c" stoichiometry="1"
constant="true"/>
                  <speciesReference species="M_nh4_c" stoichiometry="1"
constant="true"/>
                  <speciesReference species="M_nadh_c" stoichiometry="1"
constant="true"/>
                  <speciesReference species="M_pyr_c" stoichiometry="1"
constant="true"/>
            </listOfProducts>
            <fbc:geneProductAssociation>
                  <fbc:or>
                        <fbc:geneProductRef fbc:geneProduct="SAUSA300_1655"/>
                        <fbc:geneProductRef fbc:geneProduct="SAUSA300_1331"/>
                  </fbc:or>
            </fbc:geneProductAssociation>
```

```
      </reaction>

      <reaction metaid="R_ALAGLNtr" id="R_ALAGLNtr" name="Dipeptide transport
via ABC system ala-gln " reversible="false" fast="false"
fbc:lowerFluxBound="irr_lb" fbc:upperFluxBound="irr_ub">
            <listOfReactants>
                  <speciesReference species="M_h2o_c" stoichiometry="1"
constant="true"/>
                  <speciesReference species="M_ala_gln_e" stoichiometry="1"
constant="true"/>
                  <speciesReference species="M_atp_c" stoichiometry="1"
constant="true"/>
            </listOfReactants>
            <listOfProducts>
                  <speciesReference species="M_h_c" stoichiometry="1"
constant="true"/>
                  <speciesReference species="M_pi_c" stoichiometry="1"
constant="true"/>
                  <speciesReference species="M_adp_c" stoichiometry="1"
constant="true"/>
                  <speciesReference species="M_ala_gln_c" stoichiometry="1"
constant="true"/>
            </listOfProducts>
            <fbc:geneProductAssociation>
                  <fbc:or>
                        <fbc:geneProductRef fbc:geneProduct="SAUSA300_2411"/>
                        <fbc:geneProductRef fbc:geneProduct="SAUSA300_0200"/>
                        <fbc:geneProductRef fbc:geneProduct="SAUSA300_0889"/>
                        <fbc:geneProductRef fbc:geneProduct="SAUSA300_2410"/>
                        <fbc:geneProductRef fbc:geneProduct="SAUSA300_0890"/>
                        <fbc:geneProductRef fbc:geneProduct="SAUSA300_0888"/>
                        <fbc:geneProductRef fbc:geneProduct="SAUSA300_2409"/>
                        <fbc:geneProductRef fbc:geneProduct="SAUSA300_0887"/>
                        <fbc:geneProductRef fbc:geneProduct="SAUSA300_0893"/>
                        <fbc:geneProductRef fbc:geneProduct="SAUSA300_0895"/>
                        <fbc:geneProductRef fbc:geneProduct="SAUSA300_0891"/>
                        <fbc:geneProductRef fbc:geneProduct="SAUSA300_0896"/>
                        <fbc:geneProductRef fbc:geneProduct="SAUSA300_0712"/>
                  </fbc:or>
            </fbc:geneProductAssociation>
      </reaction>

      <reaction metaid="R_ALAGLUt" id="R_ALAGLUt" name="Dipeptide transport
via ABC system ala-glu " reversible="false" fast="false"
fbc:lowerFluxBound="irr_lb" fbc:upperFluxBound="irr_ub">
            <listOfReactants>
                  <speciesReference species="M_h2o_c" stoichiometry="1"
constant="true"/>
                  <speciesReference species="M_atp_c" stoichiometry="1"
constant="true"/>
                  <speciesReference species="M_ala__L_glu__L_e"
stoichiometry="1" constant="true"/>
            </listOfReactants>
            <listOfProducts>
                  <speciesReference species="M_h_c" stoichiometry="1"
constant="true"/>
                  <speciesReference species="M_pi_c" stoichiometry="1"
constant="true"/>
```

```
                  <speciesReference species="M_adp_c" stoichiometry="1"
constant="true"/>
                  <speciesReference species="M_ala__L_glu__L_c"
stoichiometry="1" constant="true"/>
            </listOfProducts>
            <fbc:geneProductAssociation>
                  <fbc:or>
                        <fbc:geneProductRef fbc:geneProduct="SAUSA300_2411"/>
                        <fbc:geneProductRef fbc:geneProduct="SAUSA300_0200"/>
                        <fbc:geneProductRef fbc:geneProduct="SAUSA300_0889"/>
                        <fbc:geneProductRef fbc:geneProduct="SAUSA300_2410"/>
                        <fbc:geneProductRef fbc:geneProduct="SAUSA300_0890"/>
                        <fbc:geneProductRef fbc:geneProduct="SAUSA300_0888"/>
                        <fbc:geneProductRef fbc:geneProduct="SAUSA300_2409"/>
                        <fbc:geneProductRef fbc:geneProduct="SAUSA300_0887"/>
                        <fbc:geneProductRef fbc:geneProduct="SAUSA300_0893"/>
                        <fbc:geneProductRef fbc:geneProduct="SAUSA300_0895"/>
                        <fbc:geneProductRef fbc:geneProduct="SAUSA300_0891"/>
                        <fbc:geneProductRef fbc:geneProduct="SAUSA300_0896"/>
                        <fbc:geneProductRef fbc:geneProduct="SAUSA300_0712"/>
                  </fbc:or>
            </fbc:geneProductAssociation>
      </reaction>

      <reaction metaid="R_ALAGLYtr" id="R_ALAGLYtr" name="Dipeptide transport
via ABC system ala-gly " reversible="false" fast="false"
fbc:lowerFluxBound="irr_lb" fbc:upperFluxBound="irr_ub">
            <listOfReactants>
                  <speciesReference species="M_h2o_c" stoichiometry="1"
constant="true"/>
                  <speciesReference species="M_atp_c" stoichiometry="1"
constant="true"/>
                  <speciesReference species="M_alagly_e" stoichiometry="1"
constant="true"/>
            </listOfReactants>
            <listOfProducts>
                  <speciesReference species="M_h_c" stoichiometry="1"
constant="true"/>
                  <speciesReference species="M_pi_c" stoichiometry="1"
constant="true"/>
                  <speciesReference species="M_adp_c" stoichiometry="1"
constant="true"/>
                  <speciesReference species="M_alagly_c" stoichiometry="1"
constant="true"/>
            </listOfProducts>
            <fbc:geneProductAssociation>
                  <fbc:or>
                        <fbc:geneProductRef fbc:geneProduct="SAUSA300_2411"/>
                        <fbc:geneProductRef fbc:geneProduct="SAUSA300_0200"/>
                        <fbc:geneProductRef fbc:geneProduct="SAUSA300_0889"/>
                        <fbc:geneProductRef fbc:geneProduct="SAUSA300_2410"/>
                        <fbc:geneProductRef fbc:geneProduct="SAUSA300_0890"/>
                        <fbc:geneProductRef fbc:geneProduct="SAUSA300_0888"/>
                        <fbc:geneProductRef fbc:geneProduct="SAUSA300_2409"/>
                        <fbc:geneProductRef fbc:geneProduct="SAUSA300_0887"/>
                        <fbc:geneProductRef fbc:geneProduct="SAUSA300_0893"/>
                        <fbc:geneProductRef fbc:geneProduct="SAUSA300_0895"/>
                        <fbc:geneProductRef fbc:geneProduct="SAUSA300_0891"/>
```

```
                        <fbc:geneProductRef fbc:geneProduct="SAUSA300_0896"/>
                        <fbc:geneProductRef fbc:geneProduct="SAUSA300_0712"/>
                  </fbc:or>
            </fbc:geneProductAssociation>
      </reaction>

      <reaction metaid="R_ALAHIStr" id="R_ALAHIStr" name="Dipeptide transport
via ABC system ala-his " reversible="false" fast="false"
fbc:lowerFluxBound="irr_lb" fbc:upperFluxBound="irr_ub">
            <listOfReactants>
                  <speciesReference species="M_h2o_c" stoichiometry="1"
constant="true"/>
                  <speciesReference species="M_atp_c" stoichiometry="1"
constant="true"/>
                  <speciesReference species="M_ala_his_e" stoichiometry="1"
constant="true"/>
            </listOfReactants>
            <listOfProducts>
                  <speciesReference species="M_h_c" stoichiometry="1"
constant="true"/>
                  <speciesReference species="M_pi_c" stoichiometry="1"
constant="true"/>
                  <speciesReference species="M_adp_c" stoichiometry="1"
constant="true"/>
                  <speciesReference species="M_ala_his_c" stoichiometry="1"
constant="true"/>
            </listOfProducts>
            <fbc:geneProductAssociation>
                  <fbc:or>
                        <fbc:geneProductRef fbc:geneProduct="SAUSA300_2411"/>
                        <fbc:geneProductRef fbc:geneProduct="SAUSA300_0200"/>
                        <fbc:geneProductRef fbc:geneProduct="SAUSA300_0889"/>
                        <fbc:geneProductRef fbc:geneProduct="SAUSA300_2410"/>
                        <fbc:geneProductRef fbc:geneProduct="SAUSA300_0890"/>
                        <fbc:geneProductRef fbc:geneProduct="SAUSA300_0888"/>
                        <fbc:geneProductRef fbc:geneProduct="SAUSA300_2409"/>
                        <fbc:geneProductRef fbc:geneProduct="SAUSA300_0887"/>
                        <fbc:geneProductRef fbc:geneProduct="SAUSA300_0893"/>
                        <fbc:geneProductRef fbc:geneProduct="SAUSA300_0895"/>
                        <fbc:geneProductRef fbc:geneProduct="SAUSA300_0891"/>
                        <fbc:geneProductRef fbc:geneProduct="SAUSA300_0896"/>
                        <fbc:geneProductRef fbc:geneProduct="SAUSA300_0712"/>
                  </fbc:or>
            </fbc:geneProductAssociation>
      </reaction>

      <reaction metaid="R_ALALEUt" id="R_ALALEUt" name="Dipeptide transport
via ABC system ala__Leu " reversible="false" fast="false"
fbc:lowerFluxBound="irr_lb" fbc:upperFluxBound="irr_ub">
            <listOfReactants>
                  <speciesReference species="M_h2o_c" stoichiometry="1"
constant="true"/>
                  <speciesReference species="M_atp_c" stoichiometry="1"
constant="true"/>
                  <speciesReference species="M_ala_leu_e" stoichiometry="1"
constant="true"/>
            </listOfReactants>
            <listOfProducts>
```

```
                  <speciesReference species="M_h_c" stoichiometry="1"
constant="true"/>
                  <speciesReference species="M_pi_c" stoichiometry="1"
constant="true"/>
                  <speciesReference species="M_adp_c" stoichiometry="1"
constant="true"/>
                  <speciesReference species="M_ala_leu_c" stoichiometry="1"
constant="true"/>
            </listOfProducts>
            <fbc:geneProductAssociation>
                  <fbc:or>
                        <fbc:geneProductRef fbc:geneProduct="SAUSA300_2411"/>
                        <fbc:geneProductRef fbc:geneProduct="SAUSA300_0200"/>
                        <fbc:geneProductRef fbc:geneProduct="SAUSA300_0889"/>
                        <fbc:geneProductRef fbc:geneProduct="SAUSA300_2410"/>
                        <fbc:geneProductRef fbc:geneProduct="SAUSA300_0890"/>
                        <fbc:geneProductRef fbc:geneProduct="SAUSA300_0888"/>
                        <fbc:geneProductRef fbc:geneProduct="SAUSA300_2409"/>
                        <fbc:geneProductRef fbc:geneProduct="SAUSA300_0887"/>
                        <fbc:geneProductRef fbc:geneProduct="SAUSA300_0893"/>
                        <fbc:geneProductRef fbc:geneProduct="SAUSA300_0895"/>
                        <fbc:geneProductRef fbc:geneProduct="SAUSA300_0891"/>
                        <fbc:geneProductRef fbc:geneProduct="SAUSA300_0896"/>
                        <fbc:geneProductRef fbc:geneProduct="SAUSA300_0712"/>
                  </fbc:or>
            </fbc:geneProductAssociation>
      </reaction>

      <reaction metaid="R_ALAR" id="R_ALAR" name="alanine racemase"
reversible="true" fast="false" fbc:lowerFluxBound="rev_lb"
fbc:upperFluxBound="rev_ub">
            <listOfReactants>
                  <speciesReference species="M_ala__L_c" stoichiometry="1"
constant="true"/>
            </listOfReactants>
            <listOfProducts>
                  <speciesReference species="M_ala__D_c" stoichiometry="1"
constant="true"/>
            </listOfProducts>
            <fbc:geneProductAssociation>
                  <fbc:geneProductRef fbc:geneProduct="SAUSA300_2027"/>
            </fbc:geneProductAssociation>
      </reaction>

      <reaction metaid="R_ALAt2r" id="R_ALAt2r" name="L-alanine reversible
transport via proton symport" reversible="false" fast="false"
fbc:lowerFluxBound="irr_lb" fbc:upperFluxBound="irr_ub">
            <listOfReactants>
                  <speciesReference species="M_h_e" stoichiometry="1"
constant="true"/>
                  <speciesReference species="M_ala__L_e" stoichiometry="1"
constant="true"/>
            </listOfReactants>
            <listOfProducts>
                  <speciesReference species="M_h_c" stoichiometry="1"
constant="true"/>
                  <speciesReference species="M_ala__L_c" stoichiometry="1"
constant="true"/>
```

```
            </listOfProducts>
            <fbc:geneProductAssociation>
                  <fbc:or>
                        <fbc:geneProductRef fbc:geneProduct="SAUSA300_1252"/>
                        <fbc:geneProductRef fbc:geneProduct="SAUSA300_0712"/>
                        <fbc:geneProductRef fbc:geneProduct="SAUSA300_1642"/>
                        <fbc:geneProductRef fbc:geneProduct="SAUSA300_0914"/>
                  </fbc:or>
            </fbc:geneProductAssociation>
      </reaction>

      <reaction metaid="R_ALAt4" id="R_ALAt4" name="Alanine-Sodium symporter"
reversible="false" fast="false" fbc:lowerFluxBound="irr_lb"
fbc:upperFluxBound="irr_ub">
            <listOfReactants>
                  <speciesReference species="M_ala__L_e" stoichiometry="1"
constant="true"/>
                  <speciesReference species="M_na1_e" stoichiometry="1"
constant="true"/>
            </listOfReactants>
            <listOfProducts>
                  <speciesReference species="M_ala__L_c" stoichiometry="1"
constant="true"/>
                  <speciesReference species="M_na1_c" stoichiometry="1"
constant="true"/>
            </listOfProducts>
            <fbc:geneProductAssociation>
                  <fbc:or>
                        <fbc:geneProductRef fbc:geneProduct="SAUSA300_1642"/>
                        <fbc:geneProductRef fbc:geneProduct="SAUSA300_1252"/>
                        <fbc:geneProductRef fbc:geneProduct="SAUSA300_0914"/>
                  </fbc:or>
            </fbc:geneProductAssociation>
      </reaction>

      <reaction metaid="R_ALATA_D" id="R_ALATA_D" name="D-alanine
transaminase" reversible="true" fast="false" fbc:lowerFluxBound="rev_lb"
fbc:upperFluxBound="rev_ub">
            <listOfReactants>
                  <speciesReference species="M_akg_c" stoichiometry="1"
constant="true"/>
                  <speciesReference species="M_ala__D_c" stoichiometry="1"
constant="true"/>
            </listOfReactants>
            <listOfProducts>
                  <speciesReference species="M_glu__D_c" stoichiometry="1"
constant="true"/>
                  <speciesReference species="M_pyr_c" stoichiometry="1"
constant="true"/>
            </listOfProducts>
            <fbc:geneProductAssociation>
                  <fbc:geneProductRef fbc:geneProduct="SAUSA300_1696"/>
            </fbc:geneProductAssociation>
      </reaction>

      <reaction metaid="R_ALATHRtr" id="R_ALATHRtr" name="Dipeptide transport
via ABC system ala-thr " reversible="false" fast="false"
fbc:lowerFluxBound="irr_lb" fbc:upperFluxBound="irr_ub">
```

```
			<listOfReactants>
				<speciesReference species="M_h2o_c" stoichiometry="1"
constant="true"/>
				<speciesReference species="M_atp_c" stoichiometry="1"
constant="true"/>
				<speciesReference species="M_ala__L_thr__L_e"
stoichiometry="1" constant="true"/>
			</listOfReactants>
			<listOfProducts>
				<speciesReference species="M_h_c" stoichiometry="1"
constant="true"/>
				<speciesReference species="M_pi_c" stoichiometry="1"
constant="true"/>
				<speciesReference species="M_adp_c" stoichiometry="1"
constant="true"/>
				<speciesReference species="M_ala__L_thr__L_c"
stoichiometry="1" constant="true"/>
			</listOfProducts>
			<fbc:geneProductAssociation>
				<fbc:or>
					<fbc:geneProductRef fbc:geneProduct="SAUSA300_2411"/>
					<fbc:geneProductRef fbc:geneProduct="SAUSA300_0200"/>
					<fbc:geneProductRef fbc:geneProduct="SAUSA300_0889"/>
					<fbc:geneProductRef fbc:geneProduct="SAUSA300_2410"/>
					<fbc:geneProductRef fbc:geneProduct="SAUSA300_0890"/>
					<fbc:geneProductRef fbc:geneProduct="SAUSA300_0888"/>
					<fbc:geneProductRef fbc:geneProduct="SAUSA300_2409"/>
					<fbc:geneProductRef fbc:geneProduct="SAUSA300_0887"/>
					<fbc:geneProductRef fbc:geneProduct="SAUSA300_0893"/>
					<fbc:geneProductRef fbc:geneProduct="SAUSA300_0895"/>
					<fbc:geneProductRef fbc:geneProduct="SAUSA300_0891"/>
					<fbc:geneProductRef fbc:geneProduct="SAUSA300_0896"/>
					<fbc:geneProductRef fbc:geneProduct="SAUSA300_0712"/>
				</fbc:or>
			</fbc:geneProductAssociation>
		</reaction>

		<reaction metaid="R_ALATRS" id="R_ALATRS" name="Alanyl-tRNA synthetase"
reversible="false" fast="false" fbc:lowerFluxBound="irr_lb"
fbc:upperFluxBound="irr_ub">
			<listOfReactants>
				<speciesReference species="M_atp_c" stoichiometry="1"
constant="true"/>
				<speciesReference species="M_ala__L_c" stoichiometry="1"
constant="true"/>
				<speciesReference species="M_trnaala_c" stoichiometry="1"
constant="true"/>
			</listOfReactants>
			<listOfProducts>
				<speciesReference species="M_ppi_c" stoichiometry="1"
constant="true"/>
				<speciesReference species="M_amp_c" stoichiometry="1"
constant="true"/>
				<speciesReference species="M_alatrna_c" stoichiometry="1"
constant="true"/>
			</listOfProducts>
			<fbc:geneProductAssociation>
				<fbc:geneProductRef fbc:geneProduct="SAUSA300_1575"/>
```

```
            </fbc:geneProductAssociation>
      </reaction>

      <reaction metaid="R_ALCD1" id="R_ALCD1" name="alcohol dehydrogenase
(methanol)" reversible="true" fast="false" fbc:lowerFluxBound="rev_lb"
fbc:upperFluxBound="rev_ub">
            <listOfReactants>
                  <speciesReference species="M_nad_c" stoichiometry="1"
constant="true"/>
                  <speciesReference species="M_meoh_c" stoichiometry="1"
constant="true"/>
            </listOfReactants>
            <listOfProducts>
                  <speciesReference species="M_h_c" stoichiometry="1"
constant="true"/>
                  <speciesReference species="M_nadh_c" stoichiometry="1"
constant="true"/>
                  <speciesReference species="M_fald_c" stoichiometry="1"
constant="true"/>
            </listOfProducts>
            <fbc:geneProductAssociation>
                  <fbc:geneProductRef fbc:geneProduct="SAUSA300_1232"/>
            </fbc:geneProductAssociation>
      </reaction>

      <reaction metaid="R_ALCD19" id="R_ALCD19" name="alcohol dehydrogenase
(glycerol)" reversible="true" fast="false" fbc:lowerFluxBound="rev_lb"
fbc:upperFluxBound="rev_ub">
            <listOfReactants>
                  <speciesReference species="M_glyc_c" stoichiometry="1"
constant="true"/>
                  <speciesReference species="M_nad_c" stoichiometry="1"
constant="true"/>
            </listOfReactants>
            <listOfProducts>
                  <speciesReference species="M_h_c" stoichiometry="1"
constant="true"/>
                  <speciesReference species="M_nadh_c" stoichiometry="1"
constant="true"/>
                  <speciesReference species="M_glyald_c" stoichiometry="1"
constant="true"/>
            </listOfProducts>
            <fbc:geneProductAssociation>
                  <fbc:or>
                        <fbc:geneProductRef fbc:geneProduct="SAUSA300_0594"/>
                        <fbc:geneProductRef fbc:geneProduct="SAUSA300_0151"/>
                        <fbc:geneProductRef fbc:geneProduct="SAUSA300_2317"/>
                        <fbc:geneProductRef fbc:geneProduct="SAUSA300_0055"/>
                  </fbc:or>
            </fbc:geneProductAssociation>
      </reaction>

      <reaction metaid="R_ALCD2x" id="R_ALCD2x" name="alcohol dehydrogenase
(ethanol)" reversible="true" fast="false" fbc:lowerFluxBound="rev_lb"
fbc:upperFluxBound="rev_ub">
            <listOfReactants>
                  <speciesReference species="M_nad_c" stoichiometry="1"
constant="true"/>
```

```
                  <speciesReference species="M_etoh_c" stoichiometry="1"
constant="true"/>
            &lt;/listOfReactants&gt;
            &lt;listOfProducts&gt;
                  <speciesReference species="M_h_c" stoichiometry="1"
constant="true"/>
                  <speciesReference species="M_nadh_c" stoichiometry="1"
constant="true"/>
                  <speciesReference species="M_acald_c" stoichiometry="1"
constant="true"/>
            &lt;/listOfProducts&gt;
            &lt;fbc:geneProductAssociation&gt;
                  &lt;fbc:geneProductRef fbc:geneProduct="SAUSA300_0594"/&gt;
            &lt;/fbc:geneProductAssociation&gt;
      &lt;/reaction&gt;

      <reaction metaid="R_ALDD22x" id="R_ALDD22x" name="aldehyde
dehydrogenase (3-aminopropanal, NAD)" reversible="false" fast="false"
fbc:lowerFluxBound="irr_lb" fbc:upperFluxBound="irr_ub">
            &lt;listOfReactants&gt;
                  <speciesReference species="M_h2o_c" stoichiometry="1"
constant="true"/>
                  <speciesReference species="M_nad_c" stoichiometry="1"
constant="true"/>
                  <speciesReference species="M_aproa_c" stoichiometry="1"
constant="true"/>
            &lt;/listOfReactants&gt;
            &lt;listOfProducts&gt;
                  <speciesReference species="M_h_c" stoichiometry="2"
constant="true"/>
                  <speciesReference species="M_nadh_c" stoichiometry="1"
constant="true"/>
                  <speciesReference species="M_ala__B_c" stoichiometry="1"
constant="true"/>
            &lt;/listOfProducts&gt;
            &lt;fbc:geneProductAssociation&gt;
                  &lt;fbc:and&gt;
                        &lt;fbc:geneProductRef fbc:geneProduct="SAUSA300_0170"/&gt;
                        &lt;fbc:geneProductRef fbc:geneProduct="SAUSA300_1901"/&gt;
                        &lt;fbc:geneProductRef fbc:geneProduct="SAUSA300_2076"/&gt;
                  &lt;/fbc:and&gt;
            &lt;/fbc:geneProductAssociation&gt;
      &lt;/reaction&gt;

      <reaction metaid="R_ALDD2x" id="R_ALDD2x" name="aldehyde dehydrogenase
(acetaldehyde, NAD)" reversible="false" fast="false"
fbc:lowerFluxBound="irr_lb" fbc:upperFluxBound="irr_ub">
            &lt;listOfReactants&gt;
                  <speciesReference species="M_h2o_c" stoichiometry="1"
constant="true"/>
                  <speciesReference species="M_nad_c" stoichiometry="1"
constant="true"/>
                  <speciesReference species="M_acald_c" stoichiometry="1"
constant="true"/>
            &lt;/listOfReactants&gt;
            &lt;listOfProducts&gt;
                  <speciesReference species="M_h_c" stoichiometry="2"
constant="true"/>
```

```
                  <speciesReference species="M_ac_c" stoichiometry="1"
constant="true"/>
                  <speciesReference species="M_nadh_c" stoichiometry="1"
constant="true"/>
            </listOfProducts>
            <fbc:geneProductAssociation>
                  <fbc:or>
                        <fbc:geneProductRef fbc:geneProduct="SAUSA300_1901"/>
                        <fbc:geneProductRef fbc:geneProduct="SAUSA300_2076"/>
                  </fbc:or>
            </fbc:geneProductAssociation>
      </reaction>

      <reaction metaid="R_ALDD31" id="R_ALDD31" name="Aminoacetaldehyde
oxidation" reversible="true" fast="false" fbc:lowerFluxBound="rev_lb"
fbc:upperFluxBound="rev_ub">
            <listOfReactants>
                  <speciesReference species="M_h2o_c" stoichiometry="1"
constant="true"/>
                  <speciesReference species="M_nad_c" stoichiometry="1"
constant="true"/>
                  <speciesReference species="M_aacald_c" stoichiometry="1"
constant="true"/>
            </listOfReactants>
            <listOfProducts>
                  <speciesReference species="M_h_c" stoichiometry="2"
constant="true"/>
                  <speciesReference species="M_nadh_c" stoichiometry="1"
constant="true"/>
                  <speciesReference species="M_gly_c" stoichiometry="1"
constant="true"/>
            </listOfProducts>
            <fbc:geneProductAssociation>
                  <fbc:or>
                        <fbc:geneProductRef fbc:geneProduct="SAUSA300_1901"/>
                        <fbc:geneProductRef fbc:geneProduct="SAUSA300_2076"/>
                  </fbc:or>
            </fbc:geneProductAssociation>
      </reaction>

      <reaction metaid="R_ALKP" id="R_ALKP" name="alkaline phosphatase"
reversible="false" fast="false" fbc:lowerFluxBound="irr_lb"
fbc:upperFluxBound="irr_ub">
            <listOfReactants>
                  <speciesReference species="M_h2o_c" stoichiometry="1"
constant="true"/>
                  <speciesReference species="M_dhap_c" stoichiometry="1"
constant="true"/>
            </listOfReactants>
            <listOfProducts>
                  <speciesReference species="M_h_c" stoichiometry="1"
constant="true"/>
                  <speciesReference species="M_pi_c" stoichiometry="1"
constant="true"/>
                  <speciesReference species="M_dha_c" stoichiometry="1"
constant="true"/>
            </listOfProducts>
            <fbc:geneProductAssociation>
```

```
                  <fbc:geneProductRef fbc:geneProduct="SAUSA300_2561"/>
            </fbc:geneProductAssociation>
      </reaction>

      <reaction metaid="R_ALPHNH" id="R_ALPHNH" name="allophanate hydrolase"
reversible="false" fast="false" fbc:lowerFluxBound="irr_lb"
fbc:upperFluxBound="irr_ub">
            <listOfReactants>
                  <speciesReference species="M_h_c" stoichiometry="3"
constant="true"/>
                  <speciesReference species="M_h2o_c" stoichiometry="1"
constant="true"/>
                  <speciesReference species="M_allphn_c" stoichiometry="1"
constant="true"/>
            </listOfReactants>
            <listOfProducts>
                  <speciesReference species="M_nh4_c" stoichiometry="2"
constant="true"/>
                  <speciesReference species="M_co2_c" stoichiometry="2"
constant="true"/>
            </listOfProducts>
            <fbc:geneProductAssociation>
                  <fbc:and>
                        <fbc:or>
                              <fbc:geneProductRef
fbc:geneProduct="SAUSA300_1565"/>
                              <fbc:geneProductRef
fbc:geneProduct="SAUSA300_0702"/>
                        </fbc:or>
                        <fbc:or>
                              <fbc:geneProductRef
fbc:geneProduct="SAUSA300_1566"/>
                              <fbc:geneProductRef
fbc:geneProduct="SAUSA300_0701"/>
                        </fbc:or>
                  </fbc:and>
            </fbc:geneProductAssociation>
      </reaction>

      <reaction metaid="R_AMAA" id="R_AMAA" name="N-acetylmuramoyl__L-alanine
amidase" reversible="false" fast="false" fbc:lowerFluxBound="irr_lb"
fbc:upperFluxBound="irr_ub">
            <listOfReactants>
                  <speciesReference species="M_h2o_c" stoichiometry="1"
constant="true"/>
                  <speciesReference species="M_acmama_c" stoichiometry="1"
constant="true"/>
            </listOfReactants>
            <listOfProducts>
                  <speciesReference species="M_ala__L_c" stoichiometry="1"
constant="true"/>
                  <speciesReference species="M_acmam_c" stoichiometry="1"
constant="true"/>
            </listOfProducts>
            <fbc:geneProductAssociation>
                  <fbc:or>
                        <fbc:geneProductRef fbc:geneProduct="SAUSA300_2579"/>
                        <fbc:geneProductRef fbc:geneProduct="SAUSA300_1588"/>
```

```
                        <fbc:geneProductRef fbc:geneProduct="SAUSA300_2256"/>
                  </fbc:or>
            </fbc:geneProductAssociation>
      </reaction>

      <reaction metaid="R_AMANAPEr" id="R_AMANAPEr" name="N-acetylmannosamine
6-phosphate epimerase" reversible="true" fast="false"
fbc:lowerFluxBound="rev_lb" fbc:upperFluxBound="rev_ub">
            <listOfReactants>
                  <speciesReference species="M_acgam6p_c" stoichiometry="1"
constant="true"/>
            </listOfReactants>
            <listOfProducts>
                  <speciesReference species="M_acmanap_c" stoichiometry="1"
constant="true"/>
            </listOfProducts>
            <fbc:geneProductAssociation>
                  <fbc:geneProductRef fbc:geneProduct="SAUSA300_0318"/>
            </fbc:geneProductAssociation>
      </reaction>

      <reaction metaid="R_AMANK" id="R_AMANK" name="N-acetyl__D-mannosamine
kinase" reversible="false" fast="false" fbc:lowerFluxBound="irr_lb"
fbc:upperFluxBound="irr_ub">
            <listOfReactants>
                  <speciesReference species="M_atp_c" stoichiometry="1"
constant="true"/>
                  <speciesReference species="M_acmana_c" stoichiometry="1"
constant="true"/>
            </listOfReactants>
            <listOfProducts>
                  <speciesReference species="M_adp_c" stoichiometry="1"
constant="true"/>
                  <speciesReference species="M_acmanap_c" stoichiometry="1"
constant="true"/>
            </listOfProducts>
            <fbc:geneProductAssociation>
                  <fbc:or>
                        <fbc:geneProductRef fbc:geneProduct="SAUSA300_0316"/>
                        <fbc:geneProductRef fbc:geneProduct="SAUSA300_0743"/>
                  </fbc:or>
            </fbc:geneProductAssociation>
      </reaction>

      <reaction metaid="R_AMAOTr" id="R_AMAOTr" name="adenosylmethionine-8-
amino-7-oxononanoate transaminase" reversible="true" fast="false"
fbc:lowerFluxBound="rev_lb" fbc:upperFluxBound="rev_ub">
            <listOfReactants>
                  <speciesReference species="M_amet_c" stoichiometry="1"
constant="true"/>
                  <speciesReference species="M_8aonn_c" stoichiometry="1"
constant="true"/>
            </listOfReactants>
            <listOfProducts>
                  <speciesReference species="M_dann_c" stoichiometry="1"
constant="true"/>
                  <speciesReference species="M_amob_c" stoichiometry="1"
constant="true"/>
```

```
            </listOfProducts>
            <fbc:geneProductAssociation>
                  <fbc:geneProductRef fbc:geneProduct="SAUSA300_2372"/>
            </fbc:geneProductAssociation>
      </reaction>

      <reaction metaid="R_AMID" id="R_AMID" name="amidase" reversible="false"
fast="false" fbc:lowerFluxBound="irr_lb" fbc:upperFluxBound="irr_ub">
            <listOfReactants>
                  <speciesReference species="M_h2o_c" stoichiometry="1"
constant="true"/>
                  <speciesReference species="M_4gudbd_c" stoichiometry="1"
constant="true"/>
            </listOfReactants>
            <listOfProducts>
                  <speciesReference species="M_nh4_c" stoichiometry="1"
constant="true"/>
                  <speciesReference species="M_4gudbutn_c" stoichiometry="1"
constant="true"/>
            </listOfProducts>
            <fbc:geneProductAssociation>
                  <fbc:or>
                        <fbc:geneProductRef fbc:geneProduct="SAUSA300_1987"/>
                        <fbc:geneProductRef fbc:geneProduct="SAUSA300_1383"/>
                  </fbc:or>
            </fbc:geneProductAssociation>
      </reaction>

      <reaction metaid="R_AMID2r" id="R_AMID2r" name="2-phenylacetamide
amidohydrolase " reversible="true" fast="false" fbc:lowerFluxBound="rev_lb"
fbc:upperFluxBound="rev_ub">
            <listOfReactants>
                  <speciesReference species="M_h_c" stoichiometry="1"
constant="true"/>
                  <speciesReference species="M_h2o_c" stoichiometry="1"
constant="true"/>
                  <speciesReference species="M_pad_c" stoichiometry="1"
constant="true"/>
            </listOfReactants>
            <listOfProducts>
                  <speciesReference species="M_nh4_c" stoichiometry="1"
constant="true"/>
                  <speciesReference species="M_pac_c" stoichiometry="1"
constant="true"/>
            </listOfProducts>
            <fbc:geneProductAssociation>
                  <fbc:geneProductRef fbc:geneProduct="SAUSA300_1987"/>
            </fbc:geneProductAssociation>
      </reaction>

      <reaction metaid="R_AMID3" id="R_AMID3" name="amidase"
reversible="false" fast="false" fbc:lowerFluxBound="irr_lb"
fbc:upperFluxBound="irr_ub">
            <listOfReactants>
                  <speciesReference species="M_h2o_c" stoichiometry="1"
constant="true"/>
                  <speciesReference species="M_iad_c" stoichiometry="1"
constant="true"/>
```

```
            </listOfReactants>
            <listOfProducts>
                  <speciesReference species="M_nh4_c" stoichiometry="1"
constant="true"/>
                  <speciesReference species="M_ind3ac_c" stoichiometry="1"
constant="true"/>
            </listOfProducts>
            <fbc:geneProductAssociation>
                  <fbc:or>
                        <fbc:geneProductRef fbc:geneProduct="SAUSA300_1987"/>
                        <fbc:geneProductRef fbc:geneProduct="SAUSA300_1383"/>
                  </fbc:or>
            </fbc:geneProductAssociation>
      </reaction>

      <reaction metaid="R_AMID4" id="R_AMID4" name="amidase"
reversible="false" fast="false" fbc:lowerFluxBound="irr_lb"
fbc:upperFluxBound="irr_ub">
            <listOfReactants>
                  <speciesReference species="M_h2o_c" stoichiometry="1"
constant="true"/>
                  <speciesReference species="M_ad_c" stoichiometry="1"
constant="true"/>
            </listOfReactants>
            <listOfProducts>
                  <speciesReference species="M_ac_c" stoichiometry="1"
constant="true"/>
                  <speciesReference species="M_nh4_c" stoichiometry="1"
constant="true"/>
            </listOfProducts>
            <fbc:geneProductAssociation>
                  <fbc:or>
                        <fbc:geneProductRef fbc:geneProduct="SAUSA300_1987"/>
                        <fbc:geneProductRef fbc:geneProduct="SAUSA300_1383"/>
                  </fbc:or>
            </fbc:geneProductAssociation>
      </reaction>

      <reaction metaid="R_AMID5" id="R_AMID5" name="amidase"
reversible="false" fast="false" fbc:lowerFluxBound="irr_lb"
fbc:upperFluxBound="irr_ub">
            <listOfReactants>
                  <speciesReference species="M_h2o_c" stoichiometry="1"
constant="true"/>
                  <speciesReference species="M_aa_c" stoichiometry="1"
constant="true"/>
            </listOfReactants>
            <listOfProducts>
                  <speciesReference species="M_nh4_c" stoichiometry="1"
constant="true"/>
                  <speciesReference species="M_acryl_c" stoichiometry="1"
constant="true"/>
            </listOfProducts>
            <fbc:geneProductAssociation>
                  <fbc:geneProductRef fbc:geneProduct="SAUSA300_1987"/>
            </fbc:geneProductAssociation>
      </reaction>
```

```
      <reaction metaid="R_AMID7" id="R_AMID7" name="Benzamide aminohydrolase"
reversible="false" fast="false" fbc:lowerFluxBound="irr_lb"
fbc:upperFluxBound="irr_ub">
            <listOfReactants>
                  <speciesReference species="M_h2o_c" stoichiometry="1"
constant="true"/>
                  <speciesReference species="M_bzmd_c" stoichiometry="1"
constant="true"/>
            </listOfReactants>
            <listOfProducts>
                  <speciesReference species="M_nh4_c" stoichiometry="1"
constant="true"/>
                  <speciesReference species="M_bz_c" stoichiometry="1"
constant="true"/>
            </listOfProducts>
            <fbc:geneProductAssociation>
                  <fbc:geneProductRef fbc:geneProduct="SAUSA300_1987"/>
            </fbc:geneProductAssociation>
      </reaction>

      <reaction metaid="R_AMPEP1" id="R_AMPEP1" name="aminopeptidase "
reversible="true" fast="false" fbc:lowerFluxBound="rev_lb"
fbc:upperFluxBound="rev_ub">
            <listOfReactants>
                  <speciesReference species="M_h2o_c" stoichiometry="1"
constant="true"/>
                  <speciesReference species="M_gly_asn__L_c"
stoichiometry="1" constant="true"/>
            </listOfReactants>
            <listOfProducts>
                  <speciesReference species="M_gly_c" stoichiometry="1"
constant="true"/>
                  <speciesReference species="M_asn__L_c" stoichiometry="1"
constant="true"/>
            </listOfProducts>
            <fbc:geneProductAssociation>
                  <fbc:or>
                        <fbc:geneProductRef fbc:geneProduct="SAUSA300_1491"/>
                        <fbc:geneProductRef fbc:geneProduct="SAUSA300_1869"/>
                        <fbc:geneProductRef fbc:geneProduct="SAUSA300_0845"/>
                        <fbc:geneProductRef fbc:geneProduct="SAUSA300_1860"/>
                  </fbc:or>
            </fbc:geneProductAssociation>
      </reaction>

      <reaction metaid="R_AMPEP10" id="R_AMPEP10" name="aminopeptidase "
reversible="true" fast="false" fbc:lowerFluxBound="rev_lb"
fbc:upperFluxBound="rev_ub">
            <listOfReactants>
                  <speciesReference species="M_h2o_c" stoichiometry="1"
constant="true"/>
                  <speciesReference species="M_gly_asp__L_c"
stoichiometry="1" constant="true"/>
            </listOfReactants>
            <listOfProducts>
                  <speciesReference species="M_gly_c" stoichiometry="1"
constant="true"/>
```

```
                  <speciesReference species="M_asp__L_c" stoichiometry="1"
constant="true"/>
            </listOfProducts>
            <fbc:geneProductAssociation>
                  <fbc:or>
                        <fbc:geneProductRef fbc:geneProduct="SAUSA300_1491"/>
                        <fbc:geneProductRef fbc:geneProduct="SAUSA300_1869"/>
                        <fbc:geneProductRef fbc:geneProduct="SAUSA300_0845"/>
                        <fbc:geneProductRef fbc:geneProduct="SAUSA300_1860"/>
                  </fbc:or>
            </fbc:geneProductAssociation>
      </reaction>

      <reaction metaid="R_AMPEP11" id="R_AMPEP11" name="aminopeptidase "
reversible="false" fast="false" fbc:lowerFluxBound="irr_lb"
fbc:upperFluxBound="irr_ub">
            <listOfReactants>
                  <speciesReference species="M_h2o_c" stoichiometry="1"
constant="true"/>
                  <speciesReference species="M_gly__pro__L_c"
stoichiometry="1" constant="true"/>
            </listOfReactants>
            <listOfProducts>
                  <speciesReference species="M_h_c" stoichiometry="1"
constant="true"/>
                  <speciesReference species="M_pro__L_c" stoichiometry="1"
constant="true"/>
                  <speciesReference species="M_gly_c" stoichiometry="1"
constant="true"/>
            </listOfProducts>
            <fbc:geneProductAssociation>
                  <fbc:or>
                        <fbc:geneProductRef fbc:geneProduct="SAUSA300_1491"/>
                        <fbc:geneProductRef fbc:geneProduct="SAUSA300_1869"/>
                        <fbc:geneProductRef fbc:geneProduct="SAUSA300_0845"/>
                        <fbc:geneProductRef fbc:geneProduct="SAUSA300_1860"/>
                  </fbc:or>
            </fbc:geneProductAssociation>
      </reaction>

      <reaction metaid="R_AMPEP12" id="R_AMPEP12" name="aminopeptidase "
reversible="true" fast="false" fbc:lowerFluxBound="rev_lb"
fbc:upperFluxBound="rev_ub">
            <listOfReactants>
                  <speciesReference species="M_h2o_c" stoichiometry="1"
constant="true"/>
                  <speciesReference species="M_ala_gln_c" stoichiometry="1"
constant="true"/>
            </listOfReactants>
            <listOfProducts>
                  <speciesReference species="M_gln__L_c" stoichiometry="1"
constant="true"/>
                  <speciesReference species="M_ala__L_c" stoichiometry="1"
constant="true"/>
            </listOfProducts>
            <fbc:geneProductAssociation>
                  <fbc:or>
                        <fbc:geneProductRef fbc:geneProduct="SAUSA300_1491"/>
```

```
                        <fbc:geneProductRef fbc:geneProduct="SAUSA300_1869"/>
                        <fbc:geneProductRef fbc:geneProduct="SAUSA300_0845"/>
                        <fbc:geneProductRef fbc:geneProduct="SAUSA300_1860"/>
                  </fbc:or>
            </fbc:geneProductAssociation>
      </reaction>

      <reaction metaid="R_AMPEP13" id="R_AMPEP13" name="aminopeptidase "
reversible="true" fast="false" fbc:lowerFluxBound="rev_lb"
fbc:upperFluxBound="rev_ub">
            <listOfReactants>
                  <speciesReference species="M_h2o_c" stoichiometry="1"
constant="true"/>
                  <speciesReference species="M_ala__L_asp__L_c"
stoichiometry="1" constant="true"/>
            </listOfReactants>
            <listOfProducts>
                  <speciesReference species="M_ala__L_c" stoichiometry="1"
constant="true"/>
                  <speciesReference species="M_asp__L_c" stoichiometry="1"
constant="true"/>
            </listOfProducts>
            <fbc:geneProductAssociation>
                  <fbc:or>
                        <fbc:geneProductRef fbc:geneProduct="SAUSA300_1491"/>
                        <fbc:geneProductRef fbc:geneProduct="SAUSA300_1869"/>
                        <fbc:geneProductRef fbc:geneProduct="SAUSA300_0845"/>
                        <fbc:geneProductRef fbc:geneProduct="SAUSA300_1860"/>
                  </fbc:or>
            </fbc:geneProductAssociation>
      </reaction>

      <reaction metaid="R_AMPEP14" id="R_AMPEP14" name="aminopeptidase "
reversible="true" fast="false" fbc:lowerFluxBound="rev_lb"
fbc:upperFluxBound="rev_ub">
            <listOfReactants>
                  <speciesReference species="M_h_c" stoichiometry="1"
constant="true"/>
                  <speciesReference species="M_h2o_c" stoichiometry="1"
constant="true"/>
                  <speciesReference species="M_met__L_ala__L_c"
stoichiometry="1" constant="true"/>
            </listOfReactants>
            <listOfProducts>
                  <speciesReference species="M_ala__L_c" stoichiometry="1"
constant="true"/>
                  <speciesReference species="M_met__L_c" stoichiometry="1"
constant="true"/>
            </listOfProducts>
            <fbc:geneProductAssociation>
                  <fbc:or>
                        <fbc:geneProductRef fbc:geneProduct="SAUSA300_1491"/>
                        <fbc:geneProductRef fbc:geneProduct="SAUSA300_1869"/>
                        <fbc:geneProductRef fbc:geneProduct="SAUSA300_0845"/>
                        <fbc:geneProductRef fbc:geneProduct="SAUSA300_1860"/>
                  </fbc:or>
            </fbc:geneProductAssociation>
      </reaction>
```

```
      <reaction metaid="R_AMPEP2" id="R_AMPEP2" name="aminopeptidase "
reversible="true" fast="false" fbc:lowerFluxBound="rev_lb"
fbc:upperFluxBound="rev_ub">
            <listOfReactants>
                  <speciesReference species="M_h2o_c" stoichiometry="1"
constant="true"/>
                  <speciesReference species="M_ala__L_thr__L_c"
stoichiometry="1" constant="true"/>
            </listOfReactants>
            <listOfProducts>
                  <speciesReference species="M_thr__L_c" stoichiometry="1"
constant="true"/>
                  <speciesReference species="M_ala__L_c" stoichiometry="1"
constant="true"/>
            </listOfProducts>
            <fbc:geneProductAssociation>
                  <fbc:or>
                        <fbc:geneProductRef fbc:geneProduct="SAUSA300_1491"/>
                        <fbc:geneProductRef fbc:geneProduct="SAUSA300_1869"/>
                        <fbc:geneProductRef fbc:geneProduct="SAUSA300_0845"/>
                        <fbc:geneProductRef fbc:geneProduct="SAUSA300_1860"/>
                  </fbc:or>
            </fbc:geneProductAssociation>
      </reaction>

      <reaction metaid="R_AMPEP3" id="R_AMPEP3" name="aminopeptidase "
reversible="true" fast="false" fbc:lowerFluxBound="rev_lb"
fbc:upperFluxBound="rev_ub">
            <listOfReactants>
                  <speciesReference species="M_h2o_c" stoichiometry="1"
constant="true"/>
                  <speciesReference species="M_gly_gln_c" stoichiometry="1"
constant="true"/>
            </listOfReactants>
            <listOfProducts>
                  <speciesReference species="M_gln__L_c" stoichiometry="1"
constant="true"/>
                  <speciesReference species="M_gly_c" stoichiometry="1"
constant="true"/>
            </listOfProducts>
            <fbc:geneProductAssociation>
                  <fbc:or>
                        <fbc:geneProductRef fbc:geneProduct="SAUSA300_1491"/>
                        <fbc:geneProductRef fbc:geneProduct="SAUSA300_1869"/>
                        <fbc:geneProductRef fbc:geneProduct="SAUSA300_0845"/>
                        <fbc:geneProductRef fbc:geneProduct="SAUSA300_1860"/>
                  </fbc:or>
            </fbc:geneProductAssociation>
      </reaction>

      <reaction metaid="R_AMPEP4" id="R_AMPEP4" name="aminopeptidase "
reversible="true" fast="false" fbc:lowerFluxBound="rev_lb"
fbc:upperFluxBound="rev_ub">
            <listOfReactants>
                  <speciesReference species="M_h2o_c" stoichiometry="1"
constant="true"/>
```

```
                  <speciesReference species="M_alagly_c" stoichiometry="1"
constant="true"/>
            </listOfReactants>
            <listOfProducts>
                  <speciesReference species="M_gly_c" stoichiometry="1"
constant="true"/>
                  <speciesReference species="M_ala__L_c" stoichiometry="1"
constant="true"/>
            </listOfProducts>
            <fbc:geneProductAssociation>
                  <fbc:or>
                        <fbc:geneProductRef fbc:geneProduct="SAUSA300_1491"/>
                        <fbc:geneProductRef fbc:geneProduct="SAUSA300_1869"/>
                        <fbc:geneProductRef fbc:geneProduct="SAUSA300_0845"/>
                        <fbc:geneProductRef fbc:geneProduct="SAUSA300_1860"/>
                  </fbc:or>
            </fbc:geneProductAssociation>
      </reaction>

      <reaction metaid="R_AMPEP5" id="R_AMPEP5" name="aminopeptidase "
reversible="true" fast="false" fbc:lowerFluxBound="rev_lb"
fbc:upperFluxBound="rev_ub">
            <listOfReactants>
                  <speciesReference species="M_h2o_c" stoichiometry="1"
constant="true"/>
                  <speciesReference species="M_ala__L_glu__L_c"
stoichiometry="1" constant="true"/>
            </listOfReactants>
            <listOfProducts>
                  <speciesReference species="M_glu__L_c" stoichiometry="1"
constant="true"/>
                  <speciesReference species="M_ala__L_c" stoichiometry="1"
constant="true"/>
            </listOfProducts>
            <fbc:geneProductAssociation>
                  <fbc:or>
                        <fbc:geneProductRef fbc:geneProduct="SAUSA300_1491"/>
                        <fbc:geneProductRef fbc:geneProduct="SAUSA300_1869"/>
                        <fbc:geneProductRef fbc:geneProduct="SAUSA300_0845"/>
                        <fbc:geneProductRef fbc:geneProduct="SAUSA300_1860"/>
                  </fbc:or>
            </fbc:geneProductAssociation>
      </reaction>

      <reaction metaid="R_AMPEP6" id="R_AMPEP6" name="aminopeptidase "
reversible="true" fast="false" fbc:lowerFluxBound="rev_lb"
fbc:upperFluxBound="rev_ub">
            <listOfReactants>
                  <speciesReference species="M_h2o_c" stoichiometry="1"
constant="true"/>
                  <speciesReference species="M_ala_leu_c" stoichiometry="1"
constant="true"/>
            </listOfReactants>
            <listOfProducts>
                  <speciesReference species="M_ala__L_c" stoichiometry="1"
constant="true"/>
                  <speciesReference species="M_leu__L_c" stoichiometry="1"
constant="true"/>
```

```
            &lt;/listOfProducts&gt;
            &lt;fbc:geneProductAssociation&gt;
                  &lt;fbc:or&gt;
                        &lt;fbc:geneProductRef fbc:geneProduct="SAUSA300_1491"/&gt;
                        &lt;fbc:geneProductRef fbc:geneProduct="SAUSA300_1869"/&gt;
                        &lt;fbc:geneProductRef fbc:geneProduct="SAUSA300_0845"/&gt;
                        &lt;fbc:geneProductRef fbc:geneProduct="SAUSA300_1860"/&gt;
                  &lt;/fbc:or&gt;
            &lt;/fbc:geneProductAssociation&gt;
      &lt;/reaction&gt;

      <reaction metaid="R_AMPEP7" id="R_AMPEP7" name="aminopeptidase "
reversible="true" fast="false" fbc:lowerFluxBound="rev_lb"
fbc:upperFluxBound="rev_ub">
            &lt;listOfReactants&gt;
                  <speciesReference species="M_h2o_c" stoichiometry="1"
constant="true"/>
                  <speciesReference species="M_ala_his_c" stoichiometry="1"
constant="true"/>
            &lt;/listOfReactants&gt;
            &lt;listOfProducts&gt;
                  <speciesReference species="M_his__L_c" stoichiometry="1"
constant="true"/>
                  <speciesReference species="M_ala__L_c" stoichiometry="1"
constant="true"/>
            &lt;/listOfProducts&gt;
            &lt;fbc:geneProductAssociation&gt;
                  &lt;fbc:or&gt;
                        &lt;fbc:geneProductRef fbc:geneProduct="SAUSA300_1491"/&gt;
                        &lt;fbc:geneProductRef fbc:geneProduct="SAUSA300_1869"/&gt;
                        &lt;fbc:geneProductRef fbc:geneProduct="SAUSA300_0845"/&gt;
                        &lt;fbc:geneProductRef fbc:geneProduct="SAUSA300_1860"/&gt;
                  &lt;/fbc:or&gt;
            &lt;/fbc:geneProductAssociation&gt;
      &lt;/reaction&gt;

      <reaction metaid="R_AMPEP8" id="R_AMPEP8" name="aminopeptidase "
reversible="true" fast="false" fbc:lowerFluxBound="rev_lb"
fbc:upperFluxBound="rev_ub">
            &lt;listOfReactants&gt;
                  <speciesReference species="M_h2o_c" stoichiometry="1"
constant="true"/>
                  <speciesReference species="M_gly_glu__L_c"
stoichiometry="1" constant="true"/>
            &lt;/listOfReactants&gt;
            &lt;listOfProducts&gt;
                  <speciesReference species="M_glu__L_c" stoichiometry="1"
constant="true"/>
                  <speciesReference species="M_gly_c" stoichiometry="1"
constant="true"/>
            &lt;/listOfProducts&gt;
            &lt;fbc:geneProductAssociation&gt;
                  &lt;fbc:or&gt;
                        &lt;fbc:geneProductRef fbc:geneProduct="SAUSA300_1491"/&gt;
                        &lt;fbc:geneProductRef fbc:geneProduct="SAUSA300_1869"/&gt;
                        &lt;fbc:geneProductRef fbc:geneProduct="SAUSA300_0845"/&gt;
                        &lt;fbc:geneProductRef fbc:geneProduct="SAUSA300_1860"/&gt;
                  &lt;/fbc:or&gt;
```

```
            &lt;/fbc:geneProductAssociation&gt;
      &lt;/reaction&gt;

      <reaction metaid="R_AMPEP9" id="R_AMPEP9" name="aminopeptidase "
reversible="true" fast="false" fbc:lowerFluxBound="rev_lb"
fbc:upperFluxBound="rev_ub">
            &lt;listOfReactants&gt;
                  <speciesReference species="M_h2o_c" stoichiometry="1"
constant="true"/>
                  <speciesReference species="M_gly_met_c" stoichiometry="1"
constant="true"/>
            &lt;/listOfReactants&gt;
            &lt;listOfProducts&gt;
                  <speciesReference species="M_gly_c" stoichiometry="1"
constant="true"/>
                  <speciesReference species="M_met__L_c" stoichiometry="1"
constant="true"/>
            &lt;/listOfProducts&gt;
            &lt;fbc:geneProductAssociation&gt;
                  &lt;fbc:or&gt;
                        &lt;fbc:geneProductRef fbc:geneProduct="SAUSA300_1491"/&gt;
                        &lt;fbc:geneProductRef fbc:geneProduct="SAUSA300_1869"/&gt;
                        &lt;fbc:geneProductRef fbc:geneProduct="SAUSA300_0845"/&gt;
                        &lt;fbc:geneProductRef fbc:geneProduct="SAUSA300_1860"/&gt;
                  &lt;/fbc:or&gt;
            &lt;/fbc:geneProductAssociation&gt;
      &lt;/reaction&gt;

      <reaction metaid="R_AMPTASECG" id="R_AMPTASECG" name="alanyl
aminopeptidase (cys-gly)" reversible="false" fast="false"
fbc:lowerFluxBound="irr_lb" fbc:upperFluxBound="irr_ub">
            &lt;listOfReactants&gt;
                  <speciesReference species="M_h2o_c" stoichiometry="1"
constant="true"/>
                  <speciesReference species="M_cgly_c" stoichiometry="1"
constant="true"/>
            &lt;/listOfReactants&gt;
            &lt;listOfProducts&gt;
                  <speciesReference species="M_gly_c" stoichiometry="1"
constant="true"/>
                  <speciesReference species="M_cys__L_c" stoichiometry="1"
constant="true"/>
            &lt;/listOfProducts&gt;
            &lt;fbc:geneProductAssociation&gt;
                  &lt;fbc:or&gt;
                        &lt;fbc:geneProductRef fbc:geneProduct="SAUSA300_1491"/&gt;
                        &lt;fbc:geneProductRef fbc:geneProduct="SAUSA300_1869"/&gt;
                        &lt;fbc:geneProductRef fbc:geneProduct="SAUSA300_0845"/&gt;
                        &lt;fbc:geneProductRef fbc:geneProduct="SAUSA300_1860"/&gt;
                  &lt;/fbc:or&gt;
            &lt;/fbc:geneProductAssociation&gt;
      &lt;/reaction&gt;

      <reaction metaid="R_ANDPP" id="R_ANDPP" name="CDP-glycerol
polyglycerophosphate glycerophosphotransferase " reversible="true"
fast="false" fbc:lowerFluxBound="rev_lb" fbc:upperFluxBound="rev_ub">
            &lt;listOfReactants&gt;
```

```
                  <speciesReference species="M_cdpglyc_c" stoichiometry="45"
constant="true"/>
                  <speciesReference species="M_nacetlbdgl_c"
stoichiometry="1" constant="true"/>
            </listOfReactants>
            <listOfProducts>
                  <speciesReference species="M_cmp_c" stoichiometry="45"
constant="true"/>
                  <speciesReference species="M_pren45_c" stoichiometry="1"
constant="true"/>
            </listOfProducts>
            <fbc:geneProductAssociation>
                  <fbc:or>
                        <fbc:geneProductRef fbc:geneProduct="SAUSA300_0626"/>
                        <fbc:geneProductRef fbc:geneProduct="SAUSA300_0247"/>
                  </fbc:or>
            </fbc:geneProductAssociation>
      </reaction>

      <reaction metaid="R_ANDUG" id="R_ANDUG" name="anteisopentadecanoyl-UDP-
glucosyltransferase monoglucosyl " reversible="true" fast="false"
fbc:lowerFluxBound="rev_lb" fbc:upperFluxBound="rev_ub">
            <listOfReactants>
                  <speciesReference species="M_udpg_c" stoichiometry="1"
constant="true"/>
                  <speciesReference species="M_12diasglyc_c"
stoichiometry="1" constant="true"/>
            </listOfReactants>
            <listOfProducts>
                  <speciesReference species="M_udp_c" stoichiometry="1"
constant="true"/>
                  <speciesReference species="M_m12diagly_c" stoichiometry="1"
constant="true"/>
            </listOfProducts>
            <fbc:geneProductAssociation>
                  <fbc:geneProductRef fbc:geneProduct="SAUSA300_0918"/>
            </fbc:geneProductAssociation>
      </reaction>

      <reaction metaid="R_ANHGLM" id="R_ANHGLM" name="anteisoheptadecanoyl-
UDP-glucosyltransferase monoglucosyl " reversible="true" fast="false"
fbc:lowerFluxBound="rev_lb" fbc:upperFluxBound="rev_ub">
            <listOfReactants>
                  <speciesReference species="M_udpg_c" stoichiometry="1"
constant="true"/>
                  <speciesReference species="M_12disgly_c" stoichiometry="1"
constant="true"/>
            </listOfReactants>
            <listOfProducts>
                  <speciesReference species="M_udp_c" stoichiometry="1"
constant="true"/>
                  <speciesReference species="M_m12dhdeglyc_c"
stoichiometry="1" constant="true"/>
            </listOfProducts>
            <fbc:geneProductAssociation>
                  <fbc:geneProductRef fbc:geneProduct="SAUSA300_0918"/>
            </fbc:geneProductAssociation>
      </reaction>
```

```
      <reaction metaid="R_ANHTPP" id="R_ANHTPP" name="anteisoheptadecanoyl-
phosphatidylserine decarboxylase " reversible="true" fast="false"
fbc:lowerFluxBound="rev_lb" fbc:upperFluxBound="rev_ub">
            <listOfReactants>
                  <speciesReference species="M_h_c" stoichiometry="2"
constant="true"/>
                  <speciesReference species="M_dianphaser_c"
stoichiometry="1" constant="true"/>
            </listOfReactants>
            <listOfProducts>
                  <speciesReference species="M_co2_c" stoichiometry="1"
constant="true"/>
                  <speciesReference species="M_dianethal_c" stoichiometry="1"
constant="true"/>
            </listOfProducts>
            <fbc:geneProductAssociation>
                  <fbc:geneProductRef fbc:geneProduct="SAUSA300_1973"/>
            </fbc:geneProductAssociation>
      </reaction>

      <reaction metaid="R_ANICARD" id="R_ANICARD" name="anteisoheptadecanoyl-
cardiolipin synthase " reversible="true" fast="false"
fbc:lowerFluxBound="rev_lb" fbc:upperFluxBound="rev_ub">
            <listOfReactants>
                  <speciesReference species="M_dianhppglyc_c"
stoichiometry="2" constant="true"/>
            </listOfReactants>
            <listOfProducts>
                  <speciesReference species="M_glyc_c" stoichiometry="1"
constant="true"/>
                  <speciesReference species="M_antcard_c" stoichiometry="1"
constant="true"/>
            </listOfProducts>
            <fbc:geneProductAssociation>
                  <fbc:or>
                        <fbc:geneProductRef fbc:geneProduct="SAUSA300_2044"/>
                        <fbc:geneProductRef fbc:geneProduct="SAUSA300_1216"/>
                  </fbc:or>
            </fbc:geneProductAssociation>
      </reaction>

      <reaction metaid="R_ANLAGL" id="R_ANLAGL"
name="anteisoheptadecanoyl__Lipoteichoic acid synthesis n=24  linked  glucose
substituted " reversible="true" fast="false" fbc:lowerFluxBound="rev_lb"
fbc:upperFluxBound="rev_ub">
            <listOfReactants>
                  <speciesReference species="M_udpg_c" stoichiometry="24"
constant="true"/>
                  <speciesReference species="M_ant24u_c" stoichiometry="1"
constant="true"/>
            </listOfReactants>
            <listOfProducts>
                  <speciesReference species="M_udp_c" stoichiometry="24"
constant="true"/>
                  <speciesReference species="M_antla24s_c" stoichiometry="1"
constant="true"/>
            </listOfProducts>
```

```
            <fbc:geneProductAssociation>
                  <fbc:or>
                        <fbc:geneProductRef fbc:geneProduct="SAUSA300_0939"/>
                        <fbc:geneProductRef fbc:geneProduct="SAUSA300_0550"/>
                        <fbc:geneProductRef fbc:geneProduct="SAUSA300_0549"/>
                  </fbc:or>
            </fbc:geneProductAssociation>
      </reaction>

      <reaction metaid="R_ANPRT" id="R_ANPRT" name="anthranilate
phosphoribosyltransferase" reversible="false" fast="false"
fbc:lowerFluxBound="irr_lb" fbc:upperFluxBound="irr_ub">
            <listOfReactants>
                  <speciesReference species="M_anth_c" stoichiometry="1"
constant="true"/>
                  <speciesReference species="M_prpp_c" stoichiometry="1"
constant="true"/>
            </listOfReactants>
            <listOfProducts>
                  <speciesReference species="M_ppi_c" stoichiometry="1"
constant="true"/>
                  <speciesReference species="M_pran_c" stoichiometry="1"
constant="true"/>
            </listOfProducts>
            <fbc:geneProductAssociation>
                  <fbc:geneProductRef fbc:geneProduct="SAUSA300_1264"/>
            </fbc:geneProductAssociation>
      </reaction>

      <reaction metaid="R_ANPTTP" id="R_ANPTTP" name="anteisopentadecanoyl-
phosphatidate cytidylyltransferase " reversible="true" fast="false"
fbc:lowerFluxBound="rev_lb" fbc:upperFluxBound="rev_ub">
            <listOfReactants>
                  <speciesReference species="M_ctp_c" stoichiometry="1"
constant="true"/>
                  <speciesReference species="M_12diasn3_c" stoichiometry="1"
constant="true"/>
            </listOfReactants>
            <listOfProducts>
                  <speciesReference species="M_ppi_c" stoichiometry="1"
constant="true"/>
                  <speciesReference species="M_c12dpdgly_c" stoichiometry="1"
constant="true"/>
            </listOfProducts>
            <fbc:geneProductAssociation>
                  <fbc:geneProductRef fbc:geneProduct="SAUSA300_1154"/>
            </fbc:geneProductAssociation>
      </reaction>

      <reaction metaid="R_ANS" id="R_ANS" name="anthranilate synthase"
reversible="false" fast="false" fbc:lowerFluxBound="irr_lb"
fbc:upperFluxBound="irr_ub">
            <listOfReactants>
                  <speciesReference species="M_gln__L_c" stoichiometry="1"
constant="true"/>
                  <speciesReference species="M_chor_c" stoichiometry="1"
constant="true"/>
            </listOfReactants>
```

```
            <listOfProducts>
                  <speciesReference species="M_h_c" stoichiometry="1"
constant="true"/>
                  <speciesReference species="M_glu__L_c" stoichiometry="1"
constant="true"/>
                  <speciesReference species="M_pyr_c" stoichiometry="1"
constant="true"/>
                  <speciesReference species="M_anth_c" stoichiometry="1"
constant="true"/>
            </listOfProducts>
            <fbc:geneProductAssociation>
                  <fbc:and>
                        <fbc:geneProductRef fbc:geneProduct="SAUSA300_1263"/>
                        <fbc:geneProductRef fbc:geneProduct="SAUSA300_1262"/>
                  </fbc:and>
            </fbc:geneProductAssociation>
      </reaction>

      <reaction metaid="R_ANS2" id="R_ANS2" name="anthranilate synthase 2"
reversible="false" fast="false" fbc:lowerFluxBound="irr_lb"
fbc:upperFluxBound="irr_ub">
            <listOfReactants>
                  <speciesReference species="M_nh4_c" stoichiometry="1"
constant="true"/>
                  <speciesReference species="M_chor_c" stoichiometry="1"
constant="true"/>
            </listOfReactants>
            <listOfProducts>
                  <speciesReference species="M_h_c" stoichiometry="1"
constant="true"/>
                  <speciesReference species="M_h2o_c" stoichiometry="1"
constant="true"/>
                  <speciesReference species="M_pyr_c" stoichiometry="1"
constant="true"/>
                  <speciesReference species="M_anth_c" stoichiometry="1"
constant="true"/>
            </listOfProducts>
            <fbc:geneProductAssociation>
                  <fbc:and>
                        <fbc:geneProductRef fbc:geneProduct="SAUSA300_1263"/>
                        <fbc:geneProductRef fbc:geneProduct="SAUSA300_1262"/>
                  </fbc:and>
            </fbc:geneProductAssociation>
      </reaction>

      <reaction metaid="R_ANT1A3P" id="R_ANT1A3P" name="anteisoheptadecanoyl-
1-acylglycerol-3-phosphate O-acyltransferase " reversible="false"
fast="false" fbc:lowerFluxBound="irr_lb" fbc:upperFluxBound="irr_ub">
            <listOfReactants>
                  <speciesReference species="M_fa12coa_c" stoichiometry="1"
constant="true"/>
                  <speciesReference species="M_iaidsg3p_c" stoichiometry="1"
constant="true"/>
            </listOfReactants>
            <listOfProducts>
                  <speciesReference species="M_coa_c" stoichiometry="1"
constant="true"/>
```

```
                  <speciesReference species="M_12ddgly3p_c" stoichiometry="1"
constant="true"/>
            </listOfProducts>
            <fbc:geneProductAssociation>
                  <fbc:geneProductRef fbc:geneProduct="SAUSA300_1673"/>
            </fbc:geneProductAssociation>
      </reaction>

      <reaction metaid="R_ANTCDPG" id="R_ANTCDPG" name="anteisoheptadecanoyl-
CDPdiacylglycerol sn-glycerol-3-phosphate 3-phosphatidyltransferase "
reversible="true" fast="false" fbc:lowerFluxBound="rev_lb"
fbc:upperFluxBound="rev_ub">
            <listOfReactants>
                  <speciesReference species="M_glyc3p_c" stoichiometry="1"
constant="true"/>
                  <speciesReference species="M_c12dihgly_c" stoichiometry="1"
constant="true"/>
            </listOfReactants>
            <listOfProducts>
                  <speciesReference species="M_cmp_c" stoichiometry="1"
constant="true"/>
                  <speciesReference species="M_diihpgp_c" stoichiometry="1"
constant="true"/>
            </listOfProducts>
            <fbc:geneProductAssociation>
                  <fbc:geneProductRef fbc:geneProduct="SAUSA300_1176"/>
            </fbc:geneProductAssociation>
      </reaction>

      <reaction metaid="R_ANTGLPT" id="R_ANTGLPT" name="anteisoheptadecanoyl-
glycerol-3-phosphate O-acyltransferase " reversible="false" fast="false"
fbc:lowerFluxBound="irr_lb" fbc:upperFluxBound="irr_ub">
            <listOfReactants>
                  <speciesReference species="M_glyc3p_c" stoichiometry="1"
constant="true"/>
                  <speciesReference species="M_fa12coa_c" stoichiometry="1"
constant="true"/>
            </listOfReactants>
            <listOfProducts>
                  <speciesReference species="M_coa_c" stoichiometry="1"
constant="true"/>
                  <speciesReference species="M_iaidsg3p_c" stoichiometry="1"
constant="true"/>
            </listOfProducts>
            <fbc:geneProductAssociation>
                  <fbc:or>
                        <fbc:geneProductRef fbc:geneProduct="SAUSA300_0190"/>
                        <fbc:geneProductRef fbc:geneProduct="SAUSA300_2166"/>
                        <fbc:and>
                              <fbc:geneProductRef
fbc:geneProduct="SAUSA300_0993"/>
                              <fbc:geneProductRef
fbc:geneProduct="SAUSA300_0994"/>
                        </fbc:and>
                        <fbc:and>
                              <fbc:geneProductRef
fbc:geneProduct="SAUSA300_2007"/>
```

```
                              <fbc:geneProductRef
fbc:geneProduct="SAUSA300_2008"/>
                        </fbc:and>
                  </fbc:or>
            </fbc:geneProductAssociation>
      </reaction>

      <reaction metaid="R_ANTGT" id="R_ANTGT" name="anteisoheptadecanoyl-UDP-
glucosyltransferase diglucosyl " reversible="true" fast="false"
fbc:lowerFluxBound="rev_lb" fbc:upperFluxBound="rev_ub">
            <listOfReactants>
                  <speciesReference species="M_udpg_c" stoichiometry="1"
constant="true"/>
                  <speciesReference species="M_m12dhdeglyc_c"
stoichiometry="1" constant="true"/>
            </listOfReactants>
            <listOfProducts>
                  <speciesReference species="M_udp_c" stoichiometry="1"
constant="true"/>
                  <speciesReference species="M_dglu12did_c" stoichiometry="1"
constant="true"/>
            </listOfProducts>
            <fbc:geneProductAssociation>
                  <fbc:geneProductRef fbc:geneProduct="SAUSA300_0918"/>
            </fbc:geneProductAssociation>
      </reaction>

      <reaction metaid="R_ANTILIPS" id="R_ANTILIPS"
name="anteisoheptadecanoyl__Lipoteichoic acid synthesis n=24  linked  N-
acetylglucosamine substituted " reversible="true" fast="false"
fbc:lowerFluxBound="rev_lb" fbc:upperFluxBound="rev_ub">
            <listOfReactants>
                  <speciesReference species="M_uacgam_c" stoichiometry="24"
constant="true"/>
                  <speciesReference species="M_ant24u_c" stoichiometry="1"
constant="true"/>
            </listOfReactants>
            <listOfProducts>
                  <speciesReference species="M_udp_c" stoichiometry="24"
constant="true"/>
                  <speciesReference species="M_ant24Nacds_c"
stoichiometry="1" constant="true"/>
            </listOfProducts>
            <fbc:geneProductAssociation>
                  <fbc:geneProductRef fbc:geneProduct="SAUSA300_0731"/>
            </fbc:geneProductAssociation>
      </reaction>

      <reaction metaid="R_ANTIP" id="R_ANTIP" name="anteisopentadecanoyl-1-
acylglycerol-3-phosphate O-acyltransferase " reversible="false" fast="false"
fbc:lowerFluxBound="irr_lb" fbc:upperFluxBound="irr_ub">
            <listOfReactants>
                  <speciesReference species="M_1aipsg3p_c" stoichiometry="1"
constant="true"/>
                  <speciesReference species="M_fa4coa_c" stoichiometry="1"
constant="true"/>
            </listOfReactants>
            <listOfProducts>
```

```
                  <speciesReference species="M_12diasn3_c" stoichiometry="1"
constant="true"/>
                  <speciesReference species="M_coa_c" stoichiometry="1"
constant="true"/>
            </listOfProducts>
            <fbc:geneProductAssociation>
                  <fbc:geneProductRef fbc:geneProduct="SAUSA300_1673"/>
            </fbc:geneProductAssociation>
      </reaction>

      <reaction metaid="R_ANTLAS" id="R_ANTLAS"
name="anteisoheptadecanoyl__Lipoteichoic acid synthesis n=24  unlinked  D-
alanine substituted " reversible="false" fast="false"
fbc:lowerFluxBound="irr_lb" fbc:upperFluxBound="irr_ub">
            <listOfReactants>
                  <speciesReference species="M_atp_c" stoichiometry="24"
constant="true"/>
                  <speciesReference species="M_ala__D_c" stoichiometry="24"
constant="true"/>
                  <speciesReference species="M_ant24u_c" stoichiometry="1"
constant="true"/>
            </listOfReactants>
            <listOfProducts>
                  <speciesReference species="M_ppi_c" stoichiometry="24"
constant="true"/>
                  <speciesReference species="M_amp_c" stoichiometry="24"
constant="true"/>
                  <speciesReference species="M_ant24ds_c" stoichiometry="1"
constant="true"/>
            </listOfProducts>
            <fbc:geneProductAssociation>
                  <fbc:geneProductRef fbc:geneProduct="SAUSA300_0838"/>
            </fbc:geneProductAssociation>
      </reaction>

      <reaction metaid="R_ANTLPSYN" id="R_ANTLPSYN"
name="anteisopentadecanoyl__Lipoteichoic acid synthesis n=24  linked  glucose
substituted " reversible="true" fast="false" fbc:lowerFluxBound="rev_lb"
fbc:upperFluxBound="rev_ub">
            <listOfReactants>
                  <speciesReference species="M_udpg_c" stoichiometry="24"
constant="true"/>
                  <speciesReference species="M_antp24u_c" stoichiometry="1"
constant="true"/>
            </listOfReactants>
            <listOfProducts>
                  <speciesReference species="M_udp_c" stoichiometry="24"
constant="true"/>
                  <speciesReference species="M_ant24glcs_c" stoichiometry="1"
constant="true"/>
            </listOfProducts>
            <fbc:geneProductAssociation>
                  <fbc:or>
                        <fbc:geneProductRef fbc:geneProduct="SAUSA300_0939"/>
                        <fbc:geneProductRef fbc:geneProduct="SAUSA300_0550"/>
                        <fbc:geneProductRef fbc:geneProduct="SAUSA300_0549"/>
                  </fbc:or>
            </fbc:geneProductAssociation>
```

```
        </reaction>

        <reaction metaid="R_ANTPAP" id="R_ANTPAP" name="anteisoheptadecanoyl-
phosphatidic acid phosphatase " reversible="false" fast="false"
fbc:lowerFluxBound="irr_lb" fbc:upperFluxBound="irr_ub">
            <listOfReactants>
                <speciesReference species="M_h2o_c" stoichiometry="1"
constant="true"/>
                <speciesReference species="M_12ddgly3p_c" stoichiometry="1"
constant="true"/>
            </listOfReactants>
            <listOfProducts>
                <speciesReference species="M_h_c" stoichiometry="1"
constant="true"/>
                <speciesReference species="M_pi_c" stoichiometry="1"
constant="true"/>
                <speciesReference species="M_12disgly_c" stoichiometry="1"
constant="true"/>
            </listOfProducts>
            <fbc:geneProductAssociation>
                <fbc:geneProductRef fbc:geneProduct="SAUSA300_1529"/>
            </fbc:geneProductAssociation>
        </reaction>

        <reaction metaid="R_ANTPCARS" id="R_ANTPCARS"
name="anteisopentadecanoyl-cardiolipin synthase " reversible="true"
fast="false" fbc:lowerFluxBound="rev_lb" fbc:upperFluxBound="rev_ub">
            <listOfReactants>
                <speciesReference species="M_dianppglyc_c"
stoichiometry="2" constant="true"/>
            </listOfReactants>
            <listOfProducts>
                <speciesReference species="M_glyc_c" stoichiometry="1"
constant="true"/>
                <speciesReference species="M_anipcard_c" stoichiometry="1"
constant="true"/>
            </listOfProducts>
            <fbc:geneProductAssociation>
                <fbc:or>
                    <fbc:geneProductRef fbc:geneProduct="SAUSA300_2044"/>
                    <fbc:geneProductRef fbc:geneProduct="SAUSA300_1216"/>
                </fbc:or>
            </fbc:geneProductAssociation>
        </reaction>

        <reaction metaid="R_ANTPCY" id="R_ANTPCY" name="anteisoheptadecanoyl-
phosphatidate cytidylyltransferase " reversible="true" fast="false"
fbc:lowerFluxBound="rev_lb" fbc:upperFluxBound="rev_ub">
            <listOfReactants>
                <speciesReference species="M_ctp_c" stoichiometry="1"
constant="true"/>
                <speciesReference species="M_12ddgly3p_c" stoichiometry="1"
constant="true"/>
            </listOfReactants>
            <listOfProducts>
                <speciesReference species="M_ppi_c" stoichiometry="1"
constant="true"/>
```

```
                  <speciesReference species="M_c12dihgly_c" stoichiometry="1"
constant="true"/>
            &lt;/listOfProducts&gt;
            &lt;fbc:geneProductAssociation&gt;
                  &lt;fbc:geneProductRef fbc:geneProduct="SAUSA300_1154"/&gt;
            &lt;/fbc:geneProductAssociation&gt;
      &lt;/reaction&gt;

      <reaction metaid="R_ANTPEDIP" id="R_ANTPEDIP"
name="anteisopentadecanoyl-CDPdiacylglycerol sn-glycerol-3-phosphate 3-
phosphatidyltransferase " reversible="true" fast="false"
fbc:lowerFluxBound="rev_lb" fbc:upperFluxBound="rev_ub">
            &lt;listOfReactants&gt;
                  <speciesReference species="M_c12dpdgly_c" stoichiometry="1"
constant="true"/>
                  <speciesReference species="M_glyc3p_c" stoichiometry="1"
constant="true"/>
            &lt;/listOfReactants&gt;
            &lt;listOfProducts&gt;
                  <speciesReference species="M_cmp_c" stoichiometry="1"
constant="true"/>
                  <speciesReference species="M_diapdpgp_c" stoichiometry="1"
constant="true"/>
            &lt;/listOfProducts&gt;
            &lt;fbc:geneProductAssociation&gt;
                  &lt;fbc:geneProductRef fbc:geneProduct="SAUSA300_1176"/&gt;
            &lt;/fbc:geneProductAssociation&gt;
      &lt;/reaction&gt;

      <reaction metaid="R_ANTPGP" id="R_ANTPGP" name="anteisopentadecanoyl-
UDP-glucosyltransferase diglucosyl " reversible="true" fast="false"
fbc:lowerFluxBound="rev_lb" fbc:upperFluxBound="rev_ub">
            &lt;listOfReactants&gt;
                  <speciesReference species="M_udpg_c" stoichiometry="1"
constant="true"/>
                  <speciesReference species="M_m12diagly_c" stoichiometry="1"
constant="true"/>
            &lt;/listOfReactants&gt;
            &lt;listOfProducts&gt;
                  <speciesReference species="M_udp_c" stoichiometry="1"
constant="true"/>
                  <speciesReference species="M_dglu12dpgly_c"
stoichiometry="1" constant="true"/>
            &lt;/listOfProducts&gt;
            &lt;fbc:geneProductAssociation&gt;
                  &lt;fbc:geneProductRef fbc:geneProduct="SAUSA300_0918"/&gt;
            &lt;/fbc:geneProductAssociation&gt;
      &lt;/reaction&gt;

      <reaction metaid="R_AOBUTDs" id="R_AOBUTDs" name="L-2-amino-3-
oxobutanoate decarboxylation (spontaneous)" reversible="false" fast="false"
fbc:lowerFluxBound="irr_lb" fbc:upperFluxBound="irr_ub">
            &lt;listOfReactants&gt;
                  <speciesReference species="M_2aobut_c" stoichiometry="1"
constant="true"/>
                  <speciesReference species="M_h_c" stoichiometry="1"
constant="true"/>
            &lt;/listOfReactants&gt;
```

```
            <listOfProducts>
                  <speciesReference species="M_aact_c" stoichiometry="1"
constant="true"/>
                  <speciesReference species="M_co2_c" stoichiometry="1"
constant="true"/>
            </listOfProducts>
      </reaction>

      <reaction metaid="R_AOXSr" id="R_AOXSr" name="8-amino-7-oxononanoate
synthase" reversible="true" fast="false" fbc:lowerFluxBound="rev_lb"
fbc:upperFluxBound="rev_ub">
            <listOfReactants>
                  <speciesReference species="M_h_c" stoichiometry="1"
constant="true"/>
                  <speciesReference species="M_ala__L_c" stoichiometry="1"
constant="true"/>
                  <speciesReference species="M_pmcoa_c" stoichiometry="1"
constant="true"/>
            </listOfReactants>
            <listOfProducts>
                  <speciesReference species="M_co2_c" stoichiometry="1"
constant="true"/>
                  <speciesReference species="M_coa_c" stoichiometry="1"
constant="true"/>
                  <speciesReference species="M_8aonn_c" stoichiometry="1"
constant="true"/>
            </listOfProducts>
            <fbc:geneProductAssociation>
                  <fbc:geneProductRef fbc:geneProduct="SAUSA300_2370"/>
            </fbc:geneProductAssociation>
      </reaction>

      <reaction metaid="R_APAT" id="R_APAT" name="Acetyl-CoA L-2 3 4 5-
tetrahydrodipicolinate N2-acetyltransferase " reversible="true" fast="false"
fbc:lowerFluxBound="rev_lb" fbc:upperFluxBound="rev_ub">
            <listOfReactants>
                  <speciesReference species="M_h2o_c" stoichiometry="1"
constant="true"/>
                  <speciesReference species="M_accoa_c" stoichiometry="1"
constant="true"/>
                  <speciesReference species="M_thdp_c" stoichiometry="1"
constant="true"/>
            </listOfReactants>
            <listOfProducts>
                  <speciesReference species="M_nal2a6o_c" stoichiometry="1"
constant="true"/>
                  <speciesReference species="M_coa_c" stoichiometry="1"
constant="true"/>
            </listOfProducts>
            <fbc:geneProductAssociation>
                  <fbc:geneProductRef fbc:geneProduct="SAUSA300_1290"/>
            </fbc:geneProductAssociation>
      </reaction>

      <reaction metaid="R_APG3PAT140a" id="R_APG3PAT140a" name="myristoyl-
glycerol-3-phosphate O-acyltransferase " reversible="false" fast="false"
fbc:lowerFluxBound="irr_lb" fbc:upperFluxBound="irr_ub">
            <listOfReactants>
```

```
                  <speciesReference species="M_glyc3p_c" stoichiometry="1"
constant="true"/>
                  <speciesReference species="M_tdcoa_c" stoichiometry="1"
constant="true"/>
            </listOfReactants>
            <listOfProducts>
                  <speciesReference species="M_coa_c" stoichiometry="1"
constant="true"/>
                  <speciesReference species="M_1tdecg3p_c" stoichiometry="1"
constant="true"/>
            </listOfProducts>
            <fbc:geneProductAssociation>
                  <fbc:and>
                        <fbc:geneProductRef fbc:geneProduct="SAUSA300_1249"/>
                        <fbc:or>
                              <fbc:geneProductRef
fbc:geneProduct="SAUSA300_1122"/>
                              <fbc:geneProductRef
fbc:geneProduct="SAUSA300_1121"/>
                        </fbc:or>
                  </fbc:and>
            </fbc:geneProductAssociation>
      </reaction>

      <reaction metaid="R_APRAUR" id="R_APRAUR" name="5-amino-6-(5-
phosphoribosylamino)uracil reductase" reversible="false" fast="false"
fbc:lowerFluxBound="irr_lb" fbc:upperFluxBound="irr_ub">
            <listOfReactants>
                  <speciesReference species="M_nadp_c" stoichiometry="1"
constant="true"/>
                  <speciesReference species="M_5aprbu_c" stoichiometry="1"
constant="true"/>
            </listOfReactants>
            <listOfProducts>
                  <speciesReference species="M_h_c" stoichiometry="1"
constant="true"/>
                  <speciesReference species="M_5apru_c" stoichiometry="1"
constant="true"/>
                  <speciesReference species="M_nadph_c" stoichiometry="1"
constant="true"/>
            </listOfProducts>
            <fbc:geneProductAssociation>
                  <fbc:geneProductRef fbc:geneProduct="SAUSA300_1715"/>
            </fbc:geneProductAssociation>
      </reaction>

      <reaction metaid="R_APRTO2" id="R_APRTO2" name="N-acetylputrescine:
oxygen oxireductase (deaminating)" reversible="false" fast="false"
fbc:lowerFluxBound="irr_lb" fbc:upperFluxBound="irr_ub">
            <listOfReactants>
                  <speciesReference species="M_h2o_c" stoichiometry="1"
constant="true"/>
                  <speciesReference species="M_o2_c" stoichiometry="1"
constant="true"/>
                  <speciesReference species="M_aprut_c" stoichiometry="1"
constant="true"/>
            </listOfReactants>
            <listOfProducts>
```

```
                  <speciesReference species="M_nh4_c" stoichiometry="1"
constant="true"/>
                  <speciesReference species="M_h2o2_c" stoichiometry="1"
constant="true"/>
                  <speciesReference species="M_n4abutn_c" stoichiometry="1"
constant="true"/>
            </listOfProducts>
            <fbc:geneProductAssociation>
                  <fbc:geneProductRef fbc:geneProduct="amx2"/>
            </fbc:geneProductAssociation>
      </reaction>

      <reaction metaid="R_ARAB__L_Et" id="R_ARAB__L_Et" name="L-arabinose
transporter" reversible="true" fast="false" fbc:lowerFluxBound="rev_lb"
fbc:upperFluxBound="rev_ub">
            <listOfReactants>
                  <speciesReference species="M_arab__L_e" stoichiometry="1"
constant="true"/>
            </listOfReactants>
            <listOfProducts>
                  <speciesReference species="M_arab__L_c" stoichiometry="1"
constant="true"/>
            </listOfProducts>
      </reaction>

      <reaction metaid="R_ARABR" id="R_ARABR" name="arabinose reductase"
reversible="true" fast="false" fbc:lowerFluxBound="rev_lb"
fbc:upperFluxBound="rev_ub">
            <listOfReactants>
                  <speciesReference species="M_nadp_c" stoichiometry="1"
constant="true"/>
                  <speciesReference species="M_abt_c" stoichiometry="1"
constant="true"/>
            </listOfReactants>
            <listOfProducts>
                  <speciesReference species="M_h_c" stoichiometry="1"
constant="true"/>
                  <speciesReference species="M_nadph_c" stoichiometry="1"
constant="true"/>
                  <speciesReference species="M_arab__L_c" stoichiometry="1"
constant="true"/>
            </listOfProducts>
            <fbc:geneProductAssociation>
                  <fbc:or>
                        <fbc:geneProductRef fbc:geneProduct="SAUSA300_0688"/>
                        <fbc:geneProductRef fbc:geneProduct="SAUSA300_1728"/>
                  </fbc:or>
            </fbc:geneProductAssociation>
      </reaction>

      <reaction metaid="R_ARGDr" id="R_ARGDr" name="arginine deiminase"
reversible="false" fast="false" fbc:lowerFluxBound="irr_lb"
fbc:upperFluxBound="irr_ub">
            <listOfReactants>
                  <speciesReference species="M_h2o_c" stoichiometry="1"
constant="true"/>
                  <speciesReference species="M_arg__L_c" stoichiometry="1"
constant="true"/>
```

```xml
            </listOfReactants>
            <listOfProducts>
                  <speciesReference species="M_nh4_c" stoichiometry="1"
constant="true"/>
                  <speciesReference species="M_citr__L_c" stoichiometry="1"
constant="true"/>
            </listOfProducts>
            <fbc:geneProductAssociation>
                  <fbc:or>
                        <fbc:geneProductRef fbc:geneProduct="SAUSA300_0065"/>
                        <fbc:geneProductRef fbc:geneProduct="SA451515_2750"/>
                        <fbc:geneProductRef fbc:geneProduct="SAUSA300_2570"/>
                  </fbc:or>
            </fbc:geneProductAssociation>
      </reaction>

      <reaction metaid="R_ARGN" id="R_ARGN" name="arginase"
reversible="false" fast="false" fbc:lowerFluxBound="irr_lb"
fbc:upperFluxBound="irr_ub">
            <listOfReactants>
                  <speciesReference species="M_h2o_c" stoichiometry="1"
constant="true"/>
                  <speciesReference species="M_arg__L_c" stoichiometry="1"
constant="true"/>
            </listOfReactants>
            <listOfProducts>
                  <speciesReference species="M_orn_c" stoichiometry="1"
constant="true"/>
                  <speciesReference species="M_urea_c" stoichiometry="1"
constant="true"/>
            </listOfProducts>
            <fbc:geneProductAssociation>
                  <fbc:geneProductRef fbc:geneProduct="SAUSA300_2114"/>
            </fbc:geneProductAssociation>
      </reaction>

      <reaction metaid="R_ARGORNt7" id="R_ARGORNt7" name="arginine/ornithine
antiporter" reversible="true" fast="false" fbc:lowerFluxBound="rev_lb"
fbc:upperFluxBound="rev_ub">
            <listOfReactants>
                  <speciesReference species="M_orn_c" stoichiometry="1"
constant="true"/>
                  <speciesReference species="M_arg__L_e" stoichiometry="1"
constant="true"/>
            </listOfReactants>
            <listOfProducts>
                  <speciesReference species="M_arg__L_c" stoichiometry="1"
constant="true"/>
                  <speciesReference species="M_orn_e" stoichiometry="1"
constant="true"/>
            </listOfProducts>
            <fbc:geneProductAssociation>
                  <fbc:or>
                        <fbc:geneProductRef fbc:geneProduct="SA451515_2748"/>
                        <fbc:geneProductRef fbc:geneProduct="SAUSA300_0064"/>
                        <fbc:geneProductRef fbc:geneProduct="SAUSA300_1064"/>
                        <fbc:geneProductRef fbc:geneProduct="SAUSA300_2568"/>
                  </fbc:or>
```

```
            </fbc:geneProductAssociation>
      </reaction>

      <reaction metaid="R_ARGSL" id="R_ARGSL" name="argininosuccinate lyase"
reversible="true" fast="false" fbc:lowerFluxBound="rev_lb"
fbc:upperFluxBound="rev_ub">
            <listOfReactants>
                  <speciesReference species="M_argsuc_c" stoichiometry="1"
constant="true"/>
            </listOfReactants>
            <listOfProducts>
                  <speciesReference species="M_fum_c" stoichiometry="1"
constant="true"/>
                  <speciesReference species="M_arg__L_c" stoichiometry="1"
constant="true"/>
            </listOfProducts>
            <fbc:geneProductAssociation>
                  <fbc:geneProductRef fbc:geneProduct="SAUSA300_0863"/>
            </fbc:geneProductAssociation>
      </reaction>

      <reaction metaid="R_ARGSSr" id="R_ARGSSr" name="argininosuccinate
synthase, reversible" reversible="false" fast="false"
fbc:lowerFluxBound="irr_lb" fbc:upperFluxBound="irr_ub">
            <listOfReactants>
                  <speciesReference species="M_atp_c" stoichiometry="1"
constant="true"/>
                  <speciesReference species="M_citr__L_c" stoichiometry="1"
constant="true"/>
                  <speciesReference species="M_asp__L_c" stoichiometry="1"
constant="true"/>
            </listOfReactants>
            <listOfProducts>
                  <speciesReference species="M_ppi_c" stoichiometry="1"
constant="true"/>
                  <speciesReference species="M_argsuc_c" stoichiometry="1"
constant="true"/>
                  <speciesReference species="M_amp_c" stoichiometry="1"
constant="true"/>
            </listOfProducts>
            <fbc:geneProductAssociation>
                  <fbc:geneProductRef fbc:geneProduct="SAUSA300_0864"/>
            </fbc:geneProductAssociation>
      </reaction>

      <reaction metaid="R_ARGTRS" id="R_ARGTRS" name="Arginyl-tRNA
synthetase" reversible="false" fast="false" fbc:lowerFluxBound="irr_lb"
fbc:upperFluxBound="irr_ub">
            <listOfReactants>
                  <speciesReference species="M_atp_c" stoichiometry="1"
constant="true"/>
                  <speciesReference species="M_arg__L_c" stoichiometry="1"
constant="true"/>
                  <speciesReference species="M_trnaarg_c" stoichiometry="1"
constant="true"/>
            </listOfReactants>
            <listOfProducts>
```

```
                  <speciesReference species="M_ppi_c" stoichiometry="1"
constant="true"/>
                  <speciesReference species="M_amp_c" stoichiometry="1"
constant="true"/>
                  <speciesReference species="M_argtrna_c" stoichiometry="1"
constant="true"/>
            </listOfProducts>
            <fbc:geneProductAssociation>
                  <fbc:geneProductRef fbc:geneProduct="SAUSA300_0596"/>
            </fbc:geneProductAssociation>
      </reaction>

      <reaction metaid="R_ARSabc" id="R_ARSabc" name="arsenobetaine transport
in via ABC system " reversible="false" fast="false"
fbc:lowerFluxBound="irr_lb" fbc:upperFluxBound="irr_ub">
            <listOfReactants>
                  <speciesReference species="M_h2o_c" stoichiometry="1"
constant="true"/>
                  <speciesReference species="M_atp_c" stoichiometry="1"
constant="true"/>
                  <speciesReference species="M_arsbet_e" stoichiometry="1"
constant="true"/>
            </listOfReactants>
            <listOfProducts>
                  <speciesReference species="M_h_c" stoichiometry="1"
constant="true"/>
                  <speciesReference species="M_pi_c" stoichiometry="1"
constant="true"/>
                  <speciesReference species="M_adp_c" stoichiometry="1"
constant="true"/>
                  <speciesReference species="M_arsbet_c" stoichiometry="1"
constant="true"/>
            </listOfProducts>
            <fbc:geneProductAssociation>
                  <fbc:geneProductRef fbc:geneProduct="SAUSA300_0706"/>
            </fbc:geneProductAssociation>
      </reaction>

      <reaction metaid="R_ARStr" id="R_ARStr" name="arsenobetaine transport
in via proton symport " reversible="false" fast="false"
fbc:lowerFluxBound="irr_lb" fbc:upperFluxBound="irr_ub">
            <listOfReactants>
                  <speciesReference species="M_h_e" stoichiometry="1"
constant="true"/>
                  <speciesReference species="M_arsbet_e" stoichiometry="1"
constant="true"/>
            </listOfReactants>
            <listOfProducts>
                  <speciesReference species="M_h_c" stoichiometry="1"
constant="true"/>
                  <speciesReference species="M_arsbet_c" stoichiometry="1"
constant="true"/>
            </listOfProducts>
            <fbc:geneProductAssociation>
                  <fbc:or>
                        <fbc:geneProductRef fbc:geneProduct="SAUSA300_1245"/>
                        <fbc:geneProductRef fbc:geneProduct="SAUSA300_2145"/>
                  </fbc:or>
```

```
            </fbc:geneProductAssociation>
      </reaction>

      <reaction metaid="R_ASAD" id="R_ASAD" name="aspartate-semialdehyde
dehydrogenase" reversible="true" fast="false" fbc:lowerFluxBound="rev_lb"
fbc:upperFluxBound="rev_ub">
            <listOfReactants>
                  <speciesReference species="M_nadph_c" stoichiometry="1"
constant="true"/>
                  <speciesReference species="M_4pasp_c" stoichiometry="1"
constant="true"/>
            </listOfReactants>
            <listOfProducts>
                  <speciesReference species="M_nadp_c" stoichiometry="1"
constant="true"/>
                  <speciesReference species="M_pi_c" stoichiometry="1"
constant="true"/>
                  <speciesReference species="M_aspsa_c" stoichiometry="1"
constant="true"/>
            </listOfProducts>
            <fbc:geneProductAssociation>
                  <fbc:geneProductRef fbc:geneProduct="SAUSA300_1287"/>
            </fbc:geneProductAssociation>
      </reaction>

      <reaction metaid="R_ASNN" id="R_ASNN" name="L-asparaginase"
reversible="false" fast="false" fbc:lowerFluxBound="irr_lb"
fbc:upperFluxBound="irr_ub">
            <listOfReactants>
                  <speciesReference species="M_h2o_c" stoichiometry="1"
constant="true"/>
                  <speciesReference species="M_asn__L_c" stoichiometry="1"
constant="true"/>
            </listOfReactants>
            <listOfProducts>
                  <speciesReference species="M_nh4_c" stoichiometry="1"
constant="true"/>
                  <speciesReference species="M_asp__L_c" stoichiometry="1"
constant="true"/>
            </listOfProducts>
            <fbc:geneProductAssociation>
                  <fbc:geneProductRef fbc:geneProduct="SAUSA300_1368"/>
            </fbc:geneProductAssociation>
      </reaction>

      <reaction metaid="R_ASNS1" id="R_ASNS1" name="asparagine synthase
(glutamine-hydrolysing)" reversible="false" fast="false"
fbc:lowerFluxBound="irr_lb" fbc:upperFluxBound="irr_ub">
            <listOfReactants>
                  <speciesReference species="M_h2o_c" stoichiometry="1"
constant="true"/>
                  <speciesReference species="M_gln__L_c" stoichiometry="1"
constant="true"/>
                  <speciesReference species="M_atp_c" stoichiometry="1"
constant="true"/>
                  <speciesReference species="M_asp__L_c" stoichiometry="1"
constant="true"/>
            </listOfReactants>
```

```
            <listOfProducts>
                  <speciesReference species="M_h_c" stoichiometry="1"
constant="true"/>
                  <speciesReference species="M_glu__L_c" stoichiometry="1"
constant="true"/>
                  <speciesReference species="M_ppi_c" stoichiometry="1"
constant="true"/>
                  <speciesReference species="M_amp_c" stoichiometry="1"
constant="true"/>
                  <speciesReference species="M_asn__L_c" stoichiometry="1"
constant="true"/>
            </listOfProducts>
            <fbc:geneProductAssociation>
                  <fbc:geneProductRef fbc:geneProduct="SAUSA300_0972"/>
            </fbc:geneProductAssociation>
      </reaction>

      <reaction metaid="R_ASNt2r" id="R_ASNt2r" name="L-asparagine reversible
transport via proton symport" reversible="false" fast="false"
fbc:lowerFluxBound="irr_lb" fbc:upperFluxBound="irr_ub">
            <listOfReactants>
                  <speciesReference species="M_h_e" stoichiometry="1"
constant="true"/>
                  <speciesReference species="M_asn__L_e" stoichiometry="1"
constant="true"/>
            </listOfReactants>
            <listOfProducts>
                  <speciesReference species="M_h_c" stoichiometry="1"
constant="true"/>
                  <speciesReference species="M_asn__L_c" stoichiometry="1"
constant="true"/>
            </listOfProducts>
            <fbc:geneProductAssociation>
                  <fbc:geneProductRef fbc:geneProduct="SAUSA300_0712"/>
            </fbc:geneProductAssociation>
      </reaction>

      <reaction metaid="R_ASNTRS" id="R_ASNTRS" name="Asparaginyl-tRNA
synthetase" reversible="false" fast="false" fbc:lowerFluxBound="irr_lb"
fbc:upperFluxBound="irr_ub">
            <listOfReactants>
                  <speciesReference species="M_atp_c" stoichiometry="1"
constant="true"/>
                  <speciesReference species="M_asn__L_c" stoichiometry="1"
constant="true"/>
                  <speciesReference species="M_trnaasn_c" stoichiometry="1"
constant="true"/>
            </listOfReactants>
            <listOfProducts>
                  <speciesReference species="M_ppi_c" stoichiometry="1"
constant="true"/>
                  <speciesReference species="M_amp_c" stoichiometry="1"
constant="true"/>
                  <speciesReference species="M_asntrna_c" stoichiometry="1"
constant="true"/>
            </listOfProducts>
            <fbc:geneProductAssociation>
                  <fbc:geneProductRef fbc:geneProduct="SAUSA300_1345"/>
```

```
            </fbc:geneProductAssociation>
      </reaction>

      <reaction metaid="R_ASO3tex" id="R_ASO3tex" name="arsenite transport
via diffusion (extracellular to periplasm)" reversible="true" fast="false"
fbc:lowerFluxBound="rev_lb" fbc:upperFluxBound="rev_ub">
            <listOfReactants>
                  <speciesReference species="M_aso3_c" stoichiometry="1"
constant="true"/>
            </listOfReactants>
            <listOfProducts>
                  <speciesReference species="M_aso3_e" stoichiometry="1"
constant="true"/>
            </listOfProducts>
            <fbc:geneProductAssociation>
                  <fbc:geneProductRef fbc:geneProduct="SAUSA300_1718"/>
            </fbc:geneProductAssociation>
      </reaction>

      <reaction metaid="R_ASO4t" id="R_ASO4t" name="Arsenate transporter "
reversible="false" fast="false" fbc:lowerFluxBound="irr_lb"
fbc:upperFluxBound="irr_ub">
            <listOfReactants>
                  <speciesReference species="M_aso4_c" stoichiometry="1"
constant="true"/>
            </listOfReactants>
            <listOfProducts>
                  <speciesReference species="M_aso4_e" stoichiometry="1"
constant="true"/>
            </listOfProducts>
            <fbc:geneProductAssociation>
                  <fbc:geneProductRef fbc:geneProduct="SAUSA300_1718"/>
            </fbc:geneProductAssociation>
      </reaction>

      <reaction metaid="R_ASP1DC" id="R_ASP1DC" name="aspartate
1__Decarboxylase" reversible="false" fast="false" fbc:lowerFluxBound="irr_lb"
fbc:upperFluxBound="irr_ub">
            <listOfReactants>
                  <speciesReference species="M_h_c" stoichiometry="1"
constant="true"/>
                  <speciesReference species="M_asp__L_c" stoichiometry="1"
constant="true"/>
            </listOfReactants>
            <listOfProducts>
                  <speciesReference species="M_co2_c" stoichiometry="1"
constant="true"/>
                  <speciesReference species="M_ala__B_c" stoichiometry="1"
constant="true"/>
            </listOfProducts>
            <fbc:geneProductAssociation>
                  <fbc:geneProductRef fbc:geneProduct="SAUSA300_2532"/>
            </fbc:geneProductAssociation>
      </reaction>

      <reaction metaid="R_ASPabc" id="R_ASPabc" name="L-aspartate transport
via ABC system" reversible="false" fast="false" fbc:lowerFluxBound="irr_lb"
fbc:upperFluxBound="irr_ub">
```

```
            <listOfReactants>
                  <speciesReference species="M_h2o_c" stoichiometry="1"
constant="true"/>
                  <speciesReference species="M_atp_c" stoichiometry="1"
constant="true"/>
                  <speciesReference species="M_asp__L_e" stoichiometry="1"
constant="true"/>
            </listOfReactants>
            <listOfProducts>
                  <speciesReference species="M_h_c" stoichiometry="1"
constant="true"/>
                  <speciesReference species="M_pi_c" stoichiometry="1"
constant="true"/>
                  <speciesReference species="M_adp_c" stoichiometry="1"
constant="true"/>
                  <speciesReference species="M_asp__L_c" stoichiometry="1"
constant="true"/>
            </listOfProducts>
            <fbc:geneProductAssociation>
                  <fbc:or>
                        <fbc:and>
                              <fbc:geneProductRef
fbc:geneProduct="SAUSA300_1868"/>
                              <fbc:geneProductRef
fbc:geneProduct="SAUSA300_1869"/>
                        </fbc:and>
                        <fbc:and>
                              <fbc:geneProductRef
fbc:geneProduct="SAUSA300_1807"/>
                              <fbc:geneProductRef
fbc:geneProduct="SAUSA300_1808"/>
                        </fbc:and>
                  </fbc:or>
            </fbc:geneProductAssociation>
      </reaction>

      <reaction metaid="R_ASPCT" id="R_ASPCT" name="aspartate
carbamoyltransferase" reversible="false" fast="false"
fbc:lowerFluxBound="irr_lb" fbc:upperFluxBound="irr_ub">
            <listOfReactants>
                  <speciesReference species="M_cbp_c" stoichiometry="1"
constant="true"/>
                  <speciesReference species="M_asp__L_c" stoichiometry="1"
constant="true"/>
            </listOfReactants>
            <listOfProducts>
                  <speciesReference species="M_h_c" stoichiometry="2"
constant="true"/>
                  <speciesReference species="M_pi_c" stoichiometry="1"
constant="true"/>
                  <speciesReference species="M_cbasp_c" stoichiometry="1"
constant="true"/>
            </listOfProducts>
            <fbc:geneProductAssociation>
                  <fbc:geneProductRef fbc:geneProduct="SAUSA300_1093"/>
            </fbc:geneProductAssociation>
      </reaction>
```

```
      <reaction metaid="R_ASPKi" id="R_ASPKi" name="aspartate kinase,
irreversible" reversible="false" fast="false" fbc:lowerFluxBound="irr_lb"
fbc:upperFluxBound="irr_ub">
            <listOfReactants>
                  <speciesReference species="M_atp_c" stoichiometry="1"
constant="true"/>
                  <speciesReference species="M_asp__L_c" stoichiometry="1"
constant="true"/>
            </listOfReactants>
            <listOfProducts>
                  <speciesReference species="M_adp_c" stoichiometry="1"
constant="true"/>
                  <speciesReference species="M_4pasp_c" stoichiometry="1"
constant="true"/>
            </listOfProducts>
            <fbc:geneProductAssociation>
                  <fbc:or>
                        <fbc:geneProductRef fbc:geneProduct="SAUSA300_1225"/>
                        <fbc:geneProductRef fbc:geneProduct="SAUSA300_1286"/>
                  </fbc:or>
            </fbc:geneProductAssociation>
      </reaction>

      <reaction metaid="R_ASPT" id="R_ASPT" name="L-aspartase"
reversible="true" fast="false" fbc:lowerFluxBound="rev_lb"
fbc:upperFluxBound="rev_ub">
            <listOfReactants>
                  <speciesReference species="M_asp__L_c" stoichiometry="1"
constant="true"/>
            </listOfReactants>
            <listOfProducts>
                  <speciesReference species="M_fum_c" stoichiometry="1"
constant="true"/>
                  <speciesReference species="M_nh4_c" stoichiometry="1"
constant="true"/>
            </listOfProducts>
            <fbc:geneProductAssociation>
                  <fbc:geneProductRef fbc:geneProduct="aspA"/>
            </fbc:geneProductAssociation>
      </reaction>

      <reaction metaid="R_ASPTA" id="R_ASPTA" name="aspartate transaminase"
reversible="true" fast="false" fbc:lowerFluxBound="rev_lb"
fbc:upperFluxBound="rev_ub">
            <listOfReactants>
                  <speciesReference species="M_akg_c" stoichiometry="1"
constant="true"/>
                  <speciesReference species="M_asp__L_c" stoichiometry="1"
constant="true"/>
            </listOfReactants>
            <listOfProducts>
                  <speciesReference species="M_glu__L_c" stoichiometry="1"
constant="true"/>
                  <speciesReference species="M_oaa_c" stoichiometry="1"
constant="true"/>
            </listOfProducts>
            <fbc:geneProductAssociation>
                  <fbc:geneProductRef fbc:geneProduct="SAUSA300_1916"/>
```

```
            </fbc:geneProductAssociation>
      </reaction>

      <reaction metaid="R_ASPTRS" id="R_ASPTRS" name="Aspartyl-tRNA
synthetase" reversible="false" fast="false" fbc:lowerFluxBound="irr_lb"
fbc:upperFluxBound="irr_ub">
            <listOfReactants>
                  <speciesReference species="M_atp_c" stoichiometry="1"
constant="true"/>
                  <speciesReference species="M_asp__L_c" stoichiometry="1"
constant="true"/>
                  <speciesReference species="M_trnaasp_c" stoichiometry="1"
constant="true"/>
            </listOfReactants>
            <listOfProducts>
                  <speciesReference species="M_ppi_c" stoichiometry="1"
constant="true"/>
                  <speciesReference species="M_amp_c" stoichiometry="1"
constant="true"/>
                  <speciesReference species="M_asptrna_c" stoichiometry="1"
constant="true"/>
            </listOfProducts>
            <fbc:geneProductAssociation>
                  <fbc:geneProductRef fbc:geneProduct="SAUSA300_1586"/>
            </fbc:geneProductAssociation>
      </reaction>

      <reaction metaid="R_AT_MBD" id="R_AT_MBD" name="S-2-methylbutanoyl-CoA
enzyme N6__Dihydrolipoyllysine " reversible="true" fast="false"
fbc:lowerFluxBound="rev_lb" fbc:upperFluxBound="rev_ub">
            <listOfReactants>
                  <speciesReference species="M_2mbcoa_c" stoichiometry="1"
constant="true"/>
                  <speciesReference species="M_dhlam_c" stoichiometry="1"
constant="true"/>
            </listOfReactants>
            <listOfProducts>
                  <speciesReference species="M_coa_c" stoichiometry="1"
constant="true"/>
                  <speciesReference species="M_2mbdhl_c" stoichiometry="1"
constant="true"/>
            </listOfProducts>
            <fbc:geneProductAssociation>
                  <fbc:geneProductRef fbc:geneProduct="SAUSA300_1464"/>
            </fbc:geneProductAssociation>
      </reaction>

      <reaction metaid="R_AT_MBD1" id="R_AT_MBD1" name="3-methylbutanoyl-CoA
enzyme N6__Dihydrolipoyllysine " reversible="true" fast="false"
fbc:lowerFluxBound="rev_lb" fbc:upperFluxBound="rev_ub">
            <listOfReactants>
                  <speciesReference species="M_ivcoa_c" stoichiometry="1"
constant="true"/>
                  <speciesReference species="M_dhlam_c" stoichiometry="1"
constant="true"/>
            </listOfReactants>
            <listOfProducts>
```

```
                  <speciesReference species="M_coa_c" stoichiometry="1"
constant="true"/>
                  <speciesReference species="M_3mbdhl_c" stoichiometry="1"
constant="true"/>
            </listOfProducts>
            <fbc:geneProductAssociation>
                  <fbc:geneProductRef fbc:geneProduct="SAUSA300_1464"/>
            </fbc:geneProductAssociation>
      </reaction>

      <reaction metaid="R_AT_MBD2" id="R_AT_MBD2" name="2-methylpropanoyl-CoA
enzyme N6__Dihydrolipoyllysine " reversible="true" fast="false"
fbc:lowerFluxBound="rev_lb" fbc:upperFluxBound="rev_ub">
            <listOfReactants>
                  <speciesReference species="M_ibcoa_c" stoichiometry="1"
constant="true"/>
                  <speciesReference species="M_dhlam_c" stoichiometry="1"
constant="true"/>
            </listOfReactants>
            <listOfProducts>
                  <speciesReference species="M_coa_c" stoichiometry="1"
constant="true"/>
                  <speciesReference species="M_2mpdhl_c" stoichiometry="1"
constant="true"/>
            </listOfProducts>
            <fbc:geneProductAssociation>
                  <fbc:geneProductRef fbc:geneProduct="SAUSA300_1464"/>
            </fbc:geneProductAssociation>
      </reaction>

      <reaction metaid="R_ATPM" id="R_ATPM" name="ATP maintenance
requirement" reversible="false" fast="false" fbc:lowerFluxBound="irr_lb"
fbc:upperFluxBound="irr_ub">
            <listOfReactants>
                  <speciesReference species="M_h2o_c" stoichiometry="1"
constant="true"/>
                  <speciesReference species="M_atp_c" stoichiometry="1"
constant="true"/>
            </listOfReactants>
            <listOfProducts>
                  <speciesReference species="M_h_c" stoichiometry="1"
constant="true"/>
                  <speciesReference species="M_pi_c" stoichiometry="1"
constant="true"/>
                  <speciesReference species="M_adp_c" stoichiometry="1"
constant="true"/>
            </listOfProducts>
            <fbc:geneProductAssociation>
                  <fbc:geneProductRef fbc:geneProduct="SAUSA300_2494"/>
            </fbc:geneProductAssociation>
      </reaction>

      <reaction metaid="R_ATPP5P" id="R_ATPP5P" name="ATP pyridoxal 5'-
phosphotransferase " reversible="false" fast="false"
fbc:lowerFluxBound="irr_lb" fbc:upperFluxBound="irr_ub">
            <listOfReactants>
                  <speciesReference species="M_atp_c" stoichiometry="1"
constant="true"/>
```

```
                  <speciesReference species="M_pydxn_c" stoichiometry="1"
constant="true"/>
            </listOfReactants>
            <listOfProducts>
                  <speciesReference species="M_adp_c" stoichiometry="1"
constant="true"/>
                  <speciesReference species="M_pdx5p_c" stoichiometry="1"
constant="true"/>
            </listOfProducts>
            <fbc:geneProductAssociation>
                  <fbc:geneProductRef fbc:geneProduct="SAUSA300_0562"/>
            </fbc:geneProductAssociation>
      </reaction>

      <reaction metaid="R_ATPPRT" id="R_ATPPRT" name="ATP
phosphoribosyltransferase" reversible="false" fast="false"
fbc:lowerFluxBound="irr_lb" fbc:upperFluxBound="irr_ub">
            <listOfReactants>
                  <speciesReference species="M_atp_c" stoichiometry="1"
constant="true"/>
                  <speciesReference species="M_prpp_c" stoichiometry="1"
constant="true"/>
            </listOfReactants>
            <listOfProducts>
                  <speciesReference species="M_ppi_c" stoichiometry="1"
constant="true"/>
                  <speciesReference species="M_prbatp_c" stoichiometry="1"
constant="true"/>
            </listOfProducts>
            <fbc:geneProductAssociation>
                  <fbc:or>
                        <fbc:geneProductRef fbc:geneProduct="SAUSA300_2613"/>
                        <fbc:geneProductRef fbc:geneProduct="SAUSA300_2612"/>
                  </fbc:or>
            </fbc:geneProductAssociation>
      </reaction>

      <reaction metaid="R_ATPS24" id="R_ATPS24" name="ATPS24"
reversible="false" fast="false" fbc:lowerFluxBound="irr_lb"
fbc:upperFluxBound="irr_ub">
            <listOfReactants>
                  <speciesReference species="M_h_e" stoichiometry="2.4"
constant="true"/>
                  <speciesReference species="M_pi_c" stoichiometry="1"
constant="true"/>
                  <speciesReference species="M_adp_c" stoichiometry="1"
constant="true"/>
            </listOfReactants>
            <listOfProducts>
                  <speciesReference species="M_h_c" stoichiometry="2.4"
constant="true"/>
                  <speciesReference species="M_h2o_c" stoichiometry="1"
constant="true"/>
                  <speciesReference species="M_atp_c" stoichiometry="1"
constant="true"/>
            </listOfProducts>
            <fbc:geneProductAssociation>
                  <fbc:and>
```

```
                        <fbc:geneProductRef fbc:geneProduct="SAUSA300_2062"/>
                        <fbc:geneProductRef fbc:geneProduct="SAUSA300_2063"/>
                        <fbc:geneProductRef fbc:geneProduct="SAUSA300_2060"/>
                        <fbc:geneProductRef fbc:geneProduct="SAUSA300_2061"/>
                        <fbc:geneProductRef fbc:geneProduct="SA451515_2204"/>
                        <fbc:geneProductRef fbc:geneProduct="SAUSA300_2064"/>
                        <fbc:geneProductRef fbc:geneProduct="SAUSA300_2059"/>
                        <fbc:geneProductRef fbc:geneProduct="SAUSA300_2058"/>
                        <fbc:geneProductRef fbc:geneProduct="SAUSA300_2057"/>
                  </fbc:and>
            </fbc:geneProductAssociation>
      </reaction>

      <reaction metaid="R_ATPX5P" id="R_ATPX5P" name="ATP pyridoxal 5'-
phosphotransferase " reversible="false" fast="false"
fbc:lowerFluxBound="irr_lb" fbc:upperFluxBound="irr_ub">
            <listOfReactants>
                  <speciesReference species="M_atp_c" stoichiometry="1"
constant="true"/>
                  <speciesReference species="M_pydam_c" stoichiometry="1"
constant="true"/>
            </listOfReactants>
            <listOfProducts>
                  <speciesReference species="M_adp_c" stoichiometry="1"
constant="true"/>
                  <speciesReference species="M_pyam5p_c" stoichiometry="1"
constant="true"/>
            </listOfProducts>
            <fbc:geneProductAssociation>
                  <fbc:geneProductRef fbc:geneProduct="SAUSA300_0562"/>
            </fbc:geneProductAssociation>
      </reaction>

      <reaction metaid="R_BDOOx" id="R_BDOOx" name="beta__D-Glucose NAD 1-
oxoreductase " reversible="false" fast="false" fbc:lowerFluxBound="irr_lb"
fbc:upperFluxBound="irr_ub">
            <listOfReactants>
                  <speciesReference species="M_nad_c" stoichiometry="1"
constant="true"/>
                  <speciesReference species="M_glc__D__B_c" stoichiometry="1"
constant="true"/>
            </listOfReactants>
            <listOfProducts>
                  <speciesReference species="M_h_c" stoichiometry="1"
constant="true"/>
                  <speciesReference species="M_nadh_c" stoichiometry="1"
constant="true"/>
                  <speciesReference species="M_dg15l" stoichiometry="1"
constant="true"/>
            </listOfProducts>
            <fbc:geneProductAssociation>
                  <fbc:geneProductRef fbc:geneProduct="SAUSA300_2416"/>
            </fbc:geneProductAssociation>
      </reaction>

      <reaction metaid="R_BDOOy" id="R_BDOOy" name="beta__D-Glucose NADP 1-
oxoreductase " reversible="false" fast="false" fbc:lowerFluxBound="irr_lb"
fbc:upperFluxBound="irr_ub">
```

```
            <listOfReactants>
                  <speciesReference species="M_nadp_c" stoichiometry="1"
constant="true"/>
                  <speciesReference species="M_glc__D__B_c" stoichiometry="1"
constant="true"/>
            </listOfReactants>
            <listOfProducts>
                  <speciesReference species="M_h_c" stoichiometry="1"
constant="true"/>
                  <speciesReference species="M_nadph_c" stoichiometry="1"
constant="true"/>
                  <speciesReference species="M_dg15l" stoichiometry="1"
constant="true"/>
            </listOfProducts>
            <fbc:geneProductAssociation>
                  <fbc:geneProductRef fbc:geneProduct="SAUSA300_2416"/>
            </fbc:geneProductAssociation>
      </reaction>

      <reaction metaid="R_BETALDHx" id="R_BETALDHx" name="betaine-aldehyde
dehydrogenase" reversible="false" fast="false" fbc:lowerFluxBound="irr_lb"
fbc:upperFluxBound="irr_ub">
            <listOfReactants>
                  <speciesReference species="M_h2o_c" stoichiometry="1"
constant="true"/>
                  <speciesReference species="M_nad_c" stoichiometry="1"
constant="true"/>
                  <speciesReference species="M_betald_c" stoichiometry="1"
constant="true"/>
            </listOfReactants>
            <listOfProducts>
                  <speciesReference species="M_h_c" stoichiometry="2"
constant="true"/>
                  <speciesReference species="M_nadh_c" stoichiometry="1"
constant="true"/>
                  <speciesReference species="M_glyb_c" stoichiometry="1"
constant="true"/>
            </listOfProducts>
            <fbc:geneProductAssociation>
                  <fbc:geneProductRef fbc:geneProduct="SAUSA300_2546"/>
            </fbc:geneProductAssociation>
      </reaction>

      <reaction metaid="R_BETALDHy" id="R_BETALDHy" name="betaine-aldehyde
dehydrogenase" reversible="false" fast="false" fbc:lowerFluxBound="irr_lb"
fbc:upperFluxBound="irr_ub">
            <listOfReactants>
                  <speciesReference species="M_h2o_c" stoichiometry="1"
constant="true"/>
                  <speciesReference species="M_nadp_c" stoichiometry="1"
constant="true"/>
                  <speciesReference species="M_betald_c" stoichiometry="1"
constant="true"/>
            </listOfReactants>
            <listOfProducts>
                  <speciesReference species="M_h_c" stoichiometry="2"
constant="true"/>
```

```
                  <speciesReference species="M_nadph_c" stoichiometry="1"
constant="true"/>
                  <speciesReference species="M_glyb_c" stoichiometry="1"
constant="true"/>
            </listOfProducts>
            <fbc:geneProductAssociation>
                  <fbc:geneProductRef fbc:geneProduct="SAUSA300_2546"/>
            </fbc:geneProductAssociation>
      </reaction>

      <reaction metaid="R_BETALOD" id="R_BETALOD" name="Choline 1-
oxidoreductase " reversible="true" fast="false" fbc:lowerFluxBound="rev_lb"
fbc:upperFluxBound="rev_ub">
            <listOfReactants>
                  <speciesReference species="M_h2o_c" stoichiometry="1"
constant="true"/>
                  <speciesReference species="M_betald_c" stoichiometry="1"
constant="true"/>
                  <speciesReference species="M_pqq_c" stoichiometry="1"
constant="true"/>
            </listOfReactants>
            <listOfProducts>
                  <speciesReference species="M_h_c" stoichiometry="1"
constant="true"/>
                  <speciesReference species="M_glyb_c" stoichiometry="1"
constant="true"/>
                  <speciesReference species="M_pqqh2_c" stoichiometry="1"
constant="true"/>
            </listOfProducts>
            <fbc:geneProductAssociation>
                  <fbc:geneProductRef fbc:geneProduct="SAUSA300_2545"/>
            </fbc:geneProductAssociation>
      </reaction>

      <reaction metaid="R_BGLK" id="R_BGLK" name="beta-glucoside kinase"
reversible="true" fast="false" fbc:lowerFluxBound="rev_lb"
fbc:upperFluxBound="rev_ub">
            <listOfReactants>
                  <speciesReference species="M_h2o_c" stoichiometry="1"
constant="true"/>
                  <speciesReference species="M_cellb_c" stoichiometry="1"
constant="true"/>
            </listOfReactants>
            <listOfProducts>
                  <speciesReference species="M_g6p_c" stoichiometry="1"
constant="true"/>
                  <speciesReference species="M_glc__D_c" stoichiometry="1"
constant="true"/>
            </listOfProducts>
            <fbc:geneProductAssociation>
                  <fbc:geneProductRef fbc:geneProduct="SAUSA300_0260"/>
            </fbc:geneProductAssociation>
      </reaction>

      <reaction metaid="R_BPNT" id="R_BPNT" name="3',5'-bisphosphate
nucleotidase" reversible="false" fast="false" fbc:lowerFluxBound="irr_lb"
fbc:upperFluxBound="irr_ub">
            <listOfReactants>
```

```
                  <speciesReference species="M_h2o_c" stoichiometry="1"
constant="true"/>
                  <speciesReference species="M_pap_c" stoichiometry="1"
constant="true"/>
            </listOfReactants>
            <listOfProducts>
                  <speciesReference species="M_h_c" stoichiometry="1"
constant="true"/>
                  <speciesReference species="M_pi_c" stoichiometry="1"
constant="true"/>
                  <speciesReference species="M_amp_c" stoichiometry="1"
constant="true"/>
            </listOfProducts>
            <fbc:geneProductAssociation>
                  <fbc:geneProductRef fbc:geneProduct="SAUSA300_1650"/>
            </fbc:geneProductAssociation>
      </reaction>

      <reaction metaid="R_BTCCPL" id="R_BTCCPL" name="biotin-carboxyl-
carrier-protein carbon__Dioxide ligase ADP-forming " reversible="false"
fast="false" fbc:lowerFluxBound="irr_lb" fbc:upperFluxBound="irr_ub">
            <listOfReactants>
                  <speciesReference species="M_atp_c" stoichiometry="1"
constant="true"/>
                  <speciesReference species="M_hco3_c" stoichiometry="1"
constant="true"/>
                  <speciesReference species="M_hcarb_c" stoichiometry="1"
constant="true"/>
            </listOfReactants>
            <listOfProducts>
                  <speciesReference species="M_h_c" stoichiometry="1"
constant="true"/>
                  <speciesReference species="M_pi_c" stoichiometry="1"
constant="true"/>
                  <speciesReference species="M_adp_c" stoichiometry="1"
constant="true"/>
                  <speciesReference species="M_carP_c" stoichiometry="1"
constant="true"/>
            </listOfProducts>
            <fbc:geneProductAssociation>
                  <fbc:and>
                        <fbc:geneProductRef fbc:geneProduct="SAUSA300_1475"/>
                        <fbc:geneProductRef fbc:geneProduct="SAUSA300_1476"/>
                        <fbc:geneProductRef fbc:geneProduct="SAUSA300_1347"/>
                  </fbc:and>
            </fbc:geneProductAssociation>
      </reaction>

      <reaction metaid="R_BTD_RR_Et" id="R_BTD_RR_Et" name=" R R  2 3
Butanediol C4H10O2 transport " reversible="true" fast="false"
fbc:lowerFluxBound="rev_lb" fbc:upperFluxBound="rev_ub">
            <listOfReactants>
                  <speciesReference species="M_btd_RR_e" stoichiometry="1"
constant="true"/>
            </listOfReactants>
            <listOfProducts>
                  <speciesReference species="M_btd_RR_c" stoichiometry="1"
constant="true"/>
```

```
            </listOfProducts>
      </reaction>

      <reaction metaid="R_BTDD_RR" id="R_BTDD_RR" name=" R R  butanediol
dehydrogenase" reversible="true" fast="false" fbc:lowerFluxBound="rev_lb"
fbc:upperFluxBound="rev_ub">
            <listOfReactants>
                  <speciesReference species="M_actn__R_c" stoichiometry="1"
constant="true"/>
                  <speciesReference species="M_h_c" stoichiometry="1"
constant="true"/>
                  <speciesReference species="M_nadh_c" stoichiometry="1"
constant="true"/>
            </listOfReactants>
            <listOfProducts>
                  <speciesReference species="M_btd_RR_c" stoichiometry="1"
constant="true"/>
                  <speciesReference species="M_nad_c" stoichiometry="1"
constant="true"/>
            </listOfProducts>
            <fbc:geneProductAssociation>
                  <fbc:geneProductRef fbc:geneProduct="SAUSA300_0129"/>
            </fbc:geneProductAssociation>
      </reaction>

      <reaction metaid="R_BTGSD" id="R_BTGSD" name="beta-glucosidase methyl-
alpha__D-glucoside " reversible="true" fast="false"
fbc:lowerFluxBound="rev_lb" fbc:upperFluxBound="rev_ub">
            <listOfReactants>
                  <speciesReference species="M_h2o_c" stoichiometry="1"
constant="true"/>
                  <speciesReference species="M_amdglc_c" stoichiometry="1"
constant="true"/>
            </listOfReactants>
            <listOfProducts>
                  <speciesReference species="M_glc__D_c" stoichiometry="1"
constant="true"/>
                  <speciesReference species="M_meoh_c" stoichiometry="1"
constant="true"/>
            </listOfProducts>
            <fbc:geneProductAssociation>
                  <fbc:geneProductRef fbc:geneProduct="SAUSA300_0260"/>
            </fbc:geneProductAssociation>
      </reaction>

      <reaction metaid="R_BTNt" id="R_BTNt" name="Biotin ABC transporter "
reversible="false" fast="false" fbc:lowerFluxBound="irr_lb"
fbc:upperFluxBound="irr_ub">
            <listOfReactants>
                  <speciesReference species="M_h2o_c" stoichiometry="1"
constant="true"/>
                  <speciesReference species="M_atp_c" stoichiometry="1"
constant="true"/>
                  <speciesReference species="M_btn_e" stoichiometry="1"
constant="true"/>
            </listOfReactants>
            <listOfProducts>
```

```
                  <speciesReference species="M_h_c" stoichiometry="1"
constant="true"/>
                  <speciesReference species="M_pi_c" stoichiometry="1"
constant="true"/>
                  <speciesReference species="M_adp_c" stoichiometry="1"
constant="true"/>
                  <speciesReference species="M_btn_c" stoichiometry="1"
constant="true"/>
            </listOfProducts>
            <fbc:geneProductAssociation>
                  <fbc:geneProductRef fbc:geneProduct="SAUSA300_2233"/>
            </fbc:geneProductAssociation>
      </reaction>

      <reaction metaid="R_BTS3r" id="R_BTS3r" name="biotin synthase"
reversible="true" fast="false" fbc:lowerFluxBound="rev_lb"
fbc:upperFluxBound="rev_ub">
            <listOfReactants>
                  <speciesReference species="M_dtbt_c" stoichiometry="1"
constant="true"/>
                  <speciesReference species="M_s_c" stoichiometry="2"
constant="true"/>
            </listOfReactants>
            <listOfProducts>
                  <speciesReference species="M_h2s_c" stoichiometry="1"
constant="true"/>
                  <speciesReference species="M_btn_c" stoichiometry="1"
constant="true"/>
            </listOfProducts>
            <fbc:geneProductAssociation>
                  <fbc:geneProductRef fbc:geneProduct="SAUSA300_2371"/>
            </fbc:geneProductAssociation>
      </reaction>

      <reaction metaid="R_BTS4" id="R_BTS4" name="Biotin synthase"
reversible="false" fast="false" fbc:lowerFluxBound="irr_lb"
fbc:upperFluxBound="irr_ub">
            <listOfReactants>
                  <speciesReference species="M_amet_c" stoichiometry="1"
constant="true"/>
                  <speciesReference species="M_dtbt_c" stoichiometry="1"
constant="true"/>
                  <speciesReference species="M_s_c" stoichiometry="1"
constant="true"/>
            </listOfReactants>
            <listOfProducts>
                  <speciesReference species="M_h_c" stoichiometry="1"
constant="true"/>
                  <speciesReference species="M_met__L_c" stoichiometry="1"
constant="true"/>
                  <speciesReference species="M_btn_c" stoichiometry="1"
constant="true"/>
                  <speciesReference species="M_dad__5_c" stoichiometry="1"
constant="true"/>
            </listOfProducts>
            <fbc:geneProductAssociation>
                  <fbc:geneProductRef fbc:geneProduct="SAUSA300_2371"/>
            </fbc:geneProductAssociation>
```

```
      </reaction>

      <reaction metaid="R_CA2abc" id="R_CA2abc" name="calcium transport via
ABC system" reversible="false" fast="false" fbc:lowerFluxBound="irr_lb"
fbc:upperFluxBound="irr_ub">
            <listOfReactants>
                  <speciesReference species="M_h2o_c" stoichiometry="1"
constant="true"/>
                  <speciesReference species="M_atp_c" stoichiometry="1"
constant="true"/>
                  <speciesReference species="M_ca2_e" stoichiometry="1"
constant="true"/>
            </listOfReactants>
            <listOfProducts>
                  <speciesReference species="M_h_c" stoichiometry="1"
constant="true"/>
                  <speciesReference species="M_pi_c" stoichiometry="1"
constant="true"/>
                  <speciesReference species="M_adp_c" stoichiometry="1"
constant="true"/>
                  <speciesReference species="M_ca2_c" stoichiometry="1"
constant="true"/>
            </listOfProducts>
      </reaction>

      <reaction metaid="R_CAT" id="R_CAT" name="catalase" reversible="false"
fast="false" fbc:lowerFluxBound="irr_lb" fbc:upperFluxBound="irr_ub">
            <listOfReactants>
                  <speciesReference species="M_h2o2_c" stoichiometry="2"
constant="true"/>
            </listOfReactants>
            <listOfProducts>
                  <speciesReference species="M_h2o_c" stoichiometry="2"
constant="true"/>
                  <speciesReference species="M_o2_c" stoichiometry="1"
constant="true"/>
            </listOfProducts>
            <fbc:geneProductAssociation>
                  <fbc:geneProductRef fbc:geneProduct="SAUSA300_1232"/>
            </fbc:geneProductAssociation>
      </reaction>

      <reaction metaid="R_CBMK" id="R_CBMK" name="Carbamate kinase"
reversible="false" fast="false" fbc:lowerFluxBound="irr_lb"
fbc:upperFluxBound="irr_ub">
            <listOfReactants>
                  <speciesReference species="M_nh4_c" stoichiometry="1"
constant="true"/>
                  <speciesReference species="M_atp_c" stoichiometry="1"
constant="true"/>
                  <speciesReference species="M_co2_c" stoichiometry="1"
constant="true"/>
            </listOfReactants>
            <listOfProducts>
                  <speciesReference species="M_h_c" stoichiometry="1"
constant="true"/>
                  <speciesReference species="M_adp_c" stoichiometry="1"
constant="true"/>
```

```
                    <speciesReference species="M_cbp_c" stoichiometry="1"
constant="true"/>
              </listOfProducts>
              <fbc:geneProductAssociation>
                    <fbc:or>
                          <fbc:geneProductRef fbc:geneProduct="SAUSA300_0061"/>
                          <fbc:geneProductRef fbc:geneProduct="SAUSA300_1063"/>
                          <fbc:geneProductRef fbc:geneProduct="SAUSA300_2567"/>
                    </fbc:or>
              </fbc:geneProductAssociation>
        </reaction>

        <reaction metaid="R_CBPS" id="R_CBPS" name="carbamoyl-phosphate
synthase (glutamine-hydrolysing)" reversible="false" fast="false"
fbc:lowerFluxBound="irr_lb" fbc:upperFluxBound="irr_ub">
              <listOfReactants>
                    <speciesReference species="M_h2o_c" stoichiometry="1"
constant="true"/>
                    <speciesReference species="M_gln__L_c" stoichiometry="1"
constant="true"/>
                    <speciesReference species="M_atp_c" stoichiometry="2"
constant="true"/>
                    <speciesReference species="M_hco3_c" stoichiometry="1"
constant="true"/>
              </listOfReactants>
              <listOfProducts>
                    <speciesReference species="M_h_c" stoichiometry="1"
constant="true"/>
                    <speciesReference species="M_glu__L_c" stoichiometry="1"
constant="true"/>
                    <speciesReference species="M_pi_c" stoichiometry="1"
constant="true"/>
                    <speciesReference species="M_adp_c" stoichiometry="2"
constant="true"/>
                    <speciesReference species="M_cbp_c" stoichiometry="1"
constant="true"/>
              </listOfProducts>
              <fbc:geneProductAssociation>
                    <fbc:and>
                          <fbc:geneProductRef fbc:geneProduct="SAUSA300_1096"/>
                          <fbc:geneProductRef fbc:geneProduct="SAUSA300_1095"/>
                    </fbc:and>
              </fbc:geneProductAssociation>
        </reaction>

        <reaction metaid="R_CDDTPP" id="R_CDDTPP" name="dTTP cytidine 5'-
phosphotransferase " reversible="false" fast="false"
fbc:lowerFluxBound="irr_lb" fbc:upperFluxBound="irr_ub">
              <listOfReactants>
                    <speciesReference species="M_cytd_c" stoichiometry="1"
constant="true"/>
                    <speciesReference species="M_dttp_c" stoichiometry="1"
constant="true"/>
              </listOfReactants>
              <listOfProducts>
                    <speciesReference species="M_cmp_c" stoichiometry="1"
constant="true"/>
```

<speciesReference species="M_dtdp_c" stoichiometry="1" constant="true"/>
</listOfProducts>
<fbc:geneProductAssociation>
<fbc:geneProductRef fbc:geneProduct="SAUSA300_1568"/>
</fbc:geneProductAssociation>
</reaction>

<reaction metaid="R_CDPMEK" id="R_CDPMEK" name="4-(cytidine 5'__Diphospho)-2-C-methyl__D-erythritol kinase" reversible="false" fast="false" fbc:lowerFluxBound="irr_lb" fbc:upperFluxBound="irr_ub">
<listOfReactants>
<speciesReference species="M_atp_c" stoichiometry="1" constant="true"/>
<speciesReference species="M_4c2me_c" stoichiometry="1" constant="true"/>
</listOfReactants>
<listOfProducts>
<speciesReference species="M_adp_c" stoichiometry="1" constant="true"/>
<speciesReference species="M_2p4c2me_c" stoichiometry="1" constant="true"/>
</listOfProducts>
<fbc:geneProductAssociation>
<fbc:geneProductRef fbc:geneProduct="SAUSA300_0472"/>
</fbc:geneProductAssociation>
</reaction>

<reaction metaid="R_CELLB_Et" id="R_CELLB_Et" name="cellobiose transport " reversible="true" fast="false" fbc:lowerFluxBound="rev_lb" fbc:upperFluxBound="rev_ub">
<listOfReactants>
<speciesReference species="M_cellb_e" stoichiometry="1" constant="true"/>
</listOfReactants>
<listOfProducts>
<speciesReference species="M_cellb_c" stoichiometry="1" constant="true"/>
</listOfProducts>
</reaction>

<reaction metaid="R_CGLYabc" id="R_CGLYabc" name="Dipeptide transport via ABC system cgly " reversible="false" fast="false" fbc:lowerFluxBound="irr_lb" fbc:upperFluxBound="irr_ub">
<listOfReactants>
<speciesReference species="M_h2o_c" stoichiometry="1" constant="true"/>
<speciesReference species="M_cgly_e" stoichiometry="1" constant="true"/>
<speciesReference species="M_atp_c" stoichiometry="1" constant="true"/>
</listOfReactants>
<listOfProducts>
<speciesReference species="M_h_c" stoichiometry="1" constant="true"/>
<speciesReference species="M_pi_c" stoichiometry="1" constant="true"/>

```
                  <speciesReference species="M_adp_c" stoichiometry="1"
constant="true"/>
                  <speciesReference species="M_cgly_c" stoichiometry="1"
constant="true"/>
            </listOfProducts>
            <fbc:geneProductAssociation>
                  <fbc:or>
                        <fbc:geneProductRef fbc:geneProduct="SAUSA300_2411"/>
                        <fbc:geneProductRef fbc:geneProduct="SAUSA300_0200"/>
                        <fbc:geneProductRef fbc:geneProduct="SAUSA300_0889"/>
                        <fbc:geneProductRef fbc:geneProduct="SAUSA300_2410"/>
                        <fbc:geneProductRef fbc:geneProduct="SAUSA300_0890"/>
                        <fbc:geneProductRef fbc:geneProduct="SAUSA300_0888"/>
                        <fbc:geneProductRef fbc:geneProduct="SAUSA300_2409"/>
                        <fbc:geneProductRef fbc:geneProduct="SAUSA300_0887"/>
                        <fbc:geneProductRef fbc:geneProduct="SAUSA300_0893"/>
                        <fbc:geneProductRef fbc:geneProduct="SAUSA300_0895"/>
                        <fbc:geneProductRef fbc:geneProduct="SAUSA300_0891"/>
                        <fbc:geneProductRef fbc:geneProduct="SAUSA300_0896"/>
                  </fbc:or>
            </fbc:geneProductAssociation>
      </reaction>

      <reaction metaid="R_CHCOAL" id="R_CHCOAL" name="6-carboxyhexanoate-CoA
ligase" reversible="false" fast="false" fbc:lowerFluxBound="irr_lb"
fbc:upperFluxBound="irr_ub">
            <listOfReactants>
                  <speciesReference species="M_h_c" stoichiometry="1"
constant="true"/>
                  <speciesReference species="M_atp_c" stoichiometry="1"
constant="true"/>
                  <speciesReference species="M_coa_c" stoichiometry="1"
constant="true"/>
                  <speciesReference species="M_pime_c" stoichiometry="1"
constant="true"/>
            </listOfReactants>
            <listOfProducts>
                  <speciesReference species="M_ppi_c" stoichiometry="1"
constant="true"/>
                  <speciesReference species="M_amp_c" stoichiometry="1"
constant="true"/>
                  <speciesReference species="M_pmcoa_c" stoichiometry="1"
constant="true"/>
            </listOfProducts>
            <fbc:geneProductAssociation>
                  <fbc:geneProductRef fbc:geneProduct="SAUSA300_2369"/>
            </fbc:geneProductAssociation>
      </reaction>

      <reaction metaid="R_CHLabc" id="R_CHLabc" name="choline transport via
ABC system" reversible="false" fast="false" fbc:lowerFluxBound="irr_lb"
fbc:upperFluxBound="irr_ub">
            <listOfReactants>
                  <speciesReference species="M_h2o_c" stoichiometry="1"
constant="true"/>
                  <speciesReference species="M_atp_c" stoichiometry="1"
constant="true"/>
```

```
                    <speciesReference species="M_chol_e" stoichiometry="1"
constant="true"/>
            </listOfReactants>
            <listOfProducts>
                    <speciesReference species="M_h_c" stoichiometry="1"
constant="true"/>
                    <speciesReference species="M_pi_c" stoichiometry="1"
constant="true"/>
                    <speciesReference species="M_adp_c" stoichiometry="1"
constant="true"/>
                    <speciesReference species="M_chol_c" stoichiometry="1"
constant="true"/>
            </listOfProducts>
            <fbc:geneProductAssociation>
                    <fbc:and>
                            <fbc:geneProductRef fbc:geneProduct="SAUSA300_2390"/>
                            <fbc:geneProductRef fbc:geneProduct="SAUSA300_2391"/>
                            <fbc:geneProductRef fbc:geneProduct="SAUSA300_2392"/>
                            <fbc:geneProductRef fbc:geneProduct="SAUSA300_2393"/>
                    </fbc:and>
            </fbc:geneProductAssociation>
      </reaction>

      <reaction metaid="R_CHLt2r" id="R_CHLt2r" name="choline transport via
proton symport, reversible" reversible="false" fast="false"
fbc:lowerFluxBound="irr_lb" fbc:upperFluxBound="irr_ub">
            <listOfReactants>
                    <speciesReference species="M_h_e" stoichiometry="1"
constant="true"/>
                    <speciesReference species="M_chol_e" stoichiometry="1"
constant="true"/>
            </listOfReactants>
            <listOfProducts>
                    <speciesReference species="M_h_c" stoichiometry="1"
constant="true"/>
                    <speciesReference species="M_chol_c" stoichiometry="1"
constant="true"/>
            </listOfProducts>
            <fbc:geneProductAssociation>
                    <fbc:geneProductRef fbc:geneProduct="SAUSA300_2549"/>
            </fbc:geneProductAssociation>
      </reaction>

      <reaction metaid="R_CHOLD" id="R_CHOLD" name="choline dehydrogenase"
reversible="false" fast="false" fbc:lowerFluxBound="irr_lb"
fbc:upperFluxBound="irr_ub">
            <listOfReactants>
                    <speciesReference species="M_nad_c" stoichiometry="1"
constant="true"/>
                    <speciesReference species="M_chol_c" stoichiometry="1"
constant="true"/>
            </listOfReactants>
            <listOfProducts>
                    <speciesReference species="M_h_c" stoichiometry="1"
constant="true"/>
                    <speciesReference species="M_nadh_c" stoichiometry="1"
constant="true"/>
```

```
                  <speciesReference species="M_betald_c" stoichiometry="1"
constant="true"/>
            </listOfProducts>
            <fbc:geneProductAssociation>
                  <fbc:geneProductRef fbc:geneProduct="SAUSA300_2545"/>
            </fbc:geneProductAssociation>
      </reaction>

      <reaction metaid="R_CHOLOD" id="R_CHOLOD" name="Choline PQQ 1-
oxidoreductase " reversible="true" fast="false" fbc:lowerFluxBound="rev_lb"
fbc:upperFluxBound="rev_ub">
            <listOfReactants>
                  <speciesReference species="M_chol_c" stoichiometry="1"
constant="true"/>
                  <speciesReference species="M_pqq_c" stoichiometry="1"
constant="true"/>
            </listOfReactants>
            <listOfProducts>
                  <speciesReference species="M_betald_c" stoichiometry="1"
constant="true"/>
                  <speciesReference species="M_pqqh2_c" stoichiometry="1"
constant="true"/>
            </listOfProducts>
            <fbc:geneProductAssociation>
                  <fbc:geneProductRef fbc:geneProduct="SAUSA300_2545"/>
            </fbc:geneProductAssociation>
      </reaction>

      <reaction metaid="R_CHOLSabc" id="R_CHOLSabc" name="Choline sulfate ABC
transporter" reversible="false" fast="false" fbc:lowerFluxBound="irr_lb"
fbc:upperFluxBound="irr_ub">
            <listOfReactants>
                  <speciesReference species="M_h2o_c" stoichiometry="1"
constant="true"/>
                  <speciesReference species="M_atp_c" stoichiometry="1"
constant="true"/>
                  <speciesReference species="M_chols_e" stoichiometry="1"
constant="true"/>
            </listOfReactants>
            <listOfProducts>
                  <speciesReference species="M_h_c" stoichiometry="1"
constant="true"/>
                  <speciesReference species="M_pi_c" stoichiometry="1"
constant="true"/>
                  <speciesReference species="M_adp_c" stoichiometry="1"
constant="true"/>
                  <speciesReference species="M_chols_c" stoichiometry="1"
constant="true"/>
            </listOfProducts>
            <fbc:geneProductAssociation>
                  <fbc:and>
                        <fbc:geneProductRef fbc:geneProduct="SAUSA300_2390"/>
                        <fbc:geneProductRef fbc:geneProduct="SAUSA300_2391"/>
                        <fbc:geneProductRef fbc:geneProduct="SAUSA300_2392"/>
                        <fbc:geneProductRef fbc:geneProduct="SAUSA300_2393"/>
                  </fbc:and>
            </fbc:geneProductAssociation>
      </reaction>
```

```
      <reaction metaid="R_CHORM" id="R_CHORM" name="chorismate mutase"
reversible="false" fast="false" fbc:lowerFluxBound="irr_lb"
fbc:upperFluxBound="irr_ub">
            <listOfReactants>
                  <speciesReference species="M_chor_c" stoichiometry="1"
constant="true"/>
            </listOfReactants>
            <listOfProducts>
                  <speciesReference species="M_pphn_c" stoichiometry="1"
constant="true"/>
            </listOfProducts>
            <fbc:geneProductAssociation>
                  <fbc:geneProductRef fbc:geneProduct="SAUSA300_1683"/>
            </fbc:geneProductAssociation>
      </reaction>

      <reaction metaid="R_CHORS" id="R_CHORS" name="chorismate synthase"
reversible="false" fast="false" fbc:lowerFluxBound="irr_lb"
fbc:upperFluxBound="irr_ub">
            <listOfReactants>
                  <speciesReference species="M_3psme_c" stoichiometry="1"
constant="true"/>
            </listOfReactants>
            <listOfProducts>
                  <speciesReference species="M_h_c" stoichiometry="1"
constant="true"/>
                  <speciesReference species="M_pi_c" stoichiometry="1"
constant="true"/>
                  <speciesReference species="M_chor_c" stoichiometry="1"
constant="true"/>
            </listOfProducts>
            <fbc:geneProductAssociation>
                  <fbc:geneProductRef fbc:geneProduct="SAUSA300_1357"/>
            </fbc:geneProductAssociation>
      </reaction>

      <reaction metaid="R_CIT_Mgt" id="R_CIT_Mgt" name="citrate-Mg transport
in via proton symport" reversible="false" fast="false"
fbc:lowerFluxBound="irr_lb" fbc:upperFluxBound="irr_ub">
            <listOfReactants>
                  <speciesReference species="M_h_e" stoichiometry="1"
constant="true"/>
                  <speciesReference species="M_Cit__Mg_e" stoichiometry="1"
constant="true"/>
            </listOfReactants>
            <listOfProducts>
                  <speciesReference species="M_h_c" stoichiometry="1"
constant="true"/>
                  <speciesReference species="M_Cit__Mg_c" stoichiometry="1"
constant="true"/>
            </listOfProducts>
            <fbc:geneProductAssociation>
                  <fbc:geneProductRef fbc:geneProduct="SAUSA300_2552"/>
            </fbc:geneProductAssociation>
      </reaction>
```

```
      <reaction metaid="R_CITL" id="R_CITL" name="Citrate lyase"
reversible="false" fast="false" fbc:lowerFluxBound="irr_lb"
fbc:upperFluxBound="irr_ub">
            <listOfReactants>
                  <speciesReference species="M_cit_c" stoichiometry="1"
constant="true"/>
            </listOfReactants>
            <listOfProducts>
                  <speciesReference species="M_ac_c" stoichiometry="1"
constant="true"/>
                  <speciesReference species="M_oaa_c" stoichiometry="1"
constant="true"/>
            </listOfProducts>
            <fbc:geneProductAssociation>
                  <fbc:geneProductRef fbc:geneProduct="SAUSA300_1641"/>
            </fbc:geneProductAssociation>
      </reaction>

      <reaction metaid="R_CITR__L_Et" id="R_CITR__L_Et" name="L-Citrulline
transport (transport mechanism unknown - evidence from biolog data)"
reversible="true" fast="false" fbc:lowerFluxBound="rev_lb"
fbc:upperFluxBound="rev_ub">
            <listOfReactants>
                  <speciesReference species="M_citr__L_e" stoichiometry="1"
constant="true"/>
            </listOfReactants>
            <listOfProducts>
                  <speciesReference species="M_citr__L_c" stoichiometry="1"
constant="true"/>
            </listOfProducts>
      </reaction>

      <reaction metaid="R_CLPNS_SA" id="R_CLPNS_SA" name="Cardiolipin
Synthase (Saureus)" reversible="true" fast="false"
fbc:lowerFluxBound="rev_lb" fbc:upperFluxBound="rev_ub">
            <listOfReactants>
                  <speciesReference species="M_pg_SA_c" stoichiometry="0.04"
constant="true"/>
            </listOfReactants>
            <listOfProducts>
                  <speciesReference species="M_glyc_c" stoichiometry="1"
constant="true"/>
                  <speciesReference species="M_clpn_SA_c"
stoichiometry="0.02" constant="true"/>
            </listOfProducts>
            <fbc:geneProductAssociation>
                  <fbc:or>
                        <fbc:geneProductRef fbc:geneProduct="SAUSA300_2044"/>
                        <fbc:geneProductRef fbc:geneProduct="SAUSA300_1216"/>
                  </fbc:or>
            </fbc:geneProductAssociation>
      </reaction>

      <reaction metaid="R_CLPNS160" id="R_CLPNS160" name="cardiolipin
synthase (n-C16:0)" reversible="true" fast="false"
fbc:lowerFluxBound="rev_lb" fbc:upperFluxBound="rev_ub">
            <listOfReactants>
```

```
                  <speciesReference species="M_pg160_c" stoichiometry="2"
constant="true"/>
            </listOfReactants>
            <listOfProducts>
                  <speciesReference species="M_glyc_c" stoichiometry="1"
constant="true"/>
                  <speciesReference species="M_clpn160_c" stoichiometry="1"
constant="true"/>
            </listOfProducts>
            <fbc:geneProductAssociation>
                  <fbc:or>
                        <fbc:geneProductRef fbc:geneProduct="SAUSA300_2044"/>
                        <fbc:geneProductRef fbc:geneProduct="SAUSA300_1216"/>
                  </fbc:or>
            </fbc:geneProductAssociation>
      </reaction>

      <reaction metaid="R_CLtex" id="R_CLtex" name="chloride (Cl-1) transport
via diffusion (extracellular to periplasm)" reversible="true" fast="false"
fbc:lowerFluxBound="rev_lb" fbc:upperFluxBound="rev_ub">
            <listOfReactants>
                  <speciesReference species="M_cl_e" stoichiometry="1"
constant="true"/>
            </listOfReactants>
            <listOfProducts>
                  <speciesReference species="M_cl_c" stoichiometry="1"
constant="true"/>
            </listOfProducts>
      </reaction>

      <reaction metaid="R_CO23OC" id="R_CO23OC" name="Catechol oxygen 2 3-
oxidoreductasedecyclizing " reversible="false" fast="false"
fbc:lowerFluxBound="irr_lb" fbc:upperFluxBound="irr_ub">
            <listOfReactants>
                  <speciesReference species="M_o2_c" stoichiometry="1"
constant="true"/>
                  <speciesReference species="M_catechol_c" stoichiometry="1"
constant="true"/>
            </listOfReactants>
            <listOfProducts>
                  <speciesReference species="M_h_c" stoichiometry="1"
constant="true"/>
                  <speciesReference species="M_2hmcnsad_c" stoichiometry="1"
constant="true"/>
            </listOfProducts>
            <fbc:geneProductAssociation>
                  <fbc:geneProductRef fbc:geneProduct="SAUSA300_2461"/>
            </fbc:geneProductAssociation>
      </reaction>

      <reaction metaid="R_CO2t" id="R_CO2t" name="CO2 transporter via
diffusion" reversible="true" fast="false" fbc:lowerFluxBound="rev_lb"
fbc:upperFluxBound="rev_ub">
            <listOfReactants>
                  <speciesReference species="M_co2_e" stoichiometry="1"
constant="true"/>
            </listOfReactants>
            <listOfProducts>
```

```
                  <speciesReference species="M_co2_c" stoichiometry="1"
constant="true"/>
            </listOfProducts>
      </reaction>

      <reaction metaid="R_COBALT2tex" id="R_COBALT2tex" name="cobalt (Co+2)
transport" reversible="true" fast="false" fbc:lowerFluxBound="rev_lb"
fbc:upperFluxBound="rev_ub">
            <listOfReactants>
                  <speciesReference species="M_cobalt2_e" stoichiometry="1"
constant="true"/>
            </listOfReactants>
            <listOfProducts>
                  <speciesReference species="M_cobalt2_c" stoichiometry="1"
constant="true"/>
            </listOfProducts>
            <fbc:geneProductAssociation>
                  <fbc:or>
                        <fbc:geneProductRef fbc:geneProduct="SAUSA300_2293"/>
                        <fbc:geneProductRef fbc:geneProduct="SAUSA300_2323"/>
                        <fbc:geneProductRef fbc:geneProduct="SAUSA300_0977"/>
                  </fbc:or>
            </fbc:geneProductAssociation>
      </reaction>

      <reaction metaid="R_CPP3FC" id="R_CPP3FC" name="Coproporphyrin III
ferrochelatas" reversible="false" fast="false" fbc:lowerFluxBound="irr_lb"
fbc:upperFluxBound="irr_ub">
            <listOfReactants>
                  <speciesReference species="M_cpp3_c" stoichiometry="1"
constant="true"/>
                  <speciesReference species="M_fe2_c" stoichiometry="1"
constant="true"/>
            </listOfReactants>
            <listOfProducts>
                  <speciesReference species="M_fecpp3_c" stoichiometry="1"
constant="true"/>
                  <speciesReference species="M_h_c" stoichiometry="2.0"
constant="true"/>
            </listOfProducts>
            <fbc:geneProductAssociation>
                  <fbc:geneProductRef fbc:geneProduct="SAUSA300_1782"/>
            </fbc:geneProductAssociation>
      </reaction>

      <reaction metaid="R_CPP3O1" id="R_CPP3O1" name="Coproporphyrinogen III
oxidase" reversible="false" fast="false" fbc:lowerFluxBound="irr_lb"
fbc:upperFluxBound="irr_ub">
            <listOfReactants>
                  <speciesReference species="M_cpppg3_c" stoichiometry="1"
constant="true"/>
                  <speciesReference species="M_fad_c" stoichiometry="1"
constant="true"/>
            </listOfReactants>
            <listOfProducts>
                  <speciesReference species="M_cpp3_c" stoichiometry="1"
constant="true"/>
```

```
                  <speciesReference species="M_fadh2_c" stoichiometry="1"
constant="true"/>
            </listOfProducts>
            <fbc:geneProductAssociation>
                  <fbc:geneProductRef fbc:geneProduct="SAUSA300_1781"/>
            </fbc:geneProductAssociation>
      </reaction>

      <reaction metaid="R_CPPPGO" id="R_CPPPGO" name="coproporphyrinogen
oxidase (O2 required)" reversible="false" fast="false"
fbc:lowerFluxBound="irr_lb" fbc:upperFluxBound="irr_ub">
            <listOfReactants>
                  <speciesReference species="M_h_c" stoichiometry="2"
constant="true"/>
                  <speciesReference species="M_o2_c" stoichiometry="1"
constant="true"/>
                  <speciesReference species="M_cpppg3_c" stoichiometry="1"
constant="true"/>
            </listOfReactants>
            <listOfProducts>
                  <speciesReference species="M_h2o_c" stoichiometry="2"
constant="true"/>
                  <speciesReference species="M_co2_c" stoichiometry="2"
constant="true"/>
                  <speciesReference species="M_pppg9_c" stoichiometry="1"
constant="true"/>
            </listOfProducts>
            <fbc:geneProductAssociation>
                  <fbc:geneProductRef fbc:geneProduct="SAUSA300_1543"/>
            </fbc:geneProductAssociation>
      </reaction>

      <reaction metaid="R_CPPPGO2" id="R_CPPPGO2" name="Oxygen Independent
coproporphyrinogen-III oxidase" reversible="true" fast="false"
fbc:lowerFluxBound="rev_lb" fbc:upperFluxBound="rev_ub">
            <listOfReactants>
                  <speciesReference species="M_amet_c" stoichiometry="2.0"
constant="true"/>
                  <speciesReference species="M_cpppg3_c" stoichiometry="1"
constant="true"/>
            </listOfReactants>
            <listOfProducts>
                  <speciesReference species="M_co2_c" stoichiometry="2.0"
constant="true"/>
                  <speciesReference species="M_dad__5_c" stoichiometry="2.0"
constant="true"/>
                  <speciesReference species="M_met__L_c" stoichiometry="2.0"
constant="true"/>
                  <speciesReference species="M_pppg9_c" stoichiometry="1"
constant="true"/>
            </listOfProducts>
            <fbc:geneProductAssociation>
                  <fbc:geneProductRef fbc:geneProduct="SAUSA300_1543"/>
            </fbc:geneProductAssociation>
      </reaction>
```

```
      <reaction metaid="R_CRNabc" id="R_CRNabc" name="L-carnitine transport
via ABC system" reversible="false" fast="false" fbc:lowerFluxBound="irr_lb"
fbc:upperFluxBound="irr_ub">
            <listOfReactants>
                  <speciesReference species="M_h2o_c" stoichiometry="1"
constant="true"/>
                  <speciesReference species="M_atp_c" stoichiometry="1"
constant="true"/>
                  <speciesReference species="M_crn_e" stoichiometry="1"
constant="true"/>
            </listOfReactants>
            <listOfProducts>
                  <speciesReference species="M_h_c" stoichiometry="1"
constant="true"/>
                  <speciesReference species="M_pi_c" stoichiometry="1"
constant="true"/>
                  <speciesReference species="M_adp_c" stoichiometry="1"
constant="true"/>
                  <speciesReference species="M_crn_c" stoichiometry="1"
constant="true"/>
            </listOfProducts>
            <fbc:geneProductAssociation>
                  <fbc:and>
                        <fbc:geneProductRef fbc:geneProduct="SAUSA300_2390"/>
                        <fbc:geneProductRef fbc:geneProduct="SAUSA300_2391"/>
                        <fbc:geneProductRef fbc:geneProduct="SAUSA300_2392"/>
                        <fbc:geneProductRef fbc:geneProduct="SAUSA300_2393"/>
                  </fbc:and>
            </fbc:geneProductAssociation>
      </reaction>

      <reaction metaid="R_CS" id="R_CS" name="citrate synthase"
reversible="false" fast="false" fbc:lowerFluxBound="irr_lb"
fbc:upperFluxBound="irr_ub">
            <listOfReactants>
                  <speciesReference species="M_h2o_c" stoichiometry="1"
constant="true"/>
                  <speciesReference species="M_accoa_c" stoichiometry="1"
constant="true"/>
                  <speciesReference species="M_oaa_c" stoichiometry="1"
constant="true"/>
            </listOfReactants>
            <listOfProducts>
                  <speciesReference species="M_h_c" stoichiometry="1"
constant="true"/>
                  <speciesReference species="M_coa_c" stoichiometry="1"
constant="true"/>
                  <speciesReference species="M_cit_c" stoichiometry="1"
constant="true"/>
            </listOfProducts>
            <fbc:geneProductAssociation>
                  <fbc:geneProductRef fbc:geneProduct="SAUSA300_1641"/>
            </fbc:geneProductAssociation>
      </reaction>

      <reaction metaid="R_CTBTabc" id="R_CTBTabc" name="Crotono-betaine ABC
transport " reversible="false" fast="false" fbc:lowerFluxBound="irr_lb"
fbc:upperFluxBound="irr_ub">
```

```
            <listOfReactants>
                  <speciesReference species="M_h2o_c" stoichiometry="1"
constant="true"/>
                  <speciesReference species="M_atp_c" stoichiometry="1"
constant="true"/>
                  <speciesReference species="M_ctbt_e" stoichiometry="1"
constant="true"/>
            </listOfReactants>
            <listOfProducts>
                  <speciesReference species="M_h_c" stoichiometry="1"
constant="true"/>
                  <speciesReference species="M_pi_c" stoichiometry="1"
constant="true"/>
                  <speciesReference species="M_adp_c" stoichiometry="1"
constant="true"/>
                  <speciesReference species="M_ctbt_c" stoichiometry="1"
constant="true"/>
            </listOfProducts>
            <fbc:geneProductAssociation>
                  <fbc:geneProductRef fbc:geneProduct="SAUSA300_0706"/>
            </fbc:geneProductAssociation>
      </reaction>

      <reaction metaid="R_CTPS1" id="R_CTPS1" name="CTP synthase (NH3)"
reversible="false" fast="false" fbc:lowerFluxBound="irr_lb"
fbc:upperFluxBound="irr_ub">
            <listOfReactants>
                  <speciesReference species="M_nh4_c" stoichiometry="1"
constant="true"/>
                  <speciesReference species="M_atp_c" stoichiometry="1"
constant="true"/>
                  <speciesReference species="M_utp_c" stoichiometry="1"
constant="true"/>
            </listOfReactants>
            <listOfProducts>
                  <speciesReference species="M_h_c" stoichiometry="2"
constant="true"/>
                  <speciesReference species="M_pi_c" stoichiometry="1"
constant="true"/>
                  <speciesReference species="M_adp_c" stoichiometry="1"
constant="true"/>
                  <speciesReference species="M_ctp_c" stoichiometry="1"
constant="true"/>
            </listOfProducts>
            <fbc:geneProductAssociation>
                  <fbc:geneProductRef fbc:geneProduct="SAUSA300_2081"/>
            </fbc:geneProductAssociation>
      </reaction>

      <reaction metaid="R_CTPS2" id="R_CTPS2" name="CTP synthase (glutamine)"
reversible="false" fast="false" fbc:lowerFluxBound="irr_lb"
fbc:upperFluxBound="irr_ub">
            <listOfReactants>
                  <speciesReference species="M_h2o_c" stoichiometry="1"
constant="true"/>
                  <speciesReference species="M_gln__L_c" stoichiometry="1"
constant="true"/>
```

```
                  <speciesReference species="M_atp_c" stoichiometry="1"
constant="true"/>
                  <speciesReference species="M_utp_c" stoichiometry="1"
constant="true"/>
            </listOfReactants>
            <listOfProducts>
                  <speciesReference species="M_h_c" stoichiometry="2"
constant="true"/>
                  <speciesReference species="M_glu__L_c" stoichiometry="1"
constant="true"/>
                  <speciesReference species="M_pi_c" stoichiometry="1"
constant="true"/>
                  <speciesReference species="M_adp_c" stoichiometry="1"
constant="true"/>
                  <speciesReference species="M_ctp_c" stoichiometry="1"
constant="true"/>
            </listOfProducts>
            <fbc:geneProductAssociation>
                  <fbc:geneProductRef fbc:geneProduct="SAUSA300_2081"/>
            </fbc:geneProductAssociation>
      </reaction>

      <reaction metaid="R_Cut1" id="R_Cut1" name="Copper Exchange via ATPase"
reversible="false" fast="false" fbc:lowerFluxBound="irr_lb"
fbc:upperFluxBound="irr_ub">
            <listOfReactants>
                  <speciesReference species="M_h2o_c" stoichiometry="1"
constant="true"/>
                  <speciesReference species="M_atp_c" stoichiometry="1"
constant="true"/>
                  <speciesReference species="M_cu2_c" stoichiometry="1"
constant="true"/>
            </listOfReactants>
            <listOfProducts>
                  <speciesReference species="M_h_c" stoichiometry="1"
constant="true"/>
                  <speciesReference species="M_pi_c" stoichiometry="1"
constant="true"/>
                  <speciesReference species="M_adp_c" stoichiometry="1"
constant="true"/>
                  <speciesReference species="M_cu2_e" stoichiometry="1"
constant="true"/>
            </listOfProducts>
            <fbc:geneProductAssociation>
                  <fbc:and>
                        <fbc:geneProductRef fbc:geneProduct="SAUSA300_2494"/>
                        <fbc:geneProductRef fbc:geneProduct="SAUSA300_2495"/>
                        <fbc:geneProductRef fbc:geneProduct="SAUSA300_0078"/>
                  </fbc:and>
            </fbc:geneProductAssociation>
      </reaction>

      <reaction metaid="R_CYSabc" id="R_CYSabc" name="L-cysteine transport
via ABC system" reversible="false" fast="false" fbc:lowerFluxBound="irr_lb"
fbc:upperFluxBound="irr_ub">
            <listOfReactants>
                  <speciesReference species="M_h2o_c" stoichiometry="1"
constant="true"/>
```

```
                  <speciesReference species="M_atp_c" stoichiometry="1"
constant="true"/>
                  <speciesReference species="M_cys__L_e" stoichiometry="1"
constant="true"/>
            </listOfReactants>
            <listOfProducts>
                  <speciesReference species="M_h_c" stoichiometry="1"
constant="true"/>
                  <speciesReference species="M_pi_c" stoichiometry="1"
constant="true"/>
                  <speciesReference species="M_adp_c" stoichiometry="1"
constant="true"/>
                  <speciesReference species="M_cys__L_c" stoichiometry="1"
constant="true"/>
            </listOfProducts>
            <fbc:geneProductAssociation>
                  <fbc:and>
                        <fbc:geneProductRef fbc:geneProduct="SAUSA300_2359"/>
                        <fbc:geneProductRef fbc:geneProduct="SAUSA300_2358"/>
                        <fbc:geneProductRef fbc:geneProduct="SAUSA300_2357"/>
                  </fbc:and>
            </fbc:geneProductAssociation>
      </reaction>

      <reaction metaid="R_CYSDS" id="R_CYSDS" name="Cysteine Desulfhydrase"
reversible="false" fast="false" fbc:lowerFluxBound="irr_lb"
fbc:upperFluxBound="irr_ub">
            <listOfReactants>
                  <speciesReference species="M_h2o_c" stoichiometry="1"
constant="true"/>
                  <speciesReference species="M_cys__L_c" stoichiometry="1"
constant="true"/>
            </listOfReactants>
            <listOfProducts>
                  <speciesReference species="M_nh4_c" stoichiometry="1"
constant="true"/>
                  <speciesReference species="M_pyr_c" stoichiometry="1"
constant="true"/>
                  <speciesReference species="M_h2s_c" stoichiometry="1"
constant="true"/>
            </listOfProducts>
            <fbc:geneProductAssociation>
                  <fbc:or>
                        <fbc:geneProductRef fbc:geneProduct="SAUSA300_0434"/>
                        <fbc:geneProductRef fbc:geneProduct="SAUSA300_0359"/>
                        <fbc:geneProductRef fbc:geneProduct="SAUSA300_0820"/>
                  </fbc:or>
            </fbc:geneProductAssociation>
      </reaction>

      <reaction metaid="R_CYSGL" id="R_CYSGL" name="Cystathionine
gamma__Lyase" reversible="false" fast="false" fbc:lowerFluxBound="irr_lb"
fbc:upperFluxBound="irr_ub">
            <listOfReactants>
                  <speciesReference species="M_acser_c" stoichiometry="1"
constant="true"/>
                  <speciesReference species="M_hcys__L_c" stoichiometry="1"
constant="true"/>
```

```
            </listOfReactants>
            <listOfProducts>
                  <speciesReference species="M_ac_c" stoichiometry="1"
constant="true"/>
                  <speciesReference species="M_cyst__L_c" stoichiometry="1"
constant="true"/>
                  <speciesReference species="M_h_c" stoichiometry="1"
constant="true"/>
            </listOfProducts>
            <fbc:geneProductAssociation>
                  <fbc:and>
                        <fbc:geneProductRef fbc:geneProduct="SAUSA300_0433"/>
                        <fbc:geneProductRef fbc:geneProduct="SAUSA300_0434"/>
                  </fbc:and>
            </fbc:geneProductAssociation>
      </reaction>

      <reaction metaid="R_CYSS" id="R_CYSS" name="cysteine synthase"
reversible="false" fast="false" fbc:lowerFluxBound="irr_lb"
fbc:upperFluxBound="irr_ub">
            <listOfReactants>
                  <speciesReference species="M_h2s_c" stoichiometry="1"
constant="true"/>
                  <speciesReference species="M_acser_c" stoichiometry="1"
constant="true"/>
            </listOfReactants>
            <listOfProducts>
                  <speciesReference species="M_h_c" stoichiometry="1"
constant="true"/>
                  <speciesReference species="M_ac_c" stoichiometry="1"
constant="true"/>
                  <speciesReference species="M_cys__L_c" stoichiometry="1"
constant="true"/>
            </listOfProducts>
            <fbc:geneProductAssociation>
                  <fbc:geneProductRef fbc:geneProduct="SAUSA300_0491"/>
            </fbc:geneProductAssociation>
      </reaction>

      <reaction metaid="R_CYSTGL" id="R_CYSTGL" name="cystathionine g__Lyase"
reversible="false" fast="false" fbc:lowerFluxBound="irr_lb"
fbc:upperFluxBound="irr_ub">
            <listOfReactants>
                  <speciesReference species="M_h2o_c" stoichiometry="1"
constant="true"/>
                  <speciesReference species="M_cyst__L_c" stoichiometry="1"
constant="true"/>
            </listOfReactants>
            <listOfProducts>
                  <speciesReference species="M_nh4_c" stoichiometry="1"
constant="true"/>
                  <speciesReference species="M_2obut_c" stoichiometry="1"
constant="true"/>
                  <speciesReference species="M_cys__L_c" stoichiometry="1"
constant="true"/>
            </listOfProducts>
            <fbc:geneProductAssociation>
                  <fbc:geneProductRef fbc:geneProduct="SAUSA300_0434"/>
```

```
			</fbc:geneProductAssociation>
		</reaction>

		<reaction metaid="R_CYSTL" id="R_CYSTL" name="cystathionine b__Lyase"
reversible="false" fast="false" fbc:lowerFluxBound="irr_lb"
fbc:upperFluxBound="irr_ub">
			<listOfReactants>
				<speciesReference species="M_h2o_c" stoichiometry="1"
constant="true"/>
				<speciesReference species="M_cyst__L_c" stoichiometry="1"
constant="true"/>
			</listOfReactants>
			<listOfProducts>
				<speciesReference species="M_nh4_c" stoichiometry="1"
constant="true"/>
				<speciesReference species="M_pyr_c" stoichiometry="1"
constant="true"/>
				<speciesReference species="M_hcys__L_c" stoichiometry="1"
constant="true"/>
			</listOfProducts>
			<fbc:geneProductAssociation>
				<fbc:geneProductRef fbc:geneProduct="SAUSA300_0359"/>
			</fbc:geneProductAssociation>
		</reaction>

		<reaction metaid="R_CYSTRS" id="R_CYSTRS" name="Cysteinyl-tRNA
synthetase" reversible="false" fast="false" fbc:lowerFluxBound="irr_lb"
fbc:upperFluxBound="irr_ub">
			<listOfReactants>
				<speciesReference species="M_atp_c" stoichiometry="1"
constant="true"/>
				<speciesReference species="M_cys__L_c" stoichiometry="1"
constant="true"/>
				<speciesReference species="M_trnacys_c" stoichiometry="1"
constant="true"/>
			</listOfReactants>
			<listOfProducts>
				<speciesReference species="M_ppi_c" stoichiometry="1"
constant="true"/>
				<speciesReference species="M_amp_c" stoichiometry="1"
constant="true"/>
				<speciesReference species="M_cystrna_c" stoichiometry="1"
constant="true"/>
			</listOfProducts>
			<fbc:geneProductAssociation>
				<fbc:geneProductRef fbc:geneProduct="SAUSA300_0515"/>
			</fbc:geneProductAssociation>
		</reaction>

		<reaction metaid="R_CYSTSr" id="R_CYSTSr" name="L-Serine hydro__Lyase
adding homocysteine " reversible="true" fast="false"
fbc:lowerFluxBound="rev_lb" fbc:upperFluxBound="rev_ub">
			<listOfReactants>
				<speciesReference species="M_hcys__L_c" stoichiometry="1"
constant="true"/>
				<speciesReference species="M_ser__L_c" stoichiometry="1"
constant="true"/>
			</listOfReactants>
```

```
            <listOfProducts>
                  <speciesReference species="M_h2o_c" stoichiometry="1"
constant="true"/>
                  <speciesReference species="M_cyst__L_c" stoichiometry="1"
constant="true"/>
            </listOfProducts>
            <fbc:geneProductAssociation>
                  <fbc:geneProductRef fbc:geneProduct="SAUSA300_0433"/>
            </fbc:geneProductAssociation>
      </reaction>

      <reaction metaid="R_CYTBD2" id="R_CYTBD2" name="cytochrome oxidase bd
(menaquinol-8: 2 protons)" reversible="false" fast="false"
fbc:lowerFluxBound="irr_lb" fbc:upperFluxBound="irr_ub">
            <listOfReactants>
                  <speciesReference species="M_h_c" stoichiometry="2"
constant="true"/>
                  <speciesReference species="M_o2_c" stoichiometry="0.5"
constant="true"/>
                  <speciesReference species="M_mql8_c" stoichiometry="1"
constant="true"/>
            </listOfReactants>
            <listOfProducts>
                  <speciesReference species="M_h_e" stoichiometry="2"
constant="true"/>
                  <speciesReference species="M_h2o_c" stoichiometry="1"
constant="true"/>
                  <speciesReference species="M_mqn8_c" stoichiometry="1"
constant="true"/>
            </listOfProducts>
            <fbc:geneProductAssociation>
                  <fbc:or>
                        <fbc:geneProductRef fbc:geneProduct="SAUSA300_0987"/>
                        <fbc:geneProductRef fbc:geneProduct="SAUSA300_0986"/>
                        <fbc:geneProductRef fbc:geneProduct="SAUSA300_1046"/>
                  </fbc:or>
            </fbc:geneProductAssociation>
      </reaction>

      <reaction metaid="R_CYTD" id="R_CYTD" name="cytidine deaminase"
reversible="false" fast="false" fbc:lowerFluxBound="irr_lb"
fbc:upperFluxBound="irr_ub">
            <listOfReactants>
                  <speciesReference species="M_h_c" stoichiometry="1"
constant="true"/>
                  <speciesReference species="M_h2o_c" stoichiometry="1"
constant="true"/>
                  <speciesReference species="M_cytd_c" stoichiometry="1"
constant="true"/>
            </listOfReactants>
            <listOfProducts>
                  <speciesReference species="M_nh4_c" stoichiometry="1"
constant="true"/>
                  <speciesReference species="M_uri_c" stoichiometry="1"
constant="true"/>
            </listOfProducts>
            <fbc:geneProductAssociation>
                  <fbc:geneProductRef fbc:geneProduct="SAUSA300_1528"/>
```

```
            </fbc:geneProductAssociation>
      </reaction>

      <reaction metaid="R_CYTDK1" id="R_CYTDK1" name="cytidine kinase (ATP)"
reversible="false" fast="false" fbc:lowerFluxBound="irr_lb"
fbc:upperFluxBound="irr_ub">
            <listOfReactants>
                  <speciesReference species="M_atp_c" stoichiometry="1"
constant="true"/>
                  <speciesReference species="M_cytd_c" stoichiometry="1"
constant="true"/>
            </listOfReactants>
            <listOfProducts>
                  <speciesReference species="M_adp_c" stoichiometry="1"
constant="true"/>
                  <speciesReference species="M_cmp_c" stoichiometry="1"
constant="true"/>
            </listOfProducts>
            <fbc:geneProductAssociation>
                  <fbc:geneProductRef fbc:geneProduct="SAUSA300_1568"/>
            </fbc:geneProductAssociation>
      </reaction>

      <reaction metaid="R_CYTDt2r" id="R_CYTDt2r" name="cytidine transport in
via proton symport, reversible" reversible="false" fast="false"
fbc:lowerFluxBound="irr_lb" fbc:upperFluxBound="irr_ub">
            <listOfReactants>
                  <speciesReference species="M_h_e" stoichiometry="1"
constant="true"/>
                  <speciesReference species="M_cytd_e" stoichiometry="1"
constant="true"/>
            </listOfReactants>
            <listOfProducts>
                  <speciesReference species="M_h_c" stoichiometry="1"
constant="true"/>
                  <speciesReference species="M_cytd_c" stoichiometry="1"
constant="true"/>
            </listOfProducts>
            <fbc:geneProductAssociation>
                  <fbc:geneProductRef fbc:geneProduct="SAUSA300_0506"/>
            </fbc:geneProductAssociation>
      </reaction>

      <reaction metaid="R_CYTDt4" id="R_CYTDt4" name="cytidine transport in
via sodium symport" reversible="false" fast="false"
fbc:lowerFluxBound="irr_lb" fbc:upperFluxBound="irr_ub">
            <listOfReactants>
                  <speciesReference species="M_na1_e" stoichiometry="1"
constant="true"/>
                  <speciesReference species="M_cytd_e" stoichiometry="1"
constant="true"/>
            </listOfReactants>
            <listOfProducts>
                  <speciesReference species="M_cytd_c" stoichiometry="1"
constant="true"/>
                  <speciesReference species="M_na1_c" stoichiometry="1"
constant="true"/>
            </listOfProducts>
```

```
            <fbc:geneProductAssociation>
                  <fbc:or>
                        <fbc:geneProductRef fbc:geneProduct="SAUSA300_0313"/>
                        <fbc:geneProductRef fbc:geneProduct="SAUSA300_0631"/>
                        <fbc:geneProductRef fbc:geneProduct="SAUSA300_0506"/>
                  </fbc:or>
            </fbc:geneProductAssociation>
      </reaction>

      <reaction metaid="R_CYTK1" id="R_CYTK1" name="cytidylate kinase (CMP)"
reversible="true" fast="false" fbc:lowerFluxBound="rev_lb"
fbc:upperFluxBound="rev_ub">
            <listOfReactants>
                  <speciesReference species="M_atp_c" stoichiometry="1"
constant="true"/>
                  <speciesReference species="M_cmp_c" stoichiometry="1"
constant="true"/>
            </listOfReactants>
            <listOfProducts>
                  <speciesReference species="M_adp_c" stoichiometry="1"
constant="true"/>
                  <speciesReference species="M_cdp_c" stoichiometry="1"
constant="true"/>
            </listOfProducts>
            <fbc:geneProductAssociation>
                  <fbc:geneProductRef fbc:geneProduct="SAUSA300_1367"/>
            </fbc:geneProductAssociation>
      </reaction>

      <reaction metaid="R_CYTK11" id="R_CYTK11" name="cytidylate kinase
(dCMP,dGTP)" reversible="true" fast="false" fbc:lowerFluxBound="rev_lb"
fbc:upperFluxBound="rev_ub">
            <listOfReactants>
                  <speciesReference species="M_cytd_c" stoichiometry="1"
constant="true"/>
                  <speciesReference species="M_dgtp_c" stoichiometry="1"
constant="true"/>
            </listOfReactants>
            <listOfProducts>
                  <speciesReference species="M_cmp_c" stoichiometry="1"
constant="true"/>
                  <speciesReference species="M_dgdp_c" stoichiometry="1"
constant="true"/>
            </listOfProducts>
            <fbc:geneProductAssociation>
                  <fbc:geneProductRef fbc:geneProduct="SAUSA300_1568"/>
            </fbc:geneProductAssociation>
      </reaction>

      <reaction metaid="R_CYTK2" id="R_CYTK2" name="cytidylate kinase (dCMP)"
reversible="true" fast="false" fbc:lowerFluxBound="rev_lb"
fbc:upperFluxBound="rev_ub">
            <listOfReactants>
                  <speciesReference species="M_atp_c" stoichiometry="1"
constant="true"/>
                  <speciesReference species="M_dcmp_c" stoichiometry="1"
constant="true"/>
            </listOfReactants>
```

```
            <listOfProducts>
                  <speciesReference species="M_adp_c" stoichiometry="1"
constant="true"/>
                  <speciesReference species="M_dcdp_c" stoichiometry="1"
constant="true"/>
            </listOfProducts>
            <fbc:geneProductAssociation>
                  <fbc:geneProductRef fbc:geneProduct="SAUSA300_1367"/>
            </fbc:geneProductAssociation>
      </reaction>

      <reaction metaid="R_DACTPP" id="R_DACTPP" name="dATP cytidine 5'-
phosphotransferase " reversible="false" fast="false"
fbc:lowerFluxBound="irr_lb" fbc:upperFluxBound="irr_ub">
            <listOfReactants>
                  <speciesReference species="M_cytd_c" stoichiometry="1"
constant="true"/>
                  <speciesReference species="M_datp_c" stoichiometry="1"
constant="true"/>
            </listOfReactants>
            <listOfProducts>
                  <speciesReference species="M_cmp_c" stoichiometry="1"
constant="true"/>
                  <speciesReference species="M_dadp_c" stoichiometry="1"
constant="true"/>
            </listOfProducts>
            <fbc:geneProductAssociation>
                  <fbc:geneProductRef fbc:geneProduct="SAUSA300_1568"/>
            </fbc:geneProductAssociation>
      </reaction>

      <reaction metaid="R_DADA5" id="R_DADA5" name="Deoxyadenosine deaminase"
reversible="true" fast="false" fbc:lowerFluxBound="rev_lb"
fbc:upperFluxBound="rev_ub">
            <listOfReactants>
                  <speciesReference species="M_dad__5_c" stoichiometry="1"
constant="true"/>
                  <speciesReference species="M_h_c" stoichiometry="1"
constant="true"/>
                  <speciesReference species="M_h2o_c" stoichiometry="1"
constant="true"/>
            </listOfReactants>
            <listOfProducts>
                  <speciesReference species="M_din_c" stoichiometry="1"
constant="true"/>
                  <speciesReference species="M_nh4_c" stoichiometry="1"
constant="true"/>
            </listOfProducts>
            <fbc:geneProductAssociation>
                  <fbc:geneProductRef fbc:geneProduct="ada"/>
            </fbc:geneProductAssociation>
      </reaction>

      <reaction metaid="R_DADK" id="R_DADK" name="deoxyadenylate kinase"
reversible="true" fast="false" fbc:lowerFluxBound="rev_lb"
fbc:upperFluxBound="rev_ub">
            <listOfReactants>
```

```
                  <speciesReference species="M_atp_c" stoichiometry="1"
constant="true"/>
                  <speciesReference species="M_damp_c" stoichiometry="1"
constant="true"/>
            </listOfReactants>
            <listOfProducts>
                  <speciesReference species="M_adp_c" stoichiometry="1"
constant="true"/>
                  <speciesReference species="M_dadp_c" stoichiometry="1"
constant="true"/>
            </listOfProducts>
            <fbc:geneProductAssociation>
                  <fbc:geneProductRef fbc:geneProduct="SAUSA300_2183"/>
            </fbc:geneProductAssociation>
      </reaction>

      <reaction metaid="R_DADNK" id="R_DADNK" name="deoxyadenosine kinase"
reversible="false" fast="false" fbc:lowerFluxBound="irr_lb"
fbc:upperFluxBound="irr_ub">
            <listOfReactants>
                  <speciesReference species="M_atp_c" stoichiometry="1"
constant="true"/>
                  <speciesReference species="M_dad__2_c" stoichiometry="1"
constant="true"/>
            </listOfReactants>
            <listOfProducts>
                  <speciesReference species="M_adp_c" stoichiometry="1"
constant="true"/>
                  <speciesReference species="M_damp_c" stoichiometry="1"
constant="true"/>
            </listOfProducts>
            <fbc:geneProductAssociation>
                  <fbc:or>
                        <fbc:geneProductRef fbc:geneProduct="SAUSA300_0541"/>
                        <fbc:geneProductRef fbc:geneProduct="SAUSA300_0542"/>
                  </fbc:or>
            </fbc:geneProductAssociation>
      </reaction>

      <reaction metaid="R_DADNt2r" id="R_DADNt2r" name="DADNt2 "
reversible="true" fast="false" fbc:lowerFluxBound="rev_lb"
fbc:upperFluxBound="rev_ub">
            <listOfReactants>
                  <speciesReference species="M_h_e" stoichiometry="1"
constant="true"/>
                  <speciesReference species="M_dad__2_e" stoichiometry="1"
constant="true"/>
            </listOfReactants>
            <listOfProducts>
                  <speciesReference species="M_h_c" stoichiometry="1"
constant="true"/>
                  <speciesReference species="M_dad__2_c" stoichiometry="1"
constant="true"/>
            </listOfProducts>
            <fbc:geneProductAssociation>
                  <fbc:geneProductRef fbc:geneProduct="SAUSA300_0506"/>
            </fbc:geneProductAssociation>
      </reaction>
```

```
      <reaction metaid="R_DAGK_SA" id="R_DAGK_SA" name="Diacylglycerol
kinase" reversible="false" fast="false" fbc:lowerFluxBound="irr_lb"
fbc:upperFluxBound="irr_ub">
            <listOfReactants>
                  <speciesReference species="M_atp_c" stoichiometry="1"
constant="true"/>
                  <speciesReference species="M_12dgr_SA_c"
stoichiometry="0.02" constant="true"/>
            </listOfReactants>
            <listOfProducts>
                  <speciesReference species="M_h_c" stoichiometry="1"
constant="true"/>
                  <speciesReference species="M_adp_c" stoichiometry="1"
constant="true"/>
                  <speciesReference species="M_pa_SA_c" stoichiometry="0.02"
constant="true"/>
            </listOfProducts>
            <fbc:geneProductAssociation>
                  <fbc:or>
                        <fbc:geneProductRef fbc:geneProduct="SAUSA300_1529"/>
                        <fbc:geneProductRef fbc:geneProduct="SAUSA300_1879"/>
                  </fbc:or>
            </fbc:geneProductAssociation>
      </reaction>

      <reaction metaid="R_DAGK120" id="R_DAGK120" name="diacylglycerol kinase
(n-C12:0)" reversible="false" fast="false" fbc:lowerFluxBound="irr_lb"
fbc:upperFluxBound="irr_ub">
            <listOfReactants>
                  <speciesReference species="M_atp_c" stoichiometry="1"
constant="true"/>
                  <speciesReference species="M_12dgr120_c" stoichiometry="1"
constant="true"/>
            </listOfReactants>
            <listOfProducts>
                  <speciesReference species="M_adp_c" stoichiometry="1"
constant="true"/>
                  <speciesReference species="M_pa120_c" stoichiometry="1"
constant="true"/>
            </listOfProducts>
            <fbc:geneProductAssociation>
                  <fbc:geneProductRef fbc:geneProduct="SAUSA300_1529"/>
            </fbc:geneProductAssociation>
      </reaction>

      <reaction metaid="R_DAGK140" id="R_DAGK140" name="diacylglycerol kinase
(n-C14:0)" reversible="false" fast="false" fbc:lowerFluxBound="irr_lb"
fbc:upperFluxBound="irr_ub">
            <listOfReactants>
                  <speciesReference species="M_atp_c" stoichiometry="1"
constant="true"/>
                  <speciesReference species="M_12dgr140_c" stoichiometry="1"
constant="true"/>
            </listOfReactants>
            <listOfProducts>
                  <speciesReference species="M_adp_c" stoichiometry="1"
constant="true"/>
```

```
                  <speciesReference species="M_pa140_c" stoichiometry="1"
constant="true"/>
            </listOfProducts>
            <fbc:geneProductAssociation>
                  <fbc:geneProductRef fbc:geneProduct="SAUSA300_1529"/>
            </fbc:geneProductAssociation>
      </reaction>

      <reaction metaid="R_DAGK141" id="R_DAGK141" name="diacylglycerol kinase
(n-C14:1)" reversible="false" fast="false" fbc:lowerFluxBound="irr_lb"
fbc:upperFluxBound="irr_ub">
            <listOfReactants>
                  <speciesReference species="M_atp_c" stoichiometry="1"
constant="true"/>
                  <speciesReference species="M_12dgr141_c" stoichiometry="1"
constant="true"/>
            </listOfReactants>
            <listOfProducts>
                  <speciesReference species="M_adp_c" stoichiometry="1"
constant="true"/>
                  <speciesReference species="M_pa141_c" stoichiometry="1"
constant="true"/>
            </listOfProducts>
            <fbc:geneProductAssociation>
                  <fbc:geneProductRef fbc:geneProduct="SAUSA300_1529"/>
            </fbc:geneProductAssociation>
      </reaction>

      <reaction metaid="R_DAGK160" id="R_DAGK160" name="diacylglycerol kinase
(n-C16:0)" reversible="false" fast="false" fbc:lowerFluxBound="irr_lb"
fbc:upperFluxBound="irr_ub">
            <listOfReactants>
                  <speciesReference species="M_atp_c" stoichiometry="1"
constant="true"/>
                  <speciesReference species="M_12dgr160_c" stoichiometry="1"
constant="true"/>
            </listOfReactants>
            <listOfProducts>
                  <speciesReference species="M_adp_c" stoichiometry="1"
constant="true"/>
                  <speciesReference species="M_pa160_c" stoichiometry="1"
constant="true"/>
            </listOfProducts>
            <fbc:geneProductAssociation>
                  <fbc:geneProductRef fbc:geneProduct="SAUSA300_1529"/>
            </fbc:geneProductAssociation>
      </reaction>

      <reaction metaid="R_DAGK161" id="R_DAGK161" name="diacylglycerol kinase
(n-C16:1)" reversible="false" fast="false" fbc:lowerFluxBound="irr_lb"
fbc:upperFluxBound="irr_ub">
            <listOfReactants>
                  <speciesReference species="M_atp_c" stoichiometry="1"
constant="true"/>
                  <speciesReference species="M_12dgr161_c" stoichiometry="1"
constant="true"/>
            </listOfReactants>
            <listOfProducts>
```

```
                  <speciesReference species="M_adp_c" stoichiometry="1"
constant="true"/>
                  <speciesReference species="M_pa161_c" stoichiometry="1"
constant="true"/>
            </listOfProducts>
            <fbc:geneProductAssociation>
                  <fbc:geneProductRef fbc:geneProduct="SAUSA300_1529"/>
            </fbc:geneProductAssociation>
      </reaction>

      <reaction metaid="R_DAGK180" id="R_DAGK180" name="diacylglycerol kinase
(n-C18:0)" reversible="false" fast="false" fbc:lowerFluxBound="irr_lb"
fbc:upperFluxBound="irr_ub">
            <listOfReactants>
                  <speciesReference species="M_atp_c" stoichiometry="1"
constant="true"/>
                  <speciesReference species="M_12dgr180_c" stoichiometry="1"
constant="true"/>
            </listOfReactants>
            <listOfProducts>
                  <speciesReference species="M_adp_c" stoichiometry="1"
constant="true"/>
                  <speciesReference species="M_pa180_c" stoichiometry="1"
constant="true"/>
            </listOfProducts>
            <fbc:geneProductAssociation>
                  <fbc:geneProductRef fbc:geneProduct="SAUSA300_1529"/>
            </fbc:geneProductAssociation>
      </reaction>

      <reaction metaid="R_DAGK181" id="R_DAGK181" name="diacylglycerol kinase
(n-C18:1)" reversible="false" fast="false" fbc:lowerFluxBound="irr_lb"
fbc:upperFluxBound="irr_ub">
            <listOfReactants>
                  <speciesReference species="M_atp_c" stoichiometry="1"
constant="true"/>
                  <speciesReference species="M_12dgr181_c" stoichiometry="1"
constant="true"/>
            </listOfReactants>
            <listOfProducts>
                  <speciesReference species="M_adp_c" stoichiometry="1"
constant="true"/>
                  <speciesReference species="M_pa181_c" stoichiometry="1"
constant="true"/>
            </listOfProducts>
            <fbc:geneProductAssociation>
                  <fbc:geneProductRef fbc:geneProduct="SAUSA300_1529"/>
            </fbc:geneProductAssociation>
      </reaction>

      <reaction metaid="R_DALAL" id="R_DALAL" name="D-alanine-
polyphosphoribitol ligase " reversible="false" fast="false"
fbc:lowerFluxBound="irr_lb" fbc:upperFluxBound="irr_ub">
            <listOfReactants>
                  <speciesReference species="M_atp_c" stoichiometry="45"
constant="true"/>
                  <speciesReference species="M_pren45_c" stoichiometry="1"
constant="true"/>
```

```
                  <speciesReference species="M_ala__D_c" stoichiometry="45"
constant="true"/>
            </listOfReactants>
            <listOfProducts>
                  <speciesReference species="M_ppi_c" stoichiometry="45"
constant="true"/>
                  <speciesReference species="M_pretechala_c"
stoichiometry="1" constant="true"/>
                  <speciesReference species="M_amp_c" stoichiometry="45"
constant="true"/>
            </listOfProducts>
            <fbc:geneProductAssociation>
                  <fbc:and>
                        <fbc:geneProductRef fbc:geneProduct="SAUSA300_0838"/>
                        <fbc:geneProductRef fbc:geneProduct="SAUSA300_0837"/>
                        <fbc:geneProductRef fbc:geneProduct="SAUSA300_0836"/>
                        <fbc:geneProductRef fbc:geneProduct="SAUSA300_0835"/>
                  </fbc:and>
            </fbc:geneProductAssociation>
      </reaction>

      <reaction metaid="R_DALAt2r" id="R_DALAt2r" name="D-alanine transport
via proton symport" reversible="true" fast="false"
fbc:lowerFluxBound="rev_lb" fbc:upperFluxBound="rev_ub">
            <listOfReactants>
                  <speciesReference species="M_h_e" stoichiometry="1"
constant="true"/>
                  <speciesReference species="M_ala__D_e" stoichiometry="1"
constant="true"/>
            </listOfReactants>
            <listOfProducts>
                  <speciesReference species="M_h_c" stoichiometry="1"
constant="true"/>
                  <speciesReference species="M_ala__D_c" stoichiometry="1"
constant="true"/>
            </listOfProducts>
            <fbc:geneProductAssociation>
                  <fbc:or>
                        <fbc:geneProductRef fbc:geneProduct="SAUSA300_1642"/>
                        <fbc:geneProductRef fbc:geneProduct="alsT"/>
                        <fbc:geneProductRef fbc:geneProduct="SAUSA300_0914"/>
                        <fbc:geneProductRef fbc:geneProduct="SAUSA300_0712"/>
                  </fbc:or>
            </fbc:geneProductAssociation>
      </reaction>

      <reaction metaid="R_DAPDA" id="R_DAPDA" name="N-
acetyl__L,L__Diaminopimelate deacetylase" reversible="true" fast="false"
fbc:lowerFluxBound="rev_lb" fbc:upperFluxBound="rev_ub">
            <listOfReactants>
                  <speciesReference species="M_h2o_c" stoichiometry="1"
constant="true"/>
                  <speciesReference species="M_n6all26d_c" stoichiometry="1"
constant="true"/>
            </listOfReactants>
            <listOfProducts>
                  <speciesReference species="M_ac_c" stoichiometry="1"
constant="true"/>
```

```
                  <speciesReference species="M_26dap__LL_c" stoichiometry="1"
constant="true"/>
            </listOfProducts>
            <fbc:geneProductAssociation>
                  <fbc:or>
                        <fbc:geneProductRef fbc:geneProduct="SAUSA300_1291"/>
                        <fbc:geneProductRef fbc:geneProduct="SAUSA300_0534"/>
                  </fbc:or>
            </fbc:geneProductAssociation>
      </reaction>

      <reaction metaid="R_DAPDC" id="R_DAPDC" name="diaminopimelate
decarboxylase" reversible="false" fast="false" fbc:lowerFluxBound="irr_lb"
fbc:upperFluxBound="irr_ub">
            <listOfReactants>
                  <speciesReference species="M_h_c" stoichiometry="1"
constant="true"/>
                  <speciesReference species="M_26dap__M_c" stoichiometry="1"
constant="true"/>
            </listOfReactants>
            <listOfProducts>
                  <speciesReference species="M_co2_c" stoichiometry="1"
constant="true"/>
                  <speciesReference species="M_lys__L_c" stoichiometry="1"
constant="true"/>
            </listOfProducts>
            <fbc:geneProductAssociation>
                  <fbc:or>
                        <fbc:geneProductRef fbc:geneProduct="SAUSA300_1293"/>
                        <fbc:geneProductRef fbc:geneProduct="SAUSA300_0125"/>
                  </fbc:or>
            </fbc:geneProductAssociation>
      </reaction>

      <reaction metaid="R_DAPE" id="R_DAPE" name="diaminopimelate epimerase"
reversible="true" fast="false" fbc:lowerFluxBound="rev_lb"
fbc:upperFluxBound="rev_ub">
            <listOfReactants>
                  <speciesReference species="M_26dap__LL_c" stoichiometry="1"
constant="true"/>
            </listOfReactants>
            <listOfProducts>
                  <speciesReference species="M_26dap__M_c" stoichiometry="1"
constant="true"/>
            </listOfProducts>
            <fbc:geneProductAssociation>
                  <fbc:or>
                        <fbc:geneProductRef fbc:geneProduct="SAUSA300_1292"/>
                        <fbc:geneProductRef fbc:geneProduct="SAUSA300_1454"/>
                  </fbc:or>
            </fbc:geneProductAssociation>
      </reaction>

      <reaction metaid="R_DASYN_SA" id="R_DASYN_SA" name="CDP__Diacylglycerol
synthetase (Saureus)" reversible="true" fast="false"
fbc:lowerFluxBound="rev_lb" fbc:upperFluxBound="rev_ub">
            <listOfReactants>
```

```
                  <speciesReference species="M_h_c" stoichiometry="1"
constant="true"/>
                  <speciesReference species="M_ctp_c" stoichiometry="1"
constant="true"/>
                  <speciesReference species="M_pa_SA_c" stoichiometry="0.02"
constant="true"/>
            </listOfReactants>
            <listOfProducts>
                  <speciesReference species="M_ppi_c" stoichiometry="1"
constant="true"/>
                  <speciesReference species="M_cdpdag_SA_c"
stoichiometry="0.02" constant="true"/>
            </listOfProducts>
            <fbc:geneProductAssociation>
                  <fbc:geneProductRef fbc:geneProduct="SAUSA300_1154"/>
            </fbc:geneProductAssociation>
      </reaction>

      <reaction metaid="R_DASYN120" id="R_DASYN120" name="CDP__Diacylglycerol
synthetase (n-C12:0)" reversible="false" fast="false"
fbc:lowerFluxBound="irr_lb" fbc:upperFluxBound="irr_ub">
            <listOfReactants>
                  <speciesReference species="M_ctp_c" stoichiometry="1"
constant="true"/>
                  <speciesReference species="M_pa120_c" stoichiometry="1"
constant="true"/>
            </listOfReactants>
            <listOfProducts>
                  <speciesReference species="M_ppi_c" stoichiometry="1"
constant="true"/>
                  <speciesReference species="M_cdpdddecg_c" stoichiometry="1"
constant="true"/>
            </listOfProducts>
            <fbc:geneProductAssociation>
                  <fbc:geneProductRef fbc:geneProduct="SAUSA300_1154"/>
            </fbc:geneProductAssociation>
      </reaction>

      <reaction metaid="R_DASYN140" id="R_DASYN140" name="CDP__Diacylglycerol
synthetase (n-C14:0)" reversible="false" fast="false"
fbc:lowerFluxBound="irr_lb" fbc:upperFluxBound="irr_ub">
            <listOfReactants>
                  <speciesReference species="M_ctp_c" stoichiometry="1"
constant="true"/>
                  <speciesReference species="M_pa140_c" stoichiometry="1"
constant="true"/>
            </listOfReactants>
            <listOfProducts>
                  <speciesReference species="M_ppi_c" stoichiometry="1"
constant="true"/>
                  <speciesReference species="M_cdpdtdecg_c" stoichiometry="1"
constant="true"/>
            </listOfProducts>
            <fbc:geneProductAssociation>
                  <fbc:geneProductRef fbc:geneProduct="SAUSA300_1154"/>
            </fbc:geneProductAssociation>
      </reaction>
```

```
      <reaction metaid="R_DASYN141" id="R_DASYN141" name="CDP__Diacylglycerol
synthetase (n-C14:1)" reversible="false" fast="false"
fbc:lowerFluxBound="irr_lb" fbc:upperFluxBound="irr_ub">
            <listOfReactants>
                  <speciesReference species="M_ctp_c" stoichiometry="1"
constant="true"/>
                  <speciesReference species="M_pa141_c" stoichiometry="1"
constant="true"/>
            </listOfReactants>
            <listOfProducts>
                  <speciesReference species="M_ppi_c" stoichiometry="1"
constant="true"/>
                  <speciesReference species="M_cdpdtdec7eg_c"
stoichiometry="1" constant="true"/>
            </listOfProducts>
            <fbc:geneProductAssociation>
                  <fbc:geneProductRef fbc:geneProduct="SAUSA300_1154"/>
            </fbc:geneProductAssociation>
      </reaction>

      <reaction metaid="R_DASYN160" id="R_DASYN160" name="CDP__Diacylglycerol
synthetase (n-C16:0)" reversible="false" fast="false"
fbc:lowerFluxBound="irr_lb" fbc:upperFluxBound="irr_ub">
            <listOfReactants>
                  <speciesReference species="M_ctp_c" stoichiometry="1"
constant="true"/>
                  <speciesReference species="M_pa160_c" stoichiometry="1"
constant="true"/>
            </listOfReactants>
            <listOfProducts>
                  <speciesReference species="M_ppi_c" stoichiometry="1"
constant="true"/>
                  <speciesReference species="M_cdpdhdecg_c" stoichiometry="1"
constant="true"/>
            </listOfProducts>
            <fbc:geneProductAssociation>
                  <fbc:geneProductRef fbc:geneProduct="SAUSA300_1154"/>
            </fbc:geneProductAssociation>
      </reaction>

      <reaction metaid="R_DASYN161" id="R_DASYN161" name="CDP__Diacylglycerol
synthetase (n-C16:1)" reversible="false" fast="false"
fbc:lowerFluxBound="irr_lb" fbc:upperFluxBound="irr_ub">
            <listOfReactants>
                  <speciesReference species="M_ctp_c" stoichiometry="1"
constant="true"/>
                  <speciesReference species="M_pa161_c" stoichiometry="1"
constant="true"/>
            </listOfReactants>
            <listOfProducts>
                  <speciesReference species="M_ppi_c" stoichiometry="1"
constant="true"/>
                  <speciesReference species="M_cdpdhdec9eg_c"
stoichiometry="1" constant="true"/>
            </listOfProducts>
            <fbc:geneProductAssociation>
                  <fbc:geneProductRef fbc:geneProduct="SAUSA300_1154"/>
            </fbc:geneProductAssociation>
```

</reaction>

<reaction metaid="R_DASYN180" id="R_DASYN180" name="CDP__Diacylglycerol synthetase (n-C18:0)" reversible="false" fast="false" fbc:lowerFluxBound="irr_lb" fbc:upperFluxBound="irr_ub">
<listOfReactants>
<speciesReference species="M_ctp_c" stoichiometry="1" constant="true"/>
<speciesReference species="M_pa180_c" stoichiometry="1" constant="true"/>
</listOfReactants>
<listOfProducts>
<speciesReference species="M_ppi_c" stoichiometry="1" constant="true"/>
<speciesReference species="M_cdpdodecg_c" stoichiometry="1" constant="true"/>
</listOfProducts>
<fbc:geneProductAssociation>
<fbc:geneProductRef fbc:geneProduct="SAUSA300_1154"/>
</fbc:geneProductAssociation>
</reaction>

<reaction metaid="R_DASYN181" id="R_DASYN181" name="CDP__Diacylglycerol synthetase (n-C18:1)" reversible="false" fast="false" fbc:lowerFluxBound="irr_lb" fbc:upperFluxBound="irr_ub">
<listOfReactants>
<speciesReference species="M_ctp_c" stoichiometry="1" constant="true"/>
<speciesReference species="M_pa181_c" stoichiometry="1" constant="true"/>
</listOfReactants>
<listOfProducts>
<speciesReference species="M_ppi_c" stoichiometry="1" constant="true"/>
<speciesReference species="M_cdpdodec11eg_c" stoichiometry="1" constant="true"/>
</listOfProducts>
<fbc:geneProductAssociation>
<fbc:geneProductRef fbc:geneProduct="SAUSA300_1154"/>
</fbc:geneProductAssociation>
</reaction>

<reaction metaid="R_DAUPP" id="R_DAUPP" name="dATP uridine 5'-phosphotransferase " reversible="false" fast="false" fbc:lowerFluxBound="irr_lb" fbc:upperFluxBound="irr_ub">
<listOfReactants>
<speciesReference species="M_uri_c" stoichiometry="1" constant="true"/>
<speciesReference species="M_datp_c" stoichiometry="1" constant="true"/>
</listOfReactants>
<listOfProducts>
<speciesReference species="M_dadp_c" stoichiometry="1" constant="true"/>
<speciesReference species="M_ump_c" stoichiometry="1" constant="true"/>
</listOfProducts>
<fbc:geneProductAssociation>

```
                <fbc:geneProductRef fbc:geneProduct="SAUSA300_1568"/>
            </fbc:geneProductAssociation>
        </reaction>

        <reaction metaid="R_DB4PS" id="R_DB4PS" name="3,4__Dihydroxy-2-
butanone-4-phosphate synthase" reversible="false" fast="false"
fbc:lowerFluxBound="irr_lb" fbc:upperFluxBound="irr_ub">
            <listOfReactants>
                <speciesReference species="M_ru5p__D_c" stoichiometry="1"
constant="true"/>
            </listOfReactants>
            <listOfProducts>
                <speciesReference species="M_h_c" stoichiometry="1"
constant="true"/>
                <speciesReference species="M_for_c" stoichiometry="1"
constant="true"/>
                <speciesReference species="M_db4p_c" stoichiometry="1"
constant="true"/>
            </listOfProducts>
            <fbc:geneProductAssociation>
                <fbc:geneProductRef fbc:geneProduct="SAUSA300_1713"/>
            </fbc:geneProductAssociation>
        </reaction>

        <reaction metaid="R_DBTS" id="R_DBTS" name="dethiobiotin synthase"
reversible="false" fast="false" fbc:lowerFluxBound="irr_lb"
fbc:upperFluxBound="irr_ub">
            <listOfReactants>
                <speciesReference species="M_atp_c" stoichiometry="1"
constant="true"/>
                <speciesReference species="M_co2_c" stoichiometry="1"
constant="true"/>
                <speciesReference species="M_dann_c" stoichiometry="1"
constant="true"/>
            </listOfReactants>
            <listOfProducts>
                <speciesReference species="M_h_c" stoichiometry="3"
constant="true"/>
                <speciesReference species="M_pi_c" stoichiometry="1"
constant="true"/>
                <speciesReference species="M_adp_c" stoichiometry="1"
constant="true"/>
                <speciesReference species="M_dtbt_c" stoichiometry="1"
constant="true"/>
            </listOfProducts>
            <fbc:geneProductAssociation>
                <fbc:geneProductRef fbc:geneProduct="SAUSA300_2373"/>
            </fbc:geneProductAssociation>
        </reaction>

        <reaction metaid="R_DCAROTDS" id="R_DCAROTDS" name="4,4'__Diapo-x-
carotene desaturase" reversible="false" fast="false"
fbc:lowerFluxBound="irr_lb" fbc:upperFluxBound="irr_ub">
            <listOfReactants>
                <speciesReference species="M_fad_c" stoichiometry="1"
constant="true"/>
                <speciesReference species="M_thdcar_c" stoichiometry="1"
constant="true"/>
```

```
            </listOfReactants>
            <listOfProducts>
                  <speciesReference species="M_fadh2_c" stoichiometry="1"
constant="true"/>
                  <speciesReference species="M_dnspen_c" stoichiometry="1"
constant="true"/>
            </listOfProducts>
            <fbc:geneProductAssociation>
                  <fbc:geneProductRef fbc:geneProduct="SAUSA300_2498"/>
            </fbc:geneProductAssociation>
      </reaction>

      <reaction metaid="R_DCMPDA" id="R_DCMPDA" name="dCMP deaminase"
reversible="false" fast="false" fbc:lowerFluxBound="irr_lb"
fbc:upperFluxBound="irr_ub">
            <listOfReactants>
                  <speciesReference species="M_h_c" stoichiometry="1"
constant="true"/>
                  <speciesReference species="M_h2o_c" stoichiometry="1"
constant="true"/>
                  <speciesReference species="M_dcmp_c" stoichiometry="1"
constant="true"/>
            </listOfReactants>
            <listOfProducts>
                  <speciesReference species="M_nh4_c" stoichiometry="1"
constant="true"/>
                  <speciesReference species="M_dump_c" stoichiometry="1"
constant="true"/>
            </listOfProducts>
            <fbc:geneProductAssociation>
                  <fbc:geneProductRef fbc:geneProduct="SAUSA300_1548"/>
            </fbc:geneProductAssociation>
      </reaction>

      <reaction metaid="R_DCPP" id="R_DCPP" name="dCTP cytidine 5'-
phosphotransferase " reversible="false" fast="false"
fbc:lowerFluxBound="irr_lb" fbc:upperFluxBound="irr_ub">
            <listOfReactants>
                  <speciesReference species="M_cytd_c" stoichiometry="1"
constant="true"/>
                  <speciesReference species="M_dctp_c" stoichiometry="1"
constant="true"/>
            </listOfReactants>
            <listOfProducts>
                  <speciesReference species="M_cmp_c" stoichiometry="1"
constant="true"/>
                  <speciesReference species="M_dcdp_c" stoichiometry="1"
constant="true"/>
            </listOfProducts>
            <fbc:geneProductAssociation>
                  <fbc:geneProductRef fbc:geneProduct="SAUSA300_1568"/>
            </fbc:geneProductAssociation>
      </reaction>

      <reaction metaid="R_DCYTD" id="R_DCYTD" name="deoxycytidine deaminase"
reversible="false" fast="false" fbc:lowerFluxBound="irr_lb"
fbc:upperFluxBound="irr_ub">
            <listOfReactants>
```

```
                  <speciesReference species="M_h_c" stoichiometry="1"
constant="true"/>
                  <speciesReference species="M_h2o_c" stoichiometry="1"
constant="true"/>
                  <speciesReference species="M_dcyt_c" stoichiometry="1"
constant="true"/>
            </listOfReactants>
            <listOfProducts>
                  <speciesReference species="M_nh4_c" stoichiometry="1"
constant="true"/>
                  <speciesReference species="M_duri_c" stoichiometry="1"
constant="true"/>
            </listOfProducts>
            <fbc:geneProductAssociation>
                  <fbc:geneProductRef fbc:geneProduct="SAUSA300_1528"/>
            </fbc:geneProductAssociation>
      </reaction>

      <reaction metaid="R_DCYTt2r" id="R_DCYTt2r" name="Deoxycytidine ion-
coupled transport " reversible="true" fast="false"
fbc:lowerFluxBound="rev_lb" fbc:upperFluxBound="rev_ub">
            <listOfReactants>
                  <speciesReference species="M_h_e" stoichiometry="1"
constant="true"/>
                  <speciesReference species="M_dcyt_e" stoichiometry="1"
constant="true"/>
            </listOfReactants>
            <listOfProducts>
                  <speciesReference species="M_h_c" stoichiometry="1"
constant="true"/>
                  <speciesReference species="M_dcyt_c" stoichiometry="1"
constant="true"/>
            </listOfProducts>
            <fbc:geneProductAssociation>
                  <fbc:geneProductRef fbc:geneProduct="SAUSA300_0506"/>
            </fbc:geneProductAssociation>
      </reaction>

      <reaction metaid="R_DDGLK" id="R_DDGLK" name="2__Dehydro-
3__Deoxygluconokinase" reversible="false" fast="false"
fbc:lowerFluxBound="irr_lb" fbc:upperFluxBound="irr_ub">
            <listOfReactants>
                  <speciesReference species="M_atp_c" stoichiometry="1"
constant="true"/>
                  <speciesReference species="M_2ddglcn_c" stoichiometry="1"
constant="true"/>
            </listOfReactants>
            <listOfProducts>
                  <speciesReference species="M_h_c" stoichiometry="1"
constant="true"/>
                  <speciesReference species="M_adp_c" stoichiometry="1"
constant="true"/>
                  <speciesReference species="M_2ddg6p_c" stoichiometry="1"
constant="true"/>
            </listOfProducts>
            <fbc:geneProductAssociation>
                  <fbc:geneProductRef fbc:geneProduct="SAUSA300_2443"/>
            </fbc:geneProductAssociation>
```

```
      </reaction>

      <reaction metaid="R_DDPA" id="R_DDPA" name="3__Deoxy__D-arabino-
heptulosonate 7-phosphate synthetase" reversible="false" fast="false"
fbc:lowerFluxBound="irr_lb" fbc:upperFluxBound="irr_ub">
            <listOfReactants>
                  <speciesReference species="M_h2o_c" stoichiometry="1"
constant="true"/>
                  <speciesReference species="M_pep_c" stoichiometry="1"
constant="true"/>
                  <speciesReference species="M_e4p_c" stoichiometry="1"
constant="true"/>
            </listOfReactants>
            <listOfProducts>
                  <speciesReference species="M_h_c" stoichiometry="1"
constant="true"/>
                  <speciesReference species="M_pi_c" stoichiometry="1"
constant="true"/>
                  <speciesReference species="M_2dda7p_c" stoichiometry="1"
constant="true"/>
            </listOfProducts>
            <fbc:geneProductAssociation>
                  <fbc:geneProductRef fbc:geneProduct="SAUSA300_1683"/>
            </fbc:geneProductAssociation>
      </reaction>

      <reaction metaid="R_DGK1" id="R_DGK1" name="deoxyguanylate kinase
(dGMP:ATP)" reversible="true" fast="false" fbc:lowerFluxBound="rev_lb"
fbc:upperFluxBound="rev_ub">
            <listOfReactants>
                  <speciesReference species="M_atp_c" stoichiometry="1"
constant="true"/>
                  <speciesReference species="M_dgmp_c" stoichiometry="1"
constant="true"/>
            </listOfReactants>
            <listOfProducts>
                  <speciesReference species="M_adp_c" stoichiometry="1"
constant="true"/>
                  <speciesReference species="M_dgdp_c" stoichiometry="1"
constant="true"/>
            </listOfProducts>
            <fbc:geneProductAssociation>
                  <fbc:geneProductRef fbc:geneProduct="SAUSA300_1102"/>
            </fbc:geneProductAssociation>
      </reaction>

      <reaction metaid="R_DGLUO" id="R_DGLUO" name="D-Glucitol NAD 2-
oxidoreductase " reversible="true" fast="false" fbc:lowerFluxBound="rev_lb"
fbc:upperFluxBound="rev_ub">
            <listOfReactants>
                  <speciesReference species="M_nad_c" stoichiometry="1"
constant="true"/>
                  <speciesReference species="M_sbt__D_c" stoichiometry="1"
constant="true"/>
            </listOfReactants>
            <listOfProducts>
                  <speciesReference species="M_h_c" stoichiometry="1"
constant="true"/>
```

```
                  <speciesReference species="M_nadh_c" stoichiometry="1"
constant="true"/>
                  <speciesReference species="M_fru_c" stoichiometry="1"
constant="true"/>
            </listOfProducts>
            <fbc:geneProductAssociation>
                  <fbc:geneProductRef fbc:geneProduct="SAUSA300_0244"/>
            </fbc:geneProductAssociation>
      </reaction>

      <reaction metaid="R_DGNSK" id="R_DGNSK" name="ATP deoxyguanosine 5'-
phosphotransferase " reversible="false" fast="false"
fbc:lowerFluxBound="irr_lb" fbc:upperFluxBound="irr_ub">
            <listOfReactants>
                  <speciesReference species="M_atp_c" stoichiometry="1"
constant="true"/>
                  <speciesReference species="M_dgsn_c" stoichiometry="1"
constant="true"/>
            </listOfReactants>
            <listOfProducts>
                  <speciesReference species="M_adp_c" stoichiometry="1"
constant="true"/>
                  <speciesReference species="M_dgmp_c" stoichiometry="1"
constant="true"/>
            </listOfProducts>
            <fbc:geneProductAssociation>
                  <fbc:or>
                        <fbc:geneProductRef fbc:geneProduct="SAUSA300_0541"/>
                        <fbc:geneProductRef fbc:geneProduct="SAUSA300_0542"/>
                  </fbc:or>
            </fbc:geneProductAssociation>
      </reaction>

      <reaction metaid="R_DGSNt2" id="R_DGSNt2" name="deoxyguanosine
transport in via proton symport" reversible="true" fast="false"
fbc:lowerFluxBound="rev_lb" fbc:upperFluxBound="rev_ub">
            <listOfReactants>
                  <speciesReference species="M_h_e" stoichiometry="1"
constant="true"/>
                  <speciesReference species="M_dgsn_e" stoichiometry="1"
constant="true"/>
            </listOfReactants>
            <listOfProducts>
                  <speciesReference species="M_h_c" stoichiometry="1"
constant="true"/>
                  <speciesReference species="M_dgsn_c" stoichiometry="1"
constant="true"/>
            </listOfProducts>
            <fbc:geneProductAssociation>
                  <fbc:geneProductRef fbc:geneProduct="SAUSA300_0506"/>
            </fbc:geneProductAssociation>
      </reaction>

      <reaction metaid="R_DGUPP" id="R_DGUPP" name="dGTP uridine 5'-
phosphotransferase " reversible="false" fast="false"
fbc:lowerFluxBound="irr_lb" fbc:upperFluxBound="irr_ub">
            <listOfReactants>
```

```
                  <speciesReference species="M_uri_c" stoichiometry="1"
constant="true"/>
                  <speciesReference species="M_dgtp_c" stoichiometry="1"
constant="true"/>
            </listOfReactants>
            <listOfProducts>
                  <speciesReference species="M_ump_c" stoichiometry="1"
constant="true"/>
                  <speciesReference species="M_dgdp_c" stoichiometry="1"
constant="true"/>
            </listOfProducts>
            <fbc:geneProductAssociation>
                  <fbc:geneProductRef fbc:geneProduct="SAUSA300_1568"/>
            </fbc:geneProductAssociation>
      </reaction>

      <reaction metaid="R_DHAD1" id="R_DHAD1" name="dihydroxy-acid
dehydratase (2,3__Dihydroxy-3-methylbutanoate)" reversible="false"
fast="false" fbc:lowerFluxBound="irr_lb" fbc:upperFluxBound="irr_ub">
            <listOfReactants>
                  <speciesReference species="M_23dhmb_c" stoichiometry="1"
constant="true"/>
            </listOfReactants>
            <listOfProducts>
                  <speciesReference species="M_h2o_c" stoichiometry="1"
constant="true"/>
                  <speciesReference species="M_3mob_c" stoichiometry="1"
constant="true"/>
            </listOfProducts>
            <fbc:geneProductAssociation>
                  <fbc:geneProductRef fbc:geneProduct="SAUSA300_2006"/>
            </fbc:geneProductAssociation>
      </reaction>

      <reaction metaid="R_DHAD2" id="R_DHAD2" name="Dihydroxy-acid
dehydratase (2,3__Dihydroxy-3-methylpentanoate)" reversible="false"
fast="false" fbc:lowerFluxBound="irr_lb" fbc:upperFluxBound="irr_ub">
            <listOfReactants>
                  <speciesReference species="M_23dhmp_c" stoichiometry="1"
constant="true"/>
            </listOfReactants>
            <listOfProducts>
                  <speciesReference species="M_h2o_c" stoichiometry="1"
constant="true"/>
                  <speciesReference species="M_2o3mpt_c" stoichiometry="1"
constant="true"/>
            </listOfProducts>
            <fbc:geneProductAssociation>
                  <fbc:geneProductRef fbc:geneProduct="SAUSA300_2006"/>
            </fbc:geneProductAssociation>
      </reaction>

      <reaction metaid="R_DHAD3" id="R_DHAD3" name="R-2 3__Dihydroxy-3-
methylpentanoate hydro__Lyase " reversible="false" fast="false"
fbc:lowerFluxBound="irr_lb" fbc:upperFluxBound="irr_ub">
            <listOfReactants>
                  <speciesReference species="M_23dhmp_c" stoichiometry="1"
constant="true"/>
```

```
            </listOfReactants>
            <listOfProducts>
                  <speciesReference species="M_h2o_c" stoichiometry="1"
constant="true"/>
                  <speciesReference species="M_3mop_c" stoichiometry="1"
constant="true"/>
            </listOfProducts>
            <fbc:geneProductAssociation>
                  <fbc:geneProductRef fbc:geneProduct="SAUSA300_2006"/>
            </fbc:geneProductAssociation>
      </reaction>

      <reaction metaid="R_DHAK" id="R_DHAK" name="dihydroxyacetone kinase"
reversible="false" fast="false" fbc:lowerFluxBound="irr_lb"
fbc:upperFluxBound="irr_ub">
            <listOfReactants>
                  <speciesReference species="M_atp_c" stoichiometry="1"
constant="true"/>
                  <speciesReference species="M_dha_c" stoichiometry="1"
constant="true"/>
            </listOfReactants>
            <listOfProducts>
                  <speciesReference species="M_h_c" stoichiometry="1"
constant="true"/>
                  <speciesReference species="M_adp_c" stoichiometry="1"
constant="true"/>
                  <speciesReference species="M_dhap_c" stoichiometry="1"
constant="true"/>
            </listOfProducts>
            <fbc:geneProductAssociation>
                  <fbc:geneProductRef fbc:geneProduct="SAUSA300_0636"/>
            </fbc:geneProductAssociation>
      </reaction>

      <reaction metaid="R_dhap_Et" id="R_dhap_Et" name="dhap transport
(transport mechanism unknown - evidence from biolog data)" reversible="true"
fast="false" fbc:lowerFluxBound="rev_lb" fbc:upperFluxBound="rev_ub">
            <listOfReactants>
                  <speciesReference species="M_dhap_e" stoichiometry="1"
constant="true"/>
            </listOfReactants>
            <listOfProducts>
                  <speciesReference species="M_dhap_c" stoichiometry="1"
constant="true"/>
            </listOfProducts>
      </reaction>

      <reaction metaid="R_DHAPT" id="R_DHAPT" name="Dihydroxyacetone
phosphotransferase" reversible="false" fast="false"
fbc:lowerFluxBound="irr_lb" fbc:upperFluxBound="irr_ub">
            <listOfReactants>
                  <speciesReference species="M_pep_c" stoichiometry="1"
constant="true"/>
                  <speciesReference species="M_dha_c" stoichiometry="1"
constant="true"/>
            </listOfReactants>
            <listOfProducts>
```

```
                  <speciesReference species="M_pyr_c" stoichiometry="1"
constant="true"/>
                  <speciesReference species="M_dhap_c" stoichiometry="1"
constant="true"/>
            </listOfProducts>
            <fbc:geneProductAssociation>
                  <fbc:or>
                        <fbc:geneProductRef fbc:geneProduct="SAUSA300_0638"/>
                        <fbc:geneProductRef fbc:geneProduct="SAUSA300_0637"/>
                        <fbc:geneProductRef fbc:geneProduct="SAUSA300_0636"/>
                  </fbc:or>
            </fbc:geneProductAssociation>
      </reaction>

      <reaction metaid="R_DHDPRy" id="R_DHDPRy" name="dihydrodipicolinate
reductase (NADPH)" reversible="true" fast="false" fbc:lowerFluxBound="rev_lb"
fbc:upperFluxBound="rev_ub">
            <listOfReactants>
                  <speciesReference species="M_nadp_c" stoichiometry="1"
constant="true"/>
                  <speciesReference species="M_thdp_c" stoichiometry="1"
constant="true"/>
            </listOfReactants>
            <listOfProducts>
                  <speciesReference species="M_h_c" stoichiometry="1"
constant="true"/>
                  <speciesReference species="M_nadph_c" stoichiometry="1"
constant="true"/>
                  <speciesReference species="M_23dhdp_c" stoichiometry="1"
constant="true"/>
            </listOfProducts>
            <fbc:geneProductAssociation>
                  <fbc:geneProductRef fbc:geneProduct="SAUSA300_1289"/>
            </fbc:geneProductAssociation>
      </reaction>

      <reaction metaid="R_DHDPS" id="R_DHDPS" name="dihydrodipicolinate
synthase" reversible="false" fast="false" fbc:lowerFluxBound="irr_lb"
fbc:upperFluxBound="irr_ub">
            <listOfReactants>
                  <speciesReference species="M_pyr_c" stoichiometry="1"
constant="true"/>
                  <speciesReference species="M_aspsa_c" stoichiometry="1"
constant="true"/>
            </listOfReactants>
            <listOfProducts>
                  <speciesReference species="M_h_c" stoichiometry="1"
constant="true"/>
                  <speciesReference species="M_h2o_c" stoichiometry="2"
constant="true"/>
                  <speciesReference species="M_23dhdp_c" stoichiometry="1"
constant="true"/>
            </listOfProducts>
            <fbc:geneProductAssociation>
                  <fbc:geneProductRef fbc:geneProduct="SAUSA300_1288"/>
            </fbc:geneProductAssociation>
      </reaction>
```

<reaction metaid="R_DHFR" id="R_DHFR" name="dihydrofolate reductase" reversible="true" fast="false" fbc:lowerFluxBound="rev_lb" fbc:upperFluxBound="rev_ub">
<listOfReactants>
<speciesReference species="M_nadp_c" stoichiometry="1" constant="true"/>
<speciesReference species="M_thf_c" stoichiometry="1" constant="true"/>
</listOfReactants>
<listOfProducts>
<speciesReference species="M_h_c" stoichiometry="1" constant="true"/>
<speciesReference species="M_nadph_c" stoichiometry="1" constant="true"/>
<speciesReference species="M_dhf_c" stoichiometry="1" constant="true"/>
</listOfProducts>
<fbc:geneProductAssociation>
<fbc:geneProductRef fbc:geneProduct="SAUSA300_1319"/>
</fbc:geneProductAssociation>
</reaction>

<reaction metaid="R_DHFS" id="R_DHFS" name="dihydrofolate synthase" reversible="false" fast="false" fbc:lowerFluxBound="irr_lb" fbc:upperFluxBound="irr_ub">
<listOfReactants>
<speciesReference species="M_glu__L_c" stoichiometry="1" constant="true"/>
<speciesReference species="M_atp_c" stoichiometry="1" constant="true"/>
<speciesReference species="M_dhpt_c" stoichiometry="1" constant="true"/>
</listOfReactants>
<listOfProducts>
<speciesReference species="M_h_c" stoichiometry="1" constant="true"/>
<speciesReference species="M_pi_c" stoichiometry="1" constant="true"/>
<speciesReference species="M_adp_c" stoichiometry="1" constant="true"/>
<speciesReference species="M_dhf_c" stoichiometry="1" constant="true"/>
</listOfProducts>
<fbc:geneProductAssociation>
<fbc:geneProductRef fbc:geneProduct="SAUSA300_1610"/>
</fbc:geneProductAssociation>
</reaction>

<reaction metaid="R_DHNAOT" id="R_DHNAOT" name="1 4__Dihydroxy-2-naphthoate octaprenyltransferase" reversible="false" fast="false" fbc:lowerFluxBound="irr_lb" fbc:upperFluxBound="irr_ub">
<listOfReactants>
<speciesReference species="M_dhna_c" stoichiometry="1" constant="true"/>
<speciesReference species="M_hepdp_c" stoichiometry="1" constant="true"/>
</listOfReactants>
<listOfProducts>

```
                  <speciesReference species="M_2dmmq7_c" stoichiometry="1"
constant="true"/>
                  <speciesReference species="M_co2_c" stoichiometry="1"
constant="true"/>
                  <speciesReference species="M_h_c" stoichiometry="1"
constant="true"/>
                  <speciesReference species="M_ppi_c" stoichiometry="1"
constant="true"/>
            </listOfProducts>
            <fbc:geneProductAssociation>
                  <fbc:geneProductRef fbc:geneProduct="SAUSA300_0944"/>
            </fbc:geneProductAssociation>
      </reaction>

      <reaction metaid="R_DHNCOAT" id="R_DHNCOAT" name="1,4__Dihydroxy-2-
napthoyl-CoA thioesterase" reversible="false" fast="false"
fbc:lowerFluxBound="irr_lb" fbc:upperFluxBound="irr_ub">
            <listOfReactants>
                  <speciesReference species="M_h2o_c" stoichiometry="1"
constant="true"/>
                  <speciesReference species="M_14dhncoa_c" stoichiometry="1"
constant="true"/>
            </listOfReactants>
            <listOfProducts>
                  <speciesReference species="M_h_c" stoichiometry="1"
constant="true"/>
                  <speciesReference species="M_coa_c" stoichiometry="1"
constant="true"/>
                  <speciesReference species="M_dhna_c" stoichiometry="1"
constant="true"/>
            </listOfProducts>
            <fbc:geneProductAssociation>
                  <fbc:or>
                        <fbc:geneProductRef fbc:geneProduct="SAUSA300_1247"/>
                        <fbc:geneProductRef fbc:geneProduct="SAUSA300_2475"/>
                  </fbc:or>
            </fbc:geneProductAssociation>
      </reaction>

      <reaction metaid="R_DHNOAT" id="R_DHNOAT" name="1 4__Dihydroxy-2-
naphthoate octaprenyltransferase " reversible="false" fast="false"
fbc:lowerFluxBound="irr_lb" fbc:upperFluxBound="irr_ub">
            <listOfReactants>
                  <speciesReference species="M_h_c" stoichiometry="1"
constant="true"/>
                  <speciesReference species="M_dhna_c" stoichiometry="1"
constant="true"/>
                  <speciesReference species="M_octdp_c" stoichiometry="1"
constant="true"/>
            </listOfReactants>
            <listOfProducts>
                  <speciesReference species="M_ppi_c" stoichiometry="1"
constant="true"/>
                  <speciesReference species="M_co2_c" stoichiometry="1"
constant="true"/>
            </listOfProducts>
            <fbc:geneProductAssociation>
                  <fbc:geneProductRef fbc:geneProduct="SAUSA300_0944"/>
```

```
            &lt;/fbc:geneProductAssociation&gt;
      &lt;/reaction&gt;

      <reaction metaid="R_DHNPA2" id="R_DHNPA2" name="dihydroneopterin
aldolase" reversible="true" fast="false" fbc:lowerFluxBound="rev_lb"
fbc:upperFluxBound="rev_ub">
            &lt;listOfReactants&gt;
                  <speciesReference species="M_dhnpt_c" stoichiometry="1"
constant="true"/>
            &lt;/listOfReactants&gt;
            &lt;listOfProducts&gt;
                  <speciesReference species="M_gcald_c" stoichiometry="1"
constant="true"/>
                  <speciesReference species="M_6hmhpt_c" stoichiometry="1"
constant="true"/>
            &lt;/listOfProducts&gt;
            &lt;fbc:geneProductAssociation&gt;
                  &lt;fbc:geneProductRef fbc:geneProduct="SAUSA300_0493"/&gt;
            &lt;/fbc:geneProductAssociation&gt;
      &lt;/reaction&gt;

      <reaction metaid="R_DHORD" id="R_DHORD" name="dihydoorotic acid
dehydrogenase" reversible="false" fast="false" fbc:lowerFluxBound="irr_lb"
fbc:upperFluxBound="irr_ub">
            &lt;listOfReactants&gt;
                  <speciesReference species="M_o2_c" stoichiometry="1"
constant="true"/>
                  <speciesReference species="M_dhor__S_c" stoichiometry="1"
constant="true"/>
            &lt;/listOfReactants&gt;
            &lt;listOfProducts&gt;
                  <speciesReference species="M_h2o2_c" stoichiometry="1"
constant="true"/>
                  <speciesReference species="M_orot_c" stoichiometry="1"
constant="true"/>
            &lt;/listOfProducts&gt;
            &lt;fbc:geneProductAssociation&gt;
                  &lt;fbc:geneProductRef fbc:geneProduct="SAUSA300_2526"/&gt;
            &lt;/fbc:geneProductAssociation&gt;
      &lt;/reaction&gt;

      <reaction metaid="R_DHORD5" id="R_DHORD5" name="dihydroorotic acid
(menaquinone-8)" reversible="false" fast="false" fbc:lowerFluxBound="irr_lb"
fbc:upperFluxBound="irr_ub">
            &lt;listOfReactants&gt;
                  <speciesReference species="M_mqn8_c" stoichiometry="1"
constant="true"/>
                  <speciesReference species="M_dhor__S_c" stoichiometry="1"
constant="true"/>
            &lt;/listOfReactants&gt;
            &lt;listOfProducts&gt;
                  <speciesReference species="M_orot_c" stoichiometry="1"
constant="true"/>
                  <speciesReference species="M_mql8_c" stoichiometry="1"
constant="true"/>
            &lt;/listOfProducts&gt;
            &lt;fbc:geneProductAssociation&gt;
                  &lt;fbc:geneProductRef fbc:geneProduct="SAUSA300_2526"/&gt;
```

```
            </fbc:geneProductAssociation>
      </reaction>

      <reaction metaid="R_DHORTS" id="R_DHORTS" name="dihydroorotase"
reversible="true" fast="false" fbc:lowerFluxBound="rev_lb"
fbc:upperFluxBound="rev_ub">
            <listOfReactants>
                  <speciesReference species="M_h2o_c" stoichiometry="1"
constant="true"/>
                  <speciesReference species="M_dhor__S_c" stoichiometry="1"
constant="true"/>
            </listOfReactants>
            <listOfProducts>
                  <speciesReference species="M_h_c" stoichiometry="1"
constant="true"/>
                  <speciesReference species="M_cbasp_c" stoichiometry="1"
constant="true"/>
            </listOfProducts>
            <fbc:geneProductAssociation>
                  <fbc:geneProductRef fbc:geneProduct="SAUSA300_1094"/>
            </fbc:geneProductAssociation>
      </reaction>

      <reaction metaid="R_DHPPDA" id="R_DHPPDA"
name="diaminohydroxyphosphoribosylaminopyrimidine deaminase"
reversible="false" fast="false" fbc:lowerFluxBound="irr_lb"
fbc:upperFluxBound="irr_ub">
            <listOfReactants>
                  <speciesReference species="M_h_c" stoichiometry="1"
constant="true"/>
                  <speciesReference species="M_h2o_c" stoichiometry="1"
constant="true"/>
                  <speciesReference species="M_25dhpp_c" stoichiometry="1"
constant="true"/>
            </listOfReactants>
            <listOfProducts>
                  <speciesReference species="M_nh4_c" stoichiometry="1"
constant="true"/>
                  <speciesReference species="M_5apru_c" stoichiometry="1"
constant="true"/>
            </listOfProducts>
            <fbc:geneProductAssociation>
                  <fbc:geneProductRef fbc:geneProduct="SAUSA300_1715"/>
            </fbc:geneProductAssociation>
      </reaction>

      <reaction metaid="R_DHPS2" id="R_DHPS2" name="dihydropteroate synthase"
reversible="false" fast="false" fbc:lowerFluxBound="irr_lb"
fbc:upperFluxBound="irr_ub">
            <listOfReactants>
                  <speciesReference species="M_6hmhptpp_c" stoichiometry="1"
constant="true"/>
                  <speciesReference species="M_4abz_c" stoichiometry="1"
constant="true"/>
            </listOfReactants>
            <listOfProducts>
                  <speciesReference species="M_ppi_c" stoichiometry="1"
constant="true"/>
```

```
                  <speciesReference species="M_dhpt_c" stoichiometry="1"
constant="true"/>
            </listOfProducts>
            <fbc:geneProductAssociation>
                  <fbc:geneProductRef fbc:geneProduct="SAUSA300_0492"/>
            </fbc:geneProductAssociation>
      </reaction>

      <reaction metaid="R_DHQD" id="R_DHQD" name="3__Dehydroquinate
dehydratase" reversible="true" fast="false" fbc:lowerFluxBound="rev_lb"
fbc:upperFluxBound="rev_ub">
            <listOfReactants>
                  <speciesReference species="M_3dhq_c" stoichiometry="1"
constant="true"/>
            </listOfReactants>
            <listOfProducts>
                  <speciesReference species="M_h2o_c" stoichiometry="1"
constant="true"/>
                  <speciesReference species="M_3dhsk_c" stoichiometry="1"
constant="true"/>
            </listOfProducts>
            <fbc:geneProductAssociation>
                  <fbc:geneProductRef fbc:geneProduct="SAUSA300_0787"/>
            </fbc:geneProductAssociation>
      </reaction>

      <reaction metaid="R_DHQS" id="R_DHQS" name="3__Dehydroquinate synthase"
reversible="false" fast="false" fbc:lowerFluxBound="irr_lb"
fbc:upperFluxBound="irr_ub">
            <listOfReactants>
                  <speciesReference species="M_2dda7p_c" stoichiometry="1"
constant="true"/>
            </listOfReactants>
            <listOfProducts>
                  <speciesReference species="M_h_c" stoichiometry="1"
constant="true"/>
                  <speciesReference species="M_pi_c" stoichiometry="1"
constant="true"/>
                  <speciesReference species="M_3dhq_c" stoichiometry="1"
constant="true"/>
            </listOfProducts>
            <fbc:geneProductAssociation>
                  <fbc:geneProductRef fbc:geneProduct="SAUSA300_1356"/>
            </fbc:geneProductAssociation>
      </reaction>

      <reaction metaid="R_DHSQSAT" id="R_DHSQSAT" name="dehydrosqualene
saturase" reversible="false" fast="false" fbc:lowerFluxBound="irr_lb"
fbc:upperFluxBound="irr_ub">
            <listOfReactants>
                  <speciesReference species="M_fad_c" stoichiometry="1"
constant="true"/>
                  <speciesReference species="M_dhsq_c" stoichiometry="1"
constant="true"/>
            </listOfReactants>
            <listOfProducts>
                  <speciesReference species="M_fadh2_c" stoichiometry="1"
constant="true"/>
```

```
                  <speciesReference species="M_dphytol_c" stoichiometry="1"
constant="true"/>
            </listOfProducts>
            <fbc:geneProductAssociation>
                  <fbc:geneProductRef fbc:geneProduct="SAUSA300_2498"/>
            </fbc:geneProductAssociation>
      </reaction>

      <reaction metaid="R_DHSQSYN" id="R_DHSQSYN" name="dehydrosqualene
synthase" reversible="false" fast="false" fbc:lowerFluxBound="irr_lb"
fbc:upperFluxBound="irr_ub">
            <listOfReactants>
                  <speciesReference species="M_psdp_c" stoichiometry="1"
constant="true"/>
            </listOfReactants>
            <listOfProducts>
                  <speciesReference species="M_ppi_c" stoichiometry="1"
constant="true"/>
                  <speciesReference species="M_dhsq_c" stoichiometry="1"
constant="true"/>
            </listOfProducts>
            <fbc:geneProductAssociation>
                  <fbc:geneProductRef fbc:geneProduct="SAUSA300_2499"/>
            </fbc:geneProductAssociation>
      </reaction>

      <reaction metaid="R_DINSt2e" id="R_DINSt2e" name="deoxyinosine
transport in via proton symport " reversible="true" fast="false"
fbc:lowerFluxBound="rev_lb" fbc:upperFluxBound="rev_ub">
            <listOfReactants>
                  <speciesReference species="M_h_e" stoichiometry="1"
constant="true"/>
                  <speciesReference species="M_din_e" stoichiometry="1"
constant="true"/>
            </listOfReactants>
            <listOfProducts>
                  <speciesReference species="M_h_c" stoichiometry="1"
constant="true"/>
                  <speciesReference species="M_din_c" stoichiometry="1"
constant="true"/>
            </listOfProducts>
            <fbc:geneProductAssociation>
                  <fbc:geneProductRef fbc:geneProduct="SAUSA300_0506"/>
            </fbc:geneProductAssociation>
      </reaction>

      <reaction metaid="R_DKMPPD2" id="R_DKMPPD2" name="2,3__Diketo-5-
methylthio-1-phosphopentane degradation reaction" reversible="false"
fast="false" fbc:lowerFluxBound="irr_lb" fbc:upperFluxBound="irr_ub">
            <listOfReactants>
                  <speciesReference species="M_h2o_c" stoichiometry="3"
constant="true"/>
                  <speciesReference species="M_dkmpp_c" stoichiometry="1"
constant="true"/>
            </listOfReactants>
            <listOfProducts>
                  <speciesReference species="M_h_c" stoichiometry="6"
constant="true"/>
```

```
                  <speciesReference species="M_pi_c" stoichiometry="1"
constant="true"/>
                  <speciesReference species="M_for_c" stoichiometry="1"
constant="true"/>
                  <speciesReference species="M_2kmb_c" stoichiometry="1"
constant="true"/>
            </listOfProducts>
            <fbc:geneProductAssociation>
                  <fbc:geneProductRef fbc:geneProduct="SAUSA300_0557"/>
            </fbc:geneProductAssociation>
      </reaction>

      <reaction metaid="R_DMATT" id="R_DMATT"
name="dimethylallyltranstransferase" reversible="false" fast="false"
fbc:lowerFluxBound="irr_lb" fbc:upperFluxBound="irr_ub">
            <listOfReactants>
                  <speciesReference species="M_ipdp_c" stoichiometry="1"
constant="true"/>
                  <speciesReference species="M_dmpp_c" stoichiometry="1"
constant="true"/>
            </listOfReactants>
            <listOfProducts>
                  <speciesReference species="M_ppi_c" stoichiometry="1"
constant="true"/>
                  <speciesReference species="M_grdp_c" stoichiometry="1"
constant="true"/>
            </listOfProducts>
            <fbc:geneProductAssociation>
                  <fbc:geneProductRef fbc:geneProduct="SAUSA300_1470"/>
            </fbc:geneProductAssociation>
      </reaction>

      <reaction metaid="R_DMPHMT" id="R_DMPHMT" name="rxn04674 "
reversible="false" fast="false" fbc:lowerFluxBound="irr_lb"
fbc:upperFluxBound="irr_ub">
            <listOfReactants>
                  <speciesReference species="M_amet_c" stoichiometry="1"
constant="true"/>
                  <speciesReference species="M_23dmphol_c" stoichiometry="1"
constant="true"/>
            </listOfReactants>
            <listOfProducts>
                  <speciesReference species="M_h_c" stoichiometry="1"
constant="true"/>
                  <speciesReference species="M_ahcys_c" stoichiometry="1"
constant="true"/>
                  <speciesReference species="M_phyQ_c" stoichiometry="1"
constant="true"/>
            </listOfProducts>
            <fbc:geneProductAssociation>
                  <fbc:geneProductRef fbc:geneProduct="SAUSA300_1360"/>
            </fbc:geneProductAssociation>
      </reaction>

      <reaction metaid="R_DNMPPA" id="R_DNMPPA" name="Dihydroneopterin
monophosphate dephosphorylase" reversible="false" fast="false"
fbc:lowerFluxBound="irr_lb" fbc:upperFluxBound="irr_ub">
            <listOfReactants>
```

```
                  <speciesReference species="M_h2o_c" stoichiometry="1"
constant="true"/>
                  <speciesReference species="M_dhpmp_c" stoichiometry="1"
constant="true"/>
            </listOfReactants>
            <listOfProducts>
                  <speciesReference species="M_pi_c" stoichiometry="1"
constant="true"/>
                  <speciesReference species="M_dhnpt_c" stoichiometry="1"
constant="true"/>
            </listOfProducts>
            <fbc:geneProductAssociation>
                  <fbc:geneProductRef fbc:geneProduct="SAUSA300_1371"/>
            </fbc:geneProductAssociation>
      </reaction>

      <reaction metaid="R_DNSPADH" id="R_DNSPADH"
name="4,4'__Diaponeurosporen-aldehyde dehydrogenase" reversible="false"
fast="false" fbc:lowerFluxBound="irr_lb" fbc:upperFluxBound="irr_ub">
            <listOfReactants>
                  <speciesReference species="M_h2o_c" stoichiometry="1"
constant="true"/>
                  <speciesReference species="M_nad_c" stoichiometry="1"
constant="true"/>
                  <speciesReference species="M_dnspal_c" stoichiometry="1"
constant="true"/>
            </listOfReactants>
            <listOfProducts>
                  <speciesReference species="M_h_c" stoichiometry="2"
constant="true"/>
                  <speciesReference species="M_nadh_c" stoichiometry="1"
constant="true"/>
                  <speciesReference species="M_dnspate_c" stoichiometry="1"
constant="true"/>
            </listOfProducts>
            <fbc:geneProductAssociation>
                  <fbc:geneProductRef fbc:geneProduct="SAUSA300_1901"/>
            </fbc:geneProductAssociation>
      </reaction>

      <reaction metaid="R_DNSPGT" id="R_DNSPGT"
name="4,4'__Diaponeurosporenoate glycosyltransferase" reversible="false"
fast="false" fbc:lowerFluxBound="irr_lb" fbc:upperFluxBound="irr_ub">
            <listOfReactants>
                  <speciesReference species="M_udpg_c" stoichiometry="1"
constant="true"/>
                  <speciesReference species="M_dnspate_c" stoichiometry="1"
constant="true"/>
            </listOfReactants>
            <listOfProducts>
                  <speciesReference species="M_udp_c" stoichiometry="1"
constant="true"/>
                  <speciesReference species="M_gdnspate_c" stoichiometry="1"
constant="true"/>
            </listOfProducts>
            <fbc:geneProductAssociation>
                  <fbc:or>
                        <fbc:geneProductRef fbc:geneProduct="SAUSA300_2500"/>
```

```
                        <fbc:geneProductRef fbc:geneProduct="SAUSA300_2583"/>
                  </fbc:or>
            </fbc:geneProductAssociation>
      </reaction>

      <reaction metaid="R_DNSPO" id="R_DNSPO" name="diaponeurosporene
oxidase" reversible="false" fast="false" fbc:lowerFluxBound="irr_lb"
fbc:upperFluxBound="irr_ub">
            <listOfReactants>
                  <speciesReference species="M_o2_c" stoichiometry="1"
constant="true"/>
                  <speciesReference species="M_dnspen_c" stoichiometry="1"
constant="true"/>
            </listOfReactants>
            <listOfProducts>
                  <speciesReference species="M_h2o_c" stoichiometry="1"
constant="true"/>
                  <speciesReference species="M_dnspal_c" stoichiometry="1"
constant="true"/>
            </listOfProducts>
            <fbc:geneProductAssociation>
                  <fbc:geneProductRef fbc:geneProduct="SAUSA300_2501"/>
            </fbc:geneProductAssociation>
      </reaction>

      <reaction metaid="R_DNTPPA" id="R_DNTPPA" name="Dihydroneopterin
triphosphate pyrophosphatase" reversible="false" fast="false"
fbc:lowerFluxBound="irr_lb" fbc:upperFluxBound="irr_ub">
            <listOfReactants>
                  <speciesReference species="M_h2o_c" stoichiometry="1"
constant="true"/>
                  <speciesReference species="M_ahdt_c" stoichiometry="1"
constant="true"/>
            </listOfReactants>
            <listOfProducts>
                  <speciesReference species="M_h_c" stoichiometry="1"
constant="true"/>
                  <speciesReference species="M_ppi_c" stoichiometry="1"
constant="true"/>
                  <speciesReference species="M_dhpmp_c" stoichiometry="1"
constant="true"/>
            </listOfProducts>
            <fbc:geneProductAssociation>
                  <fbc:geneProductRef fbc:geneProduct="SAUSA300_1371"/>
            </fbc:geneProductAssociation>
      </reaction>

      <reaction metaid="R_DPCOAK" id="R_DPCOAK" name="dephospho-CoA kinase"
reversible="false" fast="false" fbc:lowerFluxBound="irr_lb"
fbc:upperFluxBound="irr_ub">
            <listOfReactants>
                  <speciesReference species="M_atp_c" stoichiometry="1"
constant="true"/>
                  <speciesReference species="M_dpcoa_c" stoichiometry="1"
constant="true"/>
            </listOfReactants>
            <listOfProducts>
```

```
                  <speciesReference species="M_adp_c" stoichiometry="1"
constant="true"/>
                  <speciesReference species="M_coa_c" stoichiometry="1"
constant="true"/>
            </listOfProducts>
            <fbc:geneProductAssociation>
                  <fbc:geneProductRef fbc:geneProduct="SAUSA300_1634"/>
            </fbc:geneProductAssociation>
      </reaction>

      <reaction metaid="R_DPHYTOLS" id="R_DPHYTOLS"
name="4,4'__Diapophytofluene desaturase" reversible="false" fast="false"
fbc:lowerFluxBound="irr_lb" fbc:upperFluxBound="irr_ub">
            <listOfReactants>
                  <speciesReference species="M_fad_c" stoichiometry="1"
constant="true"/>
                  <speciesReference species="M_dphytol_c" stoichiometry="1"
constant="true"/>
            </listOfReactants>
            <listOfProducts>
                  <speciesReference species="M_fadh2_c" stoichiometry="1"
constant="true"/>
                  <speciesReference species="M_thdcar_c" stoichiometry="1"
constant="true"/>
            </listOfProducts>
            <fbc:geneProductAssociation>
                  <fbc:geneProductRef fbc:geneProduct="SAUSA300_2498"/>
            </fbc:geneProductAssociation>
      </reaction>

      <reaction metaid="R_DPMVD" id="R_DPMVD" name="diphosphomevalonate
decarboxylase" reversible="false" fast="false" fbc:lowerFluxBound="irr_lb"
fbc:upperFluxBound="irr_ub">
            <listOfReactants>
                  <speciesReference species="M_atp_c" stoichiometry="1"
constant="true"/>
                  <speciesReference species="M_5dpmev_c" stoichiometry="1"
constant="true"/>
            </listOfReactants>
            <listOfProducts>
                  <speciesReference species="M_pi_c" stoichiometry="1"
constant="true"/>
                  <speciesReference species="M_adp_c" stoichiometry="1"
constant="true"/>
                  <speciesReference species="M_co2_c" stoichiometry="1"
constant="true"/>
                  <speciesReference species="M_ipdp_c" stoichiometry="1"
constant="true"/>
            </listOfProducts>
            <fbc:geneProductAssociation>
                  <fbc:geneProductRef fbc:geneProduct="SAUSA300_0573"/>
            </fbc:geneProductAssociation>
      </reaction>

      <reaction metaid="R_DPR" id="R_DPR" name="2__Dehydropantoate 2-
reductase" reversible="true" fast="false" fbc:lowerFluxBound="rev_lb"
fbc:upperFluxBound="rev_ub">
            <listOfReactants>
```

```
                  <speciesReference species="M_nadp_c" stoichiometry="1"
constant="true"/>
                  <speciesReference species="M_pant__R_c" stoichiometry="1"
constant="true"/>
            </listOfReactants>
            <listOfProducts>
                  <speciesReference species="M_h_c" stoichiometry="1"
constant="true"/>
                  <speciesReference species="M_nadph_c" stoichiometry="1"
constant="true"/>
                  <speciesReference species="M_2dhp_c" stoichiometry="1"
constant="true"/>
            </listOfProducts>
            <fbc:geneProductAssociation>
                  <fbc:or>
                        <fbc:geneProductRef fbc:geneProduct="SAUSA300_2009"/>
                        <fbc:geneProductRef fbc:geneProduct="SAUSA300_2388"/>
                        <fbc:geneProductRef fbc:geneProduct="SAUSA300_2535"/>
                  </fbc:or>
            </fbc:geneProductAssociation>
      </reaction>

      <reaction metaid="R_DR1ORy" id="R_DR1ORy" name="D-Ribitol-5-phosphate
NADP 2-oxidoreductase " reversible="true" fast="false"
fbc:lowerFluxBound="rev_lb" fbc:upperFluxBound="rev_ub">
            <listOfReactants>
                  <speciesReference species="M_nadp_c" stoichiometry="1"
constant="true"/>
                  <speciesReference species="M_dr5p" stoichiometry="1"
constant="true"/>
            </listOfReactants>
            <listOfProducts>
                  <speciesReference species="M_h_c" stoichiometry="1"
constant="true"/>
                  <speciesReference species="M_nadph_c" stoichiometry="1"
constant="true"/>
                  <speciesReference species="M_ru5p__D_c" stoichiometry="1"
constant="true"/>
            </listOfProducts>
            <fbc:geneProductAssociation>
                  <fbc:geneProductRef fbc:geneProduct="SAUSA300_0250"/>
            </fbc:geneProductAssociation>
      </reaction>

      <reaction metaid="R_DRBabc" id="R_DRBabc" name="Deoxyribose transport
via ABC system " reversible="false" fast="false" fbc:lowerFluxBound="irr_lb"
fbc:upperFluxBound="irr_ub">
            <listOfReactants>
                  <speciesReference species="M_h2o_c" stoichiometry="1"
constant="true"/>
                  <speciesReference species="M_atp_c" stoichiometry="1"
constant="true"/>
                  <speciesReference species="M_drib_e" stoichiometry="1"
constant="true"/>
            </listOfReactants>
            <listOfProducts>
                  <speciesReference species="M_h_c" stoichiometry="1"
constant="true"/>
```

```
                  <speciesReference species="M_pi_c" stoichiometry="1"
constant="true"/>
                  <speciesReference species="M_adp_c" stoichiometry="1"
constant="true"/>
                  <speciesReference species="M_drib_c" stoichiometry="1"
constant="true"/>
            &lt;/listOfProducts&gt;
            &lt;fbc:geneProductAssociation&gt;
                  &lt;fbc:geneProductRef fbc:geneProduct="SAUSA300_0263"/&gt;
            &lt;/fbc:geneProductAssociation&gt;
      &lt;/reaction&gt;

      <reaction metaid="R_DRBK" id="R_DRBK" name="Deoxyribokinase"
reversible="true" fast="false" fbc:lowerFluxBound="rev_lb"
fbc:upperFluxBound="rev_ub">
            &lt;listOfReactants&gt;
                  <speciesReference species="M_adp_c" stoichiometry="1"
constant="true"/>
                  <speciesReference species="M_2dr5p_c" stoichiometry="1"
constant="true"/>
            &lt;/listOfReactants&gt;
            &lt;listOfProducts&gt;
                  <speciesReference species="M_atp_c" stoichiometry="1"
constant="true"/>
                  <speciesReference species="M_drib_c" stoichiometry="1"
constant="true"/>
            &lt;/listOfProducts&gt;
            &lt;fbc:geneProductAssociation&gt;
                  &lt;fbc:geneProductRef fbc:geneProduct="SAUSA300_0262"/&gt;
            &lt;/fbc:geneProductAssociation&gt;
      &lt;/reaction&gt;

      <reaction metaid="R_DROPPRy" id="R_DROPPRy" name="2,5__Diamino-6-
ribosylamino-4(3H)-pyrimidinone 5'-phosphate reductase (nadph)"
reversible="false" fast="false" fbc:lowerFluxBound="irr_lb"
fbc:upperFluxBound="irr_ub">
            &lt;listOfReactants&gt;
                  <speciesReference species="M_h_c" stoichiometry="1"
constant="true"/>
                  <speciesReference species="M_nadph_c" stoichiometry="1"
constant="true"/>
                  <speciesReference species="M_25dhpp_c" stoichiometry="1"
constant="true"/>
            &lt;/listOfReactants&gt;
            &lt;listOfProducts&gt;
                  <speciesReference species="M_nadp_c" stoichiometry="1"
constant="true"/>
                  <speciesReference species="M_25dthpp_c" stoichiometry="1"
constant="true"/>
            &lt;/listOfProducts&gt;
            &lt;fbc:geneProductAssociation&gt;
                  &lt;fbc:geneProductRef fbc:geneProduct="SAUSA300_1715"/&gt;
            &lt;/fbc:geneProductAssociation&gt;
      &lt;/reaction&gt;

      <reaction metaid="R_DRPA" id="R_DRPA" name="deoxyribose-phosphate
aldolase" reversible="false" fast="false" fbc:lowerFluxBound="irr_lb"
fbc:upperFluxBound="irr_ub">
```

```
            <listOfReactants>
                  <speciesReference species="M_2dr5p_c" stoichiometry="1"
constant="true"/>
            </listOfReactants>
            <listOfProducts>
                  <speciesReference species="M_acald_c" stoichiometry="1"
constant="true"/>
                  <speciesReference species="M_g3p_c" stoichiometry="1"
constant="true"/>
            </listOfProducts>
            <fbc:geneProductAssociation>
                  <fbc:or>
                        <fbc:geneProductRef fbc:geneProduct="SAUSA300_0140"/>
                        <fbc:geneProductRef fbc:geneProduct="SAUSA300_2090"/>
                  </fbc:or>
            </fbc:geneProductAssociation>
      </reaction>

      <reaction metaid="R_DRPAr" id="R_DRPAr" name="deoxyribose-phosphate
aldolase reversible" reversible="true" fast="false"
fbc:lowerFluxBound="rev_lb" fbc:upperFluxBound="rev_ub">
            <listOfReactants>
                  <speciesReference species="M_2dr5p_c" stoichiometry="1"
constant="true"/>
            </listOfReactants>
            <listOfProducts>
                  <speciesReference species="M_acald_c" stoichiometry="1"
constant="true"/>
                  <speciesReference species="M_g3p_c" stoichiometry="1"
constant="true"/>
            </listOfProducts>
            <fbc:geneProductAssociation>
                  <fbc:or>
                        <fbc:geneProductRef fbc:geneProduct="SAUSA300_0140"/>
                        <fbc:geneProductRef fbc:geneProduct="SAUSA300_2090"/>
                  </fbc:or>
            </fbc:geneProductAssociation>
      </reaction>

      <reaction metaid="R_DRTPPD" id="R_DRTPPD" name="2,5__Diamino-6-
ribitylamino-4(3H)-pyrimidinone 5'-phosphate deaminase" reversible="false"
fast="false" fbc:lowerFluxBound="irr_lb" fbc:upperFluxBound="irr_ub">
            <listOfReactants>
                  <speciesReference species="M_h_c" stoichiometry="1"
constant="true"/>
                  <speciesReference species="M_h2o_c" stoichiometry="1"
constant="true"/>
                  <speciesReference species="M_25dthpp_c" stoichiometry="1"
constant="true"/>
            </listOfReactants>
            <listOfProducts>
                  <speciesReference species="M_nh4_c" stoichiometry="1"
constant="true"/>
                  <speciesReference species="M_5aprbu_c" stoichiometry="1"
constant="true"/>
            </listOfProducts>
            <fbc:geneProductAssociation>
                  <fbc:geneProductRef fbc:geneProduct="SAUSA300_0874"/>
```

```
            </fbc:geneProductAssociation>
      </reaction>

      <reaction metaid="R_DSERt2" id="R_DSERt2" name="D-serine transport via
proton symport" reversible="true" fast="false" fbc:lowerFluxBound="rev_lb"
fbc:upperFluxBound="rev_ub">
            <listOfReactants>
                  <speciesReference species="M_h_e" stoichiometry="1"
constant="true"/>
                  <speciesReference species="M_ser__D_e" stoichiometry="1"
constant="true"/>
            </listOfReactants>
            <listOfProducts>
                  <speciesReference species="M_h_c" stoichiometry="1"
constant="true"/>
                  <speciesReference species="M_ser__D_c" stoichiometry="1"
constant="true"/>
            </listOfProducts>
            <fbc:geneProductAssociation>
                  <fbc:or>
                        <fbc:geneProductRef fbc:geneProduct="SAUSA300_1642"/>
                        <fbc:geneProductRef fbc:geneProduct="SA451515_2543"/>
                        <fbc:geneProductRef fbc:geneProduct="SAUSA300_0712"/>
                  </fbc:or>
            </fbc:geneProductAssociation>
      </reaction>

      <reaction metaid="R_DTMPK" id="R_DTMPK" name="dTMP kinase"
reversible="true" fast="false" fbc:lowerFluxBound="rev_lb"
fbc:upperFluxBound="rev_ub">
            <listOfReactants>
                  <speciesReference species="M_atp_c" stoichiometry="1"
constant="true"/>
                  <speciesReference species="M_dtmp_c" stoichiometry="1"
constant="true"/>
            </listOfReactants>
            <listOfProducts>
                  <speciesReference species="M_adp_c" stoichiometry="1"
constant="true"/>
                  <speciesReference species="M_dtdp_c" stoichiometry="1"
constant="true"/>
            </listOfProducts>
            <fbc:geneProductAssociation>
                  <fbc:geneProductRef fbc:geneProduct="SAUSA300_0459"/>
            </fbc:geneProductAssociation>
      </reaction>

      <reaction metaid="R_DTUPP" id="R_DTUPP" name="dTTP uridine 5'-
phosphotransferase " reversible="false" fast="false"
fbc:lowerFluxBound="irr_lb" fbc:upperFluxBound="irr_ub">
            <listOfReactants>
                  <speciesReference species="M_uri_c" stoichiometry="1"
constant="true"/>
                  <speciesReference species="M_dttp_c" stoichiometry="1"
constant="true"/>
            </listOfReactants>
            <listOfProducts>
```

```
                  <speciesReference species="M_ump_c" stoichiometry="1"
constant="true"/>
                  <speciesReference species="M_dtdp_c" stoichiometry="1"
constant="true"/>
            </listOfProducts>
            <fbc:geneProductAssociation>
                  <fbc:geneProductRef fbc:geneProduct="SAUSA300_1568"/>
            </fbc:geneProductAssociation>
      </reaction>

      <reaction metaid="R_DUCYTP" id="R_DUCYTP" name="dUTP cytidine 5'-
phosphotransferase " reversible="false" fast="false"
fbc:lowerFluxBound="irr_lb" fbc:upperFluxBound="irr_ub">
            <listOfReactants>
                  <speciesReference species="M_dutp_c" stoichiometry="1"
constant="true"/>
                  <speciesReference species="M_cytd_c" stoichiometry="1"
constant="true"/>
            </listOfReactants>
            <listOfProducts>
                  <speciesReference species="M_cmp_c" stoichiometry="1"
constant="true"/>
                  <speciesReference species="M_dudp_c" stoichiometry="1"
constant="true"/>
            </listOfProducts>
            <fbc:geneProductAssociation>
                  <fbc:geneProductRef fbc:geneProduct="SAUSA300_1568"/>
            </fbc:geneProductAssociation>
      </reaction>

      <reaction metaid="R_DURIK1" id="R_DURIK1" name="deoxyuridine kinase
(ATP:Deoxyuridine)" reversible="false" fast="false"
fbc:lowerFluxBound="irr_lb" fbc:upperFluxBound="irr_ub">
            <listOfReactants>
                  <speciesReference species="M_atp_c" stoichiometry="1"
constant="true"/>
                  <speciesReference species="M_duri_c" stoichiometry="1"
constant="true"/>
            </listOfReactants>
            <listOfProducts>
                  <speciesReference species="M_adp_c" stoichiometry="1"
constant="true"/>
                  <speciesReference species="M_dump_c" stoichiometry="1"
constant="true"/>
            </listOfProducts>
            <fbc:geneProductAssociation>
                  <fbc:geneProductRef fbc:geneProduct="SAUSA300_2073"/>
            </fbc:geneProductAssociation>
      </reaction>

      <reaction metaid="R_DURIPP" id="R_DURIPP" name="deoxyuridine
phosphorylase" reversible="true" fast="false" fbc:lowerFluxBound="rev_lb"
fbc:upperFluxBound="rev_ub">
            <listOfReactants>
                  <speciesReference species="M_h_c" stoichiometry="1"
constant="true"/>
                  <speciesReference species="M_pi_c" stoichiometry="1"
constant="true"/>
```

```
                  <speciesReference species="M_duri_c" stoichiometry="1"
constant="true"/>
            </listOfReactants>
            <listOfProducts>
                  <speciesReference species="M_2dr1p_c" stoichiometry="1"
constant="true"/>
                  <speciesReference species="M_ura_c" stoichiometry="1"
constant="true"/>
            </listOfProducts>
            <fbc:geneProductAssociation>
                  <fbc:or>
                        <fbc:geneProductRef fbc:geneProduct="SAUSA300_2089"/>
                        <fbc:geneProductRef fbc:geneProduct="SAUSA300_0138"/>
                        <fbc:geneProductRef fbc:geneProduct="SAUSA300_2091"/>
                  </fbc:or>
            </fbc:geneProductAssociation>
      </reaction>

      <reaction metaid="R_DURIt2r" id="R_DURIt2r" name="deoxyuridine
transport in via proton symport " reversible="true" fast="false"
fbc:lowerFluxBound="rev_lb" fbc:upperFluxBound="rev_ub">
            <listOfReactants>
                  <speciesReference species="M_h_e" stoichiometry="1"
constant="true"/>
                  <speciesReference species="M_duri_e" stoichiometry="1"
constant="true"/>
            </listOfReactants>
            <listOfProducts>
                  <speciesReference species="M_h_c" stoichiometry="1"
constant="true"/>
                  <speciesReference species="M_duri_c" stoichiometry="1"
constant="true"/>
            </listOfProducts>
            <fbc:geneProductAssociation>
                  <fbc:geneProductRef fbc:geneProduct="SAUSA300_0506"/>
            </fbc:geneProductAssociation>
      </reaction>

      <reaction metaid="R_DUTPDP" id="R_DUTPDP" name="dUTP diphosphatase"
reversible="false" fast="false" fbc:lowerFluxBound="irr_lb"
fbc:upperFluxBound="irr_ub">
            <listOfReactants>
                  <speciesReference species="M_h2o_c" stoichiometry="1"
constant="true"/>
                  <speciesReference species="M_dutp_c" stoichiometry="1"
constant="true"/>
            </listOfReactants>
            <listOfProducts>
                  <speciesReference species="M_ppi_c" stoichiometry="1"
constant="true"/>
                  <speciesReference species="M_dump_c" stoichiometry="1"
constant="true"/>
            </listOfProducts>
            <fbc:geneProductAssociation>
                  <fbc:geneProductRef fbc:geneProduct="SAUSA300_1949"/>
            </fbc:geneProductAssociation>
      </reaction>
```

```
      <reaction metaid="R_DUUPP" id="R_DUUPP" name="dUTP uridine 5'-
phosphotransferase " reversible="false" fast="false"
fbc:lowerFluxBound="irr_lb" fbc:upperFluxBound="irr_ub">
            <listOfReactants>
                  <speciesReference species="M_dutp_c" stoichiometry="1"
constant="true"/>
                  <speciesReference species="M_uri_c" stoichiometry="1"
constant="true"/>
            </listOfReactants>
            <listOfProducts>
                  <speciesReference species="M_dudp_c" stoichiometry="1"
constant="true"/>
                  <speciesReference species="M_ump_c" stoichiometry="1"
constant="true"/>
            </listOfProducts>
            <fbc:geneProductAssociation>
                  <fbc:geneProductRef fbc:geneProduct="SAUSA300_1568"/>
            </fbc:geneProductAssociation>
      </reaction>

      <reaction metaid="R_E4PD" id="R_E4PD" name="Erythrose 4-phosphate
dehydrogenase" reversible="true" fast="false" fbc:lowerFluxBound="rev_lb"
fbc:upperFluxBound="rev_ub">
            <listOfReactants>
                  <speciesReference species="M_h2o_c" stoichiometry="1"
constant="true"/>
                  <speciesReference species="M_nad_c" stoichiometry="1"
constant="true"/>
                  <speciesReference species="M_e4p_c" stoichiometry="1"
constant="true"/>
            </listOfReactants>
            <listOfProducts>
                  <speciesReference species="M_h_c" stoichiometry="2"
constant="true"/>
                  <speciesReference species="M_nadh_c" stoichiometry="1"
constant="true"/>
                  <speciesReference species="M_4per_c" stoichiometry="1"
constant="true"/>
            </listOfProducts>
            <fbc:geneProductAssociation>
                  <fbc:geneProductRef fbc:geneProduct="SAUSA300_0756"/>
            </fbc:geneProductAssociation>
      </reaction>

      <reaction metaid="R_EAR100x" id="R_EAR100x" name="enoyl-[acyl-carrier-
protein] reductase (NADH) (n-C10:0)" reversible="false" fast="false"
fbc:lowerFluxBound="irr_lb" fbc:upperFluxBound="irr_ub">
            <listOfReactants>
                  <speciesReference species="M_h_c" stoichiometry="1"
constant="true"/>
                  <speciesReference species="M_nadh_c" stoichiometry="1"
constant="true"/>
                  <speciesReference species="M_tdec2eACP_c" stoichiometry="1"
constant="true"/>
            </listOfReactants>
            <listOfProducts>
                  <speciesReference species="M_nad_c" stoichiometry="1"
constant="true"/>
```

```
                  <speciesReference species="M_dcaACP_c" stoichiometry="1"
constant="true"/>
            </listOfProducts>
            <fbc:geneProductAssociation>
                  <fbc:geneProductRef fbc:geneProduct="SAUSA300_0912"/>
            </fbc:geneProductAssociation>
      </reaction>

      <reaction metaid="R_EAR120x" id="R_EAR120x" name="enoyl-[acyl-carrier-
protein] reductase (NADH) (n-C12:0)" reversible="false" fast="false"
fbc:lowerFluxBound="irr_lb" fbc:upperFluxBound="irr_ub">
            <listOfReactants>
                  <speciesReference species="M_h_c" stoichiometry="1"
constant="true"/>
                  <speciesReference species="M_nadh_c" stoichiometry="1"
constant="true"/>
                  <speciesReference species="M_tddec2eACP_c"
stoichiometry="1" constant="true"/>
            </listOfReactants>
            <listOfProducts>
                  <speciesReference species="M_nad_c" stoichiometry="1"
constant="true"/>
                  <speciesReference species="M_ddcaACP_c" stoichiometry="1"
constant="true"/>
            </listOfProducts>
            <fbc:geneProductAssociation>
                  <fbc:geneProductRef fbc:geneProduct="SAUSA300_0912"/>
            </fbc:geneProductAssociation>
      </reaction>

      <reaction metaid="R_EAR140x" id="R_EAR140x" name="enoyl-[acyl-carrier-
protein] reductase (NADH) (n-C14:0)" reversible="false" fast="false"
fbc:lowerFluxBound="irr_lb" fbc:upperFluxBound="irr_ub">
            <listOfReactants>
                  <speciesReference species="M_h_c" stoichiometry="1"
constant="true"/>
                  <speciesReference species="M_nadh_c" stoichiometry="1"
constant="true"/>
                  <speciesReference species="M_tmrs2eACP_c" stoichiometry="1"
constant="true"/>
            </listOfReactants>
            <listOfProducts>
                  <speciesReference species="M_nad_c" stoichiometry="1"
constant="true"/>
                  <speciesReference species="M_myrsACP_c" stoichiometry="1"
constant="true"/>
            </listOfProducts>
            <fbc:geneProductAssociation>
                  <fbc:geneProductRef fbc:geneProduct="SAUSA300_0912"/>
            </fbc:geneProductAssociation>
      </reaction>

      <reaction metaid="R_EAR40x" id="R_EAR40x" name="enoyl-[acyl-carrier-
protein] reductase (NADH) (n-C4:0)" reversible="false" fast="false"
fbc:lowerFluxBound="irr_lb" fbc:upperFluxBound="irr_ub">
            <listOfReactants>
                  <speciesReference species="M_h_c" stoichiometry="1"
constant="true"/>
```

```
                <speciesReference species="M_nadh_c" stoichiometry="1"
constant="true"/>
                <speciesReference species="M_but2eACP_c" stoichiometry="1"
constant="true"/>
            </listOfReactants>
            <listOfProducts>
                <speciesReference species="M_nad_c" stoichiometry="1"
constant="true"/>
                <speciesReference species="M_butACP_c" stoichiometry="1"
constant="true"/>
            </listOfProducts>
            <fbc:geneProductAssociation>
                <fbc:geneProductRef fbc:geneProduct="SAUSA300_0912"/>
            </fbc:geneProductAssociation>
        </reaction>

        <reaction metaid="R_EAR60x" id="R_EAR60x" name="enoyl-[acyl-carrier-
protein] reductase (NADH) (n-C6:0)" reversible="false" fast="false"
fbc:lowerFluxBound="irr_lb" fbc:upperFluxBound="irr_ub">
            <listOfReactants>
                <speciesReference species="M_h_c" stoichiometry="1"
constant="true"/>
                <speciesReference species="M_nadh_c" stoichiometry="1"
constant="true"/>
                <speciesReference species="M_thex2eACP_c" stoichiometry="1"
constant="true"/>
            </listOfReactants>
            <listOfProducts>
                <speciesReference species="M_nad_c" stoichiometry="1"
constant="true"/>
                <speciesReference species="M_hexACP_c" stoichiometry="1"
constant="true"/>
            </listOfProducts>
            <fbc:geneProductAssociation>
                <fbc:geneProductRef fbc:geneProduct="SAUSA300_0912"/>
            </fbc:geneProductAssociation>
        </reaction>

        <reaction metaid="R_EAR80x" id="R_EAR80x" name="enoyl-[acyl-carrier-
protein] reductase (NADH) (n-C8:0)" reversible="false" fast="false"
fbc:lowerFluxBound="irr_lb" fbc:upperFluxBound="irr_ub">
            <listOfReactants>
                <speciesReference species="M_h_c" stoichiometry="1"
constant="true"/>
                <speciesReference species="M_nadh_c" stoichiometry="1"
constant="true"/>
                <speciesReference species="M_toct2eACP_c" stoichiometry="1"
constant="true"/>
            </listOfReactants>
            <listOfProducts>
                <speciesReference species="M_nad_c" stoichiometry="1"
constant="true"/>
                <speciesReference species="M_ocACP_c" stoichiometry="1"
constant="true"/>
            </listOfProducts>
            <fbc:geneProductAssociation>
                <fbc:geneProductRef fbc:geneProduct="SAUSA300_0912"/>
            </fbc:geneProductAssociation>
```

```
      </reaction>

      <reaction metaid="R_ECOAH1" id="R_ECOAH1" name="3-hydroxyacyl-CoA
dehydratase (3-hydroxybutanoyl-CoA)" reversible="true" fast="false"
fbc:lowerFluxBound="rev_lb" fbc:upperFluxBound="rev_ub">
            <listOfReactants>
                  <speciesReference species="M_3hbcoa_c" stoichiometry="1"
constant="true"/>
            </listOfReactants>
            <listOfProducts>
                  <speciesReference species="M_h2o_c" stoichiometry="1"
constant="true"/>
                  <speciesReference species="M_b2coa_c" stoichiometry="1"
constant="true"/>
            </listOfProducts>
            <fbc:geneProductAssociation>
                  <fbc:geneProductRef fbc:geneProduct="SAUSA300_0226"/>
            </fbc:geneProductAssociation>
      </reaction>

      <reaction metaid="R_ECOAH3" id="R_ECOAH3" name="3-hydroxyacyl-CoA
dehydratase (3-hydroxyoctanoyl-CoA)" reversible="true" fast="false"
fbc:lowerFluxBound="rev_lb" fbc:upperFluxBound="rev_ub">
            <listOfReactants>
                  <speciesReference species="M_3hocoa_c" stoichiometry="1"
constant="true"/>
            </listOfReactants>
            <listOfProducts>
                  <speciesReference species="M_h2o_c" stoichiometry="1"
constant="true"/>
                  <speciesReference species="M_oc2coa_c" stoichiometry="1"
constant="true"/>
            </listOfProducts>
            <fbc:geneProductAssociation>
                  <fbc:geneProductRef fbc:geneProduct="SAUSA300_2054"/>
            </fbc:geneProductAssociation>
      </reaction>

      <reaction metaid="R_ECOAH7" id="R_ECOAH7" name="3-hydroxyacyl-CoA
dehydratase (3-hydroxyhexadecanoyl-CoA)" reversible="true" fast="false"
fbc:lowerFluxBound="rev_lb" fbc:upperFluxBound="rev_ub">
            <listOfReactants>
                  <speciesReference species="M_3hhdcoa_c" stoichiometry="1"
constant="true"/>
            </listOfReactants>
            <listOfProducts>
                  <speciesReference species="M_h2o_c" stoichiometry="1"
constant="true"/>
                  <speciesReference species="M_hdd2coa_c" stoichiometry="1"
constant="true"/>
            </listOfProducts>
            <fbc:geneProductAssociation>
                  <fbc:geneProductRef fbc:geneProduct="SAUSA300_2054"/>
            </fbc:geneProductAssociation>
      </reaction>
```

```
	<reaction metaid="R_EDA" id="R_EDA" name="2__Dehydro-3__Deoxy-
phosphogluconate aldolase" reversible="false" fast="false"
fbc:lowerFluxBound="irr_lb" fbc:upperFluxBound="irr_ub">
		<listOfReactants>
			<speciesReference species="M_2ddg6p_c" stoichiometry="1"
constant="true"/>
		</listOfReactants>
		<listOfProducts>
			<speciesReference species="M_pyr_c" stoichiometry="1"
constant="true"/>
			<speciesReference species="M_g3p_c" stoichiometry="1"
constant="true"/>
		</listOfProducts>
		<fbc:geneProductAssociation>
			<fbc:geneProductRef fbc:geneProduct="SAUSA300_0124"/>
		</fbc:geneProductAssociation>
	</reaction>

	<reaction metaid="R_EHGLAT" id="R_EHGLAT" name="L-erythro-4-
Hydroxyglutamate:2-oxoglutarate aminotransferase" reversible="true"
fast="false" fbc:lowerFluxBound="rev_lb" fbc:upperFluxBound="rev_ub">
		<listOfReactants>
			<speciesReference species="M_akg_c" stoichiometry="1"
constant="true"/>
			<speciesReference species="M_e4hglu_c" stoichiometry="1"
constant="true"/>
		</listOfReactants>
		<listOfProducts>
			<speciesReference species="M_glu__L_c" stoichiometry="1"
constant="true"/>
			<speciesReference species="M_4h2oglt_c" stoichiometry="1"
constant="true"/>
		</listOfProducts>
		<fbc:geneProductAssociation>
			<fbc:geneProductRef fbc:geneProduct="SAUSA300_1916"/>
		</fbc:geneProductAssociation>
	</reaction>

	<reaction metaid="R_ENO" id="R_ENO" name="enolase" reversible="true"
fast="false" fbc:lowerFluxBound="rev_lb" fbc:upperFluxBound="rev_ub">
		<listOfReactants>
			<speciesReference species="M_2pg_c" stoichiometry="1"
constant="true"/>
		</listOfReactants>
		<listOfProducts>
			<speciesReference species="M_h2o_c" stoichiometry="1"
constant="true"/>
			<speciesReference species="M_pep_c" stoichiometry="1"
constant="true"/>
		</listOfProducts>
		<fbc:geneProductAssociation>
			<fbc:geneProductRef fbc:geneProduct="SAUSA300_0760"/>
		</fbc:geneProductAssociation>
	</reaction>

	<reaction metaid="R_ETHA_Et" id="R_ETHA_Et" name="Ethanolamine
transport " reversible="true" fast="false" fbc:lowerFluxBound="rev_lb"
fbc:upperFluxBound="rev_ub">
```

```
            <listOfReactants>
                  <speciesReference species="M_etha_e" stoichiometry="1"
constant="true"/>
            </listOfReactants>
            <listOfProducts>
                  <speciesReference species="M_etha_c" stoichiometry="1"
constant="true"/>
            </listOfProducts>
      </reaction>

      <reaction metaid="R_ETOHt" id="R_ETOHt" name="ethanol reversible
transport" reversible="true" fast="false" fbc:lowerFluxBound="rev_lb"
fbc:upperFluxBound="rev_ub">
            <listOfReactants>
                  <speciesReference species="M_etoh_e" stoichiometry="1"
constant="true"/>
            </listOfReactants>
            <listOfProducts>
                  <speciesReference species="M_etoh_c" stoichiometry="1"
constant="true"/>
            </listOfProducts>
      </reaction>

      <reaction metaid="EX_12ppd__R_e" id="EX_12ppd__R_e" name="(R)-Propane-
1,2__Diol Exchange" reversible="true" fast="false"
fbc:lowerFluxBound="rev_lb" fbc:upperFluxBound="rev_ub">
            <listOfReactants>
                  <speciesReference species="M_12ppd__R_e" stoichiometry="1"
constant="true"/>
            </listOfReactants>
      </reaction>

      <reaction metaid="EX_12ppd__S_e" id="EX_12ppd__S_e" name="(S)-Propane-
1,2__Diol Exchange" reversible="true" fast="false"
fbc:lowerFluxBound="rev_lb" fbc:upperFluxBound="rev_ub">
            <listOfReactants>
                  <speciesReference species="M_12ppd__S_e" stoichiometry="1"
constant="true"/>
            </listOfReactants>
      </reaction>

      <reaction metaid="EX_23ccmp_e" id="EX_23ccmp_e" name="2',3'-cyclic CMP
Exchange" reversible="true" fast="false" fbc:lowerFluxBound="rev_lb"
fbc:upperFluxBound="rev_ub">
            <listOfReactants>
                  <speciesReference species="M_23ccmp_e" stoichiometry="1"
constant="true"/>
            </listOfReactants>
      </reaction>

      <reaction metaid="EX_26dap_M_e" id="EX_26dap_M_e" name="26dap_M
Exchange" reversible="true" fast="false" fbc:lowerFluxBound="rev_lb"
fbc:upperFluxBound="rev_ub">
            <listOfReactants>
                  <speciesReference species="M_26dap__M_e" stoichiometry="1"
constant="true"/>
            </listOfReactants>
      </reaction>
```

```
      <reaction metaid="EX_2bdgsglyc_e" id="EX_2bdgsglyc_e" name="2-beta__D-
Glucosyl-sn-glycerol Exchange" reversible="true" fast="false"
fbc:lowerFluxBound="rev_lb" fbc:upperFluxBound="rev_ub">
            &lt;listOfReactants&gt;
                  <speciesReference species="M_2bdgsglyc_e" stoichiometry="1"
constant="true"/>
            &lt;/listOfReactants&gt;
      &lt;/reaction&gt;

      <reaction metaid="EX_2obut_e" id="EX_2obut_e" name="2-oxobutanoate
Exchange" reversible="true" fast="false" fbc:lowerFluxBound="rev_lb"
fbc:upperFluxBound="rev_ub">
            &lt;listOfReactants&gt;
                  <speciesReference species="M_2obut_e" stoichiometry="1"
constant="true"/>
            &lt;/listOfReactants&gt;
      &lt;/reaction&gt;

      <reaction metaid="EX_3amp_e" id="EX_3amp_e" name="3'-AMP Exchange"
reversible="true" fast="false" fbc:lowerFluxBound="rev_lb"
fbc:upperFluxBound="rev_ub">
            &lt;listOfReactants&gt;
                  <speciesReference species="M_3amp_e" stoichiometry="1"
constant="true"/>
            &lt;/listOfReactants&gt;
      &lt;/reaction&gt;

      <reaction metaid="EX_3gmp_e" id="EX_3gmp_e" name="3'-GMP Exchange"
reversible="true" fast="false" fbc:lowerFluxBound="rev_lb"
fbc:upperFluxBound="rev_ub">
            &lt;listOfReactants&gt;
                  <speciesReference species="M_3gmp_e" stoichiometry="1"
constant="true"/>
            &lt;/listOfReactants&gt;
      &lt;/reaction&gt;

      <reaction metaid="EX_4abut_e" id="EX_4abut_e" name="4-Aminobutanoate
Exchange" reversible="true" fast="false" fbc:lowerFluxBound="rev_lb"
fbc:upperFluxBound="rev_ub">
            &lt;listOfReactants&gt;
                  <speciesReference species="M_4abut_e" stoichiometry="1"
constant="true"/>
            &lt;/listOfReactants&gt;
      &lt;/reaction&gt;

      <reaction metaid="EX_4abz_e" id="EX_4abz_e" name="4-Aminobenzoate
Exchange" reversible="true" fast="false" fbc:lowerFluxBound="rev_lb"
fbc:upperFluxBound="rev_ub">
            &lt;listOfReactants&gt;
                  <speciesReference species="M_4abz_e" stoichiometry="1"
constant="true"/>
            &lt;/listOfReactants&gt;
      &lt;/reaction&gt;

      <reaction metaid="EX_4hba_e" id="EX_4hba_e" name="4-Hydroxy-benzyl
alcohol Exchange" reversible="true" fast="false" fbc:lowerFluxBound="rev_lb"
fbc:upperFluxBound="rev_ub">
```

```
            <listOfReactants>
                  <speciesReference species="M_4hba_e" stoichiometry="1"
constant="true"/>
            </listOfReactants>
      </reaction>

      <reaction metaid="EX_4hoxpacd_e" id="EX_4hoxpacd_e" name="4-
Hydroxyphenylacetaldehyde Exchange" reversible="true" fast="false"
fbc:lowerFluxBound="rev_lb" fbc:upperFluxBound="rev_ub">
            <listOfReactants>
                  <speciesReference species="M_4hoxpacd_e" stoichiometry="1"
constant="true"/>
            </listOfReactants>
      </reaction>

      <reaction metaid="EX_5dglcn_e" id="EX_5dglcn_e" name="5__Dehydro__D-
gluconate Exchange" reversible="true" fast="false"
fbc:lowerFluxBound="rev_lb" fbc:upperFluxBound="rev_ub">
            <listOfReactants>
                  <speciesReference species="M_5dglcn_e" stoichiometry="1"
constant="true"/>
            </listOfReactants>
      </reaction>

      <reaction metaid="EX_5mtr_e" id="EX_5mtr_e" name="5-Methylthio__D-
ribose Exchange" reversible="true" fast="false" fbc:lowerFluxBound="rev_lb"
fbc:upperFluxBound="rev_ub">
            <listOfReactants>
                  <speciesReference species="M_5mtr_e" stoichiometry="1"
constant="true"/>
            </listOfReactants>
      </reaction>

      <reaction metaid="EX_abt_e" id="EX_abt_e" name="arabitol Exchange"
reversible="true" fast="false" fbc:lowerFluxBound="rev_lb"
fbc:upperFluxBound="rev_ub">
            <listOfReactants>
                  <speciesReference species="M_abt_e" stoichiometry="1"
constant="true"/>
            </listOfReactants>
      </reaction>

      <reaction metaid="EX_ac_e" id="EX_ac_e" name="Acetate Exchange"
reversible="true" fast="false" fbc:lowerFluxBound="rev_lb"
fbc:upperFluxBound="rev_ub">
            <listOfReactants>
                  <speciesReference species="M_ac_e" stoichiometry="1"
constant="true"/>
            </listOfReactants>
      </reaction>

      <reaction metaid="EX_acac_e" id="EX_acac_e" name="Acetoacetate
Exchange" reversible="true" fast="false" fbc:lowerFluxBound="rev_lb"
fbc:upperFluxBound="rev_ub">
            <listOfReactants>
                  <speciesReference species="M_acac_e" stoichiometry="1"
constant="true"/>
            </listOfReactants>
```

```
      </reaction>

      <reaction metaid="EX_acgal_e" id="EX_acgal_e" name="N-Acetyl__D-
galactosamine Exchange" reversible="true" fast="false"
fbc:lowerFluxBound="rev_lb" fbc:upperFluxBound="rev_ub">
            <listOfReactants>
                  <speciesReference species="M_acgal_e" stoichiometry="1"
constant="true"/>
            </listOfReactants>
      </reaction>

      <reaction metaid="EX_acgam_e" id="EX_acgam_e" name="N-Acetyl__D-
glucosamine Exchange" reversible="true" fast="false"
fbc:lowerFluxBound="rev_lb" fbc:upperFluxBound="rev_ub">
            <listOfReactants>
                  <speciesReference species="M_acgam_e" stoichiometry="1"
constant="true"/>
            </listOfReactants>
      </reaction>

      <reaction metaid="EX_acmana_e" id="EX_acmana_e" name="N-Acetyl__D-
mannosamine Exchange" reversible="true" fast="false"
fbc:lowerFluxBound="rev_lb" fbc:upperFluxBound="rev_ub">
            <listOfReactants>
                  <speciesReference species="M_acmana_e" stoichiometry="1"
constant="true"/>
            </listOfReactants>
      </reaction>

      <reaction metaid="EX_acnam_e" id="EX_acnam_e" name="N-Acetylneuraminate
Exchange" reversible="true" fast="false" fbc:lowerFluxBound="rev_lb"
fbc:upperFluxBound="rev_ub">
            <listOfReactants>
                  <speciesReference species="M_acnam_e" stoichiometry="1"
constant="true"/>
            </listOfReactants>
      </reaction>

      <reaction metaid="EX_actn__R_e" id="EX_actn__R_e" name="acetoin
Exchange" reversible="true" fast="false" fbc:lowerFluxBound="rev_lb"
fbc:upperFluxBound="rev_ub">
            <listOfReactants>
                  <speciesReference species="M_actn__R_e" stoichiometry="1"
constant="true"/>
            </listOfReactants>
      </reaction>

      <reaction metaid="EX_ad_e" id="EX_ad_e" name="acetamide Exchange"
reversible="true" fast="false" fbc:lowerFluxBound="rev_lb"
fbc:upperFluxBound="rev_ub">
            <listOfReactants>
                  <speciesReference species="M_ad_e" stoichiometry="1"
constant="true"/>
            </listOfReactants>
      </reaction>
```

```
      <reaction metaid="EX_ade_e" id="EX_ade_e" name="Adenine Exchange"
reversible="true" fast="false" fbc:lowerFluxBound="rev_lb"
fbc:upperFluxBound="rev_ub">
            &lt;listOfReactants&gt;
                  <speciesReference species="M_ade_e" stoichiometry="1"
constant="true"/>
            &lt;/listOfReactants&gt;
      &lt;/reaction&gt;

      <reaction metaid="EX_adn_e" id="EX_adn_e" name="Adenosine Exchange"
reversible="true" fast="false" fbc:lowerFluxBound="rev_lb"
fbc:upperFluxBound="rev_ub">
            &lt;listOfReactants&gt;
                  <speciesReference species="M_adn_e" stoichiometry="1"
constant="true"/>
            &lt;/listOfReactants&gt;
      &lt;/reaction&gt;

      <reaction metaid="EX_agm_e" id="EX_agm_e" name="Agmatine Exchange"
reversible="true" fast="false" fbc:lowerFluxBound="rev_lb"
fbc:upperFluxBound="rev_ub">
            &lt;listOfReactants&gt;
                  <speciesReference species="M_agm_e" stoichiometry="1"
constant="true"/>
            &lt;/listOfReactants&gt;
      &lt;/reaction&gt;

      <reaction metaid="EX_akg_e" id="EX_akg_e" name="2-Oxoglutarate
Exchange" reversible="true" fast="false" fbc:lowerFluxBound="rev_lb"
fbc:upperFluxBound="rev_ub">
            &lt;listOfReactants&gt;
                  <speciesReference species="M_akg_e" stoichiometry="1"
constant="true"/>
            &lt;/listOfReactants&gt;
      &lt;/reaction&gt;

      <reaction metaid="EX_ala__D_e" id="EX_ala__D_e" name="D-Alanine
Exchange" reversible="true" fast="false" fbc:lowerFluxBound="rev_lb"
fbc:upperFluxBound="rev_ub">
            &lt;listOfReactants&gt;
                  <speciesReference species="M_ala__D_e" stoichiometry="1"
constant="true"/>
            &lt;/listOfReactants&gt;
      &lt;/reaction&gt;

      <reaction metaid="EX_ala__L_e" id="EX_ala__L_e" name="L-Alanine
Exchange" reversible="true" fast="false" fbc:lowerFluxBound="rev_lb"
fbc:upperFluxBound="rev_ub">
            &lt;listOfReactants&gt;
                  <speciesReference species="M_ala__L_e" stoichiometry="1"
constant="true"/>
            &lt;/listOfReactants&gt;
      &lt;/reaction&gt;

      <reaction metaid="EX_alagly_e" id="EX_alagly_e" name="L-alanylglycine
Exchange" reversible="true" fast="false" fbc:lowerFluxBound="rev_lb"
fbc:upperFluxBound="rev_ub">
            &lt;listOfReactants&gt;
```

```
                  <speciesReference species="M_alagly_e" stoichiometry="1"
constant="true"/>
            </listOfReactants>
      </reaction>

      <reaction metaid="EX_all__D_e" id="EX_all__D_e" name="D-Allose
Exchange" reversible="true" fast="false" fbc:lowerFluxBound="rev_lb"
fbc:upperFluxBound="rev_ub">
            <listOfReactants>
                  <speciesReference species="M_all__D_e" stoichiometry="1"
constant="true"/>
            </listOfReactants>
      </reaction>

      <reaction metaid="EX_alltn_e" id="EX_alltn_e" name="Allantoin Exchange"
reversible="true" fast="false" fbc:lowerFluxBound="rev_lb"
fbc:upperFluxBound="rev_ub">
            <listOfReactants>
                  <speciesReference species="M_alltn_e" stoichiometry="1"
constant="true"/>
            </listOfReactants>
      </reaction>

      <reaction metaid="EX_amp_e" id="EX_amp_e" name="AMP Exchange"
reversible="true" fast="false" fbc:lowerFluxBound="rev_lb"
fbc:upperFluxBound="rev_ub">
            <listOfReactants>
                  <speciesReference species="M_amp_e" stoichiometry="1"
constant="true"/>
            </listOfReactants>
      </reaction>

      <reaction metaid="EX_arab__L_e" id="EX_arab__L_e" name="L-Arabinose
Exchange" reversible="true" fast="false" fbc:lowerFluxBound="rev_lb"
fbc:upperFluxBound="rev_ub">
            <listOfReactants>
                  <speciesReference species="M_arab__L_e" stoichiometry="1"
constant="true"/>
            </listOfReactants>
      </reaction>

      <reaction metaid="EX_arbt_e" id="EX_arbt_e" name="Arbutin Exchange"
reversible="true" fast="false" fbc:lowerFluxBound="rev_lb"
fbc:upperFluxBound="rev_ub">
            <listOfReactants>
                  <speciesReference species="M_arbt_e" stoichiometry="1"
constant="true"/>
            </listOfReactants>
      </reaction>

      <reaction metaid="EX_arg__L_e" id="EX_arg__L_e" name="L-Arginine
Exchange" reversible="true" fast="false" fbc:lowerFluxBound="rev_lb"
fbc:upperFluxBound="rev_ub">
            <listOfReactants>
                  <speciesReference species="M_arg__L_e" stoichiometry="1"
constant="true"/>
            </listOfReactants>
      </reaction>
```

```
      <reaction metaid="EX_arsbet_e" id="EX_arsbet_e" name="ARSENOBETAINE
Exchange" reversible="true" fast="false" fbc:lowerFluxBound="rev_lb"
fbc:upperFluxBound="rev_ub">
            &lt;listOfReactants&gt;
                  <speciesReference species="M_arsbet_e" stoichiometry="1"
constant="true"/>
            &lt;/listOfReactants&gt;
      &lt;/reaction&gt;

      <reaction metaid="EX_asn__L_e" id="EX_asn__L_e" name="L-Asparagine
Exchange" reversible="true" fast="false" fbc:lowerFluxBound="rev_lb"
fbc:upperFluxBound="rev_ub">
            &lt;listOfReactants&gt;
                  <speciesReference species="M_asn__L_e" stoichiometry="1"
constant="true"/>
            &lt;/listOfReactants&gt;
      &lt;/reaction&gt;

      <reaction metaid="EX_aso3_e" id="EX_aso3_e" name="arsenite Exchange"
reversible="true" fast="false" fbc:lowerFluxBound="rev_lb"
fbc:upperFluxBound="rev_ub">
            &lt;listOfReactants&gt;
                  <speciesReference species="M_aso3_e" stoichiometry="1"
constant="true"/>
            &lt;/listOfReactants&gt;
      &lt;/reaction&gt;

      <reaction metaid="EX_aso4_e" id="EX_aso4_e" name="arsenate Exchange"
reversible="true" fast="false" fbc:lowerFluxBound="rev_lb"
fbc:upperFluxBound="rev_ub">
            &lt;listOfReactants&gt;
                  <speciesReference species="M_aso4_e" stoichiometry="1"
constant="true"/>
            &lt;/listOfReactants&gt;
      &lt;/reaction&gt;

      <reaction metaid="EX_asp__L_e" id="EX_asp__L_e" name="L-Aspartate
Exchange" reversible="true" fast="false" fbc:lowerFluxBound="rev_lb"
fbc:upperFluxBound="rev_ub">
            &lt;listOfReactants&gt;
                  <speciesReference species="M_asp__L_e" stoichiometry="1"
constant="true"/>
            &lt;/listOfReactants&gt;
      &lt;/reaction&gt;

      <reaction metaid="EX_b12_e" id="EX_b12_e" name="Vitamin_B12 Exchange"
reversible="true" fast="false" fbc:lowerFluxBound="rev_lb"
fbc:upperFluxBound="rev_ub">
            &lt;listOfReactants&gt;
                  <speciesReference species="M_b12_e" stoichiometry="1"
constant="true"/>
            &lt;/listOfReactants&gt;
      &lt;/reaction&gt;

      <reaction metaid="EX_btbet_e" id="EX_btbet_e" name="Butyro-betaine
Exchange" reversible="true" fast="false" fbc:lowerFluxBound="rev_lb"
fbc:upperFluxBound="rev_ub">
```

```
            <listOfReactants>
                  <speciesReference species="M_btbet_e" stoichiometry="1"
constant="true"/>
            </listOfReactants>
      </reaction>

      <reaction metaid="EX_btn_e" id="EX_btn_e" name="Biotin Exchange"
reversible="true" fast="false" fbc:lowerFluxBound="rev_lb"
fbc:upperFluxBound="rev_ub">
            <listOfReactants>
                  <speciesReference species="M_btn_e" stoichiometry="1"
constant="true"/>
            </listOfReactants>
      </reaction>

      <reaction metaid="EX_but_e" id="EX_but_e" name="Butyrate (n-C4:0)
Exchange" reversible="true" fast="false" fbc:lowerFluxBound="rev_lb"
fbc:upperFluxBound="rev_ub">
            <listOfReactants>
                  <speciesReference species="M_but_e" stoichiometry="1"
constant="true"/>
            </listOfReactants>
      </reaction>

      <reaction metaid="EX_butso3_e" id="EX_butso3_e" name="butanesulfonate
Exchange" reversible="true" fast="false" fbc:lowerFluxBound="rev_lb"
fbc:upperFluxBound="rev_ub">
            <listOfReactants>
                  <speciesReference species="M_butso3_e" stoichiometry="1"
constant="true"/>
            </listOfReactants>
      </reaction>

      <reaction metaid="EX_ca2_e" id="EX_ca2_e" name="Calcium Exchange"
reversible="true" fast="false" fbc:lowerFluxBound="rev_lb"
fbc:upperFluxBound="rev_ub">
            <listOfReactants>
                  <speciesReference species="M_ca2_e" stoichiometry="1"
constant="true"/>
            </listOfReactants>
      </reaction>

      <reaction metaid="EX_cbl1_e" id="EX_cbl1_e" name="Cob(I)alamin
Exchange" reversible="true" fast="false" fbc:lowerFluxBound="rev_lb"
fbc:upperFluxBound="rev_ub">
            <listOfReactants>
                  <speciesReference species="M_cbl1_e" stoichiometry="1"
constant="true"/>
            </listOfReactants>
      </reaction>

      <reaction metaid="EX_cbl2_e" id="EX_cbl2_e" name="cob(II)alamin
Exchange" reversible="true" fast="false" fbc:lowerFluxBound="rev_lb"
fbc:upperFluxBound="rev_ub">
            <listOfReactants>
                  <speciesReference species="M_cbl2_e" stoichiometry="1"
constant="true"/>
            </listOfReactants>
```

```
      </reaction>

      <reaction metaid="EX_cd2_e" id="EX_cd2_e" name="Cadmium Exchange"
reversible="true" fast="false" fbc:lowerFluxBound="rev_lb"
fbc:upperFluxBound="rev_ub">
            <listOfReactants>
                  <speciesReference species="M_cd2_e" stoichiometry="1"
constant="true"/>
            </listOfReactants>
      </reaction>

      <reaction metaid="EX_cgly_e" id="EX_cgly_e" name="Cys-Gly Exchange"
reversible="true" fast="false" fbc:lowerFluxBound="rev_lb"
fbc:upperFluxBound="rev_ub">
            <listOfReactants>
                  <speciesReference species="M_cgly_e" stoichiometry="1"
constant="true"/>
            </listOfReactants>
      </reaction>

      <reaction metaid="EX_chol_e" id="EX_chol_e" name="Choline Exchange"
reversible="true" fast="false" fbc:lowerFluxBound="rev_lb"
fbc:upperFluxBound="rev_ub">
            <listOfReactants>
                  <speciesReference species="M_chol_e" stoichiometry="1"
constant="true"/>
            </listOfReactants>
      </reaction>

      <reaction metaid="EX_chols_e" id="EX_chols_e" name="Choline sulfate
Exchange" reversible="true" fast="false" fbc:lowerFluxBound="rev_lb"
fbc:upperFluxBound="rev_ub">
            <listOfReactants>
                  <speciesReference species="M_chols_e" stoichiometry="1"
constant="true"/>
            </listOfReactants>
      </reaction>

      <reaction metaid="EX_Cit__Mg_e" id="EX_Cit__Mg_e" name="Citrate-Mg
Exchange" reversible="true" fast="false" fbc:lowerFluxBound="rev_lb"
fbc:upperFluxBound="rev_ub">
            <listOfReactants>
                  <speciesReference species="M_Cit__Mg_e" stoichiometry="1"
constant="true"/>
            </listOfReactants>
      </reaction>

      <reaction metaid="EX_cit_e" id="EX_cit_e" name="Citrate Exchange"
reversible="true" fast="false" fbc:lowerFluxBound="rev_lb"
fbc:upperFluxBound="rev_ub">
            <listOfReactants>
                  <speciesReference species="M_cit_e" stoichiometry="1"
constant="true"/>
            </listOfReactants>
      </reaction>
```

```
      <reaction metaid="EX_citr__L_e" id="EX_citr__L_e" name="L-Citrulline
Exchange" reversible="true" fast="false" fbc:lowerFluxBound="rev_lb"
fbc:upperFluxBound="rev_ub">
            <listOfReactants>
                  <speciesReference species="M_citr__L_e" stoichiometry="1"
constant="true"/>
            </listOfReactants>
      </reaction>

      <reaction metaid="EX_cl_e" id="EX_cl_e" name="Chloride Exchange"
reversible="true" fast="false" fbc:lowerFluxBound="rev_lb"
fbc:upperFluxBound="rev_ub">
            <listOfReactants>
                  <speciesReference species="M_cl_e" stoichiometry="1"
constant="true"/>
            </listOfReactants>
      </reaction>

      <reaction metaid="EX_co2_e" id="EX_co2_e" name="CO2 Exchange"
reversible="true" fast="false" fbc:lowerFluxBound="rev_lb"
fbc:upperFluxBound="rev_ub">
            <listOfReactants>
                  <speciesReference species="M_co2_e" stoichiometry="1"
constant="true"/>
            </listOfReactants>
      </reaction>

      <reaction metaid="EX_cobalt2_e" id="EX_cobalt2_e" name="Co2+ Exchange"
reversible="true" fast="false" fbc:lowerFluxBound="rev_lb"
fbc:upperFluxBound="rev_ub">
            <listOfReactants>
                  <speciesReference species="M_cobalt2_e" stoichiometry="1"
constant="true"/>
            </listOfReactants>
      </reaction>

      <reaction metaid="EX_crn_e" id="EX_crn_e" name="L-Carnitine Exchange"
reversible="true" fast="false" fbc:lowerFluxBound="rev_lb"
fbc:upperFluxBound="rev_ub">
            <listOfReactants>
                  <speciesReference species="M_crn_e" stoichiometry="1"
constant="true"/>
            </listOfReactants>
      </reaction>

      <reaction metaid="EX_csn_e" id="EX_csn_e" name="Cytosine Exchange"
reversible="true" fast="false" fbc:lowerFluxBound="rev_lb"
fbc:upperFluxBound="rev_ub">
            <listOfReactants>
                  <speciesReference species="M_csn_e" stoichiometry="1"
constant="true"/>
            </listOfReactants>
      </reaction>

      <reaction metaid="EX_ctbt_e" id="EX_ctbt_e" name="crotono-betaine
Exchange" reversible="true" fast="false" fbc:lowerFluxBound="rev_lb"
fbc:upperFluxBound="rev_ub">
            <listOfReactants>
```

```
                  <speciesReference species="M_ctbt_e" stoichiometry="1"
constant="true"/>
            </listOfReactants>
      </reaction>

      <reaction metaid="EX_cu2_e" id="EX_cu2_e" name="Cu2+ Exchange"
reversible="true" fast="false" fbc:lowerFluxBound="rev_lb"
fbc:upperFluxBound="rev_ub">
            <listOfReactants>
                  <speciesReference species="M_cu2_e" stoichiometry="1"
constant="true"/>
            </listOfReactants>
      </reaction>

      <reaction metaid="EX_cys__D_e" id="EX_cys__D_e" name="D-Cysteine
Exchange" reversible="true" fast="false" fbc:lowerFluxBound="rev_lb"
fbc:upperFluxBound="rev_ub">
            <listOfReactants>
                  <speciesReference species="M_cys__D_e" stoichiometry="1"
constant="true"/>
            </listOfReactants>
      </reaction>

      <reaction metaid="EX_cys__L_e" id="EX_cys__L_e" name="L-Cysteine
Exchange" reversible="true" fast="false" fbc:lowerFluxBound="rev_lb"
fbc:upperFluxBound="rev_ub">
            <listOfReactants>
                  <speciesReference species="M_cys__L_e" stoichiometry="1"
constant="true"/>
            </listOfReactants>
      </reaction>

      <reaction metaid="EX_cytd_e" id="EX_cytd_e" name="Cytidine Exchange"
reversible="true" fast="false" fbc:lowerFluxBound="rev_lb"
fbc:upperFluxBound="rev_ub">
            <listOfReactants>
                  <speciesReference species="M_cytd_e" stoichiometry="1"
constant="true"/>
            </listOfReactants>
      </reaction>

      <reaction metaid="EX_dad__2_e" id="EX_dad__2_e" name="Deoxyadenosine
Exchange" reversible="true" fast="false" fbc:lowerFluxBound="rev_lb"
fbc:upperFluxBound="rev_ub">
            <listOfReactants>
                  <speciesReference species="M_dad__2_e" stoichiometry="1"
constant="true"/>
            </listOfReactants>
      </reaction>

      <reaction metaid="EX_dca_e" id="EX_dca_e" name="Decanoate (n-C10:0)
Exchange" reversible="true" fast="false" fbc:lowerFluxBound="rev_lb"
fbc:upperFluxBound="rev_ub">
            <listOfReactants>
                  <speciesReference species="M_dca_e" stoichiometry="1"
constant="true"/>
            </listOfReactants>
      </reaction>
```

```
      <reaction metaid="EX_dha_e" id="EX_dha_e" name="Dihydroxyacetone
Exchange" reversible="true" fast="false" fbc:lowerFluxBound="rev_lb"
fbc:upperFluxBound="rev_ub">
            <listOfReactants>
                  <speciesReference species="M_dha_e" stoichiometry="1"
constant="true"/>
            </listOfReactants>
      </reaction>

      <reaction metaid="EX_dhap_e" id="EX_dhap_e" name="dhap Exchange"
reversible="true" fast="false" fbc:lowerFluxBound="rev_lb"
fbc:upperFluxBound="rev_ub">
            <listOfReactants>
                  <speciesReference species="M_dhap_e" stoichiometry="1"
constant="true"/>
            </listOfReactants>
      </reaction>

      <reaction metaid="EX_dhptd_c" id="EX_dhptd_c" name="4,5__Dihydroxy-2,3-
pentanedione Exchange" reversible="true" fast="false"
fbc:lowerFluxBound="rev_lb" fbc:upperFluxBound="rev_ub">
            <listOfReactants>
                  <speciesReference species="M_dhptd_c" stoichiometry="1"
constant="true"/>
            </listOfReactants>
      </reaction>

      <reaction metaid="EX_drib_e" id="EX_drib_e" name="Deoxyribose Exchange"
reversible="true" fast="false" fbc:lowerFluxBound="rev_lb"
fbc:upperFluxBound="rev_ub">
            <listOfReactants>
                  <speciesReference species="M_drib_e" stoichiometry="1"
constant="true"/>
            </listOfReactants>
      </reaction>

      <reaction metaid="EX_etha_e" id="EX_etha_e" name="Ethanolamine
Exchange" reversible="true" fast="false" fbc:lowerFluxBound="rev_lb"
fbc:upperFluxBound="rev_ub">
            <listOfReactants>
                  <speciesReference species="M_etha_e" stoichiometry="1"
constant="true"/>
            </listOfReactants>
      </reaction>

      <reaction metaid="EX_etoh_e" id="EX_etoh_e" name="Ethanol Exchange"
reversible="true" fast="false" fbc:lowerFluxBound="rev_lb"
fbc:upperFluxBound="rev_ub">
            <listOfReactants>
                  <speciesReference species="M_etoh_e" stoichiometry="1"
constant="true"/>
            </listOfReactants>
      </reaction>

      <reaction metaid="EX_f6p_e" id="EX_f6p_e" name="D-Fructose 6-phosphate
Exchange" reversible="true" fast="false" fbc:lowerFluxBound="rev_lb"
fbc:upperFluxBound="rev_ub">
```

```
            &lt;listOfReactants&gt;
                  <speciesReference species="M_f6p_e" stoichiometry="1"
constant="true"/>
            &lt;/listOfReactants&gt;
      &lt;/reaction&gt;

      <reaction metaid="EX_fe2_e" id="EX_fe2_e" name="Fe2+ Exchange"
reversible="true" fast="false" fbc:lowerFluxBound="rev_lb"
fbc:upperFluxBound="rev_ub">
            &lt;listOfReactants&gt;
                  <speciesReference species="M_fe2_e" stoichiometry="1"
constant="true"/>
            &lt;/listOfReactants&gt;
      &lt;/reaction&gt;

      <reaction metaid="EX_fe3_e" id="EX_fe3_e" name="Fe3+ Exchange"
reversible="true" fast="false" fbc:lowerFluxBound="rev_lb"
fbc:upperFluxBound="rev_ub">
            &lt;listOfReactants&gt;
                  <speciesReference species="M_fe3_e" stoichiometry="1"
constant="true"/>
            &lt;/listOfReactants&gt;
      &lt;/reaction&gt;

      <reaction metaid="EX_fe3dcit_e" id="EX_fe3dcit_e"
name="Fe(III)dicitrate Exchange" reversible="true" fast="false"
fbc:lowerFluxBound="rev_lb" fbc:upperFluxBound="rev_ub">
            &lt;listOfReactants&gt;
                  <speciesReference species="M_fe3dcit_e" stoichiometry="1"
constant="true"/>
            &lt;/listOfReactants&gt;
      &lt;/reaction&gt;

      <reaction metaid="EX_fecrm_e" id="EX_fecrm_e" name="Ferrichrome
Exchange" reversible="true" fast="false" fbc:lowerFluxBound="rev_lb"
fbc:upperFluxBound="rev_ub">
            &lt;listOfReactants&gt;
                  <speciesReference species="M_fecrm_e" stoichiometry="1"
constant="true"/>
            &lt;/listOfReactants&gt;
      &lt;/reaction&gt;

      <reaction metaid="EX_for_e" id="EX_for_e" name="Formate Exchange"
reversible="true" fast="false" fbc:lowerFluxBound="rev_lb"
fbc:upperFluxBound="rev_ub">
            &lt;listOfReactants&gt;
                  <speciesReference species="M_for_e" stoichiometry="1"
constant="true"/>
            &lt;/listOfReactants&gt;
      &lt;/reaction&gt;

      <reaction metaid="EX_fru_e" id="EX_fru_e" name="D-Fructose Exchange"
reversible="true" fast="false" fbc:lowerFluxBound="rev_lb"
fbc:upperFluxBound="rev_ub">
            &lt;listOfReactants&gt;
                  <speciesReference species="M_fru_e" stoichiometry="1"
constant="true"/>
            &lt;/listOfReactants&gt;
```

```
      </reaction>

      <reaction metaid="EX_fuc__L_e" id="EX_fuc__L_e" name="L-Fucose
Exchange" reversible="true" fast="false" fbc:lowerFluxBound="rev_lb"
fbc:upperFluxBound="rev_ub">
            <listOfReactants>
                  <speciesReference species="M_fuc__L_e" stoichiometry="1"
constant="true"/>
            </listOfReactants>
      </reaction>

      <reaction metaid="EX_fuc1p__L_e" id="EX_fuc1p__L_e" name="L-Fucose 1-
phosphate Exchange" reversible="true" fast="false"
fbc:lowerFluxBound="rev_lb" fbc:upperFluxBound="rev_ub">
            <listOfReactants>
                  <speciesReference species="M_fuc1p__L_e" stoichiometry="1"
constant="true"/>
            </listOfReactants>
      </reaction>

      <reaction metaid="EX_fum_e" id="EX_fum_e" name="Fumarate Exchange"
reversible="true" fast="false" fbc:lowerFluxBound="rev_lb"
fbc:upperFluxBound="rev_ub">
            <listOfReactants>
                  <speciesReference species="M_fum_e" stoichiometry="1"
constant="true"/>
            </listOfReactants>
      </reaction>

      <reaction metaid="EX_g1p_e" id="EX_g1p_e" name="D-Glucose 1-phosphate
Exchange" reversible="true" fast="false" fbc:lowerFluxBound="rev_lb"
fbc:upperFluxBound="rev_ub">
            <listOfReactants>
                  <speciesReference species="M_g1p_e" stoichiometry="1"
constant="true"/>
            </listOfReactants>
      </reaction>

      <reaction metaid="EX_g6p_e" id="EX_g6p_e" name="D-Glucose 6-phosphate
Exchange" reversible="true" fast="false" fbc:lowerFluxBound="rev_lb"
fbc:upperFluxBound="rev_ub">
            <listOfReactants>
                  <speciesReference species="M_g6p_e" stoichiometry="1"
constant="true"/>
            </listOfReactants>
      </reaction>

      <reaction metaid="EX_gal_e" id="EX_gal_e" name="D-Galactose Exchange"
reversible="true" fast="false" fbc:lowerFluxBound="rev_lb"
fbc:upperFluxBound="rev_ub">
            <listOfReactants>
                  <speciesReference species="M_gal_e" stoichiometry="1"
constant="true"/>
            </listOfReactants>
      </reaction>
```

```
      <reaction metaid="EX_galct__D_e" id="EX_galct__D_e" name="D-Galactarate
Exchange" reversible="true" fast="false" fbc:lowerFluxBound="rev_lb"
fbc:upperFluxBound="rev_ub">
            <listOfReactants>
                  <speciesReference species="M_galct__D_e" stoichiometry="1"
constant="true"/>
            </listOfReactants>
      </reaction>

      <reaction metaid="EX_galctn__D_e" id="EX_galctn__D_e" name="D-
Galactonate Exchange" reversible="true" fast="false"
fbc:lowerFluxBound="rev_lb" fbc:upperFluxBound="rev_ub">
            <listOfReactants>
                  <speciesReference species="M_galctn__D_e" stoichiometry="1"
constant="true"/>
            </listOfReactants>
      </reaction>

      <reaction metaid="EX_galt_e" id="EX_galt_e" name="Galactitol Exchange"
reversible="true" fast="false" fbc:lowerFluxBound="rev_lb"
fbc:upperFluxBound="rev_ub">
            <listOfReactants>
                  <speciesReference species="M_galt_e" stoichiometry="1"
constant="true"/>
            </listOfReactants>
      </reaction>

      <reaction metaid="EX_galur_e" id="EX_galur_e" name="D-Galacturonate
Exchange" reversible="true" fast="false" fbc:lowerFluxBound="rev_lb"
fbc:upperFluxBound="rev_ub">
            <listOfReactants>
                  <speciesReference species="M_galur_e" stoichiometry="1"
constant="true"/>
            </listOfReactants>
      </reaction>

      <reaction metaid="EX_gam_e" id="EX_gam_e" name="D-Glucosamine Exchange"
reversible="true" fast="false" fbc:lowerFluxBound="rev_lb"
fbc:upperFluxBound="rev_ub">
            <listOfReactants>
                  <speciesReference species="M_gam_e" stoichiometry="1"
constant="true"/>
            </listOfReactants>
      </reaction>

      <reaction metaid="EX_gam6p_e" id="EX_gam6p_e" name="D-Glucosamine 6-
phosphate Exchange" reversible="true" fast="false"
fbc:lowerFluxBound="rev_lb" fbc:upperFluxBound="rev_ub">
            <listOfReactants>
                  <speciesReference species="M_gam6p_e" stoichiometry="1"
constant="true"/>
            </listOfReactants>
      </reaction>

      <reaction metaid="EX_glc_e" id="EX_glc_e" name="D-glucose Exchange"
reversible="true" fast="false" fbc:lowerFluxBound="rev_lb"
fbc:upperFluxBound="rev_ub">
            <listOfReactants>
```

```
                  <speciesReference species="M_glc__D_e" stoichiometry="1"
constant="true"/>
            </listOfReactants>
      </reaction>

      <reaction metaid="EX_glcn_e" id="EX_glcn_e" name="D-Gluconate Exchange"
reversible="true" fast="false" fbc:lowerFluxBound="rev_lb"
fbc:upperFluxBound="rev_ub">
            <listOfReactants>
                  <speciesReference species="M_glcn_e" stoichiometry="1"
constant="true"/>
            </listOfReactants>
      </reaction>

      <reaction metaid="EX_glcr_e" id="EX_glcr_e" name="D-Glucarate Exchange"
reversible="true" fast="false" fbc:lowerFluxBound="rev_lb"
fbc:upperFluxBound="rev_ub">
            <listOfReactants>
                  <speciesReference species="M_glcr_e" stoichiometry="1"
constant="true"/>
            </listOfReactants>
      </reaction>

      <reaction metaid="EX_glcur_e" id="EX_glcur_e" name="D-Glucuronate
Exchange" reversible="true" fast="false" fbc:lowerFluxBound="rev_lb"
fbc:upperFluxBound="rev_ub">
            <listOfReactants>
                  <speciesReference species="M_glcur_e" stoichiometry="1"
constant="true"/>
            </listOfReactants>
      </reaction>

      <reaction metaid="EX_gln__L_e" id="EX_gln__L_e" name="L-Glutamine
Exchange" reversible="true" fast="false" fbc:lowerFluxBound="rev_lb"
fbc:upperFluxBound="rev_ub">
            <listOfReactants>
                  <speciesReference species="M_gln__L_e" stoichiometry="1"
constant="true"/>
            </listOfReactants>
      </reaction>

      <reaction metaid="EX_glu__L_e" id="EX_glu__L_e" name="L-Glutamate
Exchange" reversible="true" fast="false" fbc:lowerFluxBound="rev_lb"
fbc:upperFluxBound="rev_ub">
            <listOfReactants>
                  <speciesReference species="M_glu__L_e" stoichiometry="1"
constant="true"/>
            </listOfReactants>
      </reaction>

      <reaction metaid="EX_gly_e" id="EX_gly_e" name="Glycine Exchange"
reversible="true" fast="false" fbc:lowerFluxBound="rev_lb"
fbc:upperFluxBound="rev_ub">
            <listOfReactants>
                  <speciesReference species="M_gly_e" stoichiometry="1"
constant="true"/>
            </listOfReactants>
      </reaction>
```

```
      <reaction metaid="EX_glyald_e" id="EX_glyald_e" name="D-Glyceraldehyde
Exchange" reversible="true" fast="false" fbc:lowerFluxBound="rev_lb"
fbc:upperFluxBound="rev_ub">
            <listOfReactants>
                  <speciesReference species="M_glyald_e" stoichiometry="1"
constant="true"/>
            </listOfReactants>
      </reaction>

      <reaction metaid="EX_glyb_e" id="EX_glyb_e" name="Glycine betaine
Exchange" reversible="true" fast="false" fbc:lowerFluxBound="rev_lb"
fbc:upperFluxBound="rev_ub">
            <listOfReactants>
                  <speciesReference species="M_glyb_e" stoichiometry="1"
constant="true"/>
            </listOfReactants>
      </reaction>

      <reaction metaid="EX_glyc_e" id="EX_glyc_e" name="Glycerol Exchange"
reversible="true" fast="false" fbc:lowerFluxBound="rev_lb"
fbc:upperFluxBound="rev_ub">
            <listOfReactants>
                  <speciesReference species="M_glyc_e" stoichiometry="1"
constant="true"/>
            </listOfReactants>
      </reaction>

      <reaction metaid="EX_glyc3p_e" id="EX_glyc3p_e" name="Glycerol 3-
phosphate Exchange" reversible="true" fast="false"
fbc:lowerFluxBound="rev_lb" fbc:upperFluxBound="rev_ub">
            <listOfReactants>
                  <speciesReference species="M_glyc3p_e" stoichiometry="1"
constant="true"/>
            </listOfReactants>
      </reaction>

      <reaction metaid="EX_glyclt_e" id="EX_glyclt_e" name="Glycolate
Exchange" reversible="true" fast="false" fbc:lowerFluxBound="rev_lb"
fbc:upperFluxBound="rev_ub">
            <listOfReactants>
                  <speciesReference species="M_glyclt_e" stoichiometry="1"
constant="true"/>
            </listOfReactants>
      </reaction>

      <reaction metaid="EX_gsn_e" id="EX_gsn_e" name="Guanosine Exchange"
reversible="true" fast="false" fbc:lowerFluxBound="rev_lb"
fbc:upperFluxBound="rev_ub">
            <listOfReactants>
                  <speciesReference species="M_gsn_e" stoichiometry="1"
constant="true"/>
            </listOfReactants>
      </reaction>

      <reaction metaid="EX_gua_e" id="EX_gua_e" name="Guanine Exchange"
reversible="true" fast="false" fbc:lowerFluxBound="rev_lb"
fbc:upperFluxBound="rev_ub">
```

```
            <listOfReactants>
                  <speciesReference species="M_gua_e" stoichiometry="1"
constant="true"/>
            </listOfReactants>
      </reaction>

      <reaction metaid="EX_h_e" id="EX_h_e" name="H+ Exchange"
reversible="true" fast="false" fbc:lowerFluxBound="rev_lb"
fbc:upperFluxBound="rev_ub">
            <listOfReactants>
                  <speciesReference species="M_h_e" stoichiometry="1"
constant="true"/>
            </listOfReactants>
      </reaction>

      <reaction metaid="EX_h2o_e" id="EX_h2o_e" name="H2O Exchange"
reversible="true" fast="false" fbc:lowerFluxBound="rev_lb"
fbc:upperFluxBound="rev_ub">
            <listOfReactants>
                  <speciesReference species="M_h2o_e" stoichiometry="1"
constant="true"/>
            </listOfReactants>
      </reaction>

      <reaction metaid="EX_hdca_e" id="EX_hdca_e" name="Hexadecanoate (n-
C16:0) Exchange" reversible="true" fast="false" fbc:lowerFluxBound="rev_lb"
fbc:upperFluxBound="rev_ub">
            <listOfReactants>
                  <speciesReference species="M_hdca_e" stoichiometry="1"
constant="true"/>
            </listOfReactants>
      </reaction>

      <reaction metaid="EX_hg2_e" id="EX_hg2_e" name="Hg2+ Exchange"
reversible="true" fast="false" fbc:lowerFluxBound="rev_lb"
fbc:upperFluxBound="rev_ub">
            <listOfReactants>
                  <speciesReference species="M_hg2_e" stoichiometry="1"
constant="true"/>
            </listOfReactants>
      </reaction>

      <reaction metaid="EX_his__L_e" id="EX_his__L_e" name="L-Histidine
Exchange" reversible="true" fast="false" fbc:lowerFluxBound="rev_lb"
fbc:upperFluxBound="rev_ub">
            <listOfReactants>
                  <speciesReference species="M_his__L_e" stoichiometry="1"
constant="true"/>
            </listOfReactants>
      </reaction>

      <reaction metaid="EX_hom__L_e" id="EX_hom__L_e" name="L-Homoserine
Exchange" reversible="true" fast="false" fbc:lowerFluxBound="rev_lb"
fbc:upperFluxBound="rev_ub">
            <listOfReactants>
                  <speciesReference species="M_hom__L_e" stoichiometry="1"
constant="true"/>
            </listOfReactants>
```

```
      </reaction>

      <reaction metaid="EX_hxa_e" id="EX_hxa_e" name="Hexanoate (n-C6:0)
Exchange" reversible="true" fast="false" fbc:lowerFluxBound="rev_lb"
fbc:upperFluxBound="rev_ub">
            <listOfReactants>
                  <speciesReference species="M_hxa_e" stoichiometry="1"
constant="true"/>
            </listOfReactants>
      </reaction>

      <reaction metaid="EX_hxan_e" id="EX_hxan_e" name="Hypoxanthine
Exchange" reversible="true" fast="false" fbc:lowerFluxBound="rev_lb"
fbc:upperFluxBound="rev_ub">
            <listOfReactants>
                  <speciesReference species="M_hxan_e" stoichiometry="1"
constant="true"/>
            </listOfReactants>
      </reaction>

      <reaction metaid="EX_ile__L_e" id="EX_ile__L_e" name="L-Isoleucine
Exchange" reversible="true" fast="false" fbc:lowerFluxBound="rev_lb"
fbc:upperFluxBound="rev_ub">
            <listOfReactants>
                  <speciesReference species="M_ile__L_e" stoichiometry="1"
constant="true"/>
            </listOfReactants>
      </reaction>

      <reaction metaid="EX_inost_e" id="EX_inost_e" name="myo-Inositol
Exchange" reversible="true" fast="false" fbc:lowerFluxBound="rev_lb"
fbc:upperFluxBound="rev_ub">
            <listOfReactants>
                  <speciesReference species="M_inost_e" stoichiometry="1"
constant="true"/>
            </listOfReactants>
      </reaction>

      <reaction metaid="EX_ins_e" id="EX_ins_e" name="Inosine Exchange"
reversible="true" fast="false" fbc:lowerFluxBound="rev_lb"
fbc:upperFluxBound="rev_ub">
            <listOfReactants>
                  <speciesReference species="M_ins_e" stoichiometry="1"
constant="true"/>
            </listOfReactants>
      </reaction>

      <reaction metaid="EX_isetac_e" id="EX_isetac_e" name="Isethionic acid
Exchange" reversible="true" fast="false" fbc:lowerFluxBound="rev_lb"
fbc:upperFluxBound="rev_ub">
            <listOfReactants>
                  <speciesReference species="M_isetac_e" stoichiometry="1"
constant="true"/>
            </listOfReactants>
      </reaction>
```

```
      <reaction metaid="EX_k_e" id="EX_k_e" name="potassium Exchange"
reversible="true" fast="false" fbc:lowerFluxBound="rev_lb"
fbc:upperFluxBound="rev_ub">
            <listOfReactants>
                  <speciesReference species="M_k_e" stoichiometry="1"
constant="true"/>
            </listOfReactants>
      </reaction>

      <reaction metaid="EX_lac__L_e" id="EX_lac__L_e" name="L__Lactate
Exchange" reversible="true" fast="false" fbc:lowerFluxBound="rev_lb"
fbc:upperFluxBound="rev_ub">
            <listOfReactants>
                  <speciesReference species="M_lac__L_e" stoichiometry="1"
constant="true"/>
            </listOfReactants>
      </reaction>

      <reaction metaid="EX_lcts_e" id="EX_lcts_e" name="Lactose Exchange"
reversible="true" fast="false" fbc:lowerFluxBound="rev_lb"
fbc:upperFluxBound="rev_ub">
            <listOfReactants>
                  <speciesReference species="M_lcts_e" stoichiometry="1"
constant="true"/>
            </listOfReactants>
      </reaction>

      <reaction metaid="EX_leu__L_e" id="EX_leu__L_e" name="L__Leucine
Exchange" reversible="true" fast="false" fbc:lowerFluxBound="rev_lb"
fbc:upperFluxBound="rev_ub">
            <listOfReactants>
                  <speciesReference species="M_leu__L_e" stoichiometry="1"
constant="true"/>
            </listOfReactants>
      </reaction>

      <reaction metaid="EX_lys__L_e" id="EX_lys__L_e" name="L__Lysine
Exchange" reversible="true" fast="false" fbc:lowerFluxBound="rev_lb"
fbc:upperFluxBound="rev_ub">
            <listOfReactants>
                  <speciesReference species="M_lys__L_e" stoichiometry="1"
constant="true"/>
            </listOfReactants>
      </reaction>

      <reaction metaid="EX_lyx__L_e" id="EX_lyx__L_e" name="L__Lyxose
Exchange" reversible="true" fast="false" fbc:lowerFluxBound="rev_lb"
fbc:upperFluxBound="rev_ub">
            <listOfReactants>
                  <speciesReference species="M_lyx__L_e" stoichiometry="1"
constant="true"/>
            </listOfReactants>
      </reaction>

      <reaction metaid="EX_mal__D_e" id="EX_mal__D_e" name="D-Malate
Exchange" reversible="true" fast="false" fbc:lowerFluxBound="rev_lb"
fbc:upperFluxBound="rev_ub">
            <listOfReactants>
```

```
                  <speciesReference species="M_mal__D_e" stoichiometry="1"
constant="true"/>
            </listOfReactants>
      </reaction>

      <reaction metaid="EX_mal__L_e" id="EX_mal__L_e" name="L-Malate
Exchange" reversible="true" fast="false" fbc:lowerFluxBound="rev_lb"
fbc:upperFluxBound="rev_ub">
            <listOfReactants>
                  <speciesReference species="M_mal__L_e" stoichiometry="1"
constant="true"/>
            </listOfReactants>
      </reaction>

      <reaction metaid="EX_malt_e" id="EX_malt_e" name="Maltose Exchange"
reversible="true" fast="false" fbc:lowerFluxBound="rev_lb"
fbc:upperFluxBound="rev_ub">
            <listOfReactants>
                  <speciesReference species="M_malt_e" stoichiometry="1"
constant="true"/>
            </listOfReactants>
      </reaction>

      <reaction metaid="EX_malthx_e" id="EX_malthx_e" name="Maltohexaose
Exchange" reversible="true" fast="false" fbc:lowerFluxBound="rev_lb"
fbc:upperFluxBound="rev_ub">
            <listOfReactants>
                  <speciesReference species="M_malthx_e" stoichiometry="1"
constant="true"/>
            </listOfReactants>
      </reaction>

      <reaction metaid="EX_malttr_e" id="EX_malttr_e" name="Maltotriose
Exchange" reversible="true" fast="false" fbc:lowerFluxBound="rev_lb"
fbc:upperFluxBound="rev_ub">
            <listOfReactants>
                  <speciesReference species="M_malttr_e" stoichiometry="1"
constant="true"/>
            </listOfReactants>
      </reaction>

      <reaction metaid="EX_man_e" id="EX_man_e" name="D-Mannose Exchange"
reversible="true" fast="false" fbc:lowerFluxBound="rev_lb"
fbc:upperFluxBound="rev_ub">
            <listOfReactants>
                  <speciesReference species="M_man_e" stoichiometry="1"
constant="true"/>
            </listOfReactants>
      </reaction>

      <reaction metaid="EX_man6p_e" id="EX_man6p_e" name="D-Mannose 6-
phosphate Exchange" reversible="true" fast="false"
fbc:lowerFluxBound="rev_lb" fbc:upperFluxBound="rev_ub">
            <listOfReactants>
                  <speciesReference species="M_man6p_e" stoichiometry="1"
constant="true"/>
            </listOfReactants>
      </reaction>
```

<reaction metaid="EX_manl1p_e" id="EX_manl1p_e" name="manl1p Exchange"
reversible="true" fast="false" fbc:lowerFluxBound="rev_lb"
fbc:upperFluxBound="rev_ub">
<listOfReactants>
<speciesReference species="M_manl1p_e" stoichiometry="1"
constant="true"/>
</listOfReactants>
</reaction>

<reaction metaid="EX_melib_e" id="EX_melib_e" name="Melibiose Exchange"
reversible="true" fast="false" fbc:lowerFluxBound="rev_lb"
fbc:upperFluxBound="rev_ub">
<listOfReactants>
<speciesReference species="M_melib_e" stoichiometry="1"
constant="true"/>
</listOfReactants>
</reaction>

<reaction metaid="EX_met__D_e" id="EX_met__D_e" name="D-Methionine
Exchange" reversible="true" fast="false" fbc:lowerFluxBound="rev_lb"
fbc:upperFluxBound="rev_ub">
<listOfReactants>
<speciesReference species="M_met__D_e" stoichiometry="1"
constant="true"/>
</listOfReactants>
</reaction>

<reaction metaid="EX_met__L_e" id="EX_met__L_e" name="L-Methionine
Exchange" reversible="true" fast="false" fbc:lowerFluxBound="rev_lb"
fbc:upperFluxBound="rev_ub">
<listOfReactants>
<speciesReference species="M_met__L_e" stoichiometry="1"
constant="true"/>
</listOfReactants>
</reaction>

<reaction metaid="EX_metsox__R__L_e" id="EX_metsox__R__L_e" name="L-
methionine-R-sulfoxide Exchange" reversible="true" fast="false"
fbc:lowerFluxBound="rev_lb" fbc:upperFluxBound="rev_ub">
<listOfReactants>
<speciesReference species="M_metsox__R__L_e"
stoichiometry="1" constant="true"/>
</listOfReactants>
</reaction>

<reaction metaid="EX_metsox__S__L_e" id="EX_metsox__S__L_e" name="L-
Methionine Sulfoxide Exchange" reversible="true" fast="false"
fbc:lowerFluxBound="rev_lb" fbc:upperFluxBound="rev_ub">
<listOfReactants>
<speciesReference species="M_metsox__S__L_e"
stoichiometry="1" constant="true"/>
</listOfReactants>
</reaction>

<reaction metaid="EX_mg2_e" id="EX_mg2_e" name="magnesium Exchange"
reversible="true" fast="false" fbc:lowerFluxBound="rev_lb"
fbc:upperFluxBound="rev_ub">

```
            <listOfReactants>
                  <speciesReference species="M_mg2_e" stoichiometry="1"
constant="true"/>
            </listOfReactants>
      </reaction>

      <reaction metaid="EX_mn2_e" id="EX_mn2_e" name="Mn2+ Exchange"
reversible="true" fast="false" fbc:lowerFluxBound="rev_lb"
fbc:upperFluxBound="rev_ub">
            <listOfReactants>
                  <speciesReference species="M_mn2_e" stoichiometry="1"
constant="true"/>
            </listOfReactants>
      </reaction>

      <reaction metaid="EX_mnl_e" id="EX_mnl_e" name="D-Mannitol Exchange"
reversible="true" fast="false" fbc:lowerFluxBound="rev_lb"
fbc:upperFluxBound="rev_ub">
            <listOfReactants>
                  <speciesReference species="M_mnl_e" stoichiometry="1"
constant="true"/>
            </listOfReactants>
      </reaction>

      <reaction metaid="EX_mobd_e" id="EX_mobd_e" name="Molybdate Exchange"
reversible="true" fast="false" fbc:lowerFluxBound="rev_lb"
fbc:upperFluxBound="rev_ub">
            <listOfReactants>
                  <speciesReference species="M_mobd_e" stoichiometry="1"
constant="true"/>
            </listOfReactants>
      </reaction>

      <reaction metaid="EX_mso3_e" id="EX_mso3_e" name="methanesulfonate
Exchange" reversible="true" fast="false" fbc:lowerFluxBound="rev_lb"
fbc:upperFluxBound="rev_ub">
            <listOfReactants>
                  <speciesReference species="M_mso3_e" stoichiometry="1"
constant="true"/>
            </listOfReactants>
      </reaction>

      <reaction metaid="EX_na1_e" id="EX_na1_e" name="Sodium Exchange"
reversible="true" fast="false" fbc:lowerFluxBound="rev_lb"
fbc:upperFluxBound="rev_ub">
            <listOfReactants>
                  <speciesReference species="M_na1_e" stoichiometry="1"
constant="true"/>
            </listOfReactants>
      </reaction>

      <reaction metaid="EX_nac_e" id="EX_nac_e" name="Nicotinate Exchange"
reversible="true" fast="false" fbc:lowerFluxBound="rev_lb"
fbc:upperFluxBound="rev_ub">
            <listOfReactants>
                  <speciesReference species="M_nac_e" stoichiometry="1"
constant="true"/>
            </listOfReactants>
```

```
		</reaction>

		<reaction metaid="EX_ncam_e" id="EX_ncam_e" name="Nicotinamide
Exchange" reversible="true" fast="false" fbc:lowerFluxBound="rev_lb"
fbc:upperFluxBound="rev_ub">
			<listOfReactants>
				<speciesReference species="M_ncam_e" stoichiometry="1"
constant="true"/>
			</listOfReactants>
		</reaction>

		<reaction metaid="EX_nh4_e" id="EX_nh4_e" name="Ammonium Exchange"
reversible="true" fast="false" fbc:lowerFluxBound="rev_lb"
fbc:upperFluxBound="rev_ub">
			<listOfReactants>
				<speciesReference species="M_nh4_e" stoichiometry="1"
constant="true"/>
			</listOfReactants>
		</reaction>

		<reaction metaid="EX_ni2_e" id="EX_ni2_e" name="nickel Exchange"
reversible="true" fast="false" fbc:lowerFluxBound="rev_lb"
fbc:upperFluxBound="rev_ub">
			<listOfReactants>
				<speciesReference species="M_ni2_e" stoichiometry="1"
constant="true"/>
			</listOfReactants>
		</reaction>

		<reaction metaid="EX_nmn_e" id="EX_nmn_e" name="NMN Exchange"
reversible="true" fast="false" fbc:lowerFluxBound="rev_lb"
fbc:upperFluxBound="rev_ub">
			<listOfReactants>
				<speciesReference species="M_nmn_e" stoichiometry="1"
constant="true"/>
			</listOfReactants>
		</reaction>

		<reaction metaid="EX_no2_e" id="EX_no2_e" name="Nitrite Exchange"
reversible="true" fast="false" fbc:lowerFluxBound="rev_lb"
fbc:upperFluxBound="rev_ub">
			<listOfReactants>
				<speciesReference species="M_no2_e" stoichiometry="1"
constant="true"/>
			</listOfReactants>
		</reaction>

		<reaction metaid="EX_no3_e" id="EX_no3_e" name="Nitrate Exchange"
reversible="true" fast="false" fbc:lowerFluxBound="rev_lb"
fbc:upperFluxBound="rev_ub">
			<listOfReactants>
				<speciesReference species="M_no3_e" stoichiometry="1"
constant="true"/>
			</listOfReactants>
		</reaction>
```

```
      <reaction metaid="EX_o2_e" id="EX_o2_e" name="O2 Exchange"
reversible="true" fast="false" fbc:lowerFluxBound="rev_lb"
fbc:upperFluxBound="rev_ub">
            <listOfReactants>
                  <speciesReference species="M_o2_e" stoichiometry="1"
constant="true"/>
            </listOfReactants>
      </reaction>

      <reaction metaid="EX_orn_e" id="EX_orn_e" name="Ornithine Exchange"
reversible="true" fast="false" fbc:lowerFluxBound="rev_lb"
fbc:upperFluxBound="rev_ub">
            <listOfReactants>
                  <speciesReference species="M_orn_e" stoichiometry="1"
constant="true"/>
            </listOfReactants>
      </reaction>

      <reaction metaid="EX_pb_e" id="EX_pb_e" name="Pb Exchange"
reversible="true" fast="false" fbc:lowerFluxBound="rev_lb"
fbc:upperFluxBound="rev_ub">
            <listOfReactants>
                  <speciesReference species="M_pb_e" stoichiometry="1"
constant="true"/>
            </listOfReactants>
      </reaction>

      <reaction metaid="EX_peamn_e" id="EX_peamn_e" name="Phenethylamine
Exchange" reversible="true" fast="false" fbc:lowerFluxBound="rev_lb"
fbc:upperFluxBound="rev_ub">
            <listOfReactants>
                  <speciesReference species="M_peamn_e" stoichiometry="1"
constant="true"/>
            </listOfReactants>
      </reaction>

      <reaction metaid="EX_phe__L_e" id="EX_phe__L_e" name="L-Phenylalanine
Exchange" reversible="true" fast="false" fbc:lowerFluxBound="rev_lb"
fbc:upperFluxBound="rev_ub">
            <listOfReactants>
                  <speciesReference species="M_phe__L_e" stoichiometry="1"
constant="true"/>
            </listOfReactants>
      </reaction>

      <reaction metaid="EX_pheme_e" id="EX_pheme_e" name="Protoheme Exchange"
reversible="true" fast="false" fbc:lowerFluxBound="rev_lb"
fbc:upperFluxBound="rev_ub">
            <listOfReactants>
                  <speciesReference species="M_pheme_e" stoichiometry="1"
constant="true"/>
            </listOfReactants>
      </reaction>

      <reaction metaid="EX_pi_e" id="EX_pi_e" name="Phosphate Exchange"
reversible="true" fast="false" fbc:lowerFluxBound="rev_lb"
fbc:upperFluxBound="rev_ub">
            <listOfReactants>
```

<speciesReference species="M_pi_e" stoichiometry="1"
constant="true"/>
</listOfReactants>
</reaction>

<reaction metaid="EX_ppa_e" id="EX_ppa_e" name="Propionate (n-C3:0)
Exchange" reversible="true" fast="false" fbc:lowerFluxBound="rev_lb"
fbc:upperFluxBound="rev_ub">
<listOfReactants>
<speciesReference species="M_ppa_e" stoichiometry="1"
constant="true"/>
</listOfReactants>
</reaction>

<reaction metaid="EX_ppi_e" id="EX_ppi_e" name="Diphosphate Exchange"
reversible="true" fast="false" fbc:lowerFluxBound="rev_lb"
fbc:upperFluxBound="rev_ub">
<listOfReactants>
<speciesReference species="M_ppi_e" stoichiometry="1"
constant="true"/>
</listOfReactants>
</reaction>

<reaction metaid="EX_pro__L_e" id="EX_pro__L_e" name="L-Proline
Exchange" reversible="true" fast="false" fbc:lowerFluxBound="rev_lb"
fbc:upperFluxBound="rev_ub">
<listOfReactants>
<speciesReference species="M_pro__L_e" stoichiometry="1"
constant="true"/>
</listOfReactants>
</reaction>

<reaction metaid="EX_ptrc_e" id="EX_ptrc_e" name="Putrescine Exchange"
reversible="true" fast="false" fbc:lowerFluxBound="rev_lb"
fbc:upperFluxBound="rev_ub">
<listOfReactants>
<speciesReference species="M_ptrc_e" stoichiometry="1"
constant="true"/>
</listOfReactants>
</reaction>

<reaction metaid="EX_pydx_e" id="EX_pydx_e" name="Pyridoxal Exchange"
reversible="true" fast="false" fbc:lowerFluxBound="rev_lb"
fbc:upperFluxBound="rev_ub">
<listOfReactants>
<speciesReference species="M_pydx_e" stoichiometry="1"
constant="true"/>
</listOfReactants>
</reaction>

<reaction metaid="EX_pyr_e" id="EX_pyr_e" name="Pyruvate Exchange"
reversible="true" fast="false" fbc:lowerFluxBound="rev_lb"
fbc:upperFluxBound="rev_ub">
<listOfReactants>
<speciesReference species="M_pyr_e" stoichiometry="1"
constant="true"/>
</listOfReactants>
</reaction>

```
      <reaction metaid="EX_quin_e" id="EX_quin_e" name="Quinate Exchange"
reversible="true" fast="false" fbc:lowerFluxBound="rev_lb"
fbc:upperFluxBound="rev_ub">
            <listOfReactants>
                  <speciesReference species="M_quin_e" stoichiometry="1"
constant="true"/>
            </listOfReactants>
      </reaction>

      <reaction metaid="EX_rib__D_e" id="EX_rib__D_e" name="D-Ribose
Exchange" reversible="true" fast="false" fbc:lowerFluxBound="rev_lb"
fbc:upperFluxBound="rev_ub">
            <listOfReactants>
                  <speciesReference species="M_rib__D_e" stoichiometry="1"
constant="true"/>
            </listOfReactants>
      </reaction>

      <reaction metaid="EX_ribflv_e" id="EX_ribflv_e" name="Riboflavin
Exchange" reversible="true" fast="false" fbc:lowerFluxBound="rev_lb"
fbc:upperFluxBound="rev_ub">
            <listOfReactants>
                  <speciesReference species="M_ribflv_e" stoichiometry="1"
constant="true"/>
            </listOfReactants>
      </reaction>

      <reaction metaid="EX_rmn_e" id="EX_rmn_e" name="L-Rhamnose Exchange"
reversible="true" fast="false" fbc:lowerFluxBound="rev_lb"
fbc:upperFluxBound="rev_ub">
            <listOfReactants>
                  <speciesReference species="M_rmn_e" stoichiometry="1"
constant="true"/>
            </listOfReactants>
      </reaction>

      <reaction metaid="EX_salcn_e" id="EX_salcn_e" name="Salicin Exchange"
reversible="true" fast="false" fbc:lowerFluxBound="rev_lb"
fbc:upperFluxBound="rev_ub">
            <listOfReactants>
                  <speciesReference species="M_salcn_e" stoichiometry="1"
constant="true"/>
            </listOfReactants>
      </reaction>

      <reaction metaid="EX_sbt__D_e" id="EX_sbt__D_e" name="D-Sorbitol
Exchange" reversible="true" fast="false" fbc:lowerFluxBound="rev_lb"
fbc:upperFluxBound="rev_ub">
            <listOfReactants>
                  <speciesReference species="M_sbt__D_e" stoichiometry="1"
constant="true"/>
            </listOfReactants>
      </reaction>

      <reaction metaid="EX_ser__D_e" id="EX_ser__D_e" name="D-Serine
Exchange" reversible="true" fast="false" fbc:lowerFluxBound="rev_lb"
fbc:upperFluxBound="rev_ub">
```

```
            &lt;listOfReactants&gt;
                  <speciesReference species="M_ser__D_e" stoichiometry="1"
constant="true"/>
            &lt;/listOfReactants&gt;
      &lt;/reaction&gt;

      <reaction metaid="EX_ser__L_e" id="EX_ser__L_e" name="L-Serine
Exchange" reversible="true" fast="false" fbc:lowerFluxBound="rev_lb"
fbc:upperFluxBound="rev_ub">
            &lt;listOfReactants&gt;
                  <speciesReference species="M_ser__L_e" stoichiometry="1"
constant="true"/>
            &lt;/listOfReactants&gt;
      &lt;/reaction&gt;

      <reaction metaid="EX_so4_e" id="EX_so4_e" name="Sulfate Exchange"
reversible="true" fast="false" fbc:lowerFluxBound="rev_lb"
fbc:upperFluxBound="rev_ub">
            &lt;listOfReactants&gt;
                  <speciesReference species="M_so4_e" stoichiometry="1"
constant="true"/>
            &lt;/listOfReactants&gt;
      &lt;/reaction&gt;

      <reaction metaid="EX_spmd_e" id="EX_spmd_e" name="Spermidine Exchange"
reversible="true" fast="false" fbc:lowerFluxBound="rev_lb"
fbc:upperFluxBound="rev_ub">
            &lt;listOfReactants&gt;
                  <speciesReference species="M_spmd_e" stoichiometry="1"
constant="true"/>
            &lt;/listOfReactants&gt;
      &lt;/reaction&gt;

      <reaction metaid="EX_succ_e" id="EX_succ_e" name="Succinate Exchange"
reversible="true" fast="false" fbc:lowerFluxBound="rev_lb"
fbc:upperFluxBound="rev_ub">
            &lt;listOfReactants&gt;
                  <speciesReference species="M_succ_e" stoichiometry="1"
constant="true"/>
            &lt;/listOfReactants&gt;
      &lt;/reaction&gt;

      <reaction metaid="EX_sucr_e" id="EX_sucr_e" name="Sucrose Exchange"
reversible="true" fast="false" fbc:lowerFluxBound="rev_lb"
fbc:upperFluxBound="rev_ub">
            &lt;listOfReactants&gt;
                  <speciesReference species="M_sucr_e" stoichiometry="1"
constant="true"/>
            &lt;/listOfReactants&gt;
      &lt;/reaction&gt;

      <reaction metaid="EX_tartr__D_e" id="EX_tartr__D_e" name="D-tartrate
Exchange" reversible="true" fast="false" fbc:lowerFluxBound="rev_lb"
fbc:upperFluxBound="rev_ub">
            &lt;listOfReactants&gt;
                  <speciesReference species="M_tartr__D_e" stoichiometry="1"
constant="true"/>
            &lt;/listOfReactants&gt;
```

```
      &lt;/reaction&gt;

      <reaction metaid="EX_tartr__L_e" id="EX_tartr__L_e" name="L-tartrate
Exchange" reversible="true" fast="false" fbc:lowerFluxBound="rev_lb"
fbc:upperFluxBound="rev_ub">
            &lt;listOfReactants&gt;
                  <speciesReference species="M_tartr__L_e" stoichiometry="1"
constant="true"/>
            &lt;/listOfReactants&gt;
      &lt;/reaction&gt;

      <reaction metaid="EX_taur_e" id="EX_taur_e" name="Taurine Exchange"
reversible="true" fast="false" fbc:lowerFluxBound="rev_lb"
fbc:upperFluxBound="rev_ub">
            &lt;listOfReactants&gt;
                  <speciesReference species="M_taur_e" stoichiometry="1"
constant="true"/>
            &lt;/listOfReactants&gt;
      &lt;/reaction&gt;

      <reaction metaid="EX_thm_e" id="EX_thm_e" name="Thiamin Exchange"
reversible="true" fast="false" fbc:lowerFluxBound="rev_lb"
fbc:upperFluxBound="rev_ub">
            &lt;listOfReactants&gt;
                  <speciesReference species="M_thm_e" stoichiometry="1"
constant="true"/>
            &lt;/listOfReactants&gt;
      &lt;/reaction&gt;

      <reaction metaid="EX_thr__L_e" id="EX_thr__L_e" name="L-Threonine
Exchange" reversible="true" fast="false" fbc:lowerFluxBound="rev_lb"
fbc:upperFluxBound="rev_ub">
            &lt;listOfReactants&gt;
                  <speciesReference species="M_thr__L_e" stoichiometry="1"
constant="true"/>
            &lt;/listOfReactants&gt;
      &lt;/reaction&gt;

      <reaction metaid="EX_thym_e" id="EX_thym_e" name="Thymine Exchange"
reversible="true" fast="false" fbc:lowerFluxBound="rev_lb"
fbc:upperFluxBound="rev_ub">
            &lt;listOfReactants&gt;
                  <speciesReference species="M_thym_e" stoichiometry="1"
constant="true"/>
            &lt;/listOfReactants&gt;
      &lt;/reaction&gt;

      <reaction metaid="EX_thymd_e" id="EX_thymd_e" name="Thymidine Exchange"
reversible="true" fast="false" fbc:lowerFluxBound="rev_lb"
fbc:upperFluxBound="rev_ub">
            &lt;listOfReactants&gt;
                  <speciesReference species="M_thymd_e" stoichiometry="1"
constant="true"/>
            &lt;/listOfReactants&gt;
      &lt;/reaction&gt;
```

```
      <reaction metaid="EX_tre_e" id="EX_tre_e" name="Trehalose Exchange"
reversible="true" fast="false" fbc:lowerFluxBound="rev_lb"
fbc:upperFluxBound="rev_ub">
            <listOfReactants>
                  <speciesReference species="M_tre_e" stoichiometry="1"
constant="true"/>
            </listOfReactants>
      </reaction>

      <reaction metaid="EX_trp__L_e" id="EX_trp__L_e" name="L-Tryptophan
Exchange" reversible="true" fast="false" fbc:lowerFluxBound="rev_lb"
fbc:upperFluxBound="rev_ub">
            <listOfReactants>
                  <speciesReference species="M_trp__L_e" stoichiometry="1"
constant="true"/>
            </listOfReactants>
      </reaction>

      <reaction metaid="EX_tsul_e" id="EX_tsul_e" name="Thiosulfate Exchange"
reversible="true" fast="false" fbc:lowerFluxBound="rev_lb"
fbc:upperFluxBound="rev_ub">
            <listOfReactants>
                  <speciesReference species="M_tsul_e" stoichiometry="1"
constant="true"/>
            </listOfReactants>
      </reaction>

      <reaction metaid="EX_tym_e" id="EX_tym_e" name="Tyramine Exchange"
reversible="true" fast="false" fbc:lowerFluxBound="rev_lb"
fbc:upperFluxBound="rev_ub">
            <listOfReactants>
                  <speciesReference species="M_tym_e" stoichiometry="1"
constant="true"/>
            </listOfReactants>
      </reaction>

      <reaction metaid="EX_tyr__L_e" id="EX_tyr__L_e" name="L-Tyrosine
Exchange" reversible="true" fast="false" fbc:lowerFluxBound="rev_lb"
fbc:upperFluxBound="rev_ub">
            <listOfReactants>
                  <speciesReference species="M_tyr__L_e" stoichiometry="1"
constant="true"/>
            </listOfReactants>
      </reaction>

      <reaction metaid="EX_ura_e" id="EX_ura_e" name="Uracil Exchange"
reversible="true" fast="false" fbc:lowerFluxBound="rev_lb"
fbc:upperFluxBound="rev_ub">
            <listOfReactants>
                  <speciesReference species="M_ura_e" stoichiometry="1"
constant="true"/>
            </listOfReactants>
      </reaction>

      <reaction metaid="EX_urea_e" id="EX_urea_e" name="Urea Exchange"
reversible="true" fast="false" fbc:lowerFluxBound="rev_lb"
fbc:upperFluxBound="rev_ub">
            <listOfReactants>
```

```
                  <speciesReference species="M_urea_e" stoichiometry="1"
constant="true"/>
            </listOfReactants>
      </reaction>

      <reaction metaid="EX_uri_e" id="EX_uri_e" name="Uridine Exchange"
reversible="true" fast="false" fbc:lowerFluxBound="rev_lb"
fbc:upperFluxBound="rev_ub">
            <listOfReactants>
                  <speciesReference species="M_uri_e" stoichiometry="1"
constant="true"/>
            </listOfReactants>
      </reaction>

      <reaction metaid="EX_val__L_e" id="EX_val__L_e" name="L-Valine
Exchange" reversible="true" fast="false" fbc:lowerFluxBound="rev_lb"
fbc:upperFluxBound="rev_ub">
            <listOfReactants>
                  <speciesReference species="M_val__L_e" stoichiometry="1"
constant="true"/>
            </listOfReactants>
      </reaction>

      <reaction metaid="EX_xan_e" id="EX_xan_e" name="Xanthine Exchange"
reversible="true" fast="false" fbc:lowerFluxBound="rev_lb"
fbc:upperFluxBound="rev_ub">
            <listOfReactants>
                  <speciesReference species="M_xan_e" stoichiometry="1"
constant="true"/>
            </listOfReactants>
      </reaction>

      <reaction metaid="EX_xtsn_e" id="EX_xtsn_e" name="Xanthosine Exchange"
reversible="true" fast="false" fbc:lowerFluxBound="rev_lb"
fbc:upperFluxBound="rev_ub">
            <listOfReactants>
                  <speciesReference species="M_xtsn_e" stoichiometry="1"
constant="true"/>
            </listOfReactants>
      </reaction>

      <reaction metaid="EX_xyl__D_e" id="EX_xyl__D_e" name="D-Xylose
Exchange" reversible="true" fast="false" fbc:lowerFluxBound="rev_lb"
fbc:upperFluxBound="rev_ub">
            <listOfReactants>
                  <speciesReference species="M_xyl__D_e" stoichiometry="1"
constant="true"/>
            </listOfReactants>
      </reaction>

      <reaction metaid="EX_zn2_e" id="EX_zn2_e" name="Zinc Exchange"
reversible="true" fast="false" fbc:lowerFluxBound="rev_lb"
fbc:upperFluxBound="rev_ub">
            <listOfReactants>
                  <speciesReference species="M_zn2_e" stoichiometry="1"
constant="true"/>
            </listOfReactants>
      </reaction>
```

```
      <reaction metaid="R_F6Pt6_2pp" id="R_F6Pt6_2pp" name="Fructose-6-
phosphate transport via phosphate antiport (periplasm)" reversible="false"
fast="false" fbc:lowerFluxBound="irr_lb" fbc:upperFluxBound="irr_ub">
            <listOfReactants>
                  <speciesReference species="M_pi_c" stoichiometry="2"
constant="true"/>
                  <speciesReference species="M_f6p_p" stoichiometry="1"
constant="true"/>
            </listOfReactants>
            <listOfProducts>
                  <speciesReference species="M_f6p_c" stoichiometry="1"
constant="true"/>
                  <speciesReference species="M_pi_p" stoichiometry="2"
constant="true"/>
            </listOfProducts>
            <fbc:geneProductAssociation>
                  <fbc:geneProductRef fbc:geneProduct="SAUSA300_0488"/>
            </fbc:geneProductAssociation>
      </reaction>

      <reaction metaid="R_F6Ptex" id="R_F6Ptex" name="fructose 6-phosphate
transport via diffusion (extracellular to periplasm)" reversible="true"
fast="false" fbc:lowerFluxBound="rev_lb" fbc:upperFluxBound="rev_ub">
            <listOfReactants>
                  <speciesReference species="M_f6p_e" stoichiometry="1"
constant="true"/>
            </listOfReactants>
            <listOfProducts>
                  <speciesReference species="M_f6p_p" stoichiometry="1"
constant="true"/>
            </listOfProducts>
      </reaction>

      <reaction metaid="R_FACOAL140p" id="R_FACOAL140p" name="fatty-acid--CoA
ligase (tetradecanoate), peroxisomal" reversible="false" fast="false"
fbc:lowerFluxBound="irr_lb" fbc:upperFluxBound="irr_ub">
            <listOfReactants>
                  <speciesReference species="M_h_c" stoichiometry="1"
constant="true"/>
                  <speciesReference species="M_atp_c" stoichiometry="1"
constant="true"/>
                  <speciesReference species="M_coa_c" stoichiometry="1"
constant="true"/>
                  <speciesReference species="M_ttdca_c" stoichiometry="1"
constant="true"/>
            </listOfReactants>
            <listOfProducts>
                  <speciesReference species="M_ppi_c" stoichiometry="1"
constant="true"/>
                  <speciesReference species="M_amp_c" stoichiometry="1"
constant="true"/>
                  <speciesReference species="M_tdcoa_c" stoichiometry="1"
constant="true"/>
            </listOfProducts>
            <fbc:geneProductAssociation>
                  <fbc:or>
                        <fbc:geneProductRef fbc:geneProduct="SAUSA300_0228"/>
```

```
                    &lt;fbc:geneProductRef fbc:geneProduct="SAUSA300_0559"/&gt;
                &lt;/fbc:or&gt;
            &lt;/fbc:geneProductAssociation&gt;
        &lt;/reaction&gt;

        <reaction metaid="R_FACOAL150AI" id="R_FACOAL150AI"
name="FACOAL150anteiso " reversible="false" fast="false"
fbc:lowerFluxBound="irr_lb" fbc:upperFluxBound="irr_ub">
            &lt;listOfReactants&gt;
                <speciesReference species="M_h_c" stoichiometry="1"
constant="true"/>
                <speciesReference species="M_atp_c" stoichiometry="1"
constant="true"/>
                <speciesReference species="M_coa_c" stoichiometry="1"
constant="true"/>
                <speciesReference species="M_fa4_c" stoichiometry="1"
constant="true"/>
            &lt;/listOfReactants&gt;
            &lt;listOfProducts&gt;
                <speciesReference species="M_ppi_c" stoichiometry="1"
constant="true"/>
                <speciesReference species="M_amp_c" stoichiometry="1"
constant="true"/>
                <speciesReference species="M_fa4coa_c" stoichiometry="1"
constant="true"/>
            &lt;/listOfProducts&gt;
            &lt;fbc:geneProductAssociation&gt;
                &lt;fbc:or&gt;
                    &lt;fbc:geneProductRef fbc:geneProduct="SAUSA300_0228"/&gt;
                    &lt;fbc:geneProductRef fbc:geneProduct="SAUSA300_0559"/&gt;
                &lt;/fbc:or&gt;
            &lt;/fbc:geneProductAssociation&gt;
        &lt;/reaction&gt;

        <reaction metaid="R_FACOAL160" id="R_FACOAL160" name="fatty-acid--CoA
ligase (hexadecanoate)" reversible="false" fast="false"
fbc:lowerFluxBound="irr_lb" fbc:upperFluxBound="irr_ub">
            &lt;listOfReactants&gt;
                <speciesReference species="M_atp_c" stoichiometry="1"
constant="true"/>
                <speciesReference species="M_coa_c" stoichiometry="1"
constant="true"/>
                <speciesReference species="M_hdca_c" stoichiometry="1"
constant="true"/>
            &lt;/listOfReactants&gt;
            &lt;listOfProducts&gt;
                <speciesReference species="M_ppi_c" stoichiometry="1"
constant="true"/>
                <speciesReference species="M_amp_c" stoichiometry="1"
constant="true"/>
                <speciesReference species="M_pmtcoa_c" stoichiometry="1"
constant="true"/>
            &lt;/listOfProducts&gt;
            &lt;fbc:geneProductAssociation&gt;
                &lt;fbc:geneProductRef fbc:geneProduct="SAUSA300_0228"/&gt;
            &lt;/fbc:geneProductAssociation&gt;
        &lt;/reaction&gt;
```

```
      <reaction metaid="R_FACOAL160iso" id="R_FACOAL160iso"
name="FACOAL160ISO " reversible="false" fast="false"
fbc:lowerFluxBound="irr_lb" fbc:upperFluxBound="irr_ub">
            <listOfReactants>
                  <speciesReference species="M_h_c" stoichiometry="1"
constant="true"/>
                  <speciesReference species="M_atp_c" stoichiometry="1"
constant="true"/>
                  <speciesReference species="M_coa_c" stoichiometry="1"
constant="true"/>
                  <speciesReference species="M_fa6_c" stoichiometry="1"
constant="true"/>
            </listOfReactants>
            <listOfProducts>
                  <speciesReference species="M_ppi_c" stoichiometry="1"
constant="true"/>
                  <speciesReference species="M_amp_c" stoichiometry="1"
constant="true"/>
                  <speciesReference species="M_fa6coa_c" stoichiometry="1"
constant="true"/>
            </listOfProducts>
            <fbc:geneProductAssociation>
                  <fbc:or>
                        <fbc:geneProductRef
fbc:geneProduct="SA1323661_VBIStaAur292900_0569"/>
                        <fbc:geneProductRef
fbc:geneProduct="SA1323661_VBIStaAur292900_0232"/>
                        <fbc:geneProductRef
fbc:geneProduct="SA1323661_VBIStaAur292900_0185"/>
                  </fbc:or>
            </fbc:geneProductAssociation>
      </reaction>

      <reaction metaid="R_FACOALO" id="R_FACOALO" name="fatty-acid--CoA
ligase octadecanoate " reversible="false" fast="false"
fbc:lowerFluxBound="irr_lb" fbc:upperFluxBound="irr_ub">
            <listOfReactants>
                  <speciesReference species="M_h_c" stoichiometry="1"
constant="true"/>
                  <speciesReference species="M_atp_c" stoichiometry="1"
constant="true"/>
                  <speciesReference species="M_coa_c" stoichiometry="1"
constant="true"/>
                  <speciesReference species="M_ocdca_c" stoichiometry="1"
constant="true"/>
            </listOfReactants>
            <listOfProducts>
                  <speciesReference species="M_ppi_c" stoichiometry="1"
constant="true"/>
                  <speciesReference species="M_amp_c" stoichiometry="1"
constant="true"/>
                  <speciesReference species="M_stcoa_c" stoichiometry="1"
constant="true"/>
            </listOfProducts>
            <fbc:geneProductAssociation>
                  <fbc:or>
                        <fbc:geneProductRef fbc:geneProduct="SAUSA300_0228"/>
                        <fbc:geneProductRef fbc:geneProduct="SAUSA300_0559"/>
```

```
                </fbc:or>
            </fbc:geneProductAssociation>
        </reaction>

        <reaction metaid="R_FBA" id="R_FBA" name="fructose-bisphosphate
aldolase" reversible="true" fast="false" fbc:lowerFluxBound="rev_lb"
fbc:upperFluxBound="rev_ub">
            <listOfReactants>
                <speciesReference species="M_fdp_c" stoichiometry="1"
constant="true"/>
            </listOfReactants>
            <listOfProducts>
                <speciesReference species="M_dhap_c" stoichiometry="1"
constant="true"/>
                <speciesReference species="M_g3p_c" stoichiometry="1"
constant="true"/>
            </listOfProducts>
            <fbc:geneProductAssociation>
                <fbc:or>
                    <fbc:geneProductRef fbc:geneProduct="SAUSA300_2079"/>
                    <fbc:geneProductRef fbc:geneProduct="SAUSA300_2540"/>
                </fbc:or>
            </fbc:geneProductAssociation>
        </reaction>

        <reaction metaid="R_FBA2" id="R_FBA2" name="D-Fructose 1-phosphate D-
glyceraldehyde-3-phosphate__Lyase" reversible="true" fast="false"
fbc:lowerFluxBound="rev_lb" fbc:upperFluxBound="rev_ub">
            <listOfReactants>
                <speciesReference species="M_f1p_c" stoichiometry="1"
constant="true"/>
            </listOfReactants>
            <listOfProducts>
                <speciesReference species="M_dhap_c" stoichiometry="1"
constant="true"/>
                <speciesReference species="M_glyald_c" stoichiometry="1"
constant="true"/>
            </listOfProducts>
            <fbc:geneProductAssociation>
                <fbc:geneProductRef fbc:geneProduct="SAUSA300_2079"/>
            </fbc:geneProductAssociation>
        </reaction>

        <reaction metaid="R_FBA3" id="R_FBA3" name="Sedoheptulose 1,7-
bisphosphate D-glyceraldehyde-3-phosphate__Lyase" reversible="true"
fast="false" fbc:lowerFluxBound="rev_lb" fbc:upperFluxBound="rev_ub">
            <listOfReactants>
                <speciesReference species="M_s17bp_c" stoichiometry="1"
constant="true"/>
            </listOfReactants>
            <listOfProducts>
                <speciesReference species="M_dhap_c" stoichiometry="1"
constant="true"/>
                <speciesReference species="M_e4p_c" stoichiometry="1"
constant="true"/>
            </listOfProducts>
            <fbc:geneProductAssociation>
                <fbc:or>
```

```
                        <fbc:geneProductRef fbc:geneProduct="SAUSA300_2079"/>
                        <fbc:geneProductRef fbc:geneProduct="SAUSA300_2540"/>
                  </fbc:or>
            </fbc:geneProductAssociation>
      </reaction>

      <reaction metaid="R_FBP" id="R_FBP" name="fructose-bisphosphatase"
reversible="false" fast="false" fbc:lowerFluxBound="irr_lb"
fbc:upperFluxBound="irr_ub">
            <listOfReactants>
                  <speciesReference species="M_h2o_c" stoichiometry="1"
constant="true"/>
                  <speciesReference species="M_fdp_c" stoichiometry="1"
constant="true"/>
            </listOfReactants>
            <listOfProducts>
                  <speciesReference species="M_h_c" stoichiometry="1"
constant="true"/>
                  <speciesReference species="M_pi_c" stoichiometry="1"
constant="true"/>
                  <speciesReference species="M_f6p_c" stoichiometry="1"
constant="true"/>
            </listOfProducts>
            <fbc:geneProductAssociation>
                  <fbc:geneProductRef fbc:geneProduct="SAUSA300_2455"/>
            </fbc:geneProductAssociation>
      </reaction>

      <reaction metaid="R_FCLT" id="R_FCLT" name="Ferrochelatase"
reversible="false" fast="false" fbc:lowerFluxBound="irr_lb"
fbc:upperFluxBound="irr_ub">
            <listOfReactants>
                  <speciesReference species="M_ppp9_c" stoichiometry="1"
constant="true"/>
                  <speciesReference species="M_fe2_c" stoichiometry="1"
constant="true"/>
            </listOfReactants>
            <listOfProducts>
                  <speciesReference species="M_h_c" stoichiometry="2"
constant="true"/>
                  <speciesReference species="M_pheme_c" stoichiometry="1"
constant="true"/>
            </listOfProducts>
            <fbc:geneProductAssociation>
                  <fbc:geneProductRef fbc:geneProduct="SAUSA300_1782"/>
            </fbc:geneProductAssociation>
      </reaction>

      <reaction metaid="R_FDFP" id="R_FDFP" name="Farnesyl__Diphosphate
farnesyl__Diphosphate farnesyltransferase " reversible="true" fast="false"
fbc:lowerFluxBound="rev_lb" fbc:upperFluxBound="rev_ub">
            <listOfReactants>
                  <speciesReference species="M_frdp_c" stoichiometry="2"
constant="true"/>
            </listOfReactants>
            <listOfProducts>
                  <speciesReference species="M_ppi_c" stoichiometry="1"
constant="true"/>
```

```
                    <speciesReference species="M_psqldp_c" stoichiometry="1"
constant="true"/>
            </listOfProducts>
            <fbc:geneProductAssociation>
                  <fbc:geneProductRef fbc:geneProduct="SAUSA300_2499"/>
            </fbc:geneProductAssociation>
      </reaction>

      <reaction metaid="R_FDH" id="R_FDH" name="formate dehydrogenase"
reversible="false" fast="false" fbc:lowerFluxBound="irr_lb"
fbc:upperFluxBound="irr_ub">
            <listOfReactants>
                  <speciesReference species="M_nad_c" stoichiometry="1"
constant="true"/>
                  <speciesReference species="M_for_c" stoichiometry="1"
constant="true"/>
            </listOfReactants>
            <listOfProducts>
                  <speciesReference species="M_co2_c" stoichiometry="1"
constant="true"/>
                  <speciesReference species="M_nadh_c" stoichiometry="1"
constant="true"/>
            </listOfProducts>
            <fbc:geneProductAssociation>
                  <fbc:or>
                        <fbc:geneProductRef fbc:geneProduct="SAUSA300_2258"/>
                        <fbc:geneProductRef fbc:geneProduct="SAUSA300_2231"/>
                        <fbc:geneProductRef fbc:geneProduct="SAUSA300_0179"/>
                  </fbc:or>
            </fbc:geneProductAssociation>
      </reaction>

      <reaction metaid="R_FE2abc" id="R_FE2abc" name="iron (II) transport via
ABC system" reversible="false" fast="false" fbc:lowerFluxBound="irr_lb"
fbc:upperFluxBound="irr_ub">
            <listOfReactants>
                  <speciesReference species="M_h2o_c" stoichiometry="1"
constant="true"/>
                  <speciesReference species="M_atp_c" stoichiometry="1"
constant="true"/>
                  <speciesReference species="M_fe2_e" stoichiometry="1"
constant="true"/>
            </listOfReactants>
            <listOfProducts>
                  <speciesReference species="M_h_c" stoichiometry="1"
constant="true"/>
                  <speciesReference species="M_pi_c" stoichiometry="1"
constant="true"/>
                  <speciesReference species="M_adp_c" stoichiometry="1"
constant="true"/>
                  <speciesReference species="M_fe2_c" stoichiometry="1"
constant="true"/>
            </listOfProducts>
            <fbc:geneProductAssociation>
                  <fbc:or>
                        <fbc:geneProductRef fbc:geneProduct="SAUSA300_2487"/>
                        <fbc:geneProductRef fbc:geneProduct="SAUSA300_2520"/>
                        <fbc:geneProductRef fbc:geneProduct="SAUSA300_2488"/>
```

```
                        <fbc:and>
                              <fbc:geneProductRef
fbc:geneProduct="SAUSA300_0117"/>
                              <fbc:geneProductRef
fbc:geneProduct="SAUSA300_0116"/>
                              <fbc:geneProductRef
fbc:geneProduct="SAUSA300_0115"/>
                              <fbc:geneProductRef
fbc:geneProduct="SAUSA300_0633"/>
                        </fbc:and>
                        <fbc:and>
                              <fbc:geneProductRef
fbc:geneProduct="SAUSA300_2136"/>
                              <fbc:geneProductRef
fbc:geneProduct="SAUSA300_2135"/>
                              <fbc:geneProductRef
fbc:geneProduct="SAUSA300_2134"/>
                              <fbc:geneProductRef
fbc:geneProduct="SAUSA300_0633"/>
                        </fbc:and>
                  </fbc:or>
            </fbc:geneProductAssociation>
      </reaction>

      <reaction metaid="R_FE3DCITabc" id="R_FE3DCITabc" name="Iron transport
from ferric__Dicitrate via ABC system" reversible="false" fast="false"
fbc:lowerFluxBound="irr_lb" fbc:upperFluxBound="irr_ub">
            <listOfReactants>
                  <speciesReference species="M_atp_c" stoichiometry="1"
constant="true"/>
                  <speciesReference species="M_fe3dcit_e" stoichiometry="1"
constant="true"/>
                  <speciesReference species="M_h2o_c" stoichiometry="1"
constant="true"/>
            </listOfReactants>
            <listOfProducts>
                  <speciesReference species="M_adp_c" stoichiometry="1"
constant="true"/>
                  <speciesReference species="M_cit_c" stoichiometry="2"
constant="true"/>
                  <speciesReference species="M_fe3_c" stoichiometry="1"
constant="true"/>
                  <speciesReference species="M_h_c" stoichiometry="1"
constant="true"/>
                  <speciesReference species="M_pi_c" stoichiometry="1"
constant="true"/>
            </listOfProducts>
            <fbc:geneProductAssociation>
                  <fbc:and>
                        <fbc:geneProductRef fbc:geneProduct="SAUSA300_0115"/>
                        <fbc:geneProductRef fbc:geneProduct="SAUSA300_0116"/>
                        <fbc:geneProductRef fbc:geneProduct="SAUSA300_0117"/>
                  </fbc:and>
            </fbc:geneProductAssociation>
      </reaction>
```

```
      <reaction metaid="R_FE3tex" id="R_FE3tex" name="iron (III) transport"
reversible="true" fast="false" fbc:lowerFluxBound="rev_lb"
fbc:upperFluxBound="rev_ub">
            <listOfReactants>
                  <speciesReference species="M_fe3_e" stoichiometry="1"
constant="true"/>
            </listOfReactants>
            <listOfProducts>
                  <speciesReference species="M_fe3_c" stoichiometry="1"
constant="true"/>
            </listOfProducts>
            <fbc:geneProductAssociation>
                  <fbc:and>
                        <fbc:geneProductRef fbc:geneProduct="SAUSA300_0345"/>
                        <fbc:geneProductRef fbc:geneProduct="SAUSA300_0344"/>
                  </fbc:and>
            </fbc:geneProductAssociation>
      </reaction>

      <reaction metaid="R_FECPP3O" id="R_FECPP3O" name="Fe-coproporphyrin III
oxidase/dehydrogenase" reversible="false" fast="false"
fbc:lowerFluxBound="irr_lb" fbc:upperFluxBound="irr_ub">
            <listOfReactants>
                  <speciesReference species="M_amet_c" stoichiometry="2.0"
constant="true"/>
                  <speciesReference species="M_fecpp3_c" stoichiometry="1"
constant="true"/>
            </listOfReactants>
            <listOfProducts>
                  <speciesReference species="M_co2_c" stoichiometry="2.0"
constant="true"/>
                  <speciesReference species="M_dad__5_c" stoichiometry="2.0"
constant="true"/>
                  <speciesReference species="M_h_c" stoichiometry="4.0"
constant="true"/>
                  <speciesReference species="M_met__L_c" stoichiometry="2.0"
constant="true"/>
                  <speciesReference species="M_pheme_c" stoichiometry="1"
constant="true"/>
            </listOfProducts>
            <fbc:geneProductAssociation>
                  <fbc:geneProductRef fbc:geneProduct="SAUSA300_0569"/>
            </fbc:geneProductAssociation>
      </reaction>

      <reaction metaid="R_FECRMabc" id="R_FECRMabc" name="Ferrichrome ABC
transport " reversible="false" fast="false" fbc:lowerFluxBound="irr_lb"
fbc:upperFluxBound="irr_ub">
            <listOfReactants>
                  <speciesReference species="M_h2o_c" stoichiometry="1"
constant="true"/>
                  <speciesReference species="M_atp_c" stoichiometry="1"
constant="true"/>
                  <speciesReference species="M_fecrm_e" stoichiometry="1"
constant="true"/>
            </listOfReactants>
            <listOfProducts>
```

```
                  <speciesReference species="M_h_c" stoichiometry="1"
constant="true"/>
                  <speciesReference species="M_pi_c" stoichiometry="1"
constant="true"/>
                  <speciesReference species="M_adp_c" stoichiometry="1"
constant="true"/>
                  <speciesReference species="M_fecrm_c" stoichiometry="1"
constant="true"/>
            </listOfProducts>
        <fbc:geneProductAssociation>
                  <fbc:or>
                      <fbc:and>
                          <fbc:or>
                              <fbc:geneProductRef
fbc:geneProduct="SAUSA300_0719"/>
                              <fbc:geneProductRef
fbc:geneProduct="SAUSA300_0718"/>
                          </fbc:or>
                          <fbc:geneProductRef
fbc:geneProduct="SAUSA300_0721"/>
                          <fbc:geneProductRef
fbc:geneProduct="SAUSA300_0720"/>
                      </fbc:and>
                      <fbc:and>
                          <fbc:geneProductRef
fbc:geneProduct="SAUSA300_2134"/>
                          <fbc:geneProductRef
fbc:geneProduct="SAUSA300_2135"/>
                      </fbc:and>
                      <fbc:geneProductRef fbc:geneProduct="SAUSA300_0941"/>
                      <fbc:geneProductRef fbc:geneProduct="SAUSA300_2136"/>
                  </fbc:or>
            </fbc:geneProductAssociation>
      </reaction>

      <reaction metaid="R_FERO" id="R_FERO" name="FeII oxygen oxidoreductase
" reversible="true" fast="false" fbc:lowerFluxBound="rev_lb"
fbc:upperFluxBound="rev_ub">
            <listOfReactants>
                  <speciesReference species="M_h_c" stoichiometry="4"
constant="true"/>
                  <speciesReference species="M_o2_c" stoichiometry="1"
constant="true"/>
                  <speciesReference species="M_fe2_c" stoichiometry="4"
constant="true"/>
            </listOfReactants>
            <listOfProducts>
                  <speciesReference species="M_h2o_c" stoichiometry="2"
constant="true"/>
                  <speciesReference species="M_fe3_c" stoichiometry="4"
constant="true"/>
            </listOfProducts>
            <fbc:geneProductAssociation>
                  <fbc:geneProductRef fbc:geneProduct="SAUSA300_2092"/>
            </fbc:geneProductAssociation>
      </reaction>
```

```
      <reaction metaid="R_FF_10" id="R_FF_10"
name="isotetradecanoyl__Lipoteichoic acid synthesis n=24  unlinked  D-alanine
substituted " reversible="false" fast="false" fbc:lowerFluxBound="irr_lb"
fbc:upperFluxBound="irr_ub">
            <listOfReactants>
                  <speciesReference species="M_atp_c" stoichiometry="24"
constant="true"/>
                  <speciesReference species="M_ala__D_c" stoichiometry="24"
constant="true"/>
                  <speciesReference species="M_isot24u_c" stoichiometry="1"
constant="true"/>
            </listOfReactants>
            <listOfProducts>
                  <speciesReference species="M_ppi_c" stoichiometry="24"
constant="true"/>
                  <speciesReference species="M_amp_c" stoichiometry="24"
constant="true"/>
                  <speciesReference species="M_isot24ds_c" stoichiometry="1"
constant="true"/>
            </listOfProducts>
            <fbc:geneProductAssociation>
                  <fbc:geneProductRef fbc:geneProduct="SAUSA300_0838"/>
            </fbc:geneProductAssociation>
      </reaction>

      <reaction metaid="R_FF_11" id="R_FF_11" name="copper transport via
proton antiport " reversible="false" fast="false" fbc:lowerFluxBound="irr_lb"
fbc:upperFluxBound="irr_ub">
            <listOfReactants>
                  <speciesReference species="M_h_c" stoichiometry="1"
constant="true"/>
                  <speciesReference species="M_cu2_e" stoichiometry="1"
constant="true"/>
            </listOfReactants>
            <listOfProducts>
                  <speciesReference species="M_h_e" stoichiometry="1"
constant="true"/>
                  <speciesReference species="M_cu2_c" stoichiometry="1"
constant="true"/>
            </listOfProducts>
            <fbc:geneProductAssociation>
                  <fbc:geneProductRef fbc:geneProduct="SAUSA300_2494"/>
            </fbc:geneProductAssociation>
      </reaction>

      <reaction metaid="R_FF_16" id="R_FF_16" name="L-cysteine ThiI
sulfurtransferase " reversible="true" fast="false"
fbc:lowerFluxBound="rev_lb" fbc:upperFluxBound="rev_ub">
            <listOfReactants>
                  <speciesReference species="M_cys__L_c" stoichiometry="1"
constant="true"/>
                  <speciesReference species="M_c15811_c" stoichiometry="1"
constant="true"/>
            </listOfReactants>
            <listOfProducts>
                  <speciesReference species="M_ala__L_c" stoichiometry="1"
constant="true"/>
```

```
                  <speciesReference species="M_c15812_c" stoichiometry="1"
constant="true"/>
            </listOfProducts>
            <fbc:geneProductAssociation>
                  <fbc:or>
                        <fbc:geneProductRef fbc:geneProduct="SAUSA300_1662"/>
                        <fbc:geneProductRef fbc:geneProduct="SAUSA300_1579"/>
                  </fbc:or>
            </fbc:geneProductAssociation>
      </reaction>

      <reaction metaid="R_FF_17" id="R_FF_17" name="Dethiobiotin sulfur
sulfurtransferase " reversible="false" fast="false"
fbc:lowerFluxBound="irr_lb" fbc:upperFluxBound="irr_ub">
            <listOfReactants>
                  <speciesReference species="M_amet_c" stoichiometry="2"
constant="true"/>
                  <speciesReference species="M_dtbt_c" stoichiometry="1"
constant="true"/>
                  <speciesReference species="M_s_c" stoichiometry="1"
constant="true"/>
            </listOfReactants>
            <listOfProducts>
                  <speciesReference species="M_met__L_c" stoichiometry="2"
constant="true"/>
                  <speciesReference species="M_btn_c" stoichiometry="1"
constant="true"/>
                  <speciesReference species="M_dad__5_c" stoichiometry="2"
constant="true"/>
            </listOfProducts>
            <fbc:geneProductAssociation>
                  <fbc:geneProductRef fbc:geneProduct="SAUSA300_2371"/>
            </fbc:geneProductAssociation>
      </reaction>

      <reaction metaid="R_FF_18" id="R_FF_18" name="palmitoyl__Lipoteichoic
acid synthesis n=24  unlinked  D-alanine substituted " reversible="false"
fast="false" fbc:lowerFluxBound="irr_lb" fbc:upperFluxBound="irr_ub">
            <listOfReactants>
                  <speciesReference species="M_atp_c" stoichiometry="24"
constant="true"/>
                  <speciesReference species="M_ala__D_c" stoichiometry="24"
constant="true"/>
                  <speciesReference species="M_palm24u_c" stoichiometry="1"
constant="true"/>
            </listOfReactants>
            <listOfProducts>
                  <speciesReference species="M_ppi_c" stoichiometry="24"
constant="true"/>
                  <speciesReference species="M_amp_c" stoichiometry="24"
constant="true"/>
                  <speciesReference species="M_palm24alas_c"
stoichiometry="1" constant="true"/>
            </listOfProducts>
            <fbc:geneProductAssociation>
                  <fbc:geneProductRef fbc:geneProduct="SAUSA300_0838"/>
            </fbc:geneProductAssociation>
      </reaction>
```

```
      <reaction metaid="R_FF_19" id="R_FF_19"
name="isopentadecanoyl__Lipoteichoic acid synthesis n=24  unlinked  D-alanine
substituted " reversible="false" fast="false" fbc:lowerFluxBound="irr_lb"
fbc:upperFluxBound="irr_ub">
            <listOfReactants>
                  <speciesReference species="M_atp_c" stoichiometry="24"
constant="true"/>
                  <speciesReference species="M_ala__D_c" stoichiometry="24"
constant="true"/>
                  <speciesReference species="M_isop24u_c" stoichiometry="1"
constant="true"/>
            </listOfReactants>
            <listOfProducts>
                  <speciesReference species="M_ppi_c" stoichiometry="24"
constant="true"/>
                  <speciesReference species="M_amp_c" stoichiometry="24"
constant="true"/>
                  <speciesReference species="M_isop24ds_c" stoichiometry="1"
constant="true"/>
            </listOfProducts>
            <fbc:geneProductAssociation>
                  <fbc:geneProductRef fbc:geneProduct="SAUSA300_0838"/>
            </fbc:geneProductAssociation>
      </reaction>

      <reaction metaid="R_FF_2" id="R_FF_2" name="myristoyl__Lipoteichoic
acid synthesis n=24  unlinked  D-alanine substituted " reversible="false"
fast="false" fbc:lowerFluxBound="irr_lb" fbc:upperFluxBound="irr_ub">
            <listOfReactants>
                  <speciesReference species="M_atp_c" stoichiometry="24"
constant="true"/>
                  <speciesReference species="M_ala__D_c" stoichiometry="24"
constant="true"/>
                  <speciesReference species="M_myrs24u_c" stoichiometry="1"
constant="true"/>
            </listOfReactants>
            <listOfProducts>
                  <speciesReference species="M_h_c" stoichiometry="2"
constant="true"/>
                  <speciesReference species="M_ppi_c" stoichiometry="24"
constant="true"/>
                  <speciesReference species="M_amp_c" stoichiometry="24"
constant="true"/>
                  <speciesReference species="M_my424ds_c" stoichiometry="1"
constant="true"/>
            </listOfProducts>
            <fbc:geneProductAssociation>
                  <fbc:geneProductRef fbc:geneProduct="SAUSA300_0838"/>
            </fbc:geneProductAssociation>
      </reaction>

      <reaction metaid="R_FF_21" id="R_FF_21" name="Pyruvate ferricytochrome-
b1 oxidoreductase " reversible="true" fast="false"
fbc:lowerFluxBound="rev_lb" fbc:upperFluxBound="rev_ub">
            <listOfReactants>
                  <speciesReference species="M_h2o_c" stoichiometry="1"
constant="true"/>
```

```
                  <speciesReference species="M_pyr_c" stoichiometry="1"
constant="true"/>
                  <speciesReference species="M_ficytcb1_c" stoichiometry="1"
constant="true"/>
            </listOfReactants>
            <listOfProducts>
                  <speciesReference species="M_h_c" stoichiometry="2"
constant="true"/>
                  <speciesReference species="M_ac_c" stoichiometry="1"
constant="true"/>
                  <speciesReference species="M_co2_c" stoichiometry="1"
constant="true"/>
                  <speciesReference species="M_focytcb1_c" stoichiometry="1"
constant="true"/>
            </listOfProducts>
            <fbc:geneProductAssociation>
                  <fbc:geneProductRef fbc:geneProduct="SAUSA300_2477"/>
            </fbc:geneProductAssociation>
      </reaction>

      <reaction metaid="R_FF_26" id="R_FF_26" name="arbutin transport via PEP
Pyr PTS " reversible="true" fast="false" fbc:lowerFluxBound="rev_lb"
fbc:upperFluxBound="rev_ub">
            <listOfReactants>
                  <speciesReference species="M_pep_c" stoichiometry="1"
constant="true"/>
                  <speciesReference species="M_arbt_e" stoichiometry="1"
constant="true"/>
            </listOfReactants>
            <listOfProducts>
                  <speciesReference species="M_pyr_c" stoichiometry="1"
constant="true"/>
                  <speciesReference species="M_arbt6p_c" stoichiometry="1"
constant="true"/>
            </listOfProducts>
            <fbc:geneProductAssociation>
                  <fbc:and>
                        <fbc:geneProductRef fbc:geneProduct="SAUSA300_0983"/>
                        <fbc:geneProductRef fbc:geneProduct="SAUSA300_0984"/>
                  </fbc:and>
            </fbc:geneProductAssociation>
      </reaction>

      <reaction metaid="R_FF_31" id="R_FF_31" name="NADH ubiquinone
oxidoreductase " reversible="true" fast="false" fbc:lowerFluxBound="rev_lb"
fbc:upperFluxBound="rev_ub">
            <listOfReactants>
                  <speciesReference species="M_nadh_c" stoichiometry="1"
constant="true"/>
                  <speciesReference species="M_q_c" stoichiometry="1"
constant="true"/>
            </listOfReactants>
            <listOfProducts>
                  <speciesReference species="M_h_c" stoichiometry="1"
constant="true"/>
                  <speciesReference species="M_nad_c" stoichiometry="1"
constant="true"/>
```

```
                  <speciesReference species="M_qh2_c" stoichiometry="1"
constant="true"/>
            </listOfProducts>
            <fbc:geneProductAssociation>
                  <fbc:or>
                        <fbc:geneProductRef fbc:geneProduct="SAUSA300_0844"/>
                        <fbc:geneProductRef fbc:geneProduct="SAUSA300_0841"/>
                  </fbc:or>
            </fbc:geneProductAssociation>
      </reaction>

      <reaction metaid="R_FF_36" id="R_FF_36" name="isohexadecanoyl-
phosphatidic acid phosphatase " reversible="false" fast="false"
fbc:lowerFluxBound="irr_lb" fbc:upperFluxBound="irr_ub">
            <listOfReactants>
                  <speciesReference species="M_h2o_c" stoichiometry="1"
constant="true"/>
                  <speciesReference species="M_12dhsgly3p_c"
stoichiometry="1" constant="true"/>
            </listOfReactants>
            <listOfProducts>
                  <speciesReference species="M_h_c" stoichiometry="1"
constant="true"/>
                  <speciesReference species="M_pi_c" stoichiometry="1"
constant="true"/>
                  <speciesReference species="M_12dihexsgly_c"
stoichiometry="1" constant="true"/>
            </listOfProducts>
            <fbc:geneProductAssociation>
                  <fbc:geneProductRef fbc:geneProduct="SAUSA300_1529"/>
            </fbc:geneProductAssociation>
      </reaction>

      <reaction metaid="R_FF_37" id="R_FF_37" name="anteisopentadecanoyl-
phosphatidic acid phosphatase " reversible="false" fast="false"
fbc:lowerFluxBound="irr_lb" fbc:upperFluxBound="irr_ub">
            <listOfReactants>
                  <speciesReference species="M_h2o_c" stoichiometry="1"
constant="true"/>
                  <speciesReference species="M_12diasn3_c" stoichiometry="1"
constant="true"/>
            </listOfReactants>
            <listOfProducts>
                  <speciesReference species="M_h_c" stoichiometry="1"
constant="true"/>
                  <speciesReference species="M_pi_c" stoichiometry="1"
constant="true"/>
                  <speciesReference species="M_12diasglyc_c"
stoichiometry="1" constant="true"/>
            </listOfProducts>
            <fbc:geneProductAssociation>
                  <fbc:geneProductRef fbc:geneProduct="SAUSA300_1529"/>
            </fbc:geneProductAssociation>
      </reaction>

      <reaction metaid="R_FF_38" id="R_FF_38" name="isopentadecanoyl-
phosphatidic acid phosphatase " reversible="false" fast="false"
fbc:lowerFluxBound="irr_lb" fbc:upperFluxBound="irr_ub">
```

```
            <listOfReactants>
                  <speciesReference species="M_h2o_c" stoichiometry="1"
constant="true"/>
                  <speciesReference species="M_12dpsgly3p_c"
stoichiometry="1" constant="true"/>
            </listOfReactants>
            <listOfProducts>
                  <speciesReference species="M_h_c" stoichiometry="1"
constant="true"/>
                  <speciesReference species="M_pi_c" stoichiometry="1"
constant="true"/>
                  <speciesReference species="M_12dpgly_c" stoichiometry="1"
constant="true"/>
            </listOfProducts>
            <fbc:geneProductAssociation>
                  <fbc:geneProductRef fbc:geneProduct="SAUSA300_1529"/>
            </fbc:geneProductAssociation>
      </reaction>

      <reaction metaid="R_FF_39" id="R_FF_39" name="isotetradecanoyl-
phosphatidic acid phosphatase " reversible="false" fast="false"
fbc:lowerFluxBound="irr_lb" fbc:upperFluxBound="irr_ub">
            <listOfReactants>
                  <speciesReference species="M_h2o_c" stoichiometry="1"
constant="true"/>
                  <speciesReference species="M_12diidgly3p_c"
stoichiometry="1" constant="true"/>
            </listOfReactants>
            <listOfProducts>
                  <speciesReference species="M_h_c" stoichiometry="1"
constant="true"/>
                  <speciesReference species="M_pi_c" stoichiometry="1"
constant="true"/>
                  <speciesReference species="M_12ditetgly_c"
stoichiometry="1" constant="true"/>
            </listOfProducts>
            <fbc:geneProductAssociation>
                  <fbc:geneProductRef fbc:geneProduct="SAUSA300_1529"/>
            </fbc:geneProductAssociation>
      </reaction>

      <reaction metaid="R_FF_41" id="R_FF_41" name="Succinate ubiquinone
oxidoreductase " reversible="false" fast="false" fbc:lowerFluxBound="irr_lb"
fbc:upperFluxBound="irr_ub">
            <listOfReactants>
                  <speciesReference species="M_succ_c" stoichiometry="1"
constant="true"/>
                  <speciesReference species="M_q_c" stoichiometry="1"
constant="true"/>
            </listOfReactants>
            <listOfProducts>
                  <speciesReference species="M_fum_c" stoichiometry="1"
constant="true"/>
                  <speciesReference species="M_qh2_c" stoichiometry="1"
constant="true"/>
            </listOfProducts>
            <fbc:geneProductAssociation>
                  <fbc:geneProductRef fbc:geneProduct="SAUSA300_1047"/>
```

```
			</fbc:geneProductAssociation>
		</reaction>

		<reaction metaid="R_FF_42" id="R_FF_42" name="teichuronic acid n=45
unlinked  GalNAc-GlcA repeated " reversible="true" fast="false"
fbc:lowerFluxBound="rev_lb" fbc:upperFluxBound="rev_ub">
			<listOfReactants>
				<speciesReference species="M_udpacgal_c" stoichiometry="45"
constant="true"/>
				<speciesReference species="M_udpglcur_c" stoichiometry="45"
constant="true"/>
			</listOfReactants>
			<listOfProducts>
				<speciesReference species="M_h_c" stoichiometry="45"
constant="true"/>
				<speciesReference species="M_udp_c" stoichiometry="45"
constant="true"/>
				<speciesReference species="M_ump_c" stoichiometry="45"
constant="true"/>
				<speciesReference species="M_teich__45_c" stoichiometry="1"
constant="true"/>
			</listOfProducts>
			<fbc:geneProductAssociation>
				<fbc:geneProductRef fbc:geneProduct="SAUSA300_0133"/>
			</fbc:geneProductAssociation>
		</reaction>

		<reaction metaid="R_FF_5" id="R_FF_5"
name="isohexadecanoyl__Lipoteichoic acid synthesis n=24  unlinked  D-alanine
substituted " reversible="false" fast="false" fbc:lowerFluxBound="irr_lb"
fbc:upperFluxBound="irr_ub">
			<listOfReactants>
				<speciesReference species="M_atp_c" stoichiometry="24"
constant="true"/>
				<speciesReference species="M_ala__D_c" stoichiometry="24"
constant="true"/>
				<speciesReference species="M_isohdt24u_c" stoichiometry="1"
constant="true"/>
			</listOfReactants>
			<listOfProducts>
				<speciesReference species="M_ppi_c" stoichiometry="24"
constant="true"/>
				<speciesReference species="M_amp_c" stoichiometry="24"
constant="true"/>
				<speciesReference species="M_isoh24ds_c" stoichiometry="1"
constant="true"/>
			</listOfProducts>
			<fbc:geneProductAssociation>
				<fbc:geneProductRef fbc:geneProduct="SAUSA300_0838"/>
			</fbc:geneProductAssociation>
		</reaction>

		<reaction metaid="R_FF_56" id="R_FF_56" name="acyl-CoA sn-glycerol-3-
phosphate 1-O-acyltransferase " reversible="true" fast="false"
fbc:lowerFluxBound="rev_lb" fbc:upperFluxBound="rev_ub">
			<listOfReactants>
				<speciesReference species="M_glyc3p_c" stoichiometry="1"
constant="true"/>
```

```
                  <speciesReference species="M_acoa_c" stoichiometry="1"
constant="true"/>
            </listOfReactants>
            <listOfProducts>
                  <speciesReference species="M_coa_c" stoichiometry="1"
constant="true"/>
                  <speciesReference species="M_1ag3p_c" stoichiometry="1"
constant="true"/>
            </listOfProducts>
            <fbc:geneProductAssociation>
                  <fbc:or>
                        <fbc:geneProductRef fbc:geneProduct="SAUSA300_1249"/>
                        <fbc:geneProductRef fbc:geneProduct="SAUSA300_1122"/>
                        <fbc:geneProductRef fbc:geneProduct="SAUSA300_1121"/>
                  </fbc:or>
            </fbc:geneProductAssociation>
      </reaction>

      <reaction metaid="R_FF_7" id="R_FF_7" name="Unbranched
phosphatidylethanolamine synthesis " reversible="true" fast="false"
fbc:lowerFluxBound="rev_lb" fbc:upperFluxBound="rev_ub">
            <listOfReactants>
                  <speciesReference species="M_pe140_c"
stoichiometry="0.0005249" constant="true"/>
                  <speciesReference species="M_pe160_c"
stoichiometry="0.0004824" constant="true"/>
                  <speciesReference species="M_pe180_c"
stoichiometry="0.0004462" constant="true"/>
            </listOfReactants>
            <listOfProducts>
                  <speciesReference species="M_ubpeth_c" stoichiometry="1"
constant="true"/>
            </listOfProducts>
            <fbc:geneProductAssociation>
                  <fbc:geneProductRef fbc:geneProduct="SAUSA300_1973"/>
            </fbc:geneProductAssociation>
      </reaction>

      <reaction metaid="R_FF_8" id="R_FF_8" name="Nitrite acceptor
oxidoreductase " reversible="true" fast="false" fbc:lowerFluxBound="rev_lb"
fbc:upperFluxBound="rev_ub">
            <listOfReactants>
                  <speciesReference species="M_no2_c" stoichiometry="1"
constant="true"/>
                  <speciesReference species="M_ficytcc553_c"
stoichiometry="1" constant="true"/>
            </listOfReactants>
            <listOfProducts>
                  <speciesReference species="M_no3_c" stoichiometry="1"
constant="true"/>
                  <speciesReference species="M_focytc_c" stoichiometry="1"
constant="true"/>
            </listOfProducts>
            <fbc:geneProductAssociation>
                  <fbc:or>
                        <fbc:geneProductRef fbc:geneProduct="SAUSA300_2343"/>
                        <fbc:geneProductRef fbc:geneProduct="SAUSA300_2342"/>
                        <fbc:geneProductRef fbc:geneProduct="SAUSA300_2341"/>
```

```
                        <fbc:geneProductRef fbc:geneProduct="SAUSA300_2340"/>
                  </fbc:or>
            </fbc:geneProductAssociation>
      </reaction>

      <reaction metaid="R_FF_9" id="R_FF_9"
name="anteisopentadecanoyl__Lipoteichoic acid synthesis n=24  unlinked  D-
alanine substituted " reversible="false" fast="false"
fbc:lowerFluxBound="irr_lb" fbc:upperFluxBound="irr_ub">
            <listOfReactants>
                  <speciesReference species="M_h_c" stoichiometry="1"
constant="true"/>
                  <speciesReference species="M_atp_c" stoichiometry="24"
constant="true"/>
                  <speciesReference species="M_ala__D_c" stoichiometry="24"
constant="true"/>
                  <speciesReference species="M_antp24u_c" stoichiometry="1"
constant="true"/>
            </listOfReactants>
            <listOfProducts>
                  <speciesReference species="M_ppi_c" stoichiometry="24"
constant="true"/>
                  <speciesReference species="M_amp_c" stoichiometry="24"
constant="true"/>
                  <speciesReference species="M_antl24ds_c" stoichiometry="1"
constant="true"/>
            </listOfProducts>
            <fbc:geneProductAssociation>
                  <fbc:geneProductRef fbc:geneProduct="SAUSA300_0838"/>
            </fbc:geneProductAssociation>
      </reaction>

      <reaction metaid="R_FFSD" id="R_FFSD" name="beta-fructofuranosidase"
reversible="false" fast="false" fbc:lowerFluxBound="irr_lb"
fbc:upperFluxBound="irr_ub">
            <listOfReactants>
                  <speciesReference species="M_h2o_c" stoichiometry="1"
constant="true"/>
                  <speciesReference species="M_suc6p_c" stoichiometry="1"
constant="true"/>
            </listOfReactants>
            <listOfProducts>
                  <speciesReference species="M_g6p_c" stoichiometry="1"
constant="true"/>
                  <speciesReference species="M_fru_c" stoichiometry="1"
constant="true"/>
            </listOfProducts>
            <fbc:geneProductAssociation>
                  <fbc:geneProductRef fbc:geneProduct="SAUSA300_1994"/>
            </fbc:geneProductAssociation>
      </reaction>

      <reaction metaid="R_FGLU" id="R_FGLU" name="formimidoylglutamase"
reversible="false" fast="false" fbc:lowerFluxBound="irr_lb"
fbc:upperFluxBound="irr_ub">
            <listOfReactants>
                  <speciesReference species="M_glu__L_c" stoichiometry="1"
constant="true"/>
```

```
                  <speciesReference species="M_frmd_c" stoichiometry="1"
constant="true"/>
            </listOfReactants>
            <listOfProducts>
                  <speciesReference species="M_h2o_c" stoichiometry="1"
constant="true"/>
                  <speciesReference species="M_forglu_c" stoichiometry="1"
constant="true"/>
            </listOfProducts>
            <fbc:geneProductAssociation>
                  <fbc:geneProductRef fbc:geneProduct="SAUSA300_2281"/>
            </fbc:geneProductAssociation>
      </reaction>

      <reaction metaid="R_FLDO" id="R_FLDO" name="NAD(P)H-flavin
oxidoreductase" reversible="false" fast="false" fbc:lowerFluxBound="irr_lb"
fbc:upperFluxBound="irr_ub">
            <listOfReactants>
                  <speciesReference species="M_h_c" stoichiometry="1"
constant="true"/>
                  <speciesReference species="M_nadph_c" stoichiometry="1"
constant="true"/>
                  <speciesReference species="M_fmn_c" stoichiometry="1"
constant="true"/>
            </listOfReactants>
            <listOfProducts>
                  <speciesReference species="M_nadp_c" stoichiometry="1"
constant="true"/>
                  <speciesReference species="M_fmnh2_c" stoichiometry="1"
constant="true"/>
            </listOfProducts>
            <fbc:geneProductAssociation>
                  <fbc:or>
                        <fbc:geneProductRef fbc:geneProduct="SAUSA300_0340"/>
                        <fbc:geneProductRef fbc:geneProduct="SAUSA300_0545"/>
                  </fbc:or>
            </fbc:geneProductAssociation>
      </reaction>

      <reaction metaid="R_FLVR" id="R_FLVR" name="flavin reductase"
reversible="false" fast="false" fbc:lowerFluxBound="irr_lb"
fbc:upperFluxBound="irr_ub">
            <listOfReactants>
                  <speciesReference species="M_h_c" stoichiometry="1"
constant="true"/>
                  <speciesReference species="M_nadph_c" stoichiometry="1"
constant="true"/>
                  <speciesReference species="M_ribflv_c" stoichiometry="1"
constant="true"/>
            </listOfReactants>
            <listOfProducts>
                  <speciesReference species="M_nadp_c" stoichiometry="1"
constant="true"/>
                  <speciesReference species="M_rbflvrd_c" stoichiometry="1"
constant="true"/>
            </listOfProducts>
            <fbc:geneProductAssociation>
                  <fbc:or>
```

```
                        <fbc:geneProductRef fbc:geneProduct="SAUSA300_2462"/>
                        <fbc:geneProductRef fbc:geneProduct="SAUSA300_0322"/>
                  </fbc:or>
            </fbc:geneProductAssociation>
      </reaction>

      <reaction metaid="R_FLVRx" id="R_FLVRx" name="flavin reductase (NAD)"
reversible="false" fast="false" fbc:lowerFluxBound="irr_lb"
fbc:upperFluxBound="irr_ub">
            <listOfReactants>
                  <speciesReference species="M_h_c" stoichiometry="1"
constant="true"/>
                  <speciesReference species="M_nadh_c" stoichiometry="1"
constant="true"/>
                  <speciesReference species="M_ribflv_c" stoichiometry="1"
constant="true"/>
            </listOfReactants>
            <listOfProducts>
                  <speciesReference species="M_nad_c" stoichiometry="1"
constant="true"/>
                  <speciesReference species="M_rbflvrd_c" stoichiometry="1"
constant="true"/>
            </listOfProducts>
            <fbc:geneProductAssociation>
                  <fbc:or>
                        <fbc:geneProductRef fbc:geneProduct="SAUSA300_2462"/>
                        <fbc:geneProductRef fbc:geneProduct="SAUSA300_0322"/>
                  </fbc:or>
            </fbc:geneProductAssociation>
      </reaction>

      <reaction metaid="R_FMNAT" id="R_FMNAT" name="FMN adenylyltransferase"
reversible="false" fast="false" fbc:lowerFluxBound="irr_lb"
fbc:upperFluxBound="irr_ub">
            <listOfReactants>
                  <speciesReference species="M_atp_c" stoichiometry="1"
constant="true"/>
                  <speciesReference species="M_fmn_c" stoichiometry="1"
constant="true"/>
            </listOfReactants>
            <listOfProducts>
                  <speciesReference species="M_ppi_c" stoichiometry="1"
constant="true"/>
                  <speciesReference species="M_fad_c" stoichiometry="1"
constant="true"/>
            </listOfProducts>
            <fbc:geneProductAssociation>
                  <fbc:geneProductRef fbc:geneProduct="SAUSA300_1165"/>
            </fbc:geneProductAssociation>
      </reaction>

      <reaction metaid="R_FMNRx" id="R_FMNRx" name="FMN reductase"
reversible="false" fast="false" fbc:lowerFluxBound="irr_lb"
fbc:upperFluxBound="irr_ub">
            <listOfReactants>
                  <speciesReference species="M_h_c" stoichiometry="1"
constant="true"/>
```

```
                  <speciesReference species="M_nadh_c" stoichiometry="1"
constant="true"/>
                  <speciesReference species="M_fmn_c" stoichiometry="1"
constant="true"/>
            </listOfReactants>
            <listOfProducts>
                  <speciesReference species="M_nad_c" stoichiometry="1"
constant="true"/>
                  <speciesReference species="M_fmnh2_c" stoichiometry="1"
constant="true"/>
            </listOfProducts>
            <fbc:geneProductAssociation>
                  <fbc:geneProductRef fbc:geneProduct="SAUSA300_0340"/>
            </fbc:geneProductAssociation>
      </reaction>

      <reaction metaid="R_FOLR2" id="R_FOLR2" name="folate reductase"
reversible="true" fast="false" fbc:lowerFluxBound="rev_lb"
fbc:upperFluxBound="rev_ub">
            <listOfReactants>
                  <speciesReference species="M_nadp_c" stoichiometry="1"
constant="true"/>
                  <speciesReference species="M_dhf_c" stoichiometry="1"
constant="true"/>
            </listOfReactants>
            <listOfProducts>
                  <speciesReference species="M_h_c" stoichiometry="1"
constant="true"/>
                  <speciesReference species="M_nadph_c" stoichiometry="1"
constant="true"/>
                  <speciesReference species="M_fol_c" stoichiometry="1"
constant="true"/>
            </listOfProducts>
            <fbc:geneProductAssociation>
                  <fbc:geneProductRef fbc:geneProduct="SAUSA300_1319"/>
            </fbc:geneProductAssociation>
      </reaction>

      <reaction metaid="R_FORMANT" id="R_FORMANT" name="Formamidopyrimidine
nucleoside triphosphate 7 8-8 9__Dihydrolase " reversible="true" fast="false"
fbc:lowerFluxBound="rev_lb" fbc:upperFluxBound="rev_ub">
            <listOfReactants>
                  <speciesReference species="M_h2o_c" stoichiometry="1"
constant="true"/>
                  <speciesReference species="M_fapnt_c" stoichiometry="1"
constant="true"/>
            </listOfReactants>
            <listOfProducts>
                  <speciesReference species="M_h_c" stoichiometry="1"
constant="true"/>
                  <speciesReference species="M_for_c" stoichiometry="1"
constant="true"/>
                  <speciesReference species="M_ptp_c" stoichiometry="1"
constant="true"/>
            </listOfProducts>
            <fbc:geneProductAssociation>
                  <fbc:geneProductRef fbc:geneProduct="SAUSA300_0551"/>
            </fbc:geneProductAssociation>
```

```
      &lt;/reaction&gt;

      <reaction metaid="R_FORt2" id="R_FORt2" name="formate transport in via
proton symport" reversible="true" fast="false" fbc:lowerFluxBound="rev_lb"
fbc:upperFluxBound="rev_ub">
            &lt;listOfReactants&gt;
                  <speciesReference species="M_h_e" stoichiometry="1"
constant="true"/>
                  <speciesReference species="M_for_e" stoichiometry="1"
constant="true"/>
            &lt;/listOfReactants&gt;
            &lt;listOfProducts&gt;
                  <speciesReference species="M_h_c" stoichiometry="1"
constant="true"/>
                  <speciesReference species="M_for_c" stoichiometry="1"
constant="true"/>
            &lt;/listOfProducts&gt;
            &lt;fbc:geneProductAssociation&gt;
                  &lt;fbc:or&gt;
                        &lt;fbc:geneProductRef fbc:geneProduct="SAUSA300_2349"/&gt;
                        &lt;fbc:geneProductRef fbc:geneProduct="SAUSA300_0305"/&gt;
                  &lt;/fbc:or&gt;
            &lt;/fbc:geneProductAssociation&gt;
      &lt;/reaction&gt;

      <reaction metaid="R_FRD2" id="R_FRD2" name="fumarate reductase"
reversible="false" fast="false" fbc:lowerFluxBound="irr_lb"
fbc:upperFluxBound="irr_ub">
            &lt;listOfReactants&gt;
                  <speciesReference species="M_fum_c" stoichiometry="1"
constant="true"/>
                  <speciesReference species="M_mql8_c" stoichiometry="1"
constant="true"/>
            &lt;/listOfReactants&gt;
            &lt;listOfProducts&gt;
                  <speciesReference species="M_mqn8_c" stoichiometry="1"
constant="true"/>
                  <speciesReference species="M_succ_c" stoichiometry="1"
constant="true"/>
            &lt;/listOfProducts&gt;
            &lt;fbc:geneProductAssociation&gt;
                  &lt;fbc:or&gt;
                        &lt;fbc:and&gt;
                              <fbc:geneProductRef
fbc:geneProduct="SAUSA300_1048"/>
                              <fbc:geneProductRef
fbc:geneProduct="SAUSA300_1047"/>
                        &lt;/fbc:and&gt;
                        &lt;fbc:geneProductRef fbc:geneProduct="SAUSA300_1047"/&gt;
                        &lt;fbc:geneProductRef fbc:geneProduct="SAUSA300_1046"/&gt;
                  &lt;/fbc:or&gt;
            &lt;/fbc:geneProductAssociation&gt;
      &lt;/reaction&gt;

      <reaction metaid="R_FRTT" id="R_FRTT" name="farnesyltranstransferase"
reversible="false" fast="false" fbc:lowerFluxBound="irr_lb"
fbc:upperFluxBound="irr_ub">
            &lt;listOfReactants&gt;
```

```
                  <speciesReference species="M_ipdp_c" stoichiometry="1"
constant="true"/>
                  <speciesReference species="M_frdp_c" stoichiometry="1"
constant="true"/>
            </listOfReactants>
            <listOfProducts>
                  <speciesReference species="M_ppi_c" stoichiometry="1"
constant="true"/>
                  <speciesReference species="M_ggdp_c" stoichiometry="1"
constant="true"/>
            </listOfProducts>
            <fbc:geneProductAssociation>
                  <fbc:or>
                        <fbc:geneProductRef fbc:geneProduct="SAUSA300_1359"/>
                        <fbc:geneProductRef fbc:geneProduct="SAUSA300_1361"/>
                  </fbc:or>
            </fbc:geneProductAssociation>
      </reaction>

      <reaction metaid="R_FRUK" id="R_FRUK" name="fructose-1-phosphate
kinase" reversible="false" fast="false" fbc:lowerFluxBound="irr_lb"
fbc:upperFluxBound="irr_ub">
            <listOfReactants>
                  <speciesReference species="M_atp_c" stoichiometry="1"
constant="true"/>
                  <speciesReference species="M_f1p_c" stoichiometry="1"
constant="true"/>
            </listOfReactants>
            <listOfProducts>
                  <speciesReference species="M_h_c" stoichiometry="1"
constant="true"/>
                  <speciesReference species="M_adp_c" stoichiometry="1"
constant="true"/>
                  <speciesReference species="M_fdp_c" stoichiometry="1"
constant="true"/>
            </listOfProducts>
            <fbc:geneProductAssociation>
                  <fbc:geneProductRef fbc:geneProduct="SAUSA300_0684"/>
            </fbc:geneProductAssociation>
      </reaction>

      <reaction metaid="R_FRUKr" id="R_FRUKr" name="ATP D-fructose-1-
phosphate 6-phosphotransferase " reversible="false" fast="false"
fbc:lowerFluxBound="irr_lb" fbc:upperFluxBound="irr_ub">
            <listOfReactants>
                  <speciesReference species="M_atp_c" stoichiometry="1"
constant="true"/>
                  <speciesReference species="M_f1p_c" stoichiometry="1"
constant="true"/>
            </listOfReactants>
            <listOfProducts>
                  <speciesReference species="M_adp_c" stoichiometry="1"
constant="true"/>
                  <speciesReference species="M_fdp_c" stoichiometry="1"
constant="true"/>
            </listOfProducts>
            <fbc:geneProductAssociation>
                  <fbc:and>
```

```
                        <fbc:geneProductRef fbc:geneProduct="SAUSA300_2153"/>
                        <fbc:geneProductRef fbc:geneProduct="SAUSA300_0684"/>
                  </fbc:and>
            </fbc:geneProductAssociation>
      </reaction>

      <reaction metaid="R_FRUpts" id="R_FRUpts" name="D-fructose transport
via PEP:Pyr PTS" reversible="true" fast="false" fbc:lowerFluxBound="rev_lb"
fbc:upperFluxBound="rev_ub">
            <listOfReactants>
                  <speciesReference species="M_pep_c" stoichiometry="1"
constant="true"/>
                  <speciesReference species="M_fru_e" stoichiometry="1"
constant="true"/>
            </listOfReactants>
            <listOfProducts>
                  <speciesReference species="M_pyr_c" stoichiometry="1"
constant="true"/>
                  <speciesReference species="M_f1p_c" stoichiometry="1"
constant="true"/>
            </listOfProducts>
            <fbc:geneProductAssociation>
                  <fbc:and>
                        <fbc:geneProductRef fbc:geneProduct="SAUSA300_0983"/>
                        <fbc:geneProductRef fbc:geneProduct="SAUSA300_0984"/>
                        <fbc:geneProductRef fbc:geneProduct="SAUSA300_0685"/>
                  </fbc:and>
            </fbc:geneProductAssociation>
      </reaction>

      <reaction metaid="R_FTHFCL" id="R_FTHFCL" name="5-
formethyltetrahydrofolate cyclo__Ligase" reversible="false" fast="false"
fbc:lowerFluxBound="irr_lb" fbc:upperFluxBound="irr_ub">
            <listOfReactants>
                  <speciesReference species="M_atp_c" stoichiometry="1"
constant="true"/>
                  <speciesReference species="M_5fthf_c" stoichiometry="1"
constant="true"/>
            </listOfReactants>
            <listOfProducts>
                  <speciesReference species="M_pi_c" stoichiometry="1"
constant="true"/>
                  <speciesReference species="M_adp_c" stoichiometry="1"
constant="true"/>
                  <speciesReference species="M_methf_c" stoichiometry="1"
constant="true"/>
            </listOfProducts>
            <fbc:geneProductAssociation>
                  <fbc:geneProductRef fbc:geneProduct="SAUSA300_1510"/>
            </fbc:geneProductAssociation>
      </reaction>

      <reaction metaid="R_FTHFL" id="R_FTHFL" name="formate-tetrahydrofolate
ligase" reversible="false" fast="false" fbc:lowerFluxBound="irr_lb"
fbc:upperFluxBound="irr_ub">
            <listOfReactants>
                  <speciesReference species="M_atp_c" stoichiometry="1"
constant="true"/>
```

```
                  <speciesReference species="M_thf_c" stoichiometry="1"
constant="true"/>
                  <speciesReference species="M_for_c" stoichiometry="1"
constant="true"/>
            </listOfReactants>
            <listOfProducts>
                  <speciesReference species="M_pi_c" stoichiometry="1"
constant="true"/>
                  <speciesReference species="M_adp_c" stoichiometry="1"
constant="true"/>
                  <speciesReference species="M_10fthf_c" stoichiometry="1"
constant="true"/>
            </listOfProducts>
            <fbc:geneProductAssociation>
                  <fbc:geneProductRef fbc:geneProduct="SAUSA300_1678"/>
            </fbc:geneProductAssociation>
      </reaction>

      <reaction metaid="R_FUM" id="R_FUM" name="fumarase" reversible="true"
fast="false" fbc:lowerFluxBound="rev_lb" fbc:upperFluxBound="rev_ub">
            <listOfReactants>
                  <speciesReference species="M_mal__L_c" stoichiometry="1"
constant="true"/>
            </listOfReactants>
            <listOfProducts>
                  <speciesReference species="M_h2o_c" stoichiometry="1"
constant="true"/>
                  <speciesReference species="M_fum_c" stoichiometry="1"
constant="true"/>
            </listOfProducts>
            <fbc:geneProductAssociation>
                  <fbc:geneProductRef fbc:geneProduct="SAUSA300_1801"/>
            </fbc:geneProductAssociation>
      </reaction>

      <reaction metaid="R_FUMt2_3" id="R_FUMt2_3" name="Fumarate transport
via proton symport" reversible="true" fast="false"
fbc:lowerFluxBound="rev_lb" fbc:upperFluxBound="rev_ub">
            <listOfReactants>
                  <speciesReference species="M_fum_e" stoichiometry="1"
constant="true"/>
                  <speciesReference species="M_h_e" stoichiometry="3.0"
constant="true"/>
            </listOfReactants>
            <listOfProducts>
                  <speciesReference species="M_fum_c" stoichiometry="1"
constant="true"/>
                  <speciesReference species="M_h_c" stoichiometry="3.0"
constant="true"/>
            </listOfProducts>
      </reaction>

      <reaction metaid="R_G1PACT" id="R_G1PACT" name="glucosamine-1-phosphate
N-acetyltransferase" reversible="false" fast="false"
fbc:lowerFluxBound="irr_lb" fbc:upperFluxBound="irr_ub">
            <listOfReactants>
                  <speciesReference species="M_accoa_c" stoichiometry="1"
constant="true"/>
```

```
                        <speciesReference species="M_gam1p_c" stoichiometry="1"
constant="true"/>
                  </listOfReactants>
                  <listOfProducts>
                        <speciesReference species="M_h_c" stoichiometry="1"
constant="true"/>
                        <speciesReference species="M_coa_c" stoichiometry="1"
constant="true"/>
                        <speciesReference species="M_acgam1p_c" stoichiometry="1"
constant="true"/>
                  </listOfProducts>
                  <fbc:geneProductAssociation>
                        <fbc:geneProductRef fbc:geneProduct="SAUSA300_0477"/>
                  </fbc:geneProductAssociation>
            </reaction>

            <reaction metaid="R_G1SAT" id="R_G1SAT" name="glutamate-1-semialdehyde
aminotransferase" reversible="true" fast="false" fbc:lowerFluxBound="rev_lb"
fbc:upperFluxBound="rev_ub">
                  <listOfReactants>
                        <speciesReference species="M_5aop_c" stoichiometry="1"
constant="true"/>
                  </listOfReactants>
                  <listOfProducts>
                        <speciesReference species="M_glu1sa_c" stoichiometry="1"
constant="true"/>
                  </listOfProducts>
                  <fbc:geneProductAssociation>
                        <fbc:or>
                              <fbc:geneProductRef fbc:geneProduct="SAUSA300_1614"/>
                              <fbc:geneProductRef fbc:geneProduct="SAUSA300_1845"/>
                        </fbc:or>
                  </fbc:geneProductAssociation>
            </reaction>

            <reaction metaid="R_G3PAT120" id="R_G3PAT120" name="glycerol-3-
phosphate acyltransferase (C12:0)" reversible="false" fast="false"
fbc:lowerFluxBound="irr_lb" fbc:upperFluxBound="irr_ub">
                  <listOfReactants>
                        <speciesReference species="M_glyc3p_c" stoichiometry="1"
constant="true"/>
                        <speciesReference species="M_ddcaACP_c" stoichiometry="1"
constant="true"/>
                  </listOfReactants>
                  <listOfProducts>
                        <speciesReference species="M_ACP_c" stoichiometry="1"
constant="true"/>
                        <speciesReference species="M_1ddecg3p_c" stoichiometry="1"
constant="true"/>
                  </listOfProducts>
                  <fbc:geneProductAssociation>
                        <fbc:and>
                              <fbc:geneProductRef fbc:geneProduct="SAUSA300_1249"/>
                              <fbc:or>
                                    <fbc:geneProductRef
fbc:geneProduct="SAUSA300_1122"/>
                                    <fbc:geneProductRef
fbc:geneProduct="SAUSA300_1121"/>
```

```
                        </fbc:or>
                  </fbc:and>
            </fbc:geneProductAssociation>
      </reaction>

      <reaction metaid="R_G3PAT140" id="R_G3PAT140" name="glycerol-3-
phosphate acyltransferase (C14:0)" reversible="false" fast="false"
fbc:lowerFluxBound="irr_lb" fbc:upperFluxBound="irr_ub">
            <listOfReactants>
                  <speciesReference species="M_glyc3p_c" stoichiometry="1"
constant="true"/>
                  <speciesReference species="M_myrsACP_c" stoichiometry="1"
constant="true"/>
            </listOfReactants>
            <listOfProducts>
                  <speciesReference species="M_ACP_c" stoichiometry="1"
constant="true"/>
                  <speciesReference species="M_1tdecg3p_c" stoichiometry="1"
constant="true"/>
            </listOfProducts>
            <fbc:geneProductAssociation>
                  <fbc:and>
                        <fbc:geneProductRef fbc:geneProduct="SAUSA300_1249"/>
                        <fbc:or>
                              <fbc:geneProductRef
fbc:geneProduct="SAUSA300_1122"/>
                              <fbc:geneProductRef
fbc:geneProduct="SAUSA300_1121"/>
                        </fbc:or>
                  </fbc:and>
            </fbc:geneProductAssociation>
      </reaction>

      <reaction metaid="R_G3PAT141" id="R_G3PAT141" name="glycerol-3-
phosphate acyltransferase (C14:1)" reversible="false" fast="false"
fbc:lowerFluxBound="irr_lb" fbc:upperFluxBound="irr_ub">
            <listOfReactants>
                  <speciesReference species="M_glyc3p_c" stoichiometry="1"
constant="true"/>
                  <speciesReference species="M_tdeACP_c" stoichiometry="1"
constant="true"/>
            </listOfReactants>
            <listOfProducts>
                  <speciesReference species="M_ACP_c" stoichiometry="1"
constant="true"/>
                  <speciesReference species="M_1tdec7eg3p_c"
stoichiometry="1" constant="true"/>
            </listOfProducts>
            <fbc:geneProductAssociation>
                  <fbc:and>
                        <fbc:geneProductRef fbc:geneProduct="SAUSA300_1249"/>
                        <fbc:or>
                              <fbc:geneProductRef
fbc:geneProduct="SAUSA300_1122"/>
                              <fbc:geneProductRef
fbc:geneProduct="SAUSA300_1121"/>
                        </fbc:or>
                  </fbc:and>
```

```
            </fbc:geneProductAssociation>
      </reaction>

      <reaction metaid="R_G3PAT160" id="R_G3PAT160" name="glycerol-3-
phosphate acyltransferase (C16:0)" reversible="false" fast="false"
fbc:lowerFluxBound="irr_lb" fbc:upperFluxBound="irr_ub">
            <listOfReactants>
                  <speciesReference species="M_glyc3p_c" stoichiometry="1"
constant="true"/>
                  <speciesReference species="M_palmACP_c" stoichiometry="1"
constant="true"/>
            </listOfReactants>
            <listOfProducts>
                  <speciesReference species="M_ACP_c" stoichiometry="1"
constant="true"/>
                  <speciesReference species="M_1hdecg3p_c" stoichiometry="1"
constant="true"/>
            </listOfProducts>
            <fbc:geneProductAssociation>
                  <fbc:and>
                        <fbc:geneProductRef fbc:geneProduct="SAUSA300_1249"/>
                        <fbc:or>
                              <fbc:geneProductRef
fbc:geneProduct="SAUSA300_1122"/>
                              <fbc:geneProductRef
fbc:geneProduct="SAUSA300_1121"/>
                        </fbc:or>
                  </fbc:and>
            </fbc:geneProductAssociation>
      </reaction>

      <reaction metaid="R_G3PAT161" id="R_G3PAT161" name="glycerol-3-
phosphate acyltransferase (C16:1)" reversible="false" fast="false"
fbc:lowerFluxBound="irr_lb" fbc:upperFluxBound="irr_ub">
            <listOfReactants>
                  <speciesReference species="M_glyc3p_c" stoichiometry="1"
constant="true"/>
                  <speciesReference species="M_hdeACP_c" stoichiometry="1"
constant="true"/>
            </listOfReactants>
            <listOfProducts>
                  <speciesReference species="M_ACP_c" stoichiometry="1"
constant="true"/>
                  <speciesReference species="M_1hdec9eg3p_c"
stoichiometry="1" constant="true"/>
            </listOfProducts>
            <fbc:geneProductAssociation>
                  <fbc:and>
                        <fbc:geneProductRef fbc:geneProduct="SAUSA300_1249"/>
                        <fbc:or>
                              <fbc:geneProductRef
fbc:geneProduct="SAUSA300_1122"/>
                              <fbc:geneProductRef
fbc:geneProduct="SAUSA300_1121"/>
                        </fbc:or>
                  </fbc:and>
            </fbc:geneProductAssociation>
      </reaction>
```

```
      <reaction metaid="R_G3PAT180" id="R_G3PAT180" name="glycerol-3-
phosphate acyltransferase (C18:0)" reversible="false" fast="false"
fbc:lowerFluxBound="irr_lb" fbc:upperFluxBound="irr_ub">
            <listOfReactants>
                  <speciesReference species="M_glyc3p_c" stoichiometry="1"
constant="true"/>
                  <speciesReference species="M_ocdcaACP_c" stoichiometry="1"
constant="true"/>
            </listOfReactants>
            <listOfProducts>
                  <speciesReference species="M_ACP_c" stoichiometry="1"
constant="true"/>
                  <speciesReference species="M_1odecg3p_c" stoichiometry="1"
constant="true"/>
            </listOfProducts>
            <fbc:geneProductAssociation>
                  <fbc:and>
                        <fbc:geneProductRef fbc:geneProduct="SAUSA300_1249"/>
                        <fbc:or>
                              <fbc:geneProductRef
fbc:geneProduct="SAUSA300_1122"/>
                              <fbc:geneProductRef
fbc:geneProduct="SAUSA300_1121"/>
                        </fbc:or>
                  </fbc:and>
            </fbc:geneProductAssociation>
      </reaction>

      <reaction metaid="R_G3PAT181" id="R_G3PAT181" name="glycerol-3-
phosphate acyltransferase (C18:1)" reversible="false" fast="false"
fbc:lowerFluxBound="irr_lb" fbc:upperFluxBound="irr_ub">
            <listOfReactants>
                  <speciesReference species="M_glyc3p_c" stoichiometry="1"
constant="true"/>
                  <speciesReference species="M_octeACP_c" stoichiometry="1"
constant="true"/>
            </listOfReactants>
            <listOfProducts>
                  <speciesReference species="M_ACP_c" stoichiometry="1"
constant="true"/>
                  <speciesReference species="M_1odec11eg3p_c"
stoichiometry="1" constant="true"/>
            </listOfProducts>
            <fbc:geneProductAssociation>
                  <fbc:and>
                        <fbc:geneProductRef fbc:geneProduct="SAUSA300_1249"/>
                        <fbc:or>
                              <fbc:geneProductRef
fbc:geneProduct="SAUSA300_1122"/>
                              <fbc:geneProductRef
fbc:geneProduct="SAUSA300_1121"/>
                        </fbc:or>
                  </fbc:and>
            </fbc:geneProductAssociation>
      </reaction>
```

```
      <reaction metaid="R_G3PCT" id="R_G3PCT" name="glycerol-3-phosphate
cytidylyltransferase" reversible="false" fast="false"
fbc:lowerFluxBound="irr_lb" fbc:upperFluxBound="irr_ub">
            <listOfReactants>
                  <speciesReference species="M_ctp_c" stoichiometry="1"
constant="true"/>
                  <speciesReference species="M_glyc3p_c" stoichiometry="1"
constant="true"/>
            </listOfReactants>
            <listOfProducts>
                  <speciesReference species="M_ppi_c" stoichiometry="1"
constant="true"/>
                  <speciesReference species="M_cdpglyc_c" stoichiometry="1"
constant="true"/>
            </listOfProducts>
            <fbc:geneProductAssociation>
                  <fbc:geneProductRef fbc:geneProduct="SAUSA300_0628"/>
            </fbc:geneProductAssociation>
      </reaction>

      <reaction metaid="R_G3PD" id="R_G3PD" name="glycerol-3-phosphate
dehydrogenase (FAD)" reversible="false" fast="false"
fbc:lowerFluxBound="irr_lb" fbc:upperFluxBound="irr_ub">
            <listOfReactants>
                  <speciesReference species="M_glyc3p_c" stoichiometry="1"
constant="true"/>
                  <speciesReference species="M_fad_c" stoichiometry="1"
constant="true"/>
            </listOfReactants>
            <listOfProducts>
                  <speciesReference species="M_dhap_c" stoichiometry="1"
constant="true"/>
                  <speciesReference species="M_fadh2_c" stoichiometry="1"
constant="true"/>
            </listOfProducts>
            <fbc:geneProductAssociation>
                  <fbc:geneProductRef fbc:geneProduct="SAUSA300_1193"/>
            </fbc:geneProductAssociation>
      </reaction>

      <reaction metaid="R_G3PD2" id="R_G3PD2" name="glycerol-3-phosphate
dehydrogenase (NADP)" reversible="true" fast="false"
fbc:lowerFluxBound="rev_lb" fbc:upperFluxBound="rev_ub">
            <listOfReactants>
                  <speciesReference species="M_nadp_c" stoichiometry="1"
constant="true"/>
                  <speciesReference species="M_glyc3p_c" stoichiometry="1"
constant="true"/>
            </listOfReactants>
            <listOfProducts>
                  <speciesReference species="M_h_c" stoichiometry="1"
constant="true"/>
                  <speciesReference species="M_nadph_c" stoichiometry="1"
constant="true"/>
                  <speciesReference species="M_dhap_c" stoichiometry="1"
constant="true"/>
            </listOfProducts>
            <fbc:geneProductAssociation>
```

```
                <fbc:geneProductRef fbc:geneProduct="SAUSA300_1363"/>
            </fbc:geneProductAssociation>
        </reaction>

        <reaction metaid="R_G3PD6" id="R_G3PD6" name="glycerol-3-phosphate
dehydrogenase (menaquinone-8)" reversible="false" fast="false"
fbc:lowerFluxBound="irr_lb" fbc:upperFluxBound="irr_ub">
            <listOfReactants>
                <speciesReference species="M_glyc3p_c" stoichiometry="1"
constant="true"/>
                <speciesReference species="M_mqn8_c" stoichiometry="1"
constant="true"/>
            </listOfReactants>
            <listOfProducts>
                <speciesReference species="M_dhap_c" stoichiometry="1"
constant="true"/>
                <speciesReference species="M_mql8_c" stoichiometry="1"
constant="true"/>
            </listOfProducts>
            <fbc:geneProductAssociation>
                <fbc:geneProductRef fbc:geneProduct="SAUSA300_1193"/>
            </fbc:geneProductAssociation>
        </reaction>

        <reaction metaid="R_G5SAD" id="R_G5SAD" name="L-glutamate 5-
semialdehyde dehydratase spontaneous " reversible="false" fast="false"
fbc:lowerFluxBound="irr_lb" fbc:upperFluxBound="irr_ub">
            <listOfReactants>
                <speciesReference species="M_glu5sa_c" stoichiometry="1"
constant="true"/>
            </listOfReactants>
            <listOfProducts>
                <speciesReference species="M_h_c" stoichiometry="1"
constant="true"/>
                <speciesReference species="M_h2o_c" stoichiometry="1"
constant="true"/>
                <speciesReference species="M_1pyr5c_c" stoichiometry="1"
constant="true"/>
            </listOfProducts>
        </reaction>

        <reaction metaid="R_G6PDH2r" id="R_G6PDH2r" name="glucose 6-phosphate
dehydrogenase" reversible="true" fast="false" fbc:lowerFluxBound="rev_lb"
fbc:upperFluxBound="rev_ub">
            <listOfReactants>
                <speciesReference species="M_g6p_c" stoichiometry="1"
constant="true"/>
                <speciesReference species="M_nadp_c" stoichiometry="1"
constant="true"/>
            </listOfReactants>
            <listOfProducts>
                <speciesReference species="M_h_c" stoichiometry="1"
constant="true"/>
                <speciesReference species="M_nadph_c" stoichiometry="1"
constant="true"/>
                <speciesReference species="M_6pgl_c" stoichiometry="1"
constant="true"/>
            </listOfProducts>
```

```
            <fbc:geneProductAssociation>
                  <fbc:geneProductRef fbc:geneProduct="SAUSA300_1454"/>
            </fbc:geneProductAssociation>
      </reaction>

      <reaction metaid="R_G6PI3" id="R_G6PI3" name="Glucose-6-phosphate
isomerase" reversible="false" fast="false" fbc:lowerFluxBound="irr_lb"
fbc:upperFluxBound="irr_ub">
            <listOfReactants>
                  <speciesReference species="M_g6p__B_c" stoichiometry="1"
constant="true"/>
            </listOfReactants>
            <listOfProducts>
                  <speciesReference species="M_f6p_c" stoichiometry="1"
constant="true"/>
            </listOfProducts>
            <fbc:geneProductAssociation>
                  <fbc:geneProductRef fbc:geneProduct="SAUSA300_0865"/>
            </fbc:geneProductAssociation>
      </reaction>

      <reaction metaid="R_G6PP" id="R_G6PP" name="glucose-6-phosphate
phosphatase" reversible="false" fast="false" fbc:lowerFluxBound="irr_lb"
fbc:upperFluxBound="irr_ub">
            <listOfReactants>
                  <speciesReference species="M_h2o_c" stoichiometry="1"
constant="true"/>
                  <speciesReference species="M_g6p_c" stoichiometry="1"
constant="true"/>
            </listOfReactants>
            <listOfProducts>
                  <speciesReference species="M_glc__D_c" stoichiometry="1"
constant="true"/>
                  <speciesReference species="M_pi_c" stoichiometry="1"
constant="true"/>
            </listOfProducts>
            <fbc:geneProductAssociation>
                  <fbc:geneProductRef fbc:geneProduct="SAUSA300_2561"/>
            </fbc:geneProductAssociation>
      </reaction>

      <reaction metaid="R_G6Pt6_2pp" id="R_G6Pt6_2pp" name="Glucose-6-
phosphate transport via phosphate antiport (periplasm)" reversible="false"
fast="false" fbc:lowerFluxBound="irr_lb" fbc:upperFluxBound="irr_ub">
            <listOfReactants>
                  <speciesReference species="M_pi_c" stoichiometry="2"
constant="true"/>
                  <speciesReference species="M_g6p_p" stoichiometry="1"
constant="true"/>
            </listOfReactants>
            <listOfProducts>
                  <speciesReference species="M_g6p_c" stoichiometry="1"
constant="true"/>
                  <speciesReference species="M_pi_p" stoichiometry="2"
constant="true"/>
            </listOfProducts>
            <fbc:geneProductAssociation>
                  <fbc:geneProductRef fbc:geneProduct="SAUSA300_0216"/>
```

```
            </fbc:geneProductAssociation>
      </reaction>

      <reaction metaid="R_G6Ptex" id="R_G6Ptex" name="glucose 6-phosphate
transport via diffusion (extracellular to periplasm)" reversible="true"
fast="false" fbc:lowerFluxBound="rev_lb" fbc:upperFluxBound="rev_ub">
            <listOfReactants>
                  <speciesReference species="M_g6p_e" stoichiometry="1"
constant="true"/>
            </listOfReactants>
            <listOfProducts>
                  <speciesReference species="M_g6p_p" stoichiometry="1"
constant="true"/>
            </listOfProducts>
      </reaction>

      <reaction metaid="R_GAL6PI" id="R_GAL6PI" name="galactose-6-phosphate
isomerase" reversible="true" fast="false" fbc:lowerFluxBound="rev_lb"
fbc:upperFluxBound="rev_ub">
            <listOfReactants>
                  <speciesReference species="M_dgal6p_c" stoichiometry="1"
constant="true"/>
            </listOfReactants>
            <listOfProducts>
                  <speciesReference species="M_tag6p__D_c" stoichiometry="1"
constant="true"/>
            </listOfProducts>
            <fbc:geneProductAssociation>
                  <fbc:or>
                        <fbc:geneProductRef fbc:geneProduct="SAUSA300_2154"/>
                        <fbc:geneProductRef fbc:geneProduct="SAUSA300_2155"/>
                  </fbc:or>
            </fbc:geneProductAssociation>
      </reaction>

      <reaction metaid="R_GALabcpp" id="R_GALabcpp" name="D-galactose
transport via ABC system (periplasm)" reversible="false" fast="false"
fbc:lowerFluxBound="irr_lb" fbc:upperFluxBound="irr_ub">
            <listOfReactants>
                  <speciesReference species="M_h2o_c" stoichiometry="1"
constant="true"/>
                  <speciesReference species="M_atp_c" stoichiometry="1"
constant="true"/>
                  <speciesReference species="M_gal_p" stoichiometry="1"
constant="true"/>
            </listOfReactants>
            <listOfProducts>
                  <speciesReference species="M_h_c" stoichiometry="1"
constant="true"/>
                  <speciesReference species="M_pi_c" stoichiometry="1"
constant="true"/>
                  <speciesReference species="M_adp_c" stoichiometry="1"
constant="true"/>
                  <speciesReference species="M_gal_c" stoichiometry="1"
constant="true"/>
            </listOfProducts>
            <fbc:geneProductAssociation>
                  <fbc:and>
```

```
                  <fbc:geneProductRef fbc:geneProduct="SAUSA300_0208"/>
                  <fbc:geneProductRef fbc:geneProduct="SAUSA300_0209"/>
                  <fbc:geneProductRef fbc:geneProduct="SAUSA300_0210"/>
                  <fbc:geneProductRef fbc:geneProduct="SAUSA300_0211"/>
               </fbc:and>
            </fbc:geneProductAssociation>
      </reaction>

      <reaction metaid="R_GALKr" id="R_GALKr" name="galactokinase"
reversible="true" fast="false" fbc:lowerFluxBound="rev_lb"
fbc:upperFluxBound="rev_ub">
            <listOfReactants>
               <speciesReference species="M_atp_c" stoichiometry="1"
constant="true"/>
               <speciesReference species="M_gal_c" stoichiometry="1"
constant="true"/>
            </listOfReactants>
            <listOfProducts>
               <speciesReference species="M_h_c" stoichiometry="1"
constant="true"/>
               <speciesReference species="M_adp_c" stoichiometry="1"
constant="true"/>
               <speciesReference species="M_gal1p_c" stoichiometry="1"
constant="true"/>
            </listOfProducts>
            <fbc:geneProductAssociation>
               <fbc:geneProductRef fbc:geneProduct="SAUSA300_0572"/>
            </fbc:geneProductAssociation>
      </reaction>

      <reaction metaid="R_GALpts" id="R_GALpts" name="Galactose transport via
PTS" reversible="true" fast="false" fbc:lowerFluxBound="rev_lb"
fbc:upperFluxBound="rev_ub">
            <listOfReactants>
               <speciesReference species="M_gal_e" stoichiometry="1"
constant="true"/>
               <speciesReference species="M_pep_c" stoichiometry="1"
constant="true"/>
            </listOfReactants>
            <listOfProducts>
               <speciesReference species="M_dgal6p_c" stoichiometry="1"
constant="true"/>
               <speciesReference species="M_pyr_c" stoichiometry="1"
constant="true"/>
            </listOfProducts>
            <fbc:geneProductAssociation>
               <fbc:and>
                  <fbc:geneProductRef fbc:geneProduct="SAUSA300_2150"/>
                  <fbc:geneProductRef fbc:geneProduct="SAUSA300_2151"/>
                  <fbc:geneProductRef fbc:geneProduct="SAUSA300_0984"/>
                  <fbc:geneProductRef fbc:geneProduct="SAUSA300_0983"/>
               </fbc:and>
            </fbc:geneProductAssociation>
      </reaction>

      <reaction metaid="R_GALS3" id="R_GALS3" name="A-galactosidase
(melibiose)" reversible="false" fast="false" fbc:lowerFluxBound="irr_lb"
fbc:upperFluxBound="irr_ub">
```

```
            <listOfReactants>
                  <speciesReference species="M_h2o_c" stoichiometry="1"
constant="true"/>
                  <speciesReference species="M_melib_c" stoichiometry="1"
constant="true"/>
            </listOfReactants>
            <listOfProducts>
                  <speciesReference species="M_gal_c" stoichiometry="1"
constant="true"/>
                  <speciesReference species="M_glc__D_c" stoichiometry="1"
constant="true"/>
            </listOfProducts>
      </reaction>

      <reaction metaid="R_GALtex" id="R_GALtex" name="D-galactose transport
via diffusion (extracellular to periplasm)" reversible="true" fast="false"
fbc:lowerFluxBound="rev_lb" fbc:upperFluxBound="rev_ub">
            <listOfReactants>
                  <speciesReference species="M_gal_e" stoichiometry="1"
constant="true"/>
            </listOfReactants>
            <listOfProducts>
                  <speciesReference species="M_gal_p" stoichiometry="1"
constant="true"/>
            </listOfProducts>
      </reaction>

      <reaction metaid="R_GALTpts" id="R_GALTpts" name="Galactitol transport
via PEP:Pyr PTS" reversible="true" fast="false" fbc:lowerFluxBound="rev_lb"
fbc:upperFluxBound="rev_ub">
            <listOfReactants>
                  <speciesReference species="M_galt_e" stoichiometry="1"
constant="true"/>
                  <speciesReference species="M_pep_c" stoichiometry="1"
constant="true"/>
            </listOfReactants>
            <listOfProducts>
                  <speciesReference species="M_pyr_c" stoichiometry="1"
constant="true"/>
                  <speciesReference species="M_galt1p_c" stoichiometry="1"
constant="true"/>
            </listOfProducts>
            <fbc:geneProductAssociation>
                  <fbc:and>
                        <fbc:geneProductRef fbc:geneProduct="SAUSA300_0240"/>
                        <fbc:geneProductRef fbc:geneProduct="SAUSA300_0241"/>
                        <fbc:geneProductRef fbc:geneProduct="SAUSA300_0984"/>
                        <fbc:geneProductRef fbc:geneProduct="SAUSA300_0239"/>
                  </fbc:and>
            </fbc:geneProductAssociation>
      </reaction>

      <reaction metaid="R_GALUi" id="R_GALUi" name="UTP-glucose-1-phosphate
uridylyltransferase (irreversible)" reversible="false" fast="false"
fbc:lowerFluxBound="irr_lb" fbc:upperFluxBound="irr_ub">
            <listOfReactants>
                  <speciesReference species="M_h_c" stoichiometry="1"
constant="true"/>
```

```
                  <speciesReference species="M_utp_c" stoichiometry="1"
constant="true"/>
                  <speciesReference species="M_g1p_c" stoichiometry="1"
constant="true"/>
            </listOfReactants>
            <listOfProducts>
                  <speciesReference species="M_ppi_c" stoichiometry="1"
constant="true"/>
                  <speciesReference species="M_udpg_c" stoichiometry="1"
constant="true"/>
            </listOfProducts>
            <fbc:geneProductAssociation>
                  <fbc:geneProductRef fbc:geneProduct="SAUSA300_2439"/>
            </fbc:geneProductAssociation>
      </reaction>

      <reaction metaid="R_GAM6Pt6_2pp" id="R_GAM6Pt6_2pp" name="D-Glucosamine
6-phosphate transport via phosphate antiport (periplasm)" reversible="false"
fast="false" fbc:lowerFluxBound="irr_lb" fbc:upperFluxBound="irr_ub">
            <listOfReactants>
                  <speciesReference species="M_pi_c" stoichiometry="2"
constant="true"/>
                  <speciesReference species="M_gam6p_p" stoichiometry="1"
constant="true"/>
            </listOfReactants>
            <listOfProducts>
                  <speciesReference species="M_gam6p_c" stoichiometry="1"
constant="true"/>
                  <speciesReference species="M_pi_p" stoichiometry="2"
constant="true"/>
            </listOfProducts>
      </reaction>

      <reaction metaid="R_GAMAN6Ptex" id="R_GAMAN6Ptex" name="D-glucosamine
6-phosphate transport via diffusion (extracellular to periplasm)"
reversible="true" fast="false" fbc:lowerFluxBound="rev_lb"
fbc:upperFluxBound="rev_ub">
            <listOfReactants>
                  <speciesReference species="M_gam6p_e" stoichiometry="1"
constant="true"/>
            </listOfReactants>
            <listOfProducts>
                  <speciesReference species="M_gam6p_p" stoichiometry="1"
constant="true"/>
            </listOfProducts>
      </reaction>

      <reaction metaid="R_GAMpts" id="R_GAMpts" name="D-glucosamine transport
via PEP:Pyr PTS" reversible="true" fast="false" fbc:lowerFluxBound="rev_lb"
fbc:upperFluxBound="rev_ub">
            <listOfReactants>
                  <speciesReference species="M_pep_c" stoichiometry="1"
constant="true"/>
                  <speciesReference species="M_gam_e" stoichiometry="1"
constant="true"/>
            </listOfReactants>
            <listOfProducts>
```

```
                  <speciesReference species="M_pyr_c" stoichiometry="1"
constant="true"/>
                  <speciesReference species="M_gam6p_c" stoichiometry="1"
constant="true"/>
            &lt;/listOfProducts&gt;
            &lt;fbc:geneProductAssociation&gt;
                  &lt;fbc:and&gt;
                        &lt;fbc:geneProductRef fbc:geneProduct="SAUSA300_0983"/&gt;
                        &lt;fbc:geneProductRef fbc:geneProduct="SAUSA300_0984"/&gt;
                  &lt;/fbc:and&gt;
            &lt;/fbc:geneProductAssociation&gt;
      &lt;/reaction&gt;

      <reaction metaid="R_GAPD" id="R_GAPD" name="glyceraldehyde-3-phosphate
dehydrogenase" reversible="true" fast="false" fbc:lowerFluxBound="rev_lb"
fbc:upperFluxBound="rev_ub">
            &lt;listOfReactants&gt;
                  <speciesReference species="M_pi_c" stoichiometry="1"
constant="true"/>
                  <speciesReference species="M_nad_c" stoichiometry="1"
constant="true"/>
                  <speciesReference species="M_g3p_c" stoichiometry="1"
constant="true"/>
            &lt;/listOfReactants&gt;
            &lt;listOfProducts&gt;
                  <speciesReference species="M_nadh_c" stoichiometry="1"
constant="true"/>
                  <speciesReference species="M_13dpg_c" stoichiometry="1"
constant="true"/>
            &lt;/listOfProducts&gt;
            &lt;fbc:geneProductAssociation&gt;
                  &lt;fbc:or&gt;
                        &lt;fbc:geneProductRef fbc:geneProduct="SAUSA300_0756"/&gt;
                        &lt;fbc:geneProductRef fbc:geneProduct="SAUSA300_1633"/&gt;
                  &lt;/fbc:or&gt;
            &lt;/fbc:geneProductAssociation&gt;
      &lt;/reaction&gt;

      <reaction metaid="R_GARFT" id="R_GARFT" name="phosphoribosylglycinamide
formyltransferase" reversible="true" fast="false" fbc:lowerFluxBound="rev_lb"
fbc:upperFluxBound="rev_ub">
            &lt;listOfReactants&gt;
                  <speciesReference species="M_10fthf_c" stoichiometry="1"
constant="true"/>
                  <speciesReference species="M_gar_c" stoichiometry="1"
constant="true"/>
            &lt;/listOfReactants&gt;
            &lt;listOfProducts&gt;
                  <speciesReference species="M_h_c" stoichiometry="1"
constant="true"/>
                  <speciesReference species="M_fgam_c" stoichiometry="1"
constant="true"/>
                  <speciesReference species="M_thf_c" stoichiometry="1"
constant="true"/>
            &lt;/listOfProducts&gt;
            &lt;fbc:geneProductAssociation&gt;
                  &lt;fbc:geneProductRef fbc:geneProduct="SAUSA300_0974"/&gt;
            &lt;/fbc:geneProductAssociation&gt;
```

```
      </reaction>

      <reaction metaid="R_GCALDD" id="R_GCALDD" name="Glycolaldehyde
dehydrogenase" reversible="false" fast="false" fbc:lowerFluxBound="irr_lb"
fbc:upperFluxBound="irr_ub">
            <listOfReactants>
                  <speciesReference species="M_h2o_c" stoichiometry="1"
constant="true"/>
                  <speciesReference species="M_nad_c" stoichiometry="1"
constant="true"/>
                  <speciesReference species="M_gcald_c" stoichiometry="1"
constant="true"/>
            </listOfReactants>
            <listOfProducts>
                  <speciesReference species="M_h_c" stoichiometry="2"
constant="true"/>
                  <speciesReference species="M_nadh_c" stoichiometry="1"
constant="true"/>
                  <speciesReference species="M_glyclt_c" stoichiometry="1"
constant="true"/>
            </listOfProducts>
            <fbc:geneProductAssociation>
                  <fbc:or>
                        <fbc:geneProductRef fbc:geneProduct="SAUSA300_1901"/>
                        <fbc:geneProductRef fbc:geneProduct="SAUSA300_2076"/>
                  </fbc:or>
            </fbc:geneProductAssociation>
      </reaction>

      <reaction metaid="R_GCCa" id="R_GCCa" name="glycine-cleavage complex"
reversible="false" fast="false" fbc:lowerFluxBound="irr_lb"
fbc:upperFluxBound="irr_ub">
            <listOfReactants>
                  <speciesReference species="M_h_c" stoichiometry="1"
constant="true"/>
                  <speciesReference species="M_gly_c" stoichiometry="1"
constant="true"/>
                  <speciesReference species="M_lpro_c" stoichiometry="1"
constant="true"/>
            </listOfReactants>
            <listOfProducts>
                  <speciesReference species="M_co2_c" stoichiometry="1"
constant="true"/>
                  <speciesReference species="M_alpro_c" stoichiometry="1"
constant="true"/>
            </listOfProducts>
            <fbc:geneProductAssociation>
                  <fbc:and>
                        <fbc:geneProductRef fbc:geneProduct="SAUSA300_1496"/>
                        <fbc:geneProductRef fbc:geneProduct="SAUSA300_1497"/>
                        <fbc:geneProductRef fbc:geneProduct="SAUSA300_0791"/>
                  </fbc:and>
            </fbc:geneProductAssociation>
      </reaction>

      <reaction metaid="R_GCCb" id="R_GCCb" name="glycine cleavage complex"
reversible="false" fast="false" fbc:lowerFluxBound="irr_lb"
fbc:upperFluxBound="irr_ub">
```

```
            <listOfReactants>
                  <speciesReference species="M_thf_c" stoichiometry="1"
constant="true"/>
                  <speciesReference species="M_alpro_c" stoichiometry="1"
constant="true"/>
            </listOfReactants>
            <listOfProducts>
                  <speciesReference species="M_nh4_c" stoichiometry="1"
constant="true"/>
                  <speciesReference species="M_mlthf_c" stoichiometry="1"
constant="true"/>
                  <speciesReference species="M_dhlpro_c" stoichiometry="1"
constant="true"/>
            </listOfProducts>
            <fbc:geneProductAssociation>
                  <fbc:or>
                        <fbc:and>
                              <fbc:geneProductRef
fbc:geneProduct="SAUSA300_0791"/>
                              <fbc:geneProductRef
fbc:geneProduct="SAUSA300_1498"/>
                        </fbc:and>
                        <fbc:geneProductRef fbc:geneProduct="SAUSA300_0325"/>
                        <fbc:and>
                              <fbc:geneProductRef
fbc:geneProduct="SAUSA300_0791"/>
                              <fbc:geneProductRef
fbc:geneProduct="SAUSA300_1496"/>
                              <fbc:geneProductRef
fbc:geneProduct="SAUSA300_1497"/>
                        </fbc:and>
                  </fbc:or>
            </fbc:geneProductAssociation>
      </reaction>

      <reaction metaid="R_GCCc" id="R_GCCc" name="glycine-cleavage complex"
reversible="true" fast="false" fbc:lowerFluxBound="rev_lb"
fbc:upperFluxBound="rev_ub">
            <listOfReactants>
                  <speciesReference species="M_nad_c" stoichiometry="1"
constant="true"/>
                  <speciesReference species="M_dhlpro_c" stoichiometry="1"
constant="true"/>
            </listOfReactants>
            <listOfProducts>
                  <speciesReference species="M_h_c" stoichiometry="1"
constant="true"/>
                  <speciesReference species="M_nadh_c" stoichiometry="1"
constant="true"/>
                  <speciesReference species="M_lpro_c" stoichiometry="1"
constant="true"/>
            </listOfProducts>
            <fbc:geneProductAssociation>
                  <fbc:or>
                        <fbc:and>
                              <fbc:geneProductRef
fbc:geneProduct="SAUSA300_0996"/>
```

```
                              <fbc:geneProductRef
fbc:geneProduct="SAUSA300_0791"/>
                        </fbc:and>
                        <fbc:geneProductRef fbc:geneProduct="SAUSA300_0325"/>
                        <fbc:and>
                              <fbc:geneProductRef
fbc:geneProduct="SAUSA300_0791"/>
                              <fbc:geneProductRef
fbc:geneProduct="SAUSA300_1496"/>
                              <fbc:geneProductRef
fbc:geneProduct="SAUSA300_1497"/>
                        </fbc:and>
                  </fbc:or>
            </fbc:geneProductAssociation>
      </reaction>

      <reaction metaid="R_GDNSACT" id="R_GDNSACT" name="glycosyl-
4,4'__Diaponeurosporenoate acyltransferase" reversible="false" fast="false"
fbc:lowerFluxBound="irr_lb" fbc:upperFluxBound="irr_ub">
            <listOfReactants>
                  <speciesReference species="M_gdnspate_c" stoichiometry="1"
constant="true"/>
                  <speciesReference species="M_mttdca_c" stoichiometry="1"
constant="true"/>
            </listOfReactants>
            <listOfProducts>
                  <speciesReference species="M_stphxln_c" stoichiometry="1"
constant="true"/>
            </listOfProducts>
            <fbc:geneProductAssociation>
                  <fbc:geneProductRef fbc:geneProduct="SAUSA300_2502"/>
            </fbc:geneProductAssociation>
      </reaction>

      <reaction metaid="R_GF6PTAr" id="R_GF6PTAr" name="Glutamine-fructose-6-
phosphate transaminase" reversible="true" fast="false"
fbc:lowerFluxBound="rev_lb" fbc:upperFluxBound="rev_ub">
            <listOfReactants>
                  <speciesReference species="M_gln__L_c" stoichiometry="1"
constant="true"/>
                  <speciesReference species="M_f6p_c" stoichiometry="1"
constant="true"/>
            </listOfReactants>
            <listOfProducts>
                  <speciesReference species="M_glu__L_c" stoichiometry="1"
constant="true"/>
                  <speciesReference species="M_gam6p_c" stoichiometry="1"
constant="true"/>
            </listOfProducts>
            <fbc:geneProductAssociation>
                  <fbc:geneProductRef fbc:geneProduct="SAUSA300_2104"/>
            </fbc:geneProductAssociation>
      </reaction>

      <reaction metaid="R_GGTT" id="R_GGTT"
name="geranylgeranyltranstransferase" reversible="false" fast="false"
fbc:lowerFluxBound="irr_lb" fbc:upperFluxBound="irr_ub">
            <listOfReactants>
```

```
                  <speciesReference species="M_ggdp_c" stoichiometry="1"
constant="true"/>
                  <speciesReference species="M_ipdp_c" stoichiometry="1"
constant="true"/>
            &lt;/listOfReactants&gt;
            &lt;listOfProducts&gt;
                  <speciesReference species="M_ppi_c" stoichiometry="1"
constant="true"/>
                  <speciesReference species="M_pendp_c" stoichiometry="1"
constant="true"/>
            &lt;/listOfProducts&gt;
            &lt;fbc:geneProductAssociation&gt;
                  &lt;fbc:or&gt;
                        &lt;fbc:geneProductRef fbc:geneProduct="SAUSA300_1359"/&gt;
                        &lt;fbc:geneProductRef fbc:geneProduct="SAUSA300_1361"/&gt;
                  &lt;/fbc:or&gt;
            &lt;/fbc:geneProductAssociation&gt;
      &lt;/reaction&gt;

      <reaction metaid="R_GHMT2r" id="R_GHMT2r" name="glycine
hydroxymethyltransferase, reversible" reversible="true" fast="false"
fbc:lowerFluxBound="rev_lb" fbc:upperFluxBound="rev_ub">
            &lt;listOfReactants&gt;
                  <speciesReference species="M_h2o_c" stoichiometry="1"
constant="true"/>
                  <speciesReference species="M_gly_c" stoichiometry="1"
constant="true"/>
                  <speciesReference species="M_mlthf_c" stoichiometry="1"
constant="true"/>
            &lt;/listOfReactants&gt;
            &lt;listOfProducts&gt;
                  <speciesReference species="M_thf_c" stoichiometry="1"
constant="true"/>
                  <speciesReference species="M_ser__L_c" stoichiometry="1"
constant="true"/>
            &lt;/listOfProducts&gt;
            &lt;fbc:geneProductAssociation&gt;
                  &lt;fbc:geneProductRef fbc:geneProduct="SAUSA300_2067"/&gt;
            &lt;/fbc:geneProductAssociation&gt;
      &lt;/reaction&gt;

      <reaction metaid="R_GK1" id="R_GK1" name="guanylate kinase (GMP:ATP)"
reversible="true" fast="false" fbc:lowerFluxBound="rev_lb"
fbc:upperFluxBound="rev_ub">
            &lt;listOfReactants&gt;
                  <speciesReference species="M_atp_c" stoichiometry="1"
constant="true"/>
                  <speciesReference species="M_gmp_c" stoichiometry="1"
constant="true"/>
            &lt;/listOfReactants&gt;
            &lt;listOfProducts&gt;
                  <speciesReference species="M_adp_c" stoichiometry="1"
constant="true"/>
                  <speciesReference species="M_gdp_c" stoichiometry="1"
constant="true"/>
            &lt;/listOfProducts&gt;
            &lt;fbc:geneProductAssociation&gt;
                  &lt;fbc:geneProductRef fbc:geneProduct="SAUSA300_1102"/&gt;
```

```
            </fbc:geneProductAssociation>
      </reaction>

      <reaction metaid="R_GLCAH" id="R_GLCAH" name="Glycocholate
amidohydrolase " reversible="true" fast="false" fbc:lowerFluxBound="rev_lb"
fbc:upperFluxBound="rev_ub">
            <listOfReactants>
                  <speciesReference species="M_h2o_c" stoichiometry="1"
constant="true"/>
                  <speciesReference species="M_gchola_c" stoichiometry="1"
constant="true"/>
            </listOfReactants>
            <listOfProducts>
                  <speciesReference species="M_gly_c" stoichiometry="1"
constant="true"/>
                  <speciesReference species="M_cholate_c" stoichiometry="1"
constant="true"/>
            </listOfProducts>
            <fbc:geneProductAssociation>
                  <fbc:geneProductRef fbc:geneProduct="SAUSA300_0269"/>
            </fbc:geneProductAssociation>
      </reaction>

      <reaction metaid="R_GLCBPP" id="R_GLCBPP" name="ATP D-glucose 6-
phosphotransferase " reversible="false" fast="false"
fbc:lowerFluxBound="irr_lb" fbc:upperFluxBound="irr_ub">
            <listOfReactants>
                  <speciesReference species="M_atp_c" stoichiometry="1"
constant="true"/>
                  <speciesReference species="M_glc__D__B_c" stoichiometry="1"
constant="true"/>
            </listOfReactants>
            <listOfProducts>
                  <speciesReference species="M_adp_c" stoichiometry="1"
constant="true"/>
                  <speciesReference species="M_g6p__B_c" stoichiometry="1"
constant="true"/>
            </listOfProducts>
            <fbc:geneProductAssociation>
                  <fbc:geneProductRef fbc:geneProduct="SAUSA300_1507"/>
            </fbc:geneProductAssociation>
      </reaction>

      <reaction metaid="R_GLCE" id="R_GLCE" name="alpha__D-Glucose 1-
epimerase " reversible="true" fast="false" fbc:lowerFluxBound="rev_lb"
fbc:upperFluxBound="rev_ub">
            <listOfReactants>
                  <speciesReference species="M_glc__D_c" stoichiometry="1"
constant="true"/>
            </listOfReactants>
            <listOfProducts>
                  <speciesReference species="M_glc__D__B_c" stoichiometry="1"
constant="true"/>
            </listOfProducts>
            <fbc:geneProductAssociation>
                  <fbc:geneProductRef fbc:geneProduct="SAUSA300_2285"/>
            </fbc:geneProductAssociation>
      </reaction>
```

```
      <reaction metaid="R_GLCNt2r" id="R_GLCNt2r" name="D-gluconate transport
via proton symport, reversible" reversible="true" fast="false"
fbc:lowerFluxBound="rev_lb" fbc:upperFluxBound="rev_ub">
            <listOfReactants>
                  <speciesReference species="M_h_e" stoichiometry="1"
constant="true"/>
                  <speciesReference species="M_glcn_e" stoichiometry="1"
constant="true"/>
            </listOfReactants>
            <listOfProducts>
                  <speciesReference species="M_h_c" stoichiometry="1"
constant="true"/>
                  <speciesReference species="M_glcn_c" stoichiometry="1"
constant="true"/>
            </listOfProducts>
            <fbc:geneProductAssociation>
                  <fbc:geneProductRef fbc:geneProduct="SAUSA300_2442"/>
            </fbc:geneProductAssociation>
      </reaction>

      <reaction metaid="R_GLCOAD" id="R_GLCOAD" name="Glutaryl-CoA acceptor 2
3-oxidoreductase decarboxylating " reversible="true" fast="false"
fbc:lowerFluxBound="rev_lb" fbc:upperFluxBound="rev_ub">
            <listOfReactants>
                  <speciesReference species="M_h_c" stoichiometry="1"
constant="true"/>
                  <speciesReference species="M_fad_c" stoichiometry="1"
constant="true"/>
                  <speciesReference species="M_glutcoa_c" stoichiometry="1"
constant="true"/>
            </listOfReactants>
            <listOfProducts>
                  <speciesReference species="M_b2coa_c" stoichiometry="1"
constant="true"/>
                  <speciesReference species="M_co2_c" stoichiometry="1"
constant="true"/>
                  <speciesReference species="M_fadh2_c" stoichiometry="1"
constant="true"/>
            </listOfProducts>
            <fbc:geneProductAssociation>
                  <fbc:geneProductRef fbc:geneProduct="SAUSA300_0227"/>
            </fbc:geneProductAssociation>
      </reaction>

      <reaction metaid="R_GLCPcom_SA" id="R_GLCPcom_SA" name="Glucosyl
Phosphoglycerol Combination (SA)" reversible="false" fast="false"
fbc:lowerFluxBound="irr_lb" fbc:upperFluxBound="irr_ub">
            <listOfReactants>
                  <speciesReference species="M_gtca3_c" stoichiometry="2"
constant="true"/>
                  <speciesReference species="M_gtca2_c" stoichiometry="2"
constant="true"/>
                  <speciesReference species="M_gtca1_c" stoichiometry="2"
constant="true"/>
            </listOfReactants>
            <listOfProducts>
```

```
                  <speciesReference species="M_glcp_SA_c" stoichiometry="1"
constant="true"/>
            </listOfProducts>
      </reaction>

      <reaction metaid="R_GLCpts" id="R_GLCpts" name="D-glucose transport via
PEP:Pyr PTS" reversible="false" fast="false" fbc:lowerFluxBound="irr_lb"
fbc:upperFluxBound="irr_ub">
            <listOfReactants>
                  <speciesReference species="M_pep_c" stoichiometry="1"
constant="true"/>
                  <speciesReference species="M_glc__D_e" stoichiometry="1"
constant="true"/>
            </listOfReactants>
            <listOfProducts>
                  <speciesReference species="M_g6p_c" stoichiometry="1"
constant="true"/>
                  <speciesReference species="M_pyr_c" stoichiometry="1"
constant="true"/>
            </listOfProducts>
            <fbc:geneProductAssociation>
                  <fbc:or>
                        <fbc:and>
                              <fbc:geneProductRef
fbc:geneProduct="SAUSA300_0236"/>
                              <fbc:geneProductRef
fbc:geneProduct="SAUSA300_0983"/>
                              <fbc:geneProductRef
fbc:geneProduct="SAUSA300_0984"/>
                        </fbc:and>
                        <fbc:and>
                              <fbc:geneProductRef
fbc:geneProduct="SAUSA300_0191"/>
                              <fbc:geneProductRef
fbc:geneProduct="SAUSA300_0236"/>
                              <fbc:geneProductRef
fbc:geneProduct="SAUSA300_RS13740"/>
                              <fbc:geneProductRef
fbc:geneProduct="SAUSA300_2210"/>
                        </fbc:and>
                  </fbc:or>
            </fbc:geneProductAssociation>
      </reaction>

      <reaction metaid="R_GLCRAL" id="R_GLCRAL" name="5__Dehydro-
4__Deoxyglucarate aldolase" reversible="false" fast="false"
fbc:lowerFluxBound="irr_lb" fbc:upperFluxBound="irr_ub">
            <listOfReactants>
                  <speciesReference species="M_5dh4dglc_c" stoichiometry="1"
constant="true"/>
            </listOfReactants>
            <listOfProducts>
                  <speciesReference species="M_pyr_c" stoichiometry="1"
constant="true"/>
                  <speciesReference species="M_2h3oppan_c" stoichiometry="1"
constant="true"/>
            </listOfProducts>
            <fbc:geneProductAssociation>
```

```
                  <fbc:geneProductRef fbc:geneProduct="sbnG"/>
            </fbc:geneProductAssociation>
      </reaction>

      <reaction metaid="R_GLCRD" id="R_GLCRD" name="glucarate dehydratase"
reversible="false" fast="false" fbc:lowerFluxBound="irr_lb"
fbc:upperFluxBound="irr_ub">
            <listOfReactants>
                  <speciesReference species="M_glcr_c" stoichiometry="1"
constant="true"/>
            </listOfReactants>
            <listOfProducts>
                  <speciesReference species="M_h2o_c" stoichiometry="1"
constant="true"/>
                  <speciesReference species="M_5dh4dglc_c" stoichiometry="1"
constant="true"/>
            </listOfProducts>
            <fbc:geneProductAssociation>
                  <fbc:and>
                        <fbc:geneProductRef fbc:geneProduct="gudD"/>
                        <fbc:geneProductRef fbc:geneProduct="gudX"/>
                  </fbc:and>
            </fbc:geneProductAssociation>
      </reaction>

      <reaction metaid="R_GLCRt2rpp" id="R_GLCRt2rpp" name="D-glucarate
transport via proton symport, reversible (periplasm)" reversible="true"
fast="false" fbc:lowerFluxBound="rev_lb" fbc:upperFluxBound="rev_ub">
            <listOfReactants>
                  <speciesReference species="M_glcr_p" stoichiometry="1"
constant="true"/>
                  <speciesReference species="M_h_p" stoichiometry="1"
constant="true"/>
            </listOfReactants>
            <listOfProducts>
                  <speciesReference species="M_h_c" stoichiometry="1"
constant="true"/>
                  <speciesReference species="M_glcr_c" stoichiometry="1"
constant="true"/>
            </listOfProducts>
            <fbc:geneProductAssociation>
                  <fbc:geneProductRef fbc:geneProduct="D3C55_04135"/>
            </fbc:geneProductAssociation>
      </reaction>

      <reaction metaid="R_GLCRtex" id="R_GLCRtex" name="D-glucarate transport
via diffusion (extracellular to periplasm)" reversible="true" fast="false"
fbc:lowerFluxBound="rev_lb" fbc:upperFluxBound="rev_ub">
            <listOfReactants>
                  <speciesReference species="M_glcr_e" stoichiometry="1"
constant="true"/>
            </listOfReactants>
            <listOfProducts>
                  <speciesReference species="M_glcr_p" stoichiometry="1"
constant="true"/>
            </listOfProducts>
      </reaction>
```

```
      <reaction metaid="R_GLCS1" id="R_GLCS1" name="glycogen synthase
(ADPGlc)" reversible="false" fast="false" fbc:lowerFluxBound="irr_lb"
fbc:upperFluxBound="irr_ub">
            &lt;listOfReactants&gt;
                  <speciesReference species="M_adpglc_c" stoichiometry="1"
constant="true"/>
            &lt;/listOfReactants&gt;
            &lt;listOfProducts&gt;
                  <speciesReference species="M_h_c" stoichiometry="1"
constant="true"/>
                  <speciesReference species="M_adp_c" stoichiometry="1"
constant="true"/>
                  <speciesReference species="M_glycogen_c" stoichiometry="1"
constant="true"/>
            &lt;/listOfProducts&gt;
            &lt;fbc:geneProductAssociation&gt;
                  &lt;fbc:geneProductRef fbc:geneProduct="SAUSA300_1349"/&gt;
            &lt;/fbc:geneProductAssociation&gt;
      &lt;/reaction&gt;

      <reaction metaid="R_GLCURt2rpp" id="R_GLCURt2rpp" name="D-glucuronate
transport via proton symport, reversible (periplasm)" reversible="true"
fast="false" fbc:lowerFluxBound="rev_lb" fbc:upperFluxBound="rev_ub">
            &lt;listOfReactants&gt;
                  <speciesReference species="M_h_p" stoichiometry="1"
constant="true"/>
                  <speciesReference species="M_glcur_p" stoichiometry="1"
constant="true"/>
            &lt;/listOfReactants&gt;
            &lt;listOfProducts&gt;
                  <speciesReference species="M_h_c" stoichiometry="1"
constant="true"/>
                  <speciesReference species="M_glcur_c" stoichiometry="1"
constant="true"/>
            &lt;/listOfProducts&gt;
            &lt;fbc:geneProductAssociation&gt;
                  &lt;fbc:geneProductRef fbc:geneProduct="SAUSA300_2449"/&gt;
            &lt;/fbc:geneProductAssociation&gt;
      &lt;/reaction&gt;

      <reaction metaid="R_GLCURtex" id="R_GLCURtex" name="D-glucuronat
transport via diffusion (extracellular to periplasm)" reversible="true"
fast="false" fbc:lowerFluxBound="rev_lb" fbc:upperFluxBound="rev_ub">
            &lt;listOfReactants&gt;
                  <speciesReference species="M_glcur_e" stoichiometry="1"
constant="true"/>
            &lt;/listOfReactants&gt;
            &lt;listOfProducts&gt;
                  <speciesReference species="M_glcur_p" stoichiometry="1"
constant="true"/>
            &lt;/listOfProducts&gt;
            &lt;fbc:geneProductAssociation&gt;
                  &lt;fbc:geneProductRef fbc:geneProduct="SAUSA300_RS13580"/&gt;
            &lt;/fbc:geneProductAssociation&gt;
      &lt;/reaction&gt;
```

```
      <reaction metaid="R_GLGC" id="R_GLGC" name="glucose-1-phosphate
adenylyltransferase" reversible="false" fast="false"
fbc:lowerFluxBound="irr_lb" fbc:upperFluxBound="irr_ub">
            <listOfReactants>
                  <speciesReference species="M_h_c" stoichiometry="1"
constant="true"/>
                  <speciesReference species="M_atp_c" stoichiometry="1"
constant="true"/>
                  <speciesReference species="M_g1p_c" stoichiometry="1"
constant="true"/>
            </listOfReactants>
            <listOfProducts>
                  <speciesReference species="M_ppi_c" stoichiometry="1"
constant="true"/>
                  <speciesReference species="M_adpglc_c" stoichiometry="1"
constant="true"/>
            </listOfProducts>
            <fbc:geneProductAssociation>
                  <fbc:geneProductRef fbc:geneProduct="SAUSA300_2439"/>
            </fbc:geneProductAssociation>
      </reaction>

      <reaction metaid="R_GLN_NAct" id="R_GLN_NAct" name="glutamine
transport" reversible="false" fast="false" fbc:lowerFluxBound="irr_lb"
fbc:upperFluxBound="irr_ub">
            <listOfReactants>
                  <speciesReference species="M_atp_c" stoichiometry="1"
constant="true"/>
                  <speciesReference species="M_gln__L_e" stoichiometry="1"
constant="true"/>
                  <speciesReference species="M_h2o_c" stoichiometry="1"
constant="true"/>
                  <speciesReference species="M_na1_e" stoichiometry="1"
constant="true"/>
            </listOfReactants>
            <listOfProducts>
                  <speciesReference species="M_adp_c" stoichiometry="1"
constant="true"/>
                  <speciesReference species="M_gln__L_c" stoichiometry="1"
constant="true"/>
                  <speciesReference species="M_h_c" stoichiometry="1"
constant="true"/>
                  <speciesReference species="M_na1_c" stoichiometry="1"
constant="true"/>
                  <speciesReference species="M_pi_c" stoichiometry="1"
constant="true"/>
            </listOfProducts>
      </reaction>

      <reaction metaid="R_GLNS" id="R_GLNS" name="glutamine synthetase"
reversible="false" fast="false" fbc:lowerFluxBound="irr_lb"
fbc:upperFluxBound="irr_ub">
            <listOfReactants>
                  <speciesReference species="M_nh4_c" stoichiometry="1"
constant="true"/>
                  <speciesReference species="M_glu__L_c" stoichiometry="1"
constant="true"/>
```

```
                  <speciesReference species="M_atp_c" stoichiometry="1"
constant="true"/>
            </listOfReactants>
            <listOfProducts>
                  <speciesReference species="M_h_c" stoichiometry="1"
constant="true"/>
                  <speciesReference species="M_pi_c" stoichiometry="1"
constant="true"/>
                  <speciesReference species="M_gln__L_c" stoichiometry="1"
constant="true"/>
                  <speciesReference species="M_adp_c" stoichiometry="1"
constant="true"/>
            </listOfProducts>
            <fbc:geneProductAssociation>
                  <fbc:geneProductRef fbc:geneProduct="SAUSA300_1201"/>
            </fbc:geneProductAssociation>
      </reaction>

      <reaction metaid="R_GLNSP1" id="R_GLNSP1" name="glutamine synthetase
(uaaAgld)" reversible="false" fast="false" fbc:lowerFluxBound="irr_lb"
fbc:upperFluxBound="irr_ub">
            <listOfReactants>
                  <speciesReference species="M_nh4_c" stoichiometry="1"
constant="true"/>
                  <speciesReference species="M_atp_c" stoichiometry="1"
constant="true"/>
                  <speciesReference species="M_uaaAgla_c" stoichiometry="1"
constant="true"/>
            </listOfReactants>
            <listOfProducts>
                  <speciesReference species="M_h_c" stoichiometry="1"
constant="true"/>
                  <speciesReference species="M_pi_c" stoichiometry="1"
constant="true"/>
                  <speciesReference species="M_adp_c" stoichiometry="1"
constant="true"/>
                  <speciesReference species="M_uaaAgtla_c" stoichiometry="1"
constant="true"/>
            </listOfProducts>
            <fbc:geneProductAssociation>
                  <fbc:geneProductRef fbc:geneProduct="SAUSA300_1201"/>
            </fbc:geneProductAssociation>
      </reaction>

      <reaction metaid="R_GLNSP2" id="R_GLNSP2" name="glutamine synthetase
(uaaGgla)" reversible="false" fast="false" fbc:lowerFluxBound="irr_lb"
fbc:upperFluxBound="irr_ub">
            <listOfReactants>
                  <speciesReference species="M_nh4_c" stoichiometry="1"
constant="true"/>
                  <speciesReference species="M_atp_c" stoichiometry="1"
constant="true"/>
                  <speciesReference species="M_uaaGgla_c" stoichiometry="1"
constant="true"/>
            </listOfReactants>
            <listOfProducts>
                  <speciesReference species="M_h_c" stoichiometry="1"
constant="true"/>
```

<speciesReference species="M_pi_c" stoichiometry="1" constant="true"/>
<speciesReference species="M_adp_c" stoichiometry="1" constant="true"/>
<speciesReference species="M_uaaGgtla_c" stoichiometry="1" constant="true"/>
</listOfProducts>
<fbc:geneProductAssociation>
<fbc:geneProductRef fbc:geneProduct="SAUSA300_1201"/>
</fbc:geneProductAssociation>
</reaction>

<reaction metaid="R_GLNSP3" id="R_GLNSP3" name="glutamine synthetase (uaagmda)" reversible="false" fast="false" fbc:lowerFluxBound="irr_lb" fbc:upperFluxBound="irr_ub">
<listOfReactants>
<speciesReference species="M_nh4_c" stoichiometry="1" constant="true"/>
<speciesReference species="M_atp_c" stoichiometry="1" constant="true"/>
<speciesReference species="M_uaagmda_c" stoichiometry="1" constant="true"/>
</listOfReactants>
<listOfProducts>
<speciesReference species="M_h_c" stoichiometry="1" constant="true"/>
<speciesReference species="M_pi_c" stoichiometry="1" constant="true"/>
<speciesReference species="M_adp_c" stoichiometry="1" constant="true"/>
<speciesReference species="M_uaagtmda_c" stoichiometry="1" constant="true"/>
</listOfProducts>
<fbc:geneProductAssociation>
<fbc:geneProductRef fbc:geneProduct="SAUSA300_1201"/>
</fbc:geneProductAssociation>
</reaction>

<reaction metaid="R_GLTPD" id="R_GLTPD" name="Galactitol-1-phosphate dehydrogenase" reversible="true" fast="false" fbc:lowerFluxBound="rev_lb" fbc:upperFluxBound="rev_ub">
<listOfReactants>
<speciesReference species="M_nad_c" stoichiometry="1" constant="true"/>
<speciesReference species="M_galt1p_c" stoichiometry="1" constant="true"/>
</listOfReactants>
<listOfProducts>
<speciesReference species="M_h_c" stoichiometry="1" constant="true"/>
<speciesReference species="M_nadh_c" stoichiometry="1" constant="true"/>
<speciesReference species="M_tag6p__D_c" stoichiometry="1" constant="true"/>
</listOfProducts>
<fbc:geneProductAssociation>
<fbc:geneProductRef fbc:geneProduct="SAUSA300_0242"/>
</fbc:geneProductAssociation>

```
      </reaction>

      <reaction metaid="R_GLUabc" id="R_GLUabc" name="L-glutamate transport
via ABC system" reversible="false" fast="false" fbc:lowerFluxBound="irr_lb"
fbc:upperFluxBound="irr_ub">
            <listOfReactants>
                  <speciesReference species="M_h2o_c" stoichiometry="1"
constant="true"/>
                  <speciesReference species="M_atp_c" stoichiometry="1"
constant="true"/>
                  <speciesReference species="M_glu__L_e" stoichiometry="1"
constant="true"/>
            </listOfReactants>
            <listOfProducts>
                  <speciesReference species="M_h_c" stoichiometry="1"
constant="true"/>
                  <speciesReference species="M_glu__L_c" stoichiometry="1"
constant="true"/>
                  <speciesReference species="M_pi_c" stoichiometry="1"
constant="true"/>
                  <speciesReference species="M_adp_c" stoichiometry="1"
constant="true"/>
            </listOfProducts>
            <fbc:geneProductAssociation>
                  <fbc:and>
                        <fbc:geneProductRef fbc:geneProduct="SAUSA300_1807"/>
                        <fbc:geneProductRef fbc:geneProduct="SAUSA300_1808"/>
                  </fbc:and>
            </fbc:geneProductAssociation>
      </reaction>

      <reaction metaid="R_GLUDx" id="R_GLUDx" name="glutamate dehydrogenase
(NAD)" reversible="false" fast="false" fbc:lowerFluxBound="irr_lb"
fbc:upperFluxBound="irr_ub">
            <listOfReactants>
                  <speciesReference species="M_h2o_c" stoichiometry="1"
constant="true"/>
                  <speciesReference species="M_glu__L_c" stoichiometry="1"
constant="true"/>
                  <speciesReference species="M_nad_c" stoichiometry="1"
constant="true"/>
            </listOfReactants>
            <listOfProducts>
                  <speciesReference species="M_h_c" stoichiometry="1"
constant="true"/>
                  <speciesReference species="M_nh4_c" stoichiometry="1"
constant="true"/>
                  <speciesReference species="M_akg_c" stoichiometry="1"
constant="true"/>
                  <speciesReference species="M_nadh_c" stoichiometry="1"
constant="true"/>
            </listOfProducts>
            <fbc:geneProductAssociation>
                  <fbc:geneProductRef fbc:geneProduct="SAUSA300_0861"/>
            </fbc:geneProductAssociation>
      </reaction>
```

```
      <reaction metaid="R_GLULOD" id="R_GLULOD" name="L-Glutamate ferredoxin
oxidoreductase transaminating " reversible="true" fast="false"
fbc:lowerFluxBound="rev_lb" fbc:upperFluxBound="rev_ub">
            <listOfReactants>
                  <speciesReference species="M_glu__L_c" stoichiometry="2"
constant="true"/>
                  <speciesReference species="M_fdxox_c" stoichiometry="2"
constant="true"/>
            </listOfReactants>
            <listOfProducts>
                  <speciesReference species="M_h_c" stoichiometry="2"
constant="true"/>
                  <speciesReference species="M_akg_c" stoichiometry="1"
constant="true"/>
                  <speciesReference species="M_gln__L_c" stoichiometry="1"
constant="true"/>
                  <speciesReference species="M_fdxrd_c" stoichiometry="2"
constant="true"/>
            </listOfProducts>
            <fbc:geneProductAssociation>
                  <fbc:geneProductRef fbc:geneProduct="SAUSA300_2404"/>
            </fbc:geneProductAssociation>
      </reaction>

      <reaction metaid="R_GLUPEP5" id="R_GLUPEP5" name="5-Glutamyl-peptide
amino-acid 5-glutamyltransferase " reversible="true" fast="false"
fbc:lowerFluxBound="rev_lb" fbc:upperFluxBound="rev_ub">
            <listOfReactants>
                  <speciesReference species="M_glu__L_c" stoichiometry="1"
constant="true"/>
                  <speciesReference species="M_cyoala_c" stoichiometry="1"
constant="true"/>
            </listOfReactants>
            <listOfProducts>
                  <speciesReference species="M_h2o_c" stoichiometry="1"
constant="true"/>
                  <speciesReference species="M_ggbca_c" stoichiometry="1"
constant="true"/>
            </listOfProducts>
            <fbc:geneProductAssociation>
                  <fbc:geneProductRef fbc:geneProduct="SAUSA300_0204"/>
            </fbc:geneProductAssociation>
      </reaction>

      <reaction metaid="R_GLUPRT" id="R_GLUPRT" name="glutamine
phosphoribosyldiphosphate amidotransferase" reversible="false" fast="false"
fbc:lowerFluxBound="irr_lb" fbc:upperFluxBound="irr_ub">
            <listOfReactants>
                  <speciesReference species="M_h2o_c" stoichiometry="1"
constant="true"/>
                  <speciesReference species="M_gln__L_c" stoichiometry="1"
constant="true"/>
                  <speciesReference species="M_prpp_c" stoichiometry="1"
constant="true"/>
            </listOfReactants>
            <listOfProducts>
                  <speciesReference species="M_glu__L_c" stoichiometry="1"
constant="true"/>
```

```
                  <speciesReference species="M_ppi_c" stoichiometry="1"
constant="true"/>
                  <speciesReference species="M_pram_c" stoichiometry="1"
constant="true"/>
            </listOfProducts>
            <fbc:geneProductAssociation>
                  <fbc:geneProductRef fbc:geneProduct="SAUSA300_0972"/>
            </fbc:geneProductAssociation>
      </reaction>

      <reaction metaid="R_GLUR" id="R_GLUR" name="glutamate racemase"
reversible="true" fast="false" fbc:lowerFluxBound="rev_lb"
fbc:upperFluxBound="rev_ub">
            <listOfReactants>
                  <speciesReference species="M_glu__L_c" stoichiometry="1"
constant="true"/>
            </listOfReactants>
            <listOfProducts>
                  <speciesReference species="M_glu__D_c" stoichiometry="1"
constant="true"/>
            </listOfProducts>
            <fbc:geneProductAssociation>
                  <fbc:geneProductRef fbc:geneProduct="SAUSA300_1049"/>
            </fbc:geneProductAssociation>
      </reaction>

      <reaction metaid="R_GLUSx" id="R_GLUSx" name="glutamate synthase
(NADH2)" reversible="false" fast="false" fbc:lowerFluxBound="irr_lb"
fbc:upperFluxBound="irr_ub">
            <listOfReactants>
                  <speciesReference species="M_h_c" stoichiometry="1"
constant="true"/>
                  <speciesReference species="M_akg_c" stoichiometry="1"
constant="true"/>
                  <speciesReference species="M_gln__L_c" stoichiometry="1"
constant="true"/>
                  <speciesReference species="M_nadh_c" stoichiometry="1"
constant="true"/>
            </listOfReactants>
            <listOfProducts>
                  <speciesReference species="M_glu__L_c" stoichiometry="2"
constant="true"/>
                  <speciesReference species="M_nad_c" stoichiometry="1"
constant="true"/>
            </listOfProducts>
            <fbc:geneProductAssociation>
                  <fbc:and>
                        <fbc:or>
                              <fbc:geneProductRef
fbc:geneProduct="SAUSA300_2404"/>
                              <fbc:geneProductRef
fbc:geneProduct="SAUSA300_0445"/>
                        </fbc:or>
                        <fbc:geneProductRef fbc:geneProduct="SAUSA300_0446"/>
                  </fbc:and>
            </fbc:geneProductAssociation>
      </reaction>
```

```
      <reaction metaid="R_GLUSy" id="R_GLUSy" name="glutamate synthase
(NADPH)" reversible="false" fast="false" fbc:lowerFluxBound="irr_lb"
fbc:upperFluxBound="irr_ub">
            <listOfReactants>
                  <speciesReference species="M_h_c" stoichiometry="1"
constant="true"/>
                  <speciesReference species="M_nadph_c" stoichiometry="1"
constant="true"/>
                  <speciesReference species="M_akg_c" stoichiometry="1"
constant="true"/>
                  <speciesReference species="M_gln__L_c" stoichiometry="1"
constant="true"/>
            </listOfReactants>
            <listOfProducts>
                  <speciesReference species="M_nadp_c" stoichiometry="1"
constant="true"/>
                  <speciesReference species="M_glu__L_c" stoichiometry="2"
constant="true"/>
            </listOfProducts>
            <fbc:geneProductAssociation>
                  <fbc:and>
                        <fbc:geneProductRef fbc:geneProduct="SAUSA300_0446"/>
                        <fbc:geneProductRef fbc:geneProduct="SAUSA300_0445"/>
                  </fbc:and>
            </fbc:geneProductAssociation>
      </reaction>

      <reaction metaid="R_GLUt2" id="R_GLUt2" name="L-glutamate transport in
via proton symport" reversible="false" fast="false"
fbc:lowerFluxBound="irr_lb" fbc:upperFluxBound="irr_ub">
            <listOfReactants>
                  <speciesReference species="M_h_e" stoichiometry="1"
constant="true"/>
                  <speciesReference species="M_glu__L_e" stoichiometry="1"
constant="true"/>
            </listOfReactants>
            <listOfProducts>
                  <speciesReference species="M_h_c" stoichiometry="1"
constant="true"/>
                  <speciesReference species="M_glu__L_c" stoichiometry="1"
constant="true"/>
            </listOfProducts>
            <fbc:geneProductAssociation>
                  <fbc:or>
                        <fbc:geneProductRef fbc:geneProduct="SAUSA300_2329"/>
                        <fbc:geneProductRef fbc:geneProduct="SAUSA300_0712"/>
                  </fbc:or>
            </fbc:geneProductAssociation>
      </reaction>

      <reaction metaid="R_GLUt4" id="R_GLUt4" name="Na+/glutamate symport"
reversible="false" fast="false" fbc:lowerFluxBound="irr_lb"
fbc:upperFluxBound="irr_ub">
            <listOfReactants>
                  <speciesReference species="M_na1_e" stoichiometry="1"
constant="true"/>
                  <speciesReference species="M_glu__L_e" stoichiometry="1"
constant="true"/>
```

```
            </listOfReactants>
            <listOfProducts>
                  <speciesReference species="M_glu__L_c" stoichiometry="1"
constant="true"/>
                  <speciesReference species="M_na1_c" stoichiometry="1"
constant="true"/>
            </listOfProducts>
            <fbc:geneProductAssociation>
                  <fbc:or>
                        <fbc:geneProductRef fbc:geneProduct="SAUSA300_2329"/>
                        <fbc:geneProductRef fbc:geneProduct="SAUSA300_2291"/>
                  </fbc:or>
            </fbc:geneProductAssociation>
      </reaction>

      <reaction metaid="R_GLUTRR" id="R_GLUTRR" name="glutamyl-tRNA
reductase" reversible="false" fast="false" fbc:lowerFluxBound="irr_lb"
fbc:upperFluxBound="irr_ub">
            <listOfReactants>
                  <speciesReference species="M_h_c" stoichiometry="1"
constant="true"/>
                  <speciesReference species="M_nadph_c" stoichiometry="1"
constant="true"/>
                  <speciesReference species="M_glutrna_c" stoichiometry="1"
constant="true"/>
            </listOfReactants>
            <listOfProducts>
                  <speciesReference species="M_nadp_c" stoichiometry="1"
constant="true"/>
                  <speciesReference species="M_glu1sa_c" stoichiometry="1"
constant="true"/>
                  <speciesReference species="M_trnaglu_c" stoichiometry="1"
constant="true"/>
            </listOfProducts>
            <fbc:geneProductAssociation>
                  <fbc:geneProductRef fbc:geneProduct="SAUSA300_1619"/>
            </fbc:geneProductAssociation>
      </reaction>

      <reaction metaid="R_GLUTRS" id="R_GLUTRS" name="Glutamyl-tRNA
synthetase" reversible="false" fast="false" fbc:lowerFluxBound="irr_lb"
fbc:upperFluxBound="irr_ub">
            <listOfReactants>
                  <speciesReference species="M_h_c" stoichiometry="1"
constant="true"/>
                  <speciesReference species="M_glu__L_c" stoichiometry="1"
constant="true"/>
                  <speciesReference species="M_atp_c" stoichiometry="1"
constant="true"/>
                  <speciesReference species="M_trnaglu_c" stoichiometry="1"
constant="true"/>
            </listOfReactants>
            <listOfProducts>
                  <speciesReference species="M_ppi_c" stoichiometry="1"
constant="true"/>
                  <speciesReference species="M_amp_c" stoichiometry="1"
constant="true"/>
```

```
                  <speciesReference species="M_glutrna_c" stoichiometry="1"
constant="true"/>
            &lt;/listOfProducts&gt;
            &lt;fbc:geneProductAssociation&gt;
                  &lt;fbc:geneProductRef fbc:geneProduct="SAUSA300_0513"/&gt;
            &lt;/fbc:geneProductAssociation&gt;
      &lt;/reaction&gt;

      <reaction metaid="R_GLXIII" id="R_GLXIII" name="glyoxalase III "
reversible="false" fast="false" fbc:lowerFluxBound="irr_lb"
fbc:upperFluxBound="irr_ub">
            &lt;listOfReactants&gt;
                  <speciesReference species="M_h2o_c" stoichiometry="1"
constant="true"/>
                  <speciesReference species="M_mthgxl_c" stoichiometry="1"
constant="true"/>
            &lt;/listOfReactants&gt;
            &lt;listOfProducts&gt;
                  <speciesReference species="M_h_c" stoichiometry="1"
constant="true"/>
                  <speciesReference species="M_lac__D_c" stoichiometry="1"
constant="true"/>
            &lt;/listOfProducts&gt;
            &lt;fbc:geneProductAssociation&gt;
                  &lt;fbc:geneProductRef fbc:geneProduct="SAUSA300_0536"/&gt;
            &lt;/fbc:geneProductAssociation&gt;
      &lt;/reaction&gt;

      <reaction metaid="R_GLXO1" id="R_GLXO1" name="glyoxylate oxidase"
reversible="true" fast="false" fbc:lowerFluxBound="rev_lb"
fbc:upperFluxBound="rev_ub">
            &lt;listOfReactants&gt;
                  <speciesReference species="M_h2o_c" stoichiometry="1"
constant="true"/>
                  <speciesReference species="M_nad_c" stoichiometry="1"
constant="true"/>
                  <speciesReference species="M_glx_c" stoichiometry="1"
constant="true"/>
            &lt;/listOfReactants&gt;
            &lt;listOfProducts&gt;
                  <speciesReference species="M_h_c" stoichiometry="2"
constant="true"/>
                  <speciesReference species="M_nadh_c" stoichiometry="1"
constant="true"/>
                  <speciesReference species="M_oxa_c" stoichiometry="1"
constant="true"/>
            &lt;/listOfProducts&gt;
            &lt;fbc:geneProductAssociation&gt;
                  &lt;fbc:or&gt;
                        &lt;fbc:geneProductRef fbc:geneProduct="SAUSA300_1901"/&gt;
                        &lt;fbc:geneProductRef fbc:geneProduct="SAUSA300_2076"/&gt;
                  &lt;/fbc:or&gt;
            &lt;/fbc:geneProductAssociation&gt;
      &lt;/reaction&gt;

      <reaction metaid="R_GLYALDDr" id="R_GLYALDDr" name="D-Glyceraldehyde
dehydrogenase" reversible="true" fast="false" fbc:lowerFluxBound="rev_lb"
fbc:upperFluxBound="rev_ub">
```

```
            <listOfReactants>
                  <speciesReference species="M_h2o_c" stoichiometry="1"
constant="true"/>
                  <speciesReference species="M_nad_c" stoichiometry="1"
constant="true"/>
                  <speciesReference species="M_glyald_c" stoichiometry="1"
constant="true"/>
            </listOfReactants>
            <listOfProducts>
                  <speciesReference species="M_h_c" stoichiometry="2"
constant="true"/>
                  <speciesReference species="M_nadh_c" stoichiometry="1"
constant="true"/>
                  <speciesReference species="M_glyc__R_c" stoichiometry="1"
constant="true"/>
            </listOfProducts>
            <fbc:geneProductAssociation>
                  <fbc:or>
                        <fbc:geneProductRef fbc:geneProduct="SAUSA300_1901"/>
                        <fbc:geneProductRef fbc:geneProduct="SAUSA300_2076"/>
                  </fbc:or>
            </fbc:geneProductAssociation>
      </reaction>

      <reaction metaid="R_GLYALDt" id="R_GLYALDt" name="Glyceraldehyde
facilitated diffusion" reversible="true" fast="false"
fbc:lowerFluxBound="rev_lb" fbc:upperFluxBound="rev_ub">
            <listOfReactants>
                  <speciesReference species="M_glyald_e" stoichiometry="1"
constant="true"/>
            </listOfReactants>
            <listOfProducts>
                  <speciesReference species="M_glyald_c" stoichiometry="1"
constant="true"/>
            </listOfProducts>
            <fbc:geneProductAssociation>
                  <fbc:geneProductRef fbc:geneProduct="SAUSA300_1191"/>
            </fbc:geneProductAssociation>
      </reaction>

      <reaction metaid="R_GLYASNabc" id="R_GLYASNabc" name="Dipeptide
transport via ABC system gly-asn " reversible="false" fast="false"
fbc:lowerFluxBound="irr_lb" fbc:upperFluxBound="irr_ub">
            <listOfReactants>
                  <speciesReference species="M_h2o_c" stoichiometry="1"
constant="true"/>
                  <speciesReference species="M_atp_c" stoichiometry="1"
constant="true"/>
                  <speciesReference species="M_gly_asn__L_e"
stoichiometry="1" constant="true"/>
            </listOfReactants>
            <listOfProducts>
                  <speciesReference species="M_h_c" stoichiometry="1"
constant="true"/>
                  <speciesReference species="M_pi_c" stoichiometry="1"
constant="true"/>
                  <speciesReference species="M_adp_c" stoichiometry="1"
constant="true"/>
```

```
                  <speciesReference species="M_gly_asn__L_c"
stoichiometry="1" constant="true"/>
            </listOfProducts>
            <fbc:geneProductAssociation>
                  <fbc:or>
                        <fbc:geneProductRef fbc:geneProduct="SAUSA300_2411"/>
                        <fbc:geneProductRef fbc:geneProduct="SAUSA300_0200"/>
                        <fbc:geneProductRef fbc:geneProduct="SAUSA300_0889"/>
                        <fbc:geneProductRef fbc:geneProduct="SAUSA300_2410"/>
                        <fbc:geneProductRef fbc:geneProduct="SAUSA300_0890"/>
                        <fbc:geneProductRef fbc:geneProduct="SAUSA300_0888"/>
                        <fbc:geneProductRef fbc:geneProduct="SAUSA300_2409"/>
                        <fbc:geneProductRef fbc:geneProduct="SAUSA300_0887"/>
                        <fbc:geneProductRef fbc:geneProduct="SAUSA300_0893"/>
                        <fbc:geneProductRef fbc:geneProduct="SAUSA300_0895"/>
                        <fbc:geneProductRef fbc:geneProduct="SAUSA300_0891"/>
                        <fbc:geneProductRef fbc:geneProduct="SAUSA300_0896"/>
                        <fbc:geneProductRef fbc:geneProduct="SAUSA300_0712"/>
                  </fbc:or>
            </fbc:geneProductAssociation>
      </reaction>

      <reaction metaid="R_GLYASPtr" id="R_GLYASPtr" name="Dipeptide transport
via ABC system gly-asp " reversible="false" fast="false"
fbc:lowerFluxBound="irr_lb" fbc:upperFluxBound="irr_ub">
            <listOfReactants>
                  <speciesReference species="M_h2o_c" stoichiometry="1"
constant="true"/>
                  <speciesReference species="M_atp_c" stoichiometry="1"
constant="true"/>
                  <speciesReference species="M_gly_asp__L_e"
stoichiometry="1" constant="true"/>
            </listOfReactants>
            <listOfProducts>
                  <speciesReference species="M_h_c" stoichiometry="1"
constant="true"/>
                  <speciesReference species="M_pi_c" stoichiometry="1"
constant="true"/>
                  <speciesReference species="M_adp_c" stoichiometry="1"
constant="true"/>
                  <speciesReference species="M_gly_asp__L_c"
stoichiometry="1" constant="true"/>
            </listOfProducts>
            <fbc:geneProductAssociation>
                  <fbc:or>
                        <fbc:geneProductRef fbc:geneProduct="SAUSA300_2411"/>
                        <fbc:geneProductRef fbc:geneProduct="SAUSA300_0200"/>
                        <fbc:geneProductRef fbc:geneProduct="SAUSA300_0889"/>
                        <fbc:geneProductRef fbc:geneProduct="SAUSA300_2410"/>
                        <fbc:geneProductRef fbc:geneProduct="SAUSA300_0890"/>
                        <fbc:geneProductRef fbc:geneProduct="SAUSA300_0888"/>
                        <fbc:geneProductRef fbc:geneProduct="SAUSA300_2409"/>
                        <fbc:geneProductRef fbc:geneProduct="SAUSA300_0887"/>
                        <fbc:geneProductRef fbc:geneProduct="SAUSA300_0893"/>
                        <fbc:geneProductRef fbc:geneProduct="SAUSA300_0895"/>
                        <fbc:geneProductRef fbc:geneProduct="SAUSA300_0891"/>
                        <fbc:geneProductRef fbc:geneProduct="SAUSA300_0896"/>
                        <fbc:geneProductRef fbc:geneProduct="SAUSA300_0712"/>
```

```
                </fbc:or>
            </fbc:geneProductAssociation>
        </reaction>

        <reaction metaid="R_GLYAT" id="R_GLYAT" name="glycine C-
acetyltransferase" reversible="false" fast="false"
fbc:lowerFluxBound="irr_lb" fbc:upperFluxBound="irr_ub">
            <listOfReactants>
                <speciesReference species="M_gly_c" stoichiometry="1"
constant="true"/>
                <speciesReference species="M_accoa_c" stoichiometry="1"
constant="true"/>
            </listOfReactants>
            <listOfProducts>
                <speciesReference species="M_coa_c" stoichiometry="1"
constant="true"/>
                <speciesReference species="M_2aobut_c" stoichiometry="1"
constant="true"/>
            </listOfProducts>
            <fbc:geneProductAssociation>
                <fbc:geneProductRef fbc:geneProduct="SAUSA300_0535"/>
            </fbc:geneProductAssociation>
        </reaction>

        <reaction metaid="R_GLYBt2r" id="R_GLYBt2r" name="Glycine betaine
transport via proton symport, reversible" reversible="false" fast="false"
fbc:lowerFluxBound="irr_lb" fbc:upperFluxBound="irr_ub">
            <listOfReactants>
                <speciesReference species="M_h_e" stoichiometry="1"
constant="true"/>
                <speciesReference species="M_glyb_e" stoichiometry="1"
constant="true"/>
            </listOfReactants>
            <listOfProducts>
                <speciesReference species="M_h_c" stoichiometry="1"
constant="true"/>
                <speciesReference species="M_glyb_c" stoichiometry="1"
constant="true"/>
            </listOfProducts>
            <fbc:geneProductAssociation>
                <fbc:or>
                    <fbc:geneProductRef fbc:geneProduct="SAUSA300_1245"/>
                    <fbc:geneProductRef fbc:geneProduct="SAUSA300_2145"/>
                </fbc:or>
            </fbc:geneProductAssociation>
        </reaction>

        <reaction metaid="R_GLYC3Pabc" id="R_GLYC3Pabc" name="sn-Glycerol 3-
phosphate transport via ABC system" reversible="false" fast="false"
fbc:lowerFluxBound="irr_lb" fbc:upperFluxBound="irr_ub">
            <listOfReactants>
                <speciesReference species="M_h2o_c" stoichiometry="1"
constant="true"/>
                <speciesReference species="M_atp_c" stoichiometry="1"
constant="true"/>
                <speciesReference species="M_glyc3p_e" stoichiometry="1"
constant="true"/>
            </listOfReactants>
```

```
            <listOfProducts>
                  <speciesReference species="M_h_c" stoichiometry="1"
constant="true"/>
                  <speciesReference species="M_pi_c" stoichiometry="1"
constant="true"/>
                  <speciesReference species="M_adp_c" stoichiometry="1"
constant="true"/>
                  <speciesReference species="M_glyc3p_c" stoichiometry="1"
constant="true"/>
            </listOfProducts>
            <fbc:geneProductAssociation>
                  <fbc:and>
                        <fbc:geneProductRef fbc:geneProduct="SAUSA300_0211"/>
                        <fbc:geneProductRef fbc:geneProduct="SAUSA300_0210"/>
                        <fbc:geneProductRef fbc:geneProduct="SAUSA300_0208"/>
                        <fbc:geneProductRef fbc:geneProduct="SAUSA300_0209"/>
                  </fbc:and>
            </fbc:geneProductAssociation>
      </reaction>

      <reaction metaid="R_GLYC3Pt6" id="R_GLYC3Pt6" name="Glycerol-3-
phosphate : phosphate antiporter" reversible="false" fast="false"
fbc:lowerFluxBound="irr_lb" fbc:upperFluxBound="irr_ub">
            <listOfReactants>
                  <speciesReference species="M_pi_c" stoichiometry="1"
constant="true"/>
                  <speciesReference species="M_glyc3p_e" stoichiometry="1"
constant="true"/>
            </listOfReactants>
            <listOfProducts>
                  <speciesReference species="M_glyc3p_c" stoichiometry="1"
constant="true"/>
                  <speciesReference species="M_pi_e" stoichiometry="1"
constant="true"/>
            </listOfProducts>
            <fbc:geneProductAssociation>
                  <fbc:or>
                        <fbc:geneProductRef fbc:geneProduct="SAUSA300_0216"/>
                        <fbc:geneProductRef fbc:geneProduct="SAUSA300_0337"/>
                  </fbc:or>
            </fbc:geneProductAssociation>
      </reaction>

      <reaction metaid="R_GLYCK" id="R_GLYCK" name="glycerate kinase"
reversible="false" fast="false" fbc:lowerFluxBound="irr_lb"
fbc:upperFluxBound="irr_ub">
            <listOfReactants>
                  <speciesReference species="M_atp_c" stoichiometry="1"
constant="true"/>
                  <speciesReference species="M_glyc__R_c" stoichiometry="1"
constant="true"/>
            </listOfReactants>
            <listOfProducts>
                  <speciesReference species="M_adp_c" stoichiometry="1"
constant="true"/>
                  <speciesReference species="M_3pg_c" stoichiometry="1"
constant="true"/>
            </listOfProducts>
```

```
            &lt;fbc:geneProductAssociation&gt;
                  &lt;fbc:or&gt;
                        &lt;fbc:geneProductRef fbc:geneProduct="SAUSA300_2377"/&gt;
                        &lt;fbc:geneProductRef fbc:geneProduct="SAUSA300_0726"/&gt;
                  &lt;/fbc:or&gt;
            &lt;/fbc:geneProductAssociation&gt;
      &lt;/reaction&gt;

      <reaction metaid="R_GLYCt" id="R_GLYCt" name="glycerol transport via
channel" reversible="false" fast="false" fbc:lowerFluxBound="irr_lb"
fbc:upperFluxBound="irr_ub">
            &lt;listOfReactants&gt;
                  <speciesReference species="M_h_e" stoichiometry="1"
constant="true"/>
                  <speciesReference species="M_glyc3p_e" stoichiometry="1"
constant="true"/>
            &lt;/listOfReactants&gt;
            &lt;listOfProducts&gt;
                  <speciesReference species="M_h_c" stoichiometry="1"
constant="true"/>
                  <speciesReference species="M_glyc3p_c" stoichiometry="1"
constant="true"/>
            &lt;/listOfProducts&gt;
            &lt;fbc:geneProductAssociation&gt;
                  &lt;fbc:or&gt;
                        &lt;fbc:geneProductRef fbc:geneProduct="SAUSA300_0216"/&gt;
                        &lt;fbc:geneProductRef fbc:geneProduct="SAUSA300_0337"/&gt;
                  &lt;/fbc:or&gt;
            &lt;/fbc:geneProductAssociation&gt;
      &lt;/reaction&gt;

      <reaction metaid="R_GLYCt2" id="R_GLYCt2" name="glycerol transport via
symport" reversible="true" fast="false" fbc:lowerFluxBound="rev_lb"
fbc:upperFluxBound="rev_ub">
            &lt;listOfReactants&gt;
                  <speciesReference species="M_glyc_c" stoichiometry="1"
constant="true"/>
            &lt;/listOfReactants&gt;
            &lt;listOfProducts&gt;
                  <speciesReference species="M_glyc_e" stoichiometry="1"
constant="true"/>
            &lt;/listOfProducts&gt;
            &lt;fbc:geneProductAssociation&gt;
                  &lt;fbc:geneProductRef fbc:geneProduct="SAUSA300_1191"/&gt;
            &lt;/fbc:geneProductAssociation&gt;
      &lt;/reaction&gt;

      <reaction metaid="R_GLYCYSabc" id="R_GLYCYSabc" name="Gly-Cys ABC
transporters " reversible="false" fast="false" fbc:lowerFluxBound="irr_lb"
fbc:upperFluxBound="irr_ub">
            &lt;listOfReactants&gt;
                  <speciesReference species="M_h2o_c" stoichiometry="1"
constant="true"/>
                  <speciesReference species="M_atp_c" stoichiometry="1"
constant="true"/>
                  <speciesReference species="M_gly_cys_e" stoichiometry="1"
constant="true"/>
            &lt;/listOfReactants&gt;
```

```
            <listOfProducts>
                  <speciesReference species="M_pi_c" stoichiometry="1"
constant="true"/>
                  <speciesReference species="M_adp_c" stoichiometry="1"
constant="true"/>
                  <speciesReference species="M_gly_cys_c" stoichiometry="1"
constant="true"/>
            </listOfProducts>
            <fbc:geneProductAssociation>
                  <fbc:or>
                        <fbc:geneProductRef fbc:geneProduct="SAUSA300_2411"/>
                        <fbc:geneProductRef fbc:geneProduct="SAUSA300_0200"/>
                        <fbc:geneProductRef fbc:geneProduct="SAUSA300_0712"/>
                  </fbc:or>
            </fbc:geneProductAssociation>
      </reaction>

      <reaction metaid="R_GLYCYSAP" id="R_GLYCYSAP" name="Gly-Cys
aminopeptidase " reversible="true" fast="false" fbc:lowerFluxBound="rev_lb"
fbc:upperFluxBound="rev_ub">
            <listOfReactants>
                  <speciesReference species="M_h2o_c" stoichiometry="1"
constant="true"/>
                  <speciesReference species="M_gly_cys_c" stoichiometry="1"
constant="true"/>
            </listOfReactants>
            <listOfProducts>
                  <speciesReference species="M_gly_c" stoichiometry="1"
constant="true"/>
                  <speciesReference species="M_cys__L_c" stoichiometry="1"
constant="true"/>
            </listOfProducts>
            <fbc:geneProductAssociation>
                  <fbc:or>
                        <fbc:geneProductRef fbc:geneProduct="SAUSA300_1491"/>
                        <fbc:geneProductRef fbc:geneProduct="SAUSA300_1869"/>
                        <fbc:geneProductRef fbc:geneProduct="SAUSA300_0845"/>
                        <fbc:geneProductRef fbc:geneProduct="SAUSA300_1860"/>
                  </fbc:or>
            </fbc:geneProductAssociation>
      </reaction>

      <reaction metaid="R_GLYGLNtr" id="R_GLYGLNtr" name="Dipeptide transport
via ABC system gly-gln " reversible="false" fast="false"
fbc:lowerFluxBound="irr_lb" fbc:upperFluxBound="irr_ub">
            <listOfReactants>
                  <speciesReference species="M_h2o_c" stoichiometry="1"
constant="true"/>
                  <speciesReference species="M_atp_c" stoichiometry="1"
constant="true"/>
                  <speciesReference species="M_gly_gln_e" stoichiometry="1"
constant="true"/>
            </listOfReactants>
            <listOfProducts>
                  <speciesReference species="M_h_c" stoichiometry="1"
constant="true"/>
                  <speciesReference species="M_pi_c" stoichiometry="1"
constant="true"/>
```

```
                  <speciesReference species="M_adp_c" stoichiometry="1"
constant="true"/>
                  <speciesReference species="M_gly_gln_c" stoichiometry="1"
constant="true"/>
            </listOfProducts>
            <fbc:geneProductAssociation>
                  <fbc:or>
                        <fbc:geneProductRef fbc:geneProduct="SAUSA300_2411"/>
                        <fbc:geneProductRef fbc:geneProduct="SAUSA300_0200"/>
                        <fbc:geneProductRef fbc:geneProduct="SAUSA300_0889"/>
                        <fbc:geneProductRef fbc:geneProduct="SAUSA300_2410"/>
                        <fbc:geneProductRef fbc:geneProduct="SAUSA300_0890"/>
                        <fbc:geneProductRef fbc:geneProduct="SAUSA300_0888"/>
                        <fbc:geneProductRef fbc:geneProduct="SAUSA300_2409"/>
                        <fbc:geneProductRef fbc:geneProduct="SAUSA300_0887"/>
                        <fbc:geneProductRef fbc:geneProduct="SAUSA300_0893"/>
                        <fbc:geneProductRef fbc:geneProduct="SAUSA300_0895"/>
                        <fbc:geneProductRef fbc:geneProduct="SAUSA300_0891"/>
                        <fbc:geneProductRef fbc:geneProduct="SAUSA300_0896"/>
                        <fbc:geneProductRef fbc:geneProduct="SAUSA300_0712"/>
                  </fbc:or>
            </fbc:geneProductAssociation>
      </reaction>

      <reaction metaid="R_GLYGLUtr" id="R_GLYGLUtr" name="Dipeptide transport
via ABC system gly-glu " reversible="false" fast="false"
fbc:lowerFluxBound="irr_lb" fbc:upperFluxBound="irr_ub">
            <listOfReactants>
                  <speciesReference species="M_h2o_c" stoichiometry="1"
constant="true"/>
                  <speciesReference species="M_atp_c" stoichiometry="1"
constant="true"/>
                  <speciesReference species="M_gly_glu__L_e"
stoichiometry="1" constant="true"/>
            </listOfReactants>
            <listOfProducts>
                  <speciesReference species="M_h_c" stoichiometry="1"
constant="true"/>
                  <speciesReference species="M_pi_c" stoichiometry="1"
constant="true"/>
                  <speciesReference species="M_adp_c" stoichiometry="1"
constant="true"/>
                  <speciesReference species="M_gly_glu__L_c"
stoichiometry="1" constant="true"/>
            </listOfProducts>
            <fbc:geneProductAssociation>
                  <fbc:or>
                        <fbc:geneProductRef fbc:geneProduct="SAUSA300_2411"/>
                        <fbc:geneProductRef fbc:geneProduct="SAUSA300_0200"/>
                        <fbc:geneProductRef fbc:geneProduct="SAUSA300_0889"/>
                        <fbc:geneProductRef fbc:geneProduct="SAUSA300_2410"/>
                        <fbc:geneProductRef fbc:geneProduct="SAUSA300_0890"/>
                        <fbc:geneProductRef fbc:geneProduct="SAUSA300_0888"/>
                        <fbc:geneProductRef fbc:geneProduct="SAUSA300_2409"/>
                        <fbc:geneProductRef fbc:geneProduct="SAUSA300_0887"/>
                        <fbc:geneProductRef fbc:geneProduct="SAUSA300_0893"/>
                        <fbc:geneProductRef fbc:geneProduct="SAUSA300_0895"/>
                        <fbc:geneProductRef fbc:geneProduct="SAUSA300_0891"/>
```

```
                        <fbc:geneProductRef fbc:geneProduct="SAUSA300_0896"/>
                        <fbc:geneProductRef fbc:geneProduct="SAUSA300_0712"/>
                  </fbc:or>
            </fbc:geneProductAssociation>
      </reaction>

      <reaction metaid="R_GLYKr" id="R_GLYKr" name="glycerol kinase
(reversible)" reversible="false" fast="false" fbc:lowerFluxBound="irr_lb"
fbc:upperFluxBound="irr_ub">
            <listOfReactants>
                  <speciesReference species="M_atp_c" stoichiometry="1"
constant="true"/>
                  <speciesReference species="M_glyc_c" stoichiometry="1"
constant="true"/>
            </listOfReactants>
            <listOfProducts>
                  <speciesReference species="M_adp_c" stoichiometry="1"
constant="true"/>
                  <speciesReference species="M_glyc3p_c" stoichiometry="1"
constant="true"/>
            </listOfProducts>
            <fbc:geneProductAssociation>
                  <fbc:geneProductRef fbc:geneProduct="SAUSA300_1192"/>
            </fbc:geneProductAssociation>
      </reaction>

      <reaction metaid="R_GLYLEUAP" id="R_GLYLEUAP" name="Gly__Leu
aminopeptidase " reversible="true" fast="false" fbc:lowerFluxBound="rev_lb"
fbc:upperFluxBound="rev_ub">
            <listOfReactants>
                  <speciesReference species="M_h2o_c" stoichiometry="1"
constant="true"/>
                  <speciesReference species="M_gly_leu_c" stoichiometry="1"
constant="true"/>
            </listOfReactants>
            <listOfProducts>
                  <speciesReference species="M_gly_c" stoichiometry="1"
constant="true"/>
                  <speciesReference species="M_leu__L_c" stoichiometry="1"
constant="true"/>
            </listOfProducts>
            <fbc:geneProductAssociation>
                  <fbc:or>
                        <fbc:geneProductRef fbc:geneProduct="SAUSA300_1491"/>
                        <fbc:geneProductRef fbc:geneProduct="SAUSA300_1869"/>
                        <fbc:geneProductRef fbc:geneProduct="SAUSA300_0845"/>
                        <fbc:geneProductRef fbc:geneProduct="SAUSA300_1860"/>
                  </fbc:or>
            </fbc:geneProductAssociation>
      </reaction>

      <reaction metaid="R_GLYLEUtr" id="R_GLYLEUtr" name="Gly__Leu ABC
transporters " reversible="false" fast="false" fbc:lowerFluxBound="irr_lb"
fbc:upperFluxBound="irr_ub">
            <listOfReactants>
                  <speciesReference species="M_h2o_c" stoichiometry="1"
constant="true"/>
```

```
                  <speciesReference species="M_atp_c" stoichiometry="1"
constant="true"/>
                  <speciesReference species="M_gly_leu_e" stoichiometry="1"
constant="true"/>
            </listOfReactants>
            <listOfProducts>
                  <speciesReference species="M_pi_c" stoichiometry="1"
constant="true"/>
                  <speciesReference species="M_adp_c" stoichiometry="1"
constant="true"/>
                  <speciesReference species="M_gly_leu_c" stoichiometry="1"
constant="true"/>
            </listOfProducts>
            <fbc:geneProductAssociation>
                  <fbc:or>
                        <fbc:geneProductRef fbc:geneProduct="SAUSA300_2411"/>
                        <fbc:geneProductRef fbc:geneProduct="SAUSA300_0712"/>
                  </fbc:or>
            </fbc:geneProductAssociation>
      </reaction>

      <reaction metaid="R_GLYMETtr" id="R_GLYMETtr" name="Dipeptide transport
via ABC system gly-met " reversible="false" fast="false"
fbc:lowerFluxBound="irr_lb" fbc:upperFluxBound="irr_ub">
            <listOfReactants>
                  <speciesReference species="M_h2o_c" stoichiometry="1"
constant="true"/>
                  <speciesReference species="M_atp_c" stoichiometry="1"
constant="true"/>
                  <speciesReference species="M_gly_met_e" stoichiometry="1"
constant="true"/>
            </listOfReactants>
            <listOfProducts>
                  <speciesReference species="M_h_c" stoichiometry="1"
constant="true"/>
                  <speciesReference species="M_pi_c" stoichiometry="1"
constant="true"/>
                  <speciesReference species="M_adp_c" stoichiometry="1"
constant="true"/>
                  <speciesReference species="M_gly_met_c" stoichiometry="1"
constant="true"/>
            </listOfProducts>
            <fbc:geneProductAssociation>
                  <fbc:or>
                        <fbc:geneProductRef fbc:geneProduct="SAUSA300_2411"/>
                        <fbc:geneProductRef fbc:geneProduct="SAUSA300_0200"/>
                        <fbc:geneProductRef fbc:geneProduct="SAUSA300_0889"/>
                        <fbc:geneProductRef fbc:geneProduct="SAUSA300_2410"/>
                        <fbc:geneProductRef fbc:geneProduct="SAUSA300_0890"/>
                        <fbc:geneProductRef fbc:geneProduct="SAUSA300_0888"/>
                        <fbc:geneProductRef fbc:geneProduct="SAUSA300_2409"/>
                        <fbc:geneProductRef fbc:geneProduct="SAUSA300_0887"/>
                        <fbc:geneProductRef fbc:geneProduct="SAUSA300_0893"/>
                        <fbc:geneProductRef fbc:geneProduct="SAUSA300_0895"/>
                        <fbc:geneProductRef fbc:geneProduct="SAUSA300_0891"/>
                        <fbc:geneProductRef fbc:geneProduct="SAUSA300_0896"/>
                        <fbc:geneProductRef fbc:geneProduct="SAUSA300_0712"/>
                  </fbc:or>
```

```
            </fbc:geneProductAssociation>
      </reaction>

      <reaction metaid="R_GLYOX" id="R_GLYOX" name="hydroxyacylglutathione
hydrolase" reversible="false" fast="false" fbc:lowerFluxBound="irr_lb"
fbc:upperFluxBound="irr_ub">
            <listOfReactants>
                  <speciesReference species="M_h2o_c" stoichiometry="1"
constant="true"/>
                  <speciesReference species="M_lgt__S_c" stoichiometry="1"
constant="true"/>
            </listOfReactants>
            <listOfProducts>
                  <speciesReference species="M_h_c" stoichiometry="1"
constant="true"/>
                  <speciesReference species="M_lac__D_c" stoichiometry="1"
constant="true"/>
                  <speciesReference species="M_gthrd_c" stoichiometry="1"
constant="true"/>
            </listOfProducts>
            <fbc:geneProductAssociation>
                  <fbc:or>
                        <fbc:geneProductRef fbc:geneProduct="SAUSA300_1505"/>
                        <fbc:geneProductRef fbc:geneProduct="SAUSA300_0536"/>
                  </fbc:or>
            </fbc:geneProductAssociation>
      </reaction>

      <reaction metaid="R_GLYPHEAP" id="R_GLYPHEAP" name="Gly-Phe
aminopeptidase " reversible="true" fast="false" fbc:lowerFluxBound="rev_lb"
fbc:upperFluxBound="rev_ub">
            <listOfReactants>
                  <speciesReference species="M_h2o_c" stoichiometry="1"
constant="true"/>
                  <speciesReference species="M_gly_phe_c" stoichiometry="1"
constant="true"/>
            </listOfReactants>
            <listOfProducts>
                  <speciesReference species="M_gly_c" stoichiometry="1"
constant="true"/>
                  <speciesReference species="M_phe__L_c" stoichiometry="1"
constant="true"/>
            </listOfProducts>
            <fbc:geneProductAssociation>
                  <fbc:or>
                        <fbc:geneProductRef fbc:geneProduct="SAUSA300_1491"/>
                        <fbc:geneProductRef fbc:geneProduct="SAUSA300_1869"/>
                        <fbc:geneProductRef fbc:geneProduct="SAUSA300_0845"/>
                        <fbc:geneProductRef fbc:geneProduct="SAUSA300_1860"/>
                  </fbc:or>
            </fbc:geneProductAssociation>
      </reaction>

      <reaction metaid="R_GLYPHEtr" id="R_GLYPHEtr" name="Gly-Phe ABC
transporters " reversible="false" fast="false" fbc:lowerFluxBound="irr_lb"
fbc:upperFluxBound="irr_ub">
            <listOfReactants>
```

```
                  <speciesReference species="M_h2o_c" stoichiometry="1"
constant="true"/>
                  <speciesReference species="M_atp_c" stoichiometry="1"
constant="true"/>
                  <speciesReference species="M_gly_phe_e" stoichiometry="1"
constant="true"/>
            </listOfReactants>
            <listOfProducts>
                  <speciesReference species="M_pi_c" stoichiometry="1"
constant="true"/>
                  <speciesReference species="M_adp_c" stoichiometry="1"
constant="true"/>
                  <speciesReference species="M_gly_phe_c" stoichiometry="1"
constant="true"/>
            </listOfProducts>
            <fbc:geneProductAssociation>
                  <fbc:or>
                        <fbc:geneProductRef fbc:geneProduct="SAUSA300_2411"/>
                        <fbc:geneProductRef fbc:geneProduct="SAUSA300_0712"/>
                  </fbc:or>
            </fbc:geneProductAssociation>
      </reaction>

      <reaction metaid="R_GLYPRO__Ltr" id="R_GLYPRO__Ltr" name="Dipeptide
transport via ABC system gly__pro__L " reversible="false" fast="false"
fbc:lowerFluxBound="irr_lb" fbc:upperFluxBound="irr_ub">
            <listOfReactants>
                  <speciesReference species="M_h2o_c" stoichiometry="1"
constant="true"/>
                  <speciesReference species="M_atp_c" stoichiometry="1"
constant="true"/>
                  <speciesReference species="M_gly__pro__L_e"
stoichiometry="1" constant="true"/>
            </listOfReactants>
            <listOfProducts>
                  <speciesReference species="M_h_c" stoichiometry="1"
constant="true"/>
                  <speciesReference species="M_pi_c" stoichiometry="1"
constant="true"/>
                  <speciesReference species="M_adp_c" stoichiometry="1"
constant="true"/>
                  <speciesReference species="M_gly__pro__L_c"
stoichiometry="1" constant="true"/>
            </listOfProducts>
            <fbc:geneProductAssociation>
                  <fbc:or>
                        <fbc:geneProductRef fbc:geneProduct="SAUSA300_2411"/>
                        <fbc:geneProductRef fbc:geneProduct="SAUSA300_0200"/>
                        <fbc:geneProductRef fbc:geneProduct="SAUSA300_0889"/>
                        <fbc:geneProductRef fbc:geneProduct="SAUSA300_2410"/>
                        <fbc:geneProductRef fbc:geneProduct="SAUSA300_0890"/>
                        <fbc:geneProductRef fbc:geneProduct="SAUSA300_0888"/>
                        <fbc:geneProductRef fbc:geneProduct="SAUSA300_2409"/>
                        <fbc:geneProductRef fbc:geneProduct="SAUSA300_0887"/>
                        <fbc:geneProductRef fbc:geneProduct="SAUSA300_0893"/>
                        <fbc:geneProductRef fbc:geneProduct="SAUSA300_0895"/>
                        <fbc:geneProductRef fbc:geneProduct="SAUSA300_0891"/>
                        <fbc:geneProductRef fbc:geneProduct="SAUSA300_0896"/>
```

```
                        <fbc:geneProductRef fbc:geneProduct="SAUSA300_0712"/>
                  </fbc:or>
            </fbc:geneProductAssociation>
      </reaction>

      <reaction metaid="R_GLYt2" id="R_GLYt2" name="glycine transport in via
proton symport" reversible="true" fast="false" fbc:lowerFluxBound="rev_lb"
fbc:upperFluxBound="rev_ub">
            <listOfReactants>
                  <speciesReference species="M_h_e" stoichiometry="1"
constant="true"/>
                  <speciesReference species="M_gly_e" stoichiometry="1"
constant="true"/>
            </listOfReactants>
            <listOfProducts>
                  <speciesReference species="M_h_c" stoichiometry="1"
constant="true"/>
                  <speciesReference species="M_gly_c" stoichiometry="1"
constant="true"/>
            </listOfProducts>
            <fbc:geneProductAssociation>
                  <fbc:or>
                        <fbc:geneProductRef fbc:geneProduct="SAUSA300_1642"/>
                        <fbc:geneProductRef fbc:geneProduct="SA451515_2543"/>
                        <fbc:geneProductRef fbc:geneProduct="SAUSA300_0914"/>
                  </fbc:or>
            </fbc:geneProductAssociation>
      </reaction>

      <reaction metaid="R_GLYTRS" id="R_GLYTRS" name="Glycyl-tRNA synthetase"
reversible="false" fast="false" fbc:lowerFluxBound="irr_lb"
fbc:upperFluxBound="irr_ub">
            <listOfReactants>
                  <speciesReference species="M_atp_c" stoichiometry="1"
constant="true"/>
                  <speciesReference species="M_gly_c" stoichiometry="1"
constant="true"/>
                  <speciesReference species="M_trnagly_c" stoichiometry="1"
constant="true"/>
            </listOfReactants>
            <listOfProducts>
                  <speciesReference species="M_ppi_c" stoichiometry="1"
constant="true"/>
                  <speciesReference species="M_amp_c" stoichiometry="1"
constant="true"/>
                  <speciesReference species="M_glytrna_c" stoichiometry="1"
constant="true"/>
            </listOfProducts>
            <fbc:geneProductAssociation>
                  <fbc:geneProductRef fbc:geneProduct="SAUSA300_1525"/>
            </fbc:geneProductAssociation>
      </reaction>

      <reaction metaid="R_GLYTYRabc" id="R_GLYTYRabc" name="Gly-Try ABC
transporters " reversible="false" fast="false" fbc:lowerFluxBound="irr_lb"
fbc:upperFluxBound="irr_ub">
            <listOfReactants>
```

```
                  <speciesReference species="M_h2o_c" stoichiometry="1"
constant="true"/>
                  <speciesReference species="M_atp_c" stoichiometry="1"
constant="true"/>
                  <speciesReference species="M_gly_tyr_e" stoichiometry="1"
constant="true"/>
            </listOfReactants>
            <listOfProducts>
                  <speciesReference species="M_pi_c" stoichiometry="1"
constant="true"/>
                  <speciesReference species="M_adp_c" stoichiometry="1"
constant="true"/>
                  <speciesReference species="M_gly_tyr_c" stoichiometry="1"
constant="true"/>
            </listOfProducts>
            <fbc:geneProductAssociation>
                  <fbc:or>
                        <fbc:geneProductRef fbc:geneProduct="SAUSA300_2411"/>
                        <fbc:geneProductRef fbc:geneProduct="SAUSA300_0712"/>
                  </fbc:or>
            </fbc:geneProductAssociation>
      </reaction>

      <reaction metaid="R_GLYTYRAP" id="R_GLYTYRAP" name="Gly-Try
aminopeptidase " reversible="true" fast="false" fbc:lowerFluxBound="rev_lb"
fbc:upperFluxBound="rev_ub">
            <listOfReactants>
                  <speciesReference species="M_h2o_c" stoichiometry="1"
constant="true"/>
                  <speciesReference species="M_gly_tyr_c" stoichiometry="1"
constant="true"/>
            </listOfReactants>
            <listOfProducts>
                  <speciesReference species="M_gly_c" stoichiometry="1"
constant="true"/>
                  <speciesReference species="M_tyr__L_c" stoichiometry="1"
constant="true"/>
            </listOfProducts>
            <fbc:geneProductAssociation>
                  <fbc:or>
                        <fbc:geneProductRef fbc:geneProduct="SAUSA300_1491"/>
                        <fbc:geneProductRef fbc:geneProduct="SAUSA300_1869"/>
                        <fbc:geneProductRef fbc:geneProduct="SAUSA300_0845"/>
                        <fbc:geneProductRef fbc:geneProduct="SAUSA300_1860"/>
                  </fbc:or>
            </fbc:geneProductAssociation>
      </reaction>

      <reaction metaid="R_GMHEPAT" id="R_GMHEPAT" name="D-glycero__D-manno-
hepose 1-phosphate adenyltransferase" reversible="false" fast="false"
fbc:lowerFluxBound="irr_lb" fbc:upperFluxBound="irr_ub">
            <listOfReactants>
                  <speciesReference species="M_h_c" stoichiometry="1"
constant="true"/>
                  <speciesReference species="M_atp_c" stoichiometry="1"
constant="true"/>
                  <speciesReference species="M_gmhep1p_c" stoichiometry="1"
constant="true"/>
```

```
			</listOfReactants>
			<listOfProducts>
				<speciesReference species="M_ppi_c" stoichiometry="1"
constant="true"/>
				<speciesReference species="M_adphep__DD_c"
stoichiometry="1" constant="true"/>
			</listOfProducts>
			<fbc:geneProductAssociation>
				<fbc:geneProductRef fbc:geneProduct="SAUSA300_0628"/>
			</fbc:geneProductAssociation>
		</reaction>

		<reaction metaid="R_GMHEPK" id="R_GMHEPK" name="D-glycero__D-manno-
heptose 7-phosphate kinase" reversible="false" fast="false"
fbc:lowerFluxBound="irr_lb" fbc:upperFluxBound="irr_ub">
			<listOfReactants>
				<speciesReference species="M_atp_c" stoichiometry="1"
constant="true"/>
				<speciesReference species="M_gmhep7p_c" stoichiometry="1"
constant="true"/>
			</listOfReactants>
			<listOfProducts>
				<speciesReference species="M_h_c" stoichiometry="1"
constant="true"/>
				<speciesReference species="M_adp_c" stoichiometry="1"
constant="true"/>
				<speciesReference species="M_gmhep17bp_c" stoichiometry="1"
constant="true"/>
			</listOfProducts>
			<fbc:geneProductAssociation>
				<fbc:geneProductRef fbc:geneProduct="SAUSA300_0628"/>
			</fbc:geneProductAssociation>
		</reaction>

		<reaction metaid="R_GMHEPPA" id="R_GMHEPPA" name="D-glycero__D-manno-
heptose 1,7-bisphosphate phosphatase" reversible="false" fast="false"
fbc:lowerFluxBound="irr_lb" fbc:upperFluxBound="irr_ub">
			<listOfReactants>
				<speciesReference species="M_h2o_c" stoichiometry="1"
constant="true"/>
				<speciesReference species="M_gmhep17bp_c" stoichiometry="1"
constant="true"/>
			</listOfReactants>
			<listOfProducts>
				<speciesReference species="M_pi_c" stoichiometry="1"
constant="true"/>
				<speciesReference species="M_gmhep1p_c" stoichiometry="1"
constant="true"/>
			</listOfProducts>
			<fbc:geneProductAssociation>
				<fbc:geneProductRef fbc:geneProduct="SAUSA300_0540"/>
			</fbc:geneProductAssociation>
		</reaction>

		<reaction metaid="R_GMPR" id="R_GMPR" name="GMP reductase"
reversible="false" fast="false" fbc:lowerFluxBound="irr_lb"
fbc:upperFluxBound="irr_ub">
			<listOfReactants>
```

```
                  <speciesReference species="M_h_c" stoichiometry="2"
constant="true"/>
                  <speciesReference species="M_nadph_c" stoichiometry="1"
constant="true"/>
                  <speciesReference species="M_gmp_c" stoichiometry="1"
constant="true"/>
            </listOfReactants>
            <listOfProducts>
                  <speciesReference species="M_nh4_c" stoichiometry="1"
constant="true"/>
                  <speciesReference species="M_nadp_c" stoichiometry="1"
constant="true"/>
                  <speciesReference species="M_imp_c" stoichiometry="1"
constant="true"/>
            </listOfProducts>
            <fbc:geneProductAssociation>
                  <fbc:geneProductRef fbc:geneProduct="SAUSA300_1235"/>
            </fbc:geneProductAssociation>
      </reaction>

      <reaction metaid="R_GMPS2" id="R_GMPS2" name="GMP synthase"
reversible="false" fast="false" fbc:lowerFluxBound="irr_lb"
fbc:upperFluxBound="irr_ub">
            <listOfReactants>
                  <speciesReference species="M_atp_c" stoichiometry="1"
constant="true"/>
                  <speciesReference species="M_gln__L_c" stoichiometry="1"
constant="true"/>
                  <speciesReference species="M_h2o_c" stoichiometry="1"
constant="true"/>
                  <speciesReference species="M_xmp_c" stoichiometry="1"
constant="true"/>
            </listOfReactants>
            <listOfProducts>
                  <speciesReference species="M_amp_c" stoichiometry="1"
constant="true"/>
                  <speciesReference species="M_glu__L_c" stoichiometry="1"
constant="true"/>
                  <speciesReference species="M_gmp_c" stoichiometry="1"
constant="true"/>
                  <speciesReference species="M_h_c" stoichiometry="2.0"
constant="true"/>
                  <speciesReference species="M_ppi_c" stoichiometry="1"
constant="true"/>
            </listOfProducts>
            <fbc:geneProductAssociation>
                  <fbc:geneProductRef fbc:geneProduct="SAUSA300_0389"/>
            </fbc:geneProductAssociation>
      </reaction>

      <reaction metaid="R_GND" id="R_GND" name="phosphogluconate
dehydrogenase" reversible="false" fast="false" fbc:lowerFluxBound="irr_lb"
fbc:upperFluxBound="irr_ub">
            <listOfReactants>
                  <speciesReference species="M_nadp_c" stoichiometry="1"
constant="true"/>
                  <speciesReference species="M_6pgc_c" stoichiometry="1"
constant="true"/>
```

```
            </listOfReactants>
            <listOfProducts>
                  <speciesReference species="M_nadph_c" stoichiometry="1"
constant="true"/>
                  <speciesReference species="M_co2_c" stoichiometry="1"
constant="true"/>
                  <speciesReference species="M_ru5p__D_c" stoichiometry="1"
constant="true"/>
            </listOfProducts>
            <fbc:geneProductAssociation>
                  <fbc:geneProductRef fbc:geneProduct="SAUSA300_1459"/>
            </fbc:geneProductAssociation>
      </reaction>

      <reaction metaid="R_GNK" id="R_GNK" name="gluconokinase"
reversible="false" fast="false" fbc:lowerFluxBound="irr_lb"
fbc:upperFluxBound="irr_ub">
            <listOfReactants>
                  <speciesReference species="M_atp_c" stoichiometry="1"
constant="true"/>
                  <speciesReference species="M_glcn_c" stoichiometry="1"
constant="true"/>
            </listOfReactants>
            <listOfProducts>
                  <speciesReference species="M_adp_c" stoichiometry="1"
constant="true"/>
                  <speciesReference species="M_6pgc_c" stoichiometry="1"
constant="true"/>
            </listOfProducts>
            <fbc:geneProductAssociation>
                  <fbc:geneProductRef fbc:geneProduct="SAUSA300_2443"/>
            </fbc:geneProductAssociation>
      </reaction>

      <reaction metaid="R_GNNUC" id="R_GNNUC" name="gnnuc" reversible="false"
fast="false" fbc:lowerFluxBound="irr_lb" fbc:upperFluxBound="irr_ub">
            <listOfReactants>
                  <speciesReference species="M_h2o_c" stoichiometry="1"
constant="true"/>
                  <speciesReference species="M_gsn_c" stoichiometry="1"
constant="true"/>
            </listOfReactants>
            <listOfProducts>
                  <speciesReference species="M_gua_c" stoichiometry="1"
constant="true"/>
                  <speciesReference species="M_rib__D_c" stoichiometry="1"
constant="true"/>
            </listOfProducts>
            <fbc:geneProductAssociation>
                  <fbc:or>
                        <fbc:geneProductRef fbc:geneProduct="SAUSA300_2234"/>
                        <fbc:geneProductRef fbc:geneProduct="SAUSA300_0237"/>
                  </fbc:or>
            </fbc:geneProductAssociation>
      </reaction>
```

```
	<reaction metaid="R_GPDDA1" id="R_GPDDA1" name="Glycerophosphodiester
phosphodiesterase (Glycerophosphocholine)" reversible="false" fast="false"
fbc:lowerFluxBound="irr_lb" fbc:upperFluxBound="irr_ub">
		<listOfReactants>
			<speciesReference species="M_h2o_c" stoichiometry="1"
constant="true"/>
			<speciesReference species="M_g3pc_c" stoichiometry="1"
constant="true"/>
		</listOfReactants>
		<listOfProducts>
			<speciesReference species="M_h_c" stoichiometry="1"
constant="true"/>
			<speciesReference species="M_glyc3p_c" stoichiometry="1"
constant="true"/>
			<speciesReference species="M_chol_c" stoichiometry="1"
constant="true"/>
		</listOfProducts>
		<fbc:geneProductAssociation>
			<fbc:or>
				<fbc:geneProductRef fbc:geneProduct="SAUSA300_0030"/>
				<fbc:geneProductRef fbc:geneProduct="SAUSA300_0862"/>
				<fbc:geneProductRef fbc:geneProduct="SAUSA300_1667"/>
				<fbc:geneProductRef fbc:geneProduct="SAUSA300_1020"/>
				<fbc:geneProductRef fbc:geneProduct="SAUSA300_0222"/>
			</fbc:or>
		</fbc:geneProductAssociation>
	</reaction>

	<reaction metaid="R_GPDDA2" id="R_GPDDA2" name="Glycerophosphodiester
phosphodiesterase (Glycerophosphoethanolamine)" reversible="false"
fast="false" fbc:lowerFluxBound="irr_lb" fbc:upperFluxBound="irr_ub">
		<listOfReactants>
			<speciesReference species="M_h2o_c" stoichiometry="1"
constant="true"/>
			<speciesReference species="M_g3pe_c" stoichiometry="1"
constant="true"/>
		</listOfReactants>
		<listOfProducts>
			<speciesReference species="M_h_c" stoichiometry="1"
constant="true"/>
			<speciesReference species="M_glyc3p_c" stoichiometry="1"
constant="true"/>
			<speciesReference species="M_etha_c" stoichiometry="1"
constant="true"/>
		</listOfProducts>
		<fbc:geneProductAssociation>
			<fbc:or>
				<fbc:geneProductRef fbc:geneProduct="SAUSA300_0030"/>
				<fbc:geneProductRef fbc:geneProduct="SAUSA300_0862"/>
				<fbc:geneProductRef fbc:geneProduct="SAUSA300_1667"/>
				<fbc:geneProductRef fbc:geneProduct="SAUSA300_1020"/>
				<fbc:geneProductRef fbc:geneProduct="SAUSA300_0222"/>
			</fbc:or>
		</fbc:geneProductAssociation>
	</reaction>
```

```
      <reaction metaid="R_GPDDA3" id="R_GPDDA3" name="Glycerophosphodiester
phosphodiesterase (Glycerophosphoserine)" reversible="false" fast="false"
fbc:lowerFluxBound="irr_lb" fbc:upperFluxBound="irr_ub">
            <listOfReactants>
                  <speciesReference species="M_h2o_c" stoichiometry="1"
constant="true"/>
                  <speciesReference species="M_g3ps_c" stoichiometry="1"
constant="true"/>
            </listOfReactants>
            <listOfProducts>
                  <speciesReference species="M_h_c" stoichiometry="1"
constant="true"/>
                  <speciesReference species="M_glyc3p_c" stoichiometry="1"
constant="true"/>
                  <speciesReference species="M_ser__L_c" stoichiometry="1"
constant="true"/>
            </listOfProducts>
            <fbc:geneProductAssociation>
                  <fbc:or>
                        <fbc:geneProductRef fbc:geneProduct="SAUSA300_0030"/>
                        <fbc:geneProductRef fbc:geneProduct="SAUSA300_0862"/>
                        <fbc:geneProductRef fbc:geneProduct="SAUSA300_1667"/>
                        <fbc:geneProductRef fbc:geneProduct="SAUSA300_1020"/>
                        <fbc:geneProductRef fbc:geneProduct="SAUSA300_0222"/>
                  </fbc:or>
            </fbc:geneProductAssociation>
      </reaction>

      <reaction metaid="R_GPDDA4" id="R_GPDDA4" name="Glycerophosphodiester
phosphodiesterase (Glycerophosphoglycerol)" reversible="false" fast="false"
fbc:lowerFluxBound="irr_lb" fbc:upperFluxBound="irr_ub">
            <listOfReactants>
                  <speciesReference species="M_h2o_c" stoichiometry="1"
constant="true"/>
                  <speciesReference species="M_g3pg_c" stoichiometry="1"
constant="true"/>
            </listOfReactants>
            <listOfProducts>
                  <speciesReference species="M_h_c" stoichiometry="1"
constant="true"/>
                  <speciesReference species="M_glyc_c" stoichiometry="1"
constant="true"/>
                  <speciesReference species="M_glyc3p_c" stoichiometry="1"
constant="true"/>
            </listOfProducts>
            <fbc:geneProductAssociation>
                  <fbc:or>
                        <fbc:geneProductRef fbc:geneProduct="SAUSA300_0030"/>
                        <fbc:geneProductRef fbc:geneProduct="SAUSA300_0862"/>
                        <fbc:geneProductRef fbc:geneProduct="SAUSA300_1667"/>
                        <fbc:geneProductRef fbc:geneProduct="SAUSA300_1020"/>
                        <fbc:geneProductRef fbc:geneProduct="SAUSA300_0222"/>
                  </fbc:or>
            </fbc:geneProductAssociation>
      </reaction>

      <reaction metaid="R_GPDDA4pp" id="R_GPDDA4pp"
name="Glycerophosphodiester phosphodiesterase (Glycerophosphoglycerol)"
```

```
reversible="false" fast="false" fbc:lowerFluxBound="irr_lb"
fbc:upperFluxBound="irr_ub">
            <listOfReactants>
                  <speciesReference species="M_h2o_c" stoichiometry="1"
constant="true"/>
                  <speciesReference species="M_g3pg_c" stoichiometry="1"
constant="true"/>
            </listOfReactants>
            <listOfProducts>
                  <speciesReference species="M_glyc_c" stoichiometry="1"
constant="true"/>
                  <speciesReference species="M_glyc3p_c" stoichiometry="1"
constant="true"/>
            </listOfProducts>
            <fbc:geneProductAssociation>
                  <fbc:or>
                        <fbc:geneProductRef fbc:geneProduct="SAUSA300_0862"/>
                        <fbc:geneProductRef fbc:geneProduct="SAUSA300_0222"/>
                        <fbc:geneProductRef fbc:geneProduct="SAUSA300_1020"/>
                        <fbc:geneProductRef fbc:geneProduct="SAUSA300_0030"/>
                        <fbc:geneProductRef fbc:geneProduct="SAUSA300_1667"/>
                  </fbc:or>
            </fbc:geneProductAssociation>
      </reaction>

      <reaction metaid="R_GPDDA5" id="R_GPDDA5" name="Glycerophosphodiester
phosphodiesterase (Glycerophosphoinositol)" reversible="false" fast="false"
fbc:lowerFluxBound="irr_lb" fbc:upperFluxBound="irr_ub">
            <listOfReactants>
                  <speciesReference species="M_h2o_c" stoichiometry="1"
constant="true"/>
                  <speciesReference species="M_g3pi_c" stoichiometry="1"
constant="true"/>
            </listOfReactants>
            <listOfProducts>
                  <speciesReference species="M_h_c" stoichiometry="1"
constant="true"/>
                  <speciesReference species="M_glyc3p_c" stoichiometry="1"
constant="true"/>
                  <speciesReference species="M_inost_c" stoichiometry="1"
constant="true"/>
            </listOfProducts>
            <fbc:geneProductAssociation>
                  <fbc:or>
                        <fbc:geneProductRef fbc:geneProduct="SAUSA300_0030"/>
                        <fbc:geneProductRef fbc:geneProduct="SAUSA300_0862"/>
                        <fbc:geneProductRef fbc:geneProduct="SAUSA300_1667"/>
                        <fbc:geneProductRef fbc:geneProduct="SAUSA300_1020"/>
                        <fbc:geneProductRef fbc:geneProduct="SAUSA300_0222"/>
                  </fbc:or>
            </fbc:geneProductAssociation>
      </reaction>

      <reaction metaid="R_GRTT" id="R_GRTT" name="geranyltranstransferase"
reversible="false" fast="false" fbc:lowerFluxBound="irr_lb"
fbc:upperFluxBound="irr_ub">
            <listOfReactants>
```

```
                  <speciesReference species="M_ipdp_c" stoichiometry="1"
constant="true"/>
                  <speciesReference species="M_grdp_c" stoichiometry="1"
constant="true"/>
            </listOfReactants>
            <listOfProducts>
                  <speciesReference species="M_ppi_c" stoichiometry="1"
constant="true"/>
                  <speciesReference species="M_frdp_c" stoichiometry="1"
constant="true"/>
            </listOfProducts>
            <fbc:geneProductAssociation>
                  <fbc:geneProductRef fbc:geneProduct="SAUSA300_1470"/>
            </fbc:geneProductAssociation>
      </reaction>

      <reaction metaid="R_GSNt2pp" id="R_GSNt2pp" name="guanosine transport
in via proton symport (periplasm)" reversible="false" fast="false"
fbc:lowerFluxBound="irr_lb" fbc:upperFluxBound="irr_ub">
            <listOfReactants>
                  <speciesReference species="M_h_p" stoichiometry="1"
constant="true"/>
                  <speciesReference species="M_gsn_p" stoichiometry="1"
constant="true"/>
            </listOfReactants>
            <listOfProducts>
                  <speciesReference species="M_h_c" stoichiometry="1"
constant="true"/>
                  <speciesReference species="M_gsn_c" stoichiometry="1"
constant="true"/>
            </listOfProducts>
      </reaction>

      <reaction metaid="R_GTHP" id="R_GTHP" name="glutathione peroxidase"
reversible="true" fast="false" fbc:lowerFluxBound="rev_lb"
fbc:upperFluxBound="rev_ub">
            <listOfReactants>
                  <speciesReference species="M_gthrd_c" stoichiometry="2"
constant="true"/>
                  <speciesReference species="M_h2o2_c" stoichiometry="1"
constant="true"/>
            </listOfReactants>
            <listOfProducts>
                  <speciesReference species="M_h2o_c" stoichiometry="2"
constant="true"/>
                  <speciesReference species="M_gthrd2_c" stoichiometry="1"
constant="true"/>
            </listOfProducts>
            <fbc:geneProductAssociation>
                  <fbc:or>
                        <fbc:geneProductRef fbc:geneProduct="SAUSA300_2555"/>
                        <fbc:geneProductRef fbc:geneProduct="SAUSA300_1197"/>
                  </fbc:or>
            </fbc:geneProductAssociation>
      </reaction>
```

```
      <reaction metaid="R_GTHRDHpp" id="R_GTHRDHpp" name="glutathione
hydralase (periplasmic)" reversible="false" fast="false"
fbc:lowerFluxBound="irr_lb" fbc:upperFluxBound="irr_ub">
            &lt;listOfReactants&gt;
                  <speciesReference species="M_h2o_c" stoichiometry="1"
constant="true"/>
                  <speciesReference species="M_gthrd_c" stoichiometry="1"
constant="true"/>
            &lt;/listOfReactants&gt;
            &lt;listOfProducts&gt;
                  <speciesReference species="M_glu__L_c" stoichiometry="1"
constant="true"/>
                  <speciesReference species="M_cgly_c" stoichiometry="1"
constant="true"/>
            &lt;/listOfProducts&gt;
            &lt;fbc:geneProductAssociation&gt;
                  &lt;fbc:geneProductRef fbc:geneProduct="SAUSA300_0204"/&gt;
            &lt;/fbc:geneProductAssociation&gt;
      &lt;/reaction&gt;

      <reaction metaid="R_GTPCI" id="R_GTPCI" name="GTP cyclohydrolase I"
reversible="false" fast="false" fbc:lowerFluxBound="irr_lb"
fbc:upperFluxBound="irr_ub">
            &lt;listOfReactants&gt;
                  <speciesReference species="M_h2o_c" stoichiometry="1"
constant="true"/>
                  <speciesReference species="M_gtp_c" stoichiometry="1"
constant="true"/>
            &lt;/listOfReactants&gt;
            &lt;listOfProducts&gt;
                  <speciesReference species="M_h_c" stoichiometry="1"
constant="true"/>
                  <speciesReference species="M_for_c" stoichiometry="1"
constant="true"/>
                  <speciesReference species="M_ahdt_c" stoichiometry="1"
constant="true"/>
            &lt;/listOfProducts&gt;
            &lt;fbc:geneProductAssociation&gt;
                  &lt;fbc:or&gt;
                        &lt;fbc:geneProductRef fbc:geneProduct="SAUSA300_0551"/&gt;
                        &lt;fbc:geneProductRef fbc:geneProduct="SAUSA300_1519"/&gt;
                  &lt;/fbc:or&gt;
            &lt;/fbc:geneProductAssociation&gt;
      &lt;/reaction&gt;

      <reaction metaid="R_GTPCII" id="R_GTPCII" name="GTP cyclohydrolase II"
reversible="false" fast="false" fbc:lowerFluxBound="irr_lb"
fbc:upperFluxBound="irr_ub">
            &lt;listOfReactants&gt;
                  <speciesReference species="M_h2o_c" stoichiometry="3"
constant="true"/>
                  <speciesReference species="M_gtp_c" stoichiometry="1"
constant="true"/>
            &lt;/listOfReactants&gt;
            &lt;listOfProducts&gt;
                  <speciesReference species="M_h_c" stoichiometry="1"
constant="true"/>
```

```
                  <speciesReference species="M_ppi_c" stoichiometry="1"
constant="true"/>
                  <speciesReference species="M_for_c" stoichiometry="1"
constant="true"/>
                  <speciesReference species="M_25dhpp_c" stoichiometry="1"
constant="true"/>
            </listOfProducts>
            <fbc:geneProductAssociation>
                  <fbc:or>
                        <fbc:geneProductRef fbc:geneProduct="SAUSA300_1713"/>
                        <fbc:geneProductRef fbc:geneProduct="SAUSA300_1519"/>
                  </fbc:or>
            </fbc:geneProductAssociation>
      </reaction>

      <reaction metaid="R_GTPDPK" id="R_GTPDPK" name="GTP diphosphokinase"
reversible="false" fast="false" fbc:lowerFluxBound="irr_lb"
fbc:upperFluxBound="irr_ub">
            <listOfReactants>
                  <speciesReference species="M_atp_c" stoichiometry="1"
constant="true"/>
                  <speciesReference species="M_gtp_c" stoichiometry="1"
constant="true"/>
            </listOfReactants>
            <listOfProducts>
                  <speciesReference species="M_amp_c" stoichiometry="1"
constant="true"/>
                  <speciesReference species="M_gdptp_c" stoichiometry="1"
constant="true"/>
            </listOfProducts>
            <fbc:geneProductAssociation>
                  <fbc:or>
                        <fbc:geneProductRef fbc:geneProduct="SAUSA300_0907"/>
                        <fbc:geneProductRef fbc:geneProduct="SAUSA300_2446"/>
                        <fbc:geneProductRef fbc:geneProduct="SAUSA300_1590"/>
                  </fbc:or>
            </fbc:geneProductAssociation>
      </reaction>

      <reaction metaid="R_GTPHDR" id="R_GTPHDR" name="GTP 7 8-8
9__Dihydrolase " reversible="true" fast="false" fbc:lowerFluxBound="rev_lb"
fbc:upperFluxBound="rev_ub">
            <listOfReactants>
                  <speciesReference species="M_ptp_c" stoichiometry="1"
constant="true"/>
            </listOfReactants>
            <listOfProducts>
                  <speciesReference species="M_dattoo_c" stoichiometry="1"
constant="true"/>
            </listOfProducts>
            <fbc:geneProductAssociation>
                  <fbc:geneProductRef fbc:geneProduct="SAUSA300_0551"/>
            </fbc:geneProductAssociation>
      </reaction>

      <reaction metaid="R_GTPHL" id="R_GTPHL" name="GTP 7 8-8 9__Dihydrolase
" reversible="false" fast="false" fbc:lowerFluxBound="irr_lb"
fbc:upperFluxBound="irr_ub">
```

```
            <listOfReactants>
                  <speciesReference species="M_h2o_c" stoichiometry="1"
constant="true"/>
                  <speciesReference species="M_gtp_c" stoichiometry="1"
constant="true"/>
            </listOfReactants>
            <listOfProducts>
                  <speciesReference species="M_fapnt_c" stoichiometry="1"
constant="true"/>
            </listOfProducts>
            <fbc:geneProductAssociation>
                  <fbc:geneProductRef fbc:geneProduct="SAUSA300_0551"/>
            </fbc:geneProductAssociation>
      </reaction>

      <reaction metaid="R_GUAPRT" id="R_GUAPRT" name="guanine
phosphoribosyltransferase" reversible="false" fast="false"
fbc:lowerFluxBound="irr_lb" fbc:upperFluxBound="irr_ub">
            <listOfReactants>
                  <speciesReference species="M_gua_c" stoichiometry="1"
constant="true"/>
                  <speciesReference species="M_prpp_c" stoichiometry="1"
constant="true"/>
            </listOfReactants>
            <listOfProducts>
                  <speciesReference species="M_ppi_c" stoichiometry="1"
constant="true"/>
                  <speciesReference species="M_gmp_c" stoichiometry="1"
constant="true"/>
            </listOfProducts>
            <fbc:geneProductAssociation>
                  <fbc:or>
                        <fbc:geneProductRef fbc:geneProduct="SAUSA300_0488"/>
                        <fbc:geneProductRef fbc:geneProduct="SAUSA300_1591"/>
                  </fbc:or>
            </fbc:geneProductAssociation>
      </reaction>

      <reaction metaid="R_GUAt2" id="R_GUAt2" name="guanine transport in via
proton symport" reversible="false" fast="false" fbc:lowerFluxBound="irr_lb"
fbc:upperFluxBound="irr_ub">
            <listOfReactants>
                  <speciesReference species="M_h_e" stoichiometry="1"
constant="true"/>
                  <speciesReference species="M_gua_e" stoichiometry="1"
constant="true"/>
            </listOfReactants>
            <listOfProducts>
                  <speciesReference species="M_h_c" stoichiometry="1"
constant="true"/>
                  <speciesReference species="M_gua_c" stoichiometry="1"
constant="true"/>
            </listOfProducts>
      </reaction>

      <reaction metaid="R_GUI1" id="R_GUI1" name="glucuronate isomerase (D-
glucuronate)" reversible="true" fast="false" fbc:lowerFluxBound="rev_lb"
fbc:upperFluxBound="rev_ub">
```

```
            <listOfReactants>
                  <speciesReference species="M_glcur_c" stoichiometry="1"
constant="true"/>
            </listOfReactants>
            <listOfProducts>
                  <speciesReference species="M_fruur_c" stoichiometry="1"
constant="true"/>
            </listOfProducts>
      </reaction>

      <reaction metaid="R_H2Otex" id="R_H2Otex" name="H2O transport via
diffusion (extracellular to periplasm)" reversible="true" fast="false"
fbc:lowerFluxBound="rev_lb" fbc:upperFluxBound="rev_ub">
            <listOfReactants>
                  <speciesReference species="M_h2o_e" stoichiometry="1"
constant="true"/>
            </listOfReactants>
            <listOfProducts>
                  <speciesReference species="M_h2o_p" stoichiometry="1"
constant="true"/>
            </listOfProducts>
      </reaction>

      <reaction metaid="R_HACD1" id="R_HACD1" name="3-hydroxyacyl-CoA
dehydrogenase (acetoacetyl-CoA)" reversible="true" fast="false"
fbc:lowerFluxBound="rev_lb" fbc:upperFluxBound="rev_ub">
            <listOfReactants>
                  <speciesReference species="M_3hbcoa_c" stoichiometry="1"
constant="true"/>
                  <speciesReference species="M_nad_c" stoichiometry="1"
constant="true"/>
            </listOfReactants>
            <listOfProducts>
                  <speciesReference species="M_h_c" stoichiometry="1"
constant="true"/>
                  <speciesReference species="M_nadh_c" stoichiometry="1"
constant="true"/>
                  <speciesReference species="M_aacoa_c" stoichiometry="1"
constant="true"/>
            </listOfProducts>
            <fbc:geneProductAssociation>
                  <fbc:or>
                        <fbc:geneProductRef fbc:geneProduct="SAUSA300_2294"/>
                        <fbc:geneProductRef fbc:geneProduct="SAUSA300_0226"/>
                  </fbc:or>
            </fbc:geneProductAssociation>
      </reaction>

      <reaction metaid="R_HACD2" id="R_HACD2" name="3-hydroxyacyl-CoA
dehydrogenase (3-oxohexanoyl-CoA)" reversible="true" fast="false"
fbc:lowerFluxBound="rev_lb" fbc:upperFluxBound="rev_ub">
            <listOfReactants>
                  <speciesReference species="M_nad_c" stoichiometry="1"
constant="true"/>
                  <speciesReference species="M_3hhcoa_c" stoichiometry="1"
constant="true"/>
            </listOfReactants>
            <listOfProducts>
```

```
                  <speciesReference species="M_h_c" stoichiometry="1"
constant="true"/>
                  <speciesReference species="M_nadh_c" stoichiometry="1"
constant="true"/>
                  <speciesReference species="M_3ohcoa_c" stoichiometry="1"
constant="true"/>
            </listOfProducts>
            <fbc:geneProductAssociation>
                  <fbc:or>
                        <fbc:geneProductRef fbc:geneProduct="SAUSA300_2294"/>
                        <fbc:geneProductRef fbc:geneProduct="SAUSA300_0226"/>
                  </fbc:or>
            </fbc:geneProductAssociation>
      </reaction>

      <reaction metaid="R_HACD3" id="R_HACD3" name="3-hydroxyacyl-CoA
dehydrogenase (3-oxooctanoyl-CoA)" reversible="true" fast="false"
fbc:lowerFluxBound="rev_lb" fbc:upperFluxBound="rev_ub">
            <listOfReactants>
                  <speciesReference species="M_nad_c" stoichiometry="1"
constant="true"/>
                  <speciesReference species="M_3hocoa_c" stoichiometry="1"
constant="true"/>
            </listOfReactants>
            <listOfProducts>
                  <speciesReference species="M_h_c" stoichiometry="1"
constant="true"/>
                  <speciesReference species="M_nadh_c" stoichiometry="1"
constant="true"/>
                  <speciesReference species="M_3oocoa_c" stoichiometry="1"
constant="true"/>
            </listOfProducts>
            <fbc:geneProductAssociation>
                  <fbc:or>
                        <fbc:geneProductRef fbc:geneProduct="SAUSA300_2294"/>
                        <fbc:geneProductRef fbc:geneProduct="SAUSA300_0226"/>
                  </fbc:or>
            </fbc:geneProductAssociation>
      </reaction>

      <reaction metaid="R_HACD4" id="R_HACD4" name="3-hydroxyacyl-CoA
dehydrogenase (3-oxodecanoyl-CoA)" reversible="true" fast="false"
fbc:lowerFluxBound="rev_lb" fbc:upperFluxBound="rev_ub">
            <listOfReactants>
                  <speciesReference species="M_nad_c" stoichiometry="1"
constant="true"/>
                  <speciesReference species="M_3hdcoa_c" stoichiometry="1"
constant="true"/>
            </listOfReactants>
            <listOfProducts>
                  <speciesReference species="M_h_c" stoichiometry="1"
constant="true"/>
                  <speciesReference species="M_nadh_c" stoichiometry="1"
constant="true"/>
                  <speciesReference species="M_3odcoa_c" stoichiometry="1"
constant="true"/>
            </listOfProducts>
            <fbc:geneProductAssociation>
```

```
                <fbc:or>
                    <fbc:geneProductRef fbc:geneProduct="SAUSA300_2294"/>
                    <fbc:geneProductRef fbc:geneProduct="SAUSA300_0226"/>
                </fbc:or>
            </fbc:geneProductAssociation>
        </reaction>

        <reaction metaid="R_HACD5" id="R_HACD5" name="3-hydroxyacyl-CoA
dehydrogenase (3-oxododecanoyl-CoA)" reversible="true" fast="false"
fbc:lowerFluxBound="rev_lb" fbc:upperFluxBound="rev_ub">
            <listOfReactants>
                <speciesReference species="M_nad_c" stoichiometry="1"
constant="true"/>
                <speciesReference species="M_3hddcoa_c" stoichiometry="1"
constant="true"/>
            </listOfReactants>
            <listOfProducts>
                <speciesReference species="M_h_c" stoichiometry="1"
constant="true"/>
                <speciesReference species="M_nadh_c" stoichiometry="1"
constant="true"/>
                <speciesReference species="M_3oddcoa_c" stoichiometry="1"
constant="true"/>
            </listOfProducts>
            <fbc:geneProductAssociation>
                <fbc:or>
                    <fbc:geneProductRef fbc:geneProduct="SAUSA300_2294"/>
                    <fbc:geneProductRef fbc:geneProduct="SAUSA300_0226"/>
                </fbc:or>
            </fbc:geneProductAssociation>
        </reaction>

        <reaction metaid="R_HACD6" id="R_HACD6" name="3-hydroxyacyl-CoA
dehydrogenase (3-oxotetradecanoyl-CoA)" reversible="true" fast="false"
fbc:lowerFluxBound="rev_lb" fbc:upperFluxBound="rev_ub">
            <listOfReactants>
                <speciesReference species="M_nad_c" stoichiometry="1"
constant="true"/>
                <speciesReference species="M_3htdcoa_c" stoichiometry="1"
constant="true"/>
            </listOfReactants>
            <listOfProducts>
                <speciesReference species="M_h_c" stoichiometry="1"
constant="true"/>
                <speciesReference species="M_nadh_c" stoichiometry="1"
constant="true"/>
                <speciesReference species="M_3otdcoa_c" stoichiometry="1"
constant="true"/>
            </listOfProducts>
            <fbc:geneProductAssociation>
                <fbc:or>
                    <fbc:geneProductRef fbc:geneProduct="SAUSA300_2294"/>
                    <fbc:geneProductRef fbc:geneProduct="SAUSA300_0226"/>
                </fbc:or>
            </fbc:geneProductAssociation>
        </reaction>
```

```
      <reaction metaid="R_HACD7" id="R_HACD7" name="3-hydroxyacyl-CoA
dehydrogenase (3-oxohexadecanoyl-CoA)" reversible="true" fast="false"
fbc:lowerFluxBound="rev_lb" fbc:upperFluxBound="rev_ub">
            <listOfReactants>
                  <speciesReference species="M_h_c" stoichiometry="1"
constant="true"/>
                  <speciesReference species="M_nadh_c" stoichiometry="1"
constant="true"/>
                  <speciesReference species="M_3ohdcoa_c" stoichiometry="1"
constant="true"/>
            </listOfReactants>
            <listOfProducts>
                  <speciesReference species="M_nad_c" stoichiometry="1"
constant="true"/>
                  <speciesReference species="M_3hhdcoa_c" stoichiometry="1"
constant="true"/>
            </listOfProducts>
            <fbc:geneProductAssociation>
                  <fbc:geneProductRef fbc:geneProduct="SAUSA300_0226"/>
            </fbc:geneProductAssociation>
      </reaction>

      <reaction metaid="R_HACD9" id="R_HACD9" name="3-hydroxyacyl-CoA
dehydrogenase (2-Methylacetoacetyl-CoA)" reversible="true" fast="false"
fbc:lowerFluxBound="rev_lb" fbc:upperFluxBound="rev_ub">
            <listOfReactants>
                  <speciesReference species="M_nad_c" stoichiometry="1"
constant="true"/>
                  <speciesReference species="M_3hmbcoa_c" stoichiometry="1"
constant="true"/>
            </listOfReactants>
            <listOfProducts>
                  <speciesReference species="M_h_c" stoichiometry="1"
constant="true"/>
                  <speciesReference species="M_nadh_c" stoichiometry="1"
constant="true"/>
                  <speciesReference species="M_2maacoa_c" stoichiometry="1"
constant="true"/>
            </listOfProducts>
            <fbc:geneProductAssociation>
                  <fbc:or>
                        <fbc:geneProductRef fbc:geneProduct="SAUSA300_2294"/>
                        <fbc:geneProductRef fbc:geneProduct="SAUSA300_0226"/>
                  </fbc:or>
            </fbc:geneProductAssociation>
      </reaction>

      <reaction metaid="R_HADPCOADH3" id="R_HADPCOADH3" name="3-
hydroxyadipyl-CoA dehydrogenase (NAD+)" reversible="true" fast="false"
fbc:lowerFluxBound="rev_lb" fbc:upperFluxBound="rev_ub">
            <listOfReactants>
                  <speciesReference species="M_nad_c" stoichiometry="1"
constant="true"/>
                  <speciesReference species="M_3hadpcoa_c" stoichiometry="1"
constant="true"/>
            </listOfReactants>
            <listOfProducts>
```

```
                  <speciesReference species="M_h_c" stoichiometry="1"
constant="true"/>
                  <speciesReference species="M_nadh_c" stoichiometry="1"
constant="true"/>
                  <speciesReference species="M_oxadpcoa_c" stoichiometry="1"
constant="true"/>
            </listOfProducts>
            <fbc:geneProductAssociation>
                  <fbc:or>
                        <fbc:geneProductRef fbc:geneProduct="SAUSA300_2294"/>
                        <fbc:geneProductRef fbc:geneProduct="SAUSA300_0226"/>
                  </fbc:or>
            </fbc:geneProductAssociation>
      </reaction>

      <reaction metaid="R_HBZOPT" id="R_HBZOPT" name="Hydroxybenzoate
octaprenyltransferase" reversible="false" fast="false"
fbc:lowerFluxBound="irr_lb" fbc:upperFluxBound="irr_ub">
            <listOfReactants>
                  <speciesReference species="M_octdp_c" stoichiometry="1"
constant="true"/>
                  <speciesReference species="M_4hbz_c" stoichiometry="1"
constant="true"/>
            </listOfReactants>
            <listOfProducts>
                  <speciesReference species="M_ppi_c" stoichiometry="1"
constant="true"/>
                  <speciesReference species="M_3ophb_c" stoichiometry="1"
constant="true"/>
            </listOfProducts>
            <fbc:geneProductAssociation>
                  <fbc:geneProductRef fbc:geneProduct="UbiA"/>
            </fbc:geneProductAssociation>
      </reaction>

      <reaction metaid="R_HCO3E" id="R_HCO3E" name="HCO3 equilibration
reaction" reversible="true" fast="false" fbc:lowerFluxBound="rev_lb"
fbc:upperFluxBound="rev_ub">
            <listOfReactants>
                  <speciesReference species="M_h2o_c" stoichiometry="1"
constant="true"/>
                  <speciesReference species="M_co2_c" stoichiometry="1"
constant="true"/>
            </listOfReactants>
            <listOfProducts>
                  <speciesReference species="M_h_c" stoichiometry="1"
constant="true"/>
                  <speciesReference species="M_hco3_c" stoichiometry="1"
constant="true"/>
            </listOfProducts>
            <fbc:geneProductAssociation>
                  <fbc:geneProductRef fbc:geneProduct="SAUSA300_2438"/>
            </fbc:geneProductAssociation>
      </reaction>

      <reaction metaid="R_HCYSMT" id="R_HCYSMT" name="homocysteine S-
methyltransferase" reversible="false" fast="false"
fbc:lowerFluxBound="irr_lb" fbc:upperFluxBound="irr_ub">
```

```
            <listOfReactants>
                  <speciesReference species="M_hcys__L_c" stoichiometry="1"
constant="true"/>
                  <speciesReference species="M_mhpglu_c" stoichiometry="1"
constant="true"/>
            </listOfReactants>
            <listOfProducts>
                  <speciesReference species="M_met__L_c" stoichiometry="1"
constant="true"/>
                  <speciesReference species="M_hpglu_c" stoichiometry="1"
constant="true"/>
            </listOfProducts>
            <fbc:geneProductAssociation>
                  <fbc:geneProductRef fbc:geneProduct="SAUSA300_0357"/>
            </fbc:geneProductAssociation>
      </reaction>

      <reaction metaid="R_HEDMC" id="R_HEDMC" name="Hexadecanoyl-acyl-carrier
protein malonyl-CoA " reversible="false" fast="false"
fbc:lowerFluxBound="irr_lb" fbc:upperFluxBound="irr_ub">
            <listOfReactants>
                  <speciesReference species="M_h_c" stoichiometry="1"
constant="true"/>
                  <speciesReference species="M_nadh_c" stoichiometry="1"
constant="true"/>
                  <speciesReference species="M_tpalm2eACP_c"
stoichiometry="1" constant="true"/>
            </listOfReactants>
            <listOfProducts>
                  <speciesReference species="M_nad_c" stoichiometry="1"
constant="true"/>
                  <speciesReference species="M_hedacp_c" stoichiometry="1"
constant="true"/>
            </listOfProducts>
            <fbc:geneProductAssociation>
                  <fbc:geneProductRef fbc:geneProduct="SAUSA300_0912"/>
            </fbc:geneProductAssociation>
      </reaction>

      <reaction metaid="R_HEMEAS" id="R_HEMEAS" name="Heme A synthase"
reversible="false" fast="false" fbc:lowerFluxBound="irr_lb"
fbc:upperFluxBound="irr_ub">
            <listOfReactants>
                  <speciesReference species="M_h2o_c" stoichiometry="1"
constant="true"/>
                  <speciesReference species="M_hemeO_c" stoichiometry="1"
constant="true"/>
            </listOfReactants>
            <listOfProducts>
                  <speciesReference species="M_h_c" stoichiometry="4"
constant="true"/>
                  <speciesReference species="M_hemeA__1_c" stoichiometry="1"
constant="true"/>
            </listOfProducts>
            <fbc:geneProductAssociation>
                  <fbc:geneProductRef fbc:geneProduct="SAUSA300_1015"/>
            </fbc:geneProductAssociation>
      </reaction>
```

```
      <reaction metaid="R_HEMEOS" id="R_HEMEOS" name="Heme O synthase"
reversible="false" fast="false" fbc:lowerFluxBound="irr_lb"
fbc:upperFluxBound="irr_ub">
            <listOfReactants>
                  <speciesReference species="M_h2o_c" stoichiometry="1"
constant="true"/>
                  <speciesReference species="M_frdp_c" stoichiometry="1"
constant="true"/>
                  <speciesReference species="M_pheme_c" stoichiometry="1"
constant="true"/>
            </listOfReactants>
            <listOfProducts>
                  <speciesReference species="M_ppi_c" stoichiometry="1"
constant="true"/>
                  <speciesReference species="M_hemeO_c" stoichiometry="1"
constant="true"/>
            </listOfProducts>
            <fbc:geneProductAssociation>
                  <fbc:geneProductRef fbc:geneProduct="SAUSA300_1016"/>
            </fbc:geneProductAssociation>
      </reaction>

      <reaction metaid="R_HEMEti" id="R_HEMEti" name="Heme transport via ABC
system" reversible="false" fast="false" fbc:lowerFluxBound="irr_lb"
fbc:upperFluxBound="irr_ub">
            <listOfReactants>
                  <speciesReference species="M_h2o_c" stoichiometry="1"
constant="true"/>
                  <speciesReference species="M_atp_c" stoichiometry="1"
constant="true"/>
                  <speciesReference species="M_pheme_e" stoichiometry="1"
constant="true"/>
            </listOfReactants>
            <listOfProducts>
                  <speciesReference species="M_h_c" stoichiometry="1"
constant="true"/>
                  <speciesReference species="M_pi_c" stoichiometry="1"
constant="true"/>
                  <speciesReference species="M_adp_c" stoichiometry="1"
constant="true"/>
                  <speciesReference species="M_pheme_c" stoichiometry="1"
constant="true"/>
            </listOfProducts>
            <fbc:geneProductAssociation>
                  <fbc:and>
                        <fbc:geneProductRef fbc:geneProduct="SAUSA300_2134"/>
                        <fbc:geneProductRef fbc:geneProduct="SAUSA300_2135"/>
                        <fbc:or>
                              <fbc:geneProductRef
fbc:geneProduct="SAUSA300_0941"/>
                              <fbc:geneProductRef
fbc:geneProduct="SAUSA300_2136"/>
                        </fbc:or>
                  </fbc:and>
            </fbc:geneProductAssociation>
      </reaction>
```

```
      <reaction metaid="R_HETZK" id="R_HETZK" name="hydroxyethylthiazole
kinase" reversible="false" fast="false" fbc:lowerFluxBound="irr_lb"
fbc:upperFluxBound="irr_ub">
            <listOfReactants>
                  <speciesReference species="M_atp_c" stoichiometry="1"
constant="true"/>
                  <speciesReference species="M_4mhetz_c" stoichiometry="1"
constant="true"/>
            </listOfReactants>
            <listOfProducts>
                  <speciesReference species="M_adp_c" stoichiometry="1"
constant="true"/>
                  <speciesReference species="M_4mpetz_c" stoichiometry="1"
constant="true"/>
            </listOfProducts>
            <fbc:geneProductAssociation>
                  <fbc:geneProductRef fbc:geneProduct="SAUSA300_2048"/>
            </fbc:geneProductAssociation>
      </reaction>

      <reaction metaid="R_HEX1" id="R_HEX1" name="hexokinase (D-glucose:ATP)"
reversible="false" fast="false" fbc:lowerFluxBound="irr_lb"
fbc:upperFluxBound="irr_ub">
            <listOfReactants>
                  <speciesReference species="M_glc__D_c" stoichiometry="1"
constant="true"/>
                  <speciesReference species="M_atp_c" stoichiometry="1"
constant="true"/>
            </listOfReactants>
            <listOfProducts>
                  <speciesReference species="M_g6p_c" stoichiometry="1"
constant="true"/>
                  <speciesReference species="M_adp_c" stoichiometry="1"
constant="true"/>
            </listOfProducts>
            <fbc:geneProductAssociation>
                  <fbc:geneProductRef fbc:geneProduct="SAUSA300_1507"/>
            </fbc:geneProductAssociation>
      </reaction>

      <reaction metaid="R_HEX7" id="R_HEX7" name="hexokinase (D-
fructose:ATP)" reversible="false" fast="false" fbc:lowerFluxBound="irr_lb"
fbc:upperFluxBound="irr_ub">
            <listOfReactants>
                  <speciesReference species="M_atp_c" stoichiometry="1"
constant="true"/>
                  <speciesReference species="M_fru_c" stoichiometry="1"
constant="true"/>
            </listOfReactants>
            <listOfProducts>
                  <speciesReference species="M_adp_c" stoichiometry="1"
constant="true"/>
                  <speciesReference species="M_f6p_c" stoichiometry="1"
constant="true"/>
            </listOfProducts>
            <fbc:geneProductAssociation>
                  <fbc:geneProductRef fbc:geneProduct="SAUSA300_1993"/>
            </fbc:geneProductAssociation>
```

```
      </reaction>

      <reaction metaid="R_HEXAT" id="R_HEXAT" name="hexadecanoyl-ACP acyl-
carrier-protein transferase " reversible="true" fast="false"
fbc:lowerFluxBound="rev_lb" fbc:upperFluxBound="rev_ub">
            <listOfReactants>
                  <speciesReference species="M_coa_c" stoichiometry="1"
constant="true"/>
                  <speciesReference species="M_hedacp_c" stoichiometry="1"
constant="true"/>
            </listOfReactants>
            <listOfProducts>
                  <speciesReference species="M_ACP_c" stoichiometry="1"
constant="true"/>
                  <speciesReference species="M_pmtcoa_c" stoichiometry="1"
constant="true"/>
            </listOfProducts>
            <fbc:geneProductAssociation>
                  <fbc:geneProductRef fbc:geneProduct="SAUSA300_1123"/>
            </fbc:geneProductAssociation>
      </reaction>

      <reaction metaid="R_HEXTT" id="R_HEXTT" name="trans-
hexaprenyltranstransferase" reversible="false" fast="false"
fbc:lowerFluxBound="irr_lb" fbc:upperFluxBound="irr_ub">
            <listOfReactants>
                  <speciesReference species="M_ipdp_c" stoichiometry="1"
constant="true"/>
                  <speciesReference species="M_hexdp_c" stoichiometry="1"
constant="true"/>
            </listOfReactants>
            <listOfProducts>
                  <speciesReference species="M_ppi_c" stoichiometry="1"
constant="true"/>
                  <speciesReference species="M_hepdp_c" stoichiometry="1"
constant="true"/>
            </listOfProducts>
            <fbc:geneProductAssociation>
                  <fbc:and>
                        <fbc:geneProductRef fbc:geneProduct="SAUSA300_1359"/>
                        <fbc:geneProductRef fbc:geneProduct="SAUSA300_1361"/>
                  </fbc:and>
            </fbc:geneProductAssociation>
      </reaction>

      <reaction metaid="R_HIBD" id="R_HIBD" name="3-hydroxyisobutyrate
dehydrogenase" reversible="true" fast="false" fbc:lowerFluxBound="rev_lb"
fbc:upperFluxBound="rev_ub">
            <listOfReactants>
                  <speciesReference species="M_nad_c" stoichiometry="1"
constant="true"/>
                  <speciesReference species="M_3hmp_c" stoichiometry="1"
constant="true"/>
            </listOfReactants>
            <listOfProducts>
                  <speciesReference species="M_h_c" stoichiometry="1"
constant="true"/>
```

```
                  <speciesReference species="M_nadh_c" stoichiometry="1"
constant="true"/>
                  <speciesReference species="M_2mop_c" stoichiometry="1"
constant="true"/>
            </listOfProducts>
            <fbc:geneProductAssociation>
                  <fbc:or>
                        <fbc:geneProductRef fbc:geneProduct="SAUSA300_2294"/>
                        <fbc:geneProductRef fbc:geneProduct="SAUSA300_0226"/>
                  </fbc:or>
            </fbc:geneProductAssociation>
      </reaction>

      <reaction metaid="R_HISabc" id="R_HISabc" name="Histidine transporter"
reversible="true" fast="false" fbc:lowerFluxBound="rev_lb"
fbc:upperFluxBound="rev_ub">
            <listOfReactants>
                  <speciesReference species="M_atp_c" stoichiometry="1"
constant="true"/>
                  <speciesReference species="M_h2o_c" stoichiometry="1"
constant="true"/>
                  <speciesReference species="M_his__L_e" stoichiometry="1"
constant="true"/>
            </listOfReactants>
            <listOfProducts>
                  <speciesReference species="M_adp_c" stoichiometry="1"
constant="true"/>
                  <speciesReference species="M_h_c" stoichiometry="1"
constant="true"/>
                  <speciesReference species="M_his__L_c" stoichiometry="1"
constant="true"/>
                  <speciesReference species="M_pi_c" stoichiometry="1"
constant="true"/>
            </listOfProducts>
            <fbc:geneProductAssociation>
                  <fbc:geneProductRef fbc:geneProduct="hisP"/>
            </fbc:geneProductAssociation>
      </reaction>

      <reaction metaid="R_HISDr" id="R_HISDr" name="histidase r"
reversible="true" fast="false" fbc:lowerFluxBound="rev_lb"
fbc:upperFluxBound="rev_ub">
            <listOfReactants>
                  <speciesReference species="M_his__L_c" stoichiometry="1"
constant="true"/>
            </listOfReactants>
            <listOfProducts>
                  <speciesReference species="M_nh4_c" stoichiometry="1"
constant="true"/>
                  <speciesReference species="M_urcan_c" stoichiometry="1"
constant="true"/>
            </listOfProducts>
            <fbc:geneProductAssociation>
                  <fbc:geneProductRef fbc:geneProduct="SAUSA300_0008"/>
            </fbc:geneProductAssociation>
      </reaction>
```

```
      <reaction metaid="R_HISTD" id="R_HISTD" name="histidinol dehydrogenase"
reversible="false" fast="false" fbc:lowerFluxBound="irr_lb"
fbc:upperFluxBound="irr_ub">
            <listOfReactants>
                  <speciesReference species="M_h2o_c" stoichiometry="1"
constant="true"/>
                  <speciesReference species="M_nad_c" stoichiometry="2"
constant="true"/>
                  <speciesReference species="M_histd_c" stoichiometry="1"
constant="true"/>
            </listOfReactants>
            <listOfProducts>
                  <speciesReference species="M_h_c" stoichiometry="3"
constant="true"/>
                  <speciesReference species="M_his__L_c" stoichiometry="1"
constant="true"/>
                  <speciesReference species="M_nadh_c" stoichiometry="2"
constant="true"/>
            </listOfProducts>
            <fbc:geneProductAssociation>
                  <fbc:geneProductRef fbc:geneProduct="SAUSA300_2611"/>
            </fbc:geneProductAssociation>
      </reaction>

      <reaction metaid="R_HISTP" id="R_HISTP" name="histidinol-phosphatase"
reversible="false" fast="false" fbc:lowerFluxBound="irr_lb"
fbc:upperFluxBound="irr_ub">
            <listOfReactants>
                  <speciesReference species="M_h2o_c" stoichiometry="1"
constant="true"/>
                  <speciesReference species="M_hisp_c" stoichiometry="1"
constant="true"/>
            </listOfReactants>
            <listOfProducts>
                  <speciesReference species="M_h_c" stoichiometry="1"
constant="true"/>
                  <speciesReference species="M_pi_c" stoichiometry="1"
constant="true"/>
                  <speciesReference species="M_histd_c" stoichiometry="1"
constant="true"/>
            </listOfProducts>
            <fbc:geneProductAssociation>
                  <fbc:geneProductRef fbc:geneProduct="SAUSA300_1042"/>
            </fbc:geneProductAssociation>
      </reaction>

      <reaction metaid="R_HMBS" id="R_HMBS" name="hydroxymethylbilane
synthase" reversible="false" fast="false" fbc:lowerFluxBound="irr_lb"
fbc:upperFluxBound="irr_ub">
            <listOfReactants>
                  <speciesReference species="M_h2o_c" stoichiometry="1"
constant="true"/>
                  <speciesReference species="M_ppbng_c" stoichiometry="4"
constant="true"/>
            </listOfReactants>
            <listOfProducts>
                  <speciesReference species="M_nh4_c" stoichiometry="4"
constant="true"/>
```

```
                  <speciesReference species="M_hmbil_c" stoichiometry="1"
constant="true"/>
            </listOfProducts>
            <fbc:geneProductAssociation>
                  <fbc:geneProductRef fbc:geneProduct="SAUSA300_1617"/>
            </fbc:geneProductAssociation>
      </reaction>

      <reaction metaid="R_HMGCOARi" id="R_HMGCOARi"
name="Hydroxymethylglutaryl CoA reductase (ir)" reversible="false"
fast="false" fbc:lowerFluxBound="irr_lb" fbc:upperFluxBound="irr_ub">
            <listOfReactants>
                  <speciesReference species="M_h_c" stoichiometry="2"
constant="true"/>
                  <speciesReference species="M_nadph_c" stoichiometry="2"
constant="true"/>
                  <speciesReference species="M_hmgcoa_c" stoichiometry="1"
constant="true"/>
            </listOfReactants>
            <listOfProducts>
                  <speciesReference species="M_nadp_c" stoichiometry="2"
constant="true"/>
                  <speciesReference species="M_coa_c" stoichiometry="1"
constant="true"/>
                  <speciesReference species="M_mev__R_c" stoichiometry="1"
constant="true"/>
            </listOfProducts>
            <fbc:geneProductAssociation>
                  <fbc:geneProductRef fbc:geneProduct="SAUSA300_2483"/>
            </fbc:geneProductAssociation>
      </reaction>

      <reaction metaid="R_HMGCOAS" id="R_HMGCOAS" name="Hydroxymethylglutaryl
CoA synthase" reversible="false" fast="false" fbc:lowerFluxBound="irr_lb"
fbc:upperFluxBound="irr_ub">
            <listOfReactants>
                  <speciesReference species="M_h2o_c" stoichiometry="1"
constant="true"/>
                  <speciesReference species="M_accoa_c" stoichiometry="1"
constant="true"/>
                  <speciesReference species="M_aacoa_c" stoichiometry="1"
constant="true"/>
            </listOfReactants>
            <listOfProducts>
                  <speciesReference species="M_h_c" stoichiometry="1"
constant="true"/>
                  <speciesReference species="M_coa_c" stoichiometry="1"
constant="true"/>
                  <speciesReference species="M_hmgcoa_c" stoichiometry="1"
constant="true"/>
            </listOfProducts>
            <fbc:geneProductAssociation>
                  <fbc:geneProductRef fbc:geneProduct="SAUSA300_2484"/>
            </fbc:geneProductAssociation>
      </reaction>
```

```
      <reaction metaid="R_HMGCOASi" id="R_HMGCOASi"
name="Hydroxymethylglutaryl CoA synthase (ir)" reversible="false"
fast="false" fbc:lowerFluxBound="irr_lb" fbc:upperFluxBound="irr_ub">
            <listOfReactants>
                  <speciesReference species="M_h2o_c" stoichiometry="1"
constant="true"/>
                  <speciesReference species="M_accoa_c" stoichiometry="1"
constant="true"/>
                  <speciesReference species="M_aacoa_c" stoichiometry="1"
constant="true"/>
            </listOfReactants>
            <listOfProducts>
                  <speciesReference species="M_h_c" stoichiometry="1"
constant="true"/>
                  <speciesReference species="M_coa_c" stoichiometry="1"
constant="true"/>
                  <speciesReference species="M_hmgcoa_c" stoichiometry="1"
constant="true"/>
            </listOfProducts>
            <fbc:geneProductAssociation>
                  <fbc:geneProductRef fbc:geneProduct="SAUSA300_2484"/>
            </fbc:geneProductAssociation>
      </reaction>

      <reaction metaid="R_HMPK1" id="R_HMPK1" name="hydroxymethylpyrimidine
kinase (ATP)" reversible="false" fast="false" fbc:lowerFluxBound="irr_lb"
fbc:upperFluxBound="irr_ub">
            <listOfReactants>
                  <speciesReference species="M_atp_c" stoichiometry="1"
constant="true"/>
                  <speciesReference species="M_4ahmmp_c" stoichiometry="1"
constant="true"/>
            </listOfReactants>
            <listOfProducts>
                  <speciesReference species="M_adp_c" stoichiometry="1"
constant="true"/>
                  <speciesReference species="M_4ampm_c" stoichiometry="1"
constant="true"/>
            </listOfProducts>
            <fbc:geneProductAssociation>
                  <fbc:or>
                        <fbc:geneProductRef fbc:geneProduct="SAUSA300_0562"/>
                        <fbc:geneProductRef fbc:geneProduct="SAUSA300_2049"/>
                  </fbc:or>
            </fbc:geneProductAssociation>
      </reaction>

      <reaction metaid="R_HOM__L_Et" id="R_HOM__L_Et" name="L-Homoserine
transport " reversible="true" fast="false" fbc:lowerFluxBound="rev_lb"
fbc:upperFluxBound="rev_ub">
            <listOfReactants>
                  <speciesReference species="M_hom__L_e" stoichiometry="1"
constant="true"/>
            </listOfReactants>
            <listOfProducts>
                  <speciesReference species="M_hom__L_c" stoichiometry="1"
constant="true"/>
            </listOfProducts>
```

```
      </reaction>

      <reaction metaid="R_HPPK2" id="R_HPPK2" name="6-
hydroxymethyl__Dihydropterin pyrophosphokinase" reversible="false"
fast="false" fbc:lowerFluxBound="irr_lb" fbc:upperFluxBound="irr_ub">
            <listOfReactants>
                  <speciesReference species="M_atp_c" stoichiometry="1"
constant="true"/>
                  <speciesReference species="M_6hmhpt_c" stoichiometry="1"
constant="true"/>
            </listOfReactants>
            <listOfProducts>
                  <speciesReference species="M_amp_c" stoichiometry="1"
constant="true"/>
                  <speciesReference species="M_6hmhptpp_c" stoichiometry="1"
constant="true"/>
            </listOfProducts>
            <fbc:geneProductAssociation>
                  <fbc:geneProductRef fbc:geneProduct="SAUSA300_0494"/>
            </fbc:geneProductAssociation>
      </reaction>

      <reaction metaid="R_HPROzr" id="R_HPROzr" name="trans-4-Hydroxy__L-
proline NAD oxidoreductase " reversible="true" fast="false"
fbc:lowerFluxBound="rev_lb" fbc:upperFluxBound="rev_ub">
            <listOfReactants>
                  <speciesReference species="M_4hpro__LT_c" stoichiometry="1"
constant="true"/>
                  <speciesReference species="M_fad_c" stoichiometry="1"
constant="true"/>
            </listOfReactants>
            <listOfProducts>
                  <speciesReference species="M_fadh2_c" stoichiometry="1"
constant="true"/>
                  <speciesReference species="M_1p3h5c_c" stoichiometry="1"
constant="true"/>
            </listOfProducts>
            <fbc:geneProductAssociation>
                  <fbc:geneProductRef fbc:geneProduct="SAUSA300_1711"/>
            </fbc:geneProductAssociation>
      </reaction>

      <reaction metaid="R_HSDy" id="R_HSDy" name="homoserine dehydrogenase
(NADPH)" reversible="true" fast="false" fbc:lowerFluxBound="rev_lb"
fbc:upperFluxBound="rev_ub">
            <listOfReactants>
                  <speciesReference species="M_nadp_c" stoichiometry="1"
constant="true"/>
                  <speciesReference species="M_hom__L_c" stoichiometry="1"
constant="true"/>
            </listOfReactants>
            <listOfProducts>
                  <speciesReference species="M_h_c" stoichiometry="1"
constant="true"/>
                  <speciesReference species="M_nadph_c" stoichiometry="1"
constant="true"/>
                  <speciesReference species="M_aspsa_c" stoichiometry="1"
constant="true"/>
```

```
            </listOfProducts>
            <fbc:geneProductAssociation>
                  <fbc:geneProductRef fbc:geneProduct="SAUSA300_1226"/>
            </fbc:geneProductAssociation>
      </reaction>

      <reaction metaid="R_HSERTA" id="R_HSERTA" name="homoserine O-trans-
acetylase" reversible="false" fast="false" fbc:lowerFluxBound="irr_lb"
fbc:upperFluxBound="irr_ub">
            <listOfReactants>
                  <speciesReference species="M_accoa_c" stoichiometry="1"
constant="true"/>
                  <speciesReference species="M_hom__L_c" stoichiometry="1"
constant="true"/>
            </listOfReactants>
            <listOfProducts>
                  <speciesReference species="M_coa_c" stoichiometry="1"
constant="true"/>
                  <speciesReference species="M_achms_c" stoichiometry="1"
constant="true"/>
            </listOfProducts>
            <fbc:geneProductAssociation>
                  <fbc:geneProductRef fbc:geneProduct="SAUSA300_0012"/>
            </fbc:geneProductAssociation>
      </reaction>

      <reaction metaid="R_HSK" id="R_HSK" name="homoserine kinase"
reversible="false" fast="false" fbc:lowerFluxBound="irr_lb"
fbc:upperFluxBound="irr_ub">
            <listOfReactants>
                  <speciesReference species="M_atp_c" stoichiometry="1"
constant="true"/>
                  <speciesReference species="M_hom__L_c" stoichiometry="1"
constant="true"/>
            </listOfReactants>
            <listOfProducts>
                  <speciesReference species="M_adp_c" stoichiometry="1"
constant="true"/>
                  <speciesReference species="M_phom_c" stoichiometry="1"
constant="true"/>
            </listOfProducts>
            <fbc:geneProductAssociation>
                  <fbc:geneProductRef fbc:geneProduct="SAUSA300_1228"/>
            </fbc:geneProductAssociation>
      </reaction>

      <reaction metaid="R_HSTPTr" id="R_HSTPTr" name="histidinol-phosphate
transaminase reversible" reversible="true" fast="false"
fbc:lowerFluxBound="rev_lb" fbc:upperFluxBound="rev_ub">
            <listOfReactants>
                  <speciesReference species="M_akg_c" stoichiometry="1"
constant="true"/>
                  <speciesReference species="M_hisp_c" stoichiometry="1"
constant="true"/>
            </listOfReactants>
            <listOfProducts>
                  <speciesReference species="M_glu__L_c" stoichiometry="1"
constant="true"/>
```

```
                  <speciesReference species="M_imacp_c" stoichiometry="1"
constant="true"/>
            &lt;/listOfProducts&gt;
            &lt;fbc:geneProductAssociation&gt;
                  &lt;fbc:and&gt;
                        &lt;fbc:geneProductRef fbc:geneProduct="SAUSA300_0708"/&gt;
                        &lt;fbc:geneProductRef fbc:geneProduct="SAUSA300_2610"/&gt;
                  &lt;/fbc:and&gt;
            &lt;/fbc:geneProductAssociation&gt;
      &lt;/reaction&gt;

      <reaction metaid="R_HXAN_Et" id="R_HXAN_Et" name="Hypoxanthine
transport (transport mechanism unknown - evidence from biolog data)"
reversible="true" fast="false" fbc:lowerFluxBound="rev_lb"
fbc:upperFluxBound="rev_ub">
            &lt;listOfReactants&gt;
                  <speciesReference species="M_hxan_e" stoichiometry="1"
constant="true"/>
            &lt;/listOfReactants&gt;
            &lt;listOfProducts&gt;
                  <speciesReference species="M_hxan_c" stoichiometry="1"
constant="true"/>
            &lt;/listOfProducts&gt;
      &lt;/reaction&gt;

      <reaction metaid="R_HXPRT" id="R_HXPRT" name="hypoxanthine
phosphoribosyltransferase (Hypoxanthine)" reversible="false" fast="false"
fbc:lowerFluxBound="irr_lb" fbc:upperFluxBound="irr_ub">
            &lt;listOfReactants&gt;
                  <speciesReference species="M_hxan_c" stoichiometry="1"
constant="true"/>
                  <speciesReference species="M_prpp_c" stoichiometry="1"
constant="true"/>
            &lt;/listOfReactants&gt;
            &lt;listOfProducts&gt;
                  <speciesReference species="M_ppi_c" stoichiometry="1"
constant="true"/>
                  <speciesReference species="M_imp_c" stoichiometry="1"
constant="true"/>
            &lt;/listOfProducts&gt;
            &lt;fbc:geneProductAssociation&gt;
                  &lt;fbc:geneProductRef fbc:geneProduct="SAUSA300_0488"/&gt;
            &lt;/fbc:geneProductAssociation&gt;
      &lt;/reaction&gt;

      <reaction metaid="R_ICDHyr" id="R_ICDHyr" name="isocitrate
dehydrogenase (NADP)" reversible="false" fast="false"
fbc:lowerFluxBound="irr_lb" fbc:upperFluxBound="irr_ub">
            &lt;listOfReactants&gt;
                  <speciesReference species="M_nadp_c" stoichiometry="1"
constant="true"/>
                  <speciesReference species="M_icit_c" stoichiometry="1"
constant="true"/>
            &lt;/listOfReactants&gt;
            &lt;listOfProducts&gt;
                  <speciesReference species="M_nadph_c" stoichiometry="1"
constant="true"/>
```

```
                  <speciesReference species="M_akg_c" stoichiometry="1"
constant="true"/>
                  <speciesReference species="M_co2_c" stoichiometry="1"
constant="true"/>
            </listOfProducts>
            <fbc:geneProductAssociation>
                  <fbc:geneProductRef fbc:geneProduct="SAUSA300_1640"/>
            </fbc:geneProductAssociation>
      </reaction>

      <reaction metaid="R_ICHORS" id="R_ICHORS" name="isochorismate synthase"
reversible="true" fast="false" fbc:lowerFluxBound="rev_lb"
fbc:upperFluxBound="rev_ub">
            <listOfReactants>
                  <speciesReference species="M_chor_c" stoichiometry="1"
constant="true"/>
            </listOfReactants>
            <listOfProducts>
                  <speciesReference species="M_ichor_c" stoichiometry="1"
constant="true"/>
            </listOfProducts>
            <fbc:geneProductAssociation>
                  <fbc:geneProductRef fbc:geneProduct="SAUSA300_0945"/>
            </fbc:geneProductAssociation>
      </reaction>

      <reaction metaid="R_ICHORT" id="R_ICHORT" name="isochorismatase"
reversible="false" fast="false" fbc:lowerFluxBound="irr_lb"
fbc:upperFluxBound="irr_ub">
            <listOfReactants>
                  <speciesReference species="M_h2o_c" stoichiometry="1"
constant="true"/>
                  <speciesReference species="M_ichor_c" stoichiometry="1"
constant="true"/>
            </listOfReactants>
            <listOfProducts>
                  <speciesReference species="M_pyr_c" stoichiometry="1"
constant="true"/>
                  <speciesReference species="M_23ddhb_c" stoichiometry="1"
constant="true"/>
            </listOfProducts>
            <fbc:geneProductAssociation>
                  <fbc:or>
                        <fbc:geneProductRef fbc:geneProduct="SAUSA300_2580"/>
                        <fbc:geneProductRef fbc:geneProduct="SAUSA300_0189"/>
                  </fbc:or>
            </fbc:geneProductAssociation>
      </reaction>

      <reaction metaid="R_ICITRED" id="R_ICITRED" name="Isocitrate:NADP+
oxidoreductase (decarboxylating)" reversible="true" fast="false"
fbc:lowerFluxBound="rev_lb" fbc:upperFluxBound="rev_ub">
            <listOfReactants>
                  <speciesReference species="M_nadp_c" stoichiometry="1"
constant="true"/>
                  <speciesReference species="M_icit_c" stoichiometry="1"
constant="true"/>
            </listOfReactants>
```

```
            <listOfProducts>
                  <speciesReference species="M_h_c" stoichiometry="1"
constant="true"/>
                  <speciesReference species="M_nadph_c" stoichiometry="1"
constant="true"/>
                  <speciesReference species="M_osuc_c" stoichiometry="1"
constant="true"/>
            </listOfProducts>
            <fbc:geneProductAssociation>
                  <fbc:geneProductRef fbc:geneProduct="SAUSA300_1640"/>
            </fbc:geneProductAssociation>
      </reaction>

      <reaction metaid="R_IG3PS" id="R_IG3PS" name="Imidazole-glycerol-3-
phosphate synthase" reversible="false" fast="false"
fbc:lowerFluxBound="irr_lb" fbc:upperFluxBound="irr_ub">
            <listOfReactants>
                  <speciesReference species="M_gln__L_c" stoichiometry="1"
constant="true"/>
                  <speciesReference species="M_prlp_c" stoichiometry="1"
constant="true"/>
            </listOfReactants>
            <listOfProducts>
                  <speciesReference species="M_h_c" stoichiometry="1"
constant="true"/>
                  <speciesReference species="M_glu__L_c" stoichiometry="1"
constant="true"/>
                  <speciesReference species="M_aicar_c" stoichiometry="1"
constant="true"/>
                  <speciesReference species="M_eig3p_c" stoichiometry="1"
constant="true"/>
            </listOfProducts>
            <fbc:geneProductAssociation>
                  <fbc:and>
                        <fbc:geneProductRef fbc:geneProduct="SAUSA300_2608"/>
                        <fbc:geneProductRef fbc:geneProduct="SAUSA300_2606"/>
                  </fbc:and>
            </fbc:geneProductAssociation>
      </reaction>

      <reaction metaid="R_IGPDH" id="R_IGPDH" name="imidazoleglycerol-
phosphate dehydratase" reversible="false" fast="false"
fbc:lowerFluxBound="irr_lb" fbc:upperFluxBound="irr_ub">
            <listOfReactants>
                  <speciesReference species="M_eig3p_c" stoichiometry="1"
constant="true"/>
            </listOfReactants>
            <listOfProducts>
                  <speciesReference species="M_h2o_c" stoichiometry="1"
constant="true"/>
                  <speciesReference species="M_imacp_c" stoichiometry="1"
constant="true"/>
            </listOfProducts>
            <fbc:geneProductAssociation>
                  <fbc:geneProductRef fbc:geneProduct="SAUSA300_2609"/>
            </fbc:geneProductAssociation>
      </reaction>
```

```
      <reaction metaid="R_IGPS" id="R_IGPS" name="indole-3-glycerol-phosphate
synthase" reversible="false" fast="false" fbc:lowerFluxBound="irr_lb"
fbc:upperFluxBound="irr_ub">
            <listOfReactants>
                  <speciesReference species="M_h_c" stoichiometry="1"
constant="true"/>
                  <speciesReference species="M_2cpr5p_c" stoichiometry="1"
constant="true"/>
            </listOfReactants>
            <listOfProducts>
                  <speciesReference species="M_h2o_c" stoichiometry="1"
constant="true"/>
                  <speciesReference species="M_co2_c" stoichiometry="1"
constant="true"/>
                  <speciesReference species="M_3ig3p_c" stoichiometry="1"
constant="true"/>
            </listOfProducts>
            <fbc:geneProductAssociation>
                  <fbc:geneProductRef fbc:geneProduct="SAUSA300_1265"/>
            </fbc:geneProductAssociation>
      </reaction>

      <reaction metaid="R_ILASL" id="R_ILASL"
name="isoheptadecanoyl__Lipoteichoic acid synthesis n=24  linked  N-
acetylglucosamine substituted " reversible="true" fast="false"
fbc:lowerFluxBound="rev_lb" fbc:upperFluxBound="rev_ub">
            <listOfReactants>
                  <speciesReference species="M_uacgam_c" stoichiometry="24"
constant="true"/>
                  <speciesReference species="M_isolp24u_c" stoichiometry="1"
constant="true"/>
            </listOfReactants>
            <listOfProducts>
                  <speciesReference species="M_udp_c" stoichiometry="24"
constant="true"/>
                  <speciesReference species="M_isoh24s_c" stoichiometry="1"
constant="true"/>
            </listOfProducts>
            <fbc:geneProductAssociation>
                  <fbc:geneProductRef fbc:geneProduct="SAUSA300_0731"/>
            </fbc:geneProductAssociation>
      </reaction>

      <reaction metaid="R_ILEt2r" id="R_ILEt2r" name="L-isoleucine reversible
transport via proton symport" reversible="true" fast="false"
fbc:lowerFluxBound="rev_lb" fbc:upperFluxBound="rev_ub">
            <listOfReactants>
                  <speciesReference species="M_h_e" stoichiometry="1"
constant="true"/>
                  <speciesReference species="M_ile__L_e" stoichiometry="1"
constant="true"/>
            </listOfReactants>
            <listOfProducts>
                  <speciesReference species="M_h_c" stoichiometry="1"
constant="true"/>
                  <speciesReference species="M_ile__L_c" stoichiometry="1"
constant="true"/>
            </listOfProducts>
```

```
			<fbc:geneProductAssociation>
				<fbc:or>
					<fbc:geneProductRef fbc:geneProduct="SAUSA300_1300"/>
					<fbc:geneProductRef fbc:geneProduct="SAUSA300_0188"/>
					<fbc:geneProductRef fbc:geneProduct="SAUSA300_0306"/>
				</fbc:or>
			</fbc:geneProductAssociation>
		</reaction>

		<reaction metaid="R_ILETA" id="R_ILETA" name="isoleucine transaminase"
reversible="true" fast="false" fbc:lowerFluxBound="rev_lb"
fbc:upperFluxBound="rev_ub">
			<listOfReactants>
				<speciesReference species="M_akg_c" stoichiometry="1"
constant="true"/>
				<speciesReference species="M_ile__L_c" stoichiometry="1"
constant="true"/>
			</listOfReactants>
			<listOfProducts>
				<speciesReference species="M_glu__L_c" stoichiometry="1"
constant="true"/>
				<speciesReference species="M_3mop_c" stoichiometry="1"
constant="true"/>
			</listOfProducts>
			<fbc:geneProductAssociation>
				<fbc:geneProductRef fbc:geneProduct="SAUSA300_0539"/>
			</fbc:geneProductAssociation>
		</reaction>

		<reaction metaid="R_ILETRS" id="R_ILETRS" name="Isoleucyl-tRNA
synthetase" reversible="false" fast="false" fbc:lowerFluxBound="irr_lb"
fbc:upperFluxBound="irr_ub">
			<listOfReactants>
				<speciesReference species="M_atp_c" stoichiometry="1"
constant="true"/>
				<speciesReference species="M_ile__L_c" stoichiometry="1"
constant="true"/>
				<speciesReference species="M_trnaile_c" stoichiometry="1"
constant="true"/>
			</listOfReactants>
			<listOfProducts>
				<speciesReference species="M_ppi_c" stoichiometry="1"
constant="true"/>
				<speciesReference species="M_amp_c" stoichiometry="1"
constant="true"/>
				<speciesReference species="M_iletrna_c" stoichiometry="1"
constant="true"/>
			</listOfProducts>
			<fbc:geneProductAssociation>
				<fbc:geneProductRef fbc:geneProduct="SAUSA300_1087"/>
			</fbc:geneProductAssociation>
		</reaction>

		<reaction metaid="R_IMACTD" id="R_IMACTD" name="Imidazole acetaldeyde
dehydrogenase" reversible="true" fast="false" fbc:lowerFluxBound="rev_lb"
fbc:upperFluxBound="rev_ub">
			<listOfReactants>
```

```
                  <speciesReference species="M_h2o_c" stoichiometry="1"
constant="true"/>
                  <speciesReference species="M_nad_c" stoichiometry="1"
constant="true"/>
                  <speciesReference species="M_im4act_c" stoichiometry="1"
constant="true"/>
            &lt;/listOfReactants&gt;
            &lt;listOfProducts&gt;
                  <speciesReference species="M_h_c" stoichiometry="2"
constant="true"/>
                  <speciesReference species="M_nadh_c" stoichiometry="1"
constant="true"/>
                  <speciesReference species="M_im4ac_c" stoichiometry="1"
constant="true"/>
            &lt;/listOfProducts&gt;
            &lt;fbc:geneProductAssociation&gt;
                  &lt;fbc:or&gt;
                        &lt;fbc:geneProductRef fbc:geneProduct="SAUSA300_1901"/&gt;
                        &lt;fbc:geneProductRef fbc:geneProduct="SAUSA300_2076"/&gt;
                  &lt;/fbc:or&gt;
            &lt;/fbc:geneProductAssociation&gt;
      &lt;/reaction&gt;

      <reaction metaid="R_IMPC" id="R_IMPC" name="IMP cyclohydrolase"
reversible="true" fast="false" fbc:lowerFluxBound="rev_lb"
fbc:upperFluxBound="rev_ub">
            &lt;listOfReactants&gt;
                  <speciesReference species="M_h2o_c" stoichiometry="1"
constant="true"/>
                  <speciesReference species="M_imp_c" stoichiometry="1"
constant="true"/>
            &lt;/listOfReactants&gt;
            &lt;listOfProducts&gt;
                  <speciesReference species="M_fprica_c" stoichiometry="1"
constant="true"/>
            &lt;/listOfProducts&gt;
            &lt;fbc:geneProductAssociation&gt;
                  &lt;fbc:geneProductRef fbc:geneProduct="SAUSA300_0975"/&gt;
            &lt;/fbc:geneProductAssociation&gt;
      &lt;/reaction&gt;

      <reaction metaid="R_IMPD" id="R_IMPD" name="IMP dehydrogenase"
reversible="false" fast="false" fbc:lowerFluxBound="irr_lb"
fbc:upperFluxBound="irr_ub">
            &lt;listOfReactants&gt;
                  <speciesReference species="M_h2o_c" stoichiometry="1"
constant="true"/>
                  <speciesReference species="M_nad_c" stoichiometry="1"
constant="true"/>
                  <speciesReference species="M_imp_c" stoichiometry="1"
constant="true"/>
            &lt;/listOfReactants&gt;
            &lt;listOfProducts&gt;
                  <speciesReference species="M_h_c" stoichiometry="1"
constant="true"/>
                  <speciesReference species="M_nadh_c" stoichiometry="1"
constant="true"/>
```

```
                  <speciesReference species="M_xmp_c" stoichiometry="1"
constant="true"/>
            &lt;/listOfProducts&gt;
            &lt;fbc:geneProductAssociation&gt;
                  &lt;fbc:geneProductRef fbc:geneProduct="SAUSA300_0388"/&gt;
            &lt;/fbc:geneProductAssociation&gt;
      &lt;/reaction&gt;

      <reaction metaid="R_INOST_Et" id="R_INOST_Et" name="myo-Inositol
transport (transport mechanism unknown - evidence from biolog data)"
reversible="true" fast="false" fbc:lowerFluxBound="rev_lb"
fbc:upperFluxBound="rev_ub">
            &lt;listOfReactants&gt;
                  <speciesReference species="M_inost_e" stoichiometry="1"
constant="true"/>
            &lt;/listOfReactants&gt;
            &lt;listOfProducts&gt;
                  <speciesReference species="M_inost_c" stoichiometry="1"
constant="true"/>
            &lt;/listOfProducts&gt;
      &lt;/reaction&gt;

      <reaction metaid="R_INSH" id="R_INSH" name="Inosine hydrolase"
reversible="false" fast="false" fbc:lowerFluxBound="irr_lb"
fbc:upperFluxBound="irr_ub">
            &lt;listOfReactants&gt;
                  <speciesReference species="M_h2o_c" stoichiometry="1"
constant="true"/>
                  <speciesReference species="M_ins_c" stoichiometry="1"
constant="true"/>
            &lt;/listOfReactants&gt;
            &lt;listOfProducts&gt;
                  <speciesReference species="M_rib__D_c" stoichiometry="1"
constant="true"/>
                  <speciesReference species="M_hxan_c" stoichiometry="1"
constant="true"/>
            &lt;/listOfProducts&gt;
            &lt;fbc:geneProductAssociation&gt;
                  &lt;fbc:or&gt;
                        &lt;fbc:geneProductRef fbc:geneProduct="SAUSA300_2234"/&gt;
                        &lt;fbc:geneProductRef fbc:geneProduct="SAUSA300_0237"/&gt;
                  &lt;/fbc:or&gt;
            &lt;/fbc:geneProductAssociation&gt;
      &lt;/reaction&gt;

      <reaction metaid="R_INSt2r" id="R_INSt2r" name="inosine transport in
via proton symport, reversible" reversible="true" fast="false"
fbc:lowerFluxBound="rev_lb" fbc:upperFluxBound="rev_ub">
            &lt;listOfReactants&gt;
                  <speciesReference species="M_h_e" stoichiometry="1"
constant="true"/>
                  <speciesReference species="M_ins_e" stoichiometry="1"
constant="true"/>
            &lt;/listOfReactants&gt;
            &lt;listOfProducts&gt;
                  <speciesReference species="M_h_c" stoichiometry="1"
constant="true"/>
```

```
                  <speciesReference species="M_ins_c" stoichiometry="1"
constant="true"/>
            </listOfProducts>
            <fbc:geneProductAssociation>
                  <fbc:geneProductRef fbc:geneProduct="SAUSA300_0506"/>
            </fbc:geneProductAssociation>
      </reaction>

      <reaction metaid="R_IPDDI" id="R_IPDDI" name="isopentenyl__Diphosphate
D-isomerase" reversible="true" fast="false" fbc:lowerFluxBound="rev_lb"
fbc:upperFluxBound="rev_ub">
            <listOfReactants>
                  <speciesReference species="M_ipdp_c" stoichiometry="1"
constant="true"/>
            </listOfReactants>
            <listOfProducts>
                  <speciesReference species="M_dmpp_c" stoichiometry="1"
constant="true"/>
            </listOfProducts>
            <fbc:geneProductAssociation>
                  <fbc:geneProductRef fbc:geneProduct="SAUSA300_2292"/>
            </fbc:geneProductAssociation>
      </reaction>

      <reaction metaid="R_IPG3P" id="R_IPG3P" name="isopentadecanoyl-
glycerol-3-phosphate O-acyltransferase " reversible="false" fast="false"
fbc:lowerFluxBound="irr_lb" fbc:upperFluxBound="irr_ub">
            <listOfReactants>
                  <speciesReference species="M_glyc3p_c" stoichiometry="1"
constant="true"/>
                  <speciesReference species="M_fa3coa_c" stoichiometry="1"
constant="true"/>
            </listOfReactants>
            <listOfProducts>
                  <speciesReference species="M_coa_c" stoichiometry="1"
constant="true"/>
                  <speciesReference species="M_1ipsg3p_c" stoichiometry="1"
constant="true"/>
            </listOfProducts>
            <fbc:geneProductAssociation>
                  <fbc:and>
                        <fbc:geneProductRef fbc:geneProduct="SAUSA300_1249"/>
                        <fbc:or>
                              <fbc:geneProductRef
fbc:geneProduct="SAUSA300_1122"/>
                              <fbc:geneProductRef
fbc:geneProduct="SAUSA300_1121"/>
                        </fbc:or>
                  </fbc:and>
            </fbc:geneProductAssociation>
      </reaction>

      <reaction metaid="R_IPMD" id="R_IPMD" name="3-isopropylmalate
dehydrogenase" reversible="false" fast="false" fbc:lowerFluxBound="irr_lb"
fbc:upperFluxBound="irr_ub">
            <listOfReactants>
                  <speciesReference species="M_nad_c" stoichiometry="1"
constant="true"/>
```

```
                  <speciesReference species="M_3c2hmp_c" stoichiometry="1"
constant="true"/>
            </listOfReactants>
            <listOfProducts>
                  <speciesReference species="M_h_c" stoichiometry="1"
constant="true"/>
                  <speciesReference species="M_nadh_c" stoichiometry="1"
constant="true"/>
                  <speciesReference species="M_3c4mop_c" stoichiometry="1"
constant="true"/>
            </listOfProducts>
            <fbc:geneProductAssociation>
                  <fbc:geneProductRef fbc:geneProduct="SAUSA300_2011"/>
            </fbc:geneProductAssociation>
      </reaction>

      <reaction metaid="R_IPPMIa" id="R_IPPMIa" name="3-isopropylmalate
dehydratase" reversible="true" fast="false" fbc:lowerFluxBound="rev_lb"
fbc:upperFluxBound="rev_ub">
            <listOfReactants>
                  <speciesReference species="M_3c2hmp_c" stoichiometry="1"
constant="true"/>
            </listOfReactants>
            <listOfProducts>
                  <speciesReference species="M_h2o_c" stoichiometry="1"
constant="true"/>
                  <speciesReference species="M_2ippm_c" stoichiometry="1"
constant="true"/>
            </listOfProducts>
            <fbc:geneProductAssociation>
                  <fbc:and>
                        <fbc:geneProductRef fbc:geneProduct="SAUSA300_2013"/>
                        <fbc:geneProductRef fbc:geneProduct="SAUSA300_2012"/>
                  </fbc:and>
            </fbc:geneProductAssociation>
      </reaction>

      <reaction metaid="R_IPPMIb" id="R_IPPMIb" name="2-isopropylmalate
hydratase" reversible="true" fast="false" fbc:lowerFluxBound="rev_lb"
fbc:upperFluxBound="rev_ub">
            <listOfReactants>
                  <speciesReference species="M_3c3hmp_c" stoichiometry="1"
constant="true"/>
            </listOfReactants>
            <listOfProducts>
                  <speciesReference species="M_h2o_c" stoichiometry="1"
constant="true"/>
                  <speciesReference species="M_2ippm_c" stoichiometry="1"
constant="true"/>
            </listOfProducts>
            <fbc:geneProductAssociation>
                  <fbc:or>
                        <fbc:geneProductRef fbc:geneProduct="SAUSA300_2013"/>
                        <fbc:geneProductRef fbc:geneProduct="SAUSA300_2012"/>
                  </fbc:or>
            </fbc:geneProductAssociation>
      </reaction>
```

```
      <reaction metaid="R_IPPS" id="R_IPPS" name="2-isopropylmalate synthase"
reversible="false" fast="false" fbc:lowerFluxBound="irr_lb"
fbc:upperFluxBound="irr_ub">
            <listOfReactants>
                  <speciesReference species="M_h2o_c" stoichiometry="1"
constant="true"/>
                  <speciesReference species="M_3mob_c" stoichiometry="1"
constant="true"/>
                  <speciesReference species="M_accoa_c" stoichiometry="1"
constant="true"/>
            </listOfReactants>
            <listOfProducts>
                  <speciesReference species="M_h_c" stoichiometry="1"
constant="true"/>
                  <speciesReference species="M_coa_c" stoichiometry="1"
constant="true"/>
                  <speciesReference species="M_3c3hmp_c" stoichiometry="1"
constant="true"/>
            </listOfProducts>
            <fbc:geneProductAssociation>
                  <fbc:or>
                        <fbc:geneProductRef fbc:geneProduct="SAUSA300_2010"/>
                        <fbc:geneProductRef fbc:geneProduct="SAUSA300_0879"/>
                  </fbc:or>
            </fbc:geneProductAssociation>
      </reaction>

      <reaction metaid="R_ISCPG3P" id="R_ISCPG3P" name="isoheptadecanoyl-
CDPdiacylglycerol sn-glycerol-3-phosphate 3-phosphatidyltransferase "
reversible="true" fast="false" fbc:lowerFluxBound="rev_lb"
fbc:upperFluxBound="rev_ub">
            <listOfReactants>
                  <speciesReference species="M_glyc3p_c" stoichiometry="1"
constant="true"/>
                  <speciesReference species="M_c12diigly_c" stoichiometry="1"
constant="true"/>
            </listOfReactants>
            <listOfProducts>
                  <speciesReference species="M_cmp_c" stoichiometry="1"
constant="true"/>
                  <speciesReference species="M_diispgp_c" stoichiometry="1"
constant="true"/>
            </listOfProducts>
            <fbc:geneProductAssociation>
                  <fbc:geneProductRef fbc:geneProduct="SAUSA300_1176"/>
            </fbc:geneProductAssociation>
      </reaction>

      <reaction metaid="R_ISGASS" id="R_ISGASS"
name="isohexadecanoyl__Lipoteichoic acid synthesis n=24  linked  glucose
substituted " reversible="true" fast="false" fbc:lowerFluxBound="rev_lb"
fbc:upperFluxBound="rev_ub">
            <listOfReactants>
                  <speciesReference species="M_udpg_c" stoichiometry="24"
constant="true"/>
                  <speciesReference species="M_isohdt24u_c" stoichiometry="1"
constant="true"/>
            </listOfReactants>
```

```
            <listOfProducts>
                  <speciesReference species="M_udp_c" stoichiometry="24"
constant="true"/>
                  <speciesReference species="M_isodec24s_c" stoichiometry="1"
constant="true"/>
            </listOfProducts>
            <fbc:geneProductAssociation>
                  <fbc:or>
                        <fbc:geneProductRef fbc:geneProduct="SAUSA300_0939"/>
                        <fbc:geneProductRef fbc:geneProduct="SAUSA300_0550"/>
                        <fbc:geneProductRef fbc:geneProduct="SAUSA300_0549"/>
                  </fbc:or>
            </fbc:geneProductAssociation>
      </reaction>

      <reaction metaid="R_ISHCDP3" id="R_ISHCDP3" name="isohexadecanoyl-
CDPdiacylglycerol sn-glycerol-3-phosphate 3-phosphatidyltransferase "
reversible="true" fast="false" fbc:lowerFluxBound="rev_lb"
fbc:upperFluxBound="rev_ub">
            <listOfReactants>
                  <speciesReference species="M_glyc3p_c" stoichiometry="1"
constant="true"/>
                  <speciesReference species="M_c12diidgly_c"
stoichiometry="1" constant="true"/>
            </listOfReactants>
            <listOfProducts>
                  <speciesReference species="M_cmp_c" stoichiometry="1"
constant="true"/>
                  <speciesReference species="M_diihexpgp_c" stoichiometry="1"
constant="true"/>
            </listOfProducts>
            <fbc:geneProductAssociation>
                  <fbc:geneProductRef fbc:geneProduct="SAUSA300_1176"/>
            </fbc:geneProductAssociation>
      </reaction>

      <reaction metaid="R_ISHDK" id="R_ISHDK"
name="isoheptadecanoyl__Diacylglycerol kinase " reversible="false"
fast="false" fbc:lowerFluxBound="irr_lb" fbc:upperFluxBound="irr_ub">
            <listOfReactants>
                  <speciesReference species="M_atp_c" stoichiometry="1"
constant="true"/>
                  <speciesReference species="M_12diisgly_c" stoichiometry="1"
constant="true"/>
            </listOfReactants>
            <listOfProducts>
                  <speciesReference species="M_adp_c" stoichiometry="1"
constant="true"/>
                  <speciesReference species="M_12dsgly3p_c" stoichiometry="1"
constant="true"/>
            </listOfProducts>
            <fbc:geneProductAssociation>
                  <fbc:geneProductRef fbc:geneProduct="SAUSA300_1529"/>
            </fbc:geneProductAssociation>
      </reaction>

      <reaction metaid="R_ISHDLAS" id="R_ISHDLAS"
name="isohexadecanoyl__Lipoteichoic acid synthesis n=24  linked  N-
```

```
acetylglucosamine substituted " reversible="true" fast="false"
fbc:lowerFluxBound="rev_lb" fbc:upperFluxBound="rev_ub">
            <listOfReactants>
                  <speciesReference species="M_uacgam_c" stoichiometry="24"
constant="true"/>
                  <speciesReference species="M_isohdt24u_c" stoichiometry="1"
constant="true"/>
            </listOfReactants>
            <listOfProducts>
                  <speciesReference species="M_udp_c" stoichiometry="24"
constant="true"/>
                  <speciesReference species="M_ish24s_c" stoichiometry="1"
constant="true"/>
            </listOfProducts>
            <fbc:geneProductAssociation>
                  <fbc:geneProductRef fbc:geneProduct="SAUSA300_0731"/>
            </fbc:geneProductAssociation>
      </reaction>

      <reaction metaid="R_ISHEGL3P" id="R_ISHEGL3P" name="isohexadecanoyl-
glycerol-3-phosphate O-acyltransferase " reversible="false" fast="false"
fbc:lowerFluxBound="irr_lb" fbc:upperFluxBound="irr_ub">
            <listOfReactants>
                  <speciesReference species="M_glyc3p_c" stoichiometry="1"
constant="true"/>
                  <speciesReference species="M_fa6coa_c" stoichiometry="1"
constant="true"/>
            </listOfReactants>
            <listOfProducts>
                  <speciesReference species="M_coa_c" stoichiometry="1"
constant="true"/>
                  <speciesReference species="M_1ihgly3p_c" stoichiometry="1"
constant="true"/>
            </listOfProducts>
            <fbc:geneProductAssociation>
                  <fbc:and>
                        <fbc:geneProductRef fbc:geneProduct="SAUSA300_1249"/>
                        <fbc:or>
                              <fbc:geneProductRef
fbc:geneProduct="SAUSA300_1122"/>
                              <fbc:geneProductRef
fbc:geneProduct="SAUSA300_1121"/>
                        </fbc:or>
                  </fbc:and>
            </fbc:geneProductAssociation>
      </reaction>

      <reaction metaid="R_ISHGLT" id="R_ISHGLT" name="isohexadecanoyl-UDP-
glucosyltransferase monoglucosyl " reversible="true" fast="false"
fbc:lowerFluxBound="rev_lb" fbc:upperFluxBound="rev_ub">
            <listOfReactants>
                  <speciesReference species="M_udpg_c" stoichiometry="1"
constant="true"/>
                  <speciesReference species="M_12dihexsgly_c"
stoichiometry="1" constant="true"/>
            </listOfReactants>
            <listOfProducts>
```

<speciesReference species="M_udp_c" stoichiometry="1"
constant="true"/>
<speciesReference species="M_m12diihgly_c"
stoichiometry="1" constant="true"/>
</listOfProducts>
<fbc:geneProductAssociation>
<fbc:geneProductRef fbc:geneProduct="SAUSA300_0918"/>
</fbc:geneProductAssociation>
</reaction>

<reaction metaid="R_ISHPCYTT" id="R_ISHPCYTT" name="isohexadecanoyl-
phosphatidate cytidylyltransferase " reversible="true" fast="false"
fbc:lowerFluxBound="rev_lb" fbc:upperFluxBound="rev_ub">
<listOfReactants>
<speciesReference species="M_ctp_c" stoichiometry="1"
constant="true"/>
<speciesReference species="M_12dhsgly3p_c"
stoichiometry="1" constant="true"/>
</listOfReactants>
<listOfProducts>
<speciesReference species="M_ppi_c" stoichiometry="1"
constant="true"/>
<speciesReference species="M_c12diidgly_c"
stoichiometry="1" constant="true"/>
</listOfProducts>
<fbc:geneProductAssociation>
<fbc:geneProductRef fbc:geneProduct="SAUSA300_1154"/>
</fbc:geneProductAssociation>
</reaction>

<reaction metaid="R_ISO1A3P" id="R_ISO1A3P" name="isopentadecanoyl-1-
acylglycerol-3-phosphate O-acyltransferase " reversible="false" fast="false"
fbc:lowerFluxBound="irr_lb" fbc:upperFluxBound="irr_ub">
<listOfReactants>
<speciesReference species="M_fa3coa_c" stoichiometry="1"
constant="true"/>
<speciesReference species="M_1ipsg3p_c" stoichiometry="1"
constant="true"/>
</listOfReactants>
<listOfProducts>
<speciesReference species="M_coa_c" stoichiometry="1"
constant="true"/>
<speciesReference species="M_12dpsgly3p_c"
stoichiometry="1" constant="true"/>
</listOfProducts>
<fbc:geneProductAssociation>
<fbc:geneProductRef fbc:geneProduct="SAUSA300_1673"/>
</fbc:geneProductAssociation>
</reaction>

<reaction metaid="R_ISO1A3PAT" id="R_ISO1A3PAT" name="isohexadecanoyl-
1-acylglycerol-3-phosphate O-acyltransferase " reversible="false"
fast="false" fbc:lowerFluxBound="irr_lb" fbc:upperFluxBound="irr_ub">
<listOfReactants>
<speciesReference species="M_fa6coa_c" stoichiometry="1"
constant="true"/>
<speciesReference species="M_1ihgly3p_c" stoichiometry="1"
constant="true"/>

```
            </listOfReactants>
            <listOfProducts>
                  <speciesReference species="M_coa_c" stoichiometry="1"
constant="true"/>
                  <speciesReference species="M_12dhsgly3p_c"
stoichiometry="1" constant="true"/>
            </listOfProducts>
            <fbc:geneProductAssociation>
                  <fbc:geneProductRef fbc:geneProduct="SAUSA300_1673"/>
            </fbc:geneProductAssociation>
      </reaction>

      <reaction metaid="R_ISOCARDS" id="R_ISOCARDS" name="isohexadecanoyl-
cardiolipin synthase " reversible="true" fast="false"
fbc:lowerFluxBound="rev_lb" fbc:upperFluxBound="rev_ub">
            <listOfReactants>
                  <speciesReference species="M_diihexpglyc_c"
stoichiometry="2" constant="true"/>
            </listOfReactants>
            <listOfProducts>
                  <speciesReference species="M_glyc_c" stoichiometry="1"
constant="true"/>
                  <speciesReference species="M_isohdcard_c" stoichiometry="1"
constant="true"/>
            </listOfProducts>
            <fbc:geneProductAssociation>
                  <fbc:or>
                        <fbc:geneProductRef fbc:geneProduct="SAUSA300_2044"/>
                        <fbc:geneProductRef fbc:geneProduct="SAUSA300_1216"/>
                  </fbc:or>
            </fbc:geneProductAssociation>
      </reaction>

      <reaction metaid="R_ISODK" id="R_ISODK"
name="isotetradecanoyl__Diacylglycerol kinase " reversible="false"
fast="false" fbc:lowerFluxBound="irr_lb" fbc:upperFluxBound="irr_ub">
            <listOfReactants>
                  <speciesReference species="M_atp_c" stoichiometry="1"
constant="true"/>
                  <speciesReference species="M_12ditetgly_c"
stoichiometry="1" constant="true"/>
            </listOfReactants>
            <listOfProducts>
                  <speciesReference species="M_adp_c" stoichiometry="1"
constant="true"/>
                  <speciesReference species="M_12diidgly3p_c"
stoichiometry="1" constant="true"/>
            </listOfProducts>
            <fbc:geneProductAssociation>
                  <fbc:geneProductRef fbc:geneProduct="SAUSA300_1529"/>
            </fbc:geneProductAssociation>
      </reaction>

      <reaction metaid="R_ISODLIP" id="R_ISODLIP"
name="isopentadecanoyl__Lipoteichoic acid synthesis n=24  linked  glucose
substituted " reversible="true" fast="false" fbc:lowerFluxBound="rev_lb"
fbc:upperFluxBound="rev_ub">
            <listOfReactants>
```

```
                  <speciesReference species="M_udpg_c" stoichiometry="24"
constant="true"/>
                  <speciesReference species="M_isop24u_c" stoichiometry="1"
constant="true"/>
            &lt;/listOfReactants&gt;
            &lt;listOfProducts&gt;
                  <speciesReference species="M_udp_c" stoichiometry="24"
constant="true"/>
                  <speciesReference species="M_isop24glcs_c"
stoichiometry="1" constant="true"/>
            &lt;/listOfProducts&gt;
            &lt;fbc:geneProductAssociation&gt;
                  &lt;fbc:or&gt;
                        &lt;fbc:geneProductRef fbc:geneProduct="SAUSA300_0939"/&gt;
                        &lt;fbc:geneProductRef fbc:geneProduct="SAUSA300_0550"/&gt;
                        &lt;fbc:geneProductRef fbc:geneProduct="SAUSA300_0549"/&gt;
                  &lt;/fbc:or&gt;
            &lt;/fbc:geneProductAssociation&gt;
      &lt;/reaction&gt;

      <reaction metaid="R_ISOGL3P" id="R_ISOGL3P" name="2-methylpropionyl-ACP
malonyl-acyl-carrier-protein C-acyltransferase decarboxylating "
reversible="false" fast="false" fbc:lowerFluxBound="irr_lb"
fbc:upperFluxBound="irr_ub">
            &lt;listOfReactants&gt;
                  <speciesReference species="M_h_c" stoichiometry="1"
constant="true"/>
                  <speciesReference species="M_malACP_c" stoichiometry="1"
constant="true"/>
                  <speciesReference species="M_isobutACP_c" stoichiometry="1"
constant="true"/>
            &lt;/listOfReactants&gt;
            &lt;listOfProducts&gt;
                  <speciesReference species="M_ACP_c" stoichiometry="1"
constant="true"/>
                  <speciesReference species="M_co2_c" stoichiometry="1"
constant="true"/>
                  <speciesReference species="M_4m3opACP_c" stoichiometry="1"
constant="true"/>
            &lt;/listOfProducts&gt;
            &lt;fbc:geneProductAssociation&gt;
                  &lt;fbc:and&gt;
                        &lt;fbc:geneProductRef fbc:geneProduct="SAUSA300_0886"/&gt;
                        &lt;fbc:geneProductRef fbc:geneProduct="SAUSA300_0885"/&gt;
                  &lt;/fbc:and&gt;
            &lt;/fbc:geneProductAssociation&gt;
      &lt;/reaction&gt;

      <reaction metaid="R_ISOHAS" id="R_ISOHAS"
name="isoheptadecanoyl__Lipoteichoic acid synthesis n=24  linked  glucose
substituted " reversible="true" fast="false" fbc:lowerFluxBound="rev_lb"
fbc:upperFluxBound="rev_ub">
            &lt;listOfReactants&gt;
                  <speciesReference species="M_udpg_c" stoichiometry="24"
constant="true"/>
                  <speciesReference species="M_isolp24u_c" stoichiometry="1"
constant="true"/>
            &lt;/listOfReactants&gt;
```

```
            <listOfProducts>
                  <speciesReference species="M_udp_c" stoichiometry="24"
constant="true"/>
                  <speciesReference species="M_isohep24s_c" stoichiometry="1"
constant="true"/>
            </listOfProducts>
            <fbc:geneProductAssociation>
                  <fbc:or>
                        <fbc:geneProductRef fbc:geneProduct="SAUSA300_0939"/>
                        <fbc:geneProductRef fbc:geneProduct="SAUSA300_0550"/>
                        <fbc:geneProductRef fbc:geneProduct="SAUSA300_0549"/>
                  </fbc:or>
            </fbc:geneProductAssociation>
      </reaction>

      <reaction metaid="R_ISOHCARDS" id="R_ISOHCARDS" name="isoheptadecanoyl-
cardiolipin synthase " reversible="true" fast="false"
fbc:lowerFluxBound="rev_lb" fbc:upperFluxBound="rev_ub">
            <listOfReactants>
                  <speciesReference species="M_diihpgly_c" stoichiometry="2"
constant="true"/>
            </listOfReactants>
            <listOfProducts>
                  <speciesReference species="M_glyc_c" stoichiometry="1"
constant="true"/>
                  <speciesReference species="M_isohcard_c" stoichiometry="1"
constant="true"/>
            </listOfProducts>
            <fbc:geneProductAssociation>
                  <fbc:or>
                        <fbc:geneProductRef fbc:geneProduct="SAUSA300_2044"/>
                        <fbc:geneProductRef fbc:geneProduct="SAUSA300_1216"/>
                  </fbc:or>
            </fbc:geneProductAssociation>
      </reaction>

      <reaction metaid="R_ISOHEP1A3PA" id="R_ISOHEP1A3PA"
name="isoheptadecanoyl-1-acylglycerol-3-phosphate O-acyltransferase "
reversible="false" fast="false" fbc:lowerFluxBound="irr_lb"
fbc:upperFluxBound="irr_ub">
            <listOfReactants>
                  <speciesReference species="M_fa11coa_c" stoichiometry="1"
constant="true"/>
                  <speciesReference species="M_1ihsg3p_c" stoichiometry="1"
constant="true"/>
            </listOfReactants>
            <listOfProducts>
                  <speciesReference species="M_coa_c" stoichiometry="1"
constant="true"/>
                  <speciesReference species="M_12dsgly3p_c" stoichiometry="1"
constant="true"/>
            </listOfProducts>
            <fbc:geneProductAssociation>
                  <fbc:geneProductRef fbc:geneProduct="SAUSA300_1673"/>
            </fbc:geneProductAssociation>
      </reaction>
```

```
      <reaction metaid="R_ISOHEPD" id="R_ISOHEPD" name="isoheptadecanoyl-UDP-
glucosyltransferase diglucosyl " reversible="true" fast="false"
fbc:lowerFluxBound="rev_lb" fbc:upperFluxBound="rev_ub">
            <listOfReactants>
                  <speciesReference species="M_udpg_c" stoichiometry="1"
constant="true"/>
                  <speciesReference species="M_m12dhdglyc_c"
stoichiometry="1" constant="true"/>
            </listOfReactants>
            <listOfProducts>
                  <speciesReference species="M_udp_c" stoichiometry="1"
constant="true"/>
                  <speciesReference species="M_dglu12dig_c" stoichiometry="1"
constant="true"/>
            </listOfProducts>
            <fbc:geneProductAssociation>
                  <fbc:geneProductRef fbc:geneProduct="SAUSA300_0918"/>
            </fbc:geneProductAssociation>
      </reaction>

      <reaction metaid="R_ISOHEPO" id="R_ISOHEPO" name="isoheptadecanoyl-UDP-
glucosyltransferase monoglucosyl " reversible="true" fast="false"
fbc:lowerFluxBound="rev_lb" fbc:upperFluxBound="rev_ub">
            <listOfReactants>
                  <speciesReference species="M_udpg_c" stoichiometry="1"
constant="true"/>
                  <speciesReference species="M_12diisgly_c" stoichiometry="1"
constant="true"/>
            </listOfReactants>
            <listOfProducts>
                  <speciesReference species="M_udp_c" stoichiometry="1"
constant="true"/>
                  <speciesReference species="M_m12dhdglyc_c"
stoichiometry="1" constant="true"/>
            </listOfProducts>
            <fbc:geneProductAssociation>
                  <fbc:geneProductRef fbc:geneProduct="SAUSA300_0918"/>
            </fbc:geneProductAssociation>
      </reaction>

      <reaction metaid="R_ISOHEPT" id="R_ISOHEPT"
name="isoheptadecanoyl__Lipoteichoic acid synthesis n=24  unlinked  D-alanine
substituted " reversible="false" fast="false" fbc:lowerFluxBound="irr_lb"
fbc:upperFluxBound="irr_ub">
            <listOfReactants>
                  <speciesReference species="M_atp_c" stoichiometry="24"
constant="true"/>
                  <speciesReference species="M_ala__D_c" stoichiometry="24"
constant="true"/>
                  <speciesReference species="M_isolp24u_c" stoichiometry="1"
constant="true"/>
            </listOfReactants>
            <listOfProducts>
                  <speciesReference species="M_ppi_c" stoichiometry="24"
constant="true"/>
                  <speciesReference species="M_amp_c" stoichiometry="24"
constant="true"/>
```

```
                  <speciesReference species="M_iso24ds_c" stoichiometry="1"
constant="true"/>
            </listOfProducts>
            <fbc:geneProductAssociation>
                  <fbc:geneProductRef fbc:geneProduct="SAUSA300_0838"/>
            </fbc:geneProductAssociation>
      </reaction>

      <reaction metaid="R_ISOHEXDK" id="R_ISOHEXDK"
name="isohexadecanoyl__Diacylglycerol kinase " reversible="false"
fast="false" fbc:lowerFluxBound="irr_lb" fbc:upperFluxBound="irr_ub">
            <listOfReactants>
                  <speciesReference species="M_atp_c" stoichiometry="1"
constant="true"/>
                  <speciesReference species="M_12dihexsgly_c"
stoichiometry="1" constant="true"/>
            </listOfReactants>
            <listOfProducts>
                  <speciesReference species="M_adp_c" stoichiometry="1"
constant="true"/>
                  <speciesReference species="M_12dhsgly3p_c"
stoichiometry="1" constant="true"/>
            </listOfProducts>
            <fbc:geneProductAssociation>
                  <fbc:geneProductRef fbc:geneProduct="SAUSA300_1529"/>
            </fbc:geneProductAssociation>
      </reaction>

      <reaction metaid="R_ISOHG3PO" id="R_ISOHG3PO" name="isoheptadecanoyl-
glycerol-3-phosphate O-acyltransferase " reversible="false" fast="false"
fbc:lowerFluxBound="irr_lb" fbc:upperFluxBound="irr_ub">
            <listOfReactants>
                  <speciesReference species="M_glyc3p_c" stoichiometry="1"
constant="true"/>
                  <speciesReference species="M_fa11coa_c" stoichiometry="1"
constant="true"/>
            </listOfReactants>
            <listOfProducts>
                  <speciesReference species="M_coa_c" stoichiometry="1"
constant="true"/>
                  <speciesReference species="M_1ihsg3p_c" stoichiometry="1"
constant="true"/>
            </listOfProducts>
            <fbc:geneProductAssociation>
                  <fbc:and>
                        <fbc:geneProductRef fbc:geneProduct="SAUSA300_1249"/>
                        <fbc:or>
                              <fbc:geneProductRef
fbc:geneProduct="SAUSA300_1122"/>
                              <fbc:geneProductRef
fbc:geneProduct="SAUSA300_1121"/>
                        </fbc:or>
                  </fbc:and>
            </fbc:geneProductAssociation>
      </reaction>

      <reaction metaid="R_ISOHGL3AS" id="R_ISOHGL3AS"
name="anteisopentadecanoyl__Lipoteichoic acid synthesis n=24  linked  N-
```

```
acetylglucosamine substituted " reversible="true" fast="false"
fbc:lowerFluxBound="rev_lb" fbc:upperFluxBound="rev_ub">
            <listOfReactants>
                  <speciesReference species="M_uacgam_c" stoichiometry="24"
constant="true"/>
                  <speciesReference species="M_antp24u_c" stoichiometry="1"
constant="true"/>
            </listOfReactants>
            <listOfProducts>
                  <speciesReference species="M_udp_c" stoichiometry="24"
constant="true"/>
                  <speciesReference species="M_antp24s_c" stoichiometry="1"
constant="true"/>
            </listOfProducts>
            <fbc:geneProductAssociation>
                  <fbc:geneProductRef fbc:geneProduct="SAUSA300_0731"/>
            </fbc:geneProductAssociation>
      </reaction>

      <reaction metaid="R_ISOHGLT" id="R_ISOHGLT" name="isohexadecanoyl-UDP-
glucosyltransferase diglucosyl " reversible="true" fast="false"
fbc:lowerFluxBound="rev_lb" fbc:upperFluxBound="rev_ub">
            <listOfReactants>
                  <speciesReference species="M_udpg_c" stoichiometry="1"
constant="true"/>
                  <speciesReference species="M_m12diihgly_c"
stoichiometry="1" constant="true"/>
            </listOfReactants>
            <listOfProducts>
                  <speciesReference species="M_udp_c" stoichiometry="1"
constant="true"/>
                  <speciesReference species="M_dglu12dgly_c"
stoichiometry="1" constant="true"/>
            </listOfProducts>
            <fbc:geneProductAssociation>
                  <fbc:geneProductRef fbc:geneProduct="SAUSA300_0918"/>
            </fbc:geneProductAssociation>
      </reaction>

      <reaction metaid="R_ISOP3PT" id="R_ISOP3PT" name="Butyro-betaine ABC
transport " reversible="false" fast="false" fbc:lowerFluxBound="irr_lb"
fbc:upperFluxBound="irr_ub">
            <listOfReactants>
                  <speciesReference species="M_h2o_c" stoichiometry="1"
constant="true"/>
                  <speciesReference species="M_atp_c" stoichiometry="1"
constant="true"/>
                  <speciesReference species="M_btbet_e" stoichiometry="1"
constant="true"/>
            </listOfReactants>
            <listOfProducts>
                  <speciesReference species="M_h_c" stoichiometry="1"
constant="true"/>
                  <speciesReference species="M_pi_c" stoichiometry="1"
constant="true"/>
                  <speciesReference species="M_adp_c" stoichiometry="1"
constant="true"/>
```

```
                  <speciesReference species="M_btbet_c" stoichiometry="1"
constant="true"/>
            </listOfProducts>
            <fbc:geneProductAssociation>
                  <fbc:geneProductRef fbc:geneProduct="SAUSA300_0706"/>
            </fbc:geneProductAssociation>
      </reaction>

      <reaction metaid="R_ISOPCG3PT" id="R_ISOPCG3PT" name="isopentadecanoyl-
CDPdiacylglycerol sn-glycerol-3-phosphate 3-phosphatidyltransferase "
reversible="true" fast="false" fbc:lowerFluxBound="rev_lb"
fbc:upperFluxBound="rev_ub">
            <listOfReactants>
                  <speciesReference species="M_glyc3p_c" stoichiometry="1"
constant="true"/>
                  <speciesReference species="M_c12dipgly_c" stoichiometry="1"
constant="true"/>
            </listOfReactants>
            <listOfProducts>
                  <speciesReference species="M_cmp_c" stoichiometry="1"
constant="true"/>
                  <speciesReference species="M_diippgp_c" stoichiometry="1"
constant="true"/>
            </listOfProducts>
            <fbc:geneProductAssociation>
                  <fbc:geneProductRef fbc:geneProduct="SAUSA300_1176"/>
            </fbc:geneProductAssociation>
      </reaction>

      <reaction metaid="R_ISOPENM" id="R_ISOPENM" name="isopentadecanoyl-UDP-
glucosyltransferase monoglucosyl " reversible="true" fast="false"
fbc:lowerFluxBound="rev_lb" fbc:upperFluxBound="rev_ub">
            <listOfReactants>
                  <speciesReference species="M_udpg_c" stoichiometry="1"
constant="true"/>
                  <speciesReference species="M_12dpgly_c" stoichiometry="1"
constant="true"/>
            </listOfReactants>
            <listOfProducts>
                  <speciesReference species="M_udp_c" stoichiometry="1"
constant="true"/>
                  <speciesReference species="M_m12dipgly_c" stoichiometry="1"
constant="true"/>
            </listOfProducts>
            <fbc:geneProductAssociation>
                  <fbc:geneProductRef fbc:geneProduct="SAUSA300_0918"/>
            </fbc:geneProductAssociation>
      </reaction>

      <reaction metaid="R_ISOPLSNS" id="R_ISOPLSNS"
name="isopentadecanoyl__Lipoteichoic acid synthesis n=24  linked  N-
acetylglucosamine substituted " reversible="true" fast="false"
fbc:lowerFluxBound="rev_lb" fbc:upperFluxBound="rev_ub">
            <listOfReactants>
                  <speciesReference species="M_uacgam_c" stoichiometry="24"
constant="true"/>
                  <speciesReference species="M_isop24u_c" stoichiometry="1"
constant="true"/>
```

```
            </listOfReactants>
            <listOfProducts>
                  <speciesReference species="M_udp_c" stoichiometry="24"
constant="true"/>
                  <speciesReference species="M_isop24gsms_c"
stoichiometry="1" constant="true"/>
            </listOfProducts>
            <fbc:geneProductAssociation>
                  <fbc:geneProductRef fbc:geneProduct="SAUSA300_0731"/>
            </fbc:geneProductAssociation>
      </reaction>

      <reaction metaid="R_ISOPPCT" id="R_ISOPPCT" name="isopentadecanoyl-
phosphatidate cytidylyltransferase " reversible="true" fast="false"
fbc:lowerFluxBound="rev_lb" fbc:upperFluxBound="rev_ub">
            <listOfReactants>
                  <speciesReference species="M_ctp_c" stoichiometry="1"
constant="true"/>
                  <speciesReference species="M_12dpsgly3p_c"
stoichiometry="1" constant="true"/>
            </listOfReactants>
            <listOfProducts>
                  <speciesReference species="M_ppi_c" stoichiometry="1"
constant="true"/>
                  <speciesReference species="M_c12dipgly_c" stoichiometry="1"
constant="true"/>
            </listOfProducts>
            <fbc:geneProductAssociation>
                  <fbc:geneProductRef fbc:geneProduct="SAUSA300_1154"/>
            </fbc:geneProductAssociation>
      </reaction>

      <reaction metaid="R_ISOTCARS" id="R_ISOTCARS" name="isotetradecanoyl-
cardiolipin synthase " reversible="true" fast="false"
fbc:lowerFluxBound="rev_lb" fbc:upperFluxBound="rev_ub">
            <listOfReactants>
                  <speciesReference species="M_diitpgly_c" stoichiometry="2"
constant="true"/>
            </listOfReactants>
            <listOfProducts>
                  <speciesReference species="M_glyc_c" stoichiometry="1"
constant="true"/>
                  <speciesReference species="M_isotcard_c" stoichiometry="1"
constant="true"/>
            </listOfProducts>
            <fbc:geneProductAssociation>
                  <fbc:or>
                        <fbc:geneProductRef fbc:geneProduct="SAUSA300_2044"/>
                        <fbc:geneProductRef fbc:geneProduct="SAUSA300_1216"/>
                  </fbc:or>
            </fbc:geneProductAssociation>
      </reaction>

      <reaction metaid="R_ISOTETT" id="R_ISOTETT" name="isotetradecanoyl-1-
acylglycerol-3-phosphate O-acyltransferase " reversible="false" fast="false"
fbc:lowerFluxBound="irr_lb" fbc:upperFluxBound="irr_ub">
            <listOfReactants>
```

```
                  <speciesReference species="M_1stsg3p_c" stoichiometry="1"
constant="true"/>
                  <speciesReference species="M_fa1coa_c" stoichiometry="1"
constant="true"/>
            </listOfReactants>
            <listOfProducts>
                  <speciesReference species="M_coa_c" stoichiometry="1"
constant="true"/>
                  <speciesReference species="M_12diidgly3p_c"
stoichiometry="1" constant="true"/>
            </listOfProducts>
            <fbc:geneProductAssociation>
                  <fbc:geneProductRef fbc:geneProduct="SAUSA300_1673"/>
            </fbc:geneProductAssociation>
      </reaction>

      <reaction metaid="R_ISOTG3PT" id="R_ISOTG3PT" name="isotetradecanoyl-
CDPdiacylglycerol sn-glycerol-3-phosphate 3-phosphatidyltransferase "
reversible="true" fast="false" fbc:lowerFluxBound="rev_lb"
fbc:upperFluxBound="rev_ub">
            <listOfReactants>
                  <speciesReference species="M_glyc3p_c" stoichiometry="1"
constant="true"/>
                  <speciesReference species="M_c12ditgly_c" stoichiometry="1"
constant="true"/>
            </listOfReactants>
            <listOfProducts>
                  <speciesReference species="M_cmp_c" stoichiometry="1"
constant="true"/>
                  <speciesReference species="M_diidpgp_c" stoichiometry="1"
constant="true"/>
            </listOfProducts>
            <fbc:geneProductAssociation>
                  <fbc:geneProductRef fbc:geneProduct="SAUSA300_1176"/>
            </fbc:geneProductAssociation>
      </reaction>

      <reaction metaid="R_ISPDK" id="R_ISPDK"
name="isopentadecanoyl__Diacylglycerol kinase " reversible="false"
fast="false" fbc:lowerFluxBound="irr_lb" fbc:upperFluxBound="irr_ub">
            <listOfReactants>
                  <speciesReference species="M_atp_c" stoichiometry="1"
constant="true"/>
                  <speciesReference species="M_12dpgly_c" stoichiometry="1"
constant="true"/>
            </listOfReactants>
            <listOfProducts>
                  <speciesReference species="M_adp_c" stoichiometry="1"
constant="true"/>
                  <speciesReference species="M_12dpsgly3p_c"
stoichiometry="1" constant="true"/>
            </listOfProducts>
            <fbc:geneProductAssociation>
                  <fbc:geneProductRef fbc:geneProduct="SAUSA300_1529"/>
            </fbc:geneProductAssociation>
      </reaction>
```

```
      <reaction metaid="R_ISPDUGL" id="R_ISPDUGL" name="isopentadecanoyl-UDP-
glucosyltransferase diglucosyl " reversible="true" fast="false"
fbc:lowerFluxBound="rev_lb" fbc:upperFluxBound="rev_ub">
            <listOfReactants>
                  <speciesReference species="M_udpg_c" stoichiometry="1"
constant="true"/>
                  <speciesReference species="M_m12dipgly_c" stoichiometry="1"
constant="true"/>
            </listOfReactants>
            <listOfProducts>
                  <speciesReference species="M_udp_c" stoichiometry="1"
constant="true"/>
                  <speciesReference species="M_dglu12dpg_c" stoichiometry="1"
constant="true"/>
            </listOfProducts>
            <fbc:geneProductAssociation>
                  <fbc:geneProductRef fbc:geneProduct="SAUSA300_0918"/>
            </fbc:geneProductAssociation>
      </reaction>

      <reaction metaid="R_ISTETUGLT" id="R_ISTETUGLT" name="isotetradecanoyl-
UDP-glucosyltransferase monoglucosyl " reversible="true" fast="false"
fbc:lowerFluxBound="rev_lb" fbc:upperFluxBound="rev_ub">
            <listOfReactants>
                  <speciesReference species="M_udpg_c" stoichiometry="1"
constant="true"/>
                  <speciesReference species="M_12ditetgly_c"
stoichiometry="1" constant="true"/>
            </listOfReactants>
            <listOfProducts>
                  <speciesReference species="M_udp_c" stoichiometry="1"
constant="true"/>
                  <speciesReference species="M_m12diidgly_c"
stoichiometry="1" constant="true"/>
            </listOfProducts>
            <fbc:geneProductAssociation>
                  <fbc:geneProductRef fbc:geneProduct="SAUSA300_0918"/>
            </fbc:geneProductAssociation>
      </reaction>

      <reaction metaid="R_ISTLALNA" id="R_ISTLALNA"
name="isotetradecanoyl__Lipoteichoic acid synthesis n=24  linked  N-
acetylglucosamine substituted " reversible="true" fast="false"
fbc:lowerFluxBound="rev_lb" fbc:upperFluxBound="rev_ub">
            <listOfReactants>
                  <speciesReference species="M_uacgam_c" stoichiometry="24"
constant="true"/>
                  <speciesReference species="M_isot24u_c" stoichiometry="1"
constant="true"/>
            </listOfReactants>
            <listOfProducts>
                  <speciesReference species="M_udp_c" stoichiometry="24"
constant="true"/>
                  <speciesReference species="M_isot24s_c" stoichiometry="1"
constant="true"/>
            </listOfProducts>
            <fbc:geneProductAssociation>
                  <fbc:geneProductRef fbc:geneProduct="SAUSA300_0731"/>
```

```
			</fbc:geneProductAssociation>
		</reaction>

		<reaction metaid="R_ISTPCYT" id="R_ISTPCYT" name="isotetradecanoyl-
phosphatidate cytidylyltransferase " reversible="true" fast="false"
fbc:lowerFluxBound="rev_lb" fbc:upperFluxBound="rev_ub">
			<listOfReactants>
				<speciesReference species="M_ctp_c" stoichiometry="1"
constant="true"/>
				<speciesReference species="M_12diidgly3p_c"
stoichiometry="1" constant="true"/>
			</listOfReactants>
			<listOfProducts>
				<speciesReference species="M_ppi_c" stoichiometry="1"
constant="true"/>
				<speciesReference species="M_c12ditgly_c" stoichiometry="1"
constant="true"/>
			</listOfProducts>
			<fbc:geneProductAssociation>
				<fbc:geneProductRef fbc:geneProduct="SAUSA300_1154"/>
			</fbc:geneProductAssociation>
		</reaction>

		<reaction metaid="R_ISTUG" id="R_ISTUG" name="isotetradecanoyl-UDP-
glucosyltransferase diglucosyl " reversible="true" fast="false"
fbc:lowerFluxBound="rev_lb" fbc:upperFluxBound="rev_ub">
			<listOfReactants>
				<speciesReference species="M_udpg_c" stoichiometry="1"
constant="true"/>
				<speciesReference species="M_m12diidgly_c"
stoichiometry="1" constant="true"/>
			</listOfReactants>
			<listOfProducts>
				<speciesReference species="M_udp_c" stoichiometry="1"
constant="true"/>
				<speciesReference species="M_dglu12dit_c" stoichiometry="1"
constant="true"/>
			</listOfProducts>
			<fbc:geneProductAssociation>
				<fbc:geneProductRef fbc:geneProduct="SAUSA300_0918"/>
			</fbc:geneProductAssociation>
		</reaction>

		<reaction metaid="R_ITG3PS" id="R_ITG3PS" name="isotetradecanoyl-
glycerol-3-phosphate O-acyltransferase " reversible="false" fast="false"
fbc:lowerFluxBound="irr_lb" fbc:upperFluxBound="irr_ub">
			<listOfReactants>
				<speciesReference species="M_glyc3p_c" stoichiometry="1"
constant="true"/>
				<speciesReference species="M_fa1coa_c" stoichiometry="1"
constant="true"/>
			</listOfReactants>
			<listOfProducts>
				<speciesReference species="M_coa_c" stoichiometry="1"
constant="true"/>
				<speciesReference species="M_1stsg3p_c" stoichiometry="1"
constant="true"/>
			</listOfProducts>
```

```
            <fbc:geneProductAssociation>
                  <fbc:and>
                        <fbc:geneProductRef fbc:geneProduct="SAUSA300_1249"/>
                        <fbc:or>
                              <fbc:geneProductRef
fbc:geneProduct="SAUSA300_1122"/>
                              <fbc:geneProductRef
fbc:geneProduct="SAUSA300_1121"/>
                        </fbc:or>
                  </fbc:and>
            </fbc:geneProductAssociation>
      </reaction>

      <reaction metaid="R_ITLAS" id="R_ITLAS"
name="isotetradecanoyl__Lipoteichoic acid synthesis n=24  linked  glucose
substituted " reversible="true" fast="false" fbc:lowerFluxBound="rev_lb"
fbc:upperFluxBound="rev_ub">
            <listOfReactants>
                  <speciesReference species="M_udpg_c" stoichiometry="24"
constant="true"/>
                  <speciesReference species="M_isot24u_c" stoichiometry="1"
constant="true"/>
            </listOfReactants>
            <listOfProducts>
                  <speciesReference species="M_udp_c" stoichiometry="24"
constant="true"/>
                  <speciesReference species="M_isod24s_c" stoichiometry="1"
constant="true"/>
            </listOfProducts>
            <fbc:geneProductAssociation>
                  <fbc:or>
                        <fbc:geneProductRef fbc:geneProduct="SAUSA300_0939"/>
                        <fbc:geneProductRef fbc:geneProduct="SAUSA300_0550"/>
                        <fbc:geneProductRef fbc:geneProduct="SAUSA300_0549"/>
                  </fbc:or>
            </fbc:geneProductAssociation>
      </reaction>

      <reaction metaid="R_IZPN" id="R_IZPN" name="imidazolonepropionase"
reversible="false" fast="false" fbc:lowerFluxBound="irr_lb"
fbc:upperFluxBound="irr_ub">
            <listOfReactants>
                  <speciesReference species="M_h2o_c" stoichiometry="1"
constant="true"/>
                  <speciesReference species="M_4izp_c" stoichiometry="1"
constant="true"/>
            </listOfReactants>
            <listOfProducts>
                  <speciesReference species="M_forglu_c" stoichiometry="1"
constant="true"/>
            </listOfProducts>
            <fbc:geneProductAssociation>
                  <fbc:geneProductRef fbc:geneProduct="SAUSA300_2277"/>
            </fbc:geneProductAssociation>
      </reaction>
```

```
      <reaction metaid="R_Kabc" id="R_Kabc" name="Potassium ABC transporter"
reversible="false" fast="false" fbc:lowerFluxBound="irr_lb"
fbc:upperFluxBound="irr_ub">
            <listOfReactants>
                  <speciesReference species="M_h2o_c" stoichiometry="1"
constant="true"/>
                  <speciesReference species="M_atp_c" stoichiometry="1"
constant="true"/>
                  <speciesReference species="M_k_e" stoichiometry="1"
constant="true"/>
            </listOfReactants>
            <listOfProducts>
                  <speciesReference species="M_h_c" stoichiometry="1"
constant="true"/>
                  <speciesReference species="M_pi_c" stoichiometry="1"
constant="true"/>
                  <speciesReference species="M_adp_c" stoichiometry="1"
constant="true"/>
                  <speciesReference species="M_k_c" stoichiometry="1"
constant="true"/>
            </listOfProducts>
            <fbc:geneProductAssociation>
                  <fbc:or>
                        <fbc:geneProductRef fbc:geneProduct="SAUSA300_2034"/>
                        <fbc:geneProductRef fbc:geneProduct="SAUSA300_2033"/>
                        <fbc:geneProductRef fbc:geneProduct="SAUSA300_2032"/>
                  </fbc:or>
            </fbc:geneProductAssociation>
      </reaction>

      <reaction metaid="R_KARA1" id="R_KARA1" name="ketol-acid
reductoisomerase (2,3__Dihydroxy-3-methylbutanoate)" reversible="false"
fast="false" fbc:lowerFluxBound="irr_lb" fbc:upperFluxBound="irr_ub">
            <listOfReactants>
                  <speciesReference species="M_h_c" stoichiometry="1"
constant="true"/>
                  <speciesReference species="M_nadph_c" stoichiometry="1"
constant="true"/>
                  <speciesReference species="M_alac__S_c" stoichiometry="1"
constant="true"/>
            </listOfReactants>
            <listOfProducts>
                  <speciesReference species="M_nadp_c" stoichiometry="1"
constant="true"/>
                  <speciesReference species="M_23dhmb_c" stoichiometry="1"
constant="true"/>
            </listOfProducts>
            <fbc:geneProductAssociation>
                  <fbc:geneProductRef fbc:geneProduct="SAUSA300_2009"/>
            </fbc:geneProductAssociation>
      </reaction>

      <reaction metaid="R_KARA1i" id="R_KARA1i" name="acetohydroxy acid
isomeroreductase" reversible="false" fast="false" fbc:lowerFluxBound="irr_lb"
fbc:upperFluxBound="irr_ub">
            <listOfReactants>
                  <speciesReference species="M_h_c" stoichiometry="1"
constant="true"/>
```

```
                  <speciesReference species="M_nadph_c" stoichiometry="1"
constant="true"/>
                  <speciesReference species="M_alac__S_c" stoichiometry="1"
constant="true"/>
            </listOfReactants>
            <listOfProducts>
                  <speciesReference species="M_nadp_c" stoichiometry="1"
constant="true"/>
                  <speciesReference species="M_23dhmb_c" stoichiometry="1"
constant="true"/>
            </listOfProducts>
            <fbc:geneProductAssociation>
                  <fbc:geneProductRef fbc:geneProduct="SAUSA300_2009"/>
            </fbc:geneProductAssociation>
      </reaction>

      <reaction metaid="R_KARA2i" id="R_KARA2i" name="ketol-acid
reductoisomerase (2-Aceto-2-hydroxybutanoate)" reversible="false"
fast="false" fbc:lowerFluxBound="irr_lb" fbc:upperFluxBound="irr_ub">
            <listOfReactants>
                  <speciesReference species="M_h_c" stoichiometry="1"
constant="true"/>
                  <speciesReference species="M_nadph_c" stoichiometry="1"
constant="true"/>
                  <speciesReference species="M_2ahbut_c" stoichiometry="1"
constant="true"/>
            </listOfReactants>
            <listOfProducts>
                  <speciesReference species="M_nadp_c" stoichiometry="1"
constant="true"/>
                  <speciesReference species="M_23dhmp_c" stoichiometry="1"
constant="true"/>
            </listOfProducts>
            <fbc:geneProductAssociation>
                  <fbc:geneProductRef fbc:geneProduct="SAUSA300_2009"/>
            </fbc:geneProductAssociation>
      </reaction>

      <reaction metaid="R_KARA3" id="R_KARA3" name="ketol-acid
reductoisomerase ((R)-2,3__Dihydroxy-3-methylbutanoate)" reversible="true"
fast="false" fbc:lowerFluxBound="rev_lb" fbc:upperFluxBound="rev_ub">
            <listOfReactants>
                  <speciesReference species="M_nadp_c" stoichiometry="1"
constant="true"/>
                  <speciesReference species="M_23dhmp_c" stoichiometry="1"
constant="true"/>
            </listOfReactants>
            <listOfProducts>
                  <speciesReference species="M_h_c" stoichiometry="1"
constant="true"/>
                  <speciesReference species="M_nadph_c" stoichiometry="1"
constant="true"/>
                  <speciesReference species="M_3h3mop_c" stoichiometry="1"
constant="true"/>
            </listOfProducts>
            <fbc:geneProductAssociation>
                  <fbc:geneProductRef fbc:geneProduct="SAUSA300_2009"/>
            </fbc:geneProductAssociation>
```

```
      </reaction>

      <reaction metaid="R_KARA4" id="R_KARA4" name="(R)-2,3__Dihydroxy-3-
methylbutanoate:NADP+ oxidoreductase" reversible="false" fast="false"
fbc:lowerFluxBound="irr_lb" fbc:upperFluxBound="irr_ub">
            <listOfReactants>
                  <speciesReference species="M_nadp_c" stoichiometry="1"
constant="true"/>
                  <speciesReference species="M_23dhmb_c" stoichiometry="1"
constant="true"/>
            </listOfReactants>
            <listOfProducts>
                  <speciesReference species="M_h_c" stoichiometry="1"
constant="true"/>
                  <speciesReference species="M_nadph_c" stoichiometry="1"
constant="true"/>
                  <speciesReference species="M_33hmeoxobut_c"
stoichiometry="1" constant="true"/>
            </listOfProducts>
            <fbc:geneProductAssociation>
                  <fbc:geneProductRef fbc:geneProduct="SAUSA300_2009"/>
            </fbc:geneProductAssociation>
      </reaction>

      <reaction metaid="R_KAS1" id="R_KAS1" name="b-ketoacyl synthetase (Iso-
C14:0)" reversible="false" fast="false" fbc:lowerFluxBound="irr_lb"
fbc:upperFluxBound="irr_ub">
            <listOfReactants>
                  <speciesReference species="M_h_c" stoichiometry="14"
constant="true"/>
                  <speciesReference species="M_nadph_c" stoichiometry="10"
constant="true"/>
                  <speciesReference species="M_ibcoa_c" stoichiometry="1"
constant="true"/>
                  <speciesReference species="M_malcoa_c" stoichiometry="5"
constant="true"/>
            </listOfReactants>
            <listOfProducts>
                  <speciesReference species="M_h2o_c" stoichiometry="4"
constant="true"/>
                  <speciesReference species="M_nadp_c" stoichiometry="10"
constant="true"/>
                  <speciesReference species="M_co2_c" stoichiometry="5"
constant="true"/>
                  <speciesReference species="M_coa_c" stoichiometry="6"
constant="true"/>
                  <speciesReference species="M_fa1_c" stoichiometry="1"
constant="true"/>
            </listOfProducts>
            <fbc:geneProductAssociation>
                  <fbc:and>
                        <fbc:geneProductRef fbc:geneProduct="FabH"/>
                        <fbc:geneProductRef fbc:geneProduct="SAUSA300_0886"/>
                  </fbc:and>
            </fbc:geneProductAssociation>
      </reaction>
```

```
	<reaction metaid="R_KAS10" id="R_KAS10" name="b-ketoacyl synthetase (Anteiso-C17:1)" reversible="false" fast="false" fbc:lowerFluxBound="irr_lb" fbc:upperFluxBound="irr_ub">
		<listOfReactants>
			<speciesReference species="M_h_c" stoichiometry="16" constant="true"/>
			<speciesReference species="M_nadph_c" stoichiometry="11" constant="true"/>
			<speciesReference species="M_2mbcoa_c" stoichiometry="1" constant="true"/>
			<speciesReference species="M_malcoa_c" stoichiometry="6" constant="true"/>
		</listOfReactants>
		<listOfProducts>
			<speciesReference species="M_h2o_c" stoichiometry="5" constant="true"/>
			<speciesReference species="M_nadp_c" stoichiometry="11" constant="true"/>
			<speciesReference species="M_co2_c" stoichiometry="6" constant="true"/>
			<speciesReference species="M_coa_c" stoichiometry="7" constant="true"/>
			<speciesReference species="M_fa10_c" stoichiometry="1" constant="true"/>
		</listOfProducts>
		<fbc:geneProductAssociation>
			<fbc:and>
				<fbc:geneProductRef fbc:geneProduct="FabH"/>
				<fbc:geneProductRef fbc:geneProduct="SAUSA300_0886"/>
			</fbc:and>
		</fbc:geneProductAssociation>
	</reaction>

	<reaction metaid="R_KAS11" id="R_KAS11" name="b-ketoacyl synthetase (Iso-C17:0)" reversible="false" fast="false" fbc:lowerFluxBound="irr_lb" fbc:upperFluxBound="irr_ub">
		<listOfReactants>
			<speciesReference species="M_h_c" stoichiometry="17" constant="true"/>
			<speciesReference species="M_nadph_c" stoichiometry="12" constant="true"/>
			<speciesReference species="M_ivcoa_c" stoichiometry="1" constant="true"/>
			<speciesReference species="M_malcoa_c" stoichiometry="6" constant="true"/>
		</listOfReactants>
		<listOfProducts>
			<speciesReference species="M_h2o_c" stoichiometry="5" constant="true"/>
			<speciesReference species="M_nadp_c" stoichiometry="12" constant="true"/>
			<speciesReference species="M_co2_c" stoichiometry="6" constant="true"/>
			<speciesReference species="M_coa_c" stoichiometry="7" constant="true"/>
			<speciesReference species="M_fa11_c" stoichiometry="1" constant="true"/>
		</listOfProducts>
```

```
            <fbc:geneProductAssociation>
                  <fbc:and>
                        <fbc:geneProductRef fbc:geneProduct="FabH"/>
                        <fbc:geneProductRef fbc:geneProduct="SAUSA300_0886"/>
                  </fbc:and>
            </fbc:geneProductAssociation>
      </reaction>

      <reaction metaid="R_KAS12" id="R_KAS12" name="b-ketoacyl synthetase
(Anteiso-C17:0)" reversible="false" fast="false" fbc:lowerFluxBound="irr_lb"
fbc:upperFluxBound="irr_ub">
            <listOfReactants>
                  <speciesReference species="M_h_c" stoichiometry="17"
constant="true"/>
                  <speciesReference species="M_nadph_c" stoichiometry="12"
constant="true"/>
                  <speciesReference species="M_2mbcoa_c" stoichiometry="1"
constant="true"/>
                  <speciesReference species="M_malcoa_c" stoichiometry="6"
constant="true"/>
            </listOfReactants>
            <listOfProducts>
                  <speciesReference species="M_h2o_c" stoichiometry="5"
constant="true"/>
                  <speciesReference species="M_nadp_c" stoichiometry="12"
constant="true"/>
                  <speciesReference species="M_co2_c" stoichiometry="6"
constant="true"/>
                  <speciesReference species="M_coa_c" stoichiometry="7"
constant="true"/>
                  <speciesReference species="M_fa12_c" stoichiometry="1"
constant="true"/>
            </listOfProducts>
            <fbc:geneProductAssociation>
                  <fbc:and>
                        <fbc:geneProductRef fbc:geneProduct="FabH"/>
                        <fbc:geneProductRef fbc:geneProduct="SAUSA300_0886"/>
                  </fbc:and>
            </fbc:geneProductAssociation>
      </reaction>

      <reaction metaid="R_KAS13" id="R_KAS13" name="b-ketoacyl synthetase
(octadecanoate)" reversible="false" fast="false" fbc:lowerFluxBound="irr_lb"
fbc:upperFluxBound="irr_ub">
            <listOfReactants>
                  <speciesReference species="M_h_c" stoichiometry="23"
constant="true"/>
                  <speciesReference species="M_nadph_c" stoichiometry="16"
constant="true"/>
                  <speciesReference species="M_accoa_c" stoichiometry="1"
constant="true"/>
                  <speciesReference species="M_malcoa_c" stoichiometry="8"
constant="true"/>
            </listOfReactants>
            <listOfProducts>
                  <speciesReference species="M_h2o_c" stoichiometry="7"
constant="true"/>
```

```
                  <speciesReference species="M_nadp_c" stoichiometry="16"
constant="true"/>
                  <speciesReference species="M_co2_c" stoichiometry="8"
constant="true"/>
                  <speciesReference species="M_coa_c" stoichiometry="9"
constant="true"/>
                  <speciesReference species="M_ocdca_c" stoichiometry="1"
constant="true"/>
            </listOfProducts>
            <fbc:geneProductAssociation>
                  <fbc:and>
                        <fbc:geneProductRef fbc:geneProduct="FabH"/>
                        <fbc:geneProductRef fbc:geneProduct="SAUSA300_0886"/>
                  </fbc:and>
            </fbc:geneProductAssociation>
      </reaction>

      <reaction metaid="R_KAS14" id="R_KAS14" name="beta-ketoacyl-ACP
synthase" reversible="false" fast="false" fbc:lowerFluxBound="irr_lb"
fbc:upperFluxBound="irr_ub">
            <listOfReactants>
                  <speciesReference species="M_h_c" stoichiometry="1"
constant="true"/>
                  <speciesReference species="M_malACP_c" stoichiometry="1"
constant="true"/>
                  <speciesReference species="M_acACP_c" stoichiometry="1"
constant="true"/>
            </listOfReactants>
            <listOfProducts>
                  <speciesReference species="M_ACP_c" stoichiometry="1"
constant="true"/>
                  <speciesReference species="M_co2_c" stoichiometry="1"
constant="true"/>
                  <speciesReference species="M_actACP_c" stoichiometry="1"
constant="true"/>
            </listOfProducts>
            <fbc:geneProductAssociation>
                  <fbc:and>
                        <fbc:geneProductRef fbc:geneProduct="SAUSA300_0886"/>
                        <fbc:geneProductRef fbc:geneProduct="SAUSA300_0885"/>
                  </fbc:and>
            </fbc:geneProductAssociation>
      </reaction>

      <reaction metaid="R_KAS15" id="R_KAS15" name="beta-ketoacyl-ACP
synthase (2)" reversible="false" fast="false" fbc:lowerFluxBound="irr_lb"
fbc:upperFluxBound="irr_ub">
            <listOfReactants>
                  <speciesReference species="M_h_c" stoichiometry="1"
constant="true"/>
                  <speciesReference species="M_malACP_c" stoichiometry="1"
constant="true"/>
                  <speciesReference species="M_accoa_c" stoichiometry="1"
constant="true"/>
            </listOfReactants>
            <listOfProducts>
                  <speciesReference species="M_co2_c" stoichiometry="1"
constant="true"/>
```

```
                  <speciesReference species="M_coa_c" stoichiometry="1"
constant="true"/>
                  <speciesReference species="M_actACP_c" stoichiometry="1"
constant="true"/>
            </listOfProducts>
            <fbc:geneProductAssociation>
                  <fbc:and>
                        <fbc:geneProductRef fbc:geneProduct="FabH"/>
                        <fbc:geneProductRef fbc:geneProduct="SAUSA300_0886"/>
                  </fbc:and>
            </fbc:geneProductAssociation>
      </reaction>

      <reaction metaid="R_KAS16" id="R_KAS16" name="3-hydroxy-myristoyl-ACP
synthesis" reversible="false" fast="false" fbc:lowerFluxBound="irr_lb"
fbc:upperFluxBound="irr_ub">
            <listOfReactants>
                  <speciesReference species="M_h_c" stoichiometry="2"
constant="true"/>
                  <speciesReference species="M_nadph_c" stoichiometry="1"
constant="true"/>
                  <speciesReference species="M_malACP_c" stoichiometry="1"
constant="true"/>
                  <speciesReference species="M_ddcaACP_c" stoichiometry="1"
constant="true"/>
            </listOfReactants>
            <listOfProducts>
                  <speciesReference species="M_nadp_c" stoichiometry="1"
constant="true"/>
                  <speciesReference species="M_ACP_c" stoichiometry="1"
constant="true"/>
                  <speciesReference species="M_co2_c" stoichiometry="1"
constant="true"/>
                  <speciesReference species="M_3hmrsACP_c" stoichiometry="1"
constant="true"/>
            </listOfProducts>
            <fbc:geneProductAssociation>
                  <fbc:and>
                        <fbc:or>
                              <fbc:geneProductRef
fbc:geneProduct="SAUSA300_2416"/>
                              <fbc:geneProductRef fbc:geneProduct="fabG"/>
                        </fbc:or>
                        <fbc:geneProductRef fbc:geneProduct="FabH"/>
                        <fbc:geneProductRef fbc:geneProduct="SAUSA300_0886"/>
                  </fbc:and>
            </fbc:geneProductAssociation>
      </reaction>

      <reaction metaid="R_KAS17" id="R_KAS17" name="b-ketoacyl synthetase (n-
C18:1)" reversible="false" fast="false" fbc:lowerFluxBound="irr_lb"
fbc:upperFluxBound="irr_ub">
            <listOfReactants>
                  <speciesReference species="M_h_c" stoichiometry="22"
constant="true"/>
                  <speciesReference species="M_nadph_c" stoichiometry="15"
constant="true"/>
```

```
                  <speciesReference species="M_accoa_c" stoichiometry="1"
constant="true"/>
                  <speciesReference species="M_malcoa_c" stoichiometry="8"
constant="true"/>
            &lt;/listOfReactants&gt;
            &lt;listOfProducts&gt;
                  <speciesReference species="M_h2o_c" stoichiometry="7"
constant="true"/>
                  <speciesReference species="M_nadp_c" stoichiometry="15"
constant="true"/>
                  <speciesReference species="M_co2_c" stoichiometry="8"
constant="true"/>
                  <speciesReference species="M_coa_c" stoichiometry="9"
constant="true"/>
                  <speciesReference species="M_ocdcea_c" stoichiometry="1"
constant="true"/>
            &lt;/listOfProducts&gt;
            &lt;fbc:geneProductAssociation&gt;
                  &lt;fbc:and&gt;
                        &lt;fbc:geneProductRef fbc:geneProduct="FabH"/&gt;
                        &lt;fbc:geneProductRef fbc:geneProduct="SAUSA300_0886"/&gt;
                  &lt;/fbc:and&gt;
            &lt;/fbc:geneProductAssociation&gt;
      &lt;/reaction&gt;

      <reaction metaid="R_KAS19SA" id="R_KAS19SA" name="b-ketoacyl synthetase
(Anteiso-C19:0)" reversible="false" fast="false" fbc:lowerFluxBound="irr_lb"
fbc:upperFluxBound="irr_ub">
            &lt;listOfReactants&gt;
                  <speciesReference species="M_h_c" stoichiometry="20"
constant="true"/>
                  <speciesReference species="M_nadph_c" stoichiometry="14"
constant="true"/>
                  <speciesReference species="M_2mbcoa_c" stoichiometry="1"
constant="true"/>
                  <speciesReference species="M_malcoa_c" stoichiometry="7"
constant="true"/>
            &lt;/listOfReactants&gt;
            &lt;listOfProducts&gt;
                  <speciesReference species="M_h2o_c" stoichiometry="6"
constant="true"/>
                  <speciesReference species="M_nadp_c" stoichiometry="14"
constant="true"/>
                  <speciesReference species="M_co2_c" stoichiometry="7"
constant="true"/>
                  <speciesReference species="M_coa_c" stoichiometry="8"
constant="true"/>
                  <speciesReference species="M_fa19a_c" stoichiometry="1"
constant="true"/>
            &lt;/listOfProducts&gt;
            &lt;fbc:geneProductAssociation&gt;
                  &lt;fbc:and&gt;
                        &lt;fbc:geneProductRef fbc:geneProduct="FabH"/&gt;
                        &lt;fbc:geneProductRef fbc:geneProduct="SAUSA300_0886"/&gt;
                  &lt;/fbc:and&gt;
            &lt;/fbc:geneProductAssociation&gt;
      &lt;/reaction&gt;
```

```
      <reaction metaid="R_KAS2" id="R_KAS2" name="b-ketoacyl synthetase (n-C14:0)" reversible="false" fast="false" fbc:lowerFluxBound="irr_lb" fbc:upperFluxBound="irr_ub">
            <listOfReactants>
                  <speciesReference species="M_h_c" stoichiometry="17" constant="true"/>
                  <speciesReference species="M_nadph_c" stoichiometry="12" constant="true"/>
                  <speciesReference species="M_accoa_c" stoichiometry="1" constant="true"/>
                  <speciesReference species="M_malcoa_c" stoichiometry="6" constant="true"/>
            </listOfReactants>
            <listOfProducts>
                  <speciesReference species="M_h2o_c" stoichiometry="5" constant="true"/>
                  <speciesReference species="M_nadp_c" stoichiometry="12" constant="true"/>
                  <speciesReference species="M_co2_c" stoichiometry="6" constant="true"/>
                  <speciesReference species="M_coa_c" stoichiometry="7" constant="true"/>
                  <speciesReference species="M_ttdca_c" stoichiometry="1" constant="true"/>
            </listOfProducts>
            <fbc:geneProductAssociation>
                  <fbc:and>
                        <fbc:geneProductRef fbc:geneProduct="FabH"/>
                        <fbc:geneProductRef fbc:geneProduct="SAUSA300_0886"/>
                  </fbc:and>
            </fbc:geneProductAssociation>
      </reaction>

      <reaction metaid="R_KAS20SA" id="R_KAS20SA" name="b-ketoacyl synthetase (C20:0)" reversible="false" fast="false" fbc:lowerFluxBound="irr_lb" fbc:upperFluxBound="irr_ub">
            <listOfReactants>
                  <speciesReference species="M_h_c" stoichiometry="26" constant="true"/>
                  <speciesReference species="M_nadph_c" stoichiometry="18" constant="true"/>
                  <speciesReference species="M_accoa_c" stoichiometry="1" constant="true"/>
                  <speciesReference species="M_malcoa_c" stoichiometry="9" constant="true"/>
            </listOfReactants>
            <listOfProducts>
                  <speciesReference species="M_h2o_c" stoichiometry="8" constant="true"/>
                  <speciesReference species="M_nadp_c" stoichiometry="18" constant="true"/>
                  <speciesReference species="M_co2_c" stoichiometry="9" constant="true"/>
                  <speciesReference species="M_coa_c" stoichiometry="10" constant="true"/>
                  <speciesReference species="M_fa20n_c" stoichiometry="1" constant="true"/>
            </listOfProducts>
```

```
            <fbc:geneProductAssociation>
                  <fbc:and>
                        <fbc:geneProductRef fbc:geneProduct="FabH"/>
                        <fbc:geneProductRef fbc:geneProduct="SAUSA300_0886"/>
                  </fbc:and>
            </fbc:geneProductAssociation>
      </reaction>

      <reaction metaid="R_KAS3" id="R_KAS3" name="b-ketoacyl synthetase (Iso-
C15:0)" reversible="false" fast="false" fbc:lowerFluxBound="irr_lb"
fbc:upperFluxBound="irr_ub">
            <listOfReactants>
                  <speciesReference species="M_h_c" stoichiometry="14"
constant="true"/>
                  <speciesReference species="M_nadph_c" stoichiometry="10"
constant="true"/>
                  <speciesReference species="M_ivcoa_c" stoichiometry="1"
constant="true"/>
                  <speciesReference species="M_malcoa_c" stoichiometry="5"
constant="true"/>
            </listOfReactants>
            <listOfProducts>
                  <speciesReference species="M_h2o_c" stoichiometry="4"
constant="true"/>
                  <speciesReference species="M_nadp_c" stoichiometry="10"
constant="true"/>
                  <speciesReference species="M_co2_c" stoichiometry="5"
constant="true"/>
                  <speciesReference species="M_coa_c" stoichiometry="6"
constant="true"/>
                  <speciesReference species="M_fa3_c" stoichiometry="1"
constant="true"/>
            </listOfProducts>
            <fbc:geneProductAssociation>
                  <fbc:and>
                        <fbc:geneProductRef fbc:geneProduct="FabH"/>
                        <fbc:geneProductRef fbc:geneProduct="SAUSA300_0886"/>
                  </fbc:and>
            </fbc:geneProductAssociation>
      </reaction>

      <reaction metaid="R_KAS4" id="R_KAS4" name="b-ketoacyl synthetase
(Anteiso-C15:0)" reversible="false" fast="false" fbc:lowerFluxBound="irr_lb"
fbc:upperFluxBound="irr_ub">
            <listOfReactants>
                  <speciesReference species="M_h_c" stoichiometry="14"
constant="true"/>
                  <speciesReference species="M_nadph_c" stoichiometry="10"
constant="true"/>
                  <speciesReference species="M_2mbcoa_c" stoichiometry="1"
constant="true"/>
                  <speciesReference species="M_malcoa_c" stoichiometry="5"
constant="true"/>
            </listOfReactants>
            <listOfProducts>
                  <speciesReference species="M_h2o_c" stoichiometry="4"
constant="true"/>
```

```
                    <speciesReference species="M_nadp_c" stoichiometry="10"
constant="true"/>
                    <speciesReference species="M_co2_c" stoichiometry="5"
constant="true"/>
                    <speciesReference species="M_coa_c" stoichiometry="6"
constant="true"/>
                    <speciesReference species="M_fa4_c" stoichiometry="1"
constant="true"/>
              </listOfProducts>
              <fbc:geneProductAssociation>
                    <fbc:and>
                          <fbc:geneProductRef fbc:geneProduct="FabH"/>
                          <fbc:geneProductRef fbc:geneProduct="SAUSA300_0886"/>
                    </fbc:and>
              </fbc:geneProductAssociation>
        </reaction>

        <reaction metaid="R_KAS5" id="R_KAS5" name="b-ketoacyl synthetase (Iso-
C16:1)" reversible="false" fast="false" fbc:lowerFluxBound="irr_lb"
fbc:upperFluxBound="irr_ub">
              <listOfReactants>
                    <speciesReference species="M_h_c" stoichiometry="16"
constant="true"/>
                    <speciesReference species="M_nadph_c" stoichiometry="11"
constant="true"/>
                    <speciesReference species="M_ibcoa_c" stoichiometry="1"
constant="true"/>
                    <speciesReference species="M_malcoa_c" stoichiometry="6"
constant="true"/>
              </listOfReactants>
              <listOfProducts>
                    <speciesReference species="M_h2o_c" stoichiometry="5"
constant="true"/>
                    <speciesReference species="M_nadp_c" stoichiometry="11"
constant="true"/>
                    <speciesReference species="M_co2_c" stoichiometry="6"
constant="true"/>
                    <speciesReference species="M_coa_c" stoichiometry="7"
constant="true"/>
                    <speciesReference species="M_fa5_c" stoichiometry="1"
constant="true"/>
              </listOfProducts>
              <fbc:geneProductAssociation>
                    <fbc:and>
                          <fbc:geneProductRef fbc:geneProduct="FabH"/>
                          <fbc:geneProductRef fbc:geneProduct="SAUSA300_0886"/>
                    </fbc:and>
              </fbc:geneProductAssociation>
        </reaction>

        <reaction metaid="R_KAS6" id="R_KAS6" name="b-ketoacyl synthetase (Iso-
C16:0)" reversible="false" fast="false" fbc:lowerFluxBound="irr_lb"
fbc:upperFluxBound="irr_ub">
              <listOfReactants>
                    <speciesReference species="M_h_c" stoichiometry="17"
constant="true"/>
                    <speciesReference species="M_nadph_c" stoichiometry="12"
constant="true"/>
```

```
                  <speciesReference species="M_ibcoa_c" stoichiometry="1"
constant="true"/>
                  <speciesReference species="M_malcoa_c" stoichiometry="6"
constant="true"/>
            </listOfReactants>
            <listOfProducts>
                  <speciesReference species="M_h2o_c" stoichiometry="5"
constant="true"/>
                  <speciesReference species="M_nadp_c" stoichiometry="12"
constant="true"/>
                  <speciesReference species="M_co2_c" stoichiometry="6"
constant="true"/>
                  <speciesReference species="M_coa_c" stoichiometry="7"
constant="true"/>
                  <speciesReference species="M_fa6_c" stoichiometry="1"
constant="true"/>
            </listOfProducts>
            <fbc:geneProductAssociation>
                  <fbc:and>
                        <fbc:geneProductRef fbc:geneProduct="FabH"/>
                        <fbc:geneProductRef fbc:geneProduct="SAUSA300_0886"/>
                  </fbc:and>
            </fbc:geneProductAssociation>
      </reaction>

      <reaction metaid="R_KAS7" id="R_KAS7" name="b-ketoacyl synthetase (n-
C16:1)" reversible="false" fast="false" fbc:lowerFluxBound="irr_lb"
fbc:upperFluxBound="irr_ub">
            <listOfReactants>
                  <speciesReference species="M_h_c" stoichiometry="19"
constant="true"/>
                  <speciesReference species="M_nadph_c" stoichiometry="13"
constant="true"/>
                  <speciesReference species="M_accoa_c" stoichiometry="1"
constant="true"/>
                  <speciesReference species="M_malcoa_c" stoichiometry="7"
constant="true"/>
            </listOfReactants>
            <listOfProducts>
                  <speciesReference species="M_h2o_c" stoichiometry="6"
constant="true"/>
                  <speciesReference species="M_nadp_c" stoichiometry="13"
constant="true"/>
                  <speciesReference species="M_co2_c" stoichiometry="7"
constant="true"/>
                  <speciesReference species="M_coa_c" stoichiometry="8"
constant="true"/>
                  <speciesReference species="M_hdcea_c" stoichiometry="1"
constant="true"/>
            </listOfProducts>
            <fbc:geneProductAssociation>
                  <fbc:and>
                        <fbc:geneProductRef fbc:geneProduct="FabH"/>
                        <fbc:geneProductRef fbc:geneProduct="SAUSA300_0886"/>
                  </fbc:and>
            </fbc:geneProductAssociation>
      </reaction>
```

```
      <reaction metaid="R_KAS8" id="R_KAS8" name="b-ketoacyl synthetase
(palmitate, n-C16:0)" reversible="false" fast="false"
fbc:lowerFluxBound="irr_lb" fbc:upperFluxBound="irr_ub">
            &lt;listOfReactants&gt;
                  <speciesReference species="M_h_c" stoichiometry="20"
constant="true"/>
                  <speciesReference species="M_nadph_c" stoichiometry="14"
constant="true"/>
                  <speciesReference species="M_accoa_c" stoichiometry="1"
constant="true"/>
                  <speciesReference species="M_malcoa_c" stoichiometry="7"
constant="true"/>
            &lt;/listOfReactants&gt;
            &lt;listOfProducts&gt;
                  <speciesReference species="M_h2o_c" stoichiometry="6"
constant="true"/>
                  <speciesReference species="M_nadp_c" stoichiometry="14"
constant="true"/>
                  <speciesReference species="M_co2_c" stoichiometry="7"
constant="true"/>
                  <speciesReference species="M_coa_c" stoichiometry="8"
constant="true"/>
                  <speciesReference species="M_hdca_c" stoichiometry="1"
constant="true"/>
            &lt;/listOfProducts&gt;
            &lt;fbc:geneProductAssociation&gt;
                  &lt;fbc:and&gt;
                        &lt;fbc:geneProductRef fbc:geneProduct="FabH"/&gt;
                        &lt;fbc:geneProductRef fbc:geneProduct="SAUSA300_0886"/&gt;
                  &lt;/fbc:and&gt;
            &lt;/fbc:geneProductAssociation&gt;
      &lt;/reaction&gt;

      <reaction metaid="R_KAS9" id="R_KAS9" name="b-ketoacyl synthetase (Iso-
C17:1)" reversible="false" fast="false" fbc:lowerFluxBound="irr_lb"
fbc:upperFluxBound="irr_ub">
            &lt;listOfReactants&gt;
                  <speciesReference species="M_h_c" stoichiometry="16"
constant="true"/>
                  <speciesReference species="M_nadph_c" stoichiometry="11"
constant="true"/>
                  <speciesReference species="M_ivcoa_c" stoichiometry="1"
constant="true"/>
                  <speciesReference species="M_malcoa_c" stoichiometry="6"
constant="true"/>
            &lt;/listOfReactants&gt;
            &lt;listOfProducts&gt;
                  <speciesReference species="M_h2o_c" stoichiometry="5"
constant="true"/>
                  <speciesReference species="M_nadp_c" stoichiometry="11"
constant="true"/>
                  <speciesReference species="M_co2_c" stoichiometry="6"
constant="true"/>
                  <speciesReference species="M_coa_c" stoichiometry="7"
constant="true"/>
                  <speciesReference species="M_fa9_c" stoichiometry="1"
constant="true"/>
            &lt;/listOfProducts&gt;
```

```
            <fbc:geneProductAssociation>
                  <fbc:and>
                        <fbc:geneProductRef fbc:geneProduct="FabH"/>
                        <fbc:geneProductRef fbc:geneProduct="SAUSA300_0886"/>
                  </fbc:and>
            </fbc:geneProductAssociation>
      </reaction>

      <reaction metaid="R_Kt2r" id="R_Kt2r" name="potassium reversible
transport  via proton symport" reversible="false" fast="false"
fbc:lowerFluxBound="irr_lb" fbc:upperFluxBound="irr_ub">
            <listOfReactants>
                  <speciesReference species="M_h_e" stoichiometry="1"
constant="true"/>
                  <speciesReference species="M_k_e" stoichiometry="1"
constant="true"/>
            </listOfReactants>
            <listOfProducts>
                  <speciesReference species="M_h_c" stoichiometry="1"
constant="true"/>
                  <speciesReference species="M_k_c" stoichiometry="1"
constant="true"/>
            </listOfProducts>
            <fbc:geneProductAssociation>
                  <fbc:and>
                        <fbc:geneProductRef fbc:geneProduct="SAUSA300_0988"/>
                        <fbc:or>
                              <fbc:geneProductRef
fbc:geneProduct="SAUSA300_1979"/>
                              <fbc:geneProductRef
fbc:geneProduct="SAUSA300_0924"/>
                        </fbc:or>
                  </fbc:and>
            </fbc:geneProductAssociation>
      </reaction>

      <reaction metaid="R_L__LACD3" id="R_L__LACD3" name="L__Lactate
dehydrogenase (menaquinone)" reversible="true" fast="false"
fbc:lowerFluxBound="rev_lb" fbc:upperFluxBound="rev_ub">
            <listOfReactants>
                  <speciesReference species="M_lac__L_c" stoichiometry="1"
constant="true"/>
                  <speciesReference species="M_mqn8_c" stoichiometry="1"
constant="true"/>
            </listOfReactants>
            <listOfProducts>
                  <speciesReference species="M_pyr_c" stoichiometry="1"
constant="true"/>
                  <speciesReference species="M_mql8_c" stoichiometry="1"
constant="true"/>
            </listOfProducts>
            <fbc:geneProductAssociation>
                  <fbc:or>
                        <fbc:geneProductRef fbc:geneProduct="SAUSA300_2537"/>
                        <fbc:geneProductRef fbc:geneProduct="SAUSA300_0235"/>
                  </fbc:or>
            </fbc:geneProductAssociation>
      </reaction>
```

```
      <reaction metaid="R_L__LACt" id="R_L__LACt" name="L__Lactate reversible
transport via proton symport " reversible="true" fast="false"
fbc:lowerFluxBound="rev_lb" fbc:upperFluxBound="rev_ub">
            <listOfReactants>
                  <speciesReference species="M_h_e" stoichiometry="1"
constant="true"/>
                  <speciesReference species="M_lac__L_e" stoichiometry="1"
constant="true"/>
            </listOfReactants>
            <listOfProducts>
                  <speciesReference species="M_h_c" stoichiometry="1"
constant="true"/>
                  <speciesReference species="M_lac__L_c" stoichiometry="1"
constant="true"/>
            </listOfProducts>
            <fbc:geneProductAssociation>
                  <fbc:or>
                        <fbc:geneProductRef fbc:geneProduct="SAUSA300_0112"/>
                        <fbc:geneProductRef fbc:geneProduct="SA451515_2469"/>
                        <fbc:geneProductRef fbc:geneProduct="SAUSA300_2313"/>
                  </fbc:or>
            </fbc:geneProductAssociation>
      </reaction>

      <reaction metaid="R_LACpts" id="R_LACpts" name="Lactose transport via
PEP:Pyr PTS" reversible="true" fast="false" fbc:lowerFluxBound="rev_lb"
fbc:upperFluxBound="rev_ub">
            <listOfReactants>
                  <speciesReference species="M_pep_c" stoichiometry="1"
constant="true"/>
                  <speciesReference species="M_lcts_e" stoichiometry="1"
constant="true"/>
            </listOfReactants>
            <listOfProducts>
                  <speciesReference species="M_pyr_c" stoichiometry="1"
constant="true"/>
                  <speciesReference species="M_lac6p_c" stoichiometry="1"
constant="true"/>
            </listOfProducts>
            <fbc:geneProductAssociation>
                  <fbc:or>
                        <fbc:geneProductRef fbc:geneProduct="SAUSA300_2150"/>
                        <fbc:geneProductRef fbc:geneProduct="SAUSA300_2151"/>
                  </fbc:or>
            </fbc:geneProductAssociation>
      </reaction>

      <reaction metaid="R_LASP2OA" id="R_LASP2OA" name="L-Aspartate 2-
oxoglutarate aminotransferase " reversible="true" fast="false"
fbc:lowerFluxBound="rev_lb" fbc:upperFluxBound="rev_ub">
            <listOfReactants>
                  <speciesReference species="M_oaa_c" stoichiometry="1"
constant="true"/>
                  <speciesReference species="M_Largn_c" stoichiometry="1"
constant="true"/>
            </listOfReactants>
            <listOfProducts>
```

```
                  <speciesReference species="M_pphn_c" stoichiometry="1"
constant="true"/>
                  <speciesReference species="M_asp__L_c" stoichiometry="1"
constant="true"/>
            </listOfProducts>
            <fbc:geneProductAssociation>
                  <fbc:geneProductRef fbc:geneProduct="SAUSA300_0708"/>
            </fbc:geneProductAssociation>
      </reaction>

      <reaction metaid="R_LCADi" id="R_LCADi" name="lactaldehyde
dehydrogenase" reversible="false" fast="false" fbc:lowerFluxBound="irr_lb"
fbc:upperFluxBound="irr_ub">
            <listOfReactants>
                  <speciesReference species="M_h2o_c" stoichiometry="1"
constant="true"/>
                  <speciesReference species="M_nad_c" stoichiometry="1"
constant="true"/>
                  <speciesReference species="M_lald__L_c" stoichiometry="1"
constant="true"/>
            </listOfReactants>
            <listOfProducts>
                  <speciesReference species="M_h_c" stoichiometry="2"
constant="true"/>
                  <speciesReference species="M_nadh_c" stoichiometry="1"
constant="true"/>
                  <speciesReference species="M_lac__L_c" stoichiometry="1"
constant="true"/>
            </listOfProducts>
            <fbc:geneProductAssociation>
                  <fbc:geneProductRef fbc:geneProduct="SAUSA300_0170"/>
            </fbc:geneProductAssociation>
      </reaction>

      <reaction metaid="R_LDH_D" id="R_LDH_D" name="D__Lactate dehydrogenase"
reversible="true" fast="false" fbc:lowerFluxBound="rev_lb"
fbc:upperFluxBound="rev_ub">
            <listOfReactants>
                  <speciesReference species="M_nad_c" stoichiometry="1"
constant="true"/>
                  <speciesReference species="M_lac__D_c" stoichiometry="1"
constant="true"/>
            </listOfReactants>
            <listOfProducts>
                  <speciesReference species="M_h_c" stoichiometry="1"
constant="true"/>
                  <speciesReference species="M_nadh_c" stoichiometry="1"
constant="true"/>
                  <speciesReference species="M_pyr_c" stoichiometry="1"
constant="true"/>
            </listOfProducts>
            <fbc:geneProductAssociation>
                  <fbc:geneProductRef fbc:geneProduct="SAUSA300_2463"/>
            </fbc:geneProductAssociation>
      </reaction>
```

```
      <reaction metaid="R_LDH_L" id="R_LDH_L" name="L__Lactate dehydrogenase"
reversible="true" fast="false" fbc:lowerFluxBound="rev_lb"
fbc:upperFluxBound="rev_ub">
            <listOfReactants>
                  <speciesReference species="M_nad_c" stoichiometry="1"
constant="true"/>
                  <speciesReference species="M_lac__L_c" stoichiometry="1"
constant="true"/>
            </listOfReactants>
            <listOfProducts>
                  <speciesReference species="M_h_c" stoichiometry="1"
constant="true"/>
                  <speciesReference species="M_nadh_c" stoichiometry="1"
constant="true"/>
                  <speciesReference species="M_pyr_c" stoichiometry="1"
constant="true"/>
            </listOfProducts>
            <fbc:geneProductAssociation>
                  <fbc:or>
                        <fbc:geneProductRef fbc:geneProduct="SAUSA300_2537"/>
                        <fbc:geneProductRef fbc:geneProduct="SAUSA300_0235"/>
                  </fbc:or>
            </fbc:geneProductAssociation>
      </reaction>

      <reaction metaid="R_LEUt2r" id="R_LEUt2r" name="L__Leucine reversible
transport via proton symport" reversible="true" fast="false"
fbc:lowerFluxBound="rev_lb" fbc:upperFluxBound="rev_ub">
            <listOfReactants>
                  <speciesReference species="M_h_e" stoichiometry="1"
constant="true"/>
                  <speciesReference species="M_leu__L_e" stoichiometry="1"
constant="true"/>
            </listOfReactants>
            <listOfProducts>
                  <speciesReference species="M_h_c" stoichiometry="1"
constant="true"/>
                  <speciesReference species="M_leu__L_c" stoichiometry="1"
constant="true"/>
            </listOfProducts>
            <fbc:geneProductAssociation>
                  <fbc:or>
                        <fbc:geneProductRef fbc:geneProduct="SAUSA300_1300"/>
                        <fbc:geneProductRef fbc:geneProduct="SAUSA300_0188"/>
                        <fbc:geneProductRef fbc:geneProduct="SAUSA300_0306"/>
                  </fbc:or>
            </fbc:geneProductAssociation>
      </reaction>

      <reaction metaid="R_LEUTA" id="R_LEUTA" name="leucine transaminase"
reversible="true" fast="false" fbc:lowerFluxBound="rev_lb"
fbc:upperFluxBound="rev_ub">
            <listOfReactants>
                  <speciesReference species="M_akg_c" stoichiometry="1"
constant="true"/>
                  <speciesReference species="M_leu__L_c" stoichiometry="1"
constant="true"/>
            </listOfReactants>
```

```
            <listOfProducts>
                  <speciesReference species="M_glu__L_c" stoichiometry="1"
constant="true"/>
                  <speciesReference species="M_4mop_c" stoichiometry="1"
constant="true"/>
            </listOfProducts>
            <fbc:geneProductAssociation>
                  <fbc:geneProductRef fbc:geneProduct="SAUSA300_0539"/>
            </fbc:geneProductAssociation>
      </reaction>

      <reaction metaid="R_LHISO" id="R_LHISO" name="L-Histidinol NAD
oxidoreductase " reversible="false" fast="false" fbc:lowerFluxBound="irr_lb"
fbc:upperFluxBound="irr_ub">
            <listOfReactants>
                  <speciesReference species="M_nad_c" stoichiometry="1"
constant="true"/>
                  <speciesReference species="M_histd_c" stoichiometry="1"
constant="true"/>
            </listOfReactants>
            <listOfProducts>
                  <speciesReference species="M_h_c" stoichiometry="1"
constant="true"/>
                  <speciesReference species="M_nadh_c" stoichiometry="1"
constant="true"/>
                  <speciesReference species="M_htdol__L_c" stoichiometry="1"
constant="true"/>
            </listOfProducts>
            <fbc:geneProductAssociation>
                  <fbc:geneProductRef fbc:geneProduct="SAUSA300_2611"/>
            </fbc:geneProductAssociation>
      </reaction>

      <reaction metaid="R_LHIST" id="R_LHIST" name="L-Histidinal NAD
oxidoreductase " reversible="false" fast="false" fbc:lowerFluxBound="irr_lb"
fbc:upperFluxBound="irr_ub">
            <listOfReactants>
                  <speciesReference species="M_h2o_c" stoichiometry="1"
constant="true"/>
                  <speciesReference species="M_nad_c" stoichiometry="1"
constant="true"/>
                  <speciesReference species="M_htdol__L_c" stoichiometry="1"
constant="true"/>
            </listOfReactants>
            <listOfProducts>
                  <speciesReference species="M_h_c" stoichiometry="2"
constant="true"/>
                  <speciesReference species="M_his__L_c" stoichiometry="1"
constant="true"/>
                  <speciesReference species="M_nadh_c" stoichiometry="1"
constant="true"/>
            </listOfProducts>
            <fbc:geneProductAssociation>
                  <fbc:geneProductRef fbc:geneProduct="SAUSA300_2611"/>
            </fbc:geneProductAssociation>
      </reaction>
```

```
      <reaction metaid="R_LIPATPT" id="R_LIPATPT" name="Lipoate-ATP adenylate
transferase" reversible="false" fast="false" fbc:lowerFluxBound="irr_lb"
fbc:upperFluxBound="irr_ub">
            <listOfReactants>
                  <speciesReference species="M_h_c" stoichiometry="1"
constant="true"/>
                  <speciesReference species="M_atp_c" stoichiometry="1"
constant="true"/>
                  <speciesReference species="M_lipoate_c" stoichiometry="1"
constant="true"/>
            </listOfReactants>
            <listOfProducts>
                  <speciesReference species="M_ppi_c" stoichiometry="1"
constant="true"/>
                  <speciesReference species="M_lipoamp_c" stoichiometry="1"
constant="true"/>
            </listOfProducts>
            <fbc:geneProductAssociation>
                  <fbc:or>
                        <fbc:geneProductRef fbc:geneProduct="SAUSA300_0930"/>
                        <fbc:geneProductRef fbc:geneProduct="SAUSA300_0328"/>
                        <fbc:geneProductRef fbc:geneProduct="SAUSA300_1494"/>
                  </fbc:or>
            </fbc:geneProductAssociation>
      </reaction>

      <reaction metaid="R_LPLIPAL1E181pp" id="R_LPLIPAL1E181pp"
name="Lysophospholipase L1 (2-acylglycerophosphoethanolamine, n-C18:1)
(periplasm)" reversible="false" fast="false" fbc:lowerFluxBound="irr_lb"
fbc:upperFluxBound="irr_ub">
            <listOfReactants>
                  <speciesReference species="M_h2o_c" stoichiometry="1"
constant="true"/>
                  <speciesReference species="M_1agpe181_c" stoichiometry="1"
constant="true"/>
            </listOfReactants>
            <listOfProducts>
                  <speciesReference species="M_h_c" stoichiometry="1"
constant="true"/>
                  <speciesReference species="M_elaid_c" stoichiometry="1"
constant="true"/>
                  <speciesReference species="M_g3pe_c" stoichiometry="1"
constant="true"/>
            </listOfProducts>
            <fbc:geneProductAssociation>
                  <fbc:or>
                        <fbc:geneProductRef fbc:geneProduct="SAUSA300_0070"/>
                        <fbc:geneProductRef fbc:geneProduct="SAUSA300_1710"/>
                  </fbc:or>
            </fbc:geneProductAssociation>
      </reaction>

      <reaction metaid="R_LPLIPAL1G161pp" id="R_LPLIPAL1G161pp"
name="Lysophospholipase L1 (2-acylglycerophosphoglycerol, n-C16:1)
(periplasm)" reversible="false" fast="false" fbc:lowerFluxBound="irr_lb"
fbc:upperFluxBound="irr_ub">
            <listOfReactants>
```

```
                <speciesReference species="M_diipdpgly_c" stoichiometry="2"
constant="true"/>
            </listOfReactants>
            <listOfProducts>
                <speciesReference species="M_glyc_c" stoichiometry="1"
constant="true"/>
                <speciesReference species="M_isopcard_c" stoichiometry="1"
constant="true"/>
            </listOfProducts>
            <fbc:geneProductAssociation>
                <fbc:or>
                    <fbc:geneProductRef fbc:geneProduct="SAUSA300_2044"/>
                    <fbc:geneProductRef fbc:geneProduct="SAUSA300_1216"/>
                </fbc:or>
            </fbc:geneProductAssociation>
        </reaction>

        <reaction metaid="R_LPLIPAL1G180pp" id="R_LPLIPAL1G180pp"
name="Lysophospholipase L1 (2-acylglycerophosphoglycerol, n-C18:0)
(periplasm)" reversible="false" fast="false" fbc:lowerFluxBound="irr_lb"
fbc:upperFluxBound="irr_ub">
            <listOfReactants>
                <speciesReference species="M_h2o_c" stoichiometry="1"
constant="true"/>
                <speciesReference species="M_1agpg180_c" stoichiometry="1"
constant="true"/>
            </listOfReactants>
            <listOfProducts>
                <speciesReference species="M_h_c" stoichiometry="1"
constant="true"/>
                <speciesReference species="M_g3pg_c" stoichiometry="1"
constant="true"/>
                <speciesReference species="M_ocdca_c" stoichiometry="1"
constant="true"/>
            </listOfProducts>
            <fbc:geneProductAssociation>
                <fbc:or>
                    <fbc:geneProductRef fbc:geneProduct="SAUSA300_0070"/>
                    <fbc:geneProductRef fbc:geneProduct="SAUSA300_1710"/>
                </fbc:or>
            </fbc:geneProductAssociation>
        </reaction>

        <reaction metaid="R_LPLIPAL1G181pp" id="R_LPLIPAL1G181pp"
name="Lysophospholipase L1 (2-acylglycerophosphoglycerol, n-C18:1)
(periplasm)" reversible="false" fast="false" fbc:lowerFluxBound="irr_lb"
fbc:upperFluxBound="irr_ub">
            <listOfReactants>
                <speciesReference species="M_h2o_c" stoichiometry="1"
constant="true"/>
                <speciesReference species="M_1agpg181_c" stoichiometry="1"
constant="true"/>
            </listOfReactants>
            <listOfProducts>
                <speciesReference species="M_h_c" stoichiometry="1"
constant="true"/>
                <speciesReference species="M_g3pg_c" stoichiometry="1"
constant="true"/>
```

```
                  <speciesReference species="M_elaid_c" stoichiometry="1"
constant="true"/>
            </listOfProducts>
            <fbc:geneProductAssociation>
                  <fbc:or>
                        <fbc:geneProductRef fbc:geneProduct="SAUSA300_1194"/>
                        <fbc:geneProductRef fbc:geneProduct="SAUSA300_1710"/>
                  </fbc:or>
            </fbc:geneProductAssociation>
      </reaction>

      <reaction metaid="R_LPLIPAL2A161" id="R_LPLIPAL2A161"
name="Lysophospholipase L2 (2-acylglycerophosphotidate, n-C16:1)"
reversible="false" fast="false" fbc:lowerFluxBound="irr_lb"
fbc:upperFluxBound="irr_ub">
            <listOfReactants>
                  <speciesReference species="M_h2o_c" stoichiometry="1"
constant="true"/>
                  <speciesReference species="M_2hdec9eg3p_c"
stoichiometry="1" constant="true"/>
            </listOfReactants>
            <listOfProducts>
                  <speciesReference species="M_h_c" stoichiometry="1"
constant="true"/>
                  <speciesReference species="M_glyc3p_c" stoichiometry="1"
constant="true"/>
                  <speciesReference species="M_hdcea_c" stoichiometry="1"
constant="true"/>
            </listOfProducts>
            <fbc:geneProductAssociation>
                  <fbc:or>
                        <fbc:geneProductRef fbc:geneProduct="SAUSA300_0070"/>
                        <fbc:geneProductRef fbc:geneProduct="SAUSA300_1710"/>
                  </fbc:or>
            </fbc:geneProductAssociation>
      </reaction>

      <reaction metaid="R_LPLIPAL2E141" id="R_LPLIPAL2E141"
name="Lysophospholipase L2 (2-acylglycerophosphoethanolamine, n-C14:1)"
reversible="false" fast="false" fbc:lowerFluxBound="irr_lb"
fbc:upperFluxBound="irr_ub">
            <listOfReactants>
                  <speciesReference species="M_h2o_c" stoichiometry="1"
constant="true"/>
                  <speciesReference species="M_2agpe141_c" stoichiometry="1"
constant="true"/>
            </listOfReactants>
            <listOfProducts>
                  <speciesReference species="M_h_c" stoichiometry="1"
constant="true"/>
                  <speciesReference species="M_g3pe_c" stoichiometry="1"
constant="true"/>
                  <speciesReference species="M_ttdcea_c" stoichiometry="1"
constant="true"/>
            </listOfProducts>
            <fbc:geneProductAssociation>
                  <fbc:or>
                        <fbc:geneProductRef fbc:geneProduct="SAUSA300_0070"/>
```

```
                  <fbc:geneProductRef fbc:geneProduct="SAUSA300_1710"/>
            </fbc:or>
      </fbc:geneProductAssociation>
</reaction>

<reaction metaid="R_LPLIPAL2E160" id="R_LPLIPAL2E160"
name="Lysophospholipase L2 (2-acylglycerophosphoethanolamine, n-C16:0)"
reversible="false" fast="false" fbc:lowerFluxBound="irr_lb"
fbc:upperFluxBound="irr_ub">
      <listOfReactants>
            <speciesReference species="M_h2o_c" stoichiometry="1"
constant="true"/>
            <speciesReference species="M_2agpe160_c" stoichiometry="1"
constant="true"/>
      </listOfReactants>
      <listOfProducts>
            <speciesReference species="M_h_c" stoichiometry="1"
constant="true"/>
            <speciesReference species="M_g3pe_c" stoichiometry="1"
constant="true"/>
            <speciesReference species="M_hdca_c" stoichiometry="1"
constant="true"/>
      </listOfProducts>
      <fbc:geneProductAssociation>
            <fbc:or>
                  <fbc:geneProductRef fbc:geneProduct="SAUSA300_0070"/>
                  <fbc:geneProductRef fbc:geneProduct="SAUSA300_1710"/>
            </fbc:or>
      </fbc:geneProductAssociation>
</reaction>

<reaction metaid="R_LPLIPAL2G160" id="R_LPLIPAL2G160"
name="Lysophospholipase L2 (2-acylglycerophosphoglycerol, n-C16:0)"
reversible="false" fast="false" fbc:lowerFluxBound="irr_lb"
fbc:upperFluxBound="irr_ub">
      <listOfReactants>
            <speciesReference species="M_h2o_c" stoichiometry="1"
constant="true"/>
            <speciesReference species="M_2agpg160_c" stoichiometry="1"
constant="true"/>
      </listOfReactants>
      <listOfProducts>
            <speciesReference species="M_h_c" stoichiometry="1"
constant="true"/>
            <speciesReference species="M_g3pg_c" stoichiometry="1"
constant="true"/>
            <speciesReference species="M_hdca_c" stoichiometry="1"
constant="true"/>
      </listOfProducts>
      <fbc:geneProductAssociation>
            <fbc:or>
                  <fbc:geneProductRef fbc:geneProduct="SAUSA300_0070"/>
                  <fbc:geneProductRef fbc:geneProduct="SAUSA300_1710"/>
            </fbc:or>
      </fbc:geneProductAssociation>
</reaction>
```

```
      <reaction metaid="R_LPLIPAL2G180" id="R_LPLIPAL2G180"
name="Lysophospholipase L2 (2-acylglycerophosphoglycerol, n-C18:0)"
reversible="false" fast="false" fbc:lowerFluxBound="irr_lb"
fbc:upperFluxBound="irr_ub">
            <listOfReactants>
                  <speciesReference species="M_h2o_c" stoichiometry="1"
constant="true"/>
                  <speciesReference species="M_2agpg180_c" stoichiometry="1"
constant="true"/>
            </listOfReactants>
            <listOfProducts>
                  <speciesReference species="M_h_c" stoichiometry="1"
constant="true"/>
                  <speciesReference species="M_g3pg_c" stoichiometry="1"
constant="true"/>
                  <speciesReference species="M_ocdca_c" stoichiometry="1"
constant="true"/>
            </listOfProducts>
            <fbc:geneProductAssociation>
                  <fbc:or>
                        <fbc:geneProductRef fbc:geneProduct="SAUSA300_0070"/>
                        <fbc:geneProductRef fbc:geneProduct="SAUSA300_1710"/>
                  </fbc:or>
            </fbc:geneProductAssociation>
      </reaction>

      <reaction metaid="R_LYSabc" id="R_LYSabc" name="L__Lysine transport via
ABC system" reversible="false" fast="false" fbc:lowerFluxBound="irr_lb"
fbc:upperFluxBound="irr_ub">
            <listOfReactants>
                  <speciesReference species="M_h2o_c" stoichiometry="1"
constant="true"/>
                  <speciesReference species="M_atp_c" stoichiometry="1"
constant="true"/>
                  <speciesReference species="M_lys__L_e" stoichiometry="1"
constant="true"/>
            </listOfReactants>
            <listOfProducts>
                  <speciesReference species="M_h_c" stoichiometry="1"
constant="true"/>
                  <speciesReference species="M_pi_c" stoichiometry="1"
constant="true"/>
                  <speciesReference species="M_adp_c" stoichiometry="1"
constant="true"/>
                  <speciesReference species="M_lys__L_c" stoichiometry="1"
constant="true"/>
            </listOfProducts>
            <fbc:geneProductAssociation>
                  <fbc:or>
                        <fbc:geneProductRef fbc:geneProduct="SAUSA300_1231"/>
                        <fbc:geneProductRef fbc:geneProduct="SAUSA300_1628"/>
                  </fbc:or>
            </fbc:geneProductAssociation>
      </reaction>

      <reaction metaid="R_LYSDC" id="R_LYSDC" name="lysine decarboxylase"
reversible="false" fast="false" fbc:lowerFluxBound="irr_lb"
fbc:upperFluxBound="irr_ub">
```

```
            <listOfReactants>
                  <speciesReference species="M_h_c" stoichiometry="1"
constant="true"/>
                  <speciesReference species="M_lys__L_c" stoichiometry="1"
constant="true"/>
            </listOfReactants>
            <listOfProducts>
                  <speciesReference species="M_co2_c" stoichiometry="1"
constant="true"/>
                  <speciesReference species="M_15dap_c" stoichiometry="1"
constant="true"/>
            </listOfProducts>
            <fbc:geneProductAssociation>
                  <fbc:geneProductRef fbc:geneProduct="SAUSA300_0458"/>
            </fbc:geneProductAssociation>
      </reaction>

      <reaction metaid="R_LYSt2r" id="R_LYSt2r" name="L__Lysine reversible
transport via proton symport" reversible="false" fast="false"
fbc:lowerFluxBound="irr_lb" fbc:upperFluxBound="irr_ub">
            <listOfReactants>
                  <speciesReference species="M_h_e" stoichiometry="1"
constant="true"/>
                  <speciesReference species="M_lys__L_e" stoichiometry="1"
constant="true"/>
            </listOfReactants>
            <listOfProducts>
                  <speciesReference species="M_h_c" stoichiometry="1"
constant="true"/>
                  <speciesReference species="M_lys__L_c" stoichiometry="1"
constant="true"/>
            </listOfProducts>
            <fbc:geneProductAssociation>
                  <fbc:or>
                        <fbc:geneProductRef fbc:geneProduct="SAUSA300_1231"/>
                        <fbc:geneProductRef fbc:geneProduct="SAUSA300_1628"/>
                  </fbc:or>
            </fbc:geneProductAssociation>
      </reaction>

      <reaction metaid="R_LYSt3r" id="R_LYSt3r" name="L__Lysine transport out
via proton antiport reversible" reversible="true" fast="false"
fbc:lowerFluxBound="rev_lb" fbc:upperFluxBound="rev_ub">
            <listOfReactants>
                  <speciesReference species="M_h_e" stoichiometry="1"
constant="true"/>
                  <speciesReference species="M_lys__L_c" stoichiometry="1"
constant="true"/>
            </listOfReactants>
            <listOfProducts>
                  <speciesReference species="M_h_c" stoichiometry="1"
constant="true"/>
                  <speciesReference species="M_lys__L_e" stoichiometry="1"
constant="true"/>
            </listOfProducts>
            <fbc:geneProductAssociation>
                  <fbc:geneProductRef fbc:geneProduct="SAUSA300_0784"/>
            </fbc:geneProductAssociation>
```

```
      </reaction>

      <reaction metaid="R_LYSTRS" id="R_LYSTRS" name="Lysyl-tRNA synthetase"
reversible="false" fast="false" fbc:lowerFluxBound="irr_lb"
fbc:upperFluxBound="irr_ub">
            <listOfReactants>
                  <speciesReference species="M_atp_c" stoichiometry="1"
constant="true"/>
                  <speciesReference species="M_lys__L_c" stoichiometry="1"
constant="true"/>
                  <speciesReference species="M_trnalys_c" stoichiometry="1"
constant="true"/>
            </listOfReactants>
            <listOfProducts>
                  <speciesReference species="M_ppi_c" stoichiometry="1"
constant="true"/>
                  <speciesReference species="M_amp_c" stoichiometry="1"
constant="true"/>
                  <speciesReference species="M_lystrna_c" stoichiometry="1"
constant="true"/>
            </listOfProducts>
            <fbc:geneProductAssociation>
                  <fbc:geneProductRef fbc:geneProduct="SAUSA300_0496"/>
            </fbc:geneProductAssociation>
      </reaction>

      <reaction metaid="R_M1PD" id="R_M1PD" name="mannitol-1-phosphate
5__Dehydrogenase" reversible="false" fast="false" fbc:lowerFluxBound="irr_lb"
fbc:upperFluxBound="irr_ub">
            <listOfReactants>
                  <speciesReference species="M_nad_c" stoichiometry="1"
constant="true"/>
                  <speciesReference species="M_mnl1p_c" stoichiometry="1"
constant="true"/>
            </listOfReactants>
            <listOfProducts>
                  <speciesReference species="M_h_c" stoichiometry="1"
constant="true"/>
                  <speciesReference species="M_nadh_c" stoichiometry="1"
constant="true"/>
                  <speciesReference species="M_f6p_c" stoichiometry="1"
constant="true"/>
            </listOfProducts>
            <fbc:geneProductAssociation>
                  <fbc:geneProductRef fbc:geneProduct="SAUSA300_2108"/>
            </fbc:geneProductAssociation>
      </reaction>

      <reaction metaid="R_MACPD" id="R_MACPD" name="Malonyl-ACP
decarboxylase" reversible="false" fast="false" fbc:lowerFluxBound="irr_lb"
fbc:upperFluxBound="irr_ub">
            <listOfReactants>
                  <speciesReference species="M_h_c" stoichiometry="1"
constant="true"/>
                  <speciesReference species="M_malACP_c" stoichiometry="1"
constant="true"/>
            </listOfReactants>
            <listOfProducts>
```

```
                  <speciesReference species="M_co2_c" stoichiometry="1"
constant="true"/>
                  <speciesReference species="M_acACP_c" stoichiometry="1"
constant="true"/>
            </listOfProducts>
            <fbc:geneProductAssociation>
                  <fbc:geneProductRef fbc:geneProduct="SAUSA300_0886"/>
            </fbc:geneProductAssociation>
      </reaction>

      <reaction metaid="R_MAL__D_Et" id="R_MAL__D_Et" name="D-Malate
transport " reversible="true" fast="false" fbc:lowerFluxBound="rev_lb"
fbc:upperFluxBound="rev_ub">
            <listOfReactants>
                  <speciesReference species="M_mal__D_e" stoichiometry="1"
constant="true"/>
            </listOfReactants>
            <listOfProducts>
                  <speciesReference species="M_mal__D_c" stoichiometry="1"
constant="true"/>
            </listOfProducts>
      </reaction>

      <reaction metaid="R_MALT" id="R_MALT" name="alpha-glucosidase"
reversible="true" fast="false" fbc:lowerFluxBound="rev_lb"
fbc:upperFluxBound="rev_ub">
            <listOfReactants>
                  <speciesReference species="M_h2o_c" stoichiometry="1"
constant="true"/>
                  <speciesReference species="M_malt_c" stoichiometry="1"
constant="true"/>
            </listOfReactants>
            <listOfProducts>
                  <speciesReference species="M_glc__D_c" stoichiometry="2"
constant="true"/>
            </listOfProducts>
            <fbc:geneProductAssociation>
                  <fbc:geneProductRef fbc:geneProduct="SAUSA300_1456"/>
            </fbc:geneProductAssociation>
      </reaction>

      <reaction metaid="R_MALt2r" id="R_MALt2r" name="L-malate reversible
transport via proton symport" reversible="false" fast="false"
fbc:lowerFluxBound="irr_lb" fbc:upperFluxBound="irr_ub">
            <listOfReactants>
                  <speciesReference species="M_h_e" stoichiometry="1"
constant="true"/>
                  <speciesReference species="M_mal__L_e" stoichiometry="1"
constant="true"/>
            </listOfReactants>
            <listOfProducts>
                  <speciesReference species="M_h_c" stoichiometry="1"
constant="true"/>
                  <speciesReference species="M_mal__L_c" stoichiometry="1"
constant="true"/>
            </listOfProducts>
            <fbc:geneProductAssociation>
                  <fbc:geneProductRef fbc:geneProduct="SAUSA300_2627"/>
```

```
			&lt;/fbc:geneProductAssociation&gt;
		&lt;/reaction&gt;

		<reaction metaid="R_MALt4" id="R_MALt4" name="Na/malate symporter "
reversible="false" fast="false" fbc:lowerFluxBound="irr_lb"
fbc:upperFluxBound="irr_ub">
			&lt;listOfReactants&gt;
				<speciesReference species="M_na1_e" stoichiometry="1"
constant="true"/>
				<speciesReference species="M_mal__L_e" stoichiometry="1"
constant="true"/>
			&lt;/listOfReactants&gt;
			&lt;listOfProducts&gt;
				<speciesReference species="M_na1_c" stoichiometry="1"
constant="true"/>
				<speciesReference species="M_mal__L_c" stoichiometry="1"
constant="true"/>
			&lt;/listOfProducts&gt;
			&lt;fbc:geneProductAssociation&gt;
				&lt;fbc:geneProductRef fbc:geneProduct="SAUSA300_2627"/&gt;
			&lt;/fbc:geneProductAssociation&gt;
		&lt;/reaction&gt;

		<reaction metaid="R_MALTabc" id="R_MALTabc" name="maltose transport via
ABC system" reversible="false" fast="false" fbc:lowerFluxBound="irr_lb"
fbc:upperFluxBound="irr_ub">
			&lt;listOfReactants&gt;
				<speciesReference species="M_h2o_c" stoichiometry="1"
constant="true"/>
				<speciesReference species="M_atp_c" stoichiometry="1"
constant="true"/>
				<speciesReference species="M_malt_e" stoichiometry="1"
constant="true"/>
			&lt;/listOfReactants&gt;
			&lt;listOfProducts&gt;
				<speciesReference species="M_h_c" stoichiometry="1"
constant="true"/>
				<speciesReference species="M_pi_c" stoichiometry="1"
constant="true"/>
				<speciesReference species="M_adp_c" stoichiometry="1"
constant="true"/>
				<speciesReference species="M_malt_c" stoichiometry="1"
constant="true"/>
			&lt;/listOfProducts&gt;
			&lt;fbc:geneProductAssociation&gt;
				&lt;fbc:or&gt;
					&lt;fbc:and&gt;
						<fbc:geneProductRef
fbc:geneProduct="SAUSA300_0211"/>
						<fbc:geneProductRef
fbc:geneProduct="SAUSA300_0210"/>
						<fbc:geneProductRef
fbc:geneProduct="SAUSA300_0209"/>
					&lt;/fbc:and&gt;
					&lt;fbc:geneProductRef fbc:geneProduct="SAUSA300_0208"/&gt;
				&lt;/fbc:or&gt;
			&lt;/fbc:geneProductAssociation&gt;
		&lt;/reaction&gt;
```

```
      <reaction metaid="R_MALTAT" id="R_MALTAT" name="maltose O-
acetyltransferase" reversible="false" fast="false"
fbc:lowerFluxBound="irr_lb" fbc:upperFluxBound="irr_ub">
            <listOfReactants>
                  <speciesReference species="M_malt_c" stoichiometry="1"
constant="true"/>
                  <speciesReference species="M_accoa_c" stoichiometry="1"
constant="true"/>
            </listOfReactants>
            <listOfProducts>
                  <speciesReference species="M_coa_c" stoichiometry="1"
constant="true"/>
                  <speciesReference species="M_acmalt_c" stoichiometry="1"
constant="true"/>
            </listOfProducts>
            <fbc:geneProductAssociation>
                  <fbc:geneProductRef fbc:geneProduct="SAUSA300_2492"/>
            </fbc:geneProductAssociation>
      </reaction>

      <reaction metaid="R_MALTHXabc" id="R_MALTHXabc" name="maltohexaose
transport via ABC system" reversible="false" fast="false"
fbc:lowerFluxBound="irr_lb" fbc:upperFluxBound="irr_ub">
            <listOfReactants>
                  <speciesReference species="M_h2o_c" stoichiometry="1"
constant="true"/>
                  <speciesReference species="M_atp_c" stoichiometry="1"
constant="true"/>
                  <speciesReference species="M_malthx_e" stoichiometry="1"
constant="true"/>
            </listOfReactants>
            <listOfProducts>
                  <speciesReference species="M_h_c" stoichiometry="1"
constant="true"/>
                  <speciesReference species="M_pi_c" stoichiometry="1"
constant="true"/>
                  <speciesReference species="M_adp_c" stoichiometry="1"
constant="true"/>
                  <speciesReference species="M_malthx_c" stoichiometry="1"
constant="true"/>
            </listOfProducts>
            <fbc:geneProductAssociation>
                  <fbc:or>
                        <fbc:geneProductRef fbc:geneProduct="SAUSA300_0211"/>
                        <fbc:geneProductRef fbc:geneProduct="SAUSA300_0210"/>
                        <fbc:geneProductRef fbc:geneProduct="SAUSA300_0208"/>
                        <fbc:geneProductRef fbc:geneProduct="SAUSA300_0209"/>
                  </fbc:or>
            </fbc:geneProductAssociation>
      </reaction>

      <reaction metaid="R_MALTpts" id="R_MALTpts" name="maltose transport via
PEP:Pyr PTS" reversible="true" fast="false" fbc:lowerFluxBound="rev_lb"
fbc:upperFluxBound="rev_ub">
            <listOfReactants>
                  <speciesReference species="M_pep_c" stoichiometry="1"
constant="true"/>
```

```
                  <speciesReference species="M_malt_e" stoichiometry="1"
constant="true"/>
            &lt;/listOfReactants&gt;
            &lt;listOfProducts&gt;
                  <speciesReference species="M_pyr_c" stoichiometry="1"
constant="true"/>
                  <speciesReference species="M_malt6p_c" stoichiometry="1"
constant="true"/>
            &lt;/listOfProducts&gt;
            &lt;fbc:geneProductAssociation&gt;
                  &lt;fbc:and&gt;
                        &lt;fbc:geneProductRef fbc:geneProduct="SAUSA300_0236"/&gt;
                        &lt;fbc:geneProductRef fbc:geneProduct="SAUSA300_0983"/&gt;
                        &lt;fbc:geneProductRef fbc:geneProduct="SAUSA300_0984"/&gt;
                  &lt;/fbc:and&gt;
            &lt;/fbc:geneProductAssociation&gt;
      &lt;/reaction&gt;

      <reaction metaid="R_MALTTRabc" id="R_MALTTRabc" name="Maltotriose
transport via ABC system" reversible="false" fast="false"
fbc:lowerFluxBound="irr_lb" fbc:upperFluxBound="irr_ub">
            &lt;listOfReactants&gt;
                  <speciesReference species="M_h2o_c" stoichiometry="1"
constant="true"/>
                  <speciesReference species="M_atp_c" stoichiometry="1"
constant="true"/>
                  <speciesReference species="M_malttr_e" stoichiometry="1"
constant="true"/>
            &lt;/listOfReactants&gt;
            &lt;listOfProducts&gt;
                  <speciesReference species="M_h_c" stoichiometry="1"
constant="true"/>
                  <speciesReference species="M_pi_c" stoichiometry="1"
constant="true"/>
                  <speciesReference species="M_adp_c" stoichiometry="1"
constant="true"/>
                  <speciesReference species="M_malttr_c" stoichiometry="1"
constant="true"/>
            &lt;/listOfProducts&gt;
            &lt;fbc:geneProductAssociation&gt;
                  &lt;fbc:and&gt;
                        &lt;fbc:geneProductRef fbc:geneProduct="SAUSA300_0211"/&gt;
                        &lt;fbc:geneProductRef fbc:geneProduct="SAUSA300_0210"/&gt;
                        &lt;fbc:geneProductRef fbc:geneProduct="SAUSA300_0208"/&gt;
                        &lt;fbc:geneProductRef fbc:geneProduct="SAUSA300_0209"/&gt;
                  &lt;/fbc:and&gt;
            &lt;/fbc:geneProductAssociation&gt;
      &lt;/reaction&gt;

      <reaction metaid="R_MAN6PI" id="R_MAN6PI" name="mannose-6-phosphate
isomerase" reversible="false" fast="false" fbc:lowerFluxBound="irr_lb"
fbc:upperFluxBound="irr_ub">
            &lt;listOfReactants&gt;
                  <speciesReference species="M_man6p_c" stoichiometry="1"
constant="true"/>
            &lt;/listOfReactants&gt;
            &lt;listOfProducts&gt;
```

```xml
                  <speciesReference species="M_f6p_c" stoichiometry="1"
constant="true"/>
            </listOfProducts>
            <fbc:geneProductAssociation>
                  <fbc:or>
                        <fbc:geneProductRef fbc:geneProduct="SAUSA300_2577"/>
                        <fbc:geneProductRef fbc:geneProduct="SAUSA300_2096"/>
                  </fbc:or>
            </fbc:geneProductAssociation>
      </reaction>

      <reaction metaid="R_MAN6Pt6_2pp" id="R_MAN6Pt6_2pp" name="Mannose-6-
phosphate transport via phosphate antiport (periplasm)" reversible="false"
fast="false" fbc:lowerFluxBound="irr_lb" fbc:upperFluxBound="irr_ub">
            <listOfReactants>
                  <speciesReference species="M_pi_c" stoichiometry="2"
constant="true"/>
                  <speciesReference species="M_man6p_p" stoichiometry="1"
constant="true"/>
            </listOfReactants>
            <listOfProducts>
                  <speciesReference species="M_man6p_c" stoichiometry="1"
constant="true"/>
                  <speciesReference species="M_pi_p" stoichiometry="2"
constant="true"/>
            </listOfProducts>
            <fbc:geneProductAssociation>
                  <fbc:geneProductRef fbc:geneProduct="SAUSA300_2576"/>
            </fbc:geneProductAssociation>
      </reaction>

      <reaction metaid="R_MAN6Ptex" id="R_MAN6Ptex" name="Mannose 6-phosphate
transport via diffusion (extracellular to periplasm)" reversible="true"
fast="false" fbc:lowerFluxBound="rev_lb" fbc:upperFluxBound="rev_ub">
            <listOfReactants>
                  <speciesReference species="M_man6p_e" stoichiometry="1"
constant="true"/>
            </listOfReactants>
            <listOfProducts>
                  <speciesReference species="M_man6p_p" stoichiometry="1"
constant="true"/>
            </listOfProducts>
      </reaction>

      <reaction metaid="R_MANAO" id="R_MANAO" name="Mannonate oxidoreductase"
reversible="true" fast="false" fbc:lowerFluxBound="rev_lb"
fbc:upperFluxBound="rev_ub">
            <listOfReactants>
                  <speciesReference species="M_nad_c" stoichiometry="1"
constant="true"/>
                  <speciesReference species="M_mana_c" stoichiometry="1"
constant="true"/>
            </listOfReactants>
            <listOfProducts>
                  <speciesReference species="M_h_c" stoichiometry="1"
constant="true"/>
                  <speciesReference species="M_nadh_c" stoichiometry="1"
constant="true"/>
```

```
                  <speciesReference species="M_fruur_c" stoichiometry="1"
constant="true"/>
            </listOfProducts>
            <fbc:geneProductAssociation>
                  <fbc:or>
                        <fbc:geneProductRef fbc:geneProduct="uxuB"/>
                        <fbc:geneProductRef fbc:geneProduct="SAUSA300_1646"/>
                  </fbc:or>
            </fbc:geneProductAssociation>
      </reaction>

      <reaction metaid="R_manl1p_Et" id="R_manl1p_Et" name="manl1p transport
(transport mechanism unknown - evidence from biolog data)" reversible="true"
fast="false" fbc:lowerFluxBound="rev_lb" fbc:upperFluxBound="rev_ub">
            <listOfReactants>
                  <speciesReference species="M_manl1p_e" stoichiometry="1"
constant="true"/>
            </listOfReactants>
            <listOfProducts>
                  <speciesReference species="M_manl1p_c" stoichiometry="1"
constant="true"/>
            </listOfProducts>
      </reaction>

      <reaction metaid="R_MANpts" id="R_MANpts" name="D-mannose transport via
PEP:Pyr PTS" reversible="true" fast="false" fbc:lowerFluxBound="rev_lb"
fbc:upperFluxBound="rev_ub">
            <listOfReactants>
                  <speciesReference species="M_man_e" stoichiometry="1"
constant="true"/>
                  <speciesReference species="M_pep_c" stoichiometry="1"
constant="true"/>
            </listOfReactants>
            <listOfProducts>
                  <speciesReference species="M_pyr_c" stoichiometry="1"
constant="true"/>
                  <speciesReference species="M_man6p_c" stoichiometry="1"
constant="true"/>
            </listOfProducts>
            <fbc:geneProductAssociation>
                  <fbc:and>
                        <fbc:geneProductRef fbc:geneProduct="SAUSA300_2576"/>
                        <fbc:geneProductRef fbc:geneProduct="SAUSA300_0984"/>
                        <fbc:geneProductRef fbc:geneProduct="SAUSA300_0191"/>
                        <fbc:geneProductRef fbc:geneProduct="SAUSA300_0983"/>
                  </fbc:and>
            </fbc:geneProductAssociation>
      </reaction>

      <reaction metaid="R_MBCOA3" id="R_MBCOA3" name="S-2-methylbutanoyl-CoA
oxygen 2 3-oxidoreductase " reversible="false" fast="false"
fbc:lowerFluxBound="irr_lb" fbc:upperFluxBound="irr_ub">
            <listOfReactants>
                  <speciesReference species="M_o2_c" stoichiometry="1"
constant="true"/>
                  <speciesReference species="M_2mbcoa_c" stoichiometry="2"
constant="true"/>
            </listOfReactants>
```

```
            <listOfProducts>
                  <speciesReference species="M_h2o_c" stoichiometry="2"
constant="true"/>
                  <speciesReference species="M_2mb2coa_c" stoichiometry="2"
constant="true"/>
            </listOfProducts>
            <fbc:geneProductAssociation>
                  <fbc:geneProductRef fbc:geneProduct="SAUSA300_2236"/>
            </fbc:geneProductAssociation>
      </reaction>

      <reaction metaid="R_MCOATA" id="R_MCOATA" name="Malonyl-CoA-ACP
transacylase" reversible="true" fast="false" fbc:lowerFluxBound="rev_lb"
fbc:upperFluxBound="rev_ub">
            <listOfReactants>
                  <speciesReference species="M_ACP_c" stoichiometry="1"
constant="true"/>
                  <speciesReference species="M_malcoa_c" stoichiometry="1"
constant="true"/>
            </listOfReactants>
            <listOfProducts>
                  <speciesReference species="M_malACP_c" stoichiometry="1"
constant="true"/>
                  <speciesReference species="M_coa_c" stoichiometry="1"
constant="true"/>
            </listOfProducts>
            <fbc:geneProductAssociation>
                  <fbc:geneProductRef fbc:geneProduct="SAUSA300_1123"/>
            </fbc:geneProductAssociation>
      </reaction>

      <reaction metaid="R_MDH3" id="R_MDH3" name="Malate dehydrogenase
(menaquinone 8 as acceptor)" reversible="false" fast="false"
fbc:lowerFluxBound="irr_lb" fbc:upperFluxBound="irr_ub">
            <listOfReactants>
                  <speciesReference species="M_mal__L_c" stoichiometry="1"
constant="true"/>
                  <speciesReference species="M_mqn8_c" stoichiometry="1"
constant="true"/>
            </listOfReactants>
            <listOfProducts>
                  <speciesReference species="M_oaa_c" stoichiometry="1"
constant="true"/>
                  <speciesReference species="M_mql8_c" stoichiometry="1"
constant="true"/>
            </listOfProducts>
            <fbc:geneProductAssociation>
                  <fbc:or>
                        <fbc:geneProductRef fbc:geneProduct="SAUSA300_2541"/>
                        <fbc:geneProductRef fbc:geneProduct="SAUSA300_2312"/>
                  </fbc:or>
            </fbc:geneProductAssociation>
      </reaction>

      <reaction metaid="R_MDRPD" id="R_MDRPD" name="5-Methylthio-5__Deoxy__D-
ribulose 1-phosphate dehydratase" reversible="false" fast="false"
fbc:lowerFluxBound="irr_lb" fbc:upperFluxBound="irr_ub">
            <listOfReactants>
```

```
                  <speciesReference species="M_5mdru1p_c" stoichiometry="1"
constant="true"/>
            </listOfReactants>
            <listOfProducts>
                  <speciesReference species="M_h2o_c" stoichiometry="1"
constant="true"/>
                  <speciesReference species="M_dkmpp_c" stoichiometry="1"
constant="true"/>
            </listOfProducts>
      </reaction>

      <reaction metaid="R_ME1_rev" id="R_ME1_rev" name="malic enzyme"
reversible="false" fast="false" fbc:lowerFluxBound="irr_lb"
fbc:upperFluxBound="irr_ub">
            <listOfReactants>
                  <speciesReference species="M_nad_c" stoichiometry="1"
constant="true"/>
                  <speciesReference species="M_mal__L_c" stoichiometry="1"
constant="true"/>
            </listOfReactants>
            <listOfProducts>
                  <speciesReference species="M_co2_c" stoichiometry="1"
constant="true"/>
                  <speciesReference species="M_nadh_c" stoichiometry="1"
constant="true"/>
                  <speciesReference species="M_pyr_c" stoichiometry="1"
constant="true"/>
            </listOfProducts>
            <fbc:geneProductAssociation>
                  <fbc:geneProductRef fbc:geneProduct="SAUSA300_1648"/>
            </fbc:geneProductAssociation>
      </reaction>

      <reaction metaid="R_MELIB_Et" id="R_MELIB_Et" name="Melibiose transport
(transport mechanism unknown - evidence from biolog data)" reversible="true"
fast="false" fbc:lowerFluxBound="rev_lb" fbc:upperFluxBound="rev_ub">
            <listOfReactants>
                  <speciesReference species="M_melib_e" stoichiometry="1"
constant="true"/>
            </listOfReactants>
            <listOfProducts>
                  <speciesReference species="M_melib_c" stoichiometry="1"
constant="true"/>
            </listOfProducts>
      </reaction>

      <reaction metaid="R_MEPCT" id="R_MEPCT" name="2-C-methyl__D-erythritol
4-phosphate cytidylyltransferase" reversible="false" fast="false"
fbc:lowerFluxBound="irr_lb" fbc:upperFluxBound="irr_ub">
            <listOfReactants>
                  <speciesReference species="M_ctp_c" stoichiometry="1"
constant="true"/>
                  <speciesReference species="M_2me4p_c" stoichiometry="1"
constant="true"/>
            </listOfReactants>
            <listOfProducts>
                  <speciesReference species="M_ppi_c" stoichiometry="1"
constant="true"/>
```

```
                  <speciesReference species="M_4c2me_c" stoichiometry="1"
constant="true"/>
            </listOfProducts>
            <fbc:geneProductAssociation>
                  <fbc:or>
                        <fbc:geneProductRef fbc:geneProduct="SAUSA300_0249"/>
                        <fbc:geneProductRef fbc:geneProduct="SAUSA300_0245"/>
                  </fbc:or>
            </fbc:geneProductAssociation>
      </reaction>

      <reaction metaid="R_METabc" id="R_METabc" name="L-methionine transport
via ABC system" reversible="false" fast="false" fbc:lowerFluxBound="irr_lb"
fbc:upperFluxBound="irr_ub">
            <listOfReactants>
                  <speciesReference species="M_h2o_c" stoichiometry="1"
constant="true"/>
                  <speciesReference species="M_atp_c" stoichiometry="1"
constant="true"/>
                  <speciesReference species="M_met__L_e" stoichiometry="1"
constant="true"/>
            </listOfReactants>
            <listOfProducts>
                  <speciesReference species="M_h_c" stoichiometry="1"
constant="true"/>
                  <speciesReference species="M_pi_c" stoichiometry="1"
constant="true"/>
                  <speciesReference species="M_adp_c" stoichiometry="1"
constant="true"/>
                  <speciesReference species="M_met__L_c" stoichiometry="1"
constant="true"/>
            </listOfProducts>
            <fbc:geneProductAssociation>
                  <fbc:and>
                        <fbc:or>
                              <fbc:geneProductRef
fbc:geneProduct="SAUSA300_0437"/>
                              <fbc:geneProductRef
fbc:geneProduct="SAUSA300_0798"/>
                        </fbc:or>
                        <fbc:or>
                              <fbc:geneProductRef
fbc:geneProduct="SAUSA300_0797"/>
                              <fbc:geneProductRef
fbc:geneProduct="SAUSA300_0436"/>
                              <fbc:geneProductRef
fbc:geneProduct="SAUSA300_0435"/>
                        </fbc:or>
                  </fbc:and>
            </fbc:geneProductAssociation>
      </reaction>

      <reaction metaid="R_METALAtr" id="R_METALAtr" name="Dipeptide transport
via ABC system met-ala " reversible="false" fast="false"
fbc:lowerFluxBound="irr_lb" fbc:upperFluxBound="irr_ub">
            <listOfReactants>
                  <speciesReference species="M_h2o_c" stoichiometry="1"
constant="true"/>
```

```
                  <speciesReference species="M_atp_c" stoichiometry="1"
constant="true"/>
                  <speciesReference species="M_met__L_ala__L_e"
stoichiometry="1" constant="true"/>
            </listOfReactants>
            <listOfProducts>
                  <speciesReference species="M_h_c" stoichiometry="1"
constant="true"/>
                  <speciesReference species="M_pi_c" stoichiometry="1"
constant="true"/>
                  <speciesReference species="M_adp_c" stoichiometry="1"
constant="true"/>
                  <speciesReference species="M_met__L_ala__L_c"
stoichiometry="1" constant="true"/>
            </listOfProducts>
            <fbc:geneProductAssociation>
                  <fbc:or>
                        <fbc:geneProductRef fbc:geneProduct="SAUSA300_2411"/>
                        <fbc:geneProductRef fbc:geneProduct="SAUSA300_0200"/>
                        <fbc:geneProductRef fbc:geneProduct="SAUSA300_0889"/>
                        <fbc:geneProductRef fbc:geneProduct="SAUSA300_2410"/>
                        <fbc:geneProductRef fbc:geneProduct="SAUSA300_0890"/>
                        <fbc:geneProductRef fbc:geneProduct="SAUSA300_0888"/>
                        <fbc:geneProductRef fbc:geneProduct="SAUSA300_2409"/>
                        <fbc:geneProductRef fbc:geneProduct="SAUSA300_0887"/>
                        <fbc:geneProductRef fbc:geneProduct="SAUSA300_0893"/>
                        <fbc:geneProductRef fbc:geneProduct="SAUSA300_0895"/>
                        <fbc:geneProductRef fbc:geneProduct="SAUSA300_0891"/>
                        <fbc:geneProductRef fbc:geneProduct="SAUSA300_0896"/>
                        <fbc:geneProductRef fbc:geneProduct="SAUSA300_0712"/>
                  </fbc:or>
            </fbc:geneProductAssociation>
      </reaction>

      <reaction metaid="R_METAT" id="R_METAT" name="methionine
adenosyltransferase" reversible="false" fast="false"
fbc:lowerFluxBound="irr_lb" fbc:upperFluxBound="irr_ub">
            <listOfReactants>
                  <speciesReference species="M_h2o_c" stoichiometry="1"
constant="true"/>
                  <speciesReference species="M_atp_c" stoichiometry="1"
constant="true"/>
                  <speciesReference species="M_met__L_c" stoichiometry="1"
constant="true"/>
            </listOfReactants>
            <listOfProducts>
                  <speciesReference species="M_amet_c" stoichiometry="1"
constant="true"/>
                  <speciesReference species="M_pi_c" stoichiometry="1"
constant="true"/>
                  <speciesReference species="M_ppi_c" stoichiometry="1"
constant="true"/>
            </listOfProducts>
            <fbc:geneProductAssociation>
                  <fbc:geneProductRef fbc:geneProduct="SAUSA300_1730"/>
            </fbc:geneProductAssociation>
      </reaction>
```

```
      <reaction metaid="R_METB1r" id="R_METB1r" name="metb1 (rev)" 
reversible="true" fast="false" fbc:lowerFluxBound="rev_lb" 
fbc:upperFluxBound="rev_ub">
            <listOfReactants>
                  <speciesReference species="M_cys__L_c" stoichiometry="1" 
constant="true"/>
                  <speciesReference species="M_achms_c" stoichiometry="1" 
constant="true"/>
            </listOfReactants>
            <listOfProducts>
                  <speciesReference species="M_ac_c" stoichiometry="1" 
constant="true"/>
                  <speciesReference species="M_cyst__L_c" stoichiometry="1" 
constant="true"/>
            </listOfProducts>
            <fbc:geneProductAssociation>
                  <fbc:geneProductRef fbc:geneProduct="SAUSA300_0360"/>
            </fbc:geneProductAssociation>
      </reaction>

      <reaction metaid="R_METDabc" id="R_METDabc" name="D-methionine 
transport via ABC system" reversible="false" fast="false" 
fbc:lowerFluxBound="irr_lb" fbc:upperFluxBound="irr_ub">
            <listOfReactants>
                  <speciesReference species="M_h2o_c" stoichiometry="1" 
constant="true"/>
                  <speciesReference species="M_atp_c" stoichiometry="1" 
constant="true"/>
                  <speciesReference species="M_met__D_e" stoichiometry="1" 
constant="true"/>
            </listOfReactants>
            <listOfProducts>
                  <speciesReference species="M_h_c" stoichiometry="1" 
constant="true"/>
                  <speciesReference species="M_pi_c" stoichiometry="1" 
constant="true"/>
                  <speciesReference species="M_adp_c" stoichiometry="1" 
constant="true"/>
                  <speciesReference species="M_met__D_c" stoichiometry="1" 
constant="true"/>
            </listOfProducts>
            <fbc:geneProductAssociation>
                  <fbc:or>
                        <fbc:and>
                              <fbc:geneProductRef 
fbc:geneProduct="SAUSA300_0796"/>
                              <fbc:geneProductRef 
fbc:geneProduct="SAUSA300_0798"/>
                              <fbc:geneProductRef 
fbc:geneProduct="SAUSA300_0797"/>
                        </fbc:and>
                        <fbc:and>
                              <fbc:geneProductRef 
fbc:geneProduct="SAUSA300_0435"/>
                              <fbc:geneProductRef 
fbc:geneProduct="SAUSA300_0436"/>
                              <fbc:geneProductRef 
fbc:geneProduct="SAUSA300_0437"/>
```

```
                        </fbc:and>
                  </fbc:or>
            </fbc:geneProductAssociation>
      </reaction>

      <reaction metaid="R_METSOX1abc" id="R_METSOX1abc" name="L-methionine S-
oxide transport via ABC system (periplasm)" reversible="false" fast="false"
fbc:lowerFluxBound="irr_lb" fbc:upperFluxBound="irr_ub">
            <listOfReactants>
                  <speciesReference species="M_h2o_c" stoichiometry="1"
constant="true"/>
                  <speciesReference species="M_atp_c" stoichiometry="1"
constant="true"/>
                  <speciesReference species="M_metsox__S__L_e"
stoichiometry="1" constant="true"/>
            </listOfReactants>
            <listOfProducts>
                  <speciesReference species="M_h_c" stoichiometry="1"
constant="true"/>
                  <speciesReference species="M_pi_c" stoichiometry="1"
constant="true"/>
                  <speciesReference species="M_adp_c" stoichiometry="1"
constant="true"/>
                  <speciesReference species="M_metsox__S__L_c"
stoichiometry="1" constant="true"/>
            </listOfProducts>
            <fbc:geneProductAssociation>
                  <fbc:and>
                        <fbc:or>
                              <fbc:geneProductRef
fbc:geneProduct="SAUSA300_0437"/>
                              <fbc:geneProductRef
fbc:geneProduct="SAUSA300_0798"/>
                        </fbc:or>
                        <fbc:or>
                              <fbc:geneProductRef
fbc:geneProduct="SAUSA300_0797"/>
                              <fbc:geneProductRef
fbc:geneProduct="SAUSA300_0436"/>
                        </fbc:or>
                  </fbc:and>
            </fbc:geneProductAssociation>
      </reaction>

      <reaction metaid="R_METSOX2abc" id="R_METSOX2abc" name="L-methionine R-
oxide transport via ABC system (periplasm)" reversible="false" fast="false"
fbc:lowerFluxBound="irr_lb" fbc:upperFluxBound="irr_ub">
            <listOfReactants>
                  <speciesReference species="M_h2o_c" stoichiometry="1"
constant="true"/>
                  <speciesReference species="M_atp_c" stoichiometry="1"
constant="true"/>
                  <speciesReference species="M_metsox__R__L_e"
stoichiometry="1" constant="true"/>
            </listOfReactants>
            <listOfProducts>
                  <speciesReference species="M_h_c" stoichiometry="1"
constant="true"/>
```

```
                  <speciesReference species="M_pi_c" stoichiometry="1"
constant="true"/>
                  <speciesReference species="M_adp_c" stoichiometry="1"
constant="true"/>
                  <speciesReference species="M_metsox__R__L_c"
stoichiometry="1" constant="true"/>
            </listOfProducts>
            <fbc:geneProductAssociation>
                  <fbc:and>
                        <fbc:or>
                              <fbc:geneProductRef
fbc:geneProduct="SAUSA300_0437"/>
                              <fbc:geneProductRef
fbc:geneProduct="SAUSA300_0798"/>
                        </fbc:or>
                        <fbc:or>
                              <fbc:geneProductRef
fbc:geneProduct="SAUSA300_0797"/>
                              <fbc:geneProductRef
fbc:geneProduct="SAUSA300_0436"/>
                        </fbc:or>
                  </fbc:and>
            </fbc:geneProductAssociation>
      </reaction>

      <reaction metaid="R_METSOXR1" id="R_METSOXR1" name="L-methionine-S-
oxide reductase" reversible="false" fast="false" fbc:lowerFluxBound="irr_lb"
fbc:upperFluxBound="irr_ub">
            <listOfReactants>
                  <speciesReference species="M_h2o_c" stoichiometry="1"
constant="true"/>
                  <speciesReference species="M_trdox_c" stoichiometry="1"
constant="true"/>
                  <speciesReference species="M_met__L_c" stoichiometry="1"
constant="true"/>
            </listOfReactants>
            <listOfProducts>
                  <speciesReference species="M_trdrd_c" stoichiometry="1"
constant="true"/>
                  <speciesReference species="M_metsox__S__L_c"
stoichiometry="1" constant="true"/>
            </listOfProducts>
            <fbc:geneProductAssociation>
                  <fbc:or>
                        <fbc:geneProductRef fbc:geneProduct="SAUSA300_1317"/>
                        <fbc:geneProductRef fbc:geneProduct="SAUSA300_1256"/>
                        <fbc:geneProductRef fbc:geneProduct="SAUSA300_2594"/>
                  </fbc:or>
            </fbc:geneProductAssociation>
      </reaction>

      <reaction metaid="R_METSr" id="R_METSr" name="methionine synthase
reversible" reversible="true" fast="false" fbc:lowerFluxBound="rev_lb"
fbc:upperFluxBound="rev_ub">
            <listOfReactants>
                  <speciesReference species="M_5mthf_c" stoichiometry="1"
constant="true"/>
```

```
                        <speciesReference species="M_hcys__L_c" stoichiometry="1"
constant="true"/>
                  </listOfReactants>
                  <listOfProducts>
                        <speciesReference species="M_thf_c" stoichiometry="1"
constant="true"/>
                        <speciesReference species="M_met__L_c" stoichiometry="1"
constant="true"/>
                  </listOfProducts>
                  <fbc:geneProductAssociation>
                        <fbc:geneProductRef fbc:geneProduct="SAUSA300_0358"/>
                  </fbc:geneProductAssociation>
            </reaction>

            <reaction metaid="R_METt" id="R_METt" name="met/proton antiport"
reversible="true" fast="false" fbc:lowerFluxBound="rev_lb"
fbc:upperFluxBound="rev_ub">
                  <listOfReactants>
                        <speciesReference species="M_h_e" stoichiometry="1"
constant="true"/>
                        <speciesReference species="M_met__L_c" stoichiometry="1"
constant="true"/>
                  </listOfReactants>
                  <listOfProducts>
                        <speciesReference species="M_h_c" stoichiometry="1"
constant="true"/>
                        <speciesReference species="M_met__L_e" stoichiometry="1"
constant="true"/>
                  </listOfProducts>
            </reaction>

            <reaction metaid="R_METTRS" id="R_METTRS" name="Methionyl-tRNA
synthetase" reversible="false" fast="false" fbc:lowerFluxBound="irr_lb"
fbc:upperFluxBound="irr_ub">
                  <listOfReactants>
                        <speciesReference species="M_atp_c" stoichiometry="1"
constant="true"/>
                        <speciesReference species="M_met__L_c" stoichiometry="1"
constant="true"/>
                        <speciesReference species="M_trnamet_c" stoichiometry="1"
constant="true"/>
                  </listOfReactants>
                  <listOfProducts>
                        <speciesReference species="M_ppi_c" stoichiometry="1"
constant="true"/>
                        <speciesReference species="M_amp_c" stoichiometry="1"
constant="true"/>
                        <speciesReference species="M_mettrna_c" stoichiometry="1"
constant="true"/>
                  </listOfProducts>
                  <fbc:geneProductAssociation>
                        <fbc:or>
                              <fbc:geneProductRef fbc:geneProduct="SAUSA300_0467"/>
                              <fbc:geneProductRef fbc:geneProduct="SAUSA300_1109"/>
                              <fbc:geneProductRef fbc:geneProduct="SAUSA300_0959"/>
                        </fbc:or>
                  </fbc:geneProductAssociation>
            </reaction>
```

```
      <reaction metaid="R_MEVK1" id="R_MEVK1" name="mevalonate kinase (atp)"
reversible="false" fast="false" fbc:lowerFluxBound="irr_lb"
fbc:upperFluxBound="irr_ub">
            <listOfReactants>
                  <speciesReference species="M_atp_c" stoichiometry="1"
constant="true"/>
                  <speciesReference species="M_mev__R_c" stoichiometry="1"
constant="true"/>
            </listOfReactants>
            <listOfProducts>
                  <speciesReference species="M_adp_c" stoichiometry="1"
constant="true"/>
                  <speciesReference species="M_5pmev_c" stoichiometry="1"
constant="true"/>
            </listOfProducts>
            <fbc:geneProductAssociation>
                  <fbc:geneProductRef fbc:geneProduct="SAUSA300_0572"/>
            </fbc:geneProductAssociation>
      </reaction>

      <reaction metaid="R_MEVK2" id="R_MEVK2" name="mevalonate kinase (ctp)"
reversible="false" fast="false" fbc:lowerFluxBound="irr_lb"
fbc:upperFluxBound="irr_ub">
            <listOfReactants>
                  <speciesReference species="M_ctp_c" stoichiometry="1"
constant="true"/>
                  <speciesReference species="M_mev__R_c" stoichiometry="1"
constant="true"/>
            </listOfReactants>
            <listOfProducts>
                  <speciesReference species="M_h_c" stoichiometry="1"
constant="true"/>
                  <speciesReference species="M_cdp_c" stoichiometry="1"
constant="true"/>
                  <speciesReference species="M_5pmev_c" stoichiometry="1"
constant="true"/>
            </listOfProducts>
            <fbc:geneProductAssociation>
                  <fbc:geneProductRef fbc:geneProduct="SAUSA300_0572"/>
            </fbc:geneProductAssociation>
      </reaction>

      <reaction metaid="R_MEVK3" id="R_MEVK3" name="mevalonate kinase (gtp)"
reversible="false" fast="false" fbc:lowerFluxBound="irr_lb"
fbc:upperFluxBound="irr_ub">
            <listOfReactants>
                  <speciesReference species="M_mev__R_c" stoichiometry="1"
constant="true"/>
                  <speciesReference species="M_gtp_c" stoichiometry="1"
constant="true"/>
            </listOfReactants>
            <listOfProducts>
                  <speciesReference species="M_h_c" stoichiometry="1"
constant="true"/>
                  <speciesReference species="M_5pmev_c" stoichiometry="1"
constant="true"/>
```

```
                  <speciesReference species="M_gdp_c" stoichiometry="1"
constant="true"/>
            </listOfProducts>
            <fbc:geneProductAssociation>
                  <fbc:geneProductRef fbc:geneProduct="SAUSA300_0572"/>
            </fbc:geneProductAssociation>
      </reaction>

      <reaction metaid="R_MEVK4" id="R_MEVK4" name="mevalonate kinase (utp)"
reversible="false" fast="false" fbc:lowerFluxBound="irr_lb"
fbc:upperFluxBound="irr_ub">
            <listOfReactants>
                  <speciesReference species="M_mev__R_c" stoichiometry="1"
constant="true"/>
                  <speciesReference species="M_utp_c" stoichiometry="1"
constant="true"/>
            </listOfReactants>
            <listOfProducts>
                  <speciesReference species="M_h_c" stoichiometry="1"
constant="true"/>
                  <speciesReference species="M_udp_c" stoichiometry="1"
constant="true"/>
                  <speciesReference species="M_5pmev_c" stoichiometry="1"
constant="true"/>
            </listOfProducts>
            <fbc:geneProductAssociation>
                  <fbc:geneProductRef fbc:geneProduct="SAUSA300_0572"/>
            </fbc:geneProductAssociation>
      </reaction>

      <reaction metaid="R_MG2abc" id="R_MG2abc" name="magnesium transport via
ABC system" reversible="false" fast="false" fbc:lowerFluxBound="irr_lb"
fbc:upperFluxBound="irr_ub">
            <listOfReactants>
                  <speciesReference species="M_h2o_c" stoichiometry="1"
constant="true"/>
                  <speciesReference species="M_atp_c" stoichiometry="1"
constant="true"/>
                  <speciesReference species="M_mg2_e" stoichiometry="1"
constant="true"/>
            </listOfReactants>
            <listOfProducts>
                  <speciesReference species="M_h_c" stoichiometry="1"
constant="true"/>
                  <speciesReference species="M_pi_c" stoichiometry="1"
constant="true"/>
                  <speciesReference species="M_adp_c" stoichiometry="1"
constant="true"/>
                  <speciesReference species="M_mg2_c" stoichiometry="1"
constant="true"/>
            </listOfProducts>
            <fbc:geneProductAssociation>
                  <fbc:geneProductRef fbc:geneProduct="SAUSA300_2323"/>
            </fbc:geneProductAssociation>
      </reaction>
```

```
      <reaction metaid="R_MG2tex" id="R_MG2tex" name="magnesium (Mg+2)
transport via diffusion (extracellular to periplasm)" reversible="false"
fast="false" fbc:lowerFluxBound="irr_lb" fbc:upperFluxBound="irr_ub">
            <listOfReactants>
                  <speciesReference species="M_mg2_e" stoichiometry="1"
constant="true"/>
            </listOfReactants>
            <listOfProducts>
                  <speciesReference species="M_mg2_c" stoichiometry="1"
constant="true"/>
            </listOfProducts>
            <fbc:geneProductAssociation>
                  <fbc:or>
                        <fbc:geneProductRef fbc:geneProduct="SAUSA300_0910"/>
                        <fbc:geneProductRef fbc:geneProduct="SAUSA300_2293"/>
                        <fbc:geneProductRef fbc:geneProduct="SAUSA300_2323"/>
                  </fbc:or>
            </fbc:geneProductAssociation>
      </reaction>

      <reaction metaid="R_MHPGLUT" id="R_MHPGLUT" name="5-
methyltetrahydropteroyltriglutamate-homocysteine S-methyltransferase"
reversible="false" fast="false" fbc:lowerFluxBound="irr_lb"
fbc:upperFluxBound="irr_ub">
            <listOfReactants>
                  <speciesReference species="M_hcys__L_c" stoichiometry="1"
constant="true"/>
                  <speciesReference species="M_mhpglu_c" stoichiometry="1"
constant="true"/>
            </listOfReactants>
            <listOfProducts>
                  <speciesReference species="M_met__L_c" stoichiometry="1"
constant="true"/>
                  <speciesReference species="M_hpglu_c" stoichiometry="1"
constant="true"/>
            </listOfProducts>
            <fbc:geneProductAssociation>
                  <fbc:geneProductRef fbc:geneProduct="SAUSA300_0357"/>
            </fbc:geneProductAssociation>
      </reaction>

      <reaction metaid="R_MHPGLUT2" id="R_MHPGLUT2" name="5-
methyltetrahydropteroyltriglutamate methyltransferase" reversible="true"
fast="false" fbc:lowerFluxBound="rev_lb" fbc:upperFluxBound="rev_ub">
            <listOfReactants>
                  <speciesReference species="M_h_c" stoichiometry="1"
constant="true"/>
                  <speciesReference species="M_ahcys_c" stoichiometry="1"
constant="true"/>
                  <speciesReference species="M_mhpglu_c" stoichiometry="1"
constant="true"/>
            </listOfReactants>
            <listOfProducts>
                  <speciesReference species="M_amet_c" stoichiometry="1"
constant="true"/>
                  <speciesReference species="M_hpglu_c" stoichiometry="1"
constant="true"/>
            </listOfProducts>
```

```
            <fbc:geneProductAssociation>
                  <fbc:geneProductRef fbc:geneProduct="SAUSA300_0357"/>
            </fbc:geneProductAssociation>
      </reaction>

      <reaction metaid="R_MI1PP" id="R_MI1PP" name="myo-inositol 1-
phosphatase" reversible="false" fast="false" fbc:lowerFluxBound="irr_lb"
fbc:upperFluxBound="irr_ub">
            <listOfReactants>
                  <speciesReference species="M_h2o_c" stoichiometry="1"
constant="true"/>
                  <speciesReference species="M_mi1p__D_c" stoichiometry="1"
constant="true"/>
            </listOfReactants>
            <listOfProducts>
                  <speciesReference species="M_h_c" stoichiometry="1"
constant="true"/>
                  <speciesReference species="M_pi_c" stoichiometry="1"
constant="true"/>
                  <speciesReference species="M_inost_c" stoichiometry="1"
constant="true"/>
            </listOfProducts>
            <fbc:geneProductAssociation>
                  <fbc:geneProductRef fbc:geneProduct="SAUSA300_1007"/>
            </fbc:geneProductAssociation>
      </reaction>

      <reaction metaid="R_MI3PP" id="R_MI3PP" name="myo-inositol 3-
phosphatase" reversible="false" fast="false" fbc:lowerFluxBound="irr_lb"
fbc:upperFluxBound="irr_ub">
            <listOfReactants>
                  <speciesReference species="M_h2o_c" stoichiometry="1"
constant="true"/>
                  <speciesReference species="M_mi3p__D_c" stoichiometry="1"
constant="true"/>
            </listOfReactants>
            <listOfProducts>
                  <speciesReference species="M_h_c" stoichiometry="1"
constant="true"/>
                  <speciesReference species="M_pi_c" stoichiometry="1"
constant="true"/>
                  <speciesReference species="M_inost_c" stoichiometry="1"
constant="true"/>
            </listOfProducts>
            <fbc:geneProductAssociation>
                  <fbc:geneProductRef fbc:geneProduct="SAUSA300_1007"/>
            </fbc:geneProductAssociation>
      </reaction>

      <reaction metaid="R_MI4PP" id="R_MI4PP" name="myo-inositol 4-
phosphatase" reversible="false" fast="false" fbc:lowerFluxBound="irr_lb"
fbc:upperFluxBound="irr_ub">
            <listOfReactants>
                  <speciesReference species="M_h2o_c" stoichiometry="1"
constant="true"/>
                  <speciesReference species="M_mi4p__D_c" stoichiometry="1"
constant="true"/>
            </listOfReactants>
```

```
            <listOfProducts>
                  <speciesReference species="M_h_c" stoichiometry="1"
constant="true"/>
                  <speciesReference species="M_pi_c" stoichiometry="1"
constant="true"/>
                  <speciesReference species="M_inost_c" stoichiometry="1"
constant="true"/>
            </listOfProducts>
            <fbc:geneProductAssociation>
                  <fbc:geneProductRef fbc:geneProduct="SAUSA300_1007"/>
            </fbc:geneProductAssociation>
      </reaction>

      <reaction metaid="R_MLTG1" id="R_MLTG1" name="Maltodextrin glucosidase
(maltotriose)" reversible="false" fast="false" fbc:lowerFluxBound="irr_lb"
fbc:upperFluxBound="irr_ub">
            <listOfReactants>
                  <speciesReference species="M_h2o_c" stoichiometry="1"
constant="true"/>
                  <speciesReference species="M_malttr_c" stoichiometry="1"
constant="true"/>
            </listOfReactants>
            <listOfProducts>
                  <speciesReference species="M_glc__D_c" stoichiometry="1"
constant="true"/>
                  <speciesReference species="M_malt_c" stoichiometry="1"
constant="true"/>
            </listOfProducts>
            <fbc:geneProductAssociation>
                  <fbc:geneProductRef fbc:geneProduct="SAUSA300_1456"/>
            </fbc:geneProductAssociation>
      </reaction>

      <reaction metaid="R_MMSAD3" id="R_MMSAD3" name="methylmalonate-
semialdehyde dehydrogenase (malonic semialdehyde)" reversible="false"
fast="false" fbc:lowerFluxBound="irr_lb" fbc:upperFluxBound="irr_ub">
            <listOfReactants>
                  <speciesReference species="M_nad_c" stoichiometry="1"
constant="true"/>
                  <speciesReference species="M_coa_c" stoichiometry="1"
constant="true"/>
                  <speciesReference species="M_msa_c" stoichiometry="1"
constant="true"/>
            </listOfReactants>
            <listOfProducts>
                  <speciesReference species="M_co2_c" stoichiometry="1"
constant="true"/>
                  <speciesReference species="M_nadh_c" stoichiometry="1"
constant="true"/>
                  <speciesReference species="M_accoa_c" stoichiometry="1"
constant="true"/>
            </listOfProducts>
            <fbc:geneProductAssociation>
                  <fbc:geneProductRef fbc:geneProduct="SAUSA300_0225"/>
            </fbc:geneProductAssociation>
      </reaction>
```

```
      <reaction metaid="R_MNabc" id="R_MNabc" name="manganese transport via
ABC system" reversible="false" fast="false" fbc:lowerFluxBound="irr_lb"
fbc:upperFluxBound="irr_ub">
            <listOfReactants>
                  <speciesReference species="M_h2o_c" stoichiometry="1"
constant="true"/>
                  <speciesReference species="M_atp_c" stoichiometry="1"
constant="true"/>
                  <speciesReference species="M_mn2_e" stoichiometry="1"
constant="true"/>
            </listOfReactants>
            <listOfProducts>
                  <speciesReference species="M_h_c" stoichiometry="1"
constant="true"/>
                  <speciesReference species="M_pi_c" stoichiometry="1"
constant="true"/>
                  <speciesReference species="M_adp_c" stoichiometry="1"
constant="true"/>
                  <speciesReference species="M_mn2_c" stoichiometry="1"
constant="true"/>
            </listOfProducts>
            <fbc:geneProductAssociation>
                  <fbc:and>
                        <fbc:geneProductRef fbc:geneProduct="SAUSA300_0620"/>
                        <fbc:geneProductRef fbc:geneProduct="SAUSA300_0619"/>
                        <fbc:geneProductRef fbc:geneProduct="SAUSA300_0618"/>
                  </fbc:and>
            </fbc:geneProductAssociation>
      </reaction>

      <reaction metaid="R_MNLpts" id="R_MNLpts" name="mannitol transport via
PEP:Pyr PTS" reversible="true" fast="false" fbc:lowerFluxBound="rev_lb"
fbc:upperFluxBound="rev_ub">
            <listOfReactants>
                  <speciesReference species="M_pep_c" stoichiometry="1"
constant="true"/>
                  <speciesReference species="M_mnl_e" stoichiometry="1"
constant="true"/>
            </listOfReactants>
            <listOfProducts>
                  <speciesReference species="M_pyr_c" stoichiometry="1"
constant="true"/>
                  <speciesReference species="M_mnl1p_c" stoichiometry="1"
constant="true"/>
            </listOfProducts>
            <fbc:geneProductAssociation>
                  <fbc:and>
                        <fbc:geneProductRef fbc:geneProduct="SAUSA300_2106"/>
                        <fbc:geneProductRef fbc:geneProduct="SAUSA300_2107"/>
                        <fbc:geneProductRef fbc:geneProduct="SAUSA300_2105"/>
                        <fbc:geneProductRef fbc:geneProduct="SAUSA300_0983"/>
                        <fbc:geneProductRef fbc:geneProduct="SAUSA300_0984"/>
                  </fbc:and>
            </fbc:geneProductAssociation>
      </reaction>
```

```
      <reaction metaid="R_MNNH" id="R_MNNH" name="D-mannonate hydrolyase"
reversible="false" fast="false" fbc:lowerFluxBound="irr_lb"
fbc:upperFluxBound="irr_ub">
            &lt;listOfReactants&gt;
                  <speciesReference species="M_mana_c" stoichiometry="1"
constant="true"/>
            &lt;/listOfReactants&gt;
            &lt;listOfProducts&gt;
                  <speciesReference species="M_h2o_c" stoichiometry="1"
constant="true"/>
                  <speciesReference species="M_2ddglcn_c" stoichiometry="1"
constant="true"/>
            &lt;/listOfProducts&gt;
            &lt;fbc:geneProductAssociation&gt;
                  &lt;fbc:or&gt;
                        &lt;fbc:geneProductRef fbc:geneProduct="uxuAB"/&gt;
                        &lt;fbc:geneProductRef fbc:geneProduct="SAUSA300_0229"/&gt;
                  &lt;/fbc:or&gt;
            &lt;/fbc:geneProductAssociation&gt;
      &lt;/reaction&gt;

      <reaction metaid="R_MNt2" id="R_MNt2" name="manganese transport in via
proton symport" reversible="false" fast="false" fbc:lowerFluxBound="irr_lb"
fbc:upperFluxBound="irr_ub">
            &lt;listOfReactants&gt;
                  <speciesReference species="M_h_e" stoichiometry="1"
constant="true"/>
                  <speciesReference species="M_mn2_e" stoichiometry="1"
constant="true"/>
            &lt;/listOfReactants&gt;
            &lt;listOfProducts&gt;
                  <speciesReference species="M_h_c" stoichiometry="1"
constant="true"/>
                  <speciesReference species="M_mn2_c" stoichiometry="1"
constant="true"/>
            &lt;/listOfProducts&gt;
            &lt;fbc:geneProductAssociation&gt;
                  &lt;fbc:geneProductRef fbc:geneProduct="SAUSA300_1005"/&gt;
            &lt;/fbc:geneProductAssociation&gt;
      &lt;/reaction&gt;

      <reaction metaid="R_MOBDabc" id="R_MOBDabc" name="molybdate transport
via ABC system" reversible="false" fast="false" fbc:lowerFluxBound="irr_lb"
fbc:upperFluxBound="irr_ub">
            &lt;listOfReactants&gt;
                  <speciesReference species="M_h2o_c" stoichiometry="1"
constant="true"/>
                  <speciesReference species="M_atp_c" stoichiometry="1"
constant="true"/>
                  <speciesReference species="M_mobd_e" stoichiometry="1"
constant="true"/>
            &lt;/listOfReactants&gt;
            &lt;listOfProducts&gt;
                  <speciesReference species="M_h_c" stoichiometry="1"
constant="true"/>
                  <speciesReference species="M_pi_c" stoichiometry="1"
constant="true"/>
```

```
                  <speciesReference species="M_adp_c" stoichiometry="1"
constant="true"/>
                  <speciesReference species="M_mobd_c" stoichiometry="1"
constant="true"/>
            </listOfProducts>
            <fbc:geneProductAssociation>
                  <fbc:and>
                        <fbc:geneProductRef fbc:geneProduct="SAUSA300_2229"/>
                        <fbc:geneProductRef fbc:geneProduct="SAUSA300_2230"/>
                  </fbc:and>
            </fbc:geneProductAssociation>
      </reaction>

      <reaction metaid="R_MOHMT" id="R_MOHMT" name="3-methyl-2-oxobutanoate
hydroxymethyltransferase" reversible="false" fast="false"
fbc:lowerFluxBound="irr_lb" fbc:upperFluxBound="irr_ub">
            <listOfReactants>
                  <speciesReference species="M_h2o_c" stoichiometry="1"
constant="true"/>
                  <speciesReference species="M_3mob_c" stoichiometry="1"
constant="true"/>
                  <speciesReference species="M_mlthf_c" stoichiometry="1"
constant="true"/>
            </listOfReactants>
            <listOfProducts>
                  <speciesReference species="M_thf_c" stoichiometry="1"
constant="true"/>
                  <speciesReference species="M_2dhp_c" stoichiometry="1"
constant="true"/>
            </listOfProducts>
            <fbc:geneProductAssociation>
                  <fbc:geneProductRef fbc:geneProduct="SAUSA300_2534"/>
            </fbc:geneProductAssociation>
      </reaction>

      <reaction metaid="R_MTAN" id="R_MTAN" name="methylthioadenosine
nucleosidase" reversible="false" fast="false" fbc:lowerFluxBound="irr_lb"
fbc:upperFluxBound="irr_ub">
            <listOfReactants>
                  <speciesReference species="M_h2o_c" stoichiometry="1"
constant="true"/>
                  <speciesReference species="M_5mta_c" stoichiometry="1"
constant="true"/>
            </listOfReactants>
            <listOfProducts>
                  <speciesReference species="M_ade_c" stoichiometry="1"
constant="true"/>
                  <speciesReference species="M_5mtr_c" stoichiometry="1"
constant="true"/>
            </listOfProducts>
            <fbc:geneProductAssociation>
                  <fbc:geneProductRef fbc:geneProduct="SAUSA300_1558"/>
            </fbc:geneProductAssociation>
      </reaction>

      <reaction metaid="R_MTDACP" id="R_MTDACP" name="12-methyl-
tetra__Decanoyl-ACP malonyl-acyl-carrier-protein C-acyltransferase
```

```
decarboxylating " reversible="false" fast="false" fbc:lowerFluxBound="irr_lb"
fbc:upperFluxBound="irr_ub">
            <listOfReactants>
                  <speciesReference species="M_h_c" stoichiometry="1"
constant="true"/>
                  <speciesReference species="M_malACP_c" stoichiometry="1"
constant="true"/>
                  <speciesReference species="M_12methedec_c"
stoichiometry="1" constant="true"/>
            </listOfReactants>
            <listOfProducts>
                  <speciesReference species="M_ACP_c" stoichiometry="1"
constant="true"/>
                  <speciesReference species="M_co2_c" stoichiometry="1"
constant="true"/>
                  <speciesReference species="M_14m3ohdACP_c"
stoichiometry="1" constant="true"/>
            </listOfProducts>
            <fbc:geneProductAssociation>
                  <fbc:and>
                        <fbc:geneProductRef fbc:geneProduct="SAUSA300_0886"/>
                        <fbc:geneProductRef fbc:geneProduct="SAUSA300_0885"/>
                  </fbc:and>
            </fbc:geneProductAssociation>
      </reaction>

      <reaction metaid="R_MTHFC" id="R_MTHFC" name="methenyltetrahydrofolate
cyclohydrolase" reversible="true" fast="false" fbc:lowerFluxBound="rev_lb"
fbc:upperFluxBound="rev_ub">
            <listOfReactants>
                  <speciesReference species="M_h2o_c" stoichiometry="1"
constant="true"/>
                  <speciesReference species="M_methf_c" stoichiometry="1"
constant="true"/>
            </listOfReactants>
            <listOfProducts>
                  <speciesReference species="M_h_c" stoichiometry="1"
constant="true"/>
                  <speciesReference species="M_10fthf_c" stoichiometry="1"
constant="true"/>
            </listOfProducts>
            <fbc:geneProductAssociation>
                  <fbc:or>
                        <fbc:geneProductRef fbc:geneProduct="SAUSA300_0965"/>
                        <fbc:geneProductRef fbc:geneProduct="SAUSA300_1678"/>
                  </fbc:or>
            </fbc:geneProductAssociation>
      </reaction>

      <reaction metaid="R_MTHFD" id="R_MTHFD" name="methylenetetrahydrofolate
dehydrogenase (NADP)" reversible="true" fast="false"
fbc:lowerFluxBound="rev_lb" fbc:upperFluxBound="rev_ub">
            <listOfReactants>
                  <speciesReference species="M_nadp_c" stoichiometry="1"
constant="true"/>
                  <speciesReference species="M_mlthf_c" stoichiometry="1"
constant="true"/>
            </listOfReactants>
```

```
            &lt;listOfProducts&gt;
                  <speciesReference species="M_nadph_c" stoichiometry="1"
constant="true"/>
                  <speciesReference species="M_methf_c" stoichiometry="1"
constant="true"/>
            &lt;/listOfProducts&gt;
            &lt;fbc:geneProductAssociation&gt;
                  &lt;fbc:geneProductRef fbc:geneProduct="SAUSA300_0965"/&gt;
            &lt;/fbc:geneProductAssociation&gt;
      &lt;/reaction&gt;

      <reaction metaid="R_MTHFR3" id="R_MTHFR3" name="5,10-
methylenetetrahydrofolatereductase (NADPH)" reversible="true" fast="false"
fbc:lowerFluxBound="rev_lb" fbc:upperFluxBound="rev_ub">
            &lt;listOfReactants&gt;
                  <speciesReference species="M_nadp_c" stoichiometry="1"
constant="true"/>
                  <speciesReference species="M_5mthf_c" stoichiometry="1"
constant="true"/>
            &lt;/listOfReactants&gt;
            &lt;listOfProducts&gt;
                  <speciesReference species="M_h_c" stoichiometry="1"
constant="true"/>
                  <speciesReference species="M_nadph_c" stoichiometry="1"
constant="true"/>
                  <speciesReference species="M_mlthf_c" stoichiometry="1"
constant="true"/>
            &lt;/listOfProducts&gt;
            &lt;fbc:geneProductAssociation&gt;
                  &lt;fbc:geneProductRef fbc:geneProduct="SAUSA300_0358"/&gt;
            &lt;/fbc:geneProductAssociation&gt;
      &lt;/reaction&gt;

      <reaction metaid="R_MTRK" id="R_MTRK" name="5-methylthioribose kinase"
reversible="false" fast="false" fbc:lowerFluxBound="irr_lb"
fbc:upperFluxBound="irr_ub">
            &lt;listOfReactants&gt;
                  <speciesReference species="M_atp_c" stoichiometry="1"
constant="true"/>
                  <speciesReference species="M_5mtr_c" stoichiometry="1"
constant="true"/>
            &lt;/listOfReactants&gt;
            &lt;listOfProducts&gt;
                  <speciesReference species="M_h_c" stoichiometry="1"
constant="true"/>
                  <speciesReference species="M_adp_c" stoichiometry="1"
constant="true"/>
                  <speciesReference species="M_5mdr1p_c" stoichiometry="1"
constant="true"/>
            &lt;/listOfProducts&gt;
            &lt;fbc:geneProductAssociation&gt;
                  &lt;fbc:geneProductRef fbc:geneProduct="mtnK"/&gt;
            &lt;/fbc:geneProductAssociation&gt;
      &lt;/reaction&gt;

      <reaction metaid="R_MUDPACPO" id="R_MUDPACPO" name="myristoyl-UDP-
glucosyltransferase monoglucosyl " reversible="true" fast="false"
fbc:lowerFluxBound="rev_lb" fbc:upperFluxBound="rev_ub">
```

```
            <listOfReactants>
                  <speciesReference species="M_udpg_c" stoichiometry="1"
constant="true"/>
                  <speciesReference species="M_12dgr140_c" stoichiometry="1"
constant="true"/>
            </listOfReactants>
            <listOfProducts>
                  <speciesReference species="M_m12dglyc_c" stoichiometry="1"
constant="true"/>
                  <speciesReference species="M_udp_c" stoichiometry="1"
constant="true"/>
            </listOfProducts>
            <fbc:geneProductAssociation>
                  <fbc:geneProductRef fbc:geneProduct="SAUSA300_0918"/>
            </fbc:geneProductAssociation>
      </reaction>

      <reaction metaid="R_MYRCS" id="R_MYRCS" name="myristoyl-cardiolipin
synthase " reversible="true" fast="false" fbc:lowerFluxBound="rev_lb"
fbc:upperFluxBound="rev_ub">
            <listOfReactants>
                  <speciesReference species="M_pg140_c" stoichiometry="2"
constant="true"/>
            </listOfReactants>
            <listOfProducts>
                  <speciesReference species="M_glyc_c" stoichiometry="1"
constant="true"/>
                  <speciesReference species="M_mycard_c" stoichiometry="1"
constant="true"/>
            </listOfProducts>
            <fbc:geneProductAssociation>
                  <fbc:or>
                        <fbc:geneProductRef fbc:geneProduct="SAUSA300_2044"/>
                        <fbc:geneProductRef fbc:geneProduct="SAUSA300_1216"/>
                  </fbc:or>
            </fbc:geneProductAssociation>
      </reaction>

      <reaction metaid="R_MYRGLT" id="R_MYRGLT" name="myristoyl-UDP-
glucosyltransferase diglucosyl " reversible="true" fast="false"
fbc:lowerFluxBound="rev_lb" fbc:upperFluxBound="rev_ub">
            <listOfReactants>
                  <speciesReference species="M_udpg_c" stoichiometry="1"
constant="true"/>
                  <speciesReference species="M_m12dglyc_c" stoichiometry="1"
constant="true"/>
            </listOfReactants>
            <listOfProducts>
                  <speciesReference species="M_udp_c" stoichiometry="1"
constant="true"/>
                  <speciesReference species="M_dglu12m_c" stoichiometry="1"
constant="true"/>
            </listOfProducts>
            <fbc:geneProductAssociation>
                  <fbc:geneProductRef fbc:geneProduct="SAUSA300_0918"/>
            </fbc:geneProductAssociation>
      </reaction>
```

```
      <reaction metaid="R_MYRLASNS" id="R_MYRLASNS"
name="myristoyl__Lipoteichoic acid synthesis n=24  linked  N-
acetylglucosamine substituted " reversible="true" fast="false"
fbc:lowerFluxBound="rev_lb" fbc:upperFluxBound="rev_ub">
            <listOfReactants>
                  <speciesReference species="M_myrs24u_c" stoichiometry="1"
constant="true"/>
                  <speciesReference species="M_uacgam_c" stoichiometry="24"
constant="true"/>
            </listOfReactants>
            <listOfProducts>
                  <speciesReference species="M_udp_c" stoichiometry="24"
constant="true"/>
                  <speciesReference species="M_myrs24s_c" stoichiometry="1"
constant="true"/>
            </listOfProducts>
            <fbc:geneProductAssociation>
                  <fbc:geneProductRef fbc:geneProduct="SAUSA300_0731"/>
            </fbc:geneProductAssociation>
      </reaction>

      <reaction metaid="R_MYRLL" id="R_MYRLL" name="myristoyl__Lipoteichoic
acid synthesis n=24  linked  glucose substituted " reversible="true"
fast="false" fbc:lowerFluxBound="rev_lb" fbc:upperFluxBound="rev_ub">
            <listOfReactants>
                  <speciesReference species="M_udpg_c" stoichiometry="24"
constant="true"/>
                  <speciesReference species="M_myrs24u_c" stoichiometry="1"
constant="true"/>
            </listOfReactants>
            <listOfProducts>
                  <speciesReference species="M_udp_c" stoichiometry="24"
constant="true"/>
                  <speciesReference species="M_myrst24s_c" stoichiometry="1"
constant="true"/>
            </listOfProducts>
            <fbc:geneProductAssociation>
                  <fbc:or>
                        <fbc:geneProductRef fbc:geneProduct="SAUSA300_0939"/>
                        <fbc:geneProductRef fbc:geneProduct="SAUSA300_0550"/>
                        <fbc:geneProductRef fbc:geneProduct="SAUSA300_0549"/>
                  </fbc:or>
            </fbc:geneProductAssociation>
      </reaction>

      <reaction metaid="R_NA6PH" id="R_NA6PH" name="N-acetylmuramate 6-
phosphate hydrolase " reversible="true" fast="false"
fbc:lowerFluxBound="rev_lb" fbc:upperFluxBound="rev_ub">
            <listOfReactants>
                  <speciesReference species="M_h2o_c" stoichiometry="1"
constant="true"/>
                  <speciesReference species="M_acmum6p_c" stoichiometry="1"
constant="true"/>
            </listOfReactants>
            <listOfProducts>
                  <speciesReference species="M_lac__D_c" stoichiometry="1"
constant="true"/>
```

```
                  <speciesReference species="M_acgam6p_c" stoichiometry="1"
constant="true"/>
            </listOfProducts>
            <fbc:geneProductAssociation>
                  <fbc:geneProductRef fbc:geneProduct="SAUSA300_0193"/>
            </fbc:geneProductAssociation>
      </reaction>

      <reaction metaid="R_NABTNO" id="R_NABTNO" name="N4-
Acetylaminobutanal:NAD+ oxidoreductase" reversible="false" fast="false"
fbc:lowerFluxBound="irr_lb" fbc:upperFluxBound="irr_ub">
            <listOfReactants>
                  <speciesReference species="M_h2o_c" stoichiometry="1"
constant="true"/>
                  <speciesReference species="M_nad_c" stoichiometry="1"
constant="true"/>
                  <speciesReference species="M_n4abutn_c" stoichiometry="1"
constant="true"/>
            </listOfReactants>
            <listOfProducts>
                  <speciesReference species="M_h_c" stoichiometry="2"
constant="true"/>
                  <speciesReference species="M_nadh_c" stoichiometry="1"
constant="true"/>
                  <speciesReference species="M_4aabutn_c" stoichiometry="1"
constant="true"/>
            </listOfProducts>
            <fbc:geneProductAssociation>
                  <fbc:or>
                        <fbc:geneProductRef fbc:geneProduct="SAUSA300_0170"/>
                        <fbc:geneProductRef fbc:geneProduct="SAUSA300_1901"/>
                        <fbc:geneProductRef fbc:geneProduct="SAUSA300_2076"/>
                  </fbc:or>
            </fbc:geneProductAssociation>
      </reaction>

      <reaction metaid="R_NACUP" id="R_NACUP" name="Nicotinic acid uptake"
reversible="false" fast="false" fbc:lowerFluxBound="irr_lb"
fbc:upperFluxBound="irr_ub">
            <listOfReactants>
                  <speciesReference species="M_nac_e" stoichiometry="1"
constant="true"/>
            </listOfReactants>
            <listOfProducts>
                  <speciesReference species="M_nac_c" stoichiometry="1"
constant="true"/>
            </listOfProducts>
            <fbc:geneProductAssociation>
                  <fbc:geneProductRef fbc:geneProduct="niaX"/>
            </fbc:geneProductAssociation>
      </reaction>

      <reaction metaid="R_NADDPp" id="R_NADDPp" name="NAD diphosphatase,
peroxisomal" reversible="true" fast="false" fbc:lowerFluxBound="rev_lb"
fbc:upperFluxBound="rev_ub">
            <listOfReactants>
                  <speciesReference species="M_h2o_c" stoichiometry="1"
constant="true"/>
```

```
                  <speciesReference species="M_nad_c" stoichiometry="1"
constant="true"/>
            </listOfReactants>
            <listOfProducts>
                  <speciesReference species="M_amp_c" stoichiometry="1"
constant="true"/>
                  <speciesReference species="M_nmn_c" stoichiometry="1"
constant="true"/>
            </listOfProducts>
            <fbc:geneProductAssociation>
                  <fbc:geneProductRef fbc:geneProduct="SAUSA300_1885"/>
            </fbc:geneProductAssociation>
      </reaction>

      <reaction metaid="R_NADH10" id="R_NADH10" name="NADH dehydrogenase
(menaquinone-8 and 0 protons)" reversible="false" fast="false"
fbc:lowerFluxBound="irr_lb" fbc:upperFluxBound="irr_ub">
            <listOfReactants>
                  <speciesReference species="M_h_c" stoichiometry="1"
constant="true"/>
                  <speciesReference species="M_nadh_c" stoichiometry="1"
constant="true"/>
                  <speciesReference species="M_mqn8_c" stoichiometry="1"
constant="true"/>
            </listOfReactants>
            <listOfProducts>
                  <speciesReference species="M_nad_c" stoichiometry="1"
constant="true"/>
                  <speciesReference species="M_mql8_c" stoichiometry="1"
constant="true"/>
            </listOfProducts>
            <fbc:geneProductAssociation>
                  <fbc:or>
                        <fbc:geneProductRef fbc:geneProduct="SAUSA300_0425"/>
                        <fbc:geneProductRef fbc:geneProduct="SAUSA300_0844"/>
                        <fbc:geneProductRef fbc:geneProduct="SAUSA300_0841"/>
                  </fbc:or>
            </fbc:geneProductAssociation>
      </reaction>

      <reaction metaid="R_NADH7" id="R_NADH7" name="NADH dehydrogenase
(menaquinone-8and 2 protons)" reversible="false" fast="false"
fbc:lowerFluxBound="irr_lb" fbc:upperFluxBound="irr_ub">
            <listOfReactants>
                  <speciesReference species="M_h_c" stoichiometry="3"
constant="true"/>
                  <speciesReference species="M_nadh_c" stoichiometry="1"
constant="true"/>
                  <speciesReference species="M_mqn8_c" stoichiometry="1"
constant="true"/>
            </listOfReactants>
            <listOfProducts>
                  <speciesReference species="M_h_c" stoichiometry="2"
constant="true"/>
                  <speciesReference species="M_nad_c" stoichiometry="1"
constant="true"/>
                  <speciesReference species="M_mql8_c" stoichiometry="1"
constant="true"/>
```

```
            </listOfProducts>
            <fbc:geneProductAssociation>
                  <fbc:and>
                        <fbc:geneProductRef fbc:geneProduct="SAUSA300_0855"/>
                        <fbc:geneProductRef fbc:geneProduct="SAUSA300_1724"/>
                        <fbc:geneProductRef fbc:geneProduct="SAUSA300_0841"/>
                        <fbc:geneProductRef fbc:geneProduct="SAUSA300_0844"/>
                        <fbc:geneProductRef fbc:geneProduct="SAUSA300_0425"/>
                        <fbc:geneProductRef fbc:geneProduct="SAUSA300_0610"/>
                  </fbc:and>
            </fbc:geneProductAssociation>
      </reaction>

      <reaction metaid="R_NADK" id="R_NADK" name="NAD kinase"
reversible="false" fast="false" fbc:lowerFluxBound="irr_lb"
fbc:upperFluxBound="irr_ub">
            <listOfReactants>
                  <speciesReference species="M_atp_c" stoichiometry="1"
constant="true"/>
                  <speciesReference species="M_nad_c" stoichiometry="1"
constant="true"/>
            </listOfReactants>
            <listOfProducts>
                  <speciesReference species="M_nadp_c" stoichiometry="1"
constant="true"/>
                  <speciesReference species="M_adp_c" stoichiometry="1"
constant="true"/>
            </listOfProducts>
            <fbc:geneProductAssociation>
                  <fbc:geneProductRef fbc:geneProduct="SAUSA300_0908"/>
            </fbc:geneProductAssociation>
      </reaction>

      <reaction metaid="R_NADKd" id="R_NADKd" name="NAD kinase dTTP "
reversible="false" fast="false" fbc:lowerFluxBound="irr_lb"
fbc:upperFluxBound="irr_ub">
            <listOfReactants>
                  <speciesReference species="M_nad_c" stoichiometry="1"
constant="true"/>
                  <speciesReference species="M_dttp_c" stoichiometry="1"
constant="true"/>
            </listOfReactants>
            <listOfProducts>
                  <speciesReference species="M_nadp_c" stoichiometry="1"
constant="true"/>
                  <speciesReference species="M_dtdp_c" stoichiometry="1"
constant="true"/>
            </listOfProducts>
            <fbc:geneProductAssociation>
                  <fbc:geneProductRef fbc:geneProduct="SAUSA300_0908"/>
            </fbc:geneProductAssociation>
      </reaction>

      <reaction metaid="R_NADS1" id="R_NADS1" name="NAD synthase (nh3)"
reversible="false" fast="false" fbc:lowerFluxBound="irr_lb"
fbc:upperFluxBound="irr_ub">
            <listOfReactants>
```

```
                  <speciesReference species="M_nh4_c" stoichiometry="1"
constant="true"/>
                  <speciesReference species="M_atp_c" stoichiometry="1"
constant="true"/>
                  <speciesReference species="M_dnad_c" stoichiometry="1"
constant="true"/>
            </listOfReactants>
            <listOfProducts>
                  <speciesReference species="M_ppi_c" stoichiometry="1"
constant="true"/>
                  <speciesReference species="M_nad_c" stoichiometry="1"
constant="true"/>
                  <speciesReference species="M_amp_c" stoichiometry="1"
constant="true"/>
            </listOfProducts>
            <fbc:geneProductAssociation>
                  <fbc:geneProductRef fbc:geneProduct="SAUSA300_1893"/>
            </fbc:geneProductAssociation>
      </reaction>

      <reaction metaid="R_NALN6" id="R_NALN6" name="NALN6" reversible="false"
fast="false" fbc:lowerFluxBound="irr_lb" fbc:upperFluxBound="irr_ub">
            <listOfReactants>
                  <speciesReference species="M_nal2a6o_c" stoichiometry="1"
constant="true"/>
                  <speciesReference species="M_2a3pp_c" stoichiometry="1"
constant="true"/>
            </listOfReactants>
            <listOfProducts>
                  <speciesReference species="M_n6all26d_c" stoichiometry="1"
constant="true"/>
                  <speciesReference species="M_3pop_c" stoichiometry="1"
constant="true"/>
            </listOfProducts>
            <fbc:geneProductAssociation>
                  <fbc:or>
                        <fbc:geneProductRef fbc:geneProduct="SAUSA300_1669"/>
                        <fbc:geneProductRef fbc:geneProduct="SAUSA300_0952"/>
                  </fbc:or>
            </fbc:geneProductAssociation>
      </reaction>

      <reaction metaid="R_NAPRTr" id="R_NAPRTr" name="NAPRTase (rev)"
reversible="true" fast="false" fbc:lowerFluxBound="rev_lb"
fbc:upperFluxBound="rev_ub">
            <listOfReactants>
                  <speciesReference species="M_ppi_c" stoichiometry="1"
constant="true"/>
                  <speciesReference species="M_nicrnt_c" stoichiometry="1"
constant="true"/>
            </listOfReactants>
            <listOfProducts>
                  <speciesReference species="M_h_c" stoichiometry="1"
constant="true"/>
                  <speciesReference species="M_nac_c" stoichiometry="1"
constant="true"/>
                  <speciesReference species="M_prpp_c" stoichiometry="1"
constant="true"/>
```

```
			</listOfProducts>
			<fbc:geneProductAssociation>
				<fbc:geneProductRef fbc:geneProduct="SAUSA300_1894"/>
			</fbc:geneProductAssociation>
		</reaction>

		<reaction metaid="R_NAt3" id="R_NAt3" name="sodium transport out via
proton antiport" reversible="false" fast="false" fbc:lowerFluxBound="irr_lb"
fbc:upperFluxBound="irr_ub">
			<listOfReactants>
				<speciesReference species="M_h_e" stoichiometry="1"
constant="true"/>
				<speciesReference species="M_na1_c" stoichiometry="1"
constant="true"/>
			</listOfReactants>
			<listOfProducts>
				<speciesReference species="M_h_c" stoichiometry="1"
constant="true"/>
				<speciesReference species="M_na1_e" stoichiometry="1"
constant="true"/>
			</listOfProducts>
			<fbc:geneProductAssociation>
				<fbc:or>
					<fbc:geneProductRef fbc:geneProduct="SAUSA300_0611"/>
					<fbc:geneProductRef fbc:geneProduct="SAUSA300_0854"/>
					<fbc:geneProductRef fbc:geneProduct="SAUSA300_2384"/>
					<fbc:geneProductRef fbc:geneProduct="SAUSA300_0617"/>
					<fbc:geneProductRef fbc:geneProduct="SAUSA300_0855"/>
					<fbc:geneProductRef fbc:geneProduct="SAUSA300_0610"/>
					<fbc:geneProductRef fbc:geneProduct="SAUSA300_0613"/>
					<fbc:geneProductRef fbc:geneProduct="SAUSA300_0852"/>
					<fbc:geneProductRef fbc:geneProduct="SAUSA300_0614"/>
					<fbc:geneProductRef fbc:geneProduct="SAUSA300_0851"/>
					<fbc:geneProductRef fbc:geneProduct="SAUSA300_0616"/>
					<fbc:geneProductRef fbc:geneProduct="SAUSA300_0849"/>
					<fbc:geneProductRef fbc:geneProduct="SAUSA300_0612"/>
					<fbc:geneProductRef fbc:geneProduct="SAUSA300_0853"/>
					<fbc:geneProductRef fbc:geneProduct="SAUSA300_0615"/>
					<fbc:geneProductRef fbc:geneProduct="SAUSA300_0850"/>
					<fbc:geneProductRef fbc:geneProduct="SAUSA300_2250"/>
					<fbc:geneProductRef fbc:geneProduct="SAUSA300_2384"/>
					<fbc:geneProductRef fbc:geneProduct="SAUSA300_0617"/>
				</fbc:or>
			</fbc:geneProductAssociation>
		</reaction>

		<reaction metaid="R_NAt3_1" id="R_NAt3_1" name="sodium proton
antiporter (H:NA is 1:1)" reversible="false" fast="false"
fbc:lowerFluxBound="irr_lb" fbc:upperFluxBound="irr_ub">
			<listOfReactants>
				<speciesReference species="M_h_e" stoichiometry="1"
constant="true"/>
				<speciesReference species="M_na1_c" stoichiometry="1"
constant="true"/>
			</listOfReactants>
			<listOfProducts>
				<speciesReference species="M_h_c" stoichiometry="1"
constant="true"/>
```

```
                  <speciesReference species="M_na1_e" stoichiometry="1"
constant="true"/>
            </listOfProducts>
            <fbc:geneProductAssociation>
                  <fbc:and>
                        <fbc:geneProductRef fbc:geneProduct="SAUSA300_0426"/>
                        <fbc:geneProductRef fbc:geneProduct="SAUSA300_0427"/>
                        <fbc:geneProductRef fbc:geneProduct="SAUSA300_0425"/>
                  </fbc:and>
            </fbc:geneProductAssociation>
      </reaction>

      <reaction metaid="R_NAt3_15" id="R_NAt3_15" name="sodium proton
antiporter (H:NA is 1.5)" reversible="false" fast="false"
fbc:lowerFluxBound="irr_lb" fbc:upperFluxBound="irr_ub">
            <listOfReactants>
                  <speciesReference species="M_h_e" stoichiometry="3"
constant="true"/>
                  <speciesReference species="M_na1_c" stoichiometry="2"
constant="true"/>
            </listOfReactants>
            <listOfProducts>
                  <speciesReference species="M_h_c" stoichiometry="3"
constant="true"/>
                  <speciesReference species="M_na1_e" stoichiometry="2"
constant="true"/>
            </listOfProducts>
            <fbc:geneProductAssociation>
                  <fbc:or>
                        <fbc:geneProductRef fbc:geneProduct="SAUSA300_2384"/>
                        <fbc:geneProductRef fbc:geneProduct="SAUSA300_0617"/>
                        <fbc:geneProductRef fbc:geneProduct="SAUSA300_0846"/>
                  </fbc:or>
            </fbc:geneProductAssociation>
      </reaction>

      <reaction metaid="R_NAt3_2" id="R_NAt3_2" name="sodium proton
antiporter (H:NA is 2)" reversible="false" fast="false"
fbc:lowerFluxBound="irr_lb" fbc:upperFluxBound="irr_ub">
            <listOfReactants>
                  <speciesReference species="M_h_e" stoichiometry="2"
constant="true"/>
                  <speciesReference species="M_na1_c" stoichiometry="1"
constant="true"/>
            </listOfReactants>
            <listOfProducts>
                  <speciesReference species="M_h_c" stoichiometry="2"
constant="true"/>
                  <speciesReference species="M_na1_e" stoichiometry="1"
constant="true"/>
            </listOfProducts>
            <fbc:geneProductAssociation>
                  <fbc:and>
                        <fbc:geneProductRef fbc:geneProduct="SAUSA300_0851"/>
                        <fbc:geneProductRef fbc:geneProduct="SAUSA300_0852"/>
                        <fbc:geneProductRef fbc:geneProduct="SAUSA300_0849"/>
                        <fbc:geneProductRef fbc:geneProduct="SAUSA300_0850"/>
                        <fbc:geneProductRef fbc:geneProduct="SAUSA300_0855"/>
```

```
                        <fbc:geneProductRef fbc:geneProduct="SAUSA300_0853"/>
                        <fbc:geneProductRef fbc:geneProduct="SAUSA300_0854"/>
                  </fbc:and>
            </fbc:geneProductAssociation>
      </reaction>

      <reaction metaid="R_NBAH" id="R_NBAH" name="N-Benzoylglycine
amidohydrolase" reversible="true" fast="false" fbc:lowerFluxBound="rev_lb"
fbc:upperFluxBound="rev_ub">
            <listOfReactants>
                  <speciesReference species="M_h2o_c" stoichiometry="1"
constant="true"/>
                  <speciesReference species="M_bgly_c" stoichiometry="1"
constant="true"/>
            </listOfReactants>
            <listOfProducts>
                  <speciesReference species="M_bz_c" stoichiometry="1"
constant="true"/>
                  <speciesReference species="M_gly_c" stoichiometry="1"
constant="true"/>
            </listOfProducts>
            <fbc:geneProductAssociation>
                  <fbc:geneProductRef fbc:geneProduct="SAUSA300_1291"/>
            </fbc:geneProductAssociation>
      </reaction>

      <reaction metaid="R_NDPK1" id="R_NDPK1" name="nucleoside__Diphosphate
kinase (ATP:GDP)" reversible="true" fast="false" fbc:lowerFluxBound="rev_lb"
fbc:upperFluxBound="rev_ub">
            <listOfReactants>
                  <speciesReference species="M_atp_c" stoichiometry="1"
constant="true"/>
                  <speciesReference species="M_gdp_c" stoichiometry="1"
constant="true"/>
            </listOfReactants>
            <listOfProducts>
                  <speciesReference species="M_adp_c" stoichiometry="1"
constant="true"/>
                  <speciesReference species="M_gtp_c" stoichiometry="1"
constant="true"/>
            </listOfProducts>
            <fbc:geneProductAssociation>
                  <fbc:geneProductRef fbc:geneProduct="SAUSA300_1358"/>
            </fbc:geneProductAssociation>
      </reaction>

      <reaction metaid="R_NDPK2" id="R_NDPK2" name="nucleoside__Diphosphate
kinase (ATP:UDP)" reversible="true" fast="false" fbc:lowerFluxBound="rev_lb"
fbc:upperFluxBound="rev_ub">
            <listOfReactants>
                  <speciesReference species="M_atp_c" stoichiometry="1"
constant="true"/>
                  <speciesReference species="M_udp_c" stoichiometry="1"
constant="true"/>
            </listOfReactants>
            <listOfProducts>
                  <speciesReference species="M_adp_c" stoichiometry="1"
constant="true"/>
```

```
                  <speciesReference species="M_utp_c" stoichiometry="1"
constant="true"/>
            &lt;/listOfProducts&gt;
            &lt;fbc:geneProductAssociation&gt;
                  &lt;fbc:geneProductRef fbc:geneProduct="SAUSA300_1358"/&gt;
            &lt;/fbc:geneProductAssociation&gt;
      &lt;/reaction&gt;

      <reaction metaid="R_NDPK3" id="R_NDPK3" name="nucleoside__Diphosphate
kinase (ATP:CDP)" reversible="true" fast="false" fbc:lowerFluxBound="rev_lb"
fbc:upperFluxBound="rev_ub">
            &lt;listOfReactants&gt;
                  <speciesReference species="M_atp_c" stoichiometry="1"
constant="true"/>
                  <speciesReference species="M_cdp_c" stoichiometry="1"
constant="true"/>
            &lt;/listOfReactants&gt;
            &lt;listOfProducts&gt;
                  <speciesReference species="M_adp_c" stoichiometry="1"
constant="true"/>
                  <speciesReference species="M_ctp_c" stoichiometry="1"
constant="true"/>
            &lt;/listOfProducts&gt;
            &lt;fbc:geneProductAssociation&gt;
                  &lt;fbc:geneProductRef fbc:geneProduct="SAUSA300_1358"/&gt;
            &lt;/fbc:geneProductAssociation&gt;
      &lt;/reaction&gt;

      <reaction metaid="R_NDPK4" id="R_NDPK4" name="nucleoside__Diphosphate
kinase (ATP:dTDP)" reversible="true" fast="false" fbc:lowerFluxBound="rev_lb"
fbc:upperFluxBound="rev_ub">
            &lt;listOfReactants&gt;
                  <speciesReference species="M_atp_c" stoichiometry="1"
constant="true"/>
                  <speciesReference species="M_dtdp_c" stoichiometry="1"
constant="true"/>
            &lt;/listOfReactants&gt;
            &lt;listOfProducts&gt;
                  <speciesReference species="M_adp_c" stoichiometry="1"
constant="true"/>
                  <speciesReference species="M_dttp_c" stoichiometry="1"
constant="true"/>
            &lt;/listOfProducts&gt;
            &lt;fbc:geneProductAssociation&gt;
                  &lt;fbc:geneProductRef fbc:geneProduct="SAUSA300_1358"/&gt;
            &lt;/fbc:geneProductAssociation&gt;
      &lt;/reaction&gt;

      <reaction metaid="R_NDPK5" id="R_NDPK5" name="nucleoside__Diphosphate
kinase (ATP:dGDP)" reversible="true" fast="false" fbc:lowerFluxBound="rev_lb"
fbc:upperFluxBound="rev_ub">
            &lt;listOfReactants&gt;
                  <speciesReference species="M_atp_c" stoichiometry="1"
constant="true"/>
                  <speciesReference species="M_dgdp_c" stoichiometry="1"
constant="true"/>
            &lt;/listOfReactants&gt;
            &lt;listOfProducts&gt;
```

```
                  <speciesReference species="M_adp_c" stoichiometry="1"
constant="true"/>
                  <speciesReference species="M_dgtp_c" stoichiometry="1"
constant="true"/>
            </listOfProducts>
            <fbc:geneProductAssociation>
                  <fbc:geneProductRef fbc:geneProduct="SAUSA300_1358"/>
            </fbc:geneProductAssociation>
      </reaction>

      <reaction metaid="R_NDPK6" id="R_NDPK6" name="nucleoside__Diphosphate
kinase (ATP:dUDP)" reversible="true" fast="false" fbc:lowerFluxBound="rev_lb"
fbc:upperFluxBound="rev_ub">
            <listOfReactants>
                  <speciesReference species="M_atp_c" stoichiometry="1"
constant="true"/>
                  <speciesReference species="M_dudp_c" stoichiometry="1"
constant="true"/>
            </listOfReactants>
            <listOfProducts>
                  <speciesReference species="M_adp_c" stoichiometry="1"
constant="true"/>
                  <speciesReference species="M_dutp_c" stoichiometry="1"
constant="true"/>
            </listOfProducts>
            <fbc:geneProductAssociation>
                  <fbc:geneProductRef fbc:geneProduct="SAUSA300_1358"/>
            </fbc:geneProductAssociation>
      </reaction>

      <reaction metaid="R_NDPK7" id="R_NDPK7" name="nucleoside__Diphosphate
kinase (ATP:dCDP)" reversible="true" fast="false" fbc:lowerFluxBound="rev_lb"
fbc:upperFluxBound="rev_ub">
            <listOfReactants>
                  <speciesReference species="M_atp_c" stoichiometry="1"
constant="true"/>
                  <speciesReference species="M_dcdp_c" stoichiometry="1"
constant="true"/>
            </listOfReactants>
            <listOfProducts>
                  <speciesReference species="M_adp_c" stoichiometry="1"
constant="true"/>
                  <speciesReference species="M_dctp_c" stoichiometry="1"
constant="true"/>
            </listOfProducts>
            <fbc:geneProductAssociation>
                  <fbc:geneProductRef fbc:geneProduct="SAUSA300_1358"/>
            </fbc:geneProductAssociation>
      </reaction>

      <reaction metaid="R_NDPK8" id="R_NDPK8" name="nucleoside__Diphosphate
kinase (ATP:dADP)" reversible="true" fast="false" fbc:lowerFluxBound="rev_lb"
fbc:upperFluxBound="rev_ub">
            <listOfReactants>
                  <speciesReference species="M_atp_c" stoichiometry="1"
constant="true"/>
                  <speciesReference species="M_dadp_c" stoichiometry="1"
constant="true"/>
```

```
            </listOfReactants>
            <listOfProducts>
                  <speciesReference species="M_adp_c" stoichiometry="1"
constant="true"/>
                  <speciesReference species="M_datp_c" stoichiometry="1"
constant="true"/>
            </listOfProducts>
            <fbc:geneProductAssociation>
                  <fbc:geneProductRef fbc:geneProduct="SAUSA300_1358"/>
            </fbc:geneProductAssociation>
      </reaction>

      <reaction metaid="R_NDPK9" id="R_NDPK9" name="nucleoside__Diphosphate
kinase (ATP:IDP)" reversible="false" fast="false" fbc:lowerFluxBound="irr_lb"
fbc:upperFluxBound="irr_ub">
            <listOfReactants>
                  <speciesReference species="M_atp_c" stoichiometry="1"
constant="true"/>
                  <speciesReference species="M_idp_c" stoichiometry="1"
constant="true"/>
            </listOfReactants>
            <listOfProducts>
                  <speciesReference species="M_adp_c" stoichiometry="1"
constant="true"/>
                  <speciesReference species="M_itp_c" stoichiometry="1"
constant="true"/>
            </listOfProducts>
            <fbc:geneProductAssociation>
                  <fbc:geneProductRef fbc:geneProduct="SAUSA300_1358"/>
            </fbc:geneProductAssociation>
      </reaction>

      <reaction metaid="R_NH4OHDs" id="R_NH4OHDs" name="NH4OH dissociation"
reversible="true" fast="false" fbc:lowerFluxBound="rev_lb"
fbc:upperFluxBound="rev_ub">
            <listOfReactants>
                  <speciesReference species="M_h_c" stoichiometry="1"
constant="true"/>
                  <speciesReference species="M_nh4oh_c" stoichiometry="1"
constant="true"/>
            </listOfReactants>
            <listOfProducts>
                  <speciesReference species="M_h2o_c" stoichiometry="1"
constant="true"/>
                  <speciesReference species="M_nh4_c" stoichiometry="1"
constant="true"/>
            </listOfProducts>
      </reaction>

      <reaction metaid="R_NH4t4" id="R_NH4t4" name="ammonium transport out
via K+ antiport" reversible="false" fast="false" fbc:lowerFluxBound="irr_lb"
fbc:upperFluxBound="irr_ub">
            <listOfReactants>
                  <speciesReference species="M_k_c" stoichiometry="1"
constant="true"/>
                  <speciesReference species="M_nh4_e" stoichiometry="1"
constant="true"/>
            </listOfReactants>
```

```xml
            <listOfProducts>
                  <speciesReference species="M_nh4_c" stoichiometry="1"
constant="true"/>
                  <speciesReference species="M_k_e" stoichiometry="1"
constant="true"/>
            </listOfProducts>
            <fbc:geneProductAssociation>
                  <fbc:geneProductRef fbc:geneProduct="SAUSA300_1996"/>
            </fbc:geneProductAssociation>
      </reaction>

      <reaction metaid="R_NH4tex" id="R_NH4tex" name="ammonia transport via
diffusion (extracellular to periplasm)" reversible="false" fast="false"
fbc:lowerFluxBound="irr_lb" fbc:upperFluxBound="irr_ub">
            <listOfReactants>
                  <speciesReference species="M_nh4_e" stoichiometry="1"
constant="true"/>
            </listOfReactants>
            <listOfProducts>
                  <speciesReference species="M_nh4_c" stoichiometry="1"
constant="true"/>
            </listOfProducts>
            <fbc:geneProductAssociation>
                  <fbc:geneProductRef fbc:geneProduct="SAUSA300_1996"/>
            </fbc:geneProductAssociation>
      </reaction>

      <reaction metaid="R_NIabc" id="R_NIabc" name="nickel transport via ABC
system" reversible="false" fast="false" fbc:lowerFluxBound="irr_lb"
fbc:upperFluxBound="irr_ub">
            <listOfReactants>
                  <speciesReference species="M_h2o_c" stoichiometry="1"
constant="true"/>
                  <speciesReference species="M_atp_c" stoichiometry="1"
constant="true"/>
                  <speciesReference species="M_ni2_e" stoichiometry="1"
constant="true"/>
            </listOfReactants>
            <listOfProducts>
                  <speciesReference species="M_h_c" stoichiometry="1"
constant="true"/>
                  <speciesReference species="M_pi_c" stoichiometry="1"
constant="true"/>
                  <speciesReference species="M_adp_c" stoichiometry="1"
constant="true"/>
                  <speciesReference species="M_ni2_c" stoichiometry="1"
constant="true"/>
            </listOfProducts>
            <fbc:geneProductAssociation>
                  <fbc:or>
                        <fbc:and>
                              <fbc:geneProductRef
fbc:geneProduct="SAUSA300_0231"/>
                              <fbc:geneProductRef
fbc:geneProduct="SAUSA300_0232"/>
                              <fbc:geneProductRef
fbc:geneProduct="SAUSA300_0230"/>
                        </fbc:and>
```

```
                        <fbc:geneProductRef fbc:geneProduct="SAUSA300_2630"/>
                        <fbc:and>
                              <fbc:geneProductRef
fbc:geneProduct="SAUSA300_1276"/>
                              <fbc:geneProductRef
fbc:geneProduct="SAUSA300_1275"/>
                              <fbc:geneProductRef
fbc:geneProduct="SAUSA300_1274"/>
                              <fbc:geneProductRef
fbc:geneProduct="SAUSA300_1273"/>
                              <fbc:geneProductRef
fbc:geneProduct="SAUSA300_0231"/>
                        </fbc:and>
                  </fbc:or>
            </fbc:geneProductAssociation>
      </reaction>

      <reaction metaid="R_NMNHYD" id="R_NMNHYD" name="Nicotinamide
ribonucleotide phosphohydrolase" reversible="false" fast="false"
fbc:lowerFluxBound="irr_lb" fbc:upperFluxBound="irr_ub">
            <listOfReactants>
                  <speciesReference species="M_h2o_c" stoichiometry="1"
constant="true"/>
                  <speciesReference species="M_nmn_c" stoichiometry="1"
constant="true"/>
            </listOfReactants>
            <listOfProducts>
                  <speciesReference species="M_h_c" stoichiometry="1"
constant="true"/>
                  <speciesReference species="M_pi_c" stoichiometry="1"
constant="true"/>
                  <speciesReference species="M_rnam_c" stoichiometry="1"
constant="true"/>
            </listOfProducts>
            <fbc:geneProductAssociation>
                  <fbc:or>
                        <fbc:geneProductRef fbc:geneProduct="SAUSA300_0828"/>
                        <fbc:geneProductRef fbc:geneProduct="SAUSA300_0025"/>
                  </fbc:or>
            </fbc:geneProductAssociation>
      </reaction>

      <reaction metaid="R_NNAM" id="R_NNAM" name="nicotinamidase"
reversible="false" fast="false" fbc:lowerFluxBound="irr_lb"
fbc:upperFluxBound="irr_ub">
            <listOfReactants>
                  <speciesReference species="M_h2o_c" stoichiometry="1"
constant="true"/>
                  <speciesReference species="M_ncam_c" stoichiometry="1"
constant="true"/>
            </listOfReactants>
            <listOfProducts>
                  <speciesReference species="M_nh4_c" stoichiometry="1"
constant="true"/>
                  <speciesReference species="M_nac_c" stoichiometry="1"
constant="true"/>
            </listOfProducts>
            <fbc:geneProductAssociation>
```

```
                &lt;fbc:geneProductRef fbc:geneProduct="SAUSA300_1899"/&gt;
            &lt;/fbc:geneProductAssociation&gt;
        &lt;/reaction&gt;

        <reaction metaid="R_NNATr" id="R_NNATr" name="nicotinate-nucleotide
adenylyltransferase" reversible="true" fast="false"
fbc:lowerFluxBound="rev_lb" fbc:upperFluxBound="rev_ub">
            &lt;listOfReactants&gt;
                <speciesReference species="M_atp_c" stoichiometry="1"
constant="true"/>
                <speciesReference species="M_nicrnt_c" stoichiometry="1"
constant="true"/>
            &lt;/listOfReactants&gt;
            &lt;listOfProducts&gt;
                <speciesReference species="M_ppi_c" stoichiometry="1"
constant="true"/>
                <speciesReference species="M_dnad_c" stoichiometry="1"
constant="true"/>
            &lt;/listOfProducts&gt;
            &lt;fbc:geneProductAssociation&gt;
                &lt;fbc:geneProductRef fbc:geneProduct="SAUSA300_1553"/&gt;
            &lt;/fbc:geneProductAssociation&gt;
        &lt;/reaction&gt;

        <reaction metaid="R_NO2t" id="R_NO2t" name="nitrite transport in via
proton symport " reversible="false" fast="false" fbc:lowerFluxBound="irr_lb"
fbc:upperFluxBound="irr_ub">
            &lt;listOfReactants&gt;
                <speciesReference species="M_h_e" stoichiometry="1"
constant="true"/>
                <speciesReference species="M_no2_e" stoichiometry="1"
constant="true"/>
            &lt;/listOfReactants&gt;
            &lt;listOfProducts&gt;
                <speciesReference species="M_h_c" stoichiometry="1"
constant="true"/>
                <speciesReference species="M_no2_c" stoichiometry="1"
constant="true"/>
            &lt;/listOfProducts&gt;
            &lt;fbc:geneProductAssociation&gt;
                &lt;fbc:or&gt;
                    &lt;fbc:geneProductRef fbc:geneProduct="SAUSA300_2349"/&gt;
                    &lt;fbc:geneProductRef fbc:geneProduct="SAUSA300_0305"/&gt;
                    &lt;fbc:geneProductRef fbc:geneProduct="SAUSA300_2333"/&gt;
                &lt;/fbc:or&gt;
            &lt;/fbc:geneProductAssociation&gt;
        &lt;/reaction&gt;

        <reaction metaid="R_NO3R2" id="R_NO3R2" name="Nitrate reductase
(Menaquinol-8)" reversible="true" fast="false" fbc:lowerFluxBound="rev_lb"
fbc:upperFluxBound="rev_ub">
            &lt;listOfReactants&gt;
                <speciesReference species="M_h_c" stoichiometry="2"
constant="true"/>
                <speciesReference species="M_no3_c" stoichiometry="1"
constant="true"/>
                <speciesReference species="M_mql8_c" stoichiometry="1"
constant="true"/>
```

```
            </listOfReactants>
            <listOfProducts>
                  <speciesReference species="M_h_e" stoichiometry="2"
constant="true"/>
                  <speciesReference species="M_h2o_c" stoichiometry="1"
constant="true"/>
                  <speciesReference species="M_no2_c" stoichiometry="1"
constant="true"/>
                  <speciesReference species="M_mqn8_c" stoichiometry="1"
constant="true"/>
            </listOfProducts>
            <fbc:geneProductAssociation>
                  <fbc:or>
                        <fbc:and>
                              <fbc:geneProductRef
fbc:geneProduct="SAUSA300_0708"/>
                              <fbc:geneProductRef
fbc:geneProduct="SAUSA300_2610"/>
                        </fbc:and>
                        <fbc:geneProductRef fbc:geneProduct="SAUSA300_1916"/>
                  </fbc:or>
            </fbc:geneProductAssociation>
      </reaction>

      <reaction metaid="R_NO3R2pp" id="R_NO3R2pp" name="Nitrate reductase
(Menaquinol-8) (periplasm)" reversible="false" fast="false"
fbc:lowerFluxBound="irr_lb" fbc:upperFluxBound="irr_ub">
            <listOfReactants>
                  <speciesReference species="M_h_c" stoichiometry="2"
constant="true"/>
                  <speciesReference species="M_no3_c" stoichiometry="1"
constant="true"/>
                  <speciesReference species="M_mql8_c" stoichiometry="1"
constant="true"/>
            </listOfReactants>
            <listOfProducts>
                  <speciesReference species="M_h2o_c" stoichiometry="1"
constant="true"/>
                  <speciesReference species="M_no2_c" stoichiometry="1"
constant="true"/>
                  <speciesReference species="M_mqn8_c" stoichiometry="1"
constant="true"/>
                  <speciesReference species="M_h_p" stoichiometry="2"
constant="true"/>
            </listOfProducts>
            <fbc:geneProductAssociation>
                  <fbc:and>
                        <fbc:geneProductRef fbc:geneProduct="SAUSA300_2342"/>
                        <fbc:geneProductRef fbc:geneProduct="SAUSA300_2343"/>
                        <fbc:geneProductRef fbc:geneProduct="SAUSA300_2341"/>
                        <fbc:geneProductRef fbc:geneProduct="SAUSA300_2340"/>
                  </fbc:and>
            </fbc:geneProductAssociation>
      </reaction>

      <reaction metaid="R_NO3t3" id="R_NO3t3" name="nitrate transport"
reversible="false" fast="false" fbc:lowerFluxBound="irr_lb"
fbc:upperFluxBound="irr_ub">
```

```
            <listOfReactants>
                  <speciesReference species="M_h_c" stoichiometry="1"
constant="true"/>
                  <speciesReference species="M_no3_c" stoichiometry="1"
constant="true"/>
            </listOfReactants>
            <listOfProducts>
                  <speciesReference species="M_h_e" stoichiometry="1"
constant="true"/>
                  <speciesReference species="M_no3_e" stoichiometry="1"
constant="true"/>
            </listOfProducts>
            <fbc:geneProductAssociation>
                  <fbc:geneProductRef fbc:geneProduct="SAUSA300_2333"/>
            </fbc:geneProductAssociation>
      </reaction>

      <reaction metaid="R_NO3t7" id="R_NO3t7" name="nitrate transport in via
nitrite antiport" reversible="false" fast="false" fbc:lowerFluxBound="irr_lb"
fbc:upperFluxBound="irr_ub">
            <listOfReactants>
                  <speciesReference species="M_no2_c" stoichiometry="1"
constant="true"/>
                  <speciesReference species="M_no3_e" stoichiometry="1"
constant="true"/>
            </listOfReactants>
            <listOfProducts>
                  <speciesReference species="M_no3_c" stoichiometry="1"
constant="true"/>
                  <speciesReference species="M_no2_e" stoichiometry="1"
constant="true"/>
            </listOfProducts>
            <fbc:geneProductAssociation>
                  <fbc:geneProductRef fbc:geneProduct="SAUSA300_2333"/>
            </fbc:geneProductAssociation>
      </reaction>

      <reaction metaid="R_NODOx" id="R_NODOx" name="nitric oxide dioxygenase"
reversible="false" fast="false" fbc:lowerFluxBound="irr_lb"
fbc:upperFluxBound="irr_ub">
            <listOfReactants>
                  <speciesReference species="M_no_c" stoichiometry="2"
constant="true"/>
                  <speciesReference species="M_o2_c" stoichiometry="2"
constant="true"/>
                  <speciesReference species="M_nadh_c" stoichiometry="1"
constant="true"/>
            </listOfReactants>
            <listOfProducts>
                  <speciesReference species="M_h_c" stoichiometry="1"
constant="true"/>
                  <speciesReference species="M_no3_c" stoichiometry="2"
constant="true"/>
                  <speciesReference species="M_nad_c" stoichiometry="1"
constant="true"/>
            </listOfProducts>
            <fbc:geneProductAssociation>
                  <fbc:geneProductRef fbc:geneProduct="SAUSA300_0234"/>
```

```
            </fbc:geneProductAssociation>
      </reaction>

      <reaction metaid="R_NODOy" id="R_NODOy" name="nitric oxide dioxygenase"
reversible="false" fast="false" fbc:lowerFluxBound="irr_lb"
fbc:upperFluxBound="irr_ub">
            <listOfReactants>
                  <speciesReference species="M_nadph_c" stoichiometry="1"
constant="true"/>
                  <speciesReference species="M_no_c" stoichiometry="2"
constant="true"/>
                  <speciesReference species="M_o2_c" stoichiometry="2"
constant="true"/>
            </listOfReactants>
            <listOfProducts>
                  <speciesReference species="M_h_c" stoichiometry="1"
constant="true"/>
                  <speciesReference species="M_nadp_c" stoichiometry="1"
constant="true"/>
                  <speciesReference species="M_no3_c" stoichiometry="2"
constant="true"/>
            </listOfProducts>
            <fbc:geneProductAssociation>
                  <fbc:geneProductRef fbc:geneProduct="SAUSA300_0234"/>
            </fbc:geneProductAssociation>
      </reaction>

      <reaction metaid="R_NOPD" id="R_NOPD" name="D-nopaline dehydrogenase"
reversible="false" fast="false" fbc:lowerFluxBound="irr_lb"
fbc:upperFluxBound="irr_ub">
            <listOfReactants>
                  <speciesReference species="M_h2o_c" stoichiometry="1"
constant="true"/>
                  <speciesReference species="M_nadp_c" stoichiometry="1"
constant="true"/>
                  <speciesReference species="M_nop_c" stoichiometry="1"
constant="true"/>
            </listOfReactants>
            <listOfProducts>
                  <speciesReference species="M_h_c" stoichiometry="1"
constant="true"/>
                  <speciesReference species="M_nadph_c" stoichiometry="1"
constant="true"/>
                  <speciesReference species="M_akg_c" stoichiometry="1"
constant="true"/>
                  <speciesReference species="M_arg__L_c" stoichiometry="1"
constant="true"/>
            </listOfProducts>
            <fbc:geneProductAssociation>
                  <fbc:geneProductRef fbc:geneProduct="SAUSA300_2251"/>
            </fbc:geneProductAssociation>
      </reaction>

      <reaction metaid="R_NOS1" id="R_NOS1" name="Nitric Oxide Synthase
(intermediate forming)" reversible="false" fast="false"
fbc:lowerFluxBound="irr_lb" fbc:upperFluxBound="irr_ub">
            <listOfReactants>
```

```
                  <speciesReference species="M_nadph_c" stoichiometry="1"
constant="true"/>
                  <speciesReference species="M_o2_c" stoichiometry="1"
constant="true"/>
                  <speciesReference species="M_arg__L_c" stoichiometry="1"
constant="true"/>
            </listOfReactants>
            <listOfProducts>
                  <speciesReference species="M_h2o_c" stoichiometry="1"
constant="true"/>
                  <speciesReference species="M_nadp_c" stoichiometry="1"
constant="true"/>
                  <speciesReference species="M_nwharg_c" stoichiometry="1"
constant="true"/>
            </listOfProducts>
            <fbc:geneProductAssociation>
                  <fbc:geneProductRef fbc:geneProduct="SAUSA300_1895"/>
            </fbc:geneProductAssociation>
      </reaction>

      <reaction metaid="R_NOS3" id="R_NOS3" name="N-omega-Hydroxyarginine
NADPH oxygen oxidoreductase " reversible="true" fast="false"
fbc:lowerFluxBound="rev_lb" fbc:upperFluxBound="rev_ub">
            <listOfReactants>
                  <speciesReference species="M_nadph_c" stoichiometry="1"
constant="true"/>
                  <speciesReference species="M_o2_c" stoichiometry="1"
constant="true"/>
                  <speciesReference species="M_nwharg_c" stoichiometry="2"
constant="true"/>
            </listOfReactants>
            <listOfProducts>
                  <speciesReference species="M_h_c" stoichiometry="3"
constant="true"/>
                  <speciesReference species="M_nadp_c" stoichiometry="1"
constant="true"/>
                  <speciesReference species="M_no_c" stoichiometry="2"
constant="true"/>
                  <speciesReference species="M_citr__L_c" stoichiometry="2"
constant="true"/>
            </listOfProducts>
            <fbc:geneProductAssociation>
                  <fbc:geneProductRef fbc:geneProduct="SAUSA300_1895"/>
            </fbc:geneProductAssociation>
      </reaction>

      <reaction metaid="R_NP1" id="R_NP1" name="nucleotide phosphatase"
reversible="true" fast="false" fbc:lowerFluxBound="rev_lb"
fbc:upperFluxBound="rev_ub">
            <listOfReactants>
                  <speciesReference species="M_pi_c" stoichiometry="1"
constant="true"/>
                  <speciesReference species="M_nicrns_c" stoichiometry="1"
constant="true"/>
            </listOfReactants>
            <listOfProducts>
                  <speciesReference species="M_r1p_c" stoichiometry="1"
constant="true"/>
```

<speciesReference species="M_nac_c" stoichiometry="1"
constant="true"/>
</listOfProducts>
<fbc:geneProductAssociation>
<fbc:or>
<fbc:geneProductRef fbc:geneProduct="SAUSA300_0138"/>
<fbc:geneProductRef fbc:geneProduct="SAUSA300_2091"/>
</fbc:or>
</fbc:geneProductAssociation>
</reaction>

<reaction metaid="R_NPHS2" id="R_NPHS2" name="rxn05024 "
reversible="false" fast="false" fbc:lowerFluxBound="irr_lb"
fbc:upperFluxBound="irr_ub">
<listOfReactants>
<speciesReference species="M_h_c" stoichiometry="1"
constant="true"/>
<speciesReference species="M_sbzcoa_c" stoichiometry="1"
constant="true"/>
</listOfReactants>
<listOfProducts>
<speciesReference species="M_h2o_c" stoichiometry="1"
constant="true"/>
<speciesReference species="M_14dhncoa_c" stoichiometry="1"
constant="true"/>
</listOfProducts>
<fbc:geneProductAssociation>
<fbc:geneProductRef fbc:geneProduct="SAUSA300_0948"/>
</fbc:geneProductAssociation>
</reaction>

<reaction metaid="R_NPHSr" id="R_NPHSr" name="naphthoate synthase
(reversible)" reversible="true" fast="false" fbc:lowerFluxBound="rev_lb"
fbc:upperFluxBound="rev_ub">
<listOfReactants>
<speciesReference species="M_sbzcoa_c" stoichiometry="1"
constant="true"/>
</listOfReactants>
<listOfProducts>
<speciesReference species="M_coa_c" stoichiometry="1"
constant="true"/>
<speciesReference species="M_dhna_c" stoichiometry="1"
constant="true"/>
</listOfProducts>
<fbc:geneProductAssociation>
<fbc:geneProductRef fbc:geneProduct="SAUSA300_0948"/>
</fbc:geneProductAssociation>
</reaction>

<reaction metaid="R_NTD1" id="R_NTD1" name="5'-nucleotidase (dUMP)"
reversible="false" fast="false" fbc:lowerFluxBound="irr_lb"
fbc:upperFluxBound="irr_ub">
<listOfReactants>
<speciesReference species="M_h2o_c" stoichiometry="1"
constant="true"/>
<speciesReference species="M_dump_c" stoichiometry="1"
constant="true"/>
</listOfReactants>

```
			<listOfProducts>
				<speciesReference species="M_h_c" stoichiometry="1"
constant="true"/>
				<speciesReference species="M_pi_c" stoichiometry="1"
constant="true"/>
				<speciesReference species="M_duri_c" stoichiometry="1"
constant="true"/>
			</listOfProducts>
			<fbc:geneProductAssociation>
				<fbc:or>
					<fbc:geneProductRef fbc:geneProduct="SAUSA300_0025"/>
					<fbc:geneProductRef fbc:geneProduct="SAUSA300_2073"/>
				</fbc:or>
			</fbc:geneProductAssociation>
		</reaction>

		<reaction metaid="R_NTD4pp" id="R_NTD4pp" name="5'-nucleotidase (CMP)"
reversible="false" fast="false" fbc:lowerFluxBound="irr_lb"
fbc:upperFluxBound="irr_ub">
			<listOfReactants>
				<speciesReference species="M_h2o_c" stoichiometry="1"
constant="true"/>
				<speciesReference species="M_cmp_c" stoichiometry="1"
constant="true"/>
			</listOfReactants>
			<listOfProducts>
				<speciesReference species="M_h_c" stoichiometry="1"
constant="true"/>
				<speciesReference species="M_pi_c" stoichiometry="1"
constant="true"/>
				<speciesReference species="M_cytd_c" stoichiometry="1"
constant="true"/>
			</listOfProducts>
			<fbc:geneProductAssociation>
				<fbc:or>
					<fbc:geneProductRef fbc:geneProduct="SAUSA300_0828"/>
					<fbc:geneProductRef fbc:geneProduct="SAUSA300_0925"/>
				</fbc:or>
			</fbc:geneProductAssociation>
		</reaction>

		<reaction metaid="R_NTD6pp" id="R_NTD6pp" name="5'-nucleotidase (dAMP)"
reversible="false" fast="false" fbc:lowerFluxBound="irr_lb"
fbc:upperFluxBound="irr_ub">
			<listOfReactants>
				<speciesReference species="M_h2o_c" stoichiometry="1"
constant="true"/>
				<speciesReference species="M_damp_c" stoichiometry="1"
constant="true"/>
			</listOfReactants>
			<listOfProducts>
				<speciesReference species="M_h_c" stoichiometry="1"
constant="true"/>
				<speciesReference species="M_pi_c" stoichiometry="1"
constant="true"/>
				<speciesReference species="M_dad__2_c" stoichiometry="1"
constant="true"/>
			</listOfProducts>
```

```
            <fbc:geneProductAssociation>
                  <fbc:or>
                        <fbc:geneProductRef fbc:geneProduct="SAUSA300_0828"/>
                        <fbc:geneProductRef fbc:geneProduct="SAUSA300_0925"/>
                  </fbc:or>
            </fbc:geneProductAssociation>
      </reaction>

      <reaction metaid="R_NTPP1" id="R_NTPP1" name="Nucleoside triphosphate
pyrophosphorylase (dgtp)" reversible="false" fast="false"
fbc:lowerFluxBound="irr_lb" fbc:upperFluxBound="irr_ub">
            <listOfReactants>
                  <speciesReference species="M_h2o_c" stoichiometry="1"
constant="true"/>
                  <speciesReference species="M_dgtp_c" stoichiometry="1"
constant="true"/>
            </listOfReactants>
            <listOfProducts>
                  <speciesReference species="M_ppi_c" stoichiometry="1"
constant="true"/>
                  <speciesReference species="M_dgmp_c" stoichiometry="1"
constant="true"/>
            </listOfProducts>
            <fbc:geneProductAssociation>
                  <fbc:geneProductRef fbc:geneProduct="SAUSA300_1050"/>
            </fbc:geneProductAssociation>
      </reaction>

      <reaction metaid="R_NTPP10" id="R_NTPP10" name="Nucleoside triphosphate
pyrophosphorylase (ditp)" reversible="false" fast="false"
fbc:lowerFluxBound="irr_lb" fbc:upperFluxBound="irr_ub">
            <listOfReactants>
                  <speciesReference species="M_h2o_c" stoichiometry="1"
constant="true"/>
                  <speciesReference species="M_ditp_c" stoichiometry="1"
constant="true"/>
            </listOfReactants>
            <listOfProducts>
                  <speciesReference species="M_ppi_c" stoichiometry="1"
constant="true"/>
                  <speciesReference species="M_dimp_c" stoichiometry="1"
constant="true"/>
            </listOfProducts>
            <fbc:geneProductAssociation>
                  <fbc:geneProductRef fbc:geneProduct="SAUSA300_1050"/>
            </fbc:geneProductAssociation>
      </reaction>

      <reaction metaid="R_NTPP11" id="R_NTPP11" name="Nucleoside triphosphate
pyrophosphorylase (xtp)" reversible="false" fast="false"
fbc:lowerFluxBound="irr_lb" fbc:upperFluxBound="irr_ub">
            <listOfReactants>
                  <speciesReference species="M_h2o_c" stoichiometry="1"
constant="true"/>
                  <speciesReference species="M_xtp_c" stoichiometry="1"
constant="true"/>
            </listOfReactants>
            <listOfProducts>
```

```
                  <speciesReference species="M_ppi_c" stoichiometry="1"
constant="true"/>
                  <speciesReference species="M_xmp_c" stoichiometry="1"
constant="true"/>
            </listOfProducts>
            <fbc:geneProductAssociation>
                  <fbc:geneProductRef fbc:geneProduct="SAUSA300_1050"/>
            </fbc:geneProductAssociation>
      </reaction>

      <reaction metaid="R_NTPP2" id="R_NTPP2" name="Nucleoside triphosphate
pyrophosphorylase (gtp)" reversible="false" fast="false"
fbc:lowerFluxBound="irr_lb" fbc:upperFluxBound="irr_ub">
            <listOfReactants>
                  <speciesReference species="M_h2o_c" stoichiometry="1"
constant="true"/>
                  <speciesReference species="M_gtp_c" stoichiometry="1"
constant="true"/>
            </listOfReactants>
            <listOfProducts>
                  <speciesReference species="M_ppi_c" stoichiometry="1"
constant="true"/>
                  <speciesReference species="M_gmp_c" stoichiometry="1"
constant="true"/>
            </listOfProducts>
            <fbc:geneProductAssociation>
                  <fbc:geneProductRef fbc:geneProduct="SAUSA300_1050"/>
            </fbc:geneProductAssociation>
      </reaction>

      <reaction metaid="R_NTPP8" id="R_NTPP8" name="Nucleoside triphosphate
pyrophosphorylase (utp)" reversible="false" fast="false"
fbc:lowerFluxBound="irr_lb" fbc:upperFluxBound="irr_ub">
            <listOfReactants>
                  <speciesReference species="M_h2o_c" stoichiometry="1"
constant="true"/>
                  <speciesReference species="M_utp_c" stoichiometry="1"
constant="true"/>
            </listOfReactants>
            <listOfProducts>
                  <speciesReference species="M_ppi_c" stoichiometry="1"
constant="true"/>
                  <speciesReference species="M_ump_c" stoichiometry="1"
constant="true"/>
            </listOfProducts>
            <fbc:geneProductAssociation>
                  <fbc:geneProductRef fbc:geneProduct="SAUSA300_1050"/>
            </fbc:geneProductAssociation>
      </reaction>

      <reaction metaid="R_NTPP9" id="R_NTPP9" name="Nucleoside triphosphate
pyrophosphorylase (itp)" reversible="false" fast="false"
fbc:lowerFluxBound="irr_lb" fbc:upperFluxBound="irr_ub">
            <listOfReactants>
                  <speciesReference species="M_h2o_c" stoichiometry="1"
constant="true"/>
                  <speciesReference species="M_itp_c" stoichiometry="1"
constant="true"/>
```

```
            </listOfReactants>
            <listOfProducts>
                  <speciesReference species="M_ppi_c" stoichiometry="1"
constant="true"/>
                  <speciesReference species="M_imp_c" stoichiometry="1"
constant="true"/>
            </listOfProducts>
            <fbc:geneProductAssociation>
                  <fbc:geneProductRef fbc:geneProduct="SAUSA300_1050"/>
            </fbc:geneProductAssociation>
      </reaction>

      <reaction metaid="R_NTRIR2x" id="R_NTRIR2x" name="nitrite Reductase
(NADH)" reversible="false" fast="false" fbc:lowerFluxBound="irr_lb"
fbc:upperFluxBound="irr_ub">
            <listOfReactants>
                  <speciesReference species="M_h_c" stoichiometry="5"
constant="true"/>
                  <speciesReference species="M_nadh_c" stoichiometry="3"
constant="true"/>
                  <speciesReference species="M_no2_c" stoichiometry="1"
constant="true"/>
            </listOfReactants>
            <listOfProducts>
                  <speciesReference species="M_h2o_c" stoichiometry="2"
constant="true"/>
                  <speciesReference species="M_nh4_c" stoichiometry="1"
constant="true"/>
                  <speciesReference species="M_nad_c" stoichiometry="3"
constant="true"/>
            </listOfProducts>
            <fbc:geneProductAssociation>
                  <fbc:and>
                        <fbc:geneProductRef fbc:geneProduct="SAUSA300_2346"/>
                        <fbc:geneProductRef fbc:geneProduct="SAUSA300_2345"/>
                  </fbc:and>
            </fbc:geneProductAssociation>
      </reaction>

      <reaction metaid="R_NTRIR2y" id="R_NTRIR2y" name="nitrite Reductase
(NADPH)" reversible="false" fast="false" fbc:lowerFluxBound="irr_lb"
fbc:upperFluxBound="irr_ub">
            <listOfReactants>
                  <speciesReference species="M_h_c" stoichiometry="5"
constant="true"/>
                  <speciesReference species="M_nadph_c" stoichiometry="3"
constant="true"/>
                  <speciesReference species="M_no2_c" stoichiometry="1"
constant="true"/>
            </listOfReactants>
            <listOfProducts>
                  <speciesReference species="M_h2o_c" stoichiometry="2"
constant="true"/>
                  <speciesReference species="M_nh4_c" stoichiometry="1"
constant="true"/>
                  <speciesReference species="M_nadp_c" stoichiometry="3"
constant="true"/>
            </listOfProducts>
```

```
            <fbc:geneProductAssociation>
                  <fbc:or>
                        <fbc:geneProductRef fbc:geneProduct="SAUSA300_2346"/>
                        <fbc:geneProductRef fbc:geneProduct="SAUSA300_2345"/>
                  </fbc:or>
            </fbc:geneProductAssociation>
      </reaction>

      <reaction metaid="R_NTRIRx" id="R_NTRIRx" name="nitrite reductase
(NADH)" reversible="false" fast="false" fbc:lowerFluxBound="irr_lb"
fbc:upperFluxBound="irr_ub">
            <listOfReactants>
                  <speciesReference species="M_h_c" stoichiometry="4"
constant="true"/>
                  <speciesReference species="M_nadh_c" stoichiometry="3"
constant="true"/>
                  <speciesReference species="M_no2_c" stoichiometry="1"
constant="true"/>
            </listOfReactants>
            <listOfProducts>
                  <speciesReference species="M_h2o_c" stoichiometry="1"
constant="true"/>
                  <speciesReference species="M_nad_c" stoichiometry="3"
constant="true"/>
                  <speciesReference species="M_nh4oh_c" stoichiometry="1"
constant="true"/>
            </listOfProducts>
            <fbc:geneProductAssociation>
                  <fbc:and>
                        <fbc:geneProductRef fbc:geneProduct="SAUSA300_2346"/>
                        <fbc:geneProductRef fbc:geneProduct="SAUSA300_2345"/>
                  </fbc:and>
            </fbc:geneProductAssociation>
      </reaction>

      <reaction metaid="R_NTRIRy" id="R_NTRIRy" name="nitrite reductase
(NADPH)" reversible="false" fast="false" fbc:lowerFluxBound="irr_lb"
fbc:upperFluxBound="irr_ub">
            <listOfReactants>
                  <speciesReference species="M_h_c" stoichiometry="4"
constant="true"/>
                  <speciesReference species="M_nadph_c" stoichiometry="3"
constant="true"/>
                  <speciesReference species="M_no2_c" stoichiometry="1"
constant="true"/>
            </listOfReactants>
            <listOfProducts>
                  <speciesReference species="M_h2o_c" stoichiometry="1"
constant="true"/>
                  <speciesReference species="M_nadp_c" stoichiometry="3"
constant="true"/>
                  <speciesReference species="M_nh4oh_c" stoichiometry="1"
constant="true"/>
            </listOfProducts>
            <fbc:geneProductAssociation>
                  <fbc:and>
                        <fbc:geneProductRef fbc:geneProduct="SAUSA300_2346"/>
                        <fbc:geneProductRef fbc:geneProduct="SAUSA300_2345"/>
```

```
                </fbc:and>
            </fbc:geneProductAssociation>
        </reaction>

        <reaction metaid="R_O2t5i" id="R_O2t5i" name="O2 transport via
diffusion c0" reversible="true" fast="false" fbc:lowerFluxBound="rev_lb"
fbc:upperFluxBound="rev_ub">
            <listOfReactants>
                <speciesReference species="M_o2_e" stoichiometry="1"
constant="true"/>
            </listOfReactants>
            <listOfProducts>
                <speciesReference species="M_o2_c" stoichiometry="1"
constant="true"/>
            </listOfProducts>
        </reaction>

        <reaction metaid="R_OBTFL" id="R_OBTFL" name="2-Oxobutanoate formate
lyase" reversible="true" fast="false" fbc:lowerFluxBound="rev_lb"
fbc:upperFluxBound="rev_ub">
            <listOfReactants>
                <speciesReference species="M_2obut_c" stoichiometry="1"
constant="true"/>
                <speciesReference species="M_coa_c" stoichiometry="1"
constant="true"/>
            </listOfReactants>
            <listOfProducts>
                <speciesReference species="M_ppcoa_c" stoichiometry="1"
constant="true"/>
                <speciesReference species="M_for_c" stoichiometry="1"
constant="true"/>
            </listOfProducts>
            <fbc:geneProductAssociation>
                <fbc:geneProductRef fbc:geneProduct="SAUSA300_0220"/>
            </fbc:geneProductAssociation>
        </reaction>

        <reaction metaid="R_OCBT" id="R_OCBT" name="ornithine
carbamoyltransferase" reversible="true" fast="false"
fbc:lowerFluxBound="rev_lb" fbc:upperFluxBound="rev_ub">
            <listOfReactants>
                <speciesReference species="M_cbp_c" stoichiometry="1"
constant="true"/>
                <speciesReference species="M_orn_c" stoichiometry="1"
constant="true"/>
            </listOfReactants>
            <listOfProducts>
                <speciesReference species="M_h_c" stoichiometry="2"
constant="true"/>
                <speciesReference species="M_pi_c" stoichiometry="1"
constant="true"/>
                <speciesReference species="M_citr__L_c" stoichiometry="1"
constant="true"/>
            </listOfProducts>
            <fbc:geneProductAssociation>
                <fbc:or>
                    <fbc:geneProductRef fbc:geneProduct="SAUSA300_0062"/>
                    <fbc:geneProductRef fbc:geneProduct="SAUSA300_2569"/>
```

```
                        <fbc:geneProductRef fbc:geneProduct="SAUSA300_1062"/>
                  </fbc:or>
            </fbc:geneProductAssociation>
      </reaction>

      <reaction metaid="R_OCTD" id="R_OCTD" name="D-octopine dehydrogenase"
reversible="false" fast="false" fbc:lowerFluxBound="irr_lb"
fbc:upperFluxBound="irr_ub">
            <listOfReactants>
                  <speciesReference species="M_h2o_c" stoichiometry="1"
constant="true"/>
                  <speciesReference species="M_nadp_c" stoichiometry="1"
constant="true"/>
                  <speciesReference species="M_octp_c" stoichiometry="1"
constant="true"/>
            </listOfReactants>
            <listOfProducts>
                  <speciesReference species="M_h_c" stoichiometry="1"
constant="true"/>
                  <speciesReference species="M_nadph_c" stoichiometry="1"
constant="true"/>
                  <speciesReference species="M_arg__L_c" stoichiometry="1"
constant="true"/>
                  <speciesReference species="M_pyr_c" stoichiometry="1"
constant="true"/>
            </listOfProducts>
            <fbc:geneProductAssociation>
                  <fbc:geneProductRef fbc:geneProduct="SAUSA300_2251"/>
            </fbc:geneProductAssociation>
      </reaction>

      <reaction metaid="R_OIVD1r" id="R_OIVD1r" name="2-oxoisovalerate
dehydrogenase (acylating; 4-methyl-2-oxopentaoate)" reversible="false"
fast="false" fbc:lowerFluxBound="irr_lb" fbc:upperFluxBound="irr_ub">
            <listOfReactants>
                  <speciesReference species="M_nad_c" stoichiometry="1"
constant="true"/>
                  <speciesReference species="M_4mop_c" stoichiometry="1"
constant="true"/>
                  <speciesReference species="M_coa_c" stoichiometry="1"
constant="true"/>
            </listOfReactants>
            <listOfProducts>
                  <speciesReference species="M_co2_c" stoichiometry="1"
constant="true"/>
                  <speciesReference species="M_nadh_c" stoichiometry="1"
constant="true"/>
                  <speciesReference species="M_ivcoa_c" stoichiometry="1"
constant="true"/>
            </listOfProducts>
            <fbc:geneProductAssociation>
                  <fbc:and>
                        <fbc:geneProductRef fbc:geneProduct="SAUSA300_1466"/>
                        <fbc:geneProductRef fbc:geneProduct="SAUSA300_1464"/>
                        <fbc:geneProductRef fbc:geneProduct="SAUSA300_1465"/>
                  </fbc:and>
            </fbc:geneProductAssociation>
      </reaction>
```

```
      <reaction metaid="R_OIVD2" id="R_OIVD2" name="2-oxoisovalerate
dehydrogenase (acylating; 3-methyl-2-oxobutanoate)" reversible="false"
fast="false" fbc:lowerFluxBound="irr_lb" fbc:upperFluxBound="irr_ub">
            <listOfReactants>
                  <speciesReference species="M_nad_c" stoichiometry="1"
constant="true"/>
                  <speciesReference species="M_3mob_c" stoichiometry="1"
constant="true"/>
                  <speciesReference species="M_coa_c" stoichiometry="1"
constant="true"/>
            </listOfReactants>
            <listOfProducts>
                  <speciesReference species="M_co2_c" stoichiometry="1"
constant="true"/>
                  <speciesReference species="M_nadh_c" stoichiometry="1"
constant="true"/>
                  <speciesReference species="M_ibcoa_c" stoichiometry="1"
constant="true"/>
            </listOfProducts>
            <fbc:geneProductAssociation>
                  <fbc:and>
                        <fbc:geneProductRef fbc:geneProduct="SAUSA300_1466"/>
                        <fbc:geneProductRef fbc:geneProduct="SAUSA300_1464"/>
                        <fbc:geneProductRef fbc:geneProduct="SAUSA300_1465"/>
                  </fbc:and>
            </fbc:geneProductAssociation>
      </reaction>

      <reaction metaid="R_OIVD3" id="R_OIVD3" name="2-oxoisovalerate
dehydrogenase (acylating; 3-methyl-2-oxopentanoate)" reversible="false"
fast="false" fbc:lowerFluxBound="irr_lb" fbc:upperFluxBound="irr_ub">
            <listOfReactants>
                  <speciesReference species="M_nad_c" stoichiometry="1"
constant="true"/>
                  <speciesReference species="M_coa_c" stoichiometry="1"
constant="true"/>
                  <speciesReference species="M_3mop_c" stoichiometry="1"
constant="true"/>
            </listOfReactants>
            <listOfProducts>
                  <speciesReference species="M_co2_c" stoichiometry="1"
constant="true"/>
                  <speciesReference species="M_nadh_c" stoichiometry="1"
constant="true"/>
                  <speciesReference species="M_2mbcoa_c" stoichiometry="1"
constant="true"/>
            </listOfProducts>
            <fbc:geneProductAssociation>
                  <fbc:and>
                        <fbc:geneProductRef fbc:geneProduct="SAUSA300_1466"/>
                        <fbc:geneProductRef fbc:geneProduct="SAUSA300_1464"/>
                        <fbc:geneProductRef fbc:geneProduct="SAUSA300_1465"/>
                  </fbc:and>
            </fbc:geneProductAssociation>
      </reaction>
```

```
      <reaction metaid="R_OMPDC" id="R_OMPDC" name="orotidine-5'-phosphate
decarboxylase" reversible="false" fast="false" fbc:lowerFluxBound="irr_lb"
fbc:upperFluxBound="irr_ub">
            <listOfReactants>
                  <speciesReference species="M_h_c" stoichiometry="1"
constant="true"/>
                  <speciesReference species="M_orot5p_c" stoichiometry="1"
constant="true"/>
            </listOfReactants>
            <listOfProducts>
                  <speciesReference species="M_co2_c" stoichiometry="1"
constant="true"/>
                  <speciesReference species="M_ump_c" stoichiometry="1"
constant="true"/>
            </listOfProducts>
            <fbc:geneProductAssociation>
                  <fbc:geneProductRef fbc:geneProduct="SAUSA300_1097"/>
            </fbc:geneProductAssociation>
      </reaction>

      <reaction metaid="R_OPHHXy" id="R_OPHHXy" name="2-Octaprenylphenol
hydroxylase (NADPH)" reversible="false" fast="false"
fbc:lowerFluxBound="irr_lb" fbc:upperFluxBound="irr_ub">
            <listOfReactants>
                  <speciesReference species="M_h_c" stoichiometry="1"
constant="true"/>
                  <speciesReference species="M_nadph_c" stoichiometry="1"
constant="true"/>
                  <speciesReference species="M_o2_c" stoichiometry="1"
constant="true"/>
                  <speciesReference species="M_2oph_c" stoichiometry="1"
constant="true"/>
            </listOfReactants>
            <listOfProducts>
                  <speciesReference species="M_h2o_c" stoichiometry="1"
constant="true"/>
                  <speciesReference species="M_nadp_c" stoichiometry="1"
constant="true"/>
                  <speciesReference species="M_2ohph_c" stoichiometry="1"
constant="true"/>
            </listOfProducts>
            <fbc:geneProductAssociation>
                  <fbc:or>
                        <fbc:geneProductRef fbc:geneProduct="SAUSA300_2429"/>
                        <fbc:geneProductRef fbc:geneProduct="SAUSA300_1741"/>
                        <fbc:geneProductRef fbc:geneProduct="ubiI"/>
                  </fbc:or>
            </fbc:geneProductAssociation>
      </reaction>

      <reaction metaid="R_ORNCD" id="R_ORNCD" name="ornithine cyclodeaminase"
reversible="false" fast="false" fbc:lowerFluxBound="irr_lb"
fbc:upperFluxBound="irr_ub">
            <listOfReactants>
                  <speciesReference species="M_orn_c" stoichiometry="1"
constant="true"/>
            </listOfReactants>
            <listOfProducts>
```

```
                  <speciesReference species="M_h_c" stoichiometry="1"
constant="true"/>
                  <speciesReference species="M_nh4_c" stoichiometry="1"
constant="true"/>
                  <speciesReference species="M_pro__L_c" stoichiometry="1"
constant="true"/>
            &lt;/listOfProducts&gt;
            &lt;fbc:geneProductAssociation&gt;
                  &lt;fbc:geneProductRef fbc:geneProduct="SAUSA300_0119"/&gt;
            &lt;/fbc:geneProductAssociation&gt;
      &lt;/reaction&gt;

      <reaction metaid="R_ORNDC" id="R_ORNDC" name="L-Ornithine
carboxy__Lyase c0" reversible="false" fast="false"
fbc:lowerFluxBound="irr_lb" fbc:upperFluxBound="irr_ub">
            &lt;listOfReactants&gt;
                  <speciesReference species="M_h_c" stoichiometry="1"
constant="true"/>
                  <speciesReference species="M_orn_c" stoichiometry="1"
constant="true"/>
            &lt;/listOfReactants&gt;
            &lt;listOfProducts&gt;
                  <speciesReference species="M_co2_c" stoichiometry="1"
constant="true"/>
                  <speciesReference species="M_ptrc_c" stoichiometry="1"
constant="true"/>
            &lt;/listOfProducts&gt;
            &lt;fbc:geneProductAssociation&gt;
                  &lt;fbc:geneProductRef fbc:geneProduct="speC"/&gt;
            &lt;/fbc:geneProductAssociation&gt;
      &lt;/reaction&gt;

      <reaction metaid="R_ORNTAC" id="R_ORNTAC" name="ornithine
transacetylase" reversible="false" fast="false" fbc:lowerFluxBound="irr_lb"
fbc:upperFluxBound="irr_ub">
            &lt;listOfReactants&gt;
                  <speciesReference species="M_glu__L_c" stoichiometry="1"
constant="true"/>
                  <speciesReference species="M_acorn_c" stoichiometry="1"
constant="true"/>
            &lt;/listOfReactants&gt;
            &lt;listOfProducts&gt;
                  <speciesReference species="M_orn_c" stoichiometry="1"
constant="true"/>
                  <speciesReference species="M_acglu_c" stoichiometry="1"
constant="true"/>
            &lt;/listOfProducts&gt;
            &lt;fbc:geneProductAssociation&gt;
                  &lt;fbc:geneProductRef fbc:geneProduct="SAUSA300_0185"/&gt;
            &lt;/fbc:geneProductAssociation&gt;
      &lt;/reaction&gt;

      <reaction metaid="R_ORNTAr" id="R_ORNTAr" name="ornithine transaminase
reversible" reversible="true" fast="false" fbc:lowerFluxBound="rev_lb"
fbc:upperFluxBound="rev_ub">
            &lt;listOfReactants&gt;
                  <speciesReference species="M_akg_c" stoichiometry="1"
constant="true"/>
```

```
                  <speciesReference species="M_orn_c" stoichiometry="1"
constant="true"/>
            </listOfReactants>
            <listOfProducts>
                  <speciesReference species="M_glu__L_c" stoichiometry="1"
constant="true"/>
                  <speciesReference species="M_glu5sa_c" stoichiometry="1"
constant="true"/>
            </listOfProducts>
            <fbc:geneProductAssociation>
                  <fbc:geneProductRef fbc:geneProduct="SAUSA300_0860"/>
            </fbc:geneProductAssociation>
      </reaction>

      <reaction metaid="R_ORPT" id="R_ORPT" name="orotate
phosphoribosyltransferase" reversible="true" fast="false"
fbc:lowerFluxBound="rev_lb" fbc:upperFluxBound="rev_ub">
            <listOfReactants>
                  <speciesReference species="M_prpp_c" stoichiometry="1"
constant="true"/>
                  <speciesReference species="M_orot_c" stoichiometry="1"
constant="true"/>
            </listOfReactants>
            <listOfProducts>
                  <speciesReference species="M_ppi_c" stoichiometry="1"
constant="true"/>
                  <speciesReference species="M_orot5p_c" stoichiometry="1"
constant="true"/>
            </listOfProducts>
            <fbc:geneProductAssociation>
                  <fbc:geneProductRef fbc:geneProduct="SAUSA300_1098"/>
            </fbc:geneProductAssociation>
      </reaction>

      <reaction metaid="R_OSUCCL" id="R_OSUCCL" name="Oxalosuccinate
carboxy__Lyase (2-oxoglutarate-forming)" reversible="false" fast="false"
fbc:lowerFluxBound="irr_lb" fbc:upperFluxBound="irr_ub">
            <listOfReactants>
                  <speciesReference species="M_h_c" stoichiometry="1"
constant="true"/>
                  <speciesReference species="M_osuc_c" stoichiometry="1"
constant="true"/>
            </listOfReactants>
            <listOfProducts>
                  <speciesReference species="M_akg_c" stoichiometry="1"
constant="true"/>
                  <speciesReference species="M_co2_c" stoichiometry="1"
constant="true"/>
            </listOfProducts>
            <fbc:geneProductAssociation>
                  <fbc:geneProductRef fbc:geneProduct="SAUSA300_1640"/>
            </fbc:geneProductAssociation>
      </reaction>

      <reaction metaid="R_P5CD" id="R_P5CD" name="1-pyrroline-5-carboxylate
dehydrogenase" reversible="false" fast="false" fbc:lowerFluxBound="irr_lb"
fbc:upperFluxBound="irr_ub">
            <listOfReactants>
```

```
                    <speciesReference species="M_h2o_c" stoichiometry="2"
constant="true"/>
                    <speciesReference species="M_1pyr5c_c" stoichiometry="1"
constant="true"/>
                    <speciesReference species="M_nad_c" stoichiometry="1"
constant="true"/>
              </listOfReactants>
              <listOfProducts>
                    <speciesReference species="M_h_c" stoichiometry="1"
constant="true"/>
                    <speciesReference species="M_glu__L_c" stoichiometry="1"
constant="true"/>
                    <speciesReference species="M_nadh_c" stoichiometry="1"
constant="true"/>
              </listOfProducts>
              <fbc:geneProductAssociation>
                    <fbc:geneProductRef fbc:geneProduct="SAUSA300_2491"/>
              </fbc:geneProductAssociation>
        </reaction>

        <reaction metaid="R_P5CR" id="R_P5CR" name="pyrroline-5-carboxylate
reductase" reversible="false" fast="false" fbc:lowerFluxBound="irr_lb"
fbc:upperFluxBound="irr_ub">
              <listOfReactants>
                    <speciesReference species="M_nadp_c" stoichiometry="1"
constant="true"/>
                    <speciesReference species="M_pro__L_c" stoichiometry="1"
constant="true"/>
              </listOfReactants>
              <listOfProducts>
                    <speciesReference species="M_h_c" stoichiometry="1"
constant="true"/>
                    <speciesReference species="M_nadph_c" stoichiometry="1"
constant="true"/>
                    <speciesReference species="M_1pyr5c_c" stoichiometry="1"
constant="true"/>
              </listOfProducts>
              <fbc:geneProductAssociation>
                    <fbc:geneProductRef fbc:geneProduct="SAUSA300_1452"/>
              </fbc:geneProductAssociation>
        </reaction>

        <reaction metaid="R_PALASA_SA2" id="R_PALASA_SA2"
name="Phosphatidylalanine Synthase (SA) 2" reversible="true" fast="false"
fbc:lowerFluxBound="rev_lb" fbc:upperFluxBound="rev_ub">
              <listOfReactants>
                    <speciesReference species="M_ala__L_c" stoichiometry="1"
constant="true"/>
                    <speciesReference species="M_cdpdag_SA_c"
stoichiometry="0.02" constant="true"/>
              </listOfReactants>
              <listOfProducts>
                    <speciesReference species="M_h_c" stoichiometry="1"
constant="true"/>
                    <speciesReference species="M_cmp_c" stoichiometry="1"
constant="true"/>
                    <speciesReference species="M_pala_SA2_c"
stoichiometry="0.02" constant="true"/>
```

```
            </listOfProducts>
      </reaction>

      <reaction metaid="R_PALGLCT" id="R_PALGLCT" name="palmitoyl-UDP-
glucosyltransferase diglucosyl " reversible="true" fast="false"
fbc:lowerFluxBound="rev_lb" fbc:upperFluxBound="rev_ub">
            <listOfReactants>
                  <speciesReference species="M_udpg_c" stoichiometry="1"
constant="true"/>
                  <speciesReference species="M_mg12dpgly_c" stoichiometry="1"
constant="true"/>
            </listOfReactants>
            <listOfProducts>
                  <speciesReference species="M_udp_c" stoichiometry="1"
constant="true"/>
                  <speciesReference species="M_dglu12d_c" stoichiometry="1"
constant="true"/>
            </listOfProducts>
            <fbc:geneProductAssociation>
                  <fbc:geneProductRef fbc:geneProduct="SAUSA300_0918"/>
            </fbc:geneProductAssociation>
      </reaction>

      <reaction metaid="R_PALMGLTT" id="R_PALMGLTT" name="palmitoyl-UDP-
glucosyltransferase monoglucosyl " reversible="true" fast="false"
fbc:lowerFluxBound="rev_lb" fbc:upperFluxBound="rev_ub">
            <listOfReactants>
                  <speciesReference species="M_udpg_c" stoichiometry="1"
constant="true"/>
                  <speciesReference species="M_12dgr160_c" stoichiometry="1"
constant="true"/>
            </listOfReactants>
            <listOfProducts>
                  <speciesReference species="M_udp_c" stoichiometry="1"
constant="true"/>
                  <speciesReference species="M_mg12dpgly_c" stoichiometry="1"
constant="true"/>
            </listOfProducts>
            <fbc:geneProductAssociation>
                  <fbc:geneProductRef fbc:geneProduct="SAUSA300_0918"/>
            </fbc:geneProductAssociation>
      </reaction>

      <reaction metaid="R_PALMLNS" id="R_PALMLNS"
name="palmitoyl__Lipoteichoic acid synthesis n=24  linked  N-
acetylglucosamine substituted " reversible="true" fast="false"
fbc:lowerFluxBound="rev_lb" fbc:upperFluxBound="rev_ub">
            <listOfReactants>
                  <speciesReference species="M_uacgam_c" stoichiometry="24"
constant="true"/>
                  <speciesReference species="M_palm24u_c" stoichiometry="1"
constant="true"/>
            </listOfReactants>
            <listOfProducts>
                  <speciesReference species="M_udp_c" stoichiometry="24"
constant="true"/>
                  <speciesReference species="M_palm24nacs_c"
stoichiometry="1" constant="true"/>
```

```
            </listOfProducts>
            <fbc:geneProductAssociation>
                  <fbc:geneProductRef fbc:geneProduct="SAUSA300_0731"/>
            </fbc:geneProductAssociation>
      </reaction>

      <reaction metaid="R_PANTS" id="R_PANTS" name="pantothenate synthase"
reversible="false" fast="false" fbc:lowerFluxBound="irr_lb"
fbc:upperFluxBound="irr_ub">
            <listOfReactants>
                  <speciesReference species="M_atp_c" stoichiometry="1"
constant="true"/>
                  <speciesReference species="M_pant__R_c" stoichiometry="1"
constant="true"/>
                  <speciesReference species="M_ala__B_c" stoichiometry="1"
constant="true"/>
            </listOfReactants>
            <listOfProducts>
                  <speciesReference species="M_ppi_c" stoichiometry="1"
constant="true"/>
                  <speciesReference species="M_amp_c" stoichiometry="1"
constant="true"/>
                  <speciesReference species="M_pnto__R_c" stoichiometry="1"
constant="true"/>
            </listOfProducts>
            <fbc:geneProductAssociation>
                  <fbc:geneProductRef fbc:geneProduct="SAUSA300_2533"/>
            </fbc:geneProductAssociation>
      </reaction>

      <reaction metaid="R_PAPA_SA" id="R_PAPA_SA" name="Phosphatidate
phosphatase" reversible="false" fast="false" fbc:lowerFluxBound="irr_lb"
fbc:upperFluxBound="irr_ub">
            <listOfReactants>
                  <speciesReference species="M_h2o_c" stoichiometry="1"
constant="true"/>
                  <speciesReference species="M_pa_SA_c" stoichiometry="0.02"
constant="true"/>
            </listOfReactants>
            <listOfProducts>
                  <speciesReference species="M_pi_c" stoichiometry="1"
constant="true"/>
                  <speciesReference species="M_12dgr_SA_c"
stoichiometry="0.02" constant="true"/>
            </listOfProducts>
      </reaction>

      <reaction metaid="R_PAPA140" id="R_PAPA140" name="Phosphatidate
phosphatase (n-C14:0)" reversible="false" fast="false"
fbc:lowerFluxBound="irr_lb" fbc:upperFluxBound="irr_ub">
            <listOfReactants>
                  <speciesReference species="M_h2o_c" stoichiometry="1"
constant="true"/>
                  <speciesReference species="M_pa140_c" stoichiometry="1"
constant="true"/>
            </listOfReactants>
            <listOfProducts>
```

```
                  <speciesReference species="M_h_c" stoichiometry="1"
constant="true"/>
                  <speciesReference species="M_pi_c" stoichiometry="1"
constant="true"/>
                  <speciesReference species="M_12dgr140_c" stoichiometry="1"
constant="true"/>
            </listOfProducts>
            <fbc:geneProductAssociation>
                  <fbc:geneProductRef fbc:geneProduct="SAUSA300_1529"/>
            </fbc:geneProductAssociation>
      </reaction>

      <reaction metaid="R_PAPA160" id="R_PAPA160" name="Phosphatidate
phosphatase (n-C16:0)" reversible="false" fast="false"
fbc:lowerFluxBound="irr_lb" fbc:upperFluxBound="irr_ub">
            <listOfReactants>
                  <speciesReference species="M_h2o_c" stoichiometry="1"
constant="true"/>
                  <speciesReference species="M_pa160_c" stoichiometry="1"
constant="true"/>
            </listOfReactants>
            <listOfProducts>
                  <speciesReference species="M_h_c" stoichiometry="1"
constant="true"/>
                  <speciesReference species="M_pi_c" stoichiometry="1"
constant="true"/>
                  <speciesReference species="M_12dgr160_c" stoichiometry="1"
constant="true"/>
            </listOfProducts>
            <fbc:geneProductAssociation>
                  <fbc:geneProductRef fbc:geneProduct="SAUSA300_1529"/>
            </fbc:geneProductAssociation>
      </reaction>

      <reaction metaid="R_PAPA180pp" id="R_PAPA180pp" name="Phosphatidate
phosphatase (periplasmic, n-C18:0)" reversible="false" fast="false"
fbc:lowerFluxBound="irr_lb" fbc:upperFluxBound="irr_ub">
            <listOfReactants>
                  <speciesReference species="M_h2o_c" stoichiometry="1"
constant="true"/>
                  <speciesReference species="M_pa180_c" stoichiometry="1"
constant="true"/>
            </listOfReactants>
            <listOfProducts>
                  <speciesReference species="M_h_c" stoichiometry="1"
constant="true"/>
                  <speciesReference species="M_pi_c" stoichiometry="1"
constant="true"/>
                  <speciesReference species="M_12dgr180_c" stoichiometry="1"
constant="true"/>
            </listOfProducts>
            <fbc:geneProductAssociation>
                  <fbc:geneProductRef fbc:geneProduct="SAUSA300_1529"/>
            </fbc:geneProductAssociation>
      </reaction>
```

```
      <reaction metaid="R_PAPPT2" id="R_PAPPT2" name="phospho-N-
acetylmuramoyl-pentapeptide-transferase (gamma-glutamate)" reversible="true"
fast="false" fbc:lowerFluxBound="rev_lb" fbc:upperFluxBound="rev_ub">
            <listOfReactants>
                  <speciesReference species="M_udcpp_c" stoichiometry="1"
constant="true"/>
                  <speciesReference species="M_uGgla_c" stoichiometry="1"
constant="true"/>
            </listOfReactants>
            <listOfProducts>
                  <speciesReference species="M_ump_c" stoichiometry="1"
constant="true"/>
                  <speciesReference species="M_uaGgla_c" stoichiometry="1"
constant="true"/>
            </listOfProducts>
            <fbc:geneProductAssociation>
                  <fbc:geneProductRef fbc:geneProduct="SAUSA300_1076"/>
            </fbc:geneProductAssociation>
      </reaction>

      <reaction metaid="R_PAPPT3" id="R_PAPPT3" name="phospho-N-
acetylmuramoyl-pentapeptide-transferase (meso-2,6__Diaminopimelate)"
reversible="false" fast="false" fbc:lowerFluxBound="irr_lb"
fbc:upperFluxBound="irr_ub">
            <listOfReactants>
                  <speciesReference species="M_udcpp_c" stoichiometry="1"
constant="true"/>
                  <speciesReference species="M_ugmda_c" stoichiometry="1"
constant="true"/>
            </listOfReactants>
            <listOfProducts>
                  <speciesReference species="M_uagmda_c" stoichiometry="1"
constant="true"/>
                  <speciesReference species="M_ump_c" stoichiometry="1"
constant="true"/>
            </listOfProducts>
            <fbc:geneProductAssociation>
                  <fbc:geneProductRef fbc:geneProduct="SAUSA300_1076"/>
            </fbc:geneProductAssociation>
      </reaction>

      <reaction metaid="R_PAPSR" id="R_PAPSR" name="phosphoadenylyl-sulfate
reductase (thioredoxin)" reversible="false" fast="false"
fbc:lowerFluxBound="irr_lb" fbc:upperFluxBound="irr_ub">
            <listOfReactants>
                  <speciesReference species="M_trdrd_c" stoichiometry="1"
constant="true"/>
                  <speciesReference species="M_paps_c" stoichiometry="1"
constant="true"/>
            </listOfReactants>
            <listOfProducts>
                  <speciesReference species="M_h_c" stoichiometry="2"
constant="true"/>
                  <speciesReference species="M_so3_c" stoichiometry="1"
constant="true"/>
                  <speciesReference species="M_trdox_c" stoichiometry="1"
constant="true"/>
```

```
                        <speciesReference species="M_pap_c" stoichiometry="1"
constant="true"/>
                </listOfProducts>
        </reaction>

        <reaction metaid="R_PASYN_SA" id="R_PASYN_SA" name="Phosphatidic acid
synthase (Saureus)" reversible="false" fast="false"
fbc:lowerFluxBound="irr_lb" fbc:upperFluxBound="irr_ub">
                <listOfReactants>
                        <speciesReference species="M_glyc3p_c" stoichiometry="1"
constant="true"/>
                        <speciesReference species="M_hdca_c" stoichiometry="0.024"
constant="true"/>
                        <speciesReference species="M_fa4_c" stoichiometry="0.792"
constant="true"/>
                        <speciesReference species="M_ocdca_c" stoichiometry="0.116"
constant="true"/>
                        <speciesReference species="M_ttdca_c" stoichiometry="0.004"
constant="true"/>
                        <speciesReference species="M_fa12_c" stoichiometry="0.35"
constant="true"/>
                        <speciesReference species="M_fa3_c" stoichiometry="0.169"
constant="true"/>
                        <speciesReference species="M_fa6_c" stoichiometry="0.033"
constant="true"/>
                        <speciesReference species="M_fa11_c" stoichiometry="0.15"
constant="true"/>
                        <speciesReference species="M_fa1_c" stoichiometry="0.013"
constant="true"/>
                        <speciesReference species="M_fa19a_c" stoichiometry="0.186"
constant="true"/>
                        <speciesReference species="M_fa20n_c" stoichiometry="0.063"
constant="true"/>
                </listOfReactants>
                <listOfProducts>
                        <speciesReference species="M_pa_SA_c" stoichiometry="0.02"
constant="true"/>
                </listOfProducts>
                <fbc:geneProductAssociation>
                        <fbc:geneProductRef fbc:geneProduct="SAUSA300_0099"/>
                </fbc:geneProductAssociation>
        </reaction>

        <reaction metaid="R_PC" id="R_PC" name="pyruvate carboxylase"
reversible="false" fast="false" fbc:lowerFluxBound="irr_lb"
fbc:upperFluxBound="irr_ub">
                <listOfReactants>
                        <speciesReference species="M_atp_c" stoichiometry="1"
constant="true"/>
                        <speciesReference species="M_pyr_c" stoichiometry="1"
constant="true"/>
                        <speciesReference species="M_hco3_c" stoichiometry="1"
constant="true"/>
                </listOfReactants>
                <listOfProducts>
                        <speciesReference species="M_h_c" stoichiometry="1"
constant="true"/>
```

```
                  <speciesReference species="M_pi_c" stoichiometry="1"
constant="true"/>
                  <speciesReference species="M_adp_c" stoichiometry="1"
constant="true"/>
                  <speciesReference species="M_oaa_c" stoichiometry="1"
constant="true"/>
            &lt;/listOfProducts&gt;
            &lt;fbc:geneProductAssociation&gt;
                  &lt;fbc:geneProductRef fbc:geneProduct="SAUSA300_1014"/&gt;
            &lt;/fbc:geneProductAssociation&gt;
      &lt;/reaction&gt;

      <reaction metaid="R_PDH" id="R_PDH" name="pyruvate dehydrogenase"
reversible="false" fast="false" fbc:lowerFluxBound="irr_lb"
fbc:upperFluxBound="irr_ub">
            &lt;listOfReactants&gt;
                  <speciesReference species="M_nad_c" stoichiometry="1"
constant="true"/>
                  <speciesReference species="M_pyr_c" stoichiometry="1"
constant="true"/>
                  <speciesReference species="M_coa_c" stoichiometry="1"
constant="true"/>
            &lt;/listOfReactants&gt;
            &lt;listOfProducts&gt;
                  <speciesReference species="M_co2_c" stoichiometry="1"
constant="true"/>
                  <speciesReference species="M_nadh_c" stoichiometry="1"
constant="true"/>
                  <speciesReference species="M_accoa_c" stoichiometry="1"
constant="true"/>
            &lt;/listOfProducts&gt;
            &lt;fbc:geneProductAssociation&gt;
                  &lt;fbc:and&gt;
                        &lt;fbc:geneProductRef fbc:geneProduct="SAUSA300_0993"/&gt;
                        &lt;fbc:geneProductRef fbc:geneProduct="SAUSA300_0994"/&gt;
                        &lt;fbc:geneProductRef fbc:geneProduct="SAUSA300_0996"/&gt;
                        &lt;fbc:geneProductRef fbc:geneProduct="SAUSA300_0995"/&gt;
                  &lt;/fbc:and&gt;
            &lt;/fbc:geneProductAssociation&gt;
      &lt;/reaction&gt;

      <reaction metaid="R_PDHbr" id="R_PDHbr" name="pyruvate dehydrogenase
(dihydrolipoamide) reversible" reversible="true" fast="false"
fbc:lowerFluxBound="rev_lb" fbc:upperFluxBound="rev_ub">
            &lt;listOfReactants&gt;
                  <speciesReference species="M_accoa_c" stoichiometry="1"
constant="true"/>
                  <speciesReference species="M_dhlam_c" stoichiometry="1"
constant="true"/>
            &lt;/listOfReactants&gt;
            &lt;listOfProducts&gt;
                  <speciesReference species="M_coa_c" stoichiometry="1"
constant="true"/>
                  <speciesReference species="M_adhlam_c" stoichiometry="1"
constant="true"/>
            &lt;/listOfProducts&gt;
            &lt;fbc:geneProductAssociation&gt;
                  &lt;fbc:geneProductRef fbc:geneProduct="SAUSA300_0995"/&gt;
```

```
            </fbc:geneProductAssociation>
      </reaction>

      <reaction metaid="R_PDHcr" id="R_PDHcr" name="Pyruvate dehydrogenase
(dihydrolipoamide dehydrogenase) reversible" reversible="false" fast="false"
fbc:lowerFluxBound="irr_lb" fbc:upperFluxBound="irr_ub">
            <listOfReactants>
                  <speciesReference species="M_nad_c" stoichiometry="1"
constant="true"/>
                  <speciesReference species="M_dhlam_c" stoichiometry="1"
constant="true"/>
            </listOfReactants>
            <listOfProducts>
                  <speciesReference species="M_h_c" stoichiometry="1"
constant="true"/>
                  <speciesReference species="M_nadh_c" stoichiometry="1"
constant="true"/>
                  <speciesReference species="M_lpam_c" stoichiometry="1"
constant="true"/>
            </listOfProducts>
            <fbc:geneProductAssociation>
                  <fbc:or>
                        <fbc:geneProductRef fbc:geneProduct="SAUSA300_1467"/>
                        <fbc:geneProductRef fbc:geneProduct="SAUSA300_0576"/>
                        <fbc:geneProductRef fbc:geneProduct="SAUSA300_0996"/>
                  </fbc:or>
            </fbc:geneProductAssociation>
      </reaction>

      <reaction metaid="R_PEPC" id="R_PEPC" name="Phosphoenolpyruvate
carboxylase" reversible="true" fast="false" fbc:lowerFluxBound="rev_lb"
fbc:upperFluxBound="rev_ub">
            <listOfReactants>
                  <speciesReference species="M_pep_c" stoichiometry="1"
constant="true"/>
                  <speciesReference species="M_hco3_c" stoichiometry="1"
constant="true"/>
            </listOfReactants>
            <listOfProducts>
                  <speciesReference species="M_pi_c" stoichiometry="1"
constant="true"/>
                  <speciesReference species="M_oaa_c" stoichiometry="1"
constant="true"/>
            </listOfProducts>
            <fbc:geneProductAssociation>
                  <fbc:geneProductRef fbc:geneProduct="SAUSA300_1731"/>
            </fbc:geneProductAssociation>
      </reaction>

      <reaction metaid="R_PEPSYN" id="R_PEPSYN" name="Peptidoglycan subunit
synthesis " reversible="true" fast="false" fbc:lowerFluxBound="rev_lb"
fbc:upperFluxBound="rev_ub">
            <listOfReactants>
                  <speciesReference species="M_pep_p1_c" stoichiometry="1"
constant="true"/>
                  <speciesReference species="M_uaagmda_c" stoichiometry="1"
constant="true"/>
            </listOfReactants>
```

```
            <listOfProducts>
                  <speciesReference species="M_udcpdp_c" stoichiometry="1"
constant="true"/>
                  <speciesReference species="M_pep_p_c" stoichiometry="1"
constant="true"/>
            </listOfProducts>
            <fbc:geneProductAssociation>
                  <fbc:or>
                        <fbc:geneProductRef fbc:geneProduct="SAUSA300_1341"/>
                        <fbc:geneProductRef fbc:geneProduct="SAUSA300_1676"/>
                  </fbc:or>
            </fbc:geneProductAssociation>
      </reaction>

      <reaction metaid="R_PFK" id="R_PFK" name="phosphofructokinase"
reversible="false" fast="false" fbc:lowerFluxBound="irr_lb"
fbc:upperFluxBound="irr_ub">
            <listOfReactants>
                  <speciesReference species="M_atp_c" stoichiometry="1"
constant="true"/>
                  <speciesReference species="M_f6p_c" stoichiometry="1"
constant="true"/>
            </listOfReactants>
            <listOfProducts>
                  <speciesReference species="M_adp_c" stoichiometry="1"
constant="true"/>
                  <speciesReference species="M_fdp_c" stoichiometry="1"
constant="true"/>
            </listOfProducts>
            <fbc:geneProductAssociation>
                  <fbc:geneProductRef fbc:geneProduct="SAUSA300_1645"/>
            </fbc:geneProductAssociation>
      </reaction>

      <reaction metaid="R_PFK_2" id="R_PFK_2" name="Phosphofructokinase"
reversible="false" fast="false" fbc:lowerFluxBound="irr_lb"
fbc:upperFluxBound="irr_ub">
            <listOfReactants>
                  <speciesReference species="M_atp_c" stoichiometry="1"
constant="true"/>
                  <speciesReference species="M_tag6p__D_c" stoichiometry="1"
constant="true"/>
            </listOfReactants>
            <listOfProducts>
                  <speciesReference species="M_adp_c" stoichiometry="1"
constant="true"/>
                  <speciesReference species="M_tagdp__D_c" stoichiometry="1"
constant="true"/>
            </listOfProducts>
            <fbc:geneProductAssociation>
                  <fbc:or>
                        <fbc:and>
                              <fbc:geneProductRef
fbc:geneProduct="SAUSA300_2153"/>
                              <fbc:geneProductRef
fbc:geneProduct="SAUSA300_0684"/>
                        </fbc:and>
                        <fbc:geneProductRef fbc:geneProduct="SAUSA300_1645"/>
```

```
                  &lt;/fbc:or&gt;
            &lt;/fbc:geneProductAssociation&gt;
      &lt;/reaction&gt;

      <reaction metaid="R_PFK_3" id="R_PFK_3" name="phosphofructokinase
(s7p)" reversible="false" fast="false" fbc:lowerFluxBound="irr_lb"
fbc:upperFluxBound="irr_ub">
            &lt;listOfReactants&gt;
                  <speciesReference species="M_atp_c" stoichiometry="1"
constant="true"/>
                  <speciesReference species="M_s7p_c" stoichiometry="1"
constant="true"/>
            &lt;/listOfReactants&gt;
            &lt;listOfProducts&gt;
                  <speciesReference species="M_adp_c" stoichiometry="1"
constant="true"/>
                  <speciesReference species="M_s17bp_c" stoichiometry="1"
constant="true"/>
            &lt;/listOfProducts&gt;
            &lt;fbc:geneProductAssociation&gt;
                  &lt;fbc:geneProductRef fbc:geneProduct="SAUSA300_1645"/&gt;
            &lt;/fbc:geneProductAssociation&gt;
      &lt;/reaction&gt;

      <reaction metaid="R_PFLr" id="R_PFLr" name="pyruvate formate lyase"
reversible="true" fast="false" fbc:lowerFluxBound="rev_lb"
fbc:upperFluxBound="rev_ub">
            &lt;listOfReactants&gt;
                  <speciesReference species="M_pyr_c" stoichiometry="1"
constant="true"/>
                  <speciesReference species="M_coa_c" stoichiometry="1"
constant="true"/>
            &lt;/listOfReactants&gt;
            &lt;listOfProducts&gt;
                  <speciesReference species="M_accoa_c" stoichiometry="1"
constant="true"/>
                  <speciesReference species="M_for_c" stoichiometry="1"
constant="true"/>
            &lt;/listOfProducts&gt;
            &lt;fbc:geneProductAssociation&gt;
                  &lt;fbc:or&gt;
                        &lt;fbc:geneProductRef fbc:geneProduct="SAUSA300_0220"/&gt;
                        &lt;fbc:geneProductRef fbc:geneProduct="SAUSA300_0221"/&gt;
                  &lt;/fbc:or&gt;
            &lt;/fbc:geneProductAssociation&gt;
      &lt;/reaction&gt;

      <reaction metaid="R_PGAMT" id="R_PGAMT" name="phosphoglucosamine
mutase" reversible="true" fast="false" fbc:lowerFluxBound="rev_lb"
fbc:upperFluxBound="rev_ub">
            &lt;listOfReactants&gt;
                  <speciesReference species="M_gam1p_c" stoichiometry="1"
constant="true"/>
            &lt;/listOfReactants&gt;
            &lt;listOfProducts&gt;
                  <speciesReference species="M_gam6p_c" stoichiometry="1"
constant="true"/>
            &lt;/listOfProducts&gt;
```

```
            <fbc:geneProductAssociation>
                  <fbc:geneProductRef fbc:geneProduct="SAUSA300_2111"/>
            </fbc:geneProductAssociation>
      </reaction>

      <reaction metaid="R_PGCDr" id="R_PGCDr" name="phosphoglycerate
dehydrogenase reversible" reversible="true" fast="false"
fbc:lowerFluxBound="rev_lb" fbc:upperFluxBound="rev_ub">
            <listOfReactants>
                  <speciesReference species="M_nad_c" stoichiometry="1"
constant="true"/>
                  <speciesReference species="M_3pg_c" stoichiometry="1"
constant="true"/>
            </listOfReactants>
            <listOfProducts>
                  <speciesReference species="M_h_c" stoichiometry="1"
constant="true"/>
                  <speciesReference species="M_nadh_c" stoichiometry="1"
constant="true"/>
                  <speciesReference species="M_3php_c" stoichiometry="1"
constant="true"/>
            </listOfProducts>
            <fbc:geneProductAssociation>
                  <fbc:geneProductRef fbc:geneProduct="SAUSA300_1670"/>
            </fbc:geneProductAssociation>
      </reaction>

      <reaction metaid="R_PGI" id="R_PGI" name="glucose-6-phosphate
isomerase" reversible="true" fast="false" fbc:lowerFluxBound="rev_lb"
fbc:upperFluxBound="rev_ub">
            <listOfReactants>
                  <speciesReference species="M_g6p_c" stoichiometry="1"
constant="true"/>
            </listOfReactants>
            <listOfProducts>
                  <speciesReference species="M_f6p_c" stoichiometry="1"
constant="true"/>
            </listOfProducts>
            <fbc:geneProductAssociation>
                  <fbc:geneProductRef fbc:geneProduct="SAUSA300_0865"/>
            </fbc:geneProductAssociation>
      </reaction>

      <reaction metaid="R_PGK" id="R_PGK" name="phosphoglycerate kinase"
reversible="true" fast="false" fbc:lowerFluxBound="rev_lb"
fbc:upperFluxBound="rev_ub">
            <listOfReactants>
                  <speciesReference species="M_h_c" stoichiometry="1"
constant="true"/>
                  <speciesReference species="M_atp_c" stoichiometry="1"
constant="true"/>
                  <speciesReference species="M_3pg_c" stoichiometry="1"
constant="true"/>
            </listOfReactants>
            <listOfProducts>
                  <speciesReference species="M_adp_c" stoichiometry="1"
constant="true"/>
```

```
                  <speciesReference species="M_13dpg_c" stoichiometry="1"
constant="true"/>
            &lt;/listOfProducts&gt;
            &lt;fbc:geneProductAssociation&gt;
                  &lt;fbc:geneProductRef fbc:geneProduct="SAUSA300_0757"/&gt;
            &lt;/fbc:geneProductAssociation&gt;
      &lt;/reaction&gt;

      <reaction metaid="R_PGLer" id="R_PGLer" name="6-
phosphogluconolactonase, endoplasmic reticulum" reversible="true"
fast="false" fbc:lowerFluxBound="rev_lb" fbc:upperFluxBound="rev_ub">
            &lt;listOfReactants&gt;
                  <speciesReference species="M_h2o_c" stoichiometry="1"
constant="true"/>
                  <speciesReference species="M_6pgl_c" stoichiometry="1"
constant="true"/>
            &lt;/listOfReactants&gt;
            &lt;listOfProducts&gt;
                  <speciesReference species="M_h_c" stoichiometry="1"
constant="true"/>
                  <speciesReference species="M_6pgc_c" stoichiometry="1"
constant="true"/>
            &lt;/listOfProducts&gt;
            &lt;fbc:geneProductAssociation&gt;
                  &lt;fbc:geneProductRef fbc:geneProduct="SAUSA300_1902"/&gt;
            &lt;/fbc:geneProductAssociation&gt;
      &lt;/reaction&gt;

      <reaction metaid="R_PGLYCP" id="R_PGLYCP" name="Phosphoglycolate
phosphatase" reversible="false" fast="false" fbc:lowerFluxBound="irr_lb"
fbc:upperFluxBound="irr_ub">
            &lt;listOfReactants&gt;
                  <speciesReference species="M_h2o_c" stoichiometry="1"
constant="true"/>
                  <speciesReference species="M_2pglyc_c" stoichiometry="1"
constant="true"/>
            &lt;/listOfReactants&gt;
            &lt;listOfProducts&gt;
                  <speciesReference species="M_h_c" stoichiometry="1"
constant="true"/>
                  <speciesReference species="M_pi_c" stoichiometry="1"
constant="true"/>
                  <speciesReference species="M_glyclt_c" stoichiometry="1"
constant="true"/>
            &lt;/listOfProducts&gt;
            &lt;fbc:geneProductAssociation&gt;
                  &lt;fbc:or&gt;
                        &lt;fbc:geneProductRef fbc:geneProduct="SAUSA300_0557"/&gt;
                        &lt;fbc:geneProductRef fbc:geneProduct="SAUSA300_0833"/&gt;
                  &lt;/fbc:or&gt;
            &lt;/fbc:geneProductAssociation&gt;
      &lt;/reaction&gt;

      <reaction metaid="R_PGLYSA_SA2" id="R_PGLYSA_SA2"
name="Phosphatidylglycine Synthase (SA) 2" reversible="true" fast="false"
fbc:lowerFluxBound="rev_lb" fbc:upperFluxBound="rev_ub">
            &lt;listOfReactants&gt;
```

```
                  <speciesReference species="M_gly_c" stoichiometry="1"
constant="true"/>
                  <speciesReference species="M_cdpdag_SA_c"
stoichiometry="0.02" constant="true"/>
            </listOfReactants>
            <listOfProducts>
                  <speciesReference species="M_h_c" stoichiometry="1"
constant="true"/>
                  <speciesReference species="M_cmp_c" stoichiometry="1"
constant="true"/>
                  <speciesReference species="M_pgly_SA2_c"
stoichiometry="0.02" constant="true"/>
            </listOfProducts>
      </reaction>

      <reaction metaid="R_PGM" id="R_PGM" name="phosphoglycerate mutase"
reversible="true" fast="false" fbc:lowerFluxBound="rev_lb"
fbc:upperFluxBound="rev_ub">
            <listOfReactants>
                  <speciesReference species="M_2pg_c" stoichiometry="1"
constant="true"/>
            </listOfReactants>
            <listOfProducts>
                  <speciesReference species="M_3pg_c" stoichiometry="1"
constant="true"/>
            </listOfProducts>
            <fbc:geneProductAssociation>
                  <fbc:or>
                        <fbc:geneProductRef fbc:geneProduct="SAUSA300_2362"/>
                        <fbc:geneProductRef fbc:geneProduct="SAUSA300_0759"/>
                        <fbc:geneProductRef fbc:geneProduct="SAUSA300_0375"/>
                  </fbc:or>
            </fbc:geneProductAssociation>
      </reaction>

      <reaction metaid="R_PGMT" id="R_PGMT" name="phosphoglucomutase"
reversible="true" fast="false" fbc:lowerFluxBound="rev_lb"
fbc:upperFluxBound="rev_ub">
            <listOfReactants>
                  <speciesReference species="M_g1p_c" stoichiometry="1"
constant="true"/>
            </listOfReactants>
            <listOfProducts>
                  <speciesReference species="M_g6p_c" stoichiometry="1"
constant="true"/>
            </listOfProducts>
            <fbc:geneProductAssociation>
                  <fbc:geneProductRef fbc:geneProduct="SAUSA300_2111"/>
            </fbc:geneProductAssociation>
      </reaction>

      <reaction metaid="R_PGPP_SA" id="R_PGPP_SA" name="Phosphatidylglycerol
phosphate phosphatase (Saureus)" reversible="false" fast="false"
fbc:lowerFluxBound="irr_lb" fbc:upperFluxBound="irr_ub">
            <listOfReactants>
                  <speciesReference species="M_h2o_c" stoichiometry="1"
constant="true"/>
```

```
                    <speciesReference species="M_pgp_SA_c" stoichiometry="0.02"
constant="true"/>
              </listOfReactants>
              <listOfProducts>
                    <speciesReference species="M_pi_c" stoichiometry="1"
constant="true"/>
                    <speciesReference species="M_pg_SA_c" stoichiometry="0.02"
constant="true"/>
              </listOfProducts>
              <fbc:geneProductAssociation>
                    <fbc:geneProductRef fbc:geneProduct="SAUSA300_1310"/>
              </fbc:geneProductAssociation>
        </reaction>

        <reaction metaid="R_PGSA_SA" id="R_PGSA_SA" name="Phosphatidylglycerol
synthase (Saureus)" reversible="true" fast="false"
fbc:lowerFluxBound="rev_lb" fbc:upperFluxBound="rev_ub">
              <listOfReactants>
                    <speciesReference species="M_glyc3p_c" stoichiometry="1"
constant="true"/>
                    <speciesReference species="M_cdpdag_SA_c"
stoichiometry="0.02" constant="true"/>
              </listOfReactants>
              <listOfProducts>
                    <speciesReference species="M_h_c" stoichiometry="1"
constant="true"/>
                    <speciesReference species="M_cmp_c" stoichiometry="1"
constant="true"/>
                    <speciesReference species="M_pgp_SA_c" stoichiometry="0.02"
constant="true"/>
              </listOfProducts>
              <fbc:geneProductAssociation>
                    <fbc:geneProductRef fbc:geneProduct="SAUSA300_1176"/>
              </fbc:geneProductAssociation>
        </reaction>

        <reaction metaid="R_PGSA120" id="R_PGSA120" name="Phosphatidylglycerol
synthase (n-C12:0)" reversible="false" fast="false"
fbc:lowerFluxBound="irr_lb" fbc:upperFluxBound="irr_ub">
              <listOfReactants>
                    <speciesReference species="M_glyc3p_c" stoichiometry="1"
constant="true"/>
                    <speciesReference species="M_cdpdddecg_c" stoichiometry="1"
constant="true"/>
              </listOfReactants>
              <listOfProducts>
                    <speciesReference species="M_cmp_c" stoichiometry="1"
constant="true"/>
                    <speciesReference species="M_pgp120_c" stoichiometry="1"
constant="true"/>
              </listOfProducts>
              <fbc:geneProductAssociation>
                    <fbc:geneProductRef fbc:geneProduct="SAUSA300_1176"/>
              </fbc:geneProductAssociation>
        </reaction>
```

```
      <reaction metaid="R_PGSA140" id="R_PGSA140" name="Phosphatidylglycerol
synthase (n-C14:0)" reversible="false" fast="false"
fbc:lowerFluxBound="irr_lb" fbc:upperFluxBound="irr_ub">
            <listOfReactants>
                  <speciesReference species="M_glyc3p_c" stoichiometry="1"
constant="true"/>
                  <speciesReference species="M_cdpdtdecg_c" stoichiometry="1"
constant="true"/>
            </listOfReactants>
            <listOfProducts>
                  <speciesReference species="M_cmp_c" stoichiometry="1"
constant="true"/>
                  <speciesReference species="M_pgp140_c" stoichiometry="1"
constant="true"/>
            </listOfProducts>
            <fbc:geneProductAssociation>
                  <fbc:geneProductRef fbc:geneProduct="SAUSA300_1176"/>
            </fbc:geneProductAssociation>
      </reaction>

      <reaction metaid="R_PGSA141" id="R_PGSA141" name="Phosphatidylglycerol
synthase (n-C14:1)" reversible="false" fast="false"
fbc:lowerFluxBound="irr_lb" fbc:upperFluxBound="irr_ub">
            <listOfReactants>
                  <speciesReference species="M_glyc3p_c" stoichiometry="1"
constant="true"/>
                  <speciesReference species="M_cdpdtdec7eg_c"
stoichiometry="1" constant="true"/>
            </listOfReactants>
            <listOfProducts>
                  <speciesReference species="M_cmp_c" stoichiometry="1"
constant="true"/>
                  <speciesReference species="M_pgp141_c" stoichiometry="1"
constant="true"/>
            </listOfProducts>
            <fbc:geneProductAssociation>
                  <fbc:geneProductRef fbc:geneProduct="SAUSA300_1176"/>
            </fbc:geneProductAssociation>
      </reaction>

      <reaction metaid="R_PGSA160" id="R_PGSA160" name="Phosphatidylglycerol
synthase (n-C16:0)" reversible="false" fast="false"
fbc:lowerFluxBound="irr_lb" fbc:upperFluxBound="irr_ub">
            <listOfReactants>
                  <speciesReference species="M_glyc3p_c" stoichiometry="1"
constant="true"/>
                  <speciesReference species="M_cdpdhdecg_c" stoichiometry="1"
constant="true"/>
            </listOfReactants>
            <listOfProducts>
                  <speciesReference species="M_cmp_c" stoichiometry="1"
constant="true"/>
                  <speciesReference species="M_pgp160_c" stoichiometry="1"
constant="true"/>
            </listOfProducts>
            <fbc:geneProductAssociation>
                  <fbc:geneProductRef fbc:geneProduct="SAUSA300_1176"/>
            </fbc:geneProductAssociation>
```

```
      </reaction>

      <reaction metaid="R_PGSA161" id="R_PGSA161" name="Phosphatidylglycerol
synthase (n-C16:1)" reversible="false" fast="false"
fbc:lowerFluxBound="irr_lb" fbc:upperFluxBound="irr_ub">
            <listOfReactants>
                  <speciesReference species="M_glyc3p_c" stoichiometry="1"
constant="true"/>
                  <speciesReference species="M_cdpdhdec9eg_c"
stoichiometry="1" constant="true"/>
            </listOfReactants>
            <listOfProducts>
                  <speciesReference species="M_cmp_c" stoichiometry="1"
constant="true"/>
                  <speciesReference species="M_pgp161_c" stoichiometry="1"
constant="true"/>
            </listOfProducts>
            <fbc:geneProductAssociation>
                  <fbc:geneProductRef fbc:geneProduct="SAUSA300_1176"/>
            </fbc:geneProductAssociation>
      </reaction>

      <reaction metaid="R_PGSA180" id="R_PGSA180" name="Phosphatidylglycerol
synthase (n-C18:0)" reversible="false" fast="false"
fbc:lowerFluxBound="irr_lb" fbc:upperFluxBound="irr_ub">
            <listOfReactants>
                  <speciesReference species="M_glyc3p_c" stoichiometry="1"
constant="true"/>
                  <speciesReference species="M_cdpdodecg_c" stoichiometry="1"
constant="true"/>
            </listOfReactants>
            <listOfProducts>
                  <speciesReference species="M_cmp_c" stoichiometry="1"
constant="true"/>
                  <speciesReference species="M_pgp180_c" stoichiometry="1"
constant="true"/>
            </listOfProducts>
            <fbc:geneProductAssociation>
                  <fbc:geneProductRef fbc:geneProduct="SAUSA300_1176"/>
            </fbc:geneProductAssociation>
      </reaction>

      <reaction metaid="R_PGSA181" id="R_PGSA181" name="Phosphatidylglycerol
synthase (n-C18:1)" reversible="false" fast="false"
fbc:lowerFluxBound="irr_lb" fbc:upperFluxBound="irr_ub">
            <listOfReactants>
                  <speciesReference species="M_glyc3p_c" stoichiometry="1"
constant="true"/>
                  <speciesReference species="M_cdpdodec11eg_c"
stoichiometry="1" constant="true"/>
            </listOfReactants>
            <listOfProducts>
                  <speciesReference species="M_cmp_c" stoichiometry="1"
constant="true"/>
                  <speciesReference species="M_pgp181_c" stoichiometry="1"
constant="true"/>
            </listOfProducts>
            <fbc:geneProductAssociation>
```

```
                  <fbc:geneProductRef fbc:geneProduct="SAUSA300_1176"/>
            </fbc:geneProductAssociation>
      </reaction>

      <reaction metaid="R_PHETA1" id="R_PHETA1" name="phenylalanine
transaminase" reversible="true" fast="false" fbc:lowerFluxBound="rev_lb"
fbc:upperFluxBound="rev_ub">
            <listOfReactants>
                  <speciesReference species="M_akg_c" stoichiometry="1"
constant="true"/>
                  <speciesReference species="M_phe__L_c" stoichiometry="1"
constant="true"/>
            </listOfReactants>
            <listOfProducts>
                  <speciesReference species="M_glu__L_c" stoichiometry="1"
constant="true"/>
                  <speciesReference species="M_phpyr_c" stoichiometry="1"
constant="true"/>
            </listOfProducts>
            <fbc:geneProductAssociation>
                  <fbc:or>
                        <fbc:and>
                              <fbc:geneProductRef
fbc:geneProduct="SAUSA300_0708"/>
                              <fbc:geneProductRef
fbc:geneProduct="SAUSA300_2610"/>
                        </fbc:and>
                        <fbc:geneProductRef fbc:geneProduct="SAUSA300_1916"/>
                  </fbc:or>
            </fbc:geneProductAssociation>
      </reaction>

      <reaction metaid="R_PIabc" id="R_PIabc" name="phosphate transport via
ABC system" reversible="false" fast="false" fbc:lowerFluxBound="irr_lb"
fbc:upperFluxBound="irr_ub">
            <listOfReactants>
                  <speciesReference species="M_h2o_c" stoichiometry="1"
constant="true"/>
                  <speciesReference species="M_atp_c" stoichiometry="1"
constant="true"/>
                  <speciesReference species="M_pi_e" stoichiometry="1"
constant="true"/>
            </listOfReactants>
            <listOfProducts>
                  <speciesReference species="M_h_c" stoichiometry="1"
constant="true"/>
                  <speciesReference species="M_pi_c" stoichiometry="2"
constant="true"/>
                  <speciesReference species="M_adp_c" stoichiometry="1"
constant="true"/>
            </listOfProducts>
            <fbc:geneProductAssociation>
                  <fbc:and>
                        <fbc:geneProductRef fbc:geneProduct="SAUSA300_1283"/>
                        <fbc:geneProductRef fbc:geneProduct="SAUSA300_1282"/>
                        <fbc:geneProductRef fbc:geneProduct="SAUSA300_1281"/>
                        <fbc:geneProductRef fbc:geneProduct="SAUSA300_1280"/>
                  </fbc:and>
```

```
            </fbc:geneProductAssociation>
      </reaction>

      <reaction metaid="R_PIt2" id="R_PIt2" name="phosphate transport in via
proton symport" reversible="false" fast="false" fbc:lowerFluxBound="irr_lb"
fbc:upperFluxBound="irr_ub">
            <listOfReactants>
                  <speciesReference species="M_h_c" stoichiometry="1"
constant="true"/>
                  <speciesReference species="M_pi_c" stoichiometry="1"
constant="true"/>
            </listOfReactants>
            <listOfProducts>
                  <speciesReference species="M_h_e" stoichiometry="1"
constant="true"/>
                  <speciesReference species="M_pi_e" stoichiometry="1"
constant="true"/>
            </listOfProducts>
            <fbc:geneProductAssociation>
                  <fbc:geneProductRef fbc:geneProduct="SAUSA300_0650"/>
            </fbc:geneProductAssociation>
      </reaction>

      <reaction metaid="R_PIt2rpp" id="R_PIt2rpp" name="phosphate reversible
transport via symport (periplasm)" reversible="true" fast="false"
fbc:lowerFluxBound="rev_lb" fbc:upperFluxBound="rev_ub">
            <listOfReactants>
                  <speciesReference species="M_h_p" stoichiometry="1"
constant="true"/>
                  <speciesReference species="M_pi_p" stoichiometry="1"
constant="true"/>
            </listOfReactants>
            <listOfProducts>
                  <speciesReference species="M_h_c" stoichiometry="1"
constant="true"/>
                  <speciesReference species="M_pi_c" stoichiometry="1"
constant="true"/>
            </listOfProducts>
      </reaction>

      <reaction metaid="R_PIt7" id="R_PIt7" name="phosphate transport in/out
via three Na+ symporter" reversible="false" fast="false"
fbc:lowerFluxBound="irr_lb" fbc:upperFluxBound="irr_ub">
            <listOfReactants>
                  <speciesReference species="M_na1_e" stoichiometry="3"
constant="true"/>
                  <speciesReference species="M_pi_e" stoichiometry="1"
constant="true"/>
            </listOfReactants>
            <listOfProducts>
                  <speciesReference species="M_pi_c" stoichiometry="1"
constant="true"/>
                  <speciesReference species="M_na1_c" stoichiometry="3"
constant="true"/>
            </listOfProducts>
            <fbc:geneProductAssociation>
                  <fbc:geneProductRef fbc:geneProduct="SAUSA300_0107"/>
            </fbc:geneProductAssociation>
```

```
      </reaction>

      <reaction metaid="R_PLAMGLS" id="R_PLAMGLS"
name="palmitoyl__Lipoteichoic acid synthesis n=24  linked  glucose
substituted " reversible="true" fast="false" fbc:lowerFluxBound="rev_lb"
fbc:upperFluxBound="rev_ub">
            <listOfReactants>
                  <speciesReference species="M_udpg_c" stoichiometry="24"
constant="true"/>
                  <speciesReference species="M_palm24u_c" stoichiometry="1"
constant="true"/>
            </listOfReactants>
            <listOfProducts>
                  <speciesReference species="M_udp_c" stoichiometry="24"
constant="true"/>
                  <speciesReference species="M_palmt24s_c" stoichiometry="1"
constant="true"/>
            </listOfProducts>
            <fbc:geneProductAssociation>
                  <fbc:or>
                        <fbc:geneProductRef fbc:geneProduct="SAUSA300_0939"/>
                        <fbc:geneProductRef fbc:geneProduct="SAUSA300_0550"/>
                        <fbc:geneProductRef fbc:geneProduct="SAUSA300_0549"/>
                  </fbc:or>
            </fbc:geneProductAssociation>
      </reaction>

      <reaction metaid="R_PLEUSA_SA2" id="R_PLEUSA_SA2"
name="Phosphatidylleucine Synthase (SA) 2" reversible="true" fast="false"
fbc:lowerFluxBound="rev_lb" fbc:upperFluxBound="rev_ub">
            <listOfReactants>
                  <speciesReference species="M_leu__L_c" stoichiometry="1"
constant="true"/>
                  <speciesReference species="M_cdpdag_SA_c"
stoichiometry="0.02" constant="true"/>
            </listOfReactants>
            <listOfProducts>
                  <speciesReference species="M_h_c" stoichiometry="1"
constant="true"/>
                  <speciesReference species="M_cmp_c" stoichiometry="1"
constant="true"/>
                  <speciesReference species="M_pleu_SA2_c"
stoichiometry="0.02" constant="true"/>
            </listOfProducts>
      </reaction>

      <reaction metaid="R_PLIPA2A180b" id="R_PLIPA2A180b" name="stearoyl-
glycerol-3-phosphate O-acyltransferase " reversible="false" fast="false"
fbc:lowerFluxBound="irr_lb" fbc:upperFluxBound="irr_ub">
            <listOfReactants>
                  <speciesReference species="M_glyc3p_c" stoichiometry="1"
constant="true"/>
                  <speciesReference species="M_stcoa_c" stoichiometry="1"
constant="true"/>
            </listOfReactants>
            <listOfProducts>
                  <speciesReference species="M_coa_c" stoichiometry="1"
constant="true"/>
```

```
                  <speciesReference species="M_1odecg3p_c" stoichiometry="1"
constant="true"/>
            </listOfProducts>
            <fbc:geneProductAssociation>
                  <fbc:and>
                        <fbc:geneProductRef fbc:geneProduct="SAUSA300_1249"/>
                        <fbc:or>
                              <fbc:geneProductRef
fbc:geneProduct="SAUSA300_1122"/>
                              <fbc:geneProductRef
fbc:geneProduct="SAUSA300_1121"/>
                        </fbc:or>
                  </fbc:and>
            </fbc:geneProductAssociation>
      </reaction>

      <reaction metaid="R_PLMET" id="R_PLMET" name="Plasmenylethanolamine
ethanolamine phosphohydrolase " reversible="true" fast="false"
fbc:lowerFluxBound="rev_lb" fbc:upperFluxBound="rev_ub">
            <listOfReactants>
                  <speciesReference species="M_h_c" stoichiometry="1"
constant="true"/>
                  <speciesReference species="M_h2o_c" stoichiometry="1"
constant="true"/>
                  <speciesReference species="M_3h5ox" stoichiometry="1"
constant="true"/>
            </listOfReactants>
            <listOfProducts>
                  <speciesReference species="M_ethamp_c" stoichiometry="1"
constant="true"/>
                  <speciesReference species="M_acgly" stoichiometry="1"
constant="true"/>
            </listOfProducts>
            <fbc:geneProductAssociation>
                  <fbc:geneProductRef fbc:geneProduct="SAUSA300_1973"/>
            </fbc:geneProductAssociation>
      </reaction>

      <reaction metaid="R_PLPS" id="R_PLPS" name="Pyridoxal-5-phosphate
synthase" reversible="false" fast="false" fbc:lowerFluxBound="irr_lb"
fbc:upperFluxBound="irr_ub">
            <listOfReactants>
                  <speciesReference species="M_gln__L_c" stoichiometry="1"
constant="true"/>
                  <speciesReference species="M_ru5p__D_c" stoichiometry="1"
constant="true"/>
                  <speciesReference species="M_g3p_c" stoichiometry="1"
constant="true"/>
            </listOfReactants>
            <listOfProducts>
                  <speciesReference species="M_h_c" stoichiometry="2"
constant="true"/>
                  <speciesReference species="M_h2o_c" stoichiometry="3"
constant="true"/>
                  <speciesReference species="M_glu__L_c" stoichiometry="1"
constant="true"/>
                  <speciesReference species="M_pi_c" stoichiometry="1"
constant="true"/>
```

```
                  <speciesReference species="M_pydx5p_c" stoichiometry="1"
constant="true"/>
            </listOfProducts>
            <fbc:geneProductAssociation>
                  <fbc:or>
                        <fbc:geneProductRef fbc:geneProduct="SAUSA300_0505"/>
                        <fbc:geneProductRef fbc:geneProduct="SAUSA300_0504"/>
                        <fbc:geneProductRef fbc:geneProduct="SAUSA300_1082"/>
                  </fbc:or>
            </fbc:geneProductAssociation>
      </reaction>

      <reaction metaid="R_PLYAGTR" id="R_PLYAGTR" name="Polyglycerol-
phosphate alpha-glucosyltransferase " reversible="true" fast="false"
fbc:lowerFluxBound="rev_lb" fbc:upperFluxBound="rev_ub">
            <listOfReactants>
                  <speciesReference species="M_pren45_c" stoichiometry="1"
constant="true"/>
                  <speciesReference species="M_udpg_c" stoichiometry="45"
constant="true"/>
            </listOfReactants>
            <listOfProducts>
                  <speciesReference species="M_udp_c" stoichiometry="45"
constant="true"/>
                  <speciesReference species="M_pren45glc_c" stoichiometry="1"
constant="true"/>
            </listOfProducts>
            <fbc:geneProductAssociation>
                  <fbc:or>
                        <fbc:geneProductRef fbc:geneProduct="SAUSA300_0939"/>
                        <fbc:geneProductRef fbc:geneProduct="SAUSA300_0550"/>
                        <fbc:geneProductRef fbc:geneProduct="SAUSA300_0549"/>
                  </fbc:or>
            </fbc:geneProductAssociation>
      </reaction>

      <reaction metaid="R_PLYSSA_SA2" id="R_PLYSSA_SA2"
name="Phosphatidyllysine Synthase (SA) 2" reversible="true" fast="false"
fbc:lowerFluxBound="rev_lb" fbc:upperFluxBound="rev_ub">
            <listOfReactants>
                  <speciesReference species="M_lys__L_c" stoichiometry="1"
constant="true"/>
                  <speciesReference species="M_cdpdag_SA_c"
stoichiometry="0.02" constant="true"/>
            </listOfReactants>
            <listOfProducts>
                  <speciesReference species="M_h_c" stoichiometry="1"
constant="true"/>
                  <speciesReference species="M_cmp_c" stoichiometry="1"
constant="true"/>
                  <speciesReference species="M_plys_SA2_c"
stoichiometry="0.02" constant="true"/>
            </listOfProducts>
      </reaction>

      <reaction metaid="R_PMANM" id="R_PMANM" name="phosphomannomutase"
reversible="true" fast="false" fbc:lowerFluxBound="rev_lb"
fbc:upperFluxBound="rev_ub">
```

```
            <listOfReactants>
                  <speciesReference species="M_man6p_c" stoichiometry="1"
constant="true"/>
            </listOfReactants>
            <listOfProducts>
                  <speciesReference species="M_man1p_c" stoichiometry="1"
constant="true"/>
            </listOfProducts>
            <fbc:geneProductAssociation>
                  <fbc:geneProductRef fbc:geneProduct="SAUSA300_2433"/>
            </fbc:geneProductAssociation>
      </reaction>

      <reaction metaid="R_PMDPHT" id="R_PMDPHT" name="pyrimidine phosphatase"
reversible="false" fast="false" fbc:lowerFluxBound="irr_lb"
fbc:upperFluxBound="irr_ub">
            <listOfReactants>
                  <speciesReference species="M_h2o_c" stoichiometry="1"
constant="true"/>
                  <speciesReference species="M_5aprbu_c" stoichiometry="1"
constant="true"/>
            </listOfReactants>
            <listOfProducts>
                  <speciesReference species="M_pi_c" stoichiometry="1"
constant="true"/>
                  <speciesReference species="M_4r5au_c" stoichiometry="1"
constant="true"/>
            </listOfProducts>
            <fbc:geneProductAssociation>
                  <fbc:and>
                        <fbc:geneProductRef fbc:geneProduct="SAUSA300_1714"/>
                        <fbc:geneProductRef fbc:geneProduct="SAUSA300_1712"/>
                  </fbc:and>
            </fbc:geneProductAssociation>
      </reaction>

      <reaction metaid="R_PMEVK" id="R_PMEVK" name="phosphomevalonate kinase"
reversible="false" fast="false" fbc:lowerFluxBound="irr_lb"
fbc:upperFluxBound="irr_ub">
            <listOfReactants>
                  <speciesReference species="M_atp_c" stoichiometry="1"
constant="true"/>
                  <speciesReference species="M_5pmev_c" stoichiometry="1"
constant="true"/>
            </listOfReactants>
            <listOfProducts>
                  <speciesReference species="M_adp_c" stoichiometry="1"
constant="true"/>
                  <speciesReference species="M_5dpmev_c" stoichiometry="1"
constant="true"/>
            </listOfProducts>
            <fbc:geneProductAssociation>
                  <fbc:geneProductRef fbc:geneProduct="SAUSA300_0574"/>
            </fbc:geneProductAssociation>
      </reaction>
```

```
      <reaction metaid="R_PMPK" id="R_PMPK" name="phosphomethylpyrimidine
kinase" reversible="false" fast="false" fbc:lowerFluxBound="irr_lb"
fbc:upperFluxBound="irr_ub">
            <listOfReactants>
                  <speciesReference species="M_atp_c" stoichiometry="1"
constant="true"/>
                  <speciesReference species="M_4ampm_c" stoichiometry="1"
constant="true"/>
            </listOfReactants>
            <listOfProducts>
                  <speciesReference species="M_adp_c" stoichiometry="1"
constant="true"/>
                  <speciesReference species="M_2mahmp_c" stoichiometry="1"
constant="true"/>
            </listOfProducts>
            <fbc:geneProductAssociation>
                  <fbc:or>
                        <fbc:geneProductRef fbc:geneProduct="SAUSA300_0562"/>
                        <fbc:geneProductRef fbc:geneProduct="SAUSA300_2049"/>
                  </fbc:or>
            </fbc:geneProductAssociation>
      </reaction>

      <reaction metaid="R_PMPTCA" id="R_PMPTCA" name="palmitoyl-1-
acylglycerol-3-phosphate O-acyltransferase " reversible="false" fast="false"
fbc:lowerFluxBound="irr_lb" fbc:upperFluxBound="irr_ub">
            <listOfReactants>
                  <speciesReference species="M_1hdecg3p_c" stoichiometry="1"
constant="true"/>
                  <speciesReference species="M_pmtcoa_c" stoichiometry="1"
constant="true"/>
            </listOfReactants>
            <listOfProducts>
                  <speciesReference species="M_coa_c" stoichiometry="1"
constant="true"/>
                  <speciesReference species="M_pa160_c" stoichiometry="1"
constant="true"/>
            </listOfProducts>
            <fbc:geneProductAssociation>
                  <fbc:geneProductRef fbc:geneProduct="SAUSA300_1673"/>
            </fbc:geneProductAssociation>
      </reaction>

      <reaction metaid="R_PMTSS" id="R_PMTSS" name="peptide-methionine
thioredoxin__Disulfide S-oxidoreductase " reversible="true" fast="false"
fbc:lowerFluxBound="rev_lb" fbc:upperFluxBound="rev_ub">
            <listOfReactants>
                  <speciesReference species="M_h2o_c" stoichiometry="1"
constant="true"/>
                  <speciesReference species="M_trdox_c" stoichiometry="1"
constant="true"/>
                  <speciesReference species="M_pep_met__L_c"
stoichiometry="1" constant="true"/>
            </listOfReactants>
            <listOfProducts>
                  <speciesReference species="M_trdrd_c" stoichiometry="1"
constant="true"/>
```

```
                  <speciesReference species="M_pep_met__LRS_c"
stoichiometry="1" constant="true"/>
            </listOfProducts>
            <fbc:geneProductAssociation>
                  <fbc:geneProductRef fbc:geneProduct="SAUSA300_1316"/>
            </fbc:geneProductAssociation>
      </reaction>

      <reaction metaid="R_PNP" id="R_PNP" name="purine-nucleoside
phosphorylase" reversible="true" fast="false" fbc:lowerFluxBound="rev_lb"
fbc:upperFluxBound="rev_ub">
            <listOfReactants>
                  <speciesReference species="M_pi_c" stoichiometry="1"
constant="true"/>
                  <speciesReference species="M_rnam_c" stoichiometry="1"
constant="true"/>
            </listOfReactants>
            <listOfProducts>
                  <speciesReference species="M_r1p_c" stoichiometry="1"
constant="true"/>
                  <speciesReference species="M_ncam_c" stoichiometry="1"
constant="true"/>
            </listOfProducts>
            <fbc:geneProductAssociation>
                  <fbc:or>
                        <fbc:geneProductRef fbc:geneProduct="SAUSA300_0138"/>
                        <fbc:geneProductRef fbc:geneProduct="SAUSA300_2091"/>
                  </fbc:or>
            </fbc:geneProductAssociation>
      </reaction>

      <reaction metaid="R_PNPA3" id="R_PNPA3" name="Protein-Npai-
phosphohistidine sugar Npai-phosphotransferase " reversible="true"
fast="false" fbc:lowerFluxBound="rev_lb" fbc:upperFluxBound="rev_ub">
            <listOfReactants>
                  <speciesReference species="M_h_c" stoichiometry="1"
constant="true"/>
                  <speciesReference species="M_malt_c" stoichiometry="1"
constant="true"/>
                  <speciesReference species="M_pplhis" stoichiometry="1"
constant="true"/>
            </listOfReactants>
            <listOfProducts>
                  <speciesReference species="M_malt6p_c" stoichiometry="1"
constant="true"/>
                  <speciesReference species="M_phis__L" stoichiometry="1"
constant="true"/>
            </listOfProducts>
            <fbc:geneProductAssociation>
                  <fbc:geneProductRef fbc:geneProduct="SAUSA300_1315"/>
            </fbc:geneProductAssociation>
      </reaction>

      <reaction metaid="R_PNPA5" id="R_PNPA5" name="Protein-Npai-
phosphohistidine sugar Npai-phosphotransferase " reversible="true"
fast="false" fbc:lowerFluxBound="rev_lb" fbc:upperFluxBound="rev_ub">
            <listOfReactants>
```

```
                  <speciesReference species="M_h_c" stoichiometry="1"
constant="true"/>
                  <speciesReference species="M_pplhis" stoichiometry="1"
constant="true"/>
                  <speciesReference species="M_rbl__B_c" stoichiometry="1"
constant="true"/>
            </listOfReactants>
            <listOfProducts>
                  <speciesReference species="M_salc6p_c" stoichiometry="1"
constant="true"/>
                  <speciesReference species="M_phis__L" stoichiometry="1"
constant="true"/>
            </listOfProducts>
            <fbc:geneProductAssociation>
                  <fbc:geneProductRef fbc:geneProduct="SAUSA300_1315"/>
            </fbc:geneProductAssociation>
      </reaction>

      <reaction metaid="R_PNPA6" id="R_PNPA6" name="Protein-Npai-
phosphohistidine sugar Npai-phosphotransferase " reversible="true"
fast="false" fbc:lowerFluxBound="rev_lb" fbc:upperFluxBound="rev_ub">
            <listOfReactants>
                  <speciesReference species="M_h_c" stoichiometry="1"
constant="true"/>
                  <speciesReference species="M_pplhis" stoichiometry="1"
constant="true"/>
                  <speciesReference species="M_arbt_c" stoichiometry="1"
constant="true"/>
            </listOfReactants>
            <listOfProducts>
                  <speciesReference species="M_arbt6p_c" stoichiometry="1"
constant="true"/>
                  <speciesReference species="M_phis__L" stoichiometry="1"
constant="true"/>
            </listOfProducts>
            <fbc:geneProductAssociation>
                  <fbc:geneProductRef fbc:geneProduct="SAUSA300_2270"/>
            </fbc:geneProductAssociation>
      </reaction>

      <reaction metaid="R_PNTK" id="R_PNTK" name="pantothenate kinase"
reversible="false" fast="false" fbc:lowerFluxBound="irr_lb"
fbc:upperFluxBound="irr_ub">
            <listOfReactants>
                  <speciesReference species="M_atp_c" stoichiometry="1"
constant="true"/>
                  <speciesReference species="M_pnto__R_c" stoichiometry="1"
constant="true"/>
            </listOfReactants>
            <listOfProducts>
                  <speciesReference species="M_adp_c" stoichiometry="1"
constant="true"/>
                  <speciesReference species="M_4ppan_c" stoichiometry="1"
constant="true"/>
            </listOfProducts>
            <fbc:geneProductAssociation>
                  <fbc:geneProductRef fbc:geneProduct="SAUSA300_2084"/>
            </fbc:geneProductAssociation>
```

```
      </reaction>

      <reaction metaid="R_POLYAO" id="R_POLYAO" name="polyamine oxidase"
reversible="false" fast="false" fbc:lowerFluxBound="irr_lb"
fbc:upperFluxBound="irr_ub">
            <listOfReactants>
                  <speciesReference species="M_h2o_c" stoichiometry="1"
constant="true"/>
                  <speciesReference species="M_o2_c" stoichiometry="1"
constant="true"/>
                  <speciesReference species="M_N1aspmd_c" stoichiometry="1"
constant="true"/>
            </listOfReactants>
            <listOfProducts>
                  <speciesReference species="M_h2o2_c" stoichiometry="1"
constant="true"/>
                  <speciesReference species="M_aprut_c" stoichiometry="1"
constant="true"/>
                  <speciesReference species="M_aproa_c" stoichiometry="1"
constant="true"/>
            </listOfProducts>
      </reaction>

      <reaction metaid="R_POX2" id="R_POX2" name="Pyruvate Oxidase (2)"
reversible="false" fast="false" fbc:lowerFluxBound="irr_lb"
fbc:upperFluxBound="irr_ub">
            <listOfReactants>
                  <speciesReference species="M_h_c" stoichiometry="1"
constant="true"/>
                  <speciesReference species="M_h2o_c" stoichiometry="1"
constant="true"/>
                  <speciesReference species="M_o2_c" stoichiometry="1.5"
constant="true"/>
                  <speciesReference species="M_pi_c" stoichiometry="1"
constant="true"/>
                  <speciesReference species="M_pyr_c" stoichiometry="1"
constant="true"/>
            </listOfReactants>
            <listOfProducts>
                  <speciesReference species="M_co2_c" stoichiometry="1"
constant="true"/>
                  <speciesReference species="M_actp_c" stoichiometry="1"
constant="true"/>
                  <speciesReference species="M_h2o2_c" stoichiometry="2"
constant="true"/>
            </listOfProducts>
            <fbc:geneProductAssociation>
                  <fbc:geneProductRef fbc:geneProduct="SAUSA300_2477"/>
            </fbc:geneProductAssociation>
      </reaction>

      <reaction metaid="R_POX3" id="R_POX3" name="Pyruvate oxidase 3"
reversible="false" fast="false" fbc:lowerFluxBound="irr_lb"
fbc:upperFluxBound="irr_ub">
            <listOfReactants>
                  <speciesReference species="M_h2o_c" stoichiometry="1"
constant="true"/>
```

```
                    <speciesReference species="M_mqn8_c" stoichiometry="1"
constant="true"/>
                    <speciesReference species="M_pyr_c" stoichiometry="1"
constant="true"/>
              </listOfReactants>
              <listOfProducts>
                    <speciesReference species="M_ac_c" stoichiometry="1"
constant="true"/>
                    <speciesReference species="M_mql8_c" stoichiometry="1"
constant="true"/>
                    <speciesReference species="M_co2_c" stoichiometry="1"
constant="true"/>
              </listOfProducts>
              <fbc:geneProductAssociation>
                    <fbc:geneProductRef fbc:geneProduct="SAUSA300_2477"/>
              </fbc:geneProductAssociation>
        </reaction>

        <reaction metaid="R_PPA" id="R_PPA" name="inorganic diphosphatase"
reversible="false" fast="false" fbc:lowerFluxBound="irr_lb"
fbc:upperFluxBound="irr_ub">
              <listOfReactants>
                    <speciesReference species="M_h2o_c" stoichiometry="1"
constant="true"/>
                    <speciesReference species="M_ppi_c" stoichiometry="1"
constant="true"/>
              </listOfReactants>
              <listOfProducts>
                    <speciesReference species="M_h_c" stoichiometry="1"
constant="true"/>
                    <speciesReference species="M_pi_c" stoichiometry="2"
constant="true"/>
              </listOfProducts>
              <fbc:geneProductAssociation>
                    <fbc:geneProductRef fbc:geneProduct="SAUSA300_1900"/>
              </fbc:geneProductAssociation>
        </reaction>

        <reaction metaid="R_PPAKr" id="R_PPAKr" name="Propionate kinase"
reversible="true" fast="false" fbc:lowerFluxBound="rev_lb"
fbc:upperFluxBound="rev_ub">
              <listOfReactants>
                    <speciesReference species="M_h_c" stoichiometry="1"
constant="true"/>
                    <speciesReference species="M_atp_c" stoichiometry="1"
constant="true"/>
                    <speciesReference species="M_ppa_c" stoichiometry="1"
constant="true"/>
              </listOfReactants>
              <listOfProducts>
                    <speciesReference species="M_adp_c" stoichiometry="1"
constant="true"/>
                    <speciesReference species="M_ppap_c" stoichiometry="1"
constant="true"/>
              </listOfProducts>
              <fbc:geneProductAssociation>
                    <fbc:geneProductRef fbc:geneProduct="SAUSA300_1657"/>
              </fbc:geneProductAssociation>
```

</reaction>

<reaction metaid="R_PPBNGS" id="R_PPBNGS" name="porphobilinogen synthase" reversible="false" fast="false" fbc:lowerFluxBound="irr_lb" fbc:upperFluxBound="irr_ub">
<listOfReactants>
<speciesReference species="M_5aop_c" stoichiometry="2" constant="true"/>
</listOfReactants>
<listOfProducts>
<speciesReference species="M_h_c" stoichiometry="1" constant="true"/>
<speciesReference species="M_h2o_c" stoichiometry="2" constant="true"/>
<speciesReference species="M_ppbng_c" stoichiometry="1" constant="true"/>
</listOfProducts>
<fbc:geneProductAssociation>
<fbc:geneProductRef fbc:geneProduct="SAUSA300_1615"/>
</fbc:geneProductAssociation>
</reaction>

<reaction metaid="R_PPCDC" id="R_PPCDC" name="phosphopantothenoylcysteine decarboxylase" reversible="false" fast="false" fbc:lowerFluxBound="irr_lb" fbc:upperFluxBound="irr_ub">
<listOfReactants>
<speciesReference species="M_h_c" stoichiometry="1" constant="true"/>
<speciesReference species="M_4ppcys_c" stoichiometry="1" constant="true"/>
</listOfReactants>
<listOfProducts>
<speciesReference species="M_co2_c" stoichiometry="1" constant="true"/>
<speciesReference species="M_pan4p_c" stoichiometry="1" constant="true"/>
</listOfProducts>
<fbc:geneProductAssociation>
<fbc:geneProductRef fbc:geneProduct="SAUSA300_1104"/>
</fbc:geneProductAssociation>
</reaction>

<reaction metaid="R_PPCK" id="R_PPCK" name="phosphoenolpyruvate carboxykinase" reversible="false" fast="false" fbc:lowerFluxBound="irr_lb" fbc:upperFluxBound="irr_ub">
<listOfReactants>
<speciesReference species="M_h_c" stoichiometry="1" constant="true"/>
<speciesReference species="M_atp_c" stoichiometry="1" constant="true"/>
<speciesReference species="M_oaa_c" stoichiometry="1" constant="true"/>
</listOfReactants>
<listOfProducts>
<speciesReference species="M_adp_c" stoichiometry="1" constant="true"/>
<speciesReference species="M_co2_c" stoichiometry="1" constant="true"/>

```
                  <speciesReference species="M_pep_c" stoichiometry="1"
constant="true"/>
            </listOfProducts>
            <fbc:geneProductAssociation>
                  <fbc:geneProductRef fbc:geneProduct="SAUSA300_1731"/>
            </fbc:geneProductAssociation>
      </reaction>

      <reaction metaid="R_PPCOAC" id="R_PPCOAC" name="Propionyl-CoA
carboxylase" reversible="false" fast="false" fbc:lowerFluxBound="irr_lb"
fbc:upperFluxBound="irr_ub">
            <listOfReactants>
                  <speciesReference species="M_atp_c" stoichiometry="1"
constant="true"/>
                  <speciesReference species="M_ppcoa_c" stoichiometry="1"
constant="true"/>
                  <speciesReference species="M_hco3_c" stoichiometry="1"
constant="true"/>
            </listOfReactants>
            <listOfProducts>
                  <speciesReference species="M_h_c" stoichiometry="1"
constant="true"/>
                  <speciesReference species="M_pi_c" stoichiometry="1"
constant="true"/>
                  <speciesReference species="M_adp_c" stoichiometry="1"
constant="true"/>
                  <speciesReference species="M_mmcoa__S_c" stoichiometry="1"
constant="true"/>
            </listOfProducts>
            <fbc:geneProductAssociation>
                  <fbc:or>
                        <fbc:and>
                              <fbc:geneProductRef
fbc:geneProduct="SAUSA300_1647"/>
                              <fbc:geneProductRef
fbc:geneProduct="SAUSA300_1646"/>
                        </fbc:and>
                        <fbc:geneProductRef fbc:geneProduct="SAUSA300_1563"/>
                  </fbc:or>
            </fbc:geneProductAssociation>
      </reaction>

      <reaction metaid="R_PPCOAL" id="R_PPCOAL" name="Propanoate CoA ligase
AMP-forming " reversible="false" fast="false" fbc:lowerFluxBound="irr_lb"
fbc:upperFluxBound="irr_ub">
            <listOfReactants>
                  <speciesReference species="M_ppi_c" stoichiometry="1"
constant="true"/>
                  <speciesReference species="M_ppadn_c" stoichiometry="1"
constant="true"/>
            </listOfReactants>
            <listOfProducts>
                  <speciesReference species="M_h_c" stoichiometry="1"
constant="true"/>
                  <speciesReference species="M_atp_c" stoichiometry="1"
constant="true"/>
                  <speciesReference species="M_ppa_c" stoichiometry="1"
constant="true"/>
```

```
            </listOfProducts>
            <fbc:geneProductAssociation>
                  <fbc:geneProductRef fbc:geneProduct="SAUSA300_1679"/>
            </fbc:geneProductAssociation>
      </reaction>

      <reaction metaid="R_PPItex" id="R_PPItex" name="Diphosphate
transporter, periplasm" reversible="false" fast="false"
fbc:lowerFluxBound="irr_lb" fbc:upperFluxBound="irr_ub">
            <listOfReactants>
                  <speciesReference species="M_h_c" stoichiometry="1"
constant="true"/>
                  <speciesReference species="M_ppi_c" stoichiometry="1"
constant="true"/>
            </listOfReactants>
            <listOfProducts>
                  <speciesReference species="M_h_e" stoichiometry="1"
constant="true"/>
                  <speciesReference species="M_ppi_e" stoichiometry="1"
constant="true"/>
            </listOfProducts>
            <fbc:geneProductAssociation>
                  <fbc:geneProductRef fbc:geneProduct="SAUSA300_0650"/>
            </fbc:geneProductAssociation>
      </reaction>

      <reaction metaid="R_PPM" id="R_PPM" name="phosphopentomutase"
reversible="true" fast="false" fbc:lowerFluxBound="rev_lb"
fbc:upperFluxBound="rev_ub">
            <listOfReactants>
                  <speciesReference species="M_r1p_c" stoichiometry="1"
constant="true"/>
            </listOfReactants>
            <listOfProducts>
                  <speciesReference species="M_r5p_c" stoichiometry="1"
constant="true"/>
            </listOfProducts>
            <fbc:geneProductAssociation>
                  <fbc:geneProductRef fbc:geneProduct="SAUSA300_0141"/>
            </fbc:geneProductAssociation>
      </reaction>

      <reaction metaid="R_PPM2" id="R_PPM2" name="phosphopentomutase 2
(deoxyribose)" reversible="true" fast="false" fbc:lowerFluxBound="rev_lb"
fbc:upperFluxBound="rev_ub">
            <listOfReactants>
                  <speciesReference species="M_2dr1p_c" stoichiometry="1"
constant="true"/>
            </listOfReactants>
            <listOfProducts>
                  <speciesReference species="M_2dr5p_c" stoichiometry="1"
constant="true"/>
            </listOfProducts>
            <fbc:geneProductAssociation>
                  <fbc:geneProductRef fbc:geneProduct="SAUSA300_0141"/>
            </fbc:geneProductAssociation>
      </reaction>
```

```
      <reaction metaid="R_PPNCL2" id="R_PPNCL2" name="phosphopantothenate-
cysteine ligase" reversible="false" fast="false" fbc:lowerFluxBound="irr_lb"
fbc:upperFluxBound="irr_ub">
            <listOfReactants>
                  <speciesReference species="M_ctp_c" stoichiometry="1"
constant="true"/>
                  <speciesReference species="M_cys__L_c" stoichiometry="1"
constant="true"/>
                  <speciesReference species="M_4ppan_c" stoichiometry="1"
constant="true"/>
            </listOfReactants>
            <listOfProducts>
                  <speciesReference species="M_ppi_c" stoichiometry="1"
constant="true"/>
                  <speciesReference species="M_cmp_c" stoichiometry="1"
constant="true"/>
                  <speciesReference species="M_4ppcys_c" stoichiometry="1"
constant="true"/>
            </listOfProducts>
            <fbc:geneProductAssociation>
                  <fbc:geneProductRef fbc:geneProduct="SAUSA300_1104"/>
            </fbc:geneProductAssociation>
      </reaction>

      <reaction metaid="R_PPND" id="R_PPND" name="prephenate dehydrogenase"
reversible="false" fast="false" fbc:lowerFluxBound="irr_lb"
fbc:upperFluxBound="irr_ub">
            <listOfReactants>
                  <speciesReference species="M_nad_c" stoichiometry="1"
constant="true"/>
                  <speciesReference species="M_pphn_c" stoichiometry="1"
constant="true"/>
            </listOfReactants>
            <listOfProducts>
                  <speciesReference species="M_co2_c" stoichiometry="1"
constant="true"/>
                  <speciesReference species="M_nadh_c" stoichiometry="1"
constant="true"/>
                  <speciesReference species="M_34hpp_c" stoichiometry="1"
constant="true"/>
            </listOfProducts>
            <fbc:geneProductAssociation>
                  <fbc:geneProductRef fbc:geneProduct="SAUSA300_1260"/>
            </fbc:geneProductAssociation>
      </reaction>

      <reaction metaid="R_PPNDH" id="R_PPNDH" name="prephenate dehydratase"
reversible="false" fast="false" fbc:lowerFluxBound="irr_lb"
fbc:upperFluxBound="irr_ub">
            <listOfReactants>
                  <speciesReference species="M_h_c" stoichiometry="1"
constant="true"/>
                  <speciesReference species="M_pphn_c" stoichiometry="1"
constant="true"/>
            </listOfReactants>
            <listOfProducts>
                  <speciesReference species="M_h2o_c" stoichiometry="1"
constant="true"/>
```

```
                  <speciesReference species="M_co2_c" stoichiometry="1"
constant="true"/>
                  <speciesReference species="M_phpyr_c" stoichiometry="1"
constant="true"/>
            </listOfProducts>
            <fbc:geneProductAssociation>
                  <fbc:geneProductRef fbc:geneProduct="SAUSA300_1896"/>
            </fbc:geneProductAssociation>
      </reaction>

      <reaction metaid="R_PPPGO" id="R_PPPGO" name="protoporphyrinogen
oxidase (aerobic)" reversible="false" fast="false"
fbc:lowerFluxBound="irr_lb" fbc:upperFluxBound="irr_ub">
            <listOfReactants>
                  <speciesReference species="M_o2_c" stoichiometry="3"
constant="true"/>
                  <speciesReference species="M_pppg9_c" stoichiometry="2"
constant="true"/>
            </listOfReactants>
            <listOfProducts>
                  <speciesReference species="M_h2o_c" stoichiometry="6"
constant="true"/>
                  <speciesReference species="M_ppp9_c" stoichiometry="2"
constant="true"/>
            </listOfProducts>
            <fbc:geneProductAssociation>
                  <fbc:geneProductRef fbc:geneProduct="SAUSA300_1781"/>
            </fbc:geneProductAssociation>
      </reaction>

      <reaction metaid="R_PPPGO2" id="R_PPPGO2" name="Protoporphyrinogen
oxidase (anaerobic, menaquinone)" reversible="true" fast="false"
fbc:lowerFluxBound="rev_lb" fbc:upperFluxBound="rev_ub">
            <listOfReactants>
                  <speciesReference species="M_mqn8_c" stoichiometry="3.0"
constant="true"/>
                  <speciesReference species="M_pppg9_c" stoichiometry="1"
constant="true"/>
            </listOfReactants>
            <listOfProducts>
                  <speciesReference species="M_mql8_c" stoichiometry="3.0"
constant="true"/>
                  <speciesReference species="M_ppp9_c" stoichiometry="1"
constant="true"/>
            </listOfProducts>
            <fbc:geneProductAssociation>
                  <fbc:geneProductRef fbc:geneProduct="SAUSA300_1781"/>
            </fbc:geneProductAssociation>
      </reaction>

      <reaction metaid="R_PPTT" id="R_PPTT" name="trans-
pentaprenyltranstransferase" reversible="false" fast="false"
fbc:lowerFluxBound="irr_lb" fbc:upperFluxBound="irr_ub">
            <listOfReactants>
                  <speciesReference species="M_ipdp_c" stoichiometry="1"
constant="true"/>
                  <speciesReference species="M_pendp_c" stoichiometry="1"
constant="true"/>
```

```
            </listOfReactants>
            <listOfProducts>
                  <speciesReference species="M_ppi_c" stoichiometry="1"
constant="true"/>
                  <speciesReference species="M_hexdp_c" stoichiometry="1"
constant="true"/>
            </listOfProducts>
            <fbc:geneProductAssociation>
                  <fbc:or>
                        <fbc:geneProductRef fbc:geneProduct="SAUSA300_1359"/>
                        <fbc:geneProductRef fbc:geneProduct="SAUSA300_1361"/>
                  </fbc:or>
            </fbc:geneProductAssociation>
      </reaction>

      <reaction metaid="R_PRAGS" id="R_PRAGS" name="phosphoribosylglycinamide
synthetase" reversible="false" fast="false" fbc:lowerFluxBound="irr_lb"
fbc:upperFluxBound="irr_ub">
            <listOfReactants>
                  <speciesReference species="M_atp_c" stoichiometry="1"
constant="true"/>
                  <speciesReference species="M_gly_c" stoichiometry="1"
constant="true"/>
                  <speciesReference species="M_pram_c" stoichiometry="1"
constant="true"/>
            </listOfReactants>
            <listOfProducts>
                  <speciesReference species="M_h_c" stoichiometry="1"
constant="true"/>
                  <speciesReference species="M_pi_c" stoichiometry="1"
constant="true"/>
                  <speciesReference species="M_adp_c" stoichiometry="1"
constant="true"/>
                  <speciesReference species="M_gar_c" stoichiometry="1"
constant="true"/>
            </listOfProducts>
            <fbc:geneProductAssociation>
                  <fbc:geneProductRef fbc:geneProduct="SAUSA300_0976"/>
            </fbc:geneProductAssociation>
      </reaction>

      <reaction metaid="R_PRAI" id="R_PRAI" name="phosphoribosylanthranilate
isomerase" reversible="true" fast="false" fbc:lowerFluxBound="rev_lb"
fbc:upperFluxBound="rev_ub">
            <listOfReactants>
                  <speciesReference species="M_pran_c" stoichiometry="1"
constant="true"/>
            </listOfReactants>
            <listOfProducts>
                  <speciesReference species="M_2cpr5p_c" stoichiometry="1"
constant="true"/>
            </listOfProducts>
            <fbc:geneProductAssociation>
                  <fbc:geneProductRef fbc:geneProduct="SAUSA300_1266"/>
            </fbc:geneProductAssociation>
      </reaction>
```

```
      <reaction metaid="R_PRAIS" id="R_PRAIS"
name="phosphoribosylaminoimidazole synthase" reversible="false" fast="false"
fbc:lowerFluxBound="irr_lb" fbc:upperFluxBound="irr_ub">
            <listOfReactants>
                  <speciesReference species="M_atp_c" stoichiometry="1"
constant="true"/>
                  <speciesReference species="M_fpram_c" stoichiometry="1"
constant="true"/>
            </listOfReactants>
            <listOfProducts>
                  <speciesReference species="M_h_c" stoichiometry="1"
constant="true"/>
                  <speciesReference species="M_pi_c" stoichiometry="1"
constant="true"/>
                  <speciesReference species="M_adp_c" stoichiometry="1"
constant="true"/>
                  <speciesReference species="M_air_c" stoichiometry="1"
constant="true"/>
            </listOfProducts>
            <fbc:geneProductAssociation>
                  <fbc:geneProductRef fbc:geneProduct="SAUSA300_0973"/>
            </fbc:geneProductAssociation>
      </reaction>

      <reaction metaid="R_PRAMPC" id="R_PRAMPC" name="phosphoribosyl-AMP
cyclohydrolase" reversible="false" fast="false" fbc:lowerFluxBound="irr_lb"
fbc:upperFluxBound="irr_ub">
            <listOfReactants>
                  <speciesReference species="M_h2o_c" stoichiometry="1"
constant="true"/>
                  <speciesReference species="M_prbamp_c" stoichiometry="1"
constant="true"/>
            </listOfReactants>
            <listOfProducts>
                  <speciesReference species="M_prfp_c" stoichiometry="1"
constant="true"/>
            </listOfProducts>
            <fbc:geneProductAssociation>
                  <fbc:geneProductRef fbc:geneProduct="SAUSA300_2605"/>
            </fbc:geneProductAssociation>
      </reaction>

      <reaction metaid="R_PRASCS" id="R_PRASCS"
name="phosphoribosylaminoimidazolesuccinocarboxamide synthase"
reversible="false" fast="false" fbc:lowerFluxBound="irr_lb"
fbc:upperFluxBound="irr_ub">
            <listOfReactants>
                  <speciesReference species="M_atp_c" stoichiometry="1"
constant="true"/>
                  <speciesReference species="M_asp__L_c" stoichiometry="1"
constant="true"/>
                  <speciesReference species="M_5aizc_c" stoichiometry="1"
constant="true"/>
            </listOfReactants>
            <listOfProducts>
                  <speciesReference species="M_h_c" stoichiometry="1"
constant="true"/>
```

```
                  <speciesReference species="M_pi_c" stoichiometry="1"
constant="true"/>
                  <speciesReference species="M_adp_c" stoichiometry="1"
constant="true"/>
                  <speciesReference species="M_25aics_c" stoichiometry="1"
constant="true"/>
            </listOfProducts>
            <fbc:geneProductAssociation>
                  <fbc:or>
                        <fbc:geneProductRef fbc:geneProduct="SAUSA300_0967"/>
                        <fbc:geneProductRef fbc:geneProduct="SAUSA300_0966"/>
                        <fbc:geneProductRef fbc:geneProduct="SAUSA300_0968"/>
                  </fbc:or>
            </fbc:geneProductAssociation>
      </reaction>

      <reaction metaid="R_PRATPP" id="R_PRATPP" name="phosphoribosyl-ATP
pyrophosphatase" reversible="false" fast="false" fbc:lowerFluxBound="irr_lb"
fbc:upperFluxBound="irr_ub">
            <listOfReactants>
                  <speciesReference species="M_h2o_c" stoichiometry="1"
constant="true"/>
                  <speciesReference species="M_prbatp_c" stoichiometry="1"
constant="true"/>
            </listOfReactants>
            <listOfProducts>
                  <speciesReference species="M_ppi_c" stoichiometry="1"
constant="true"/>
                  <speciesReference species="M_prbamp_c" stoichiometry="1"
constant="true"/>
            </listOfProducts>
            <fbc:geneProductAssociation>
                  <fbc:geneProductRef fbc:geneProduct="SAUSA300_2605"/>
            </fbc:geneProductAssociation>
      </reaction>

      <reaction metaid="R_PRFGS" id="R_PRFGS"
name="phosphoribosylformylglycinamidine synthase" reversible="false"
fast="false" fbc:lowerFluxBound="irr_lb" fbc:upperFluxBound="irr_ub">
            <listOfReactants>
                  <speciesReference species="M_h2o_c" stoichiometry="1"
constant="true"/>
                  <speciesReference species="M_gln__L_c" stoichiometry="1"
constant="true"/>
                  <speciesReference species="M_atp_c" stoichiometry="1"
constant="true"/>
                  <speciesReference species="M_fgam_c" stoichiometry="1"
constant="true"/>
            </listOfReactants>
            <listOfProducts>
                  <speciesReference species="M_h_c" stoichiometry="1"
constant="true"/>
                  <speciesReference species="M_glu__L_c" stoichiometry="1"
constant="true"/>
                  <speciesReference species="M_pi_c" stoichiometry="1"
constant="true"/>
                  <speciesReference species="M_adp_c" stoichiometry="1"
constant="true"/>
```

```
                  <speciesReference species="M_fpram_c" stoichiometry="1"
constant="true"/>
            </listOfProducts>
            <fbc:geneProductAssociation>
                  <fbc:and>
                        <fbc:geneProductRef fbc:geneProduct="SAUSA300_0969"/>
                        <fbc:geneProductRef fbc:geneProduct="SAUSA300_0970"/>
                        <fbc:geneProductRef fbc:geneProduct="SAUSA300_0971"/>
                  </fbc:and>
            </fbc:geneProductAssociation>
      </reaction>

      <reaction metaid="R_PRMICIi" id="R_PRMICIi" name="1-(5-phosphoribosyl)-
5-[(5-phosphoribosylamino)methylideneamino)imidazole-4-carboxamide isomerase
(irreversible)" reversible="true" fast="false" fbc:lowerFluxBound="rev_lb"
fbc:upperFluxBound="rev_ub">
            <listOfReactants>
                  <speciesReference species="M_prfp_c" stoichiometry="1"
constant="true"/>
            </listOfReactants>
            <listOfProducts>
                  <speciesReference species="M_prlp_c" stoichiometry="1"
constant="true"/>
            </listOfProducts>
            <fbc:geneProductAssociation>
                  <fbc:geneProductRef fbc:geneProduct="SAUSA300_2607"/>
            </fbc:geneProductAssociation>
      </reaction>

      <reaction metaid="R_PRO1x" id="R_PRO1x" name="proline oxidase (L-
proline, NAD)" reversible="false" fast="false" fbc:lowerFluxBound="irr_lb"
fbc:upperFluxBound="irr_ub">
            <listOfReactants>
                  <speciesReference species="M_pro__L_c" stoichiometry="1"
constant="true"/>
                  <speciesReference species="M_nad_c" stoichiometry="1"
constant="true"/>
            </listOfReactants>
            <listOfProducts>
                  <speciesReference species="M_h_c" stoichiometry="1"
constant="true"/>
                  <speciesReference species="M_1pyr5c_c" stoichiometry="1"
constant="true"/>
                  <speciesReference species="M_nadh_c" stoichiometry="1"
constant="true"/>
            </listOfProducts>
            <fbc:geneProductAssociation>
                  <fbc:geneProductRef fbc:geneProduct="SAUSA300_1452"/>
            </fbc:geneProductAssociation>
      </reaction>

      <reaction metaid="R_PROabc" id="R_PROabc" name="L-proline transport via
ABC system" reversible="false" fast="false" fbc:lowerFluxBound="irr_lb"
fbc:upperFluxBound="irr_ub">
            <listOfReactants>
                  <speciesReference species="M_h2o_c" stoichiometry="1"
constant="true"/>
```

```
                  <speciesReference species="M_atp_c" stoichiometry="1"
constant="true"/>
                  <speciesReference species="M_pro__L_e" stoichiometry="1"
constant="true"/>
            </listOfReactants>
            <listOfProducts>
                  <speciesReference species="M_h_c" stoichiometry="1"
constant="true"/>
                  <speciesReference species="M_pi_c" stoichiometry="1"
constant="true"/>
                  <speciesReference species="M_adp_c" stoichiometry="1"
constant="true"/>
                  <speciesReference species="M_pro__L_c" stoichiometry="1"
constant="true"/>
            </listOfProducts>
            <fbc:geneProductAssociation>
                  <fbc:and>
                        <fbc:geneProductRef fbc:geneProduct="SAUSA300_0707"/>
                        <fbc:geneProductRef fbc:geneProduct="SAUSA300_0706"/>
                  </fbc:and>
            </fbc:geneProductAssociation>
      </reaction>

      <reaction metaid="R_PROD2" id="R_PROD2" name="Proline dehydrogenase"
reversible="false" fast="false" fbc:lowerFluxBound="irr_lb"
fbc:upperFluxBound="irr_ub">
            <listOfReactants>
                  <speciesReference species="M_pro__L_c" stoichiometry="1"
constant="true"/>
                  <speciesReference species="M_fad_c" stoichiometry="1"
constant="true"/>
            </listOfReactants>
            <listOfProducts>
                  <speciesReference species="M_1pyr5c_c" stoichiometry="1"
constant="true"/>
                  <speciesReference species="M_fadh2_c" stoichiometry="1"
constant="true"/>
            </listOfProducts>
            <fbc:geneProductAssociation>
                  <fbc:geneProductRef fbc:geneProduct="SAUSA300_1711"/>
            </fbc:geneProductAssociation>
      </reaction>

      <reaction metaid="R_PROt" id="R_PROt" name="EX pro L e "
reversible="false" fast="false" fbc:lowerFluxBound="irr_lb"
fbc:upperFluxBound="irr_ub">
            <listOfReactants>
                  <speciesReference species="M_pro__L_e" stoichiometry="1"
constant="true"/>
            </listOfReactants>
            <listOfProducts>
                  <speciesReference species="M_pro__L_c" stoichiometry="1"
constant="true"/>
            </listOfProducts>
            <fbc:geneProductAssociation>
                  <fbc:geneProductRef fbc:geneProduct="SAUSA300_0558"/>
            </fbc:geneProductAssociation>
      </reaction>
```

```
      <reaction metaid="R_PROt2r" id="R_PROt2r" name="L-proline reversible
transport via proton symport" reversible="false" fast="false"
fbc:lowerFluxBound="irr_lb" fbc:upperFluxBound="irr_ub">
            <listOfReactants>
                  <speciesReference species="M_h_e" stoichiometry="1"
constant="true"/>
                  <speciesReference species="M_pro__L_e" stoichiometry="1"
constant="true"/>
            </listOfReactants>
            <listOfProducts>
                  <speciesReference species="M_h_c" stoichiometry="1"
constant="true"/>
                  <speciesReference species="M_pro__L_c" stoichiometry="1"
constant="true"/>
            </listOfProducts>
            <fbc:geneProductAssociation>
                  <fbc:geneProductRef fbc:geneProduct="SAUSA300_0558"/>
            </fbc:geneProductAssociation>
      </reaction>

      <reaction metaid="R_PROt4" id="R_PROt4" name="Na+/Proline__L symporter"
reversible="false" fast="false" fbc:lowerFluxBound="irr_lb"
fbc:upperFluxBound="irr_ub">
            <listOfReactants>
                  <speciesReference species="M_na1_e" stoichiometry="1"
constant="true"/>
                  <speciesReference species="M_pro__L_e" stoichiometry="1"
constant="true"/>
            </listOfReactants>
            <listOfProducts>
                  <speciesReference species="M_pro__L_c" stoichiometry="1"
constant="true"/>
                  <speciesReference species="M_na1_c" stoichiometry="1"
constant="true"/>
            </listOfProducts>
            <fbc:geneProductAssociation>
                  <fbc:geneProductRef fbc:geneProduct="SAUSA300_1883"/>
            </fbc:geneProductAssociation>
      </reaction>

      <reaction metaid="R_PRPPS" id="R_PRPPS"
name="phosphoribosylpyrophosphate synthetase" reversible="true" fast="false"
fbc:lowerFluxBound="rev_lb" fbc:upperFluxBound="rev_ub">
            <listOfReactants>
                  <speciesReference species="M_atp_c" stoichiometry="1"
constant="true"/>
                  <speciesReference species="M_r5p_c" stoichiometry="1"
constant="true"/>
            </listOfReactants>
            <listOfProducts>
                  <speciesReference species="M_amp_c" stoichiometry="1"
constant="true"/>
                  <speciesReference species="M_prpp_c" stoichiometry="1"
constant="true"/>
            </listOfProducts>
            <fbc:geneProductAssociation>
                  <fbc:geneProductRef fbc:geneProduct="SAUSA300_0478"/>
```

```
            </fbc:geneProductAssociation>
      </reaction>

      <reaction metaid="R_PSCVT" id="R_PSCVT" name="3-phosphoshikimate 1-
carboxyvinyltransferase" reversible="true" fast="false"
fbc:lowerFluxBound="rev_lb" fbc:upperFluxBound="rev_ub">
            <listOfReactants>
                  <speciesReference species="M_pep_c" stoichiometry="1"
constant="true"/>
                  <speciesReference species="M_skm5p_c" stoichiometry="1"
constant="true"/>
            </listOfReactants>
            <listOfProducts>
                  <speciesReference species="M_h_c" stoichiometry="1"
constant="true"/>
                  <speciesReference species="M_pi_c" stoichiometry="1"
constant="true"/>
                  <speciesReference species="M_3psme_c" stoichiometry="1"
constant="true"/>
            </listOfProducts>
            <fbc:geneProductAssociation>
                  <fbc:geneProductRef fbc:geneProduct="SAUSA300_1355"/>
            </fbc:geneProductAssociation>
      </reaction>

      <reaction metaid="R_PSD_SA" id="R_PSD_SA" name="Phosphatidylserine
decarboxylase (Saureus)" reversible="false" fast="false"
fbc:lowerFluxBound="irr_lb" fbc:upperFluxBound="irr_ub">
            <listOfReactants>
                  <speciesReference species="M_h_c" stoichiometry="1"
constant="true"/>
                  <speciesReference species="M_ps_SA_c" stoichiometry="0.02"
constant="true"/>
            </listOfReactants>
            <listOfProducts>
                  <speciesReference species="M_co2_c" stoichiometry="1"
constant="true"/>
                  <speciesReference species="M_pe_SA_c" stoichiometry="0.02"
constant="true"/>
            </listOfProducts>
            <fbc:geneProductAssociation>
                  <fbc:geneProductRef fbc:geneProduct="psd"/>
            </fbc:geneProductAssociation>
      </reaction>

      <reaction metaid="R_PSERT" id="R_PSERT" name="phosphoserine
transaminase" reversible="false" fast="false" fbc:lowerFluxBound="irr_lb"
fbc:upperFluxBound="irr_ub">
            <listOfReactants>
                  <speciesReference species="M_akg_c" stoichiometry="1"
constant="true"/>
                  <speciesReference species="M_pser__L_c" stoichiometry="1"
constant="true"/>
            </listOfReactants>
            <listOfProducts>
                  <speciesReference species="M_glu__L_c" stoichiometry="1"
constant="true"/>
```

```
                  <speciesReference species="M_3php_c" stoichiometry="1"
constant="true"/>
            </listOfProducts>
            <fbc:geneProductAssociation>
                  <fbc:geneProductRef fbc:geneProduct="SAUSA300_1669"/>
            </fbc:geneProductAssociation>
      </reaction>

      <reaction metaid="R_PSP_L" id="R_PSP_L" name="phosphoserine phosphatase
(L-serine)" reversible="false" fast="false" fbc:lowerFluxBound="irr_lb"
fbc:upperFluxBound="irr_ub">
            <listOfReactants>
                  <speciesReference species="M_h2o_c" stoichiometry="1"
constant="true"/>
                  <speciesReference species="M_pser__L_c" stoichiometry="1"
constant="true"/>
            </listOfReactants>
            <listOfProducts>
                  <speciesReference species="M_h_c" stoichiometry="1"
constant="true"/>
                  <speciesReference species="M_pi_c" stoichiometry="1"
constant="true"/>
                  <speciesReference species="M_ser__L_c" stoichiometry="1"
constant="true"/>
            </listOfProducts>
            <fbc:geneProductAssociation>
                  <fbc:geneProductRef fbc:geneProduct="SAUSA300_1671"/>
            </fbc:geneProductAssociation>
      </reaction>

      <reaction metaid="R_PSQSYN" id="R_PSQSYN" name="presqualene synthase"
reversible="false" fast="false" fbc:lowerFluxBound="irr_lb"
fbc:upperFluxBound="irr_ub">
            <listOfReactants>
                  <speciesReference species="M_frdp_c" stoichiometry="2"
constant="true"/>
            </listOfReactants>
            <listOfProducts>
                  <speciesReference species="M_ppi_c" stoichiometry="1"
constant="true"/>
                  <speciesReference species="M_psdp_c" stoichiometry="1"
constant="true"/>
            </listOfProducts>
            <fbc:geneProductAssociation>
                  <fbc:geneProductRef fbc:geneProduct="SAUSA300_2499"/>
            </fbc:geneProductAssociation>
      </reaction>

      <reaction metaid="R_PSSA_SA" id="R_PSSA_SA" name="Phosphatidylserine
syntase (Saureus)" reversible="true" fast="false" fbc:lowerFluxBound="rev_lb"
fbc:upperFluxBound="rev_ub">
            <listOfReactants>
                  <speciesReference species="M_ser__L_c" stoichiometry="1"
constant="true"/>
                  <speciesReference species="M_cdpdag_SA_c"
stoichiometry="0.02" constant="true"/>
            </listOfReactants>
            <listOfProducts>
```

```
                  <speciesReference species="M_h_c" stoichiometry="1"
constant="true"/>
                  <speciesReference species="M_cmp_c" stoichiometry="1"
constant="true"/>
                  <speciesReference species="M_ps_SA_c" stoichiometry="0.02"
constant="true"/>
            </listOfProducts>
            <fbc:geneProductAssociation>
                  <fbc:geneProductRef fbc:geneProduct="pss"/>
            </fbc:geneProductAssociation>
      </reaction>

      <reaction metaid="R_PTA2" id="R_PTA2" name="Phosphate
acetyltransferase" reversible="false" fast="false"
fbc:lowerFluxBound="irr_lb" fbc:upperFluxBound="irr_ub">
            <listOfReactants>
                  <speciesReference species="M_h_c" stoichiometry="1"
constant="true"/>
                  <speciesReference species="M_pi_c" stoichiometry="1"
constant="true"/>
                  <speciesReference species="M_ppcoa_c" stoichiometry="1"
constant="true"/>
            </listOfReactants>
            <listOfProducts>
                  <speciesReference species="M_ppap_c" stoichiometry="1"
constant="true"/>
                  <speciesReference species="M_coa_c" stoichiometry="1"
constant="true"/>
            </listOfProducts>
            <fbc:geneProductAssociation>
                  <fbc:geneProductRef fbc:geneProduct="SAUSA300_0570"/>
            </fbc:geneProductAssociation>
      </reaction>

      <reaction metaid="R_PTAr" id="R_PTAr" name="phosphotransacetylase"
reversible="false" fast="false" fbc:lowerFluxBound="irr_lb"
fbc:upperFluxBound="irr_ub">
            <listOfReactants>
                  <speciesReference species="M_h_c" stoichiometry="1"
constant="true"/>
                  <speciesReference species="M_pi_c" stoichiometry="1"
constant="true"/>
                  <speciesReference species="M_accoa_c" stoichiometry="1"
constant="true"/>
            </listOfReactants>
            <listOfProducts>
                  <speciesReference species="M_coa_c" stoichiometry="1"
constant="true"/>
                  <speciesReference species="M_actp_c" stoichiometry="1"
constant="true"/>
            </listOfProducts>
            <fbc:geneProductAssociation>
                  <fbc:geneProductRef fbc:geneProduct="SAUSA300_0570"/>
            </fbc:geneProductAssociation>
      </reaction>
```

```
      <reaction metaid="R_PTHPS" id="R_PTHPS" name="6-
pyruvoyltetrahydropterin synthase" reversible="true" fast="false"
fbc:lowerFluxBound="rev_lb" fbc:upperFluxBound="rev_ub">
            <listOfReactants>
                  <speciesReference species="M_ahdt_c" stoichiometry="1"
constant="true"/>
            </listOfReactants>
            <listOfProducts>
                  <speciesReference species="M_h_c" stoichiometry="1"
constant="true"/>
                  <speciesReference species="M_pppi_c" stoichiometry="1"
constant="true"/>
                  <speciesReference species="M_6pthp_c" stoichiometry="1"
constant="true"/>
            </listOfProducts>
            <fbc:geneProductAssociation>
                  <fbc:geneProductRef fbc:geneProduct="SAUSA300_0696"/>
            </fbc:geneProductAssociation>
      </reaction>

      <reaction metaid="R_PTPATi" id="R_PTPATi" name="pantetheine-phosphate
adenylyltransferase" reversible="false" fast="false"
fbc:lowerFluxBound="irr_lb" fbc:upperFluxBound="irr_ub">
            <listOfReactants>
                  <speciesReference species="M_h_c" stoichiometry="1"
constant="true"/>
                  <speciesReference species="M_atp_c" stoichiometry="1"
constant="true"/>
                  <speciesReference species="M_pan4p_c" stoichiometry="1"
constant="true"/>
            </listOfReactants>
            <listOfProducts>
                  <speciesReference species="M_ppi_c" stoichiometry="1"
constant="true"/>
                  <speciesReference species="M_dpcoa_c" stoichiometry="1"
constant="true"/>
            </listOfProducts>
            <fbc:geneProductAssociation>
                  <fbc:geneProductRef fbc:geneProduct="SAUSA300_1024"/>
            </fbc:geneProductAssociation>
      </reaction>

      <reaction metaid="R_PTRCabc" id="R_PTRCabc" name="putrescine transport
via ABC system" reversible="false" fast="false" fbc:lowerFluxBound="irr_lb"
fbc:upperFluxBound="irr_ub">
            <listOfReactants>
                  <speciesReference species="M_h2o_c" stoichiometry="1"
constant="true"/>
                  <speciesReference species="M_atp_c" stoichiometry="1"
constant="true"/>
                  <speciesReference species="M_ptrc_e" stoichiometry="1"
constant="true"/>
            </listOfReactants>
            <listOfProducts>
                  <speciesReference species="M_h_c" stoichiometry="1"
constant="true"/>
                  <speciesReference species="M_pi_c" stoichiometry="1"
constant="true"/>
```

```
                  <speciesReference species="M_adp_c" stoichiometry="1"
constant="true"/>
                  <speciesReference species="M_ptrc_c" stoichiometry="1"
constant="true"/>
            </listOfProducts>
            <fbc:geneProductAssociation>
                  <fbc:and>
                        <fbc:geneProductRef fbc:geneProduct="SAUSA300_0999"/>
                        <fbc:geneProductRef fbc:geneProduct="SAUSA300_1001"/>
                        <fbc:geneProductRef fbc:geneProduct="SAUSA300_1000"/>
                        <fbc:geneProductRef fbc:geneProduct="SAUSA300_1002"/>
                  </fbc:and>
            </fbc:geneProductAssociation>
      </reaction>

      <reaction metaid="R_PUNP1" id="R_PUNP1" name="purine-nucleoside
phosphorylase (Adenosine)" reversible="true" fast="false"
fbc:lowerFluxBound="rev_lb" fbc:upperFluxBound="rev_ub">
            <listOfReactants>
                  <speciesReference species="M_h_c" stoichiometry="1"
constant="true"/>
                  <speciesReference species="M_pi_c" stoichiometry="1"
constant="true"/>
                  <speciesReference species="M_adn_c" stoichiometry="1"
constant="true"/>
            </listOfReactants>
            <listOfProducts>
                  <speciesReference species="M_r1p_c" stoichiometry="1"
constant="true"/>
                  <speciesReference species="M_ade_c" stoichiometry="1"
constant="true"/>
            </listOfProducts>
            <fbc:geneProductAssociation>
                  <fbc:or>
                        <fbc:geneProductRef fbc:geneProduct="SAUSA300_0138"/>
                        <fbc:geneProductRef fbc:geneProduct="SAUSA300_2091"/>
                  </fbc:or>
            </fbc:geneProductAssociation>
      </reaction>

      <reaction metaid="R_PUNP2" id="R_PUNP2" name="purine-nucleoside
phosphorylase (Deoxyadenosine)" reversible="true" fast="false"
fbc:lowerFluxBound="rev_lb" fbc:upperFluxBound="rev_ub">
            <listOfReactants>
                  <speciesReference species="M_h_c" stoichiometry="1"
constant="true"/>
                  <speciesReference species="M_pi_c" stoichiometry="1"
constant="true"/>
                  <speciesReference species="M_dad__2_c" stoichiometry="1"
constant="true"/>
            </listOfReactants>
            <listOfProducts>
                  <speciesReference species="M_ade_c" stoichiometry="1"
constant="true"/>
                  <speciesReference species="M_2dr1p_c" stoichiometry="1"
constant="true"/>
            </listOfProducts>
            <fbc:geneProductAssociation>
```

```
                    <fbc:or>
                          <fbc:geneProductRef fbc:geneProduct="SAUSA300_0138"/>
                          <fbc:geneProductRef fbc:geneProduct="SAUSA300_2091"/>
                    </fbc:or>
              </fbc:geneProductAssociation>
        </reaction>

        <reaction metaid="R_PUNP3" id="R_PUNP3" name="purine-nucleoside
phosphorylase (Guanosine)" reversible="false" fast="false"
fbc:lowerFluxBound="irr_lb" fbc:upperFluxBound="irr_ub">
              <listOfReactants>
                    <speciesReference species="M_h_c" stoichiometry="1"
constant="true"/>
                    <speciesReference species="M_pi_c" stoichiometry="1"
constant="true"/>
                    <speciesReference species="M_gsn_c" stoichiometry="1"
constant="true"/>
              </listOfReactants>
              <listOfProducts>
                    <speciesReference species="M_r1p_c" stoichiometry="1"
constant="true"/>
                    <speciesReference species="M_gua_c" stoichiometry="1"
constant="true"/>
              </listOfProducts>
              <fbc:geneProductAssociation>
                    <fbc:or>
                          <fbc:geneProductRef fbc:geneProduct="SAUSA300_0138"/>
                          <fbc:geneProductRef fbc:geneProduct="SAUSA300_2091"/>
                    </fbc:or>
              </fbc:geneProductAssociation>
        </reaction>

        <reaction metaid="R_PUNP4" id="R_PUNP4" name="purine-nucleoside
phosphorylase (Deoxyguanosine)" reversible="true" fast="false"
fbc:lowerFluxBound="rev_lb" fbc:upperFluxBound="rev_ub">
              <listOfReactants>
                    <speciesReference species="M_h_c" stoichiometry="1"
constant="true"/>
                    <speciesReference species="M_pi_c" stoichiometry="1"
constant="true"/>
                    <speciesReference species="M_dgsn_c" stoichiometry="1"
constant="true"/>
              </listOfReactants>
              <listOfProducts>
                    <speciesReference species="M_2dr1p_c" stoichiometry="1"
constant="true"/>
                    <speciesReference species="M_gua_c" stoichiometry="1"
constant="true"/>
              </listOfProducts>
              <fbc:geneProductAssociation>
                    <fbc:or>
                          <fbc:geneProductRef fbc:geneProduct="SAUSA300_0138"/>
                          <fbc:geneProductRef fbc:geneProduct="SAUSA300_2091"/>
                    </fbc:or>
              </fbc:geneProductAssociation>
        </reaction>
```

<reaction metaid="R_PUNP5" id="R_PUNP5" name="purine-nucleoside phosphorylase (Inosine)" reversible="true" fast="false" fbc:lowerFluxBound="rev_lb" fbc:upperFluxBound="rev_ub">
<listOfReactants>
<speciesReference species="M_h_c" stoichiometry="1" constant="true"/>
<speciesReference species="M_pi_c" stoichiometry="1" constant="true"/>
<speciesReference species="M_ins_c" stoichiometry="1" constant="true"/>
</listOfReactants>
<listOfProducts>
<speciesReference species="M_r1p_c" stoichiometry="1" constant="true"/>
<speciesReference species="M_hxan_c" stoichiometry="1" constant="true"/>
</listOfProducts>
<fbc:geneProductAssociation>
<fbc:or>
<fbc:geneProductRef fbc:geneProduct="SAUSA300_0138"/>
<fbc:geneProductRef fbc:geneProduct="SAUSA300_2091"/>
</fbc:or>
</fbc:geneProductAssociation>
</reaction>

<reaction metaid="R_PUNP6" id="R_PUNP6" name="purine-nucleoside phosphorylase (Deoxyinosine)" reversible="true" fast="false" fbc:lowerFluxBound="rev_lb" fbc:upperFluxBound="rev_ub">
<listOfReactants>
<speciesReference species="M_h_c" stoichiometry="1" constant="true"/>
<speciesReference species="M_pi_c" stoichiometry="1" constant="true"/>
<speciesReference species="M_din_c" stoichiometry="1" constant="true"/>
</listOfReactants>
<listOfProducts>
<speciesReference species="M_2dr1p_c" stoichiometry="1" constant="true"/>
<speciesReference species="M_hxan_c" stoichiometry="1" constant="true"/>
</listOfProducts>
<fbc:geneProductAssociation>
<fbc:or>
<fbc:geneProductRef fbc:geneProduct="SAUSA300_0138"/>
<fbc:geneProductRef fbc:geneProduct="SAUSA300_2091"/>
</fbc:or>
</fbc:geneProductAssociation>
</reaction>

<reaction metaid="R_PUNP7" id="R_PUNP7" name="purine-nucleoside phosphorylase (Xanthosine)" reversible="true" fast="false" fbc:lowerFluxBound="rev_lb" fbc:upperFluxBound="rev_ub">
<listOfReactants>
<speciesReference species="M_h_c" stoichiometry="1" constant="true"/>
<speciesReference species="M_pi_c" stoichiometry="1" constant="true"/>

```
                  <speciesReference species="M_xtsn_c" stoichiometry="1"
constant="true"/>
            </listOfReactants>
            <listOfProducts>
                  <speciesReference species="M_xan_c" stoichiometry="1"
constant="true"/>
                  <speciesReference species="M_r1p_c" stoichiometry="1"
constant="true"/>
            </listOfProducts>
            <fbc:geneProductAssociation>
                  <fbc:or>
                        <fbc:geneProductRef fbc:geneProduct="SAUSA300_0138"/>
                        <fbc:geneProductRef fbc:geneProduct="SAUSA300_2091"/>
                  </fbc:or>
            </fbc:geneProductAssociation>
      </reaction>

      <reaction metaid="R_PUTA3" id="R_PUTA3" name="puta3" reversible="true"
fast="false" fbc:lowerFluxBound="rev_lb" fbc:upperFluxBound="rev_ub">
            <listOfReactants>
                  <speciesReference species="M_h2o_c" stoichiometry="1"
constant="true"/>
                  <speciesReference species="M_nad_c" stoichiometry="1"
constant="true"/>
                  <speciesReference species="M_glu5sa_c" stoichiometry="1"
constant="true"/>
            </listOfReactants>
            <listOfProducts>
                  <speciesReference species="M_h_c" stoichiometry="2"
constant="true"/>
                  <speciesReference species="M_glu__L_c" stoichiometry="1"
constant="true"/>
                  <speciesReference species="M_nadh_c" stoichiometry="1"
constant="true"/>
            </listOfProducts>
            <fbc:geneProductAssociation>
                  <fbc:geneProductRef fbc:geneProduct="SAUSA300_2491"/>
            </fbc:geneProductAssociation>
      </reaction>

      <reaction metaid="R_PYDXK" id="R_PYDXK" name="pyridoxal kinase"
reversible="false" fast="false" fbc:lowerFluxBound="irr_lb"
fbc:upperFluxBound="irr_ub">
            <listOfReactants>
                  <speciesReference species="M_atp_c" stoichiometry="1"
constant="true"/>
                  <speciesReference species="M_pydx_c" stoichiometry="1"
constant="true"/>
            </listOfReactants>
            <listOfProducts>
                  <speciesReference species="M_adp_c" stoichiometry="1"
constant="true"/>
                  <speciesReference species="M_pydx5p_c" stoichiometry="1"
constant="true"/>
            </listOfProducts>
            <fbc:geneProductAssociation>
                  <fbc:geneProductRef fbc:geneProduct="SAUSA300_0562"/>
            </fbc:geneProductAssociation>
```

```
      </reaction>

      <reaction metaid="R_PYK" id="R_PYK" name="pyruvate kinase"
reversible="false" fast="false" fbc:lowerFluxBound="irr_lb"
fbc:upperFluxBound="irr_ub">
            <listOfReactants>
                  <speciesReference species="M_adp_c" stoichiometry="1"
constant="true"/>
                  <speciesReference species="M_pep_c" stoichiometry="1"
constant="true"/>
            </listOfReactants>
            <listOfProducts>
                  <speciesReference species="M_atp_c" stoichiometry="1"
constant="true"/>
                  <speciesReference species="M_pyr_c" stoichiometry="1"
constant="true"/>
            </listOfProducts>
            <fbc:geneProductAssociation>
                  <fbc:geneProductRef fbc:geneProduct="SAUSA300_1644"/>
            </fbc:geneProductAssociation>
      </reaction>

      <reaction metaid="R_PYK3" id="R_PYK3" name="Pyruvate kinase(3)"
reversible="true" fast="false" fbc:lowerFluxBound="rev_lb"
fbc:upperFluxBound="rev_ub">
            <listOfReactants>
                  <speciesReference species="M_h_c" stoichiometry="1"
constant="true"/>
                  <speciesReference species="M_gdp_c" stoichiometry="1"
constant="true"/>
                  <speciesReference species="M_pep_c" stoichiometry="1"
constant="true"/>
            </listOfReactants>
            <listOfProducts>
                  <speciesReference species="M_gtp_c" stoichiometry="1"
constant="true"/>
                  <speciesReference species="M_pyr_c" stoichiometry="1"
constant="true"/>
            </listOfProducts>
            <fbc:geneProductAssociation>
                  <fbc:geneProductRef fbc:geneProduct="SAUSA300_1644"/>
            </fbc:geneProductAssociation>
      </reaction>

      <reaction metaid="R_PYNP1" id="R_PYNP1" name="pyrimidine-nucleoside
phosphorylase (cytosine)" reversible="true" fast="false"
fbc:lowerFluxBound="rev_lb" fbc:upperFluxBound="rev_ub">
            <listOfReactants>
                  <speciesReference species="M_h_c" stoichiometry="1"
constant="true"/>
                  <speciesReference species="M_pi_c" stoichiometry="1"
constant="true"/>
                  <speciesReference species="M_cytd_c" stoichiometry="1"
constant="true"/>
            </listOfReactants>
            <listOfProducts>
                  <speciesReference species="M_r1p_c" stoichiometry="1"
constant="true"/>
```

```
                  <speciesReference species="M_csn_c" stoichiometry="1"
constant="true"/>
            </listOfProducts>
            <fbc:geneProductAssociation>
                  <fbc:geneProductRef fbc:geneProduct="SAUSA300_2089"/>
            </fbc:geneProductAssociation>
      </reaction>

      <reaction metaid="R_PYNP2r" id="R_PYNP2r" name="pyrimidine-nucleoside
phosphorylase (uracil)" reversible="false" fast="false"
fbc:lowerFluxBound="irr_lb" fbc:upperFluxBound="irr_ub">
            <listOfReactants>
                  <speciesReference species="M_h_c" stoichiometry="1"
constant="true"/>
                  <speciesReference species="M_pi_c" stoichiometry="1"
constant="true"/>
                  <speciesReference species="M_uri_c" stoichiometry="1"
constant="true"/>
            </listOfReactants>
            <listOfProducts>
                  <speciesReference species="M_r1p_c" stoichiometry="1"
constant="true"/>
                  <speciesReference species="M_ura_c" stoichiometry="1"
constant="true"/>
            </listOfProducts>
            <fbc:geneProductAssociation>
                  <fbc:geneProductRef fbc:geneProduct="SAUSA300_2089"/>
            </fbc:geneProductAssociation>
      </reaction>

      <reaction metaid="R_PYRt2" id="R_PYRt2" name="Pyruvate-Proton symport"
reversible="true" fast="false" fbc:lowerFluxBound="rev_lb"
fbc:upperFluxBound="rev_ub">
            <listOfReactants>
                  <speciesReference species="M_h_e" stoichiometry="1"
constant="true"/>
                  <speciesReference species="M_pyr_e" stoichiometry="1"
constant="true"/>
            </listOfReactants>
            <listOfProducts>
                  <speciesReference species="M_h_c" stoichiometry="1"
constant="true"/>
                  <speciesReference species="M_pyr_c" stoichiometry="1"
constant="true"/>
            </listOfProducts>
      </reaction>

      <reaction metaid="R_PYRZAM" id="R_PYRZAM" name="Pyrazinamidase"
reversible="false" fast="false" fbc:lowerFluxBound="irr_lb"
fbc:upperFluxBound="irr_ub">
            <listOfReactants>
                  <speciesReference species="M_h2o_c" stoichiometry="1"
constant="true"/>
                  <speciesReference species="M_malm_c" stoichiometry="1"
constant="true"/>
            </listOfReactants>
            <listOfProducts>
```

```
                  <speciesReference species="M_nh4_c" stoichiometry="1"
constant="true"/>
                  <speciesReference species="M_male_c" stoichiometry="1"
constant="true"/>
            &lt;/listOfProducts&gt;
            &lt;fbc:geneProductAssociation&gt;
                  &lt;fbc:geneProductRef fbc:geneProduct="SAUSA300_1899"/&gt;
            &lt;/fbc:geneProductAssociation&gt;
      &lt;/reaction&gt;

      <reaction metaid="R_RAFH" id="R_RAFH" name="raffinose hydrolyzing
enzyme" reversible="true" fast="false" fbc:lowerFluxBound="rev_lb"
fbc:upperFluxBound="rev_ub">
            &lt;listOfReactants&gt;
                  <speciesReference species="M_h2o_c" stoichiometry="1"
constant="true"/>
                  <speciesReference species="M_raffin_c" stoichiometry="1"
constant="true"/>
            &lt;/listOfReactants&gt;
            &lt;listOfProducts&gt;
                  <speciesReference species="M_fru_c" stoichiometry="1"
constant="true"/>
                  <speciesReference species="M_melib_c" stoichiometry="1"
constant="true"/>
            &lt;/listOfProducts&gt;
            &lt;fbc:geneProductAssociation&gt;
                  &lt;fbc:geneProductRef fbc:geneProduct="SAUSA300_1994"/&gt;
            &lt;/fbc:geneProductAssociation&gt;
      &lt;/reaction&gt;

      <reaction metaid="R_RBFK" id="R_RBFK" name="riboflavin kinase"
reversible="false" fast="false" fbc:lowerFluxBound="irr_lb"
fbc:upperFluxBound="irr_ub">
            &lt;listOfReactants&gt;
                  <speciesReference species="M_atp_c" stoichiometry="1"
constant="true"/>
                  <speciesReference species="M_ribflv_c" stoichiometry="1"
constant="true"/>
            &lt;/listOfReactants&gt;
            &lt;listOfProducts&gt;
                  <speciesReference species="M_adp_c" stoichiometry="1"
constant="true"/>
                  <speciesReference species="M_fmn_c" stoichiometry="1"
constant="true"/>
            &lt;/listOfProducts&gt;
            &lt;fbc:geneProductAssociation&gt;
                  &lt;fbc:geneProductRef fbc:geneProduct="SAUSA300_1165"/&gt;
            &lt;/fbc:geneProductAssociation&gt;
      &lt;/reaction&gt;

      <reaction metaid="R_RBFSa" id="R_RBFSa" name="riboflavin synthase"
reversible="false" fast="false" fbc:lowerFluxBound="irr_lb"
fbc:upperFluxBound="irr_ub">
            &lt;listOfReactants&gt;
                  <speciesReference species="M_4r5au_c" stoichiometry="1"
constant="true"/>
                  <speciesReference species="M_db4p_c" stoichiometry="1"
constant="true"/>
```

```
            </listOfReactants>
            <listOfProducts>
                  <speciesReference species="M_h_c" stoichiometry="1"
constant="true"/>
                  <speciesReference species="M_h2o_c" stoichiometry="2"
constant="true"/>
                  <speciesReference species="M_pi_c" stoichiometry="1"
constant="true"/>
                  <speciesReference species="M_dmlz_c" stoichiometry="1"
constant="true"/>
            </listOfProducts>
            <fbc:geneProductAssociation>
                  <fbc:geneProductRef fbc:geneProduct="SAUSA300_1712"/>
            </fbc:geneProductAssociation>
      </reaction>

      <reaction metaid="R_RBFSb" id="R_RBFSb" name="riboflavin synthase"
reversible="false" fast="false" fbc:lowerFluxBound="irr_lb"
fbc:upperFluxBound="irr_ub">
            <listOfReactants>
                  <speciesReference species="M_dmlz_c" stoichiometry="2"
constant="true"/>
            </listOfReactants>
            <listOfProducts>
                  <speciesReference species="M_ribflv_c" stoichiometry="1"
constant="true"/>
                  <speciesReference species="M_4r5au_c" stoichiometry="1"
constant="true"/>
            </listOfProducts>
            <fbc:geneProductAssociation>
                  <fbc:and>
                        <fbc:geneProductRef fbc:geneProduct="SAUSA300_1714"/>
                        <fbc:geneProductRef fbc:geneProduct="SAUSA300_1712"/>
                  </fbc:and>
            </fbc:geneProductAssociation>
      </reaction>

      <reaction metaid="R_RBK_Dr" id="R_RBK_Dr" name="D-ribulokinase
(reversible)" reversible="false" fast="false" fbc:lowerFluxBound="irr_lb"
fbc:upperFluxBound="irr_ub">
            <listOfReactants>
                  <speciesReference species="M_atp_c" stoichiometry="1"
constant="true"/>
                  <speciesReference species="M_rbl__D_c" stoichiometry="1"
constant="true"/>
            </listOfReactants>
            <listOfProducts>
                  <speciesReference species="M_adp_c" stoichiometry="1"
constant="true"/>
                  <speciesReference species="M_ru5p__D_c" stoichiometry="1"
constant="true"/>
            </listOfProducts>
            <fbc:geneProductAssociation>
                  <fbc:geneProductRef fbc:geneProduct="SAUSA300_0537"/>
            </fbc:geneProductAssociation>
      </reaction>
```

```
      <reaction metaid="R_RBK_L1" id="R_RBK_L1" name="L-ribulokinase (L-
ribulose)" reversible="false" fast="false" fbc:lowerFluxBound="irr_lb"
fbc:upperFluxBound="irr_ub">
            <listOfReactants>
                  <speciesReference species="M_atp_c" stoichiometry="1"
constant="true"/>
                  <speciesReference species="M_rbl__L_c" stoichiometry="1"
constant="true"/>
            </listOfReactants>
            <listOfProducts>
                  <speciesReference species="M_adp_c" stoichiometry="1"
constant="true"/>
                  <speciesReference species="M_ru5p__L_c" stoichiometry="1"
constant="true"/>
            </listOfProducts>
            <fbc:geneProductAssociation>
                  <fbc:geneProductRef fbc:geneProduct="SAUSA300_0537"/>
            </fbc:geneProductAssociation>
      </reaction>

      <reaction metaid="R_RBKr" id="R_RBKr" name="ribokinase reversible"
reversible="false" fast="false" fbc:lowerFluxBound="irr_lb"
fbc:upperFluxBound="irr_ub">
            <listOfReactants>
                  <speciesReference species="M_atp_c" stoichiometry="1"
constant="true"/>
                  <speciesReference species="M_rib__D_c" stoichiometry="1"
constant="true"/>
            </listOfReactants>
            <listOfProducts>
                  <speciesReference species="M_adp_c" stoichiometry="1"
constant="true"/>
                  <speciesReference species="M_r5p_c" stoichiometry="1"
constant="true"/>
            </listOfProducts>
            <fbc:geneProductAssociation>
                  <fbc:geneProductRef fbc:geneProduct="SAUSA300_0262"/>
            </fbc:geneProductAssociation>
      </reaction>

      <reaction metaid="R_RE0453C" id="R_RE0453C"
name="Nucleoside__Diphosphate Kinase" reversible="true" fast="false"
fbc:lowerFluxBound="rev_lb" fbc:upperFluxBound="rev_ub">
            <listOfReactants>
                  <speciesReference species="M_dtdp_c" stoichiometry="1"
constant="true"/>
                  <speciesReference species="M_datp_c" stoichiometry="1"
constant="true"/>
            </listOfReactants>
            <listOfProducts>
                  <speciesReference species="M_dadp_c" stoichiometry="1"
constant="true"/>
                  <speciesReference species="M_dttp_c" stoichiometry="1"
constant="true"/>
            </listOfProducts>
            <fbc:geneProductAssociation>
                  <fbc:geneProductRef fbc:geneProduct="SAUSA300_1358"/>
            </fbc:geneProductAssociation>
```

```
      </reaction>

      <reaction metaid="R_RETOx" id="R_RETOx" name="Retinol NAD
oxidoreductase " reversible="true" fast="false" fbc:lowerFluxBound="rev_lb"
fbc:upperFluxBound="rev_ub">
            <listOfReactants>
                  <speciesReference species="M_nad_c" stoichiometry="1"
constant="true"/>
                  <speciesReference species="M_retinol_c" stoichiometry="1"
constant="true"/>
            </listOfReactants>
            <listOfProducts>
                  <speciesReference species="M_h_c" stoichiometry="1"
constant="true"/>
                  <speciesReference species="M_nadh_c" stoichiometry="1"
constant="true"/>
                  <speciesReference species="M_retinal_c" stoichiometry="1"
constant="true"/>
            </listOfProducts>
            <fbc:geneProductAssociation>
                  <fbc:geneProductRef fbc:geneProduct="SAUSA300_2147"/>
            </fbc:geneProductAssociation>
      </reaction>

      <reaction metaid="R_RHCCE" id="R_RHCCE" name="S-ribosylhomocysteine
cleavage enzyme" reversible="false" fast="false" fbc:lowerFluxBound="irr_lb"
fbc:upperFluxBound="irr_ub">
            <listOfReactants>
                  <speciesReference species="M_rhcys_c" stoichiometry="1"
constant="true"/>
            </listOfReactants>
            <listOfProducts>
                  <speciesReference species="M_hcys__L_c" stoichiometry="1"
constant="true"/>
                  <speciesReference species="M_dhptd_c" stoichiometry="1"
constant="true"/>
            </listOfProducts>
            <fbc:geneProductAssociation>
                  <fbc:geneProductRef fbc:geneProduct="SAUSA300_2088"/>
            </fbc:geneProductAssociation>
      </reaction>

      <reaction metaid="R_RIBabc" id="R_RIBabc" name="D-ribose transport via
ABC system" reversible="false" fast="false" fbc:lowerFluxBound="irr_lb"
fbc:upperFluxBound="irr_ub">
            <listOfReactants>
                  <speciesReference species="M_h2o_c" stoichiometry="1"
constant="true"/>
                  <speciesReference species="M_atp_c" stoichiometry="1"
constant="true"/>
                  <speciesReference species="M_rib__D_e" stoichiometry="1"
constant="true"/>
            </listOfReactants>
            <listOfProducts>
                  <speciesReference species="M_h_c" stoichiometry="1"
constant="true"/>
                  <speciesReference species="M_pi_c" stoichiometry="1"
constant="true"/>
```

```
                  <speciesReference species="M_adp_c" stoichiometry="1"
constant="true"/>
                  <speciesReference species="M_rib__D_c" stoichiometry="1"
constant="true"/>
            &lt;/listOfProducts&gt;
            &lt;fbc:geneProductAssociation&gt;
                  &lt;fbc:or&gt;
                        &lt;fbc:geneProductRef fbc:geneProduct="SAUSA300_0263"/&gt;
                        &lt;fbc:geneProductRef fbc:geneProduct="SAUSA300_0264"/&gt;
                  &lt;/fbc:or&gt;
            &lt;/fbc:geneProductAssociation&gt;
      &lt;/reaction&gt;

      <reaction metaid="R_RIBabc2" id="R_RIBabc2" name="D-ribose transport
out via ABC system " reversible="false" fast="false"
fbc:lowerFluxBound="irr_lb" fbc:upperFluxBound="irr_ub">
            &lt;listOfReactants&gt;
                  <speciesReference species="M_h2o_c" stoichiometry="1"
constant="true"/>
                  <speciesReference species="M_atp_c" stoichiometry="1"
constant="true"/>
                  <speciesReference species="M_rib__D_c" stoichiometry="1"
constant="true"/>
            &lt;/listOfReactants&gt;
            &lt;listOfProducts&gt;
                  <speciesReference species="M_h_c" stoichiometry="1"
constant="true"/>
                  <speciesReference species="M_pi_c" stoichiometry="1"
constant="true"/>
                  <speciesReference species="M_adp_c" stoichiometry="1"
constant="true"/>
                  <speciesReference species="M_rib__D_e" stoichiometry="1"
constant="true"/>
            &lt;/listOfProducts&gt;
            &lt;fbc:geneProductAssociation&gt;
                  &lt;fbc:or&gt;
                        &lt;fbc:geneProductRef fbc:geneProduct="SAUSA300_0264"/&gt;
                        &lt;fbc:geneProductRef fbc:geneProduct="SAUSA300_0263"/&gt;
                  &lt;/fbc:or&gt;
            &lt;/fbc:geneProductAssociation&gt;
      &lt;/reaction&gt;

      <reaction metaid="R_RIBFLVt2" id="R_RIBFLVt2" name="riboflavin
transport in via proton symport" reversible="true" fast="false"
fbc:lowerFluxBound="rev_lb" fbc:upperFluxBound="rev_ub">
            &lt;listOfReactants&gt;
                  <speciesReference species="M_h_e" stoichiometry="1"
constant="true"/>
                  <speciesReference species="M_ribflv_e" stoichiometry="1"
constant="true"/>
            &lt;/listOfReactants&gt;
            &lt;listOfProducts&gt;
                  <speciesReference species="M_h_c" stoichiometry="1"
constant="true"/>
                  <speciesReference species="M_ribflv_c" stoichiometry="1"
constant="true"/>
            &lt;/listOfProducts&gt;
            &lt;fbc:geneProductAssociation&gt;
```

```xml
                  &lt;fbc:geneProductRef fbc:geneProduct="SAUSA300_1374"/&gt;
            &lt;/fbc:geneProductAssociation&gt;
      &lt;/reaction&gt;

      <reaction metaid="R_RNDR1" id="R_RNDR1"
name="ribonucleoside__Diphosphate reductase (ADP)" reversible="false"
fast="false" fbc:lowerFluxBound="irr_lb" fbc:upperFluxBound="irr_ub">
            &lt;listOfReactants&gt;
                  <speciesReference species="M_adp_c" stoichiometry="1"
constant="true"/>
                  <speciesReference species="M_trdrd_c" stoichiometry="1"
constant="true"/>
            &lt;/listOfReactants&gt;
            &lt;listOfProducts&gt;
                  <speciesReference species="M_h2o_c" stoichiometry="1"
constant="true"/>
                  <speciesReference species="M_trdox_c" stoichiometry="1"
constant="true"/>
                  <speciesReference species="M_dadp_c" stoichiometry="1"
constant="true"/>
            &lt;/listOfProducts&gt;
            &lt;fbc:geneProductAssociation&gt;
                  &lt;fbc:and&gt;
                        &lt;fbc:geneProductRef fbc:geneProduct="SAUSA300_0716"/&gt;
                        &lt;fbc:geneProductRef fbc:geneProduct="SAUSA300_0717"/&gt;
                        &lt;fbc:geneProductRef fbc:geneProduct="SAUSA300_0715"/&gt;
                  &lt;/fbc:and&gt;
            &lt;/fbc:geneProductAssociation&gt;
      &lt;/reaction&gt;

      <reaction metaid="R_RNDR2" id="R_RNDR2"
name="ribonucleoside__Diphosphate reductase (GDP)" reversible="false"
fast="false" fbc:lowerFluxBound="irr_lb" fbc:upperFluxBound="irr_ub">
            &lt;listOfReactants&gt;
                  <speciesReference species="M_trdrd_c" stoichiometry="1"
constant="true"/>
                  <speciesReference species="M_gdp_c" stoichiometry="1"
constant="true"/>
            &lt;/listOfReactants&gt;
            &lt;listOfProducts&gt;
                  <speciesReference species="M_h2o_c" stoichiometry="1"
constant="true"/>
                  <speciesReference species="M_trdox_c" stoichiometry="1"
constant="true"/>
                  <speciesReference species="M_dgdp_c" stoichiometry="1"
constant="true"/>
            &lt;/listOfProducts&gt;
            &lt;fbc:geneProductAssociation&gt;
                  &lt;fbc:and&gt;
                        &lt;fbc:geneProductRef fbc:geneProduct="SAUSA300_0716"/&gt;
                        &lt;fbc:geneProductRef fbc:geneProduct="SAUSA300_0717"/&gt;
                        &lt;fbc:geneProductRef fbc:geneProduct="SAUSA300_0715"/&gt;
                  &lt;/fbc:and&gt;
            &lt;/fbc:geneProductAssociation&gt;
      &lt;/reaction&gt;
```

```
      <reaction metaid="R_RNDR3" id="R_RNDR3"
name="ribonucleoside__Diphosphate reductase (CDP)" reversible="false"
fast="false" fbc:lowerFluxBound="irr_lb" fbc:upperFluxBound="irr_ub">
            <listOfReactants>
                  <speciesReference species="M_trdrd_c" stoichiometry="1"
constant="true"/>
                  <speciesReference species="M_cdp_c" stoichiometry="1"
constant="true"/>
            </listOfReactants>
            <listOfProducts>
                  <speciesReference species="M_h2o_c" stoichiometry="1"
constant="true"/>
                  <speciesReference species="M_trdox_c" stoichiometry="1"
constant="true"/>
                  <speciesReference species="M_dcdp_c" stoichiometry="1"
constant="true"/>
            </listOfProducts>
            <fbc:geneProductAssociation>
                  <fbc:and>
                        <fbc:geneProductRef fbc:geneProduct="SAUSA300_0716"/>
                        <fbc:geneProductRef fbc:geneProduct="SAUSA300_0717"/>
                        <fbc:geneProductRef fbc:geneProduct="SAUSA300_0715"/>
                  </fbc:and>
            </fbc:geneProductAssociation>
      </reaction>

      <reaction metaid="R_RNDR4" id="R_RNDR4"
name="ribonucleoside__Diphosphate reductase (UDP)" reversible="false"
fast="false" fbc:lowerFluxBound="irr_lb" fbc:upperFluxBound="irr_ub">
            <listOfReactants>
                  <speciesReference species="M_udp_c" stoichiometry="1"
constant="true"/>
                  <speciesReference species="M_trdrd_c" stoichiometry="1"
constant="true"/>
            </listOfReactants>
            <listOfProducts>
                  <speciesReference species="M_h2o_c" stoichiometry="1"
constant="true"/>
                  <speciesReference species="M_trdox_c" stoichiometry="1"
constant="true"/>
                  <speciesReference species="M_dudp_c" stoichiometry="1"
constant="true"/>
            </listOfProducts>
            <fbc:geneProductAssociation>
                  <fbc:and>
                        <fbc:geneProductRef fbc:geneProduct="SAUSA300_0716"/>
                        <fbc:geneProductRef fbc:geneProduct="SAUSA300_0717"/>
                        <fbc:geneProductRef fbc:geneProduct="SAUSA300_0715"/>
                  </fbc:and>
            </fbc:geneProductAssociation>
      </reaction>

      <reaction metaid="R_RNTR1" id="R_RNTR1" name="ribonucleoside-
triphosphate reductase (ATP)" reversible="false" fast="false"
fbc:lowerFluxBound="irr_lb" fbc:upperFluxBound="irr_ub">
            <listOfReactants>
                  <speciesReference species="M_atp_c" stoichiometry="1"
constant="true"/>
```

```
                  <speciesReference species="M_trdrd_c" stoichiometry="1"
constant="true"/>
            </listOfReactants>
            <listOfProducts>
                  <speciesReference species="M_h2o_c" stoichiometry="1"
constant="true"/>
                  <speciesReference species="M_trdox_c" stoichiometry="1"
constant="true"/>
                  <speciesReference species="M_datp_c" stoichiometry="1"
constant="true"/>
            </listOfProducts>
            <fbc:geneProductAssociation>
                  <fbc:and>
                        <fbc:geneProductRef fbc:geneProduct="SAUSA300_2551"/>
                        <fbc:geneProductRef fbc:geneProduct="SAUSA300_2550"/>
                  </fbc:and>
            </fbc:geneProductAssociation>
      </reaction>

      <reaction metaid="R_RNTR2" id="R_RNTR2" name="ribonucleoside-
triphosphate reductase (GTP)" reversible="false" fast="false"
fbc:lowerFluxBound="irr_lb" fbc:upperFluxBound="irr_ub">
            <listOfReactants>
                  <speciesReference species="M_trdrd_c" stoichiometry="1"
constant="true"/>
                  <speciesReference species="M_gtp_c" stoichiometry="1"
constant="true"/>
            </listOfReactants>
            <listOfProducts>
                  <speciesReference species="M_h2o_c" stoichiometry="1"
constant="true"/>
                  <speciesReference species="M_trdox_c" stoichiometry="1"
constant="true"/>
                  <speciesReference species="M_dgtp_c" stoichiometry="1"
constant="true"/>
            </listOfProducts>
            <fbc:geneProductAssociation>
                  <fbc:and>
                        <fbc:geneProductRef fbc:geneProduct="SAUSA300_2551"/>
                        <fbc:geneProductRef fbc:geneProduct="SAUSA300_2550"/>
                  </fbc:and>
            </fbc:geneProductAssociation>
      </reaction>

      <reaction metaid="R_RNTR3" id="R_RNTR3" name="ribonucleoside-
triphosphate reductase (CTP)" reversible="false" fast="false"
fbc:lowerFluxBound="irr_lb" fbc:upperFluxBound="irr_ub">
            <listOfReactants>
                  <speciesReference species="M_ctp_c" stoichiometry="1"
constant="true"/>
                  <speciesReference species="M_trdrd_c" stoichiometry="1"
constant="true"/>
            </listOfReactants>
            <listOfProducts>
                  <speciesReference species="M_h2o_c" stoichiometry="1"
constant="true"/>
                  <speciesReference species="M_trdox_c" stoichiometry="1"
constant="true"/>
```

```xml
                  <speciesReference species="M_dctp_c" stoichiometry="1"
constant="true"/>
            </listOfProducts>
            <fbc:geneProductAssociation>
                  <fbc:and>
                        <fbc:geneProductRef fbc:geneProduct="SAUSA300_2551"/>
                        <fbc:geneProductRef fbc:geneProduct="SAUSA300_2550"/>
                  </fbc:and>
            </fbc:geneProductAssociation>
      </reaction>

      <reaction metaid="R_RNTR4" id="R_RNTR4" name="ribonucleoside-
triphosphate reductase (UTP)" reversible="false" fast="false"
fbc:lowerFluxBound="irr_lb" fbc:upperFluxBound="irr_ub">
            <listOfReactants>
                  <speciesReference species="M_trdrd_c" stoichiometry="1"
constant="true"/>
                  <speciesReference species="M_utp_c" stoichiometry="1"
constant="true"/>
            </listOfReactants>
            <listOfProducts>
                  <speciesReference species="M_h2o_c" stoichiometry="1"
constant="true"/>
                  <speciesReference species="M_trdox_c" stoichiometry="1"
constant="true"/>
                  <speciesReference species="M_dutp_c" stoichiometry="1"
constant="true"/>
            </listOfProducts>
            <fbc:geneProductAssociation>
                  <fbc:and>
                        <fbc:geneProductRef fbc:geneProduct="SAUSA300_2551"/>
                        <fbc:geneProductRef fbc:geneProduct="SAUSA300_2550"/>
                  </fbc:and>
            </fbc:geneProductAssociation>
      </reaction>

      <reaction metaid="R_RPE" id="R_RPE" name="ribulose 5-phosphate 3-
epimerase" reversible="true" fast="false" fbc:lowerFluxBound="rev_lb"
fbc:upperFluxBound="rev_ub">
            <listOfReactants>
                  <speciesReference species="M_ru5p__D_c" stoichiometry="1"
constant="true"/>
            </listOfReactants>
            <listOfProducts>
                  <speciesReference species="M_xu5p__D_c" stoichiometry="1"
constant="true"/>
            </listOfProducts>
            <fbc:geneProductAssociation>
                  <fbc:geneProductRef fbc:geneProduct="SAUSA300_1115"/>
            </fbc:geneProductAssociation>
      </reaction>

      <reaction metaid="R_RPI" id="R_RPI" name="ribose-5-phosphate isomerase"
reversible="true" fast="false" fbc:lowerFluxBound="rev_lb"
fbc:upperFluxBound="rev_ub">
            <listOfReactants>
                  <speciesReference species="M_r5p_c" stoichiometry="1"
constant="true"/>
```

```
            &lt;/listOfReactants&gt;
            &lt;listOfProducts&gt;
                  <speciesReference species="M_ru5p__D_c" stoichiometry="1"
constant="true"/>
            &lt;/listOfProducts&gt;
            &lt;fbc:geneProductAssociation&gt;
                  &lt;fbc:geneProductRef fbc:geneProduct="SAUSA300_2283"/&gt;
            &lt;/fbc:geneProductAssociation&gt;
      &lt;/reaction&gt;

      <reaction metaid="R_RSLML" id="R_RSLML" name="R-S__Lactoylglutathione
methylglyoxal__Lyase isomerizing " reversible="true" fast="false"
fbc:lowerFluxBound="rev_lb" fbc:upperFluxBound="rev_ub">
            &lt;listOfReactants&gt;
                  <speciesReference species="M_lgt__S_c" stoichiometry="1"
constant="true"/>
            &lt;/listOfReactants&gt;
            &lt;listOfProducts&gt;
                  <speciesReference species="M_gthrd_c" stoichiometry="1"
constant="true"/>
                  <speciesReference species="M_mthgxl_c" stoichiometry="1"
constant="true"/>
            &lt;/listOfProducts&gt;
            &lt;fbc:geneProductAssociation&gt;
                  &lt;fbc:geneProductRef fbc:geneProduct="SAUSA300_1458"/&gt;
            &lt;/fbc:geneProductAssociation&gt;
      &lt;/reaction&gt;

      <reaction metaid="R_RU5PS" id="R_RU5PS" name="Ribulose-5-P Synthetase"
reversible="true" fast="false" fbc:lowerFluxBound="rev_lb"
fbc:upperFluxBound="rev_ub">
            &lt;listOfReactants&gt;
                  <speciesReference species="M_ru5p__D_c" stoichiometry="1"
constant="true"/>
                  <speciesReference species="M_fald_c" stoichiometry="1"
constant="true"/>
            &lt;/listOfReactants&gt;
            &lt;listOfProducts&gt;
                  <speciesReference species="M_ah6p__D_c" stoichiometry="1"
constant="true"/>
            &lt;/listOfProducts&gt;
            &lt;fbc:geneProductAssociation&gt;
                  &lt;fbc:geneProductRef fbc:geneProduct="SAUSA300_0555"/&gt;
            &lt;/fbc:geneProductAssociation&gt;
      &lt;/reaction&gt;

      <reaction metaid="R_S1AGPA" id="R_S1AGPA" name="stearoyl-1-
acylglycerol-3-phosphate O-acyltransferase " reversible="false" fast="false"
fbc:lowerFluxBound="irr_lb" fbc:upperFluxBound="irr_ub">
            &lt;listOfReactants&gt;
                  <speciesReference species="M_stcoa_c" stoichiometry="1"
constant="true"/>
                  <speciesReference species="M_1odecg3p_c" stoichiometry="1"
constant="true"/>
            &lt;/listOfReactants&gt;
            &lt;listOfProducts&gt;
                  <speciesReference species="M_coa_c" stoichiometry="1"
constant="true"/>
```

```
                  <speciesReference species="M_pa180_c" stoichiometry="1"
constant="true"/>
            </listOfProducts>
            <fbc:geneProductAssociation>
                  <fbc:geneProductRef fbc:geneProduct="SAUSA300_1673"/>
            </fbc:geneProductAssociation>
      </reaction>

      <reaction metaid="R_S7PIr" id="R_S7PIr" name="sedoheptulose 7-phosphate
isomerase (reversible)" reversible="true" fast="false"
fbc:lowerFluxBound="rev_lb" fbc:upperFluxBound="rev_ub">
            <listOfReactants>
                  <speciesReference species="M_s7p_c" stoichiometry="1"
constant="true"/>
            </listOfReactants>
            <listOfProducts>
                  <speciesReference species="M_gmhep7p_c" stoichiometry="1"
constant="true"/>
            </listOfProducts>
            <fbc:geneProductAssociation>
                  <fbc:geneProductRef fbc:geneProduct="SAUSA300_0317"/>
            </fbc:geneProductAssociation>
      </reaction>

      <reaction metaid="R_Free_FA" id="R_Free_FA" name="Free Fatty Acid
composition for SA" reversible="false" fast="false"
fbc:lowerFluxBound="irr_lb" fbc:upperFluxBound="irr_ub">
            <listOfReactants>
                  <speciesReference species="M_hdca_c" stoichiometry="10.1"
constant="true"/>
                  <speciesReference species="M_fa4_c" stoichiometry="32.7"
constant="true"/>
                  <speciesReference species="M_fa12_c" stoichiometry="18.1"
constant="true"/>
                  <speciesReference species="M_fa3_c" stoichiometry="19.3"
constant="true"/>
                  <speciesReference species="M_fa6_c" stoichiometry="4.7"
constant="true"/>
                  <speciesReference species="M_fa11_c" stoichiometry="7.7"
constant="true"/>
            </listOfReactants>
            <listOfProducts>
                  <speciesReference species="M_SA_FREE_FA_c"
stoichiometry="1" constant="true"/>
            </listOfProducts>
      </reaction>

      <reaction metaid="R_SADT2" id="R_SADT2" name="Sulfate
adenyltransferase" reversible="false" fast="false"
fbc:lowerFluxBound="irr_lb" fbc:upperFluxBound="irr_ub">
            <listOfReactants>
                  <speciesReference species="M_h2o_c" stoichiometry="1"
constant="true"/>
                  <speciesReference species="M_atp_c" stoichiometry="1"
constant="true"/>
                  <speciesReference species="M_so4_c" stoichiometry="1"
constant="true"/>
```

```
                  <speciesReference species="M_gtp_c" stoichiometry="1"
constant="true"/>
            </listOfReactants>
            <listOfProducts>
                  <speciesReference species="M_pi_c" stoichiometry="1"
constant="true"/>
                  <speciesReference species="M_ppi_c" stoichiometry="1"
constant="true"/>
                  <speciesReference species="M_gdp_c" stoichiometry="1"
constant="true"/>
                  <speciesReference species="M_aps_c" stoichiometry="1"
constant="true"/>
            </listOfProducts>
            <fbc:geneProductAssociation>
                  <fbc:geneProductRef fbc:geneProduct="SAUSA300_0533"/>
            </fbc:geneProductAssociation>
      </reaction>

      <reaction metaid="R_SALCOD" id="R_SALCOD" name="salicylate NADH oxygen
oxidoreductase 1-hydroxylating  " reversible="false" fast="false"
fbc:lowerFluxBound="irr_lb" fbc:upperFluxBound="irr_ub">
            <listOfReactants>
                  <speciesReference species="M_h_c" stoichiometry="2"
constant="true"/>
                  <speciesReference species="M_o2_c" stoichiometry="1"
constant="true"/>
                  <speciesReference species="M_nadh_c" stoichiometry="1"
constant="true"/>
                  <speciesReference species="M_salc_c" stoichiometry="1"
constant="true"/>
            </listOfReactants>
            <listOfProducts>
                  <speciesReference species="M_h2o_c" stoichiometry="1"
constant="true"/>
                  <speciesReference species="M_co2_c" stoichiometry="1"
constant="true"/>
                  <speciesReference species="M_nad_c" stoichiometry="1"
constant="true"/>
                  <speciesReference species="M_catechol_c" stoichiometry="1"
constant="true"/>
            </listOfProducts>
            <fbc:geneProductAssociation>
                  <fbc:geneProductRef fbc:geneProduct="SAUSA300_2255"/>
            </fbc:geneProductAssociation>
      </reaction>

      <reaction metaid="R_SALCpts" id="R_SALCpts" name="salicin transport via
PEP:Pyr PTS" reversible="false" fast="false" fbc:lowerFluxBound="irr_lb"
fbc:upperFluxBound="irr_ub">
            <listOfReactants>
                  <speciesReference species="M_pep_c" stoichiometry="1"
constant="true"/>
                  <speciesReference species="M_rbl__B_e" stoichiometry="1"
constant="true"/>
            </listOfReactants>
            <listOfProducts>
                  <speciesReference species="M_pyr_c" stoichiometry="1"
constant="true"/>
```

```
                  <speciesReference species="M_salc6p_c" stoichiometry="1"
constant="true"/>
            </listOfProducts>
            <fbc:geneProductAssociation>
                  <fbc:and>
                        <fbc:geneProductRef fbc:geneProduct="SAUSA300_0983"/>
                        <fbc:geneProductRef fbc:geneProduct="SAUSA300_0984"/>
                  </fbc:and>
            </fbc:geneProductAssociation>
      </reaction>

      <reaction metaid="R_SALHL" id="R_SALHL" name="rxn03906 "
reversible="false" fast="false" fbc:lowerFluxBound="irr_lb"
fbc:upperFluxBound="irr_ub">
            <listOfReactants>
                  <speciesReference species="M_h_c" stoichiometry="2"
constant="true"/>
                  <speciesReference species="M_o2_c" stoichiometry="1"
constant="true"/>
                  <speciesReference species="M_nadh_c" stoichiometry="1"
constant="true"/>
                  <speciesReference species="M_1h2nap_c" stoichiometry="1"
constant="true"/>
            </listOfReactants>
            <listOfProducts>
                  <speciesReference species="M_h2o_c" stoichiometry="1"
constant="true"/>
                  <speciesReference species="M_co2_c" stoichiometry="1"
constant="true"/>
                  <speciesReference species="M_nad_c" stoichiometry="1"
constant="true"/>
                  <speciesReference species="M_12napdol_c" stoichiometry="1"
constant="true"/>
            </listOfProducts>
            <fbc:geneProductAssociation>
                  <fbc:geneProductRef fbc:geneProduct="SAUSA300_2255"/>
            </fbc:geneProductAssociation>
      </reaction>

      <reaction metaid="R_SALOR" id="R_SALOR" name="salicylate NADH oxygen
oxidoreductase " reversible="false" fast="false" fbc:lowerFluxBound="irr_lb"
fbc:upperFluxBound="irr_ub">
            <listOfReactants>
                  <speciesReference species="M_h_c" stoichiometry="2"
constant="true"/>
                  <speciesReference species="M_o2_c" stoichiometry="1"
constant="true"/>
                  <speciesReference species="M_nadh_c" stoichiometry="1"
constant="true"/>
                  <speciesReference species="M_4hmsial_c" stoichiometry="1"
constant="true"/>
            </listOfReactants>
            <listOfProducts>
                  <speciesReference species="M_h2o_c" stoichiometry="1"
constant="true"/>
                  <speciesReference species="M_co2_c" stoichiometry="1"
constant="true"/>
```

```
                  <speciesReference species="M_nad_c" stoichiometry="1"
constant="true"/>
                  <speciesReference species="M_3hmcat_c" stoichiometry="1"
constant="true"/>
            </listOfProducts>
            <fbc:geneProductAssociation>
                  <fbc:geneProductRef fbc:geneProduct="SAUSA300_2255"/>
            </fbc:geneProductAssociation>
      </reaction>

      <reaction metaid="R_SAS24" id="R_SAS24" name="rxn07624 "
reversible="true" fast="false" fbc:lowerFluxBound="rev_lb"
fbc:upperFluxBound="rev_ub">
            <listOfReactants>
                  <speciesReference species="M_coa_c" stoichiometry="1"
constant="true"/>
                  <speciesReference species="M_35oxcoa" stoichiometry="1"
constant="true"/>
            </listOfReactants>
            <listOfProducts>
                  <speciesReference species="M_3hbcoa_c" stoichiometry="1"
constant="true"/>
                  <speciesReference species="M_accoa_c" stoichiometry="1"
constant="true"/>
            </listOfProducts>
            <fbc:geneProductAssociation>
                  <fbc:geneProductRef fbc:geneProduct="SAUSA300_2505"/>
            </fbc:geneProductAssociation>
      </reaction>

      <reaction metaid="R_SAS65" id="R_SAS65" name="rxn11965 "
reversible="true" fast="false" fbc:lowerFluxBound="rev_lb"
fbc:upperFluxBound="rev_ub">
            <listOfReactants>
                  <speciesReference species="M_h_c" stoichiometry="1"
constant="true"/>
                  <speciesReference species="M_pplhis" stoichiometry="1"
constant="true"/>
                  <speciesReference species="M_tre_c" stoichiometry="1"
constant="true"/>
            </listOfReactants>
            <listOfProducts>
                  <speciesReference species="M_tre6p_c" stoichiometry="1"
constant="true"/>
                  <speciesReference species="M_phis__L" stoichiometry="1"
constant="true"/>
            </listOfProducts>
            <fbc:geneProductAssociation>
                  <fbc:geneProductRef fbc:geneProduct="SAUSA300_1315"/>
            </fbc:geneProductAssociation>
      </reaction>

      <reaction metaid="R_SAS89" id="R_SAS89" name="rxn07486 "
reversible="true" fast="false" fbc:lowerFluxBound="rev_lb"
fbc:upperFluxBound="rev_ub">
            <listOfReactants>
                  <speciesReference species="M_h_c" stoichiometry="1"
constant="true"/>
```

```
                  <speciesReference species="M_pplhis" stoichiometry="1"
constant="true"/>
                  <speciesReference species="M_l23uo" stoichiometry="1"
constant="true"/>
            </listOfReactants>
            <listOfProducts>
                  <speciesReference species="M_ascb6p_c" stoichiometry="1"
constant="true"/>
                  <speciesReference species="M_phis__L" stoichiometry="1"
constant="true"/>
            </listOfProducts>
            <fbc:geneProductAssociation>
                  <fbc:geneProductRef fbc:geneProduct="SAUSA300_0332"/>
            </fbc:geneProductAssociation>
      </reaction>

      <reaction metaid="R_SAS96" id="R_SAS96" name="rxn03796 "
reversible="true" fast="false" fbc:lowerFluxBound="rev_lb"
fbc:upperFluxBound="rev_ub">
            <listOfReactants>
                  <speciesReference species="M_coa_c" stoichiometry="1"
constant="true"/>
                  <speciesReference species="M_3o3pc" stoichiometry="1"
constant="true"/>
            </listOfReactants>
            <listOfProducts>
                  <speciesReference species="M_accoa_c" stoichiometry="1"
constant="true"/>
                  <speciesReference species="M_benzcoa_c" stoichiometry="1"
constant="true"/>
            </listOfProducts>
            <fbc:geneProductAssociation>
                  <fbc:geneProductRef fbc:geneProduct="SAUSA300_2505"/>
            </fbc:geneProductAssociation>
      </reaction>

      <reaction metaid="R_SBTpts" id="R_SBTpts" name="D-sorbitol transport
via PEP:Pyr PTS" reversible="true" fast="false" fbc:lowerFluxBound="rev_lb"
fbc:upperFluxBound="rev_ub">
            <listOfReactants>
                  <speciesReference species="M_pep_c" stoichiometry="1"
constant="true"/>
                  <speciesReference species="M_sbt__D_e" stoichiometry="1"
constant="true"/>
            </listOfReactants>
            <listOfProducts>
                  <speciesReference species="M_pyr_c" stoichiometry="1"
constant="true"/>
                  <speciesReference species="M_sbt6p_c" stoichiometry="1"
constant="true"/>
            </listOfProducts>
            <fbc:geneProductAssociation>
                  <fbc:and>
                        <fbc:geneProductRef fbc:geneProduct="SAUSA300_0983"/>
                        <fbc:geneProductRef fbc:geneProduct="SAUSA300_0984"/>
                  </fbc:and>
            </fbc:geneProductAssociation>
      </reaction>
```

```
      <reaction metaid="R_SDPDS" id="R_SDPDS" name="succinyl__Diaminopimelate
desuccinylase" reversible="false" fast="false" fbc:lowerFluxBound="irr_lb"
fbc:upperFluxBound="irr_ub">
            <listOfReactants>
                  <speciesReference species="M_h2o_c" stoichiometry="1"
constant="true"/>
                  <speciesReference species="M_sl26da_c" stoichiometry="1"
constant="true"/>
            </listOfReactants>
            <listOfProducts>
                  <speciesReference species="M_26dap__LL_c" stoichiometry="1"
constant="true"/>
                  <speciesReference species="M_succ_c" stoichiometry="1"
constant="true"/>
            </listOfProducts>
            <fbc:geneProductAssociation>
                  <fbc:or>
                        <fbc:geneProductRef fbc:geneProduct="SAUSA300_1697"/>
                        <fbc:geneProductRef fbc:geneProduct="SAUSA300_1976"/>
                  </fbc:or>
            </fbc:geneProductAssociation>
      </reaction>

      <reaction metaid="R_SERAT" id="R_SERAT" name="serine O-
acetyltransferase" reversible="true" fast="false" fbc:lowerFluxBound="rev_lb"
fbc:upperFluxBound="rev_ub">
            <listOfReactants>
                  <speciesReference species="M_accoa_c" stoichiometry="1"
constant="true"/>
                  <speciesReference species="M_ser__L_c" stoichiometry="1"
constant="true"/>
            </listOfReactants>
            <listOfProducts>
                  <speciesReference species="M_acser_c" stoichiometry="1"
constant="true"/>
                  <speciesReference species="M_coa_c" stoichiometry="1"
constant="true"/>
            </listOfProducts>
            <fbc:geneProductAssociation>
                  <fbc:geneProductRef fbc:geneProduct="SAUSA300_0514"/>
            </fbc:geneProductAssociation>
      </reaction>

      <reaction metaid="R_SERD_L" id="R_SERD_L" name="L-serine deaminase"
reversible="false" fast="false" fbc:lowerFluxBound="irr_lb"
fbc:upperFluxBound="irr_ub">
            <listOfReactants>
                  <speciesReference species="M_ser__L_c" stoichiometry="1"
constant="true"/>
            </listOfReactants>
            <listOfProducts>
                  <speciesReference species="M_nh4_c" stoichiometry="1"
constant="true"/>
                  <speciesReference species="M_pyr_c" stoichiometry="1"
constant="true"/>
            </listOfProducts>
            <fbc:geneProductAssociation>
```

```
                  <fbc:and>
                        <fbc:geneProductRef fbc:geneProduct="SAUSA300_2470"/>
                        <fbc:geneProductRef fbc:geneProduct="SAUSA300_2469"/>
                  </fbc:and>
            </fbc:geneProductAssociation>
      </reaction>

      <reaction metaid="R_SERD_Lr" id="R_SERD_Lr" name="L-serine deaminase
(rev)" reversible="false" fast="false" fbc:lowerFluxBound="irr_lb"
fbc:upperFluxBound="irr_ub">
            <listOfReactants>
                  <speciesReference species="M_ser__L_c" stoichiometry="1"
constant="true"/>
            </listOfReactants>
            <listOfProducts>
                  <speciesReference species="M_nh4_c" stoichiometry="1"
constant="true"/>
                  <speciesReference species="M_pyr_c" stoichiometry="1"
constant="true"/>
            </listOfProducts>
            <fbc:geneProductAssociation>
                  <fbc:and>
                        <fbc:geneProductRef fbc:geneProduct="SAUSA300_2470"/>
                        <fbc:geneProductRef fbc:geneProduct="SAUSA300_2469"/>
                  </fbc:and>
            </fbc:geneProductAssociation>
      </reaction>

      <reaction metaid="R_SERt2r" id="R_SERt2r" name="L-serine reversible
transport via proton symport" reversible="true" fast="false"
fbc:lowerFluxBound="rev_lb" fbc:upperFluxBound="rev_ub">
            <listOfReactants>
                  <speciesReference species="M_h_e" stoichiometry="1"
constant="true"/>
                  <speciesReference species="M_ser__L_e" stoichiometry="1"
constant="true"/>
            </listOfReactants>
            <listOfProducts>
                  <speciesReference species="M_h_c" stoichiometry="1"
constant="true"/>
                  <speciesReference species="M_ser__L_c" stoichiometry="1"
constant="true"/>
            </listOfProducts>
            <fbc:geneProductAssociation>
                  <fbc:or>
                        <fbc:geneProductRef fbc:geneProduct="SAUSA300_1642"/>
                        <fbc:geneProductRef fbc:geneProduct="SA451515_2543"/>
                  </fbc:or>
            </fbc:geneProductAssociation>
      </reaction>

      <reaction metaid="R_SERTRS" id="R_SERTRS" name="Seryl-tRNA synthetase"
reversible="false" fast="false" fbc:lowerFluxBound="irr_lb"
fbc:upperFluxBound="irr_ub">
            <listOfReactants>
                  <speciesReference species="M_atp_c" stoichiometry="1"
constant="true"/>
```

```
                  <speciesReference species="M_ser__L_c" stoichiometry="1"
constant="true"/>
                  <speciesReference species="M_trnaser_c" stoichiometry="1"
constant="true"/>
            </listOfReactants>
            <listOfProducts>
                  <speciesReference species="M_ppi_c" stoichiometry="1"
constant="true"/>
                  <speciesReference species="M_amp_c" stoichiometry="1"
constant="true"/>
                  <speciesReference species="M_sertrna_c" stoichiometry="1"
constant="true"/>
            </listOfProducts>
            <fbc:geneProductAssociation>
                  <fbc:geneProductRef fbc:geneProduct="SAUSA300_0009"/>
            </fbc:geneProductAssociation>
      </reaction>

      <reaction metaid="R_SHCHCS2" id="R_SHCHCS2" name="2-succinyl-6-hydroxy-
2,4-cyclohexadiene 1-carboxylate synthase" reversible="false" fast="false"
fbc:lowerFluxBound="irr_lb" fbc:upperFluxBound="irr_ub">
            <listOfReactants>
                  <speciesReference species="M_ichor_c" stoichiometry="1"
constant="true"/>
                  <speciesReference species="M_ssaltpp_c" stoichiometry="1"
constant="true"/>
            </listOfReactants>
            <listOfProducts>
                  <speciesReference species="M_2shchc_c" stoichiometry="1"
constant="true"/>
                  <speciesReference species="M_thmpp_c" stoichiometry="1"
constant="true"/>
                  <speciesReference species="M_pyr_c" stoichiometry="1"
constant="true"/>
            </listOfProducts>
            <fbc:geneProductAssociation>
                  <fbc:geneProductRef fbc:geneProduct="SAUSA300_0946"/>
            </fbc:geneProductAssociation>
      </reaction>

      <reaction metaid="R_SHCHD2" id="R_SHCHD2" name="sirohydrochlorin
dehydrogenase (NAD)" reversible="false" fast="false"
fbc:lowerFluxBound="irr_lb" fbc:upperFluxBound="irr_ub">
            <listOfReactants>
                  <speciesReference species="M_dscl_c" stoichiometry="1"
constant="true"/>
                  <speciesReference species="M_nad_c" stoichiometry="1"
constant="true"/>
            </listOfReactants>
            <listOfProducts>
                  <speciesReference species="M_h_c" stoichiometry="1"
constant="true"/>
                  <speciesReference species="M_nadh_c" stoichiometry="1"
constant="true"/>
                  <speciesReference species="M_scl_c" stoichiometry="1"
constant="true"/>
            </listOfProducts>
            <fbc:geneProductAssociation>
```

```
                    <fbc:or>
                        <fbc:geneProductRef fbc:geneProduct="SAUSA300_2553"/>
                        <fbc:geneProductRef fbc:geneProduct="SAUSA300_2344"/>
                    </fbc:or>
                </fbc:geneProductAssociation>
            </reaction>

            <reaction metaid="R_SHCHF" id="R_SHCHF" name="sirohydrochlorin
ferrochelatase" reversible="true" fast="false" fbc:lowerFluxBound="rev_lb"
fbc:upperFluxBound="rev_ub">
                <listOfReactants>
                    <speciesReference species="M_h_c" stoichiometry="2"
constant="true"/>
                    <speciesReference species="M_sheme_c" stoichiometry="1"
constant="true"/>
                </listOfReactants>
                <listOfProducts>
                    <speciesReference species="M_fe2_c" stoichiometry="1"
constant="true"/>
                    <speciesReference species="M_scl_c" stoichiometry="1"
constant="true"/>
                </listOfProducts>
                <fbc:geneProductAssociation>
                    <fbc:geneProductRef fbc:geneProduct="SAUSA300_2347"/>
                </fbc:geneProductAssociation>
            </reaction>

            <reaction metaid="R_SHK3Dr" id="R_SHK3Dr" name="shikimate
dehydrogenase" reversible="true" fast="false" fbc:lowerFluxBound="rev_lb"
fbc:upperFluxBound="rev_ub">
                <listOfReactants>
                    <speciesReference species="M_nadp_c" stoichiometry="1"
constant="true"/>
                    <speciesReference species="M_skm_c" stoichiometry="1"
constant="true"/>
                </listOfReactants>
                <listOfProducts>
                    <speciesReference species="M_h_c" stoichiometry="1"
constant="true"/>
                    <speciesReference species="M_nadph_c" stoichiometry="1"
constant="true"/>
                    <speciesReference species="M_3dhsk_c" stoichiometry="1"
constant="true"/>
                </listOfProducts>
                <fbc:geneProductAssociation>
                    <fbc:geneProductRef fbc:geneProduct="SAUSA300_1555"/>
                </fbc:geneProductAssociation>
            </reaction>

            <reaction metaid="R_SHKK" id="R_SHKK" name="shikimate kinase"
reversible="false" fast="false" fbc:lowerFluxBound="irr_lb"
fbc:upperFluxBound="irr_ub">
                <listOfReactants>
                    <speciesReference species="M_atp_c" stoichiometry="1"
constant="true"/>
                    <speciesReference species="M_skm_c" stoichiometry="1"
constant="true"/>
                </listOfReactants>
```

```
            <listOfProducts>
                  <speciesReference species="M_adp_c" stoichiometry="1"
constant="true"/>
                  <speciesReference species="M_skm5p_c" stoichiometry="1"
constant="true"/>
            </listOfProducts>
            <fbc:geneProductAssociation>
                  <fbc:geneProductRef fbc:geneProduct="SAUSA300_1499"/>
            </fbc:geneProductAssociation>
      </reaction>

      <reaction metaid="R_SHS1" id="R_SHS1" name="Siroheme synthsis"
reversible="true" fast="false" fbc:lowerFluxBound="rev_lb"
fbc:upperFluxBound="rev_ub">
            <listOfReactants>
                  <speciesReference species="M_fe2_c" stoichiometry="1"
constant="true"/>
                  <speciesReference species="M_amet_c" stoichiometry="2.0"
constant="true"/>
                  <speciesReference species="M_nad_c" stoichiometry="1"
constant="true"/>
                  <speciesReference species="M_uppg3_c" stoichiometry="1"
constant="true"/>
            </listOfReactants>
            <listOfProducts>
                  <speciesReference species="M_h_c" stoichiometry="5.0"
constant="true"/>
                  <speciesReference species="M_ahcys_c" stoichiometry="2.0"
constant="true"/>
                  <speciesReference species="M_sheme_c" stoichiometry="1"
constant="true"/>
                  <speciesReference species="M_nadh_c" stoichiometry="1"
constant="true"/>
            </listOfProducts>
            <fbc:geneProductAssociation>
                  <fbc:geneProductRef fbc:geneProduct="SAUSA300_2553"/>
            </fbc:geneProductAssociation>
      </reaction>

      <reaction metaid="R_SHSL1r" id="R_SHSL1r" name="O-succinylhomoserine
lyase reversible" reversible="true" fast="false" fbc:lowerFluxBound="rev_lb"
fbc:upperFluxBound="rev_ub">
            <listOfReactants>
                  <speciesReference species="M_cyst__L_c" stoichiometry="1"
constant="true"/>
                  <speciesReference species="M_succ_c" stoichiometry="1"
constant="true"/>
            </listOfReactants>
            <listOfProducts>
                  <speciesReference species="M_cys__L_c" stoichiometry="1"
constant="true"/>
                  <speciesReference species="M_suchms_c" stoichiometry="1"
constant="true"/>
            </listOfProducts>
            <fbc:geneProductAssociation>
                  <fbc:geneProductRef fbc:geneProduct="SAUSA300_0360"/>
            </fbc:geneProductAssociation>
      </reaction>
```

```
      <reaction metaid="R_SHSL4r" id="R_SHSL4r" name="O-succinylhomoserine
lyase (elimination), reversible" reversible="false" fast="false"
fbc:lowerFluxBound="irr_lb" fbc:upperFluxBound="irr_ub">
            <listOfReactants>
                  <speciesReference species="M_h2o_c" stoichiometry="1"
constant="true"/>
                  <speciesReference species="M_suchms_c" stoichiometry="1"
constant="true"/>
            </listOfReactants>
            <listOfProducts>
                  <speciesReference species="M_h_c" stoichiometry="1"
constant="true"/>
                  <speciesReference species="M_nh4_c" stoichiometry="1"
constant="true"/>
                  <speciesReference species="M_2obut_c" stoichiometry="1"
constant="true"/>
                  <speciesReference species="M_succ_c" stoichiometry="1"
constant="true"/>
            </listOfProducts>
            <fbc:geneProductAssociation>
                  <fbc:geneProductRef fbc:geneProduct="SAUSA300_0360"/>
            </fbc:geneProductAssociation>
      </reaction>

      <reaction metaid="R_SIALO" id="R_SIALO" name="salicylate NADH oxygen
oxidoreductase " reversible="false" fast="false" fbc:lowerFluxBound="irr_lb"
fbc:upperFluxBound="irr_ub">
            <listOfReactants>
                  <speciesReference species="M_h_c" stoichiometry="2"
constant="true"/>
                  <speciesReference species="M_o2_c" stoichiometry="1"
constant="true"/>
                  <speciesReference species="M_nadh_c" stoichiometry="1"
constant="true"/>
                  <speciesReference species="M_mcres_c" stoichiometry="1"
constant="true"/>
            </listOfReactants>
            <listOfProducts>
                  <speciesReference species="M_h2o_c" stoichiometry="1"
constant="true"/>
                  <speciesReference species="M_co2_c" stoichiometry="1"
constant="true"/>
                  <speciesReference species="M_nad_c" stoichiometry="1"
constant="true"/>
                  <speciesReference species="M_4mcat_c" stoichiometry="1"
constant="true"/>
            </listOfProducts>
            <fbc:geneProductAssociation>
                  <fbc:geneProductRef fbc:geneProduct="SAUSA300_2255"/>
            </fbc:geneProductAssociation>
      </reaction>

      <reaction metaid="R_SIAOO" id="R_SIAOO" name="salicylate NADH oxygen
oxidoreductase " reversible="false" fast="false" fbc:lowerFluxBound="irr_lb"
fbc:upperFluxBound="irr_ub">
            <listOfReactants>
```

```
                  <speciesReference species="M_h_c" stoichiometry="2"
constant="true"/>
                  <speciesReference species="M_o2_c" stoichiometry="1"
constant="true"/>
                  <speciesReference species="M_nadh_c" stoichiometry="1"
constant="true"/>
                  <speciesReference species="M_msial_c" stoichiometry="1"
constant="true"/>
            </listOfReactants>
            <listOfProducts>
                  <speciesReference species="M_h2o_c" stoichiometry="1"
constant="true"/>
                  <speciesReference species="M_co2_c" stoichiometry="1"
constant="true"/>
                  <speciesReference species="M_nad_c" stoichiometry="1"
constant="true"/>
                  <speciesReference species="M_3mcat_c" stoichiometry="1"
constant="true"/>
            </listOfProducts>
            <fbc:geneProductAssociation>
                  <fbc:geneProductRef fbc:geneProduct="SAUSA300_2255"/>
            </fbc:geneProductAssociation>
      </reaction>

      <reaction metaid="R_SMALO" id="R_SMALO" name="S-Malate acceptor
oxidoreductase " reversible="false" fast="false" fbc:lowerFluxBound="irr_lb"
fbc:upperFluxBound="irr_ub">
            <listOfReactants>
                  <speciesReference species="M_mal__L_c" stoichiometry="1"
constant="true"/>
                  <speciesReference species="M_fad_c" stoichiometry="1"
constant="true"/>
            </listOfReactants>
            <listOfProducts>
                  <speciesReference species="M_oaa_c" stoichiometry="1"
constant="true"/>
                  <speciesReference species="M_fadh2_c" stoichiometry="1"
constant="true"/>
            </listOfProducts>
            <fbc:geneProductAssociation>
                  <fbc:or>
                        <fbc:geneProductRef fbc:geneProduct="SAUSA300_2541"/>
                        <fbc:geneProductRef fbc:geneProduct="SAUSA300_2312"/>
                  </fbc:or>
            </fbc:geneProductAssociation>
      </reaction>

      <reaction metaid="R_SO4t2" id="R_SO4t2" name="sulfate transport in via
proton symport" reversible="true" fast="false" fbc:lowerFluxBound="rev_lb"
fbc:upperFluxBound="rev_ub">
            <listOfReactants>
                  <speciesReference species="M_h_e" stoichiometry="1"
constant="true"/>
                  <speciesReference species="M_so4_e" stoichiometry="1"
constant="true"/>
            </listOfReactants>
            <listOfProducts>
```

```
                  <speciesReference species="M_h_c" stoichiometry="1"
constant="true"/>
                  <speciesReference species="M_so4_c" stoichiometry="1"
constant="true"/>
            </listOfProducts>
      </reaction>

      <reaction metaid="R_SPMDabc" id="R_SPMDabc" name="spermidine transport
via ABC system" reversible="false" fast="false" fbc:lowerFluxBound="irr_lb"
fbc:upperFluxBound="irr_ub">
            <listOfReactants>
                  <speciesReference species="M_h2o_c" stoichiometry="1"
constant="true"/>
                  <speciesReference species="M_atp_c" stoichiometry="1"
constant="true"/>
                  <speciesReference species="M_spmd_e" stoichiometry="1"
constant="true"/>
            </listOfReactants>
            <listOfProducts>
                  <speciesReference species="M_h_c" stoichiometry="1"
constant="true"/>
                  <speciesReference species="M_pi_c" stoichiometry="1"
constant="true"/>
                  <speciesReference species="M_adp_c" stoichiometry="1"
constant="true"/>
                  <speciesReference species="M_spmd_c" stoichiometry="1"
constant="true"/>
            </listOfProducts>
            <fbc:geneProductAssociation>
                  <fbc:and>
                        <fbc:geneProductRef fbc:geneProduct="SAUSA300_0999"/>
                        <fbc:geneProductRef fbc:geneProduct="SAUSA300_1001"/>
                        <fbc:geneProductRef fbc:geneProduct="SAUSA300_1000"/>
                        <fbc:geneProductRef fbc:geneProduct="SAUSA300_1002"/>
                  </fbc:and>
            </fbc:geneProductAssociation>
      </reaction>

      <reaction metaid="R_SPMDAT1" id="R_SPMDAT1" name="Spermidine
acetyltransferase" reversible="false" fast="false"
fbc:lowerFluxBound="irr_lb" fbc:upperFluxBound="irr_ub">
            <listOfReactants>
                  <speciesReference species="M_accoa_c" stoichiometry="1"
constant="true"/>
                  <speciesReference species="M_spmd_c" stoichiometry="1"
constant="true"/>
            </listOfReactants>
            <listOfProducts>
                  <speciesReference species="M_h_c" stoichiometry="1"
constant="true"/>
                  <speciesReference species="M_coa_c" stoichiometry="1"
constant="true"/>
                  <speciesReference species="M_N1aspmd_c" stoichiometry="1"
constant="true"/>
            </listOfProducts>
            <fbc:geneProductAssociation>
                  <fbc:geneProductRef fbc:geneProduct="SAUSA300_0053"/>
            </fbc:geneProductAssociation>
```

```
      </reaction>

      <reaction metaid="R_SPMDAT2" id="R_SPMDAT2" name="Spermidine
acetyltransferase (N8)" reversible="false" fast="false"
fbc:lowerFluxBound="irr_lb" fbc:upperFluxBound="irr_ub">
            <listOfReactants>
                  <speciesReference species="M_accoa_c" stoichiometry="1"
constant="true"/>
                  <speciesReference species="M_spmd_c" stoichiometry="1"
constant="true"/>
            </listOfReactants>
            <listOfProducts>
                  <speciesReference species="M_h_c" stoichiometry="1"
constant="true"/>
                  <speciesReference species="M_coa_c" stoichiometry="1"
constant="true"/>
                  <speciesReference species="M_n8aspmd_c" stoichiometry="1"
constant="true"/>
            </listOfProducts>
            <fbc:geneProductAssociation>
                  <fbc:geneProductRef fbc:geneProduct="SAUSA300_0053"/>
            </fbc:geneProductAssociation>
      </reaction>

      <reaction metaid="R_SPODM" id="R_SPODM" name="superoxide dismutase"
reversible="false" fast="false" fbc:lowerFluxBound="irr_lb"
fbc:upperFluxBound="irr_ub">
            <listOfReactants>
                  <speciesReference species="M_o2s_c" stoichiometry="2"
constant="true"/>
            </listOfReactants>
            <listOfProducts>
                  <speciesReference species="M_o2_c" stoichiometry="1"
constant="true"/>
                  <speciesReference species="M_h2o2_c" stoichiometry="1"
constant="true"/>
            </listOfProducts>
            <fbc:geneProductAssociation>
                  <fbc:or>
                        <fbc:geneProductRef fbc:geneProduct="SAUSA300_0135"/>
                        <fbc:geneProductRef fbc:geneProduct="SAUSA300_1513"/>
                  </fbc:or>
            </fbc:geneProductAssociation>
      </reaction>

      <reaction metaid="R_SQDS" id="R_SQDS" name="rxn07468 "
reversible="false" fast="false" fbc:lowerFluxBound="irr_lb"
fbc:upperFluxBound="irr_ub">
            <listOfReactants>
                  <speciesReference species="M_dhsql_c" stoichiometry="1"
constant="true"/>
            </listOfReactants>
            <listOfProducts>
                  <speciesReference species="M_h_c" stoichiometry="6"
constant="true"/>
                  <speciesReference species="M_44dneu_c" stoichiometry="1"
constant="true"/>
            </listOfProducts>
```

```
			<fbc:geneProductAssociation>
				<fbc:geneProductRef fbc:geneProduct="SAUSA300_2498"/>
			</fbc:geneProductAssociation>
		</reaction>

		<reaction metaid="R_SQLS" id="R_SQLS" name="Squalene synthase"
reversible="false" fast="false" fbc:lowerFluxBound="irr_lb"
fbc:upperFluxBound="irr_ub">
			<listOfReactants>
				<speciesReference species="M_h_c" stoichiometry="1"
constant="true"/>
				<speciesReference species="M_nadph_c" stoichiometry="1"
constant="true"/>
				<speciesReference species="M_frdp_c" stoichiometry="2"
constant="true"/>
			</listOfReactants>
			<listOfProducts>
				<speciesReference species="M_nadp_c" stoichiometry="1"
constant="true"/>
				<speciesReference species="M_ppi_c" stoichiometry="2"
constant="true"/>
				<speciesReference species="M_sql_c" stoichiometry="1"
constant="true"/>
			</listOfProducts>
			<fbc:geneProductAssociation>
				<fbc:geneProductRef fbc:geneProduct="SAUSA300_2498"/>
			</fbc:geneProductAssociation>
		</reaction>

		<reaction metaid="R_SSALy" id="R_SSALy" name="succinate-semialdehyde
dehydrogenase (NADP)" reversible="false" fast="false"
fbc:lowerFluxBound="irr_lb" fbc:upperFluxBound="irr_ub">
			<listOfReactants>
				<speciesReference species="M_h2o_c" stoichiometry="1"
constant="true"/>
				<speciesReference species="M_nadp_c" stoichiometry="1"
constant="true"/>
				<speciesReference species="M_sucsal_c" stoichiometry="1"
constant="true"/>
			</listOfReactants>
			<listOfProducts>
				<speciesReference species="M_h_c" stoichiometry="2"
constant="true"/>
				<speciesReference species="M_nadph_c" stoichiometry="1"
constant="true"/>
				<speciesReference species="M_succ_c" stoichiometry="1"
constant="true"/>
			</listOfProducts>
			<fbc:geneProductAssociation>
				<fbc:geneProductRef fbc:geneProduct="SAUSA300_2076"/>
			</fbc:geneProductAssociation>
		</reaction>

		<reaction metaid="R_STEARCS" id="R_STEARCS" name="stearoyl-cardiolipin
synthase " reversible="true" fast="false" fbc:lowerFluxBound="rev_lb"
fbc:upperFluxBound="rev_ub">
			<listOfReactants>
```

```
                  <speciesReference species="M_pg180_c" stoichiometry="2" 
constant="true"/>
            &lt;/listOfReactants&gt;
            &lt;listOfProducts&gt;
                  <speciesReference species="M_glyc_c" stoichiometry="1" 
constant="true"/>
                  <speciesReference species="M_stecard_c" stoichiometry="1" 
constant="true"/>
            &lt;/listOfProducts&gt;
            &lt;fbc:geneProductAssociation&gt;
                  &lt;fbc:or&gt;
                        &lt;fbc:geneProductRef fbc:geneProduct="SAUSA300_2044"/&gt;
                        &lt;fbc:geneProductRef fbc:geneProduct="SAUSA300_1216"/&gt;
                  &lt;/fbc:or&gt;
            &lt;/fbc:geneProductAssociation&gt;
      &lt;/reaction&gt;

      <reaction metaid="R_STEARGLM" id="R_STEARGLM" name="stearoyl-UDP-
glucosyltransferase monoglucosyl " reversible="true" fast="false" 
fbc:lowerFluxBound="rev_lb" fbc:upperFluxBound="rev_ub">
            &lt;listOfReactants&gt;
                  <speciesReference species="M_udpg_c" stoichiometry="1" 
constant="true"/>
                  <speciesReference species="M_12dgr180_c" stoichiometry="1" 
constant="true"/>
            &lt;/listOfReactants&gt;
            &lt;listOfProducts&gt;
                  <speciesReference species="M_udp_c" stoichiometry="1" 
constant="true"/>
                  <speciesReference species="M_m12daglyc_c" stoichiometry="1" 
constant="true"/>
            &lt;/listOfProducts&gt;
            &lt;fbc:geneProductAssociation&gt;
                  &lt;fbc:geneProductRef fbc:geneProduct="SAUSA300_0918"/&gt;
            &lt;/fbc:geneProductAssociation&gt;
      &lt;/reaction&gt;

      <reaction metaid="R_STEARGLT" id="R_STEARGLT" name="stearoyl-UDP-
glucosyltransferase diglucosyl " reversible="true" fast="false" 
fbc:lowerFluxBound="rev_lb" fbc:upperFluxBound="rev_ub">
            &lt;listOfReactants&gt;
                  <speciesReference species="M_udpg_c" stoichiometry="1" 
constant="true"/>
                  <speciesReference species="M_m12daglyc_c" stoichiometry="1" 
constant="true"/>
            &lt;/listOfReactants&gt;
            &lt;listOfProducts&gt;
                  <speciesReference species="M_udp_c" stoichiometry="1" 
constant="true"/>
                  <speciesReference species="M_dglu12g_c" stoichiometry="1" 
constant="true"/>
            &lt;/listOfProducts&gt;
            &lt;fbc:geneProductAssociation&gt;
                  &lt;fbc:geneProductRef fbc:geneProduct="SAUSA300_0918"/&gt;
            &lt;/fbc:geneProductAssociation&gt;
      &lt;/reaction&gt;
```

```
      <reaction metaid="R_STEARLCS" id="R_STEARLCS"
name="stearoyl__Lipoteichoic acid synthesis n=24  unlinked  D-alanine
substituted " reversible="false" fast="false" fbc:lowerFluxBound="irr_lb"
fbc:upperFluxBound="irr_ub">
            <listOfReactants>
                  <speciesReference species="M_atp_c" stoichiometry="24"
constant="true"/>
                  <speciesReference species="M_ala__D_c" stoichiometry="24"
constant="true"/>
                  <speciesReference species="M_stear24u_c" stoichiometry="1"
constant="true"/>
            </listOfReactants>
            <listOfProducts>
                  <speciesReference species="M_ppi_c" stoichiometry="24"
constant="true"/>
                  <speciesReference species="M_amp_c" stoichiometry="24"
constant="true"/>
                  <speciesReference species="M_stear24ds_c" stoichiometry="1"
constant="true"/>
            </listOfProducts>
            <fbc:geneProductAssociation>
                  <fbc:geneProductRef fbc:geneProduct="SAUSA300_0838"/>
            </fbc:geneProductAssociation>
      </reaction>

      <reaction metaid="R_STEARLS" id="R_STEARLS"
name="stearoyl__Lipoteichoic acid synthesis n=24  linked  glucose substituted
" reversible="true" fast="false" fbc:lowerFluxBound="rev_lb"
fbc:upperFluxBound="rev_ub">
            <listOfReactants>
                  <speciesReference species="M_udpg_c" stoichiometry="24"
constant="true"/>
                  <speciesReference species="M_stear24u_c" stoichiometry="1"
constant="true"/>
            </listOfReactants>
            <listOfProducts>
                  <speciesReference species="M_udp_c" stoichiometry="24"
constant="true"/>
                  <speciesReference species="M_steart24s_c" stoichiometry="1"
constant="true"/>
            </listOfProducts>
            <fbc:geneProductAssociation>
                  <fbc:or>
                        <fbc:geneProductRef fbc:geneProduct="SAUSA300_0939"/>
                        <fbc:geneProductRef fbc:geneProduct="SAUSA300_0550"/>
                        <fbc:geneProductRef fbc:geneProduct="SAUSA300_0549"/>
                  </fbc:or>
            </fbc:geneProductAssociation>
      </reaction>

      <reaction metaid="R_STEARNS" id="R_STEARNS"
name="stearoyl__Lipoteichoic acid synthesis n=24  linked  N-acetylglucosamine
substituted " reversible="true" fast="false" fbc:lowerFluxBound="rev_lb"
fbc:upperFluxBound="rev_ub">
            <listOfReactants>
                  <speciesReference species="M_uacgam_c" stoichiometry="24"
constant="true"/>
```

```
                  <speciesReference species="M_stear24u_c" stoichiometry="1"
constant="true"/>
            </listOfReactants>
            <listOfProducts>
                  <speciesReference species="M_udp_c" stoichiometry="24"
constant="true"/>
                  <speciesReference species="M_stear24s_c" stoichiometry="1"
constant="true"/>
            </listOfProducts>
            <fbc:geneProductAssociation>
                  <fbc:geneProductRef fbc:geneProduct="SAUSA300_0731"/>
            </fbc:geneProductAssociation>
      </reaction>

      <reaction metaid="R_STEARPT" id="R_STEARPT" name="isoheptadecanoyl-
phosphatidate cytidylyltransferase " reversible="true" fast="false"
fbc:lowerFluxBound="rev_lb" fbc:upperFluxBound="rev_ub">
            <listOfReactants>
                  <speciesReference species="M_ctp_c" stoichiometry="1"
constant="true"/>
                  <speciesReference species="M_12dsgly3p_c" stoichiometry="1"
constant="true"/>
            </listOfReactants>
            <listOfProducts>
                  <speciesReference species="M_ppi_c" stoichiometry="1"
constant="true"/>
                  <speciesReference species="M_c12diigly_c" stoichiometry="1"
constant="true"/>
            </listOfProducts>
            <fbc:geneProductAssociation>
                  <fbc:geneProductRef fbc:geneProduct="SAUSA300_1154"/>
            </fbc:geneProductAssociation>
      </reaction>

      <reaction metaid="R_SUCBZL" id="R_SUCBZL" name="o-succinylbenzoate-CoA
ligase" reversible="false" fast="false" fbc:lowerFluxBound="irr_lb"
fbc:upperFluxBound="irr_ub">
            <listOfReactants>
                  <speciesReference species="M_h_c" stoichiometry="1"
constant="true"/>
                  <speciesReference species="M_sucbz_c" stoichiometry="1"
constant="true"/>
                  <speciesReference species="M_atp_c" stoichiometry="1"
constant="true"/>
                  <speciesReference species="M_coa_c" stoichiometry="1"
constant="true"/>
            </listOfReactants>
            <listOfProducts>
                  <speciesReference species="M_ppi_c" stoichiometry="1"
constant="true"/>
                  <speciesReference species="M_amp_c" stoichiometry="1"
constant="true"/>
                  <speciesReference species="M_sbzcoa_c" stoichiometry="1"
constant="true"/>
            </listOfProducts>
            <fbc:geneProductAssociation>
                  <fbc:geneProductRef fbc:geneProduct="SAUSA300_1737"/>
            </fbc:geneProductAssociation>
```

```
      &lt;/reaction&gt;

      <reaction metaid="R_SUCBZS" id="R_SUCBZS" name="O-succinylbenzoate-CoA
synthase" reversible="false" fast="false" fbc:lowerFluxBound="irr_lb"
fbc:upperFluxBound="irr_ub">
            &lt;listOfReactants&gt;
                  <speciesReference species="M_2shchc_c" stoichiometry="1"
constant="true"/>
            &lt;/listOfReactants&gt;
            &lt;listOfProducts&gt;
                  <speciesReference species="M_h2o_c" stoichiometry="1"
constant="true"/>
                  <speciesReference species="M_sucbz_c" stoichiometry="1"
constant="true"/>
            &lt;/listOfProducts&gt;
            &lt;fbc:geneProductAssociation&gt;
                  &lt;fbc:geneProductRef fbc:geneProduct="SAUSA300_1735"/&gt;
            &lt;/fbc:geneProductAssociation&gt;
      &lt;/reaction&gt;

      <reaction metaid="R_SUCCt8" id="R_SUCCt8" name="Na+/succinate
symporter" reversible="false" fast="false" fbc:lowerFluxBound="irr_lb"
fbc:upperFluxBound="irr_ub">
            &lt;listOfReactants&gt;
                  <speciesReference species="M_na1_e" stoichiometry="1"
constant="true"/>
                  <speciesReference species="M_succ_e" stoichiometry="1"
constant="true"/>
            &lt;/listOfReactants&gt;
            &lt;listOfProducts&gt;
                  <speciesReference species="M_na1_c" stoichiometry="1"
constant="true"/>
                  <speciesReference species="M_succ_c" stoichiometry="1"
constant="true"/>
            &lt;/listOfProducts&gt;
            &lt;fbc:geneProductAssociation&gt;
                  &lt;fbc:geneProductRef fbc:geneProduct="SAUSA300_1897"/&gt;
            &lt;/fbc:geneProductAssociation&gt;
      &lt;/reaction&gt;

      <reaction metaid="R_SUCD1" id="R_SUCD1" name="succinate dehydrogenase"
reversible="false" fast="false" fbc:lowerFluxBound="irr_lb"
fbc:upperFluxBound="irr_ub">
            &lt;listOfReactants&gt;
                  <speciesReference species="M_fad_c" stoichiometry="1"
constant="true"/>
                  <speciesReference species="M_succ_c" stoichiometry="1"
constant="true"/>
            &lt;/listOfReactants&gt;
            &lt;listOfProducts&gt;
                  <speciesReference species="M_fum_c" stoichiometry="1"
constant="true"/>
                  <speciesReference species="M_fadh2_c" stoichiometry="1"
constant="true"/>
            &lt;/listOfProducts&gt;
            &lt;fbc:geneProductAssociation&gt;
                  &lt;fbc:and&gt;
                        &lt;fbc:geneProductRef fbc:geneProduct="SAUSA300_1048"/&gt;
```

```
                        <fbc:geneProductRef fbc:geneProduct="SAUSA300_1047"/>
                        <fbc:geneProductRef fbc:geneProduct="SAUSA300_1046"/>
                  </fbc:and>
            </fbc:geneProductAssociation>
      </reaction>

      <reaction metaid="R_SUCOAS" id="R_SUCOAS" name="succinyl-CoA synthetase
(ADP-forming)" reversible="true" fast="false" fbc:lowerFluxBound="rev_lb"
fbc:upperFluxBound="rev_ub">
            <listOfReactants>
                  <speciesReference species="M_atp_c" stoichiometry="1"
constant="true"/>
                  <speciesReference species="M_coa_c" stoichiometry="1"
constant="true"/>
                  <speciesReference species="M_succ_c" stoichiometry="1"
constant="true"/>
            </listOfReactants>
            <listOfProducts>
                  <speciesReference species="M_pi_c" stoichiometry="1"
constant="true"/>
                  <speciesReference species="M_adp_c" stoichiometry="1"
constant="true"/>
                  <speciesReference species="M_succoa_c" stoichiometry="1"
constant="true"/>
            </listOfProducts>
            <fbc:geneProductAssociation>
                  <fbc:and>
                        <fbc:geneProductRef fbc:geneProduct="SAUSA300_1139"/>
                        <fbc:geneProductRef fbc:geneProduct="SAUSA300_1138"/>
                  </fbc:and>
            </fbc:geneProductAssociation>
      </reaction>

      <reaction metaid="R_SUCpts" id="R_SUCpts" name="sucrose transport via
PEP:Pyr PTS" reversible="true" fast="false" fbc:lowerFluxBound="rev_lb"
fbc:upperFluxBound="rev_ub">
            <listOfReactants>
                  <speciesReference species="M_pep_c" stoichiometry="1"
constant="true"/>
                  <speciesReference species="M_sucr_e" stoichiometry="1"
constant="true"/>
            </listOfReactants>
            <listOfProducts>
                  <speciesReference species="M_pyr_c" stoichiometry="1"
constant="true"/>
                  <speciesReference species="M_suc6p_c" stoichiometry="1"
constant="true"/>
            </listOfProducts>
            <fbc:geneProductAssociation>
                  <fbc:and>
                        <fbc:geneProductRef fbc:geneProduct="SAUSA300_0984"/>
                        <fbc:geneProductRef fbc:geneProduct="SAUSA300_0983"/>
                        <fbc:geneProductRef fbc:geneProduct="SAUSA300_2324"/>
                  </fbc:and>
            </fbc:geneProductAssociation>
      </reaction>
```

```
      <reaction metaid="R_SUCR" id="R_SUCR" name="sucrose hydrolyzing enzyme"
reversible="false" fast="false" fbc:lowerFluxBound="irr_lb"
fbc:upperFluxBound="irr_ub">
            <listOfReactants>
                  <speciesReference species="M_h2o_c" stoichiometry="1"
constant="true"/>
                  <speciesReference species="M_sucr_c" stoichiometry="1"
constant="true"/>
            </listOfReactants>
            <listOfProducts>
                  <speciesReference species="M_glc__D_c" stoichiometry="1"
constant="true"/>
                  <speciesReference species="M_fru_c" stoichiometry="1"
constant="true"/>
            </listOfProducts>
            <fbc:geneProductAssociation>
                  <fbc:geneProductRef fbc:geneProduct="SAUSA300_1994"/>
            </fbc:geneProductAssociation>
      </reaction>

      <reaction metaid="R_SULR" id="R_SULR" name="sulfite reductase (NADPH2)"
reversible="false" fast="false" fbc:lowerFluxBound="irr_lb"
fbc:upperFluxBound="irr_ub">
            <listOfReactants>
                  <speciesReference species="M_h_c" stoichiometry="4"
constant="true"/>
                  <speciesReference species="M_nadph_c" stoichiometry="3"
constant="true"/>
                  <speciesReference species="M_so3_c" stoichiometry="1"
constant="true"/>
            </listOfReactants>
            <listOfProducts>
                  <speciesReference species="M_h2o_c" stoichiometry="3"
constant="true"/>
                  <speciesReference species="M_nadp_c" stoichiometry="3"
constant="true"/>
                  <speciesReference species="M_h2s_c" stoichiometry="1"
constant="true"/>
            </listOfProducts>
            <fbc:geneProductAssociation>
                  <fbc:or>
                        <fbc:geneProductRef fbc:geneProduct="SAUSA300_2554"/>
                        <fbc:geneProductRef fbc:geneProduct="SA451515_2734"/>
                  </fbc:or>
            </fbc:geneProductAssociation>
      </reaction>

      <reaction metaid="R_t_actn__R" id="R_t_actn__R" name="transporter for
acetoin" reversible="true" fast="false" fbc:lowerFluxBound="rev_lb"
fbc:upperFluxBound="rev_ub">
            <listOfReactants>
                  <speciesReference species="M_actn__R_c" stoichiometry="1"
constant="true"/>
            </listOfReactants>
            <listOfProducts>
                  <speciesReference species="M_actn__R_e" stoichiometry="1"
constant="true"/>
            </listOfProducts>
```

```
      </reaction>

      <reaction metaid="R_t_glyclt" id="R_t_glyclt" name="Glycolate
transport" reversible="true" fast="false" fbc:lowerFluxBound="rev_lb"
fbc:upperFluxBound="rev_ub">
            <listOfReactants>
                  <speciesReference species="M_glyclt_c" stoichiometry="1"
constant="true"/>
            </listOfReactants>
            <listOfProducts>
                  <speciesReference species="M_glyclt_e" stoichiometry="1"
constant="true"/>
            </listOfProducts>
      </reaction>

      <reaction metaid="R_TACDex" id="R_TACDex" name="Teichoic acid D-alanine
substituted Exchange " reversible="true" fast="false"
fbc:lowerFluxBound="rev_lb" fbc:upperFluxBound="rev_ub">
            <listOfReactants>
                  <speciesReference species="M_pep_p1_c" stoichiometry="1"
constant="true"/>
                  <speciesReference species="M_pretechala_c"
stoichiometry="1" constant="true"/>
            </listOfReactants>
            <listOfProducts>
                  <speciesReference species="M_udcpp_c" stoichiometry="1"
constant="true"/>
                  <speciesReference species="M_glyteala_c" stoichiometry="1"
constant="true"/>
            </listOfProducts>
            <fbc:geneProductAssociation>
                  <fbc:and>
                        <fbc:or>
                              <fbc:geneProductRef
fbc:geneProduct="SAUSA300_1851"/>
                              <fbc:geneProductRef
fbc:geneProduct="SAUSA300_0625"/>
                        </fbc:or>
                        <fbc:or>
                              <fbc:geneProductRef
fbc:geneProduct="SAUSA300_0624"/>
                              <fbc:geneProductRef
fbc:geneProduct="SAUSA300_1852"/>
                        </fbc:or>
                  </fbc:and>
            </fbc:geneProductAssociation>
      </reaction>

      <reaction metaid="R_TAGO" id="R_TAGO" name="Tag O reaction for
initiation of teichoic acid" reversible="false" fast="false"
fbc:lowerFluxBound="irr_lb" fbc:upperFluxBound="irr_ub">
            <listOfReactants>
                  <speciesReference species="M_h2o_c" stoichiometry="2"
constant="true"/>
                  <speciesReference species="M_uacgam_c" stoichiometry="1"
constant="true"/>
            </listOfReactants>
            <listOfProducts>
```

```
                  <speciesReference species="M_h_c" stoichiometry="2"
constant="true"/>
                  <speciesReference species="M_pi_c" stoichiometry="1"
constant="true"/>
                  <speciesReference species="M_ump_c" stoichiometry="1"
constant="true"/>
                  <speciesReference species="M_acgam_c" stoichiometry="1"
constant="true"/>
            </listOfProducts>
            <fbc:geneProductAssociation>
                  <fbc:geneProductRef fbc:geneProduct="SAUSA300_0247"/>
            </fbc:geneProductAssociation>
      </reaction>

      <reaction metaid="R_TAHL" id="R_TAHL" name="Taurocholate amidohydrolase
" reversible="true" fast="false" fbc:lowerFluxBound="rev_lb"
fbc:upperFluxBound="rev_ub">
            <listOfReactants>
                  <speciesReference species="M_h2o_c" stoichiometry="1"
constant="true"/>
                  <speciesReference species="M_tchola_c" stoichiometry="1"
constant="true"/>
            </listOfReactants>
            <listOfProducts>
                  <speciesReference species="M_cholate_c" stoichiometry="1"
constant="true"/>
                  <speciesReference species="M_taur_c" stoichiometry="1"
constant="true"/>
            </listOfProducts>
            <fbc:geneProductAssociation>
                  <fbc:geneProductRef fbc:geneProduct="SAUSA300_0269"/>
            </fbc:geneProductAssociation>
      </reaction>

      <reaction metaid="R_TALA" id="R_TALA" name="transaldolase"
reversible="true" fast="false" fbc:lowerFluxBound="rev_lb"
fbc:upperFluxBound="rev_ub">
            <listOfReactants>
                  <speciesReference species="M_s7p_c" stoichiometry="1"
constant="true"/>
                  <speciesReference species="M_g3p_c" stoichiometry="1"
constant="true"/>
            </listOfReactants>
            <listOfProducts>
                  <speciesReference species="M_f6p_c" stoichiometry="1"
constant="true"/>
                  <speciesReference species="M_e4p_c" stoichiometry="1"
constant="true"/>
            </listOfProducts>
            <fbc:geneProductAssociation>
                  <fbc:geneProductRef fbc:geneProduct="SAUSA300_1725"/>
            </fbc:geneProductAssociation>
      </reaction>

      <reaction metaid="R_TDACPT" id="R_TDACPT" name="tetradecanoyl-ACP acyl-
carrier-protein transferase " reversible="true" fast="false"
fbc:lowerFluxBound="rev_lb" fbc:upperFluxBound="rev_ub">
            <listOfReactants>
```

```
                  <speciesReference species="M_coa_c" stoichiometry="1"
constant="true"/>
                  <speciesReference species="M_myrsACP_c" stoichiometry="1"
constant="true"/>
            </listOfReactants>
            <listOfProducts>
                  <speciesReference species="M_ACP_c" stoichiometry="1"
constant="true"/>
                  <speciesReference species="M_tdcoa_c" stoichiometry="1"
constant="true"/>
            </listOfProducts>
            <fbc:geneProductAssociation>
                  <fbc:geneProductRef fbc:geneProduct="SAUSA300_1123"/>
            </fbc:geneProductAssociation>
      </reaction>

      <reaction metaid="R_TECA1S" id="R_TECA1S" name="glycerol teichoic acid
synthesis (no sub, n=25)" reversible="false" fast="false"
fbc:lowerFluxBound="irr_lb" fbc:upperFluxBound="irr_ub">
            <listOfReactants>
                  <speciesReference species="M_cdpglyc_c" stoichiometry="25"
constant="true"/>
                  <speciesReference species="M_uacmam_c" stoichiometry="1"
constant="true"/>
                  <speciesReference species="M_acgam_c" stoichiometry="1"
constant="true"/>
            </listOfReactants>
            <listOfProducts>
                  <speciesReference species="M_h_c" stoichiometry="26"
constant="true"/>
                  <speciesReference species="M_cmp_c" stoichiometry="25"
constant="true"/>
                  <speciesReference species="M_udp_c" stoichiometry="1"
constant="true"/>
                  <speciesReference species="M_gtca1_c" stoichiometry="1"
constant="true"/>
            </listOfProducts>
            <fbc:geneProductAssociation>
                  <fbc:and>
                        <fbc:geneProductRef fbc:geneProduct="SAUSA300_0625"/>
                        <fbc:geneProductRef fbc:geneProduct="SAUSA300_0248"/>
                        <fbc:geneProductRef fbc:geneProduct="SAUSA300_0626"/>
                        <fbc:geneProductRef fbc:geneProduct="dltD"/>
                        <fbc:geneProductRef fbc:geneProduct="SAUSA300_0628"/>
                        <fbc:geneProductRef fbc:geneProduct="SAUSA300_0624"/>
                        <fbc:geneProductRef fbc:geneProduct="SAUSA300_0627"/>
                        <fbc:or>
                              <fbc:geneProductRef
fbc:geneProduct="SAUSA300_0549"/>
                              <fbc:geneProductRef
fbc:geneProduct="SAUSA300_0550"/>
                        </fbc:or>
                        <fbc:geneProductRef fbc:geneProduct="SAUSA300_0623"/>
                  </fbc:and>
            </fbc:geneProductAssociation>
      </reaction>
```

```
      <reaction metaid="R_TECA2S" id="R_TECA2S" name="glycerol techoic acid
synthesis (D-ala, n=25)" reversible="false" fast="false"
fbc:lowerFluxBound="irr_lb" fbc:upperFluxBound="irr_ub">
            <listOfReactants>
                  <speciesReference species="M_atp_c" stoichiometry="25"
constant="true"/>
                  <speciesReference species="M_cdpglyc_c" stoichiometry="25"
constant="true"/>
                  <speciesReference species="M_ala__D_c" stoichiometry="25"
constant="true"/>
                  <speciesReference species="M_uacmam_c" stoichiometry="1"
constant="true"/>
                  <speciesReference species="M_acgam_c" stoichiometry="1"
constant="true"/>
            </listOfReactants>
            <listOfProducts>
                  <speciesReference species="M_h_c" stoichiometry="26"
constant="true"/>
                  <speciesReference species="M_ppi_c" stoichiometry="25"
constant="true"/>
                  <speciesReference species="M_cmp_c" stoichiometry="25"
constant="true"/>
                  <speciesReference species="M_udp_c" stoichiometry="1"
constant="true"/>
                  <speciesReference species="M_amp_c" stoichiometry="25"
constant="true"/>
                  <speciesReference species="M_gtca2_c" stoichiometry="1"
constant="true"/>
            </listOfProducts>
            <fbc:geneProductAssociation>
                  <fbc:and>
                        <fbc:geneProductRef fbc:geneProduct="SAUSA300_0625"/>
                        <fbc:geneProductRef fbc:geneProduct="SAUSA300_0248"/>
                        <fbc:geneProductRef fbc:geneProduct="SAUSA300_0626"/>
                        <fbc:geneProductRef fbc:geneProduct="dltD"/>
                        <fbc:geneProductRef fbc:geneProduct="SAUSA300_0628"/>
                        <fbc:geneProductRef fbc:geneProduct="SAUSA300_0624"/>
                        <fbc:geneProductRef fbc:geneProduct="SAUSA300_0627"/>
                        <fbc:or>
                              <fbc:geneProductRef
fbc:geneProduct="SAUSA300_0549"/>
                              <fbc:geneProductRef
fbc:geneProduct="SAUSA300_0550"/>
                        </fbc:or>
                        <fbc:geneProductRef fbc:geneProduct="SAUSA300_0623"/>
                  </fbc:and>
            </fbc:geneProductAssociation>
      </reaction>

      <reaction metaid="R_TECA3S" id="R_TECA3S" name="glycerol teichoic acid
synthesis (n=25, glucose sub)" reversible="false" fast="false"
fbc:lowerFluxBound="irr_lb" fbc:upperFluxBound="irr_ub">
            <listOfReactants>
                  <speciesReference species="M_cdpglyc_c" stoichiometry="25"
constant="true"/>
                  <speciesReference species="M_udpg_c" stoichiometry="25"
constant="true"/>
```

```
                  <speciesReference species="M_uacmam_c" stoichiometry="1"
constant="true"/>
                  <speciesReference species="M_acgam_c" stoichiometry="1"
constant="true"/>
            </listOfReactants>
            <listOfProducts>
                  <speciesReference species="M_h_c" stoichiometry="51"
constant="true"/>
                  <speciesReference species="M_cmp_c" stoichiometry="25"
constant="true"/>
                  <speciesReference species="M_udp_c" stoichiometry="26"
constant="true"/>
                  <speciesReference species="M_gtca3_c" stoichiometry="1"
constant="true"/>
            </listOfProducts>
            <fbc:geneProductAssociation>
                  <fbc:and>
                        <fbc:geneProductRef fbc:geneProduct="SAUSA300_0625"/>
                        <fbc:geneProductRef fbc:geneProduct="SAUSA300_0248"/>
                        <fbc:geneProductRef fbc:geneProduct="SAUSA300_0626"/>
                        <fbc:geneProductRef fbc:geneProduct="dltD"/>
                        <fbc:geneProductRef fbc:geneProduct="SAUSA300_0628"/>
                        <fbc:geneProductRef fbc:geneProduct="SAUSA300_0624"/>
                        <fbc:geneProductRef fbc:geneProduct="SAUSA300_0627"/>
                        <fbc:or>
                              <fbc:geneProductRef
fbc:geneProduct="SAUSA300_0549"/>
                              <fbc:geneProductRef
fbc:geneProduct="SAUSA300_0550"/>
                        </fbc:or>
                        <fbc:geneProductRef fbc:geneProduct="SAUSA300_0623"/>
                  </fbc:and>
            </fbc:geneProductAssociation>
      </reaction>

      <reaction metaid="R_TECA4S" id="R_TECA4S" name="minor teichoic acid
synthesis (n=30)" reversible="true" fast="false" fbc:lowerFluxBound="rev_lb"
fbc:upperFluxBound="rev_ub">
            <listOfReactants>
                  <speciesReference species="M_h2o_c" stoichiometry="1"
constant="true"/>
                  <speciesReference species="M_udpg_c" stoichiometry="30"
constant="true"/>
                  <speciesReference species="M_udpacgal_c" stoichiometry="30"
constant="true"/>
            </listOfReactants>
            <listOfProducts>
                  <speciesReference species="M_udp_c" stoichiometry="30"
constant="true"/>
                  <speciesReference species="M_ump_c" stoichiometry="30"
constant="true"/>
                  <speciesReference species="M_tcam_c" stoichiometry="1"
constant="true"/>
            </listOfProducts>
            <fbc:geneProductAssociation>
                  <fbc:geneProductRef fbc:geneProduct="SAUSA300_0703"/>
            </fbc:geneProductAssociation>
      </reaction>
```

```
      <reaction metaid="R_TECAex" id="R_TECAex" name="Teichoic acid glucose
substituted Exchange " reversible="true" fast="false"
fbc:lowerFluxBound="rev_lb" fbc:upperFluxBound="rev_ub">
            &lt;listOfReactants&gt;
                  <speciesReference species="M_pep_p1_c" stoichiometry="1"
constant="true"/>
                  <speciesReference species="M_pren45glc_c" stoichiometry="1"
constant="true"/>
            &lt;/listOfReactants&gt;
            &lt;listOfProducts&gt;
                  <speciesReference species="M_udcpp_c" stoichiometry="1"
constant="true"/>
                  <speciesReference species="M_glytglc_c" stoichiometry="1"
constant="true"/>
            &lt;/listOfProducts&gt;
            &lt;fbc:geneProductAssociation&gt;
                  &lt;fbc:and&gt;
                        &lt;fbc:or&gt;
                              <fbc:geneProductRef
fbc:geneProduct="SAUSA300_1851"/>
                              <fbc:geneProductRef
fbc:geneProduct="SAUSA300_0625"/>
                        &lt;/fbc:or&gt;
                        &lt;fbc:or&gt;
                              <fbc:geneProductRef
fbc:geneProduct="SAUSA300_0624"/>
                              <fbc:geneProductRef
fbc:geneProduct="SAUSA300_1852"/>
                        &lt;/fbc:or&gt;
                  &lt;/fbc:and&gt;
            &lt;/fbc:geneProductAssociation&gt;
      &lt;/reaction&gt;

      <reaction metaid="R_TECHUex" id="R_TECHUex" name="Teichoic acid
unsubstituted Exchange " reversible="true" fast="false"
fbc:lowerFluxBound="rev_lb" fbc:upperFluxBound="rev_ub">
            &lt;listOfReactants&gt;
                  <speciesReference species="M_pren45_c" stoichiometry="1"
constant="true"/>
                  <speciesReference species="M_pep_p1_c" stoichiometry="1"
constant="true"/>
            &lt;/listOfReactants&gt;
            &lt;listOfProducts&gt;
                  <speciesReference species="M_udcpp_c" stoichiometry="1"
constant="true"/>
                  <speciesReference species="M_glytu_c" stoichiometry="1"
constant="true"/>
            &lt;/listOfProducts&gt;
            &lt;fbc:geneProductAssociation&gt;
                  &lt;fbc:and&gt;
                        &lt;fbc:or&gt;
                              <fbc:geneProductRef
fbc:geneProduct="SAUSA300_1851"/>
                              <fbc:geneProductRef
fbc:geneProduct="SAUSA300_0625"/>
                        &lt;/fbc:or&gt;
                        &lt;fbc:or&gt;
```

```
                              <fbc:geneProductRef
fbc:geneProduct="SAUSA300_0624"/>
                              <fbc:geneProductRef
fbc:geneProduct="SAUSA300_1852"/>
                        </fbc:or>
                  </fbc:and>
            </fbc:geneProductAssociation>
      </reaction>

      <reaction metaid="R_TGBPA" id="R_TGBPA" name="Tagatose-bisphosphate
aldolase" reversible="true" fast="false" fbc:lowerFluxBound="rev_lb"
fbc:upperFluxBound="rev_ub">
            <listOfReactants>
                  <speciesReference species="M_tagdp__D_c" stoichiometry="1"
constant="true"/>
            </listOfReactants>
            <listOfProducts>
                  <speciesReference species="M_dhap_c" stoichiometry="1"
constant="true"/>
                  <speciesReference species="M_g3p_c" stoichiometry="1"
constant="true"/>
            </listOfProducts>
            <fbc:geneProductAssociation>
                  <fbc:geneProductRef fbc:geneProduct="SAUSA300_2152"/>
            </fbc:geneProductAssociation>
      </reaction>

      <reaction metaid="R_THDPS" id="R_THDPS" name="tetrahydrodipicolinate
succinylase" reversible="false" fast="false" fbc:lowerFluxBound="irr_lb"
fbc:upperFluxBound="irr_ub">
            <listOfReactants>
                  <speciesReference species="M_h2o_c" stoichiometry="1"
constant="true"/>
                  <speciesReference species="M_thdp_c" stoichiometry="1"
constant="true"/>
                  <speciesReference species="M_succoa_c" stoichiometry="1"
constant="true"/>
            </listOfReactants>
            <listOfProducts>
                  <speciesReference species="M_coa_c" stoichiometry="1"
constant="true"/>
                  <speciesReference species="M_sl2a6o_c" stoichiometry="1"
constant="true"/>
            </listOfProducts>
            <fbc:geneProductAssociation>
                  <fbc:geneProductRef fbc:geneProduct="SAUSA300_1290"/>
            </fbc:geneProductAssociation>
      </reaction>

      <reaction metaid="R_THFGLUS" id="R_THFGLUS" name="Tetrahydrofolate:L-
glutamate gamma__Ligase (ADP-forming)" reversible="false" fast="false"
fbc:lowerFluxBound="irr_lb" fbc:upperFluxBound="irr_ub">
            <listOfReactants>
                  <speciesReference species="M_glu__L_c" stoichiometry="1"
constant="true"/>
                  <speciesReference species="M_atp_c" stoichiometry="1"
constant="true"/>
```

```
                  <speciesReference species="M_thf_c" stoichiometry="1"
constant="true"/>
            </listOfReactants>
            <listOfProducts>
                  <speciesReference species="M_h_c" stoichiometry="1"
constant="true"/>
                  <speciesReference species="M_pi_c" stoichiometry="1"
constant="true"/>
                  <speciesReference species="M_adp_c" stoichiometry="1"
constant="true"/>
                  <speciesReference species="M_thfglu_c" stoichiometry="1"
constant="true"/>
            </listOfProducts>
            <fbc:geneProductAssociation>
                  <fbc:geneProductRef fbc:geneProduct="SAUSA300_1610"/>
            </fbc:geneProductAssociation>
      </reaction>

      <reaction metaid="R_THIORDXi" id="R_THIORDXi" name="hydrogen peroxide
reductase (thioredoxin)" reversible="false" fast="false"
fbc:lowerFluxBound="irr_lb" fbc:upperFluxBound="irr_ub">
            <listOfReactants>
                  <speciesReference species="M_trdrd_c" stoichiometry="1"
constant="true"/>
                  <speciesReference species="M_h2o2_c" stoichiometry="1"
constant="true"/>
            </listOfReactants>
            <listOfProducts>
                  <speciesReference species="M_h2o_c" stoichiometry="2"
constant="true"/>
                  <speciesReference species="M_trdox_c" stoichiometry="1"
constant="true"/>
            </listOfProducts>
            <fbc:geneProductAssociation>
                  <fbc:or>
                        <fbc:geneProductRef fbc:geneProduct="SAUSA300_0795"/>
                        <fbc:geneProductRef fbc:geneProduct="SAUSA300_0789"/>
                        <fbc:geneProductRef fbc:geneProduct="SAUSA300_2474"/>
                        <fbc:geneProductRef fbc:geneProduct="SAUSA300_1909"/>
                        <fbc:geneProductRef fbc:geneProduct="SAUSA300_1044"/>
                  </fbc:or>
            </fbc:geneProductAssociation>
      </reaction>

      <reaction metaid="R_THMabc" id="R_THMabc" name="thiamine transport via
ABC system" reversible="false" fast="false" fbc:lowerFluxBound="irr_lb"
fbc:upperFluxBound="irr_ub">
            <listOfReactants>
                  <speciesReference species="M_h2o_c" stoichiometry="1"
constant="true"/>
                  <speciesReference species="M_atp_c" stoichiometry="1"
constant="true"/>
                  <speciesReference species="M_thm_e" stoichiometry="1"
constant="true"/>
            </listOfReactants>
            <listOfProducts>
                  <speciesReference species="M_h_c" stoichiometry="1"
constant="true"/>
```

```
                  <speciesReference species="M_pi_c" stoichiometry="1"
constant="true"/>
                  <speciesReference species="M_adp_c" stoichiometry="1"
constant="true"/>
                  <speciesReference species="M_thm_c" stoichiometry="1"
constant="true"/>
            &lt;/listOfProducts&gt;
            &lt;fbc:geneProductAssociation&gt;
                  &lt;fbc:or&gt;
                        &lt;fbc:and&gt;
                              &lt;fbc:or&gt;
                                    <fbc:geneProductRef
fbc:geneProduct="SAUSA300_0977"/>
                                    <fbc:geneProductRef
fbc:geneProduct="SAUSA300_2616"/>
                              &lt;/fbc:or&gt;
                              &lt;fbc:or&gt;
                                    <fbc:geneProductRef
fbc:geneProduct="SAUSA300_2617"/>
                                    <fbc:geneProductRef
fbc:geneProduct="SAUSA300_0978"/>
                              &lt;/fbc:or&gt;
                              <fbc:geneProductRef
fbc:geneProduct="SAUSA300_0979"/>
                        &lt;/fbc:and&gt;
                        &lt;fbc:and&gt;
                              &lt;fbc:or&gt;
                                    <fbc:geneProductRef
fbc:geneProduct="SAUSA300_2176"/>
                                    <fbc:geneProductRef
fbc:geneProduct="SAUSA300_2175"/>
                              &lt;/fbc:or&gt;
                              <fbc:geneProductRef
fbc:geneProduct="SAUSA300_2174"/>
                        &lt;/fbc:and&gt;
                  &lt;/fbc:or&gt;
            &lt;/fbc:geneProductAssociation&gt;
      &lt;/reaction&gt;

      <reaction metaid="R_THMDt2r" id="R_THMDt2r" name="thymidine transport
in via proton symport, reversible" reversible="false" fast="false"
fbc:lowerFluxBound="irr_lb" fbc:upperFluxBound="irr_ub">
            &lt;listOfReactants&gt;
                  <speciesReference species="M_h_e" stoichiometry="1"
constant="true"/>
                  <speciesReference species="M_thymd_e" stoichiometry="1"
constant="true"/>
            &lt;/listOfReactants&gt;
            &lt;listOfProducts&gt;
                  <speciesReference species="M_h_c" stoichiometry="1"
constant="true"/>
                  <speciesReference species="M_thymd_c" stoichiometry="1"
constant="true"/>
            &lt;/listOfProducts&gt;
            &lt;fbc:geneProductAssociation&gt;
                  &lt;fbc:geneProductRef fbc:geneProduct="SAUSA300_0506"/&gt;
            &lt;/fbc:geneProductAssociation&gt;
      &lt;/reaction&gt;
```

```
      <reaction metaid="R_THMDt4" id="R_THMDt4" name="thymidine transport in
via sodium symport" reversible="false" fast="false"
fbc:lowerFluxBound="irr_lb" fbc:upperFluxBound="irr_ub">
            <listOfReactants>
                  <speciesReference species="M_na1_e" stoichiometry="1"
constant="true"/>
                  <speciesReference species="M_thymd_e" stoichiometry="1"
constant="true"/>
            </listOfReactants>
            <listOfProducts>
                  <speciesReference species="M_na1_c" stoichiometry="1"
constant="true"/>
                  <speciesReference species="M_thymd_c" stoichiometry="1"
constant="true"/>
            </listOfProducts>
            <fbc:geneProductAssociation>
                  <fbc:or>
                        <fbc:geneProductRef fbc:geneProduct="SAUSA300_0313"/>
                        <fbc:geneProductRef fbc:geneProduct="SAUSA300_0631"/>
                        <fbc:geneProductRef fbc:geneProduct="SAUSA300_0506"/>
                  </fbc:or>
            </fbc:geneProductAssociation>
      </reaction>

      <reaction metaid="R_THRAr" id="R_THRAr" name="Threonine Aldolase"
reversible="true" fast="false" fbc:lowerFluxBound="rev_lb"
fbc:upperFluxBound="rev_ub">
            <listOfReactants>
                  <speciesReference species="M_thr__L_c" stoichiometry="1"
constant="true"/>
            </listOfReactants>
            <listOfProducts>
                  <speciesReference species="M_acald_c" stoichiometry="1"
constant="true"/>
                  <speciesReference species="M_gly_c" stoichiometry="1"
constant="true"/>
            </listOfProducts>
            <fbc:geneProductAssociation>
                  <fbc:geneProductRef fbc:geneProduct="SAUSA300_1214"/>
            </fbc:geneProductAssociation>
      </reaction>

      <reaction metaid="R_THRD" id="R_THRD" name="L-threonine dehydrogenase"
reversible="false" fast="false" fbc:lowerFluxBound="irr_lb"
fbc:upperFluxBound="irr_ub">
            <listOfReactants>
                  <speciesReference species="M_nad_c" stoichiometry="1"
constant="true"/>
                  <speciesReference species="M_thr__L_c" stoichiometry="1"
constant="true"/>
            </listOfReactants>
            <listOfProducts>
                  <speciesReference species="M_h_c" stoichiometry="1"
constant="true"/>
                  <speciesReference species="M_nadh_c" stoichiometry="1"
constant="true"/>
```

```
                  <speciesReference species="M_2aobut_c" stoichiometry="1"
constant="true"/>
            </listOfProducts>
            <fbc:geneProductAssociation>
                  <fbc:geneProductRef fbc:geneProduct="SAUSA300_0538"/>
            </fbc:geneProductAssociation>
      </reaction>

      <reaction metaid="R_THRD_L" id="R_THRD_L" name="L-threonine deaminase"
reversible="false" fast="false" fbc:lowerFluxBound="irr_lb"
fbc:upperFluxBound="irr_ub">
            <listOfReactants>
                  <speciesReference species="M_thr__L_c" stoichiometry="1"
constant="true"/>
            </listOfReactants>
            <listOfProducts>
                  <speciesReference species="M_nh4_c" stoichiometry="1"
constant="true"/>
                  <speciesReference species="M_2obut_c" stoichiometry="1"
constant="true"/>
            </listOfProducts>
            <fbc:geneProductAssociation>
                  <fbc:or>
                        <fbc:geneProductRef fbc:geneProduct="SAUSA300_1330"/>
                        <fbc:geneProductRef fbc:geneProduct="SAUSA300_2014"/>
                  </fbc:or>
            </fbc:geneProductAssociation>
      </reaction>

      <reaction metaid="R_THRS" id="R_THRS" name="threonine synthase"
reversible="false" fast="false" fbc:lowerFluxBound="irr_lb"
fbc:upperFluxBound="irr_ub">
            <listOfReactants>
                  <speciesReference species="M_h2o_c" stoichiometry="1"
constant="true"/>
                  <speciesReference species="M_phom_c" stoichiometry="1"
constant="true"/>
            </listOfReactants>
            <listOfProducts>
                  <speciesReference species="M_h_c" stoichiometry="1"
constant="true"/>
                  <speciesReference species="M_pi_c" stoichiometry="1"
constant="true"/>
                  <speciesReference species="M_thr__L_c" stoichiometry="1"
constant="true"/>
            </listOfProducts>
            <fbc:geneProductAssociation>
                  <fbc:geneProductRef fbc:geneProduct="SAUSA300_1227"/>
            </fbc:geneProductAssociation>
      </reaction>

      <reaction metaid="R_THRt2" id="R_THRt2" name="L-threonine transport in
via proton symport" reversible="false" fast="false"
fbc:lowerFluxBound="irr_lb" fbc:upperFluxBound="irr_ub">
            <listOfReactants>
                  <speciesReference species="M_h_e" stoichiometry="1"
constant="true"/>
```

```
                  <speciesReference species="M_thr__L_e" stoichiometry="1"
constant="true"/>
            </listOfReactants>
            <listOfProducts>
                  <speciesReference species="M_h_c" stoichiometry="1"
constant="true"/>
                  <speciesReference species="M_thr__L_c" stoichiometry="1"
constant="true"/>
            </listOfProducts>
            <fbc:geneProductAssociation>
                  <fbc:geneProductRef fbc:geneProduct="SAUSA300_1642"/>
            </fbc:geneProductAssociation>
      </reaction>

      <reaction metaid="R_THRTRS" id="R_THRTRS" name="Threonyl-tRNA
synthetase" reversible="false" fast="false" fbc:lowerFluxBound="irr_lb"
fbc:upperFluxBound="irr_ub">
            <listOfReactants>
                  <speciesReference species="M_atp_c" stoichiometry="1"
constant="true"/>
                  <speciesReference species="M_thr__L_c" stoichiometry="1"
constant="true"/>
                  <speciesReference species="M_trnathr_c" stoichiometry="1"
constant="true"/>
            </listOfReactants>
            <listOfProducts>
                  <speciesReference species="M_ppi_c" stoichiometry="1"
constant="true"/>
                  <speciesReference species="M_amp_c" stoichiometry="1"
constant="true"/>
                  <speciesReference species="M_thrtrna_c" stoichiometry="1"
constant="true"/>
            </listOfProducts>
            <fbc:geneProductAssociation>
                  <fbc:geneProductRef fbc:geneProduct="SAUSA300_1629"/>
            </fbc:geneProductAssociation>
      </reaction>

      <reaction metaid="R_THYMt3pp" id="R_THYMt3pp" name="thymine transport
out via proton antiport (periplasm)" reversible="false" fast="false"
fbc:lowerFluxBound="irr_lb" fbc:upperFluxBound="irr_ub">
            <listOfReactants>
                  <speciesReference species="M_h_p" stoichiometry="1"
constant="true"/>
                  <speciesReference species="M_thym_c" stoichiometry="1"
constant="true"/>
            </listOfReactants>
            <listOfProducts>
                  <speciesReference species="M_h_c" stoichiometry="1"
constant="true"/>
                  <speciesReference species="M_thym_p" stoichiometry="1"
constant="true"/>
            </listOfProducts>
      </reaction>

      <reaction metaid="R_THYMtex" id="R_THYMtex" name="thymine transport via
diffusion (extracellular to periplasm)" reversible="true" fast="false"
fbc:lowerFluxBound="rev_lb" fbc:upperFluxBound="rev_ub">
```

```
            <listOfReactants>
                  <speciesReference species="M_thym_e" stoichiometry="1"
constant="true"/>
            </listOfReactants>
            <listOfProducts>
                  <speciesReference species="M_thym_p" stoichiometry="1"
constant="true"/>
            </listOfProducts>
      </reaction>

      <reaction metaid="R_THZPSN" id="R_THZPSN" name="thiazole phosphate
synthesis" reversible="false" fast="false" fbc:lowerFluxBound="irr_lb"
fbc:upperFluxBound="irr_ub">
            <listOfReactants>
                  <speciesReference species="M_atp_c" stoichiometry="1"
constant="true"/>
                  <speciesReference species="M_cys__L_c" stoichiometry="1"
constant="true"/>
                  <speciesReference species="M_tyr__L_c" stoichiometry="1"
constant="true"/>
                  <speciesReference species="M_dxyl5p_c" stoichiometry="1"
constant="true"/>
            </listOfReactants>
            <listOfProducts>
                  <speciesReference species="M_h2o_c" stoichiometry="1"
constant="true"/>
                  <speciesReference species="M_ppi_c" stoichiometry="1"
constant="true"/>
                  <speciesReference species="M_co2_c" stoichiometry="1"
constant="true"/>
                  <speciesReference species="M_ala__L_c" stoichiometry="1"
constant="true"/>
                  <speciesReference species="M_amp_c" stoichiometry="1"
constant="true"/>
                  <speciesReference species="M_4mpetz_c" stoichiometry="1"
constant="true"/>
                  <speciesReference species="M_4hba_c" stoichiometry="1"
constant="true"/>
            </listOfProducts>
            <fbc:geneProductAssociation>
                  <fbc:or>
                        <fbc:geneProductRef fbc:geneProduct="SAUSA300_1662"/>
                        <fbc:geneProductRef fbc:geneProduct="SAUSA300_1579"/>
                  </fbc:or>
            </fbc:geneProductAssociation>
      </reaction>

      <reaction metaid="R_TKT1" id="R_TKT1" name="transketolase"
reversible="true" fast="false" fbc:lowerFluxBound="rev_lb"
fbc:upperFluxBound="rev_ub">
            <listOfReactants>
                  <speciesReference species="M_s7p_c" stoichiometry="1"
constant="true"/>
                  <speciesReference species="M_g3p_c" stoichiometry="1"
constant="true"/>
            </listOfReactants>
            <listOfProducts>
```

```
                  <speciesReference species="M_r5p_c" stoichiometry="1"
constant="true"/>
                  <speciesReference species="M_xu5p__D_c" stoichiometry="1"
constant="true"/>
            </listOfProducts>
            <fbc:geneProductAssociation>
                  <fbc:geneProductRef fbc:geneProduct="SAUSA300_1239"/>
            </fbc:geneProductAssociation>
      </reaction>

      <reaction metaid="R_TKT2" id="R_TKT2" name="transketolase"
reversible="true" fast="false" fbc:lowerFluxBound="rev_lb"
fbc:upperFluxBound="rev_ub">
            <listOfReactants>
                  <speciesReference species="M_f6p_c" stoichiometry="1"
constant="true"/>
                  <speciesReference species="M_g3p_c" stoichiometry="1"
constant="true"/>
            </listOfReactants>
            <listOfProducts>
                  <speciesReference species="M_xu5p__D_c" stoichiometry="1"
constant="true"/>
                  <speciesReference species="M_e4p_c" stoichiometry="1"
constant="true"/>
            </listOfProducts>
            <fbc:geneProductAssociation>
                  <fbc:geneProductRef fbc:geneProduct="SAUSA300_1239"/>
            </fbc:geneProductAssociation>
      </reaction>

      <reaction metaid="R_TMDK1" id="R_TMDK1" name="thymidine kinase
(ATP:thymidine)" reversible="false" fast="false" fbc:lowerFluxBound="irr_lb"
fbc:upperFluxBound="irr_ub">
            <listOfReactants>
                  <speciesReference species="M_atp_c" stoichiometry="1"
constant="true"/>
                  <speciesReference species="M_thymd_c" stoichiometry="1"
constant="true"/>
            </listOfReactants>
            <listOfProducts>
                  <speciesReference species="M_adp_c" stoichiometry="1"
constant="true"/>
                  <speciesReference species="M_dtmp_c" stoichiometry="1"
constant="true"/>
            </listOfProducts>
            <fbc:geneProductAssociation>
                  <fbc:geneProductRef fbc:geneProduct="SAUSA300_2073"/>
            </fbc:geneProductAssociation>
      </reaction>

      <reaction metaid="R_TMDPK" id="R_TMDPK" name="thiamine diphosphokinase"
reversible="false" fast="false" fbc:lowerFluxBound="irr_lb"
fbc:upperFluxBound="irr_ub">
            <listOfReactants>
                  <speciesReference species="M_atp_c" stoichiometry="1"
constant="true"/>
                  <speciesReference species="M_thm_c" stoichiometry="1"
constant="true"/>
```

```
            </listOfReactants>
            <listOfProducts>
                  <speciesReference species="M_thmpp_c" stoichiometry="1"
constant="true"/>
                  <speciesReference species="M_amp_c" stoichiometry="1"
constant="true"/>
            </listOfProducts>
            <fbc:geneProductAssociation>
                  <fbc:geneProductRef fbc:geneProduct="SAUSA300_1116"/>
            </fbc:geneProductAssociation>
      </reaction>

      <reaction metaid="R_TMDPP" id="R_TMDPP" name="thymidine phosphorylase"
reversible="true" fast="false" fbc:lowerFluxBound="rev_lb"
fbc:upperFluxBound="rev_ub">
            <listOfReactants>
                  <speciesReference species="M_pi_c" stoichiometry="1"
constant="true"/>
                  <speciesReference species="M_thymd_c" stoichiometry="1"
constant="true"/>
            </listOfReactants>
            <listOfProducts>
                  <speciesReference species="M_2dr1p_c" stoichiometry="1"
constant="true"/>
                  <speciesReference species="M_thym_c" stoichiometry="1"
constant="true"/>
            </listOfProducts>
            <fbc:geneProductAssociation>
                  <fbc:geneProductRef fbc:geneProduct="SAUSA300_2089"/>
            </fbc:geneProductAssociation>
      </reaction>

      <reaction metaid="R_TMDS" id="R_TMDS" name="thymidylate synthase"
reversible="false" fast="false" fbc:lowerFluxBound="irr_lb"
fbc:upperFluxBound="irr_ub">
            <listOfReactants>
                  <speciesReference species="M_mlthf_c" stoichiometry="1"
constant="true"/>
                  <speciesReference species="M_dump_c" stoichiometry="1"
constant="true"/>
            </listOfReactants>
            <listOfProducts>
                  <speciesReference species="M_dhf_c" stoichiometry="1"
constant="true"/>
                  <speciesReference species="M_dtmp_c" stoichiometry="1"
constant="true"/>
            </listOfProducts>
            <fbc:geneProductAssociation>
                  <fbc:geneProductRef fbc:geneProduct="SAUSA300_1320"/>
            </fbc:geneProductAssociation>
      </reaction>

      <reaction metaid="R_TMPPP" id="R_TMPPP" name="thiamine-phosphate
diphosphorylase" reversible="false" fast="false" fbc:lowerFluxBound="irr_lb"
fbc:upperFluxBound="irr_ub">
            <listOfReactants>
                  <speciesReference species="M_h_c" stoichiometry="1"
constant="true"/>
```

```
            <speciesReference species="M_2mahmp_c" stoichiometry="1"
constant="true"/>
            <speciesReference species="M_4mpetz_c" stoichiometry="1"
constant="true"/>
        &lt;/listOfReactants&gt;
        &lt;listOfProducts&gt;
            <speciesReference species="M_ppi_c" stoichiometry="1"
constant="true"/>
            <speciesReference species="M_thmmp_c" stoichiometry="1"
constant="true"/>
        &lt;/listOfProducts&gt;
        &lt;fbc:geneProductAssociation&gt;
            &lt;fbc:geneProductRef fbc:geneProduct="SAUSA300_2047"/&gt;
        &lt;/fbc:geneProductAssociation&gt;
    &lt;/reaction&gt;

    <reaction metaid="R_TPI" id="R_TPI" name="triose-phosphate isomerase"
reversible="true" fast="false" fbc:lowerFluxBound="rev_lb"
fbc:upperFluxBound="rev_ub">
        &lt;listOfReactants&gt;
            <speciesReference species="M_g3p_c" stoichiometry="1"
constant="true"/>
        &lt;/listOfReactants&gt;
        &lt;listOfProducts&gt;
            <speciesReference species="M_dhap_c" stoichiometry="1"
constant="true"/>
        &lt;/listOfProducts&gt;
        &lt;fbc:geneProductAssociation&gt;
            &lt;fbc:geneProductRef fbc:geneProduct="SAUSA300_0758"/&gt;
        &lt;/fbc:geneProductAssociation&gt;
    &lt;/reaction&gt;

    <reaction metaid="R_TRDR" id="R_TRDR" name="thioredoxin reductase
(NADPH)" reversible="false" fast="false" fbc:lowerFluxBound="irr_lb"
fbc:upperFluxBound="irr_ub">
        &lt;listOfReactants&gt;
            <speciesReference species="M_h_c" stoichiometry="1"
constant="true"/>
            <speciesReference species="M_nadph_c" stoichiometry="1"
constant="true"/>
            <speciesReference species="M_trdox_c" stoichiometry="1"
constant="true"/>
        &lt;/listOfReactants&gt;
        &lt;listOfProducts&gt;
            <speciesReference species="M_nadp_c" stoichiometry="1"
constant="true"/>
            <speciesReference species="M_trdrd_c" stoichiometry="1"
constant="true"/>
        &lt;/listOfProducts&gt;
        &lt;fbc:geneProductAssociation&gt;
            &lt;fbc:or&gt;
                &lt;fbc:geneProductRef fbc:geneProduct="SAUSA300_1369"/&gt;
                &lt;fbc:geneProductRef fbc:geneProduct="SAUSA300_0747"/&gt;
                &lt;fbc:geneProductRef fbc:geneProduct="SAUSA300_2319"/&gt;
            &lt;/fbc:or&gt;
        &lt;/fbc:geneProductAssociation&gt;
    &lt;/reaction&gt;
```

```xml
      <reaction metaid="R_TRE6PH" id="R_TRE6PH" name="trehalose-6-phosphate
hydrolase" reversible="false" fast="false" fbc:lowerFluxBound="irr_lb"
fbc:upperFluxBound="irr_ub">
            <listOfReactants>
                  <speciesReference species="M_h2o_c" stoichiometry="1"
constant="true"/>
                  <speciesReference species="M_tre6p_c" stoichiometry="1"
constant="true"/>
            </listOfReactants>
            <listOfProducts>
                  <speciesReference species="M_g6p_c" stoichiometry="1"
constant="true"/>
                  <speciesReference species="M_glc__D_c" stoichiometry="1"
constant="true"/>
            </listOfProducts>
            <fbc:geneProductAssociation>
                  <fbc:geneProductRef fbc:geneProduct="SAUSA300_0449"/>
            </fbc:geneProductAssociation>
      </reaction>

      <reaction metaid="R_TREpts" id="R_TREpts" name="trehalose transport via
PEP:Pyr PTS" reversible="true" fast="false" fbc:lowerFluxBound="rev_lb"
fbc:upperFluxBound="rev_ub">
            <listOfReactants>
                  <speciesReference species="M_pep_c" stoichiometry="1"
constant="true"/>
                  <speciesReference species="M_tre_e" stoichiometry="1"
constant="true"/>
            </listOfReactants>
            <listOfProducts>
                  <speciesReference species="M_pyr_c" stoichiometry="1"
constant="true"/>
                  <speciesReference species="M_tre6p_c" stoichiometry="1"
constant="true"/>
            </listOfProducts>
            <fbc:geneProductAssociation>
                  <fbc:and>
                        <fbc:geneProductRef fbc:geneProduct="SAUSA300_0448"/>
                        <fbc:geneProductRef fbc:geneProduct="SAUSA300_0983"/>
                        <fbc:geneProductRef fbc:geneProduct="SAUSA300_0984"/>
                  </fbc:and>
            </fbc:geneProductAssociation>
      </reaction>

      <reaction metaid="R_TRO2EAO" id="R_TRO2EAO" name="trans-Octodec-2-
enoyl-ACP NAD oxidoreductase A-specific " reversible="false" fast="false"
fbc:lowerFluxBound="irr_lb" fbc:upperFluxBound="irr_ub">
            <listOfReactants>
                  <speciesReference species="M_h_c" stoichiometry="1"
constant="true"/>
                  <speciesReference species="M_nadh_c" stoichiometry="1"
constant="true"/>
                  <speciesReference species="M_to2eACP_c" stoichiometry="1"
constant="true"/>
            </listOfReactants>
            <listOfProducts>
                  <speciesReference species="M_nad_c" stoichiometry="1"
constant="true"/>
```

```
                  <speciesReference species="M_octACP_c" stoichiometry="1"
constant="true"/>
            &lt;/listOfProducts&gt;
            &lt;fbc:geneProductAssociation&gt;
                  &lt;fbc:geneProductRef fbc:geneProduct="SAUSA300_0912"/&gt;
            &lt;/fbc:geneProductAssociation&gt;
      &lt;/reaction&gt;

      <reaction metaid="R_TRO2EHL" id="R_TRO2EHL" name="trans-Octodec-2-
enoyl-ACP hydro__Lyase " reversible="true" fast="false"
fbc:lowerFluxBound="rev_lb" fbc:upperFluxBound="rev_ub">
            &lt;listOfReactants&gt;
                  <speciesReference species="M_3hodACP_c" stoichiometry="1"
constant="true"/>
            &lt;/listOfReactants&gt;
            &lt;listOfProducts&gt;
                  <speciesReference species="M_h2o_c" stoichiometry="1"
constant="true"/>
                  <speciesReference species="M_to2eACP_c" stoichiometry="1"
constant="true"/>
            &lt;/listOfProducts&gt;
            &lt;fbc:geneProductAssociation&gt;
                  &lt;fbc:geneProductRef fbc:geneProduct="SAUSA300_2054"/&gt;
            &lt;/fbc:geneProductAssociation&gt;
      &lt;/reaction&gt;

      <reaction metaid="R_TRPS1" id="R_TRPS1" name="tryptophan synthase
(indoleglycerol phosphate)" reversible="false" fast="false"
fbc:lowerFluxBound="irr_lb" fbc:upperFluxBound="irr_ub">
            &lt;listOfReactants&gt;
                  <speciesReference species="M_3ig3p_c" stoichiometry="1"
constant="true"/>
                  <speciesReference species="M_ser__L_c" stoichiometry="1"
constant="true"/>
            &lt;/listOfReactants&gt;
            &lt;listOfProducts&gt;
                  <speciesReference species="M_h2o_c" stoichiometry="1"
constant="true"/>
                  <speciesReference species="M_g3p_c" stoichiometry="1"
constant="true"/>
                  <speciesReference species="M_trp__L_c" stoichiometry="1"
constant="true"/>
            &lt;/listOfProducts&gt;
            &lt;fbc:geneProductAssociation&gt;
                  &lt;fbc:and&gt;
                        &lt;fbc:geneProductRef fbc:geneProduct="SAUSA300_1267"/&gt;
                        &lt;fbc:geneProductRef fbc:geneProduct="SAUSA300_1268"/&gt;
                  &lt;/fbc:and&gt;
            &lt;/fbc:geneProductAssociation&gt;
      &lt;/reaction&gt;

      <reaction metaid="R_TRPS2" id="R_TRPS2" name="tryptophan synthase
(indole)" reversible="false" fast="false" fbc:lowerFluxBound="irr_lb"
fbc:upperFluxBound="irr_ub">
            &lt;listOfReactants&gt;
                  <speciesReference species="M_indole_c" stoichiometry="1"
constant="true"/>
```

```
                  <speciesReference species="M_ser__L_c" stoichiometry="1"
constant="true"/>
            </listOfReactants>
            <listOfProducts>
                  <speciesReference species="M_h2o_c" stoichiometry="1"
constant="true"/>
                  <speciesReference species="M_trp__L_c" stoichiometry="1"
constant="true"/>
            </listOfProducts>
            <fbc:geneProductAssociation>
                  <fbc:and>
                        <fbc:geneProductRef fbc:geneProduct="SAUSA300_1267"/>
                        <fbc:geneProductRef fbc:geneProduct="SAUSA300_1268"/>
                  </fbc:and>
            </fbc:geneProductAssociation>
      </reaction>

      <reaction metaid="R_TRPS3r" id="R_TRPS3r" name="tryptophan synthase
(indoleglycerol phosphate)" reversible="false" fast="false"
fbc:lowerFluxBound="irr_lb" fbc:upperFluxBound="irr_ub">
            <listOfReactants>
                  <speciesReference species="M_3ig3p_c" stoichiometry="1"
constant="true"/>
            </listOfReactants>
            <listOfProducts>
                  <speciesReference species="M_g3p_c" stoichiometry="1"
constant="true"/>
                  <speciesReference species="M_indole_c" stoichiometry="1"
constant="true"/>
            </listOfProducts>
            <fbc:geneProductAssociation>
                  <fbc:and>
                        <fbc:geneProductRef fbc:geneProduct="SAUSA300_1267"/>
                        <fbc:geneProductRef fbc:geneProduct="SAUSA300_1268"/>
                  </fbc:and>
            </fbc:geneProductAssociation>
      </reaction>

      <reaction metaid="R_TRPt2r" id="R_TRPt2r" name="L tryptophan reversible
transport via proton symport" reversible="true" fast="false"
fbc:lowerFluxBound="rev_lb" fbc:upperFluxBound="rev_ub">
            <listOfReactants>
                  <speciesReference species="M_h_e" stoichiometry="1"
constant="true"/>
                  <speciesReference species="M_trp__L_e" stoichiometry="1"
constant="true"/>
            </listOfReactants>
            <listOfProducts>
                  <speciesReference species="M_h_c" stoichiometry="1"
constant="true"/>
                  <speciesReference species="M_trp__L_c" stoichiometry="1"
constant="true"/>
            </listOfProducts>
            <fbc:geneProductAssociation>
                  <fbc:geneProductRef fbc:geneProduct="SAUSA300_0712"/>
            </fbc:geneProductAssociation>
      </reaction>
```

```
      <reaction metaid="R_TRSARr" id="R_TRSARr" name="tartronate semialdehyde
reductase" reversible="true" fast="false" fbc:lowerFluxBound="rev_lb"
fbc:upperFluxBound="rev_ub">
            &lt;listOfReactants&gt;
                  <speciesReference species="M_h_c" stoichiometry="1"
constant="true"/>
                  <speciesReference species="M_nadh_c" stoichiometry="1"
constant="true"/>
                  <speciesReference species="M_2h3oppan_c" stoichiometry="1"
constant="true"/>
            &lt;/listOfReactants&gt;
            &lt;listOfProducts&gt;
                  <speciesReference species="M_nad_c" stoichiometry="1"
constant="true"/>
                  <speciesReference species="M_glyc__R_c" stoichiometry="1"
constant="true"/>
            &lt;/listOfProducts&gt;
      &lt;/reaction&gt;

      <reaction metaid="R_TYRt2r" id="R_TYRt2r" name="L-tyrosine reversible
transport via proton symport" reversible="true" fast="false"
fbc:lowerFluxBound="rev_lb" fbc:upperFluxBound="rev_ub">
            &lt;listOfReactants&gt;
                  <speciesReference species="M_h_e" stoichiometry="1"
constant="true"/>
                  <speciesReference species="M_tyr__L_e" stoichiometry="1"
constant="true"/>
            &lt;/listOfReactants&gt;
            &lt;listOfProducts&gt;
                  <speciesReference species="M_h_c" stoichiometry="1"
constant="true"/>
                  <speciesReference species="M_tyr__L_c" stoichiometry="1"
constant="true"/>
            &lt;/listOfProducts&gt;
            &lt;fbc:geneProductAssociation&gt;
                  &lt;fbc:geneProductRef fbc:geneProduct="SAUSA300_2383"/&gt;
            &lt;/fbc:geneProductAssociation&gt;
      &lt;/reaction&gt;

      <reaction metaid="R_TYRTA" id="R_TYRTA" name="tyrosine transaminase"
reversible="true" fast="false" fbc:lowerFluxBound="rev_lb"
fbc:upperFluxBound="rev_ub">
            &lt;listOfReactants&gt;
                  <speciesReference species="M_akg_c" stoichiometry="1"
constant="true"/>
                  <speciesReference species="M_tyr__L_c" stoichiometry="1"
constant="true"/>
            &lt;/listOfReactants&gt;
            &lt;listOfProducts&gt;
                  <speciesReference species="M_glu__L_c" stoichiometry="1"
constant="true"/>
                  <speciesReference species="M_34hpp_c" stoichiometry="1"
constant="true"/>
            &lt;/listOfProducts&gt;
            &lt;fbc:geneProductAssociation&gt;
                  &lt;fbc:or&gt;
                        &lt;fbc:geneProductRef fbc:geneProduct="SAUSA300_1916"/&gt;
                        &lt;fbc:and&gt;
```

```
                              <fbc:geneProductRef
fbc:geneProduct="SAUSA300_0708"/>
                              <fbc:geneProductRef
fbc:geneProduct="SAUSA300_2610"/>
                        </fbc:and>
                  </fbc:or>
            </fbc:geneProductAssociation>
      </reaction>

      <reaction metaid="R_UAAGDS" id="R_UAAGDS" name="UDP-N-
acetylmuramoyl__L-alanyl__D-glutamyl-meso-2,6__Diaminopimelate synthetase"
reversible="false" fast="false" fbc:lowerFluxBound="irr_lb"
fbc:upperFluxBound="irr_ub">
            <listOfReactants>
                  <speciesReference species="M_atp_c" stoichiometry="1"
constant="true"/>
                  <speciesReference species="M_26dap__M_c" stoichiometry="1"
constant="true"/>
                  <speciesReference species="M_uamag_c" stoichiometry="1"
constant="true"/>
            </listOfReactants>
            <listOfProducts>
                  <speciesReference species="M_h_c" stoichiometry="1"
constant="true"/>
                  <speciesReference species="M_pi_c" stoichiometry="1"
constant="true"/>
                  <speciesReference species="M_adp_c" stoichiometry="1"
constant="true"/>
                  <speciesReference species="M_ugmd_c" stoichiometry="1"
constant="true"/>
            </listOfProducts>
            <fbc:geneProductAssociation>
                  <fbc:and>
                        <fbc:geneProductRef fbc:geneProduct="SAUSA300_0919"/>
                        <fbc:geneProductRef fbc:geneProduct="SAUSA300_1873"/>
                  </fbc:and>
            </fbc:geneProductAssociation>
      </reaction>

      <reaction metaid="R_UAG2E" id="R_UAG2E" name="UDP-N-acetylglucosamine
2-epimerase" reversible="true" fast="false" fbc:lowerFluxBound="rev_lb"
fbc:upperFluxBound="rev_ub">
            <listOfReactants>
                  <speciesReference species="M_uacgam_c" stoichiometry="1"
constant="true"/>
            </listOfReactants>
            <listOfProducts>
                  <speciesReference species="M_uacmam_c" stoichiometry="1"
constant="true"/>
            </listOfProducts>
            <fbc:geneProductAssociation>
                  <fbc:or>
                        <fbc:geneProductRef fbc:geneProduct="SAUSA300_0158"/>
                        <fbc:geneProductRef fbc:geneProduct="SAUSA300_2065"/>
                        <fbc:geneProductRef fbc:geneProduct="SAUSA300_2065"/>
                        <fbc:geneProductRef fbc:geneProduct="SAUSA300_0167"/>
                  </fbc:or>
            </fbc:geneProductAssociation>
```

```xml
      </reaction>

      <reaction metaid="R_UAG2EMA" id="R_UAG2EMA" name="UDP-N-acetyl__D-
glucosamine 2-epimerase (Hydrolysis)" reversible="true" fast="false"
fbc:lowerFluxBound="rev_lb" fbc:upperFluxBound="rev_ub">
            <listOfReactants>
                  <speciesReference species="M_h2o_c" stoichiometry="1"
constant="true"/>
                  <speciesReference species="M_uacgam_c" stoichiometry="1"
constant="true"/>
            </listOfReactants>
            <listOfProducts>
                  <speciesReference species="M_acmana_c" stoichiometry="1"
constant="true"/>
                  <speciesReference species="M_udp_c" stoichiometry="1"
constant="true"/>
            </listOfProducts>
            <fbc:geneProductAssociation>
                  <fbc:or>
                        <fbc:geneProductRef fbc:geneProduct="SAUSA300_0158"/>
                        <fbc:geneProductRef fbc:geneProduct="SAUSA300_2065"/>
                        <fbc:geneProductRef fbc:geneProduct="SAUSA300_0130"/>
                  </fbc:or>
            </fbc:geneProductAssociation>
      </reaction>

      <reaction metaid="R_UAG4E" id="R_UAG4E" name="UDP-N-acetylglucosamine
4-epimerase" reversible="true" fast="false" fbc:lowerFluxBound="rev_lb"
fbc:upperFluxBound="rev_ub">
            <listOfReactants>
                  <speciesReference species="M_uacgam_c" stoichiometry="1"
constant="true"/>
            </listOfReactants>
            <listOfProducts>
                  <speciesReference species="M_udpacgal_c" stoichiometry="1"
constant="true"/>
            </listOfProducts>
            <fbc:geneProductAssociation>
                  <fbc:or>
                        <fbc:geneProductRef fbc:geneProduct="SAUSA300_0130"/>
                        <fbc:geneProductRef fbc:geneProduct="SAUSA300_0165"/>
                  </fbc:or>
            </fbc:geneProductAssociation>
      </reaction>

      <reaction metaid="R_UAGCVT" id="R_UAGCVT" name="UDP-N-acetylglucosamine
1-carboxyvinyltransferase" reversible="false" fast="false"
fbc:lowerFluxBound="irr_lb" fbc:upperFluxBound="irr_ub">
            <listOfReactants>
                  <speciesReference species="M_uacgam_c" stoichiometry="1"
constant="true"/>
                  <speciesReference species="M_pep_c" stoichiometry="1"
constant="true"/>
            </listOfReactants>
            <listOfProducts>
                  <speciesReference species="M_h_c" stoichiometry="1"
constant="true"/>
```

```
                  <speciesReference species="M_pi_c" stoichiometry="1"
constant="true"/>
                  <speciesReference species="M_uaccg_c" stoichiometry="1"
constant="true"/>
            </listOfProducts>
            <fbc:geneProductAssociation>
                  <fbc:or>
                        <fbc:geneProductRef fbc:geneProduct="SAUSA300_2055"/>
                        <fbc:geneProductRef fbc:geneProduct="SAUSA300_2078"/>
                  </fbc:or>
            </fbc:geneProductAssociation>
      </reaction>

      <reaction metaid="R_UAGDP" id="R_UAGDP" name="UDP-N-acetylglucosamine
diphosphorylase" reversible="false" fast="false" fbc:lowerFluxBound="irr_lb"
fbc:upperFluxBound="irr_ub">
            <listOfReactants>
                  <speciesReference species="M_utp_c" stoichiometry="1"
constant="true"/>
                  <speciesReference species="M_acgam1p_c" stoichiometry="1"
constant="true"/>
            </listOfReactants>
            <listOfProducts>
                  <speciesReference species="M_ppi_c" stoichiometry="1"
constant="true"/>
                  <speciesReference species="M_uacgam_c" stoichiometry="1"
constant="true"/>
            </listOfProducts>
            <fbc:geneProductAssociation>
                  <fbc:or>
                        <fbc:geneProductRef fbc:geneProduct="SAUSA300_2130"/>
                        <fbc:geneProductRef fbc:geneProduct="SAUSA300_0477"/>
                  </fbc:or>
            </fbc:geneProductAssociation>
      </reaction>

      <reaction metaid="R_UAGPT2" id="R_UAGPT2" name="UDP-N-
acetylglucosamine-N-acetylmuramyl-(pentapeptide)pyrophosphoryl-undecaprenol
N-acetylglucosamine transferase" reversible="true" fast="false"
fbc:lowerFluxBound="rev_lb" fbc:upperFluxBound="rev_ub">
            <listOfReactants>
                  <speciesReference species="M_uacgam_c" stoichiometry="1"
constant="true"/>
                  <speciesReference species="M_uaGgla_c" stoichiometry="1"
constant="true"/>
            </listOfReactants>
            <listOfProducts>
                  <speciesReference species="M_udp_c" stoichiometry="1"
constant="true"/>
                  <speciesReference species="M_uaaGgla_c" stoichiometry="1"
constant="true"/>
            </listOfProducts>
            <fbc:geneProductAssociation>
                  <fbc:geneProductRef fbc:geneProduct="SAUSA300_1311"/>
            </fbc:geneProductAssociation>
      </reaction>
```

```
      <reaction metaid="R_UAGPT3" id="R_UAGPT3" name="UDP-N-
acetylglucosamine-N-acetylmuramyl-(pentapeptide)pyrophosphoryl-undecaprenol
N-acetylglucosamine transferase" reversible="false" fast="false"
fbc:lowerFluxBound="irr_lb" fbc:upperFluxBound="irr_ub">
            <listOfReactants>
                  <speciesReference species="M_uacgam_c" stoichiometry="1"
constant="true"/>
                  <speciesReference species="M_uagmda_c" stoichiometry="1"
constant="true"/>
            </listOfReactants>
            <listOfProducts>
                  <speciesReference species="M_udp_c" stoichiometry="1"
constant="true"/>
                  <speciesReference species="M_uaagmda_c" stoichiometry="1"
constant="true"/>
            </listOfProducts>
            <fbc:geneProductAssociation>
                  <fbc:geneProductRef fbc:geneProduct="SAUSA300_1311"/>
            </fbc:geneProductAssociation>
      </reaction>

      <reaction metaid="R_UAMAGS" id="R_UAMAGS" name="UDP-N-
acetylmuramoyl__L-alanyl__D-glutamate synthetase" reversible="false"
fast="false" fbc:lowerFluxBound="irr_lb" fbc:upperFluxBound="irr_ub">
            <listOfReactants>
                  <speciesReference species="M_glu__D_c" stoichiometry="1"
constant="true"/>
                  <speciesReference species="M_atp_c" stoichiometry="1"
constant="true"/>
                  <speciesReference species="M_uama_c" stoichiometry="1"
constant="true"/>
            </listOfReactants>
            <listOfProducts>
                  <speciesReference species="M_h_c" stoichiometry="1"
constant="true"/>
                  <speciesReference species="M_pi_c" stoichiometry="1"
constant="true"/>
                  <speciesReference species="M_adp_c" stoichiometry="1"
constant="true"/>
                  <speciesReference species="M_uamag_c" stoichiometry="1"
constant="true"/>
            </listOfProducts>
            <fbc:geneProductAssociation>
                  <fbc:geneProductRef fbc:geneProduct="SAUSA300_1077"/>
            </fbc:geneProductAssociation>
      </reaction>

      <reaction metaid="R_UAMAS" id="R_UAMAS" name="UDP-N-acetylmuramoyl__L-
alanine synthetase" reversible="false" fast="false"
fbc:lowerFluxBound="irr_lb" fbc:upperFluxBound="irr_ub">
            <listOfReactants>
                  <speciesReference species="M_atp_c" stoichiometry="1"
constant="true"/>
                  <speciesReference species="M_ala__L_c" stoichiometry="1"
constant="true"/>
                  <speciesReference species="M_uamr_c" stoichiometry="1"
constant="true"/>
            </listOfReactants>
```

<listOfProducts>
<speciesReference species="M_h_c" stoichiometry="1"
constant="true"/>
<speciesReference species="M_pi_c" stoichiometry="1"
constant="true"/>
<speciesReference species="M_adp_c" stoichiometry="1"
constant="true"/>
<speciesReference species="M_uama_c" stoichiometry="1"
constant="true"/>
</listOfProducts>
<fbc:geneProductAssociation>
<fbc:geneProductRef fbc:geneProduct="SAUSA300_1686"/>
</fbc:geneProductAssociation>
</reaction>

<reaction metaid="R_UAPGR" id="R_UAPGR" name="UDP-N-
acetylenolpyruvoylglucosamine reductase" reversible="false" fast="false"
fbc:lowerFluxBound="irr_lb" fbc:upperFluxBound="irr_ub">
<listOfReactants>
<speciesReference species="M_h_c" stoichiometry="1"
constant="true"/>
<speciesReference species="M_nadph_c" stoichiometry="1"
constant="true"/>
<speciesReference species="M_uaccg_c" stoichiometry="1"
constant="true"/>
</listOfReactants>
<listOfProducts>
<speciesReference species="M_nadp_c" stoichiometry="1"
constant="true"/>
<speciesReference species="M_uamr_c" stoichiometry="1"
constant="true"/>
</listOfProducts>
<fbc:geneProductAssociation>
<fbc:geneProductRef fbc:geneProduct="SAUSA300_0722"/>
</fbc:geneProductAssociation>
</reaction>

<reaction metaid="R_UCPP" id="R_UCPP" name="UTP cytidine 5'-
phosphotransferase " reversible="false" fast="false"
fbc:lowerFluxBound="irr_lb" fbc:upperFluxBound="irr_ub">
<listOfReactants>
<speciesReference species="M_cytd_c" stoichiometry="1"
constant="true"/>
<speciesReference species="M_utp_c" stoichiometry="1"
constant="true"/>
</listOfReactants>
<listOfProducts>
<speciesReference species="M_cmp_c" stoichiometry="1"
constant="true"/>
<speciesReference species="M_udp_c" stoichiometry="1"
constant="true"/>
</listOfProducts>
<fbc:geneProductAssociation>
<fbc:geneProductRef fbc:geneProduct="SAUSA300_1568"/>
</fbc:geneProductAssociation>
</reaction>

```
      <reaction metaid="R_UDALDL" id="R_UDALDL" name="UDP-N-
acetylmuramoyl__L-alanyl__D-glutamate L__Lysine ligase " reversible="false"
fast="false" fbc:lowerFluxBound="irr_lb" fbc:upperFluxBound="irr_ub">
            <listOfReactants>
                  <speciesReference species="M_atp_c" stoichiometry="1"
constant="true"/>
                  <speciesReference species="M_lys__L_c" stoichiometry="1"
constant="true"/>
                  <speciesReference species="M_uamag_c" stoichiometry="1"
constant="true"/>
            </listOfReactants>
            <listOfProducts>
                  <speciesReference species="M_h_c" stoichiometry="1"
constant="true"/>
                  <speciesReference species="M_pi_c" stoichiometry="1"
constant="true"/>
                  <speciesReference species="M_adp_c" stoichiometry="1"
constant="true"/>
                  <speciesReference species="M_uGgl_c" stoichiometry="1"
constant="true"/>
            </listOfProducts>
            <fbc:geneProductAssociation>
                  <fbc:geneProductRef fbc:geneProduct="SAUSA300_0919"/>
            </fbc:geneProductAssociation>
      </reaction>

      <reaction metaid="R_UDCPDPpp" id="R_UDCPDPpp"
name="undecaprenyl__Diphosphatase (periplasm)" reversible="false"
fast="false" fbc:lowerFluxBound="irr_lb" fbc:upperFluxBound="irr_ub">
            <listOfReactants>
                  <speciesReference species="M_h2o_c" stoichiometry="1"
constant="true"/>
                  <speciesReference species="M_udcpdp_c" stoichiometry="1"
constant="true"/>
            </listOfReactants>
            <listOfProducts>
                  <speciesReference species="M_h_c" stoichiometry="1"
constant="true"/>
                  <speciesReference species="M_pi_c" stoichiometry="1"
constant="true"/>
                  <speciesReference species="M_udcpp_c" stoichiometry="1"
constant="true"/>
            </listOfProducts>
            <fbc:geneProductAssociation>
                  <fbc:geneProductRef fbc:geneProduct="SAUSA300_0669"/>
            </fbc:geneProductAssociation>
      </reaction>

      <reaction metaid="R_UDCPDPS" id="R_UDCPDPS" name="Undecaprenyl
diphosphate synthase" reversible="false" fast="false"
fbc:lowerFluxBound="irr_lb" fbc:upperFluxBound="irr_ub">
            <listOfReactants>
                  <speciesReference species="M_ipdp_c" stoichiometry="8"
constant="true"/>
                  <speciesReference species="M_frdp_c" stoichiometry="1"
constant="true"/>
            </listOfReactants>
            <listOfProducts>
```

```
                  <speciesReference species="M_ppi_c" stoichiometry="8"
constant="true"/>
                  <speciesReference species="M_udcpdp_c" stoichiometry="1"
constant="true"/>
            &lt;/listOfProducts&gt;
            &lt;fbc:geneProductAssociation&gt;
                  &lt;fbc:geneProductRef fbc:geneProduct="SAUSA300_1153"/&gt;
            &lt;/fbc:geneProductAssociation&gt;
      &lt;/reaction&gt;

      <reaction metaid="R_UDCPKr" id="R_UDCPKr" name="undecaprenol kinase
(reversible)" reversible="false" fast="false" fbc:lowerFluxBound="irr_lb"
fbc:upperFluxBound="irr_ub">
            &lt;listOfReactants&gt;
                  <speciesReference species="M_atp_c" stoichiometry="1"
constant="true"/>
                  <speciesReference species="M_udcp_c" stoichiometry="1"
constant="true"/>
            &lt;/listOfReactants&gt;
            &lt;listOfProducts&gt;
                  <speciesReference species="M_h_c" stoichiometry="1"
constant="true"/>
                  <speciesReference species="M_adp_c" stoichiometry="1"
constant="true"/>
                  <speciesReference species="M_udcpp_c" stoichiometry="1"
constant="true"/>
            &lt;/listOfProducts&gt;
            &lt;fbc:geneProductAssociation&gt;
                  &lt;fbc:geneProductRef fbc:geneProduct="SAUSA300_0669"/&gt;
            &lt;/fbc:geneProductAssociation&gt;
      &lt;/reaction&gt;

      <reaction metaid="R_UDCTPP" id="R_UDCTPP" name="dCTP uridine 5'-
phosphotransferase " reversible="false" fast="false"
fbc:lowerFluxBound="irr_lb" fbc:upperFluxBound="irr_ub">
            &lt;listOfReactants&gt;
                  <speciesReference species="M_dctp_c" stoichiometry="1"
constant="true"/>
                  <speciesReference species="M_uri_c" stoichiometry="1"
constant="true"/>
            &lt;/listOfReactants&gt;
            &lt;listOfProducts&gt;
                  <speciesReference species="M_ump_c" stoichiometry="1"
constant="true"/>
                  <speciesReference species="M_dcdp_c" stoichiometry="1"
constant="true"/>
            &lt;/listOfProducts&gt;
            &lt;fbc:geneProductAssociation&gt;
                  &lt;fbc:geneProductRef fbc:geneProduct="SAUSA300_1568"/&gt;
            &lt;/fbc:geneProductAssociation&gt;
      &lt;/reaction&gt;

      <reaction metaid="R_UDNDMx" id="R_UDNDMx" name="UDP-N-acetyl__D-
mannosamine NAD 1-oxidoreductase " reversible="true" fast="false"
fbc:lowerFluxBound="rev_lb" fbc:upperFluxBound="rev_ub">
            &lt;listOfReactants&gt;
                  <speciesReference species="M_h2o_c" stoichiometry="1"
constant="true"/>
```

```
                  <speciesReference species="M_nad_c" stoichiometry="2"
constant="true"/>
                  <speciesReference species="M_uacmam_c" stoichiometry="1"
constant="true"/>
            </listOfReactants>
            <listOfProducts>
                  <speciesReference species="M_h_c" stoichiometry="3"
constant="true"/>
                  <speciesReference species="M_nadh_c" stoichiometry="2"
constant="true"/>
                  <speciesReference species="M_uacmamu_c" stoichiometry="1"
constant="true"/>
            </listOfProducts>
            <fbc:geneProductAssociation>
                  <fbc:geneProductRef fbc:geneProduct="SAUSA300_0166"/>
            </fbc:geneProductAssociation>
      </reaction>

      <reaction metaid="R_UDPDPS" id="R_UDPDPS"
name="undecaprenyl__Diphosphate synthase" reversible="false" fast="false"
fbc:lowerFluxBound="irr_lb" fbc:upperFluxBound="irr_ub">
            <listOfReactants>
                  <speciesReference species="M_ipdp_c" stoichiometry="1"
constant="true"/>
                  <speciesReference species="M_decdp_c" stoichiometry="1"
constant="true"/>
            </listOfReactants>
            <listOfProducts>
                  <speciesReference species="M_ppi_c" stoichiometry="1"
constant="true"/>
                  <speciesReference species="M_udcpdp_c" stoichiometry="1"
constant="true"/>
            </listOfProducts>
            <fbc:geneProductAssociation>
                  <fbc:geneProductRef fbc:geneProduct="SAUSA300_1153"/>
            </fbc:geneProductAssociation>
      </reaction>

      <reaction metaid="R_UDPG12dgrGT_SA2" id="R_UDPG12dgrGT_SA2"
name="UDPglucose:1,2__Diacylglycerol 3__D-glucosyltransferase"
reversible="false" fast="false" fbc:lowerFluxBound="irr_lb"
fbc:upperFluxBound="irr_ub">
            <listOfReactants>
                  <speciesReference species="M_udpg_c" stoichiometry="50"
constant="true"/>
                  <speciesReference species="M_12dgr_SA_c" stoichiometry="1"
constant="true"/>
            </listOfReactants>
            <listOfProducts>
                  <speciesReference species="M_udp_c" stoichiometry="50"
constant="true"/>
                  <speciesReference species="M_3g12dgr_SA2_c"
stoichiometry="1" constant="true"/>
            </listOfProducts>
            <fbc:geneProductAssociation>
                  <fbc:geneProductRef fbc:geneProduct="SAUSA300_0918"/>
            </fbc:geneProductAssociation>
      </reaction>
```

```
      <reaction metaid="R_UDPG3g12dgrGT_SA2" id="R_UDPG3g12dgrGT_SA2"
name="UDPglucose:3g12dgr 3__D-glucosyltransferase 2" reversible="false"
fast="false" fbc:lowerFluxBound="irr_lb" fbc:upperFluxBound="irr_ub">
            <listOfReactants>
                  <speciesReference species="M_udpg_c" stoichiometry="50"
constant="true"/>
                  <speciesReference species="M_3g12dgr_SA2_c"
stoichiometry="1" constant="true"/>
            </listOfReactants>
            <listOfProducts>
                  <speciesReference species="M_udp_c" stoichiometry="50"
constant="true"/>
                  <speciesReference species="M_dgdcg_SA2_c" stoichiometry="1"
constant="true"/>
            </listOfProducts>
            <fbc:geneProductAssociation>
                  <fbc:geneProductRef fbc:geneProduct="SAUSA300_0918"/>
            </fbc:geneProductAssociation>
      </reaction>

      <reaction metaid="R_UDPG4E" id="R_UDPG4E" name="UDPglucose 4-epimerase"
reversible="true" fast="false" fbc:lowerFluxBound="rev_lb"
fbc:upperFluxBound="rev_ub">
            <listOfReactants>
                  <speciesReference species="M_udpg_c" stoichiometry="1"
constant="true"/>
            </listOfReactants>
            <listOfProducts>
                  <speciesReference species="M_udpgal_c" stoichiometry="1"
constant="true"/>
            </listOfProducts>
            <fbc:geneProductAssociation>
                  <fbc:or>
                        <fbc:geneProductRef fbc:geneProduct="SAUSA300_0130"/>
                        <fbc:geneProductRef fbc:geneProduct="SAUSA300_0165"/>
                  </fbc:or>
            </fbc:geneProductAssociation>
      </reaction>

      <reaction metaid="R_UDUPP" id="R_UDUPP" name="UTP uridine 5'-
phosphotransferase " reversible="false" fast="false"
fbc:lowerFluxBound="irr_lb" fbc:upperFluxBound="irr_ub">
            <listOfReactants>
                  <speciesReference species="M_utp_c" stoichiometry="1"
constant="true"/>
                  <speciesReference species="M_uri_c" stoichiometry="1"
constant="true"/>
            </listOfReactants>
            <listOfProducts>
                  <speciesReference species="M_udp_c" stoichiometry="1"
constant="true"/>
                  <speciesReference species="M_ump_c" stoichiometry="1"
constant="true"/>
            </listOfProducts>
            <fbc:geneProductAssociation>
                  <fbc:geneProductRef fbc:geneProduct="SAUSA300_1568"/>
            </fbc:geneProductAssociation>
```

```
      </reaction>

      <reaction metaid="R_UGLT" id="R_UGLT" name="UDPglucose--hexose-1-
phosphate uridylyltransferase" reversible="true" fast="false"
fbc:lowerFluxBound="rev_lb" fbc:upperFluxBound="rev_ub">
            <listOfReactants>
                  <speciesReference species="M_udpg_c" stoichiometry="1"
constant="true"/>
                  <speciesReference species="M_gal1p_c" stoichiometry="1"
constant="true"/>
            </listOfReactants>
            <listOfProducts>
                  <speciesReference species="M_g1p_c" stoichiometry="1"
constant="true"/>
                  <speciesReference species="M_udpgal_c" stoichiometry="1"
constant="true"/>
            </listOfProducts>
            <fbc:geneProductAssociation>
                  <fbc:geneProductRef fbc:geneProduct="SAUSA300_2439"/>
            </fbc:geneProductAssociation>
      </reaction>

      <reaction metaid="R_UGMDDS" id="R_UGMDDS" name="UDP-N-
acetylmuramoyl__L-alanyl__D-glutamyl-meso-2,6__Diaminopimeloyl__D-alanyl__D-
alanine synthetase" reversible="false" fast="false"
fbc:lowerFluxBound="irr_lb" fbc:upperFluxBound="irr_ub">
            <listOfReactants>
                  <speciesReference species="M_atp_c" stoichiometry="1"
constant="true"/>
                  <speciesReference species="M_alaala_c" stoichiometry="1"
constant="true"/>
                  <speciesReference species="M_ugmd_c" stoichiometry="1"
constant="true"/>
            </listOfReactants>
            <listOfProducts>
                  <speciesReference species="M_h_c" stoichiometry="1"
constant="true"/>
                  <speciesReference species="M_pi_c" stoichiometry="1"
constant="true"/>
                  <speciesReference species="M_adp_c" stoichiometry="1"
constant="true"/>
                  <speciesReference species="M_ugmda_c" stoichiometry="1"
constant="true"/>
            </listOfProducts>
            <fbc:geneProductAssociation>
                  <fbc:geneProductRef fbc:geneProduct="SAUSA300_2038"/>
            </fbc:geneProductAssociation>
      </reaction>

      <reaction metaid="R_UMPK" id="R_UMPK" name="UMP kinase"
reversible="true" fast="false" fbc:lowerFluxBound="rev_lb"
fbc:upperFluxBound="rev_ub">
            <listOfReactants>
                  <speciesReference species="M_atp_c" stoichiometry="1"
constant="true"/>
                  <speciesReference species="M_ump_c" stoichiometry="1"
constant="true"/>
            </listOfReactants>
```

```
            <listOfProducts>
                  <speciesReference species="M_adp_c" stoichiometry="1"
constant="true"/>
                  <speciesReference species="M_udp_c" stoichiometry="1"
constant="true"/>
            </listOfProducts>
            <fbc:geneProductAssociation>
                  <fbc:or>
                        <fbc:geneProductRef fbc:geneProduct="SAUSA300_1151"/>
                        <fbc:geneProductRef fbc:geneProduct="SAUSA300_1367"/>
                  </fbc:or>
            </fbc:geneProductAssociation>
      </reaction>

      <reaction metaid="R_UNDBD" id="R_UNDBD" name="UDP-N-acetyl__D-
mannosamine N-acetyl-beta__D- " reversible="true" fast="false"
fbc:lowerFluxBound="rev_lb" fbc:upperFluxBound="rev_ub">
            <listOfReactants>
                  <speciesReference species="M_uacmam_c" stoichiometry="1"
constant="true"/>
                  <speciesReference species="M_unaga_c" stoichiometry="1"
constant="true"/>
            </listOfReactants>
            <listOfProducts>
                  <speciesReference species="M_nacetlbdgl_c"
stoichiometry="1" constant="true"/>
                  <speciesReference species="M_udp_c" stoichiometry="1"
constant="true"/>
            </listOfProducts>
            <fbc:geneProductAssociation>
                  <fbc:geneProductRef fbc:geneProduct="SAUSA300_0623"/>
            </fbc:geneProductAssociation>
      </reaction>

      <reaction metaid="R_UPP3MT" id="R_UPP3MT" name="uroporphyrinogen
methyltransferase" reversible="false" fast="false"
fbc:lowerFluxBound="irr_lb" fbc:upperFluxBound="irr_ub">
            <listOfReactants>
                  <speciesReference species="M_amet_c" stoichiometry="2"
constant="true"/>
                  <speciesReference species="M_uppg3_c" stoichiometry="1"
constant="true"/>
            </listOfReactants>
            <listOfProducts>
                  <speciesReference species="M_h_c" stoichiometry="2"
constant="true"/>
                  <speciesReference species="M_ahcys_c" stoichiometry="2"
constant="true"/>
                  <speciesReference species="M_dscl_c" stoichiometry="1"
constant="true"/>
            </listOfProducts>
            <fbc:geneProductAssociation>
                  <fbc:or>
                        <fbc:geneProductRef fbc:geneProduct="SAUSA300_2344"/>
                        <fbc:geneProductRef fbc:geneProduct="SAUSA300_0464"/>
                  </fbc:or>
            </fbc:geneProductAssociation>
      </reaction>
```

```
      <reaction metaid="R_UPP3S" id="R_UPP3S" name="uroporphyrinogen-III
synthase" reversible="false" fast="false" fbc:lowerFluxBound="irr_lb"
fbc:upperFluxBound="irr_ub">
            <listOfReactants>
                  <speciesReference species="M_hmbil_c" stoichiometry="1"
constant="true"/>
            </listOfReactants>
            <listOfProducts>
                  <speciesReference species="M_h2o_c" stoichiometry="1"
constant="true"/>
                  <speciesReference species="M_uppg3_c" stoichiometry="1"
constant="true"/>
            </listOfProducts>
            <fbc:geneProductAssociation>
                  <fbc:geneProductRef fbc:geneProduct="SAUSA300_1616"/>
            </fbc:geneProductAssociation>
      </reaction>

      <reaction metaid="R_UPPDC1" id="R_UPPDC1" name="uroporphyrinogen
decarboxylase (uroporphyrinogen III)" reversible="false" fast="false"
fbc:lowerFluxBound="irr_lb" fbc:upperFluxBound="irr_ub">
            <listOfReactants>
                  <speciesReference species="M_h_c" stoichiometry="4"
constant="true"/>
                  <speciesReference species="M_uppg3_c" stoichiometry="1"
constant="true"/>
            </listOfReactants>
            <listOfProducts>
                  <speciesReference species="M_co2_c" stoichiometry="4"
constant="true"/>
                  <speciesReference species="M_cpppg3_c" stoichiometry="1"
constant="true"/>
            </listOfProducts>
            <fbc:geneProductAssociation>
                  <fbc:geneProductRef fbc:geneProduct="SAUSA300_1783"/>
            </fbc:geneProductAssociation>
      </reaction>

      <reaction metaid="R_UPPDC2" id="R_UPPDC2" name="uroporphyrinogen
decarboxylase (uroporphyrinogen I)" reversible="true" fast="false"
fbc:lowerFluxBound="rev_lb" fbc:upperFluxBound="rev_ub">
            <listOfReactants>
                  <speciesReference species="M_h_c" stoichiometry="4"
constant="true"/>
                  <speciesReference species="M_uppg1_c" stoichiometry="1"
constant="true"/>
            </listOfReactants>
            <listOfProducts>
                  <speciesReference species="M_co2_c" stoichiometry="4"
constant="true"/>
                  <speciesReference species="M_cpppg1_c" stoichiometry="1"
constant="true"/>
            </listOfProducts>
            <fbc:geneProductAssociation>
                  <fbc:geneProductRef fbc:geneProduct="SAUSA300_1783"/>
            </fbc:geneProductAssociation>
      </reaction>
```

```
      <reaction metaid="R_UPPRTr" id="R_UPPRTr" name="uracil
phosphoribosyltransferase (r)" reversible="true" fast="false"
fbc:lowerFluxBound="rev_lb" fbc:upperFluxBound="rev_ub">
            <listOfReactants>
                  <speciesReference species="M_prpp_c" stoichiometry="1"
constant="true"/>
                  <speciesReference species="M_ura_c" stoichiometry="1"
constant="true"/>
            </listOfReactants>
            <listOfProducts>
                  <speciesReference species="M_ppi_c" stoichiometry="1"
constant="true"/>
                  <speciesReference species="M_ump_c" stoichiometry="1"
constant="true"/>
            </listOfProducts>
            <fbc:geneProductAssociation>
                  <fbc:or>
                        <fbc:geneProductRef fbc:geneProduct="SAUSA300_1091"/>
                        <fbc:geneProductRef fbc:geneProduct="SAUSA300_2066"/>
                  </fbc:or>
            </fbc:geneProductAssociation>
      </reaction>

      <reaction metaid="R_URAt2" id="R_URAt2" name="uracil transport in via
proton symport" reversible="true" fast="false" fbc:lowerFluxBound="rev_lb"
fbc:upperFluxBound="rev_ub">
            <listOfReactants>
                  <speciesReference species="M_h_e" stoichiometry="1"
constant="true"/>
                  <speciesReference species="M_ura_e" stoichiometry="1"
constant="true"/>
            </listOfReactants>
            <listOfProducts>
                  <speciesReference species="M_h_c" stoichiometry="1"
constant="true"/>
                  <speciesReference species="M_ura_c" stoichiometry="1"
constant="true"/>
            </listOfProducts>
            <fbc:geneProductAssociation>
                  <fbc:geneProductRef fbc:geneProduct="SAUSA300_1092"/>
            </fbc:geneProductAssociation>
      </reaction>

      <reaction metaid="R_URCN" id="R_URCN" name="urocanase"
reversible="true" fast="false" fbc:lowerFluxBound="rev_lb"
fbc:upperFluxBound="rev_ub">
            <listOfReactants>
                  <speciesReference species="M_4izp_c" stoichiometry="1"
constant="true"/>
            </listOfReactants>
            <listOfProducts>
                  <speciesReference species="M_h2o_c" stoichiometry="1"
constant="true"/>
                  <speciesReference species="M_urcan_c" stoichiometry="1"
constant="true"/>
            </listOfProducts>
            <fbc:geneProductAssociation>
```

```
                <fbc:geneProductRef fbc:geneProduct="SAUSA300_2278"/>
            </fbc:geneProductAssociation>
        </reaction>

        <reaction metaid="R_UREA" id="R_UREA" name="urease" reversible="false"
fast="false" fbc:lowerFluxBound="irr_lb" fbc:upperFluxBound="irr_ub">
            <listOfReactants>
                <speciesReference species="M_h_c" stoichiometry="2"
constant="true"/>
                <speciesReference species="M_h2o_c" stoichiometry="1"
constant="true"/>
                <speciesReference species="M_urea_c" stoichiometry="1"
constant="true"/>
            </listOfReactants>
            <listOfProducts>
                <speciesReference species="M_nh4_c" stoichiometry="2"
constant="true"/>
                <speciesReference species="M_co2_c" stoichiometry="1"
constant="true"/>
            </listOfProducts>
            <fbc:geneProductAssociation>
                <fbc:or>
                    <fbc:and>
                        <fbc:geneProductRef
fbc:geneProduct="SAUSA300_2240"/>
                        <fbc:geneProductRef
fbc:geneProduct="SAUSA300_2238"/>
                        <fbc:geneProductRef
fbc:geneProduct="SAUSA300_2239"/>
                    </fbc:and>
                    <fbc:and>
                        <fbc:geneProductRef
fbc:geneProduct="SAUSA300_2242"/>
                        <fbc:geneProductRef
fbc:geneProduct="SAUSA300_2243"/>
                        <fbc:geneProductRef
fbc:geneProduct="SAUSA300_2241"/>
                        <fbc:geneProductRef
fbc:geneProduct="SAUSA300_2244"/>
                    </fbc:and>
                </fbc:or>
            </fbc:geneProductAssociation>
        </reaction>

        <reaction metaid="R_UREAt" id="R_UREAt" name="Urea transport via
facilitate diffusion" reversible="true" fast="false"
fbc:lowerFluxBound="rev_lb" fbc:upperFluxBound="rev_ub">
            <listOfReactants>
                <speciesReference species="M_urea_e" stoichiometry="1"
constant="true"/>
            </listOfReactants>
            <listOfProducts>
                <speciesReference species="M_urea_c" stoichiometry="1"
constant="true"/>
            </listOfProducts>
            <fbc:geneProductAssociation>
                <fbc:geneProductRef fbc:geneProduct="SAUSA300_2237"/>
            </fbc:geneProductAssociation>
```

```
      </reaction>

      <reaction metaid="R_URFGTT" id="R_URFGTT" name="UDP__L-
rhamnose:flavonol-3-O__D-glucoside L-rhamnosyltransferase" reversible="false"
fast="false" fbc:lowerFluxBound="irr_lb" fbc:upperFluxBound="irr_ub">
            <listOfReactants>
                  <speciesReference species="M_amet_c" stoichiometry="1"
constant="true"/>
                  <speciesReference species="M_2ombz_c" stoichiometry="1"
constant="true"/>
            </listOfReactants>
            <listOfProducts>
                  <speciesReference species="M_h_c" stoichiometry="1"
constant="true"/>
                  <speciesReference species="M_ahcys_c" stoichiometry="1"
constant="true"/>
                  <speciesReference species="M_2ommb_c" stoichiometry="1"
constant="true"/>
            </listOfProducts>
            <fbc:geneProductAssociation>
                  <fbc:geneProductRef fbc:geneProduct="SAUSA300_1360"/>
            </fbc:geneProductAssociation>
      </reaction>

      <reaction metaid="R_URIDK2r" id="R_URIDK2r" name="uridylate kinase
(dUMP)" reversible="false" fast="false" fbc:lowerFluxBound="irr_lb"
fbc:upperFluxBound="irr_ub">
            <listOfReactants>
                  <speciesReference species="M_atp_c" stoichiometry="1"
constant="true"/>
                  <speciesReference species="M_dump_c" stoichiometry="1"
constant="true"/>
            </listOfReactants>
            <listOfProducts>
                  <speciesReference species="M_adp_c" stoichiometry="1"
constant="true"/>
                  <speciesReference species="M_dudp_c" stoichiometry="1"
constant="true"/>
            </listOfProducts>
            <fbc:geneProductAssociation>
                  <fbc:or>
                        <fbc:geneProductRef fbc:geneProduct="SAUSA300_0459"/>
                        <fbc:geneProductRef fbc:geneProduct="SAUSA300_1151"/>
                  </fbc:or>
            </fbc:geneProductAssociation>
      </reaction>

      <reaction metaid="R_URIK1" id="R_URIK1" name="uridine kinase
(ATP:Uridine)" reversible="false" fast="false" fbc:lowerFluxBound="irr_lb"
fbc:upperFluxBound="irr_ub">
            <listOfReactants>
                  <speciesReference species="M_atp_c" stoichiometry="1"
constant="true"/>
                  <speciesReference species="M_uri_c" stoichiometry="1"
constant="true"/>
            </listOfReactants>
            <listOfProducts>
```

```
                  <speciesReference species="M_adp_c" stoichiometry="1"
constant="true"/>
                  <speciesReference species="M_ump_c" stoichiometry="1"
constant="true"/>
            &lt;/listOfProducts&gt;
            &lt;fbc:geneProductAssociation&gt;
                  &lt;fbc:geneProductRef fbc:geneProduct="SAUSA300_1568"/&gt;
            &lt;/fbc:geneProductAssociation&gt;
      &lt;/reaction&gt;

      <reaction metaid="R_URIK2" id="R_URIK2" name="uridine kinase
(GTP:Uridine)" reversible="false" fast="false" fbc:lowerFluxBound="irr_lb"
fbc:upperFluxBound="irr_ub">
            &lt;listOfReactants&gt;
                  <speciesReference species="M_uri_c" stoichiometry="1"
constant="true"/>
                  <speciesReference species="M_gtp_c" stoichiometry="1"
constant="true"/>
            &lt;/listOfReactants&gt;
            &lt;listOfProducts&gt;
                  <speciesReference species="M_ump_c" stoichiometry="1"
constant="true"/>
                  <speciesReference species="M_gdp_c" stoichiometry="1"
constant="true"/>
            &lt;/listOfProducts&gt;
            &lt;fbc:geneProductAssociation&gt;
                  &lt;fbc:geneProductRef fbc:geneProduct="SAUSA300_1568"/&gt;
            &lt;/fbc:geneProductAssociation&gt;
      &lt;/reaction&gt;

      <reaction metaid="R_URIK3" id="R_URIK3" name="uridine kinase
(ITP:Uridine)" reversible="false" fast="false" fbc:lowerFluxBound="irr_lb"
fbc:upperFluxBound="irr_ub">
            &lt;listOfReactants&gt;
                  <speciesReference species="M_uri_c" stoichiometry="1"
constant="true"/>
                  <speciesReference species="M_itp_c" stoichiometry="1"
constant="true"/>
            &lt;/listOfReactants&gt;
            &lt;listOfProducts&gt;
                  <speciesReference species="M_ump_c" stoichiometry="1"
constant="true"/>
                  <speciesReference species="M_idp_c" stoichiometry="1"
constant="true"/>
            &lt;/listOfProducts&gt;
            &lt;fbc:geneProductAssociation&gt;
                  &lt;fbc:geneProductRef fbc:geneProduct="SAUSA300_1568"/&gt;
            &lt;/fbc:geneProductAssociation&gt;
      &lt;/reaction&gt;

      <reaction metaid="R_URIt2r" id="R_URIt2r" name="uridine transport in
via proton symport, reversible" reversible="false" fast="false"
fbc:lowerFluxBound="irr_lb" fbc:upperFluxBound="irr_ub">
            &lt;listOfReactants&gt;
                  <speciesReference species="M_h_e" stoichiometry="1"
constant="true"/>
                  <speciesReference species="M_uri_e" stoichiometry="1"
constant="true"/>
```

```
            </listOfReactants>
            <listOfProducts>
                  <speciesReference species="M_h_c" stoichiometry="1"
constant="true"/>
                  <speciesReference species="M_uri_c" stoichiometry="1"
constant="true"/>
            </listOfProducts>
            <fbc:geneProductAssociation>
                  <fbc:geneProductRef fbc:geneProduct="SAUSA300_0506"/>
            </fbc:geneProductAssociation>
      </reaction>

      <reaction metaid="R_URIt4" id="R_URIt4" name="uridine transport in via
sodium symport" reversible="false" fast="false" fbc:lowerFluxBound="irr_lb"
fbc:upperFluxBound="irr_ub">
            <listOfReactants>
                  <speciesReference species="M_na1_e" stoichiometry="1"
constant="true"/>
                  <speciesReference species="M_uri_e" stoichiometry="1"
constant="true"/>
            </listOfReactants>
            <listOfProducts>
                  <speciesReference species="M_na1_c" stoichiometry="1"
constant="true"/>
                  <speciesReference species="M_uri_c" stoichiometry="1"
constant="true"/>
            </listOfProducts>
            <fbc:geneProductAssociation>
                  <fbc:or>
                        <fbc:geneProductRef fbc:geneProduct="SAUSA300_0313"/>
                        <fbc:geneProductRef fbc:geneProduct="SAUSA300_0631"/>
                        <fbc:geneProductRef fbc:geneProduct="SAUSA300_0506"/>
                  </fbc:or>
            </fbc:geneProductAssociation>
      </reaction>

      <reaction metaid="R_UUPP" id="R_UUPP" name="ITP cytidine 5'-
phosphotransferase " reversible="false" fast="false"
fbc:lowerFluxBound="irr_lb" fbc:upperFluxBound="irr_ub">
            <listOfReactants>
                  <speciesReference species="M_cytd_c" stoichiometry="1"
constant="true"/>
                  <speciesReference species="M_itp_c" stoichiometry="1"
constant="true"/>
            </listOfReactants>
            <listOfProducts>
                  <speciesReference species="M_cmp_c" stoichiometry="1"
constant="true"/>
                  <speciesReference species="M_idp_c" stoichiometry="1"
constant="true"/>
            </listOfProducts>
            <fbc:geneProductAssociation>
                  <fbc:geneProductRef fbc:geneProduct="SAUSA300_1367"/>
            </fbc:geneProductAssociation>
      </reaction>
```

```
      <reaction metaid="R_VALt2r" id="R_VALt2r" name="L-valine reversible
transport via proton symport" reversible="true" fast="false"
fbc:lowerFluxBound="rev_lb" fbc:upperFluxBound="rev_ub">
            <listOfReactants>
                  <speciesReference species="M_h_e" stoichiometry="1"
constant="true"/>
                  <speciesReference species="M_val__L_e" stoichiometry="1"
constant="true"/>
            </listOfReactants>
            <listOfProducts>
                  <speciesReference species="M_h_c" stoichiometry="1"
constant="true"/>
                  <speciesReference species="M_val__L_c" stoichiometry="1"
constant="true"/>
            </listOfProducts>
            <fbc:geneProductAssociation>
                  <fbc:or>
                        <fbc:geneProductRef fbc:geneProduct="SAUSA300_1300"/>
                        <fbc:geneProductRef fbc:geneProduct="SAUSA300_0188"/>
                        <fbc:geneProductRef fbc:geneProduct="SAUSA300_0306"/>
                  </fbc:or>
            </fbc:geneProductAssociation>
      </reaction>

      <reaction metaid="R_VALTA" id="R_VALTA" name="valine transaminase"
reversible="true" fast="false" fbc:lowerFluxBound="rev_lb"
fbc:upperFluxBound="rev_ub">
            <listOfReactants>
                  <speciesReference species="M_akg_c" stoichiometry="1"
constant="true"/>
                  <speciesReference species="M_val__L_c" stoichiometry="1"
constant="true"/>
            </listOfReactants>
            <listOfProducts>
                  <speciesReference species="M_glu__L_c" stoichiometry="1"
constant="true"/>
                  <speciesReference species="M_3mob_c" stoichiometry="1"
constant="true"/>
            </listOfProducts>
            <fbc:geneProductAssociation>
                  <fbc:geneProductRef fbc:geneProduct="SAUSA300_0539"/>
            </fbc:geneProductAssociation>
      </reaction>

      <reaction metaid="R_XANt" id="R_XANt" name="xanthine reversible
transport" reversible="false" fast="false" fbc:lowerFluxBound="irr_lb"
fbc:upperFluxBound="irr_ub">
            <listOfReactants>
                  <speciesReference species="M_xan_e" stoichiometry="1"
constant="true"/>
            </listOfReactants>
            <listOfProducts>
                  <speciesReference species="M_xan_c" stoichiometry="1"
constant="true"/>
            </listOfProducts>
            <fbc:geneProductAssociation>
                  <fbc:geneProductRef fbc:geneProduct="SAUSA300_0387"/>
            </fbc:geneProductAssociation>
```

```
      </reaction>

      <reaction metaid="R_XANt2" id="R_XANt2" name="xanthine transport in via
proton symport" reversible="false" fast="false" fbc:lowerFluxBound="irr_lb"
fbc:upperFluxBound="irr_ub">
            <listOfReactants>
                  <speciesReference species="M_xan_e" stoichiometry="1"
constant="true"/>
                  <speciesReference species="M_h_e" stoichiometry="1"
constant="true"/>
            </listOfReactants>
            <listOfProducts>
                  <speciesReference species="M_h_c" stoichiometry="1"
constant="true"/>
                  <speciesReference species="M_xan_c" stoichiometry="1"
constant="true"/>
            </listOfProducts>
            <fbc:geneProductAssociation>
                  <fbc:geneProductRef fbc:geneProduct="SAUSA300_0387"/>
            </fbc:geneProductAssociation>
      </reaction>

      <reaction metaid="R_XPPT" id="R_XPPT" name="xanthine
phosphoribosyltransferase" reversible="false" fast="false"
fbc:lowerFluxBound="irr_lb" fbc:upperFluxBound="irr_ub">
            <listOfReactants>
                  <speciesReference species="M_xan_c" stoichiometry="1"
constant="true"/>
                  <speciesReference species="M_prpp_c" stoichiometry="1"
constant="true"/>
            </listOfReactants>
            <listOfProducts>
                  <speciesReference species="M_ppi_c" stoichiometry="1"
constant="true"/>
                  <speciesReference species="M_xmp_c" stoichiometry="1"
constant="true"/>
            </listOfProducts>
            <fbc:geneProductAssociation>
                  <fbc:or>
                        <fbc:geneProductRef fbc:geneProduct="SAUSA300_0488"/>
                        <fbc:geneProductRef fbc:geneProduct="SAUSA300_0386"/>
                  </fbc:or>
            </fbc:geneProductAssociation>
      </reaction>

      <reaction metaid="R_XTSNH" id="R_XTSNH" name="Xanthosine hydrolase"
reversible="false" fast="false" fbc:lowerFluxBound="irr_lb"
fbc:upperFluxBound="irr_ub">
            <listOfReactants>
                  <speciesReference species="M_h2o_c" stoichiometry="1"
constant="true"/>
                  <speciesReference species="M_xtsn_c" stoichiometry="1"
constant="true"/>
            </listOfReactants>
            <listOfProducts>
                  <speciesReference species="M_xan_c" stoichiometry="1"
constant="true"/>
```

```
                  <speciesReference species="M_rib__D_c" stoichiometry="1"
constant="true"/>
            </listOfProducts>
            <fbc:geneProductAssociation>
                  <fbc:or>
                        <fbc:geneProductRef fbc:geneProduct="SAUSA300_2234"/>
                        <fbc:geneProductRef fbc:geneProduct="SAUSA300_0237"/>
                  </fbc:or>
            </fbc:geneProductAssociation>
      </reaction>

      <reaction metaid="R_XYLOD" id="R_XYLOD" name="Xylitol NAD 2-
oxidoreductase D-xylulose-forming " reversible="true" fast="false"
fbc:lowerFluxBound="rev_lb" fbc:upperFluxBound="rev_ub">
            <listOfReactants>
                  <speciesReference species="M_nad_c" stoichiometry="1"
constant="true"/>
                  <speciesReference species="M_xylt_c" stoichiometry="1"
constant="true"/>
            </listOfReactants>
            <listOfProducts>
                  <speciesReference species="M_h_c" stoichiometry="1"
constant="true"/>
                  <speciesReference species="M_nadh_c" stoichiometry="1"
constant="true"/>
                  <speciesReference species="M_rbl__L_c" stoichiometry="1"
constant="true"/>
            </listOfProducts>
            <fbc:geneProductAssociation>
                  <fbc:geneProductRef fbc:geneProduct="SAUSA300_0244"/>
            </fbc:geneProductAssociation>
      </reaction>

      <reaction metaid="R_YUMPS" id="R_YUMPS" name="yUMP synthetase"
reversible="true" fast="false" fbc:lowerFluxBound="rev_lb"
fbc:upperFluxBound="rev_ub">
            <listOfReactants>
                  <speciesReference species="M_r5p_c" stoichiometry="1"
constant="true"/>
                  <speciesReference species="M_ura_c" stoichiometry="1"
constant="true"/>
            </listOfReactants>
            <listOfProducts>
                  <speciesReference species="M_h2o_c" stoichiometry="1"
constant="true"/>
                  <speciesReference species="M_psd5p_c" stoichiometry="1"
constant="true"/>
            </listOfProducts>
            <fbc:geneProductAssociation>
                  <fbc:or>
                        <fbc:and>
                              <fbc:geneProductRef
fbc:geneProduct="SAUSA300_1090"/>
                              <fbc:geneProductRef
fbc:geneProduct="SAUSA300_1443"/>
                              <fbc:geneProductRef
fbc:geneProduct="SAUSA300_1699"/>
                        </fbc:and>
```

```
                        <fbc:and>
                              <fbc:geneProductRef
fbc:geneProduct="SAUSA300_2173"/>
                              <fbc:geneProductRef
fbc:geneProduct="SAUSA300_1164"/>
                        </fbc:and>
                  </fbc:or>
            </fbc:geneProductAssociation>
      </reaction>

      <reaction metaid="R_Zn2tex" id="R_Zn2tex" name="zinc (Zn+2) transport
via diffusion (extracellular to periplasm)" reversible="false" fast="false"
fbc:lowerFluxBound="irr_lb" fbc:upperFluxBound="irr_ub">
            <listOfReactants>
                  <speciesReference species="M_zn2_e" stoichiometry="1"
constant="true"/>
            </listOfReactants>
            <listOfProducts>
                  <speciesReference species="M_zn2_c" stoichiometry="1"
constant="true"/>
            </listOfProducts>
      </reaction>

      <reaction metaid="R_ZNabc" id="R_ZNabc" name="zinc transport via ABC
system" reversible="false" fast="false" fbc:lowerFluxBound="irr_lb"
fbc:upperFluxBound="irr_ub">
            <listOfReactants>
                  <speciesReference species="M_h2o_c" stoichiometry="1"
constant="true"/>
                  <speciesReference species="M_atp_c" stoichiometry="1"
constant="true"/>
                  <speciesReference species="M_zn2_e" stoichiometry="1"
constant="true"/>
            </listOfReactants>
            <listOfProducts>
                  <speciesReference species="M_h_c" stoichiometry="1"
constant="true"/>
                  <speciesReference species="M_pi_c" stoichiometry="1"
constant="true"/>
                  <speciesReference species="M_adp_c" stoichiometry="1"
constant="true"/>
                  <speciesReference species="M_zn2_c" stoichiometry="1"
constant="true"/>
            </listOfProducts>
            <fbc:geneProductAssociation>
                  <fbc:or>
                        <fbc:and>
                              <fbc:geneProductRef
fbc:geneProduct="SAUSA300_2351"/>
                              <fbc:geneProductRef
fbc:geneProduct="SAUSA300_1516"/>
                              <fbc:geneProductRef
fbc:geneProduct="SAUSA300_1515"/>
                        </fbc:and>
                        <fbc:and>
                              <fbc:geneProductRef
fbc:geneProduct="SAUSA300_2407"/>
```

```
                              <fbc:geneProductRef
fbc:geneProduct="SAUSA300_2408"/>
                              <fbc:geneProductRef
fbc:geneProduct="SAUSA300_2409"/>
                              <fbc:geneProductRef
fbc:geneProduct="SAUSA300_2410"/>
                              <fbc:geneProductRef
fbc:geneProduct="SAUSA300_2411"/>
                        </fbc:and>
                  </fbc:or>
            </fbc:geneProductAssociation>
      </reaction>

      <reaction metaid="R_PGLYST" id="R_PGLYST" name="Phosphatidylglycerol
lysyltransferase" reversible="false" fast="false" fbc:lowerFluxBound="irr_lb"
fbc:upperFluxBound="irr_ub">
            <listOfReactants>
                  <speciesReference species="M_lys__L_c" stoichiometry="1"
constant="true"/>
                  <speciesReference species="M_pg_SA_c" stoichiometry="1"
constant="true"/>
            </listOfReactants>
            <listOfProducts>
                  <speciesReference species="M_pglys_SA_c" stoichiometry="1"
constant="true"/>
            </listOfProducts>
            <fbc:geneProductAssociation>
                  <fbc:geneProductRef fbc:geneProduct="SAUSA300_1255"/>
            </fbc:geneProductAssociation>
      </reaction>

      <reaction metaid="R_LIPAMPL" id="R_LIPAMPL" name="Lipoyl-adenylate
protein ligase" reversible="false" fast="false" fbc:lowerFluxBound="irr_lb"
fbc:upperFluxBound="irr_ub">
            <listOfReactants>
                  <speciesReference species="M_lipoamp_c" stoichiometry="1"
constant="true"/>
            </listOfReactants>
            <listOfProducts>
                  <speciesReference species="M_amp_c" stoichiometry="1"
constant="true"/>
                  <speciesReference species="M_lipopb_c" stoichiometry="1"
constant="true"/>
            </listOfProducts>
            <fbc:geneProductAssociation>
                  <fbc:or>
                        <fbc:geneProductRef fbc:geneProduct="SAUSA300_0930"/>
                        <fbc:geneProductRef fbc:geneProduct="SAUSA300_0328"/>
                  </fbc:or>
            </fbc:geneProductAssociation>
      </reaction>

      <reaction metaid="R_LIPOPBt" id="R_LIPOPBt" name="Lipoate transport via
proton symport" reversible="false" fast="false" fbc:lowerFluxBound="irr_lb"
fbc:upperFluxBound="irr_ub">
            <listOfReactants>
                  <speciesReference species="M_h_e" stoichiometry="1"
constant="true"/>
```

```
                  <speciesReference species="M_lipoate_e" stoichiometry="1"
constant="true"/>
            </listOfReactants>
            <listOfProducts>
                  <speciesReference species="M_h_c" stoichiometry="1"
constant="true"/>
                  <speciesReference species="M_lipoate_c" stoichiometry="1"
constant="true"/>
            </listOfProducts>
            <fbc:geneProductAssociation>
                  <fbc:or>
                        <fbc:geneProductRef fbc:geneProduct="SAUSA300_0930"/>
                        <fbc:geneProductRef fbc:geneProduct="SAUSA300_0328"/>
                  </fbc:or>
            </fbc:geneProductAssociation>
      </reaction>

      <reaction metaid="R_LIPOS" id="R_LIPOS" name="Lipoate synthase"
reversible="false" fast="false" fbc:lowerFluxBound="irr_lb"
fbc:upperFluxBound="irr_ub">
            <listOfReactants>
                  <speciesReference species="M_4fe4s_c" stoichiometry="1"
constant="true"/>
                  <speciesReference species="M_amet_c" stoichiometry="2.0"
constant="true"/>
                  <speciesReference species="M_h_c" stoichiometry="1"
constant="true"/>
                  <speciesReference species="M_nad_c" stoichiometry="1"
constant="true"/>
                  <speciesReference species="M_octapb_c" stoichiometry="1"
constant="true"/>
            </listOfReactants>
            <listOfProducts>
                  <speciesReference species="M_2fe2s_c" stoichiometry="1"
constant="true"/>
                  <speciesReference species="M_dad__5_c" stoichiometry="2.0"
constant="true"/>
                  <speciesReference species="M_fe2_c" stoichiometry="2.0"
constant="true"/>
                  <speciesReference species="M_lipopb_c" stoichiometry="1"
constant="true"/>
                  <speciesReference species="M_met__L_c" stoichiometry="2.0"
constant="true"/>
                  <speciesReference species="M_nadh_c" stoichiometry="1"
constant="true"/>
            </listOfProducts>
            <fbc:geneProductAssociation>
                  <fbc:geneProductRef fbc:geneProduct="SAUSA300_0829"/>
            </fbc:geneProductAssociation>
      </reaction>

      <reaction metaid="R_S2FE2SR" id="R_S2FE2SR" name="SUF [2Fe-2S]
regeneration" reversible="false" fast="false" fbc:lowerFluxBound="irr_lb"
fbc:upperFluxBound="irr_ub">
            <listOfReactants>
                  <speciesReference species="M_2fe1s_c" stoichiometry="1"
constant="true"/>
```

```
                  <speciesReference species="M_atp_c" stoichiometry="1"
constant="true"/>
                  <speciesReference species="M_h2o_c" stoichiometry="1"
constant="true"/>
                  <speciesReference species="M_sufbcd_c" stoichiometry="1"
constant="true"/>
                  <speciesReference species="M_sufsesh_c" stoichiometry="1"
constant="true"/>
            </listOfReactants>
            <listOfProducts>
                  <speciesReference species="M_adp_c" stoichiometry="1"
constant="true"/>
                  <speciesReference species="M_h_c" stoichiometry="5.0"
constant="true"/>
                  <speciesReference species="M_pi_c" stoichiometry="1"
constant="true"/>
                  <speciesReference species="M_sufbcd_2fe2s_c"
stoichiometry="1" constant="true"/>
                  <speciesReference species="M_sufse_c" stoichiometry="1"
constant="true"/>
            </listOfProducts>
            <fbc:geneProductAssociation>
                  <fbc:and>
                        <fbc:geneProductRef fbc:geneProduct="SAUSA300_0820"/>
                        <fbc:geneProductRef fbc:geneProduct="SAUSA300_0821"/>
                        <fbc:geneProductRef fbc:geneProduct="SAUSA300_0819"/>
                        <fbc:geneProductRef fbc:geneProduct="SAUSA300_0822"/>
                        <fbc:geneProductRef fbc:geneProduct="SAUSA300_0818"/>
                  </fbc:and>
            </fbc:geneProductAssociation>
      </reaction>

      <reaction metaid="R_S2FE2SS2" id="R_S2FE2SS2" name="SUF [2Fe-2S]
Synthesis II" reversible="false" fast="false" fbc:lowerFluxBound="irr_lb"
fbc:upperFluxBound="irr_ub">
            <listOfReactants>
                  <speciesReference species="M_atp_c" stoichiometry="1"
constant="true"/>
                  <speciesReference species="M_fadh2_c" stoichiometry="1"
constant="true"/>
                  <speciesReference species="M_fe2_c" stoichiometry="2.0"
constant="true"/>
                  <speciesReference species="M_h2o_c" stoichiometry="1"
constant="true"/>
                  <speciesReference species="M_sufbcd_2fe2s_c"
stoichiometry="1" constant="true"/>
                  <speciesReference species="M_sufsesh_c" stoichiometry="2.0"
constant="true"/>
            </listOfReactants>
            <listOfProducts>
                  <speciesReference species="M_adp_c" stoichiometry="1"
constant="true"/>
                  <speciesReference species="M_fad_c" stoichiometry="1"
constant="true"/>
                  <speciesReference species="M_h_c" stoichiometry="7.0"
constant="true"/>
                  <speciesReference species="M_pi_c" stoichiometry="1"
constant="true"/>
```

```
                  <speciesReference species="M_sufbcd_2fe2s2_c"
stoichiometry="1" constant="true"/>
                  <speciesReference species="M_sufse_c" stoichiometry="2.0"
constant="true"/>
            </listOfProducts>
            <fbc:geneProductAssociation>
                  <fbc:and>
                        <fbc:geneProductRef fbc:geneProduct="SAUSA300_0820"/>
                        <fbc:geneProductRef fbc:geneProduct="SAUSA300_0821"/>
                        <fbc:geneProductRef fbc:geneProduct="SAUSA300_0819"/>
                        <fbc:geneProductRef fbc:geneProduct="SAUSA300_0822"/>
                        <fbc:geneProductRef fbc:geneProduct="SAUSA300_0818"/>
                  </fbc:and>
            </fbc:geneProductAssociation>
      </reaction>

      <reaction metaid="R_S2FE2ST" id="R_S2FE2ST" name="SUF [2Fe-2S]
Transfer" reversible="false" fast="false" fbc:lowerFluxBound="irr_lb"
fbc:upperFluxBound="irr_ub">
            <listOfReactants>
                  <speciesReference species="M_h_c" stoichiometry="4.0"
constant="true"/>
                  <speciesReference species="M_sufbcd_2fe2s_c"
stoichiometry="1" constant="true"/>
            </listOfReactants>
            <listOfProducts>
                  <speciesReference species="M_2fe2s_c" stoichiometry="1"
constant="true"/>
                  <speciesReference species="M_sufbcd_c" stoichiometry="1"
constant="true"/>
            </listOfProducts>
            <fbc:geneProductAssociation>
                  <fbc:and>
                        <fbc:or>
                              <fbc:geneProductRef
fbc:geneProduct="SAUSA300_0843"/>
                              <fbc:geneProductRef
fbc:geneProduct="SAUSA300_0839"/>
                              <fbc:geneProductRef
fbc:geneProduct="SAUSA300_0875"/>
                        </fbc:or>
                        <fbc:geneProductRef fbc:geneProduct="SAUSA300_0819"/>
                        <fbc:geneProductRef fbc:geneProduct="SAUSA300_0822"/>
                        <fbc:geneProductRef fbc:geneProduct="SAUSA300_0818"/>
                  </fbc:and>
            </fbc:geneProductAssociation>
      </reaction>

      <reaction metaid="R_S4FE4SR" id="R_S4FE4SR" name="SUF [4Fe-4S]
Reduction" reversible="false" fast="false" fbc:lowerFluxBound="irr_lb"
fbc:upperFluxBound="irr_ub">
            <listOfReactants>
                  <speciesReference species="M_fadh2_c" stoichiometry="1"
constant="true"/>
                  <speciesReference species="M_h_c" stoichiometry="2.0"
constant="true"/>
                  <speciesReference species="M_sufbcd_2fe2s2_c"
stoichiometry="1" constant="true"/>
```

```xml
            </listOfReactants>
            <listOfProducts>
                  <speciesReference species="M_fad_c" stoichiometry="1"
constant="true"/>
                  <speciesReference species="M_sufbcd_4fe4s_c"
stoichiometry="1" constant="true"/>
            </listOfProducts>
            <fbc:geneProductAssociation>
                  <fbc:and>
                        <fbc:geneProductRef fbc:geneProduct="SAUSA300_0819"/>
                        <fbc:geneProductRef fbc:geneProduct="SAUSA300_0822"/>
                        <fbc:geneProductRef fbc:geneProduct="SAUSA300_0818"/>
                  </fbc:and>
            </fbc:geneProductAssociation>
      </reaction>

      <reaction metaid="R_S4FE4ST" id="R_S4FE4ST" name="SUF [4Fe-4S]
Transfer" reversible="false" fast="false" fbc:lowerFluxBound="irr_lb"
fbc:upperFluxBound="irr_ub">
            <listOfReactants>
                  <speciesReference species="M_h_c" stoichiometry="4.0"
constant="true"/>
                  <speciesReference species="M_sufbcd_4fe4s_c"
stoichiometry="1" constant="true"/>
            </listOfReactants>
            <listOfProducts>
                  <speciesReference species="M_4fe4s_c" stoichiometry="1"
constant="true"/>
                  <speciesReference species="M_sufbcd_c" stoichiometry="1"
constant="true"/>
            </listOfProducts>
            <fbc:geneProductAssociation>
                  <fbc:and>
                        <fbc:or>
                              <fbc:geneProductRef
fbc:geneProduct="SAUSA300_0843"/>
                              <fbc:geneProductRef
fbc:geneProduct="SAUSA300_0839"/>
                              <fbc:geneProductRef
fbc:geneProduct="SAUSA300_0875"/>
                        </fbc:or>
                        <fbc:geneProductRef fbc:geneProduct="SAUSA300_0819"/>
                        <fbc:geneProductRef fbc:geneProduct="SAUSA300_0822"/>
                        <fbc:geneProductRef fbc:geneProduct="SAUSA300_0818"/>
                  </fbc:and>
            </fbc:geneProductAssociation>
      </reaction>

      <reaction metaid="R_temp_sink" id="R_temp_sink" name="sink for
consumption of FeS protein" reversible="true" fast="false"
fbc:lowerFluxBound="rev_lb" fbc:upperFluxBound="rev_ub">
            <listOfReactants>
                  <speciesReference species="M_2fe1s_c" stoichiometry="1e-05"
constant="true"/>
            </listOfReactants>
      </reaction>
```

```
      <reaction metaid="R_BTS5" id="R_BTS5" name="Biotin synthase"
reversible="false" fast="false" fbc:lowerFluxBound="irr_lb"
fbc:upperFluxBound="irr_ub">
            <listOfReactants>
                  <speciesReference species="M_2fe2s_c" stoichiometry="1"
constant="true"/>
                  <speciesReference species="M_amet_c" stoichiometry="1"
constant="true"/>
                  <speciesReference species="M_dtbt_c" stoichiometry="1"
constant="true"/>
            </listOfReactants>
            <listOfProducts>
                  <speciesReference species="M_2fe1s_c" stoichiometry="1"
constant="true"/>
                  <speciesReference species="M_btn_c" stoichiometry="1"
constant="true"/>
                  <speciesReference species="M_dad__5_c" stoichiometry="1"
constant="true"/>
                  <speciesReference species="M_h_c" stoichiometry="1"
constant="true"/>
                  <speciesReference species="M_met__L_c" stoichiometry="1"
constant="true"/>
            </listOfProducts>
            <fbc:geneProductAssociation>
                  <fbc:geneProductRef fbc:geneProduct="B7H15_RS13455"/>
            </fbc:geneProductAssociation>
      </reaction>

      <reaction metaid="R_OCTNLL" id="R_OCTNLL" name="Octanoate
non__Lipoylated apo domain ligase" reversible="false" fast="false"
fbc:lowerFluxBound="irr_lb" fbc:upperFluxBound="irr_ub">
            <listOfReactants>
                  <speciesReference species="M_atp_c" stoichiometry="1"
constant="true"/>
                  <speciesReference species="M_h_c" stoichiometry="1"
constant="true"/>
                  <speciesReference species="M_octa_c" stoichiometry="1"
constant="true"/>
            </listOfReactants>
            <listOfProducts>
                  <speciesReference species="M_amp_c" stoichiometry="1"
constant="true"/>
                  <speciesReference species="M_octapb_c" stoichiometry="1"
constant="true"/>
                  <speciesReference species="M_ppi_c" stoichiometry="1"
constant="true"/>
            </listOfProducts>
            <fbc:geneProductAssociation>
                  <fbc:geneProductRef fbc:geneProduct="SAUSA300_0571"/>
            </fbc:geneProductAssociation>
      </reaction>

      <reaction metaid="R_FA80ACPHi" id="R_FA80ACPHi" name="Fatty-acyl-ACP
hydrolase" reversible="false" fast="false" fbc:lowerFluxBound="irr_lb"
fbc:upperFluxBound="irr_ub">
            <listOfReactants>
                  <speciesReference species="M_h2o_c" stoichiometry="1"
constant="true"/>
```

```
                  <speciesReference species="M_ocACP_c" stoichiometry="1"
constant="true"/>
            </listOfReactants>
            <listOfProducts>
                  <speciesReference species="M_ACP_c" stoichiometry="1"
constant="true"/>
                  <speciesReference species="M_h_c" stoichiometry="1"
constant="true"/>
                  <speciesReference species="M_octa_c" stoichiometry="1"
constant="true"/>
            </listOfProducts>
            <fbc:geneProductAssociation>
                  <fbc:geneProductRef fbc:geneProduct="SAUSA300_1494"/>
            </fbc:geneProductAssociation>
      </reaction>

      <reaction metaid="R_5DOAN" id="R_5DOAN" name="5'__Deoxyadenosine
nuclosidase" reversible="false" fast="false" fbc:lowerFluxBound="irr_lb"
fbc:upperFluxBound="irr_ub">
            <listOfReactants>
                  <speciesReference species="M_dad__5_c" stoichiometry="1"
constant="true"/>
                  <speciesReference species="M_h2o_c" stoichiometry="1"
constant="true"/>
            </listOfReactants>
            <listOfProducts>
                  <speciesReference species="M_5drib_c" stoichiometry="1"
constant="true"/>
                  <speciesReference species="M_ade_c" stoichiometry="1"
constant="true"/>
            </listOfProducts>
            <fbc:geneProductAssociation>
                  <fbc:geneProductRef fbc:geneProduct="SAUSA300_1558"/>
            </fbc:geneProductAssociation>
      </reaction>

      <reaction metaid="R_SCYSDS" id="R_SCYSDS" name="SUF Cysteine
desulfuration" reversible="false" fast="false" fbc:lowerFluxBound="irr_lb"
fbc:upperFluxBound="irr_ub">
            <listOfReactants>
                  <speciesReference species="M_cys__L_c" stoichiometry="1"
constant="true"/>
                  <speciesReference species="M_sufse_c" stoichiometry="1"
constant="true"/>
            </listOfReactants>
            <listOfProducts>
                  <speciesReference species="M_ala__L_c" stoichiometry="1"
constant="true"/>
                  <speciesReference species="M_sufsesh_c" stoichiometry="1"
constant="true"/>
            </listOfProducts>
            <fbc:geneProductAssociation>
                  <fbc:and>
                        <fbc:geneProductRef fbc:geneProduct="SAUSA300_0820"/>
                        <fbc:geneProductRef fbc:geneProduct="SAUSA300_0821"/>
                  </fbc:and>
            </fbc:geneProductAssociation>
      </reaction>
```

```xml
      <reaction metaid="R_HPIMHL" id="R_HPIMHL" name="3-Hydroxypimeloyl-ACP
methyl ester hydro__Lyase" reversible="false" fast="false"
fbc:lowerFluxBound="irr_lb" fbc:upperFluxBound="irr_ub">
            <listOfReactants>
                  <speciesReference species="M_3hpmeACP_c" stoichiometry="1"
constant="true"/>
            </listOfReactants>
            <listOfProducts>
                  <speciesReference species="M_epmeACP_c" stoichiometry="1"
constant="true"/>
                  <speciesReference species="M_h2o_c" stoichiometry="1"
constant="true"/>
            </listOfProducts>
            <fbc:geneProductAssociation>
                  <fbc:geneProductRef fbc:geneProduct="SAUSA300_2054"/>
            </fbc:geneProductAssociation>
      </reaction>

      <reaction metaid="R_KGLACPR" id="R_KGLACPR" name="3-Ketoglutaryl-ACP
methyl ester reduction" reversible="false" fast="false"
fbc:lowerFluxBound="irr_lb" fbc:upperFluxBound="irr_ub">
            <listOfReactants>
                  <speciesReference species="M_h_c" stoichiometry="1"
constant="true"/>
                  <speciesReference species="M_nadph_c" stoichiometry="1"
constant="true"/>
                  <speciesReference species="M_skgmeACP_c" stoichiometry="1"
constant="true"/>
            </listOfReactants>
            <listOfProducts>
                  <speciesReference species="M_3hgmeACP_c" stoichiometry="1"
constant="true"/>
                  <speciesReference species="M_nadp_c" stoichiometry="1"
constant="true"/>
            </listOfProducts>
            <fbc:geneProductAssociation>
                  <fbc:geneProductRef fbc:geneProduct="SAUSA300_1124"/>
            </fbc:geneProductAssociation>
      </reaction>

      <reaction metaid="R_KPIMD" id="R_KPIMD" name="Enoylglutaryl-ACP methyl
ester C-acyltransferase decarboxylating" reversible="false" fast="false"
fbc:lowerFluxBound="irr_lb" fbc:upperFluxBound="irr_ub">
            <listOfReactants>
                  <speciesReference species="M_glmeACP_c" stoichiometry="1"
constant="true"/>
                  <speciesReference species="M_h_c" stoichiometry="1"
constant="true"/>
                  <speciesReference species="M_malACP_c" stoichiometry="1"
constant="true"/>
            </listOfReactants>
            <listOfProducts>
                  <speciesReference species="M_ACP_c" stoichiometry="1"
constant="true"/>
                  <speciesReference species="M_co2_c" stoichiometry="1"
constant="true"/>
```

```
                  <speciesReference species="M_skpmeACP_c" stoichiometry="1"
constant="true"/>
            </listOfProducts>
            <fbc:geneProductAssociation>
                  <fbc:and>
                        <fbc:geneProductRef fbc:geneProduct="SAUSA300_0885"/>
                        <fbc:geneProductRef fbc:geneProduct="SAUSA300_0886"/>
                  </fbc:and>
            </fbc:geneProductAssociation>
      </reaction>

      <reaction metaid="R_KPIMR" id="R_KPIMR" name="3-Ketopimeloyl-ACP methyl
ester reductase" reversible="false" fast="false" fbc:lowerFluxBound="irr_lb"
fbc:upperFluxBound="irr_ub">
            <listOfReactants>
                  <speciesReference species="M_h_c" stoichiometry="1"
constant="true"/>
                  <speciesReference species="M_nadph_c" stoichiometry="1"
constant="true"/>
                  <speciesReference species="M_skpmeACP_c" stoichiometry="1"
constant="true"/>
            </listOfReactants>
            <listOfProducts>
                  <speciesReference species="M_3hpmeACP_c" stoichiometry="1"
constant="true"/>
                  <speciesReference species="M_nadp_c" stoichiometry="1"
constant="true"/>
            </listOfProducts>
            <fbc:geneProductAssociation>
                  <fbc:geneProductRef fbc:geneProduct="SAUSA300_1124"/>
            </fbc:geneProductAssociation>
      </reaction>

      <reaction metaid="R_PIMR" id="R_PIMR" name="Enoylpimeloyl-ACP methyl
ester reduction" reversible="false" fast="false" fbc:lowerFluxBound="irr_lb"
fbc:upperFluxBound="irr_ub">
            <listOfReactants>
                  <speciesReference species="M_epmeACP_c" stoichiometry="1"
constant="true"/>
                  <speciesReference species="M_h_c" stoichiometry="1"
constant="true"/>
                  <speciesReference species="M_nadph_c" stoichiometry="1"
constant="true"/>
            </listOfReactants>
            <listOfProducts>
                  <speciesReference species="M_nadp_c" stoichiometry="1"
constant="true"/>
                  <speciesReference species="M_pmlmeACP_c" stoichiometry="1"
constant="true"/>
            </listOfProducts>
            <fbc:geneProductAssociation>
                  <fbc:geneProductRef fbc:geneProduct="SAUSA300_0912"/>
            </fbc:geneProductAssociation>
      </reaction>

      <reaction metaid="R_EGLACPR" id="R_EGLACPR" name="Enoylglutaryl-ACP
methyl ester reduction" reversible="false" fast="false"
fbc:lowerFluxBound="irr_lb" fbc:upperFluxBound="irr_ub">
```

```
            <listOfReactants>
                  <speciesReference species="M_egmeACP_c" stoichiometry="1"
constant="true"/>
                  <speciesReference species="M_h_c" stoichiometry="1"
constant="true"/>
                  <speciesReference species="M_nadph_c" stoichiometry="1"
constant="true"/>
            </listOfReactants>
            <listOfProducts>
                  <speciesReference species="M_glmeACP_c" stoichiometry="1"
constant="true"/>
                  <speciesReference species="M_nadp_c" stoichiometry="1"
constant="true"/>
            </listOfProducts>
            <fbc:geneProductAssociation>
                  <fbc:geneProductRef fbc:geneProduct="SAUSA300_0912"/>
            </fbc:geneProductAssociation>
      </reaction>

      <reaction metaid="R_HGLACPHL" id="R_HGLACPHL" name="3-Hydroxyglutaryl-
ACP methyl ester hydro__Lyase" reversible="false" fast="false"
fbc:lowerFluxBound="irr_lb" fbc:upperFluxBound="irr_ub">
            <listOfReactants>
                  <speciesReference species="M_3hgmeACP_c" stoichiometry="1"
constant="true"/>
            </listOfReactants>
            <listOfProducts>
                  <speciesReference species="M_egmeACP_c" stoichiometry="1"
constant="true"/>
                  <speciesReference species="M_h2o_c" stoichiometry="1"
constant="true"/>
            </listOfProducts>
            <fbc:geneProductAssociation>
                  <fbc:geneProductRef fbc:geneProduct="SAUSA300_2054"/>
            </fbc:geneProductAssociation>
      </reaction>

      <reaction metaid="R_MALACPD" id="R_MALACPD" name="Malonyl-ACP methyl
ester C-acyltransferase decarboxylating" reversible="false" fast="false"
fbc:lowerFluxBound="irr_lb" fbc:upperFluxBound="irr_ub">
            <listOfReactants>
                  <speciesReference species="M_h_c" stoichiometry="1"
constant="true"/>
                  <speciesReference species="M_malACP_c" stoichiometry="1"
constant="true"/>
                  <speciesReference species="M_malmeACP_c" stoichiometry="1"
constant="true"/>
            </listOfReactants>
            <listOfProducts>
                  <speciesReference species="M_ACP_c" stoichiometry="1"
constant="true"/>
                  <speciesReference species="M_co2_c" stoichiometry="1"
constant="true"/>
                  <speciesReference species="M_skgmeACP_c" stoichiometry="1"
constant="true"/>
            </listOfProducts>
            <fbc:geneProductAssociation>
                  <fbc:and>
```

```
                        <fbc:geneProductRef fbc:geneProduct="SAUSA300_0885"/>
                        <fbc:geneProductRef fbc:geneProduct="SAUSA300_0886"/>
                  </fbc:and>
            </fbc:geneProductAssociation>
      </reaction>

      <reaction metaid="R_MALCOAM" id="R_MALCOAM" name="Malonyl-coa
methylation" reversible="false" fast="false" fbc:lowerFluxBound="irr_lb"
fbc:upperFluxBound="irr_ub">
            <listOfReactants>
                  <speciesReference species="M_amet_c" stoichiometry="1"
constant="true"/>
                  <speciesReference species="M_malACP_c" stoichiometry="1"
constant="true"/>
            </listOfReactants>
            <listOfProducts>
                  <speciesReference species="M_ahcys_c" stoichiometry="1"
constant="true"/>
                  <speciesReference species="M_malmeACP_c" stoichiometry="1"
constant="true"/>
            </listOfProducts>
      </reaction>

      <reaction metaid="R_DM_amob_c" id="R_DM_amob_c" name="Sink needed to
allow S-Adenosyl-4-methylthio-2-oxobutanoate to leave system"
reversible="false" fast="false" fbc:lowerFluxBound="irr_lb"
fbc:upperFluxBound="irr_ub">
            <listOfReactants>
                  <speciesReference species="M_amob_c" stoichiometry="1"
constant="true"/>
            </listOfReactants>
      </reaction>

      <reaction metaid="R_AOXSr2" id="R_AOXSr2" name="8-amino-7-oxononanoate
synthase" reversible="true" fast="false" fbc:lowerFluxBound="rev_lb"
fbc:upperFluxBound="rev_ub">
            <listOfReactants>
                  <speciesReference species="M_ala__L_c" stoichiometry="1"
constant="true"/>
                  <speciesReference species="M_pmACP_c" stoichiometry="1"
constant="true"/>
            </listOfReactants>
            <listOfProducts>
                  <speciesReference species="M_8aonn_c" stoichiometry="1"
constant="true"/>
                  <speciesReference species="M_co2_c" stoichiometry="1"
constant="true"/>
                  <speciesReference species="M_ACP_c" stoichiometry="1"
constant="true"/>
            </listOfProducts>
            <fbc:geneProductAssociation>
                  <fbc:geneProductRef fbc:geneProduct="B7H15_RS13450"/>
            </fbc:geneProductAssociation>
      </reaction>

      <reaction metaid="R_CHCOAL2" id="R_CHCOAL2" name="6-carboxyhexanoate-
CoA ligase" reversible="false" fast="false" fbc:lowerFluxBound="irr_lb"
fbc:upperFluxBound="irr_ub">
```

```
            <listOfReactants>
                  <speciesReference species="M_h2o_c" stoichiometry="1"
constant="true"/>
                  <speciesReference species="M_pmlmeACP_c" stoichiometry="1"
constant="true"/>
            </listOfReactants>
            <listOfProducts>
                  <speciesReference species="M_meoh_c" stoichiometry="1"
constant="true"/>
                  <speciesReference species="M_pmACP_c" stoichiometry="1"
constant="true"/>
            </listOfProducts>
            <fbc:geneProductAssociation>
                  <fbc:geneProductRef fbc:geneProduct="B7H15_RS13445"/>
            </fbc:geneProductAssociation>
      </reaction>

      <reaction metaid="R_DATPHs" id="R_DATPHs" name="DATP amine hydrolysis
(spontaneous)" reversible="false" fast="false" fbc:lowerFluxBound="irr_lb"
fbc:upperFluxBound="irr_ub">
            <listOfReactants>
                  <speciesReference species="M_datp_c" stoichiometry="1"
constant="true"/>
                  <speciesReference species="M_h2o_c" stoichiometry="1"
constant="true"/>
                  <speciesReference species="M_h_c" stoichiometry="1"
constant="true"/>
            </listOfReactants>
            <listOfProducts>
                  <speciesReference species="M_ditp_c" stoichiometry="1"
constant="true"/>
                  <speciesReference species="M_nh4_c" stoichiometry="1"
constant="true"/>
            </listOfProducts>
            <fbc:geneProductAssociation>
                  <fbc:geneProductRef fbc:geneProduct="s0001"/>
            </fbc:geneProductAssociation>
      </reaction>

      <reaction metaid="R_NTP11" id="R_NTP11" name="Nucleoside-triphosphatase
(dITP)" reversible="false" fast="false" fbc:lowerFluxBound="irr_lb"
fbc:upperFluxBound="irr_ub">
            <listOfReactants>
                  <speciesReference species="M_ditp_c" stoichiometry="1"
constant="true"/>
                  <speciesReference species="M_h2o_c" stoichiometry="1"
constant="true"/>
            </listOfReactants>
            <listOfProducts>
                  <speciesReference species="M_didp_c" stoichiometry="1"
constant="true"/>
                  <speciesReference species="M_h_c" stoichiometry="1"
constant="true"/>
                  <speciesReference species="M_pi_c" stoichiometry="1"
constant="true"/>
            </listOfProducts>
            <fbc:geneProductAssociation>
                  <fbc:geneProductRef fbc:geneProduct="SAUSA300_1050"/>
```

```
            </fbc:geneProductAssociation>
      </reaction>

      <reaction metaid="R_PAPT" id="R_PAPT" name="phospho-N-acetylmuramoyl-
pentapeptide-transferase" reversible="true" fast="false"
fbc:lowerFluxBound="rev_lb" fbc:upperFluxBound="rev_ub">
            <listOfReactants>
                  <speciesReference species="M_uamaglaa_c" stoichiometry="1"
constant="true"/>
                  <speciesReference species="M_udcpp_c" stoichiometry="1"
constant="true"/>
            </listOfReactants>
            <listOfProducts>
                  <speciesReference species="M_ump_c" stoichiometry="1"
constant="true"/>
                  <speciesReference species="M_updpamaglaa_c"
stoichiometry="1" constant="true"/>
            </listOfProducts>
            <fbc:geneProductAssociation>
                  <fbc:geneProductRef fbc:geneProduct="SAUSA300_1076"/>
            </fbc:geneProductAssociation>
      </reaction>

      <reaction metaid="R_UAATAAL" id="R_UAATAAL" name="UDP-N-acetylmuramoyl-
tripeptide-__D-alanyl__D-alanine ligase" reversible="false" fast="false"
fbc:lowerFluxBound="irr_lb" fbc:upperFluxBound="irr_ub">
            <listOfReactants>
                  <speciesReference species="M_alaala_c" stoichiometry="1"
constant="true"/>
                  <speciesReference species="M_atp_c" stoichiometry="1"
constant="true"/>
                  <speciesReference species="M_uamagl_c" stoichiometry="1"
constant="true"/>
            </listOfReactants>
            <listOfProducts>
                  <speciesReference species="M_adp_c" stoichiometry="1"
constant="true"/>
                  <speciesReference species="M_h_c" stoichiometry="1"
constant="true"/>
                  <speciesReference species="M_pi_c" stoichiometry="1"
constant="true"/>
                  <speciesReference species="M_uamaglaa_c" stoichiometry="1"
constant="true"/>
            </listOfProducts>
            <fbc:geneProductAssociation>
                  <fbc:or>
                        <fbc:geneProductRef fbc:geneProduct="SAUSA300_2038"/>
                        <fbc:geneProductRef fbc:geneProduct="SAUSA300_1873"/>
                  </fbc:or>
            </fbc:geneProductAssociation>
      </reaction>

      <reaction metaid="R_UAMAGLL" id="R_UAMAGLL" name="UDP-N-
acetylmuramoyl__L-alanyl__D-glutamate-__Lysine ligase" reversible="false"
fast="false" fbc:lowerFluxBound="irr_lb" fbc:upperFluxBound="irr_ub">
            <listOfReactants>
                  <speciesReference species="M_atp_c" stoichiometry="1"
constant="true"/>
```

```
                  <speciesReference species="M_lys__L_c" stoichiometry="1"
constant="true"/>
                  <speciesReference species="M_uamag_c" stoichiometry="1"
constant="true"/>
            </listOfReactants>
            <listOfProducts>
                  <speciesReference species="M_adp_c" stoichiometry="1"
constant="true"/>
                  <speciesReference species="M_h_c" stoichiometry="1"
constant="true"/>
                  <speciesReference species="M_pi_c" stoichiometry="1"
constant="true"/>
                  <speciesReference species="M_uamagl_c" stoichiometry="1"
constant="true"/>
            </listOfProducts>
            <fbc:geneProductAssociation>
                  <fbc:geneProductRef fbc:geneProduct="SAUSA300_0919"/>
            </fbc:geneProductAssociation>
      </reaction>

      <reaction metaid="R_LTAS" id="R_LTAS" name="lipoteichoic acid synthase"
reversible="false" fast="false" fbc:lowerFluxBound="irr_lb"
fbc:upperFluxBound="irr_ub">
            <listOfReactants>
                  <speciesReference species="M_3g12dgr_SA2_c"
stoichiometry="1" constant="true"/>
                  <speciesReference species="M_pg_SA_c" stoichiometry="20.0"
constant="true"/>
            </listOfReactants>
            <listOfProducts>
                  <speciesReference species="M_LTA_c" stoichiometry="1"
constant="true"/>
                  <speciesReference species="M_dag_SA_c" stoichiometry="20.0"
constant="true"/>
            </listOfProducts>
            <fbc:geneProductAssociation>
                  <fbc:geneProductRef fbc:geneProduct="SAUSA300_0703"/>
            </fbc:geneProductAssociation>
      </reaction>

      <reaction metaid="R_DAGK2" id="R_DAGK2" name="diacylglycerol kinase"
reversible="false" fast="false" fbc:lowerFluxBound="irr_lb"
fbc:upperFluxBound="irr_ub">
            <listOfReactants>
                  <speciesReference species="M_atp_c" stoichiometry="1"
constant="true"/>
                  <speciesReference species="M_dag_SA_c" stoichiometry="1"
constant="true"/>
            </listOfReactants>
            <listOfProducts>
                  <speciesReference species="M_adp_c" stoichiometry="1"
constant="true"/>
                  <speciesReference species="M_h_c" stoichiometry="1"
constant="true"/>
                  <speciesReference species="M_pa_SA_c" stoichiometry="1"
constant="true"/>
            </listOfProducts>
            <fbc:geneProductAssociation>
```

```
                <fbc:geneProductRef fbc:geneProduct="SAUSA300_1879"/>
            </fbc:geneProductAssociation>
        </reaction>

        <reaction metaid="R_ACPAL" id="R_ACPAL" name="D-alanine--[D-alanyl
carrier protein] ligase" reversible="false" fast="false"
fbc:lowerFluxBound="irr_lb" fbc:upperFluxBound="irr_ub">
            <listOfReactants>
                <speciesReference species="M_ACP_c" stoichiometry="1"
constant="true"/>
                <speciesReference species="M_ala__D_c" stoichiometry="1"
constant="true"/>
                <speciesReference species="M_atp_c" stoichiometry="1"
constant="true"/>
            </listOfReactants>
            <listOfProducts>
                <speciesReference species="M_alaACP_c" stoichiometry="1"
constant="true"/>
                <speciesReference species="M_amp_c" stoichiometry="1"
constant="true"/>
                <speciesReference species="M_ppi_c" stoichiometry="1"
constant="true"/>
            </listOfProducts>
            <fbc:geneProductAssociation>
                <fbc:and>
                    <fbc:geneProductRef fbc:geneProduct="SAUSA300_0837"/>
                    <fbc:geneProductRef fbc:geneProduct="SAUSA300_0835"/>
                </fbc:and>
            </fbc:geneProductAssociation>
        </reaction>

        <reaction metaid="R_LIP2GT" id="R_LIP2GT" name="N-acetylmuramoyl__L-
alanyl__D-glutamyl__L__Lysyl__D-alanyl__D-alanine__Diphosphoundecaprenyl-N-
acetylglucosamine:glycine transferase" reversible="false" fast="false"
fbc:lowerFluxBound="irr_lb" fbc:upperFluxBound="irr_ub">
            <listOfReactants>
                <speciesReference species="M_gly_c" stoichiometry="1"
constant="true"/>
                <speciesReference species="M_h_c" stoichiometry="2.0"
constant="true"/>
                <speciesReference species="M_lip2_c" stoichiometry="1"
constant="true"/>
            </listOfReactants>
            <listOfProducts>
                <speciesReference species="M_h2o_c" stoichiometry="1"
constant="true"/>
                <speciesReference species="M_lip2g_c" stoichiometry="1"
constant="true"/>
            </listOfProducts>
            <fbc:geneProductAssociation>
                <fbc:geneProductRef fbc:geneProduct="SAUSA300_1269"/>
            </fbc:geneProductAssociation>
        </reaction>

        <reaction metaid="R_LIP2GT2" id="R_LIP2GT2" name="N-acetylmuramoyl__L-
alanyl__D-glutamyl__L__Lysyl-(glycine)__D-alanyl__D-
alanine__Diphosphoundecaprenyl-N-acetylglucosamine:glycine transferase"
```

```
reversible="false" fast="false" fbc:lowerFluxBound="irr_lb"
fbc:upperFluxBound="irr_ub">
            <listOfReactants>
                  <speciesReference species="M_gly_c" stoichiometry="2.0"
constant="true"/>
                  <speciesReference species="M_lip2g_c" stoichiometry="1"
constant="true"/>
            </listOfReactants>
            <listOfProducts>
                  <speciesReference species="M_h2o_c" stoichiometry="2.0"
constant="true"/>
                  <speciesReference species="M_lip2g3_c" stoichiometry="1"
constant="true"/>
            </listOfProducts>
            <fbc:geneProductAssociation>
                  <fbc:geneProductRef fbc:geneProduct="SAUSA300_1270"/>
            </fbc:geneProductAssociation>
      </reaction>

      <reaction metaid="R_LIP2GT3" id="R_LIP2GT3" name="N-acetylmuramoyl__L-
alanyl__D-glutamyl__L__Lysyl-(glycine)3__D-alanyl__D-
alanine__Diphosphoundecaprenyl-N-acetylglucosamine:glycine transferase"
reversible="false" fast="false" fbc:lowerFluxBound="irr_lb"
fbc:upperFluxBound="irr_ub">
            <listOfReactants>
                  <speciesReference species="M_gly_c" stoichiometry="2.0"
constant="true"/>
                  <speciesReference species="M_lip2g3_c" stoichiometry="1"
constant="true"/>
            </listOfReactants>
            <listOfProducts>
                  <speciesReference species="M_h2o_c" stoichiometry="2.0"
constant="true"/>
                  <speciesReference species="M_pepm_c" stoichiometry="1"
constant="true"/>
            </listOfProducts>
            <fbc:geneProductAssociation>
                  <fbc:geneProductRef fbc:geneProduct="SAUSA300_2214"/>
            </fbc:geneProductAssociation>
      </reaction>

      <reaction metaid="R_PDTS" id="R_PDTS" name="peptidoglycan dimer
transpeptidation (short strand)" reversible="false" fast="false"
fbc:lowerFluxBound="irr_lb" fbc:upperFluxBound="irr_ub">
            <listOfReactants>
                  <speciesReference species="M_h2o_c" stoichiometry="9.0"
constant="true"/>
                  <speciesReference species="M_pepd_c" stoichiometry="6.0"
constant="true"/>
            </listOfReactants>
            <listOfProducts>
                  <speciesReference species="M_PG_ST_c" stoichiometry="1"
constant="true"/>
                  <speciesReference species="M_ala__D_c" stoichiometry="12.0"
constant="true"/>
                  <speciesReference species="M_h_c" stoichiometry="6.0"
constant="true"/>
```

```
                  <speciesReference species="M_udcpdp_c" stoichiometry="5.0"
constant="true"/>
            &lt;/listOfProducts&gt;
            &lt;fbc:geneProductAssociation&gt;
                  &lt;fbc:or&gt;
                        &lt;fbc:and&gt;
                              <fbc:geneProductRef
fbc:geneProduct="SAUSA300_1075"/>
                              <fbc:geneProductRef
fbc:geneProduct="SAUSA300_1341"/>
                              <fbc:geneProductRef
fbc:geneProduct="SAUSA300_1855"/>
                        &lt;/fbc:and&gt;
                        &lt;fbc:and&gt;
                              <fbc:geneProductRef
fbc:geneProduct="SAUSA300_1075"/>
                              <fbc:geneProductRef
fbc:geneProduct="SAUSA300_0032"/>
                              <fbc:geneProductRef
fbc:geneProduct="SAUSA300_1855"/>
                        &lt;/fbc:and&gt;
                  &lt;/fbc:or&gt;
            &lt;/fbc:geneProductAssociation&gt;
      &lt;/reaction&gt;

      <reaction metaid="R_PEPG" id="R_PEPG" name="monofunctional
peptidoglycan glycosyltransferase" reversible="false" fast="false"
fbc:lowerFluxBound="irr_lb" fbc:upperFluxBound="irr_ub">
            &lt;listOfReactants&gt;
                  <speciesReference species="M_pepm_c" stoichiometry="2.0"
constant="true"/>
            &lt;/listOfReactants&gt;
            &lt;listOfProducts&gt;
                  <speciesReference species="M_h_c" stoichiometry="1"
constant="true"/>
                  <speciesReference species="M_pepd_c" stoichiometry="1"
constant="true"/>
                  <speciesReference species="M_udcpdp_c" stoichiometry="1"
constant="true"/>
            &lt;/listOfProducts&gt;
            &lt;fbc:geneProductAssociation&gt;
                  &lt;fbc:or&gt;
                        &lt;fbc:geneProductRef fbc:geneProduct="SAUSA300_1855"/&gt;
                        &lt;fbc:geneProductRef fbc:geneProduct="SAUSA300_1341"/&gt;
                  &lt;/fbc:or&gt;
            &lt;/fbc:geneProductAssociation&gt;
      &lt;/reaction&gt;

      <reaction metaid="R_RBT5PD" id="R_RBT5PD" name="ribitol-5-phosphate
1__Dehydrogenase" reversible="false" fast="false" fbc:lowerFluxBound="irr_lb"
fbc:upperFluxBound="irr_ub">
            &lt;listOfReactants&gt;
                  <speciesReference species="M_h_c" stoichiometry="1"
constant="true"/>
                  <speciesReference species="M_nadph_c" stoichiometry="1"
constant="true"/>
                  <speciesReference species="M_ru5p__D_c" stoichiometry="1"
constant="true"/>
```

```xml
            </listOfReactants>
            <listOfProducts>
                  <speciesReference species="M_nadp_c" stoichiometry="1"
constant="true"/>
                  <speciesReference species="M_rbt5p_c" stoichiometry="1"
constant="true"/>
            </listOfProducts>
            <fbc:geneProductAssociation>
                  <fbc:geneProductRef fbc:geneProduct="SAUSA300_0250"/>
            </fbc:geneProductAssociation>
      </reaction>

      <reaction metaid="R_RBTCT" id="R_RBTCT" name="ribitol-5-phosphate
cytidylyltransferase" reversible="false" fast="false"
fbc:lowerFluxBound="irr_lb" fbc:upperFluxBound="irr_ub">
            <listOfReactants>
                  <speciesReference species="M_ctp_c" stoichiometry="1"
constant="true"/>
                  <speciesReference species="M_h_c" stoichiometry="1"
constant="true"/>
                  <speciesReference species="M_rbt5p_c" stoichiometry="1"
constant="true"/>
            </listOfReactants>
            <listOfProducts>
                  <speciesReference species="M_cdprib_c" stoichiometry="1"
constant="true"/>
                  <speciesReference species="M_ppi_c" stoichiometry="1"
constant="true"/>
            </listOfProducts>
            <fbc:geneProductAssociation>
                  <fbc:geneProductRef fbc:geneProduct="SAUSA300_0249"/>
            </fbc:geneProductAssociation>
      </reaction>

      <reaction metaid="R_TAAL" id="R_TAAL" name="D-alanine carrier
protein:PG D-alanyltransferase" reversible="false" fast="false"
fbc:lowerFluxBound="irr_lb" fbc:upperFluxBound="irr_ub">
            <listOfReactants>
                  <speciesReference species="M_alaACP_c" stoichiometry="1"
constant="true"/>
                  <speciesReference species="M_pg_SA_c" stoichiometry="1"
constant="true"/>
            </listOfReactants>
            <listOfProducts>
                  <speciesReference species="M_ACP_c" stoichiometry="1"
constant="true"/>
                  <speciesReference species="M_pg_SA_ala_c" stoichiometry="1"
constant="true"/>
            </listOfProducts>
            <fbc:geneProductAssociation>
                  <fbc:geneProductRef fbc:geneProduct="SAUSA300_0838"/>
            </fbc:geneProductAssociation>
      </reaction>

      <reaction metaid="R_UAAPUAT" id="R_UAAPUAT" name="UDP-N-
acetylglucosamine--N-acetylmuramyl-(pentapeptide) pyrophosphoryl-undecaprenol
N-acetylglucosamine transferase" reversible="false" fast="false"
fbc:lowerFluxBound="irr_lb" fbc:upperFluxBound="irr_ub">
```

```
            <listOfReactants>
                  <speciesReference species="M_uacgam_c" stoichiometry="1"
constant="true"/>
                  <speciesReference species="M_updpamaglaa_c"
stoichiometry="1" constant="true"/>
            </listOfReactants>
            <listOfProducts>
                  <speciesReference species="M_h_c" stoichiometry="1"
constant="true"/>
                  <speciesReference species="M_lip2_c" stoichiometry="1"
constant="true"/>
                  <speciesReference species="M_udp_c" stoichiometry="1"
constant="true"/>
            </listOfProducts>
            <fbc:geneProductAssociation>
                  <fbc:geneProductRef fbc:geneProduct="SAUSA300_1311"/>
            </fbc:geneProductAssociation>
      </reaction>

      <reaction metaid="R_WTAAT" id="R_WTAAT" name="teichoic acid D-
alanyltransferase" reversible="false" fast="false"
fbc:lowerFluxBound="irr_lb" fbc:upperFluxBound="irr_ub">
            <listOfReactants>
                  <speciesReference species="M_WTA40r_c" stoichiometry="1"
constant="true"/>
                  <speciesReference species="M_pg_SA_ala_c"
stoichiometry="20.0" constant="true"/>
            </listOfReactants>
            <listOfProducts>
                  <speciesReference species="M_WTA40r_ala_c"
stoichiometry="1" constant="true"/>
                  <speciesReference species="M_pg_SA_c" stoichiometry="20.0"
constant="true"/>
            </listOfProducts>
            <fbc:geneProductAssociation>
                  <fbc:and>
                        <fbc:geneProductRef fbc:geneProduct="SAUSA300_0838"/>
                        <fbc:geneProductRef fbc:geneProduct="SAUSA300_0836"/>
                  </fbc:and>
            </fbc:geneProductAssociation>
      </reaction>

      <reaction metaid="R_WTAGLCNACT" id="R_WTAGLCNACT" name="poly(ribitol
phosphate) teichoic acid alpha-O-GlcNAc transferase" reversible="false"
fast="false" fbc:lowerFluxBound="irr_lb" fbc:upperFluxBound="irr_ub">
            <listOfReactants>
                  <speciesReference species="M_WTA40r_c" stoichiometry="1"
constant="true"/>
                  <speciesReference species="M_uacgam_c" stoichiometry="20.0"
constant="true"/>
            </listOfReactants>
            <listOfProducts>
                  <speciesReference species="M_WTA40r_glcnac_c"
stoichiometry="1" constant="true"/>
                  <speciesReference species="M_h_c" stoichiometry="1"
constant="true"/>
                  <speciesReference species="M_udp_c" stoichiometry="20.0"
constant="true"/>
```

```
            </listOfProducts>
            <fbc:geneProductAssociation>
                  <fbc:and>
                        <fbc:or>
                              <fbc:geneProductRef
fbc:geneProduct="SAUSA300_0939"/>
                              <fbc:geneProductRef
fbc:geneProduct="SAUSA300_0549"/>
                              <fbc:geneProductRef
fbc:geneProduct="SAUSA300_0550"/>
                        </fbc:or>
                        <fbc:geneProductRef fbc:geneProduct="SAUSA300_0252"/>
                  </fbc:and>
            </fbc:geneProductAssociation>
      </reaction>

      <reaction metaid="R_WTAGPT" id="R_WTAGPT" name="teichoic acid glycerol-
phosphate transferase" reversible="false" fast="false"
fbc:lowerFluxBound="irr_lb" fbc:upperFluxBound="irr_ub">
            <listOfReactants>
                  <speciesReference species="M_cdpglyc_c" stoichiometry="1"
constant="true"/>
                  <speciesReference species="M_nacetlbdgl_c"
stoichiometry="1" constant="true"/>
            </listOfReactants>
            <listOfProducts>
                  <speciesReference species="M_cmp_c" stoichiometry="1"
constant="true"/>
                  <speciesReference species="M_h_c" stoichiometry="1"
constant="true"/>
                  <speciesReference species="M_nacetlbdglg_c"
stoichiometry="1" constant="true"/>
            </listOfProducts>
            <fbc:geneProductAssociation>
                  <fbc:geneProductRef fbc:geneProduct="SAUSA300_0626"/>
            </fbc:geneProductAssociation>
      </reaction>

      <reaction metaid="R_WTAGPT2" id="R_WTAGPT2" name="teichoic acid
glycerol-phosphate transferase 2" reversible="false" fast="false"
fbc:lowerFluxBound="irr_lb" fbc:upperFluxBound="irr_ub">
            <listOfReactants>
                  <speciesReference species="M_cdpglyc_c" stoichiometry="1"
constant="true"/>
                  <speciesReference species="M_nacetlbdglg_c"
stoichiometry="1" constant="true"/>
            </listOfReactants>
            <listOfProducts>
                  <speciesReference species="M_cmp_c" stoichiometry="1"
constant="true"/>
                  <speciesReference species="M_h_c" stoichiometry="1"
constant="true"/>
                  <speciesReference species="M_nacetlbdglg2_c"
stoichiometry="1" constant="true"/>
            </listOfProducts>
            <fbc:geneProductAssociation>
                  <fbc:geneProductRef fbc:geneProduct="SAUSA300_0248"/>
            </fbc:geneProductAssociation>
```

```
      </reaction>

      <reaction metaid="R_WTAPGT1" id="R_WTAPGT1" name="0.0"
reversible="false" fast="false" fbc:lowerFluxBound="irr_lb"
fbc:upperFluxBound="irr_ub">
            <listOfReactants>
                  <speciesReference species="M_PG_ST_c" stoichiometry="0.42"
constant="true"/>
                  <speciesReference species="M_WTA40r_ala_c"
stoichiometry="0.33" constant="true"/>
            </listOfReactants>
            <listOfProducts>
                  <speciesReference species="M_WTA40raPG_c" stoichiometry="1"
constant="true"/>
            </listOfProducts>
            <fbc:geneProductAssociation>
                  <fbc:and>
                        <fbc:geneProductRef fbc:geneProduct="SAUSA300_1257"/>
                        <fbc:geneProductRef fbc:geneProduct="SAUSA300_0624"/>
                        <fbc:geneProductRef fbc:geneProduct="SAUSA300_0625"/>
                  </fbc:and>
            </fbc:geneProductAssociation>
      </reaction>

      <reaction metaid="R_WTAPGT2" id="R_WTAPGT2" name="0.0"
reversible="false" fast="false" fbc:lowerFluxBound="irr_lb"
fbc:upperFluxBound="irr_ub">
            <listOfReactants>
                  <speciesReference species="M_PG_ST_c" stoichiometry="0.42"
constant="true"/>
                  <speciesReference species="M_WTA40r_c" stoichiometry="0.33"
constant="true"/>
            </listOfReactants>
            <listOfProducts>
                  <speciesReference species="M_WTA40rPG_c" stoichiometry="1"
constant="true"/>
            </listOfProducts>
            <fbc:geneProductAssociation>
                  <fbc:and>
                        <fbc:geneProductRef fbc:geneProduct="SAUSA300_1257"/>
                        <fbc:geneProductRef fbc:geneProduct="SAUSA300_0624"/>
                        <fbc:geneProductRef fbc:geneProduct="SAUSA300_0625"/>
                  </fbc:and>
            </fbc:geneProductAssociation>
      </reaction>

      <reaction metaid="R_WTAPGT3" id="R_WTAPGT3" name="0.0"
reversible="false" fast="false" fbc:lowerFluxBound="irr_lb"
fbc:upperFluxBound="irr_ub">
            <listOfReactants>
                  <speciesReference species="M_PG_ST_c" stoichiometry="0.42"
constant="true"/>
                  <speciesReference species="M_WTA40r_glcnac_c"
stoichiometry="0.33" constant="true"/>
            </listOfReactants>
            <listOfProducts>
                  <speciesReference species="M_WTA40rgPG_c" stoichiometry="1"
constant="true"/>
```

```
            </listOfProducts>
            <fbc:geneProductAssociation>
                  <fbc:and>
                        <fbc:geneProductRef fbc:geneProduct="SAUSA300_1257"/>
                        <fbc:geneProductRef fbc:geneProduct="SAUSA300_0624"/>
                        <fbc:geneProductRef fbc:geneProduct="SAUSA300_0625"/>
                  </fbc:and>
            </fbc:geneProductAssociation>
      </reaction>

      <reaction metaid="R_WTARPP" id="R_WTARPP" name="teichoic acid ribitol-
phosphate primase/polymerase" reversible="false" fast="false"
fbc:lowerFluxBound="irr_lb" fbc:upperFluxBound="irr_ub">
            <listOfReactants>
                  <speciesReference species="M_cdprib_c" stoichiometry="40.0"
constant="true"/>
                  <speciesReference species="M_nacetlbdglg2_c"
stoichiometry="1" constant="true"/>
            </listOfReactants>
            <listOfProducts>
                  <speciesReference species="M_WTA40r_c" stoichiometry="1"
constant="true"/>
                  <speciesReference species="M_cmp_c" stoichiometry="40.0"
constant="true"/>
                  <speciesReference species="M_h_c" stoichiometry="40.0"
constant="true"/>
            </listOfProducts>
            <fbc:geneProductAssociation>
                  <fbc:geneProductRef fbc:geneProduct="SAUSA300_0251"/>
            </fbc:geneProductAssociation>
      </reaction>

      <reaction metaid="R_Sa_biomass_CDMG" id="R_Sa_biomass_CDMG"
name="Biomass in CDMG" reversible="false" fast="false"
fbc:lowerFluxBound="irr_lb" fbc:upperFluxBound="irr_ub">
            <listOfReactants>
                  <speciesReference species="M_10fthf_c"
stoichiometry="0.00022788" constant="true"/>
                  <speciesReference species="M_12dgr_SA_c"
stoichiometry="0.00429192" constant="true"/>
                  <speciesReference species="M_accoa_c"
stoichiometry="4.72111E-05" constant="true"/>
                  <speciesReference species="M_acgam_c"
stoichiometry="0.38831654" constant="true"/>
                  <speciesReference species="M_acgam1p_c"
stoichiometry="1.246700472" constant="true"/>
                  <speciesReference species="M_ala__L_c"
stoichiometry="0.524932431" constant="true"/>
                  <speciesReference species="M_amp_c"
stoichiometry="3.178064317" constant="true"/>
                  <speciesReference species="M_arg__L_c"
stoichiometry="0.302263551" constant="true"/>
                  <speciesReference species="M_asn__L_c"
stoichiometry="0.246325532" constant="true"/>
                  <speciesReference species="M_asp__L_c"
stoichiometry="0.246325532" constant="true"/>
                  <speciesReference species="M_atp_c"
stoichiometry="55.30935548" constant="true"/>
```

<speciesReference species="M_ca2_c"
stoichiometry="0.005318915" constant="true"/>
<speciesReference species="M_cl_c"
stoichiometry="0.005318915" constant="true"/>
<speciesReference species="M_clpn_SA_c"
stoichiometry="0.002564933" constant="true"/>
<speciesReference species="M_cmp_c"
stoichiometry="2.248148391" constant="true"/>
<speciesReference species="M_coa_c"
stoichiometry="0.000588606" constant="true"/>
<speciesReference species="M_cobalt2_c"
stoichiometry="2.55471E-05" constant="true"/>
<speciesReference species="M_ctp_c"
stoichiometry="0.136431951" constant="true"/>
<speciesReference species="M_cu2_c"
stoichiometry="0.000724517" constant="true"/>
<speciesReference species="M_cys__L_c"
stoichiometry="0.093584286" constant="true"/>
<speciesReference species="M_damp_c"
stoichiometry="0.776633081" constant="true"/>
<speciesReference species="M_datp_c"
stoichiometry="0.020723841" constant="true"/>
<speciesReference species="M_dcmp_c"
stoichiometry="0.541599385" constant="true"/>
<speciesReference species="M_dctp_c"
stoichiometry="0.010116668" constant="true"/>
<speciesReference species="M_dgdcg_SA2_c"
stoichiometry="0.001233212" constant="true"/>
<speciesReference species="M_dgmp_c"
stoichiometry="0.613131379" constant="true"/>
<speciesReference species="M_dgtp_c"
stoichiometry="0.010116668" constant="true"/>
<speciesReference species="M_dtmp_c"
stoichiometry="0.745976512" constant="true"/>
<speciesReference species="M_dttp_c"
stoichiometry="0.020723841" constant="true"/>
<speciesReference species="M_fad_c"
stoichiometry="0.00022788" constant="true"/>
<speciesReference species="M_fe2_c"
stoichiometry="0.006861962" constant="true"/>
<speciesReference species="M_fe3_c"
stoichiometry="0.007978883" constant="true"/>
<speciesReference species="M_gam1p_c"
stoichiometry="0.235033695" constant="true"/>
<speciesReference species="M_glc__D_c"
stoichiometry="0.068313055" constant="true"/>
<speciesReference species="M_glcp_SA_c"
stoichiometry="0.054159939" constant="true"/>
<speciesReference species="M_gln__L_c"
stoichiometry="0.268919423" constant="true"/>
<speciesReference species="M_glu__L_c"
stoichiometry="0.268919423" constant="true"/>
<speciesReference species="M_gly_c"
stoichiometry="0.626048014" constant="true"/>
<speciesReference species="M_gtp_c"
stoichiometry="0.060822633" constant="true"/>
<speciesReference species="M_h2o_c"
stoichiometry="49.66517457" constant="true"/>

```
                  <speciesReference species="M_hemeO_c"
stoichiometry="0.010218856" constant="true"/>
                  <speciesReference species="M_his__L_c"
stoichiometry="0.096811401" constant="true"/>
                  <speciesReference species="M_ile__L_c"
stoichiometry="0.296888433" constant="true"/>
                  <speciesReference species="M_k_c"
stoichiometry="0.199461857" constant="true"/>
                  <speciesReference species="M_leu__L_c"
stoichiometry="0.460390134" constant="true"/>
                  <speciesReference species="M_LTA_c"
stoichiometry="0.055897144" constant="true"/>
                  <speciesReference species="M_lys__L_c"
stoichiometry="0.350670274" constant="true"/>
                  <speciesReference species="M_met__L_c"
stoichiometry="0.157053603" constant="true"/>
                  <speciesReference species="M_mg2_c"
stoichiometry="0.008864858" constant="true"/>
                  <speciesReference species="M_mlthf_c"
stoichiometry="0.00022788" constant="true"/>
                  <speciesReference species="M_mn2_c"
stoichiometry="0.000706123" constant="true"/>
                  <speciesReference species="M_mobd_c"
stoichiometry="7.1532E-06" constant="true"/>
                  <speciesReference species="M_mqn8_c"
stoichiometry="1.64524E-10" constant="true"/>
                  <speciesReference species="M_na1_c"
stoichiometry="0.284625805" constant="true"/>
                  <speciesReference species="M_nad_c"
stoichiometry="0.001871073" constant="true"/>
                  <speciesReference species="M_nadh_c"
stoichiometry="4.72111E-05" constant="true"/>
                  <speciesReference species="M_nadp_c"
stoichiometry="0.000456783" constant="true"/>
                  <speciesReference species="M_nadph_c"
stoichiometry="0.000381981" constant="true"/>
                  <speciesReference species="M_nh4_c"
stoichiometry="0.013297798" constant="true"/>
                  <speciesReference species="M_ni2_c"
stoichiometry="0.000330069" constant="true"/>
                  <speciesReference species="M_pg_SA_c"
stoichiometry="0.023605558" constant="true"/>
                  <speciesReference species="M_pgp_SA_c"
stoichiometry="0.010218856" constant="true"/>
                  <speciesReference species="M_phe__L_c"
stoichiometry="0.189324751" constant="true"/>
                  <speciesReference species="M_ppi_c"
stoichiometry="0.142307793" constant="true"/>
                  <speciesReference species="M_pro__L_c"
stoichiometry="0.225898038" constant="true"/>
                  <speciesReference species="M_ribflv_c"
stoichiometry="0.000106787" constant="true"/>
                  <speciesReference species="M_SA_FREE_FA_c"
stoichiometry="0.01" constant="true"/>
                  <speciesReference species="M_ser__L_c"
stoichiometry="0.220512701" constant="true"/>
                  <speciesReference species="M_sheme_c"
stoichiometry="0.00022788" constant="true"/>
```

```
                  <speciesReference species="M_so4_c"
stoichiometry="0.00443294" constant="true"/>
                  <speciesReference species="M_succoa_c"
stoichiometry="4.72111E-05" constant="true"/>
                  <speciesReference species="M_tcam_c"
stoichiometry="0.029225929" constant="true"/>
                  <speciesReference species="M_thf_c"
stoichiometry="0.00022788" constant="true"/>
                  <speciesReference species="M_thm_c"
stoichiometry="0.00022788" constant="true"/>
                  <speciesReference species="M_thmpp_c"
stoichiometry="0.00022788" constant="true"/>
                  <speciesReference species="M_thr__L_c"
stoichiometry="0.259242166" constant="true"/>
                  <speciesReference species="M_trp__L_c"
stoichiometry="0.058087045" constant="true"/>
                  <speciesReference species="M_tyr__L_c"
stoichiometry="0.140918029" constant="true"/>
                  <speciesReference species="M_uaagmda_c"
stoichiometry="5.62037E-05" constant="true"/>
                  <speciesReference species="M_uamr_c"
stoichiometry="1.818956426" constant="true"/>
                  <speciesReference species="M_udcpdp_c"
stoichiometry="5.62037E-05" constant="true"/>
                  <speciesReference species="M_ump_c"
stoichiometry="2.442306662" constant="true"/>
                  <speciesReference species="M_utp_c"
stoichiometry="0.062048896" constant="true"/>
                  <speciesReference species="M_val__L_c"
stoichiometry="0.432421124" constant="true"/>
                  <speciesReference species="M_WTA40raPG_c"
stoichiometry="0.040262294" constant="true"/>
                  <speciesReference species="M_WTA40rgPG_c"
stoichiometry="0.036481317" constant="true"/>
                  <speciesReference species="M_WTA40rPG_c"
stoichiometry="0.046700173" constant="true"/>
                  <speciesReference species="M_zn2_c"
stoichiometry="0.000348463" constant="true"/>
            </listOfReactants>
            <listOfProducts>
                  <speciesReference species="M_pi_c" stoichiometry="55.309"
constant="true"/>
                  <speciesReference species="M_h_c" stoichiometry="55.309"
constant="true"/>
                  <speciesReference species="M_adp_c" stoichiometry="55.309"
constant="true"/>
            </listOfProducts>
      </reaction>

      <reaction metaid="R_Sa_biomass_universal" id="R_Sa_biomass_universal"
name="Universal biomass" reversible="false" fast="false"
fbc:lowerFluxBound="irr_lb" fbc:upperFluxBound="irr_ub">
            <listOfReactants>
                  <speciesReference species="M_10fthf_c"
stoichiometry="0.00022788" constant="true"/>
                  <speciesReference species="M_12dgr_SA_c"
stoichiometry="0.00429192" constant="true"/>
```

<speciesReference species="M_accoa_c"
stoichiometry="4.72111E-05" constant="true"/>
<speciesReference species="M_acgam_c"
stoichiometry="0.38831654" constant="true"/>
<speciesReference species="M_acgam1p_c"
stoichiometry="1.246700472" constant="true"/>
<speciesReference species="M_ala__L_c"
stoichiometry="0.524932431" constant="true"/>
<speciesReference species="M_amp_c"
stoichiometry="3.178064317" constant="true"/>
<speciesReference species="M_arg__L_c"
stoichiometry="0.302263551" constant="true"/>
<speciesReference species="M_asn__L_c"
stoichiometry="0.246325532" constant="true"/>
<speciesReference species="M_asp__L_c"
stoichiometry="0.246325532" constant="true"/>
<speciesReference species="M_atp_c"
stoichiometry="55.30935548" constant="true"/>
<speciesReference species="M_ca2_c"
stoichiometry="0.005318915" constant="true"/>
<speciesReference species="M_cl_c"
stoichiometry="0.005318915" constant="true"/>
<speciesReference species="M_clpn_SA_c"
stoichiometry="0.002564933" constant="true"/>
<speciesReference species="M_cmp_c"
stoichiometry="2.248148391" constant="true"/>
<speciesReference species="M_coa_c"
stoichiometry="0.000588606" constant="true"/>
<speciesReference species="M_cobalt2_c"
stoichiometry="2.55471E-05" constant="true"/>
<speciesReference species="M_ctp_c"
stoichiometry="0.136431951" constant="true"/>
<speciesReference species="M_cu2_c"
stoichiometry="0.000724517" constant="true"/>
<speciesReference species="M_cys__L_c"
stoichiometry="0.093584286" constant="true"/>
<speciesReference species="M_damp_c"
stoichiometry="0.776633081" constant="true"/>
<speciesReference species="M_datp_c"
stoichiometry="0.020723841" constant="true"/>
<speciesReference species="M_dcmp_c"
stoichiometry="0.541599385" constant="true"/>
<speciesReference species="M_dctp_c"
stoichiometry="0.010116668" constant="true"/>
<speciesReference species="M_dgdcg_SA2_c"
stoichiometry="0.001233212" constant="true"/>
<speciesReference species="M_dgmp_c"
stoichiometry="0.613131379" constant="true"/>
<speciesReference species="M_dgtp_c"
stoichiometry="0.010116668" constant="true"/>
<speciesReference species="M_dtmp_c"
stoichiometry="0.745976512" constant="true"/>
<speciesReference species="M_dttp_c"
stoichiometry="0.020723841" constant="true"/>
<speciesReference species="M_fad_c"
stoichiometry="0.00022788" constant="true"/>
<speciesReference species="M_fe2_c"
stoichiometry="0.006861962" constant="true"/>

```
                    <speciesReference species="M_fe3_c"
stoichiometry="0.007978883" constant="true"/>
                    <speciesReference species="M_gam1p_c"
stoichiometry="0.235033695" constant="true"/>
                    <speciesReference species="M_glc__D_c"
stoichiometry="0.068313055" constant="true"/>
                    <speciesReference species="M_glcp_SA_c"
stoichiometry="0.054159939" constant="true"/>
                    <speciesReference species="M_gln__L_c"
stoichiometry="0.268919423" constant="true"/>
                    <speciesReference species="M_glu__L_c"
stoichiometry="0.268919423" constant="true"/>
                    <speciesReference species="M_gly_c"
stoichiometry="0.626048014" constant="true"/>
                    <speciesReference species="M_gtp_c"
stoichiometry="0.060822633" constant="true"/>
                    <speciesReference species="M_h2o_c"
stoichiometry="49.66517457" constant="true"/>
                    <speciesReference species="M_hemeO_c"
stoichiometry="0.010218856" constant="true"/>
                    <speciesReference species="M_his__L_c"
stoichiometry="0.096811401" constant="true"/>
                    <speciesReference species="M_ile__L_c"
stoichiometry="0.296888433" constant="true"/>
                    <speciesReference species="M_k_c"
stoichiometry="0.199461857" constant="true"/>
                    <speciesReference species="M_leu__L_c"
stoichiometry="0.460390134" constant="true"/>
                    <speciesReference species="M_LTA_c"
stoichiometry="0.055897144" constant="true"/>
                    <speciesReference species="M_lys__L_c"
stoichiometry="0.350670274" constant="true"/>
                    <speciesReference species="M_met__L_c"
stoichiometry="0.157053603" constant="true"/>
                    <speciesReference species="M_mg2_c"
stoichiometry="0.008864858" constant="true"/>
                    <speciesReference species="M_mlthf_c"
stoichiometry="0.00022788" constant="true"/>
                    <speciesReference species="M_mn2_c"
stoichiometry="0.000706123" constant="true"/>
                    <speciesReference species="M_mobd_c"
stoichiometry="7.1532E-06" constant="true"/>
                    <speciesReference species="M_mqn8_c"
stoichiometry="1.64524E-10" constant="true"/>
                    <speciesReference species="M_na1_c"
stoichiometry="0.284625805" constant="true"/>
                    <speciesReference species="M_nad_c"
stoichiometry="0.001871073" constant="true"/>
                    <speciesReference species="M_nadh_c"
stoichiometry="4.72111E-05" constant="true"/>
                    <speciesReference species="M_nadp_c"
stoichiometry="0.000456783" constant="true"/>
                    <speciesReference species="M_nadph_c"
stoichiometry="0.000381981" constant="true"/>
                    <speciesReference species="M_nh4_c"
stoichiometry="0.013297798" constant="true"/>
                    <speciesReference species="M_ni2_c"
stoichiometry="0.000330069" constant="true"/>
```

```
                  <speciesReference species="M_pg_SA_c"
stoichiometry="0.023605558" constant="true"/>
                  <speciesReference species="M_pgp_SA_c"
stoichiometry="0.010218856" constant="true"/>
                  <speciesReference species="M_phe__L_c"
stoichiometry="0.189324751" constant="true"/>
                  <speciesReference species="M_ppi_c"
stoichiometry="0.142307793" constant="true"/>
                  <speciesReference species="M_pro__L_c"
stoichiometry="0.225898038" constant="true"/>
                  <speciesReference species="M_ribflv_c"
stoichiometry="0.000106787" constant="true"/>
                  <speciesReference species="M_SA_FREE_FA_c"
stoichiometry="0.01" constant="true"/>
                  <speciesReference species="M_ser__L_c"
stoichiometry="0.220512701" constant="true"/>
                  <speciesReference species="M_sheme_c"
stoichiometry="0.00022788" constant="true"/>
                  <speciesReference species="M_so4_c"
stoichiometry="0.00443294" constant="true"/>
                  <speciesReference species="M_succoa_c"
stoichiometry="4.72111E-05" constant="true"/>
                  <speciesReference species="M_tcam_c"
stoichiometry="0.029225929" constant="true"/>
                  <speciesReference species="M_thf_c"
stoichiometry="0.00022788" constant="true"/>
                  <speciesReference species="M_thm_c"
stoichiometry="0.00022788" constant="true"/>
                  <speciesReference species="M_thmpp_c"
stoichiometry="0.00022788" constant="true"/>
                  <speciesReference species="M_thr__L_c"
stoichiometry="0.259242166" constant="true"/>
                  <speciesReference species="M_trp__L_c"
stoichiometry="0.058087045" constant="true"/>
                  <speciesReference species="M_tyr__L_c"
stoichiometry="0.140918029" constant="true"/>
                  <speciesReference species="M_uaagmda_c"
stoichiometry="5.62037E-05" constant="true"/>
                  <speciesReference species="M_uamr_c"
stoichiometry="1.818956426" constant="true"/>
                  <speciesReference species="M_udcpdp_c"
stoichiometry="5.62037E-05" constant="true"/>
                  <speciesReference species="M_ump_c"
stoichiometry="2.442306662" constant="true"/>
                  <speciesReference species="M_utp_c"
stoichiometry="0.062048896" constant="true"/>
                  <speciesReference species="M_val__L_c"
stoichiometry="0.432421124" constant="true"/>
                  <speciesReference species="M_zn2_c"
stoichiometry="0.000348463" constant="true"/>
            </listOfReactants>
            <listOfProducts>
                  <speciesReference species="M_pi_c" stoichiometry="55.309"
constant="true"/>
                  <speciesReference species="M_h_c" stoichiometry="55.309"
constant="true"/>
                  <speciesReference species="M_adp_c" stoichiometry="55.309"
constant="true"/>
```

```
            </listOfProducts>
      </reaction>

</listOfReactions>

      <fbc:listOfObjectives fbc:activeObjective = "obj">
            <fbc:objective fbc:id = "obj" fbc:type = "maximize">
                  <fbc:listOfFluxObjectives>
                        <fbc:fluxObjective fbc:reaction =
"R_Sa_biomass_universal" fbc:coefficient = "1"/>
                  </fbc:listOfFluxObjectives>
            </fbc:objective>
      </fbc:listOfObjectives>

      <fbc:listOfGeneProducts>
            <fbc:geneProduct metaid="ada" fbc:id="ada" fbc:label="ada"/>
            <fbc:geneProduct metaid="alsT" fbc:id="alsT" fbc:label="alsT"/>
            <fbc:geneProduct metaid="amx2" fbc:id="amx2" fbc:label="amx2"/>
            <fbc:geneProduct metaid="aspA" fbc:id="aspA" fbc:label="aspA"/>
            <fbc:geneProduct metaid="B7H15_RS13445" fbc:id="B7H15_RS13445"
fbc:label="B7H15_RS13445"/>
            <fbc:geneProduct metaid="B7H15_RS13450" fbc:id="B7H15_RS13450"
fbc:label="B7H15_RS13450"/>
            <fbc:geneProduct metaid="B7H15_RS13455" fbc:id="B7H15_RS13455"
fbc:label="B7H15_RS13455"/>
            <fbc:geneProduct metaid="D3C55_04135" fbc:id="D3C55_04135"
fbc:label="D3C55_04135"/>
            <fbc:geneProduct metaid="dltD" fbc:id="dltD" fbc:label="dltD"/>
            <fbc:geneProduct metaid="fabG" fbc:id="fabG" fbc:label="fabG"/>
            <fbc:geneProduct metaid="FabH" fbc:id="FabH" fbc:label="FabH"/>
            <fbc:geneProduct metaid="gudD" fbc:id="gudD" fbc:label="gudD"/>
            <fbc:geneProduct metaid="gudX" fbc:id="gudX" fbc:label="gudX"/>
            <fbc:geneProduct metaid="hisP" fbc:id="hisP" fbc:label="hisP"/>
            <fbc:geneProduct metaid="mtnK" fbc:id="mtnK" fbc:label="mtnK"/>
            <fbc:geneProduct metaid="niaX" fbc:id="niaX" fbc:label="niaX"/>
            <fbc:geneProduct metaid="psd" fbc:id="psd" fbc:label="psd"/>
            <fbc:geneProduct metaid="pss" fbc:id="pss" fbc:label="pss"/>
            <fbc:geneProduct metaid="s0001" fbc:id="s0001"
fbc:label="s0001"/>
            <fbc:geneProduct metaid="SA1323661_VBIStaAur292900_0185"
fbc:id="SA1323661_VBIStaAur292900_0185"
fbc:label="SA1323661_VBIStaAur292900_0185"/>
            <fbc:geneProduct metaid="SA1323661_VBIStaAur292900_0232"
fbc:id="SA1323661_VBIStaAur292900_0232"
fbc:label="SA1323661_VBIStaAur292900_0232"/>
            <fbc:geneProduct metaid="SA1323661_VBIStaAur292900_0569"
fbc:id="SA1323661_VBIStaAur292900_0569"
fbc:label="SA1323661_VBIStaAur292900_0569"/>
            <fbc:geneProduct metaid="SA451515_2204" fbc:id="SA451515_2204"
fbc:label="SA451515_2204"/>
            <fbc:geneProduct metaid="SA451515_2469" fbc:id="SA451515_2469"
fbc:label="SA451515_2469"/>
            <fbc:geneProduct metaid="SA451515_2543" fbc:id="SA451515_2543"
fbc:label="SA451515_2543"/>
            <fbc:geneProduct metaid="SA451515_255" fbc:id="SA451515_255"
fbc:label="SA451515_255"/>
            <fbc:geneProduct metaid="SA451515_2734" fbc:id="SA451515_2734"
fbc:label="SA451515_2734"/>
```

<fbc:geneProduct metaid="SA451515_2748" fbc:id="SA451515_2748" fbc:label="SA451515_2748"/>
<fbc:geneProduct metaid="SA451515_2750" fbc:id="SA451515_2750" fbc:label="SA451515_2750"/>
<fbc:geneProduct metaid="SAUSA300_0008" fbc:id="SAUSA300_0008" fbc:label="SAUSA300_0008"/>
<fbc:geneProduct metaid="SAUSA300_0009" fbc:id="SAUSA300_0009" fbc:label="SAUSA300_0009"/>
<fbc:geneProduct metaid="SAUSA300_0012" fbc:id="SAUSA300_0012" fbc:label="SAUSA300_0012"/>
<fbc:geneProduct metaid="SAUSA300_0017" fbc:id="SAUSA300_0017" fbc:label="SAUSA300_0017"/>
<fbc:geneProduct metaid="SAUSA300_0025" fbc:id="SAUSA300_0025" fbc:label="SAUSA300_0025"/>
<fbc:geneProduct metaid="SAUSA300_0030" fbc:id="SAUSA300_0030" fbc:label="SAUSA300_0030"/>
<fbc:geneProduct metaid="SAUSA300_0032" fbc:id="SAUSA300_0032" fbc:label="SAUSA300_0032"/>
<fbc:geneProduct metaid="SAUSA300_0053" fbc:id="SAUSA300_0053" fbc:label="SAUSA300_0053"/>
<fbc:geneProduct metaid="SAUSA300_0055" fbc:id="SAUSA300_0055" fbc:label="SAUSA300_0055"/>
<fbc:geneProduct metaid="SAUSA300_0061" fbc:id="SAUSA300_0061" fbc:label="SAUSA300_0061"/>
<fbc:geneProduct metaid="SAUSA300_0062" fbc:id="SAUSA300_0062" fbc:label="SAUSA300_0062"/>
<fbc:geneProduct metaid="SAUSA300_0064" fbc:id="SAUSA300_0064" fbc:label="SAUSA300_0064"/>
<fbc:geneProduct metaid="SAUSA300_0065" fbc:id="SAUSA300_0065" fbc:label="SAUSA300_0065"/>
<fbc:geneProduct metaid="SAUSA300_0070" fbc:id="SAUSA300_0070" fbc:label="SAUSA300_0070"/>
<fbc:geneProduct metaid="SAUSA300_0078" fbc:id="SAUSA300_0078" fbc:label="SAUSA300_0078"/>
<fbc:geneProduct metaid="SAUSA300_0099" fbc:id="SAUSA300_0099" fbc:label="SAUSA300_0099"/>
<fbc:geneProduct metaid="SAUSA300_0107" fbc:id="SAUSA300_0107" fbc:label="SAUSA300_0107"/>
<fbc:geneProduct metaid="SAUSA300_0112" fbc:id="SAUSA300_0112" fbc:label="SAUSA300_0112"/>
<fbc:geneProduct metaid="SAUSA300_0115" fbc:id="SAUSA300_0115" fbc:label="SAUSA300_0115"/>
<fbc:geneProduct metaid="SAUSA300_0116" fbc:id="SAUSA300_0116" fbc:label="SAUSA300_0116"/>
<fbc:geneProduct metaid="SAUSA300_0117" fbc:id="SAUSA300_0117" fbc:label="SAUSA300_0117"/>
<fbc:geneProduct metaid="SAUSA300_0119" fbc:id="SAUSA300_0119" fbc:label="SAUSA300_0119"/>
<fbc:geneProduct metaid="SAUSA300_0124" fbc:id="SAUSA300_0124" fbc:label="SAUSA300_0124"/>
<fbc:geneProduct metaid="SAUSA300_0125" fbc:id="SAUSA300_0125" fbc:label="SAUSA300_0125"/>
<fbc:geneProduct metaid="SAUSA300_0129" fbc:id="SAUSA300_0129" fbc:label="SAUSA300_0129"/>
<fbc:geneProduct metaid="SAUSA300_0130" fbc:id="SAUSA300_0130" fbc:label="SAUSA300_0130"/>
<fbc:geneProduct metaid="SAUSA300_0133" fbc:id="SAUSA300_0133" fbc:label="SAUSA300_0133"/>

<fbc:geneProduct metaid="SAUSA300_0135" fbc:id="SAUSA300_0135" fbc:label="SAUSA300_0135"/>
<fbc:geneProduct metaid="SAUSA300_0138" fbc:id="SAUSA300_0138" fbc:label="SAUSA300_0138"/>
<fbc:geneProduct metaid="SAUSA300_0140" fbc:id="SAUSA300_0140" fbc:label="SAUSA300_0140"/>
<fbc:geneProduct metaid="SAUSA300_0141" fbc:id="SAUSA300_0141" fbc:label="SAUSA300_0141"/>
<fbc:geneProduct metaid="SAUSA300_0147" fbc:id="SAUSA300_0147" fbc:label="SAUSA300_0147"/>
<fbc:geneProduct metaid="SAUSA300_0151" fbc:id="SAUSA300_0151" fbc:label="SAUSA300_0151"/>
<fbc:geneProduct metaid="SAUSA300_0158" fbc:id="SAUSA300_0158" fbc:label="SAUSA300_0158"/>
<fbc:geneProduct metaid="SAUSA300_0165" fbc:id="SAUSA300_0165" fbc:label="SAUSA300_0165"/>
<fbc:geneProduct metaid="SAUSA300_0166" fbc:id="SAUSA300_0166" fbc:label="SAUSA300_0166"/>
<fbc:geneProduct metaid="SAUSA300_0167" fbc:id="SAUSA300_0167" fbc:label="SAUSA300_0167"/>
<fbc:geneProduct metaid="SAUSA300_0170" fbc:id="SAUSA300_0170" fbc:label="SAUSA300_0170"/>
<fbc:geneProduct metaid="SAUSA300_0177" fbc:id="SAUSA300_0177" fbc:label="SAUSA300_0177"/>
<fbc:geneProduct metaid="SAUSA300_0179" fbc:id="SAUSA300_0179" fbc:label="SAUSA300_0179"/>
<fbc:geneProduct metaid="SAUSA300_0184" fbc:id="SAUSA300_0184" fbc:label="SAUSA300_0184"/>
<fbc:geneProduct metaid="SAUSA300_0185" fbc:id="SAUSA300_0185" fbc:label="SAUSA300_0185"/>
<fbc:geneProduct metaid="SAUSA300_0186" fbc:id="SAUSA300_0186" fbc:label="SAUSA300_0186"/>
<fbc:geneProduct metaid="SAUSA300_0187" fbc:id="SAUSA300_0187" fbc:label="SAUSA300_0187"/>
<fbc:geneProduct metaid="SAUSA300_0188" fbc:id="SAUSA300_0188" fbc:label="SAUSA300_0188"/>
<fbc:geneProduct metaid="SAUSA300_0189" fbc:id="SAUSA300_0189" fbc:label="SAUSA300_0189"/>
<fbc:geneProduct metaid="SAUSA300_0190" fbc:id="SAUSA300_0190" fbc:label="SAUSA300_0190"/>
<fbc:geneProduct metaid="SAUSA300_0191" fbc:id="SAUSA300_0191" fbc:label="SAUSA300_0191"/>
<fbc:geneProduct metaid="SAUSA300_0193" fbc:id="SAUSA300_0193" fbc:label="SAUSA300_0193"/>
<fbc:geneProduct metaid="SAUSA300_0200" fbc:id="SAUSA300_0200" fbc:label="SAUSA300_0200"/>
<fbc:geneProduct metaid="SAUSA300_0204" fbc:id="SAUSA300_0204" fbc:label="SAUSA300_0204"/>
<fbc:geneProduct metaid="SAUSA300_0206" fbc:id="SAUSA300_0206" fbc:label="SAUSA300_0206"/>
<fbc:geneProduct metaid="SAUSA300_0208" fbc:id="SAUSA300_0208" fbc:label="SAUSA300_0208"/>
<fbc:geneProduct metaid="SAUSA300_0209" fbc:id="SAUSA300_0209" fbc:label="SAUSA300_0209"/>
<fbc:geneProduct metaid="SAUSA300_0210" fbc:id="SAUSA300_0210" fbc:label="SAUSA300_0210"/>
<fbc:geneProduct metaid="SAUSA300_0211" fbc:id="SAUSA300_0211" fbc:label="SAUSA300_0211"/>

<fbc:geneProduct metaid="SAUSA300_0216" fbc:id="SAUSA300_0216" fbc:label="SAUSA300_0216"/>
<fbc:geneProduct metaid="SAUSA300_0220" fbc:id="SAUSA300_0220" fbc:label="SAUSA300_0220"/>
<fbc:geneProduct metaid="SAUSA300_0221" fbc:id="SAUSA300_0221" fbc:label="SAUSA300_0221"/>
<fbc:geneProduct metaid="SAUSA300_0222" fbc:id="SAUSA300_0222" fbc:label="SAUSA300_0222"/>
<fbc:geneProduct metaid="SAUSA300_0225" fbc:id="SAUSA300_0225" fbc:label="SAUSA300_0225"/>
<fbc:geneProduct metaid="SAUSA300_0226" fbc:id="SAUSA300_0226" fbc:label="SAUSA300_0226"/>
<fbc:geneProduct metaid="SAUSA300_0227" fbc:id="SAUSA300_0227" fbc:label="SAUSA300_0227"/>
<fbc:geneProduct metaid="SAUSA300_0228" fbc:id="SAUSA300_0228" fbc:label="SAUSA300_0228"/>
<fbc:geneProduct metaid="SAUSA300_0229" fbc:id="SAUSA300_0229" fbc:label="SAUSA300_0229"/>
<fbc:geneProduct metaid="SAUSA300_0230" fbc:id="SAUSA300_0230" fbc:label="SAUSA300_0230"/>
<fbc:geneProduct metaid="SAUSA300_0231" fbc:id="SAUSA300_0231" fbc:label="SAUSA300_0231"/>
<fbc:geneProduct metaid="SAUSA300_0232" fbc:id="SAUSA300_0232" fbc:label="SAUSA300_0232"/>
<fbc:geneProduct metaid="SAUSA300_0234" fbc:id="SAUSA300_0234" fbc:label="SAUSA300_0234"/>
<fbc:geneProduct metaid="SAUSA300_0235" fbc:id="SAUSA300_0235" fbc:label="SAUSA300_0235"/>
<fbc:geneProduct metaid="SAUSA300_0236" fbc:id="SAUSA300_0236" fbc:label="SAUSA300_0236"/>
<fbc:geneProduct metaid="SAUSA300_0237" fbc:id="SAUSA300_0237" fbc:label="SAUSA300_0237"/>
<fbc:geneProduct metaid="SAUSA300_0239" fbc:id="SAUSA300_0239" fbc:label="SAUSA300_0239"/>
<fbc:geneProduct metaid="SAUSA300_0240" fbc:id="SAUSA300_0240" fbc:label="SAUSA300_0240"/>
<fbc:geneProduct metaid="SAUSA300_0241" fbc:id="SAUSA300_0241" fbc:label="SAUSA300_0241"/>
<fbc:geneProduct metaid="SAUSA300_0242" fbc:id="SAUSA300_0242" fbc:label="SAUSA300_0242"/>
<fbc:geneProduct metaid="SAUSA300_0244" fbc:id="SAUSA300_0244" fbc:label="SAUSA300_0244"/>
<fbc:geneProduct metaid="SAUSA300_0245" fbc:id="SAUSA300_0245" fbc:label="SAUSA300_0245"/>
<fbc:geneProduct metaid="SAUSA300_0247" fbc:id="SAUSA300_0247" fbc:label="SAUSA300_0247"/>
<fbc:geneProduct metaid="SAUSA300_0248" fbc:id="SAUSA300_0248" fbc:label="SAUSA300_0248"/>
<fbc:geneProduct metaid="SAUSA300_0249" fbc:id="SAUSA300_0249" fbc:label="SAUSA300_0249"/>
<fbc:geneProduct metaid="SAUSA300_0250" fbc:id="SAUSA300_0250" fbc:label="SAUSA300_0250"/>
<fbc:geneProduct metaid="SAUSA300_0251" fbc:id="SAUSA300_0251" fbc:label="SAUSA300_0251"/>
<fbc:geneProduct metaid="SAUSA300_0252" fbc:id="SAUSA300_0252" fbc:label="SAUSA300_0252"/>
<fbc:geneProduct metaid="SAUSA300_0259" fbc:id="SAUSA300_0259" fbc:label="SAUSA300_0259"/>

<fbc:geneProduct metaid="SAUSA300_0260" fbc:id="SAUSA300_0260"
fbc:label="SAUSA300_0260"/>
<fbc:geneProduct metaid="SAUSA300_0262" fbc:id="SAUSA300_0262"
fbc:label="SAUSA300_0262"/>
<fbc:geneProduct metaid="SAUSA300_0263" fbc:id="SAUSA300_0263"
fbc:label="SAUSA300_0263"/>
<fbc:geneProduct metaid="SAUSA300_0264" fbc:id="SAUSA300_0264"
fbc:label="SAUSA300_0264"/>
<fbc:geneProduct metaid="SAUSA300_0269" fbc:id="SAUSA300_0269"
fbc:label="SAUSA300_0269"/>
<fbc:geneProduct metaid="SAUSA300_0305" fbc:id="SAUSA300_0305"
fbc:label="SAUSA300_0305"/>
<fbc:geneProduct metaid="SAUSA300_0306" fbc:id="SAUSA300_0306"
fbc:label="SAUSA300_0306"/>
<fbc:geneProduct metaid="SAUSA300_0313" fbc:id="SAUSA300_0313"
fbc:label="SAUSA300_0313"/>
<fbc:geneProduct metaid="SAUSA300_0315" fbc:id="SAUSA300_0315"
fbc:label="SAUSA300_0315"/>
<fbc:geneProduct metaid="SAUSA300_0316" fbc:id="SAUSA300_0316"
fbc:label="SAUSA300_0316"/>
<fbc:geneProduct metaid="SAUSA300_0317" fbc:id="SAUSA300_0317"
fbc:label="SAUSA300_0317"/>
<fbc:geneProduct metaid="SAUSA300_0318" fbc:id="SAUSA300_0318"
fbc:label="SAUSA300_0318"/>
<fbc:geneProduct metaid="SAUSA300_0322" fbc:id="SAUSA300_0322"
fbc:label="SAUSA300_0322"/>
<fbc:geneProduct metaid="SAUSA300_0325" fbc:id="SAUSA300_0325"
fbc:label="SAUSA300_0325"/>
<fbc:geneProduct metaid="SAUSA300_0328" fbc:id="SAUSA300_0328"
fbc:label="SAUSA300_0328"/>
<fbc:geneProduct metaid="SAUSA300_0332" fbc:id="SAUSA300_0332"
fbc:label="SAUSA300_0332"/>
<fbc:geneProduct metaid="SAUSA300_0337" fbc:id="SAUSA300_0337"
fbc:label="SAUSA300_0337"/>
<fbc:geneProduct metaid="SAUSA300_0340" fbc:id="SAUSA300_0340"
fbc:label="SAUSA300_0340"/>
<fbc:geneProduct metaid="SAUSA300_0344" fbc:id="SAUSA300_0344"
fbc:label="SAUSA300_0344"/>
<fbc:geneProduct metaid="SAUSA300_0345" fbc:id="SAUSA300_0345"
fbc:label="SAUSA300_0345"/>
<fbc:geneProduct metaid="SAUSA300_0355" fbc:id="SAUSA300_0355"
fbc:label="SAUSA300_0355"/>
<fbc:geneProduct metaid="SAUSA300_0357" fbc:id="SAUSA300_0357"
fbc:label="SAUSA300_0357"/>
<fbc:geneProduct metaid="SAUSA300_0358" fbc:id="SAUSA300_0358"
fbc:label="SAUSA300_0358"/>
<fbc:geneProduct metaid="SAUSA300_0359" fbc:id="SAUSA300_0359"
fbc:label="SAUSA300_0359"/>
<fbc:geneProduct metaid="SAUSA300_0360" fbc:id="SAUSA300_0360"
fbc:label="SAUSA300_0360"/>
<fbc:geneProduct metaid="SAUSA300_0375" fbc:id="SAUSA300_0375"
fbc:label="SAUSA300_0375"/>
<fbc:geneProduct metaid="SAUSA300_0386" fbc:id="SAUSA300_0386"
fbc:label="SAUSA300_0386"/>
<fbc:geneProduct metaid="SAUSA300_0387" fbc:id="SAUSA300_0387"
fbc:label="SAUSA300_0387"/>
<fbc:geneProduct metaid="SAUSA300_0388" fbc:id="SAUSA300_0388"
fbc:label="SAUSA300_0388"/>

<fbc:geneProduct metaid="SAUSA300_0389" fbc:id="SAUSA300_0389" fbc:label="SAUSA300_0389"/>
<fbc:geneProduct metaid="SAUSA300_0425" fbc:id="SAUSA300_0425" fbc:label="SAUSA300_0425"/>
<fbc:geneProduct metaid="SAUSA300_0426" fbc:id="SAUSA300_0426" fbc:label="SAUSA300_0426"/>
<fbc:geneProduct metaid="SAUSA300_0427" fbc:id="SAUSA300_0427" fbc:label="SAUSA300_0427"/>
<fbc:geneProduct metaid="SAUSA300_0433" fbc:id="SAUSA300_0433" fbc:label="SAUSA300_0433"/>
<fbc:geneProduct metaid="SAUSA300_0434" fbc:id="SAUSA300_0434" fbc:label="SAUSA300_0434"/>
<fbc:geneProduct metaid="SAUSA300_0435" fbc:id="SAUSA300_0435" fbc:label="SAUSA300_0435"/>
<fbc:geneProduct metaid="SAUSA300_0436" fbc:id="SAUSA300_0436" fbc:label="SAUSA300_0436"/>
<fbc:geneProduct metaid="SAUSA300_0437" fbc:id="SAUSA300_0437" fbc:label="SAUSA300_0437"/>
<fbc:geneProduct metaid="SAUSA300_0445" fbc:id="SAUSA300_0445" fbc:label="SAUSA300_0445"/>
<fbc:geneProduct metaid="SAUSA300_0446" fbc:id="SAUSA300_0446" fbc:label="SAUSA300_0446"/>
<fbc:geneProduct metaid="SAUSA300_0448" fbc:id="SAUSA300_0448" fbc:label="SAUSA300_0448"/>
<fbc:geneProduct metaid="SAUSA300_0449" fbc:id="SAUSA300_0449" fbc:label="SAUSA300_0449"/>
<fbc:geneProduct metaid="SAUSA300_0458" fbc:id="SAUSA300_0458" fbc:label="SAUSA300_0458"/>
<fbc:geneProduct metaid="SAUSA300_0459" fbc:id="SAUSA300_0459" fbc:label="SAUSA300_0459"/>
<fbc:geneProduct metaid="SAUSA300_0464" fbc:id="SAUSA300_0464" fbc:label="SAUSA300_0464"/>
<fbc:geneProduct metaid="SAUSA300_0467" fbc:id="SAUSA300_0467" fbc:label="SAUSA300_0467"/>
<fbc:geneProduct metaid="SAUSA300_0472" fbc:id="SAUSA300_0472" fbc:label="SAUSA300_0472"/>
<fbc:geneProduct metaid="SAUSA300_0477" fbc:id="SAUSA300_0477" fbc:label="SAUSA300_0477"/>
<fbc:geneProduct metaid="SAUSA300_0478" fbc:id="SAUSA300_0478" fbc:label="SAUSA300_0478"/>
<fbc:geneProduct metaid="SAUSA300_0488" fbc:id="SAUSA300_0488" fbc:label="SAUSA300_0488"/>
<fbc:geneProduct metaid="SAUSA300_0491" fbc:id="SAUSA300_0491" fbc:label="SAUSA300_0491"/>
<fbc:geneProduct metaid="SAUSA300_0492" fbc:id="SAUSA300_0492" fbc:label="SAUSA300_0492"/>
<fbc:geneProduct metaid="SAUSA300_0493" fbc:id="SAUSA300_0493" fbc:label="SAUSA300_0493"/>
<fbc:geneProduct metaid="SAUSA300_0494" fbc:id="SAUSA300_0494" fbc:label="SAUSA300_0494"/>
<fbc:geneProduct metaid="SAUSA300_0496" fbc:id="SAUSA300_0496" fbc:label="SAUSA300_0496"/>
<fbc:geneProduct metaid="SAUSA300_0504" fbc:id="SAUSA300_0504" fbc:label="SAUSA300_0504"/>
<fbc:geneProduct metaid="SAUSA300_0505" fbc:id="SAUSA300_0505" fbc:label="SAUSA300_0505"/>
<fbc:geneProduct metaid="SAUSA300_0506" fbc:id="SAUSA300_0506" fbc:label="SAUSA300_0506"/>

<fbc:geneProduct metaid="SAUSA300_0513" fbc:id="SAUSA300_0513" fbc:label="SAUSA300_0513"/>
<fbc:geneProduct metaid="SAUSA300_0514" fbc:id="SAUSA300_0514" fbc:label="SAUSA300_0514"/>
<fbc:geneProduct metaid="SAUSA300_0515" fbc:id="SAUSA300_0515" fbc:label="SAUSA300_0515"/>
<fbc:geneProduct metaid="SAUSA300_0533" fbc:id="SAUSA300_0533" fbc:label="SAUSA300_0533"/>
<fbc:geneProduct metaid="SAUSA300_0534" fbc:id="SAUSA300_0534" fbc:label="SAUSA300_0534"/>
<fbc:geneProduct metaid="SAUSA300_0535" fbc:id="SAUSA300_0535" fbc:label="SAUSA300_0535"/>
<fbc:geneProduct metaid="SAUSA300_0536" fbc:id="SAUSA300_0536" fbc:label="SAUSA300_0536"/>
<fbc:geneProduct metaid="SAUSA300_0537" fbc:id="SAUSA300_0537" fbc:label="SAUSA300_0537"/>
<fbc:geneProduct metaid="SAUSA300_0538" fbc:id="SAUSA300_0538" fbc:label="SAUSA300_0538"/>
<fbc:geneProduct metaid="SAUSA300_0539" fbc:id="SAUSA300_0539" fbc:label="SAUSA300_0539"/>
<fbc:geneProduct metaid="SAUSA300_0540" fbc:id="SAUSA300_0540" fbc:label="SAUSA300_0540"/>
<fbc:geneProduct metaid="SAUSA300_0541" fbc:id="SAUSA300_0541" fbc:label="SAUSA300_0541"/>
<fbc:geneProduct metaid="SAUSA300_0542" fbc:id="SAUSA300_0542" fbc:label="SAUSA300_0542"/>
<fbc:geneProduct metaid="SAUSA300_0543" fbc:id="SAUSA300_0543" fbc:label="SAUSA300_0543"/>
<fbc:geneProduct metaid="SAUSA300_0545" fbc:id="SAUSA300_0545" fbc:label="SAUSA300_0545"/>
<fbc:geneProduct metaid="SAUSA300_0549" fbc:id="SAUSA300_0549" fbc:label="SAUSA300_0549"/>
<fbc:geneProduct metaid="SAUSA300_0550" fbc:id="SAUSA300_0550" fbc:label="SAUSA300_0550"/>
<fbc:geneProduct metaid="SAUSA300_0551" fbc:id="SAUSA300_0551" fbc:label="SAUSA300_0551"/>
<fbc:geneProduct metaid="SAUSA300_0554" fbc:id="SAUSA300_0554" fbc:label="SAUSA300_0554"/>
<fbc:geneProduct metaid="SAUSA300_0555" fbc:id="SAUSA300_0555" fbc:label="SAUSA300_0555"/>
<fbc:geneProduct metaid="SAUSA300_0556" fbc:id="SAUSA300_0556" fbc:label="SAUSA300_0556"/>
<fbc:geneProduct metaid="SAUSA300_0557" fbc:id="SAUSA300_0557" fbc:label="SAUSA300_0557"/>
<fbc:geneProduct metaid="SAUSA300_0558" fbc:id="SAUSA300_0558" fbc:label="SAUSA300_0558"/>
<fbc:geneProduct metaid="SAUSA300_0559" fbc:id="SAUSA300_0559" fbc:label="SAUSA300_0559"/>
<fbc:geneProduct metaid="SAUSA300_0560" fbc:id="SAUSA300_0560" fbc:label="SAUSA300_0560"/>
<fbc:geneProduct metaid="SAUSA300_0562" fbc:id="SAUSA300_0562" fbc:label="SAUSA300_0562"/>
<fbc:geneProduct metaid="SAUSA300_0569" fbc:id="SAUSA300_0569" fbc:label="SAUSA300_0569"/>
<fbc:geneProduct metaid="SAUSA300_0570" fbc:id="SAUSA300_0570" fbc:label="SAUSA300_0570"/>
<fbc:geneProduct metaid="SAUSA300_0571" fbc:id="SAUSA300_0571" fbc:label="SAUSA300_0571"/>

<fbc:geneProduct metaid="SAUSA300_0572" fbc:id="SAUSA300_0572" fbc:label="SAUSA300_0572"/>
<fbc:geneProduct metaid="SAUSA300_0573" fbc:id="SAUSA300_0573" fbc:label="SAUSA300_0573"/>
<fbc:geneProduct metaid="SAUSA300_0574" fbc:id="SAUSA300_0574" fbc:label="SAUSA300_0574"/>
<fbc:geneProduct metaid="SAUSA300_0576" fbc:id="SAUSA300_0576" fbc:label="SAUSA300_0576"/>
<fbc:geneProduct metaid="SAUSA300_0594" fbc:id="SAUSA300_0594" fbc:label="SAUSA300_0594"/>
<fbc:geneProduct metaid="SAUSA300_0596" fbc:id="SAUSA300_0596" fbc:label="SAUSA300_0596"/>
<fbc:geneProduct metaid="SAUSA300_0610" fbc:id="SAUSA300_0610" fbc:label="SAUSA300_0610"/>
<fbc:geneProduct metaid="SAUSA300_0611" fbc:id="SAUSA300_0611" fbc:label="SAUSA300_0611"/>
<fbc:geneProduct metaid="SAUSA300_0612" fbc:id="SAUSA300_0612" fbc:label="SAUSA300_0612"/>
<fbc:geneProduct metaid="SAUSA300_0613" fbc:id="SAUSA300_0613" fbc:label="SAUSA300_0613"/>
<fbc:geneProduct metaid="SAUSA300_0614" fbc:id="SAUSA300_0614" fbc:label="SAUSA300_0614"/>
<fbc:geneProduct metaid="SAUSA300_0615" fbc:id="SAUSA300_0615" fbc:label="SAUSA300_0615"/>
<fbc:geneProduct metaid="SAUSA300_0616" fbc:id="SAUSA300_0616" fbc:label="SAUSA300_0616"/>
<fbc:geneProduct metaid="SAUSA300_0617" fbc:id="SAUSA300_0617" fbc:label="SAUSA300_0617"/>
<fbc:geneProduct metaid="SAUSA300_0618" fbc:id="SAUSA300_0618" fbc:label="SAUSA300_0618"/>
<fbc:geneProduct metaid="SAUSA300_0619" fbc:id="SAUSA300_0619" fbc:label="SAUSA300_0619"/>
<fbc:geneProduct metaid="SAUSA300_0620" fbc:id="SAUSA300_0620" fbc:label="SAUSA300_0620"/>
<fbc:geneProduct metaid="SAUSA300_0623" fbc:id="SAUSA300_0623" fbc:label="SAUSA300_0623"/>
<fbc:geneProduct metaid="SAUSA300_0624" fbc:id="SAUSA300_0624" fbc:label="SAUSA300_0624"/>
<fbc:geneProduct metaid="SAUSA300_0625" fbc:id="SAUSA300_0625" fbc:label="SAUSA300_0625"/>
<fbc:geneProduct metaid="SAUSA300_0626" fbc:id="SAUSA300_0626" fbc:label="SAUSA300_0626"/>
<fbc:geneProduct metaid="SAUSA300_0627" fbc:id="SAUSA300_0627" fbc:label="SAUSA300_0627"/>
<fbc:geneProduct metaid="SAUSA300_0628" fbc:id="SAUSA300_0628" fbc:label="SAUSA300_0628"/>
<fbc:geneProduct metaid="SAUSA300_0631" fbc:id="SAUSA300_0631" fbc:label="SAUSA300_0631"/>
<fbc:geneProduct metaid="SAUSA300_0633" fbc:id="SAUSA300_0633" fbc:label="SAUSA300_0633"/>
<fbc:geneProduct metaid="SAUSA300_0636" fbc:id="SAUSA300_0636" fbc:label="SAUSA300_0636"/>
<fbc:geneProduct metaid="SAUSA300_0637" fbc:id="SAUSA300_0637" fbc:label="SAUSA300_0637"/>
<fbc:geneProduct metaid="SAUSA300_0638" fbc:id="SAUSA300_0638" fbc:label="SAUSA300_0638"/>
<fbc:geneProduct metaid="SAUSA300_0650" fbc:id="SAUSA300_0650" fbc:label="SAUSA300_0650"/>

<fbc:geneProduct metaid="SAUSA300_0669" fbc:id="SAUSA300_0669" fbc:label="SAUSA300_0669"/>
<fbc:geneProduct metaid="SAUSA300_0684" fbc:id="SAUSA300_0684" fbc:label="SAUSA300_0684"/>
<fbc:geneProduct metaid="SAUSA300_0685" fbc:id="SAUSA300_0685" fbc:label="SAUSA300_0685"/>
<fbc:geneProduct metaid="SAUSA300_0686" fbc:id="SAUSA300_0686" fbc:label="SAUSA300_0686"/>
<fbc:geneProduct metaid="SAUSA300_0688" fbc:id="SAUSA300_0688" fbc:label="SAUSA300_0688"/>
<fbc:geneProduct metaid="SAUSA300_0696" fbc:id="SAUSA300_0696" fbc:label="SAUSA300_0696"/>
<fbc:geneProduct metaid="SAUSA300_0699" fbc:id="SAUSA300_0699" fbc:label="SAUSA300_0699"/>
<fbc:geneProduct metaid="SAUSA300_0700" fbc:id="SAUSA300_0700" fbc:label="SAUSA300_0700"/>
<fbc:geneProduct metaid="SAUSA300_0701" fbc:id="SAUSA300_0701" fbc:label="SAUSA300_0701"/>
<fbc:geneProduct metaid="SAUSA300_0702" fbc:id="SAUSA300_0702" fbc:label="SAUSA300_0702"/>
<fbc:geneProduct metaid="SAUSA300_0703" fbc:id="SAUSA300_0703" fbc:label="SAUSA300_0703"/>
<fbc:geneProduct metaid="SAUSA300_0706" fbc:id="SAUSA300_0706" fbc:label="SAUSA300_0706"/>
<fbc:geneProduct metaid="SAUSA300_0707" fbc:id="SAUSA300_0707" fbc:label="SAUSA300_0707"/>
<fbc:geneProduct metaid="SAUSA300_0708" fbc:id="SAUSA300_0708" fbc:label="SAUSA300_0708"/>
<fbc:geneProduct metaid="SAUSA300_0712" fbc:id="SAUSA300_0712" fbc:label="SAUSA300_0712"/>
<fbc:geneProduct metaid="SAUSA300_0715" fbc:id="SAUSA300_0715" fbc:label="SAUSA300_0715"/>
<fbc:geneProduct metaid="SAUSA300_0716" fbc:id="SAUSA300_0716" fbc:label="SAUSA300_0716"/>
<fbc:geneProduct metaid="SAUSA300_0717" fbc:id="SAUSA300_0717" fbc:label="SAUSA300_0717"/>
<fbc:geneProduct metaid="SAUSA300_0718" fbc:id="SAUSA300_0718" fbc:label="SAUSA300_0718"/>
<fbc:geneProduct metaid="SAUSA300_0719" fbc:id="SAUSA300_0719" fbc:label="SAUSA300_0719"/>
<fbc:geneProduct metaid="SAUSA300_0720" fbc:id="SAUSA300_0720" fbc:label="SAUSA300_0720"/>
<fbc:geneProduct metaid="SAUSA300_0721" fbc:id="SAUSA300_0721" fbc:label="SAUSA300_0721"/>
<fbc:geneProduct metaid="SAUSA300_0722" fbc:id="SAUSA300_0722" fbc:label="SAUSA300_0722"/>
<fbc:geneProduct metaid="SAUSA300_0726" fbc:id="SAUSA300_0726" fbc:label="SAUSA300_0726"/>
<fbc:geneProduct metaid="SAUSA300_0731" fbc:id="SAUSA300_0731" fbc:label="SAUSA300_0731"/>
<fbc:geneProduct metaid="SAUSA300_0743" fbc:id="SAUSA300_0743" fbc:label="SAUSA300_0743"/>
<fbc:geneProduct metaid="SAUSA300_0747" fbc:id="SAUSA300_0747" fbc:label="SAUSA300_0747"/>
<fbc:geneProduct metaid="SAUSA300_0756" fbc:id="SAUSA300_0756" fbc:label="SAUSA300_0756"/>
<fbc:geneProduct metaid="SAUSA300_0757" fbc:id="SAUSA300_0757" fbc:label="SAUSA300_0757"/>

<fbc:geneProduct metaid="SAUSA300_0758" fbc:id="SAUSA300_0758" fbc:label="SAUSA300_0758"/>
<fbc:geneProduct metaid="SAUSA300_0759" fbc:id="SAUSA300_0759" fbc:label="SAUSA300_0759"/>
<fbc:geneProduct metaid="SAUSA300_0760" fbc:id="SAUSA300_0760" fbc:label="SAUSA300_0760"/>
<fbc:geneProduct metaid="SAUSA300_0784" fbc:id="SAUSA300_0784" fbc:label="SAUSA300_0784"/>
<fbc:geneProduct metaid="SAUSA300_0787" fbc:id="SAUSA300_0787" fbc:label="SAUSA300_0787"/>
<fbc:geneProduct metaid="SAUSA300_0789" fbc:id="SAUSA300_0789" fbc:label="SAUSA300_0789"/>
<fbc:geneProduct metaid="SAUSA300_0791" fbc:id="SAUSA300_0791" fbc:label="SAUSA300_0791"/>
<fbc:geneProduct metaid="SAUSA300_0795" fbc:id="SAUSA300_0795" fbc:label="SAUSA300_0795"/>
<fbc:geneProduct metaid="SAUSA300_0796" fbc:id="SAUSA300_0796" fbc:label="SAUSA300_0796"/>
<fbc:geneProduct metaid="SAUSA300_0797" fbc:id="SAUSA300_0797" fbc:label="SAUSA300_0797"/>
<fbc:geneProduct metaid="SAUSA300_0798" fbc:id="SAUSA300_0798" fbc:label="SAUSA300_0798"/>
<fbc:geneProduct metaid="SAUSA300_0818" fbc:id="SAUSA300_0818" fbc:label="SAUSA300_0818"/>
<fbc:geneProduct metaid="SAUSA300_0819" fbc:id="SAUSA300_0819" fbc:label="SAUSA300_0819"/>
<fbc:geneProduct metaid="SAUSA300_0820" fbc:id="SAUSA300_0820" fbc:label="SAUSA300_0820"/>
<fbc:geneProduct metaid="SAUSA300_0821" fbc:id="SAUSA300_0821" fbc:label="SAUSA300_0821"/>
<fbc:geneProduct metaid="SAUSA300_0822" fbc:id="SAUSA300_0822" fbc:label="SAUSA300_0822"/>
<fbc:geneProduct metaid="SAUSA300_0828" fbc:id="SAUSA300_0828" fbc:label="SAUSA300_0828"/>
<fbc:geneProduct metaid="SAUSA300_0829" fbc:id="SAUSA300_0829" fbc:label="SAUSA300_0829"/>
<fbc:geneProduct metaid="SAUSA300_0833" fbc:id="SAUSA300_0833" fbc:label="SAUSA300_0833"/>
<fbc:geneProduct metaid="SAUSA300_0834" fbc:id="SAUSA300_0834" fbc:label="SAUSA300_0834"/>
<fbc:geneProduct metaid="SAUSA300_0835" fbc:id="SAUSA300_0835" fbc:label="SAUSA300_0835"/>
<fbc:geneProduct metaid="SAUSA300_0836" fbc:id="SAUSA300_0836" fbc:label="SAUSA300_0836"/>
<fbc:geneProduct metaid="SAUSA300_0837" fbc:id="SAUSA300_0837" fbc:label="SAUSA300_0837"/>
<fbc:geneProduct metaid="SAUSA300_0838" fbc:id="SAUSA300_0838" fbc:label="SAUSA300_0838"/>
<fbc:geneProduct metaid="SAUSA300_0839" fbc:id="SAUSA300_0839" fbc:label="SAUSA300_0839"/>
<fbc:geneProduct metaid="SAUSA300_0841" fbc:id="SAUSA300_0841" fbc:label="SAUSA300_0841"/>
<fbc:geneProduct metaid="SAUSA300_0843" fbc:id="SAUSA300_0843" fbc:label="SAUSA300_0843"/>
<fbc:geneProduct metaid="SAUSA300_0844" fbc:id="SAUSA300_0844" fbc:label="SAUSA300_0844"/>
<fbc:geneProduct metaid="SAUSA300_0845" fbc:id="SAUSA300_0845" fbc:label="SAUSA300_0845"/>

```
            <fbc:geneProduct metaid="SAUSA300_0846" fbc:id="SAUSA300_0846"
fbc:label="SAUSA300_0846"/>
            <fbc:geneProduct metaid="SAUSA300_0849" fbc:id="SAUSA300_0849"
fbc:label="SAUSA300_0849"/>
            <fbc:geneProduct metaid="SAUSA300_0850" fbc:id="SAUSA300_0850"
fbc:label="SAUSA300_0850"/>
            <fbc:geneProduct metaid="SAUSA300_0851" fbc:id="SAUSA300_0851"
fbc:label="SAUSA300_0851"/>
            <fbc:geneProduct metaid="SAUSA300_0852" fbc:id="SAUSA300_0852"
fbc:label="SAUSA300_0852"/>
            <fbc:geneProduct metaid="SAUSA300_0853" fbc:id="SAUSA300_0853"
fbc:label="SAUSA300_0853"/>
            <fbc:geneProduct metaid="SAUSA300_0854" fbc:id="SAUSA300_0854"
fbc:label="SAUSA300_0854"/>
            <fbc:geneProduct metaid="SAUSA300_0855" fbc:id="SAUSA300_0855"
fbc:label="SAUSA300_0855"/>
            <fbc:geneProduct metaid="SAUSA300_0860" fbc:id="SAUSA300_0860"
fbc:label="SAUSA300_0860"/>
            <fbc:geneProduct metaid="SAUSA300_0861" fbc:id="SAUSA300_0861"
fbc:label="SAUSA300_0861"/>
            <fbc:geneProduct metaid="SAUSA300_0862" fbc:id="SAUSA300_0862"
fbc:label="SAUSA300_0862"/>
            <fbc:geneProduct metaid="SAUSA300_0863" fbc:id="SAUSA300_0863"
fbc:label="SAUSA300_0863"/>
            <fbc:geneProduct metaid="SAUSA300_0864" fbc:id="SAUSA300_0864"
fbc:label="SAUSA300_0864"/>
            <fbc:geneProduct metaid="SAUSA300_0865" fbc:id="SAUSA300_0865"
fbc:label="SAUSA300_0865"/>
            <fbc:geneProduct metaid="SAUSA300_0874" fbc:id="SAUSA300_0874"
fbc:label="SAUSA300_0874"/>
            <fbc:geneProduct metaid="SAUSA300_0875" fbc:id="SAUSA300_0875"
fbc:label="SAUSA300_0875"/>
            <fbc:geneProduct metaid="SAUSA300_0879" fbc:id="SAUSA300_0879"
fbc:label="SAUSA300_0879"/>
            <fbc:geneProduct metaid="SAUSA300_0885" fbc:id="SAUSA300_0885"
fbc:label="SAUSA300_0885"/>
            <fbc:geneProduct metaid="SAUSA300_0886" fbc:id="SAUSA300_0886"
fbc:label="SAUSA300_0886"/>
            <fbc:geneProduct metaid="SAUSA300_0887" fbc:id="SAUSA300_0887"
fbc:label="SAUSA300_0887"/>
            <fbc:geneProduct metaid="SAUSA300_0888" fbc:id="SAUSA300_0888"
fbc:label="SAUSA300_0888"/>
            <fbc:geneProduct metaid="SAUSA300_0889" fbc:id="SAUSA300_0889"
fbc:label="SAUSA300_0889"/>
            <fbc:geneProduct metaid="SAUSA300_0890" fbc:id="SAUSA300_0890"
fbc:label="SAUSA300_0890"/>
            <fbc:geneProduct metaid="SAUSA300_0891" fbc:id="SAUSA300_0891"
fbc:label="SAUSA300_0891"/>
            <fbc:geneProduct metaid="SAUSA300_0893" fbc:id="SAUSA300_0893"
fbc:label="SAUSA300_0893"/>
            <fbc:geneProduct metaid="SAUSA300_0895" fbc:id="SAUSA300_0895"
fbc:label="SAUSA300_0895"/>
            <fbc:geneProduct metaid="SAUSA300_0896" fbc:id="SAUSA300_0896"
fbc:label="SAUSA300_0896"/>
            <fbc:geneProduct metaid="SAUSA300_0905" fbc:id="SAUSA300_0905"
fbc:label="SAUSA300_0905"/>
            <fbc:geneProduct metaid="SAUSA300_0907" fbc:id="SAUSA300_0907"
fbc:label="SAUSA300_0907"/>
```

<fbc:geneProduct metaid="SAUSA300_0908" fbc:id="SAUSA300_0908" fbc:label="SAUSA300_0908"/>
<fbc:geneProduct metaid="SAUSA300_0910" fbc:id="SAUSA300_0910" fbc:label="SAUSA300_0910"/>
<fbc:geneProduct metaid="SAUSA300_0912" fbc:id="SAUSA300_0912" fbc:label="SAUSA300_0912"/>
<fbc:geneProduct metaid="SAUSA300_0914" fbc:id="SAUSA300_0914" fbc:label="SAUSA300_0914"/>
<fbc:geneProduct metaid="SAUSA300_0918" fbc:id="SAUSA300_0918" fbc:label="SAUSA300_0918"/>
<fbc:geneProduct metaid="SAUSA300_0919" fbc:id="SAUSA300_0919" fbc:label="SAUSA300_0919"/>
<fbc:geneProduct metaid="SAUSA300_0924" fbc:id="SAUSA300_0924" fbc:label="SAUSA300_0924"/>
<fbc:geneProduct metaid="SAUSA300_0925" fbc:id="SAUSA300_0925" fbc:label="SAUSA300_0925"/>
<fbc:geneProduct metaid="SAUSA300_0930" fbc:id="SAUSA300_0930" fbc:label="SAUSA300_0930"/>
<fbc:geneProduct metaid="SAUSA300_0939" fbc:id="SAUSA300_0939" fbc:label="SAUSA300_0939"/>
<fbc:geneProduct metaid="SAUSA300_0941" fbc:id="SAUSA300_0941" fbc:label="SAUSA300_0941"/>
<fbc:geneProduct metaid="SAUSA300_0944" fbc:id="SAUSA300_0944" fbc:label="SAUSA300_0944"/>
<fbc:geneProduct metaid="SAUSA300_0945" fbc:id="SAUSA300_0945" fbc:label="SAUSA300_0945"/>
<fbc:geneProduct metaid="SAUSA300_0946" fbc:id="SAUSA300_0946" fbc:label="SAUSA300_0946"/>
<fbc:geneProduct metaid="SAUSA300_0947" fbc:id="SAUSA300_0947" fbc:label="SAUSA300_0947"/>
<fbc:geneProduct metaid="SAUSA300_0948" fbc:id="SAUSA300_0948" fbc:label="SAUSA300_0948"/>
<fbc:geneProduct metaid="SAUSA300_0952" fbc:id="SAUSA300_0952" fbc:label="SAUSA300_0952"/>
<fbc:geneProduct metaid="SAUSA300_0959" fbc:id="SAUSA300_0959" fbc:label="SAUSA300_0959"/>
<fbc:geneProduct metaid="SAUSA300_0965" fbc:id="SAUSA300_0965" fbc:label="SAUSA300_0965"/>
<fbc:geneProduct metaid="SAUSA300_0966" fbc:id="SAUSA300_0966" fbc:label="SAUSA300_0966"/>
<fbc:geneProduct metaid="SAUSA300_0967" fbc:id="SAUSA300_0967" fbc:label="SAUSA300_0967"/>
<fbc:geneProduct metaid="SAUSA300_0968" fbc:id="SAUSA300_0968" fbc:label="SAUSA300_0968"/>
<fbc:geneProduct metaid="SAUSA300_0969" fbc:id="SAUSA300_0969" fbc:label="SAUSA300_0969"/>
<fbc:geneProduct metaid="SAUSA300_0970" fbc:id="SAUSA300_0970" fbc:label="SAUSA300_0970"/>
<fbc:geneProduct metaid="SAUSA300_0971" fbc:id="SAUSA300_0971" fbc:label="SAUSA300_0971"/>
<fbc:geneProduct metaid="SAUSA300_0972" fbc:id="SAUSA300_0972" fbc:label="SAUSA300_0972"/>
<fbc:geneProduct metaid="SAUSA300_0973" fbc:id="SAUSA300_0973" fbc:label="SAUSA300_0973"/>
<fbc:geneProduct metaid="SAUSA300_0974" fbc:id="SAUSA300_0974" fbc:label="SAUSA300_0974"/>
<fbc:geneProduct metaid="SAUSA300_0975" fbc:id="SAUSA300_0975" fbc:label="SAUSA300_0975"/>

<fbc:geneProduct metaid="SAUSA300_0976" fbc:id="SAUSA300_0976" fbc:label="SAUSA300_0976"/>
<fbc:geneProduct metaid="SAUSA300_0977" fbc:id="SAUSA300_0977" fbc:label="SAUSA300_0977"/>
<fbc:geneProduct metaid="SAUSA300_0978" fbc:id="SAUSA300_0978" fbc:label="SAUSA300_0978"/>
<fbc:geneProduct metaid="SAUSA300_0979" fbc:id="SAUSA300_0979" fbc:label="SAUSA300_0979"/>
<fbc:geneProduct metaid="SAUSA300_0983" fbc:id="SAUSA300_0983" fbc:label="SAUSA300_0983"/>
<fbc:geneProduct metaid="SAUSA300_0984" fbc:id="SAUSA300_0984" fbc:label="SAUSA300_0984"/>
<fbc:geneProduct metaid="SAUSA300_0986" fbc:id="SAUSA300_0986" fbc:label="SAUSA300_0986"/>
<fbc:geneProduct metaid="SAUSA300_0987" fbc:id="SAUSA300_0987" fbc:label="SAUSA300_0987"/>
<fbc:geneProduct metaid="SAUSA300_0988" fbc:id="SAUSA300_0988" fbc:label="SAUSA300_0988"/>
<fbc:geneProduct metaid="SAUSA300_0993" fbc:id="SAUSA300_0993" fbc:label="SAUSA300_0993"/>
<fbc:geneProduct metaid="SAUSA300_0994" fbc:id="SAUSA300_0994" fbc:label="SAUSA300_0994"/>
<fbc:geneProduct metaid="SAUSA300_0995" fbc:id="SAUSA300_0995" fbc:label="SAUSA300_0995"/>
<fbc:geneProduct metaid="SAUSA300_0996" fbc:id="SAUSA300_0996" fbc:label="SAUSA300_0996"/>
<fbc:geneProduct metaid="SAUSA300_0999" fbc:id="SAUSA300_0999" fbc:label="SAUSA300_0999"/>
<fbc:geneProduct metaid="SAUSA300_1000" fbc:id="SAUSA300_1000" fbc:label="SAUSA300_1000"/>
<fbc:geneProduct metaid="SAUSA300_1001" fbc:id="SAUSA300_1001" fbc:label="SAUSA300_1001"/>
<fbc:geneProduct metaid="SAUSA300_1002" fbc:id="SAUSA300_1002" fbc:label="SAUSA300_1002"/>
<fbc:geneProduct metaid="SAUSA300_1005" fbc:id="SAUSA300_1005" fbc:label="SAUSA300_1005"/>
<fbc:geneProduct metaid="SAUSA300_1007" fbc:id="SAUSA300_1007" fbc:label="SAUSA300_1007"/>
<fbc:geneProduct metaid="SAUSA300_1014" fbc:id="SAUSA300_1014" fbc:label="SAUSA300_1014"/>
<fbc:geneProduct metaid="SAUSA300_1015" fbc:id="SAUSA300_1015" fbc:label="SAUSA300_1015"/>
<fbc:geneProduct metaid="SAUSA300_1016" fbc:id="SAUSA300_1016" fbc:label="SAUSA300_1016"/>
<fbc:geneProduct metaid="SAUSA300_1020" fbc:id="SAUSA300_1020" fbc:label="SAUSA300_1020"/>
<fbc:geneProduct metaid="SAUSA300_1024" fbc:id="SAUSA300_1024" fbc:label="SAUSA300_1024"/>
<fbc:geneProduct metaid="SAUSA300_1042" fbc:id="SAUSA300_1042" fbc:label="SAUSA300_1042"/>
<fbc:geneProduct metaid="SAUSA300_1044" fbc:id="SAUSA300_1044" fbc:label="SAUSA300_1044"/>
<fbc:geneProduct metaid="SAUSA300_1046" fbc:id="SAUSA300_1046" fbc:label="SAUSA300_1046"/>
<fbc:geneProduct metaid="SAUSA300_1047" fbc:id="SAUSA300_1047" fbc:label="SAUSA300_1047"/>
<fbc:geneProduct metaid="SAUSA300_1048" fbc:id="SAUSA300_1048" fbc:label="SAUSA300_1048"/>

<fbc:geneProduct metaid="SAUSA300_1049" fbc:id="SAUSA300_1049" fbc:label="SAUSA300_1049"/>
<fbc:geneProduct metaid="SAUSA300_1050" fbc:id="SAUSA300_1050" fbc:label="SAUSA300_1050"/>
<fbc:geneProduct metaid="SAUSA300_1062" fbc:id="SAUSA300_1062" fbc:label="SAUSA300_1062"/>
<fbc:geneProduct metaid="SAUSA300_1063" fbc:id="SAUSA300_1063" fbc:label="SAUSA300_1063"/>
<fbc:geneProduct metaid="SAUSA300_1064" fbc:id="SAUSA300_1064" fbc:label="SAUSA300_1064"/>
<fbc:geneProduct metaid="SAUSA300_1075" fbc:id="SAUSA300_1075" fbc:label="SAUSA300_1075"/>
<fbc:geneProduct metaid="SAUSA300_1076" fbc:id="SAUSA300_1076" fbc:label="SAUSA300_1076"/>
<fbc:geneProduct metaid="SAUSA300_1077" fbc:id="SAUSA300_1077" fbc:label="SAUSA300_1077"/>
<fbc:geneProduct metaid="SAUSA300_1082" fbc:id="SAUSA300_1082" fbc:label="SAUSA300_1082"/>
<fbc:geneProduct metaid="SAUSA300_1087" fbc:id="SAUSA300_1087" fbc:label="SAUSA300_1087"/>
<fbc:geneProduct metaid="SAUSA300_1090" fbc:id="SAUSA300_1090" fbc:label="SAUSA300_1090"/>
<fbc:geneProduct metaid="SAUSA300_1091" fbc:id="SAUSA300_1091" fbc:label="SAUSA300_1091"/>
<fbc:geneProduct metaid="SAUSA300_1092" fbc:id="SAUSA300_1092" fbc:label="SAUSA300_1092"/>
<fbc:geneProduct metaid="SAUSA300_1093" fbc:id="SAUSA300_1093" fbc:label="SAUSA300_1093"/>
<fbc:geneProduct metaid="SAUSA300_1094" fbc:id="SAUSA300_1094" fbc:label="SAUSA300_1094"/>
<fbc:geneProduct metaid="SAUSA300_1095" fbc:id="SAUSA300_1095" fbc:label="SAUSA300_1095"/>
<fbc:geneProduct metaid="SAUSA300_1096" fbc:id="SAUSA300_1096" fbc:label="SAUSA300_1096"/>
<fbc:geneProduct metaid="SAUSA300_1097" fbc:id="SAUSA300_1097" fbc:label="SAUSA300_1097"/>
<fbc:geneProduct metaid="SAUSA300_1098" fbc:id="SAUSA300_1098" fbc:label="SAUSA300_1098"/>
<fbc:geneProduct metaid="SAUSA300_1102" fbc:id="SAUSA300_1102" fbc:label="SAUSA300_1102"/>
<fbc:geneProduct metaid="SAUSA300_1104" fbc:id="SAUSA300_1104" fbc:label="SAUSA300_1104"/>
<fbc:geneProduct metaid="SAUSA300_1109" fbc:id="SAUSA300_1109" fbc:label="SAUSA300_1109"/>
<fbc:geneProduct metaid="SAUSA300_1115" fbc:id="SAUSA300_1115" fbc:label="SAUSA300_1115"/>
<fbc:geneProduct metaid="SAUSA300_1116" fbc:id="SAUSA300_1116" fbc:label="SAUSA300_1116"/>
<fbc:geneProduct metaid="SAUSA300_1121" fbc:id="SAUSA300_1121" fbc:label="SAUSA300_1121"/>
<fbc:geneProduct metaid="SAUSA300_1122" fbc:id="SAUSA300_1122" fbc:label="SAUSA300_1122"/>
<fbc:geneProduct metaid="SAUSA300_1123" fbc:id="SAUSA300_1123" fbc:label="SAUSA300_1123"/>
<fbc:geneProduct metaid="SAUSA300_1124" fbc:id="SAUSA300_1124" fbc:label="SAUSA300_1124"/>
<fbc:geneProduct metaid="SAUSA300_1138" fbc:id="SAUSA300_1138" fbc:label="SAUSA300_1138"/>

<fbc:geneProduct metaid="SAUSA300_1139" fbc:id="SAUSA300_1139" fbc:label="SAUSA300_1139"/>
<fbc:geneProduct metaid="SAUSA300_1151" fbc:id="SAUSA300_1151" fbc:label="SAUSA300_1151"/>
<fbc:geneProduct metaid="SAUSA300_1153" fbc:id="SAUSA300_1153" fbc:label="SAUSA300_1153"/>
<fbc:geneProduct metaid="SAUSA300_1154" fbc:id="SAUSA300_1154" fbc:label="SAUSA300_1154"/>
<fbc:geneProduct metaid="SAUSA300_1164" fbc:id="SAUSA300_1164" fbc:label="SAUSA300_1164"/>
<fbc:geneProduct metaid="SAUSA300_1165" fbc:id="SAUSA300_1165" fbc:label="SAUSA300_1165"/>
<fbc:geneProduct metaid="SAUSA300_1173" fbc:id="SAUSA300_1173" fbc:label="SAUSA300_1173"/>
<fbc:geneProduct metaid="SAUSA300_1176" fbc:id="SAUSA300_1176" fbc:label="SAUSA300_1176"/>
<fbc:geneProduct metaid="SAUSA300_1182" fbc:id="SAUSA300_1182" fbc:label="SAUSA300_1182"/>
<fbc:geneProduct metaid="SAUSA300_1183" fbc:id="SAUSA300_1183" fbc:label="SAUSA300_1183"/>
<fbc:geneProduct metaid="SAUSA300_1191" fbc:id="SAUSA300_1191" fbc:label="SAUSA300_1191"/>
<fbc:geneProduct metaid="SAUSA300_1192" fbc:id="SAUSA300_1192" fbc:label="SAUSA300_1192"/>
<fbc:geneProduct metaid="SAUSA300_1193" fbc:id="SAUSA300_1193" fbc:label="SAUSA300_1193"/>
<fbc:geneProduct metaid="SAUSA300_1194" fbc:id="SAUSA300_1194" fbc:label="SAUSA300_1194"/>
<fbc:geneProduct metaid="SAUSA300_1197" fbc:id="SAUSA300_1197" fbc:label="SAUSA300_1197"/>
<fbc:geneProduct metaid="SAUSA300_1201" fbc:id="SAUSA300_1201" fbc:label="SAUSA300_1201"/>
<fbc:geneProduct metaid="SAUSA300_1214" fbc:id="SAUSA300_1214" fbc:label="SAUSA300_1214"/>
<fbc:geneProduct metaid="SAUSA300_1216" fbc:id="SAUSA300_1216" fbc:label="SAUSA300_1216"/>
<fbc:geneProduct metaid="SAUSA300_1225" fbc:id="SAUSA300_1225" fbc:label="SAUSA300_1225"/>
<fbc:geneProduct metaid="SAUSA300_1226" fbc:id="SAUSA300_1226" fbc:label="SAUSA300_1226"/>
<fbc:geneProduct metaid="SAUSA300_1227" fbc:id="SAUSA300_1227" fbc:label="SAUSA300_1227"/>
<fbc:geneProduct metaid="SAUSA300_1228" fbc:id="SAUSA300_1228" fbc:label="SAUSA300_1228"/>
<fbc:geneProduct metaid="SAUSA300_1231" fbc:id="SAUSA300_1231" fbc:label="SAUSA300_1231"/>
<fbc:geneProduct metaid="SAUSA300_1232" fbc:id="SAUSA300_1232" fbc:label="SAUSA300_1232"/>
<fbc:geneProduct metaid="SAUSA300_1235" fbc:id="SAUSA300_1235" fbc:label="SAUSA300_1235"/>
<fbc:geneProduct metaid="SAUSA300_1239" fbc:id="SAUSA300_1239" fbc:label="SAUSA300_1239"/>
<fbc:geneProduct metaid="SAUSA300_1245" fbc:id="SAUSA300_1245" fbc:label="SAUSA300_1245"/>
<fbc:geneProduct metaid="SAUSA300_1246" fbc:id="SAUSA300_1246" fbc:label="SAUSA300_1246"/>
<fbc:geneProduct metaid="SAUSA300_1247" fbc:id="SAUSA300_1247" fbc:label="SAUSA300_1247"/>

<fbc:geneProduct metaid="SAUSA300_1249" fbc:id="SAUSA300_1249" fbc:label="SAUSA300_1249"/>
<fbc:geneProduct metaid="SAUSA300_1252" fbc:id="SAUSA300_1252" fbc:label="SAUSA300_1252"/>
<fbc:geneProduct metaid="SAUSA300_1255" fbc:id="SAUSA300_1255" fbc:label="SAUSA300_1255"/>
<fbc:geneProduct metaid="SAUSA300_1256" fbc:id="SAUSA300_1256" fbc:label="SAUSA300_1256"/>
<fbc:geneProduct metaid="SAUSA300_1257" fbc:id="SAUSA300_1257" fbc:label="SAUSA300_1257"/>
<fbc:geneProduct metaid="SAUSA300_1258" fbc:id="SAUSA300_1258" fbc:label="SAUSA300_1258"/>
<fbc:geneProduct metaid="SAUSA300_1260" fbc:id="SAUSA300_1260" fbc:label="SAUSA300_1260"/>
<fbc:geneProduct metaid="SAUSA300_1262" fbc:id="SAUSA300_1262" fbc:label="SAUSA300_1262"/>
<fbc:geneProduct metaid="SAUSA300_1263" fbc:id="SAUSA300_1263" fbc:label="SAUSA300_1263"/>
<fbc:geneProduct metaid="SAUSA300_1264" fbc:id="SAUSA300_1264" fbc:label="SAUSA300_1264"/>
<fbc:geneProduct metaid="SAUSA300_1265" fbc:id="SAUSA300_1265" fbc:label="SAUSA300_1265"/>
<fbc:geneProduct metaid="SAUSA300_1266" fbc:id="SAUSA300_1266" fbc:label="SAUSA300_1266"/>
<fbc:geneProduct metaid="SAUSA300_1267" fbc:id="SAUSA300_1267" fbc:label="SAUSA300_1267"/>
<fbc:geneProduct metaid="SAUSA300_1268" fbc:id="SAUSA300_1268" fbc:label="SAUSA300_1268"/>
<fbc:geneProduct metaid="SAUSA300_1269" fbc:id="SAUSA300_1269" fbc:label="SAUSA300_1269"/>
<fbc:geneProduct metaid="SAUSA300_1270" fbc:id="SAUSA300_1270" fbc:label="SAUSA300_1270"/>
<fbc:geneProduct metaid="SAUSA300_1273" fbc:id="SAUSA300_1273" fbc:label="SAUSA300_1273"/>
<fbc:geneProduct metaid="SAUSA300_1274" fbc:id="SAUSA300_1274" fbc:label="SAUSA300_1274"/>
<fbc:geneProduct metaid="SAUSA300_1275" fbc:id="SAUSA300_1275" fbc:label="SAUSA300_1275"/>
<fbc:geneProduct metaid="SAUSA300_1276" fbc:id="SAUSA300_1276" fbc:label="SAUSA300_1276"/>
<fbc:geneProduct metaid="SAUSA300_1280" fbc:id="SAUSA300_1280" fbc:label="SAUSA300_1280"/>
<fbc:geneProduct metaid="SAUSA300_1281" fbc:id="SAUSA300_1281" fbc:label="SAUSA300_1281"/>
<fbc:geneProduct metaid="SAUSA300_1282" fbc:id="SAUSA300_1282" fbc:label="SAUSA300_1282"/>
<fbc:geneProduct metaid="SAUSA300_1283" fbc:id="SAUSA300_1283" fbc:label="SAUSA300_1283"/>
<fbc:geneProduct metaid="SAUSA300_1286" fbc:id="SAUSA300_1286" fbc:label="SAUSA300_1286"/>
<fbc:geneProduct metaid="SAUSA300_1287" fbc:id="SAUSA300_1287" fbc:label="SAUSA300_1287"/>
<fbc:geneProduct metaid="SAUSA300_1288" fbc:id="SAUSA300_1288" fbc:label="SAUSA300_1288"/>
<fbc:geneProduct metaid="SAUSA300_1289" fbc:id="SAUSA300_1289" fbc:label="SAUSA300_1289"/>
<fbc:geneProduct metaid="SAUSA300_1290" fbc:id="SAUSA300_1290" fbc:label="SAUSA300_1290"/>

<fbc:geneProduct metaid="SAUSA300_1291" fbc:id="SAUSA300_1291" fbc:label="SAUSA300_1291"/>
<fbc:geneProduct metaid="SAUSA300_1292" fbc:id="SAUSA300_1292" fbc:label="SAUSA300_1292"/>
<fbc:geneProduct metaid="SAUSA300_1293" fbc:id="SAUSA300_1293" fbc:label="SAUSA300_1293"/>
<fbc:geneProduct metaid="SAUSA300_1297" fbc:id="SAUSA300_1297" fbc:label="SAUSA300_1297"/>
<fbc:geneProduct metaid="SAUSA300_1300" fbc:id="SAUSA300_1300" fbc:label="SAUSA300_1300"/>
<fbc:geneProduct metaid="SAUSA300_1305" fbc:id="SAUSA300_1305" fbc:label="SAUSA300_1305"/>
<fbc:geneProduct metaid="SAUSA300_1306" fbc:id="SAUSA300_1306" fbc:label="SAUSA300_1306"/>
<fbc:geneProduct metaid="SAUSA300_1310" fbc:id="SAUSA300_1310" fbc:label="SAUSA300_1310"/>
<fbc:geneProduct metaid="SAUSA300_1311" fbc:id="SAUSA300_1311" fbc:label="SAUSA300_1311"/>
<fbc:geneProduct metaid="SAUSA300_1315" fbc:id="SAUSA300_1315" fbc:label="SAUSA300_1315"/>
<fbc:geneProduct metaid="SAUSA300_1316" fbc:id="SAUSA300_1316" fbc:label="SAUSA300_1316"/>
<fbc:geneProduct metaid="SAUSA300_1317" fbc:id="SAUSA300_1317" fbc:label="SAUSA300_1317"/>
<fbc:geneProduct metaid="SAUSA300_1319" fbc:id="SAUSA300_1319" fbc:label="SAUSA300_1319"/>
<fbc:geneProduct metaid="SAUSA300_1320" fbc:id="SAUSA300_1320" fbc:label="SAUSA300_1320"/>
<fbc:geneProduct metaid="SAUSA300_1330" fbc:id="SAUSA300_1330" fbc:label="SAUSA300_1330"/>
<fbc:geneProduct metaid="SAUSA300_1331" fbc:id="SAUSA300_1331" fbc:label="SAUSA300_1331"/>
<fbc:geneProduct metaid="SAUSA300_1341" fbc:id="SAUSA300_1341" fbc:label="SAUSA300_1341"/>
<fbc:geneProduct metaid="SAUSA300_1345" fbc:id="SAUSA300_1345" fbc:label="SAUSA300_1345"/>
<fbc:geneProduct metaid="SAUSA300_1347" fbc:id="SAUSA300_1347" fbc:label="SAUSA300_1347"/>
<fbc:geneProduct metaid="SAUSA300_1349" fbc:id="SAUSA300_1349" fbc:label="SAUSA300_1349"/>
<fbc:geneProduct metaid="SAUSA300_1355" fbc:id="SAUSA300_1355" fbc:label="SAUSA300_1355"/>
<fbc:geneProduct metaid="SAUSA300_1356" fbc:id="SAUSA300_1356" fbc:label="SAUSA300_1356"/>
<fbc:geneProduct metaid="SAUSA300_1357" fbc:id="SAUSA300_1357" fbc:label="SAUSA300_1357"/>
<fbc:geneProduct metaid="SAUSA300_1358" fbc:id="SAUSA300_1358" fbc:label="SAUSA300_1358"/>
<fbc:geneProduct metaid="SAUSA300_1359" fbc:id="SAUSA300_1359" fbc:label="SAUSA300_1359"/>
<fbc:geneProduct metaid="SAUSA300_1360" fbc:id="SAUSA300_1360" fbc:label="SAUSA300_1360"/>
<fbc:geneProduct metaid="SAUSA300_1361" fbc:id="SAUSA300_1361" fbc:label="SAUSA300_1361"/>
<fbc:geneProduct metaid="SAUSA300_1363" fbc:id="SAUSA300_1363" fbc:label="SAUSA300_1363"/>
<fbc:geneProduct metaid="SAUSA300_1367" fbc:id="SAUSA300_1367" fbc:label="SAUSA300_1367"/>

<fbc:geneProduct metaid="SAUSA300_1368" fbc:id="SAUSA300_1368" fbc:label="SAUSA300_1368"/>
<fbc:geneProduct metaid="SAUSA300_1369" fbc:id="SAUSA300_1369" fbc:label="SAUSA300_1369"/>
<fbc:geneProduct metaid="SAUSA300_1371" fbc:id="SAUSA300_1371" fbc:label="SAUSA300_1371"/>
<fbc:geneProduct metaid="SAUSA300_1374" fbc:id="SAUSA300_1374" fbc:label="SAUSA300_1374"/>
<fbc:geneProduct metaid="SAUSA300_1383" fbc:id="SAUSA300_1383" fbc:label="SAUSA300_1383"/>
<fbc:geneProduct metaid="SAUSA300_1443" fbc:id="SAUSA300_1443" fbc:label="SAUSA300_1443"/>
<fbc:geneProduct metaid="SAUSA300_1449" fbc:id="SAUSA300_1449" fbc:label="SAUSA300_1449"/>
<fbc:geneProduct metaid="SAUSA300_1452" fbc:id="SAUSA300_1452" fbc:label="SAUSA300_1452"/>
<fbc:geneProduct metaid="SAUSA300_1454" fbc:id="SAUSA300_1454" fbc:label="SAUSA300_1454"/>
<fbc:geneProduct metaid="SAUSA300_1456" fbc:id="SAUSA300_1456" fbc:label="SAUSA300_1456"/>
<fbc:geneProduct metaid="SAUSA300_1458" fbc:id="SAUSA300_1458" fbc:label="SAUSA300_1458"/>
<fbc:geneProduct metaid="SAUSA300_1459" fbc:id="SAUSA300_1459" fbc:label="SAUSA300_1459"/>
<fbc:geneProduct metaid="SAUSA300_1464" fbc:id="SAUSA300_1464" fbc:label="SAUSA300_1464"/>
<fbc:geneProduct metaid="SAUSA300_1465" fbc:id="SAUSA300_1465" fbc:label="SAUSA300_1465"/>
<fbc:geneProduct metaid="SAUSA300_1466" fbc:id="SAUSA300_1466" fbc:label="SAUSA300_1466"/>
<fbc:geneProduct metaid="SAUSA300_1467" fbc:id="SAUSA300_1467" fbc:label="SAUSA300_1467"/>
<fbc:geneProduct metaid="SAUSA300_1470" fbc:id="SAUSA300_1470" fbc:label="SAUSA300_1470"/>
<fbc:geneProduct metaid="SAUSA300_1475" fbc:id="SAUSA300_1475" fbc:label="SAUSA300_1475"/>
<fbc:geneProduct metaid="SAUSA300_1476" fbc:id="SAUSA300_1476" fbc:label="SAUSA300_1476"/>
<fbc:geneProduct metaid="SAUSA300_1491" fbc:id="SAUSA300_1491" fbc:label="SAUSA300_1491"/>
<fbc:geneProduct metaid="SAUSA300_1494" fbc:id="SAUSA300_1494" fbc:label="SAUSA300_1494"/>
<fbc:geneProduct metaid="SAUSA300_1496" fbc:id="SAUSA300_1496" fbc:label="SAUSA300_1496"/>
<fbc:geneProduct metaid="SAUSA300_1497" fbc:id="SAUSA300_1497" fbc:label="SAUSA300_1497"/>
<fbc:geneProduct metaid="SAUSA300_1498" fbc:id="SAUSA300_1498" fbc:label="SAUSA300_1498"/>
<fbc:geneProduct metaid="SAUSA300_1499" fbc:id="SAUSA300_1499" fbc:label="SAUSA300_1499"/>
<fbc:geneProduct metaid="SAUSA300_1505" fbc:id="SAUSA300_1505" fbc:label="SAUSA300_1505"/>
<fbc:geneProduct metaid="SAUSA300_1507" fbc:id="SAUSA300_1507" fbc:label="SAUSA300_1507"/>
<fbc:geneProduct metaid="SAUSA300_1510" fbc:id="SAUSA300_1510" fbc:label="SAUSA300_1510"/>
<fbc:geneProduct metaid="SAUSA300_1513" fbc:id="SAUSA300_1513" fbc:label="SAUSA300_1513"/>

<fbc:geneProduct metaid="SAUSA300_1515" fbc:id="SAUSA300_1515" fbc:label="SAUSA300_1515"/>
<fbc:geneProduct metaid="SAUSA300_1516" fbc:id="SAUSA300_1516" fbc:label="SAUSA300_1516"/>
<fbc:geneProduct metaid="SAUSA300_1519" fbc:id="SAUSA300_1519" fbc:label="SAUSA300_1519"/>
<fbc:geneProduct metaid="SAUSA300_1525" fbc:id="SAUSA300_1525" fbc:label="SAUSA300_1525"/>
<fbc:geneProduct metaid="SAUSA300_1528" fbc:id="SAUSA300_1528" fbc:label="SAUSA300_1528"/>
<fbc:geneProduct metaid="SAUSA300_1529" fbc:id="SAUSA300_1529" fbc:label="SAUSA300_1529"/>
<fbc:geneProduct metaid="SAUSA300_1543" fbc:id="SAUSA300_1543" fbc:label="SAUSA300_1543"/>
<fbc:geneProduct metaid="SAUSA300_1548" fbc:id="SAUSA300_1548" fbc:label="SAUSA300_1548"/>
<fbc:geneProduct metaid="SAUSA300_1553" fbc:id="SAUSA300_1553" fbc:label="SAUSA300_1553"/>
<fbc:geneProduct metaid="SAUSA300_1555" fbc:id="SAUSA300_1555" fbc:label="SAUSA300_1555"/>
<fbc:geneProduct metaid="SAUSA300_1558" fbc:id="SAUSA300_1558" fbc:label="SAUSA300_1558"/>
<fbc:geneProduct metaid="SAUSA300_1563" fbc:id="SAUSA300_1563" fbc:label="SAUSA300_1563"/>
<fbc:geneProduct metaid="SAUSA300_1564" fbc:id="SAUSA300_1564" fbc:label="SAUSA300_1564"/>
<fbc:geneProduct metaid="SAUSA300_1565" fbc:id="SAUSA300_1565" fbc:label="SAUSA300_1565"/>
<fbc:geneProduct metaid="SAUSA300_1566" fbc:id="SAUSA300_1566" fbc:label="SAUSA300_1566"/>
<fbc:geneProduct metaid="SAUSA300_1568" fbc:id="SAUSA300_1568" fbc:label="SAUSA300_1568"/>
<fbc:geneProduct metaid="SAUSA300_1575" fbc:id="SAUSA300_1575" fbc:label="SAUSA300_1575"/>
<fbc:geneProduct metaid="SAUSA300_1579" fbc:id="SAUSA300_1579" fbc:label="SAUSA300_1579"/>
<fbc:geneProduct metaid="SAUSA300_1586" fbc:id="SAUSA300_1586" fbc:label="SAUSA300_1586"/>
<fbc:geneProduct metaid="SAUSA300_1588" fbc:id="SAUSA300_1588" fbc:label="SAUSA300_1588"/>
<fbc:geneProduct metaid="SAUSA300_1590" fbc:id="SAUSA300_1590" fbc:label="SAUSA300_1590"/>
<fbc:geneProduct metaid="SAUSA300_1591" fbc:id="SAUSA300_1591" fbc:label="SAUSA300_1591"/>
<fbc:geneProduct metaid="SAUSA300_1610" fbc:id="SAUSA300_1610" fbc:label="SAUSA300_1610"/>
<fbc:geneProduct metaid="SAUSA300_1614" fbc:id="SAUSA300_1614" fbc:label="SAUSA300_1614"/>
<fbc:geneProduct metaid="SAUSA300_1615" fbc:id="SAUSA300_1615" fbc:label="SAUSA300_1615"/>
<fbc:geneProduct metaid="SAUSA300_1616" fbc:id="SAUSA300_1616" fbc:label="SAUSA300_1616"/>
<fbc:geneProduct metaid="SAUSA300_1617" fbc:id="SAUSA300_1617" fbc:label="SAUSA300_1617"/>
<fbc:geneProduct metaid="SAUSA300_1619" fbc:id="SAUSA300_1619" fbc:label="SAUSA300_1619"/>
<fbc:geneProduct metaid="SAUSA300_1628" fbc:id="SAUSA300_1628" fbc:label="SAUSA300_1628"/>

<fbc:geneProduct metaid="SAUSA300_1629" fbc:id="SAUSA300_1629" fbc:label="SAUSA300_1629"/>
<fbc:geneProduct metaid="SAUSA300_1633" fbc:id="SAUSA300_1633" fbc:label="SAUSA300_1633"/>
<fbc:geneProduct metaid="SAUSA300_1634" fbc:id="SAUSA300_1634" fbc:label="SAUSA300_1634"/>
<fbc:geneProduct metaid="SAUSA300_1640" fbc:id="SAUSA300_1640" fbc:label="SAUSA300_1640"/>
<fbc:geneProduct metaid="SAUSA300_1641" fbc:id="SAUSA300_1641" fbc:label="SAUSA300_1641"/>
<fbc:geneProduct metaid="SAUSA300_1642" fbc:id="SAUSA300_1642" fbc:label="SAUSA300_1642"/>
<fbc:geneProduct metaid="SAUSA300_1644" fbc:id="SAUSA300_1644" fbc:label="SAUSA300_1644"/>
<fbc:geneProduct metaid="SAUSA300_1645" fbc:id="SAUSA300_1645" fbc:label="SAUSA300_1645"/>
<fbc:geneProduct metaid="SAUSA300_1646" fbc:id="SAUSA300_1646" fbc:label="SAUSA300_1646"/>
<fbc:geneProduct metaid="SAUSA300_1647" fbc:id="SAUSA300_1647" fbc:label="SAUSA300_1647"/>
<fbc:geneProduct metaid="SAUSA300_1648" fbc:id="SAUSA300_1648" fbc:label="SAUSA300_1648"/>
<fbc:geneProduct metaid="SAUSA300_1650" fbc:id="SAUSA300_1650" fbc:label="SAUSA300_1650"/>
<fbc:geneProduct metaid="SAUSA300_1655" fbc:id="SAUSA300_1655" fbc:label="SAUSA300_1655"/>
<fbc:geneProduct metaid="SAUSA300_1657" fbc:id="SAUSA300_1657" fbc:label="SAUSA300_1657"/>
<fbc:geneProduct metaid="SAUSA300_1662" fbc:id="SAUSA300_1662" fbc:label="SAUSA300_1662"/>
<fbc:geneProduct metaid="SAUSA300_1667" fbc:id="SAUSA300_1667" fbc:label="SAUSA300_1667"/>
<fbc:geneProduct metaid="SAUSA300_1669" fbc:id="SAUSA300_1669" fbc:label="SAUSA300_1669"/>
<fbc:geneProduct metaid="SAUSA300_1670" fbc:id="SAUSA300_1670" fbc:label="SAUSA300_1670"/>
<fbc:geneProduct metaid="SAUSA300_1671" fbc:id="SAUSA300_1671" fbc:label="SAUSA300_1671"/>
<fbc:geneProduct metaid="SAUSA300_1672" fbc:id="SAUSA300_1672" fbc:label="SAUSA300_1672"/>
<fbc:geneProduct metaid="SAUSA300_1673" fbc:id="SAUSA300_1673" fbc:label="SAUSA300_1673"/>
<fbc:geneProduct metaid="SAUSA300_1676" fbc:id="SAUSA300_1676" fbc:label="SAUSA300_1676"/>
<fbc:geneProduct metaid="SAUSA300_1678" fbc:id="SAUSA300_1678" fbc:label="SAUSA300_1678"/>
<fbc:geneProduct metaid="SAUSA300_1679" fbc:id="SAUSA300_1679" fbc:label="SAUSA300_1679"/>
<fbc:geneProduct metaid="SAUSA300_1681" fbc:id="SAUSA300_1681" fbc:label="SAUSA300_1681"/>
<fbc:geneProduct metaid="SAUSA300_1683" fbc:id="SAUSA300_1683" fbc:label="SAUSA300_1683"/>
<fbc:geneProduct metaid="SAUSA300_1686" fbc:id="SAUSA300_1686" fbc:label="SAUSA300_1686"/>
<fbc:geneProduct metaid="SAUSA300_1696" fbc:id="SAUSA300_1696" fbc:label="SAUSA300_1696"/>
<fbc:geneProduct metaid="SAUSA300_1697" fbc:id="SAUSA300_1697" fbc:label="SAUSA300_1697"/>

<fbc:geneProduct metaid="SAUSA300_1699" fbc:id="SAUSA300_1699" fbc:label="SAUSA300_1699"/>
<fbc:geneProduct metaid="SAUSA300_1710" fbc:id="SAUSA300_1710" fbc:label="SAUSA300_1710"/>
<fbc:geneProduct metaid="SAUSA300_1711" fbc:id="SAUSA300_1711" fbc:label="SAUSA300_1711"/>
<fbc:geneProduct metaid="SAUSA300_1712" fbc:id="SAUSA300_1712" fbc:label="SAUSA300_1712"/>
<fbc:geneProduct metaid="SAUSA300_1713" fbc:id="SAUSA300_1713" fbc:label="SAUSA300_1713"/>
<fbc:geneProduct metaid="SAUSA300_1714" fbc:id="SAUSA300_1714" fbc:label="SAUSA300_1714"/>
<fbc:geneProduct metaid="SAUSA300_1715" fbc:id="SAUSA300_1715" fbc:label="SAUSA300_1715"/>
<fbc:geneProduct metaid="SAUSA300_1718" fbc:id="SAUSA300_1718" fbc:label="SAUSA300_1718"/>
<fbc:geneProduct metaid="SAUSA300_1724" fbc:id="SAUSA300_1724" fbc:label="SAUSA300_1724"/>
<fbc:geneProduct metaid="SAUSA300_1725" fbc:id="SAUSA300_1725" fbc:label="SAUSA300_1725"/>
<fbc:geneProduct metaid="SAUSA300_1728" fbc:id="SAUSA300_1728" fbc:label="SAUSA300_1728"/>
<fbc:geneProduct metaid="SAUSA300_1730" fbc:id="SAUSA300_1730" fbc:label="SAUSA300_1730"/>
<fbc:geneProduct metaid="SAUSA300_1731" fbc:id="SAUSA300_1731" fbc:label="SAUSA300_1731"/>
<fbc:geneProduct metaid="SAUSA300_1735" fbc:id="SAUSA300_1735" fbc:label="SAUSA300_1735"/>
<fbc:geneProduct metaid="SAUSA300_1737" fbc:id="SAUSA300_1737" fbc:label="SAUSA300_1737"/>
<fbc:geneProduct metaid="SAUSA300_1741" fbc:id="SAUSA300_1741" fbc:label="SAUSA300_1741"/>
<fbc:geneProduct metaid="SAUSA300_1781" fbc:id="SAUSA300_1781" fbc:label="SAUSA300_1781"/>
<fbc:geneProduct metaid="SAUSA300_1782" fbc:id="SAUSA300_1782" fbc:label="SAUSA300_1782"/>
<fbc:geneProduct metaid="SAUSA300_1783" fbc:id="SAUSA300_1783" fbc:label="SAUSA300_1783"/>
<fbc:geneProduct metaid="SAUSA300_1801" fbc:id="SAUSA300_1801" fbc:label="SAUSA300_1801"/>
<fbc:geneProduct metaid="SAUSA300_1807" fbc:id="SAUSA300_1807" fbc:label="SAUSA300_1807"/>
<fbc:geneProduct metaid="SAUSA300_1808" fbc:id="SAUSA300_1808" fbc:label="SAUSA300_1808"/>
<fbc:geneProduct metaid="SAUSA300_1845" fbc:id="SAUSA300_1845" fbc:label="SAUSA300_1845"/>
<fbc:geneProduct metaid="SAUSA300_1851" fbc:id="SAUSA300_1851" fbc:label="SAUSA300_1851"/>
<fbc:geneProduct metaid="SAUSA300_1852" fbc:id="SAUSA300_1852" fbc:label="SAUSA300_1852"/>
<fbc:geneProduct metaid="SAUSA300_1855" fbc:id="SAUSA300_1855" fbc:label="SAUSA300_1855"/>
<fbc:geneProduct metaid="SAUSA300_1860" fbc:id="SAUSA300_1860" fbc:label="SAUSA300_1860"/>
<fbc:geneProduct metaid="SAUSA300_1868" fbc:id="SAUSA300_1868" fbc:label="SAUSA300_1868"/>
<fbc:geneProduct metaid="SAUSA300_1869" fbc:id="SAUSA300_1869" fbc:label="SAUSA300_1869"/>

<fbc:geneProduct metaid="SAUSA300_1872" fbc:id="SAUSA300_1872" fbc:label="SAUSA300_1872"/>
<fbc:geneProduct metaid="SAUSA300_1873" fbc:id="SAUSA300_1873" fbc:label="SAUSA300_1873"/>
<fbc:geneProduct metaid="SAUSA300_1879" fbc:id="SAUSA300_1879" fbc:label="SAUSA300_1879"/>
<fbc:geneProduct metaid="SAUSA300_1883" fbc:id="SAUSA300_1883" fbc:label="SAUSA300_1883"/>
<fbc:geneProduct metaid="SAUSA300_1885" fbc:id="SAUSA300_1885" fbc:label="SAUSA300_1885"/>
<fbc:geneProduct metaid="SAUSA300_1889" fbc:id="SAUSA300_1889" fbc:label="SAUSA300_1889"/>
<fbc:geneProduct metaid="SAUSA300_1893" fbc:id="SAUSA300_1893" fbc:label="SAUSA300_1893"/>
<fbc:geneProduct metaid="SAUSA300_1894" fbc:id="SAUSA300_1894" fbc:label="SAUSA300_1894"/>
<fbc:geneProduct metaid="SAUSA300_1895" fbc:id="SAUSA300_1895" fbc:label="SAUSA300_1895"/>
<fbc:geneProduct metaid="SAUSA300_1896" fbc:id="SAUSA300_1896" fbc:label="SAUSA300_1896"/>
<fbc:geneProduct metaid="SAUSA300_1897" fbc:id="SAUSA300_1897" fbc:label="SAUSA300_1897"/>
<fbc:geneProduct metaid="SAUSA300_1899" fbc:id="SAUSA300_1899" fbc:label="SAUSA300_1899"/>
<fbc:geneProduct metaid="SAUSA300_1900" fbc:id="SAUSA300_1900" fbc:label="SAUSA300_1900"/>
<fbc:geneProduct metaid="SAUSA300_1901" fbc:id="SAUSA300_1901" fbc:label="SAUSA300_1901"/>
<fbc:geneProduct metaid="SAUSA300_1902" fbc:id="SAUSA300_1902" fbc:label="SAUSA300_1902"/>
<fbc:geneProduct metaid="SAUSA300_1909" fbc:id="SAUSA300_1909" fbc:label="SAUSA300_1909"/>
<fbc:geneProduct metaid="SAUSA300_1916" fbc:id="SAUSA300_1916" fbc:label="SAUSA300_1916"/>
<fbc:geneProduct metaid="SAUSA300_1949" fbc:id="SAUSA300_1949" fbc:label="SAUSA300_1949"/>
<fbc:geneProduct metaid="SAUSA300_1973" fbc:id="SAUSA300_1973" fbc:label="SAUSA300_1973"/>
<fbc:geneProduct metaid="SAUSA300_1976" fbc:id="SAUSA300_1976" fbc:label="SAUSA300_1976"/>
<fbc:geneProduct metaid="SAUSA300_1979" fbc:id="SAUSA300_1979" fbc:label="SAUSA300_1979"/>
<fbc:geneProduct metaid="SAUSA300_1987" fbc:id="SAUSA300_1987" fbc:label="SAUSA300_1987"/>
<fbc:geneProduct metaid="SAUSA300_1993" fbc:id="SAUSA300_1993" fbc:label="SAUSA300_1993"/>
<fbc:geneProduct metaid="SAUSA300_1994" fbc:id="SAUSA300_1994" fbc:label="SAUSA300_1994"/>
<fbc:geneProduct metaid="SAUSA300_1996" fbc:id="SAUSA300_1996" fbc:label="SAUSA300_1996"/>
<fbc:geneProduct metaid="SAUSA300_2006" fbc:id="SAUSA300_2006" fbc:label="SAUSA300_2006"/>
<fbc:geneProduct metaid="SAUSA300_2007" fbc:id="SAUSA300_2007" fbc:label="SAUSA300_2007"/>
<fbc:geneProduct metaid="SAUSA300_2008" fbc:id="SAUSA300_2008" fbc:label="SAUSA300_2008"/>
<fbc:geneProduct metaid="SAUSA300_2009" fbc:id="SAUSA300_2009" fbc:label="SAUSA300_2009"/>

<fbc:geneProduct metaid="SAUSA300_2010" fbc:id="SAUSA300_2010" fbc:label="SAUSA300_2010"/>
<fbc:geneProduct metaid="SAUSA300_2011" fbc:id="SAUSA300_2011" fbc:label="SAUSA300_2011"/>
<fbc:geneProduct metaid="SAUSA300_2012" fbc:id="SAUSA300_2012" fbc:label="SAUSA300_2012"/>
<fbc:geneProduct metaid="SAUSA300_2013" fbc:id="SAUSA300_2013" fbc:label="SAUSA300_2013"/>
<fbc:geneProduct metaid="SAUSA300_2014" fbc:id="SAUSA300_2014" fbc:label="SAUSA300_2014"/>
<fbc:geneProduct metaid="SAUSA300_2027" fbc:id="SAUSA300_2027" fbc:label="SAUSA300_2027"/>
<fbc:geneProduct metaid="SAUSA300_2028" fbc:id="SAUSA300_2028" fbc:label="SAUSA300_2028"/>
<fbc:geneProduct metaid="SAUSA300_2032" fbc:id="SAUSA300_2032" fbc:label="SAUSA300_2032"/>
<fbc:geneProduct metaid="SAUSA300_2033" fbc:id="SAUSA300_2033" fbc:label="SAUSA300_2033"/>
<fbc:geneProduct metaid="SAUSA300_2034" fbc:id="SAUSA300_2034" fbc:label="SAUSA300_2034"/>
<fbc:geneProduct metaid="SAUSA300_2038" fbc:id="SAUSA300_2038" fbc:label="SAUSA300_2038"/>
<fbc:geneProduct metaid="SAUSA300_2039" fbc:id="SAUSA300_2039" fbc:label="SAUSA300_2039"/>
<fbc:geneProduct metaid="SAUSA300_2044" fbc:id="SAUSA300_2044" fbc:label="SAUSA300_2044"/>
<fbc:geneProduct metaid="SAUSA300_2047" fbc:id="SAUSA300_2047" fbc:label="SAUSA300_2047"/>
<fbc:geneProduct metaid="SAUSA300_2048" fbc:id="SAUSA300_2048" fbc:label="SAUSA300_2048"/>
<fbc:geneProduct metaid="SAUSA300_2049" fbc:id="SAUSA300_2049" fbc:label="SAUSA300_2049"/>
<fbc:geneProduct metaid="SAUSA300_2054" fbc:id="SAUSA300_2054" fbc:label="SAUSA300_2054"/>
<fbc:geneProduct metaid="SAUSA300_2055" fbc:id="SAUSA300_2055" fbc:label="SAUSA300_2055"/>
<fbc:geneProduct metaid="SAUSA300_2057" fbc:id="SAUSA300_2057" fbc:label="SAUSA300_2057"/>
<fbc:geneProduct metaid="SAUSA300_2058" fbc:id="SAUSA300_2058" fbc:label="SAUSA300_2058"/>
<fbc:geneProduct metaid="SAUSA300_2059" fbc:id="SAUSA300_2059" fbc:label="SAUSA300_2059"/>
<fbc:geneProduct metaid="SAUSA300_2060" fbc:id="SAUSA300_2060" fbc:label="SAUSA300_2060"/>
<fbc:geneProduct metaid="SAUSA300_2061" fbc:id="SAUSA300_2061" fbc:label="SAUSA300_2061"/>
<fbc:geneProduct metaid="SAUSA300_2062" fbc:id="SAUSA300_2062" fbc:label="SAUSA300_2062"/>
<fbc:geneProduct metaid="SAUSA300_2063" fbc:id="SAUSA300_2063" fbc:label="SAUSA300_2063"/>
<fbc:geneProduct metaid="SAUSA300_2064" fbc:id="SAUSA300_2064" fbc:label="SAUSA300_2064"/>
<fbc:geneProduct metaid="SAUSA300_2065" fbc:id="SAUSA300_2065" fbc:label="SAUSA300_2065"/>
<fbc:geneProduct metaid="SAUSA300_2066" fbc:id="SAUSA300_2066" fbc:label="SAUSA300_2066"/>
<fbc:geneProduct metaid="SAUSA300_2067" fbc:id="SAUSA300_2067" fbc:label="SAUSA300_2067"/>

<fbc:geneProduct metaid="SAUSA300_2073" fbc:id="SAUSA300_2073" fbc:label="SAUSA300_2073"/>
<fbc:geneProduct metaid="SAUSA300_2076" fbc:id="SAUSA300_2076" fbc:label="SAUSA300_2076"/>
<fbc:geneProduct metaid="SAUSA300_2078" fbc:id="SAUSA300_2078" fbc:label="SAUSA300_2078"/>
<fbc:geneProduct metaid="SAUSA300_2079" fbc:id="SAUSA300_2079" fbc:label="SAUSA300_2079"/>
<fbc:geneProduct metaid="SAUSA300_2081" fbc:id="SAUSA300_2081" fbc:label="SAUSA300_2081"/>
<fbc:geneProduct metaid="SAUSA300_2084" fbc:id="SAUSA300_2084" fbc:label="SAUSA300_2084"/>
<fbc:geneProduct metaid="SAUSA300_2088" fbc:id="SAUSA300_2088" fbc:label="SAUSA300_2088"/>
<fbc:geneProduct metaid="SAUSA300_2089" fbc:id="SAUSA300_2089" fbc:label="SAUSA300_2089"/>
<fbc:geneProduct metaid="SAUSA300_2090" fbc:id="SAUSA300_2090" fbc:label="SAUSA300_2090"/>
<fbc:geneProduct metaid="SAUSA300_2091" fbc:id="SAUSA300_2091" fbc:label="SAUSA300_2091"/>
<fbc:geneProduct metaid="SAUSA300_2092" fbc:id="SAUSA300_2092" fbc:label="SAUSA300_2092"/>
<fbc:geneProduct metaid="SAUSA300_2096" fbc:id="SAUSA300_2096" fbc:label="SAUSA300_2096"/>
<fbc:geneProduct metaid="SAUSA300_2104" fbc:id="SAUSA300_2104" fbc:label="SAUSA300_2104"/>
<fbc:geneProduct metaid="SAUSA300_2105" fbc:id="SAUSA300_2105" fbc:label="SAUSA300_2105"/>
<fbc:geneProduct metaid="SAUSA300_2106" fbc:id="SAUSA300_2106" fbc:label="SAUSA300_2106"/>
<fbc:geneProduct metaid="SAUSA300_2107" fbc:id="SAUSA300_2107" fbc:label="SAUSA300_2107"/>
<fbc:geneProduct metaid="SAUSA300_2108" fbc:id="SAUSA300_2108" fbc:label="SAUSA300_2108"/>
<fbc:geneProduct metaid="SAUSA300_2111" fbc:id="SAUSA300_2111" fbc:label="SAUSA300_2111"/>
<fbc:geneProduct metaid="SAUSA300_2114" fbc:id="SAUSA300_2114" fbc:label="SAUSA300_2114"/>
<fbc:geneProduct metaid="SAUSA300_2130" fbc:id="SAUSA300_2130" fbc:label="SAUSA300_2130"/>
<fbc:geneProduct metaid="SAUSA300_2134" fbc:id="SAUSA300_2134" fbc:label="SAUSA300_2134"/>
<fbc:geneProduct metaid="SAUSA300_2135" fbc:id="SAUSA300_2135" fbc:label="SAUSA300_2135"/>
<fbc:geneProduct metaid="SAUSA300_2136" fbc:id="SAUSA300_2136" fbc:label="SAUSA300_2136"/>
<fbc:geneProduct metaid="SAUSA300_2145" fbc:id="SAUSA300_2145" fbc:label="SAUSA300_2145"/>
<fbc:geneProduct metaid="SAUSA300_2147" fbc:id="SAUSA300_2147" fbc:label="SAUSA300_2147"/>
<fbc:geneProduct metaid="SAUSA300_2149" fbc:id="SAUSA300_2149" fbc:label="SAUSA300_2149"/>
<fbc:geneProduct metaid="SAUSA300_2150" fbc:id="SAUSA300_2150" fbc:label="SAUSA300_2150"/>
<fbc:geneProduct metaid="SAUSA300_2151" fbc:id="SAUSA300_2151" fbc:label="SAUSA300_2151"/>
<fbc:geneProduct metaid="SAUSA300_2152" fbc:id="SAUSA300_2152" fbc:label="SAUSA300_2152"/>

<fbc:geneProduct metaid="SAUSA300_2153" fbc:id="SAUSA300_2153" fbc:label="SAUSA300_2153"/>
<fbc:geneProduct metaid="SAUSA300_2154" fbc:id="SAUSA300_2154" fbc:label="SAUSA300_2154"/>
<fbc:geneProduct metaid="SAUSA300_2155" fbc:id="SAUSA300_2155" fbc:label="SAUSA300_2155"/>
<fbc:geneProduct metaid="SAUSA300_2165" fbc:id="SAUSA300_2165" fbc:label="SAUSA300_2165"/>
<fbc:geneProduct metaid="SAUSA300_2166" fbc:id="SAUSA300_2166" fbc:label="SAUSA300_2166"/>
<fbc:geneProduct metaid="SAUSA300_2173" fbc:id="SAUSA300_2173" fbc:label="SAUSA300_2173"/>
<fbc:geneProduct metaid="SAUSA300_2174" fbc:id="SAUSA300_2174" fbc:label="SAUSA300_2174"/>
<fbc:geneProduct metaid="SAUSA300_2175" fbc:id="SAUSA300_2175" fbc:label="SAUSA300_2175"/>
<fbc:geneProduct metaid="SAUSA300_2176" fbc:id="SAUSA300_2176" fbc:label="SAUSA300_2176"/>
<fbc:geneProduct metaid="SAUSA300_2183" fbc:id="SAUSA300_2183" fbc:label="SAUSA300_2183"/>
<fbc:geneProduct metaid="SAUSA300_2210" fbc:id="SAUSA300_2210" fbc:label="SAUSA300_2210"/>
<fbc:geneProduct metaid="SAUSA300_2214" fbc:id="SAUSA300_2214" fbc:label="SAUSA300_2214"/>
<fbc:geneProduct metaid="SAUSA300_2229" fbc:id="SAUSA300_2229" fbc:label="SAUSA300_2229"/>
<fbc:geneProduct metaid="SAUSA300_2230" fbc:id="SAUSA300_2230" fbc:label="SAUSA300_2230"/>
<fbc:geneProduct metaid="SAUSA300_2231" fbc:id="SAUSA300_2231" fbc:label="SAUSA300_2231"/>
<fbc:geneProduct metaid="SAUSA300_2233" fbc:id="SAUSA300_2233" fbc:label="SAUSA300_2233"/>
<fbc:geneProduct metaid="SAUSA300_2234" fbc:id="SAUSA300_2234" fbc:label="SAUSA300_2234"/>
<fbc:geneProduct metaid="SAUSA300_2236" fbc:id="SAUSA300_2236" fbc:label="SAUSA300_2236"/>
<fbc:geneProduct metaid="SAUSA300_2237" fbc:id="SAUSA300_2237" fbc:label="SAUSA300_2237"/>
<fbc:geneProduct metaid="SAUSA300_2238" fbc:id="SAUSA300_2238" fbc:label="SAUSA300_2238"/>
<fbc:geneProduct metaid="SAUSA300_2239" fbc:id="SAUSA300_2239" fbc:label="SAUSA300_2239"/>
<fbc:geneProduct metaid="SAUSA300_2240" fbc:id="SAUSA300_2240" fbc:label="SAUSA300_2240"/>
<fbc:geneProduct metaid="SAUSA300_2241" fbc:id="SAUSA300_2241" fbc:label="SAUSA300_2241"/>
<fbc:geneProduct metaid="SAUSA300_2242" fbc:id="SAUSA300_2242" fbc:label="SAUSA300_2242"/>
<fbc:geneProduct metaid="SAUSA300_2243" fbc:id="SAUSA300_2243" fbc:label="SAUSA300_2243"/>
<fbc:geneProduct metaid="SAUSA300_2244" fbc:id="SAUSA300_2244" fbc:label="SAUSA300_2244"/>
<fbc:geneProduct metaid="SAUSA300_2250" fbc:id="SAUSA300_2250" fbc:label="SAUSA300_2250"/>
<fbc:geneProduct metaid="SAUSA300_2251" fbc:id="SAUSA300_2251" fbc:label="SAUSA300_2251"/>
<fbc:geneProduct metaid="SAUSA300_2254" fbc:id="SAUSA300_2254" fbc:label="SAUSA300_2254"/>

<fbc:geneProduct metaid="SAUSA300_2255" fbc:id="SAUSA300_2255" fbc:label="SAUSA300_2255"/>
<fbc:geneProduct metaid="SAUSA300_2256" fbc:id="SAUSA300_2256" fbc:label="SAUSA300_2256"/>
<fbc:geneProduct metaid="SAUSA300_2258" fbc:id="SAUSA300_2258" fbc:label="SAUSA300_2258"/>
<fbc:geneProduct metaid="SAUSA300_2270" fbc:id="SAUSA300_2270" fbc:label="SAUSA300_2270"/>
<fbc:geneProduct metaid="SAUSA300_2277" fbc:id="SAUSA300_2277" fbc:label="SAUSA300_2277"/>
<fbc:geneProduct metaid="SAUSA300_2278" fbc:id="SAUSA300_2278" fbc:label="SAUSA300_2278"/>
<fbc:geneProduct metaid="SAUSA300_2281" fbc:id="SAUSA300_2281" fbc:label="SAUSA300_2281"/>
<fbc:geneProduct metaid="SAUSA300_2283" fbc:id="SAUSA300_2283" fbc:label="SAUSA300_2283"/>
<fbc:geneProduct metaid="SAUSA300_2285" fbc:id="SAUSA300_2285" fbc:label="SAUSA300_2285"/>
<fbc:geneProduct metaid="SAUSA300_2291" fbc:id="SAUSA300_2291" fbc:label="SAUSA300_2291"/>
<fbc:geneProduct metaid="SAUSA300_2292" fbc:id="SAUSA300_2292" fbc:label="SAUSA300_2292"/>
<fbc:geneProduct metaid="SAUSA300_2293" fbc:id="SAUSA300_2293" fbc:label="SAUSA300_2293"/>
<fbc:geneProduct metaid="SAUSA300_2294" fbc:id="SAUSA300_2294" fbc:label="SAUSA300_2294"/>
<fbc:geneProduct metaid="SAUSA300_2312" fbc:id="SAUSA300_2312" fbc:label="SAUSA300_2312"/>
<fbc:geneProduct metaid="SAUSA300_2313" fbc:id="SAUSA300_2313" fbc:label="SAUSA300_2313"/>
<fbc:geneProduct metaid="SAUSA300_2317" fbc:id="SAUSA300_2317" fbc:label="SAUSA300_2317"/>
<fbc:geneProduct metaid="SAUSA300_2319" fbc:id="SAUSA300_2319" fbc:label="SAUSA300_2319"/>
<fbc:geneProduct metaid="SAUSA300_2323" fbc:id="SAUSA300_2323" fbc:label="SAUSA300_2323"/>
<fbc:geneProduct metaid="SAUSA300_2324" fbc:id="SAUSA300_2324" fbc:label="SAUSA300_2324"/>
<fbc:geneProduct metaid="SAUSA300_2329" fbc:id="SAUSA300_2329" fbc:label="SAUSA300_2329"/>
<fbc:geneProduct metaid="SAUSA300_2333" fbc:id="SAUSA300_2333" fbc:label="SAUSA300_2333"/>
<fbc:geneProduct metaid="SAUSA300_2340" fbc:id="SAUSA300_2340" fbc:label="SAUSA300_2340"/>
<fbc:geneProduct metaid="SAUSA300_2341" fbc:id="SAUSA300_2341" fbc:label="SAUSA300_2341"/>
<fbc:geneProduct metaid="SAUSA300_2342" fbc:id="SAUSA300_2342" fbc:label="SAUSA300_2342"/>
<fbc:geneProduct metaid="SAUSA300_2343" fbc:id="SAUSA300_2343" fbc:label="SAUSA300_2343"/>
<fbc:geneProduct metaid="SAUSA300_2344" fbc:id="SAUSA300_2344" fbc:label="SAUSA300_2344"/>
<fbc:geneProduct metaid="SAUSA300_2345" fbc:id="SAUSA300_2345" fbc:label="SAUSA300_2345"/>
<fbc:geneProduct metaid="SAUSA300_2346" fbc:id="SAUSA300_2346" fbc:label="SAUSA300_2346"/>
<fbc:geneProduct metaid="SAUSA300_2347" fbc:id="SAUSA300_2347" fbc:label="SAUSA300_2347"/>

<fbc:geneProduct metaid="SAUSA300_2349" fbc:id="SAUSA300_2349" fbc:label="SAUSA300_2349"/>
<fbc:geneProduct metaid="SAUSA300_2351" fbc:id="SAUSA300_2351" fbc:label="SAUSA300_2351"/>
<fbc:geneProduct metaid="SAUSA300_2357" fbc:id="SAUSA300_2357" fbc:label="SAUSA300_2357"/>
<fbc:geneProduct metaid="SAUSA300_2358" fbc:id="SAUSA300_2358" fbc:label="SAUSA300_2358"/>
<fbc:geneProduct metaid="SAUSA300_2359" fbc:id="SAUSA300_2359" fbc:label="SAUSA300_2359"/>
<fbc:geneProduct metaid="SAUSA300_2362" fbc:id="SAUSA300_2362" fbc:label="SAUSA300_2362"/>
<fbc:geneProduct metaid="SAUSA300_2369" fbc:id="SAUSA300_2369" fbc:label="SAUSA300_2369"/>
<fbc:geneProduct metaid="SAUSA300_2370" fbc:id="SAUSA300_2370" fbc:label="SAUSA300_2370"/>
<fbc:geneProduct metaid="SAUSA300_2371" fbc:id="SAUSA300_2371" fbc:label="SAUSA300_2371"/>
<fbc:geneProduct metaid="SAUSA300_2372" fbc:id="SAUSA300_2372" fbc:label="SAUSA300_2372"/>
<fbc:geneProduct metaid="SAUSA300_2373" fbc:id="SAUSA300_2373" fbc:label="SAUSA300_2373"/>
<fbc:geneProduct metaid="SAUSA300_2377" fbc:id="SAUSA300_2377" fbc:label="SAUSA300_2377"/>
<fbc:geneProduct metaid="SAUSA300_2383" fbc:id="SAUSA300_2383" fbc:label="SAUSA300_2383"/>
<fbc:geneProduct metaid="SAUSA300_2384" fbc:id="SAUSA300_2384" fbc:label="SAUSA300_2384"/>
<fbc:geneProduct metaid="SAUSA300_2388" fbc:id="SAUSA300_2388" fbc:label="SAUSA300_2388"/>
<fbc:geneProduct metaid="SAUSA300_2390" fbc:id="SAUSA300_2390" fbc:label="SAUSA300_2390"/>
<fbc:geneProduct metaid="SAUSA300_2391" fbc:id="SAUSA300_2391" fbc:label="SAUSA300_2391"/>
<fbc:geneProduct metaid="SAUSA300_2392" fbc:id="SAUSA300_2392" fbc:label="SAUSA300_2392"/>
<fbc:geneProduct metaid="SAUSA300_2393" fbc:id="SAUSA300_2393" fbc:label="SAUSA300_2393"/>
<fbc:geneProduct metaid="SAUSA300_2404" fbc:id="SAUSA300_2404" fbc:label="SAUSA300_2404"/>
<fbc:geneProduct metaid="SAUSA300_2407" fbc:id="SAUSA300_2407" fbc:label="SAUSA300_2407"/>
<fbc:geneProduct metaid="SAUSA300_2408" fbc:id="SAUSA300_2408" fbc:label="SAUSA300_2408"/>
<fbc:geneProduct metaid="SAUSA300_2409" fbc:id="SAUSA300_2409" fbc:label="SAUSA300_2409"/>
<fbc:geneProduct metaid="SAUSA300_2410" fbc:id="SAUSA300_2410" fbc:label="SAUSA300_2410"/>
<fbc:geneProduct metaid="SAUSA300_2411" fbc:id="SAUSA300_2411" fbc:label="SAUSA300_2411"/>
<fbc:geneProduct metaid="SAUSA300_2416" fbc:id="SAUSA300_2416" fbc:label="SAUSA300_2416"/>
<fbc:geneProduct metaid="SAUSA300_2429" fbc:id="SAUSA300_2429" fbc:label="SAUSA300_2429"/>
<fbc:geneProduct metaid="SAUSA300_2433" fbc:id="SAUSA300_2433" fbc:label="SAUSA300_2433"/>
<fbc:geneProduct metaid="SAUSA300_2438" fbc:id="SAUSA300_2438" fbc:label="SAUSA300_2438"/>

<fbc:geneProduct metaid="SAUSA300_2439" fbc:id="SAUSA300_2439" fbc:label="SAUSA300_2439"/>
<fbc:geneProduct metaid="SAUSA300_2442" fbc:id="SAUSA300_2442" fbc:label="SAUSA300_2442"/>
<fbc:geneProduct metaid="SAUSA300_2443" fbc:id="SAUSA300_2443" fbc:label="SAUSA300_2443"/>
<fbc:geneProduct metaid="SAUSA300_2446" fbc:id="SAUSA300_2446" fbc:label="SAUSA300_2446"/>
<fbc:geneProduct metaid="SAUSA300_2449" fbc:id="SAUSA300_2449" fbc:label="SAUSA300_2449"/>
<fbc:geneProduct metaid="SAUSA300_2455" fbc:id="SAUSA300_2455" fbc:label="SAUSA300_2455"/>
<fbc:geneProduct metaid="SAUSA300_2461" fbc:id="SAUSA300_2461" fbc:label="SAUSA300_2461"/>
<fbc:geneProduct metaid="SAUSA300_2462" fbc:id="SAUSA300_2462" fbc:label="SAUSA300_2462"/>
<fbc:geneProduct metaid="SAUSA300_2463" fbc:id="SAUSA300_2463" fbc:label="SAUSA300_2463"/>
<fbc:geneProduct metaid="SAUSA300_2469" fbc:id="SAUSA300_2469" fbc:label="SAUSA300_2469"/>
<fbc:geneProduct metaid="SAUSA300_2470" fbc:id="SAUSA300_2470" fbc:label="SAUSA300_2470"/>
<fbc:geneProduct metaid="SAUSA300_2474" fbc:id="SAUSA300_2474" fbc:label="SAUSA300_2474"/>
<fbc:geneProduct metaid="SAUSA300_2475" fbc:id="SAUSA300_2475" fbc:label="SAUSA300_2475"/>
<fbc:geneProduct metaid="SAUSA300_2477" fbc:id="SAUSA300_2477" fbc:label="SAUSA300_2477"/>
<fbc:geneProduct metaid="SAUSA300_2483" fbc:id="SAUSA300_2483" fbc:label="SAUSA300_2483"/>
<fbc:geneProduct metaid="SAUSA300_2484" fbc:id="SAUSA300_2484" fbc:label="SAUSA300_2484"/>
<fbc:geneProduct metaid="SAUSA300_2487" fbc:id="SAUSA300_2487" fbc:label="SAUSA300_2487"/>
<fbc:geneProduct metaid="SAUSA300_2488" fbc:id="SAUSA300_2488" fbc:label="SAUSA300_2488"/>
<fbc:geneProduct metaid="SAUSA300_2491" fbc:id="SAUSA300_2491" fbc:label="SAUSA300_2491"/>
<fbc:geneProduct metaid="SAUSA300_2492" fbc:id="SAUSA300_2492" fbc:label="SAUSA300_2492"/>
<fbc:geneProduct metaid="SAUSA300_2494" fbc:id="SAUSA300_2494" fbc:label="SAUSA300_2494"/>
<fbc:geneProduct metaid="SAUSA300_2495" fbc:id="SAUSA300_2495" fbc:label="SAUSA300_2495"/>
<fbc:geneProduct metaid="SAUSA300_2498" fbc:id="SAUSA300_2498" fbc:label="SAUSA300_2498"/>
<fbc:geneProduct metaid="SAUSA300_2499" fbc:id="SAUSA300_2499" fbc:label="SAUSA300_2499"/>
<fbc:geneProduct metaid="SAUSA300_2500" fbc:id="SAUSA300_2500" fbc:label="SAUSA300_2500"/>
<fbc:geneProduct metaid="SAUSA300_2501" fbc:id="SAUSA300_2501" fbc:label="SAUSA300_2501"/>
<fbc:geneProduct metaid="SAUSA300_2502" fbc:id="SAUSA300_2502" fbc:label="SAUSA300_2502"/>
<fbc:geneProduct metaid="SAUSA300_2505" fbc:id="SAUSA300_2505" fbc:label="SAUSA300_2505"/>
<fbc:geneProduct metaid="SAUSA300_2520" fbc:id="SAUSA300_2520" fbc:label="SAUSA300_2520"/>

```
            <fbc:geneProduct metaid="SAUSA300_2526" fbc:id="SAUSA300_2526"
fbc:label="SAUSA300_2526"/>
            <fbc:geneProduct metaid="SAUSA300_2532" fbc:id="SAUSA300_2532"
fbc:label="SAUSA300_2532"/>
            <fbc:geneProduct metaid="SAUSA300_2533" fbc:id="SAUSA300_2533"
fbc:label="SAUSA300_2533"/>
            <fbc:geneProduct metaid="SAUSA300_2534" fbc:id="SAUSA300_2534"
fbc:label="SAUSA300_2534"/>
            <fbc:geneProduct metaid="SAUSA300_2535" fbc:id="SAUSA300_2535"
fbc:label="SAUSA300_2535"/>
            <fbc:geneProduct metaid="SAUSA300_2536" fbc:id="SAUSA300_2536"
fbc:label="SAUSA300_2536"/>
            <fbc:geneProduct metaid="SAUSA300_2537" fbc:id="SAUSA300_2537"
fbc:label="SAUSA300_2537"/>
            <fbc:geneProduct metaid="SAUSA300_2539" fbc:id="SAUSA300_2539"
fbc:label="SAUSA300_2539"/>
            <fbc:geneProduct metaid="SAUSA300_2540" fbc:id="SAUSA300_2540"
fbc:label="SAUSA300_2540"/>
            <fbc:geneProduct metaid="SAUSA300_2541" fbc:id="SAUSA300_2541"
fbc:label="SAUSA300_2541"/>
            <fbc:geneProduct metaid="SAUSA300_2542" fbc:id="SAUSA300_2542"
fbc:label="SAUSA300_2542"/>
            <fbc:geneProduct metaid="SAUSA300_2545" fbc:id="SAUSA300_2545"
fbc:label="SAUSA300_2545"/>
            <fbc:geneProduct metaid="SAUSA300_2546" fbc:id="SAUSA300_2546"
fbc:label="SAUSA300_2546"/>
            <fbc:geneProduct metaid="SAUSA300_2549" fbc:id="SAUSA300_2549"
fbc:label="SAUSA300_2549"/>
            <fbc:geneProduct metaid="SAUSA300_2550" fbc:id="SAUSA300_2550"
fbc:label="SAUSA300_2550"/>
            <fbc:geneProduct metaid="SAUSA300_2551" fbc:id="SAUSA300_2551"
fbc:label="SAUSA300_2551"/>
            <fbc:geneProduct metaid="SAUSA300_2552" fbc:id="SAUSA300_2552"
fbc:label="SAUSA300_2552"/>
            <fbc:geneProduct metaid="SAUSA300_2553" fbc:id="SAUSA300_2553"
fbc:label="SAUSA300_2553"/>
            <fbc:geneProduct metaid="SAUSA300_2554" fbc:id="SAUSA300_2554"
fbc:label="SAUSA300_2554"/>
            <fbc:geneProduct metaid="SAUSA300_2555" fbc:id="SAUSA300_2555"
fbc:label="SAUSA300_2555"/>
            <fbc:geneProduct metaid="SAUSA300_2561" fbc:id="SAUSA300_2561"
fbc:label="SAUSA300_2561"/>
            <fbc:geneProduct metaid="SAUSA300_2567" fbc:id="SAUSA300_2567"
fbc:label="SAUSA300_2567"/>
            <fbc:geneProduct metaid="SAUSA300_2568" fbc:id="SAUSA300_2568"
fbc:label="SAUSA300_2568"/>
            <fbc:geneProduct metaid="SAUSA300_2569" fbc:id="SAUSA300_2569"
fbc:label="SAUSA300_2569"/>
            <fbc:geneProduct metaid="SAUSA300_2570" fbc:id="SAUSA300_2570"
fbc:label="SAUSA300_2570"/>
            <fbc:geneProduct metaid="SAUSA300_2576" fbc:id="SAUSA300_2576"
fbc:label="SAUSA300_2576"/>
            <fbc:geneProduct metaid="SAUSA300_2577" fbc:id="SAUSA300_2577"
fbc:label="SAUSA300_2577"/>
            <fbc:geneProduct metaid="SAUSA300_2579" fbc:id="SAUSA300_2579"
fbc:label="SAUSA300_2579"/>
            <fbc:geneProduct metaid="SAUSA300_2580" fbc:id="SAUSA300_2580"
fbc:label="SAUSA300_2580"/>
```

```
            <fbc:geneProduct metaid="SAUSA300_2583" fbc:id="SAUSA300_2583"
fbc:label="SAUSA300_2583"/>
            <fbc:geneProduct metaid="SAUSA300_2594" fbc:id="SAUSA300_2594"
fbc:label="SAUSA300_2594"/>
            <fbc:geneProduct metaid="SAUSA300_2597" fbc:id="SAUSA300_2597"
fbc:label="SAUSA300_2597"/>
            <fbc:geneProduct metaid="SAUSA300_2605" fbc:id="SAUSA300_2605"
fbc:label="SAUSA300_2605"/>
            <fbc:geneProduct metaid="SAUSA300_2606" fbc:id="SAUSA300_2606"
fbc:label="SAUSA300_2606"/>
            <fbc:geneProduct metaid="SAUSA300_2607" fbc:id="SAUSA300_2607"
fbc:label="SAUSA300_2607"/>
            <fbc:geneProduct metaid="SAUSA300_2608" fbc:id="SAUSA300_2608"
fbc:label="SAUSA300_2608"/>
            <fbc:geneProduct metaid="SAUSA300_2609" fbc:id="SAUSA300_2609"
fbc:label="SAUSA300_2609"/>
            <fbc:geneProduct metaid="SAUSA300_2610" fbc:id="SAUSA300_2610"
fbc:label="SAUSA300_2610"/>
            <fbc:geneProduct metaid="SAUSA300_2611" fbc:id="SAUSA300_2611"
fbc:label="SAUSA300_2611"/>
            <fbc:geneProduct metaid="SAUSA300_2612" fbc:id="SAUSA300_2612"
fbc:label="SAUSA300_2612"/>
            <fbc:geneProduct metaid="SAUSA300_2613" fbc:id="SAUSA300_2613"
fbc:label="SAUSA300_2613"/>
            <fbc:geneProduct metaid="SAUSA300_2616" fbc:id="SAUSA300_2616"
fbc:label="SAUSA300_2616"/>
            <fbc:geneProduct metaid="SAUSA300_2617" fbc:id="SAUSA300_2617"
fbc:label="SAUSA300_2617"/>
            <fbc:geneProduct metaid="SAUSA300_2627" fbc:id="SAUSA300_2627"
fbc:label="SAUSA300_2627"/>
            <fbc:geneProduct metaid="SAUSA300_2630" fbc:id="SAUSA300_2630"
fbc:label="SAUSA300_2630"/>
            <fbc:geneProduct metaid="SAUSA300_RS13580"
fbc:id="SAUSA300_RS13580" fbc:label="SAUSA300_RS13580"/>
            <fbc:geneProduct metaid="SAUSA300_RS13740"
fbc:id="SAUSA300_RS13740" fbc:label="SAUSA300_RS13740"/>
            <fbc:geneProduct metaid="sbnG" fbc:id="sbnG" fbc:label="sbnG"/>
            <fbc:geneProduct metaid="speC" fbc:id="speC" fbc:label="speC"/>
            <fbc:geneProduct metaid="UbiA" fbc:id="UbiA" fbc:label="UbiA"/>
            <fbc:geneProduct metaid="ubiI" fbc:id="ubiI" fbc:label="ubiI"/>
            <fbc:geneProduct metaid="uxuAB" fbc:id="uxuAB"
fbc:label="uxuAB"/>
            <fbc:geneProduct metaid="uxuB" fbc:id="uxuB" fbc:label="uxuB"/>
      </fbc:listOfGeneProducts>

      </model>
</sbml>
```
